# A Michaelis‐Arbuzov‐Type Pathway to a Protected 2ʹ‐Deoxy‐2ʹ‐Selenomethyl‐Adenosine‐3ʹ,5ʹ‐Phosphoroselenolate Guanosine Dinucleotide for Use in Modified m7G Cap Synthesis

**DOI:** 10.1002/chem.202502321

**Published:** 2026-01-10

**Authors:** K. Lawrence E. Hale, Helen O. McCarthy, Mark G. McLaughlin

**Affiliations:** ^1^ School of Chemistry and Chemical Engineering Queen's University Belfast Belfast Northern Ireland UK; ^2^ School of Pharmacy Queen's University Belfast Belfast Northern Ireland UK

**Keywords:** 5ʹ‐O‐phosphorylation, m7G cap, Michaelis‐Arbuzov selenocyanate ligation, nucleoside O2ʹ‐triflate displacements, nucleoside selenomethylation, n_o_→ σ*_P‐O_ hyperconjugation, solid CPR II, the phosphite α‐effect, Vicinal Triflate Effect

## Abstract

In this paper, a solution‐phase total synthesis is described of the 5ʹ‐O‐phosphorylated 2ʹ‐deoxy‐2ʹ‐selenomethyl‐adenosine 3ʹ,5ʹ‐phosphoroselenolate guanosine dinucleotide (**4**) using a rt, 2,6‐lutidine‐mediated, nucleoside 3ʹ‐H‐phosphonate/5ʹ‐selenocyanate Michaelis‐Arbuzov ligative coupling, whose potential mechanism is discussed herein. Our route to **4** is predicated upon the highly efficient new 2ʹ‐O‐triflate selenocyanate anion displacement of **10** in MeCN, to obtain the 2ʹ‐selenocyanate **9**, which was allied with an Amosova NaBH_4_/MeI mediated reductive selenomethylation in MeOH. A novel solid CPR II‐mediated 5ʹ‐O‐phosphitylation of the dinucleotide alcohol **6** and a 70% aqueous *t*‐BuO_2_H oxidation successfully installed the 5ʹ‐O‐phosphate moiety within **4** without oxidizing the 2ʹ‐selenomethyl group. It is envisaged that **4** will be of value for a future total synthesis of the m7G cap **1**; a molecule of potential utility for therapeutic mRNA manufacture.

## Graphical abstract

Here we report a synthesis of the dinucleotide **4** for a projected future total synthesis of the m7G cap **1**. Highlights of the route include: 1) a 3ʹ‐H‐phosphonate/5ʹ‐selenocyanate Michaelis‐Arbuzov coupling to forge the 3ʹ,5ʹ‐phosphoroselenolate; 2) an Amosova reductive selenomethylation to install the 2ʹ‐SeMe; 3) a solid CPR II 5ʹ‐O‐phosphitylation; and 4) an aq. *t*BuO_2_H 5ʹ‐O‐phosphite oxidation without accompanying selenoxide formation. Also, MeCN has been found to be especially beneficial for the S*_N_* 2 reactions of **10** and the iodide **12**.

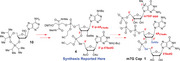

## 1 Introduction

**SCHEME 1:**
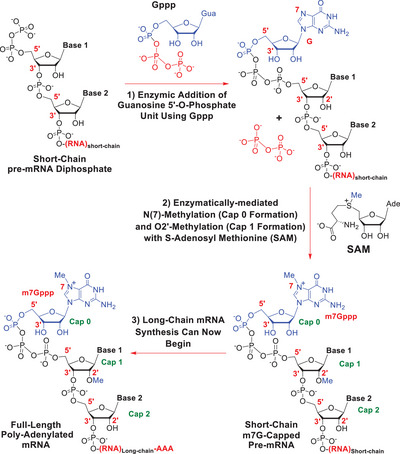
A summary of how m7G cap formation occurs biochemically *in vivo*.

7‐Methyl guanosine (m7G) capping of short‐chain oligo‐ and poly‐nucleotides is one of the key events in helping *long‐chain* polyadenylated polynucleotide transcripts form from linear DNA coding templates, through the action of RNA polymerases, both *in vivo* and *in vitro* [1, 2, 3, 4, 5, 6, 7, 8]. The presence of an m7G cap is an essential requirement for the export of full‐length mRNA transcripts from the nucleus into the cytoplasm, the stabilization of those transcripts, and their subsequent molecular recognition by the eukaryotic initiation factor 4E (eIF4E) protein, so enabling the 80S ribosome to fully form and subsequently translate the transcripts into full‐length proteins [9, 10]. Importantly, 2ʹ‐O‐methylation of the cap 1 adenosine sub‐structure in m7G‐capped mRNAs (Scheme 1) further renders them nonimmunogenic and unable to be recognized as foreign RNA, by a host's immune system [11]. It does this by preventing the cap region from interacting with the RIG1 receptor. It will thus be appreciated that m7G capping is a key operation in cellular homeostasis and *in vitro* mRNA manufacture, and there are many reviews written upon it [1, 2, 3, 4, 5, 6, 7, 8]. The main biochemical events that underpin biological capping are detailed in Scheme 1 and involve multiple enzymes working in tandem.

Following the successful introduction of the Pfizer/ BioNTech [12, 13, 14] and Moderna [15] COVID19 mRNA vaccines in 2020, there has been growing interest in improving the technologies that are currently available for *in vitro* mRNA manufacture, and one component of the manufacturing process that has garnered special attention has been the synthesis of modified m7G caps for use in the *in vitro* transcription process [16, 17, 18, 19, 20, 21, 22]. This is due to the significant potential that modified m7G caps have for greatly increasing mRNA transcription yields, and providing more enzymatically‐stabilised mRNA structures that can message for much longer within the body. Of course, a longer duration of translational messaging could be expected to lead to improved therapeutic protein yields within a patient and this, in turn, could be expected to result in significantly enhanced *in vivo* drug efficacy for those therapeutic mRNAs.

Consequentially, a key design element for all new synthetic m7G cap structures is the ability to confer much greater stability and resistance on the resulting capped mRNA transcripts toward cytoplasmic Dcp1 and Dcp 2 decapping enzymes, and decapping 5ʹ3ʹ‐exonucleases such as DXO [23, 24]. The primary aim is not to totally inhibit these types of enzyme, but instead, to slow their ability to process and decap the newly fashioned therapeutic mRNAs, since the former action might render such capped mRNAs potentially oncogenic [23, 24].

The next generation caps will also need to be designed to allow for more convenient and improved capped mRNA drug purification, easier drug formulation and delivery, while also conferring extremely low levels of immunogenicity on the resulting drugs, most especially the inability to induce anaphylactic shock or more general systemic toxicity.

While there have been many important advances made in modified m7G cap synthesis in recent years, most especially by the teams of Kore [19, 21, 25, 26, 27] and Jemielity [23, 24, 28, 29, 30, 31, 32], there has not yet been any *universal m7G cap* so far identified that excels in *all* aspects of chemical and biological performance, be it the ease of cap synthesis, the cheapness of synthesis, the ability of the cap to markedly increase transcription yields, or the capacity of the cap to confer the necessary levels of enzymic resistance upon the resulting capped mRNAs, to so prevent premature cleavage by decapping Dcp 1 and 2 and DXO 5ʹ3ʹ‐exonuclease enzymes [23, 24, 28, 29, 30, 31, 32], whilst also not causing inhibition of those enzymes.

Generally, most structural modifications that enhance one area of performance are often detrimental to other aspects of performance, or the overall ease of cap synthesis, or the final production cost. Either that or they increase the environmental footprint of the chemistry needed to synthesize the cap or the cap's ability to inhibit Dcp enzymes and 5ʹ3ʹ‐exonucleases.

**SCHEME 2:**
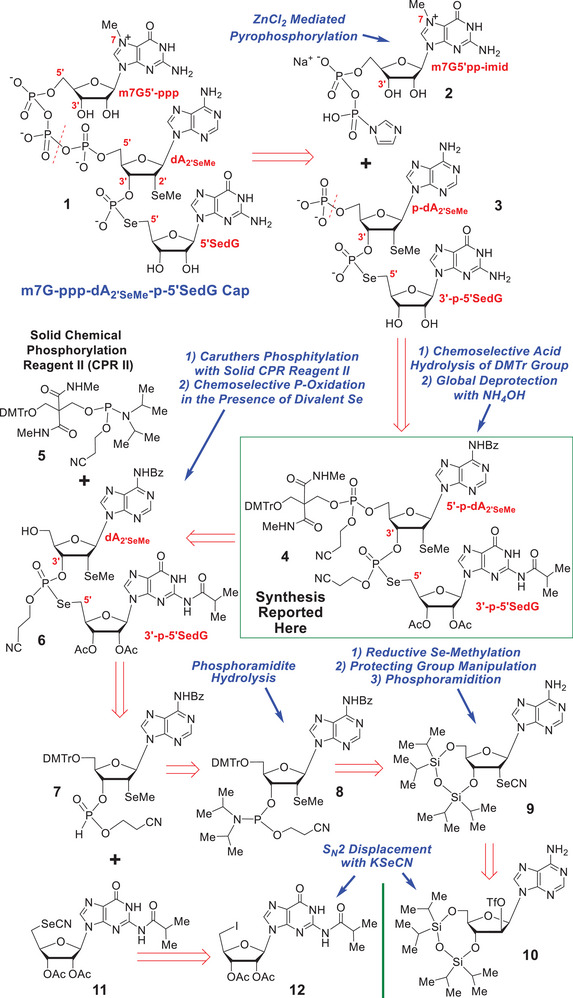
Retrosynthetic planning for the m7G cap 1 and its anticipated dinucleotide precursor **4**.

As part of a general program aimed at designing new m7G caps with the requisite universality of scope and performance for future manufacturing, we became interested in developing the synthetic methodology needed to synthesize the cap1/cap2 dinucleotide structure **4** (Scheme 2), which we hoped would serve as an advanced intermediate in a future total synthesis of the modified m7G cap **1** (see Scheme 2 for the structure of **1**).

In this connection, we here describe a synthesis of the advanced intermediate **4** (Scheme 2), by a route that is both economic in cost and scalable, and which showcases many important new methodological developments of more widespread significance and utility to the oligo‐ and poly‐nucleotide synthetic field.

## 2 Results and Discussion

For some time now, the conformationally mobile C‐terminal loop of the eIF4E protein has been known to be the primary receptor of the second, C(2ʹ)‐O‐methylated, cap 1 nucleotide of fully elaborated m7G capped mRNAs [9]. It has also been recognized that this region contains multiple hydrophobic amino‐acid arrays. So, from a new m7G cap design perspective, we reasoned that if we could replace the C(2ʹ)‐OMe of the cap 1 adenosine nucleotide with a much larger and more hydrophobic C(2ʹ)‐SeMe group, this might significantly increase the hydrophobicity and tightness of binding of the cap 1 motif to the conformationally flexible C‐terminal eIF4E region, and potentially enhance the overall binding affinity of the resulting capped mRNAs for the eIF4E protein. Most especially, if a second divalent Se donor group could simultaneously be positioned within the adjoining cap 2 structure, just 5 atoms removed from the new C(2ʹ)‐SeMe, since this would potentially allow for bidentate or even tridentate (via the dipolar P = O) cooperative chelative‐type hydrophobic/electrostatic/hole interactions between the cap and the protein, provided the Se atom was part of a 5ʹ3ʹ‐phosphoroselenolate internucleotide linkage, in what would potentially be an example of Williams’ well‐known “Gulliver Effect” in small molecule‐protein‐binding [33, 34].

The fact that Jemielity [28] had previously demonstrated that when O‐ or S‐atoms are replaced by a more polarisable Se within a cap structure, this can often have a beneficial effect on m7G cap performance, further attracted us in this general direction.

We reasoned that if we could promote a much tighter *overall* hydrophobic and electrostatic interaction between the capped mRNAs and the eIF4E protein, we might be able to potentially enhance translational messaging, to increase the level of therapeutic protein expressed within a treated patient's diseased cells.

Moreover, because of the close structural similarity between an OMe and a SeMe group in 2ʹ‐SeMe and 2ʹ‐OMe uridine [35], which both strongly favor the *endo*‐3ʹ/North conformation being adopted by the sugar ring, we strongly suspected that a 2ʹ‐SeMe would thus very effectively mimic the 2ʹ‐OMe adenosine in a cap 1, but potentially even more effectively prevent the resulting m7G‐capped mRNAs from interacting with and activating the RIG1 innate immune receptor (to activate the immune system) [11], due to the greatly increased size of the Se‐atom compared to an O‐atom. We thus considered that therapeutic mRNAs bearing the m7G cap **1** might have a very good chance of successfully evading host immune system recognition and being nonimmunogenic, while also undergoing much slower decapping by cytoplasmic DXO exonucleases [7, 23, 24].

Jemielity has also reported that O2ʹ‐methylation of the second Cap 1 nucleotide can significantly increase protein production in immature dendritic cells [23, 24, 32]. It was thus of interest to see whether the presence of a C2ʹ‐SeMe in therapeutic m7G‐capped therapeutic mRNAs might further improve protein expression in target cells.

Another design perspective underpinning our selection of **1** as a prospective m7G cap, and intermediate **4** as its immediate synthetic precursor, was the fact that Huang [36] and Micura [37, 38, 39] have both previously found that having a C2ʹ‐SeMe residue resident within long‐chain DNAs and RNAs can confer added enzymic and chemical stability upon such transcripts.

The presence of multiple C(2ʹ)‐SeMe residues in analogous DNA sequences has also often imparted significant crystallinity upon the resulting polynucleotide sequences, and so if a similar situation prevailed here, this might not only facilitate final capped mRNA purification, it might also greatly assist future chemobiological X‐ray crystallographic investigations into how such capped mRNAs interact with their protein targets, by providing multiple heavy atom centers within the cap structure to enhance anomalous scattering and help with solution of the X‐ray crystallographic phasing problem [36, 37, 38, 39].

Now that the logic underpinning our design of the modified m7G cap **1** has been espoused, our retrosynthetic planning for **1** and its dinucleotide precursor **4** (Scheme 2) will be presented.

Central to our strategy was the classic Mukaiyama m7G 5’‐diphosphoroimidazolide coupling [40] between **2** and **3** mediated by ZnCl_2_ in DMF [25, 26, 27, 28, 29, 30, 31, 32]. A type of coupling popularized by Jemielity and Kore [25, 26, 27, 28, 29, 30, 31, 32] for modified m7G cap synthesis. The 5ʹ‐O‐monophosphate **3** would itself be obtained from the protected dinucleotide **4** by chemoselective acid hydrolysis of its dimethoxytrityl (DMTr) group followed by global deprotection of all the protecting groups with concentrated aqueous ammonia. Further disconnection of **4** at its cap 1 P‐O bond thereafter led to the alcohol **6** and phosphoramidite **5** as potential precursors. The latter is currently sold commercially under the trade name of solid Chemical Phosphorylation Reagent II (CPR II) [41].

Notwithstanding its commercial availability, the use of solid CPR II (**5**) for nucleoside 5ʹ‐hydroxyl *O*‐phosphorylation has so far only been described in a very limited way [41, 42, 43, 44], with no experimental procedures having been reported either for the O‐phosphitylation or the I_2_/H_2_O/pyridine/THF oxidation steps that deliver the O5ʹ‐phosphate. That said, representative conditions have been devised for the later 5ʹ‐O‐phosphate deprotections.

Given this dearth of experimental information on the use of solid CPR II in 5ʹ‐*O*‐phosphorylation, we thought it useful that we properly define its synthetic potential in the context of a solution‐phase synthesis of the synthetically‐challenging dinucleotide **4**. Such an application would require a standard Caruthers phosphitylation reaction [45, 46, 47, 48, 49, 50] being implemented upon **5** and **6**, followed by a highly chemoselective P‐oxidation in the presence of a readily oxidizable divalent SeMe group. An oxidation that had no literature precedent at the very outset of this study, and which would likely require new synthetic technology being developed to attain success.

Indeed, all of the prior work on solid‐supported 2ʹ‐selenomethyl polynucleotide synthesis by Huang [36] and Micura [37, 38, 39], had always unavoidably oxidized the divalent selenoalkyl functionality to the selenoxide during the phosphite to phosphate transition with I_2_/H_2_O/pyridine/THF, which then required a reduction with a massive excess of 1,4‐dithiothreitol to restore the SeMe functionality. It was thus of great interest to see whether such a phosphite oxidation could be achieved *chemoselectively* in the demanding test environment provided by **4** without reliance on such reduction tactics.

Our further retrosynthetic dissection of the dinucleotide **6** thereafter led to a modified Michaelis‐Arbuzov‐type reaction [51, 52, 53] being selected between the 3ʹ‐H‐phosphonate **7** and the 5ʹ‐selenocyanate **11** for formation of the unusual 3ʹ,5ʹ‐phosphoroselenolate linkage that would ultimately reside within **4**. If successful, this would be the first time such a phosphoroselenolate linkage had been fashioned in a *ribo*‐nucleotide system that possessed a bulky substituent at C2ʹ, directly adjacent to the 3ʹ‐H‐phosphonate coupling site. While such ligations have been shown to be perfectly viable in unhindered 2ʹ‐deoxy‐3ʹ‐H‐phosphonate nucleotide systems [51, 52], there was no prior literature precedent in more sterically crowded situations when we commenced this work. A successful union between **7** and **11** would thus significantly expand the scope of this novel *room temperature* ligation technology.

3ʹ‐H‐Phosphonate **7** was considered accessible through the phosphoramidite hydrolysis [51, 52] of **8**, which itself would be accessed from **9** by reductive selenomethylation of the selenocyanate moiety in **9**, O‐desilylation, regioselective dimethoxytritylation, and Caruthers phosphoramidation [45, 46, 47, 48, 49, 50]. A KSeCN S*_N_* 2 displacement was envisaged for securing **9** from **10**, and **11** from iodide **12**.

**SCHEME 3:**
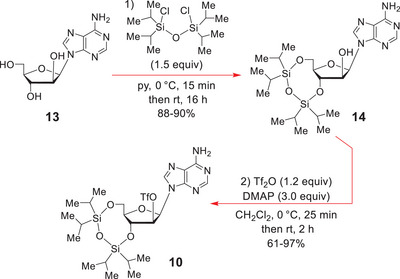
Synthesis of the C(2ʹ)‐O‐triflate **10**.

Our synthetic route to the 3ʹ‐H‐phosphonate **7** commenced from commercially available D‐*arabino*‐adenosine **13** (Scheme 3) which was converted into Robins’ protected O‐triflate ester **10** [54] by a modification of literature procedures (see Supporting Information).

Specifically, **13** was regioselectively O‐silylated with 1,3‐dichloro‐1,1,3,3‐tetraisopropyldisiloxane (1.5 equiv) in dry pyridine under an N_2_ atmosphere, to give **14**, using a new adaption of Robins’ published protocol for adenosine [55]; the starting sugar having been carefully dehydrated by azeotropic evaporation from dry pyridine.

**SCHEME 4:**
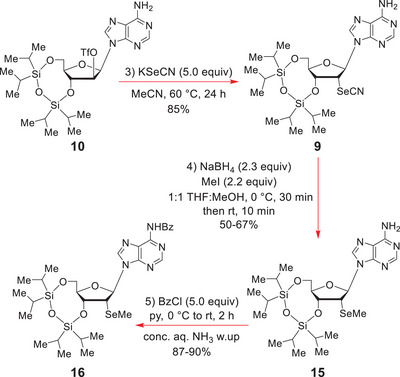
O(2ʹ)‐Triflate ester displacement, Amosova [60] reductive selenomethylation and N(6)‐protection.

An O‐triflate ester was specifically selected for this S*_N_* 2 displacement with KSeCN to obtain **9** (Scheme 4), in order to benefit from the “Vicinal Triflate Effect” [56, 57, 58]; a phenomenon that Hale and Manaviazar [56, 57] have stated can significantly lower adjacent opposing dipolar repulsions in developing S*_N_* 2 transition states of systems of this sort. In this instance, the opposing dipoles would arise from the C(1ʹ)‐O, the C(1ʹ)‐N and the C(3ʹ)‐O bonds.

Now, despite these dipoles each being substantially lowered by the O(2ʹ)‐triflate ester in **10**, still, this S*_N_* 2 displacement took 24 h to reach completion when the reactants were heated at 60°C in MeCN (Scheme 4).

Our next objective was to convert the selenocyanate **9** into the 2ʹ‐selenomethyl ether **15**, by dissolving it in dry THF, and adding 10.0 equiv of dry MeOH followed by 2.0 equiv of Bu_3_P. It had been hoped that this would form the Bu_3_P^+^‐SeR ion and thence its Bu_3_P^+^‐OMe counterpart, which would then be attacked by the nucleoside selenyl anion to give **15**, analogously to Grieco's classical phenylselenation of alcohols [59]. Disappointingly, this reaction failed.

Thereafter, we became aware of Amosova's [60] *one‐pot* reductive alkylation of alkyl selenocyanates to give selenoethers, which significantly extended Spears [61] and Krief's [62] earlier studies on the reduction of selenocyanates with metal borohydrides [61, 62]. Encouraged by Amosova's positive results, we subjected **9** to a similar 0°C reductive alkylation with NaBH_4_ and MeI in THF:MeOH (1:1). TLC analysis quickly revealed that all the starting selenocyanate **9** had been consumed within 0.5 h, and that a single slightly faster‐moving product had formed, which corresponded to **15**.

This new selenomethylation protocol offers special synthetic advantages inasmuch as it now totally removes the need to use prohibitively expensive MeSeNa in THF/EtOH for the S*_N_* 2 displacement of **10** and its congeners [37].

**SCHEME 5:**
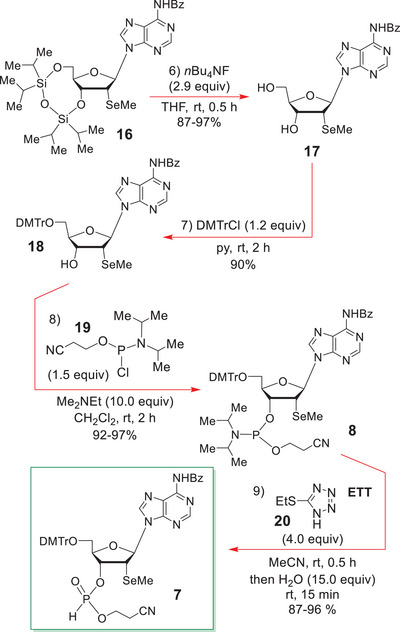
Completion of the synthesis of the 3ʹ‐H‐phosphonate **7**.

The C(6)‐NH_2_ of the adenine sub‐unit in **15** was now protected as an N‐benzamide with benzoyl chloride in pyridine (Scheme 4) to prevent unwanted side reactions from occurring later on during the phosphoramidite‐forming step. The 3ʹ,5ʹ‐*O*‐(1,1,3,3‐tetraisopropyl‐1,3‐disiloxanyl) ether was then cleaved from **16** by treatment with *n*‐Bu_4_NF in THF at rt (Scheme 5).

The next step in the sequence was the selective O‐tritylation of **17** with 4,4ʹ‐dimethoxytrityl chloride (1.2 equiv) in pyridine at rt over 2 h. Once more this reaction proceeded cleanly, affording the 5ʹ‐O‐dimethoxytrityl (DMTr) ether **18** in 90% yield.

The phosphoramidite **8** was best synthesized under modified Caruthers phosphitylation conditions [45, 46, 47, 48, 49, 50] where the alcohol **18** was successively treated with Me_2_NEt (10.0 equiv) and 2‐cyanoethyl *N,N*‐diisopropylchlorophosphoramidite (1.5 equiv) at rt for 2 h and quenched with MeOH. The slightly impure **8** (formed in *ca*. 92–97% yield after precipitative removal of the cyanoethyl methyl phosphoramidate by‐product with EtOAc/hexane) was then sufficiently pure for use in the hydrolytic H‐phosphonate‐forming step [51, 52, 53], which entailed reacting **8** with 5‐(ethylthio)‐1H‐tetrazole **20** (ETT) in MeCN for 0.5 h, before adding H_2_O and stirring for another 15 min.

**SCHEME 6:**
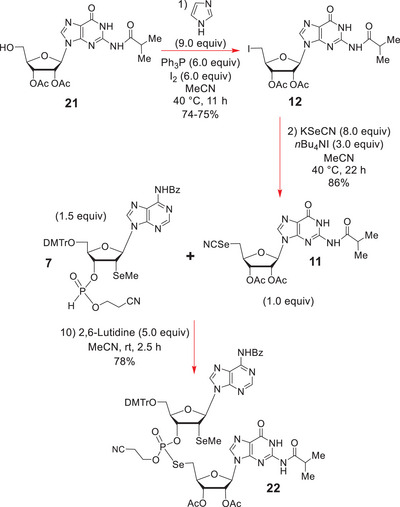
Synthesis of the phosphoroselenolate **22** via the 2,6‐lutidine mediated Michaelis‐Arbuzov‐type reaction of H‐phosphonate **7** with 5‐selenocyanate **11**.

With the 3ʹ‐(cyanomethyl)‐H‐phosphonate **7** in hand, attention turned to the preparation of its 5ʹ‐selenocyanate G coupling partner **11** from the iodide **12** (Scheme 6). The latter was obtained by Garegg iodination [63, 64, 65] of **21** with Ph_3_P, I_2_, and imidazole in MeCN at 40°C, with strict adherence to the 11 h reaction duration, to reduce base‐induced cyclonucleoside formation, which significantly erodes yield beyond that timeframe.

Iodide **12** was found to undergo a smooth S*_N_* 2 displacement, *without cyclonucleoside formation*, when it was exposed to a significant excess of KSeCN and *n*Bu_4_NI in MeCN at 40°C for 22 h; a reaction that provided **11** in 86% yield. *n*Bu_4_NI was added to help to solubilize the excess KSeCN and create a quite high concentration of the more soluble *n*Bu_4_NSeCN *in situ*.

With multi‐gram quantities of **11** and **7** secured, the key, 2,6‐lutidine‐promoted, 5ʹ‐selenocyanate/3ʹ‐H‐phosphonate Michaelis ‐Arbuzov‐type ligation reaction [51, 52] was attempted to obtain the phosphoroselenolate dinucleotide **22** (Scheme 6). In MeCN, this provided the desired O‐cyanoethylated phosphoroselenolate dinucleotide **22** as a 1.0:0.9 mixture of two P‐diastereoisomers in 78% yield after 2.5 h of stirring at rt, as evidenced by the presence of two ^31^P NMR signals for **22** at δ 22.12 (^1^ *J*_P‐Se_ = 507.0 Hz) and 21.47 (^1^ *J*_P‐Se_ = 502.1 Hz) ppm in MeCN (D_2_O, external lock) [51, 52, 53, 66].

**SCHEME 7:**
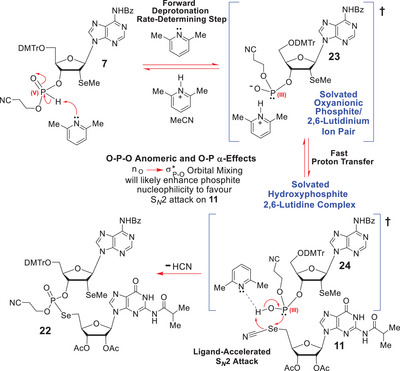
The ligand‐accelerated S*_N_* 2 mechanism that we tentatively suggest is leading to the phosphoroselenolate dinucleotide **22**, and how n_o_→σ*_P‐O_ hyperconjugative electron‐delocalization would likely enhance phosphite lone pair reactivity by substantive orbital mixing.

Mechanistically, one can envisage the coupling of **7** with **11** (Scheme 7) proceeding via a reversible, rate‐determining, 2,6‐lutidine‐promoted deprotonation of the acidic P(V)‐H‐phosphonate diester **7** to give the solvated 2,6‐lutidinium alkoxyanionic P(III) phosphite ion pair **23**, whose P(III)‐phosphite oxyanion **23** can subsequently protonate within the solvent cage to give **24** [67, 68, 69, 70]. The latter can then engage in a facile irreversible Atherton‐Todd S*_N_* 2‐type attack [68, 69, 70] on **11** (Scheme 7), which would very likely be strongly favored by the substantial α‐effect that would operate within **24**, as a result of the strong hyperconjugative n_O_→σ*_P‐O_ electron donation that would occur within **24** [71, 72, 73, 74, 75, 76, 77]. A phenomenon that would only be further magnified by ligand‐accelerated catalysis [78] as a result of complexation with the 2,6‐lutidine.

Such a mechanistic proposal would closely align with the oxy‐anionic mechanism suggested by Lewis and Spears [79] for the I_2_/H_2_O oxidation of H‐phosphonate to phosphate, under NH_3_ catalysis, where a significant *k*_H_/*k*_D_ kinetic isotope effect (1.84) was observed for the H‐phosphonate H‐atom abstraction step. The latter finding firmly established this as the rate‐determining step in this analogous multi‐step sequence. Our newly postulated mechanism also aligns favorably with the mechanistic proposal of Haake and Springs [80] on the Et_3_N‐catalysed nucleophilic addition of dimethyl H‐phosphonate to α‐chloroacetone, which revealed that such reactions are zero order in the α‐chloroacetone component, and first order in the phosphite and Et_3_N components, being second order overall. The mechanism of Scheme 7 not only accommodates both those observations, but also mechanistically aligns with the past work of Gancarz and Gancarz [67, 68, 69, 70] who kinetically defined the nature of the active nucleophile in amine‐catalysed reactions of H‐phosphonates with other electrophiles. These workers showing that such reactions typically proceed via a 1:1 associated complex of the amine and hydroxyphosphite nucleophile.

We have thus proposed that a fast protonation of the oxyanion **23** is occurring within the solvated ion cage to give **24**, on the basis of Guthrie having calculated that (EtO)_2_P‐O^−^ would abstract a proton from H_2_O with a *k* value of 1.9 × 10^8^ M^−1^ s^−1^ at 298 K [81]. The latter is extremely fast and comparable in magnitude to the rate of H‐atom abstraction of a 2,2‐dimethylvinyl radical from Bu_3_SnH in PhMe; a well‐established bimolecular process that is known to be extremely fast and facile [82].

Nonetheless, given the recent DFT studies of Keglevich et al. [83]. on the tautomerism of H‐phosphonates, and the identification of pathways that can involve three H‐phosphonates working in concert, we cannot rule out potential contributions from higher‐order mechanistic pathways.

**SCHEME 8:**
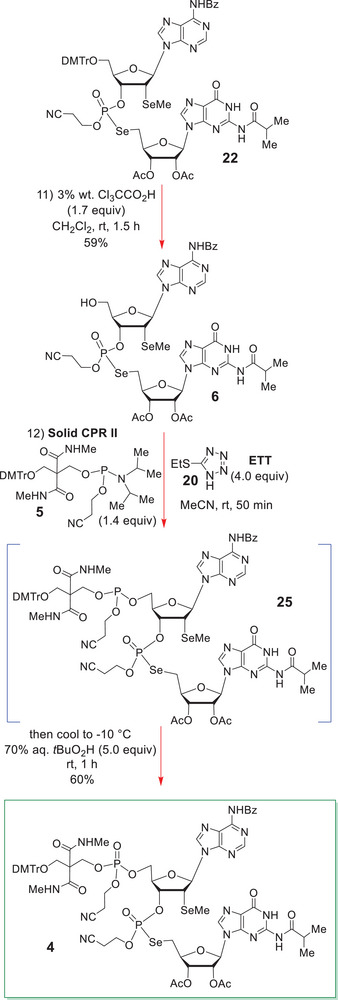
Conversion of **22** into **4** via **25**.

With efficient synthetic access to **22** now secure, we could investigate its conversion into the dinucleotide target **25** and thence **4** (Scheme 8).

A 1.5 h treatment of the dinucleotide **22** with 3% wt. trichloroacetic acid in CH_2_Cl_2_ at rt cleanly detached its DMTr group and provided the alcohol **6** as an inseparable 1:1 mixture of P‐diastereomers in 59% yield. The final step of our synthesis of the dinucleotide **4** was our conversion of its precursor alcohol **6** into the 5ʹ‐O‐phosphate **4** through use of the solid Chemical Phosphorylation Reagent II (**5**) [41, 42, 43, 44] (commonly known as solid CPR II).

**SCHEME 9:**
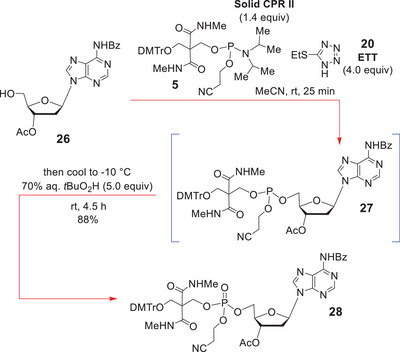
Evaluation of the solid CPR II reagent mediated 5ʹ‐O‐phosphorylation of **26** in solution‐phase.

Now although solid CPR II (**5**) has been sold commercially for well over two decades, there have been no proper experimental descriptions supplied for of its usage, and this remained the case at the very outset of this work. There were only reports of the final oligonucleotide deprotections and their detachment from solid supports by sequential treatment with 3% dichloroacetic acid in CH_2_Cl_2_ (for O‐detritylation) and conc. aqueous NH_3_ treatment at rt, or amine variants thereof. Given the lack of a proper experimental protocol [41, 42, 43, 44] for O‐phosphitylation with solid CPR II, or phosphite triester oxidation, we decided to test its viability in the model system **26** (Scheme 9).

Accordingly, **26** was reacted with solid CPR II (1.4 equiv) in MeCN (0.11 M with respect to **26**) at rt for 25 min, but instead of 1H‐tetrazole being added as the acid activator for the phosphoramidite **5**, instead, 2‐ethylthiotetrazole (ETT, 4.0 equiv) was utilized for O‐phosphitylation [45, 46, 47, 48, 49, 50], since it could potentially act as a selenomethyl protectant for the subsequent chemoselective oxidation that would be performed on **25** (see Scheme 8).

With this in mind, 5.0 equiv of 70% *aqueous t*‐butyl hydroperoxide [26] was employed for the conversion of **26** into **28** via **27**, analogously to Kore [26]. It turned out that this new combined one‐pot procedure worked very well, it affording the desired 5ʹ‐O‐phosphate **28** in 88% overall yield (Scheme 9).

Now despite this model study having carefully defined experimental conditions for effecting the desired 5ʹ‐O‐phosphorylation of **26**, it gave little real indication about whether the selenomethyl functionality of **6** and **4** would survive the *t*BuO_2_H [26] oxidation step (and remain unoxidized), but saying this, we did anticipate that if a significant excess of ETT was still present, this might improve our general prospects for leaving the Se‐functionality unoxidized.

In the event, when we applied the above general reaction procedure to **6** (Scheme 8), **4** was formed as an inseparable mixture of four P‐diastereoisomers in 60% overall yield. Proof that the 5ʹ‐O‐phosphate group had been successfully installed was provided by the four highly characteristic ^31^P NMR signals at δ ‐2.40, ‐2.48, ‐2.50 and ‐2.63 ppm in the spectrum of **4** in CDCl_3_ (see SI). Additionally, the ^13^C NMR DEPT spectrum of **4** in CDCl_3_ most definitely showed the presence of four *unoxidized* –SeMe carbons at δ 4.70, 4.65, 4.56 and 4.53 ppm. There were also four ^31^P NMR signals to be found at δ 21.02 (^1^ *J*_P‐Se_ = 513.4 Hz), 20.96 (s, ^1^ *J*_P‐Se_ = 513.4 Hz), 20.89 (^1^ *J*_P‐Se_ = 511.8 Hz), 20.85 (^1^ *J*_P‐Se_ = 511.8 Hz) ppm, which also confirmed that the phosphoroselenolate linkage had survived this prolonged 70% aqueous *t*‐butyl hydroperoxide treatment, which was by no means guaranteed at the very outset of this study [26].

**FIGURE 1:**
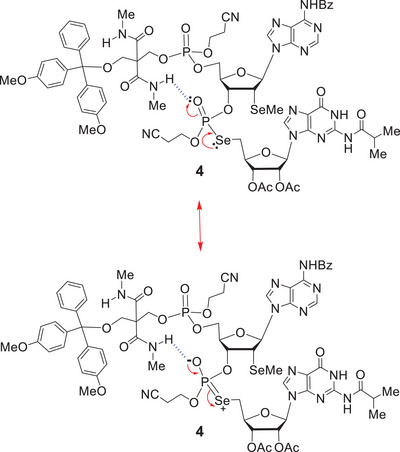
The H‐bonding that was occurring between the N‐methylamido‐NH groups and the O3ʹ‐P = O was unambiguously confirmed by the dramatic, time‐averaged, downfield chemical shifts of these amide protons, which appeared at δ 8.040, 8.035, 8.022, and 8.017 ppm respectively [87]. It is presumed that this internal H‐bonding restricts the free rotation of a significant number of the bonds within **4**, promoting the occurrence of the rotameric equilibria directly observed in the ^1^H and ^13^C NMR spectra of **4**. We believe that this rotameric effect is primarily due to the long O3ʹ‐P = O bond lengths in **4** and magnified delocalisative resonance with the Se (n_Se_→σ*_P = O_ hyperconjugation).

One particularly notable feature of the ^1^H decoupled ^13^C NMR spectrum of **4**, which deserves special comment, is the observation that it existed as interconverting rotamers on the ^13^C NMR timescale (see Figure 1), which meant that many signals were simply not visible, except through DEPT or 2D ^1^H/^13^C HSQC spectroscopy [84, 85]. The invisibility or disappearance of many signals in the ^13^C NMR spectra of molecules that are present in rotameric equilibria is a well‐known effect in NMR spectroscopy, it having previously been encountered in various solution‐phase peptide syntheses; two very good examples being the 2009 total syntheses of (+)‐azinothricin and (+)‐kettapeptin [86]. With regard to the H‐bond‐mediated restricted rotation that was seen with **4**, this was a most unexpected event, for it had not previously been observed with **28**.

However, now that it has been encountered here, such phenomena may well be routinely detected in the NMR spectra of other 3ʹ‐O‐phosphorylated systems where the solid CPR II reagent (**5**) is used for solution‐phase 5ʹ‐O‐phosphorylation.

As a result of a good synthetic route to the dinucleotide **4** having been successfully developed, we believe that we are in a very good position to complete a future total synthesis of the m7G trinucleotide cap **1**. Further work along these lines will be reported in due course, as will our application of such an m7G cap in mRNA synthesis applications *in vitro*.

Finally, given the excellent outcome of the S*_N_* 2 displacement that was conducted with the D‐*arabino*‐2ʹ‐O‐triflate ester **10** and KSeCN, we decided to evaluate the efficacy of MeCN for other S*_N_* 2 displacements of this type (Table 1), and we have found it to be an *exceptional* solvent for such reactions.

**TABLE 1:** The facile S*_N_* 2 displacement of O2ʹ‐triflate **10** in MeCN with different nucleophiles.

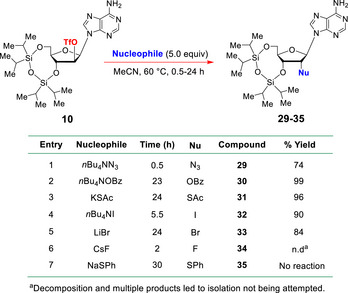

In this connection, we initially decided to examine the azide displacement shown in Table 1 in MeCN (Entry 1). This was selected because the only existing report of such an azide displacement on **10** was by Robins [54], who used thermally‐sensitive, explosive, anhydrous lithium azide to effect the S*_N_* 2 reaction in dry DMF over 0.5 h at rt. Unfortunately, dry LiN_3_ is not commercially available in solid form, and it must be prepared by the user at great personal risk. Of course, this makes the Robins [54] and Micura [88] protocols most unattractive as safe and convenient methods for the preparation of **29** [54] and its N^6^‐acetyl derivative [88].

By way of contrast, our new azidation procedure with MeCN uses commercially available *n*Bu_4_NN_3_ (5.0 equiv), which is an air‐stable, nonthermally‐sensitive solid, and the displacement reaction that we effected was complete within 0.5 h at 60°C, giving **29** cleanly as a readily observable, slower‐moving, product on TLC, isolable in 74% yield. Our new protocol is thus much more practical and far safer to execute on scale, and it is also very easy to monitor by TLC, unlike related displacements in DMF and HMPA. This is a particularly significant development, for there is growing interest in the preparation of 2ʹ‐azido‐RNAs [88, 89, 90] and 2ʹ‐azido‐m7G caps.

We next examined the S*_N_* 2 displacements of **10** [91, 92, 93, 94, 95, 96, 97] in MeCN with *n*Bu_4_NOBz, KSAc, *n*Bu_4_NI, and LiBr (Table 1, Entries 2–5). In every case, 5.0 equiv of the active nucleophile was used and the reactions were heated at 60°C. As can be seen, each of them worked very cleanly and successfully. But that is not to say that failures were not occasionally encountered using this method. This turned out to be so when CsF and NaSPh were used as nucleophiles. In the former case (Entry 6), extensive decomposition was observed, presumably due to competing O‐silyl ether cleavage. In the attempted displacement of O‐triflate **10** with PhSNa (5.0 equiv) (Entry 7), only starting material was ever recovered following 30 h of heating at 60°C. However, we would say that these two outcomes are much more representative of the exceptions rather than the norms.

Indeed, in the vast majority of cases we examined, excellent outcomes were typically obtained, and the results we have representatively gathered here have clearly paved the way for making other C2ʹ‐modified adenosine phosphoroselenolate m7G cap precursors using the same basic synthetic strategy as that used to access the dinucleotide **4**, most especially now that these important C2ʹ‐displacement breakthroughs have been made.

## 3 Conclusions

In this paper, a highly effective synthesis of the phosphoroselenolate dinucleotide m7G cap precursor **4** has been achieved from readily available commercial starting materials by a route that is 14 steps overall, and which has a longest linear sequence of 12 steps. Each of the individual reactions in this synthesis has also been performed reasonably efficiently on large scale. We further anticipate that many future chemoselective applications of **4** will prove possible in oligo‐ and poly‐nucleotide synthesis due to the protecting group arrangement it possesses.

From a methodological perspective, a number of important highlights have emerged from our synthesis of **4**. First, a new, cheap, and practical method has been developed for preparing 2ʹ‐deoxy‐2ʹ‐selenomethyl‐nucleosides based upon nucleoside 2ʹ‐O‐triflate displacement with KSeCN in MeCN, and Amosova reductive selenomethylation [60] with NaBH_4_ and MeI in THF/MeOH. This method now removes the need to use extremely expensive and noxious dimethyldiselenide alongside NaBH_4_ for this purpose.

Our synthesis of **4** has also highlighted how the *room temperature*, 2,6‐lutidine‐mediated, Michaelis‐Arbuzov‐type ligation reaction between nucleoside 5ʹ‐selenocyanates and nucleoside 3ʹ‐H‐phosphonates [51, 52] can be successfully applied in sterically hindered 3ʹ‐H‐phosphonate ribonucleotide systems in which there is a large bulky substituent stationed at C2ʹ.

This is the first time that such success has been recorded in a sterically‐demanding system of this sort. Earlier reported ligations have always been conducted with unhindered 2ʹ‐deoxy‐nucleoside 3ʹ‐H‐phosphonates. As such, the work reported here significantly expands the scope of this extremely mild and useful method for preparing oligonucleotides with novel 3ʹ,5ʹ‐phosphoroselenolate linkages [51, 52, 66].

The present paper also provides a tentative, yet powerful proposal concerning the possible mechanism of this novel Michaelis‐Arbuzov‐type reaction [51, 52, 53], which has never previously been discussed. The proposal provided here also aligns with past literature and related kinetic studies in this area.

Finally, after more than two decades since it was first introduced, we have now defined, reproducible, solution‐phase experimental conditions for the 5ʹ‐O‐phosphorylation of oligonucleotides with solid CPR II. However, even more importantly, and potentially of much greater chemical value, we have applied Kore's new chemoselective oxidation protocol (of 70% aqueous *t*BuO_2_H [26]) for the conversion of intermediary phosphite triesters into the corresponding phosphotriesters *in the presence of other readily oxidizable functionality, such as selenomethyl ethers*.

This had never previously been done using other pre‐existing technologies, which have predominantly employed I_2_/py/H_2_O for phosphite oxidation (or variants thereof). Not surprisingly, such conditions typically cause significant ‐SeMe or selenoalkyl oxidation when they are applied.

As such, our new ETT‐mediated phosphitylation with solid CPR II, allied with the Kore *in situ* 70% aqueous *t*BuO_2_H oxidation [26], represents a significant and major synthetic advance that should prove of long‐lasting value to the field. It certainly provides a far more effective and reliable alternative to the Yoshikawa POCl_3_/(MeO)_3_P = O O‐phosphorylation [98], whose harshness can frequently result in unwanted side‐reactions and low product yields in many systems [98, 99]. Indeed, when we ourselves attempted to apply the classic Yoshikawa protocol [98, 99] to the alcohol **6**, we found that it resulted in the formation of a highly complex, unprocessable, reaction mixture that contained a large number of products. So, without this new synthetic method having been introduced, the O‐phosphorylated dinucleotide **4** might not have been synthesized, particularly if other well‐known one‐pot O‐phosphorylation alternatives [26] such as bis(2‐cyano‐ethyl)‐N,N‐diisopropyl‐phosphoramidite/1H‐tetrazole (allied with 70% aqueous *t*‐BuO_2_H) [26] had failed in such a capacity. Clearly, the introduction of this new solid CPR II method offers a distinct and viable alternative to the standard technologies in such situations.

Now as already detailed, one unforeseen consequence of using the solid CPR II method for the solution‐phase 5ʹ‐O‐phosphorylation of alcohols is the potential occurrence of rotamers in oligonucleotide systems that contain a 3ʹ‐O‐phosphoroselenolate or 3ʹ‐O‐phosphate group. This is securely documented here for the very first time.

Lastly, in the present paper, we have highlighted the *exceptional* properties of MeCN as a dipolar aprotic reaction solvent for performing clean S*_N_* 2 displacements (in high yield) on the phosphonium ion derived from alcohol **21**, the iodide **12**, and the representative nucleoside 2ʹ‐O‐triflate ester **10**, which clearly benefits from the workings of the Vicinal Triflate Effect [56, 57]. The fact that each of these reactions *can now be easily monitored by TLC (due to its volatility), stands in stark contrast* to many identical or similar displacement reactions that have been conducted in dry HMPA, NMP [65] or DMF [91, 92, 93, 94, 95, 96, 97]. In our view, this significant practical advantage of MeCN makes its use extremely attractive. Indeed, when Secrist's team [97] attempted to follow the course of the corresponding triflate displacements of the 2‐chloro‐*arabino*‐adenosine analogue of **10** with LiBr and LiI in HMPA, they could only reliably estimate reaction progression through constant HPLC monitoring over 48 h. Not all organic chemistry laboratories have such equipment, and this mode of routine reaction monitoring is most inconvenient, to say the least. Additionally, even newly purchased bottles of dry HMPA and DMF can sometimes contain significant amounts of Me_2_NH, arising from gradual decomposition of the solvent upon prolonged storage, and this can significantly compromise the outcomes of displacements in some instances. These same issues can also plague S*_N_* 2 reactions run in those reaction solvents at higher temperatures [100]. Use of MeCN can side‐step all of these problems, while also frequently leading to much higher reaction yields.

In closing, we trust that other workers in the field will find the various synthetic and mechanistic contributions that have been made here, long‐lasting, useful, enabling and chemically insightful.

## Conflicts of Interest

The authors declare no conflict of interest.

## Supporting information



Experimental procedures and full characterization data including spectra are given in the Supporting Information.
**Supporting File**: chem70615‐sup‐0001‐SuppMat.pdf.
