# Supplementary material for: A Michaelis‐Arbuzov‐Type Pathway to a Protected 2ʹ‐Deoxy‐2ʹ‐Selenomethyl‐Adenosine‐3ʹ,5ʹ‐Phosphoroselenolate Guanosine Dinucleotide for Use in Modified m7G Cap Synthesis
Source: Chemistry. 2026 Jan 10;32(10):e02321. doi: 10.1002/chem.202502321 (PMC12995851; doi:10.1002/chem.202502321)

(346 Pages Inclusive of Cover Pages)

Table of Contents

Part A. Experimental Section

S1. General Experimental Protocols 6

S2. Experimental Procedures 7

Compound **14** 7

Compound **10** 8

Compound **9** 9

Compound **15** 10

Compound **16** 11

Compound **17** 12

Compound **18** 13

Compound **8** 14

Compound **7** 15

Compound **12** 15

Compound **11** 16

Compound **22** 17

Compound **6** 18

Compound **4** 20

Compound **28** 22

Compound **29** 24

Compound **30** 25

Compound **31** 26

Compound **32** 27

Compound **33** 28

Part B. Copies of Spectra

S3. Copies of NMR Spectra of All Compounds Reported 30

Compound **14** 31

Compound **10** 44

Compound **9** 58

Compound **15** 71

Compound **16** 94

Compound **17** 108

Compound **18** 122

Compound **8** 145

Compound **7** 151

Compound **12** 154

Compound **11** 167

Compound **22** 181

Compound **6** 184

Compound **4** 214

Compound **28** 250

Compound **29** 257

Compound **30** 270

Compound **31** 285

Compound **32** 299

Compound **33** 314

S4. Copies of High-Resolution Mass Spectra of Compounds Reported 326

Part A. Experimental Section

S1. General Experimental Protocols

Unless stated otherwise, all reactions were run in dry solvents under an N_2 atmosphere. As regards the THF, CH_2Cl_2 and pyridine, these were all freshly distilled from CaH_2 under an N_2 atmosphere prior to use. Dry PhMe was used as supplied from Sigma-Aldrich. All dry solvents were withdrawn with a dry N_2 flushed syringe fitted with a Luer-locked needle. Solid Chemical Phosphorylation Reagent II (also known as solid CPR II) **5** was purchased from commercial sources and used as supplied. SiO_2 flash chromatography was performed on Fluorochem silica gel 60Å. The petrol used was the 40-60 °C b.p. fraction; it was distilled prior to use for chromatography. HPLC grade EtOAc was used for all chromatographic purifications. TLC analysis and preparative TLC were performed on Merck glass-backed TLC plates coated with silica gel 60 F_{254}. Plates were typically stained with either anisaldehyde/H_2SO_4 or phosphomolybdic acids stains. NMR analyses were carried out on either a Bruker Avance III HD Ascend 600 instrument operating at a frequency of 600.1337 MHz or on a Bruker 400 Ultrashield Plus instrument operating at a resonance frequency of 400.1100 MHz. All ^{1}H NMR spectra in $CDCl_3$ were referenced upon tetramethylsilane (TMS) at δ 0.00 ppm or the δ 77.00 ppm triplet for ^{13}C spectra. All ^{19}F or ^{31}P NMR spectra were recorded in deuterated NMR solvents and were internally machine referenced with respect to the deuterium lock frequency of the deuterated NMR solvent used. As such, the chemical shifts reported are considered absolute values. To analyse and process the NMR spectra, TopSpin™ software was used. The chemical shifts are reported in parts per million (ppm) and coupling constants (*J*) are reported in Hertz (Hz). ^{1}H NMR spectra are reported as follows: δ/ppm (multiplicity, coupling constant, number of protons, assignment of peak). The multiplicities are abbreviated as follows: s = singlet, d = doublet, t = triplet, q = quartet, m = multiplet. High-resolution mass spectra (HRMS) were recorded by Analytical Services and Environmental Projects (ASEP) at Queen's University Belfast on a Waters LCT Premier ToF mass spectrometer using the electrospray ionization (ESI) technique. All HRMS were recorded by Mr Conor McGrann of the QUB School of Chemistry and Chemical Engineering.

S2. Experimental Procedures

3′,5′-*O*-(1,1,3,3-Tetraisopropyl-1,3-disiloxanyl)-β-D-*arabino*-furanosyl)adenine (14)

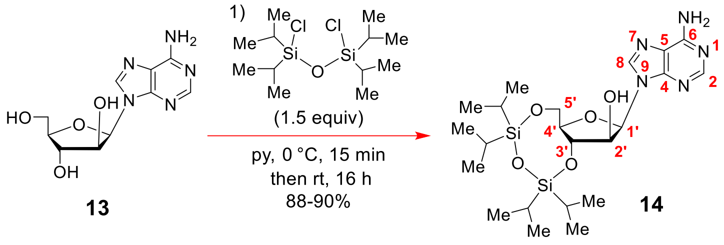

Prior to use, commercially supplied 1-β-D-*arabino*-furanosyladenine **13** (which exists as a hydrate) was dried by azeotropic distillation from dry, freshly-distilled pyridine, several times, on a rotary evaporator connected to a *high vacuum pump* and a dry ice/acetone cooling condenser. When all of the pyridine had been removed at 40-50 °C under reduced pressure, the pre-weighed flask containing the now dry 1-β-D-*arabino*-furanosyladenine **13** was fitted with a 3-way tap and connected to the high vacuum for 4-5 h. The vacuum was ultimately released with N_2 from a fitted-balloon; with the 3-way-tap finally being closed to preserve the N_2 atmosphere inside the flask. The balloon was removed and the pre-weighed 3-way tap and reaction flask, now containing dry **13** under N_2, was accurately weighed and used for the synthetic conversion of **13** into **14**.

To a vigorously stirred 0 °C solution of this *pre-dried* 9-(β-D-*arabino*-furanosyl)adenine **13** (5.00 g, 18.71 mmol) in anhydrous pyridine (100.00 mL) under N_2 was slowly added 1,3-dichloro-1,1,3,3-tetraisopropyldisiloxane (8.98 mL, 28.06 mmol, 1.5 equiv) dropwise via syringe over 15 min. The solution was stirred at rt overnight, whereafter TLC indicated that a single faster-moving product had formed. The reaction mixture was quenched by the careful addition of ice-cold H_2O (5 mL), and the reaction solvent removed by concentration *in vacuo* on a rotary evaporator. The crude residue was thereafter extracted with EtOAc (200 mL) and washed successively with H_2O (200 mL), saturated aqueous $NaHCO_3$ (100 mL) and brine (100 mL). The organic layer was then dried over $MgSO_4$, filtered and concentrated *in vacuo*. The crude residue was initially purified by SiO_2 flash chromatography with petrol/EtOAc (1:2). However, a second SiO_2 flash chromatographic purification with CH_2Cl_2/MeOH (gradient elution 20:1→15:1) led to highly pure product **14** (8.54 g, 90%) as an amorphous white solid.

^{1}H NMR of **14** (600.13 MHz, $CDCl_3$): δ 8.14 (s, 1H, H2), 8.12 (s, 1H, H8), 6.41 (s, 1H, -NH_2), 6.21 (d, *J* = 6.0 Hz, 1H, H1′), 5.72 (br s, 2H, -NH_2), 4.65 (t, *J* = 7.2 Hz, 6.0 Hz, 1H, H2′), 4.58 (t, *J* = 7.8 Hz, 1H, H3′), 4.07 (dd, *J* = 13.2 Hz, 3.6 Hz, 1H, H5′a), 4.04 (dd, 1H, *J* = 13.2 Hz, 3.6 Hz, H5′b), 3.85 (dt, *J* = 7.8 Hz, 3.0 Hz, 1H, H4′), 2.60 (broad s, 1H, OH), 1.20-0.90 (complex m, 28H, -O-($i$$Pr_2$-Si-O-Si-$i$$Pr_2$)-O-) ppm.

^{13}C NMR of **14** (150.92 MHz, $CDCl_3$): δ 155.6 (C2), 152.5 (C6), 149.3 (C4), 140.0 (C8), 119.5 (C5), 83.9 (C1′), 81.2 (C4′), 76.5 (C2′), 74.4 (C3′), 61.4 (C5′), 17.5 (Me), 17.4 (Me), 17.3 (2 x Me), 17.0 (Me) 16.93 (2 x Me), 16.89 (Me), 13.5 (Si$\underline{C}$H), 13.0 (Si$\underline{C}$H), 12.9 (Si$\underline{C}$H), 12.4 (Si$\underline{C}$H) ppm.

HRMS (ESI) Calcd. for $[C_{22}H_{39}N_5O_5Si_2+H]^+$: 510.2568. Found: 510.2566.

3′,5′-O-(1,1,3,3-Tetraisopropyl-1,3-disiloxanyl)-2-O-trifluoromethanesulfonyl-β-D-*arabino*-furanosyl)adenine (10)

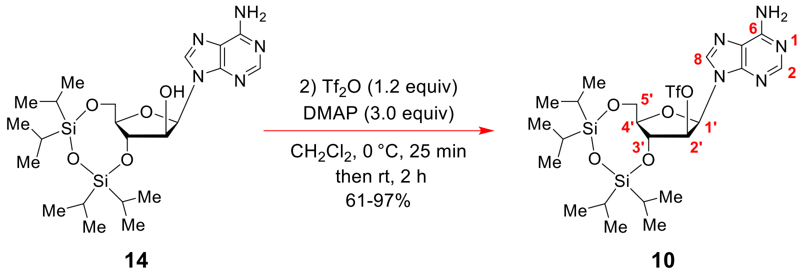

To a stirred 0 °C solution of **14** (1.52 g, 2.98 mmol) in dry CH_2Cl_2 (15.69 mL) was added 4-dimethylaminopyridine (1.09 g, 8.95 mmol, 3.0 equiv) in one portion and this was followed by a dropwise addition of trifluoromethanesulfonic anhydride (0.60 mL, 3.58 mmol, 1.2 equiv) over 2 min. After 23 min, the ice bath was removed and the reactants and allowed to warm to rt, being stirred for 2 h once the cooling bath had been removed. To work up the reaction mixture, it was treated with 1% aqueous AcOH (100 mL) and extracted thereafter with CH_2Cl_2 (2 x 75 mL). The combined organic extracts were washed with saturated aqueous $NaHCO_3$ (50 mL), H_2O (50 mL) and brine (50 mL). The organic layer was dried over $MgSO_4$, filtered and concentrated *in vacuo*. The crude residue was purified by gradient elution SiO_2 flash chromatography with petrol/EtOAc (3:1→2:1→1:1) to give **10** (1.86 g, 97%) as a runny oil which would foam when dried further on vacuum pump.

^{1}H NMR of **10** (600.13 MHz, $CDCl_3$): δ 8.33 (s, 1H, H2), 7.93 (s, 1H, H8), 6.40 (d, J = 6.0 Hz, 1H, H1′), 5.89 (s, 2H, -NH_2), 5.48 (dd, J = 6.6 Hz, 6.0 Hz, 1H, H2′), 5.39 (t, J = 7.2 Hz, 6.6 Hz, 1H, H3′), 4.22 (dd, J = 12.6 Hz, 6.6 Hz, 1H, H5′a), 4.09 (dd, J = 12.6 Hz, 3.6 Hz, 1H, H5′b), 3.96 (ddd, J = 7.8 Hz, 7.2 Hz, 6.6 Hz, 3.6 Hz, 1H, H4′) 1.30-0.86 (complex m, 28 H, -O-($i$$Pr_2$-Si-O-Si-$i$$Pr_2$)-O-) ppm.

^{13}C NMR of **10** (150.92 MHz, $CDCl_3$): δ 155.6 (C6), 153.2 (C2), 149.6 (C4), 139.6 (C8), 119.8 (C5), 118.2 (q, $J_{C\text{-}F}$ = 319.9 Hz, CF_3), 88.4 (C2′), 81.2 (C1′), 80.8 (C4′), 74.4 (C3′), 62.1 (C5′),

17.4 (Me), 17.33 (Me), 17.29 (Me), 17.25 (Me), 16.83 (Me), 16.75 (Me), 16.73 (Me), 16.71 (Me), 13.2 (SiCH), 13.1 (SiCH), 13.0 (SiCH), 12.6 (SiCH) ppm.

^{19}F NMR of **10** (564.6 MHz, $CDCl_3$): δ -74.39 ppm.

LRMS (ESI) Calcd. for $[C_{23}H_{38}F_3N_5O_7SSi_2+H]^+$: 642.2061. Found: 642.1479.

2′-Deoxy-2′-selenocyano-3′,5′-O-(1,1,3,3-tetraisopropyl-1,3-disiloxanyl)adenosine (9)

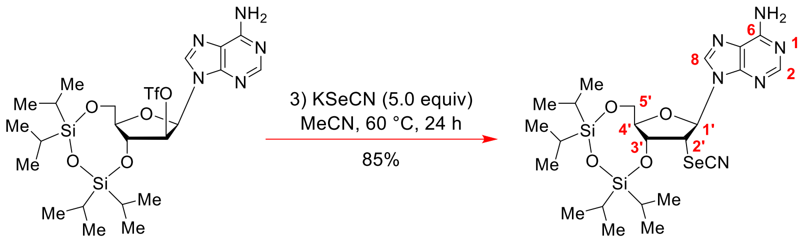

10 **9**

To a stirred solution of **10** (0.50 g, 0.78 mmol) in dry acetonitrile (8.03 mL), in a 50 mL pear-shaped flask, was added potassium selenocyanate (0.56 g, 3.90 mmol, 5.0 equiv) under N_2. The reaction mixture was heated at 60 °C for 24 h, whereafter it was diluted with CH_2Cl_2 (5 mL) and rotary evaporated to dryness. The crude residue was then treated with H_2O (100 mL) and extracted with CH_2Cl_2 (70 mL x 3), before the combined organic extracts were washed with brine (50 mL). The organic layer was then separated, dried over $MgSO_4$, filtered and concentrated *in vacuo*. The crude residue was purified by gradient elution SiO_2 flash chromatography with petrol/EtOAc (2:1→1:1) to give **9**[44a] (0.40 g, 85%), as a white/cream solid.

^{1}H NMR of **9** (600.13 MHz, $CDCl_3$): δ 8.26 (s, 1H, H2), 7.90 (s, 1H, H8), 6.47 (d, *J* = 3.0 Hz, 1H, H1′), 5.89 (s, 2H, -NH_2), 5.59 (dd, *J* = 7.2 Hz, 6.6 Hz, 1H, H3′), 5.31 (dd, *J* = 7.8 Hz, 3.0 Hz, 1H, H2′), 4.19 (ddd, *J* = 7.2 Hz, 6.6 Hz, 4.2 Hz, 1H, H4′), 4.09 (dd, *J* = 12.0 Hz, 7.2 Hz, 1H, H5′a), 4.06 (dd, *J* = 12.0 Hz, 4.2 Hz, 1H, H5′b), 1.20-0.90 (complex m, 28 H, -O-(*i*Pr_2-Si-O-Si-*i*Pr_2)-O-) ppm.

^{13}C NMR of **9** (150.92 MHz, $CDCl_3$): δ 155.7 (C6), 153.0 (C2), 149.0 (C4), 139.8 (C8), 120.7 (C5), 101.8 (SeCN), 90.5 (C1′), 84.4 (C4′), 72.8 (C3′), 63.0 (C5′), 52.7 (C2′), 17.4 (Me), 17.3 (2 x Me), 17.21 (Me), 17.15 (Me), 17.0 (Me), 16.87 (Me), 16.84 (Me), 13.2 (SiCH), 13.1 (SiCH), 12.7 (2 x SiCH) ppm.

^{77}Se NMR of **9** (114.5 MHz, $CDCl_3$): δ 238.85 ppm.

HRMS (ESI) Calcd. for $[C_{23}H_{38}N_6O_4SeSi_2+H]^+$: 599.1737. Found: 599.1736.

2′-Deoxy-2′-selenomethyl-3′,5′-O-(1,1,3,3-tetraisopropyl-1,3-disiloxanyl)adenosine (15)

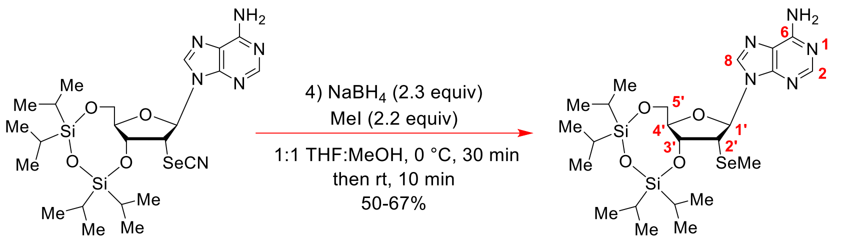

9 15

To a stirred solution of **9** (4.00 g, 6.69 mmol) in dry THF (6.70 mL) and dry MeOH (6.70 mL), in a 100 mL round-bottomed flask, was added MeI (0.92 mL, 14.72 mmol, 2.2 equiv) under N_2. $NaBH_4$ (0.58 g, 15.39 mmol, 2.3 equiv) was added in small portions and upon each addition fizzing was observed. The reaction mixture was stirred at 0 °C for 30 min and then left to warm to rt for 10 min, whereafter the crude residue was then treated with H_2O (100 mL) and extracted with $CHCl_3$ (100 mL x 3). The combined organic extracts were washed with a final time with H_2O (100 mL). The organic layer was then separated, dried over Na_2SO_4, filtered and concentrated *in vacuo*. The crude residue was purified by gradient elution SiO_2 flash chromatography with CH_2Cl_2/MeOH (60:1→40:1) to give **15** (2.64 g, 67%), as a white/cream solid.

^{1}H NMR of **15** (400.11 MHz, $CDCl_3$): δ 8.33 (s, 1H, H2), 8.04 (s, 1H, H8), 6.28 (d, J = 4.0 Hz, 1H, H1′), 5.84 (s, 2H, -NH_2), 4.91 (t, J = 6.8 Hz, 1H, H3′), 4.18 (complex m, 1H, H4′), 4.12 (dd, J = 6.8 Hz, 4.0 Hz, 1H, H2′), 4.09-4.08 (m, 2H, H5′a, H5′b), 1.96 (s, $J_{77Se\text{-}CH3}$ = 11.2 Hz, 3H, SeMe), 1.15-0.95 (complex m, 28 H, -O-($i$$Pr_2$-Si-O-Si-$i$$Pr_2$)-O-) ppm.

^{13}C NMR of **15** (100.61 MHz, $CDCl_3$): δ 155.5 (C6), 153.1 (C2), 149.4 (C4), 139.0 (C8), 120.3 (C5), 90.1 (C1′), 84.7 (C4′), 71.8 (C3′), 62.0 (C5′), 47.0 ($J_{77Se\text{-}13C2'}$ = 77.1 Hz, C2′), 17.48 (Me), 17.37 (Me), 17.33 (Me), 17.31 (Me), 17.15 (Me), 17.02 (Me), 17.00 (Me), 16.90 (Me), 13.5 (Si$\underline{C}$H), 13.2 (Si$\underline{C}$H), 13.0 (Si$\underline{C}$H), 12.7 (Si$\underline{C}$H), 3.3 ($J_{77Se\text{-}13CH3'}$ = 60.4 Hz, SeMe) ppm.

LRMS (ESI) Calcd. for $[C_{23}H_{41}N_5O_4SeSi_2+H]^+$: 588.1941. Found: 588.1693.

N^6-Benzoyl-2′-deoxy-2′-selenomethyl-3′,5′-O-(1,1,3,3-tetraisopropyl-1,3-disiloxanyl)-adenosine (16)

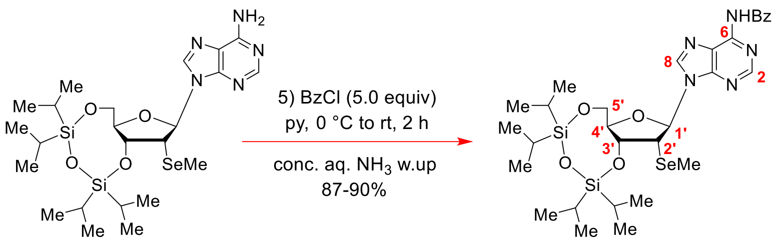

15 **16**

To a stirred 0 °C solution of **15** (2.45 g, 4.18 mmol) in dry pyridine (41.76 mL) in a 100 mL round-bottomed flask was added benzoyl chloride (2.43 mL, 20.88 mmol, 5.0 equiv) dropwise, under N_2. After the addition was complete, the ice bath was removed, and the reaction mixture was stirred at rt for 2 h. It was then cooled with an ice bath before cold H_2O (8.2 mL) and aqueous NH_4OH (35% ammonia solution) (8.2 mL) was added. After 25 min stirring, the crude residue was then extracted with EtOAc (100 mL x 3) and washed successively with saturated aqueous $NaHCO_3$ (200 mL) and brine (100 mL). The organic layer was then separated, dried over Na_2SO_4, filtered and concentrated *in vacuo*. The crude residue was purified by SiO_2 flash chromatography with CH_2Cl_2/MeOH (150:1) to give **16** (2.59 g, 90%), as a white/cream solid.

^{1}H NMR of **16** (400.11 MHz, $CDCl_3$): δ 9.18 (s, 1H, BzNH), 8.78 (s, 1H, H2), 8.25 (s, 1H, H8), 8.02 (d, J = 7.6 Hz, 2H, 2 *ortho*-protons of Bz), 7.60 (dd, J = 7.6 Hz, 7.2 Hz, 1H, *para*-proton of Bz), 7.51 (dd, J = 8.0 Hz, 7.2 Hz, 2H, 2 *meta*-protons of Bz), 6.36 (d, J = 3.6 Hz, 1H, H1′), 4.92 (dd, J = 7.2 Hz, 6.8 Hz, 1H, H3′), 4.20 (m, 1H, H4′), 4.13 (dd, J = 6.8 Hz, 3.6 Hz, 1H, H2′), 4.12-4.03 (complex m, 2H, H5′a, H5′b), 2.01 (s, $J_{77Se\text{-}CH3}$ = 11.2 Hz, 3H, SeMe), 1.14-0.96 (complex m, 28 H, -O-(iPr$_2$-Si-O-Si-iPr$_2$)-O-) ppm.

^{13}C NMR of **16** (100.61 MHz, $CDCl_3$): δ 164.6 (C=O of BzNH), 152.7 (C2), 151.1 (C6), 149.6 (C4), 141.3 (C8), 133.6 (quaternary carbon of BzNH), 132.7 (*para*-carbon of Bz), 128.8 (*meta*-carbons of Bz), 127.8 (*ortho*-carbons of Bz), 123.6 (C5), 90.1 (C1′), 84.6 (C4′), 71.4 (C3′), 61.6 (C5′), 47.1 ($J_{77Se\text{-}13C2'}$ = 76.5 Hz, C2′), 17.44 (Me), 17.34 (Me), 17.28 (2 x Me), 17.10 (Me), 16.96 (2 x Me), 16.86 (Me), 13.4 (Si$\underline{C}$H), 13.1 (Si$\underline{C}$H), 12.9 (Si$\underline{C}$H), 12.6 (Si$\underline{C}$H), 3.4 ($J_{77Se\text{-}13CH3}$ = 60.4 Hz, SeMe) ppm.

HRMS (ESI) Calcd. for $[C_{30}H_{45}N_5O_5{}^{80}SeSi_2+H]^+$: 692.2205. Found: 692.2183.

N^6-Benzoyl-2′-deoxy-2′-selenomethyl-adenosine (17)

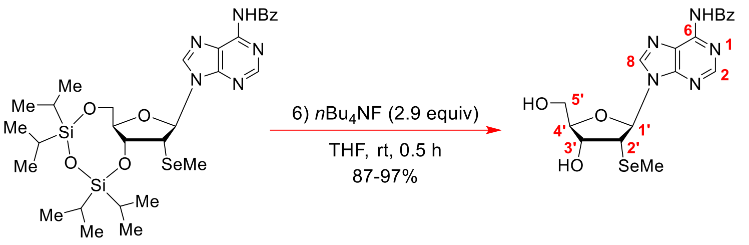

To a stirred rt solution of **16** (2.66 g, 3.85 mmol) in dry THF (16.74 mL) in a 100 mL round-bottomed flask was added tetra-*n*-butylammonium fluoride (1M solution in THF, 11.17 mL, 11.17 mmol, 2.9 equiv) dropwise maintaining an N_2 atmosphere. The reaction mixture was stirred at rt for 30 min, and then extracted with $CHCl_3$ (100 mL x 3) and washed with H_2O (100 mL). The organic layer was then separated, dried over Na_2SO_4, filtered and concentrated *in vacuo*. The crude residue was purified by gradient elution SiO_2 flash chromatography with CH_2Cl_2/MeOH (50:1→40:1→30:1) to give **17** (1.56 g, 90%), as a white solid.

^{1}H NMR of **17** (400.11 MHz, $CDCl_3$): δ 9.32 (s, 1H, BzNH), 8.79 (s, 1H, H2), 8.10 (s, 1H, H8), 8.04 (d, 2H, 2 *ortho*-protons of Bz), 7.62 (tt, J = 7.2 Hz, 1.2 Hz, 1H, *para*-proton of Bz), 7.53 (t, J = 7.2 Hz, 2H, 2 *meta*-protons of Bz), 6.04 (broad dd, J = 12.0 Hz, 1.6 Hz, 1H, 5′-OH), 6.00 (d, J = 9.6 Hz, 1H, H1′), 4.61 (d, J = 4.4 Hz, 1H, H3′), 4.38 (s, 1H, H4′), 4.24 (dd, J = 9.6 Hz, 4.4 Hz, 1H, H2′), 4.02 (dt, J = 12.8 Hz, 1.6 Hz, 1H, H5′a), 3.81 (td, J = 12.8 Hz, 1.6 Hz, 1H, H5′b), 3.49 (s, 1H, 3’-OH), 1.73 (s, $J_{77Se\text{-}CH3}$ = 10.8 Hz, 3H, SeMe) ppm.

^{13}C NMR of **17** (100.61 MHz, $CDCl_3$): δ 164.6 (C=O of BzNH), 152.2 (C2), 150.45 (C6), 150.41 (C4), 143.0 (C8), 133.4 (quaternary carbon of Bz), 133.0 (*para*-carbon of Bz), 128.9 (*meta*-carbons of Bz), 127.9 (*ortho*-carbons of Bz), 124.4 (C5), 92.5 ($^3J_{Se\text{-}C1'}$ = 19.1 Hz, C1′), 88.3 (C4′), 73.4 (C3′), 63.4 (C5′), 49.9 ($J_{77Se\text{-}13C2'}$ = 80.5 Hz, C2′), 4.5 ($J_{77Se\text{-}13CH3}$ = 58.4 Hz, SeMe) ppm.

HRMS (ESI) Calcd. for $[C_{18}H_{19}N_5O_4Se+H]^+$: 450.0681. Found: 450.0670.

2′-Deoxy-5′-O-(4,4′-dimethoxytrityl)-2′-selenomethyl-adenosine (18)

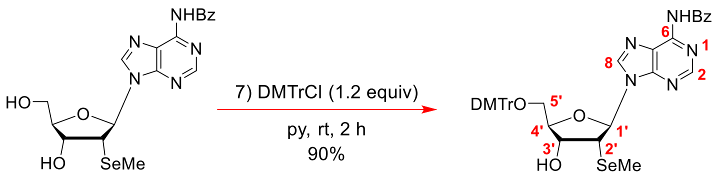

To a stirred solution of **17** (1.56 g, 3.48 mmol) in dry pyridine (11.59 mL) in a 50 mL round-bottomed flask, was added 4,4′-dimethoxytrityl chloride (1.41 g, 4.18 mmol, 1.2 equiv) under N_2. The reaction mixture was stirred at rt for 2 h and then the crude residue was extracted with CH_2Cl_2 (100 mL x 3) and washed successively with saturated aqueous $NaHCO_3$ (200 mL). The organic layer was then separated, dried over Na_2SO_4, filtered and concentrated *in vacuo*. The crude residue was purified by gradient elution SiO_2 flash chromatography with CH_2Cl_2/EtOAc (neat CH_2Cl_2→8:1→6:1→2:1) to give **18** (2.35 g, 90%).

^{1}H NMR of **18** (400.11 MHz, $CDCl_3$): δ 9.14 (broad s, 1H, BzNH), 8.68 (s, 1H, H2), 8.16 (s, 1H, H8), 8.03 (d, *J* = 7.2 Hz, 2H, 2 *ortho*-protons of Bz), 7.60 (tt, *J* = 7.6 Hz, 1.2 Hz, 1H, *para*-proton of Bz), 7.52 (dd, *J* = 7.6 Hz, 7.2 Hz, 2H, 2 *meta*-protons of Bz), 7.42 (d, *J* = 7.2 Hz, 2H, 2 *ortho*-protons of Ph in DMTr), 7.31 (d, *J* = 8.8 Hz, 4H, 4 *meta*-protons of PMP), 7.30-7.18 (m, 3H, 2 *meta*-protons and the *para*-proton of Ph in DMTr), 6.80 (d, *J* = 8.4 Hz, 4H, 4 *ortho*-protons of PMP), 6.23 (d, *J* = 8.4 Hz, 1H, H1′), 4.50 (d, *J* = 5.2 Hz, 1.6 Hz, 1H, H3′), 4.41 (dd, *J* = 8.4 Hz, 5.2 Hz, 1H, H2′), 4.32 (ddd, *J* = 4.4 Hz, 2.0 Hz, 1H, H4′), 3.77 (s, 6H, 2 x MeO), 3.75 (broad d, *J* = 1.6 Hz, 1H, 3′-OH), 3.51 (dd, *J* = 10.4 Hz, 4.4 Hz, 1H, H5′a), 3.42 (dd, *J* = 10.4 Hz, 4.0 Hz, 1H, H5′b), 1.91 (s, $J_{77Se\text{-}CH3}$= 10.8 Hz, 3H, SeMe) ppm. (**N.B.** PMP = 4-MeO-C_6H_4 of DMTr).

^{13}C NMR of **18** (100.61 MHz, $CDCl_3$): δ 164.6 (broad C=O of BzNH), 158.6 (2 x quaternary carbons of PMP), 152.6 (C2), 151.9 (C6), 149.6 (C4), 144.4 (2 x quaternary carbons of PMP), 142.1 (C8), 135.5 (quaternary carbon of Ph in DMTr), 133.6 (quaternary carbons of Bz), 132.8 (*para*-carbon of Bz), 130.03-130.02 (4 *meta*-carbons of PMP), 128.8 (2 *meta*-carbons of Bz), 128.1 (2 *ortho*-carbons of Bz), 127.9 (2 *meta*- and 2 *ortho*-carbons of Ph of DMTr), 127.0 (1 *para*-carbon of Ph of DMTr), 123.4 (C5), 113.2 (4 *ortho*-carbons of PMP), 88.9 (C1′), 86.7 (C4′), 85.1 (exocyclic quaternary carbon of DMTr), 72.6 (C3′), 63.6 (C5′), 55.21 (OMe), 55.14 (OMe), 48.8 (C2′), 4.6 ($J_{77Se\text{-}13\underline{C}H3}$ = 61.4 Hz, SeMe) ppm. (**N.B.** PMP = 4-MeO-C_6H_4 of DMTr).

HRMS (ESI) Calcd. for $[C_{39}H_{38}N_5O_6Se+H]^+$: 752.1987. Found: 752.2040.

N⁶-Benzoyl-2′-deoxy-5′-O-(4,4′-dimethoxytrityl)-2′-selenomethyl-adenosinyl-3′-(2-cyano-ethyl-N,N-diisopropyl)phosphoramidite (8)

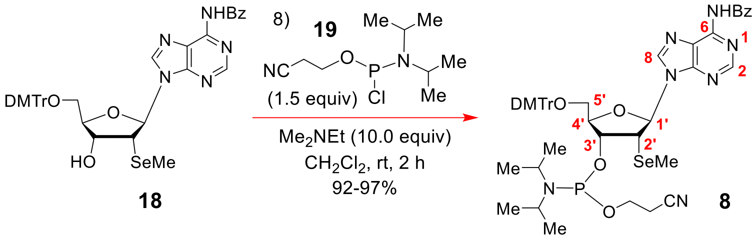

To a 150 mL pear-shaped flask charged with alcohol **18** (1.85 g, 2.46 mmol) and dry CH_2Cl_2 (22.40 mL) under N_2 was added Me_2NEt (2.67 mL, 24.64 mmol, 10.0 equiv) dropwise. Using a gas-tight syringe 2-cyanoethyl *N,N*-diisopropylchlorophosphoramidite (0.82 mL, 3.70 mmol, 1.5 equiv) was added dropwise over 30 sec. The reaction mixture was stirred at rt for 1 h 40 min. The reaction mixture was then quenched with dry MeOH (1 mL) and left to stir for 20 min. The crude residue was then extracted with CH_2Cl_2 (100 mL x 3) and washed with saturated aqueous $NaHCO_3$ (200 mL). The organic layer was then separated, dried over Na_2SO_4, filtered and concentrated *in vacuo*. The crude residue was instead precipitated using EtOAc (3.5 mL) initially to dissolve, hexane (4 mL) to precipitate, and more EtOAc (0.2 mL) to redissolve. This solution was then added dropwise to a vigorously stirred solution of hexane (190 mL) in a 500 mL conical flask. The solid precipitated out in the hexane following this addition and made the previously clear solution turn cloudy. The solid by-product was filtered off using a size 4 sinter funnel, washed with hexane (3 x 10 mL), and the resulting filtrate concentrated *in vacuo* to give crude **8** (2.28 g, 97%), which was used directly without further purification for the next step.

^{31}P NMR of **8** (161.98 MHz, $CDCl_3$): δ 151.11 and 149.52 ppm (both s, 2 x P-diastereoisomers). The crude, partially-purified, NMR sample also contained a small amount of a reagent-derived P(V) phosphoramidate impurity that resonated at δ 14.15 (s) ppm as a minor by-product. The fact that it only gave rise to a single peak showed that it was not nucleoside-related. The ^{31}P spectra of **8** in MeCN with an external D_2O insert has also been provided in Part B of the SI for useful comparison.

HRMS (ESI) for **8** Calcd. for $[C_{48}H_{54}N_7O_7PSe+H]^+$: 952.3066. Found: 952.3110.

Important Note: The crude residue of **8** must *not* be purified by SiO_2 flash chromatography due to the fact that **8** decomposes on normal commercial silica gel; Et_3N-deactivated SiO_2 is also of little value here, since it cleaves off the cyanoethyl group; the above partial purification method must therefore be always adhered to in this instance.

N^6-Benzoyl-2′-Deoxy-5′-O-(4,4’-dimethoxytrityl)-2′-selenomethyl-adenosinyl-3′-(2-cyanoethyl)-H-phosphonate (7)

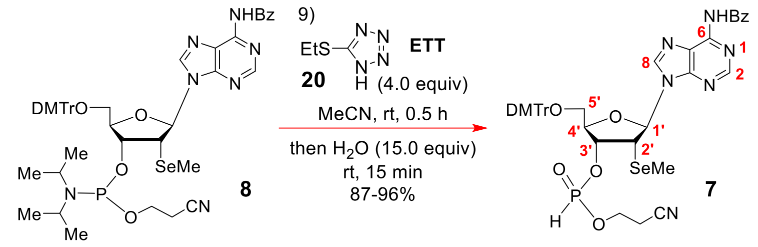

To a stirred solution of **8** (0.44 g, 0.46 mmol) in dry MeCN (3.30 mL) in a 25 mL pear-shaped flask, was added in one portion 5-(ethylthio)-1H-tetrazole (ETT) (**20**) (0.24 g, 1.85 mmol, 4.0 equiv) under N_2. The reaction mixture was stirred at rt for 0.5 h before H_2O (0.13 mL, 6.94 mmol, 15.0 equiv) was added and the reaction stirred for a further 15 min. The crude residue was then extracted with EtOAc (2 x 100 mL) and washed successively with saturated aqueous $NaHCO_3$ (200 mL) and brine (100 mL). The organic layer was then separated, dried over Na_2SO_4, filtered and concentrated *in vacuo*. The crude residue of **7** (0.37 g, 92%) was not purified by SiO_2 flash chromatography as it decomposed on silica gel.

^{31}P NMR of **7** (161.98 MHz, D_2O External Lock, MeCN): 8.36 and 8.28 ppm (both s, 2 x P-diastereoisomers).

HRMS (ESI) Calcd. for $[C_{42}H_{41}N_6O_8PSe+H]^+$: 869.1967. Found: 869.1985.

2′,3′-Di-O-acetyl-5′-deoxy-5′-iodo-N^2-isobutyramido-guanosine (12)

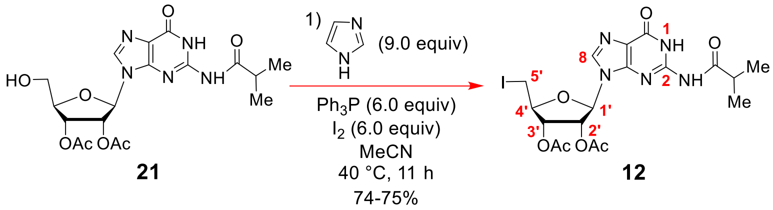

To a vigorously stirred rt solution of N^2(*i*Bu)-2’,3’-di-O-acetyl guanosine **21** (1.50 g, 3.43 mmol) *<u>in a 100 mL round-bottomed flask</u>* in dry MeCN (27.43 mL) under N_2 was successively added imidazole (2.10 g, 30.86 mmol, 9.0 equiv) and Ph_3P (5.40 g, 20.58 mmol, 6.0 equiv) in single portions. After 5 min of *vigorous stirring*, I_2 (5.22 g, 20.58 mmol, 6.0 equiv) was added portionwise over several minutes. Heat was given off following each I_2 addition and a golden

yellow solid gradually precipitated from the solution. When complete, the reaction mixture was transferred to an oil bath and heated at 40 °C with stirring for 10.5 h. It was then diluted with CH_2Cl_2 (200 mL) and saturated aqueous $Na_2S_2O_3$ (150 mL). The organic layer was separated and washed with saturated aqueous $Na_2S_2O_3$ (3 x 50 mL) and brine (3 x 50 mL). The organic layer was further separated, dried over Na_2SO_4, filtered and concentrated *in vacuo*. The crude residue was purified by gradient elution SiO_2 flash chromatography with CH_2Cl_2/EtOAc (6:1→4:1→1:1) as the eluent to give the iodide **12** (1.40 g, 75%). An analytical sample was obtained after carrying out a second SiO_2 flash chromatography with CH_2Cl_2/MeOH (40:1).

^{1}H NMR of **12** (399.91 MHz, $CDCl_3$): δ 12.29 (s, 1H, C2-NH), 10.01 (s, 1H, N(1)-H), 7.87 (s, 1H, H8), 6.12 (apparent triplet, J = 5.2 Hz, 5.2 Hz, 1H, H2′), 5.94 (d, J = 4.8 Hz, 1H, H1′), 5.72 (dd, J = 5.6 Hz, 5.2 Hz, 1H, H3′), 4.20 (dd, J = 11.2 Hz, 5.6 Hz, 1H, H4′), 3.43 (dd, J = 11.2 Hz, 5.2 Hz, 1H, H5′a), 3.39 (dd, J = 10.8 Hz, 6.0 Hz, 1H, H5′b), 2.82 (septet, J = 6.8 Hz, 1H, CH of *i*Bu), 2.11 (s, 3H, OAc), 2.09 (s, 3H, OAc), 1.27 (d, J = 6.8 Hz, 3H, Me of *i*Bu), 1.23 (d, J = 6.8 Hz, 3H, Me of *i*Bu) ppm.

^{13}C NMR of **12** (100.61 MHz, $CDCl_3$): δ 179.5 (C2), 169.7 (C=O of OAc), 169.4 (C=O of OAc), 155.6 (C=O of *i*Bu and C6 overlapped), 148.1 (C4), 138.7 (C8), 121.9 (C5), 86.7 (C1′), 80.8 (C4′), 73.1 (C3′), 72.5 (C2′), 36.2 (-HN-C(O)-<u>C</u>H(Me)$_2$), 20.6 (Me of OAc), 20.4 (Me of OAc), 19.0 (Me of *i*Bu), 18.9 (Me of *i*Bu), 4.3 (C5′) ppm.

HRMS (ESI) Calcd. for $[C_{18}H_{22}IN_5O_7+H]^+$: 548.0642. Found: 548.0674.

2′,3′-Di-O-acetyl-5′-deoxy-5′-selenocyanato-N^2-isobutyramido-guanosine (11)

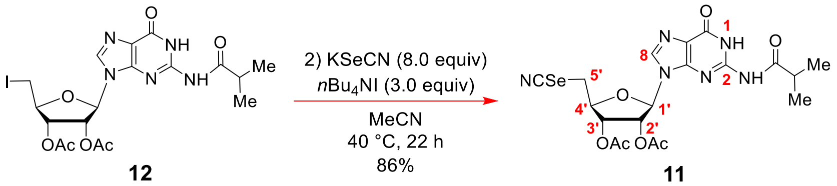

12 → 11

To a stirred solution of iodide **12** (0.34 g, 0.62 mmol) in dry MeCN (2.14 mL) in a 25 mL pear-shaped flask, was added KSeCN (0.72 g, 4.97 mmol, 8.0 equiv) and *n*Bu_4NI (0.69 g, 1.86 mmol, 3.0 equiv) under N_2. The reaction mixture was stirred at 40 °C for 22 h. The reaction mixture was quenched by adding BnBr (0.52 mL, 4.35 mmol, 7.0 equiv) and stirring for 30 min. To work up the reaction mixture, it was treated with 1% aqueous hydrochloric acid (100 mL) and extracted thereafter with CH_2Cl_2 (3 x 100 mL). The combined organic extracts were washed

with H_2O (100 mL) and brine (50 mL). The organic layer was then separated, dried over Na_2SO_4, filtered and concentrated *in vacuo*. The crude residue was purified by SiO_2 flash chromatography with EtOAc/MeOH (80:1) to give selenocyanate **11** (0.28 g, 86%). An analytical NMR was obtained by carrying out a second SiO_2 flash chromatography with EtOAc/MeOH (80:1).

^{1}H NMR of **11** (400.11 MHz, $CDCl_3$): δ 12.37 (s, 1H, C2-NH), 10.01 (s, 1H, N(1)-H), 7.76 (s, 1H, H8), 6.10 (apparent t, J = 4.8 Hz, 4.8 Hz, 1H, H2′), 5.97 (dd, J = 5.6 Hz, 5.2 Hz, 1H, H3′), 5.90 (d, J = 4.0 Hz, 1H, H1′), 4.48 (dd, J = 11.2 Hz, 5.6 Hz, 1H, H4′), 3.58-3.38 (complex m, 2H, H5′a and H5′b), 2.79 (septet, J = 6.8 Hz, 1H, CH of *i*Bu), 2.12 (s, 3H, OAc), 2.08 (s, 3H, OAc), 1.27 (d, J = 6.8 Hz, 3H, Me of *i*Bu), 1.24 (d, J = 7.2 Hz, 3H, Me of *i*Bu) ppm.

^{13}C NMR of **11** (100.61 MHz, $CDCl_3$): δ 179.7 (C2), 169.7 (C=O of OAc), 169.4 (C=O of OAc), 155.7 (C=O of *i*Bu and C6 overlapped), 148.2 and 148.0 (C4-tautomers/rotamers), 139.3 (C8), 122.1 (C5), 102.3 (SeCN), 87.6 (C1′), 80.0 (C4′), 72.5 (C2′), 72.3 (C3′), 36.2 (-HN-C(O)-<u>C</u>H(Me)$_2$), 30.3 (C5′), 20.5 (Me of OAc), 20.4 (Me of OAc), 19.0 (Me of *i*Bu), 18.9 (Me of *i*Bu).

HRMS (ESI) Calcd. for $[C_{19}H_{22}N_6O_7Se\text{-}H]^+$: 525.0637. Found: 525.0660.

N^6-Benzoyl-2′-deoxy-5′-O-(4,4′-dimethoxytrityl)-2′-selenomethyl-adenosinyl-[3′→5′-(2-cyanoethyl)phosphoroselenolyl)]-5′-deoxy-2′,3′-di-O-acetyl-N^2-isobutyramido-guanosine (22)

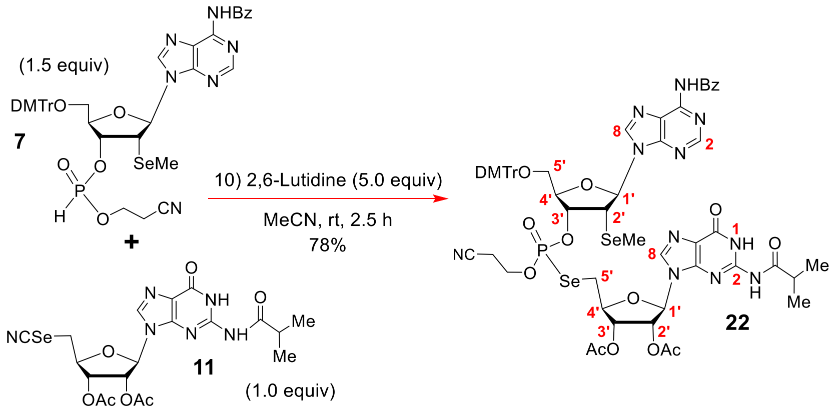

To a stirred rt solution of the 5′-selenocyanate **11** (0.65 g, 1.24 mmol) in dry MeCN (15.46 mL), in a 50 mL round-bottomed flask under N_2, was added 2,6-lutidine (0.72 mL, 6.19 mmol, 5.0 equiv) dropwise via a syringe. The solid 3′-H-phosphonate **7** (1.61 g, 1.86 mmol, 1.5 equiv) was then added in one portion and the reaction mixture was allowed to stir at rt for 2.5 h. The reaction mixture was then diluted with EtOAc (150 mL) and this solution successively washed with saturated aqueous $NaHCO_3$ (100 mL) and brine (100 mL). The organic layer was then separated, dried over Na_2SO_4, filtered and concentrated *in vacuo*. The crude residue was

thereafter purified by gradient elution SiO_2 flash chromatography with EtOAc/MeOH/pyridine (100:1:0.1→80:1:0.8) to give **22** (1.32 g, 78%).

^{31}P NMR of **22** (161.98 MHz, D_2O External Lock, MeCN): δ 22.12 ($^1J_{P\text{-}Se}$ = 507.0 Hz) and 21.47 ($^1J_{P\text{-}Se}$ = 502.1 Hz) ppm.

^{77}Se NMR of **22** (114.44 MHz, D_2O External Lock containing 0.25M KSeCN, MeCN): δ 118.17 (d, $^1J_{P\text{-}Se}$ = 502.4 Hz, -[Se-P(O)(OCH_2CH_2CN)]-, 97.92 (d, $^1J_{P\text{-}Se}$ = 505.8 Hz, -[Se-P(O)(OCH_2CH_2CN)]-, 74.23 (s, -SeMe), 68.30 (s, -SeMe), -329.00 (s, KSeCN standard) ppm.

HRMS (ESI) Calcd. for $[C_{60}H_{62}N_{11}O_{15}PSe_2+H]^+$ 1368.2573. Found: 1368.2234. **HRMS** (ESI) Calcd. for $[C_{60}H_{62}N_{11}O_{15}PSe_2-H]^+$: 1366.2417. Found: 1366.2332.

N^6-Benzoyl-2′-deoxy-2′-selenomethyl-adenosinyl-[3′→5′-(2-cyanoethyl) phosphoro-selenolyl)]-5′-deoxy-2′,3′-di-O-acetyl-N^2-isobutyramido-guanosine (6)

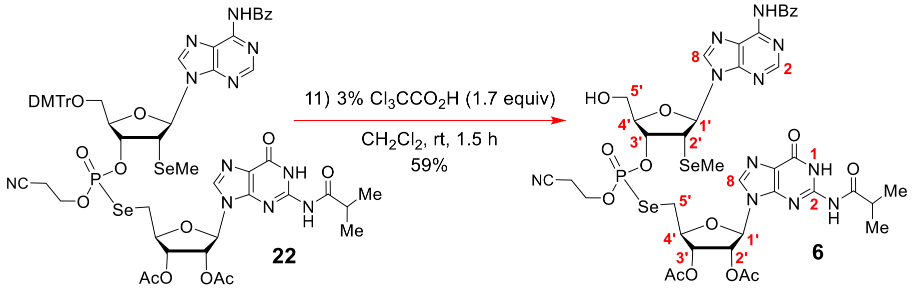

(The 3% wt. Cl_3CO_2H solution used here was prepared by adding 3.00 g of Cl_3CO_2H to 100.00 mL of CH_2Cl_2).

To the dimethoxytrityl ether **22** (0.37 g, 0.27 mmol) in a 25 mL pear-shaped flask under N_2 was added the above 3% wt. trichloroacetic acid solution in CH_2Cl_2 (2.51 mL, 0.46 mmol, *ca.* 1.7 equiv). The reaction mixture was thereafter stirred at rt for 1.5 h. The reaction mixture was then concentrated to dryness *in vacuo*. The crude residue was purified by gradient elution SiO_2 flash chromatography with CH_2Cl_2/MeOH (40:1→30:1→20:1) to give **6** as an amorphous solid (0.17 g, 59%).

^{31}P NMR of **10** (161.98 MHz, $CDCl_3$): δ 21.20 (s, $^1J_{P\text{-}Se}$ = 511.9 Hz) and 20.92 (s, $^1J_{P\text{-}Se}$ = 508.6 Hz) ppm.

^{31}**P NMR** of **10** (161.98 MHz, D_2O External Lock, MeCN): δ 21.96 (s, $^1J_{P\text{-}Se}$ = 505.4 Hz) and 21.17 (s, $^1J_{P\text{-}Se}$ = 502.1 Hz) ppm.

^{1}**H NMR** of the two P-diastereoisomers of **6** (400.11 MHz, $CDCl_3$): δ 12.31 and 12.27 (2 x s, 1H, G-C2-NH), 10.29 and 10.15 (2 x s, 1H, G-N(1)-H), 9.26 and 9.23 (2 x s, 1H, BzNH of A), 8.77 (1 x s, 1H, two overlapped H2 diastereoisomers of the A), 8.31 and 8.19 (2 x s, 1H, H8 of A), 8.03 (d, *J* = 7.6 Hz, 2H, 2 *ortho*-protons of Bz on A), 7.682 and 7.678 (2 x s, 1H, H8 of G), 7.62 (apparent triplet, *J* = 7.2 Hz, 7.2 Hz , 1H, *para*-proton of Bz on A), 7.53 (dd, *J* = 7.6 Hz, 7.2 Hz, 2H, 2 *meta*-protons of Bz on A), 6.45 (dd, *J* = 6.4 Hz, 5.6 Hz, 1H, H2′ of G), 6.33 (d, *J* = 10.0 Hz, 1H, H1′ of A) and 6.16 (d, *J* = 9.6 Hz, H1′ of A), 5.96 (d, *J* = 7.2 Hz, 1H, H1′ of G) and 5.92 (d, *J* = 6.8 Hz, 1H, H1′ of G), 5.89 (apparent d, *J* = 5.2 Hz, 1H, H3′ of G) and 5.86 (m, *J* = 4.8 Hz, 1.6 Hz, 1H, H3′ of G), 5.48 (dd, *J* = 9.6 Hz, 4.8 Hz, 1H, H3′ of A) and 5.37 (dd, *J* = 10.0 Hz, 4.8 Hz, 1H, H3′ of A), 4.63 and 4.59 (2 x s, 1H, C5′-OH of A), 4.51 (m, 2H, H4′ of A partially superimposed upon -O$\underline{CH_2}CH_2$CN) superimposed upon 4.44-4.34 (complex m, 3H, H5′a of A superimposed upon H4′ of G and -O$\underline{CH_2}CH_2$CN) partially superimposed upon 4.32 (m, 1H, H2′ of A), 4.08 (m, 1H, H5′a of G) partially superimposed on H5′b of A), 3.94-3.80 (broad m, 1H, H5′b of G), 3.38 (m, 1H, H5′a and H5′b of G), 2.96 (d, *J* = 5.2 Hz, 1H, -O$CH_2\underline{CH_2}$CN), 2.95 (d, *J* = 5.6 Hz, 1H, -O$CH_2\underline{CH_2}$CN), 2.87 (t, 2H, *J* = 6.0 Hz, -O$CH_2\underline{CH_2}$CN), 2.76 (m, 1H, CH of *i*Bu), 2.20 and 2.18 (2 x s, 3H, OAc of G), 2.00 and 1.97 (2 x s, 3H, OAc of G), 1.62 and 1.61 (2 x s, 3H, SeMe of A), 1.24 (m, 6H, Me of *i*Bu of G) ppm.

^{13}**C NMR** of the two P-diastereoisomers of **6** (100.61 MHz, $CDCl_3$): δ 179.6 (C2 of G), 169.84 and 169.81 (C=O of OAc of G), 169.4 and 169.2 (C=O of OAc of G), 164.5 (C=O of BzNH of A), 155.4 (C=O of *i*Bu of G and C6 of G overlapped), 152.3 and 152.2 (C2 of A), 150.6-150.35 (C6 of A, 2 rotamers of 2 P-diastereoisomers), 148.2 and 148.1 (C4 of A), 147.7 and 147.6 (C4 of G), 143.2 and 143.0 (C8 of A), 139.4 and 139.2 (C8 of G), 133.3 (quaternary carbons of Bz on A), 133.0 (*para*-carbon of Bz on A), 128.9 (2 *meta*-carbons of Bz on A), 127.9 (2 *ortho*-carbons of Bz on A), 124.5 (C5 of A), 122.9 and 122.8 (C5 of G), 116.7 and 116.1 (CN), 93.3 and 92.8 (C1′ of A), 87.7 and 87.6 (C1′ of G), 87.2 and 87.0 (C4′ of A), 82.8 (d, $^2J_{P\text{-}C3'}$ = 8.0 Hz, C3′ of A), 82.7 (d, $^2J_{P\text{-}C3'}$ = 4.0 Hz, C3′ of A), 82.1 (d, $^2J_{P\text{-}C5'}$ = 8.0 Hz, C4′ of G), 82.0 (d, $^2J_{P\text{-}C5'}$ = 8.0 Hz, C4′ of G), 73.1 and 72.8 (C3′ of G), 71.0 and 70.7 (C2′ of G), 63.1 (d, $^2J_{P\text{-}C}$ = 7.0 Hz, -O$\underline{CH_2}CH_2$CN), 62.8 and 62.7 (C5′ of A), 62.5 (d, $^2J_{P\text{-}C}$ = 5.0 Hz, -O$\underline{CH_2}CH_2$CN), 45.4 (d, $^3J_{P\text{-}C}$ = 8.0 Hz, C2′ of A), 45.0 (d, $^3J_{P\text{-}C}$ = 8.0 Hz, C2′ of A), 35.94 and 35.92 (-HN-C(O)-$\underline{C}$H(Me)$_2$), 27.4 and 26.5 (C5′ of G), 20.8, 20.7, 20.33, 20.29 (4 x s, Me of OAc of G), 19.6 (d, $^3J_{P\text{-}C}$ = 8.0 Hz, $\underline{C}H_2$CN), and 19.4 (d, $^3J_{P\text{-}C}$ = 9.0 Hz, $\underline{C}H_2$CN), 19.3, 19.1, 18.9, 18.7 (Me of *i*Bu), 5.0 and 4.5 (2 x SeMe) ppm.

HRMS (ESI) Calcd. for $[C_{39}H_{44}N_{11}O_{14}PSe_2+H]^+$: 1066.1266. Found: 1066.1235. **HRMS** (ESI) Calcd. for $[C_{39}H_{44}N_{11}O_{14}PSe_2-H]^+$: 1064.1110. Found: 1064.1218.

N^6-Benzoyl-2′-deoxy-5′-O-[2-((2-cyanomethyl)phosphoryloxy-methyl)-2-(4,4′-dimethoxy-trityloxymethyl)-N^1,N^2-dimethyl-malonamido]-2′-selenomethyl-adenosinyl-[3′→5′-(2-cyanoethyl)phosphoroselenolyl)]-5′-deoxy-2′,3′-di-O-acetyl-N^2-isobutyramido-guanosine (4)

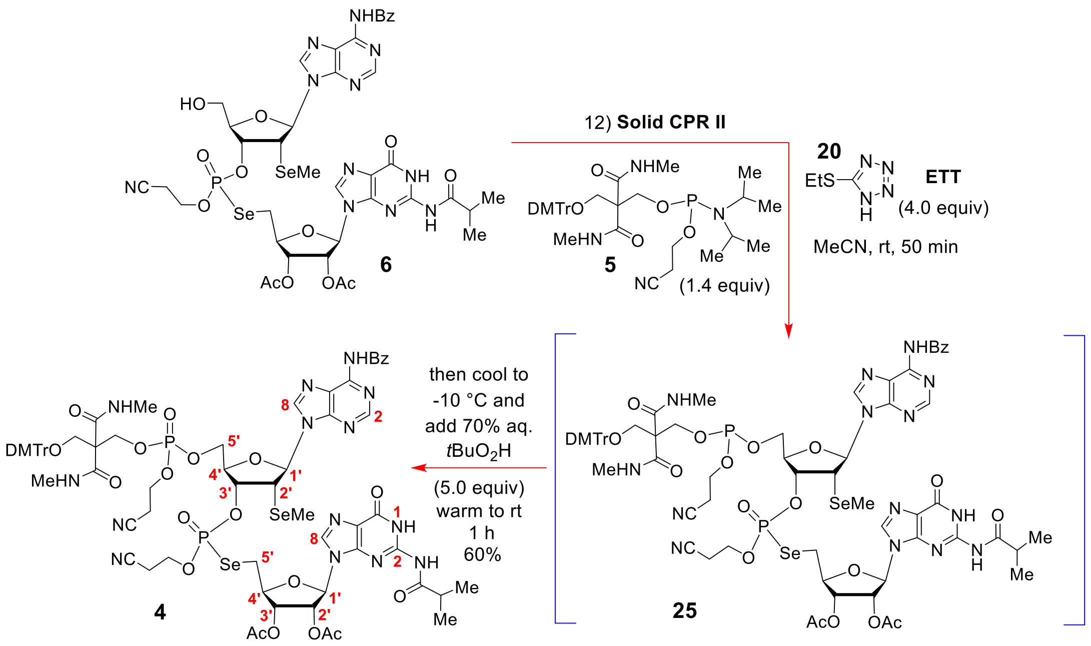

To a 10 mL pear-shaped flask was added alcohol **6** (50.0 mg, 0.047 mmol) followed by solid CPR II (**5**) (45.6 mg, 0.066 mmol, 1.4 equiv). The flask was fitted with a closed 3-way tap and an N_2-filled balloon and then placed under high vacuum for 1 h, before an N_2 atmosphere was introduced into the flask. MeCN (0.42 mL) was then added under N_2, and the contents of the flask were gently warmed to assist in dissolution, but this proved ineffective. However, as soon as 5-ethylthio-1H-tetrazole (**20**) (ETT) (24.5 mg, 0.188 mmol, 4.0 equiv) was added in a single portion, the solids quickly dissolved and a colourless solution was soon formed. The homogenous reaction mixture was stirred at rt for 50 min, whereafter TLC analysis revealed that no starting alcohol **6** was present and two faster-moving products had formed, which corresponded to **25**. The reaction flask was then placed in a salt-ice bath and cooled to -10 °C, whereafter aqueous *t*BuO$_2$H (70% solution)[11a] (0.023 mL, 0.235 mmol, 5.0 equiv) was added. The reaction mixture was then warmed to rt where it was stirred for 1 h. TLC analysis then revealed that a single slower-moving product had formed (relative to **6**); it subsequently turned out to be the 5′-O-phosphate **4**. It stained purple/brown after developing the TLC plate in anisaldehyde/H_2SO_4 stain and heating. The reaction flask was then diluted with EtOAc (5 ml), transferred to a separatory funnel, and reaction flask washed with more EtOAc (5 mL) and the washings added to the separatory funnel. Saturated aqueous $NaHCO_3$ (10 mL) was added to the separatory funnel, and the two layers were vigorously shaken and separated. The aqueous layer was then extracted with more EtOAc (3 x 10 mL), and the combined organic extracts were dried over Na_2SO_4, filtered and concentrated *in vacuo*. The crude residue was purified by

gradient elution SiO_2 flash chromatography with CH_2Cl_2/MeOH/pyridine (50:1:0.5→40:1:0.4→30:1:0.3) to give **4** (47.0 mg, 60%) as a white amorphous solid.

^{31}P NMR of the four P-diastereoisomers **4** (161.98 MHz, $CDCl_3$): δ 21.02 (s, $^1J_{P\text{-}Se}$ = 513.4 Hz, Se-P(O)-), 20.96 (s, $^1J_{P\text{-}Se}$ = 513.4 Hz, Se-P(O)-), 20.89 (s, $^1J_{P\text{-}Se}$ = 511.8 Hz, Se-P(O)-), 20.85 (s, $^1J_{P\text{-}Se}$ = 511.8 Hz, Se-P(O)-), -2.25 (s, P=O minor rotamer), -2.40 (s, P=O), -2.48 (s, P=O), -2.50 (s, P=O), -2.58 (s, P=O minor rotamer), -2.63 (s, P=O) ppm.

^{1}H NMR of the four P-diastereoisomers of **4** (400.11 MHz, $CDCl_3$): δ 12.29, 12.28, 12.24 and 12.23 (4 x s, 1H, G-C2-NH), 10.04, 9.99, 9.86 and 9.83 (2 x s, 1H, G-N(1)-H), 9.12 (2 x s, 1H, BzNH of A), 8.87 and 8.79 (1 x s, three overlapped H2 diastereoisomers of the A and 1 x s, one H2 diastereoisomer of the A, together 1H), 8.42, 8.40, 8.36 and 8.30 (4 x s, 1H, H8 of A), 8.040, 8.035, 8.022 and 8.017 (4 x s, 2 x MeNHC(O)-, each 1H), 7.724, 7.716, 7.712 and 7.70 (2 x s, 1H, H8 of G), 7.62 (apparent triplet, *J* = 7.6 Hz, 7.2 Hz, 1H, *para*-proton of Bz on A), 7.53 (dd, *J* = 8.0 Hz, 7.2 Hz, 2H, 2 *meta*-protons of Bz on A), 7.38 (complex m, 2H, *ortho*-H atoms of BzNH), 7.31-7.14 (complex m, 9H, DMTr), 6.82 (2 x superimposed 4H, *J* = 8.8 Hz, *ortho*-H atoms adjacent to the OMe of the DMTr), 6.34 (complex m, 2H, H2′ of G and H1′ of A), 5.95 (3 x superimposed d, *J* = 6.8 Hz, 1H, H1′ of G), 5.82 (complex m, 1H, H3′ of G), 5.44 (dd, *J* = 10.0 Hz, 5.6 Hz, 1H, H3′ of A), 4.71-4.54 (complex m, 4H, H2′ and H4′ of A plus $-OCH_2CH_2CN$), 4.54-4.25 (complex m, 7H, CH_2ODMTr and $CH_2OP(O)O_2$- superimposed upon H4′ of G and $-OCH_2CH_2CN$), 3.88 (m, 1H, H5′a of G), 3.76 (s, 6H, 2 x OMe of DMTr), 3.67-3.54 (complex m, 1H, H5′a and b of A), 3.45 (m, 1H, H5′b of G), 2.92 (m, 1H, $-OCH_2CH_2CN$), 2.83 (t, 2H, *J* = 6.0 Hz, $-OCH_2CH_2CN$), 2.74 and 2.72 (2 x s, 6H, NHMe) superimposed upon 2.74 (m, 1H, CH of *i*Bu), 2.17 and 2.168 (2 x s, 3H, OAc of G), 2.05, 2.03 and 2.02 (2 x s, 3H, OAc of G), 1.82, 1.81 and 1.80 (3 x s, 3H, SeMe of A), 1.24 (complex m, 6H, Me of *i*Bu of G) ppm.

Tentatively assigned **^{13}C NMR** peaks for the 4 P-Diastereomers of **4** (100.61 MHz, $CDCl_3$): δ 180.10, 179.67, 179.54 and 179.52 (C2 of G), 169.8, 169.48, 169.47, 169.39, 168.96, 168.89, 168.87, 168.84 and 168.81 (C=O of OAc of G), 164.57 and 164.54 (C=O of BzNH of A), 158.59 (quaternary C of PMP1), 156.15 and 156.13 (quaternary C of PMP2), 155.42, 155.41 and 154.40 (C=O of *i*Bu of G), 152.89, 152.80 and 152.78 (C6 of A), 149.86 and 149.82 (C4 of A), 148.23, 148.20, 148.13 (C4 of G), 143.9, 142.62, 142.56, 142.40 and 142.31 (C8 of A), 139.24, 139.21, 139.04 and 139.00 (C8 of G), 134.75 (quaternary carbon of Ph in DMTr), 133.53 (quaternary C of BzNH of A), 132.89 and 132.82 (*para*-carbon of Bz on A), 129.98 (4 *meta*-carbons of PMP of DMTr), 129.14 (*para*-carbon of Ph of DMTr), 128.9 (2 *meta*-carbons of Bz on A), 128.01 (2 *meta*-carbons of Ph of DMTr[44]), 127.9 (2 *ortho*-carbons of Bz on A and 2 *ortho*-carbons of Ph of DMTr[44]), 127.1 (1 *para*-carbon of Ph of DMTr), 116.74, 116.60 and 116.55 (2 x quaternary CN of 2 x $-OCH_2CH_2CN$ groups) 113.36 and 113.17 (4 *ortho*-carbons of PMP), 90.11, 89.97, 89.92 and 89.82 (C1′ of A), 87.51, 87.44, 87.33 and 87.26 (C1′ of G), 82.74 and

82.57 (C4′ of A), 82.00, 81.87, 81.66 and 81.56 (C4′ of G), 80.27 and 80.21 (C3′ of A), 73.01 and 72.87 (C3′ of G), 71.46 and 71.24 (C2′ of G), 68.74 and 68.68 (-$\underline{C}H_2OP(O)O_2$-), 66.57, 66.50, 66.35 and 66.30 (-$\underline{C}H_2$ODMTr), 63.42, 63.36, 63.32 and 63.28 (C5′ of A), 62.90 and 62.85 (C5′ of A), 62.67, 62.62, 62.57, 62.51 and 62.46 (2 x -O$\underline{CH_2}CH_2$CN), 55.2 (OMe of DMTr), 42.8 and 42.7 (C2′ of A), 36.02 (2 x -C(O)-NH$\underline{Me}$), 29.7 (C5′ of G), 26.6 ($\underline{C}$H of *i*Bu), 20.75, 20.71 and 20.39 (OAc-Me of G), 19.60, 19.51 and 19.44 (2 x -$OCH_2\underline{C}H_2$CN), 19.26, 19.24, 19.0 and 18.8 (Me groups of *i*Bu of G), 4.70, 4.65, 4.56 and 4.53 (4 x Se$\underline{Me}$) ppm. (PMP = *p*-$MeOC_6H_4$ of DMTr group).

Important Note: This ^{13}C NMR spectrum was complicated by the occurrence of rotamers, which made many of the aforementioned DEPT observable or ^{1}H/^{13}C cross peak observable signals practically invisible in the normal ^{1}H-decoupled ^{13}C NMR spectrum in $CDCl_3$.

HRMS (ESI) Calcd. for $[C_{70}H_{78}N_{14}O_{21}P_2Se_2+H]^+$: 1673.3350. Found: 1673.2803. **HRMS** (ESI) Calcd. for $[C_{70}H_{78}N_{14}O_{21}P_2Se_2+Na]^+$: 1695.3169. Found: 1695.2469.

3′-O-Acetyl-N^6-benzoyl-2′-deoxy-5′-O-[2-O-(2-(cyanomethyl)-phosphoryloxymethyl]-2-(4,4′-dimethoxytrityl)oxymethyl)-N^1,N^2-dimethylmalonamido]-adenosine (28)

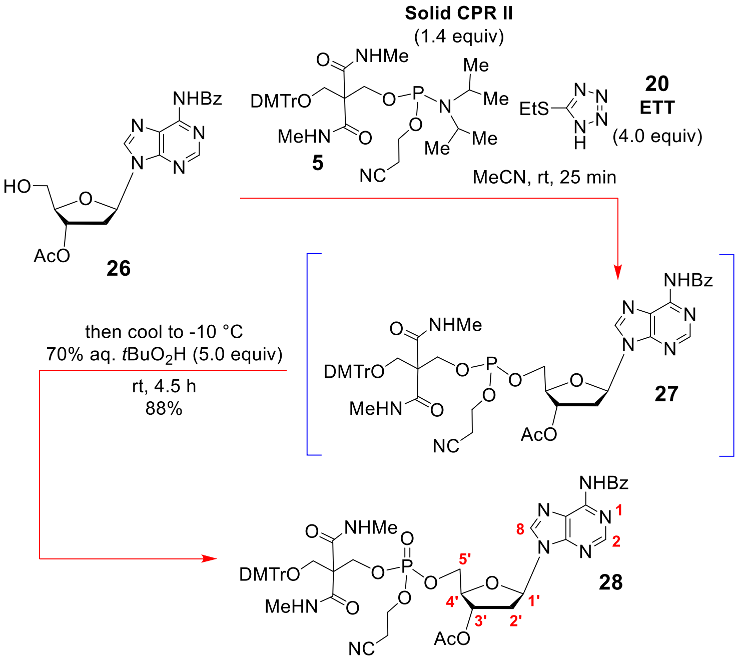

To a 10 mL pear-shaped flask was added 3′-O-acetyl-N^6-benzamido-2′-deoxy-D-adenosine (**26**) (0.10 g, 0.25 mmol) followed by Solid CPR II (**5**) (0.24 g, 0.35 mmol, 1.4 equiv). The flask was fitted with a closed 3-way tap and an N_2-filled balloon and then placed under high vacuum

for 1 h, before an N_2 atmosphere was introduced into the flask. MeCN (2.24 mL) was then added under N_2, and the contents of the flask were gently warmed to assist in dissolution, but this proved ineffective. However, as soon as 5-ethylthio-1H-tetrazole (ETT) (0.13 g, 1.01 mmol, 4.0 equiv) was added in a single portion, the solids quickly dissolved and a colourless solution was soon formed. The homogenous reaction mixture was stirred at rt for 25 min, whereafter TLC analysis revealed that no starting alcohol **26** was present. The reaction flask was then placed in a salt-ice bath and cooled to -10 °C. Aqueous *t*BuO_2H (70% solution)[11a] (0.12 mL, 1.26 mmol, 5.0 equiv) was added. The reaction mixture was then stirred for 4.5 h, but TLC analysis revealed that the reaction had actually completed after just 50 min at rt. It was then diluted with EtOAc (5 mL), transferred to a separatory funnel, and reaction flask washed with more EtOAc (10 mL), and the washings added to the separatory funnel. Saturated aqueous $NaHCO_3$ (10 mL) was added to the separatory funnel and the two layers vigorously shaken and separated. The aqueous layer was then extracted with more EtOAc (3 x 10 mL), and the combined organic extracts dried over Na_2SO_4, filtered and concentrated *in vacuo*. The crude residue was purified by gradient elution SiO_2 flash chromatography with CH_2Cl_2/MeOH/pyridine (50:1:0.5→40:1:0.4→30:1:0.3) to give **28** (0.22 g, 88%) as a white amorphous solid.

^{31}P NMR of the two P-diastereoisomers of **28** (161.98 MHz, $CDCl_3$): δ -2.24 (s, -$(O)_3P$=O) ppm.

^{1}H NMR of the two P-diastereoisomers of **28** (400.11 MHz, $CDCl_3$): δ 9.16 and 9.14 (2 x s, 1H, N<u>H</u>Bz of 2′-dA), 8.81 (1 x s, 1H, two overlapped H2 diastereoisomers of the 2′-dA), 8.38 and 8.36 (2 x s, 1H, H8 of 2′-dA), 8.03 and 8.01 (2 x s, each 1H, each H-bonded, 2 x MeN<u>H</u>C(O)), 7.60 (dd, 1H, *J* = 7.6 Hz, 7.2 Hz, *para*-H of NHBz), 7.51 (t, 2H, *J* = 7.6 Hz, *meta*-H atoms of NHBz), 7.38 (d, 2H, *J* = 7.6 Hz, *ortho*-H atoms of NHBz), 7.42-7.11 (complex m, 9H, DMTr), 6.83 (d, 4H, *J* = 8.8 Hz, *ortho*-H atoms adjacent to the OMe of the DMTr), 6.55 (broadened t, 1H, *J* = 6.4 Hz, H1′), 5.48 (narrow m, 1H, H3′), 4.58 (d, 1H, *J* = 11.2 Hz, -CH_2OP(O)) and 4.57 (d, 1H, *J* = 10.8 Hz, -CH_2OP(O)), 4.32 (m, 1H, H4′) partially superimposed upon 4.31 (m, 2H, -OC<u>H</u>$_2$$CH_2$CN), 4.16 (m, 2H, H5′a and H5′b), 2.98 (m, 1H, *J* = 14.4 Hz, 6.4 Hz, H2′a), 2.75 and 2.74 (2 x s, each 3H, NH<u>Me</u>), 2.69 (m, 2H, -CH_2CN), 2.67 (m, 1H, *J* = 6.4 Hz, H2′b), 2.14 (s, 3H, OAc) ppm.

HRMS (ESI) Calcd. for $[C_{50}H_{53}N_8O_{13}P+H]^+$: 1005.3548. Found: 1005.3576.

2′-Azido-2′-deoxy-3′,5′-O-(1,1,3,3-tetraisopropyl-1,3-disiloxanyl)-adenosine (29)

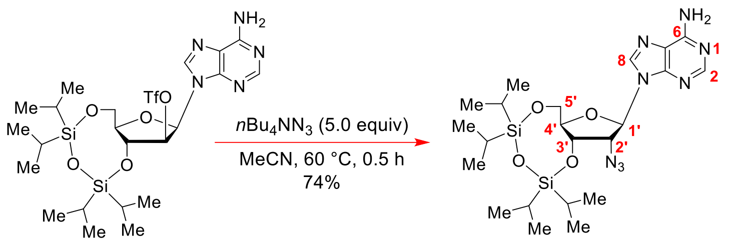

To a stirred solution of O2′-triflate **10** (0.50 g, 0.78 mmol) in dry acetonitrile (8.03 mL) in a 50 mL pear-shaped flask was added tetra-*n*-butylammonium azide (1.11 g, 3.90 mmol, 5.0 equiv). The reaction mixture was stirred at 60 °C under N_2 for 0.5 h. The reaction mixture was thereafter diluted with EtOAc (5 mL) and rotary evaporated to dryness. The reaction mixture was extracted with EtOAc (70 mL x 3) and H_2O (100 mL). The organic layer was separated, dried over $MgSO_4$, filtered and concentrated *in vacuo*. The crude residue was purified by repeat SiO_2 flash chromatography with petrol/EtOAc (3:2→1:1), followed by a second column purification with distilled CH_2Cl_2/MeOH (50:1) which gave the inverted C2′-*ribo*-azide **29**[23] (0.31 g, 74%).

^{1}H NMR of **29** (600.13 MHz, $CDCl_3$): δ 8.32 (s, 1H, H8), 8.00 (s, 1H, H2), 5.77 (d, $J_{1'2'}$ = 0.6 Hz, 1H, H1′), 5.68 (br s, 2H, -NH_2), 5.18 (dd, $J_{3'4'}$ = 9.0 Hz, $J_{2'3'}$ = 5.4 Hz, 1H, H3′), 4.61 (d, $J_{2'3'}$ = 5.4 Hz, 1H, H2′), 4.17 (dd, $J_{5'a5'b}$ = 13.2 Hz, 1.8 Hz, 1H, H5′a), 4.11 (dt, $J_{4'5'a}$ = 11.4 Hz, $J_{3'4'}$ = 9.0 Hz, $J_{4'5'b}$ = 3.0 Hz, 1H, H4′), 4.04 (dd, $J_{5'a5'b}$ = 13.2 Hz, $J_{4'5'b}$ = 2.4 Hz, 1H, H5′b), 1.10-0.90 (complex m, 28 H, -O-(*i*Pr_2-Si-O-Si-*i*Pr_2)-O-) ppm.

^{13}C NMR of **29** (150.92 MHz, $CDCl_3$): δ 155.4 (C6), 153.2 (C8), 149.0 (C4), 139.2 (C2), 120.4 (C5), 87.5 (C1′), 81.7 (C4′), 71.1 (C3′), 65.4 (C2′), 60.1 (C5′), 17.4 (Me), 17.3 (Me), 17.26 (2 x Me), 17.19 (Me), 17.0 (Me), 16.92 (Me), 16.85 (Me), 13.4 (Si<u>C</u>H), 13.0 (Si<u>C</u>H), 12.73 (Si<u>C</u>H), 12.70 (Si<u>C</u>H) ppm.

HRMS (ESI) Calcd. for $[C_{22}H_{38}N_8O_4Si_2+H]^+$: 535.2633. Found: 535.2645.

2′-O-Benzoyl-3′,5′-O-(1,1,3,3-tetraisopropyl-1,3-disiloxanyl)-adenosine (30)

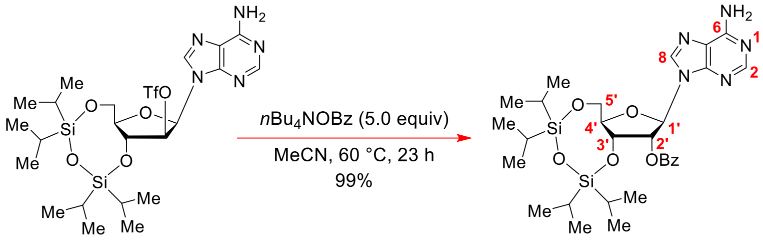

To a stirred solution of O2′-triflate **10** (0.20 g, 0.31 mmol) in dry acetonitrile (3.21 mL) in a 50 mL pear-shaped flask was added tetra-*n*-butylammonium benzoate (0.57 g, 1.56 mmol, 5.0 equiv). The reaction mixture was stirred for 23 h under N_2 at 60 °C. It was then diluted with CH_2Cl_2 (5 mL) and rotary evaporated to dryness. The crude residue was then treated with H_2O (100 mL) and extracted with CH_2Cl_2 (70 mL x 3), before the combined organic extracts were washed with brine (50 mL). The separated organic layer was dried over $MgSO_4$, filtered and concentrated *in vacuo*. The crude residue was then purified by repeat SiO_2 flash chromatography initially with petrol/EtOAc (1:1), followed by a second flash chromatographic purification using distilled CH_2Cl_2/MeOH (50:1) to give the pure O-benzoate **30** (0.19 g, 99%).

^{1}H NMR of **30** (600.13 MHz, $CDCl_3$): δ 8.30 (s, 1H, H8), 8.10 (m, 2H, *ortho*-H atoms of Ph), 8.01 (s, 1H, H2), 7.59 (m, 1H, *para*-H of Ph), 7.47 (m, 2H, *meta*-H atoms of Ph), 6.18 (d, $J_{1'2'}$ = 0.6 Hz, 1H, H1′), 6.06 (s, 2H, -NH_2), 6.02 (dd, $J_{1'2'}$ = 1.2 Hz, $J_{2'3'}$ = 5.4 Hz, 1H, H2′), 5.26 (dd, $J_{3'4'}$ = 8.4 Hz, $J_{2'3'}$ = 5.4 Hz, 1H, H3′), 4.23-4.17 (complex m, 2H, H4′ and H5′), 4.08 (complex m, 1H, H5′), 1.20-0.75 (complex m, 28 H, -O-(*i*Pr_2-Si-O-Si-*i*Pr_2)-O-) ppm.

^{13}C NMR of **30** (150.92 MHz, $CDCl_3$): δ 165.3 (C=O of the -Bz group), 155.6 (C6), 153.1 (C8), 149.2 (C4), 139.2 (C2), 133.3 (*para*-C of Bz), 129.8 (2 x *ortho*-C of Bz), 129.6 (quaternary C of Bz) 128.4 (2 x *meta*-C of Bz), 120.2 (C5), 87.6 (C1′), 82.3 (C4′), 76.4 (C2′), 69.6 (C3′), 60.9 (C5′), 17.4 (Me), 17.34 (Me), 17.30 (Me), 17.26 (Me), 16.92 (Me), 16.9 (Me), 16.76 (Me), 16.74 (Me), 13.3 (SiCH), 13.0 (SiCH), 12.8 (SiCH), 12.6 (SiCH) ppm.

HRMS (ESI) Calcd. for $[C_{29}H_{43}N_5O_6Si_2+H]^+$: 614.2830. Found: 614.2844.

2′-Deoxy-3′,5′-O-(1,1,3,3-tetraisopropyl-1,3-disiloxanyl)-2′-thioacetyl-adenosine (31)

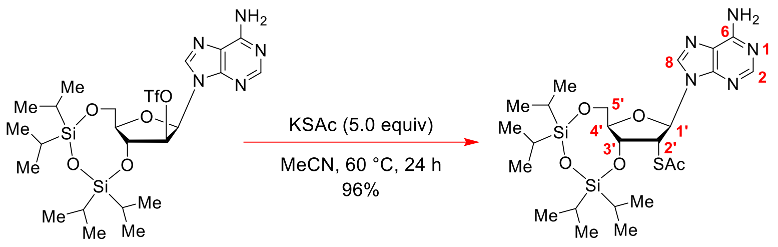

10 31

To a stirred solution of O2′-triflate **10** (0.20 g, 0.31 mmol) in dry acetonitrile (8.03 mL) in a 50 mL pear-shaped flask was added potassium thioacetate (0.18 g, 1.56 mmol, 5.0 equiv). The reaction mixture was stirred at 60 °C for 24 h under N_2. The reaction mixture was diluted with CH_2Cl_2 (5 mL) and rotary evaporated to dryness. The crude residue was then treated with H_2O (100 mL) and extracted with CH_2Cl_2 (70 mL x 3), before the combined organic extracts were washed with brine (50 mL). The organic extract was dried over $MgSO_4$, filtered and concentrated *in vacuo*. The crude residue was then repeat purified by SiO_2 flash chromatography with petrol/EtOAc (1:1), followed by a second column of distilled CH_2Cl_2/MeOH (50:1) to give pure S-thioacetate **31**[45a] (0.17 g, 96%).

^{1}H NMR of **31** (600.13 MHz, $CDCl_3$): δ 8.32 (s, 1H, H8), 7.95 (s, 1H, H2), 6.06 (d, $J_{1'2'}$ = 4.8 Hz, 1H, H1′), 5.66 (br s, 2H, -NH_2), 5.27 (overlapped dd, $J_{3'4'}$ = 7.2 Hz, $J_{2'3'}$ = 7.2 Hz, 1H, H3′), 4.70 (dd, $J_{2'3'}$ = 7.2 Hz, $J_{1'2'}$ = 4.8 Hz, 1H, H2′), 4.09-4.02 (m, 3H, H4′ and H5′a and H5′b), 2.33 (s, 3H, -SAc), 1.18-0.75 (complex m, 28 H, -O-(*i*Pr_2-Si-O-Si-*i*Pr_2)-O-) ppm.

^{13}C NMR of **31** (150.92 MHz, $CDCl_3$): δ 194.8 (C=O of the -SAc), 155.4 (C6), 153.1 (C8), 149.6 (C4), 139.6 (C2), 120.2 (C5), 88.7 (C1′), 84.9 (C4′), 71.4 (C3′), 62.6 (C5′), 51.5 (C2′), 30.5 (Me of the -SAc), 17.5 (Me), 17.36 (Me), 17.32 (Me), 17.31 (Me), 17.1 (Me), 16.99 (Me), 16.96 (Me), 16.91 (Me), 13.3 (Si<u>C</u>H), 13.2 (Si<u>C</u>H), 12.9 (Si<u>C</u>H), 12.7 (Si<u>C</u>H) ppm.

HRMS (ESI) Calcd. for $[C_{24}H_{41}N_5O_5SSi_2+H]^+$: 568.2445. Found: 568.2445.

2′-Deoxy-2′-iodo-3′,5′-O-(1,1,3,3-tetraisopropyl-1,3-disiloxanyl)-adenosine (32)

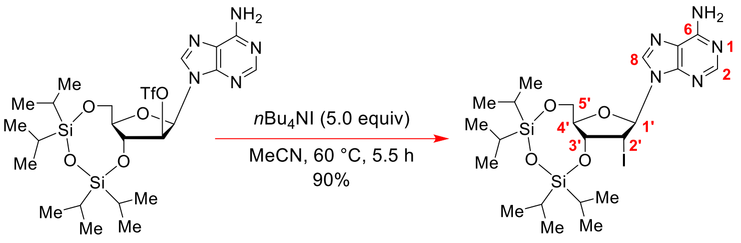

To a stirred solution of O2′-triflate **10** (0.20 g, 0.31 mmol) in dry acetonitrile (3.21 mL), in a 50 mL pear-shaped flask, was added tetra-*n*-butylammonium iodide (0.58 g, 1.56 mmol, 5.0 equiv). The reaction flask was wrapped in tin foil, to exclude light, and the reaction mixture was stirred at 60 °C for 5.5 h. The reaction mixture was then diluted with CH_2Cl_2 (5 mL) and rotary evaporated to dryness. The crude residue was then treated with H_2O (100 mL) and extracted with CH_2Cl_2 (70 mL x 3), before the combined organic extracts were washed with brine (50 mL). The organic layer was then dried over $MgSO_4$, filtered and concentrated *in vacuo*. The crude residue was purified by gradient elution SiO_2 flash chromatography with petrol/EtOAc (2:1→1:1). A second SiO_2 flash chromatographic purification using distilled CH_2Cl_2/MeOH (50:1) afforded the analytically pure iodide **32**[43] (0.17 g, 90%).

^{1}H NMR of **32** (600.13 MHz, $CDCl_3$): δ 8.33 (s, 1H, H8), 8.04 (s, 1H, H2), 6.55 (dd, $J_{1'2'}$ = 2.4 Hz, 1H, H1′), 5.64 (s, 2H, -NH_2), 5.03 (dd, $J_{2'3'}$= 6.6 Hz, $J_{1'2'}$= 2.4 Hz, 1H, H2′), 4.21 (m, $J_{3'4'}$ = 7.2 Hz, $J_{2'3'}$ = 6.0 Hz, 1H, H3′), 4.18-4.14 (m, 2H, H4′ and H5′a), 4.05 (m, 1H, H5′b), 1.13-0.96 (complex m, 28 H, -O-(*i*Pr_2-Si-O-Si-*i*Pr_2)-O-) ppm.

^{13}C NMR of **32** (150.92 MHz, $CDCl_3$): δ 155.4 (C6), 153.2 (C8), 149.1 (C4), 139.0 (C2), 120.4 (C5), 92.1 (C1′), 84.0 (C4′), 68.5 (C3′), 60.7 (C5′), 34.1 (C2’), 17.4 (Me), 17.3 (Me), 17.28 (Me), 17.25 (Me), 17.1 (Me), 17.03 (Me), 17.0 (Me), 16.96 (Me), 13.4 (Si<u>C</u>H), 13.0 (Si<u>C</u>H), 12.8 (Si<u>C</u>H), 12.7 (Si<u>C</u>H) ppm.

2′-Bromo-2′-deoxy-3′,5′-O-(1,1,3,3-tetraisopropyl-1,3-disiloxanyl)-adenosine (33)

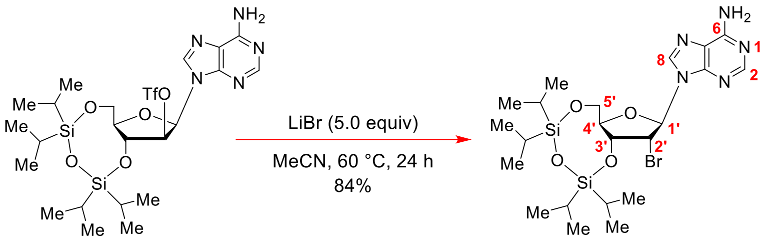

10 **33**

To a stirred solution of O2′-triflate **10** (0.20 g, 0.31 mmol) in dry acetonitrile (3.21 mL), in a 50 mL pear-shaped flask under N_2, was added lithium bromide (0.14 g, 1.56 mmol, 5.0 equiv). The reaction mixture was heated with stirring for 24 h at 60 °C. The reaction mixture was then diluted with CH_2Cl_2 (5 mL) and rotary evaporated to dryness. The crude residue was then treated with H_2O (100 mL) and extracted with CH_2Cl_2 (70 mL x 3), before the combined organic extracts were washed with brine (50 mL). The organic layer was dried over $MgSO_4$, filtered and concentrated *in vacuo*. The crude residue was purified by SiO_2 flash chromatography with distilled CH_2Cl_2/MeOH (50:1) to give bromide **33**[43] as an oil (0.15 g, 84%).

^{1}H NMR of **33** (600.13 MHz, $CDCl_3$): δ 8.32 (s, 1H, H8), 8.09 (s, 1H, H2), 6.41 (d, $J_{1'2'}$ = 1.2 Hz, 1H, H1′), 6.18 (s, 2H, -NH_2), 4.92 (dd, $J_{2'3'}$= 6.0 Hz, $J_{1'2'}$ = 1.2 Hz, 1H, H2′), 4.83 (m, $J_{3'4'}$= 8.4 Hz, $J_{2'3'}$ = 6.0 Hz, 1H, H3’), 4.22 (m, 1H, H4′) 4.20 (m, 1H H5′a), 4.05 (m, 1H, H5′b), 1.10-0.85 (complex m, 28 H, -O-(*i*Pr_2-Si-O-Si-*i*Pr_2)-O-) ppm.

^{13}C NMR of **33** (150.92 MHz, $CDCl_3$): δ 155.6 (C6), 153.2 (C8), 149.0 (C4), 138.8 (C2), 120.3 (C5), 90.7 (C1′), 82.6 (C4′), 68.2 (C3′), 60.2 (C5′), 54.5 (C2′), 17.4 (Me), 17.3 (Me), 17.24 (Me), 17.21 (Me), 17.1 (Me), 16.92 (Me), 16.87 (Me), 16.8 (Me), 13.3 (Si<u>C</u>H), 12.9 (Si<u>C</u>H), 12.8 (Si<u>C</u>H), 12.6 (Si<u>C</u>H) ppm.

HRMS (ESI) Calcd. for $[C_{22}H_{38}BrN_5O_4Si_2+H]^+$: 572.1724. Found: 572.1725.

Part B. Copies of Spectra

S3. Copies of NMR Spectra of All Compounds Reported

^{1}H NMR spectrum of compound 14

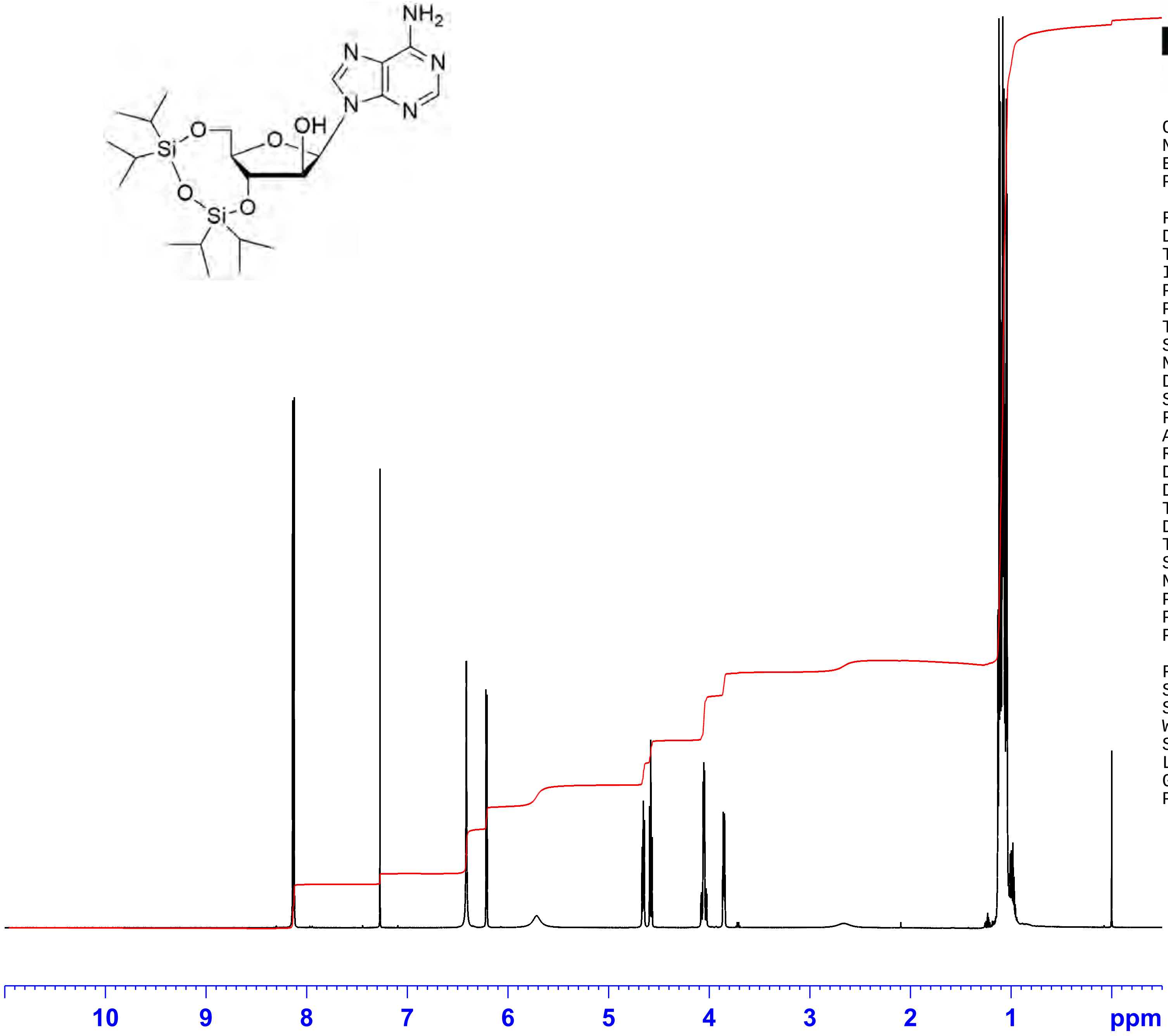


```
Current Data Parameters
NAME              LH-I-49
EXPNO                  10
PROCNO                  1

F2 - Acquisition Parameters
Date_            20220111
Time                11.19 h
INSTRUM             spect
PROBHD   Z114607_0188 (
PULPROG              zg30
TD                 180286
SOLVENT             CDCl3
NS                     16
DS                      0
SWH             18028.846 Hz
FIDRES           0.200003 Hz
AQ              4.9999318 sec
RG                  31.58
DW                 27.733 usec
DE                   8.00 usec
TE                  300.0 K
D1             0.10000000 sec
TD0                     1
SFO1          600.1337060 MHz
NUC1                   1H
P0                   3.33 usec
P1                  10.00 usec
PLW1          26.60000038 W

F2 - Processing parameters
SI                 262144
SF            600.1300080 MHz
WDW                    EM
SSB      0
LB                   0.10 Hz
GB       0
PC                   1.00
```

Expanded region of the ^{1}H NMR spectrum of compound 14

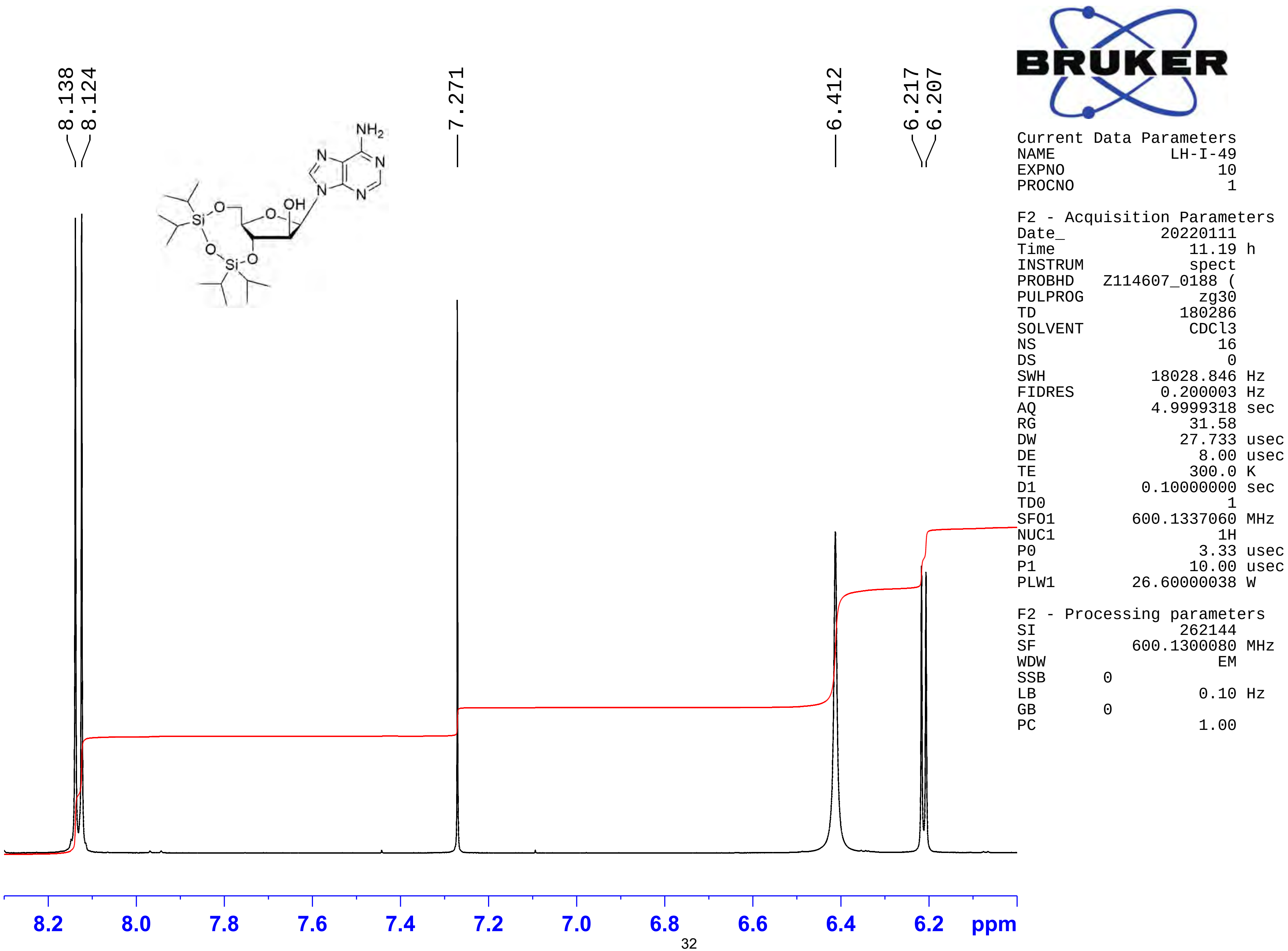

Expanded region of the ^{1}H NMR spectrum of compound 14

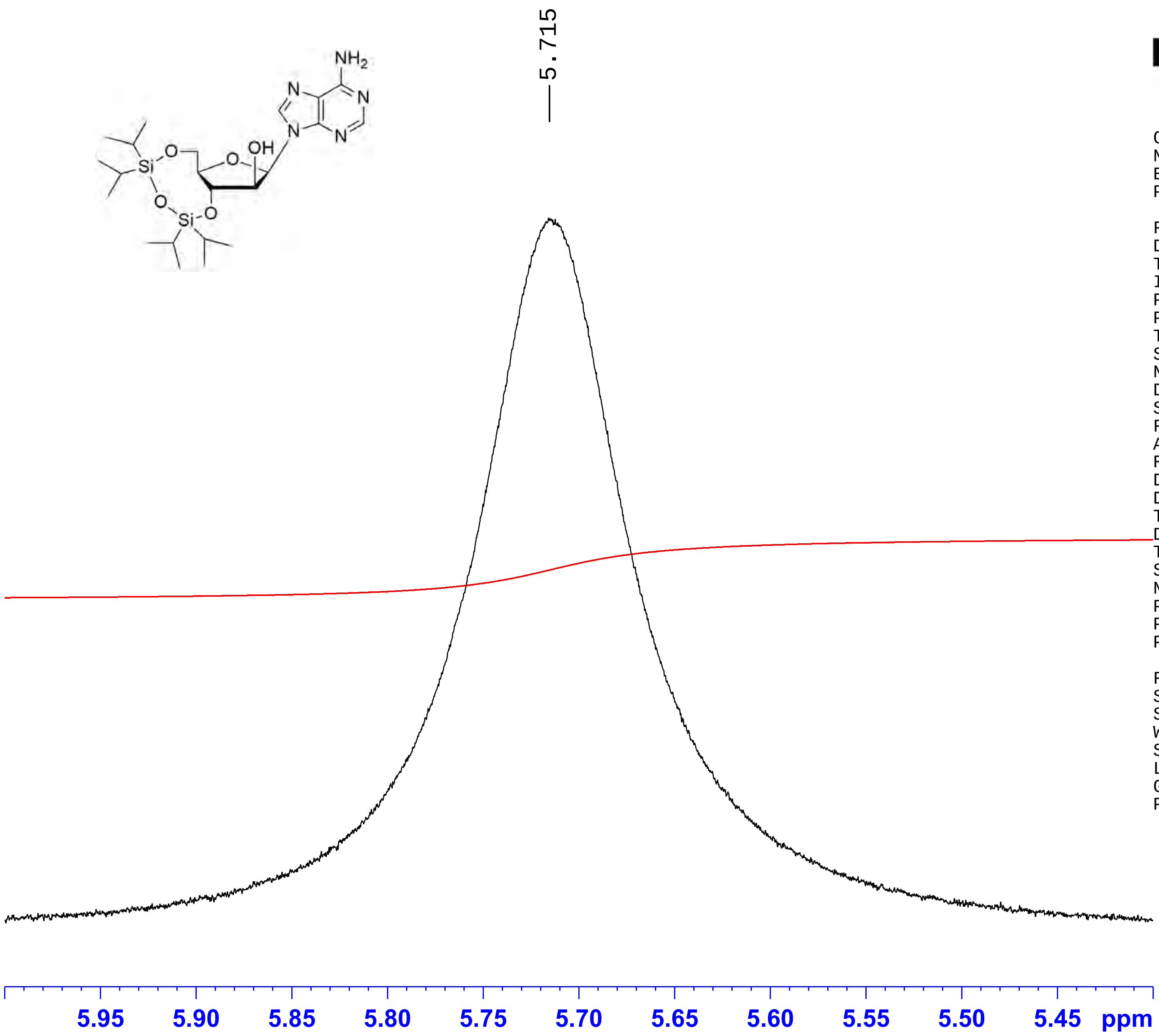


```
Current Data Parameters
NAME              LH-I-49
EXPNO                  10
PROCNO                  1

F2 - Acquisition Parameters
Date_            20220111
Time                11.19 h
INSTRUM             spect
PROBHD   Z114607_0188 (
PULPROG              zg30
TD                 180286
SOLVENT             CDCl3
NS                     16
DS                      0
SWH             18028.846 Hz
FIDRES           0.200003 Hz
AQ              4.9999318 sec
RG                  31.58
DW                 27.733 usec
DE                   8.00 usec
TE                  300.0 K
D1             0.10000000 sec
TD0                     1
SFO1          600.1337060 MHz
NUC1                   1H
P0                   3.33 usec
P1                  10.00 usec
PLW1          26.60000038 W

F2 - Processing parameters
SI                 262144
SF            600.1300080 MHz
WDW                    EM
SSB      0
LB                   0.10 Hz
GB       0
PC                   1.00
```

Expanded region of the ^{1}H NMR spectrum of compound 14

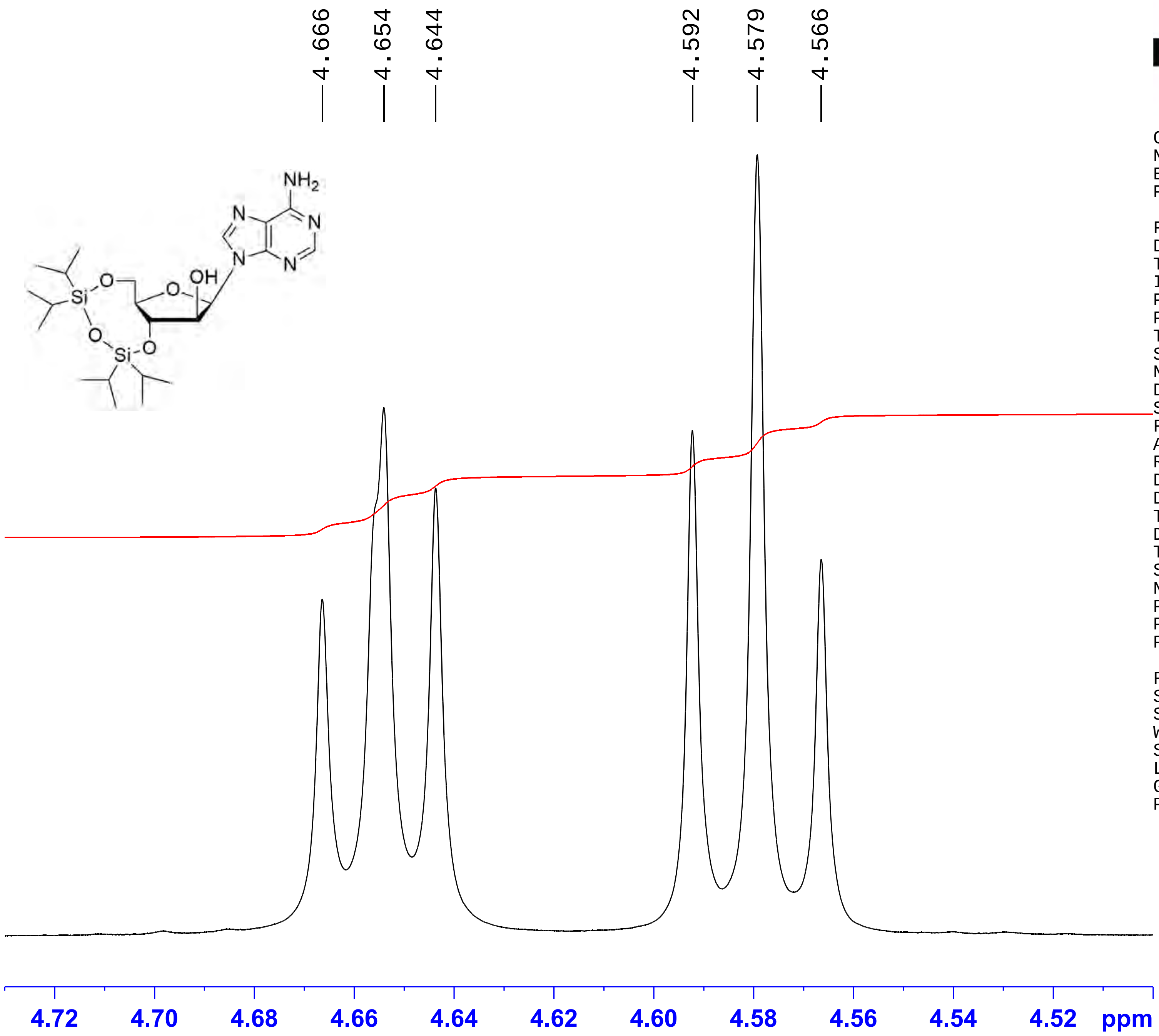

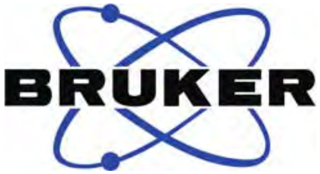

```
Current Data Parameters
NAME              LH-I-49
EXPNO                  10
PROCNO                  1

F2 - Acquisition Parameters
Date_            20220111
Time                11.19 h
INSTRUM             spect
PROBHD   Z114607_0188 (
PULPROG              zg30
TD                 180286
SOLVENT             CDCl3
NS                     16
DS                      0
SWH             18028.846 Hz
FIDRES           0.200003 Hz
AQ              4.9999318 sec
RG                  31.58
DW                 27.733 usec
DE                   8.00 usec
TE                  300.0 K
D1             0.10000000 sec
TD0                     1
SFO1          600.1337060 MHz
NUC1                   1H
P0                   3.33 usec
P1                  10.00 usec
PLW1          26.60000038 W

F2 - Processing parameters
SI                 262144
SF            600.1300080 MHz
WDW                    EM
SSB       0
LB                   0.10 Hz
GB        0
PC                   1.00
```

Expanded region of the ^{1}H NMR spectrum of compound 14

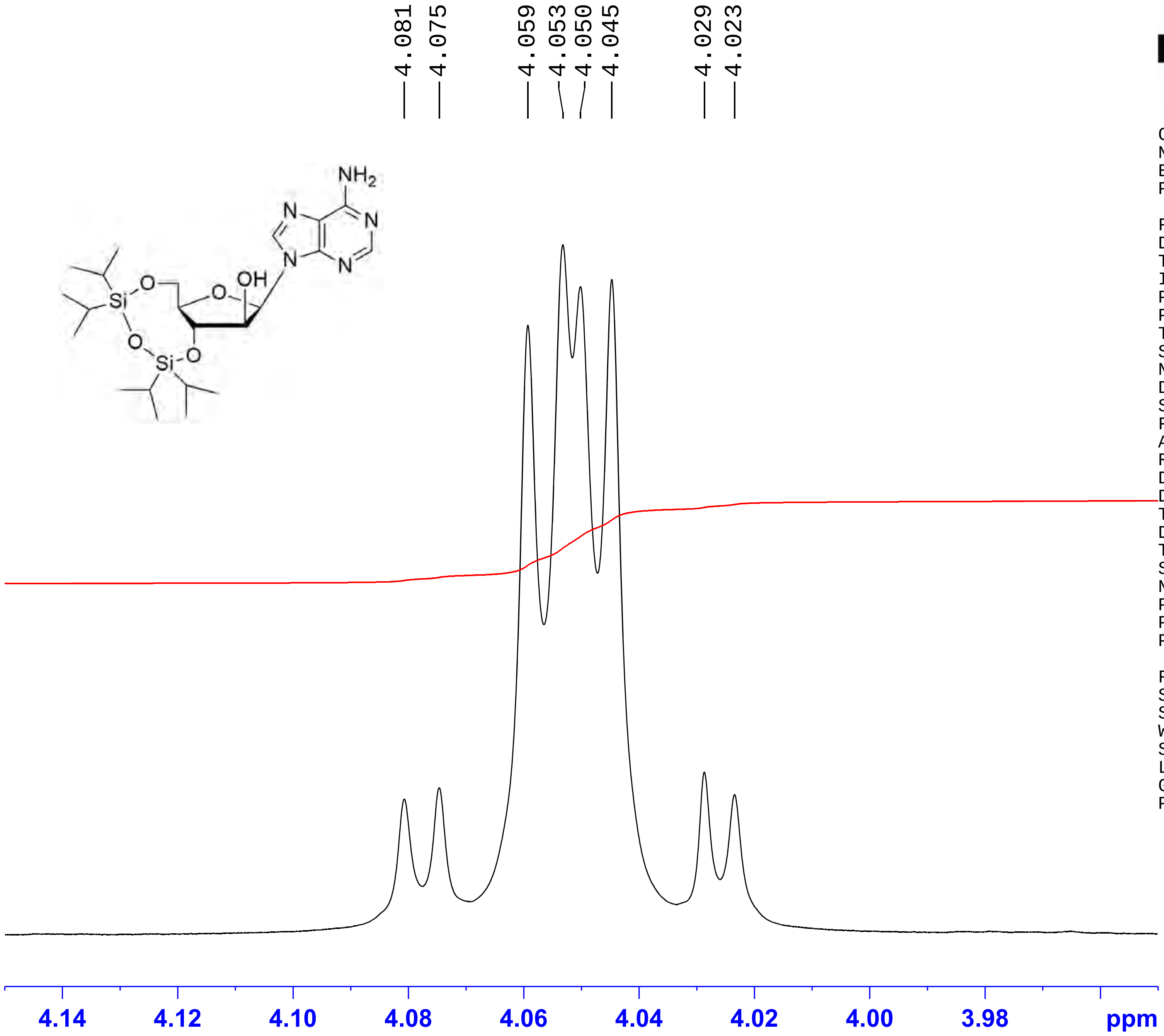


```
Current Data Parameters
NAME              LH-I-49
EXPNO                  10
PROCNO                  1

F2 - Acquisition Parameters
Date_            20220111
Time                11.19 h
INSTRUM             spect
PROBHD    Z114607_0188 (
PULPROG              zg30
TD                 180286
SOLVENT             CDCl3
NS                     16
DS                      0
SWH             18028.846 Hz
FIDRES           0.200003 Hz
AQ              4.9999318 sec
RG                  31.58
DW                 27.733 usec
DE                   8.00 usec
TE                  300.0 K
D1             0.10000000 sec
TD0                     1
SFO1          600.1337060 MHz
NUC1                   1H
P0                   3.33 usec
P1                  10.00 usec
PLW1          26.60000038 W

F2 - Processing parameters
SI                 262144
SF            600.1300080 MHz
WDW                    EM
SSB       0
LB                   0.10 Hz
GB        0
PC                   1.00
```

Expanded region of the ^{1}H NMR spectrum of compound 14

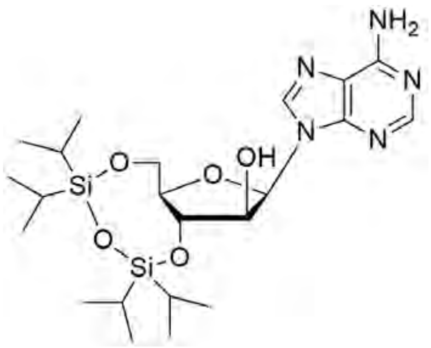

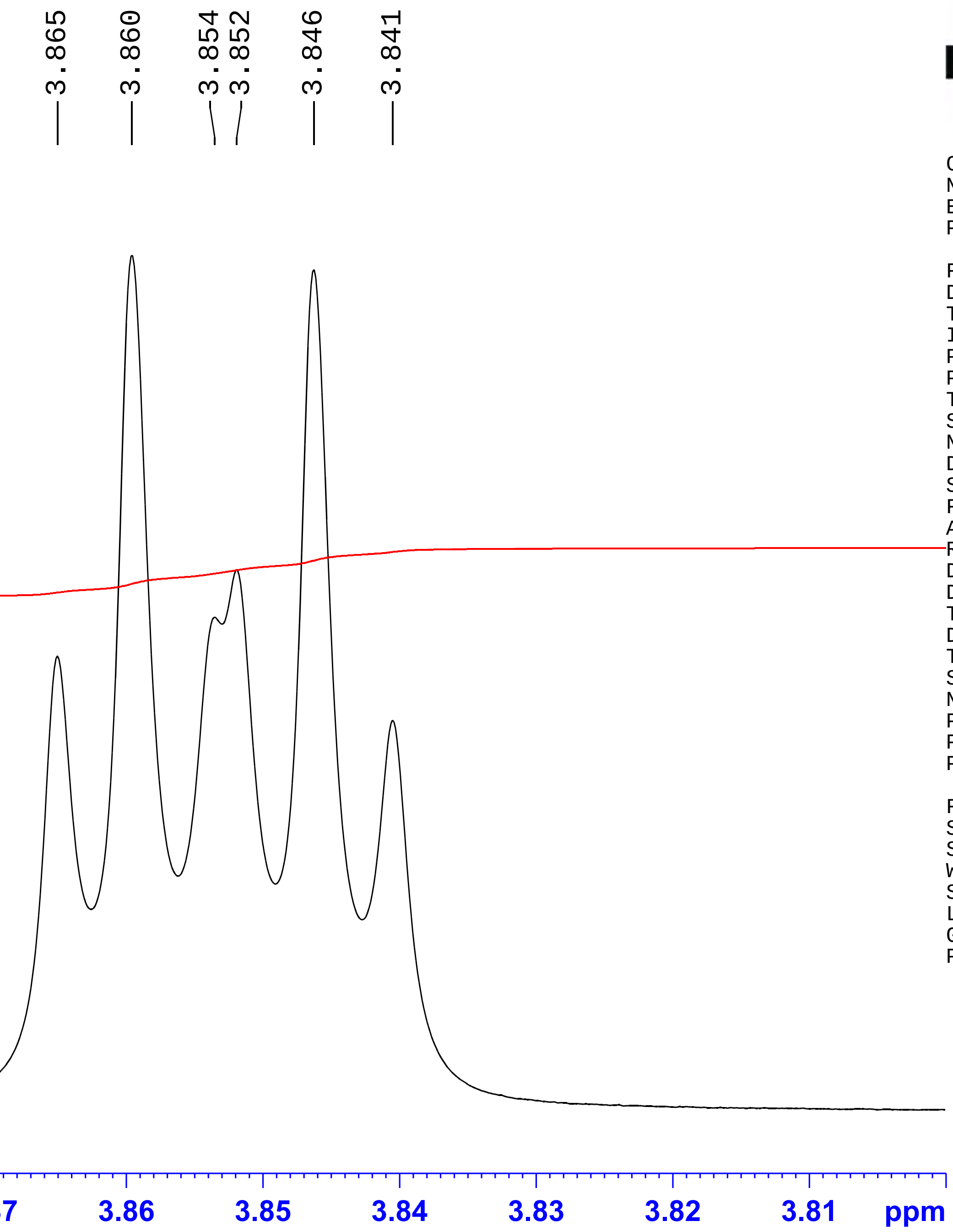


```
Current Data Parameters
NAME              LH-I-49
EXPNO                  10
PROCNO                  1

F2 - Acquisition Parameters
Date_            20220111
Time                11.19 h
INSTRUM             spect
PROBHD   Z114607_0188 (
PULPROG              zg30
TD                 180286
SOLVENT             CDCl3
NS                     16
DS                      0
SWH             18028.846 Hz
FIDRES           0.200003 Hz
AQ              4.9999318 sec
RG                  31.58
DW                 27.733 usec
DE                   8.00 usec
TE                  300.0 K
D1             0.10000000 sec
TD0                     1
SFO1          600.1337060 MHz
NUC1                   1H
P0                   3.33 usec
P1                  10.00 usec
PLW1          26.60000038 W

F2 - Processing parameters
SI                 262144
SF            600.1300080 MHz
WDW                    EM
SSB      0
LB                   0.10 Hz
GB       0
PC                   1.00
```

^{13}C NMR spectrum of compound 14

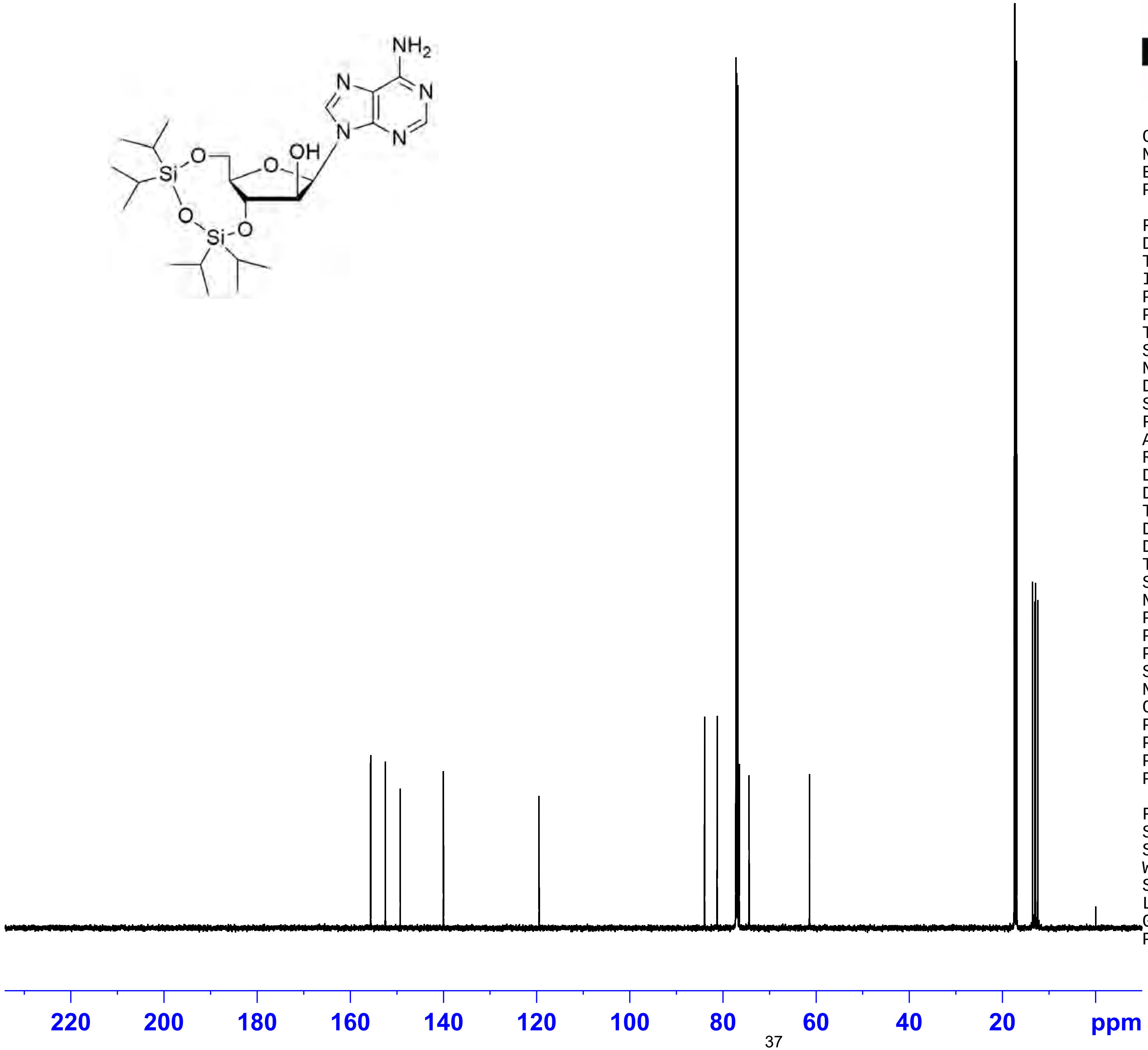

BRUKER

Current Data Parameters
NAME LH-I-49
EXPNO 11
PROCNO 1

F2 - Acquisition Parameters
Date_ 20220111
Time 12.04 h
INSTRUM spect
PROBHD Z114607_0188 (
PULPROG zgpg30
TD 119044
SOLVENT CDCl3
NS 1000
DS 4
SWH 37500.000 Hz
FIDRES 0.630019 Hz
AQ 1.5872533 sec
RG 186.92
DW 13.333 usec
DE 6.53 usec
TE 300.0 K
D1 1.00000000 sec
D11 0.03000000 sec
TD0 1
SFO1 150.9194058 MHz
NUC1 13C
P0 3.93 usec
P1 11.80 usec
PLW1 85.00000000 W
SFO2 600.1324005 MHz
NUC2 1H
CPDPRG[2 waltz64
PCPD2 70.00 usec
PLW2 27.00000000 W
PLW12 0.57327998 W
PLW13 0.28836000 W

F2 - Processing parameters
SI 131072
SF 150.9028141 MHz
WDW EM
SSB 0
LB 1.00 Hz
GB 0
PC 1.40

Expanded region of the ^{13}C NMR spectrum of compound 14

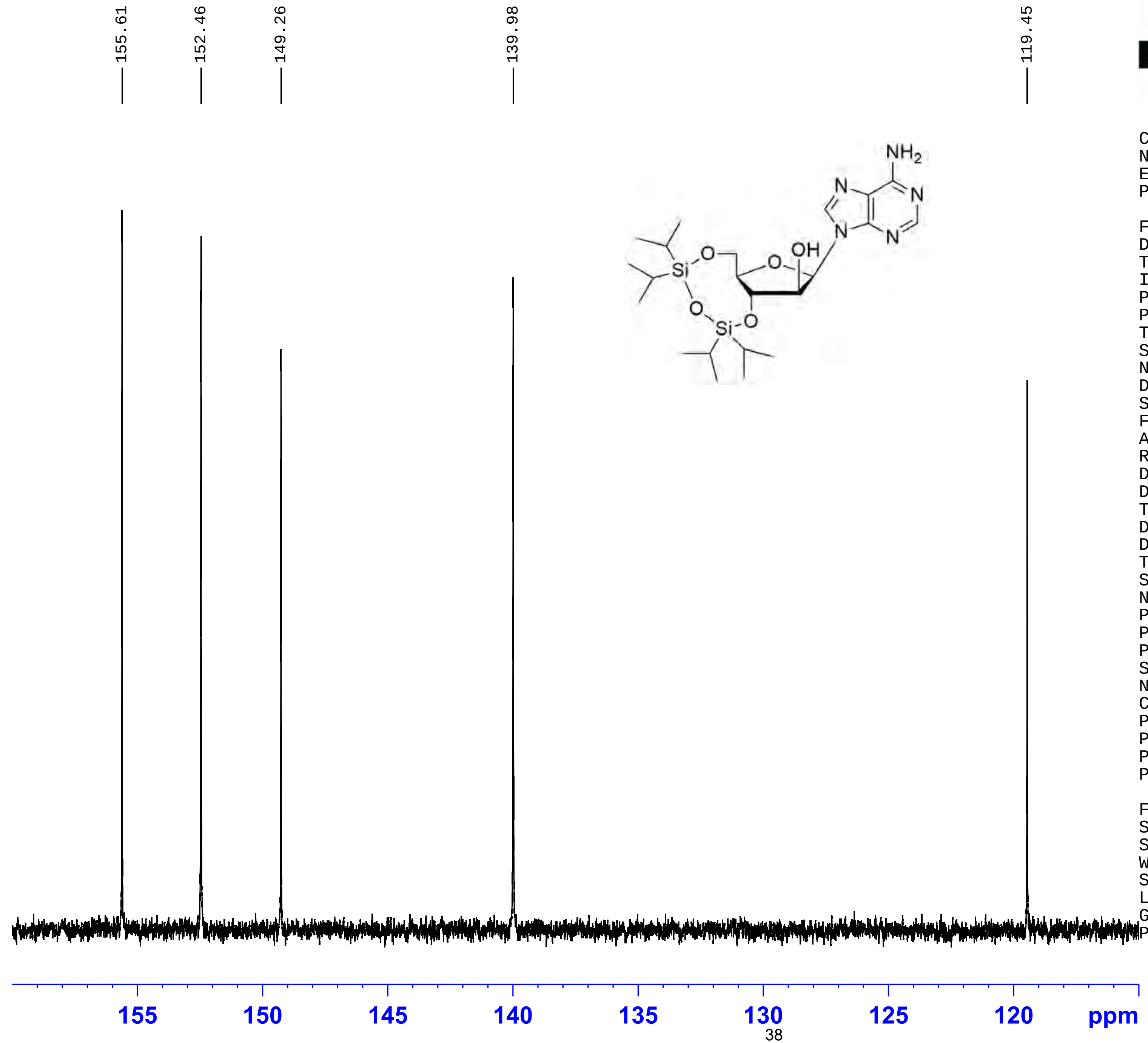

Current Data Parameters

NAME	LH-I-49
EXPNO	11
PROCNO	1

F2 - Acquisition Parameters

Date_	20220111
Time	12.04 h
INSTRUM	spect
PROBHD	Z114607_0188 (
PULPROG	zgpg30
TD	119044
SOLVENT	CDCl3
NS	1000
DS	4
SWH	37500.000 Hz
FIDRES	0.630019 Hz
AQ	1.5872533 sec
RG	186.92
DW	13.333 usec
DE	6.53 usec
TE	300.0 K
D1	1.00000000 sec
D11	0.03000000 sec
TD0	1
SFO1	150.9194058 MHz
NUC1	13C
P0	3.93 usec
P1	11.80 usec
PLW1	85.00000000 W
SFO2	600.1324005 MHz
NUC2	1H
CPDPRG[2	waltz64
PCPD2	70.00 usec
PLW2	27.00000000 W
PLW12	0.57327998 W
PLW13	0.28836000 W

F2 - Processing parameters

SI	131072
SF	150.9028141 MHz
WDW	EM
SSB	0
LB	1.00 Hz
GB	0
PC	1.40

Expanded region of the ^{13}C NMR spectrum of compound 14

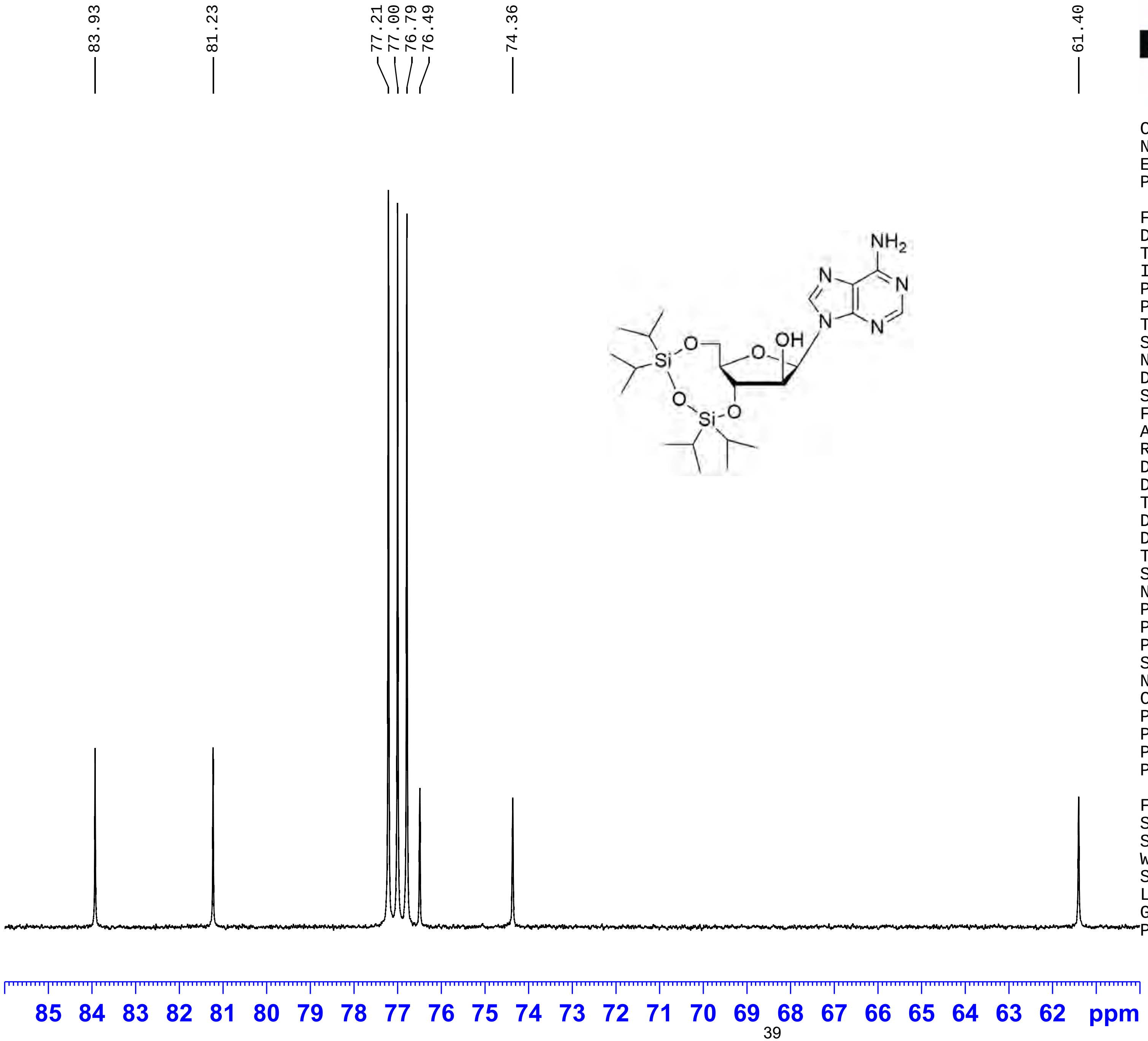


```
Current Data Parameters
NAME              LH-I-49
EXPNO                  11
PROCNO                  1

F2 - Acquisition Parameters
Date_            20220111
Time                12.04 h
INSTRUM             spect
PROBHD   Z114607_0188 (
PULPROG           zgpg30
TD                119044
SOLVENT            CDCl3
NS                  1000
DS                     4
SWH            37500.000 Hz
FIDRES          0.630019 Hz
AQ             1.5872533 sec
RG                186.92
DW                13.333 usec
DE                  6.53 usec
TE                 300.0 K
D1            1.00000000 sec
D11           0.03000000 sec
TD0                    1
SFO1         150.9194058 MHz
NUC1                 13C
P0                  3.93 usec
P1                 11.80 usec
PLW1         85.00000000 W
SFO2         600.1324005 MHz
NUC2                  1H
CPDPRG[2         waltz64
PCPD2              70.00 usec
PLW2         27.00000000 W
PLW12         0.57327998 W
PLW13         0.28836000 W

F2 - Processing parameters
SI                131072
SF           150.9028141 MHz
WDW                   EM
SSB      0
LB                  1.00 Hz
GB       0
PC                  1.40
```

Expanded region of the ^{13}C NMR spectrum of compound 14

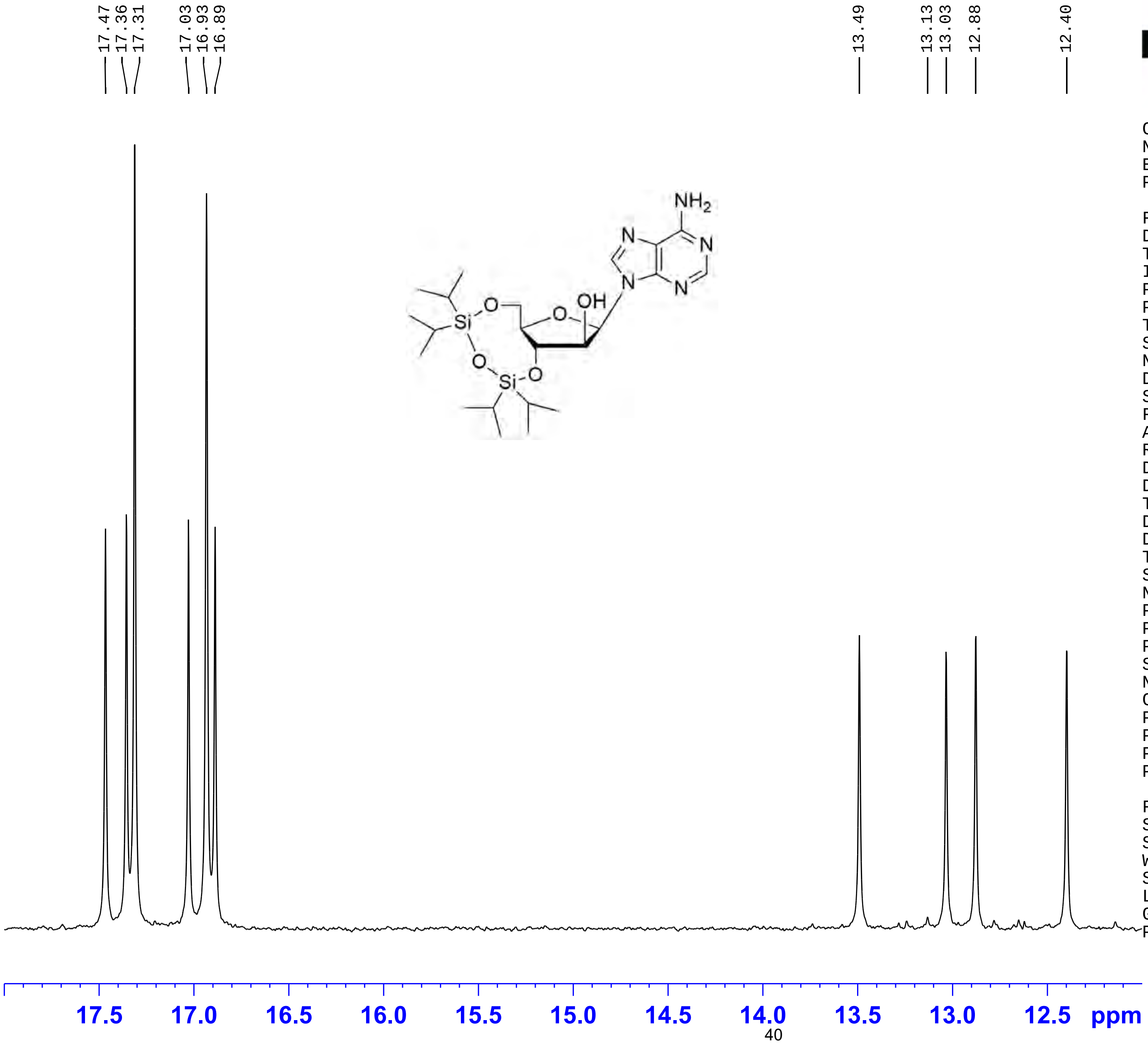


```
Current Data Parameters
NAME              LH-I-49
EXPNO                  11
PROCNO                  1

F2 - Acquisition Parameters
Date_            20220111
Time                12.04 h
INSTRUM             spect
PROBHD    Z114607_0188 (
PULPROG           zgpg30
TD                119044
SOLVENT            CDCl3
NS                  1000
DS                     4
SWH            37500.000 Hz
FIDRES          0.630019 Hz
AQ             1.5872533 sec
RG                186.92
DW                13.333 usec
DE                  6.53 usec
TE                 300.0 K
D1            1.00000000 sec
D11           0.03000000 sec
TD0                    1
SFO1         150.9194058 MHz
NUC1                 13C
P0                  3.93 usec
P1                 11.80 usec
PLW1         85.00000000 W
SFO2         600.1324005 MHz
NUC2                  1H
CPDPRG[2         waltz64
PCPD2              70.00 usec
PLW2         27.00000000 W
PLW12         0.57327998 W
PLW13         0.28836000 W

F2 - Processing parameters
SI                131072
SF           150.9028141 MHz
WDW                   EM
SSB      0
LB                  1.00 Hz
GB       0
PC                  1.40
```

^{13}C DEPT-135 NMR spectrum of compound 14

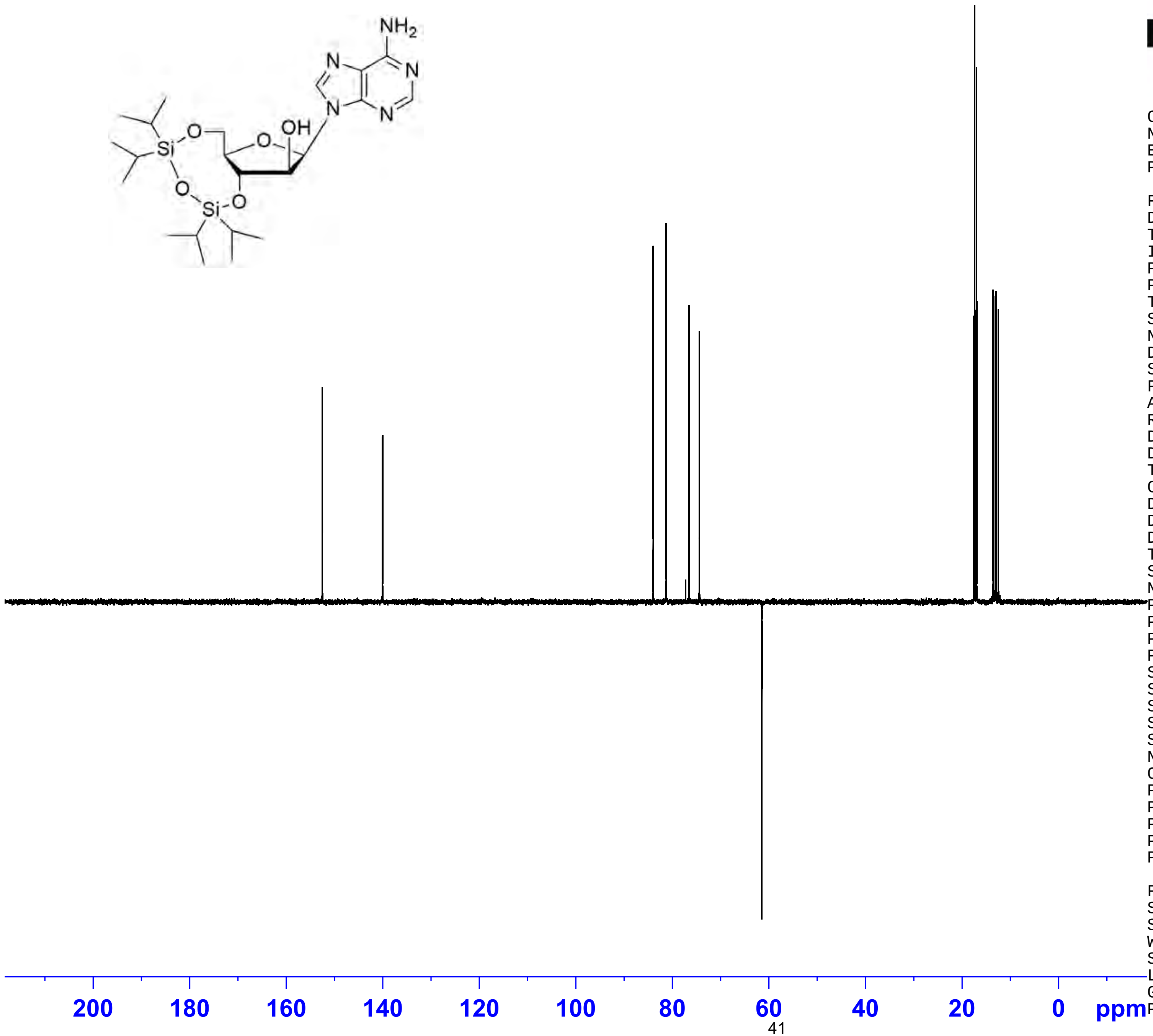

Current Data Parameters
NAME LH-I-49
EXPNO 12
PROCNO 1

F2 - Acquisition Parameters
Date_ 20220111
Time 12.27 h
INSTRUM spect
PROBHD Z114607_0188 (
PULPROG deptsp135.b
TD 119044
SOLVENT CDCl3
NS 500
DS 4
SWH 35714.285 Hz
FIDRES 0.600018 Hz
AQ 1.6666160 sec
RG 186.92
DW 14.000 usec
DE 7.44 usec
TE 300.0 K
CNST2 145.0000000
D1 1.00000000 sec
D2 0.00344828 sec
D12 0.00002000 sec
TD0 1
SFO1 150.9178962 MHz
NUC1 13C
P1 11.80 usec
P13 2000.00 usec
PLW0 0 W
PLW1 85.00000000 W
SPNAM[5] Crp60comp.4
SPOAL5 0.500
SPOFFS5 0 Hz
SPW5 18.08300018 W
SFO2 600.1324005 MHz
NUC2 1H
CPDPRG[2 waltz64
P3 10.20 usec
P4 20.40 usec
PCPD2 70.00 usec
PLW2 27.00000000 W
PLW12 0.57327998 W

F2 - Processing parameters
SI 131072
SF 150.9028085 MHz
WDW EM
SSB 0
LB 1.00 Hz
GB 0
PC 1.40

^{1}H-^{1}H COSY NMR spectrum of compound 14

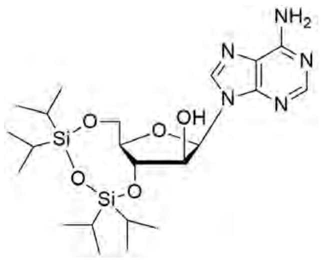

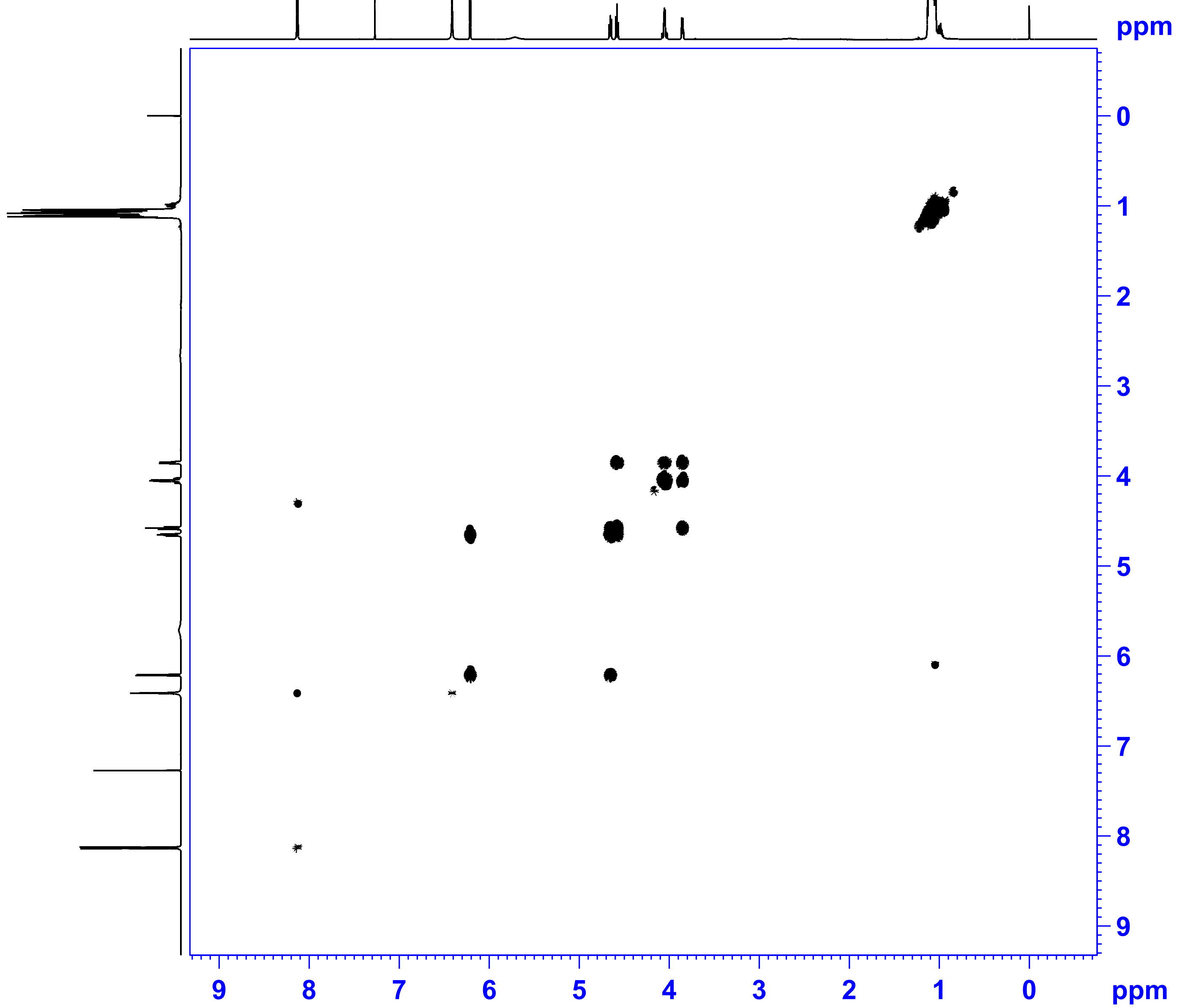


```
Current Data Parameters
NAME              LH-I-49
EXPNO                  13
PROCNO                  1

F2 - Acquisition Parameters
Date_            20220111
Time                12.38 h
INSTRUM             spect
PROBHD    Z114607_0188 (
PULPROG     cosygpmfppqf
TD                   2048
SOLVENT             CDCl3
NS                      2
DS                      8
SWH              6048.387 Hz
FIDRES           5.906628 Hz
AQ              0.1693013 sec
RG                 186.92
DW                 82.667 usec
DE                   6.50 usec
TE                  300.0 K
D0             0.00000300 sec
D1             0.89145529 sec
D11            0.03000000 sec
D12            0.00002000 sec
D13            0.00000400 sec
D16            0.00020000 sec
IN0            0.00016540 sec
TDav                    1
SFO1          600.1325802 MHz
NUC1                   1H
P1                  10.00 usec
P17               2500.00 usec
PLW1          26.60000038 W
PLW10          4.25600004 W
GPNAM[1]       SMSQ10.100
GPZ1                16.00 %
GPNAM[2]       SMSQ10.100
GPZ2                12.00 %
GPNAM[3]       SMSQ10.100
GPZ3                40.00 %
P16               1000.00 usec

F1 - Acquisition parameters
TD                    256
SFO1             600.1326 MHz
FIDRES          47.233978 Hz
SW                 10.074 ppm
FnMODE                 QF

F2 - Processing parameters
SI                   1024
SF            600.1300080 MHz
WDW                  SINE
SSB      0
LB       0 Hz
GB       0
PC                   1.40

F1 - Processing parameters
SI                   1024
MC2                    QF
SF            600.1300074 MHz
WDW                  SINE
SSB      0
LB       0 Hz
GB       0
```

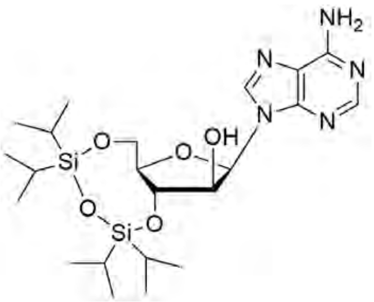

^{1}H-^{13}C HSQC NMR spectrum of compound 14

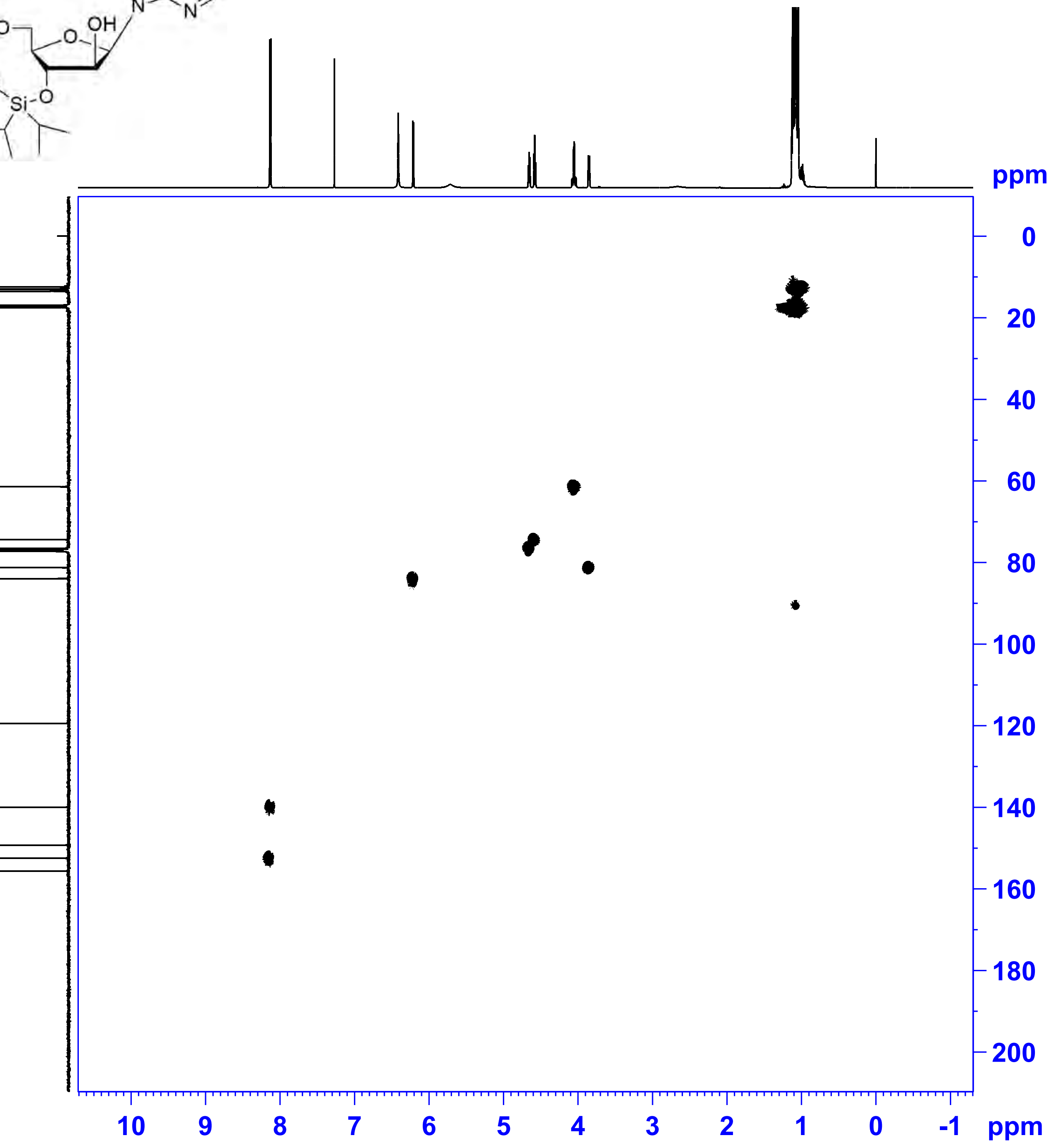


```
Current Data Parameters
NAME              LH-I-49
EXPNO                  14
PROCNO                  1

F2 - Acquisition Parameters
Date_            20220111
Time                12.47 h
INSTRUM             spect
PROBHD   Z114607_0188 (
PULPROG  hsqcedetgpsp.3
TD                   1024
SOLVENT             CDCl3
NS                      2
DS                     32
SWH              7211.539 Hz
FIDRES          14.085036 Hz
AQ              0.0709973 sec
RG                 186.92
DW                 69.333 usec
DE                   6.50 usec
TE                  300.2 K
CNST2       145.0000000
D0           0.00000300 sec
D1           0.80000001 sec
D4           0.00172414 sec
D11          0.03000000 sec
D16          0.00020000 sec
D21          0.00360000 sec
IN0          0.00001510 sec
TDav                    1
ZGOPTNS
SFO1        600.1328223 MHz
NUC1                   1H
P1                  10.00 usec
P2                  20.00 usec
PLW1         26.60000038 W
SFO2        150.9178988 MHz
NUC2                  13C
CPDPRG[2            garp4
P3                  11.80 usec
P14                500.00 usec
P31               1730.00 usec
PCPD2               60.00 usec
PLW0     0 W
PLW2         85.00000000 W
PLW12         3.28760004 W
SPNAM[3] Crp60,0.5,20.1
SPOAL3              0.500
SPOFFS3  0 Hz
SPW3         18.08300018 W
SPNAM[18  Crp60_xfilt.2
SPOAL18             0.500
SPOFFS18 0 Hz
SPW18         5.22629976 W
GPNAM[1]      SMSQ10.100
GPZ1                80.00 %
GPNAM[2]      SMSQ10.100
GPZ2                20.10 %
P16               1000.00 usec

F1 - Acquisition parameters
TD                    256
SFO1             150.9179 MHz
FIDRES         258.692047 Hz
SW                219.408 ppm
FnMODE     Echo-Antiecho

F2 - Processing parameters
SI                   1024
SF          600.1300000 MHz
WDW                 QSINE
SSB                     2
LB       0 Hz
GB       0
PC                   1.40

F1 - Processing parameters
SI                   1024
MC2          echo-antiecho
SF          150.9028085 MHz
WDW                 QSINE
SSB                     2
LB       0 Hz
GB       0
```

^{1}H NMR spectrum of compound 10

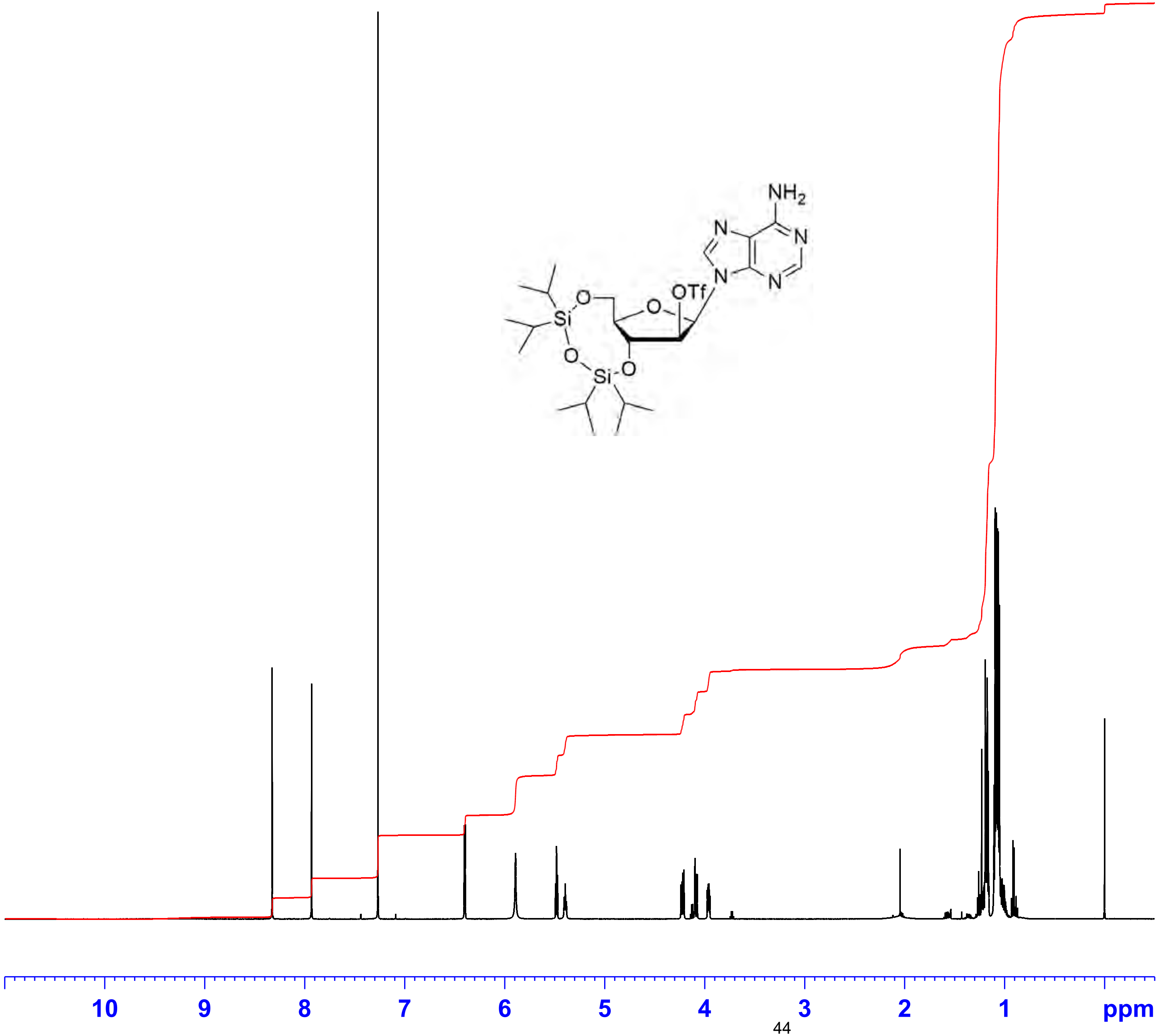

Current Data Parameters
NAME LH-I-50
EXPNO 10
PROCNO 1

F2 - Acquisition Parameters
Date_ 20220111
Time 20.01 h
INSTRUM spect
PROBHD Z114607_0188 (
PULPROG zg30
TD 180286
SOLVENT CDCl3
NS 16
DS 0
SWH 18028.846 Hz
FIDRES 0.200003 Hz
AQ 4.9999318 sec
RG 55.43
DW 27.733 usec
DE 8.00 usec
TE 300.0 K
D1 0.10000000 sec
TD0 1
SFO1 600.1337060 MHz
NUC1 1H
P0 3.33 usec
P1 10.00 usec
PLW1 26.60000038 W

F2 - Processing parameters
SI 262144
SF 600.1300101 MHz
WDW EM
SSB 0
LB 0.10 Hz
GB 0
PC 1.00

Expanded region of the ^1H NMR spectrum of compound 10

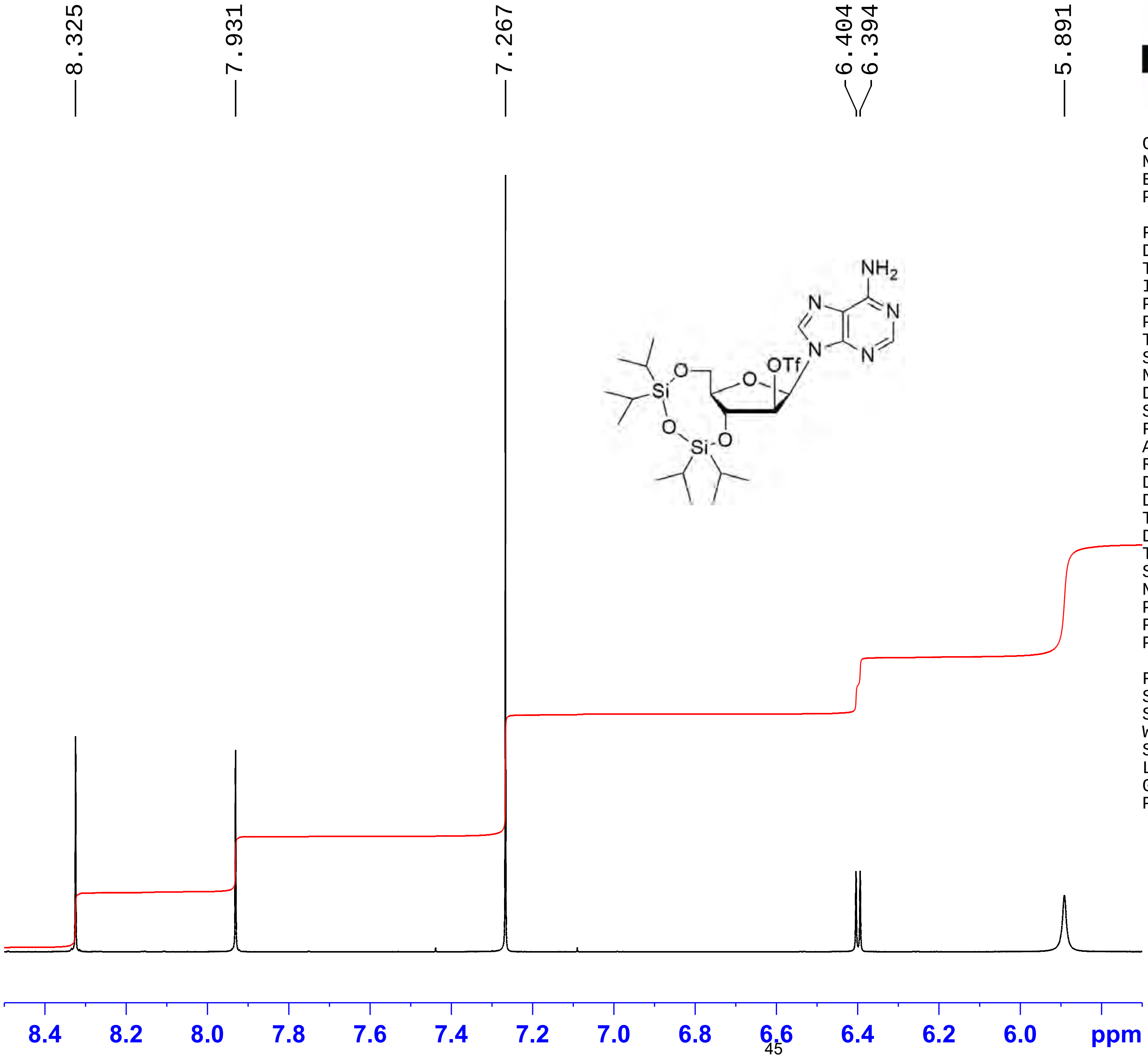

Current Data Parameters
NAME LH-I-50
EXPNO 10
PROCNO 1

F2 - Acquisition Parameters
Date_ 20220111
Time 20.01 h
INSTRUM spect
PROBHD Z114607_0188 (
PULPROG zg30
TD 180286
SOLVENT CDCl3
NS 16
DS 0
SWH 18028.846 Hz
FIDRES 0.200003 Hz
AQ 4.9999318 sec
RG 55.43
DW 27.733 usec
DE 8.00 usec
TE 300.0 K
D1 0.10000000 sec
TD0 1
SFO1 600.1337060 MHz
NUC1 1H
P0 3.33 usec
P1 10.00 usec
PLW1 26.60000038 W

F2 - Processing parameters
SI 262144
SF 600.1300101 MHz
WDW EM
SSB 0
LB 0.10 Hz
GB 0
PC 1.00

Expanded region of the ^{1}H NMR spectrum of compound 10

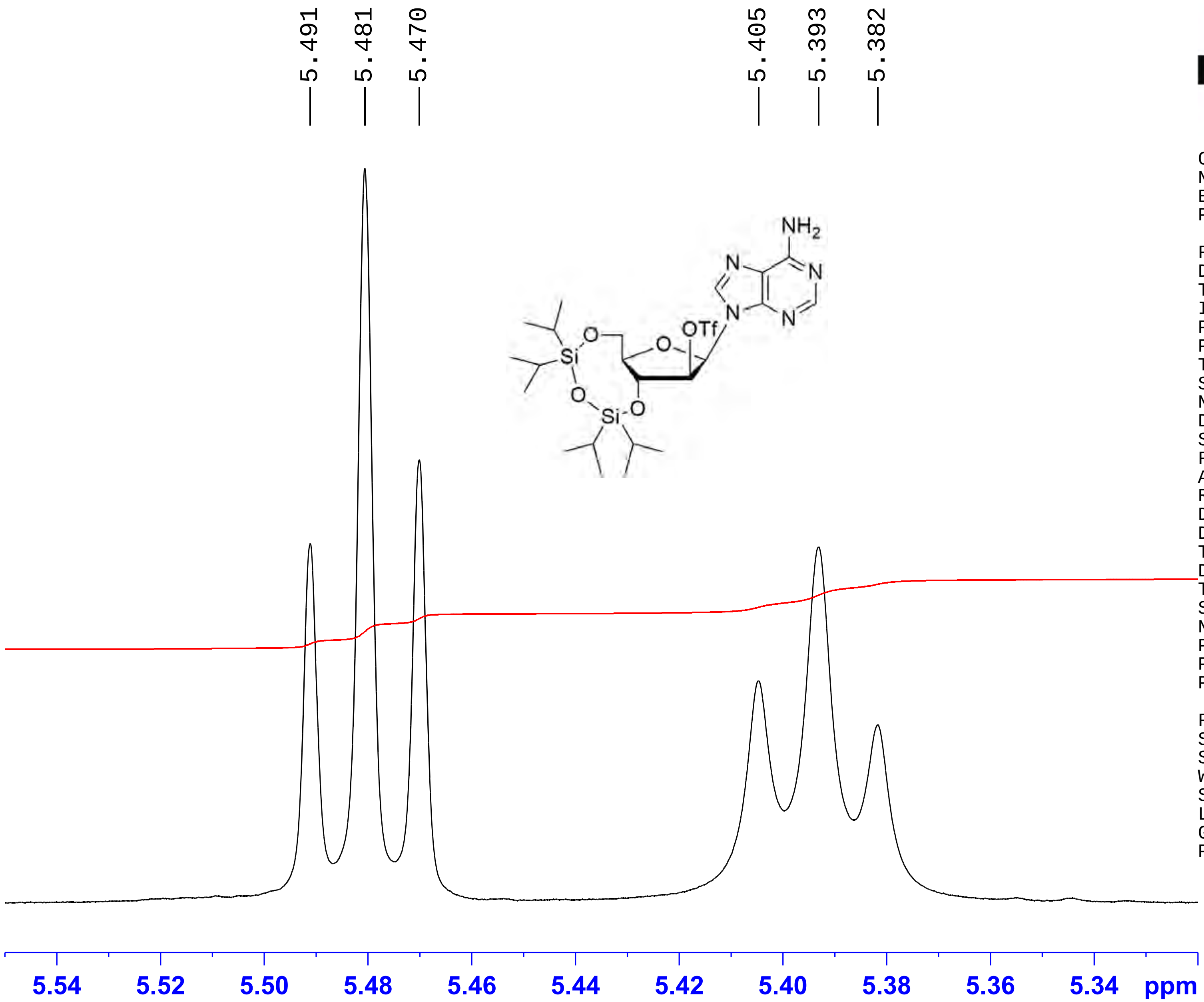

Current Data Parameters
NAME LH-I-50
EXPNO 10
PROCNO 1

F2 - Acquisition Parameters
Date_ 20220111
Time 20.01 h
INSTRUM spect
PROBHD Z114607_0188 (
PULPROG zg30
TD 180286
SOLVENT CDCl3
NS 16
DS 0
SWH 18028.846 Hz
FIDRES 0.200003 Hz
AQ 4.9999318 sec
RG 55.43
DW 27.733 usec
DE 8.00 usec
TE 300.0 K
D1 0.10000000 sec
TD0 1
SFO1 600.1337060 MHz
NUC1 1H
P0 3.33 usec
P1 10.00 usec
PLW1 26.60000038 W

F2 - Processing parameters
SI 262144
SF 600.1300101 MHz
WDW EM
SSB 0
LB 0.10 Hz
GB 0
PC 1.00

Expanded region of the ^{1}H NMR spectrum of compound 10

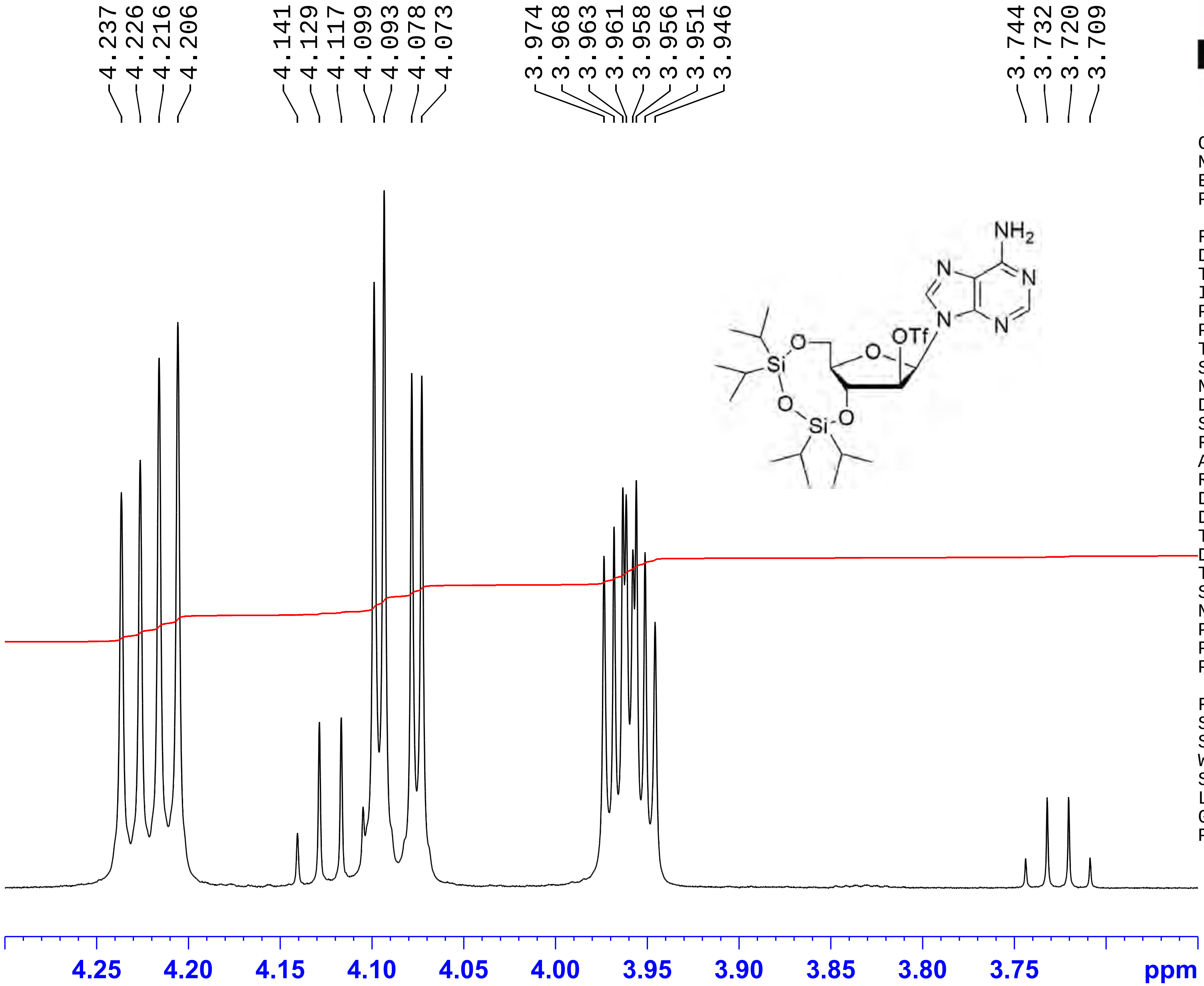

Current Data Parameters
NAME LH-I-50
EXPNO 10
PROCNO 1

F2 - Acquisition Parameters
Date_ 20220111
Time 20.01 h
INSTRUM spect
PROBHD Z114607_0188 (
PULPROG zg30
TD 180286
SOLVENT CDCl3
NS 16
DS 0
SWH 18028.846 Hz
FIDRES 0.200003 Hz
AQ 4.9999318 sec
RG 55.43
DW 27.733 usec
DE 8.00 usec
TE 300.0 K
D1 0.10000000 sec
TD0 1
SFO1 600.1337060 MHz
NUC1 1H
P0 3.33 usec
P1 10.00 usec
PLW1 26.60000038 W

F2 - Processing parameters
SI 262144
SF 600.1300101 MHz
WDW EM
SSB 0
LB 0.10 Hz
GB 0
PC 1.00

Expanded region of the ^{1}H NMR spectrum of compound 10

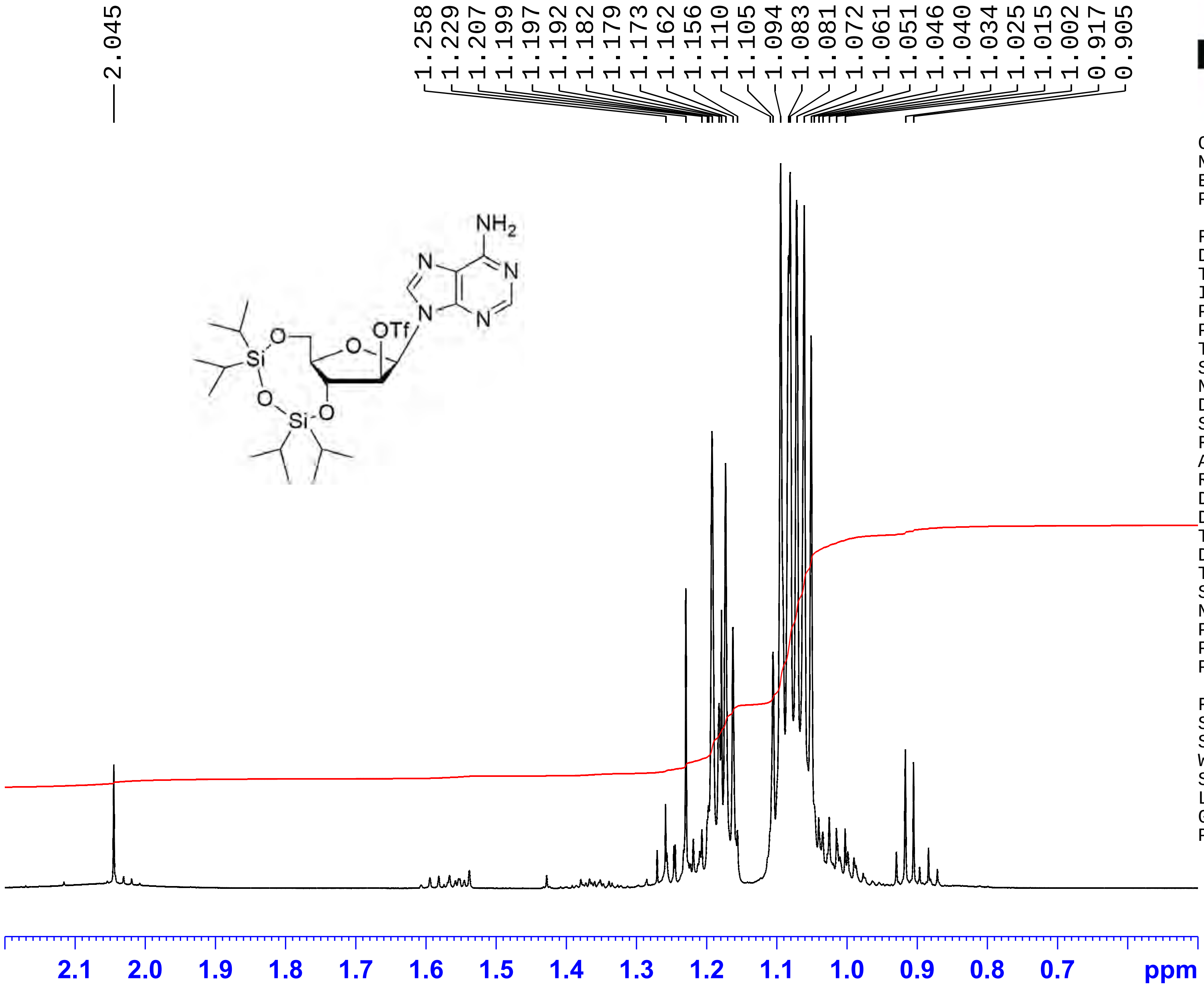


```
Current Data Parameters
NAME              LH-I-50
EXPNO                  10
PROCNO                  1

F2 - Acquisition Parameters
Date_            20220111
Time                20.01 h
INSTRUM             spect
PROBHD   Z114607_0188 (
PULPROG              zg30
TD                 180286
SOLVENT             CDCl3
NS                     16
DS                      0
SWH             18028.846 Hz
FIDRES           0.200003 Hz
AQ              4.9999318 sec
RG                  55.43
DW                 27.733 usec
DE                   8.00 usec
TE                  300.0 K
D1             0.10000000 sec
TD0                     1
SFO1          600.1337060 MHz
NUC1                   1H
P0                   3.33 usec
P1                  10.00 usec
PLW1          26.60000038 W

F2 - Processing parameters
SI                 262144
SF            600.1300101 MHz
WDW                    EM
SSB      0
LB                   0.10 Hz
GB       0
PC                   1.00
```

^{13}C NMR spectrum of compound 10

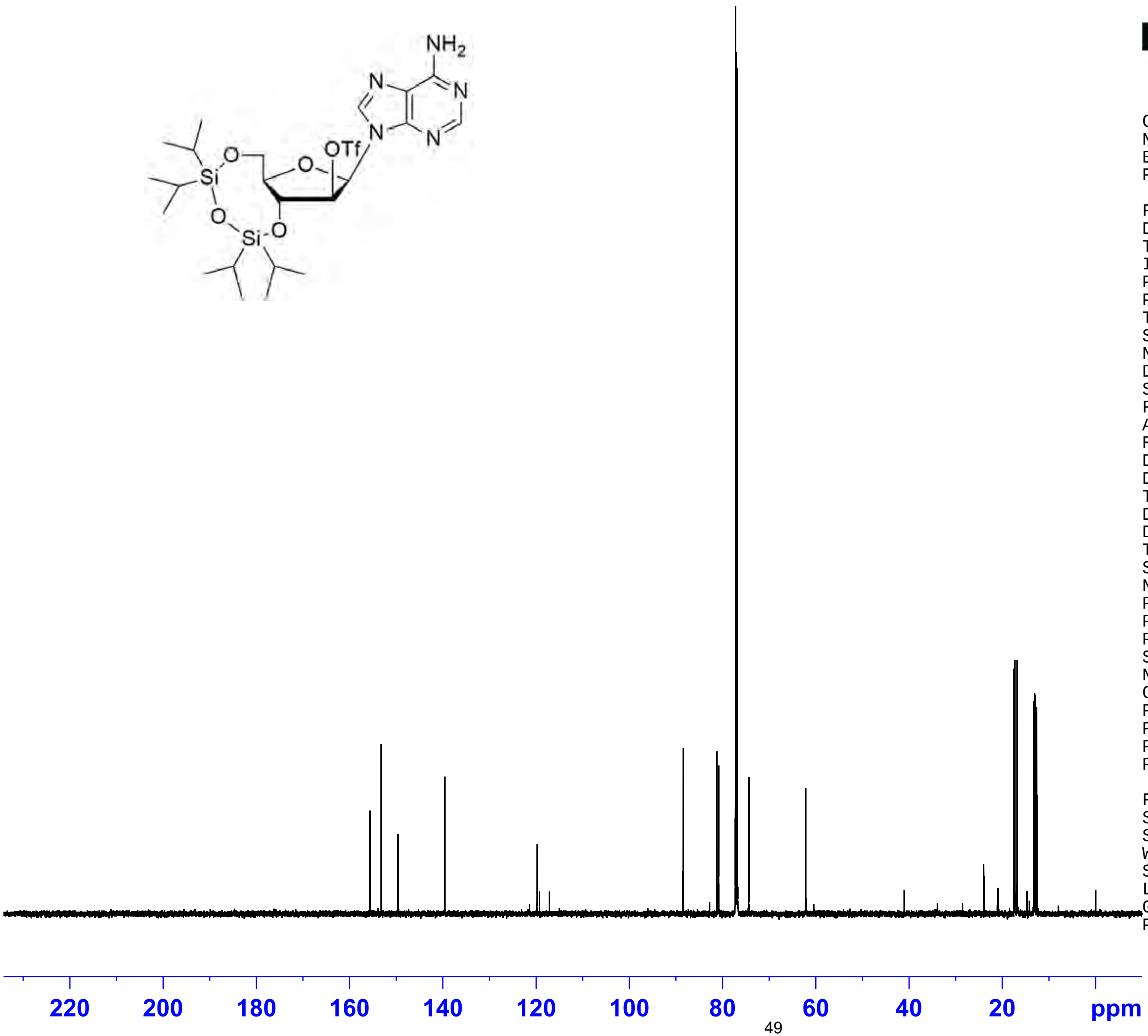

Current Data Parameters
NAME LH-I-50
EXPNO 11
PROCNO 1

F2 - Acquisition Parameters
Date_ 20220111
Time 20.46 h
INSTRUM spect
PROBHD Z114607_0188 (
PULPROG zgpg30
TD 119044
SOLVENT CDCl3
NS 1000
DS 4
SWH 37500.000 Hz
FIDRES 0.630019 Hz
AQ 1.5872533 sec
RG 186.92
DW 13.333 usec
DE 6.53 usec
TE 300.0 K
D1 1.00000000 sec
D11 0.03000000 sec
TD0 1
SFO1 150.9194058 MHz
NUC1 13C
P0 3.93 usec
P1 11.80 usec
PLW1 85.00000000 W
SFO2 600.1324005 MHz
NUC2 1H
CPDPRG[2 waltz64
PCPD2 70.00 usec
PLW2 27.00000000 W
PLW12 0.57327998 W
PLW13 0.28836000 W

F2 - Processing parameters
SI 131072
SF 150.9028118 MHz
WDW EM
SSB 0
LB 1.00 Hz
GB 0
PC 1.40

Expanded region of the ^{13}C NMR spectrum of compound 10

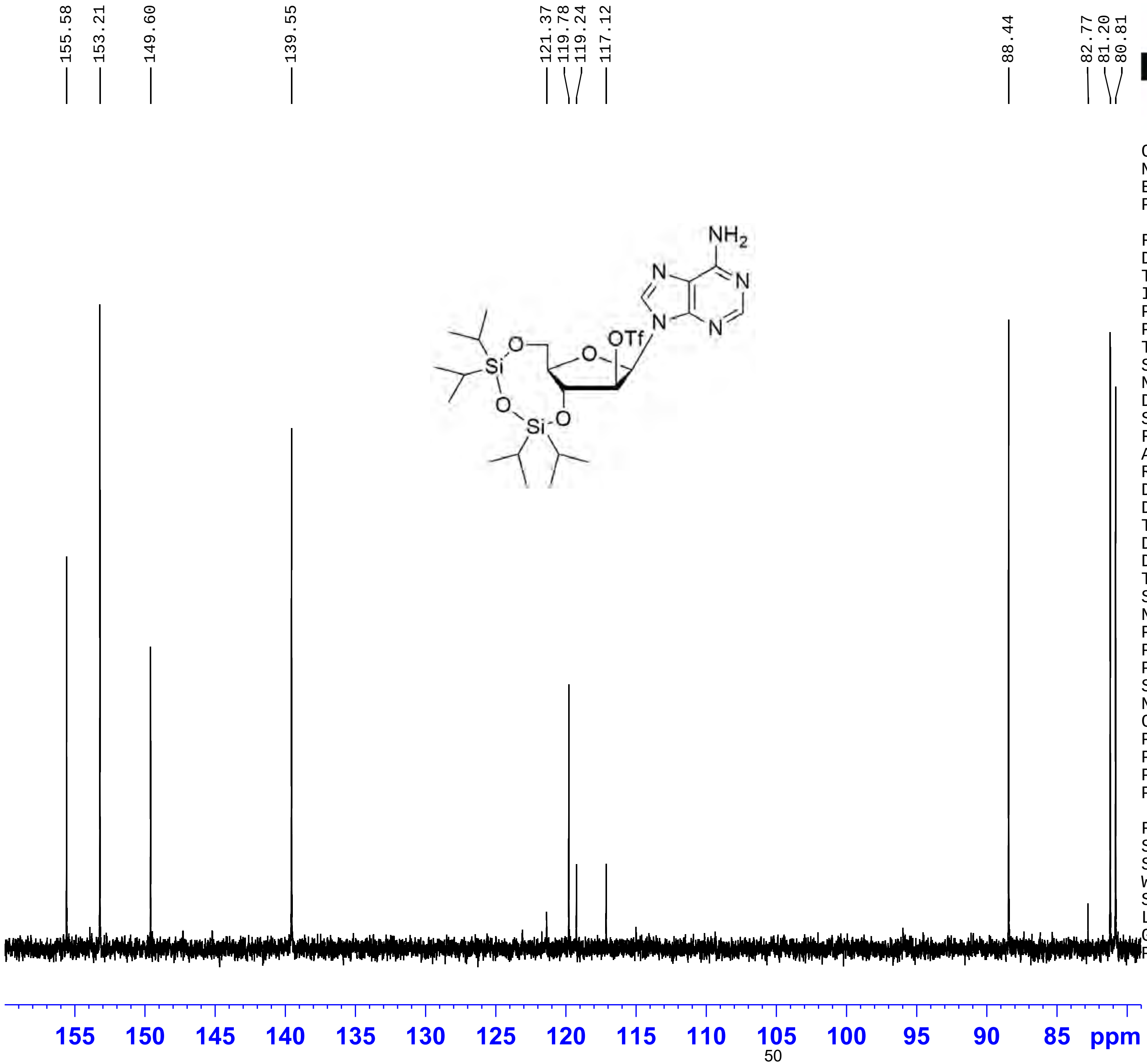


```
Current Data Parameters
NAME              LH-I-50
EXPNO                  11
PROCNO                  1

F2 - Acquisition Parameters
Date_            20220111
Time                20.46 h
INSTRUM             spect
PROBHD   Z114607_0188 (
PULPROG           zgpg30
TD                119044
SOLVENT            CDCl3
NS                  1000
DS                     4
SWH            37500.000 Hz
FIDRES          0.630019 Hz
AQ             1.5872533 sec
RG                186.92
DW                13.333 usec
DE                  6.53 usec
TE                 300.0 K
D1            1.00000000 sec
D11           0.03000000 sec
TD0                    1
SFO1         150.9194058 MHz
NUC1                 13C
P0                  3.93 usec
P1                 11.80 usec
PLW1         85.00000000 W
SFO2         600.1324005 MHz
NUC2                  1H
CPDPRG[2          waltz64
PCPD2              70.00 usec
PLW2         27.00000000 W
PLW12         0.57327998 W
PLW13         0.28836000 W

F2 - Processing parameters
SI                131072
SF           150.9028118 MHz
WDW                   EM
SSB      0
LB                  1.00 Hz
GB       0
PC                  1.40
```

Expanded region of the ^{13}C NMR spectrum of compound 10

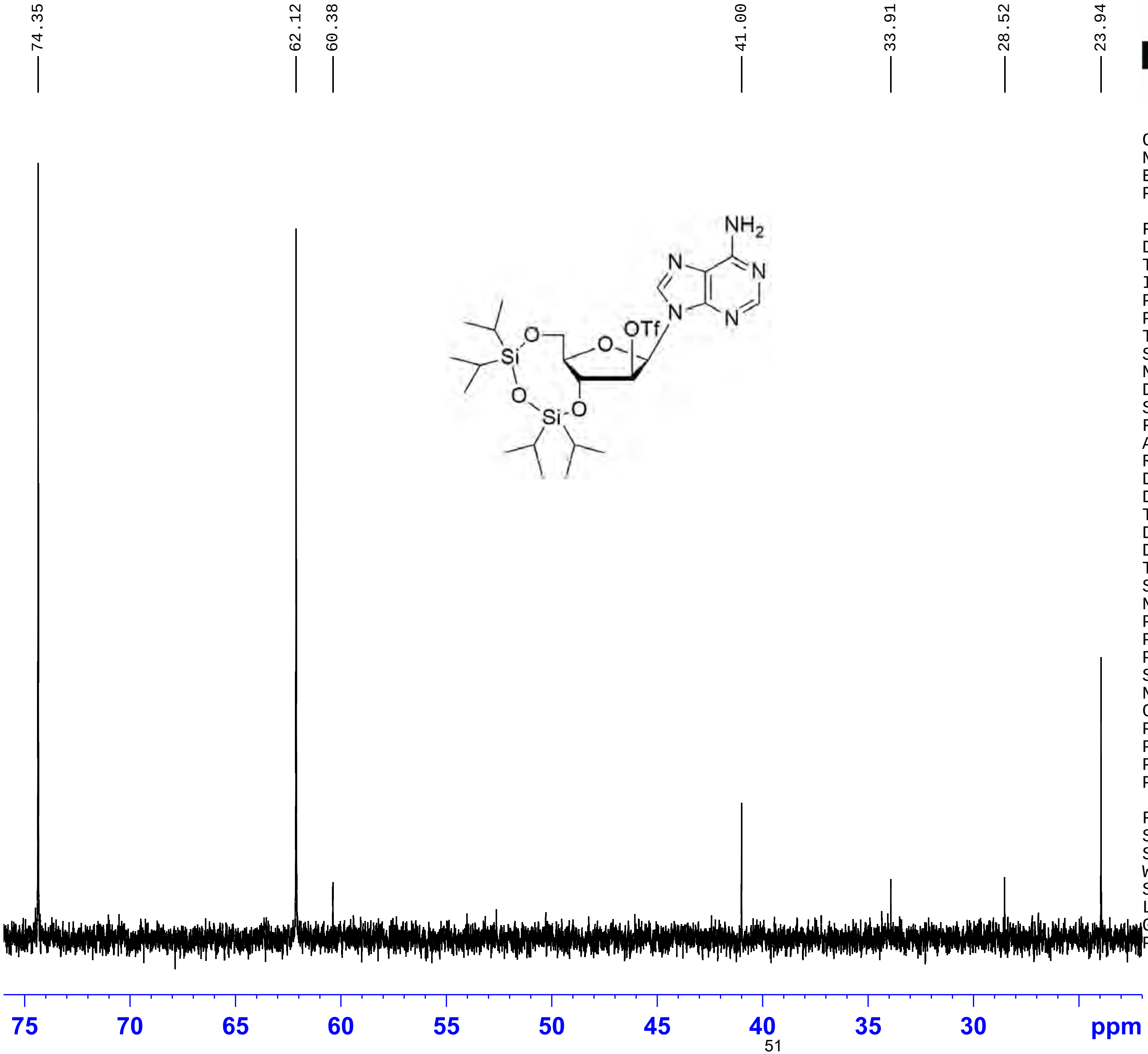

Current Data Parameters
NAME LH-I-50
EXPNO 11
PROCNO 1

F2 - Acquisition Parameters
Date_ 20220111
Time 20.46 h
INSTRUM spect
PROBHD Z114607_0188 (
PULPROG zgpg30
TD 119044
SOLVENT CDCl3
NS 1000
DS 4
SWH 37500.000 Hz
FIDRES 0.630019 Hz
AQ 1.5872533 sec
RG 186.92
DW 13.333 usec
DE 6.53 usec
TE 300.0 K
D1 1.00000000 sec
D11 0.03000000 sec
TD0 1
SFO1 150.9194058 MHz
NUC1 13C
P0 3.93 usec
P1 11.80 usec
PLW1 85.00000000 W
SFO2 600.1324005 MHz
NUC2 1H
CPDPRG[2 waltz64
PCPD2 70.00 usec
PLW2 27.00000000 W
PLW12 0.57327998 W
PLW13 0.28836000 W

F2 - Processing parameters
SI 131072
SF 150.9028118 MHz
WDW EM
SSB 0
LB 1.00 Hz
GB 0
PC 1.40

Expanded region of the ^{13}C NMR spectrum of compound 10

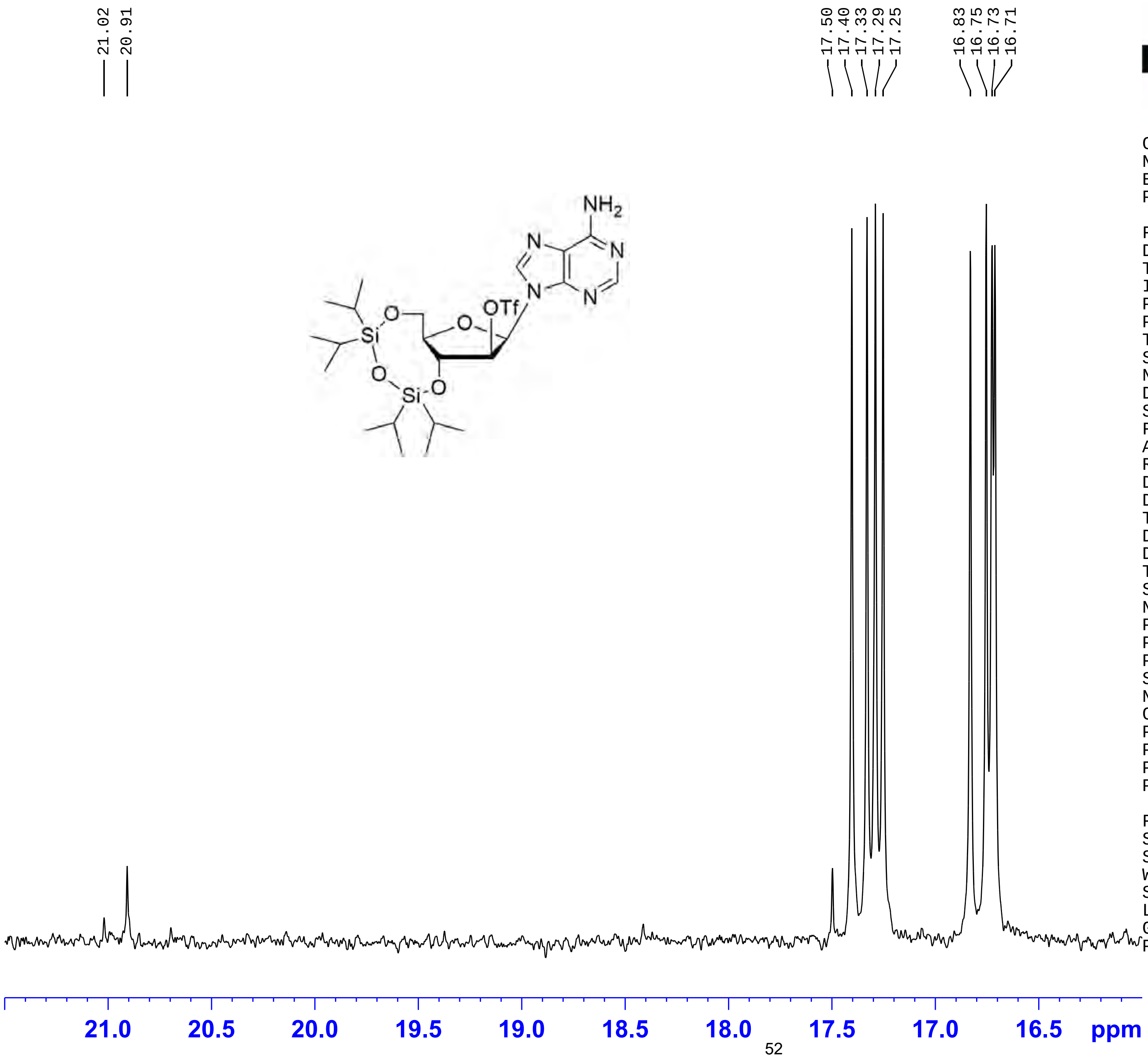

Current Data Parameters
NAME LH-I-50
EXPNO 11
PROCNO 1

F2 - Acquisition Parameters
Date_ 20220111
Time 20.46 h
INSTRUM spect
PROBHD Z114607_0188 (
PULPROG zgpg30
TD 119044
SOLVENT CDCl3
NS 1000
DS 4
SWH 37500.000 Hz
FIDRES 0.630019 Hz
AQ 1.5872533 sec
RG 186.92
DW 13.333 usec
DE 6.53 usec
TE 300.0 K
D1 1.00000000 sec
D11 0.03000000 sec
TD0 1
SFO1 150.9194058 MHz
NUC1 13C
P0 3.93 usec
P1 11.80 usec
PLW1 85.00000000 W
SFO2 600.1324005 MHz
NUC2 1H
CPDPRG[2 waltz64
PCPD2 70.00 usec
PLW2 27.00000000 W
PLW12 0.57327998 W
PLW13 0.28836000 W

F2 - Processing parameters
SI 131072
SF 150.9028118 MHz
WDW EM
SSB 0
LB 1.00 Hz
GB 0
PC 1.40

Expanded region of the ^{13}C NMR spectrum of compound 10

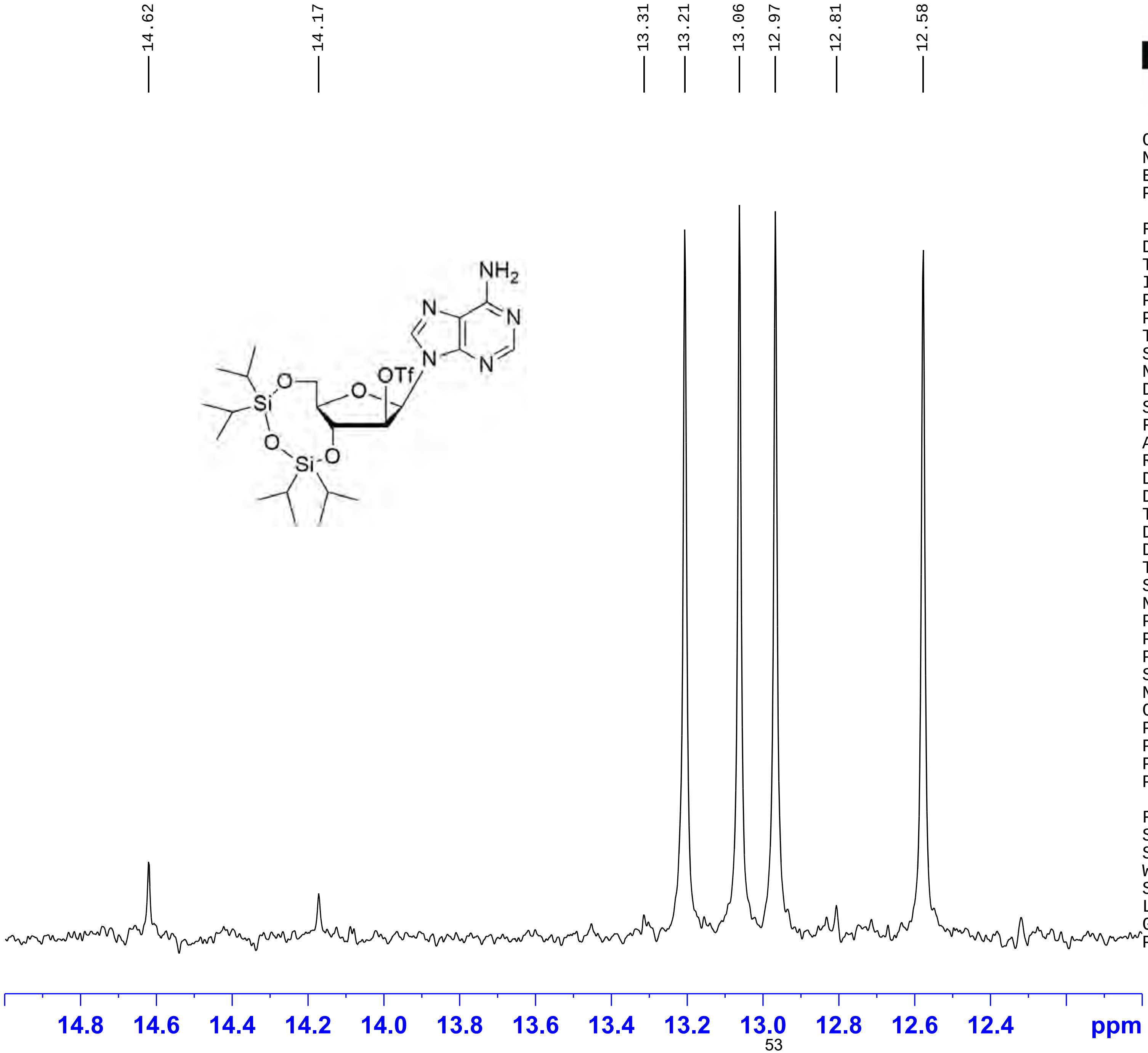

BRUKER

```
Current Data Parameters
NAME              LH-I-50
EXPNO                  11
PROCNO                  1

F2 - Acquisition Parameters
Date_            20220111
Time                20.46 h
INSTRUM             spect
PROBHD   Z114607_0188 (
PULPROG           zgpg30
TD                119044
SOLVENT            CDCl3
NS                  1000
DS                     4
SWH            37500.000 Hz
FIDRES          0.630019 Hz
AQ             1.5872533 sec
RG                186.92
DW                13.333 usec
DE                  6.53 usec
TE                 300.0 K
D1            1.00000000 sec
D11           0.03000000 sec
TD0                    1
SFO1         150.9194058 MHz
NUC1                 13C
P0                  3.93 usec
P1                 11.80 usec
PLW1         85.00000000 W
SFO2         600.1324005 MHz
NUC2                  1H
CPDPRG[2         waltz64
PCPD2              70.00 usec
PLW2         27.00000000 W
PLW12         0.57327998 W
PLW13         0.28836000 W

F2 - Processing parameters
SI                131072
SF           150.9028118 MHz
WDW                   EM
SSB      0
LB                  1.00 Hz
GB       0
PC                  1.40
```

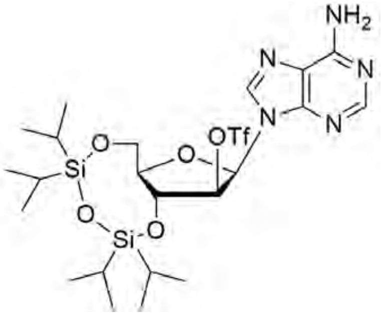

^{13}C DEPT-135 NMR spectrum of compound 10

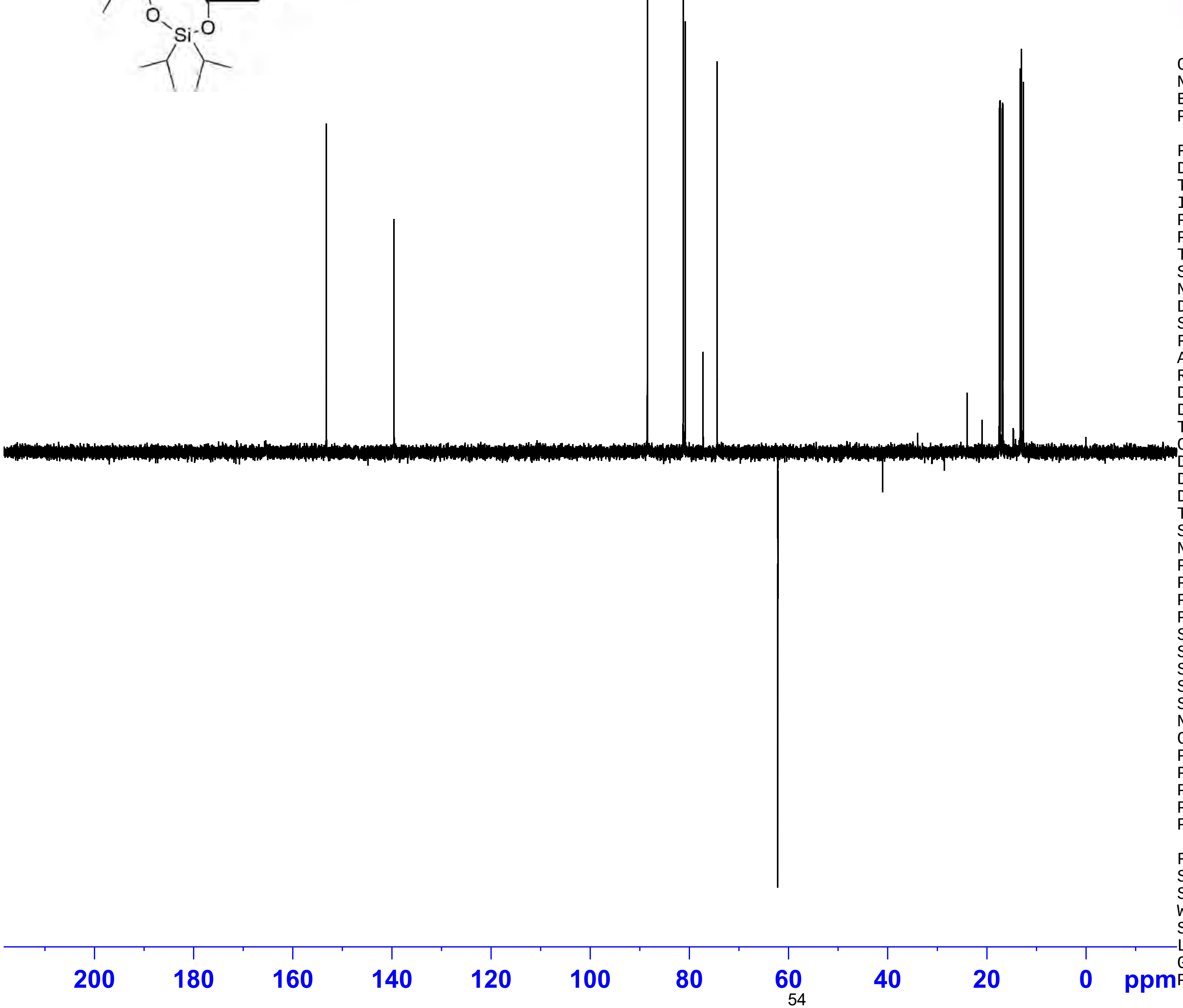

Current Data Parameters
NAME LH-I-50
EXPNO 12
PROCNO 1

F2 - Acquisition Parameters
Date_ 20220111
Time 21.09 h
INSTRUM spect
PROBHD Z114607_0188 (
PULPROG deptsp135.b
TD 119044
SOLVENT CDCl3
NS 500
DS 4
SWH 35714.285 Hz
FIDRES 0.600018 Hz
AQ 1.6666160 sec
RG 186.92
DW 14.000 usec
DE 7.44 usec
TE 300.0 K
CNST2 145.0000000
D1 1.00000000 sec
D2 0.00344828 sec
D12 0.00002000 sec
TD0 1
SFO1 150.9178962 MHz
NUC1 13C
P1 11.80 usec
P13 2000.00 usec
PLW0 0 W
PLW1 85.00000000 W
SPNAM[5] Crp60comp.4
SPOAL5 0.500
SPOFFS5 0 Hz
SPW5 18.08300018 W
SFO2 600.1324005 MHz
NUC2 1H
CPDPRG[2 waltz64
P3 10.20 usec
P4 20.40 usec
PCPD2 70.00 usec
PLW2 27.00000000 W
PLW12 0.57327998 W

F2 - Processing parameters
SI 131072
SF 150.9028085 MHz
WDW EM
SSB 0
LB 1.00 Hz
GB 0
PC 1.40

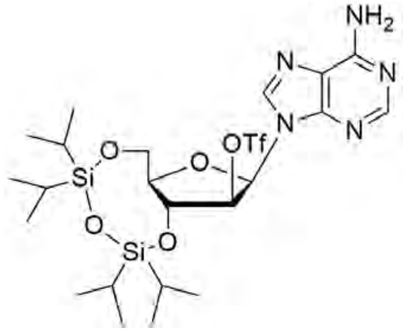

^1H-^1H COSY NMR spectrum of compound 10

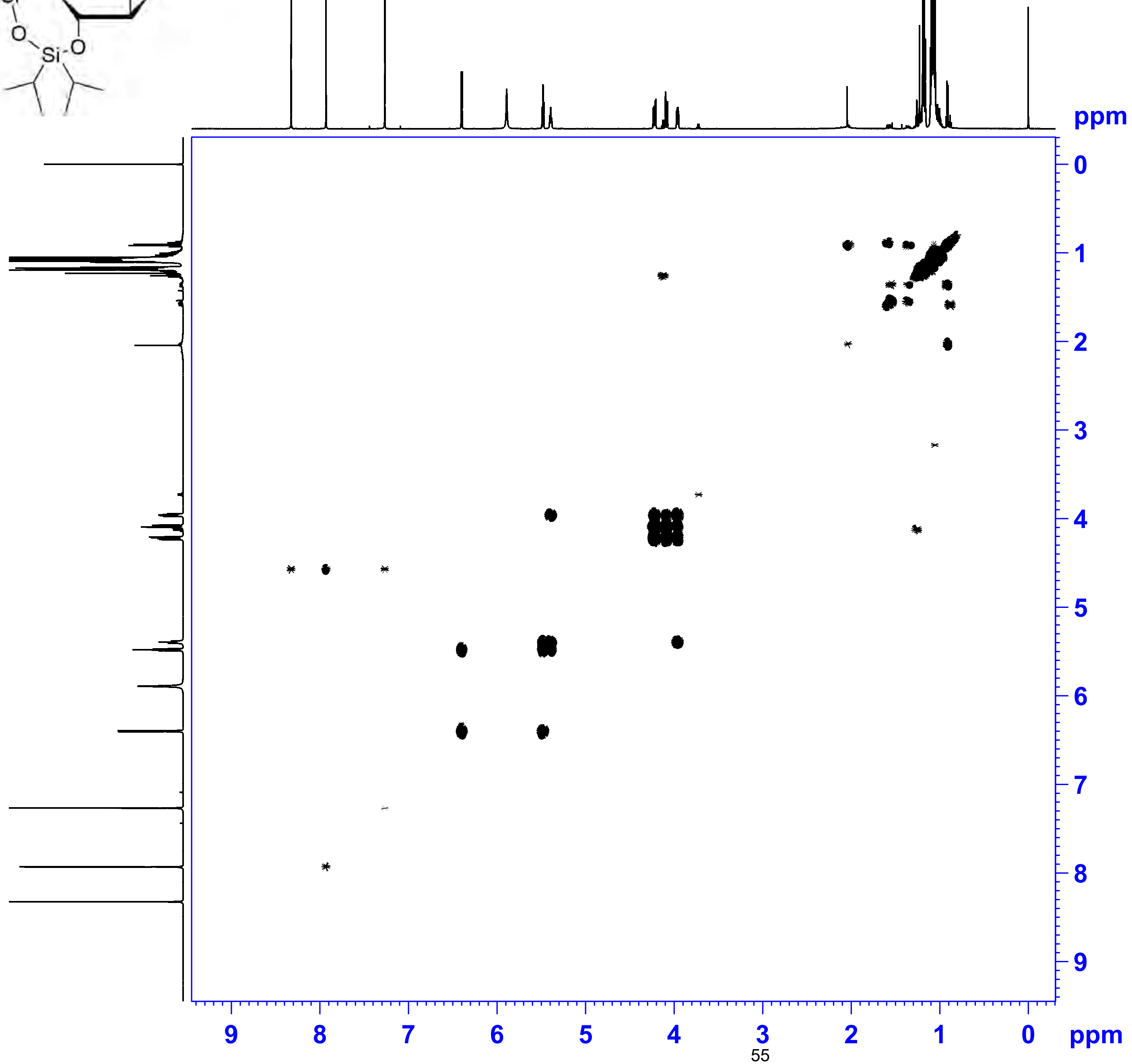

```
Current Data Parameters
NAME              LH-I-50
EXPNO                  13
PROCNO                  1

F2 - Acquisition Parameters
Date_            20220111
Time                21.20 h
INSTRUM             spect
PROBHD   Z114607_0188 (
PULPROG     cosygpmfppqf
TD                   2048
SOLVENT             CDCl3
NS                      2
DS                      8
SWH              5854.801 Hz
FIDRES           5.717579 Hz
AQ              0.1748992 sec
RG                 186.92
DW                 85.400 usec
DE                   6.50 usec
TE                  300.0 K
D0             0.00000300 sec
D1             0.88531131 sec
D11            0.03000000 sec
D12            0.00002000 sec
D13            0.00000400 sec
D16            0.00020000 sec
IN0            0.00017080 sec
TDav                    1
SFO1          600.1327534 MHz
NUC1                   1H
P1                  10.00 usec
P17               2500.00 usec
PLW1          26.60000038 W
PLW10          4.25600004 W
GPNAM[1]       SMSQ10.100
GPZ1                16.00 %
GPNAM[2]       SMSQ10.100
GPZ2                12.00 %
GPNAM[3]       SMSQ10.100
GPZ3                40.00 %
P16               1000.00 usec

F1 - Acquisition parameters
TD                    256
SFO1             600.1328 MHz
FIDRES          45.740631 Hz
SW                  9.756 ppm
FnMODE                 QF

F2 - Processing parameters
SI                   1024
SF            600.1300100 MHz
WDW                  SINE
SSB      0
LB       0 Hz
GB       0
PC                   1.40

F1 - Processing parameters
SI                   1024
MC2                    QF
SF            600.1300101 MHz
WDW                  SINE
SSB      0
LB       0 Hz
GB       0
```

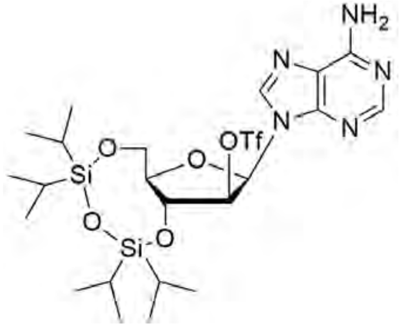

^{1}H-^{13}C HSQC NMR spectrum of compound 10

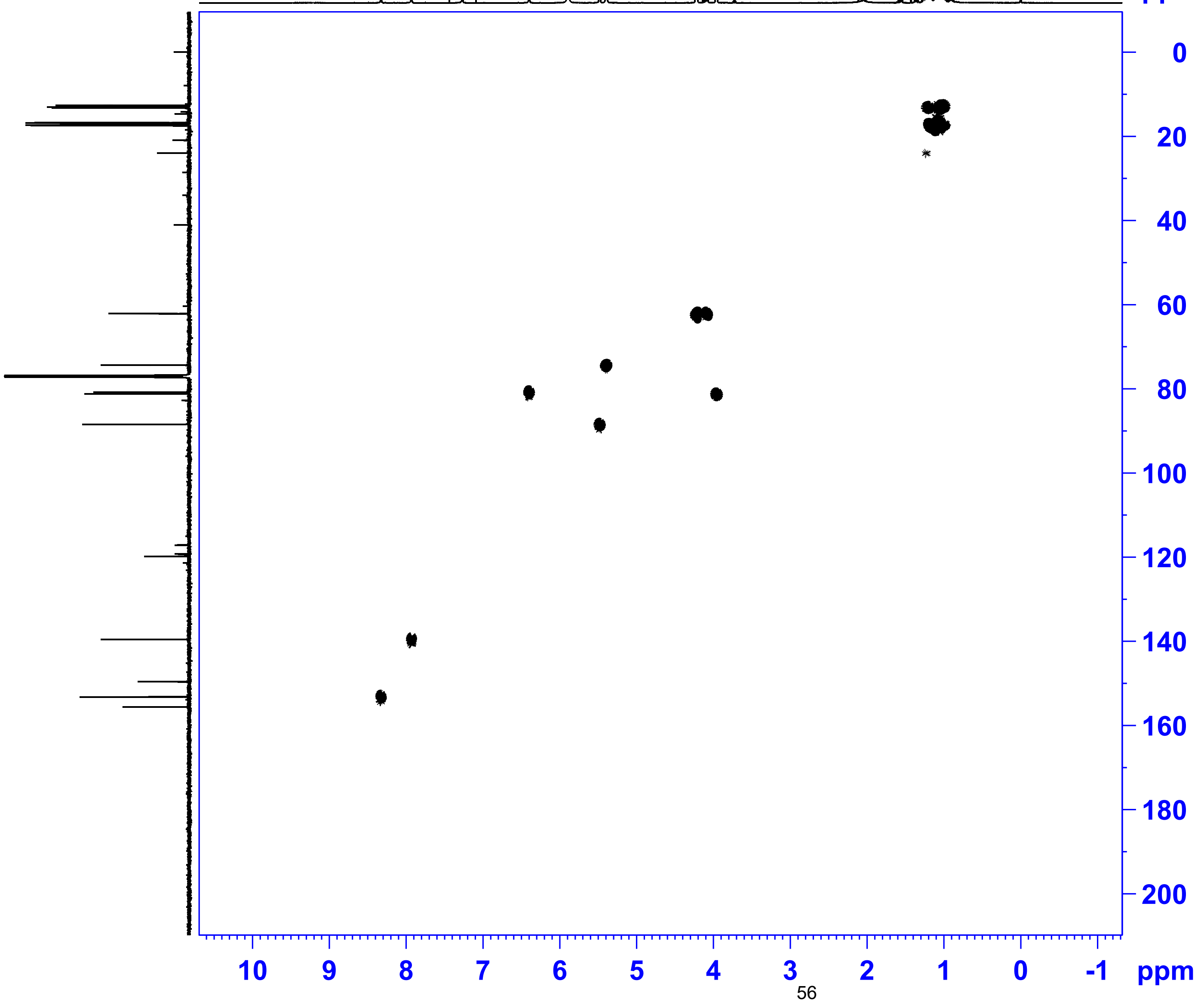


```
Current Data Parameters
NAME               LH-I-50
EXPNO                   14
PROCNO                   1

F2 - Acquisition Parameters
Date_             20220111
Time                 21.29 h
INSTRUM              spect
PROBHD   Z114607_0188 (
PULPROG  hsqcedetgpsp.3
TD                    1024
SOLVENT              CDCl3
NS                       2
DS                      32
SWH               7211.539 Hz
FIDRES           14.085036 Hz
AQ               0.0709973 sec
RG                  186.92
DW                  69.333 usec
DE                    6.50 usec
TE                   300.3 K
CNST2          145.0000000
D0              0.00000300 sec
D1              0.80000001 sec
D4              0.00172414 sec
D11             0.03000000 sec
D16             0.00020000 sec
D21             0.00360000 sec
IN0             0.00001510 sec
TDav                     1
ZGOPTNS
SFO1          600.1328223 MHz
NUC1                    1H
P1                   10.00 usec
P2                   20.00 usec
PLW1           26.60000038 W
SFO2          150.9178988 MHz
NUC2                   13C
CPDPRG[2             garp4
P3                   11.80 usec
P14                 500.00 usec
P31                1730.00 usec
PCPD2                60.00 usec
PLW0     0 W
PLW2           85.00000000 W
PLW12           3.28760004 W
SPNAM[3] Crp60,0.5,20.1
SPOAL3               0.500
SPOFFS3  0 Hz
SPW3           18.08300018 W
SPNAM[18  Crp60_xfilt.2
SPOAL18              0.500
SPOFFS18 0 Hz
SPW18           5.22629976 W
GPNAM[1]       SMSQ10.100
GPZ1                 80.00 %
GPNAM[2]       SMSQ10.100
GPZ2                 20.10 %
P16                1000.00 usec

F1 - Acquisition parameters
TD                     256
SFO1              150.9179 MHz
FIDRES          258.692047 Hz
SW                 219.408 ppm
FnMODE        Echo-Antiecho

F2 - Processing parameters
SI                    1024
SF            600.1300096 MHz
WDW                  QSINE
SSB                      2
LB       0 Hz
GB       0
PC                    1.40

F1 - Processing parameters
SI                    1024
MC2          echo-antiecho
SF            150.9027916 MHz
WDW                  QSINE
SSB                      2
LB       0 Hz
GB       0
```

^{19}F NMR spectrum of compound 10

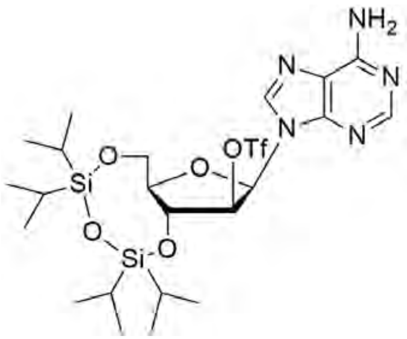

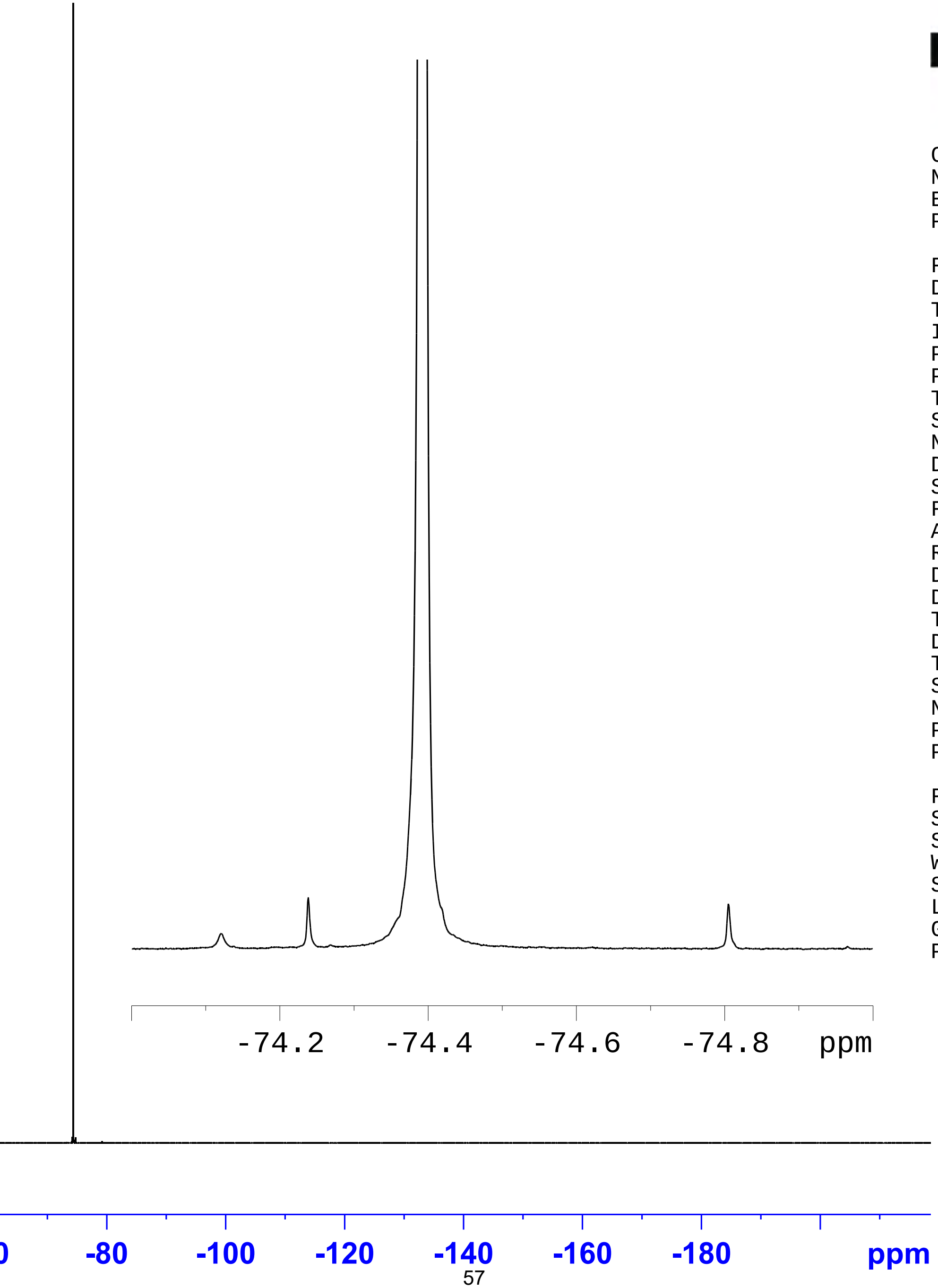


```
Current Data Parameters
NAME              LH-I-50
EXPNO                  15
PROCNO                  1

F2 - Acquisition Parameters
Date_            20220111
Time                21.32 h
INSTRUM             spect
PROBHD    Z114607_0188 (
PULPROG           zgflqn
TD                261896
SOLVENT            CDCl3
NS                    16
DS                     4
SWH           133928.578 Hz
FIDRES          1.022761 Hz
AQ             0.9777451 sec
RG                186.92
DW                 3.733 usec
DE                  6.70 usec
TE                 300.0 K
D1            4.00000000 sec
TD0                    1
SFO1         564.6299217 MHz
NUC1                 19F
P1                 12.00 usec
PLW1         49.00000000 W

F2 - Processing parameters
SI                262144
SF           564.6863882 MHz
WDW                   EM
SSB      0
LB                  0.50 Hz
GB       0
PC                  2.00
```

^{1}H NMR spectrum of compound 9

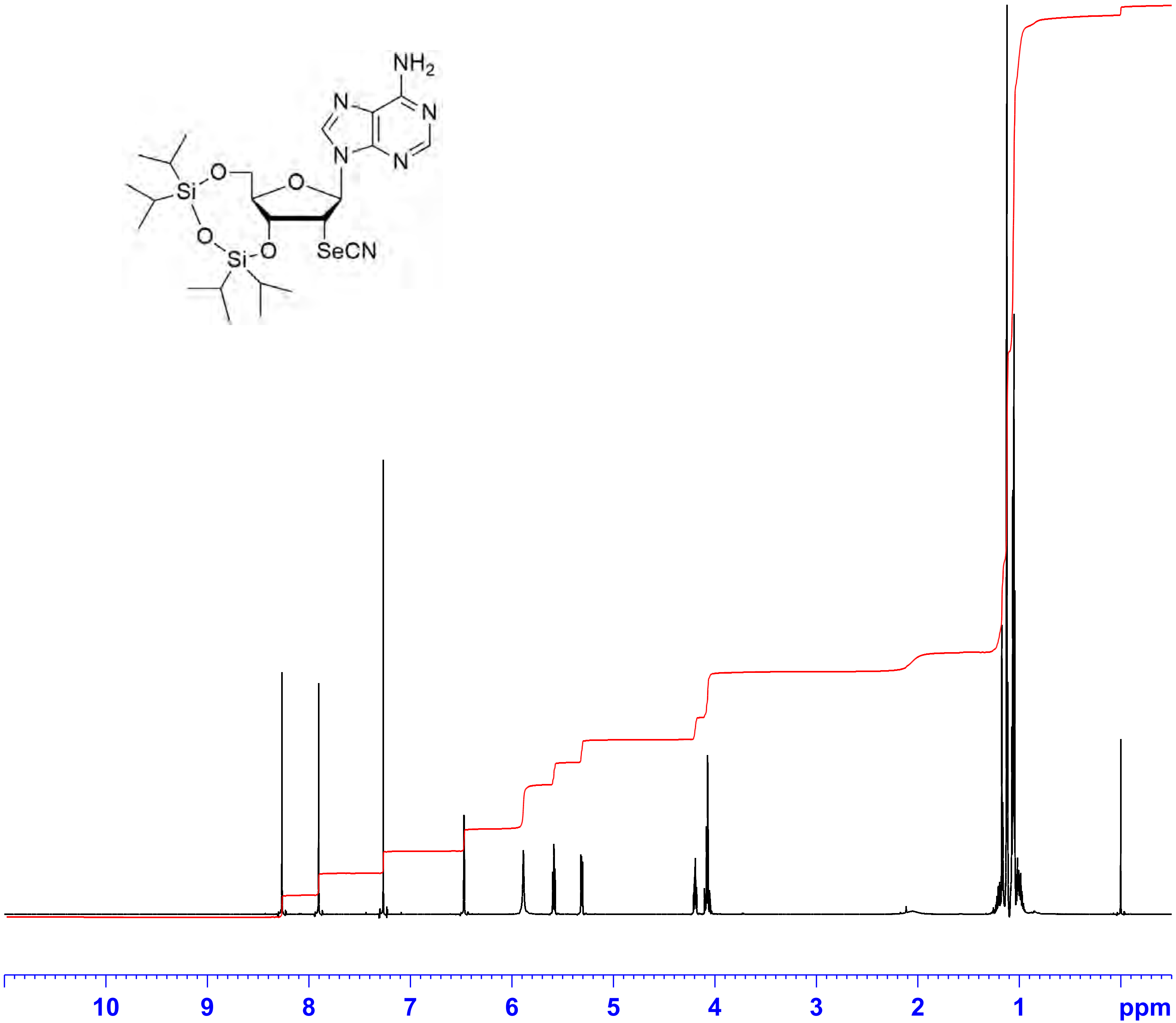


```
Current Data Parameters
NAME              LH-I-55
EXPNO                  10
PROCNO                  1

F2 - Acquisition Parameters
Date_            20220309
Time                14.25 h
INSTRUM             spect
PROBHD    Z114607_0188 (
PULPROG              zg30
TD                 180286
SOLVENT             CDCl3
NS                     16
DS                      0
SWH             18028.846 Hz
FIDRES           0.200003 Hz
AQ              4.9999318 sec
RG                  55.43
DW                 27.733 usec
DE                   8.00 usec
TE                  300.0 K
D1             0.10000000 sec
TD0                     1
SFO1          600.1337060 MHz
NUC1                   1H
P0                   3.33 usec
P1                  10.00 usec
PLW1          26.60000038 W

F2 - Processing parameters
SI                 262144
SF            600.1300113 MHz
WDW                    EM
SSB      0
LB                   0.10 Hz
GB       0
PC                   1.00
```

Expanded region of the ^{1}H NMR spectrum of compound 9

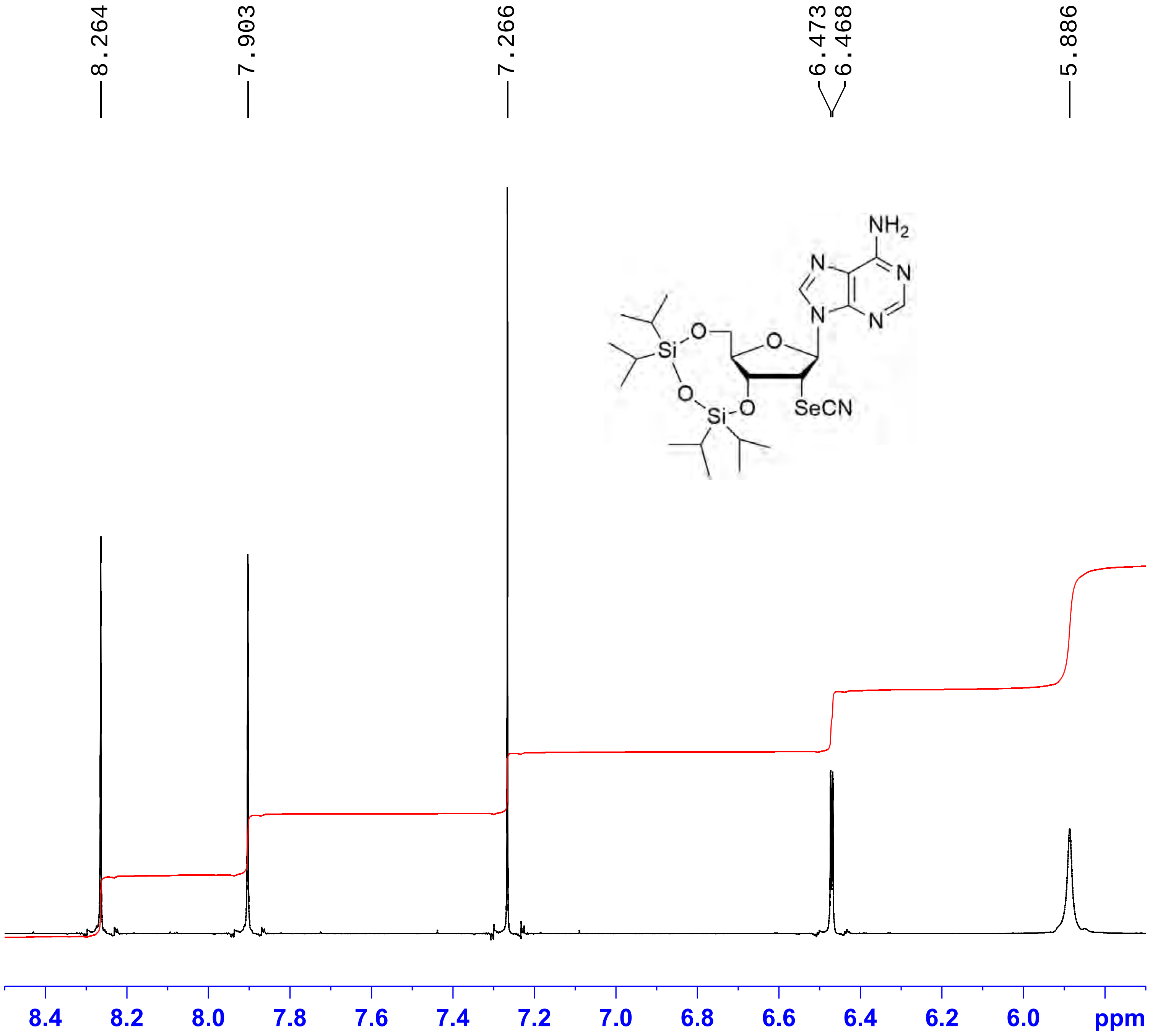


```
Current Data Parameters
NAME             LH-I-55
EXPNO                 10
PROCNO                 1

F2 - Acquisition Parameters
Date_           20220309
Time               14.25 h
INSTRUM            spect
PROBHD    Z114607_0188 (
PULPROG             zg30
TD                180286
SOLVENT            CDCl3
NS                    16
DS                     0
SWH            18028.846 Hz
FIDRES          0.200003 Hz
AQ             4.9999318 sec
RG                 55.43
DW                27.733 usec
DE                  8.00 usec
TE                 300.0 K
D1            0.10000000 sec
TD0                    1
SFO1         600.1337060 MHz
NUC1                  1H
P0                  3.33 usec
P1                 10.00 usec
PLW1         26.60000038 W

F2 - Processing parameters
SI                262144
SF           600.1300113 MHz
WDW                   EM
SSB      0
LB                  0.10 Hz
GB       0
PC                  1.00
```

Expanded region of the ^{1}H NMR spectrum of compound 9

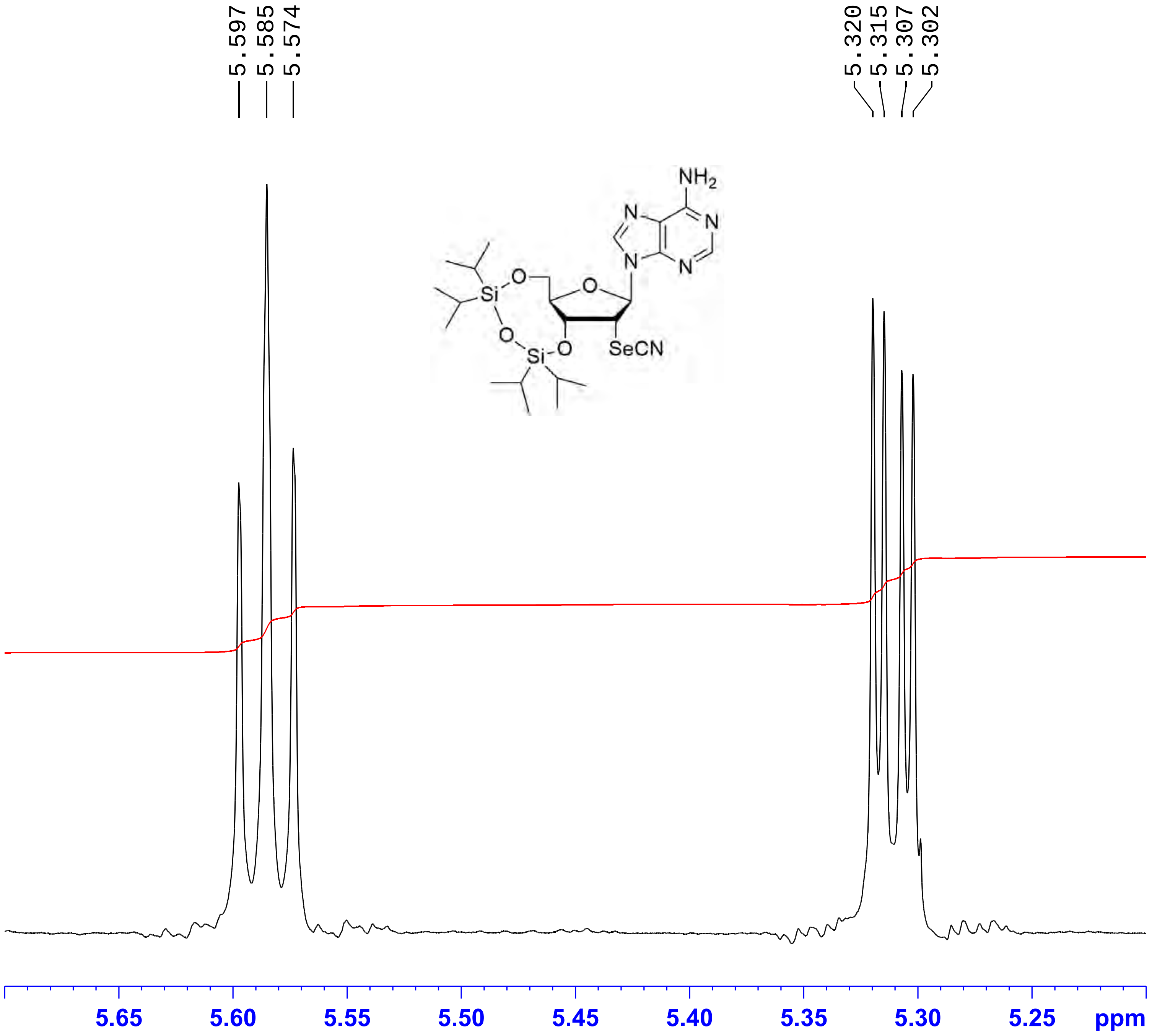


```
Current Data Parameters
NAME              LH-I-55
EXPNO                  10
PROCNO                  1

F2 - Acquisition Parameters
Date_            20220309
Time                14.25 h
INSTRUM             spect
PROBHD      Z114607_0188 (
PULPROG              zg30
TD                 180286
SOLVENT             CDCl3
NS                     16
DS                      0
SWH             18028.846 Hz
FIDRES           0.200003 Hz
AQ              4.9999318 sec
RG                  55.43
DW                 27.733 usec
DE                   8.00 usec
TE                  300.0 K
D1             0.10000000 sec
TD0                     1
SFO1          600.1337060 MHz
NUC1                   1H
P0                   3.33 usec
P1                  10.00 usec
PLW1          26.60000038 W

F2 - Processing parameters
SI                 262144
SF            600.1300113 MHz
WDW                    EM
SSB      0
LB                   0.10 Hz
GB       0
PC                   1.00
```

Expanded region of the ^{1}H NMR spectrum of compound 9

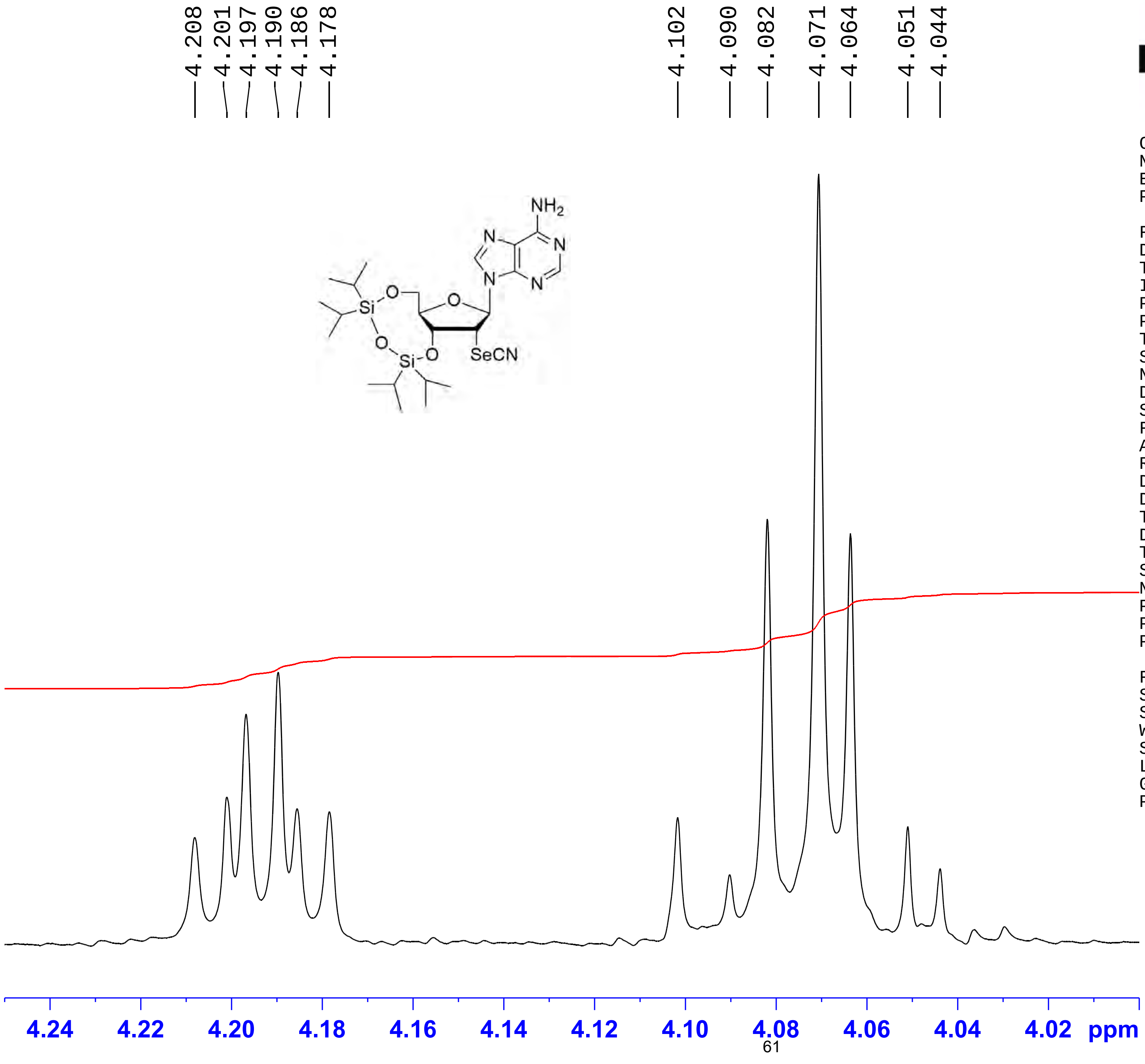


```
Current Data Parameters
NAME            LH-I-55
EXPNO                10
PROCNO                1

F2 - Acquisition Parameters
Date_          20220309
Time              14.25 h
INSTRUM           spect
PROBHD   Z114607_0188 (
PULPROG            zg30
TD               180286
SOLVENT           CDCl3
NS                   16
DS                    0
SWH           18028.846 Hz
FIDRES         0.200003 Hz
AQ            4.9999318 sec
RG                55.43
DW               27.733 usec
DE                 8.00 usec
TE                300.0 K
D1           0.10000000 sec
TD0                   1
SFO1        600.1337060 MHz
NUC1                 1H
P0                 3.33 usec
P1                10.00 usec
PLW1        26.60000038 W

F2 - Processing parameters
SI               262144
SF          600.1300113 MHz
WDW                  EM
SSB      0
LB                 0.10 Hz
GB       0
PC                 1.00
```

^{13}C NMR spectrum of compound 9

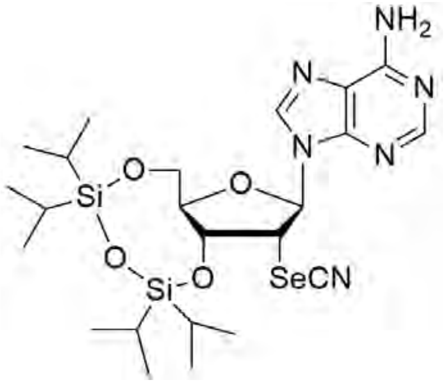

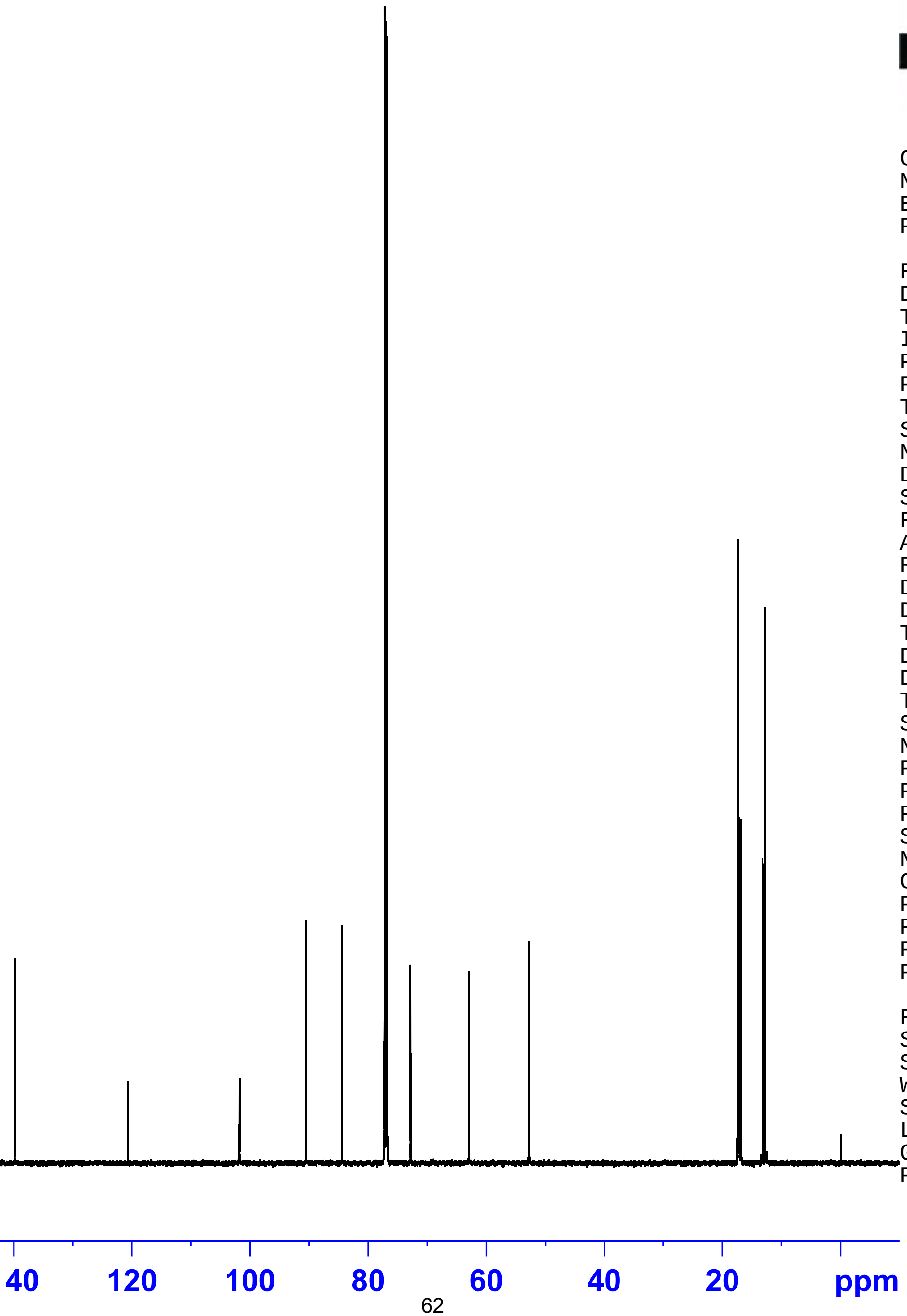

BRUKER

Current Data Parameters
NAME LH-I-55
EXPNO 11
PROCNO 1

F2 - Acquisition Parameters
Date_ 20220309
Time 15.55 h
INSTRUM spect
PROBHD Z114607_0188 (
PULPROG zgpg30
TD 119044
SOLVENT CDCl3
NS 2000
DS 4
SWH 37500.000 Hz
FIDRES 0.630019 Hz
AQ 1.5872533 sec
RG 186.92
DW 13.333 usec
DE 6.53 usec
TE 300.0 K
D1 1.00000000 sec
D11 0.03000000 sec
TD0 1
SFO1 150.9194058 MHz
NUC1 13C
P0 3.93 usec
P1 11.80 usec
PLW1 85.00000000 W
SFO2 600.1324005 MHz
NUC2 1H
CPDPRG[2 waltz64
PCPD2 70.00 usec
PLW2 27.00000000 W
PLW12 0.57327998 W
PLW13 0.28836000 W

F2 - Processing parameters
SI 131072
SF 150.9028135 MHz
WDW EM
SSB 0
LB 1.00 Hz
GB 0
PC 1.40

Expanded region of the ^{13}C NMR spectrum of compound 9

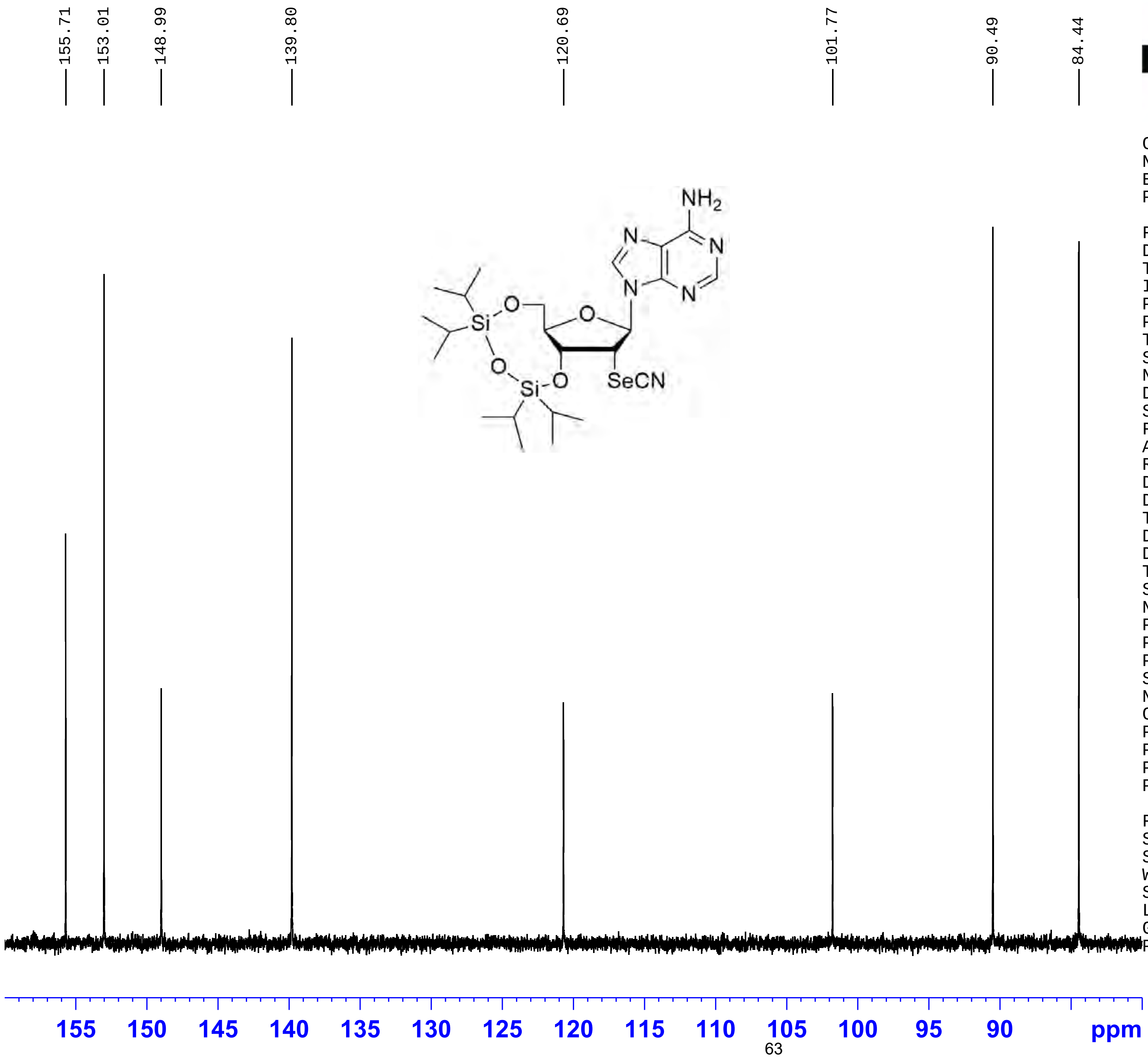


```
Current Data Parameters
NAME              LH-I-55
EXPNO                  11
PROCNO                  1

F2 - Acquisition Parameters
Date_            20220309
Time                15.55 h
INSTRUM             spect
PROBHD   Z114607_0188 (
PULPROG           zgpg30
TD                119044
SOLVENT            CDCl3
NS                  2000
DS                     4
SWH            37500.000 Hz
FIDRES          0.630019 Hz
AQ             1.5872533 sec
RG                186.92
DW                13.333 usec
DE                  6.53 usec
TE                 300.0 K
D1            1.00000000 sec
D11           0.03000000 sec
TD0                    1
SFO1         150.9194058 MHz
NUC1                 13C
P0                  3.93 usec
P1                 11.80 usec
PLW1         85.00000000 W
SFO2         600.1324005 MHz
NUC2                  1H
CPDPRG[2         waltz64
PCPD2              70.00 usec
PLW2         27.00000000 W
PLW12         0.57327998 W
PLW13         0.28836000 W

F2 - Processing parameters
SI                131072
SF           150.9028135 MHz
WDW                   EM
SSB      0
LB                  1.00 Hz
GB       0
PC                  1.40
```

Expanded region of the ^{13}C NMR spectrum of compound 9

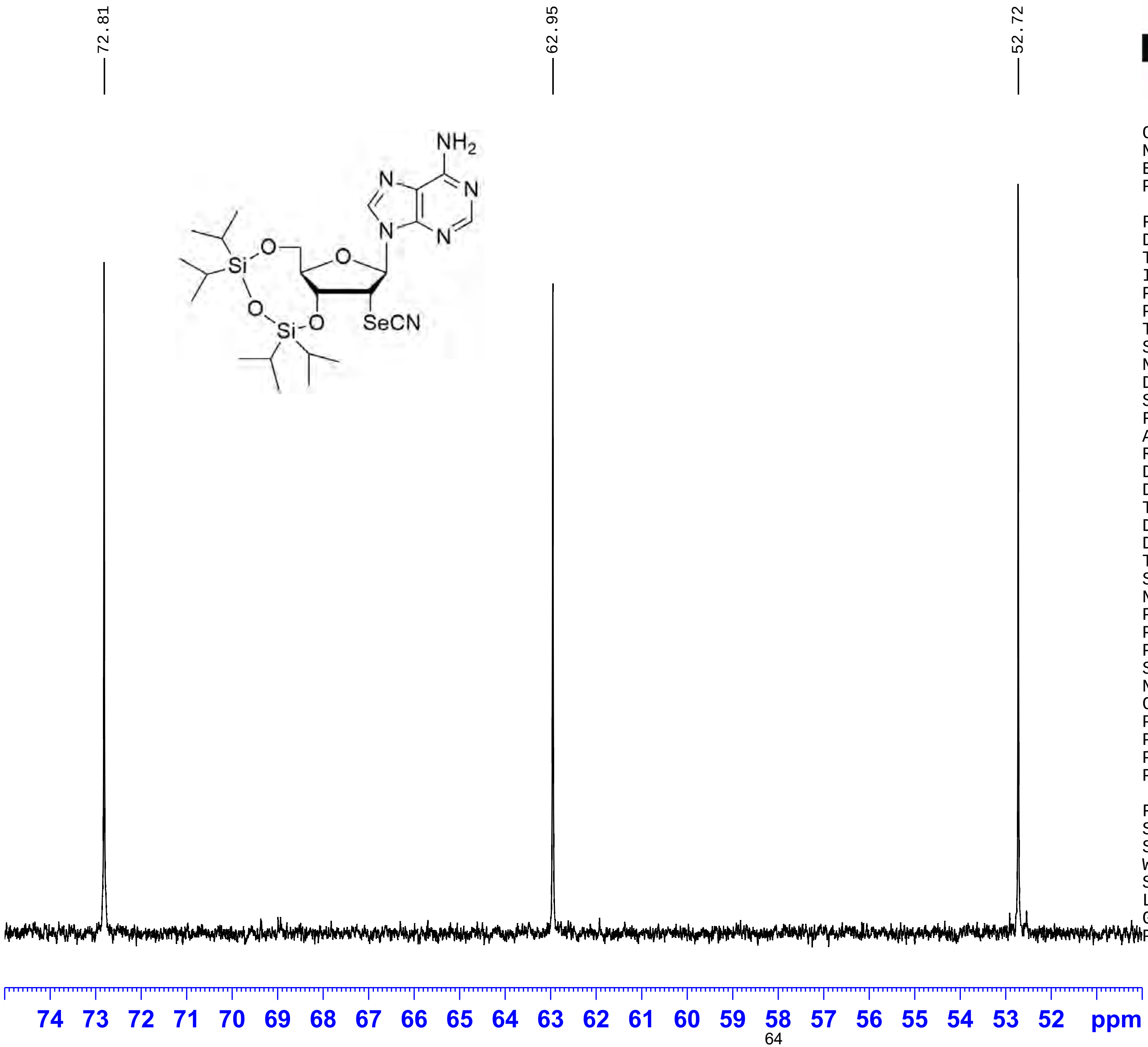


```
Current Data Parameters
NAME              LH-I-55
EXPNO                  11
PROCNO                  1

F2 - Acquisition Parameters
Date_            20220309
Time                15.55 h
INSTRUM             spect
PROBHD    Z114607_0188 (
PULPROG           zgpg30
TD                 119044
SOLVENT             CDCl3
NS                   2000
DS                      4
SWH             37500.000 Hz
FIDRES           0.630019 Hz
AQ              1.5872533 sec
RG                 186.92
DW                 13.333 usec
DE                   6.53 usec
TE                  300.0 K
D1             1.00000000 sec
D11            0.03000000 sec
TD0                     1
SFO1          150.9194058 MHz
NUC1                  13C
P0                   3.93 usec
P1                  11.80 usec
PLW1          85.00000000 W
SFO2          600.1324005 MHz
NUC2                   1H
CPDPRG[2          waltz64
PCPD2               70.00 usec
PLW2          27.00000000 W
PLW12          0.57327998 W
PLW13          0.28836000 W

F2 - Processing parameters
SI                 131072
SF            150.9028135 MHz
WDW                    EM
SSB      0
LB                   1.00 Hz
GB       0
PC                   1.40
```

Expanded region of the ^{13}C NMR spectrum of compound 9

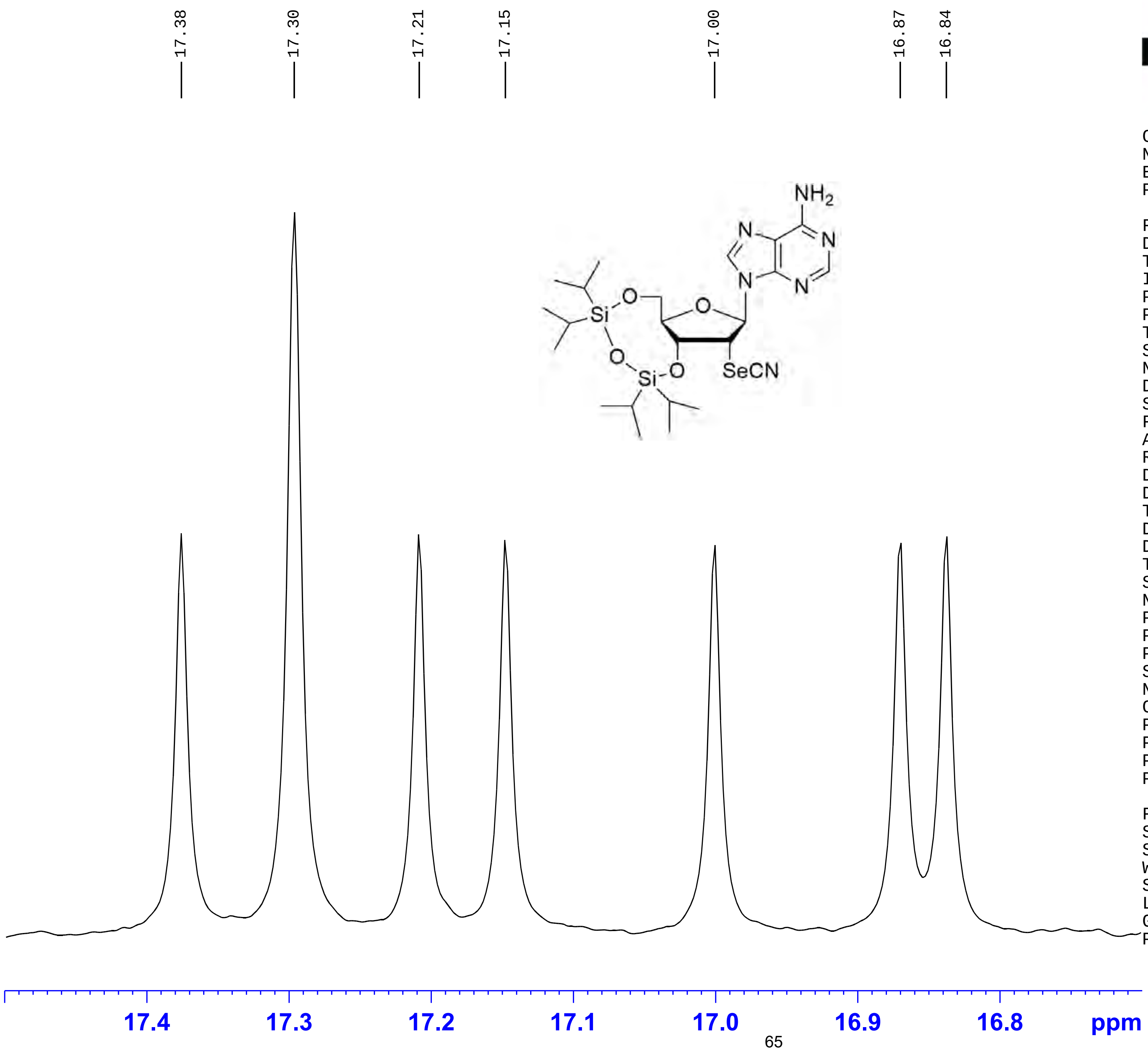

Current Data Parameters		
NAME	LH-I-55	
EXPNO	11	
PROCNO	1	
F2 - Acquisition Parameters		
Date_	20220309	
Time	15.55	h
INSTRUM	spect	
PROBHD	Z114607_0188 (	
PULPROG	zgpg30	
TD	119044	
SOLVENT	CDCl3	
NS	2000	
DS	4	
SWH	37500.000	Hz
FIDRES	0.630019	Hz
AQ	1.5872533	sec
RG	186.92	
DW	13.333	usec
DE	6.53	usec
TE	300.0	K
D1	1.00000000	sec
D11	0.03000000	sec
TD0	1	
SFO1	150.9194058	MHz
NUC1	13C	
P0	3.93	usec
P1	11.80	usec
PLW1	85.00000000	W
SFO2	600.1324005	MHz
NUC2	1H	
CPDPRG[2	waltz64	
PCPD2	70.00	usec
PLW2	27.00000000	W
PLW12	0.57327998	W
PLW13	0.28836000	W
F2 - Processing parameters		
SI	131072	
SF	150.9028135	MHz
WDW	EM	
SSB	0	
LB	1.00	Hz
GB	0	
PC	1.40	

Expanded region of the ^{13}C NMR spectrum of compound 9

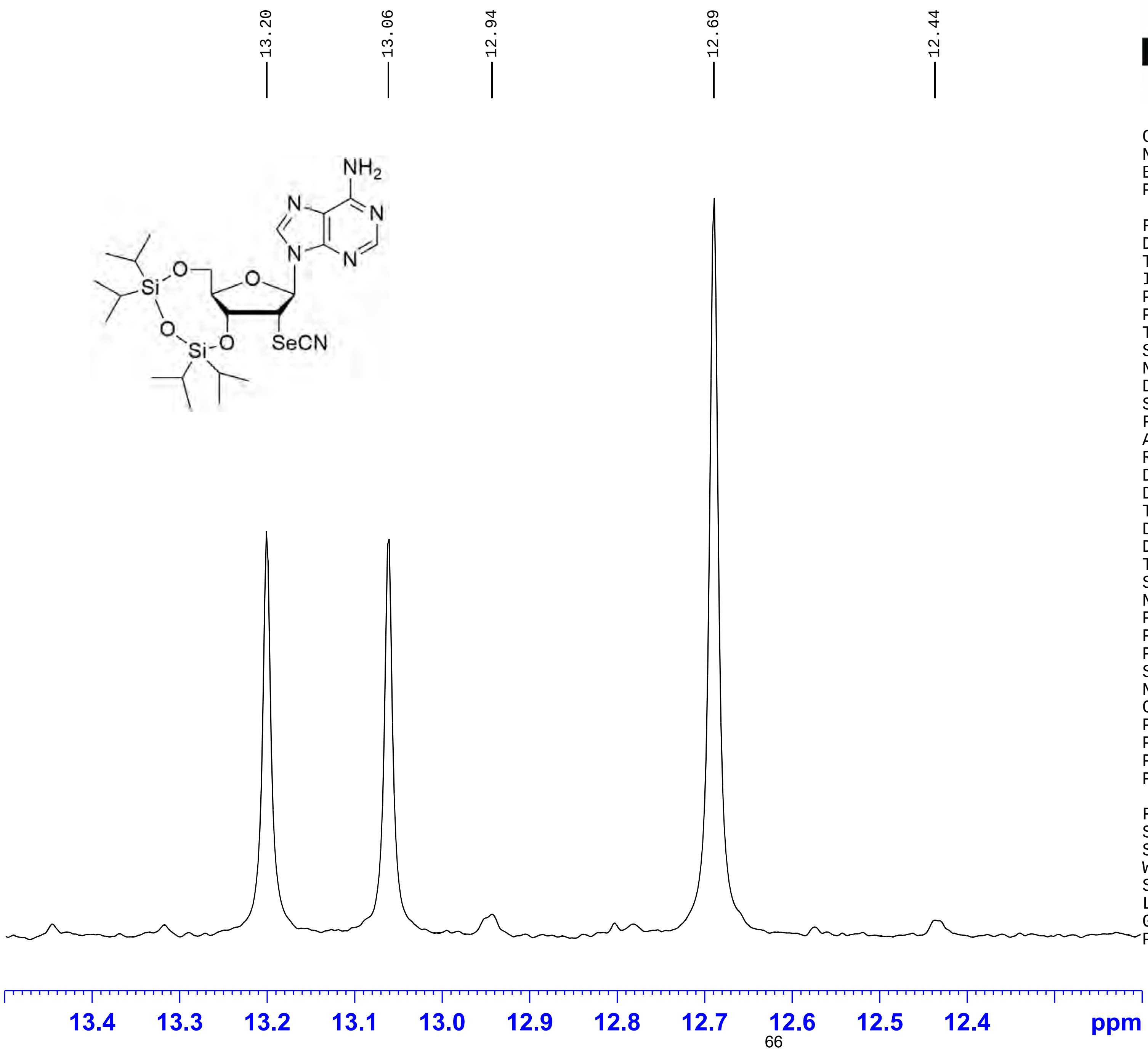


```
Current Data Parameters
NAME              LH-I-55
EXPNO                  11
PROCNO                  1

F2 - Acquisition Parameters
Date_            20220309
Time                15.55 h
INSTRUM             spect
PROBHD    Z114607_0188 (
PULPROG           zgpg30
TD                119044
SOLVENT            CDCl3
NS                   2000
DS                      4
SWH             37500.000 Hz
FIDRES           0.630019 Hz
AQ              1.5872533 sec
RG                 186.92
DW                 13.333 usec
DE                   6.53 usec
TE                  300.0 K
D1             1.00000000 sec
D11            0.03000000 sec
TD0                     1
SFO1          150.9194058 MHz
NUC1                  13C
P0                   3.93 usec
P1                  11.80 usec
PLW1          85.00000000 W
SFO2          600.1324005 MHz
NUC2                   1H
CPDPRG[2          waltz64
PCPD2               70.00 usec
PLW2          27.00000000 W
PLW12          0.57327998 W
PLW13          0.28836000 W

F2 - Processing parameters
SI                 131072
SF            150.9028135 MHz
WDW                    EM
SSB       0
LB                   1.00 Hz
GB        0
PC                   1.40
```

^{13}C DEPT-135 NMR spectrum of compound 9

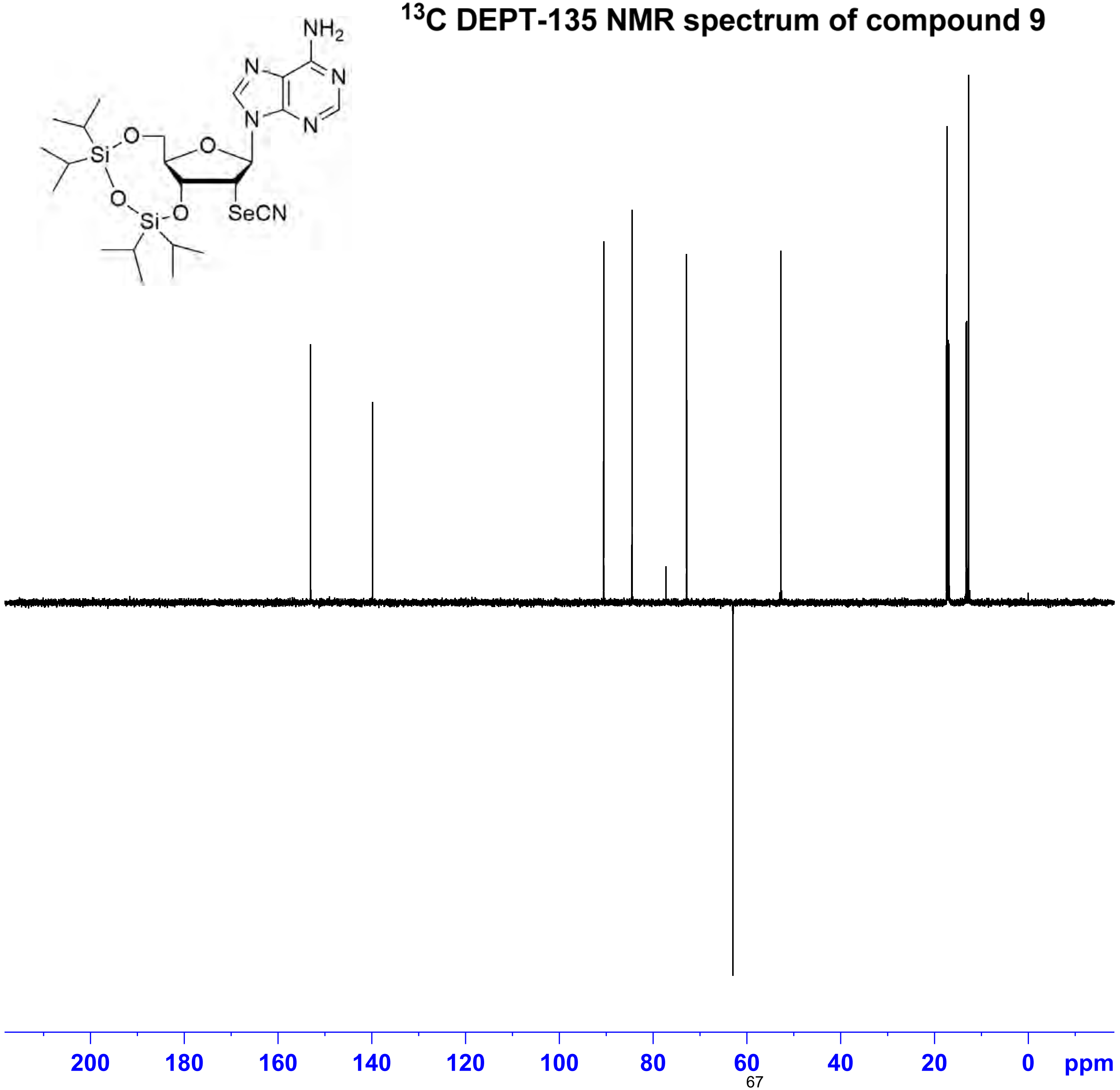


```
Current Data Parameters
NAME                LH-I-55
EXPNO                    12
PROCNO                    1

F2 - Acquisition Parameters
Date_              20220309
Time                  16.40 h
INSTRUM               spect
PROBHD     Z114607_0188 (
PULPROG        deptsp135.b
TD                   119044
SOLVENT               CDCl3
NS                     1000
DS                        4
SWH               35714.285 Hz
FIDRES             0.600018 Hz
AQ                1.6666160 sec
RG                   186.92
DW                   14.000 usec
DE                     7.44 usec
TE                    300.0 K
CNST2           145.0000000
D1               1.00000000 sec
D2               0.00344828 sec
D12              0.00002000 sec
TD0                       1
SFO1            150.9178962 MHz
NUC1                    13C
P1                    11.80 usec
P13                 2000.00 usec
PLW0        0 W
PLW1            85.00000000 W
SPNAM[5]        Crp60comp.4
SPOAL5                0.500
SPOFFS5     0 Hz
SPW5            18.08300018 W
SFO2            600.1324005 MHz
NUC2                     1H
CPDPRG[2            waltz64
P3                    10.20 usec
P4                    20.40 usec
PCPD2                 70.00 usec
PLW2            27.00000000 W
PLW12            0.57327998 W

F2 - Processing parameters
SI                   131072
SF              150.9028085 MHz
WDW                      EM
SSB         0
LB                     1.00 Hz
GB          0
PC                     1.40
```

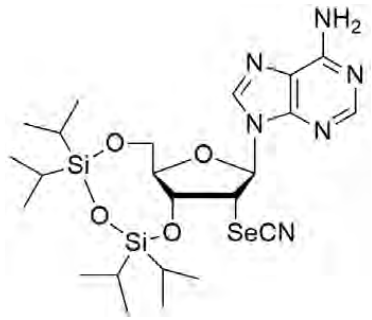

^{1}H-^{1}H COSY NMR spectrum of compound 9

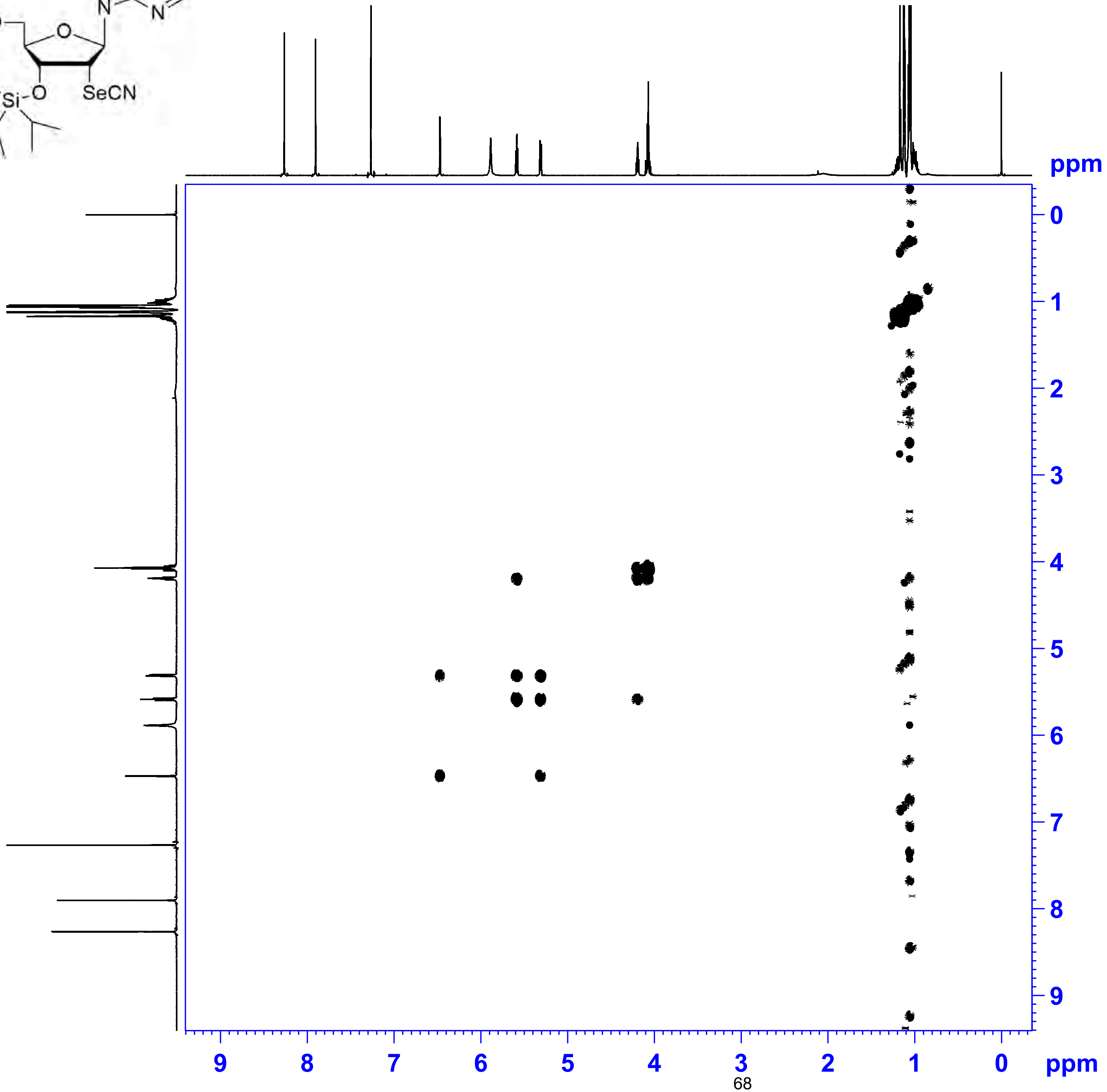

Current Data Parameters
NAME LH-I-55
EXPNO 13
PROCNO 1

F2 - Acquisition Parameters
Date_ 20220309
Time 16.51 h
INSTRUM spect
PROBHD Z114607_0188 (
PULPROG cosygpmfppqf
TD 2048
SOLVENT CDCl3
NS 2
DS 8
SWH 5854.801 Hz
FIDRES 5.717579 Hz
AQ 0.1748992 sec
RG 186.92
DW 85.400 usec
DE 6.50 usec
TE 300.0 K
D0 0.00000300 sec
D1 0.88531131 sec
D11 0.03000000 sec
D12 0.00002000 sec
D13 0.00000400 sec
D16 0.00020000 sec
IN0 0.00017080 sec
TDav 1
SFO1 600.1327260 MHz
NUC1 1H
P1 10.00 usec
P17 2500.00 usec
PLW1 26.60000038 W
PLW10 4.25600004 W
GPNAM[1] SMSQ10.100
GPZ1 16.00 %
GPNAM[2] SMSQ10.100
GPZ2 12.00 %
GPNAM[3] SMSQ10.100
GPZ3 40.00 %
P16 1000.00 usec

F1 - Acquisition parameters
TD 256
SFO1 600.1327 MHz
FIDRES 45.740631 Hz
SW 9.756 ppm
FnMODE QF

F2 - Processing parameters
SI 1024
SF 600.1300095 MHz
WDW SINE
SSB 0
LB 0 Hz
GB 0
PC 1.40

F1 - Processing parameters
SI 1024
MC2 QF
SF 600.1300087 MHz
WDW SINE
SSB 0
LB 0 Hz
GB 0

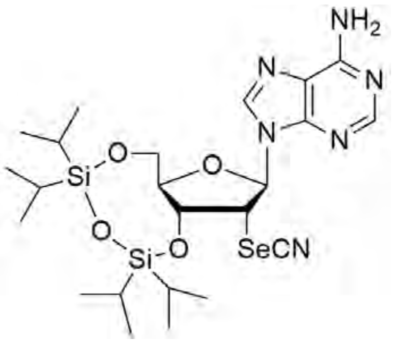

^{1}H-^{13}C HSQC NMR spectrum of compound 9

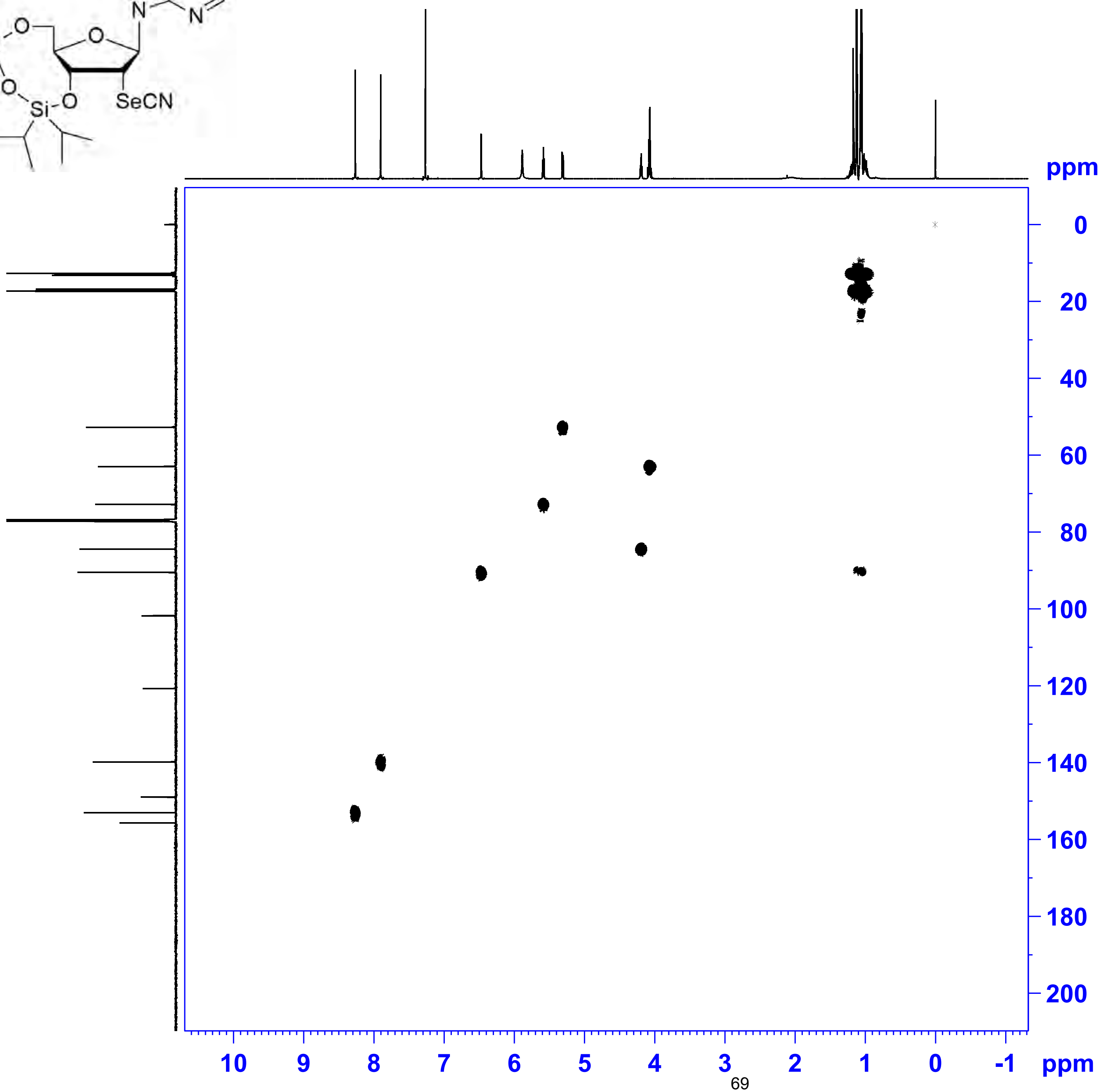


```
Current Data Parameters
NAME              LH-I-55
EXPNO                  14
PROCNO                  1

F2 - Acquisition Parameters
Date_            20220309
Time                17.00 h
INSTRUM             spect
PROBHD   Z114607_0188 (
PULPROG  hsqcedetgpsp.3
TD                   1024
SOLVENT             CDCl3
NS                      2
DS                     32
SWH              7211.539 Hz
FIDRES          14.085036 Hz
AQ              0.0709973 sec
RG                 186.92
DW                 69.333 usec
DE                   6.50 usec
TE                  300.3 K
CNST2         145.0000000
D0             0.00000300 sec
D1             0.80000001 sec
D4             0.00172414 sec
D11            0.03000000 sec
D16            0.00020000 sec
D21            0.00360000 sec
IN0            0.00001510 sec
TDav                    1
ZGOPTNS
SFO1         600.1328223 MHz
NUC1                   1H
P1                  10.00 usec
P2                  20.00 usec
PLW1          26.60000038 W
SFO2         150.9178988 MHz
NUC2                  13C
CPDPRG[2            garp4
P3                  11.80 usec
P14                500.00 usec
P31               1730.00 usec
PCPD2               60.00 usec
PLW0     0 W
PLW2          85.00000000 W
PLW12          3.28760004 W
SPNAM[3] Crp60,0.5,20.1
SPOAL3              0.500
SPOFFS3  0 Hz
SPW3          18.08300018 W
SPNAM[18  Crp60_xfilt.2
SPOAL18             0.500
SPOFFS18 0 Hz
SPW18          5.22629976 W
GPNAM[1]       SMSQ10.100
GPZ1                80.00 %
GPNAM[2]       SMSQ10.100
GPZ2                20.10 %
P16               1000.00 usec

F1 - Acquisition parameters
TD                    256
SFO1             150.9179 MHz
FIDRES         258.692047 Hz
SW                219.408 ppm
FnMODE       Echo-Antiecho

F2 - Processing parameters
SI                   1024
SF          600.1300095 MHz
WDW                 QSINE
SSB                     2
LB       0 Hz
GB       0
PC                   1.40

F1 - Processing parameters
SI                   1024
MC2         echo-antiecho
SF          150.9027945 MHz
WDW                 QSINE
SSB                     2
LB       0 Hz
GB       0
```

^{77}Se NMR spectrum of compound 9

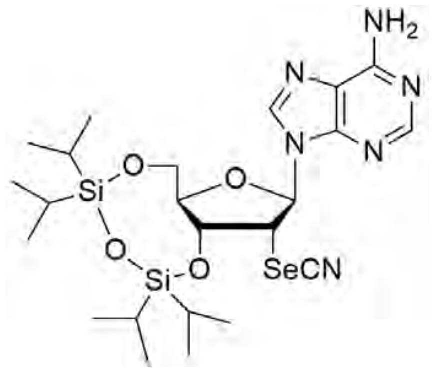

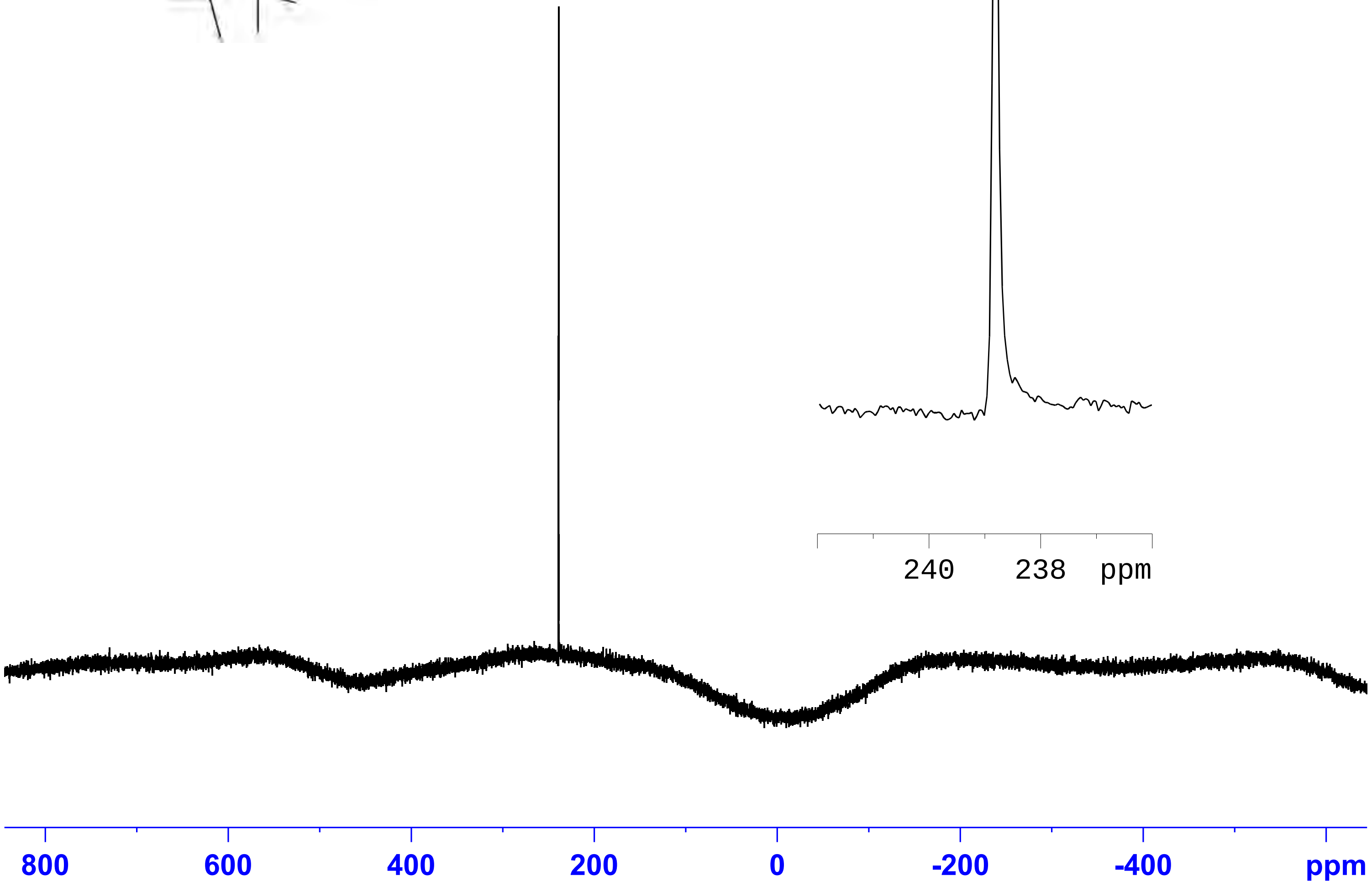

BRUKER

```
Current Data Parameters
NAME              LH-I-55
EXPNO                  15
PROCNO                  1

F2 - Acquisition Parameters
Date_            20220127
Time                 3.36 h
INSTRUM             spect
PROBHD   Z114607_0188 (
PULPROG                zg
TD                  65536
SOLVENT             CDCl3
NS                  25000
DS                      4
SWH            170454.547 Hz
FIDRES           5.201860 Hz
AQ              0.1922389 sec
RG                 186.92
DW                  2.933 usec
DE                   6.50 usec
TE                  300.0 K
D1             1.00000000 sec
TD0                     1
SFO1          114.4653164 MHz
NUC1                 77Se
P1                  10.00 usec
PLW1          60.00000000 W

F2 - Processing parameters
SI                  32768
SF            114.4538710 MHz
WDW                    EM
SSB      0
LB                   5.00 Hz
GB       0
PC                   1.40
```

^{1}H NMR spectrum of compound 15

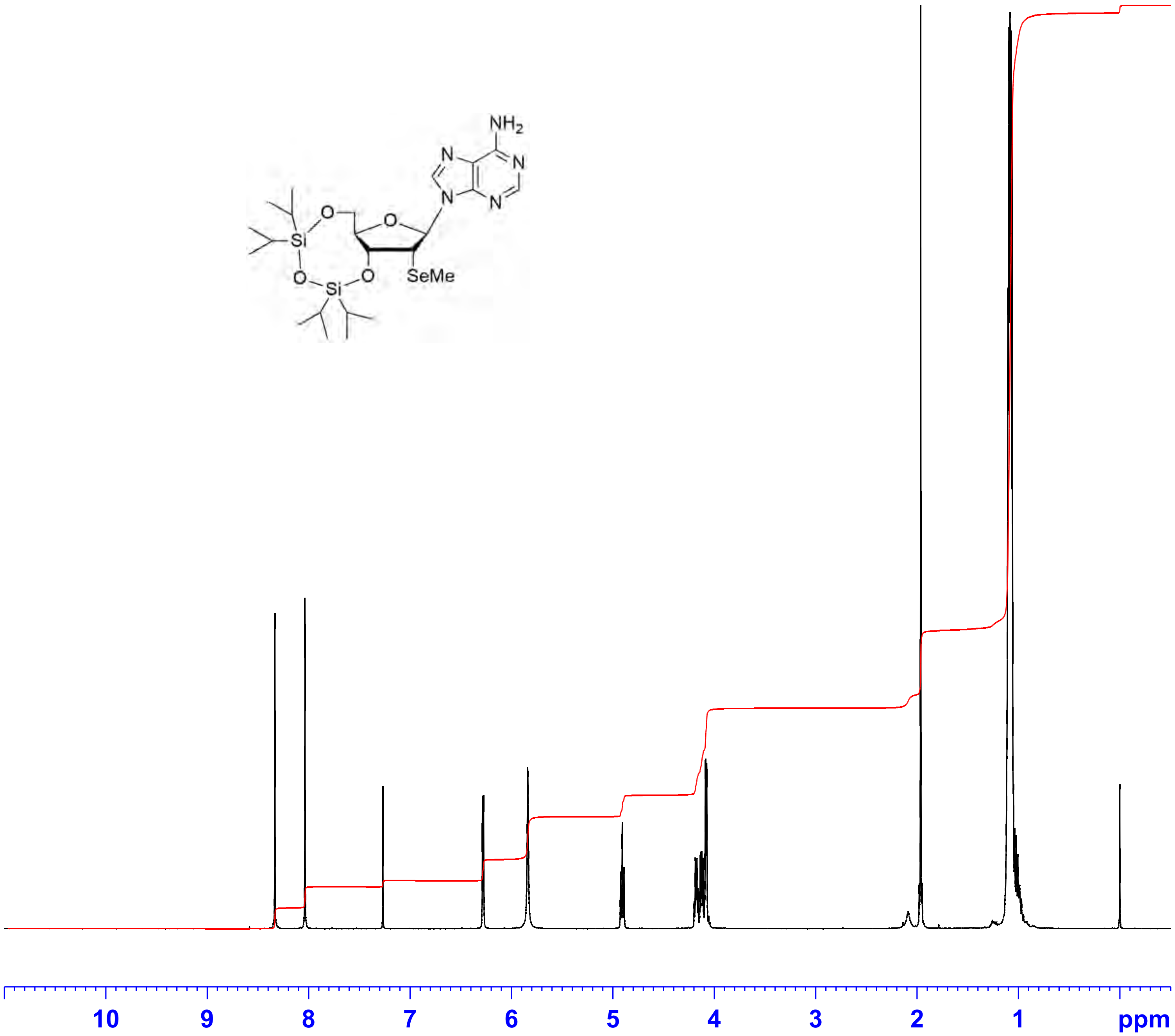

Current Data Parameters
NAME LH-II-69 OLD NMR
EXPNO 10
PROCNO 1

F2 - Acquisition Parameters
Date_ 20230614
Time 14.07 h
INSTRUM AVIII_400
PROBHD Z108618_0146 (
PULPROG zg30
TD 65536
SOLVENT CDCl3
NS 16
DS 2
SWH 8223.685 Hz
FIDRES 0.250967 Hz
AQ 3.9845889 sec
RG 80.6
DW 60.800 usec
DE 17.42 usec
TE 300.0 K
D1 1.00000000 sec
TD0 1
SFO1 400.1124708 MHz
NUC1 1H
P0 5.00 usec
P1 15.00 usec
PLW1 17.29199982 W

F2 - Processing parameters
SI 32768
SF 400.1100067 MHz
WDW EM
SSB 0
LB 0.30 Hz
GB 0
PC 1.00

Expanded region of the ¹H NMR spectrum of compound 15

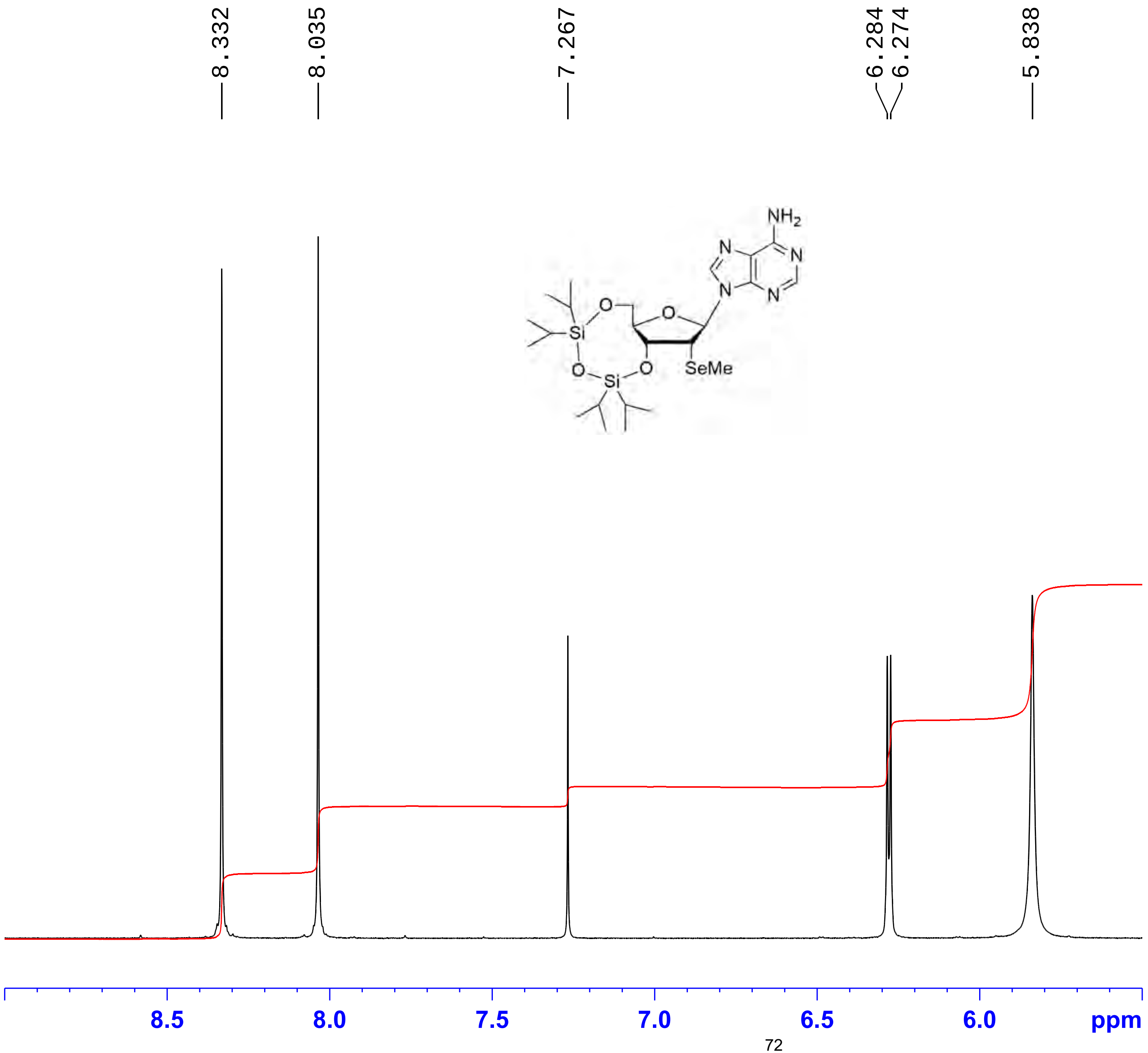


```
Current Data Parameters
NAME      LH-II-69 OLD NMR
EXPNO                   10
PROCNO                   1

F2 - Acquisition Parameters
Date_             20230614
Time                 14.07 h
INSTRUM          AVIII_400
PROBHD    Z108618_0146 (
PULPROG               zg30
TD                   65536
SOLVENT              CDCl3
NS                      16
DS                       2
SWH               8223.685 Hz
FIDRES            0.250967 Hz
AQ               3.9845889 sec
RG                    80.6
DW                  60.800 usec
DE                   17.42 usec
TE                   300.0 K
D1              1.00000000 sec
TD0                      1
SFO1           400.1124708 MHz
NUC1                    1H
P0                    5.00 usec
P1                   15.00 usec
PLW1           17.29199982 W

F2 - Processing parameters
SI                   32768
SF             400.1100067 MHz
WDW                     EM
SSB       0
LB                    0.30 Hz
GB        0
PC                    1.00
```

Expanded region of the ^{1}H NMR spectrum of compound 15

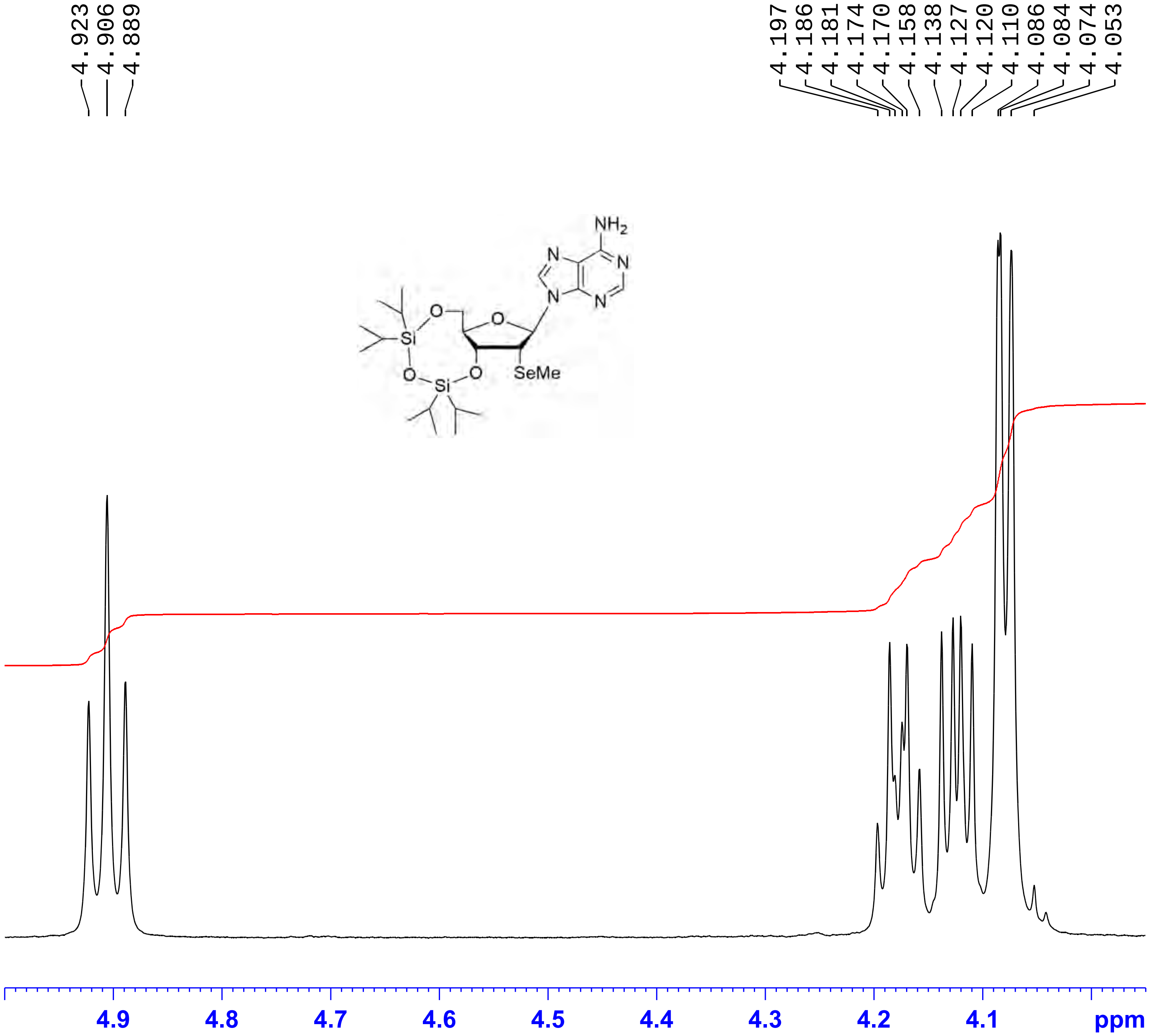


```
Current Data Parameters
NAME      LH-II-69 OLD NMR
EXPNO                   10
PROCNO                   1

F2 - Acquisition Parameters
Date_             20230614
Time                 14.07 h
INSTRUM           AVIII_400
PROBHD    Z108618_0146 (
PULPROG               zg30
TD                   65536
SOLVENT              CDCl3
NS                      16
DS                       2
SWH               8223.685 Hz
FIDRES            0.250967 Hz
AQ               3.9845889 sec
RG                    80.6
DW                  60.800 usec
DE                   17.42 usec
TE                   300.0 K
D1              1.00000000 sec
TD0                      1
SFO1           400.1124708 MHz
NUC1                    1H
P0                    5.00 usec
P1                   15.00 usec
PLW1           17.29199982 W

F2 - Processing parameters
SI                   32768
SF             400.1100067 MHz
WDW                     EM
SSB       0
LB                    0.30 Hz
GB        0
PC                    1.00
```

Expanded region of the ^{1}H NMR spectrum of compound 15

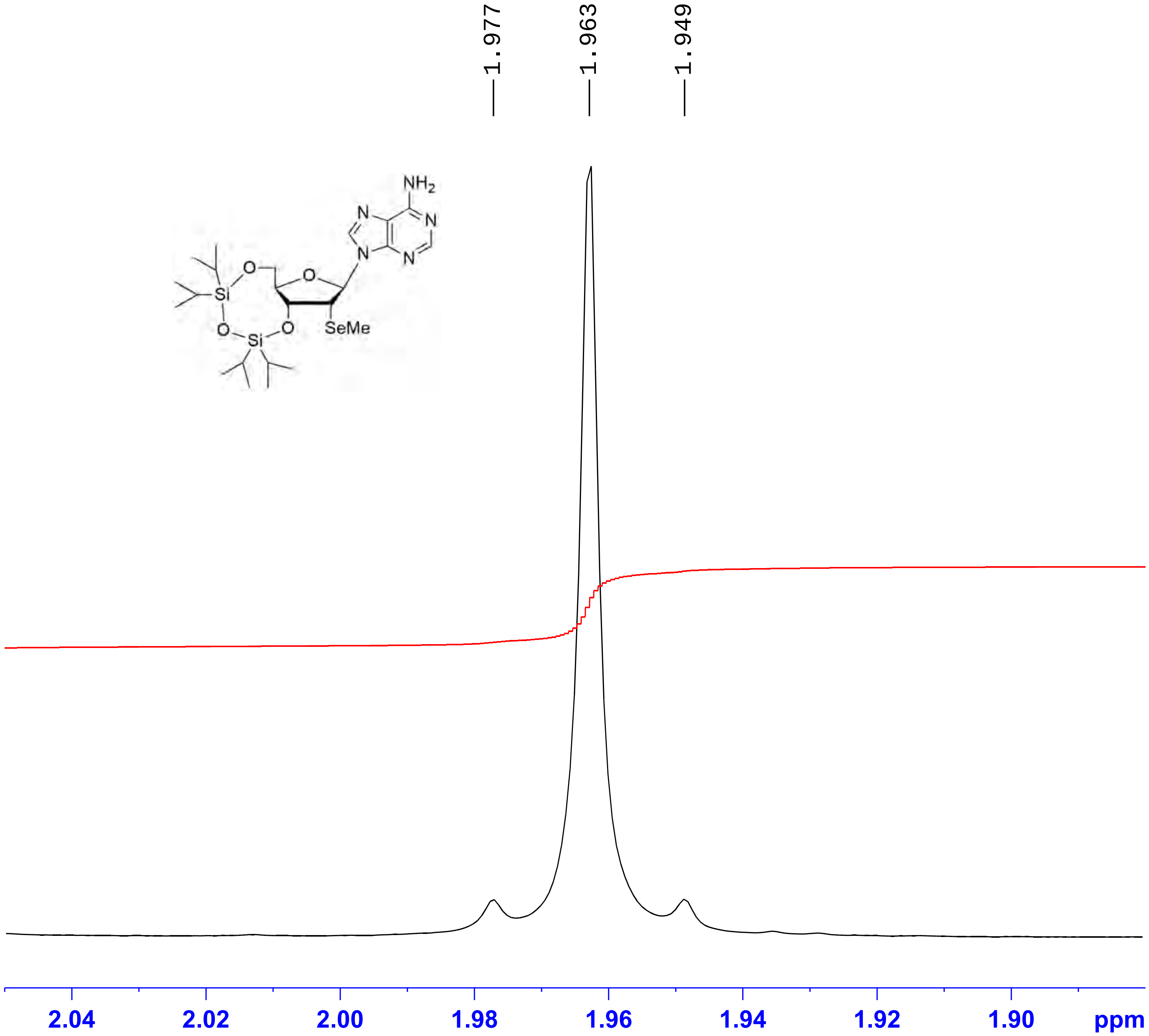

BRUKER

Current Data Parameters
NAME LH-II-69 OLD NMR
EXPNO 10
PROCNO 1

F2 - Acquisition Parameters
Date_ 20230614
Time 14.07 h
INSTRUM AVIII_400
PROBHD Z108618_0146 (
PULPROG zg30
TD 65536
SOLVENT CDCl3
NS 16
DS 2
SWH 8223.685 Hz
FIDRES 0.250967 Hz
AQ 3.9845889 sec
RG 80.6
DW 60.800 usec
DE 17.42 usec
TE 300.0 K
D1 1.00000000 sec
TD0 1
SFO1 400.1124708 MHz
NUC1 1H
P0 5.00 usec
P1 15.00 usec
PLW1 17.29199982 W

F2 - Processing parameters
SI 32768
SF 400.1100067 MHz
WDW EM
SSB 0
LB 0.30 Hz
GB 0
PC 1.00

Expanded region of the ^1H NMR spectrum of compound 15

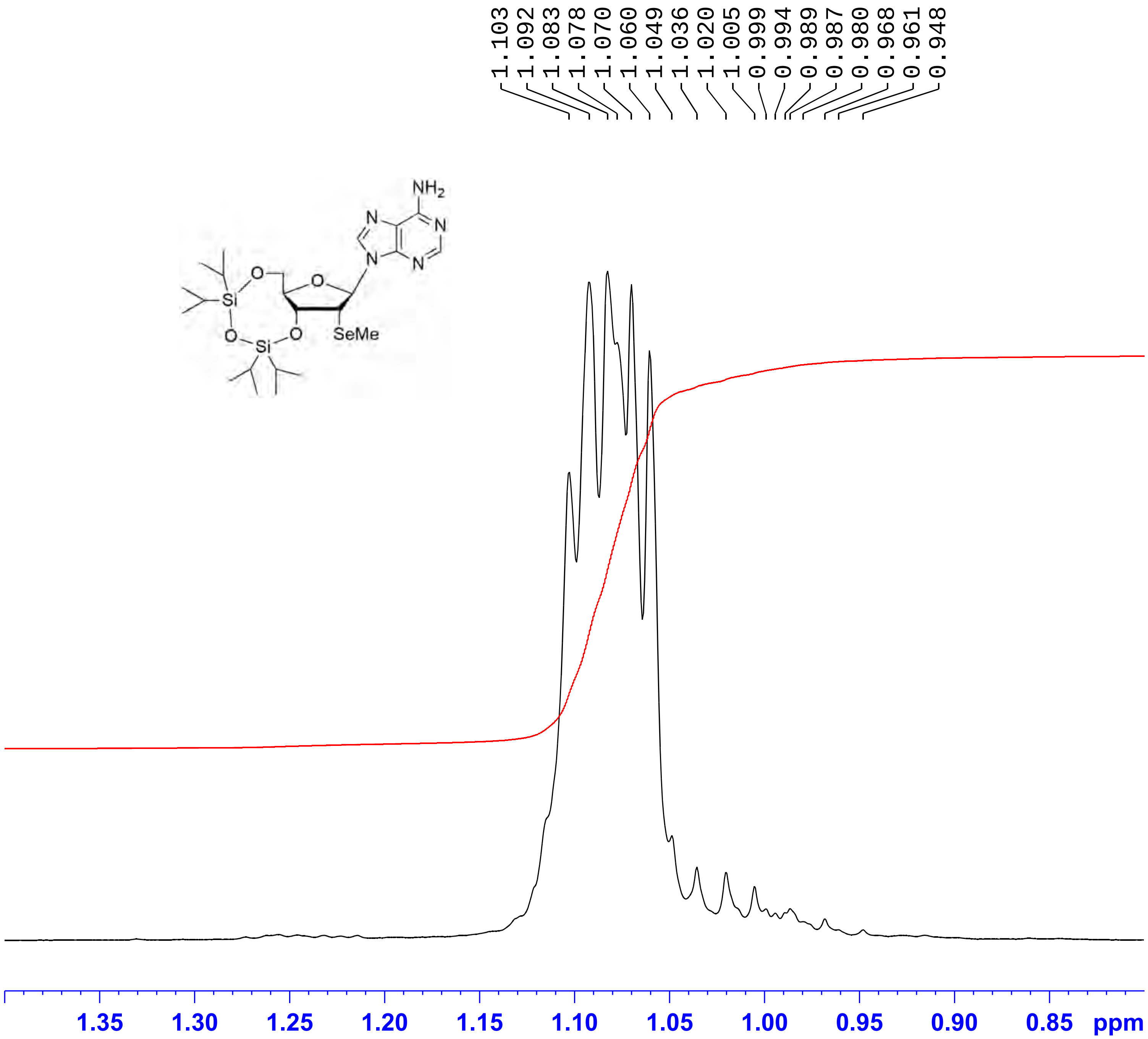

Current Data Parameters
NAME LH-II-69 OLD NMR
EXPNO 10
PROCNO 1

F2 - Acquisition Parameters
Date_ 20230614
Time 14.07 h
INSTRUM AVIII_400
PROBHD Z108618_0146 (
PULPROG zg30
TD 65536
SOLVENT CDCl3
NS 16
DS 2
SWH 8223.685 Hz
FIDRES 0.250967 Hz
AQ 3.9845889 sec
RG 80.6
DW 60.800 usec
DE 17.42 usec
TE 300.0 K
D1 1.00000000 sec
TD0 1
SFO1 400.1124708 MHz
NUC1 1H
P0 5.00 usec
P1 15.00 usec
PLW1 17.29199982 W

F2 - Processing parameters
SI 32768
SF 400.1100067 MHz
WDW EM
SSB 0
LB 0.30 Hz
GB 0
PC 1.00

^{13}C NMR spectrum of compound 15

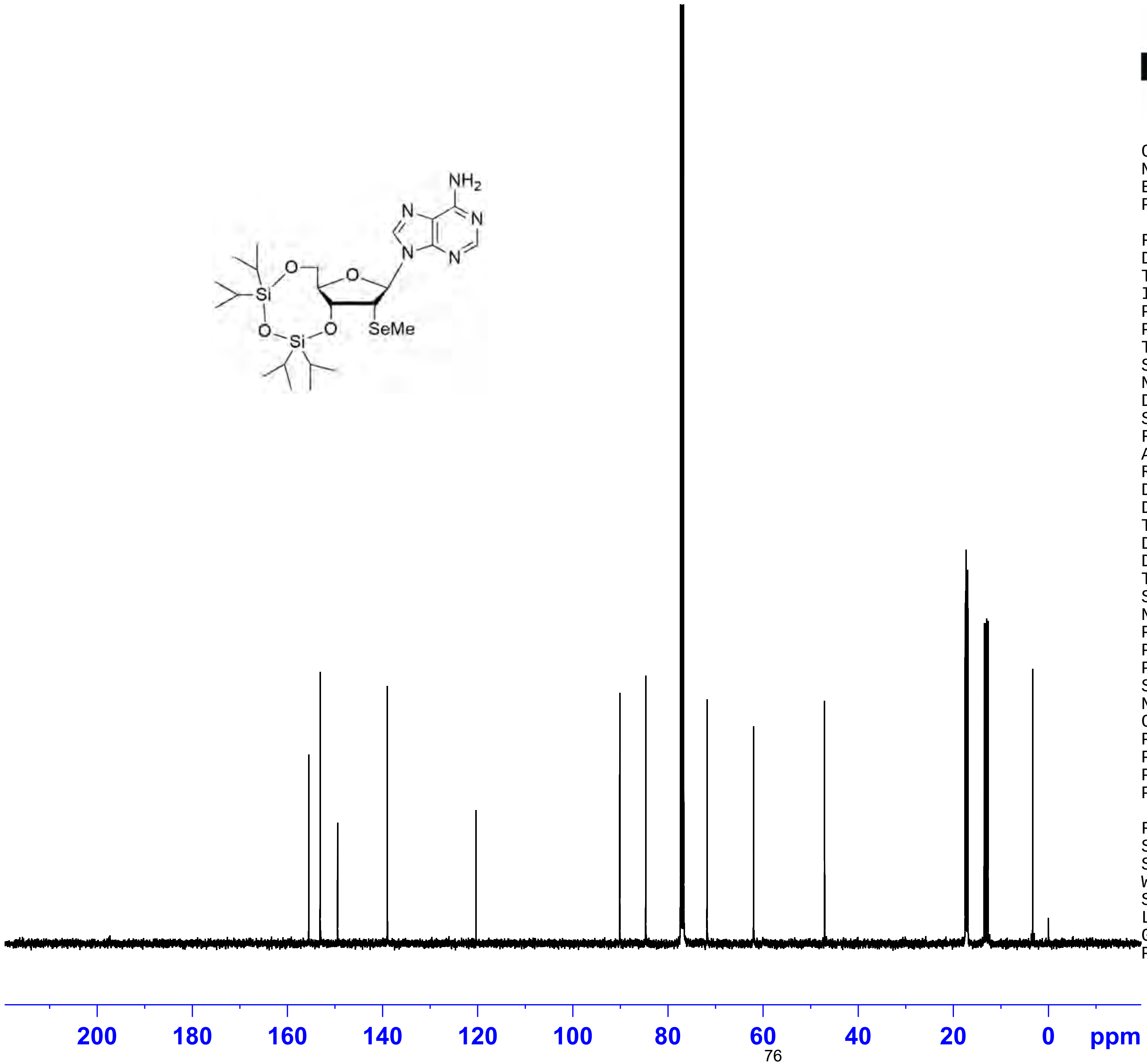

Current Data Parameters
NAME LH-II-69 OLD NMR
EXPNO 11
PROCNO 1

F2 - Acquisition Parameters
Date_ 20230614
Time 21.46 h
INSTRUM AVIII_400
PROBHD Z108618_0146 (
PULPROG zgpg30
TD 96150
SOLVENT CDCl3
NS 2500
DS 4
SWH 24038.461 Hz
FIDRES 0.500020 Hz
AQ 1.9999200 sec
RG 2050
DW 20.800 usec
DE 6.50 usec
TE 300.0 K
D1 1.00000000 sec
D11 0.03000000 sec
TD0 1
SFO1 100.6178003 MHz
NUC1 13C
P0 2.90 usec
P1 8.70 usec
PLW1 96.68000031 W
SFO2 400.1116004 MHz
NUC2 1H
CPDPRG[2 waltz64
PCPD2 90.00 usec
PLW2 17.29199982 W
PLW12 0.48032999 W
PLW13 0.24160001 W

F2 - Processing parameters
SI 131072
SF 100.6077420 MHz
WDW EM
SSB 0
LB 1.00 Hz
GB 0
PC 1.40

Expanded region of the ^{13}C NMR spectrum of compound 15

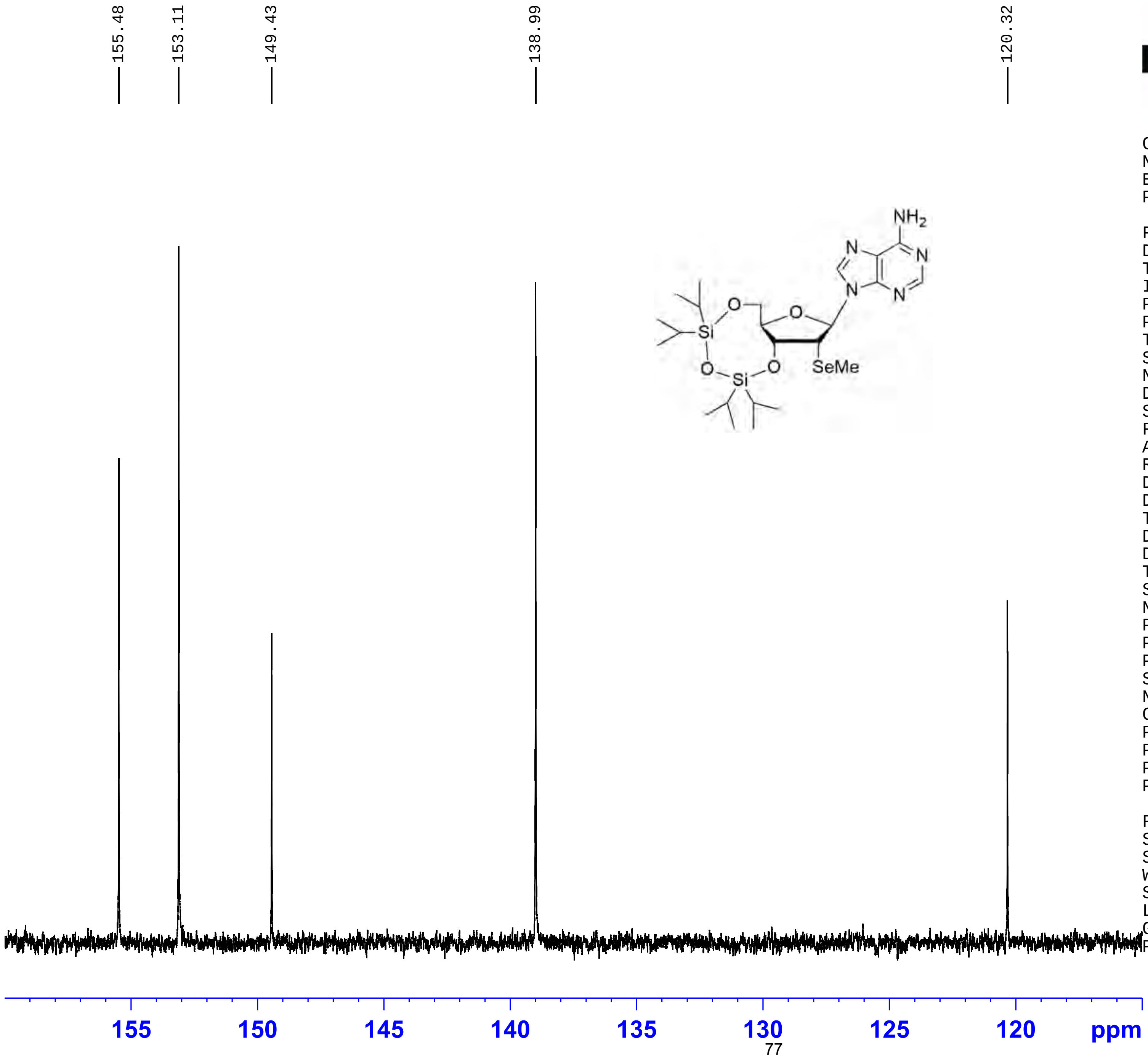

Current Data Parameters
NAME LH-II-69 OLD NMR
EXPNO 11
PROCNO 1

F2 - Acquisition Parameters
Date_ 20230614
Time 21.46 h
INSTRUM AVIII_400
PROBHD Z108618_0146 (
PULPROG zgpg30
TD 96150
SOLVENT CDCl3
NS 2500
DS 4
SWH 24038.461 Hz
FIDRES 0.500020 Hz
AQ 1.9999200 sec
RG 2050
DW 20.800 usec
DE 6.50 usec
TE 300.0 K
D1 1.00000000 sec
D11 0.03000000 sec
TD0 1
SFO1 100.6178003 MHz
NUC1 13C
P0 2.90 usec
P1 8.70 usec
PLW1 96.68000031 W
SFO2 400.1116004 MHz
NUC2 1H
CPDPRG[2 waltz64
PCPD2 90.00 usec
PLW2 17.29199982 W
PLW12 0.48032999 W
PLW13 0.24160001 W

F2 - Processing parameters
SI 131072
SF 100.6077420 MHz
WDW EM
SSB 0
LB 1.00 Hz
GB 0
PC 1.40

Expanded region of the ^{13}C NMR spectrum of compound 15

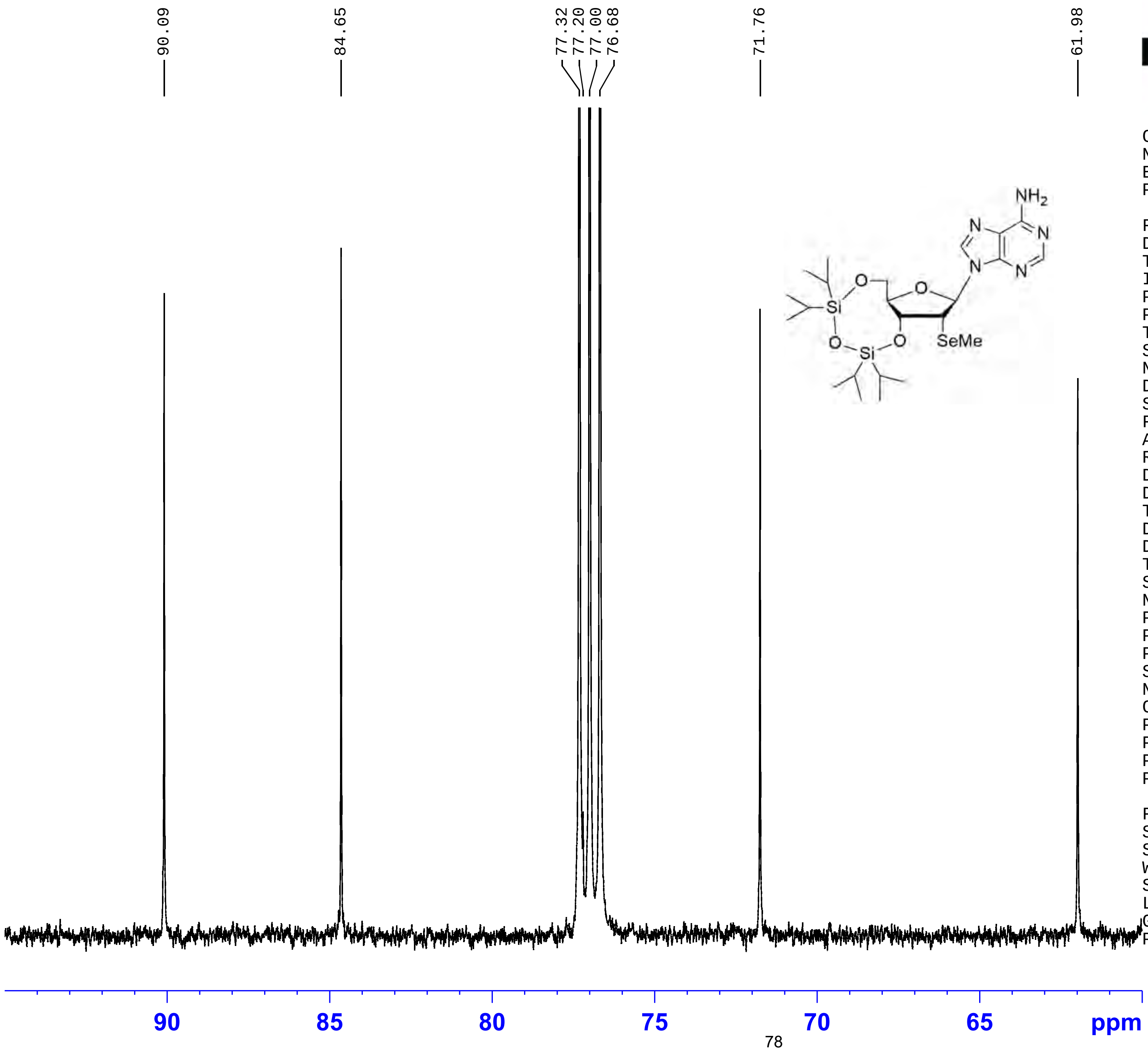


```
Current Data Parameters
NAME       LH-II-69 OLD NMR
EXPNO                    11
PROCNO                    1

F2 - Acquisition Parameters
Date_              20230614
Time                  21.46 h
INSTRUM           AVIII_400
PROBHD    Z108618_0146 (
PULPROG             zgpg30
TD                   96150
SOLVENT              CDCl3
NS                    2500
DS                       4
SWH              24038.461 Hz
FIDRES            0.500020 Hz
AQ               1.9999200 sec
RG                    2050
DW                  20.800 usec
DE                    6.50 usec
TE                   300.0 K
D1              1.00000000 sec
D11             0.03000000 sec
TD0                      1
SFO1           100.6178003 MHz
NUC1                   13C
P0                    2.90 usec
P1                    8.70 usec
PLW1           96.68000031 W
SFO2           400.1116004 MHz
NUC2                    1H
CPDPRG[2           waltz64
PCPD2                90.00 usec
PLW2           17.29199982 W
PLW12           0.48032999 W
PLW13           0.24160001 W

F2 - Processing parameters
SI                  131072
SF             100.6077420 MHz
WDW                     EM
SSB       0
LB                    1.00 Hz
GB        0
PC                    1.40
```

Expanded region of the ^{13}C NMR spectrum of compound 15

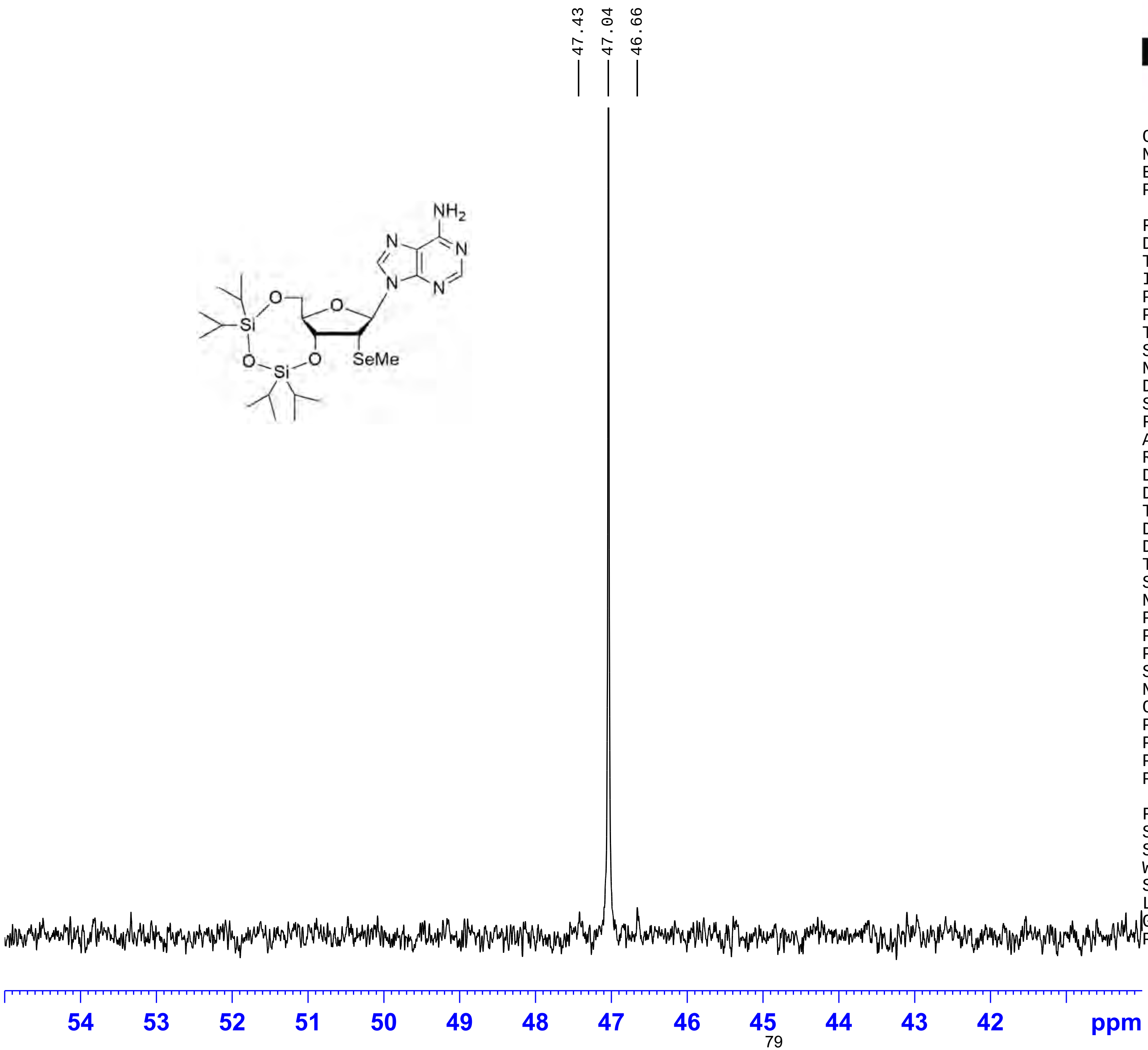


```
Current Data Parameters
NAME        LH-II-69 OLD NMR
EXPNO                     11
PROCNO                     1

F2 - Acquisition Parameters
Date_               20230614
Time                   21.46 h
INSTRUM             AVIII_400
PROBHD         Z108618_0146 (
PULPROG              zgpg30
TD                     96150
SOLVENT                CDCl3
NS                      2500
DS                         4
SWH                24038.461 Hz
FIDRES              0.500020 Hz
AQ                 1.9999200 sec
RG                      2050
DW                    20.800 usec
DE                      6.50 usec
TE                     300.0 K
D1                1.00000000 sec
D11               0.03000000 sec
TD0                        1
SFO1             100.6178003 MHz
NUC1                     13C
P0                      2.90 usec
P1                      8.70 usec
PLW1             96.68000031 W
SFO2             400.1116004 MHz
NUC2                      1H
CPDPRG[2             waltz64
PCPD2                  90.00 usec
PLW2             17.29199982 W
PLW12             0.48032999 W
PLW13             0.24160001 W

F2 - Processing parameters
SI                    131072
SF               100.6077420 MHz
WDW                       EM
SSB      0
LB                      1.00 Hz
GB       0
PC                      1.40
```

Expanded region of the ^{13}C NMR spectrum of compound 15

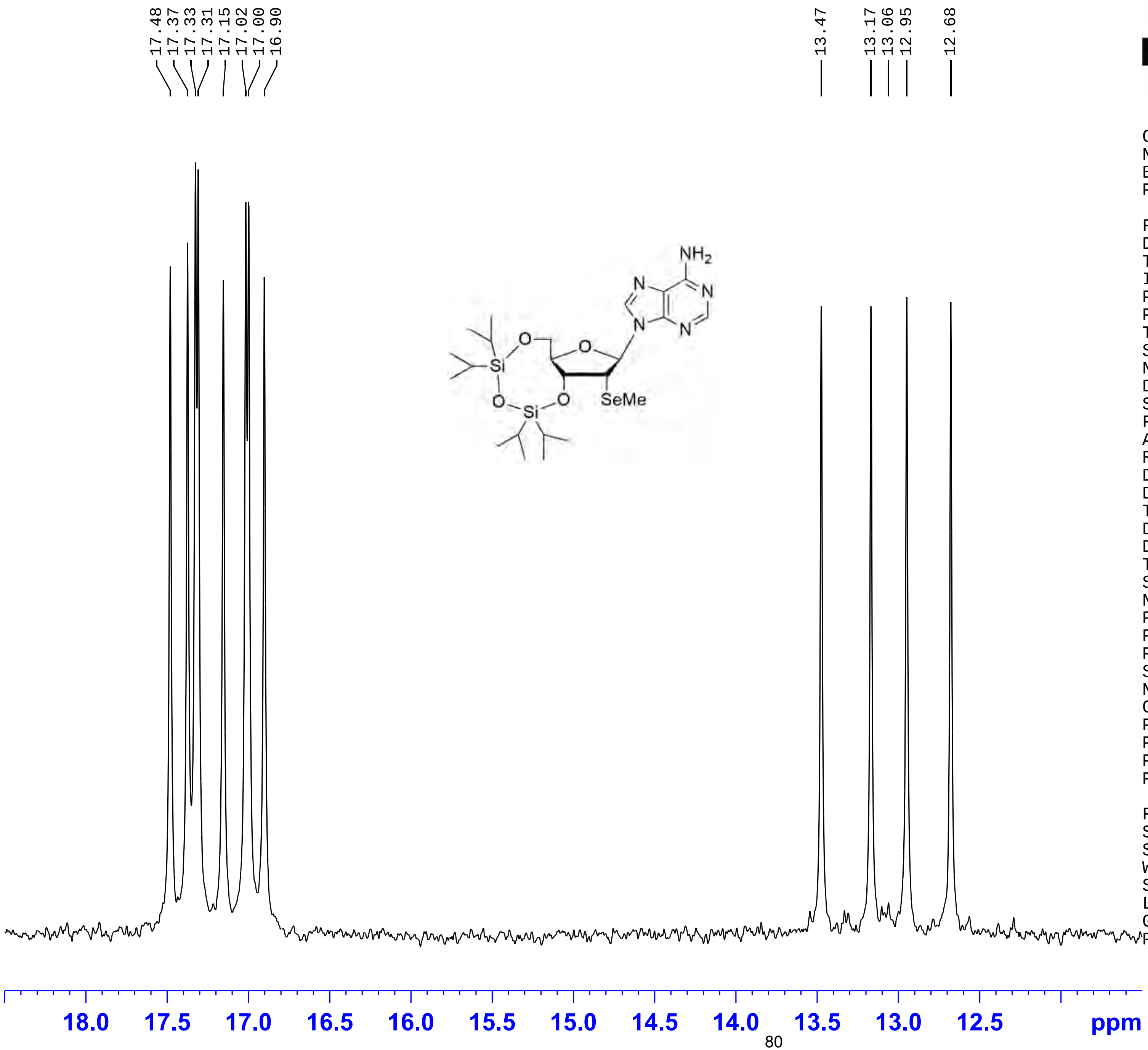

Current Data Parameters
NAME LH-II-69 OLD NMR
EXPNO 11
PROCNO 1

F2 - Acquisition Parameters
Date_ 20230614
Time 21.46 h
INSTRUM AVIII_400
PROBHD Z108618_0146 (
PULPROG zgpg30
TD 96150
SOLVENT CDCl3
NS 2500
DS 4
SWH 24038.461 Hz
FIDRES 0.500020 Hz
AQ 1.9999200 sec
RG 2050
DW 20.800 usec
DE 6.50 usec
TE 300.0 K
D1 1.00000000 sec
D11 0.03000000 sec
TD0 1
SFO1 100.6178003 MHz
NUC1 13C
P0 2.90 usec
P1 8.70 usec
PLW1 96.68000031 W
SFO2 400.1116004 MHz
NUC2 1H
CPDPRG[2 waltz64
PCPD2 90.00 usec
PLW2 17.29199982 W
PLW12 0.48032999 W
PLW13 0.24160001 W

F2 - Processing parameters
SI 131072
SF 100.6077420 MHz
WDW EM
SSB 0
LB 1.00 Hz
GB 0
PC 1.40

Expanded region of the ^{13}C NMR spectrum of compound 15

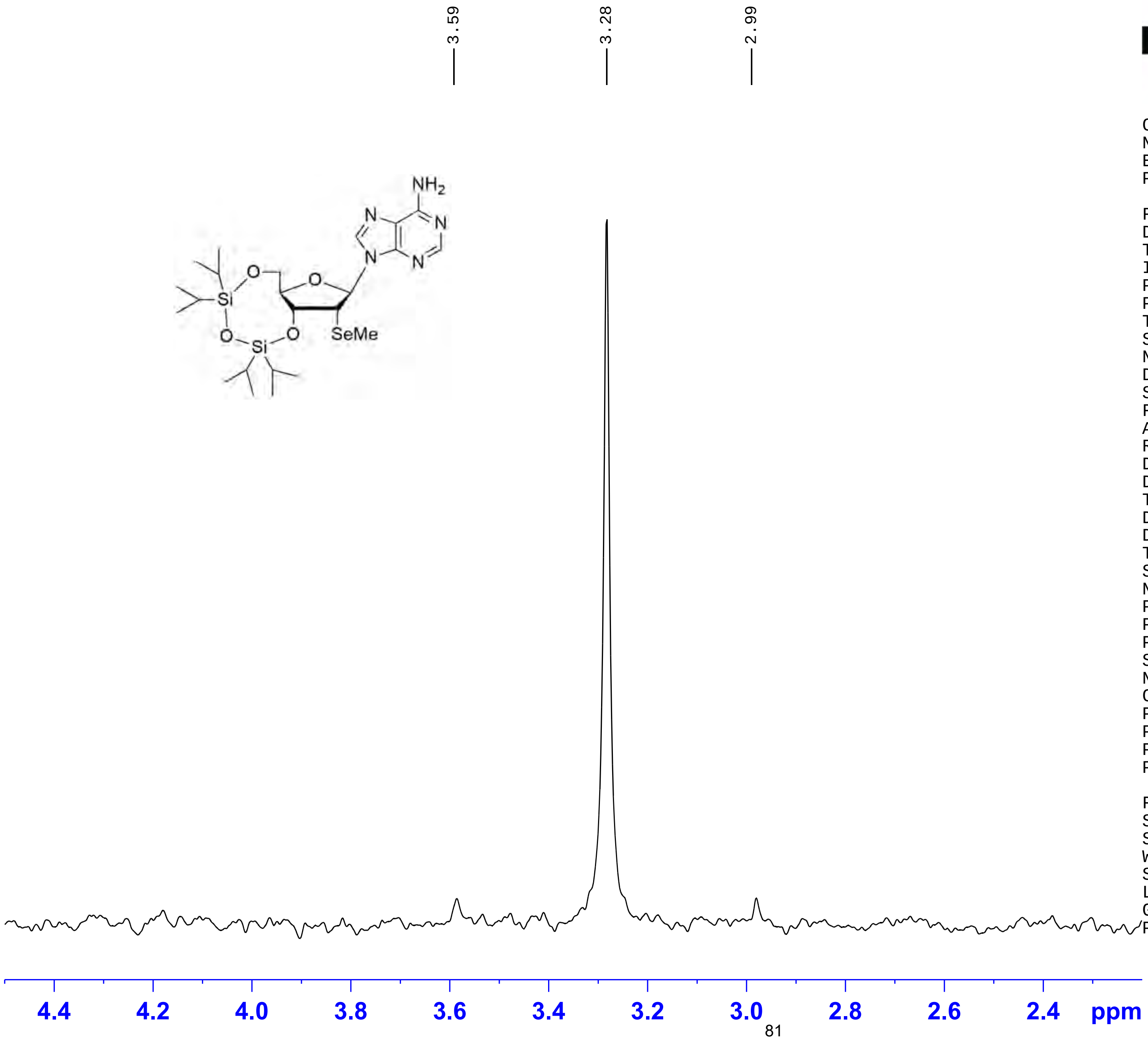

Current Data Parameters

NAME	LH-II-69 OLD NMR
EXPNO	11
PROCNO	1

F2 - Acquisition Parameters

Date_	20230614
Time	21.46 h
INSTRUM	AVIII_400
PROBHD	Z108618_0146 (
PULPROG	zgpg30
TD	96150
SOLVENT	CDCl3
NS	2500
DS	4
SWH	24038.461 Hz
FIDRES	0.500020 Hz
AQ	1.9999200 sec
RG	2050
DW	20.800 usec
DE	6.50 usec
TE	300.0 K
D1	1.00000000 sec
D11	0.03000000 sec
TD0	1
SFO1	100.6178003 MHz
NUC1	13C
P0	2.90 usec
P1	8.70 usec
PLW1	96.68000031 W
SFO2	400.1116004 MHz
NUC2	1H
CPDPRG[2	waltz64
PCPD2	90.00 usec
PLW2	17.29199982 W
PLW12	0.48032999 W
PLW13	0.24160001 W

F2 - Processing parameters

SI	131072
SF	100.6077420 MHz
WDW	EM
SSB	0
LB	1.00 Hz
GB	0
PC	1.40

^{1}H-coupled ^{13}C NMR spectrum of compound 15

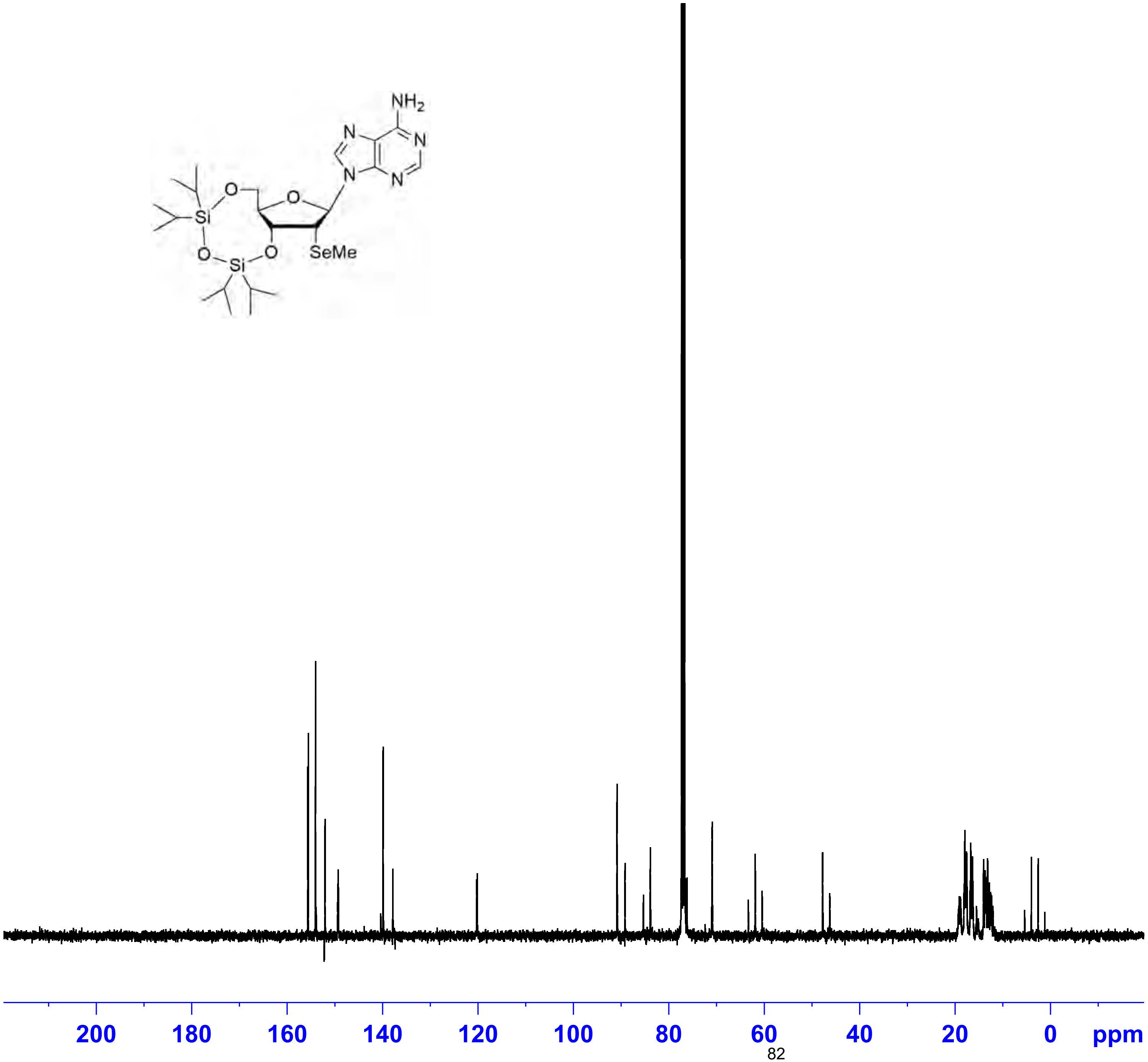

Current Data Parameters
NAME LH-II-69 Analytical
EXPNO 11
PROCNO 1

F2 - Acquisition Parameters
Date_ 20230608
Time 3.39 h
INSTRUM AVIII_400
PROBHD Z108618_0817 (
PULPROG zgpg30
TD 96150
SOLVENT CDCl3
NS 5000
DS 4
SWH 24038.461 Hz
FIDRES 0.500020 Hz
AQ 1.9999200 sec
RG 161
DW 20.800 usec
DE 6.50 usec
TE 294.8 K
D1 1.00000000 sec
D11 0.03000000 sec
TD0 1
SFO1 100.5675047 MHz
NUC1 13C
P0 2.00 usec
P1 6.00 usec
PLW1 96.68000031 W
SFO2 399.9115996 MHz
NUC2 1H
CPDPRG[2 waltz64
PCPD2 90.00 usec
PLW2 31.62299919 W
PLW12 1.88960004 W
PLW13 0.95043999 W

F2 - Processing parameters
SI 131072
SF 100.5574557 MHz
WDW EM
SSB 0
LB 1.00 Hz
GB 0
PC 1.40

Expanded region of the ^{1}H-coupled ^{13}C NMR spectrum of compound 15

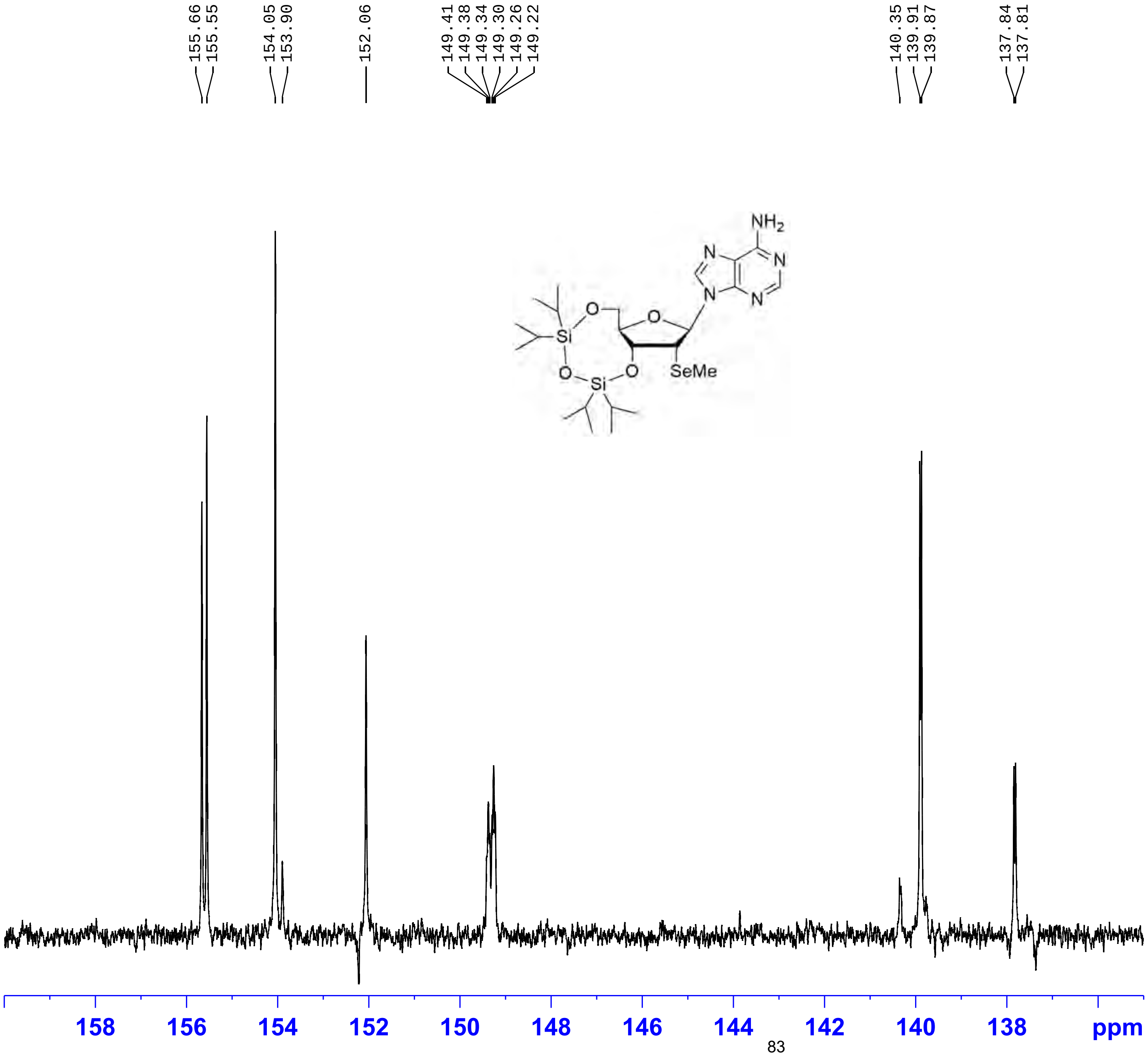

Current Data Parameters
NAME LH-II-69 Analytical
EXPNO 11
PROCNO 1

F2 - Acquisition Parameters
Date_ 20230608
Time 3.39 h
INSTRUM AVIII_400
PROBHD Z108618_0817 (
PULPROG zgpg30
TD 96150
SOLVENT CDCl3
NS 5000
DS 4
SWH 24038.461 Hz
FIDRES 0.500020 Hz
AQ 1.9999200 sec
RG 161
DW 20.800 usec
DE 6.50 usec
TE 294.8 K
D1 1.00000000 sec
D11 0.03000000 sec
TD0 1
SFO1 100.5675047 MHz
NUC1 13C
P0 2.00 usec
P1 6.00 usec
PLW1 96.68000031 W
SFO2 399.9115996 MHz
NUC2 1H
CPDPRG[2 waltz64
PCPD2 90.00 usec
PLW2 31.62299919 W
PLW12 1.88960004 W
PLW13 0.95043999 W

F2 - Processing parameters
SI 131072
SF 100.5574557 MHz
WDW EM
SSB 0
LB 1.00 Hz
GB 0
PC 1.40

Expanded region of the ^{1}H-coupled ^{13}C NMR spectrum of compound 15

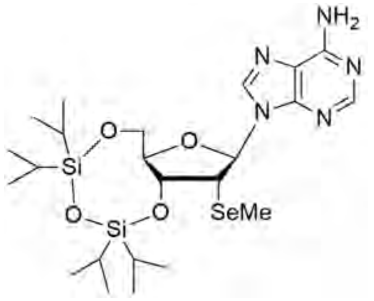

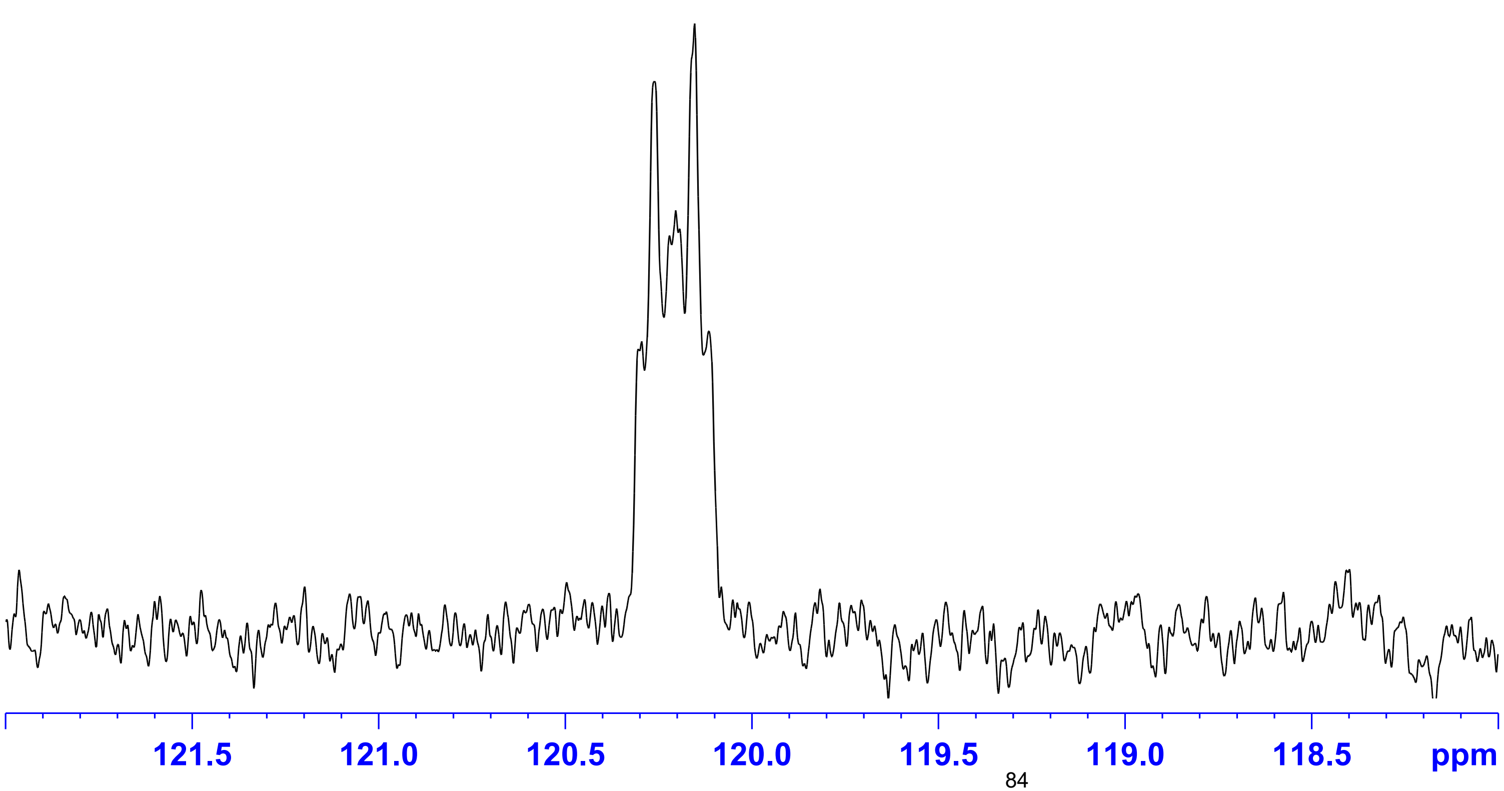


```
Current Data Parameters
NAME       LH-II-69 Analytical
EXPNO                    11
PROCNO                    1

F2 - Acquisition Parameters
Date_              20230608
Time                   3.39 h
INSTRUM            AVIII_400
PROBHD    Z108618_0817 (
PULPROG             zgpg30
TD                   96150
SOLVENT              CDCl3
NS                    5000
DS                       4
SWH              24038.461 Hz
FIDRES            0.500020 Hz
AQ               1.9999200 sec
RG                     161
DW                  20.800 usec
DE                    6.50 usec
TE                   294.8 K
D1              1.00000000 sec
D11             0.03000000 sec
TD0                      1
SFO1           100.5675047 MHz
NUC1                   13C
P0                    2.00 usec
P1                    6.00 usec
PLW1           96.68000031 W
SFO2           399.9115996 MHz
NUC2                    1H
CPDPRG[2           waltz64
PCPD2                90.00 usec
PLW2           31.62299919 W
PLW12           1.88960004 W
PLW13           0.95043999 W

F2 - Processing parameters
SI                  131072
SF             100.5574557 MHz
WDW                     EM
SSB      0
LB                    1.00 Hz
GB       0
PC                    1.40
```

Expanded region of the ^{1}H-coupled ^{13}C NMR spectrum of compound 15

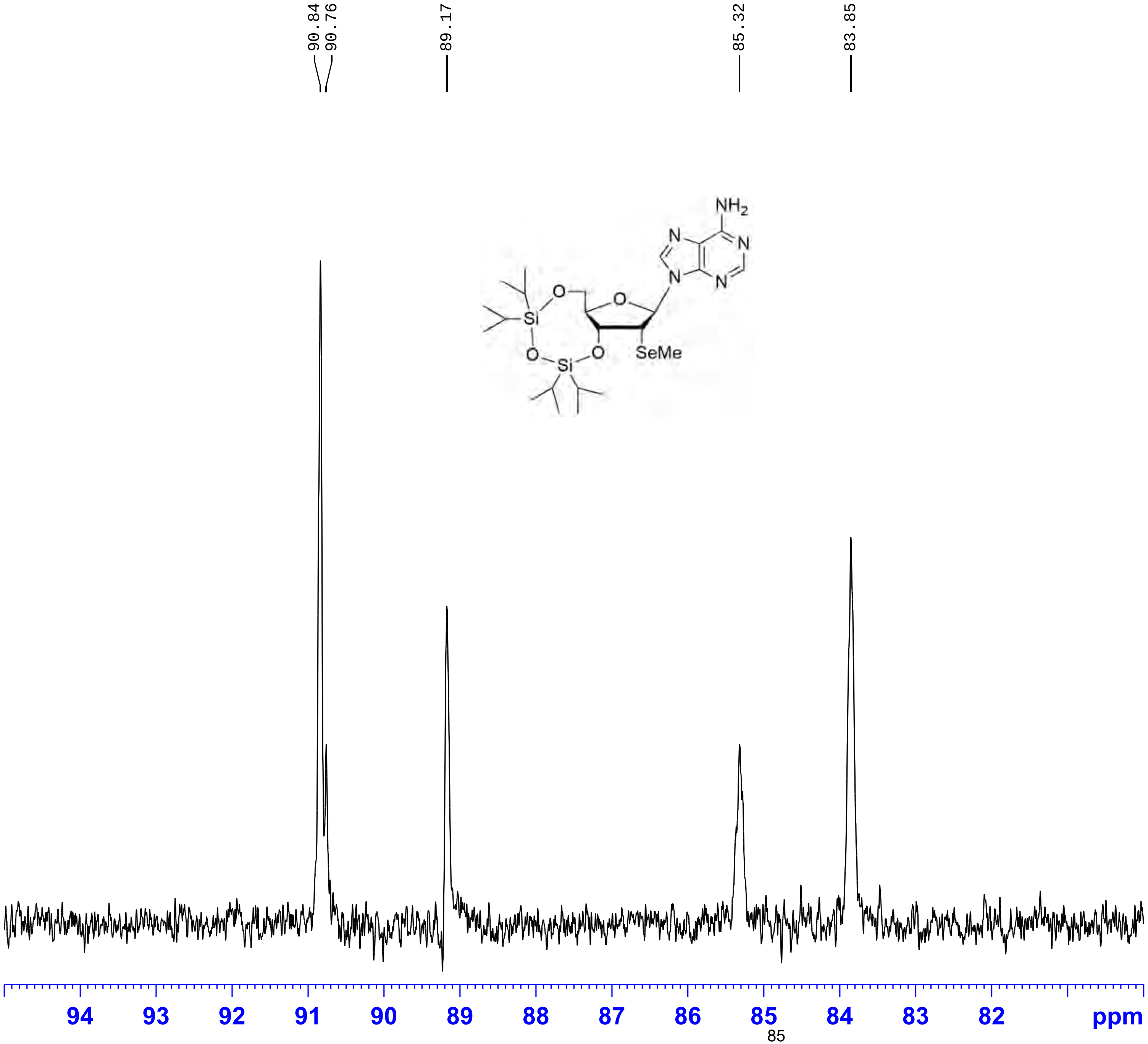

Current Data Parameters
NAME LH-II-69 Analytical
EXPNO 11
PROCNO 1

F2 - Acquisition Parameters
Date_ 20230608
Time 3.39 h
INSTRUM AVIII_400
PROBHD Z108618_0817 (
PULPROG zgpg30
TD 96150
SOLVENT CDCl3
NS 5000
DS 4
SWH 24038.461 Hz
FIDRES 0.500020 Hz
AQ 1.9999200 sec
RG 161
DW 20.800 usec
DE 6.50 usec
TE 294.8 K
D1 1.00000000 sec
D11 0.03000000 sec
TD0 1
SFO1 100.5675047 MHz
NUC1 13C
P0 2.00 usec
P1 6.00 usec
PLW1 96.68000031 W
SFO2 399.9115996 MHz
NUC2 1H
CPDPRG[2 waltz64
PCPD2 90.00 usec
PLW2 31.62299919 W
PLW12 1.88960004 W
PLW13 0.95043999 W

F2 - Processing parameters
SI 131072
SF 100.5574557 MHz
WDW EM
SSB 0
LB 1.00 Hz
GB 0
PC 1.40

Expanded region of the ^{1}H-coupled ^{13}C NMR spectrum of compound 15

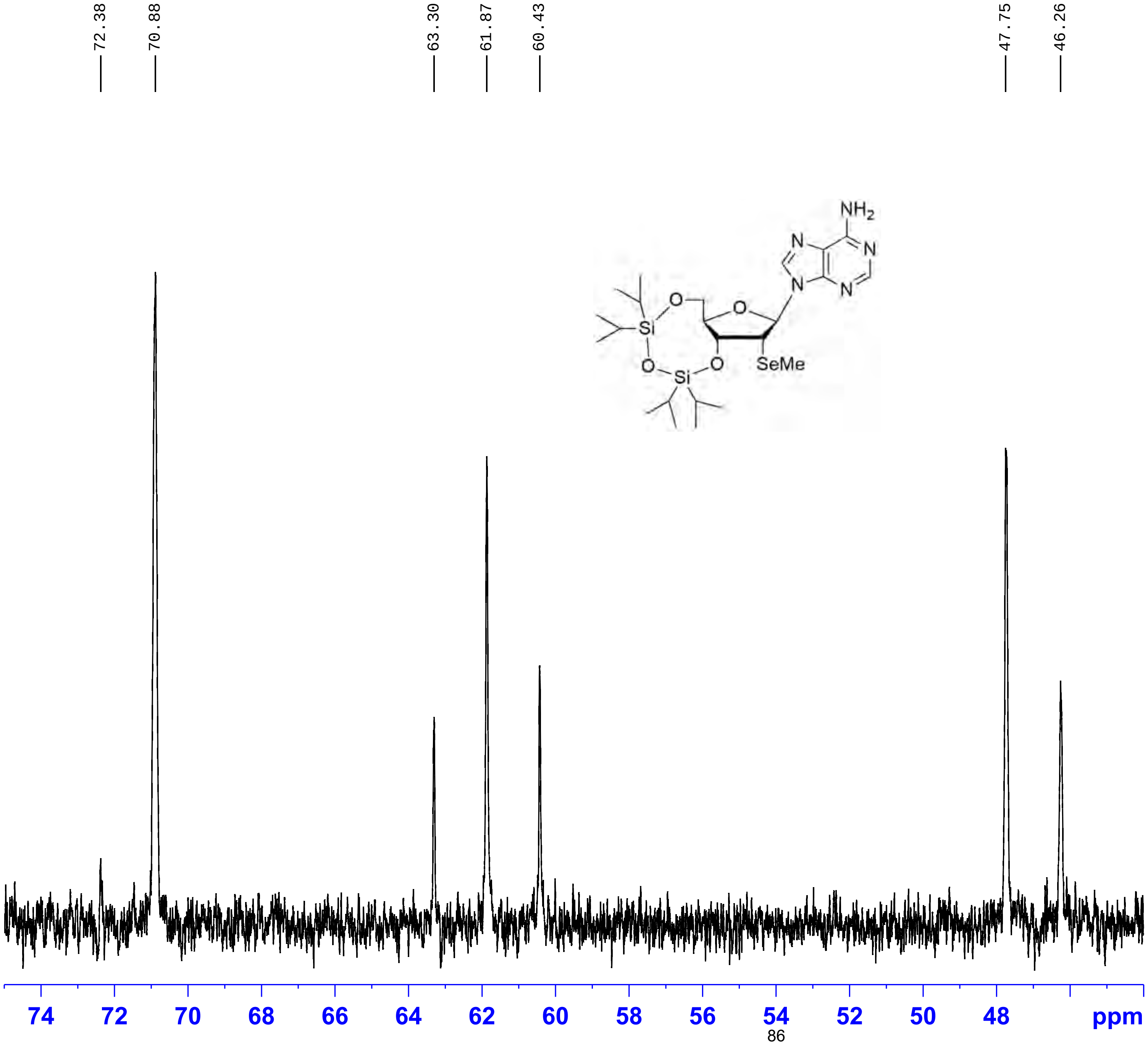


```
Current Data Parameters
NAME      LH-II-69 Analytical
EXPNO                    11
PROCNO                    1

F2 - Acquisition Parameters
Date_              20230608
Time                  3.39 h
INSTRUM           AVIII_400
PROBHD     Z108618_0817 (
PULPROG             zgpg30
TD                   96150
SOLVENT              CDCl3
NS                    5000
DS                       4
SWH              24038.461 Hz
FIDRES            0.500020 Hz
AQ               1.9999200 sec
RG                     161
DW                  20.800 usec
DE                    6.50 usec
TE                   294.8 K
D1              1.00000000 sec
D11             0.03000000 sec
TD0                      1
SFO1           100.5675047 MHz
NUC1                   13C
P0                    2.00 usec
P1                    6.00 usec
PLW1           96.68000031 W
SFO2           399.9115996 MHz
NUC2                    1H
CPDPRG[2           waltz64
PCPD2                90.00 usec
PLW2           31.62299919 W
PLW12           1.88960004 W
PLW13           0.95043999 W

F2 - Processing parameters
SI                  131072
SF             100.5574557 MHz
WDW                     EM
SSB       0
LB                    1.00 Hz
GB        0
PC                    1.40
```

Expanded region of the ^{1}H-coupled ^{13}C NMR spectrum of compound 15

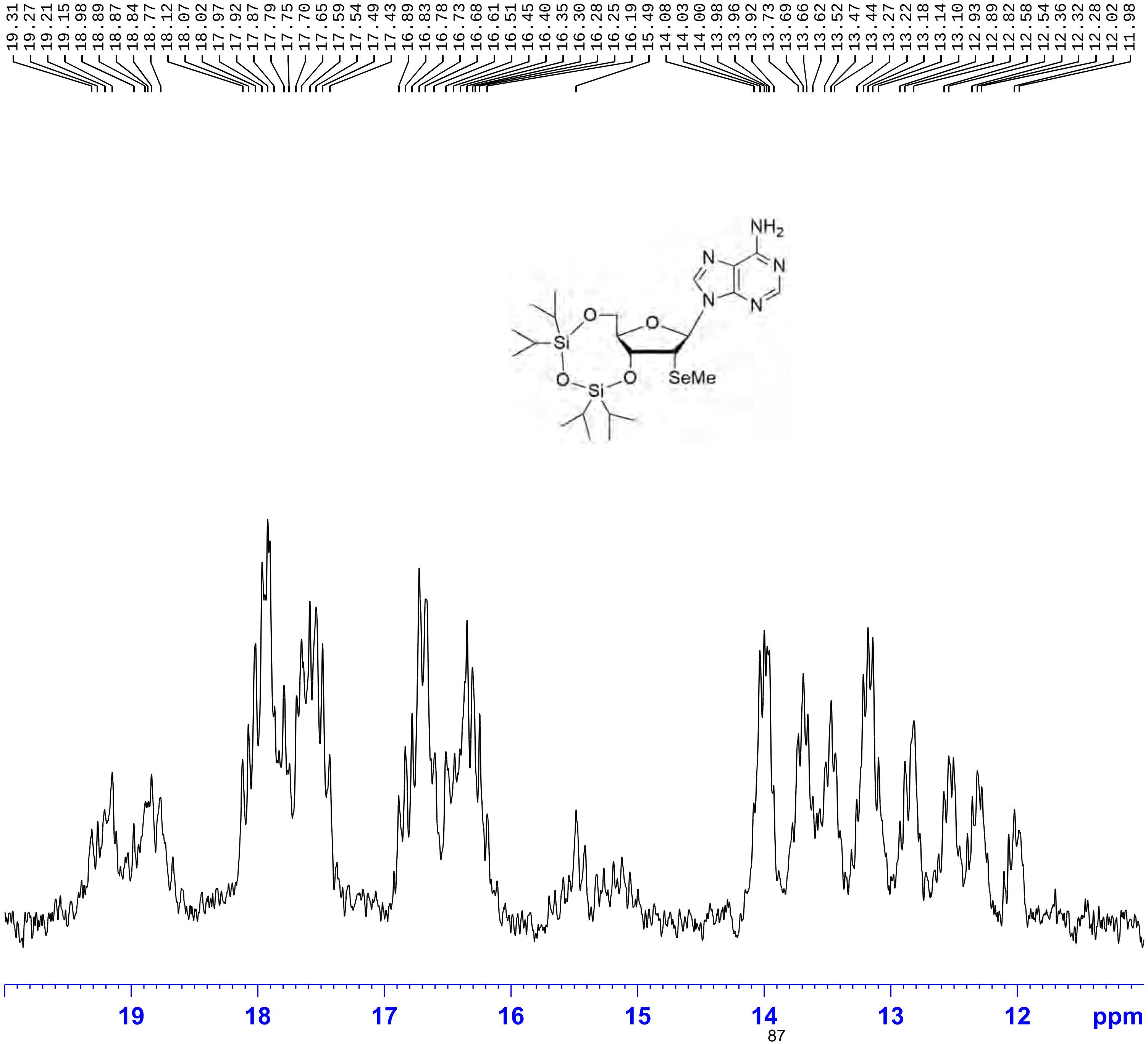

Current Data Parameters

NAME	LH-II-69 Analytical
EXPNO	11
PROCNO	1

F2 - Acquisition Parameters

Date_	20230608
Time	3.39 h
INSTRUM	AVIII_400
PROBHD	Z108618_0817 (
PULPROG	zgpg30
TD	96150
SOLVENT	CDCl3
NS	5000
DS	4
SWH	24038.461 Hz
FIDRES	0.500020 Hz
AQ	1.9999200 sec
RG	161
DW	20.800 usec
DE	6.50 usec
TE	294.8 K
D1	1.00000000 sec
D11	0.03000000 sec
TD0	1
SFO1	100.5675047 MHz
NUC1	13C
P0	2.00 usec
P1	6.00 usec
PLW1	96.68000031 W
SFO2	399.9115996 MHz
NUC2	1H
CPDPRG[2	waltz64
PCPD2	90.00 usec
PLW2	31.62299919 W
PLW12	1.88960004 W
PLW13	0.95043999 W

F2 - Processing parameters

SI	131072
SF	100.5574557 MHz
WDW	EM
SSB	0
LB	1.00 Hz
GB	0
PC	1.40

Expanded region of the ^{1}H-coupled ^{13}C NMR spectrum of compound 15

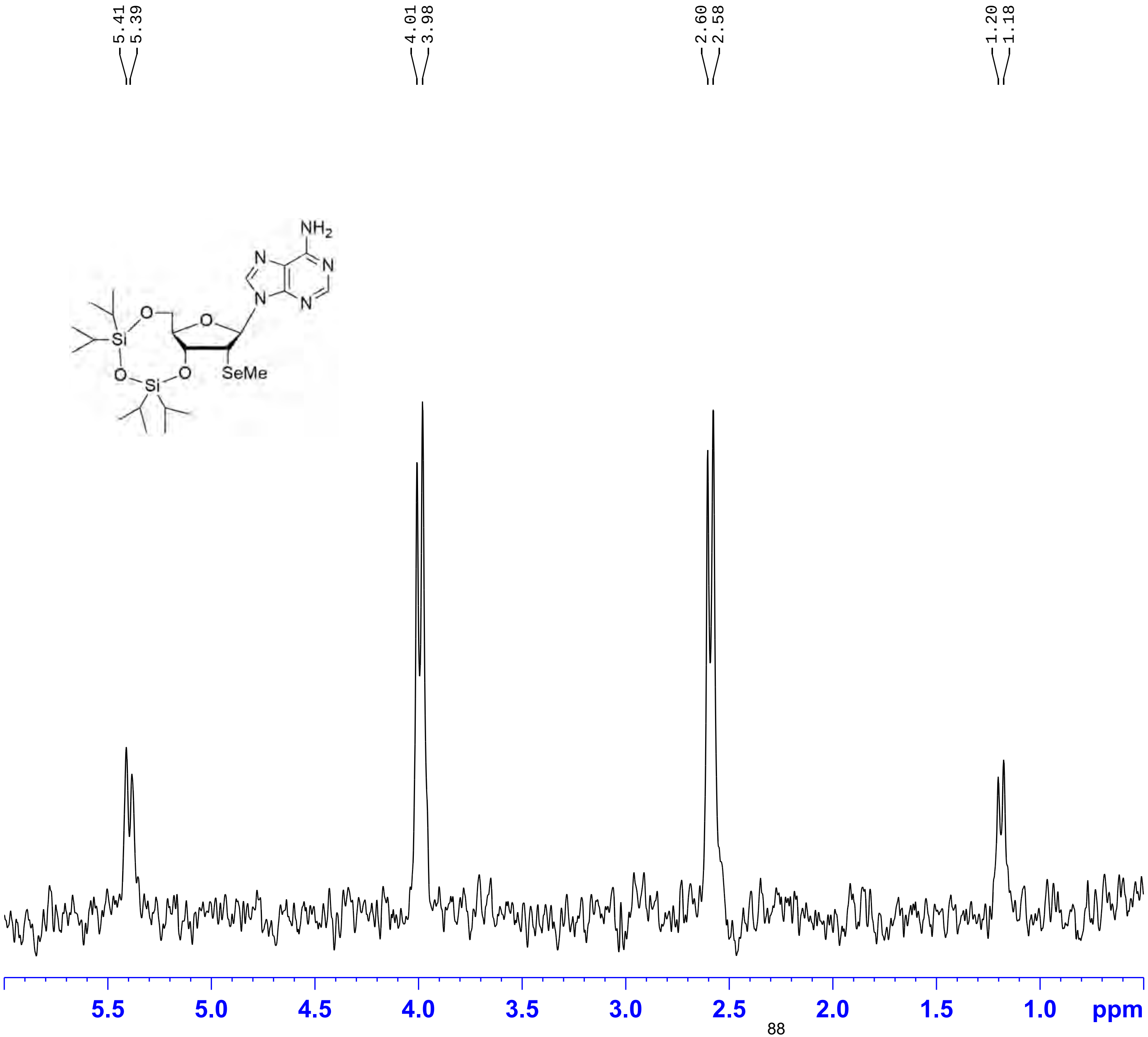


```
Current Data Parameters
NAME      LH-II-69 Analytical
EXPNO                     11
PROCNO                     1

F2 - Acquisition Parameters
Date_               20230608
Time                    3.39 h
INSTRUM             AVIII_400
PROBHD     Z108618_0817 (
PULPROG              zgpg30
TD                    96150
SOLVENT               CDCl3
NS                     5000
DS                        4
SWH               24038.461 Hz
FIDRES             0.500020 Hz
AQ                1.9999200 sec
RG                      161
DW                   20.800 usec
DE                     6.50 usec
TE                    294.8 K
D1               1.00000000 sec
D11              0.03000000 sec
TD0                       1
SFO1            100.5675047 MHz
NUC1                    13C
P0                     2.00 usec
P1                     6.00 usec
PLW1            96.68000031 W
SFO2            399.9115996 MHz
NUC2                     1H
CPDPRG[2            waltz64
PCPD2                 90.00 usec
PLW2            31.62299919 W
PLW12            1.88960004 W
PLW13            0.95043999 W

F2 - Processing parameters
SI                   131072
SF              100.5574557 MHz
WDW                      EM
SSB      0
LB                     1.00 Hz
GB       0
PC                     1.40
```

^{13}C DEPT-135 NMR spectrum of compound 15

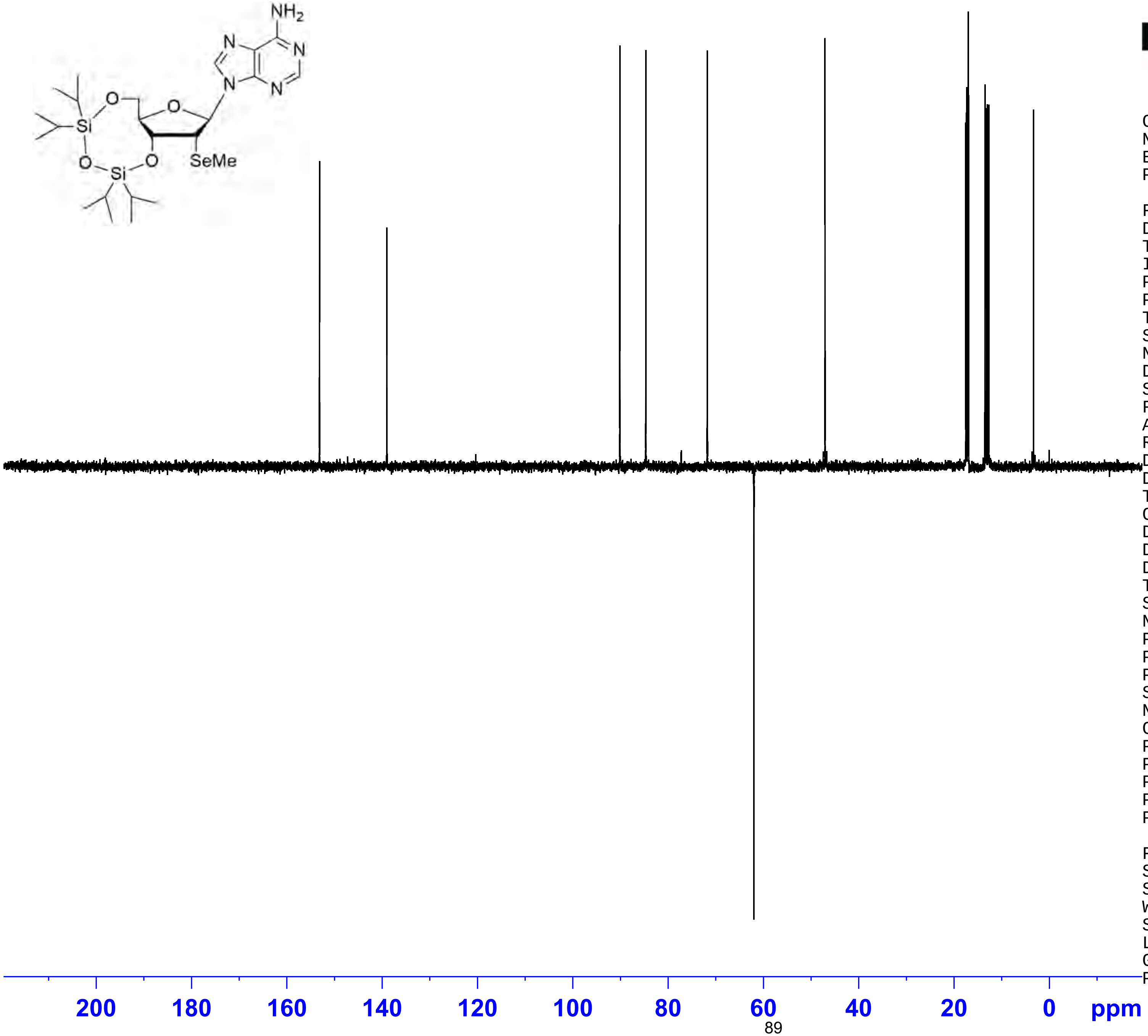

Current Data Parameters
NAME LH-II-69 OLD NMR
EXPNO 13
PROCNO 1

F2 - Acquisition Parameters
Date_ 20230615
Time 0.40 h
INSTRUM AVIII_400
PROBHD Z108618_0146 (
PULPROG dept135
TD 65536
SOLVENT CDCl3
NS 1500
DS 4
SWH 24038.461 Hz
FIDRES 0.733596 Hz
AQ 1.3631488 sec
RG 2050
DW 20.800 usec
DE 6.50 usec
TE 300.0 K
CNST2 145.0000000
D1 2.00000000 sec
D2 0.00344828 sec
D12 0.00002000 sec
TD0 1
SFO1 100.6178003 MHz
NUC1 13C
P1 8.70 usec
P2 17.40 usec
PLW1 96.68000031 W
SFO2 400.1116004 MHz
NUC2 1H
CPDPRG[2 waltz64
P3 15.00 usec
P4 30.00 usec
PCPD2 90.00 usec
PLW2 17.29199982 W
PLW12 0.48032999 W

F2 - Processing parameters
SI 32768
SF 100.6077400 MHz
WDW EM
SSB 0
LB 1.00 Hz
GB 0
PC 1.40

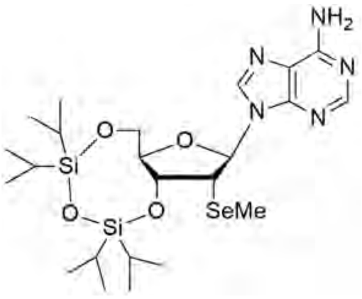

^{1}H-^{1}H COSY NMR spectrum of compound 15

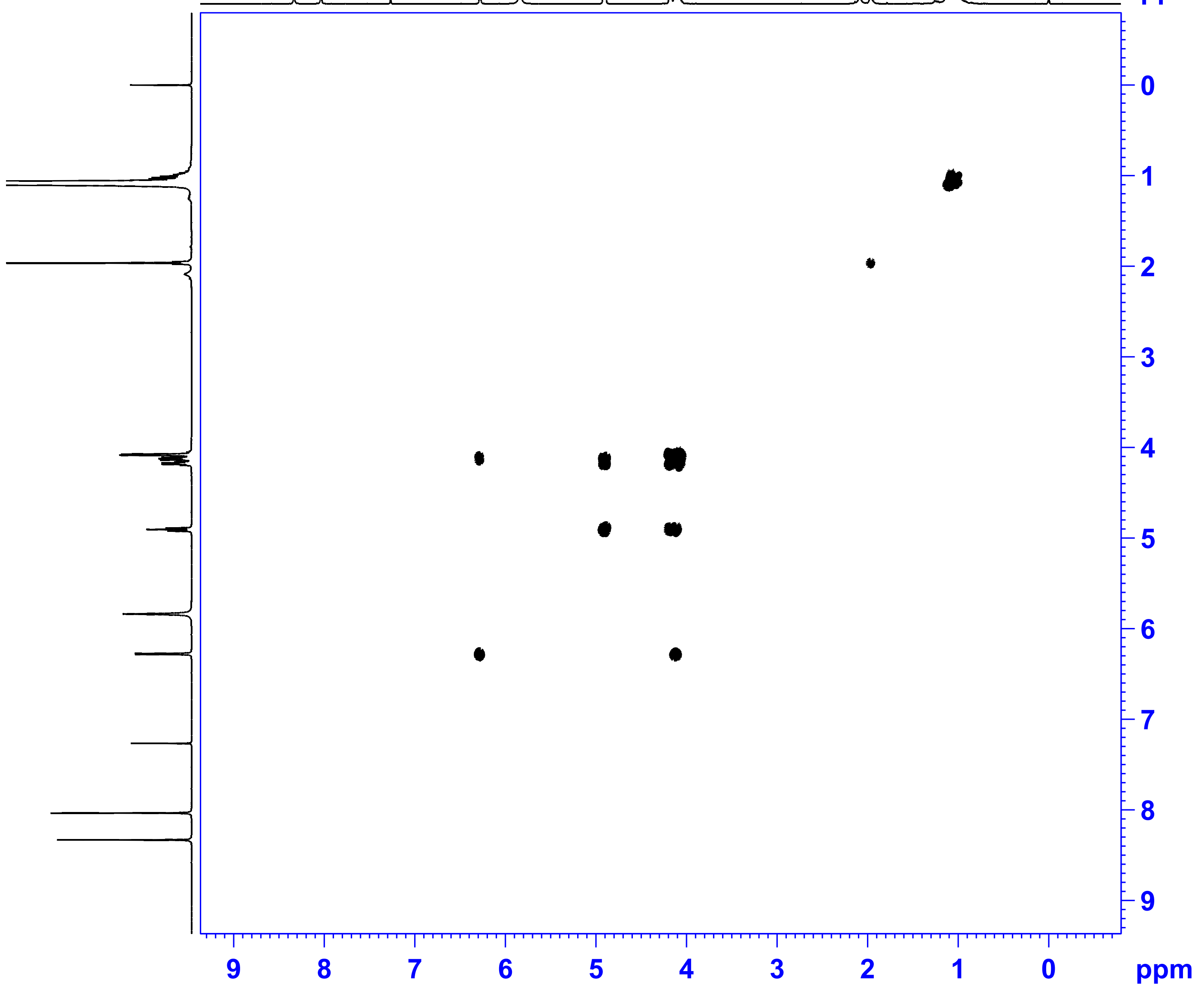

Current Data Parameters
NAME LH-II-69 Analytical
EXPNO 13
PROCNO 1

F2 - Acquisition Parameters
Date_ 20230608
Time 6.33 h
INSTRUM AVIII_400
PROBHD Z108618_0817 (
PULPROG cosygpmfqf
TD 2048
SOLVENT CDCl3
NS 3
DS 8
SWH 4065.041 Hz
FIDRES 3.969766 Hz
AQ 0.2519040 sec
RG 1620
DW 123.000 usec
DE 6.50 usec
TE 294.1 K
D0 0.00000300 sec
D1 1.94005799 sec
D13 0.00000400 sec
D16 0.00020000 sec
IN0 0.00024600 sec
TDav 1
SFO1 399.9117159 MHz
NUC1 1H
P1 500.00 usec
PLW1 31.62299919 W
GPNAM[1] SINE.100
GPZ1 16.00 %
GPNAM[2] SINE.100
GPZ2 12.00 %
GPNAM[3] SINE.100
GPZ3 40.00 %
P16 1000.00 usec

F1 - Acquisition parameters
TD 256
SFO1 399.9117 MHz
FIDRES 31.758131 Hz
SW 10.165 ppm
FnMODE QF

F2 - Processing parameters
SI 1024
SF 399.9100025 MHz
WDW SINE
SSB 0
LB 0 Hz
GB 0
PC 1.40

F1 - Processing parameters
SI 1024
MC2 QF
SF 399.9100025 MHz
WDW SINE
SSB 0
LB 0 Hz
GB 0

Expanded region of ^{1}H-^{1}H COSY NMR spectrum of compound 15

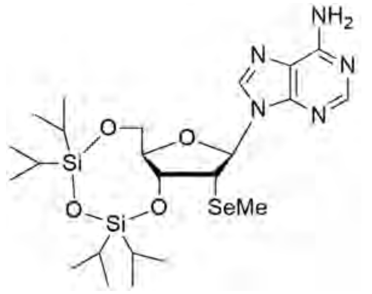

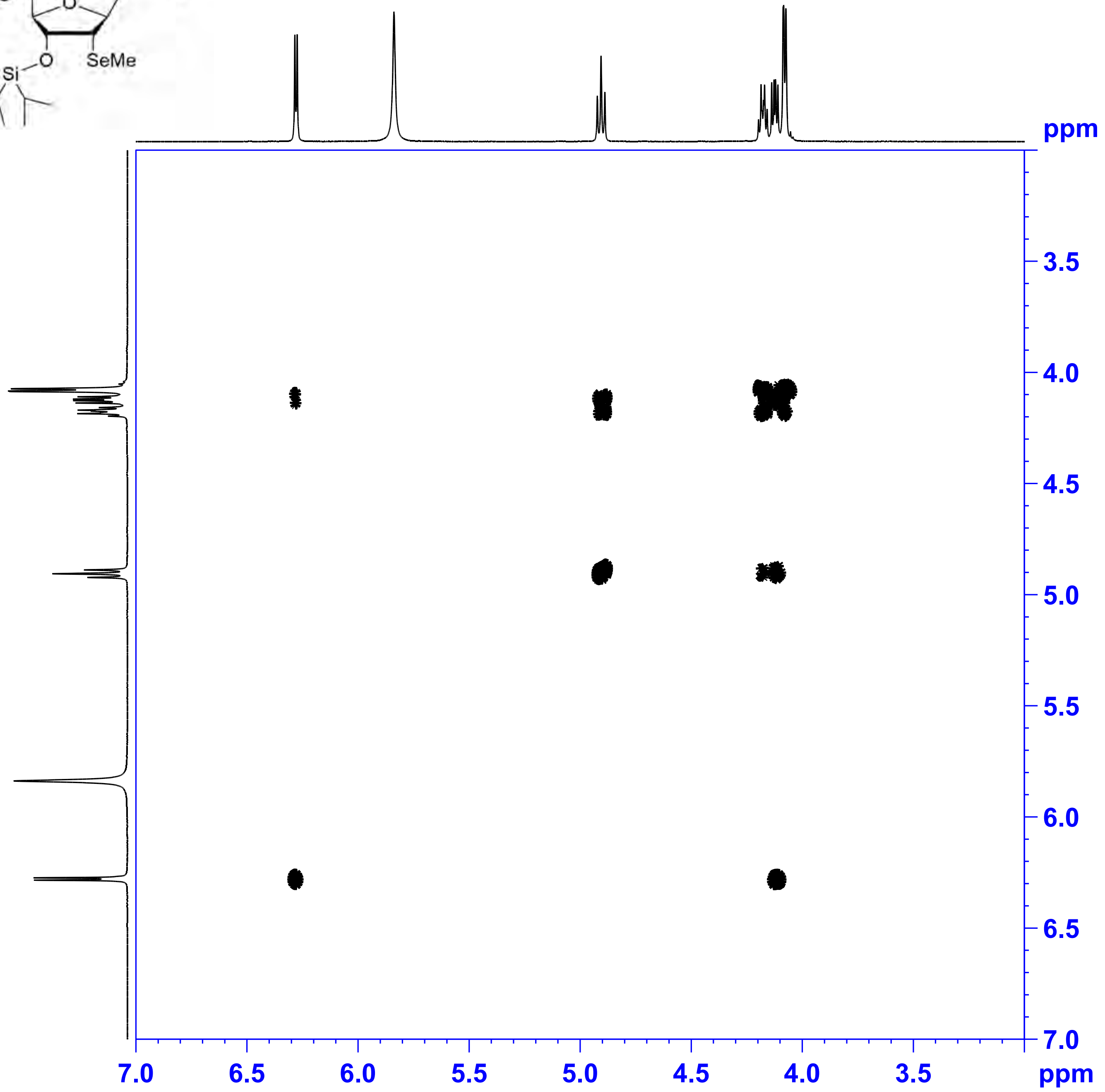

BRUKER

Current Data Parameters
NAME LH-II-69 Analytical
EXPNO 13
PROCNO 1

F2 - Acquisition Parameters
Date_ 20230608
Time 6.33 h
INSTRUM AVIII_400
PROBHD Z108618_0817 (
PULPROG cosygpmfqf
TD 2048
SOLVENT CDCl3
NS 3
DS 8
SWH 4065.041 Hz
FIDRES 3.969766 Hz
AQ 0.2519040 sec
RG 1620
DW 123.000 usec
DE 6.50 usec
TE 294.1 K
D0 0.00000300 sec
D1 1.94005799 sec
D13 0.00000400 sec
D16 0.00020000 sec
IN0 0.00024600 sec
TDav 1
SFO1 399.9117159 MHz
NUC1 1H
P1 500.00 usec
PLW1 31.62299919 W
GPNAM[1] SINE.100
GPZ1 16.00 %
GPNAM[2] SINE.100
GPZ2 12.00 %
GPNAM[3] SINE.100
GPZ3 40.00 %
P16 1000.00 usec

F1 - Acquisition parameters
TD 256
SFO1 399.9117 MHz
FIDRES 31.758131 Hz
SW 10.165 ppm
FnMODE QF

F2 - Processing parameters
SI 1024
SF 399.9100025 MHz
WDW SINE
SSB 0
LB 0 Hz
GB 0
PC 1.40

F1 - Processing parameters
SI 1024
MC2 QF
SF 399.9100025 MHz
WDW SINE
SSB 0
LB 0 Hz
GB 0

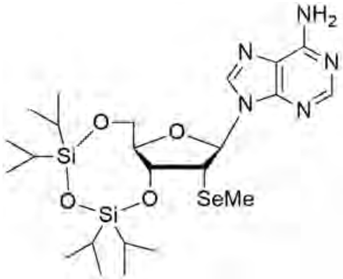

Expanded region of ¹H-¹H COSY NMR spectrum of compound 15

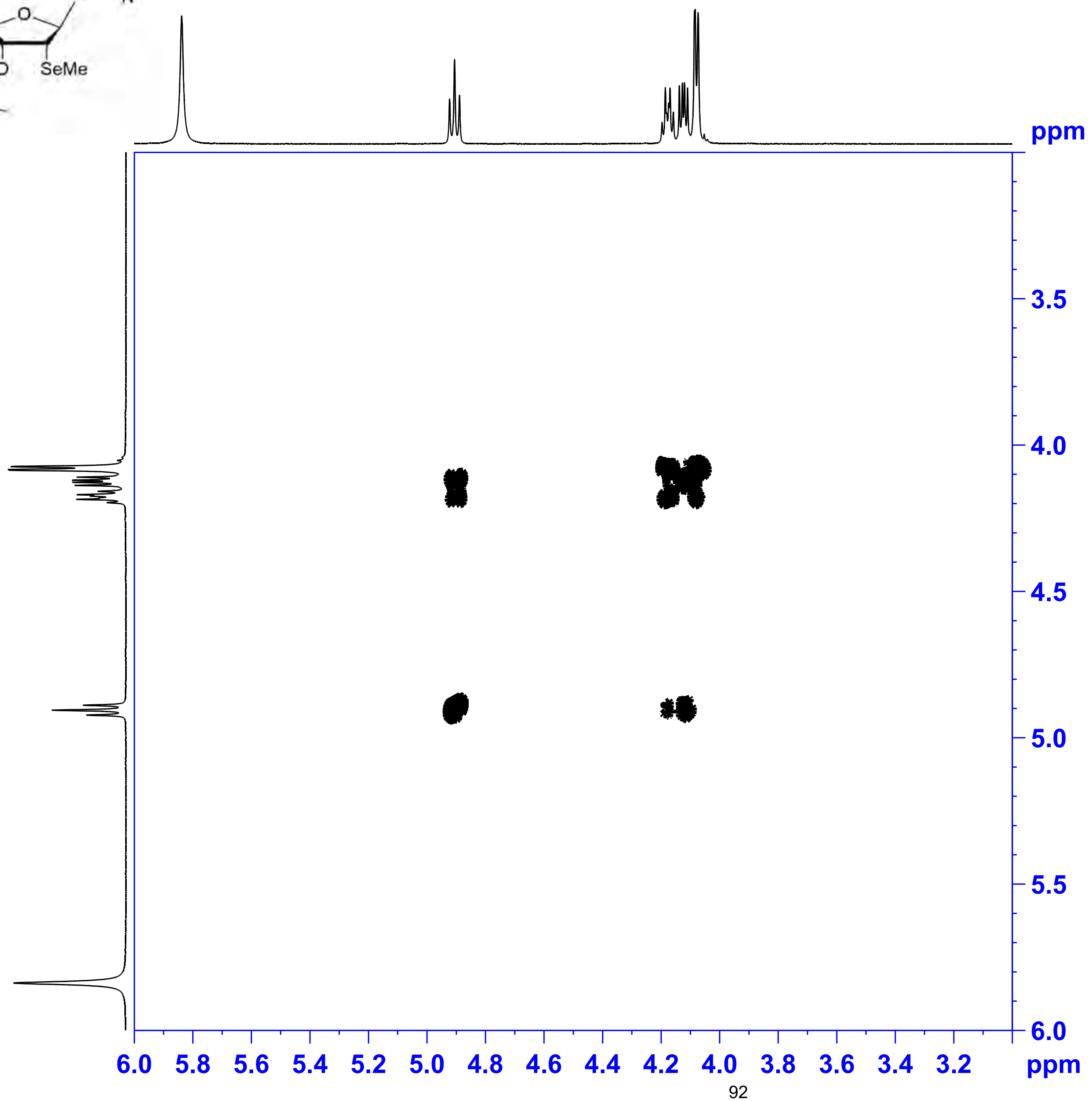

Current Data Parameters
NAME LH-II-69 Analytical
EXPNO 13
PROCNO 1

F2 - Acquisition Parameters

Date_	20230608	
Time	6.33	h
INSTRUM	AVIII_400	
PROBHD	Z108618_0817 (	
PULPROG	cosygpmfqf	
TD	2048	
SOLVENT	CDCl3	
NS	3	
DS	8	
SWH	4065.041	Hz
FIDRES	3.969766	Hz
AQ	0.2519040	sec
RG	1620	
DW	123.000	usec
DE	6.50	usec
TE	294.1	K
D0	0.00000300	sec
D1	1.94005799	sec
D13	0.00000400	sec
D16	0.00020000	sec
IN0	0.00024600	sec
TDav	1	
SFO1	399.9117159	MHz
NUC1	1H	
P1	500.00	usec
PLW1	31.62299919	W
GPNAM[1]	SINE.100	
GPZ1	16.00	%
GPNAM[2]	SINE.100	
GPZ2	12.00	%
GPNAM[3]	SINE.100	
GPZ3	40.00	%
P16	1000.00	usec

F1 - Acquisition parameters

TD	256	
SFO1	399.9117	MHz
FIDRES	31.758131	Hz
SW	10.165	ppm
FnMODE	QF	

F2 - Processing parameters

SI	1024	
SF	399.9100025	MHz
WDW	SINE	
SSB	0	
LB	0	Hz
GB	0	
PC	1.40	

F1 - Processing parameters

SI	1024	
MC2	QF	
SF	399.9100025	MHz
WDW	SINE	
SSB	0	
LB	0	Hz
GB	0	

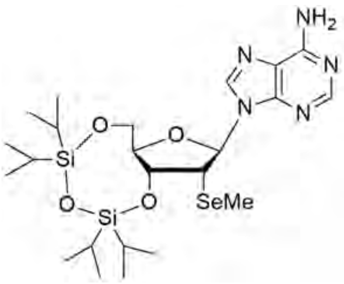

^{1}H-^{13}C HSQC NMR spectrum of compound 15

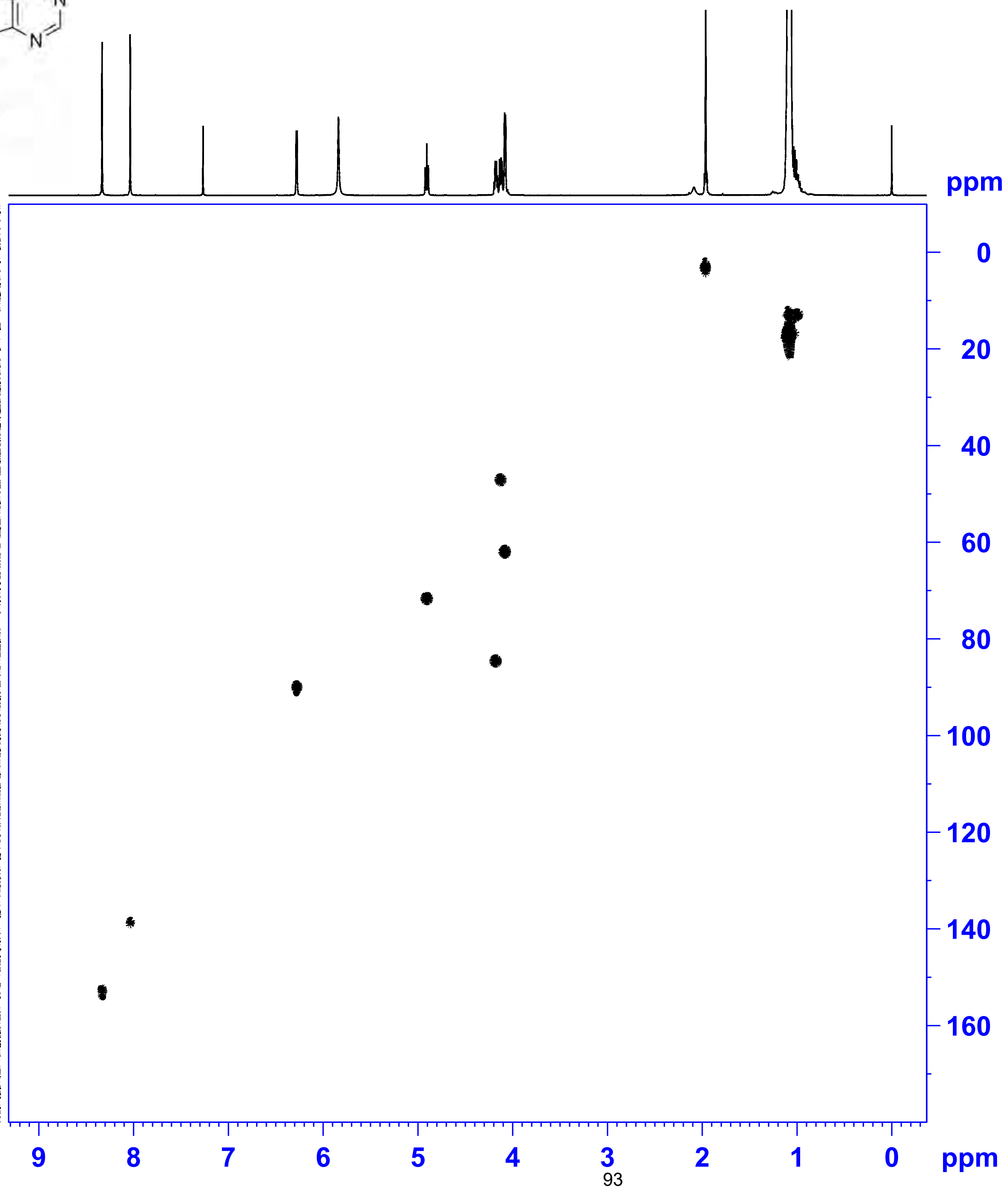

Current Data Parameters
NAME LH-II-69 OLD NMR
EXPNO 15
PROCNO 1

F2 - Acquisition Parameters
Date_ 20230615
Time 1.09 h
INSTRUM AVIII_400
PROBHD Z108618_0146 (
PULPROG hsqcedetgp
TD 1024
SOLVENT CDCl3
NS 4
DS 16
SWH 3875.969 Hz
FIDRES 7.570252 Hz
AQ 0.1320960 sec
RG 912
DW 129.000 usec
DE 6.50 usec
TE 300.0 K
CNST2 145.0000000
D0 0.00000300 sec
D1 1.45965397 sec
D4 0.00172414 sec
D11 0.03000000 sec
D16 0.00020000 sec
D21 0.00345000 sec
IN0 0.00002260 sec
TDav 1
ZGOPTNS
SFO1 400.1117973 MHz
NUC1 1H
P1 15.00 usec
P2 30.00 usec
PLW1 17.29199982 W
SFO2 100.6178008 MHz
NUC2 13C
CPDPRG[2 garp
P3 8.70 usec
P4 17.40 usec
PCPD2 56.50 usec
PLW2 96.68000031 W
PLW12 3.16230011 W
GPNAM[1] SINE.100
GPZ1 80.00 %
GPNAM[2] SINE.100
GPZ2 20.10 %
P16 1000.00 usec

F1 - Acquisition parameters
TD 256
SFO1 100.6178 MHz
FIDRES 172.842926 Hz
SW 219.881 ppm
FnMODE Echo-Antiecho

F2 - Processing parameters
SI 1024
SF 400.1100067 MHz
WDW QSINE
SSB 2
LB 0 Hz
GB 0
PC 1.40

F1 - Processing parameters
SI 1024
MC2 echo-antiecho
SF 100.6077400 MHz
WDW QSINE
SSB 2
LB 0 Hz
GB 0

^{1}H NMR spectrum of compound 16

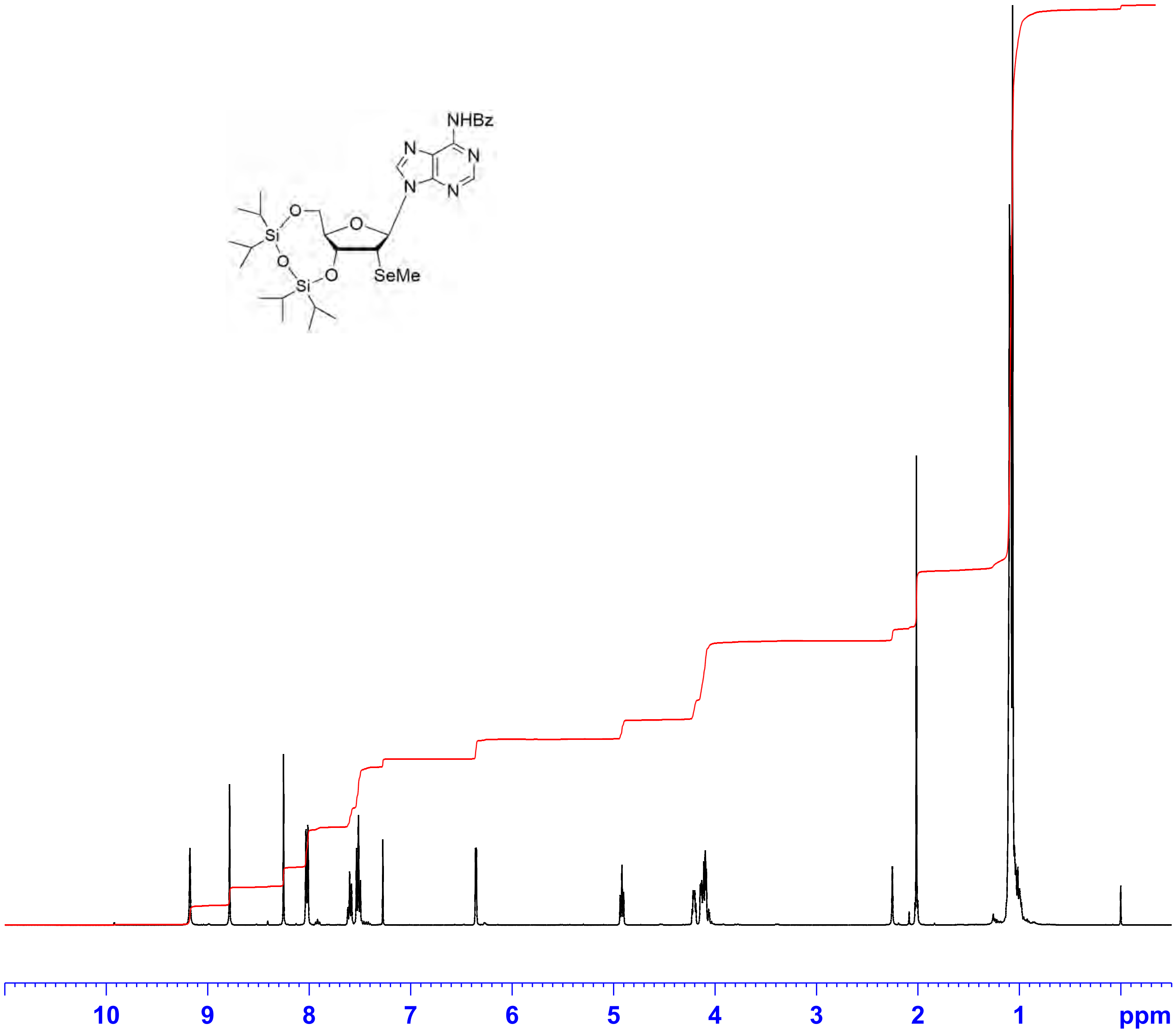


```
Current Data Parameters
NAME      LH-II-70 OLD NMR
EXPNO                   10
PROCNO                   1

F2 - Acquisition Parameters
Date_             20230610
Time                 16.06 h
INSTRUM           AVIII_400
PROBHD    Z108618_0146 (
PULPROG               zg30
TD                   65536
SOLVENT              CDCl3
NS                      16
DS                       2
SWH               8223.685 Hz
FIDRES            0.250967 Hz
AQ               3.9845889 sec
RG                    80.6
DW                  60.800 usec
DE                   17.42 usec
TE                   300.0 K
D1              1.00000000 sec
TD0                      1
SFO1           400.1124708 MHz
NUC1                    1H
P0                    5.00 usec
P1                   15.00 usec
PLW1           17.29199982 W

F2 - Processing parameters
SI                   32768
SF             400.1100037 MHz
WDW                     EM
SSB       0
LB                    0.30 Hz
GB        0
PC                    1.00
```

Expanded region of the ^{1}H NMR spectrum of compound 16

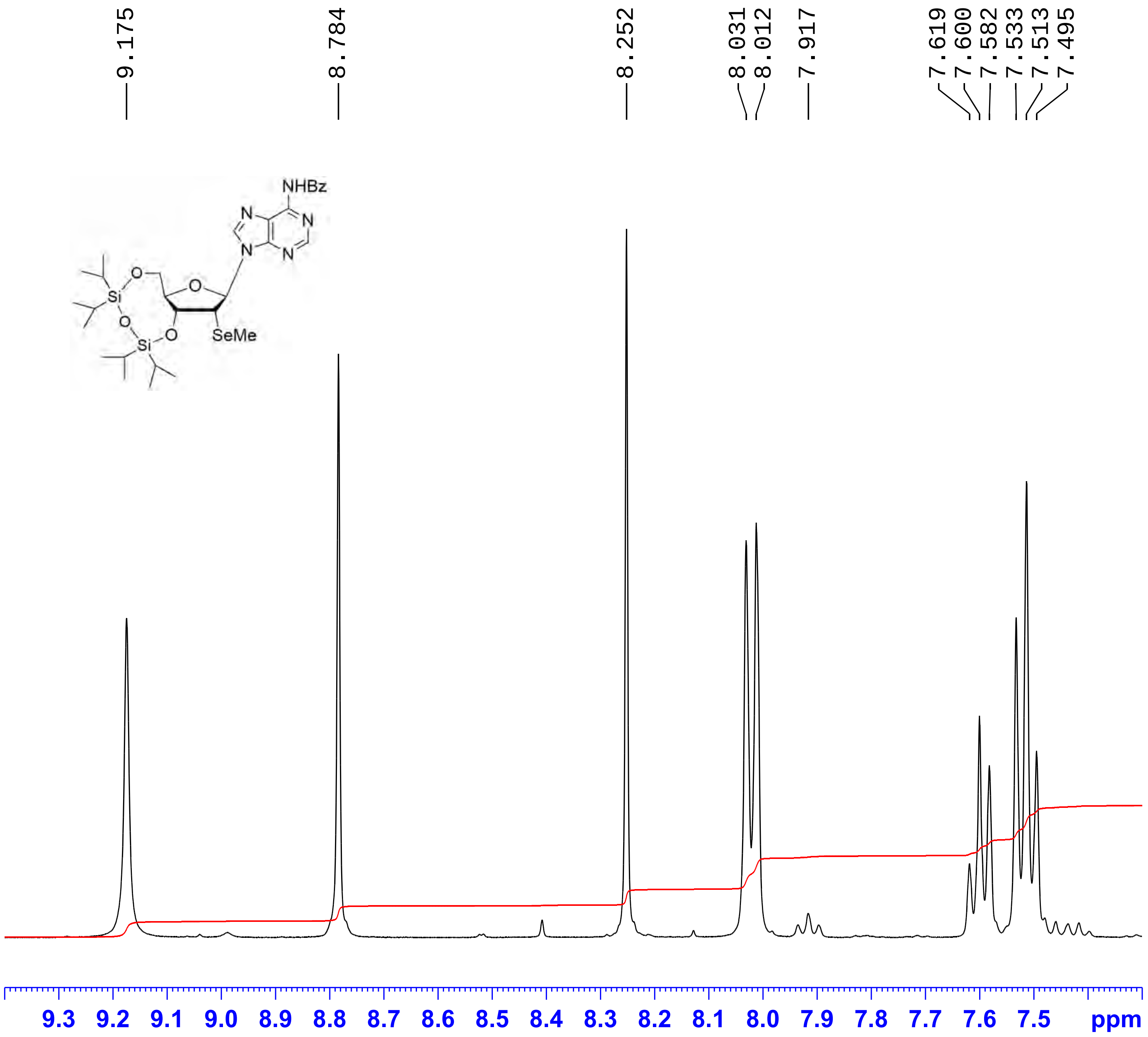


```
Current Data Parameters
NAME      LH-II-70 OLD NMR
EXPNO                   10
PROCNO                   1

F2 - Acquisition Parameters
Date_             20230610
Time                 16.06 h
INSTRUM           AVIII_400
PROBHD    Z108618_0146 (
PULPROG               zg30
TD                   65536
SOLVENT              CDCl3
NS                      16
DS                       2
SWH               8223.685 Hz
FIDRES            0.250967 Hz
AQ               3.9845889 sec
RG                    80.6
DW                  60.800 usec
DE                   17.42 usec
TE                   300.0 K
D1              1.00000000 sec
TD0                      1
SFO1           400.1124708 MHz
NUC1                    1H
P0                    5.00 usec
P1                   15.00 usec
PLW1           17.29199982 W

F2 - Processing parameters
SI                   32768
SF             400.1100037 MHz
WDW                     EM
SSB      0
LB                    0.30 Hz
GB       0
PC                    1.00
```

Expanded region of the ^{1}H NMR spectrum of compound 16

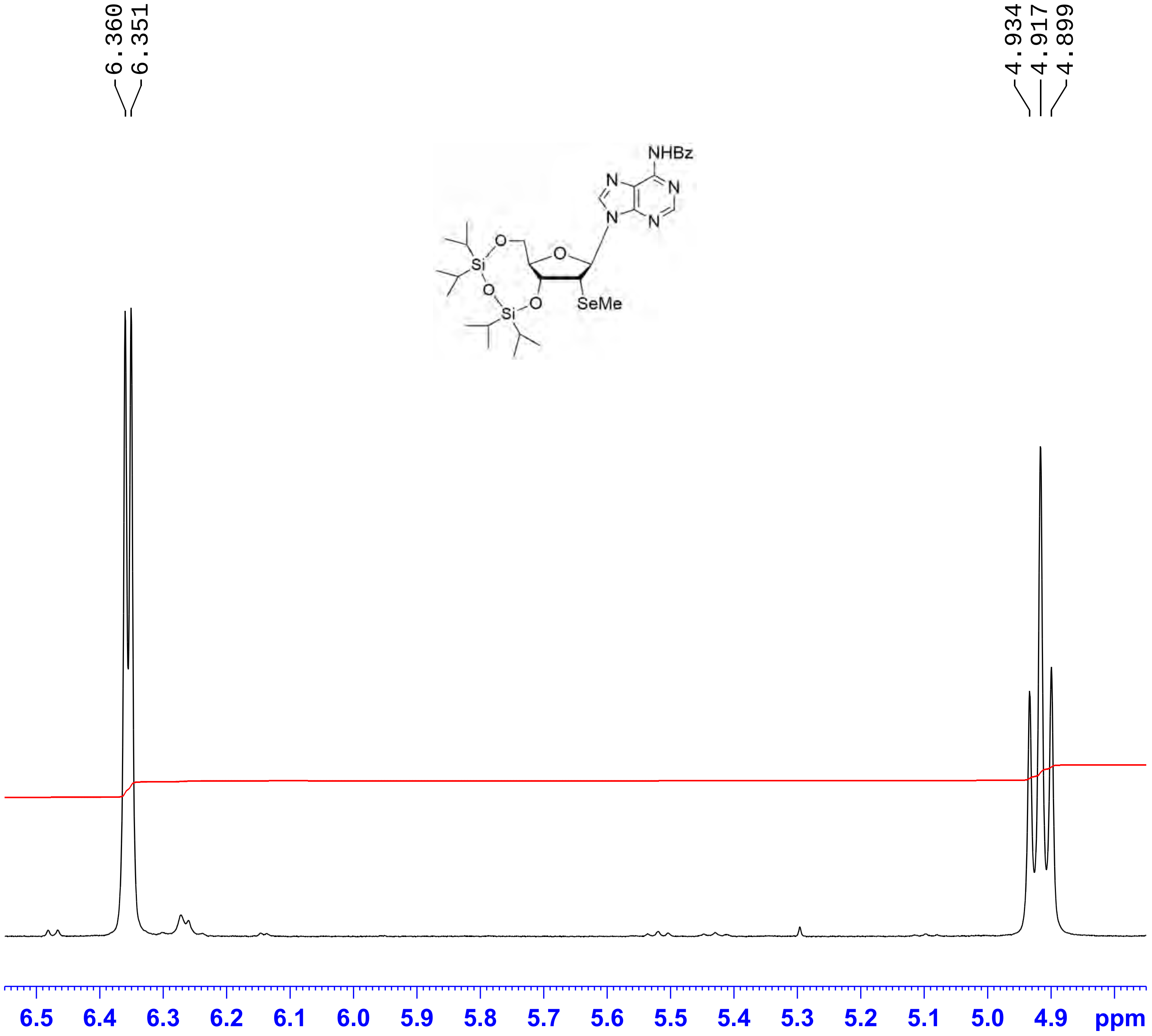


```
Current Data Parameters
NAME      LH-II-70 OLD NMR
EXPNO                   10
PROCNO                   1

F2 - Acquisition Parameters
Date_             20230610
Time                 16.06 h
INSTRUM           AVIII_400
PROBHD    Z108618_0146 (
PULPROG               zg30
TD                   65536
SOLVENT              CDCl3
NS                      16
DS                       2
SWH               8223.685 Hz
FIDRES            0.250967 Hz
AQ               3.9845889 sec
RG                    80.6
DW                  60.800 usec
DE                   17.42 usec
TE                   300.0 K
D1              1.00000000 sec
TD0                      1
SFO1           400.1124708 MHz
NUC1                    1H
P0                    5.00 usec
P1                   15.00 usec
PLW1           17.29199982 W

F2 - Processing parameters
SI                   32768
SF             400.1100037 MHz
WDW                     EM
SSB      0
LB                    0.30 Hz
GB       0
PC                    1.00
```

Expanded region of the ^{1}H NMR spectrum of compound 16

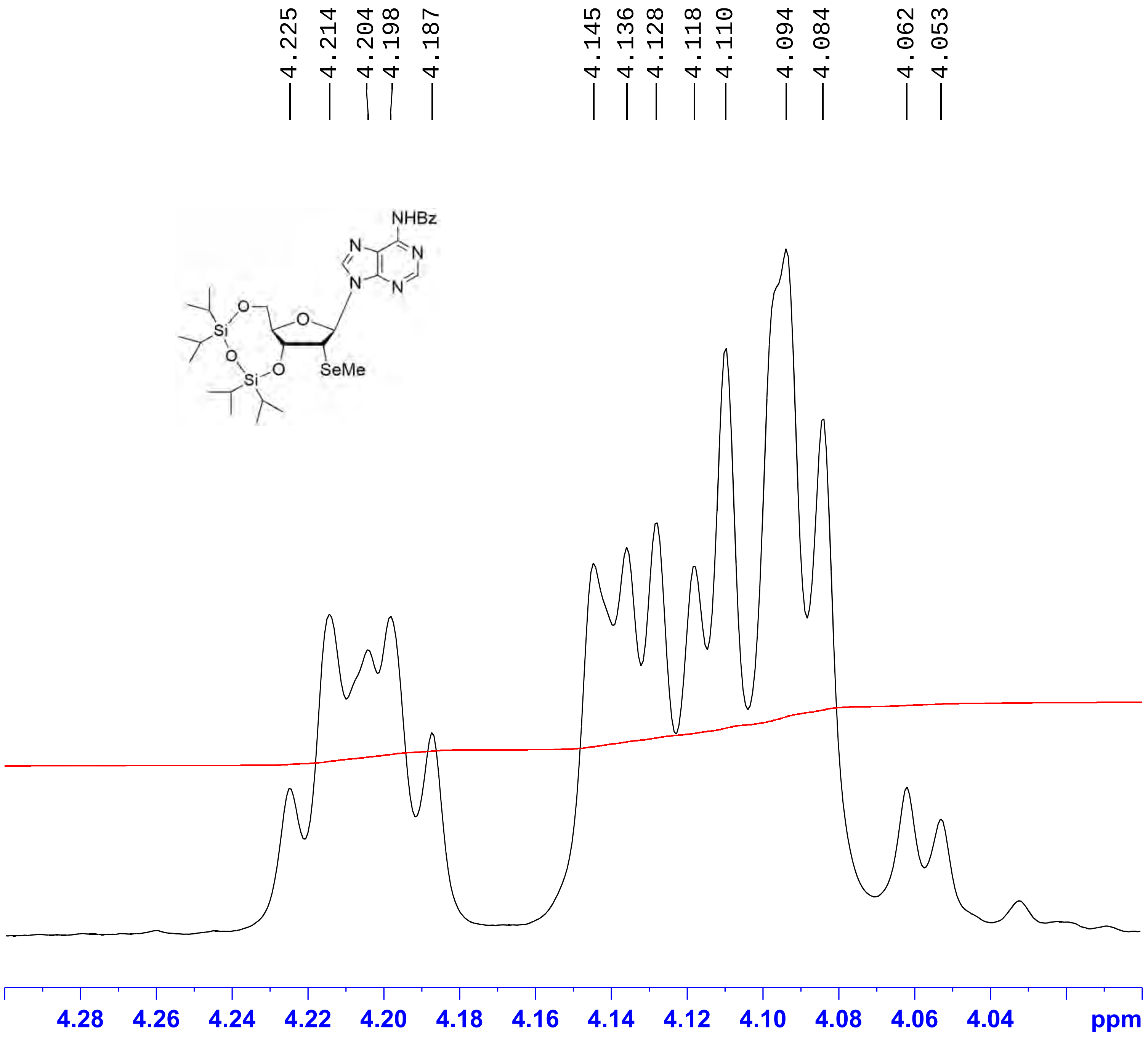


```
Current Data Parameters
NAME       LH-II-70 OLD NMR
EXPNO                    10
PROCNO                    1

F2 - Acquisition Parameters
Date_              20230610
Time                  16.06 h
INSTRUM            AVIII_400
PROBHD    Z108618_0146 (
PULPROG                zg30
TD                    65536
SOLVENT               CDCl3
NS                       16
DS                        2
SWH                8223.685 Hz
FIDRES             0.250967 Hz
AQ                3.9845889 sec
RG                     80.6
DW                   60.800 usec
DE                    17.42 usec
TE                    300.0 K
D1               1.00000000 sec
TD0                       1
SFO1            400.1124708 MHz
NUC1                     1H
P0                     5.00 usec
P1                    15.00 usec
PLW1            17.29199982 W

F2 - Processing parameters
SI                    32768
SF              400.1100037 MHz
WDW                      EM
SSB      0
LB                     0.30 Hz
GB       0
PC                     1.00
```

Expanded region of the ^{1}H NMR spectrum of compound 16

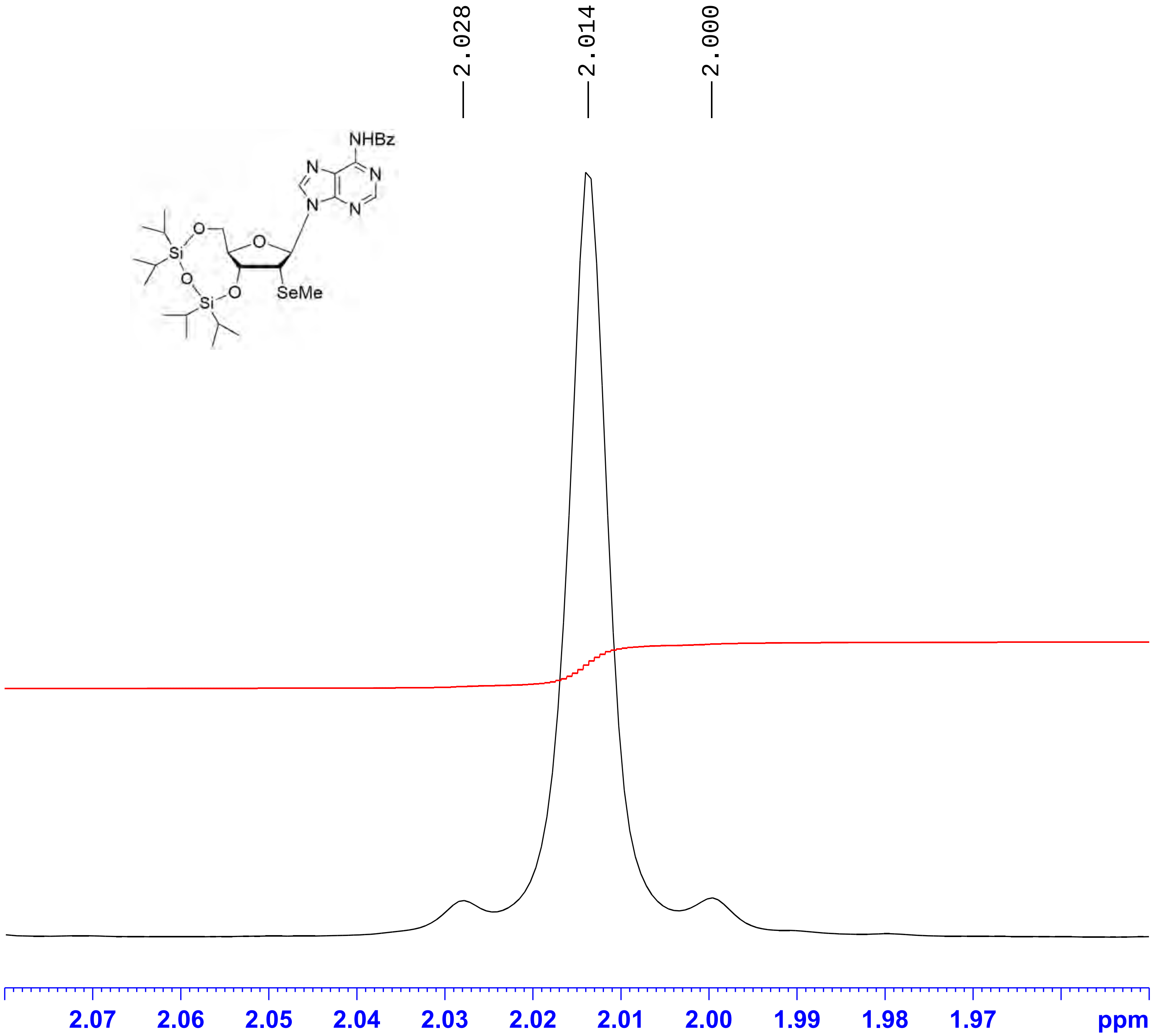

Current Data Parameters
NAME LH-II-70 OLD NMR
EXPNO 10
PROCNO 1

F2 - Acquisition Parameters
Date_ 20230610
Time 16.06 h
INSTRUM AVIII_400
PROBHD Z108618_0146 (
PULPROG zg30
TD 65536
SOLVENT CDCl3
NS 16
DS 2
SWH 8223.685 Hz
FIDRES 0.250967 Hz
AQ 3.9845889 sec
RG 80.6
DW 60.800 usec
DE 17.42 usec
TE 300.0 K
D1 1.00000000 sec
TD0 1
SFO1 400.1124708 MHz
NUC1 1H
P0 5.00 usec
P1 15.00 usec
PLW1 17.29199982 W

F2 - Processing parameters
SI 32768
SF 400.1100037 MHz
WDW EM
SSB 0
LB 0.30 Hz
GB 0
PC 1.00

Expanded region of the ^{1}H NMR spectrum of compound 16

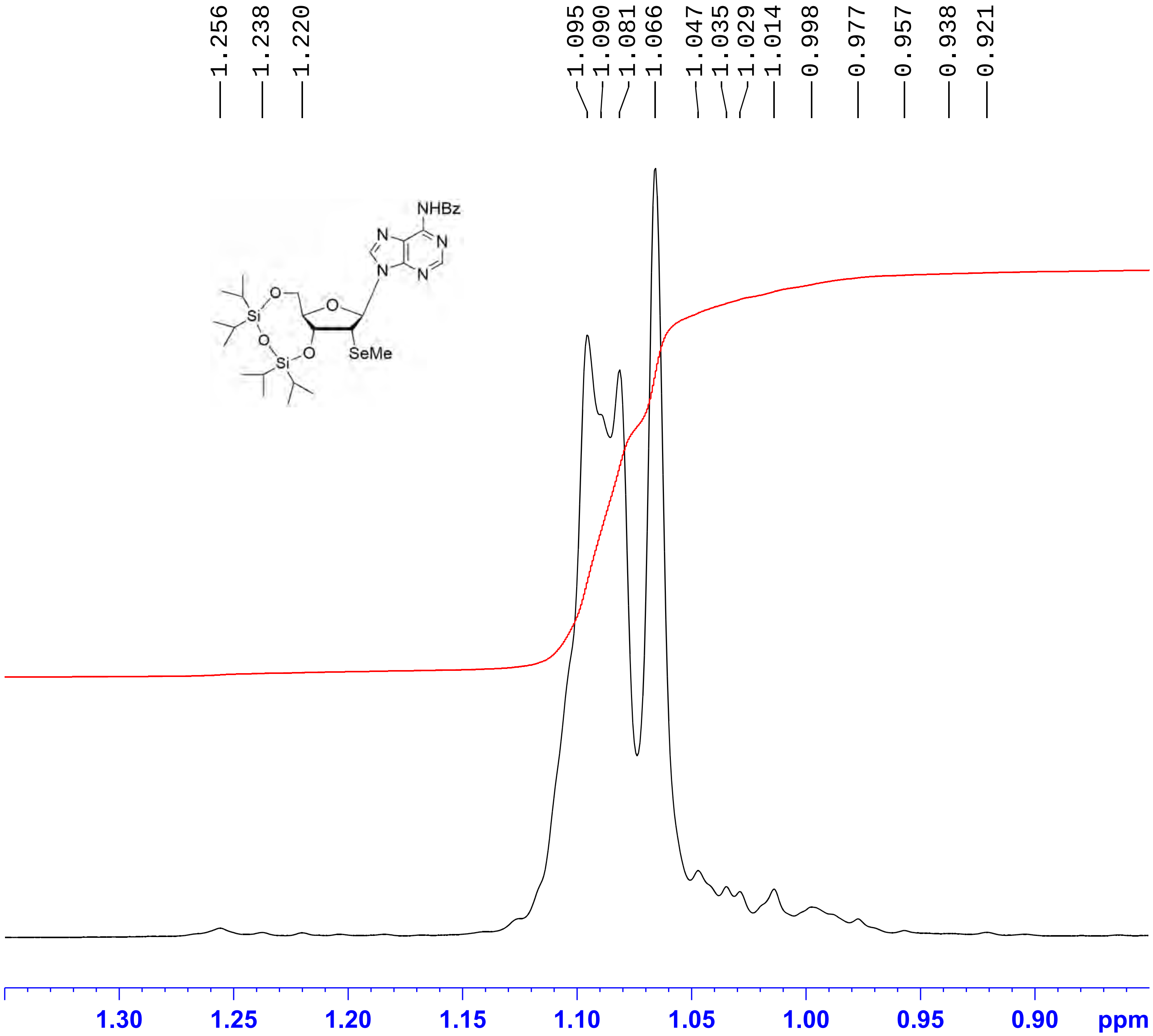


```
Current Data Parameters
NAME       LH-II-70 OLD NMR
EXPNO                    10
PROCNO                    1

F2 - Acquisition Parameters
Date_              20230610
Time                  16.06 h
INSTRUM           AVIII_400
PROBHD     Z108618_0146 (
PULPROG                zg30
TD                    65536
SOLVENT               CDCl3
NS                       16
DS                        2
SWH                8223.685 Hz
FIDRES             0.250967 Hz
AQ                3.9845889 sec
RG                     80.6
DW                   60.800 usec
DE                    17.42 usec
TE                    300.0 K
D1               1.00000000 sec
TD0                       1
SFO1            400.1124708 MHz
NUC1                     1H
P0                     5.00 usec
P1                    15.00 usec
PLW1            17.29199982 W

F2 - Processing parameters
SI                    32768
SF              400.1100037 MHz
WDW                      EM
SSB       0
LB                     0.30 Hz
GB        0
PC                     1.00
```

^{13}C NMR spectrum of compound 16

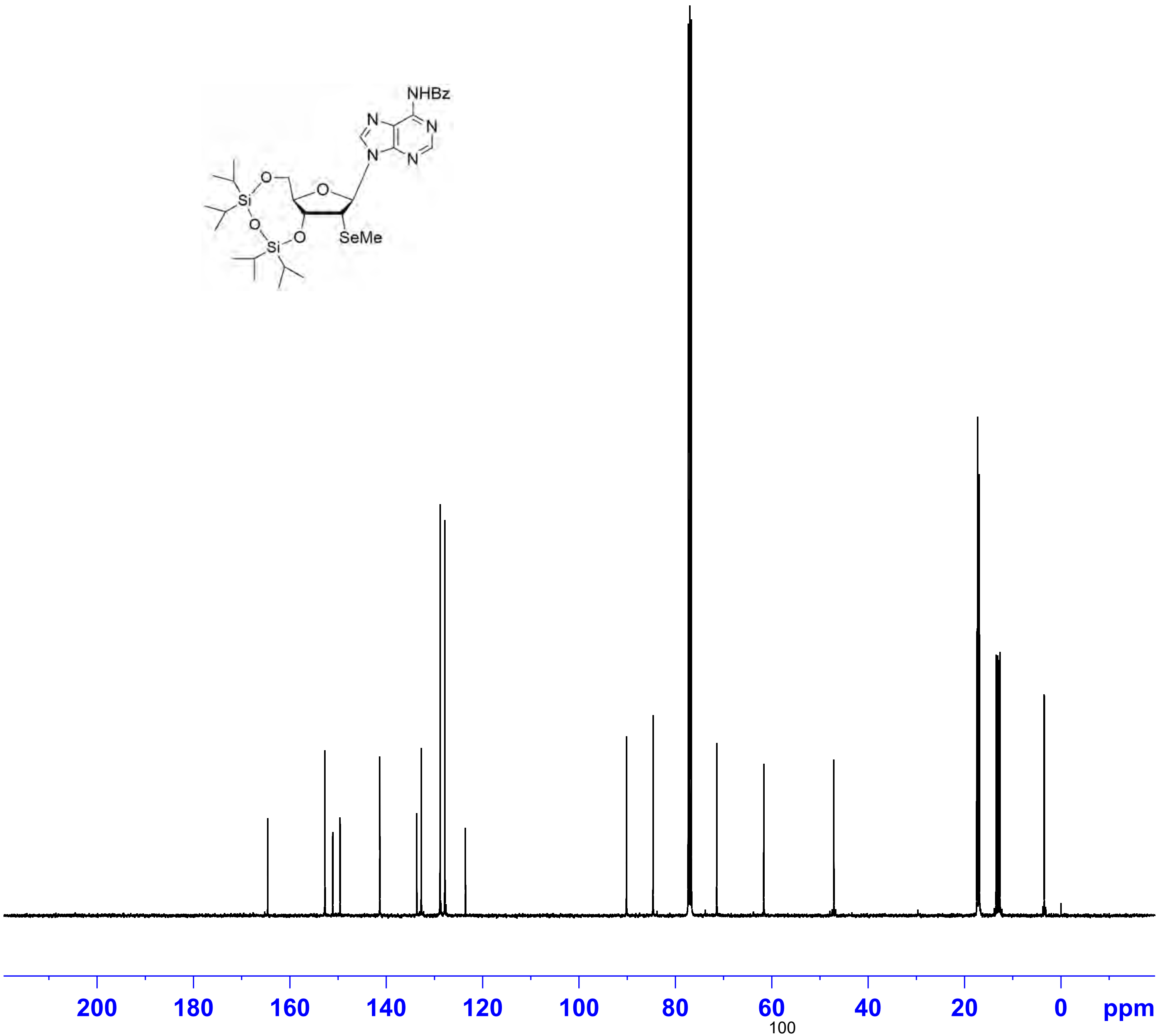

Current Data Parameters
NAME LH-II-70 OLD NMR
EXPNO 11
PROCNO 1

F2 - Acquisition Parameters
Date_ 20230610
Time 20.25 h
INSTRUM AVIII_400
PROBHD Z108618_0146 (
PULPROG zgpg30
TD 96150
SOLVENT CDCl3
NS 5000
DS 4
SWH 24038.461 Hz
FIDRES 0.500020 Hz
AQ 1.9999200 sec
RG 2050
DW 20.800 usec
DE 6.50 usec
TE 300.0 K
D1 1.00000000 sec
D11 0.03000000 sec
TD0 1
SFO1 100.6178003 MHz
NUC1 13C
P0 2.90 usec
P1 8.70 usec
PLW1 96.68000031 W
SFO2 400.1116004 MHz
NUC2 1H
CPDPRG[2 waltz64
PCPD2 90.00 usec
PLW2 17.29199982 W
PLW12 0.48032999 W
PLW13 0.24160001 W

F2 - Processing parameters
SI 131072
SF 100.6077459 MHz
WDW EM
SSB 0
LB 1.00 Hz
GB 0
PC 1.40

Expanded region of the ^{13}C NMR spectrum of compound 16

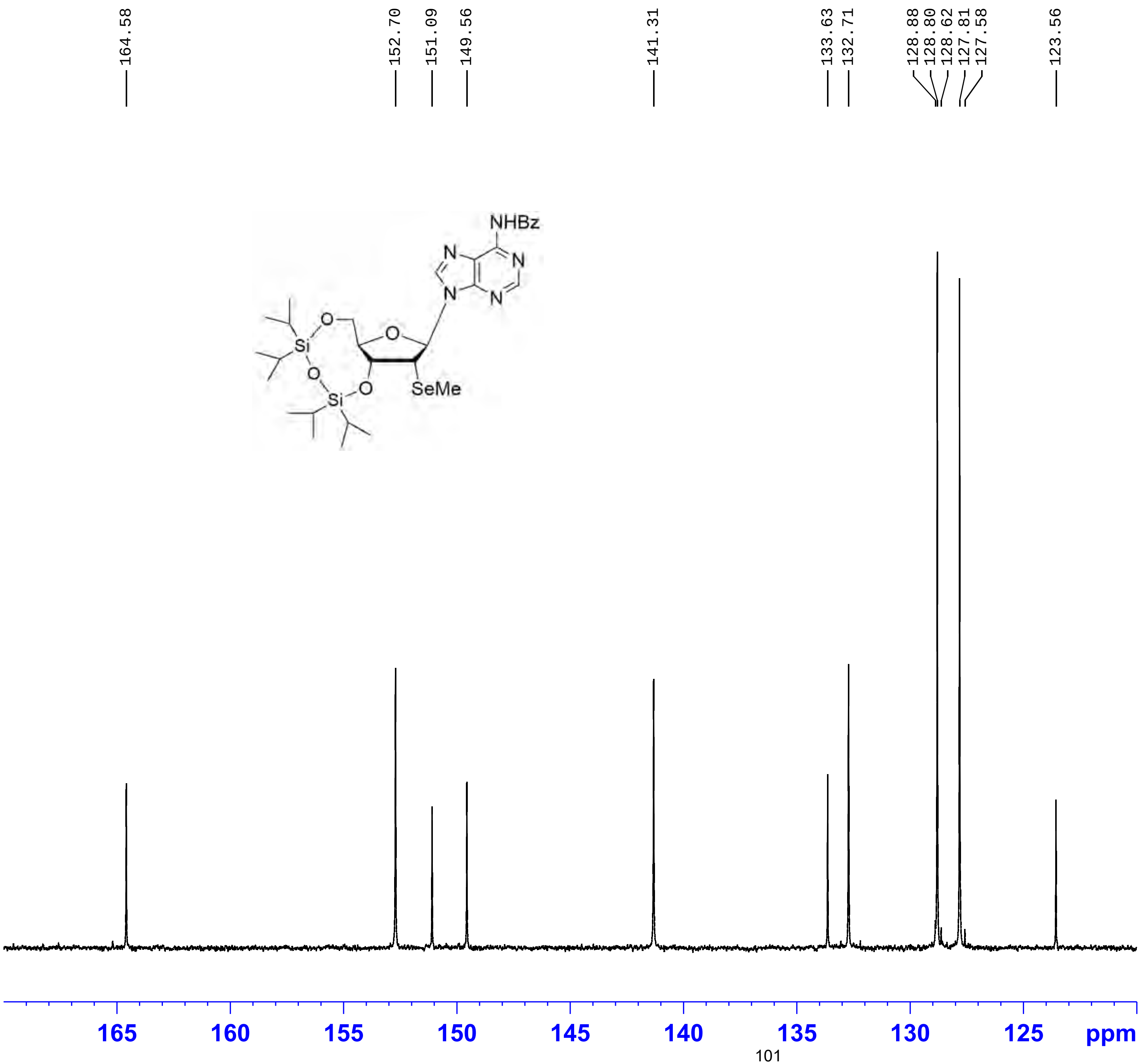


```
Current Data Parameters
NAME      LH-II-70 OLD NMR
EXPNO                   11
PROCNO                   1

F2 - Acquisition Parameters
Date_             20230610
Time                 20.25 h
INSTRUM           AVIII_400
PROBHD   Z108618_0146 (
PULPROG            zgpg30
TD                  96150
SOLVENT             CDCl3
NS                   5000
DS                      4
SWH             24038.461 Hz
FIDRES           0.500020 Hz
AQ              1.9999200 sec
RG                   2050
DW                 20.800 usec
DE                   6.50 usec
TE                  300.0 K
D1            1.00000000 sec
D11           0.03000000 sec
TD0                     1
SFO1          100.6178003 MHz
NUC1                  13C
P0                   2.90 usec
P1                   8.70 usec
PLW1          96.68000031 W
SFO2          400.1116004 MHz
NUC2                   1H
CPDPRG[2           waltz64
PCPD2               90.00 usec
PLW2          17.29199982 W
PLW12          0.48032999 W
PLW13          0.24160001 W

F2 - Processing parameters
SI                 131072
SF            100.6077459 MHz
WDW                    EM
SSB        0
LB                   1.00 Hz
GB         0
PC                   1.40
```

Expanded region of the ^{13}C NMR spectrum of compound 16

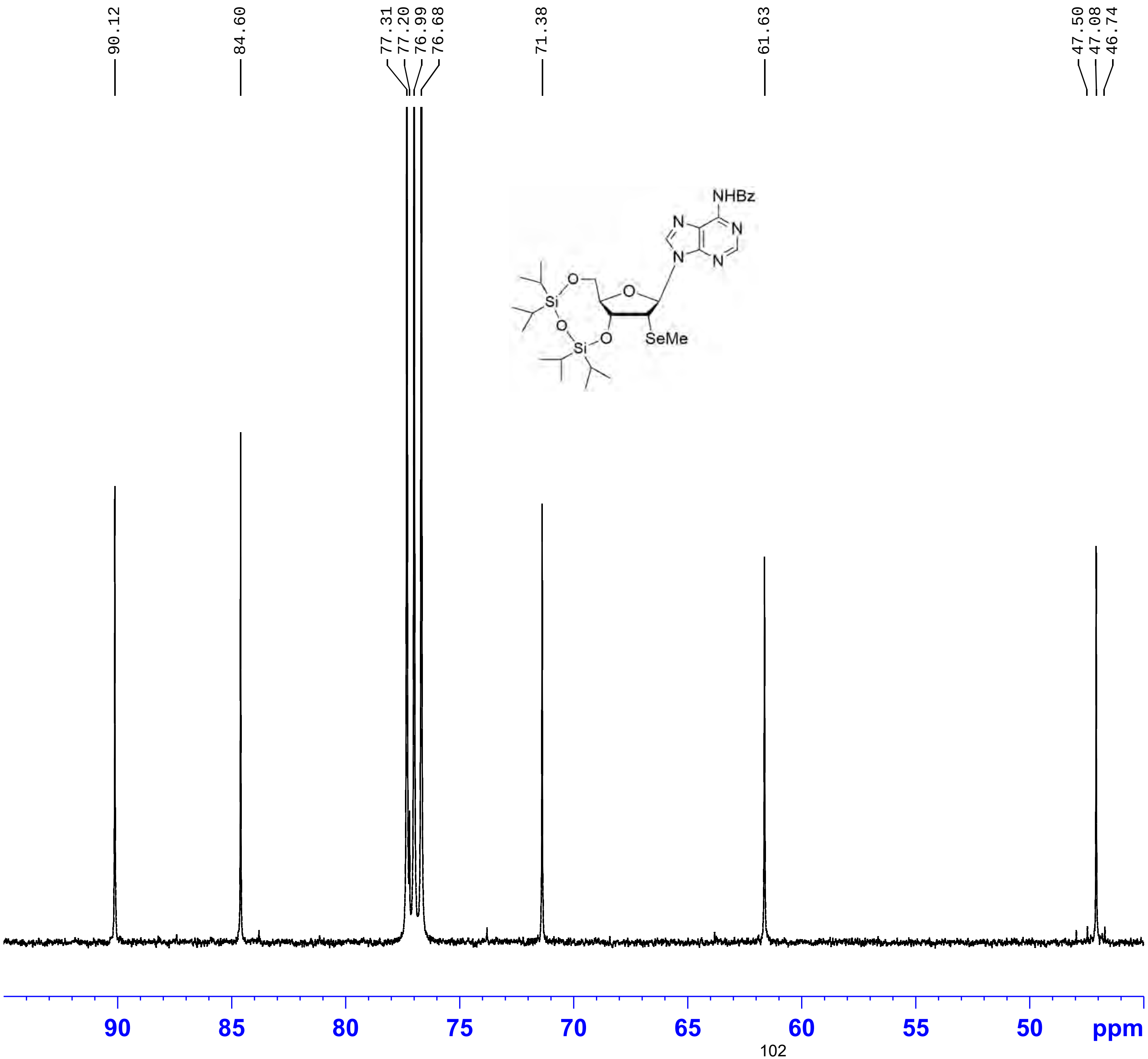


```
Current Data Parameters
NAME       LH-II-70 OLD NMR
EXPNO                    11
PROCNO                    1

F2 - Acquisition Parameters
Date_              20230610
Time                  20.25 h
INSTRUM           AVIII_400
PROBHD     Z108618_0146 (
PULPROG             zgpg30
TD                    96150
SOLVENT               CDCl3
NS                     5000
DS                        4
SWH               24038.461 Hz
FIDRES             0.500020 Hz
AQ                1.9999200 sec
RG                     2050
DW                   20.800 usec
DE                     6.50 usec
TE                    300.0 K
D1               1.00000000 sec
D11              0.03000000 sec
TD0                       1
SFO1            100.6178003 MHz
NUC1                    13C
P0                     2.90 usec
P1                     8.70 usec
PLW1            96.68000031 W
SFO2            400.1116004 MHz
NUC2                     1H
CPDPRG[2            waltz64
PCPD2                 90.00 usec
PLW2            17.29199982 W
PLW12            0.48032999 W
PLW13            0.24160001 W

F2 - Processing parameters
SI                   131072
SF              100.6077459 MHz
WDW                      EM
SSB        0
LB                     1.00 Hz
GB         0
PC                     1.40
```

Expanded region of the ^{13}C NMR spectrum of compound 16

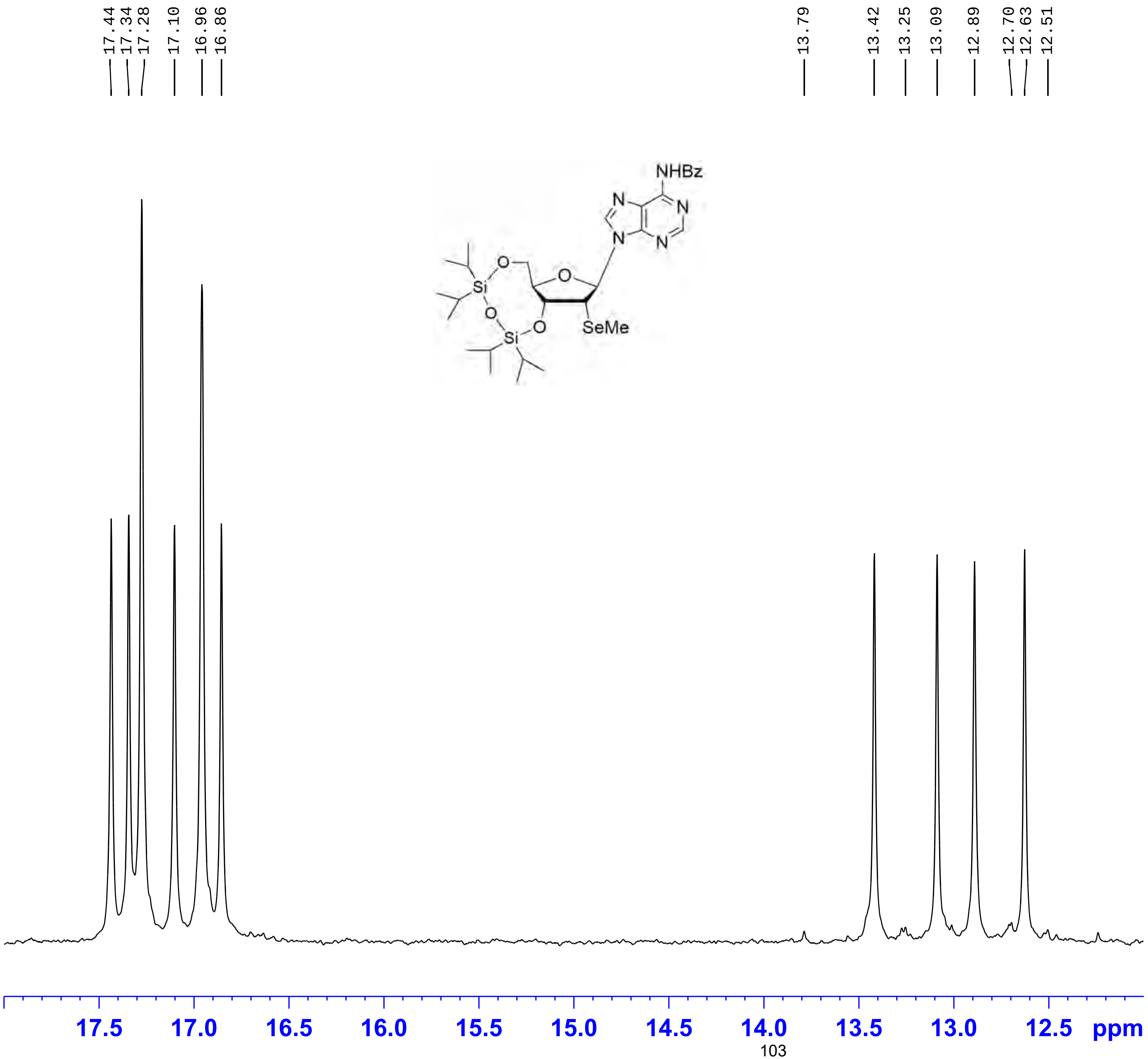


```
Current Data Parameters
NAME       LH-II-70 OLD NMR
EXPNO                    11
PROCNO                    1

F2 - Acquisition Parameters
Date_              20230610
Time                  20.25 h
INSTRUM           AVIII_400
PROBHD      Z108618_0146 (
PULPROG              zgpg30
TD                    96150
SOLVENT               CDCl3
NS                     5000
DS                        4
SWH               24038.461 Hz
FIDRES             0.500020 Hz
AQ                1.9999200 sec
RG                     2050
DW                   20.800 usec
DE                     6.50 usec
TE                    300.0 K
D1               1.00000000 sec
D11              0.03000000 sec
TD0                       1
SFO1            100.6178003 MHz
NUC1                    13C
P0                     2.90 usec
P1                     8.70 usec
PLW1            96.68000031 W
SFO2            400.1116004 MHz
NUC2                     1H
CPDPRG[2            waltz64
PCPD2                 90.00 usec
PLW2            17.29199982 W
PLW12            0.48032999 W
PLW13            0.24160001 W

F2 - Processing parameters
SI                   131072
SF              100.6077459 MHz
WDW                      EM
SSB          0
LB                     1.00 Hz
GB           0
PC                     1.40
```

Expanded region of the ^{13}C NMR spectrum of compound 16

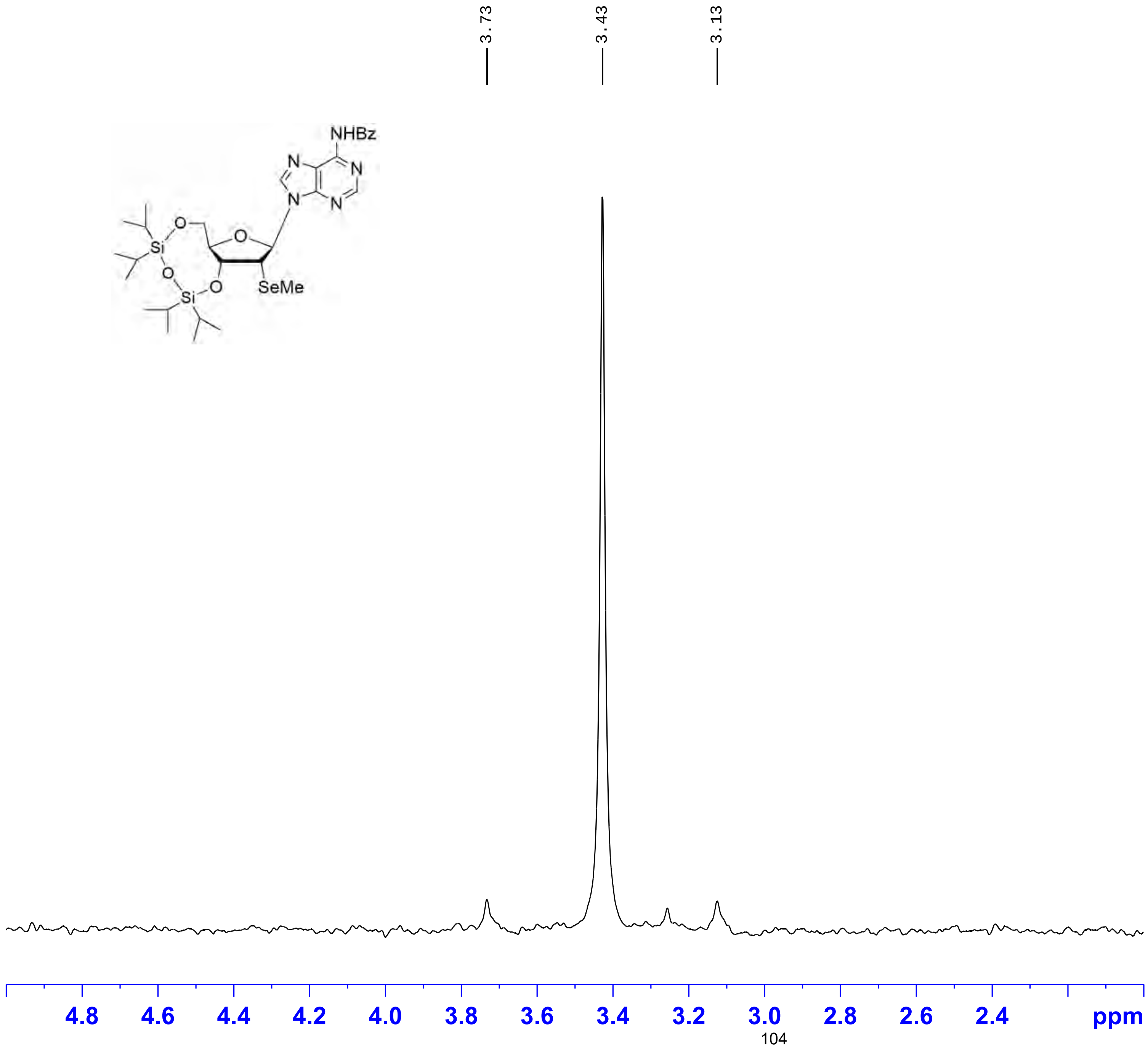


```
Current Data Parameters
NAME      LH-II-70 OLD NMR
EXPNO                   11
PROCNO                   1

F2 - Acquisition Parameters
Date_             20230610
Time                 20.25 h
INSTRUM          AVIII_400
PROBHD   Z108618_0146 (
PULPROG            zgpg30
TD                  96150
SOLVENT             CDCl3
NS                   5000
DS                      4
SWH             24038.461 Hz
FIDRES           0.500020 Hz
AQ              1.9999200 sec
RG                   2050
DW                 20.800 usec
DE                   6.50 usec
TE                  300.0 K
D1             1.00000000 sec
D11            0.03000000 sec
TD0                     1
SFO1          100.6178003 MHz
NUC1                  13C
P0                   2.90 usec
P1                   8.70 usec
PLW1          96.68000031 W
SFO2          400.1116004 MHz
NUC2                   1H
CPDPRG[2          waltz64
PCPD2               90.00 usec
PLW2          17.29199982 W
PLW12          0.48032999 W
PLW13          0.24160001 W

F2 - Processing parameters
SI                 131072
SF            100.6077459 MHz
WDW                    EM
SSB        0
LB                   1.00 Hz
GB         0
PC                   1.40
```

^{13}C DEPT-135 NMR spectrum of compound 16

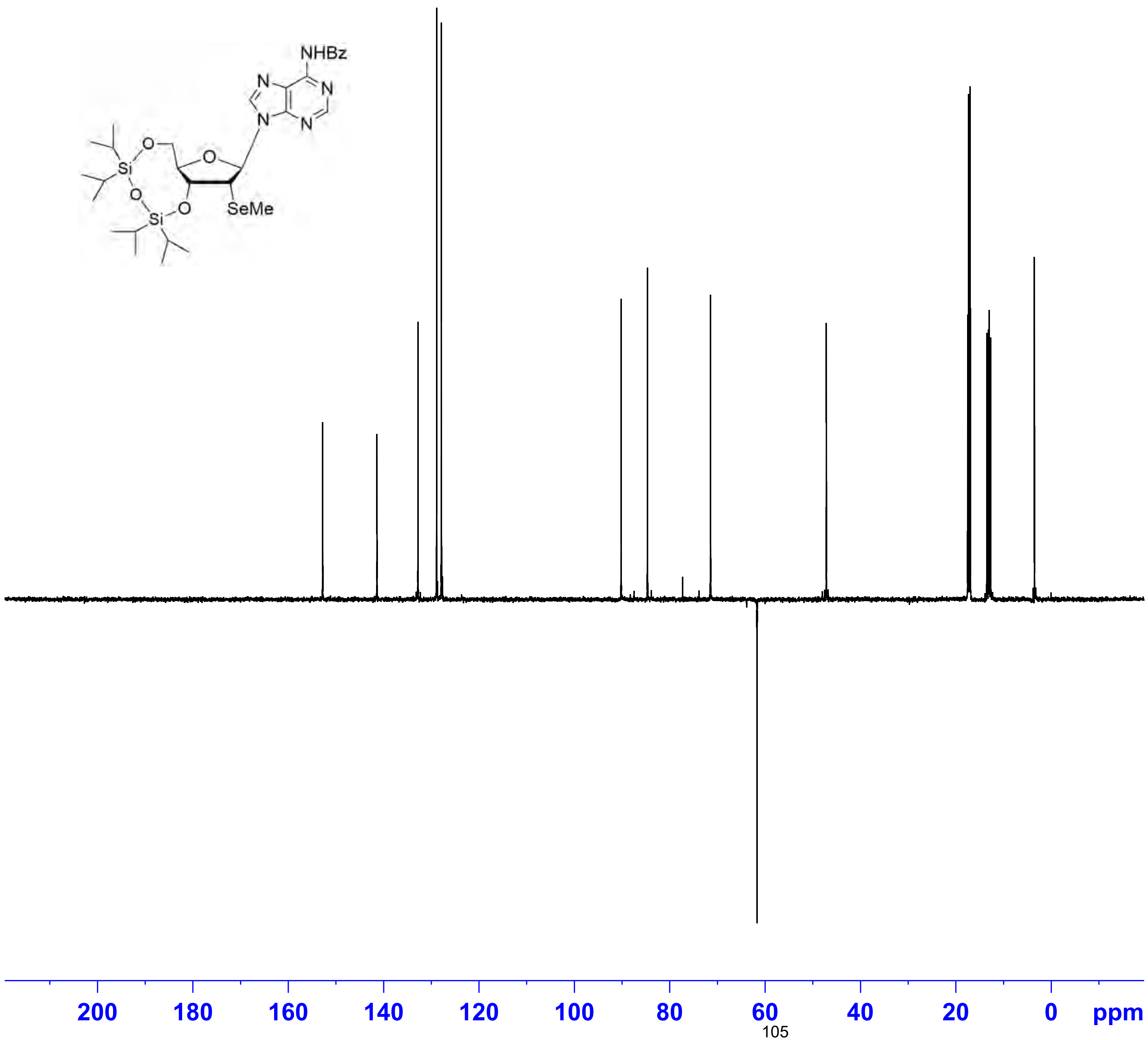

BRUKER

Current Data Parameters
NAME LH-II-70 OLD NMR
EXPNO 13
PROCNO 1

F2 - Acquisition Parameters
Date_ 20230611
Time 0.19 h
INSTRUM AVIII_400
PROBHD Z108618_0146 (
PULPROG dept135
TD 65536
SOLVENT CDCl3
NS 2000
DS 4
SWH 24038.461 Hz
FIDRES 0.733596 Hz
AQ 1.3631488 sec
RG 2050
DW 20.800 usec
DE 6.50 usec
TE 300.0 K
CNST2 145.0000000
D1 2.00000000 sec
D2 0.00344828 sec
D12 0.00002000 sec
TD0 1
SFO1 100.6178003 MHz
NUC1 13C
P1 8.70 usec
P2 17.40 usec
PLW1 96.68000031 W
SFO2 400.1116004 MHz
NUC2 1H
CPDPRG[2 waltz64
P3 15.00 usec
P4 30.00 usec
PCPD2 90.00 usec
PLW2 17.29199982 W
PLW12 0.48032999 W

F2 - Processing parameters
SI 32768
SF 100.6077400 MHz
WDW EM
SSB 0
LB 1.00 Hz
GB 0
PC 1.40

^{1}H-^{1}H COSY NMR spectrum of compound 16

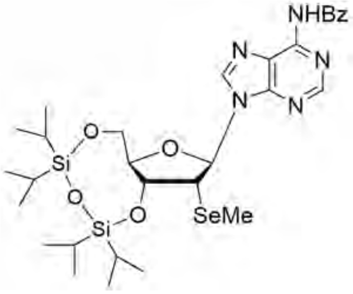

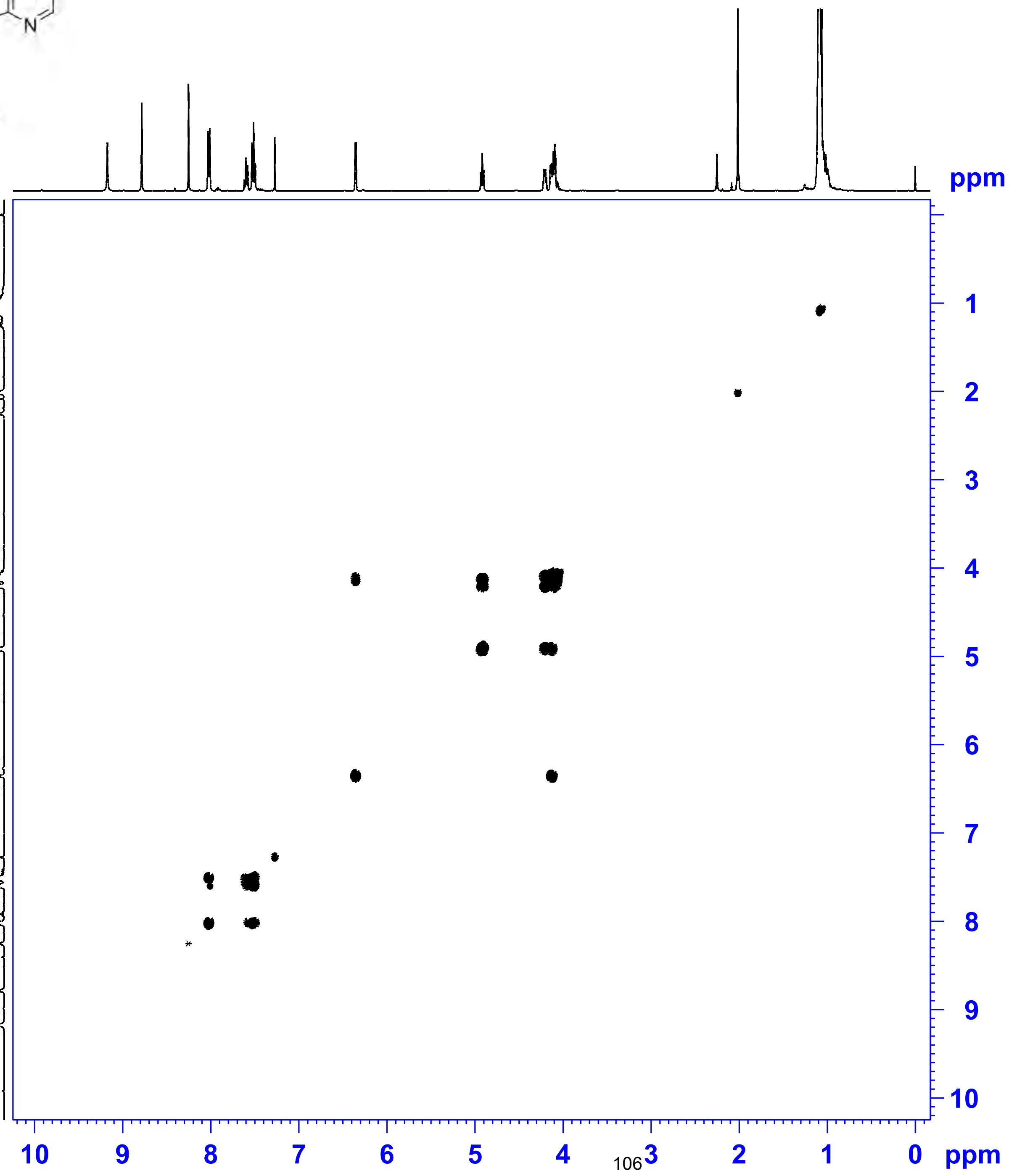

Current Data Parameters
NAME LH-II-70
EXPNO 13
PROCNO 1

F2 - Acquisition Parameters
Date_ 20230610
Time 2.13 h
INSTRUM AVIII_400
PROBHD Z108618_0817 (
PULPROG cosygpmfqf
TD 2048
SOLVENT CDCl3
NS 3
DS 8
SWH 4166.667 Hz
FIDRES 4.069010 Hz
AQ 0.2457600 sec
RG 1620
DW 120.000 usec
DE 6.50 usec
TE 294.5 K
D0 0.00000300 sec
D1 1.94620299 sec
D13 0.00000400 sec
D16 0.00020000 sec
IN0 0.00024000 sec
TDav 1
SFO1 399.9120187 MHz
NUC1 1H
P1 500.00 usec
PLW1 31.62299919 W
GPNAM[1] SINE.100
GPZ1 16.00 %
GPNAM[2] SINE.100
GPZ2 12.00 %
GPNAM[3] SINE.100
GPZ3 40.00 %
P16 1000.00 usec

F1 - Acquisition parameters
TD 256
SFO1 399.912 MHz
FIDRES 32.552082 Hz
SW 10.419 ppm
FnMODE QF

F2 - Processing parameters
SI 1024
SF 399.9100041 MHz
WDW SINE
SSB 0
LB 0 Hz
GB 0
PC 1.40

F1 - Processing parameters
SI 1024
MC2 QF
SF 399.9100041 MHz
WDW SINE
SSB 0
LB 0 Hz
GB 0

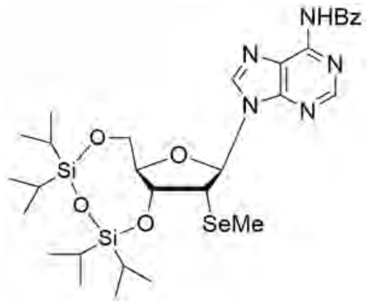

^{1}H-^{13}C HSQC NMR spectrum of compound 16

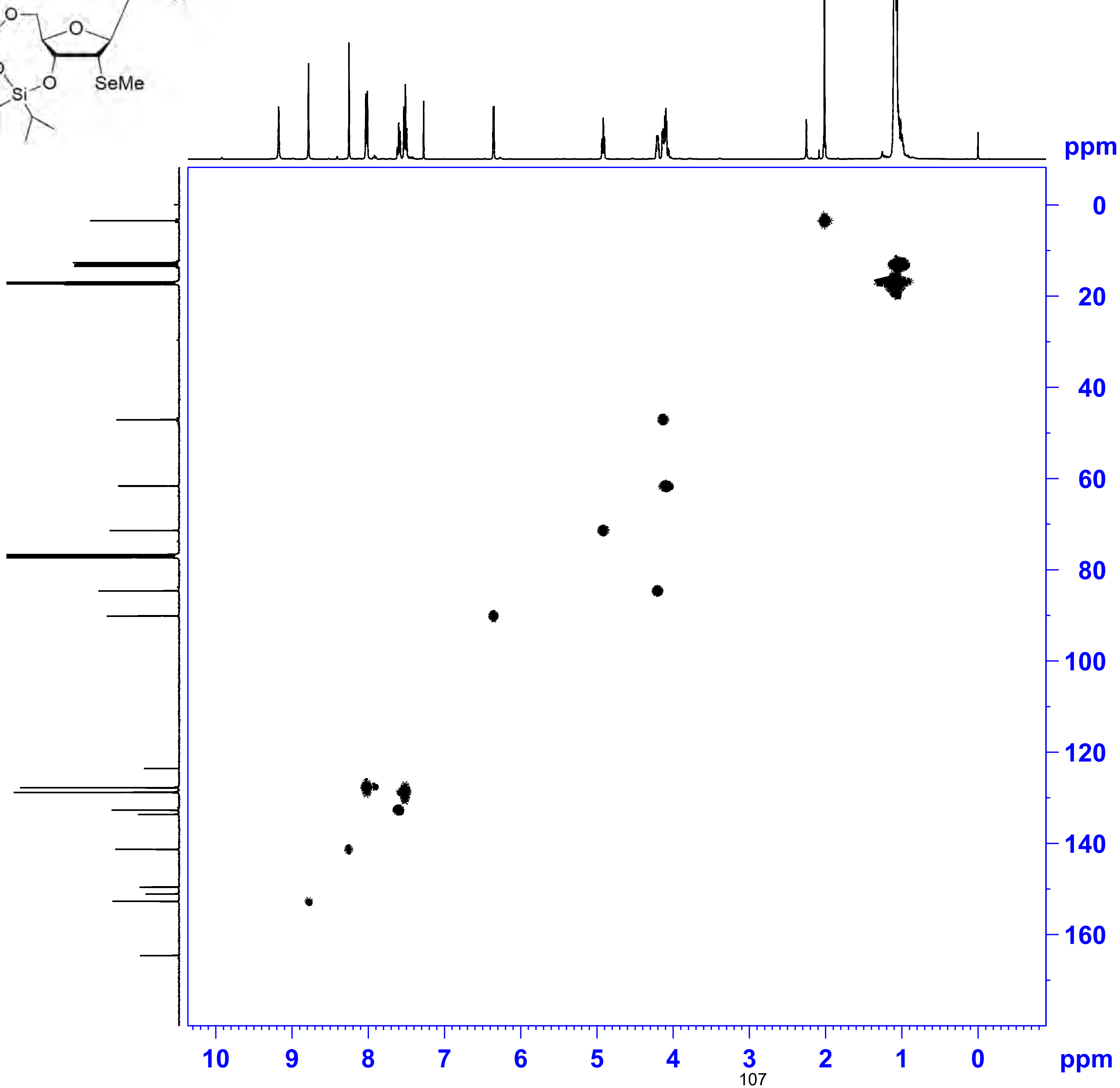


```
Current Data Parameters
NAME      LH-II-70 OLD NMR
EXPNO                   15
PROCNO                   1

F2 - Acquisition Parameters
Date_             20230611
Time                0.49 h
INSTRUM          AVIII_400
PROBHD     Z108618_0146 (
PULPROG         hsqcedetgp
TD                    1024
SOLVENT              CDCl3
NS                       4
DS                      16
SWH               4504.504 Hz
FIDRES            8.797860 Hz
AQ               0.1136640 sec
RG                    1620
DW                 111.000 usec
DE                    6.50 usec
TE                   300.0 K
CNST2          145.0000000
D0              0.00000300 sec
D1              1.45965397 sec
D4              0.00172414 sec
D11             0.03000000 sec
D16             0.00020000 sec
D21             0.00345000 sec
IN0             0.00003000 sec
TDav                     1
ZGOPTNS
SFO1           400.1118992 MHz
NUC1                    1H
P1                   15.00 usec
P2                   30.00 usec
PLW1           17.29199982 W
SFO2           100.6152434 MHz
NUC2                   13C
CPDPRG[2              garp
P3                    8.70 usec
P4                   17.40 usec
PCPD2                56.50 usec
PLW2           96.68000031 W
PLW12           3.16230011 W
GPNAM[1]        SMSQ10.100
GPZ1                 80.00 %
GPNAM[2]        SMSQ10.100
GPZ2                 20.10 %
P16                1000.00 usec

F1 - Acquisition parameters
TD                     256
SFO1              100.6152 MHz
FIDRES          130.208328 Hz
SW                 165.648 ppm
FnMODE        Echo-Antiecho

F2 - Processing parameters
SI                    1024
SF             400.1100037 MHz
WDW                  QSINE
SSB                      2
LB                  0 Hz
GB                  0
PC                    1.40

F1 - Processing parameters
SI                    1024
MC2           echo-antiecho
SF             100.6077400 MHz
WDW                  QSINE
SSB                      2
LB                  0 Hz
GB                  0
```

^{1}H NMR spectrum of compound 17

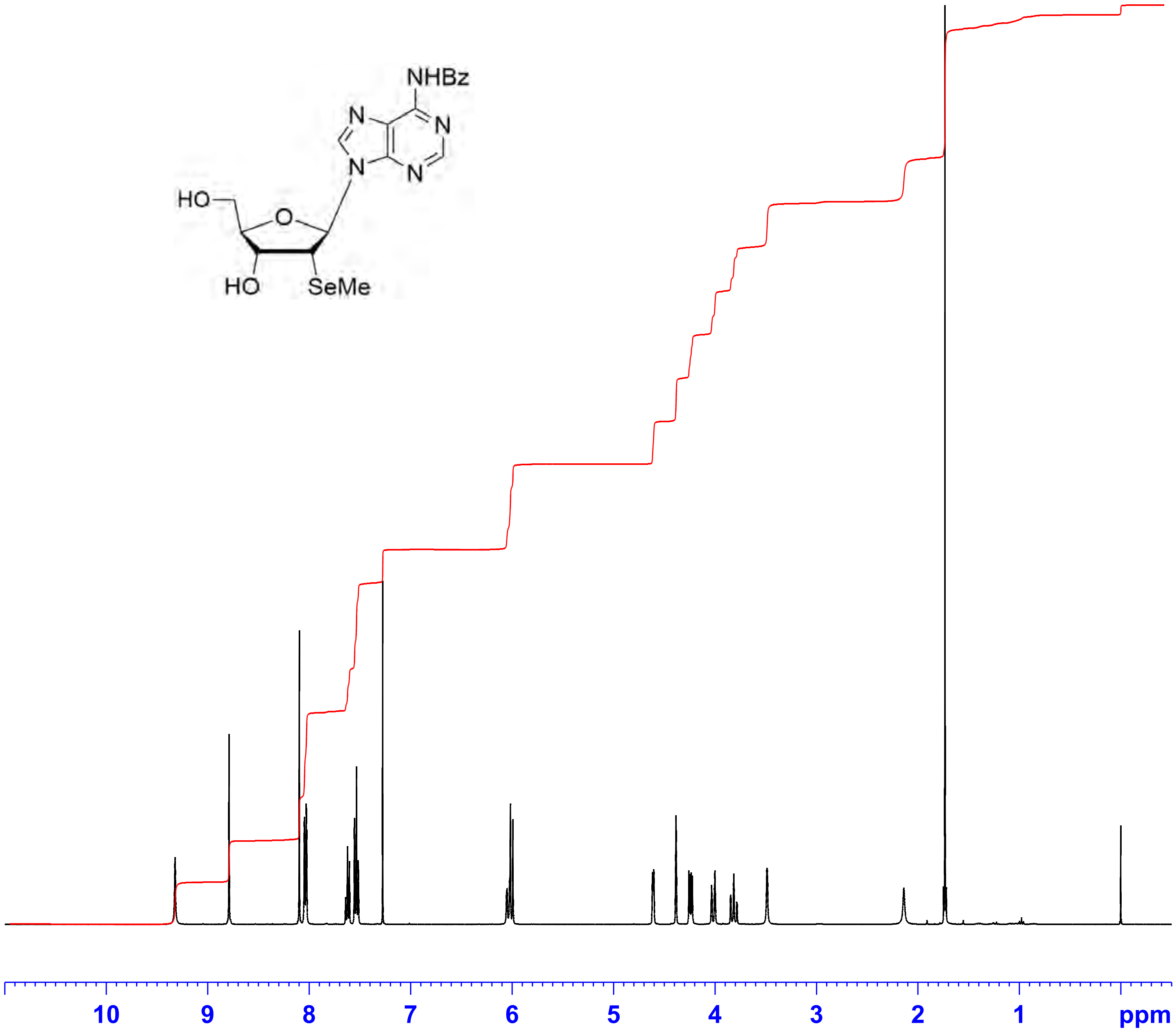

Current Data Parameters
NAME LH-II-71 OLD NMR
EXPNO 10
PROCNO 1

F2 - Acquisition Parameters
Date_ 20230613
Time 16.33 h
INSTRUM AVIII_400
PROBHD Z108618_0146 (
PULPROG zg30
TD 65536
SOLVENT CDCl3
NS 16
DS 2
SWH 8223.685 Hz
FIDRES 0.250967 Hz
AQ 3.9845889 sec
RG 144
DW 60.800 usec
DE 17.42 usec
TE 300.0 K
D1 1.00000000 sec
TD0 1
SFO1 400.1124708 MHz
NUC1 1H
P0 5.00 usec
P1 15.00 usec
PLW1 17.29199982 W

F2 - Processing parameters
SI 32768
SF 400.1100036 MHz
WDW EM
SSB 0
LB 0.30 Hz
GB 0
PC 1.00

Expanded region of the ^{1}H NMR spectrum of compound 17

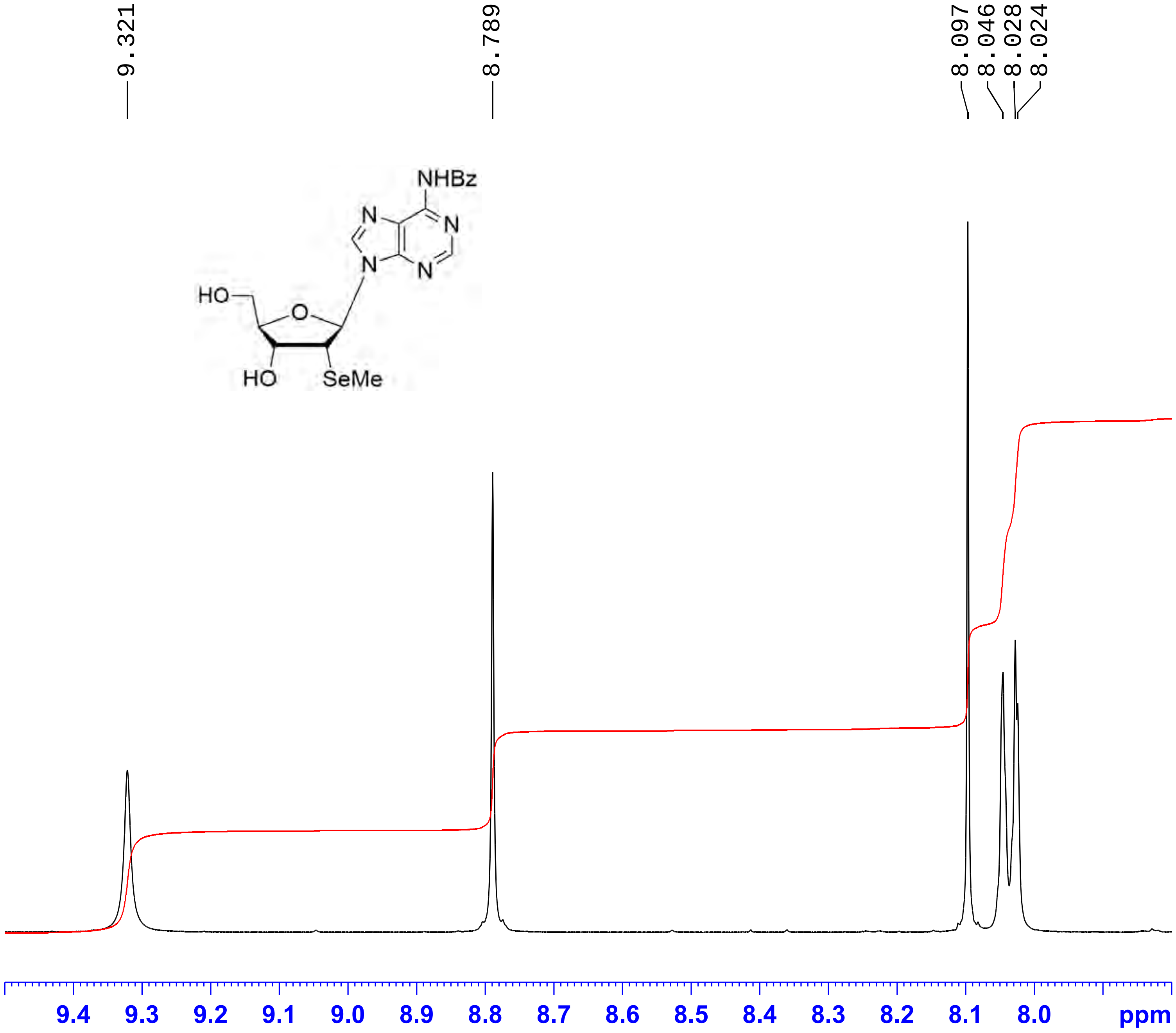


```
Current Data Parameters
NAME       LH-II-71 OLD NMR
EXPNO                    10
PROCNO                    1

F2 - Acquisition Parameters
Date_              20230613
Time                  16.33 h
INSTRUM           AVIII_400
PROBHD     Z108618_0146 (
PULPROG                zg30
TD                    65536
SOLVENT               CDCl3
NS                       16
DS                        2
SWH                8223.685 Hz
FIDRES             0.250967 Hz
AQ                3.9845889 sec
RG                      144
DW                   60.800 usec
DE                    17.42 usec
TE                    300.0 K
D1               1.00000000 sec
TD0                       1
SFO1            400.1124708 MHz
NUC1                     1H
P0                     5.00 usec
P1                    15.00 usec
PLW1            17.29199982 W

F2 - Processing parameters
SI                    32768
SF              400.1100036 MHz
WDW                      EM
SSB         0
LB                     0.30 Hz
GB          0
PC                     1.00
```

Expanded region of the ^{1}H NMR spectrum of compound 17

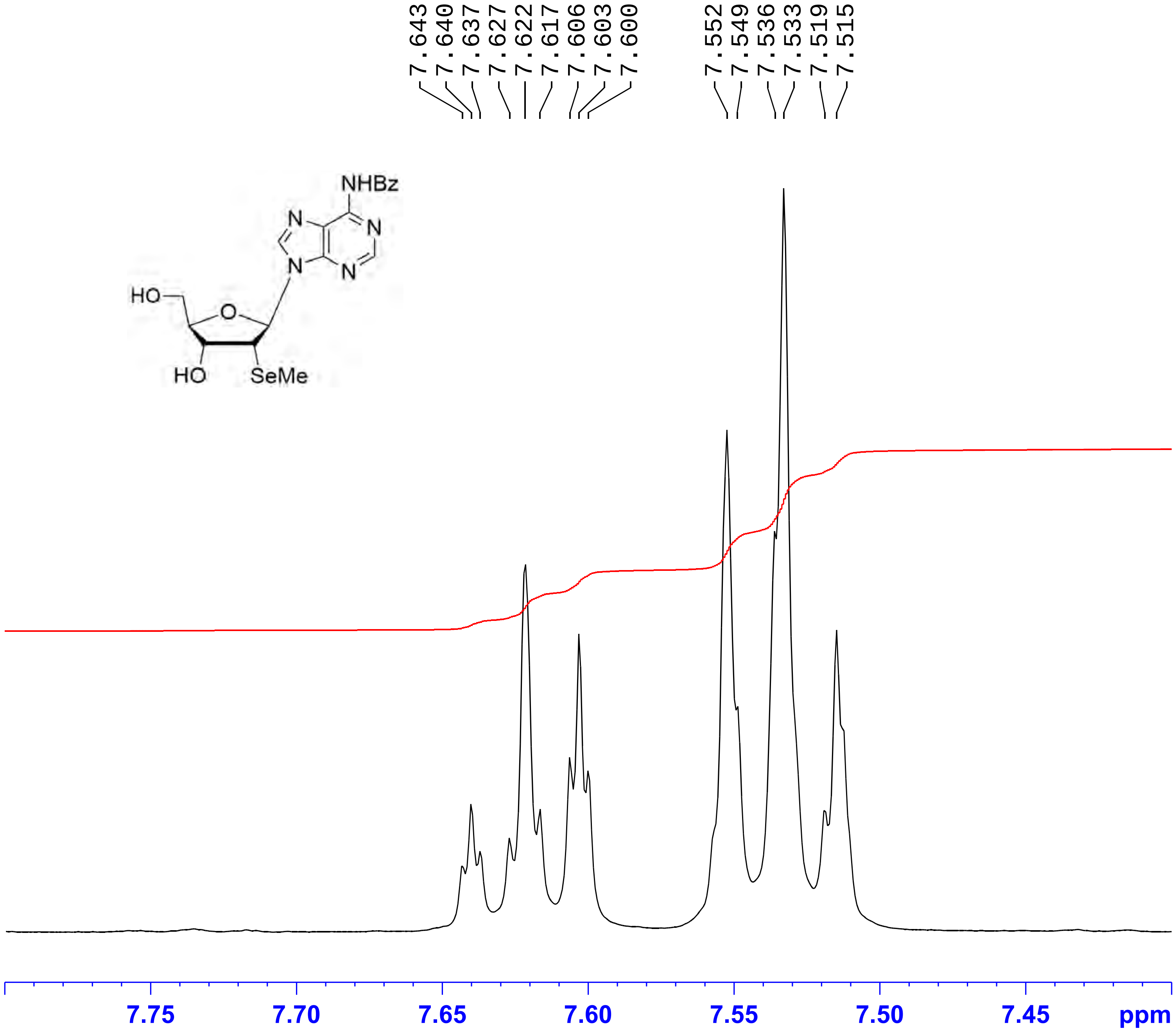


```
Current Data Parameters
NAME       LH-II-71 OLD NMR
EXPNO                    10
PROCNO                    1

F2 - Acquisition Parameters
Date_              20230613
Time                  16.33 h
INSTRUM           AVIII_400
PROBHD     Z108618_0146 (
PULPROG                zg30
TD                    65536
SOLVENT               CDCl3
NS                       16
DS                        2
SWH                8223.685 Hz
FIDRES             0.250967 Hz
AQ                3.9845889 sec
RG                      144
DW                   60.800 usec
DE                    17.42 usec
TE                    300.0 K
D1               1.00000000 sec
TD0                       1
SFO1            400.1124708 MHz
NUC1                     1H
P0                     5.00 usec
P1                    15.00 usec
PLW1            17.29199982 W

F2 - Processing parameters
SI                    32768
SF              400.1100036 MHz
WDW                      EM
SSB       0
LB                     0.30 Hz
GB        0
PC                     1.00
```

Expanded region of the ^{1}H NMR spectrum of compound 17

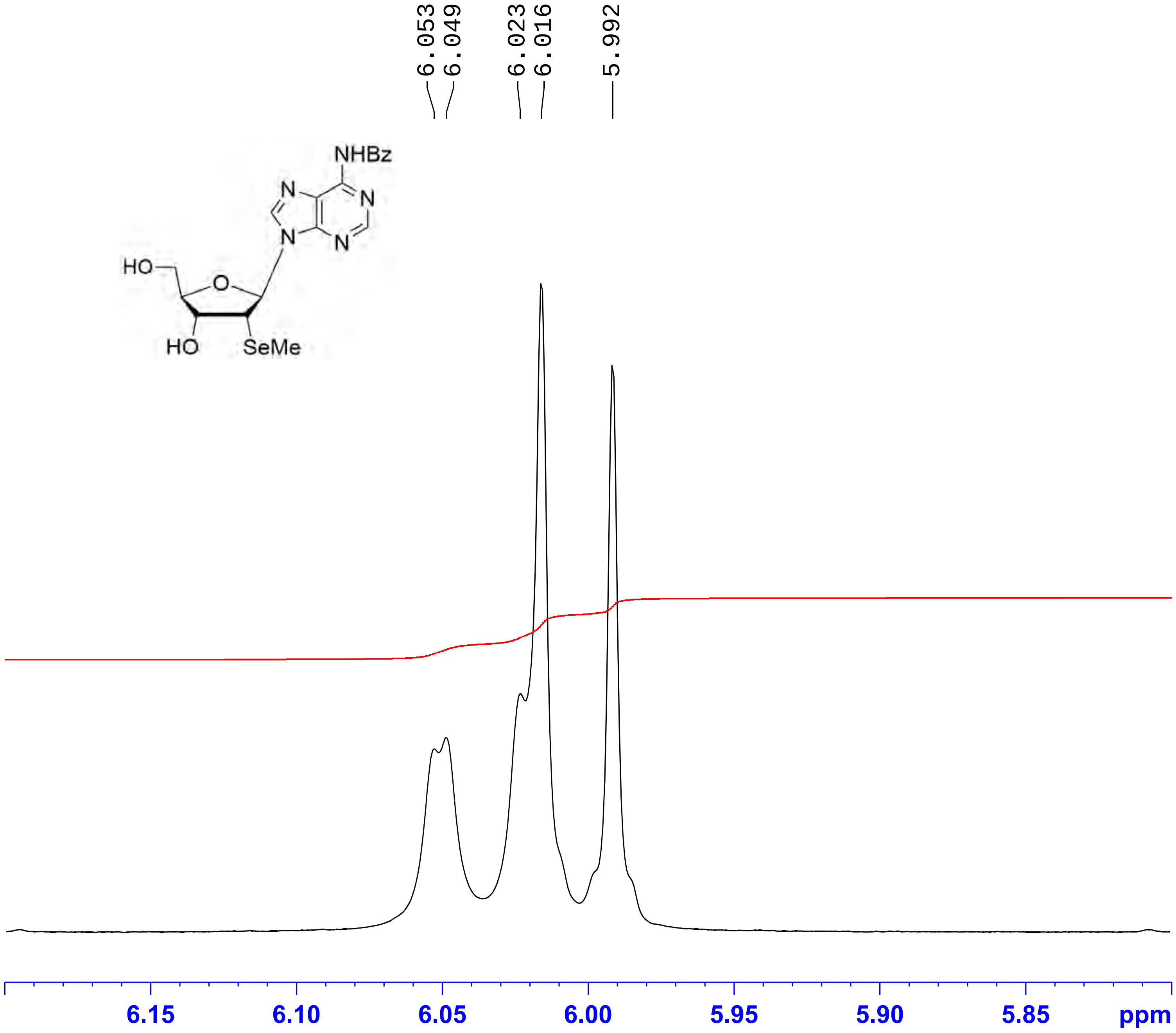

Current Data Parameters
NAME LH-II-71 OLD NMR
EXPNO 10
PROCNO 1

F2 - Acquisition Parameters
Date_ 20230613
Time 16.33 h
INSTRUM AVIII_400
PROBHD Z108618_0146 (
PULPROG zg30
TD 65536
SOLVENT CDCl3
NS 16
DS 2
SWH 8223.685 Hz
FIDRES 0.250967 Hz
AQ 3.9845889 sec
RG 144
DW 60.800 usec
DE 17.42 usec
TE 300.0 K
D1 1.00000000 sec
TD0 1
SFO1 400.1124708 MHz
NUC1 1H
P0 5.00 usec
P1 15.00 usec
PLW1 17.29199982 W

F2 - Processing parameters
SI 32768
SF 400.1100036 MHz
WDW EM
SSB 0
LB 0.30 Hz
GB 0
PC 1.00

Expanded region of the ^{1}H NMR spectrum of compound 17

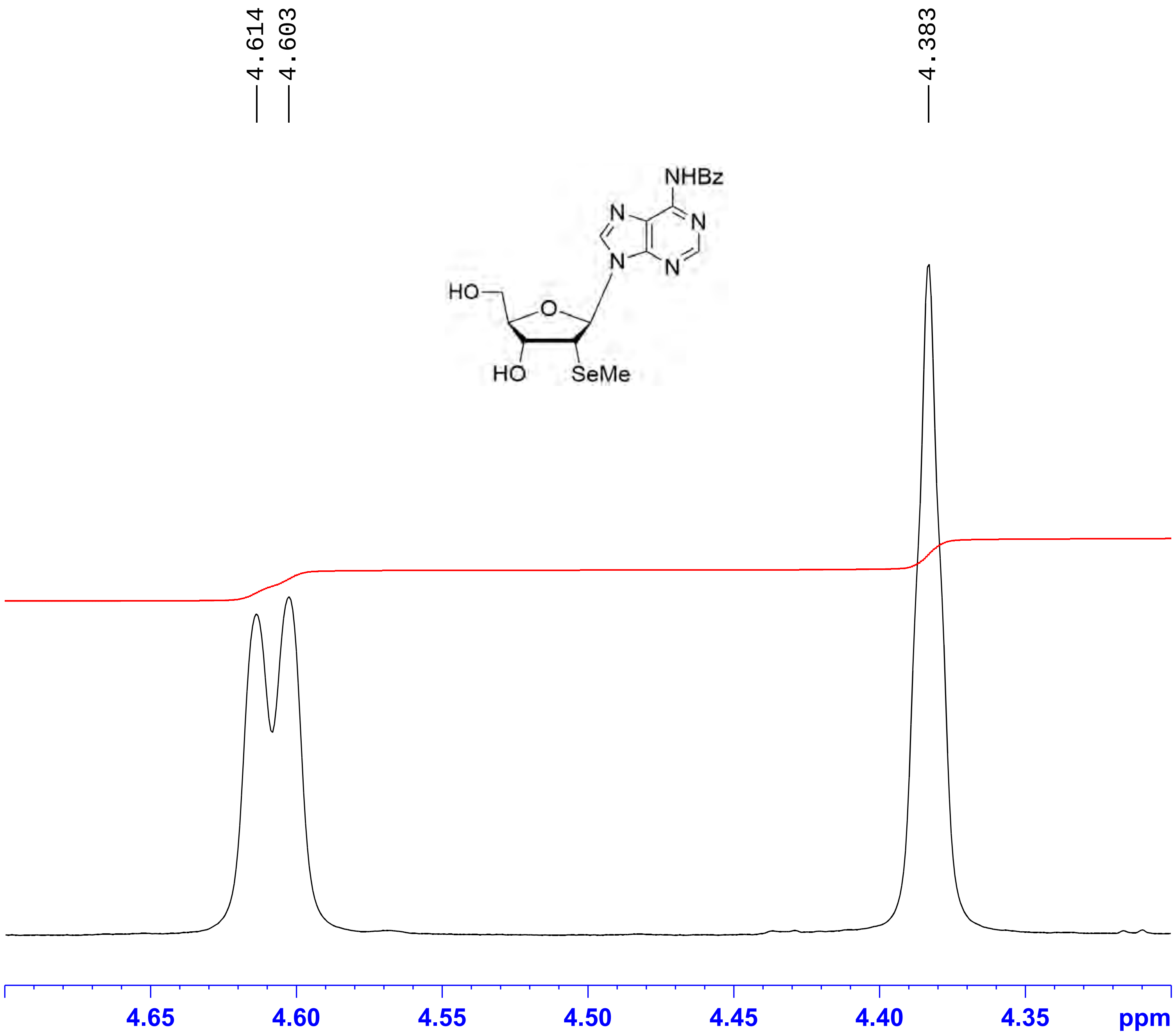


```
Current Data Parameters
NAME       LH-II-71 OLD NMR
EXPNO                    10
PROCNO                    1

F2 - Acquisition Parameters
Date_              20230613
Time                  16.33 h
INSTRUM          AVIII_400
PROBHD     Z108618_0146 (
PULPROG                zg30
TD                    65536
SOLVENT               CDCl3
NS                       16
DS                        2
SWH                8223.685 Hz
FIDRES             0.250967 Hz
AQ                3.9845889 sec
RG                      144
DW                   60.800 usec
DE                    17.42 usec
TE                    300.0 K
D1               1.00000000 sec
TD0                       1
SFO1            400.1124708 MHz
NUC1                     1H
P0                     5.00 usec
P1                    15.00 usec
PLW1            17.29199982 W

F2 - Processing parameters
SI                    32768
SF              400.1100036 MHz
WDW                      EM
SSB       0
LB                     0.30 Hz
GB        0
PC                     1.00
```

Expanded region of the ^{1}H NMR spectrum of compound 17

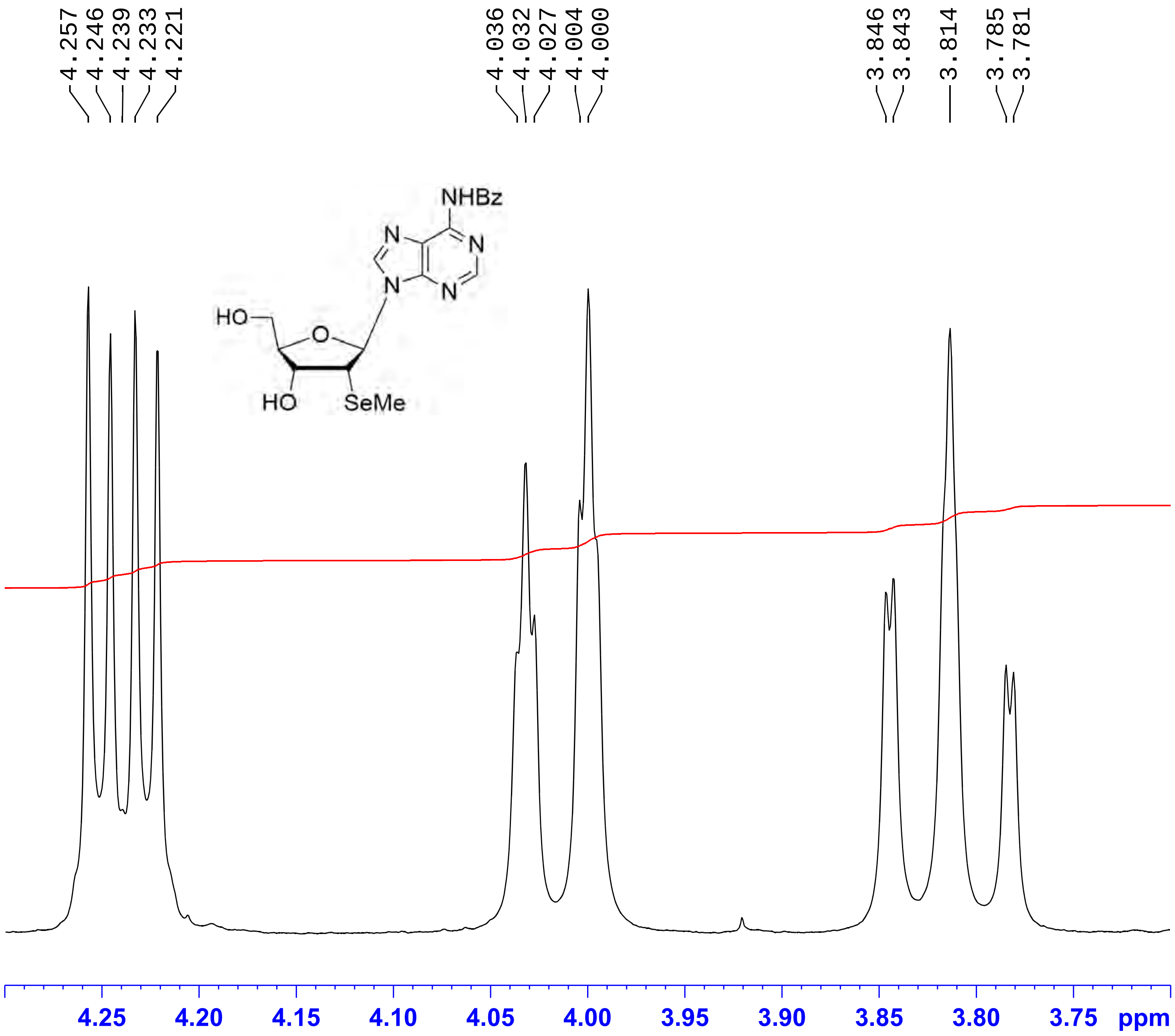


```
Current Data Parameters
NAME       LH-II-71 OLD NMR
EXPNO                    10
PROCNO                    1

F2 - Acquisition Parameters
Date_              20230613
Time                  16.33 h
INSTRUM           AVIII_400
PROBHD     Z108618_0146 (
PULPROG                zg30
TD                    65536
SOLVENT               CDCl3
NS                       16
DS                        2
SWH                8223.685 Hz
FIDRES             0.250967 Hz
AQ                3.9845889 sec
RG                      144
DW                   60.800 usec
DE                    17.42 usec
TE                    300.0 K
D1               1.00000000 sec
TD0                       1
SFO1            400.1124708 MHz
NUC1                     1H
P0                     5.00 usec
P1                    15.00 usec
PLW1            17.29199982 W

F2 - Processing parameters
SI                    32768
SF              400.1100036 MHz
WDW                      EM
SSB        0
LB                     0.30 Hz
GB         0
PC                     1.00
```

Expanded region of the ^{1}H NMR spectrum of compound 17

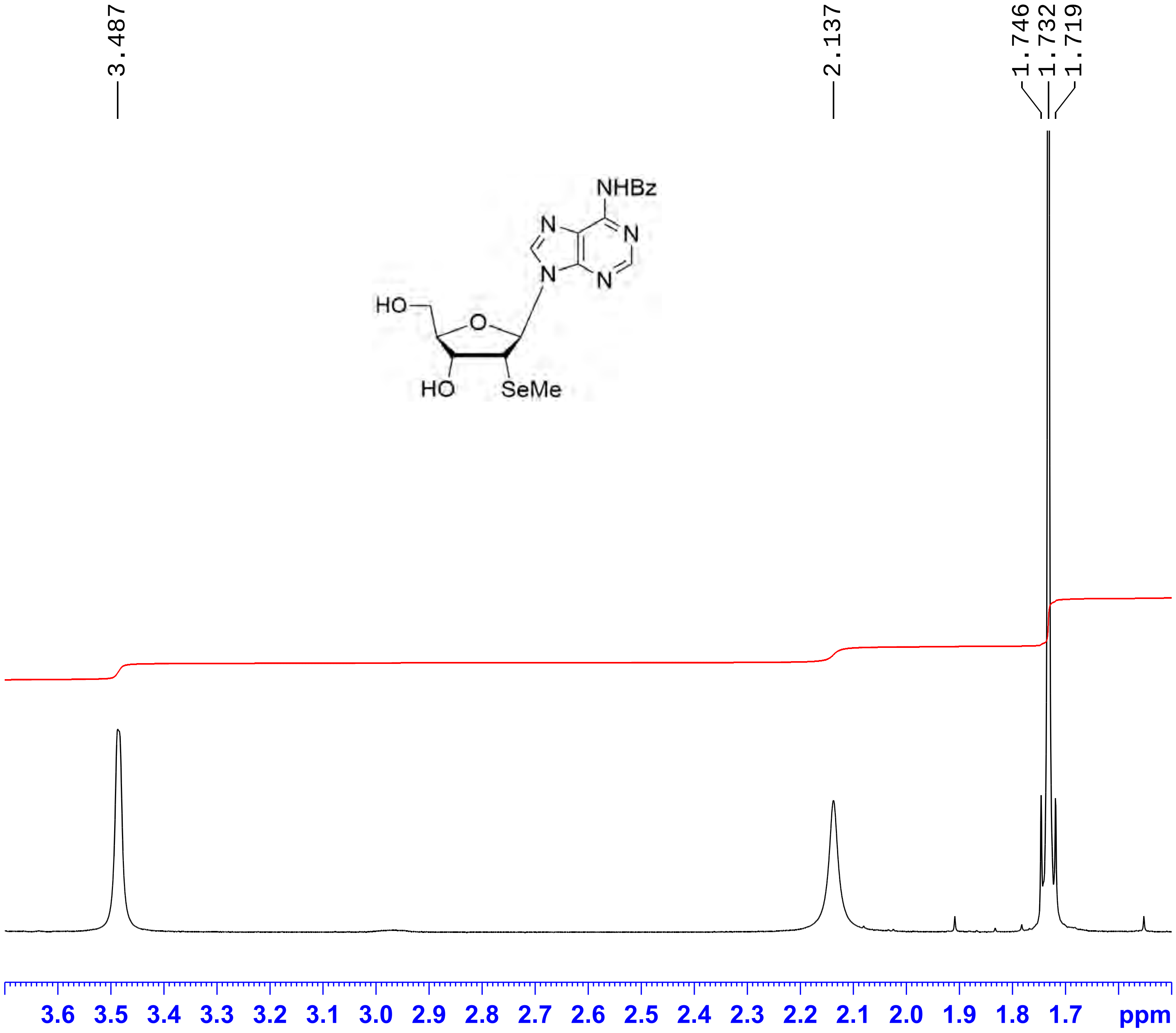

Current Data Parameters
NAME LH-II-71 OLD NMR
EXPNO 10
PROCNO 1

F2 - Acquisition Parameters
Date_ 20230613
Time 16.33 h
INSTRUM AVIII_400
PROBHD Z108618_0146 (
PULPROG zg30
TD 65536
SOLVENT CDCl3
NS 16
DS 2
SWH 8223.685 Hz
FIDRES 0.250967 Hz
AQ 3.9845889 sec
RG 144
DW 60.800 usec
DE 17.42 usec
TE 300.0 K
D1 1.00000000 sec
TD0 1
SFO1 400.1124708 MHz
NUC1 1H
P0 5.00 usec
P1 15.00 usec
PLW1 17.29199982 W

F2 - Processing parameters
SI 32768
SF 400.1100036 MHz
WDW EM
SSB 0
LB 0.30 Hz
GB 0
PC 1.00

^{13}C NMR spectrum of compound 17

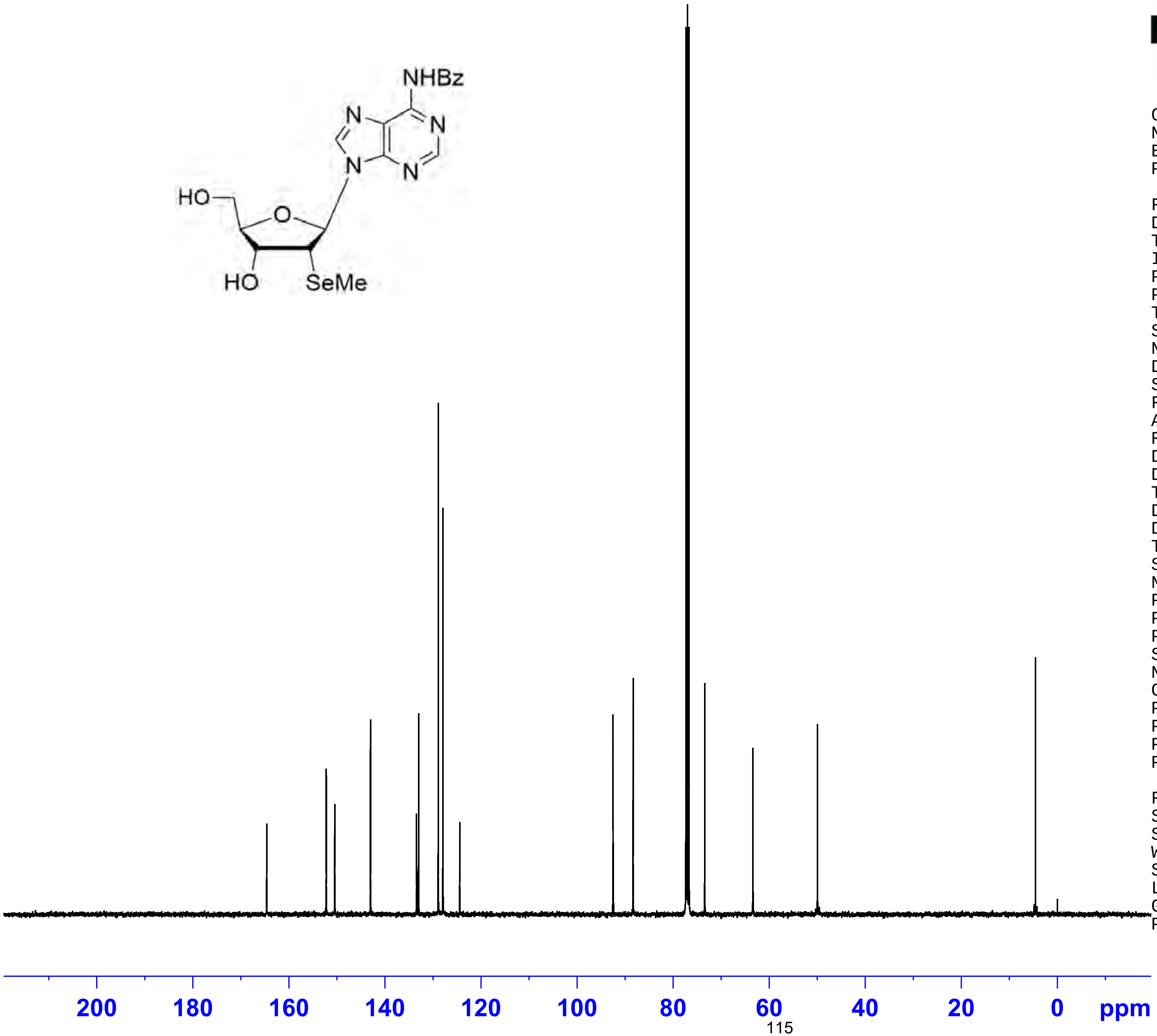

Current Data Parameters

NAME	LH-II-71 OLD NMR
EXPNO	11
PROCNO	1

F2 - Acquisition Parameters

Date_	20230614
Time	0.55 h
INSTRUM	AVIII_400
PROBHD	Z108618_0146 (
PULPROG	zgpg30
TD	96150
SOLVENT	CDCl3
NS	3000
DS	4
SWH	24038.461 Hz
FIDRES	0.500020 Hz
AQ	1.9999200 sec
RG	2050
DW	20.800 usec
DE	6.50 usec
TE	300.0 K
D1	1.00000000 sec
D11	0.03000000 sec
TD0	1
SFO1	100.6178003 MHz
NUC1	13C
P0	2.90 usec
P1	8.70 usec
PLW1	96.68000031 W
SFO2	400.1116004 MHz
NUC2	1H
CPDPRG[2	waltz64
PCPD2	90.00 usec
PLW2	17.29199982 W
PLW12	0.48032999 W
PLW13	0.24160001 W

F2 - Processing parameters

SI	131072
SF	100.6077459 MHz
WDW	EM
SSB	0
LB	1.00 Hz
GB	0
PC	1.40

Expanded region of the ^{13}C NMR spectrum of compound 17

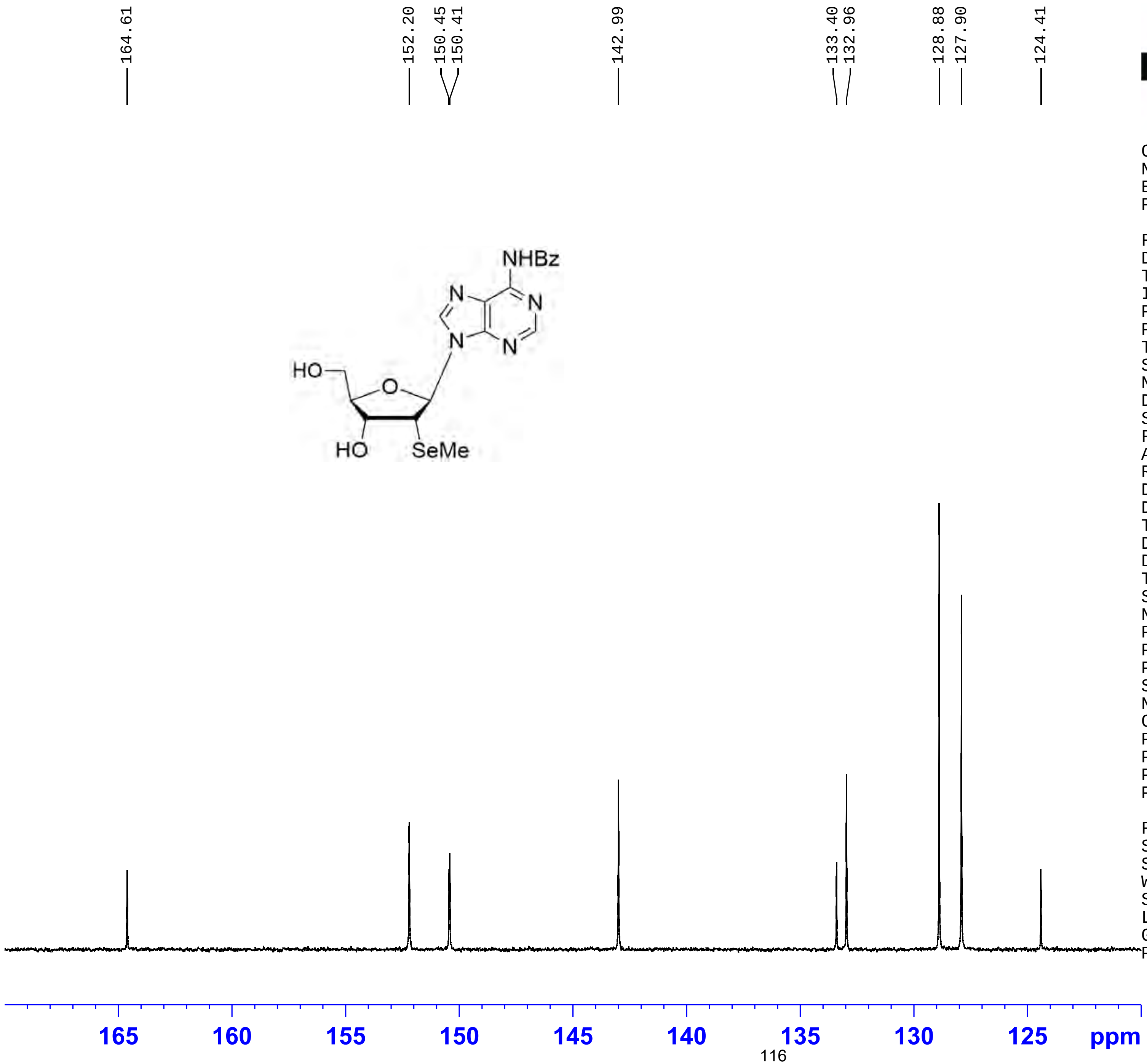

BRUKER

Current Data Parameters
NAME LH-II-71 OLD NMR
EXPNO 11
PROCNO 1

F2 - Acquisition Parameters

Date_	20230614
Time	0.55 h
INSTRUM	AVIII_400
PROBHD	Z108618_0146 (
PULPROG	zgpg30
TD	96150
SOLVENT	CDCl3
NS	3000
DS	4
SWH	24038.461 Hz
FIDRES	0.500020 Hz
AQ	1.9999200 sec
RG	2050
DW	20.800 usec
DE	6.50 usec
TE	300.0 K
D1	1.00000000 sec
D11	0.03000000 sec
TD0	1
SFO1	100.6178003 MHz
NUC1	13C
P0	2.90 usec
P1	8.70 usec
PLW1	96.68000031 W
SFO2	400.1116004 MHz
NUC2	1H
CPDPRG[2	waltz64
PCPD2	90.00 usec
PLW2	17.29199982 W
PLW12	0.48032999 W
PLW13	0.24160001 W

F2 - Processing parameters

SI	131072
SF	100.6077459 MHz
WDW	EM
SSB	0
LB	1.00 Hz
GB	0
PC	1.40

Expanded region of the ^{13}C NMR spectrum of compound 17

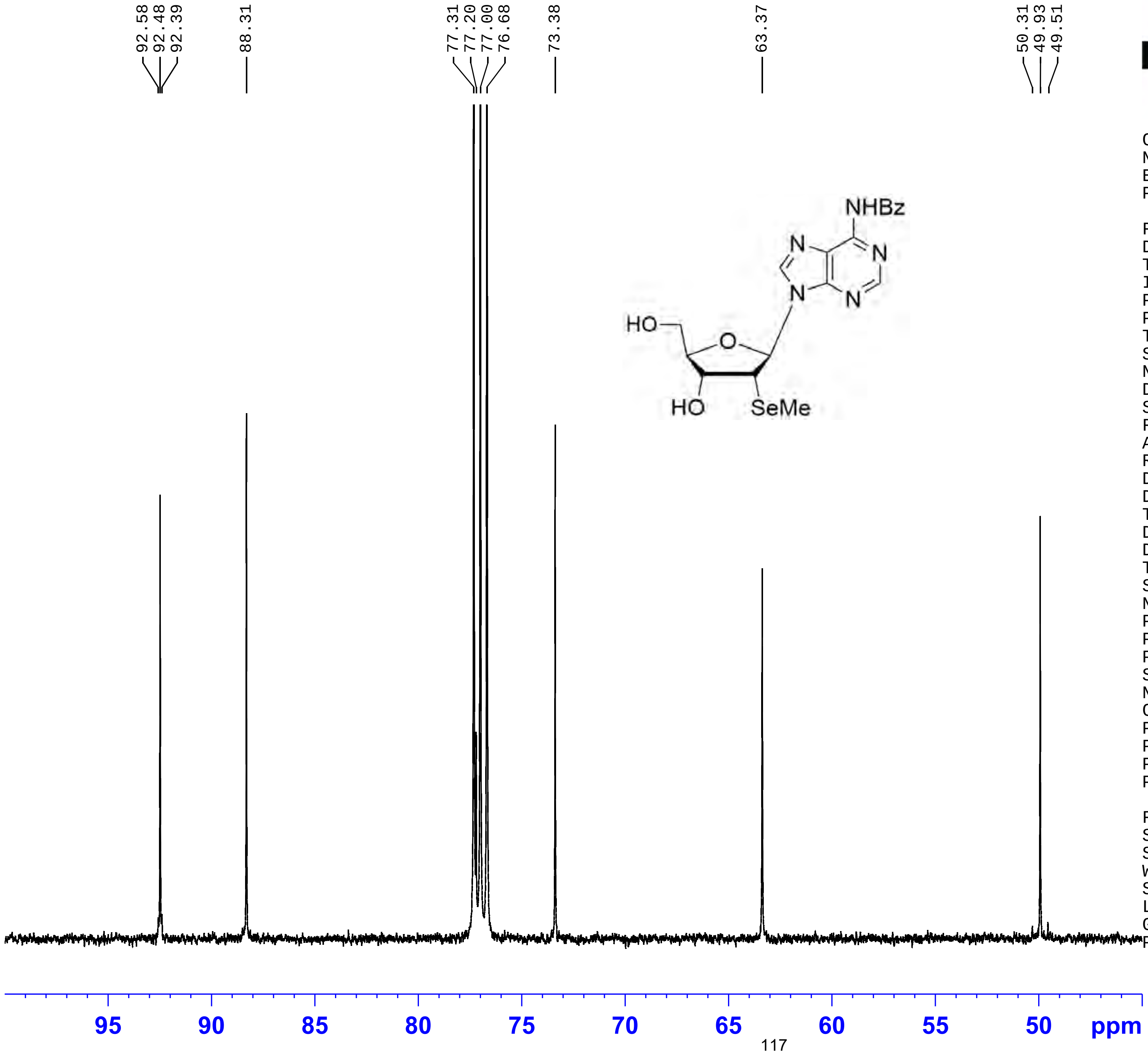

Current Data Parameters
NAME LH-II-71 OLD NMR
EXPNO 11
PROCNO 1

F2 - Acquisition Parameters
Date_ 20230614
Time 0.55 h
INSTRUM AVIII_400
PROBHD Z108618_0146 (
PULPROG zgpg30
TD 96150
SOLVENT CDCl3
NS 3000
DS 4
SWH 24038.461 Hz
FIDRES 0.500020 Hz
AQ 1.9999200 sec
RG 2050
DW 20.800 usec
DE 6.50 usec
TE 300.0 K
D1 1.00000000 sec
D11 0.03000000 sec
TD0 1
SFO1 100.6178003 MHz
NUC1 13C
P0 2.90 usec
P1 8.70 usec
PLW1 96.68000031 W
SFO2 400.1116004 MHz
NUC2 1H
CPDPRG[2 waltz64
PCPD2 90.00 usec
PLW2 17.29199982 W
PLW12 0.48032999 W
PLW13 0.24160001 W

F2 - Processing parameters
SI 131072
SF 100.6077459 MHz
WDW EM
SSB 0
LB 1.00 Hz
GB 0
PC 1.40

Expanded region of the ^{13}C NMR spectrum of compound 17

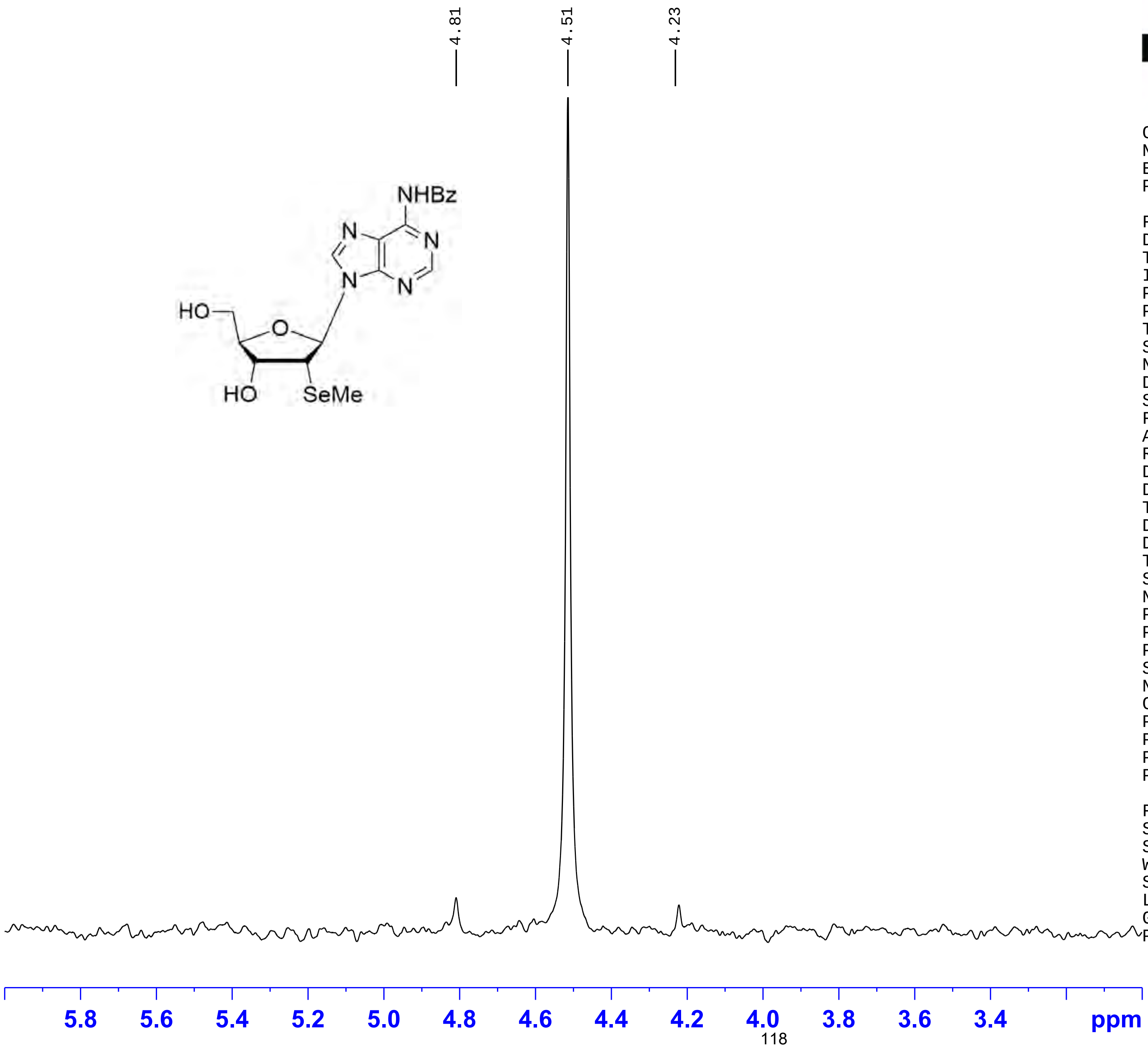

Current Data Parameters

NAME	LH-II-71 OLD NMR
EXPNO	11
PROCNO	1

F2 - Acquisition Parameters

Date_	20230614
Time	0.55 h
INSTRUM	AVIII_400
PROBHD	Z108618_0146 (
PULPROG	zgpg30
TD	96150
SOLVENT	CDCl3
NS	3000
DS	4
SWH	24038.461 Hz
FIDRES	0.500020 Hz
AQ	1.9999200 sec
RG	2050
DW	20.800 usec
DE	6.50 usec
TE	300.0 K
D1	1.00000000 sec
D11	0.03000000 sec
TD0	1
SFO1	100.6178003 MHz
NUC1	13C
P0	2.90 usec
P1	8.70 usec
PLW1	96.68000031 W
SFO2	400.1116004 MHz
NUC2	1H
CPDPRG[2	waltz64
PCPD2	90.00 usec
PLW2	17.29199982 W
PLW12	0.48032999 W
PLW13	0.24160001 W

F2 - Processing parameters

SI	131072
SF	100.6077459 MHz
WDW	EM
SSB	0
LB	1.00 Hz
GB	0
PC	1.40

^{13}C DEPT-135 NMR spectrum of compound 17

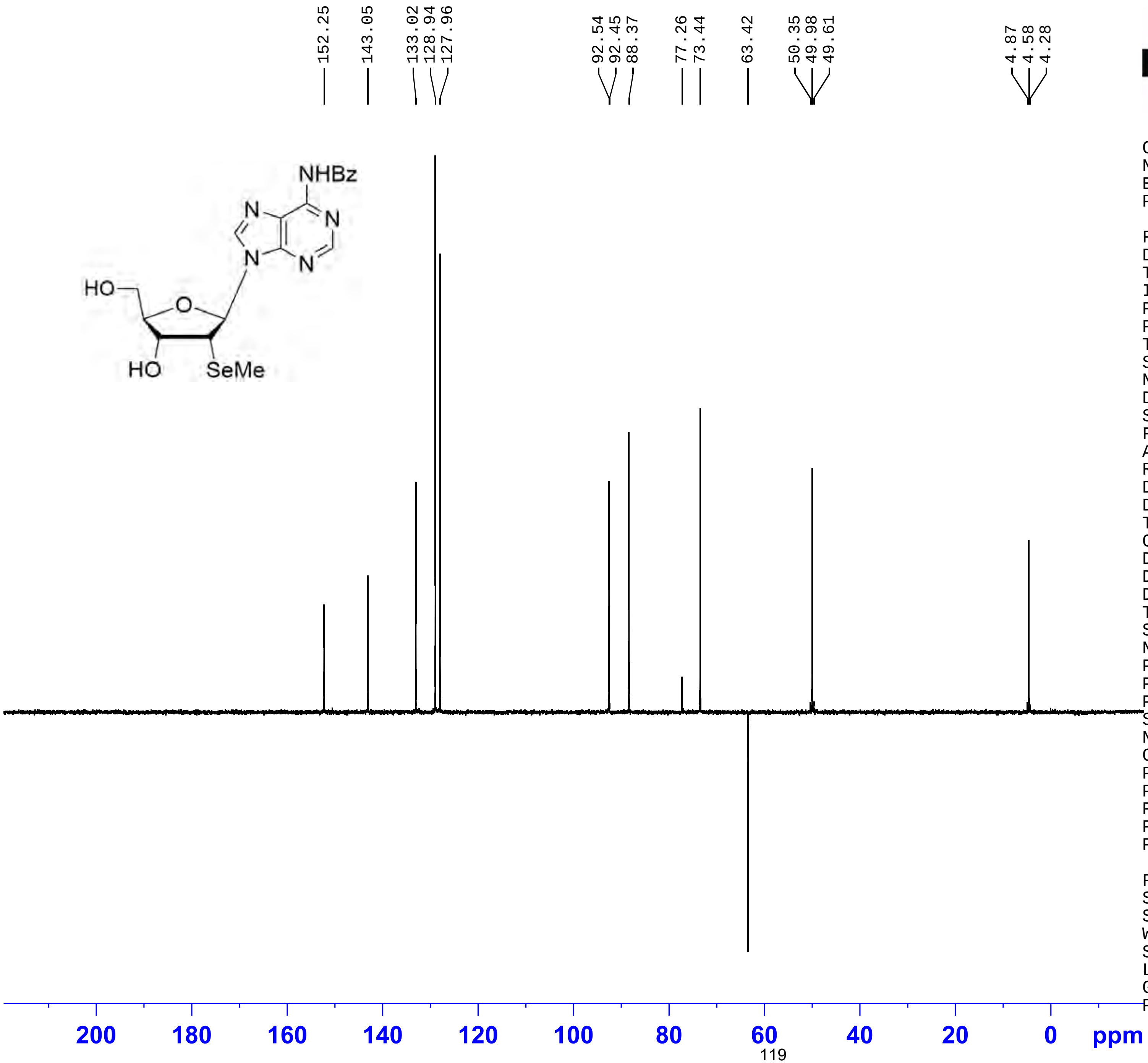

Current Data Parameters
NAME LH-II-71 OLD NMR
EXPNO 13
PROCNO 1

F2 - Acquisition Parameters
Date_ 20230614
Time 3.48 h
INSTRUM AVIII_400
PROBHD Z108618_0146 (
PULPROG dept135
TD 65536
SOLVENT CDCl3
NS 1500
DS 4
SWH 24038.461 Hz
FIDRES 0.733596 Hz
AQ 1.3631488 sec
RG 2050
DW 20.800 usec
DE 6.50 usec
TE 300.0 K
CNST2 145.0000000
D1 2.00000000 sec
D2 0.00344828 sec
D12 0.00002000 sec
TD0 1
SFO1 100.6178003 MHz
NUC1 13C
P1 8.70 usec
P2 17.40 usec
PLW1 96.68000031 W
SFO2 400.1116004 MHz
NUC2 1H
CPDPRG[2 waltz64
P3 15.00 usec
P4 30.00 usec
PCPD2 90.00 usec
PLW2 17.29199982 W
PLW12 0.48032999 W

F2 - Processing parameters
SI 32768
SF 100.6077400 MHz
WDW EM
SSB 0
LB 1.00 Hz
GB 0
PC 1.40

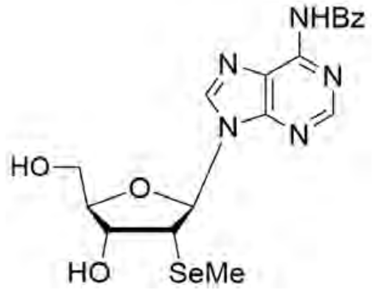

^{1}H-^{1}H COSY NMR spectrum of compound 17

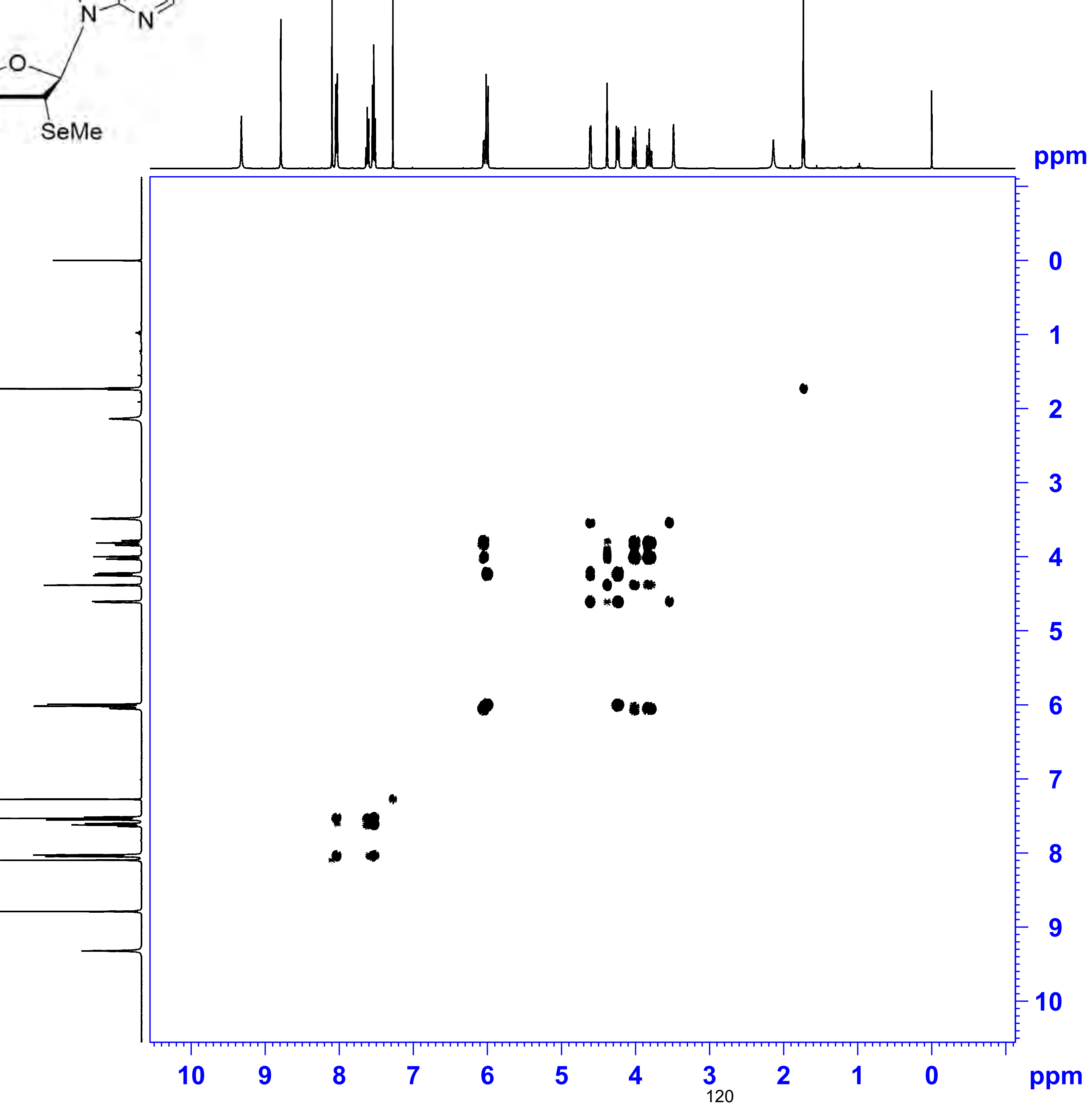


```
Current Data Parameters
NAME      LH-II-71 NEW NMR
EXPNO                   13
PROCNO                   1

F2 - Acquisition Parameters
Date_             20230614
Time                 23.37 h
INSTRUM           AVIII_400
PROBHD    Z108618_0817 (
PULPROG        cosygpmfqf
TD                    2048
SOLVENT              CDCl3
NS                       3
DS                       8
SWH               4672.897 Hz
FIDRES            4.563376 Hz
AQ               0.2191360 sec
RG                    1620
DW                 107.000 usec
DE                    6.50 usec
TE                   297.6 K
D0              0.00000300 sec
D1              1.97282600 sec
D13             0.00000400 sec
D16             0.00020000 sec
IN0             0.00021400 sec
TDav                     1
SFO1           399.9118881 MHz
NUC1                    1H
P1                  500.00 usec
PLW1           31.62299919 W
GPNAM[1]          SINE.100
GPZ1                 16.00 %
GPNAM[2]          SINE.100
GPZ2                 12.00 %
GPNAM[3]          SINE.100
GPZ3                 40.00 %
P16                1000.00 usec

F1 - Acquisition parameters
TD                     256
SFO1              399.9119 MHz
FIDRES           36.507008 Hz
SW                  11.685 ppm
FnMODE                  QF

F2 - Processing parameters
SI                    1024
SF             399.9100031 MHz
WDW                   SINE
SSB       0
LB        0 Hz
GB        0
PC                    1.40

F1 - Processing parameters
SI                    1024
MC2                     QF
SF             399.9100031 MHz
WDW                   SINE
SSB       0
LB        0 Hz
GB        0
```

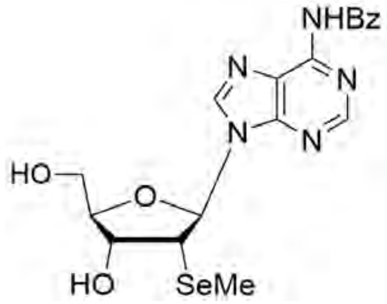

^{1}H-^{13}C HSQC NMR spectrum of compound 17

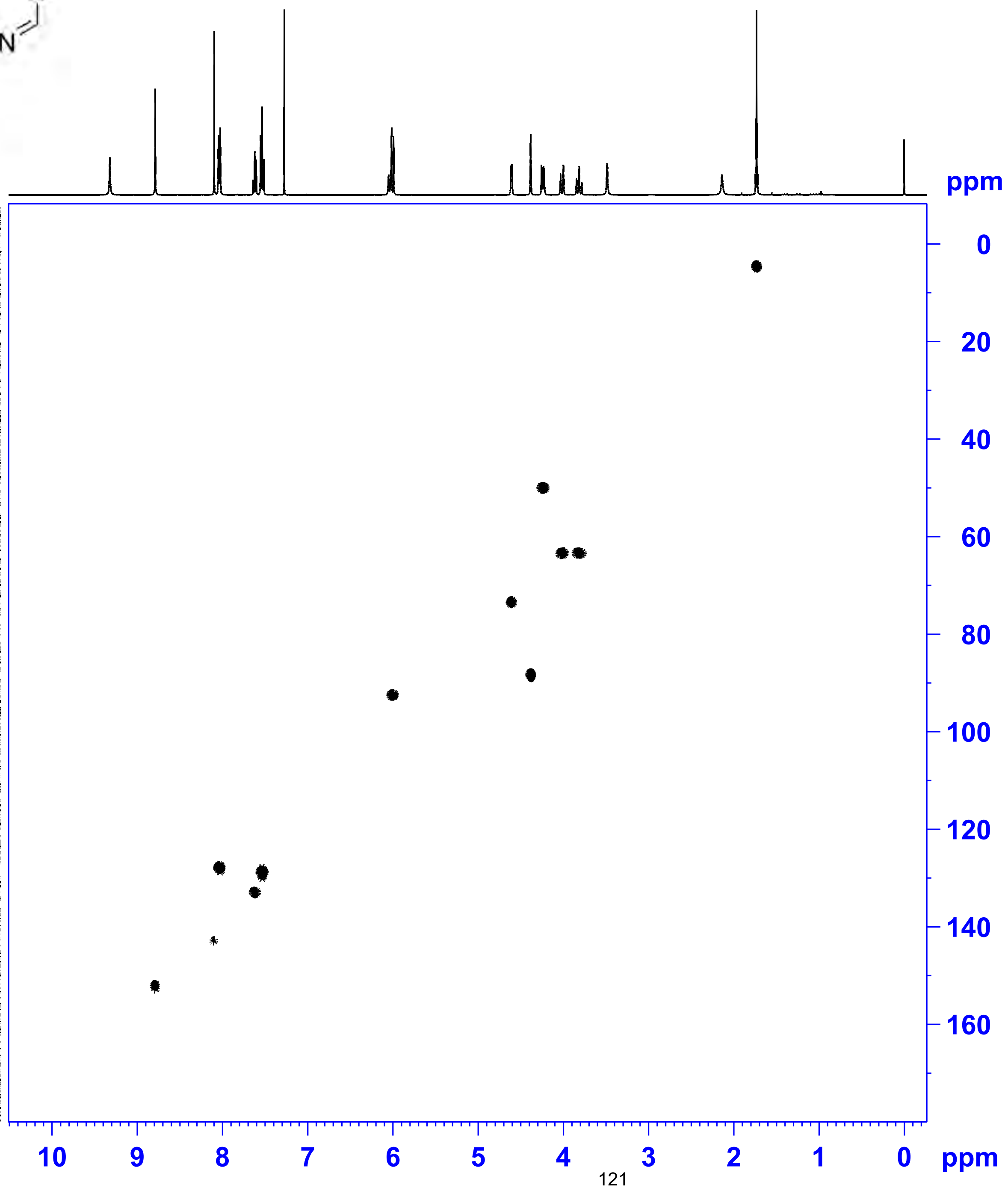


```
Current Data Parameters
NAME      LH-II-71 OLD NMR
EXPNO                   15
PROCNO                   1

F2 - Acquisition Parameters
Date_             20230614
Time                  4.18 h
INSTRUM          AVIII_400
PROBHD    Z108618_0146 (
PULPROG        hsqcedetgp
TD                    1024
SOLVENT              CDCl3
NS                       4
DS                      16
SWH               4310.345 Hz
FIDRES            8.418642 Hz
AQ               0.1187840 sec
RG                    1290
DW                 116.000 usec
DE                    6.50 usec
TE                   300.0 K
CNST2          145.0000000
D0              0.00000300 sec
D1              1.45965397 sec
D4              0.00172414 sec
D11             0.03000000 sec
D16             0.00020000 sec
D21             0.00345000 sec
IN0             0.00003000 sec
TDav                     1
ZGOPTNS
SFO1           400.1120536 MHz
NUC1                    1H
P1                   15.00 usec
P2                   30.00 usec
PLW1           17.29199982 W
SFO2           100.6152434 MHz
NUC2                   13C
CPDPRG[2              garp
P3                    8.70 usec
P4                   17.40 usec
PCPD2                56.50 usec
PLW2           96.68000031 W
PLW12           3.16230011 W
GPNAM[1]       SMSQ10.100
GPZ1                 80.00 %
GPNAM[2]       SMSQ10.100
GPZ2                 20.10 %
P16                1000.00 usec

F1 - Acquisition parameters
TD                     256
SFO1              100.6152 MHz
FIDRES          130.208328 Hz
SW                 165.648 ppm
FnMODE       Echo-Antiecho

F2 - Processing parameters
SI                    1024
SF             400.1100036 MHz
WDW                  QSINE
SSB                      2
LB                0 Hz
GB                0
PC                    1.40

F1 - Processing parameters
SI                    1024
MC2          echo-antiecho
SF             100.6077400 MHz
WDW                  QSINE
SSB                      2
LB                0 Hz
GB                0
```

^{1}H NMR spectrum of compound 18

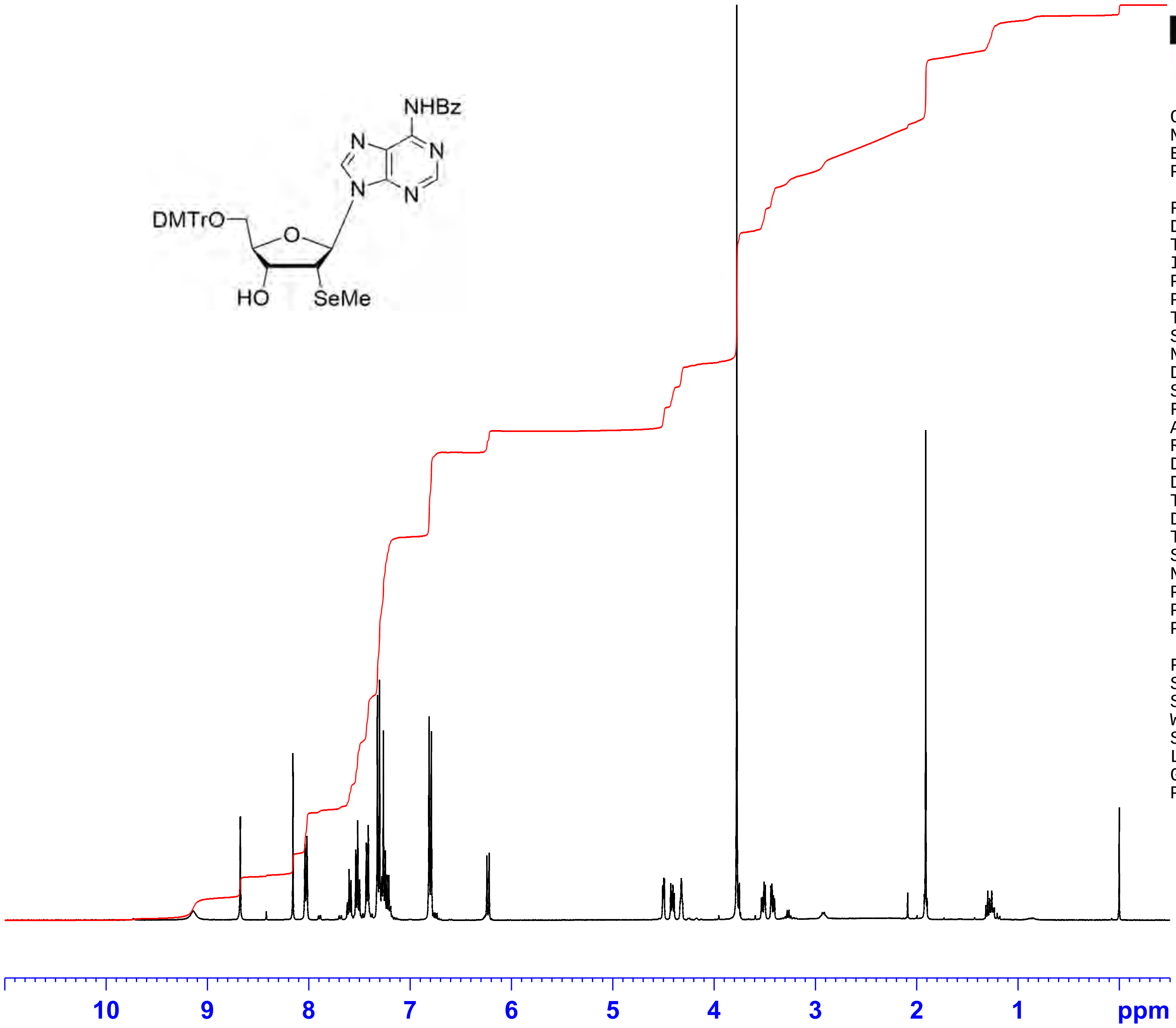


```
Current Data Parameters
NAME       LH-II-72 OLD NMR
EXPNO                    10
PROCNO                    1

F2 - Acquisition Parameters
Date_              20230625
Time                  14.11 h
INSTRUM           AVIII_400
PROBHD     Z108618_0146 (
PULPROG                zg30
TD                    65536
SOLVENT               CDCl3
NS                       16
DS                        2
SWH                8223.685 Hz
FIDRES             0.250967 Hz
AQ                3.9845889 sec
RG                      161
DW                   60.800 usec
DE                    17.42 usec
TE                    300.0 K
D1               1.00000000 sec
TD0                       1
SFO1            400.1124708 MHz
NUC1                     1H
P0                     5.00 usec
P1                    15.00 usec
PLW1            17.29199982 W

F2 - Processing parameters
SI                    32768
SF              400.1100086 MHz
WDW                      EM
SSB          0
LB                     0.30 Hz
GB           0
PC                     1.00
```

Expanded region of the ^{1}H NMR spectrum of compound 18

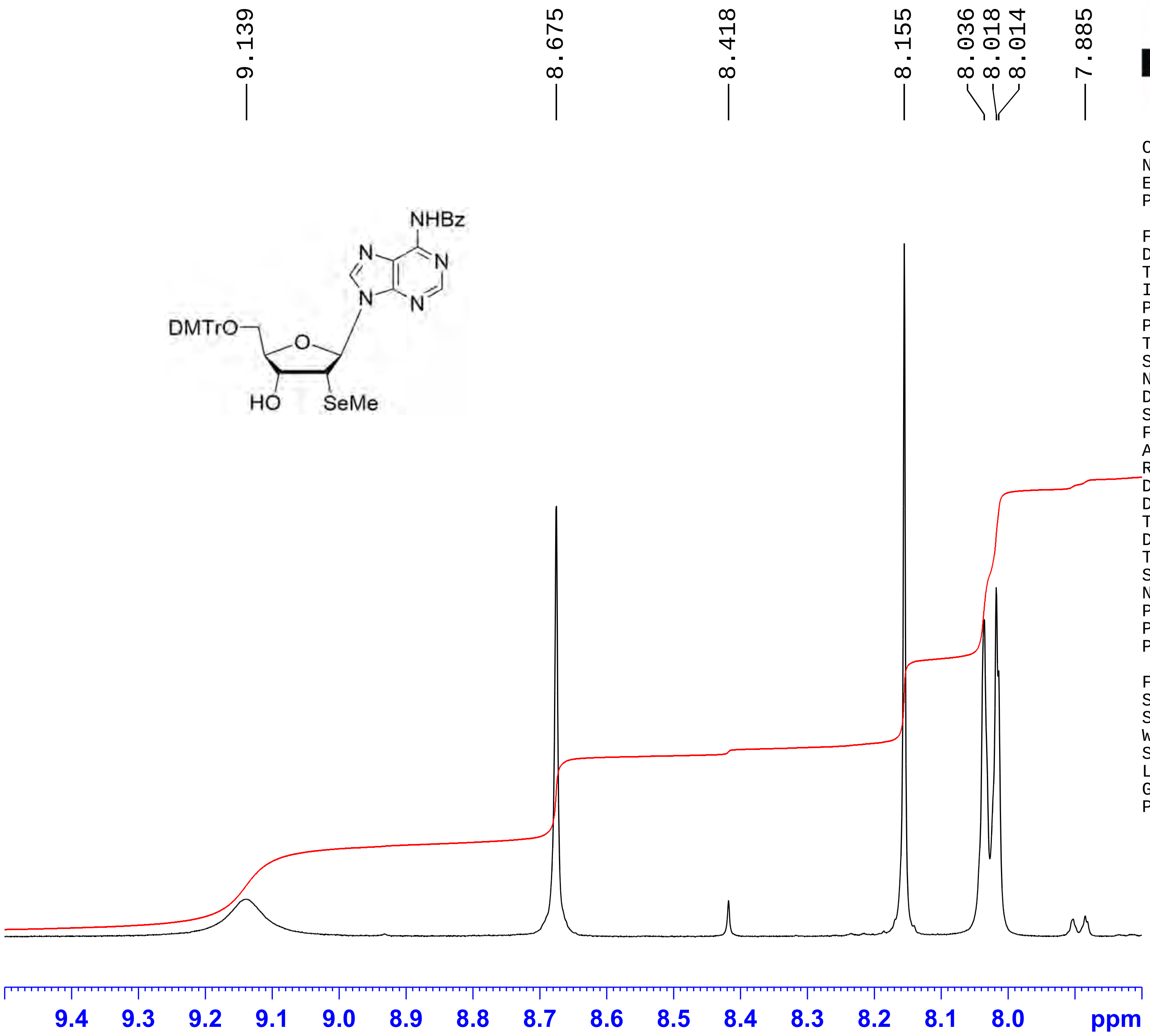


```
Current Data Parameters
NAME       LH-II-72 OLD NMR
EXPNO                    10
PROCNO                    1

F2 - Acquisition Parameters
Date_              20230625
Time                  14.11 h
INSTRUM           AVIII_400
PROBHD   Z108618_0146 (
PULPROG                zg30
TD                    65536
SOLVENT               CDCl3
NS                       16
DS                        2
SWH                8223.685 Hz
FIDRES             0.250967 Hz
AQ                3.9845889 sec
RG                      161
DW                   60.800 usec
DE                    17.42 usec
TE                    300.0 K
D1               1.00000000 sec
TD0                       1
SFO1            400.1124708 MHz
NUC1                     1H
P0                     5.00 usec
P1                    15.00 usec
PLW1            17.29199982 W

F2 - Processing parameters
SI                    32768
SF              400.1100086 MHz
WDW                      EM
SSB      0
LB                     0.30 Hz
GB       0
PC                     1.00
```

Expanded region of the ^{1}H NMR spectrum of compound 18

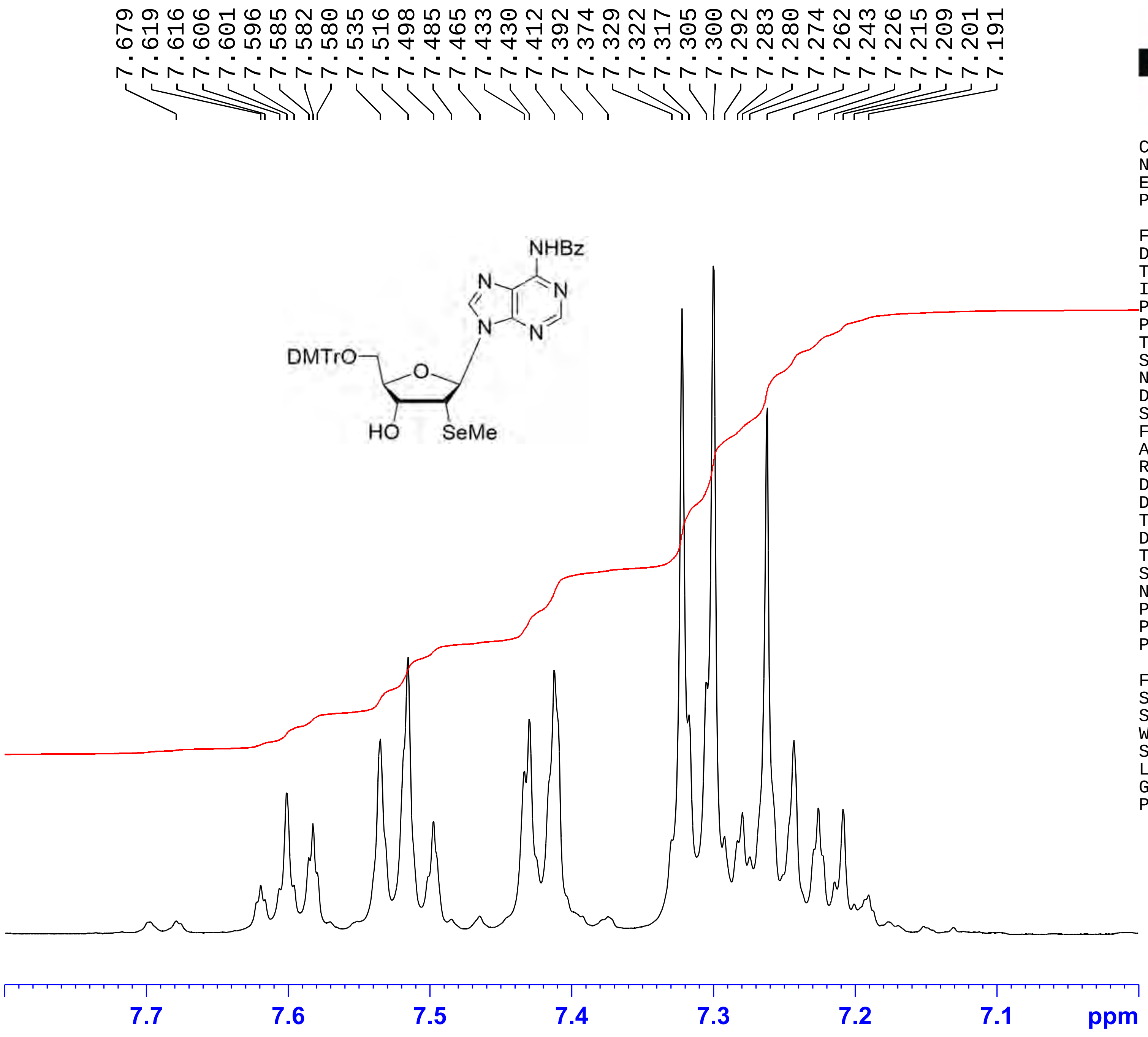

BRUKER

Current Data Parameters		
NAME	LH-II-72 OLD NMR	
EXPNO	10	
PROCNO	1	

F2 - Acquisition Parameters		
Date_	20230625	
Time	14.11	h
INSTRUM	AVIII_400	
PROBHD	Z108618_0146 (	
PULPROG	zg30	
TD	65536	
SOLVENT	CDCl3	
NS	16	
DS	2	
SWH	8223.685	Hz
FIDRES	0.250967	Hz
AQ	3.9845889	sec
RG	161	
DW	60.800	usec
DE	17.42	usec
TE	300.0	K
D1	1.00000000	sec
TD0	1	
SFO1	400.1124708	MHz
NUC1	1H	
P0	5.00	usec
P1	15.00	usec
PLW1	17.29199982	W

F2 - Processing parameters		
SI	32768	
SF	400.1100086	MHz
WDW	EM	
SSB	0	
LB	0.30	Hz
GB	0	
PC	1.00	

Expanded region of the ^{1}H NMR spectrum of compound 18

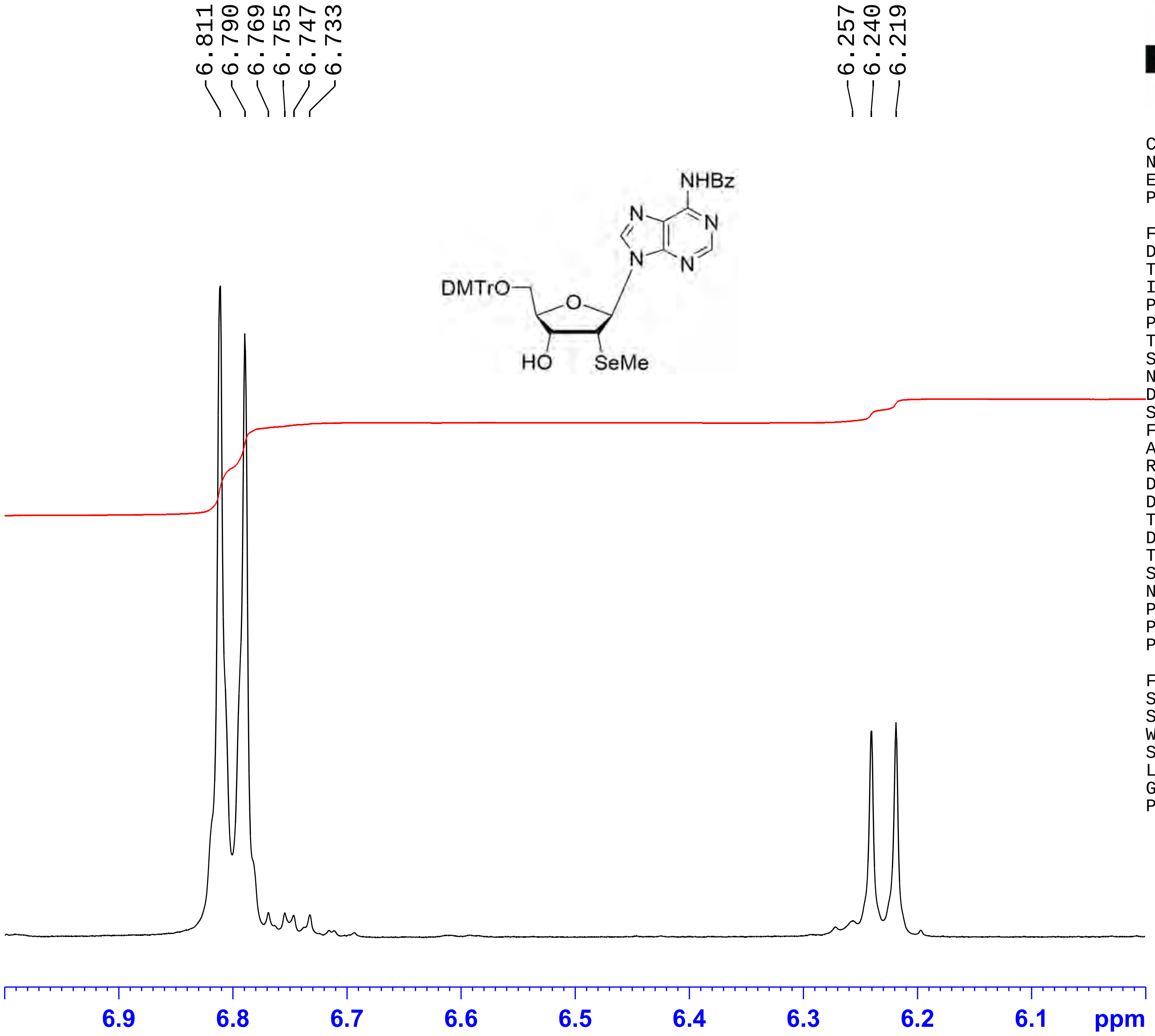

BRUKER

Current Data Parameters
NAME LH-II-72 OLD NMR
EXPNO 10
PROCNO 1

F2 - Acquisition Parameters
Date_ 20230625
Time 14.11 h
INSTRUM AVIII_400
PROBHD Z108618_0146 (
PULPROG zg30
TD 65536
SOLVENT CDCl3
NS 16
DS 2
SWH 8223.685 Hz
FIDRES 0.250967 Hz
AQ 3.9845889 sec
RG 161
DW 60.800 usec
DE 17.42 usec
TE 300.0 K
D1 1.00000000 sec
TD0 1
SFO1 400.1124708 MHz
NUC1 1H
P0 5.00 usec
P1 15.00 usec
PLW1 17.29199982 W

F2 - Processing parameters
SI 32768
SF 400.1100086 MHz
WDW EM
SSB 0
LB 0.30 Hz
GB 0
PC 1.00

Expanded region of the ^{1}H NMR spectrum of compound 18

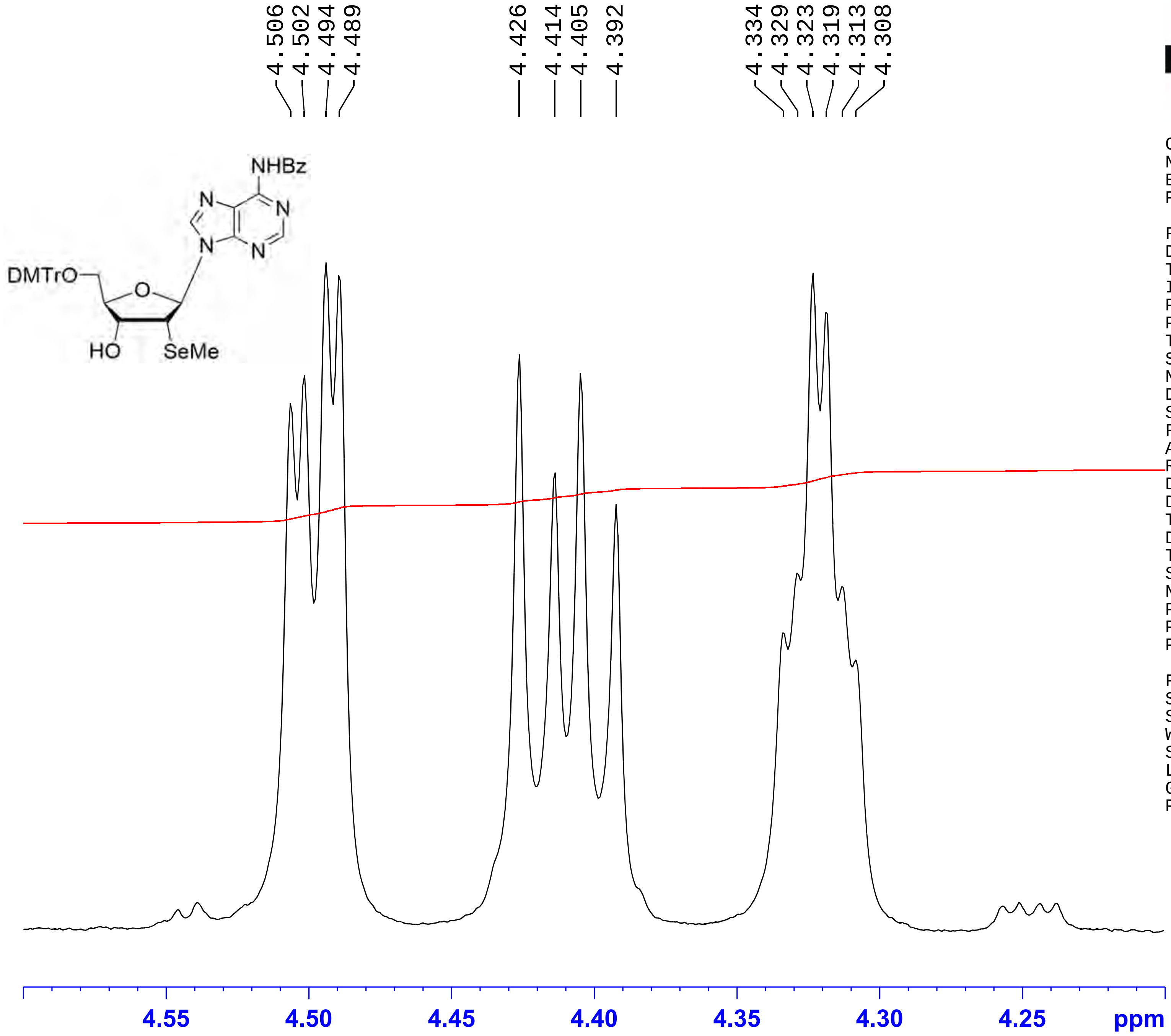

BRUKER

```
Current Data Parameters
NAME      LH-II-72 OLD NMR
EXPNO                   10
PROCNO                   1

F2 - Acquisition Parameters
Date_             20230625
Time                 14.11 h
INSTRUM           AVIII_400
PROBHD    Z108618_0146 (
PULPROG               zg30
TD                   65536
SOLVENT              CDCl3
NS                      16
DS                       2
SWH               8223.685 Hz
FIDRES            0.250967 Hz
AQ               3.9845889 sec
RG                     161
DW                  60.800 usec
DE                   17.42 usec
TE                   300.0 K
D1              1.00000000 sec
TD0                      1
SFO1           400.1124708 MHz
NUC1                    1H
P0                    5.00 usec
P1                   15.00 usec
PLW1           17.29199982 W

F2 - Processing parameters
SI                   32768
SF             400.1100086 MHz
WDW                     EM
SSB       0
LB                    0.30 Hz
GB        0
PC                    1.00
```

Expanded region of the ^{1}H NMR spectrum of compound 18

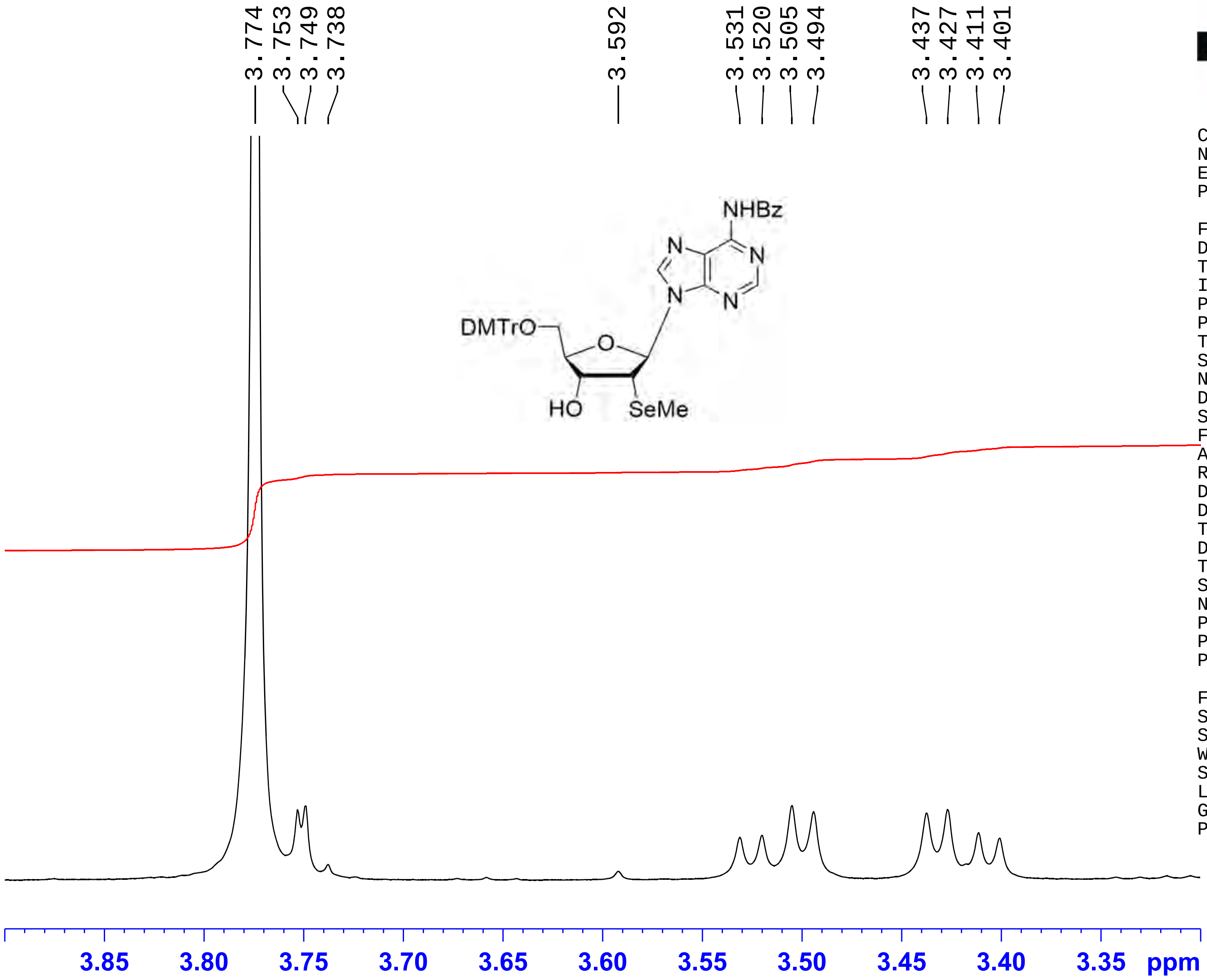


```
Current Data Parameters
NAME       LH-II-72 OLD NMR
EXPNO                    10
PROCNO                    1

F2 - Acquisition Parameters
Date_              20230625
Time                  14.11 h
INSTRUM            AVIII_400
PROBHD     Z108618_0146 (
PULPROG                zg30
TD                    65536
SOLVENT               CDCl3
NS                       16
DS                        2
SWH                8223.685 Hz
FIDRES             0.250967 Hz
AQ                3.9845889 sec
RG                      161
DW                   60.800 usec
DE                    17.42 usec
TE                    300.0 K
D1               1.00000000 sec
TD0                       1
SFO1            400.1124708 MHz
NUC1                     1H
P0                     5.00 usec
P1                    15.00 usec
PLW1            17.29199982 W

F2 - Processing parameters
SI                    32768
SF              400.1100086 MHz
WDW                      EM
SSB        0
LB                     0.30 Hz
GB         0
PC                     1.00
```

Expanded region of the ^{1}H NMR spectrum of compound 18

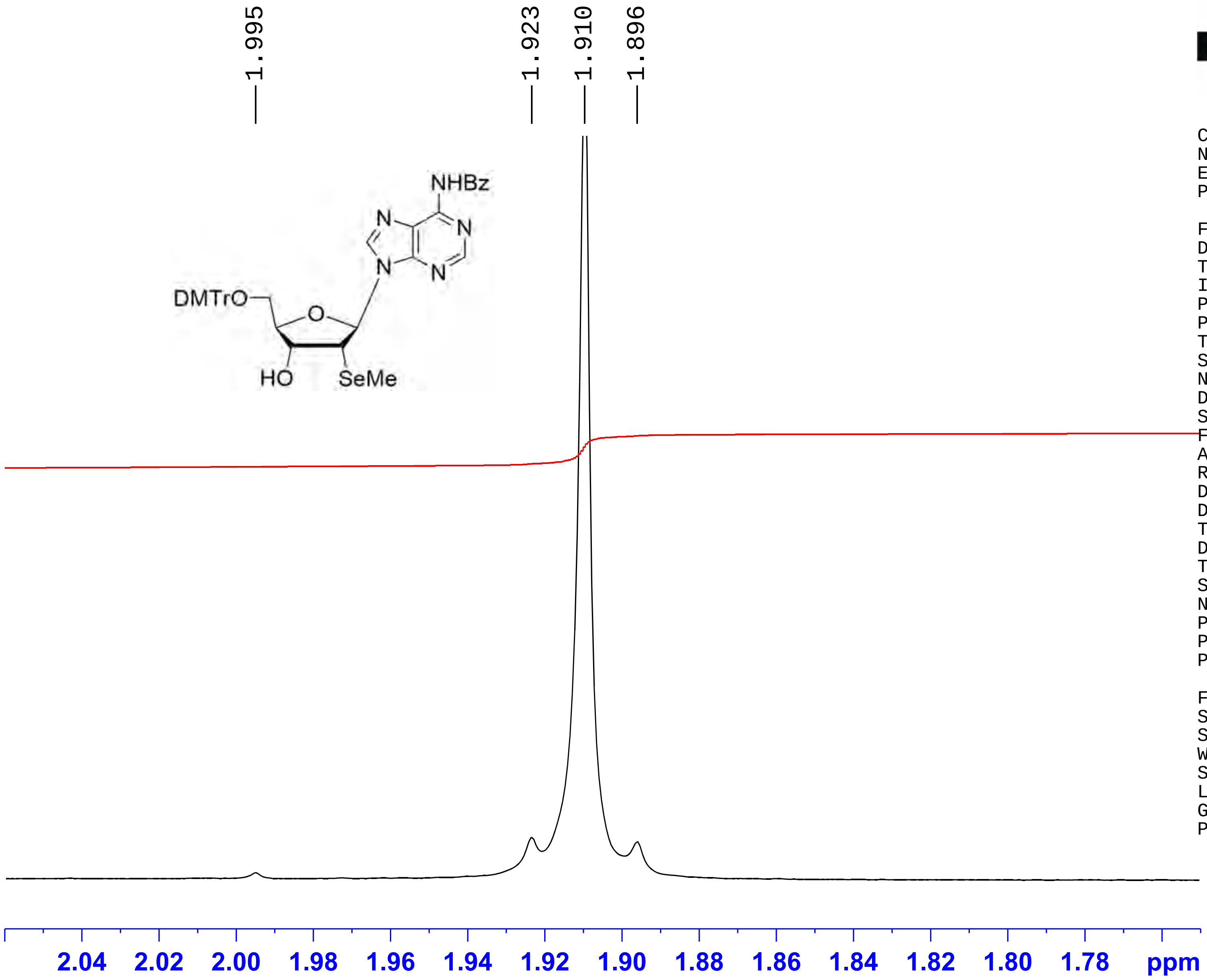

Current Data Parameters
NAME LH-II-72 OLD NMR
EXPNO 10
PROCNO 1

F2 - Acquisition Parameters
Date_ 20230625
Time 14.11 h
INSTRUM AVIII_400
PROBHD Z108618_0146 (
PULPROG zg30
TD 65536
SOLVENT CDCl3
NS 16
DS 2
SWH 8223.685 Hz
FIDRES 0.250967 Hz
AQ 3.9845889 sec
RG 161
DW 60.800 usec
DE 17.42 usec
TE 300.0 K
D1 1.00000000 sec
TD0 1
SFO1 400.1124708 MHz
NUC1 1H
P0 5.00 usec
P1 15.00 usec
PLW1 17.29199982 W

F2 - Processing parameters
SI 32768
SF 400.1100086 MHz
WDW EM
SSB 0
LB 0.30 Hz
GB 0
PC 1.00

^{13}C NMR spectrum of compound 18

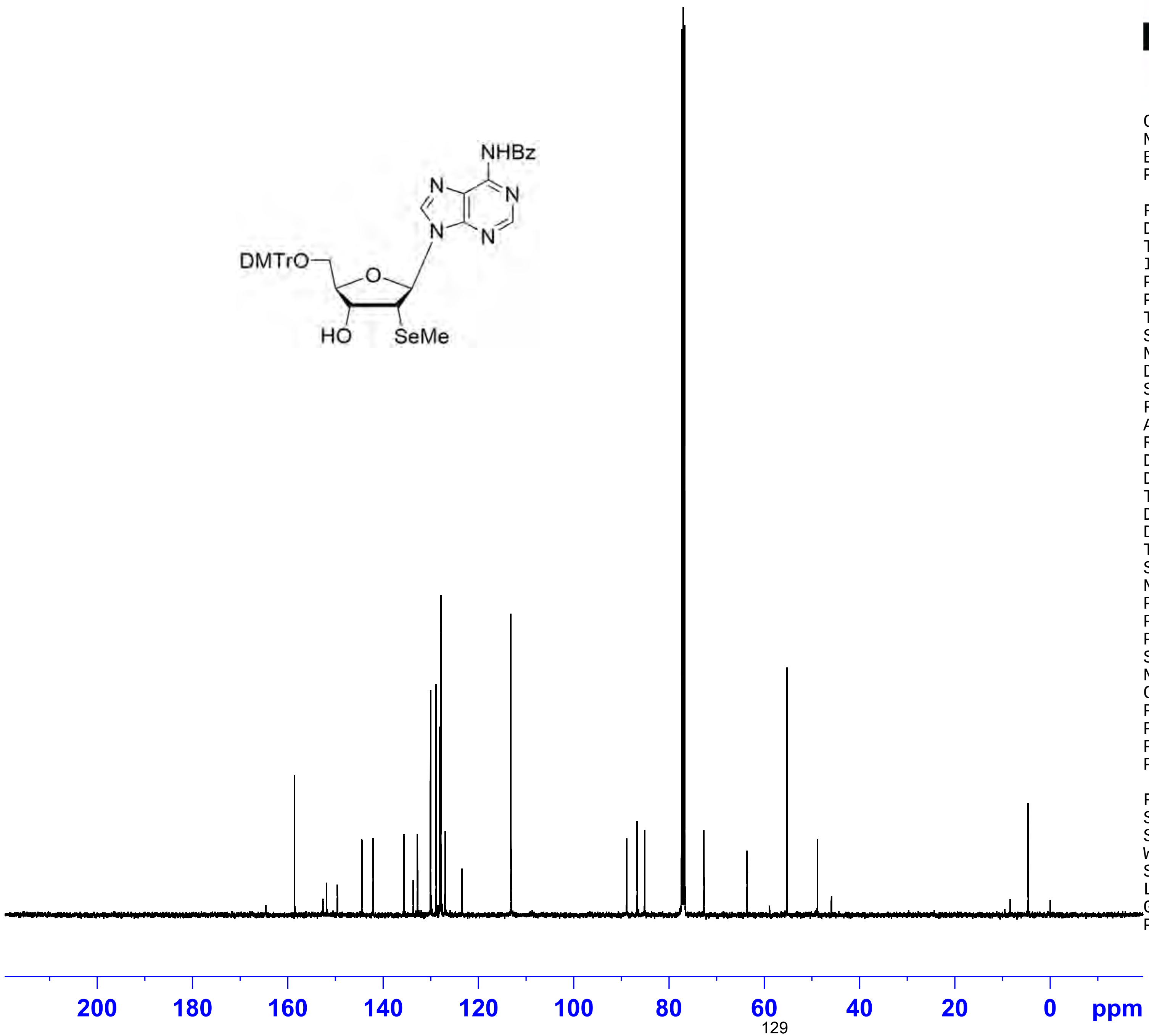

Current Data Parameters
NAME LH-II-72 OLD NMR
EXPNO 11
PROCNO 1

F2 - Acquisition Parameters
Date_ 20230625
Time 16.46 h
INSTRUM AVIII_400
PROBHD Z108618_0146 (
PULPROG zgpg30
TD 96150
SOLVENT CDCl3
NS 3000
DS 4
SWH 24038.461 Hz
FIDRES 0.500020 Hz
AQ 1.9999200 sec
RG 2050
DW 20.800 usec
DE 6.50 usec
TE 300.0 K
D1 1.00000000 sec
D11 0.03000000 sec
TD0 1
SFO1 100.6178003 MHz
NUC1 13C
P0 2.90 usec
P1 8.70 usec
PLW1 96.68000031 W
SFO2 400.1116004 MHz
NUC2 1H
CPDPRG[2 waltz64
PCPD2 90.00 usec
PLW2 17.29199982 W
PLW12 0.48032999 W
PLW13 0.24160001 W

F2 - Processing parameters
SI 131072
SF 100.6077448 MHz
WDW EM
SSB 0
LB 1.00 Hz
GB 0
PC 1.40

Expanded region of the ^{13}C NMR spectrum of compound 18

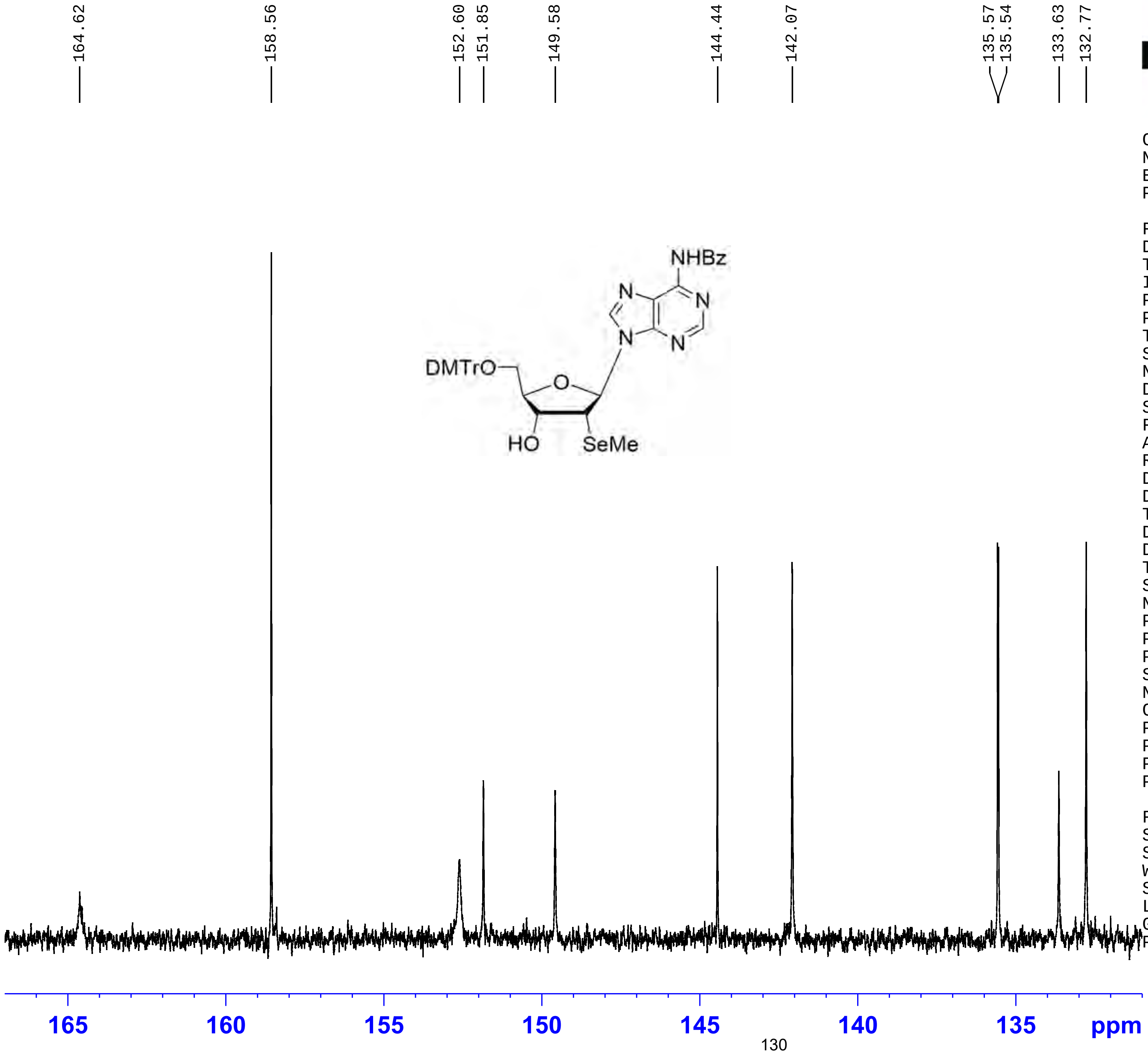


```
Current Data Parameters
NAME       LH-II-72 OLD NMR
EXPNO                    11
PROCNO                    1

F2 - Acquisition Parameters
Date_              20230625
Time                  16.46 h
INSTRUM            AVIII_400
PROBHD     Z108618_0146 (
PULPROG              zgpg30
TD                    96150
SOLVENT               CDCl3
NS                     3000
DS                        4
SWH               24038.461 Hz
FIDRES             0.500020 Hz
AQ                1.9999200 sec
RG                     2050
DW                   20.800 usec
DE                     6.50 usec
TE                    300.0 K
D1               1.00000000 sec
D11              0.03000000 sec
TD0                       1
SFO1            100.6178003 MHz
NUC1                    13C
P0                     2.90 usec
P1                     8.70 usec
PLW1            96.68000031 W
SFO2            400.1116004 MHz
NUC2                     1H
CPDPRG[2            waltz64
PCPD2                 90.00 usec
PLW2            17.29199982 W
PLW12            0.48032999 W
PLW13            0.24160001 W

F2 - Processing parameters
SI                   131072
SF              100.6077448 MHz
WDW                      EM
SSB        0
LB                     1.00 Hz
GB         0
PC                     1.40
```

Expanded region of the ^{13}C NMR spectrum of compound 18

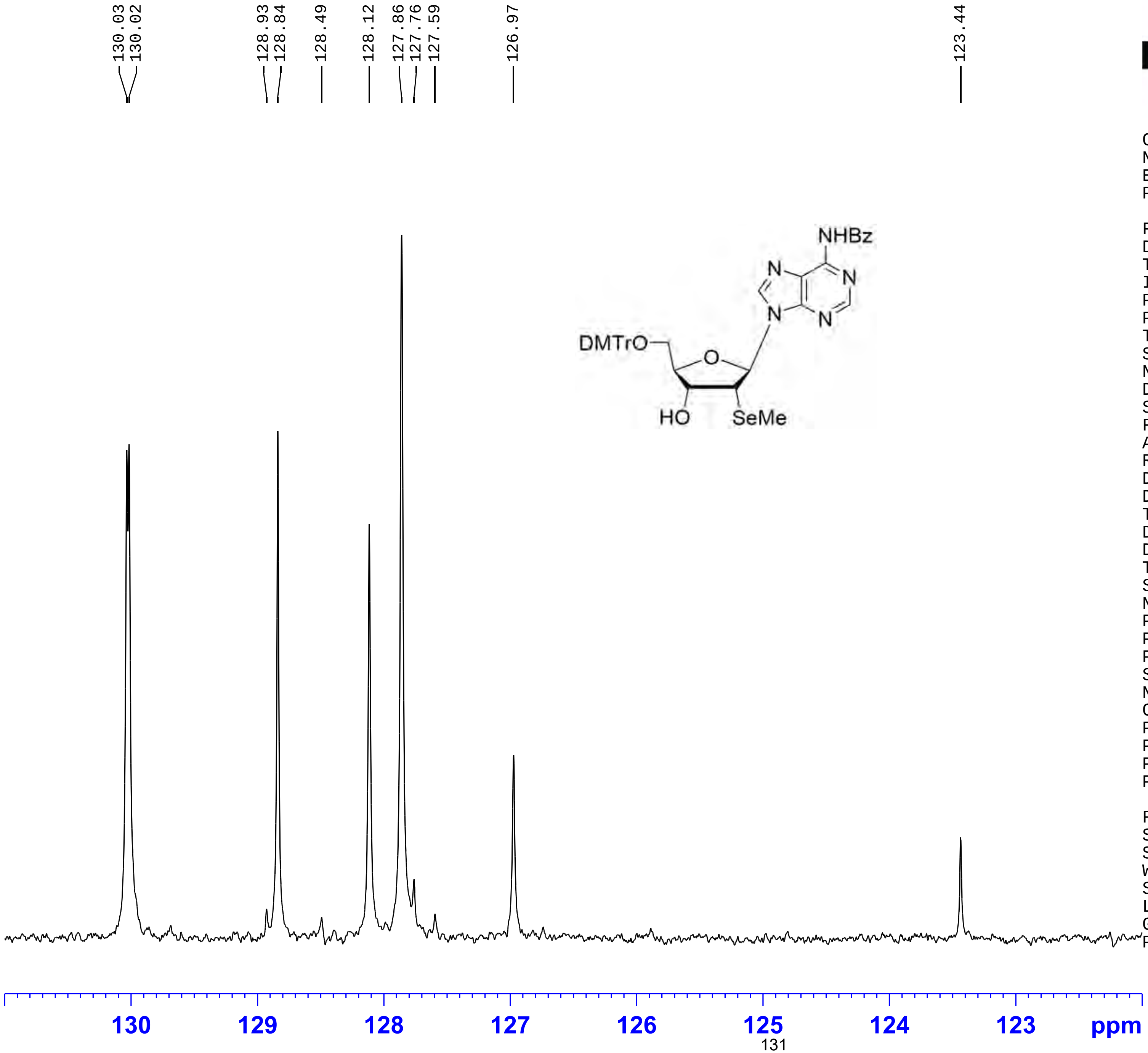

Current Data Parameters
NAME LH-II-72 OLD NMR
EXPNO 11
PROCNO 1

F2 - Acquisition Parameters
Date_ 20230625
Time 16.46 h
INSTRUM AVIII_400
PROBHD Z108618_0146 (
PULPROG zgpg30
TD 96150
SOLVENT CDCl3
NS 3000
DS 4
SWH 24038.461 Hz
FIDRES 0.500020 Hz
AQ 1.9999200 sec
RG 2050
DW 20.800 usec
DE 6.50 usec
TE 300.0 K
D1 1.00000000 sec
D11 0.03000000 sec
TD0 1
SFO1 100.6178003 MHz
NUC1 13C
P0 2.90 usec
P1 8.70 usec
PLW1 96.68000031 W
SFO2 400.1116004 MHz
NUC2 1H
CPDPRG[2 waltz64
PCPD2 90.00 usec
PLW2 17.29199982 W
PLW12 0.48032999 W
PLW13 0.24160001 W

F2 - Processing parameters
SI 131072
SF 100.6077448 MHz
WDW EM
SSB 0
LB 1.00 Hz
GB 0
PC 1.40

Expanded region of the ^{13}C NMR spectrum of compound 18

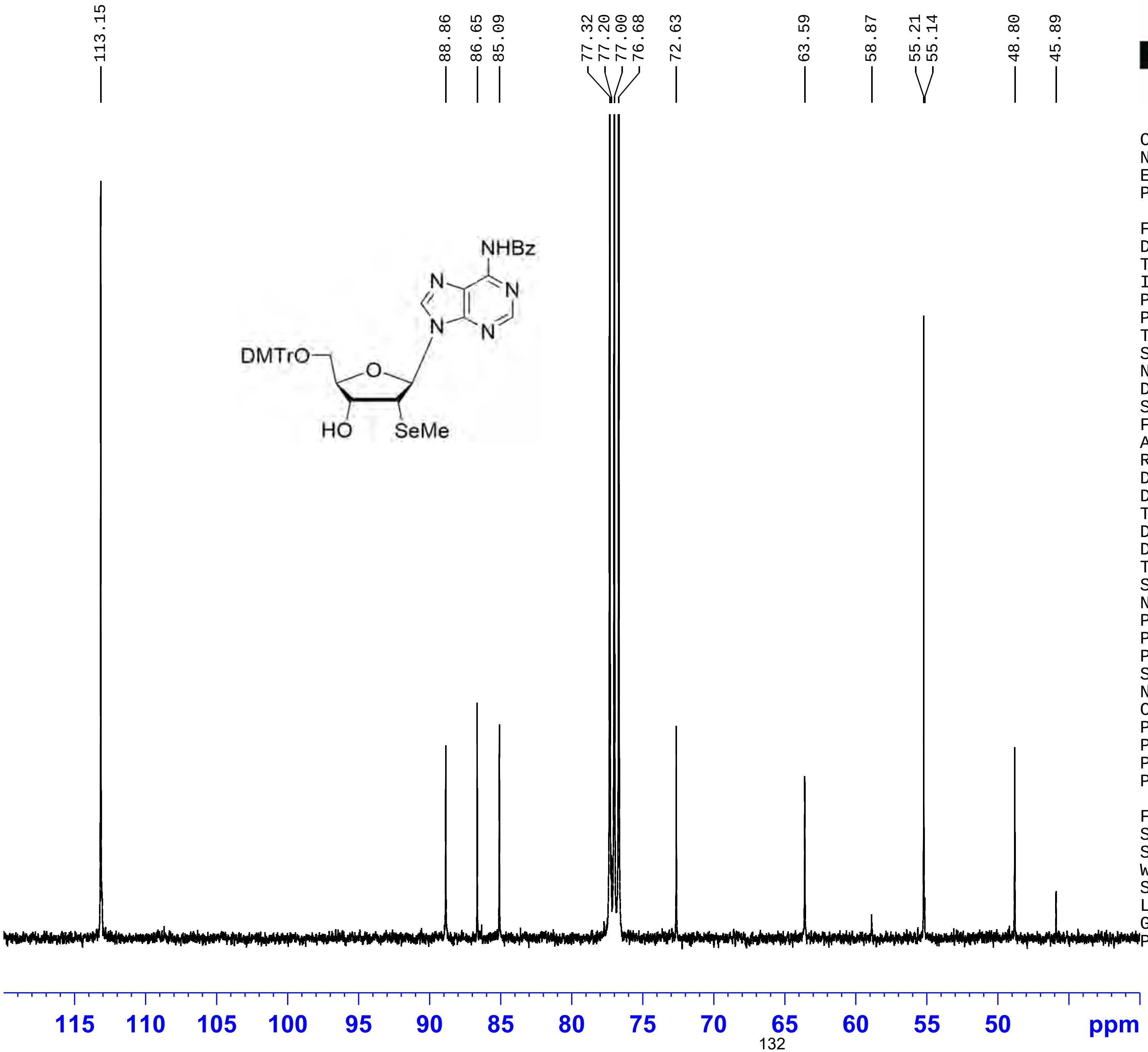

BRUKER

Current Data Parameters
NAME LH-II-72 OLD NMR
EXPNO 11
PROCNO 1

F2 - Acquisition Parameters
Date_ 20230625
Time 16.46 h
INSTRUM AVIII_400
PROBHD Z108618_0146 (
PULPROG zgpg30
TD 96150
SOLVENT CDCl3
NS 3000
DS 4
SWH 24038.461 Hz
FIDRES 0.500020 Hz
AQ 1.9999200 sec
RG 2050
DW 20.800 usec
DE 6.50 usec
TE 300.0 K
D1 1.00000000 sec
D11 0.03000000 sec
TD0 1
SFO1 100.6178003 MHz
NUC1 13C
P0 2.90 usec
P1 8.70 usec
PLW1 96.68000031 W
SFO2 400.1116004 MHz
NUC2 1H
CPDPRG[2 waltz64
PCPD2 90.00 usec
PLW2 17.29199982 W
PLW12 0.48032999 W
PLW13 0.24160001 W

F2 - Processing parameters
SI 131072
SF 100.6077448 MHz
WDW EM
SSB 0
LB 1.00 Hz
GB 0
PC 1.40

Expanded region of the ^{13}C NMR spectrum of compound 18

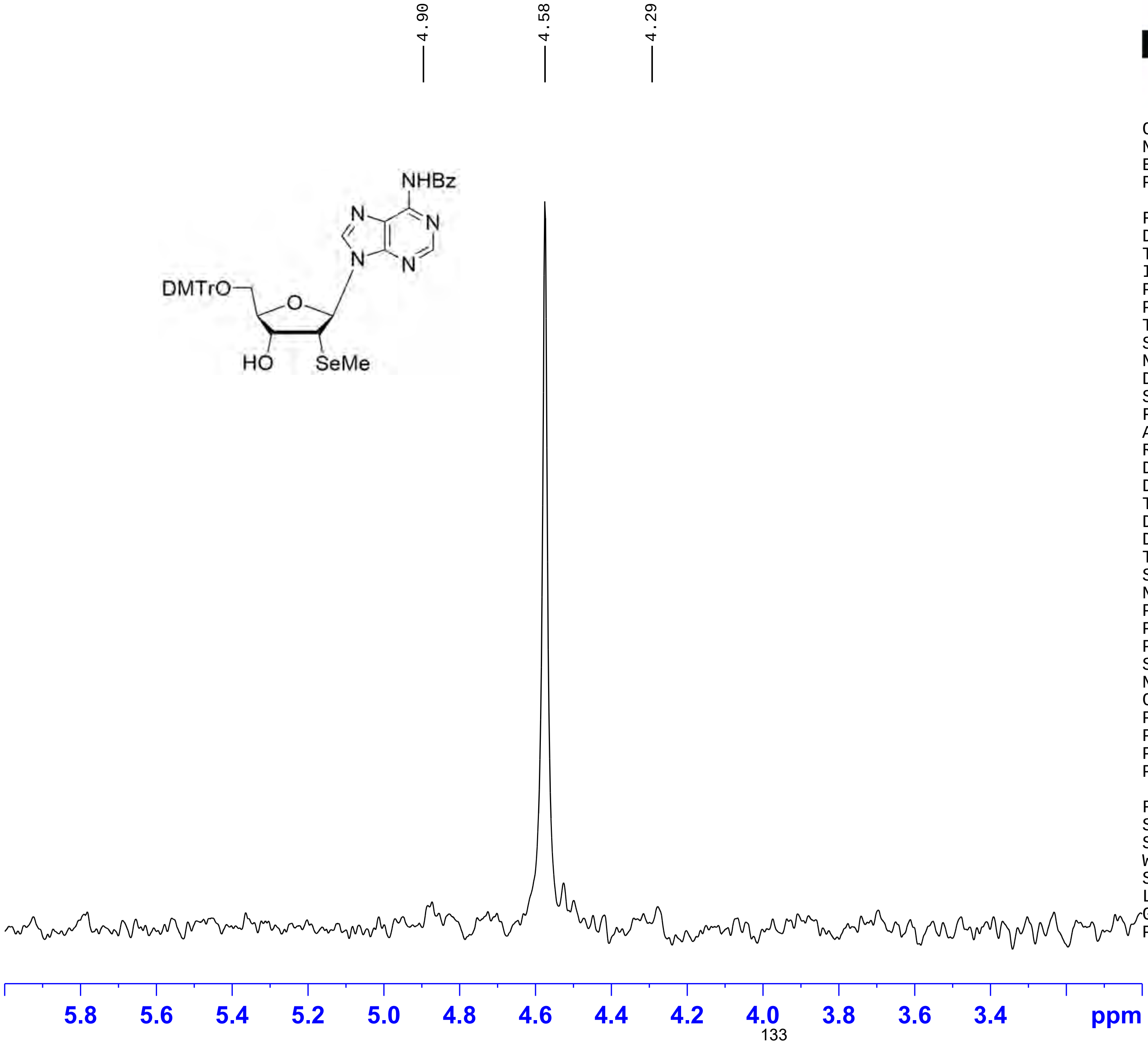


```
Current Data Parameters
NAME      LH-II-72 OLD NMR
EXPNO                   11
PROCNO                   1

F2 - Acquisition Parameters
Date_             20230625
Time                 16.46 h
INSTRUM          AVIII_400
PROBHD   Z108618_0146 (
PULPROG            zgpg30
TD                   96150
SOLVENT              CDCl3
NS                    3000
DS                       4
SWH              24038.461 Hz
FIDRES            0.500020 Hz
AQ               1.9999200 sec
RG                    2050
DW                  20.800 usec
DE                    6.50 usec
TE                   300.0 K
D1              1.00000000 sec
D11             0.03000000 sec
TD0                      1
SFO1           100.6178003 MHz
NUC1                   13C
P0                    2.90 usec
P1                    8.70 usec
PLW1           96.68000031 W
SFO2           400.1116004 MHz
NUC2                    1H
CPDPRG[2           waltz64
PCPD2                90.00 usec
PLW2           17.29199982 W
PLW12           0.48032999 W
PLW13           0.24160001 W

F2 - Processing parameters
SI                  131072
SF             100.6077448 MHz
WDW                     EM
SSB      0
LB                    1.00 Hz
GB       0
PC                    1.40
```

^{13}C DEPT-135 NMR spectrum of compound 18

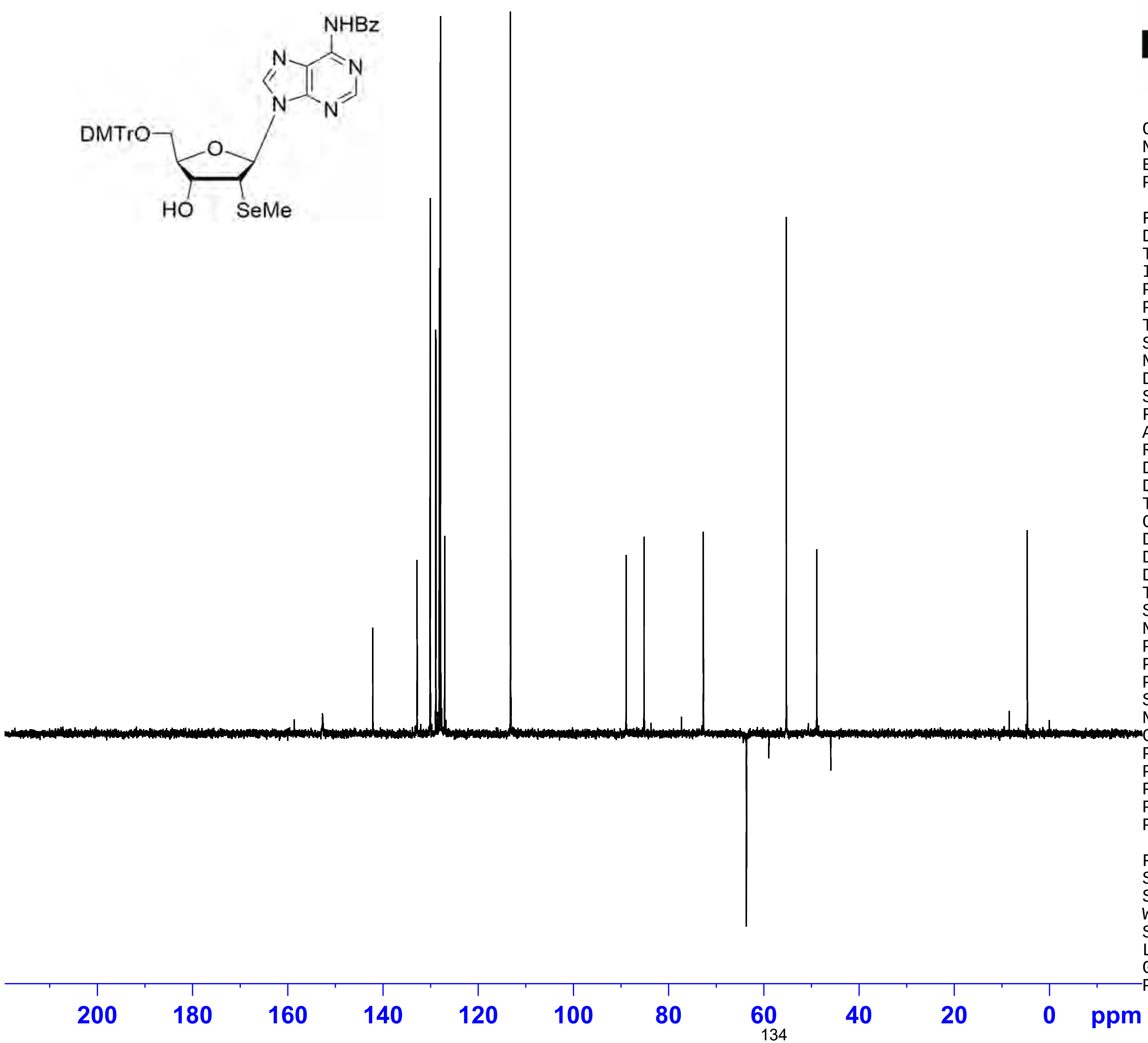

Current Data Parameters
NAME LH-II-72 OLD NMR
EXPNO 12
PROCNO 1

F2 - Acquisition Parameters
Date_ 20230625
Time 18.30 h
INSTRUM AVIII_400
PROBHD Z108618_0146 (
PULPROG dept135
TD 65536
SOLVENT CDCl3
NS 1500
DS 4
SWH 24038.461 Hz
FIDRES 0.733596 Hz
AQ 1.3631488 sec
RG 2050
DW 20.800 usec
DE 6.50 usec
TE 300.0 K
CNST2 145.0000000
D1 2.00000000 sec
D2 0.00344828 sec
D12 0.00002000 sec
TD0 1
SFO1 100.6178003 MHz
NUC1 13C
P1 8.70 usec
P2 17.40 usec
PLW1 96.68000031 W
SFO2 400.1116004 MHz
NUC2 1H
CPDPRG[2 waltz64
P3 15.00 usec
P4 30.00 usec
PCPD2 90.00 usec
PLW2 17.29199982 W
PLW12 0.48032999 W

F2 - Processing parameters
SI 32768
SF 100.6077400 MHz
WDW EM
SSB 0
LB 1.00 Hz
GB 0
PC 1.40

^{13}C DEPT-135 NMR spectrum of compound 18

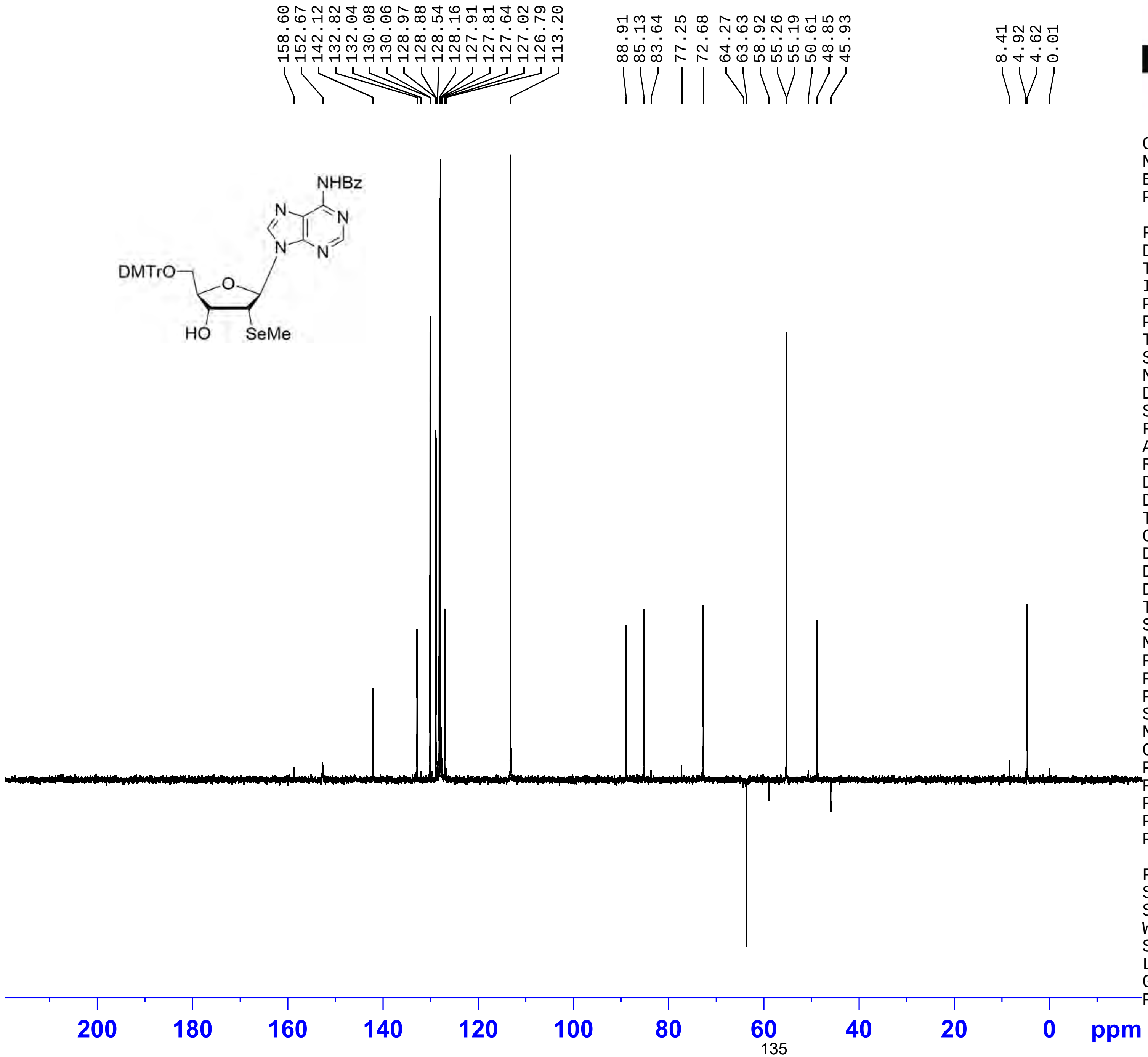


```
Current Data Parameters
NAME        LH-II-72 OLD NMR
EXPNO                     12
PROCNO                     1

F2 - Acquisition Parameters
Date_               20230625
Time                   18.30 h
INSTRUM             AVIII_400
PROBHD       Z108618_0146 (
PULPROG              dept135
TD                     65536
SOLVENT                CDCl3
NS                      1500
DS                         4
SWH                24038.461 Hz
FIDRES              0.733596 Hz
AQ                 1.3631488 sec
RG                      2050
DW                    20.800 usec
DE                      6.50 usec
TE                     300.0 K
CNST2            145.0000000
D1                2.00000000 sec
D2                0.00344828 sec
D12               0.00002000 sec
TD0                        1
SFO1             100.6178003 MHz
NUC1                     13C
P1                      8.70 usec
P2                     17.40 usec
PLW1             96.68000031 W
SFO2             400.1116004 MHz
NUC2                      1H
CPDPRG[2             waltz64
P3                     15.00 usec
P4                     30.00 usec
PCPD2                  90.00 usec
PLW2             17.29199982 W
PLW12             0.48032999 W

F2 - Processing parameters
SI                     32768
SF               100.6077400 MHz
WDW                       EM
SSB       0
LB                      1.00 Hz
GB        0
PC                      1.40
```

Expanded region of the ^{13}C DEPT-135 NMR spectrum of compound 18

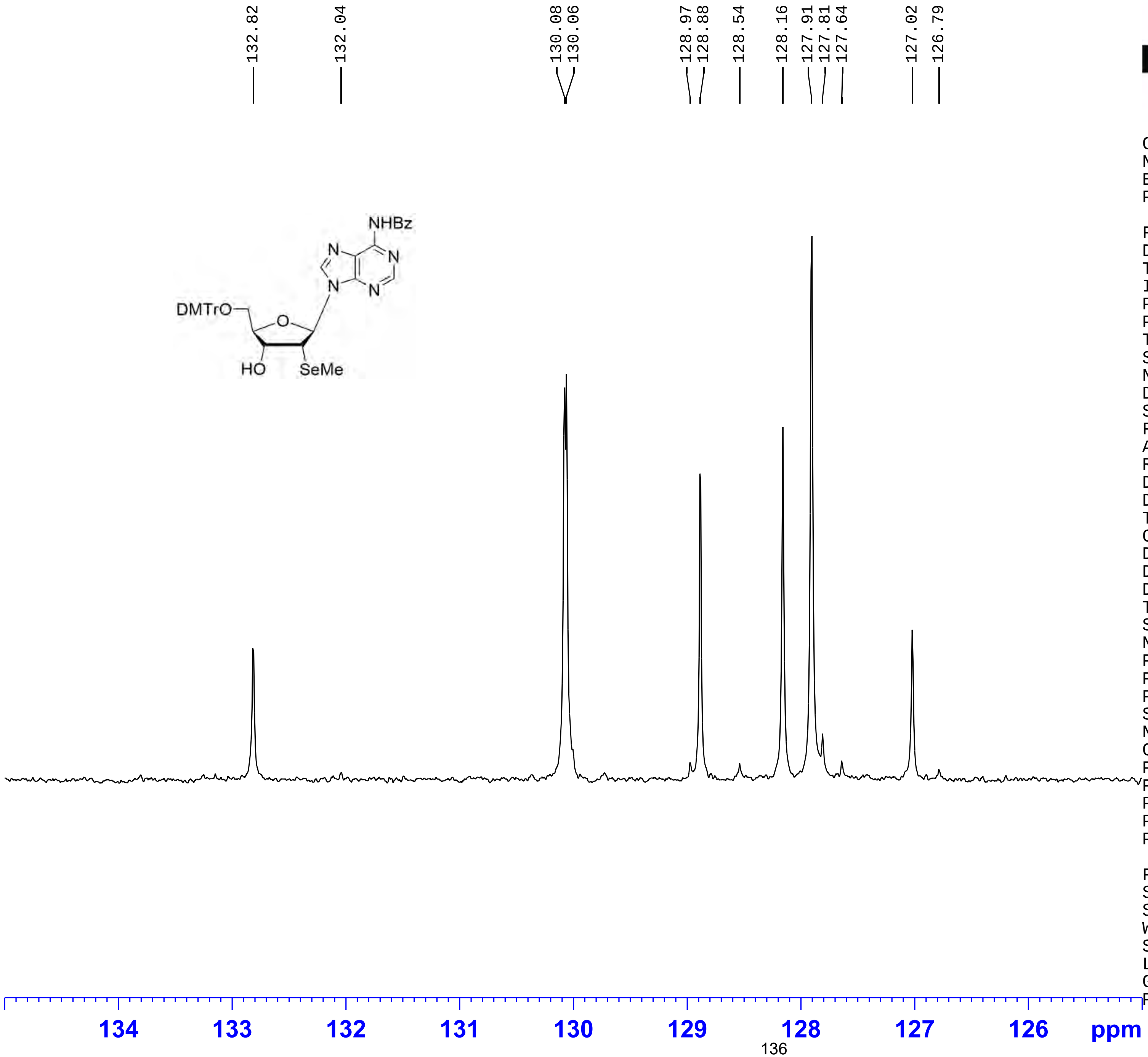


```
Current Data Parameters
NAME      LH-II-72 OLD NMR
EXPNO                   12
PROCNO                   1

F2 - Acquisition Parameters
Date_             20230625
Time                 18.30 h
INSTRUM          AVIII_400
PROBHD     Z108618_0146 (
PULPROG            dept135
TD                   65536
SOLVENT              CDCl3
NS                    1500
DS                       4
SWH              24038.461 Hz
FIDRES            0.733596 Hz
AQ               1.3631488 sec
RG                    2050
DW                  20.800 usec
DE                    6.50 usec
TE                   300.0 K
CNST2         145.0000000
D1             2.00000000 sec
D2             0.00344828 sec
D12            0.00002000 sec
TD0                      1
SFO1          100.6178003 MHz
NUC1                   13C
P1                    8.70 usec
P2                   17.40 usec
PLW1          96.68000031 W
SFO2          400.1116004 MHz
NUC2                    1H
CPDPRG[2           waltz64
P3                   15.00 usec
P4                   30.00 usec
PCPD2                90.00 usec
PLW2          17.29199982 W
PLW12          0.48032999 W

F2 - Processing parameters
SI                   32768
SF            100.6077400 MHz
WDW                     EM
SSB      0
LB                    1.00 Hz
GB       0
PC                    1.40
```

Expanded region of the ^{13}C DEPT-135 NMR spectrum of compound 18

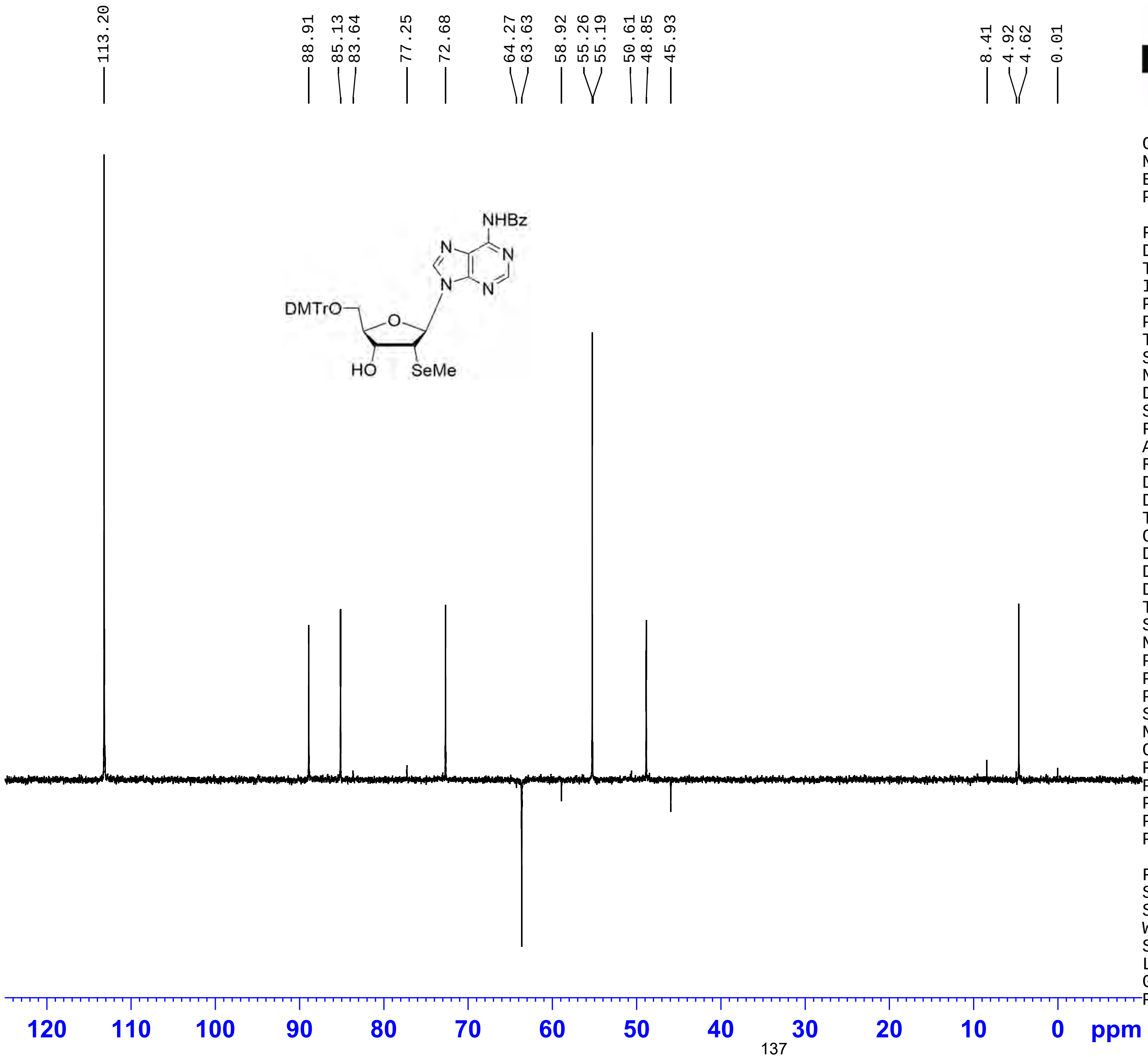

Current Data Parameters
NAME LH-II-72 OLD NMR
EXPNO 12
PROCNO 1

F2 - Acquisition Parameters
Date_ 20230625
Time 18.30 h
INSTRUM AVIII_400
PROBHD Z108618_0146 (
PULPROG dept135
TD 65536
SOLVENT CDCl3
NS 1500
DS 4
SWH 24038.461 Hz
FIDRES 0.733596 Hz
AQ 1.3631488 sec
RG 2050
DW 20.800 usec
DE 6.50 usec
TE 300.0 K
CNST2 145.0000000
D1 2.00000000 sec
D2 0.00344828 sec
D12 0.00002000 sec
TD0 1
SFO1 100.6178003 MHz
NUC1 13C
P1 8.70 usec
P2 17.40 usec
PLW1 96.68000031 W
SFO2 400.1116004 MHz
NUC2 1H
CPDPRG[2 waltz64
P3 15.00 usec
P4 30.00 usec
PCPD2 90.00 usec
PLW2 17.29199982 W
PLW12 0.48032999 W

F2 - Processing parameters
SI 32768
SF 100.6077400 MHz
WDW EM
SSB 0
LB 1.00 Hz
GB 0
PC 1.40

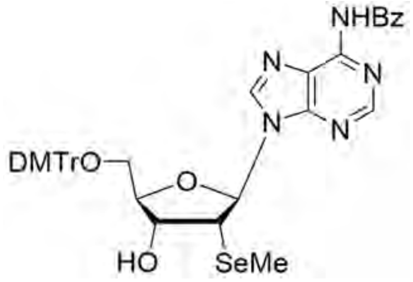

^{1}H-^{1}H COSY NMR spectrum of compound 18

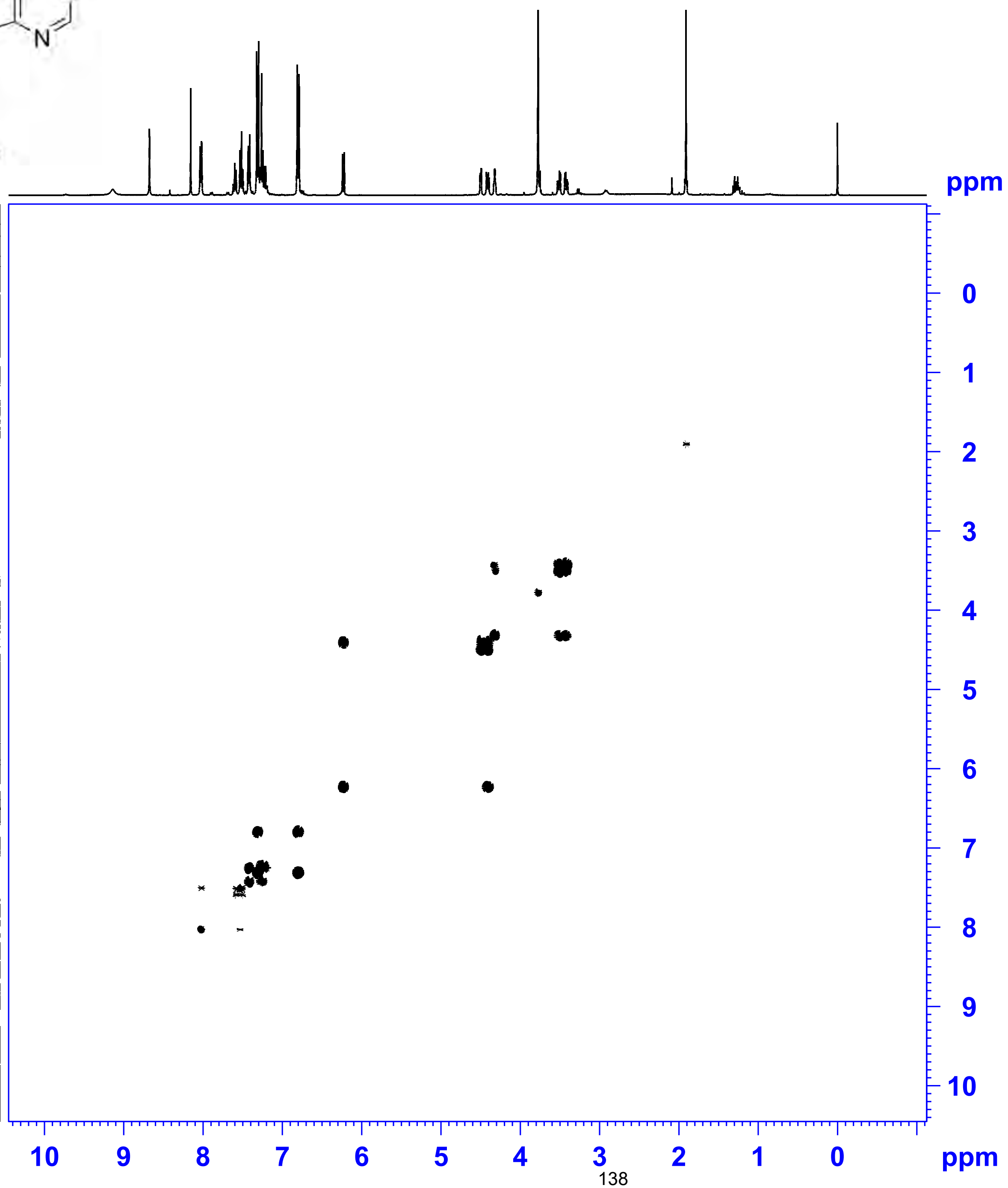


```
Current Data Parameters
NAME      LH-II-72 NEW NMR
EXPNO                   11
PROCNO                   1

F2 - Acquisition Parameters
Date_             20230625
Time                 20.32 h
INSTRUM          AVIII_400
PROBHD     Z108618_0817 (
PULPROG          cosygpmfqf
TD                    2048
SOLVENT              CDCl3
NS                       3
DS                       8
SWH               4629.629 Hz
FIDRES            4.521122 Hz
AQ               0.2211840 sec
RG                    1620
DW                 108.000 usec
DE                    6.50 usec
TE                   294.7 K
D0              0.00000300 sec
D1              1.97077799 sec
D13             0.00000400 sec
D16             0.00020000 sec
IN0             0.00021600 sec
TDav                     1
SFO1           399.9118735 MHz
NUC1                    1H
P1                  500.00 usec
PLW1           31.62299919 W
GPNAM[1]          SINE.100
GPZ1                 16.00 %
GPNAM[2]          SINE.100
GPZ2                 12.00 %
GPNAM[3]          SINE.100
GPZ3                 40.00 %
P16                1000.00 usec

F1 - Acquisition parameters
TD                     256
SFO1              399.9119 MHz
FIDRES           36.168980 Hz
SW                  11.577 ppm
FnMODE                  QF

F2 - Processing parameters
SI                    1024
SF             399.9100084 MHz
WDW                   SINE
SSB        0
LB         0 Hz
GB         0
PC                    1.40

F1 - Processing parameters
SI                    1024
MC2                     QF
SF             399.9100084 MHz
WDW                   SINE
SSB        0
LB         0 Hz
GB         0
```

Expanded region of ^{1}H-^{1}H COSY NMR spectrum of compound 18

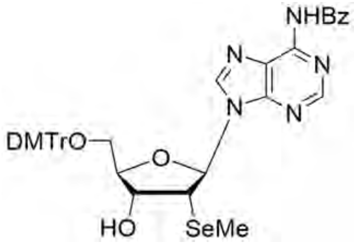

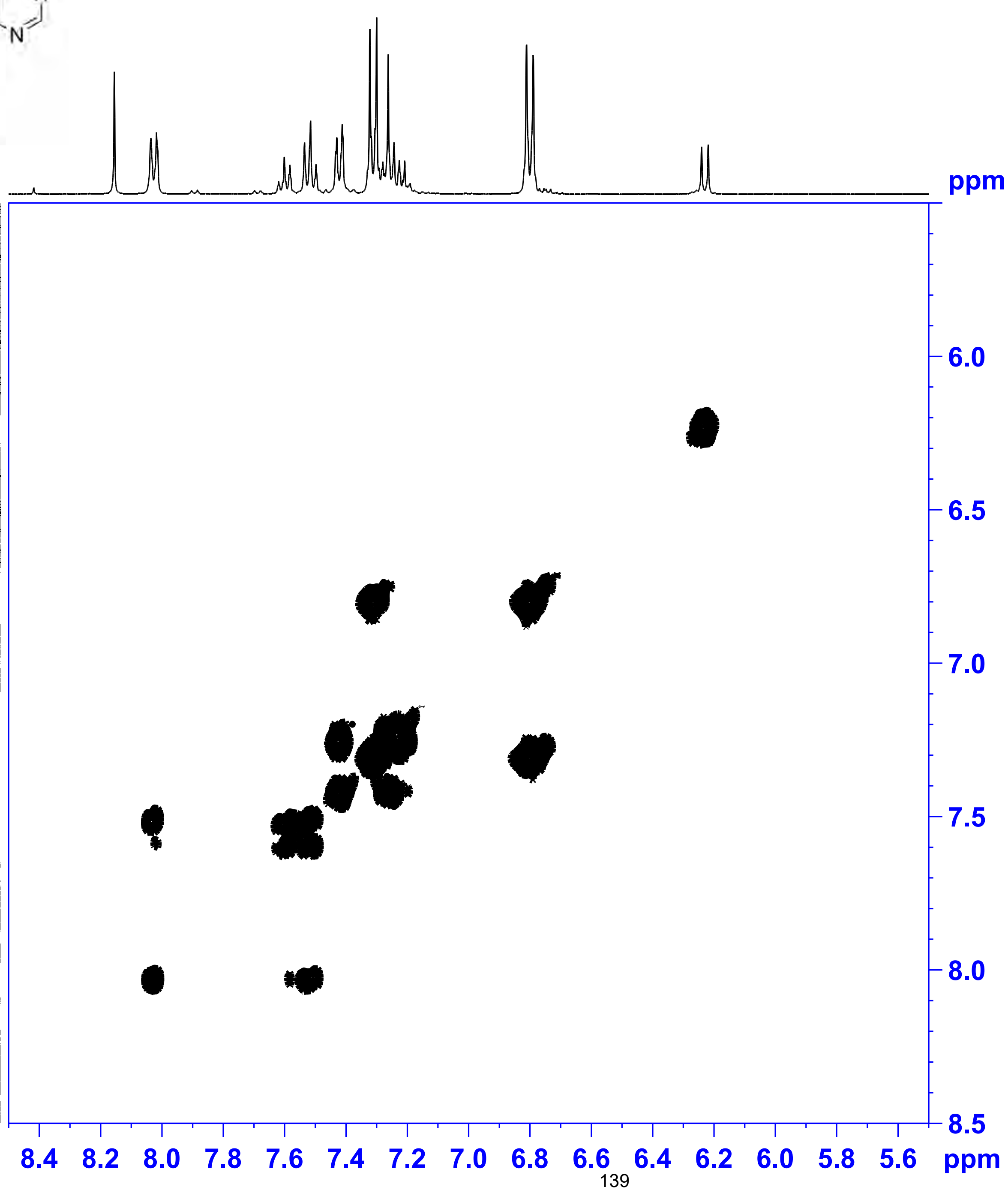


```
Current Data Parameters
NAME      LH-II-72 NEW NMR
EXPNO                   11
PROCNO                   1

F2 - Acquisition Parameters
Date_             20230625
Time                 20.32 h
INSTRUM          AVIII_400
PROBHD    Z108618_0817 (
PULPROG         cosygpmfqf
TD                    2048
SOLVENT              CDCl3
NS                       3
DS                       8
SWH               4629.629 Hz
FIDRES            4.521122 Hz
AQ               0.2211840 sec
RG                    1620
DW                 108.000 usec
DE                    6.50 usec
TE                   294.7 K
D0              0.00000300 sec
D1              1.97077799 sec
D13             0.00000400 sec
D16             0.00020000 sec
IN0             0.00021600 sec
TDav                     1
SFO1           399.9118735 MHz
NUC1                    1H
P1                  500.00 usec
PLW1           31.62299919 W
GPNAM[1]          SINE.100
GPZ1                 16.00 %
GPNAM[2]          SINE.100
GPZ2                 12.00 %
GPNAM[3]          SINE.100
GPZ3                 40.00 %
P16                1000.00 usec

F1 - Acquisition parameters
TD                     256
SFO1              399.9119 MHz
FIDRES           36.168980 Hz
SW                  11.577 ppm
FnMODE                  QF

F2 - Processing parameters
SI                    1024
SF             399.9100084 MHz
WDW                   SINE
SSB       0
LB        0 Hz
GB        0
PC                    1.40

F1 - Processing parameters
SI                    1024
MC2                     QF
SF             399.9100084 MHz
WDW                   SINE
SSB       0
LB        0 Hz
GB        0
```

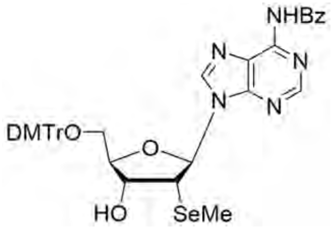

Expanded region of ^{1}H-^{1}H COSY NMR spectrum of compound 18

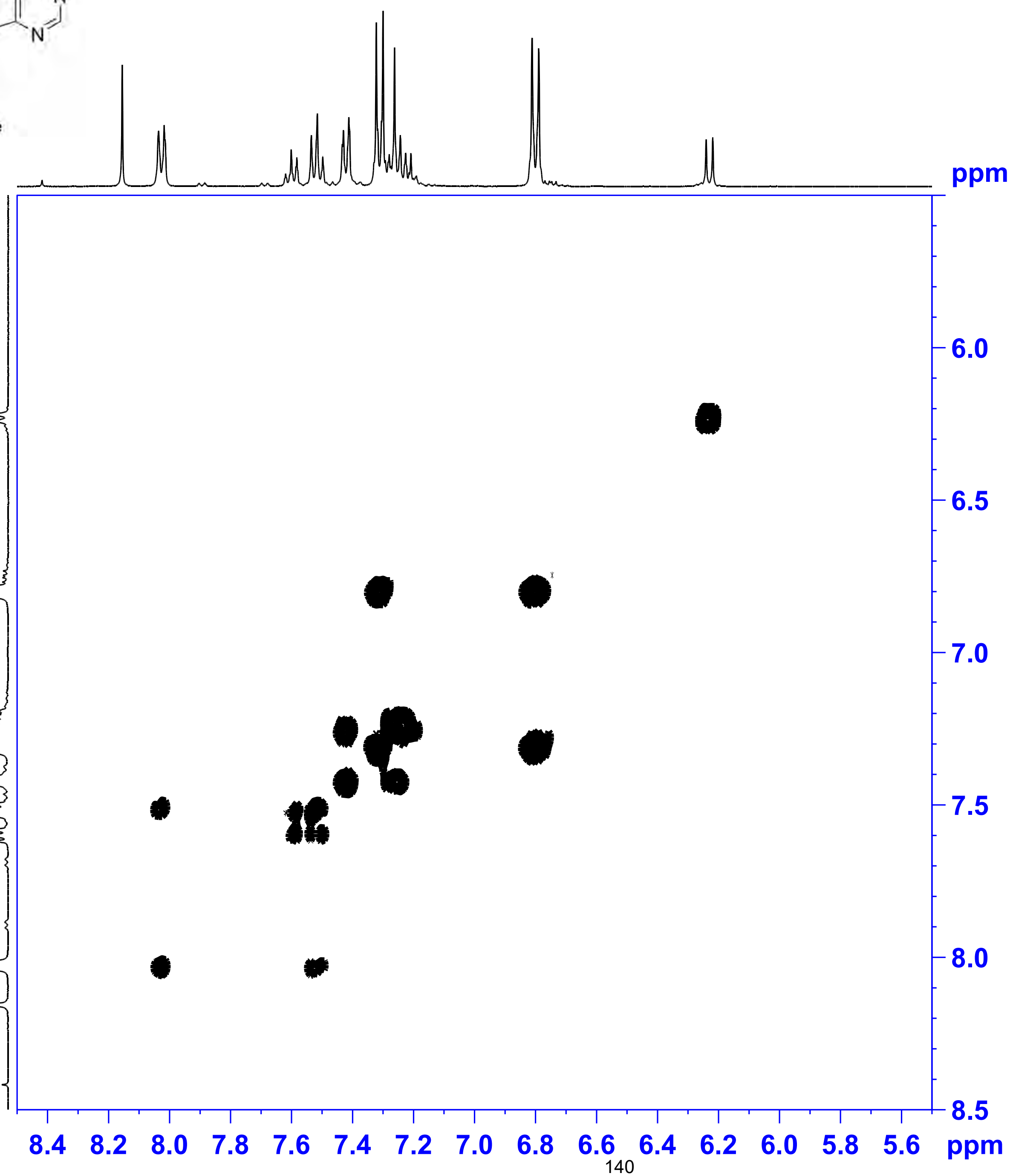


```
Current Data Parameters
NAME      LH-II-72 NEW NMR
EXPNO                   11
PROCNO                   1

F2 - Acquisition Parameters
Date_             20230625
Time                 20.32 h
INSTRUM           AVIII_400
PROBHD   Z108618_0817 (
PULPROG         cosygpmfqf
TD                    2048
SOLVENT              CDCl3
NS                       3
DS                       8
SWH               4629.629 Hz
FIDRES            4.521122 Hz
AQ               0.2211840 sec
RG                    1620
DW                 108.000 usec
DE                    6.50 usec
TE                   294.7 K
D0              0.00000300 sec
D1              1.97077799 sec
D13             0.00000400 sec
D16             0.00020000 sec
IN0             0.00021600 sec
TDav                     1
SFO1           399.9118735 MHz
NUC1                    1H
P1                  500.00 usec
PLW1           31.62299919 W
GPNAM[1]          SINE.100
GPZ1                 16.00 %
GPNAM[2]          SINE.100
GPZ2                 12.00 %
GPNAM[3]          SINE.100
GPZ3                 40.00 %
P16                1000.00 usec

F1 - Acquisition parameters
TD                     256
SFO1              399.9119 MHz
FIDRES           36.168980 Hz
SW                  11.577 ppm
FnMODE                  QF

F2 - Processing parameters
SI                    1024
SF             399.9100084 MHz
WDW                   SINE
SSB      0
LB       0 Hz
GB       0
PC                    1.40

F1 - Processing parameters
SI                    1024
MC2                     QF
SF             399.9100084 MHz
WDW                   SINE
SSB      0
LB       0 Hz
GB       0
```

Expanded region of ^{1}H-^{1}H COSY NMR spectrum of compound 18

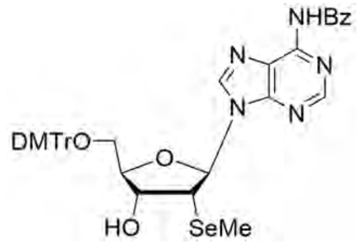

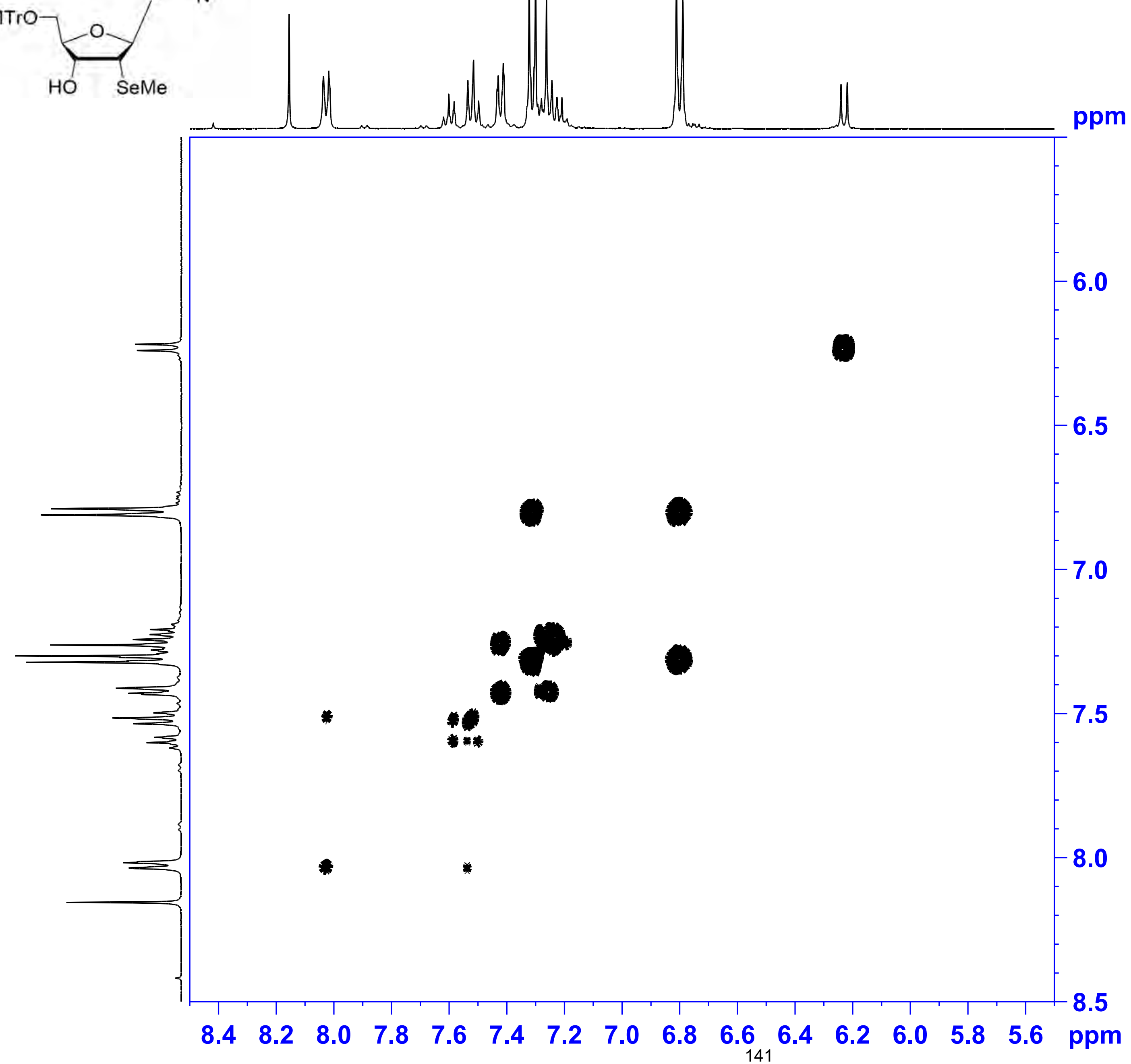


```
Current Data Parameters
NAME      LH-II-72 NEW NMR
EXPNO                   11
PROCNO                   1

F2 - Acquisition Parameters
Date_             20230625
Time                 20.32 h
INSTRUM          AVIII_400
PROBHD   Z108618_0817 (
PULPROG         cosygpmfqf
TD                    2048
SOLVENT              CDCl3
NS                       3
DS                       8
SWH               4629.629 Hz
FIDRES            4.521122 Hz
AQ               0.2211840 sec
RG                    1620
DW                 108.000 usec
DE                    6.50 usec
TE                   294.7 K
D0              0.00000300 sec
D1              1.97077799 sec
D13             0.00000400 sec
D16             0.00020000 sec
IN0             0.00021600 sec
TDav                     1
SFO1           399.9118735 MHz
NUC1                    1H
P1                  500.00 usec
PLW1           31.62299919 W
GPNAM[1]          SINE.100
GPZ1                 16.00 %
GPNAM[2]          SINE.100
GPZ2                 12.00 %
GPNAM[3]          SINE.100
GPZ3                 40.00 %
P16                1000.00 usec

F1 - Acquisition parameters
TD                     256
SFO1              399.9119 MHz
FIDRES           36.168980 Hz
SW                  11.577 ppm
FnMODE                  QF

F2 - Processing parameters
SI                    1024
SF             399.9100084 MHz
WDW                   SINE
SSB      0
LB       0 Hz
GB       0
PC                    1.40

F1 - Processing parameters
SI                    1024
MC2                     QF
SF             399.9100084 MHz
WDW                   SINE
SSB      0
LB       0 Hz
GB       0
```

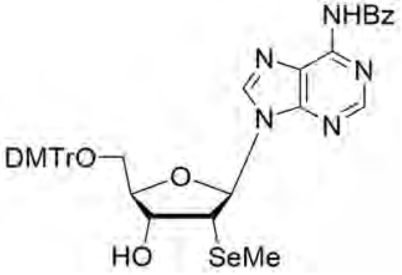

Expanded region of ¹H-¹H COSY NMR spectrum of compound 18

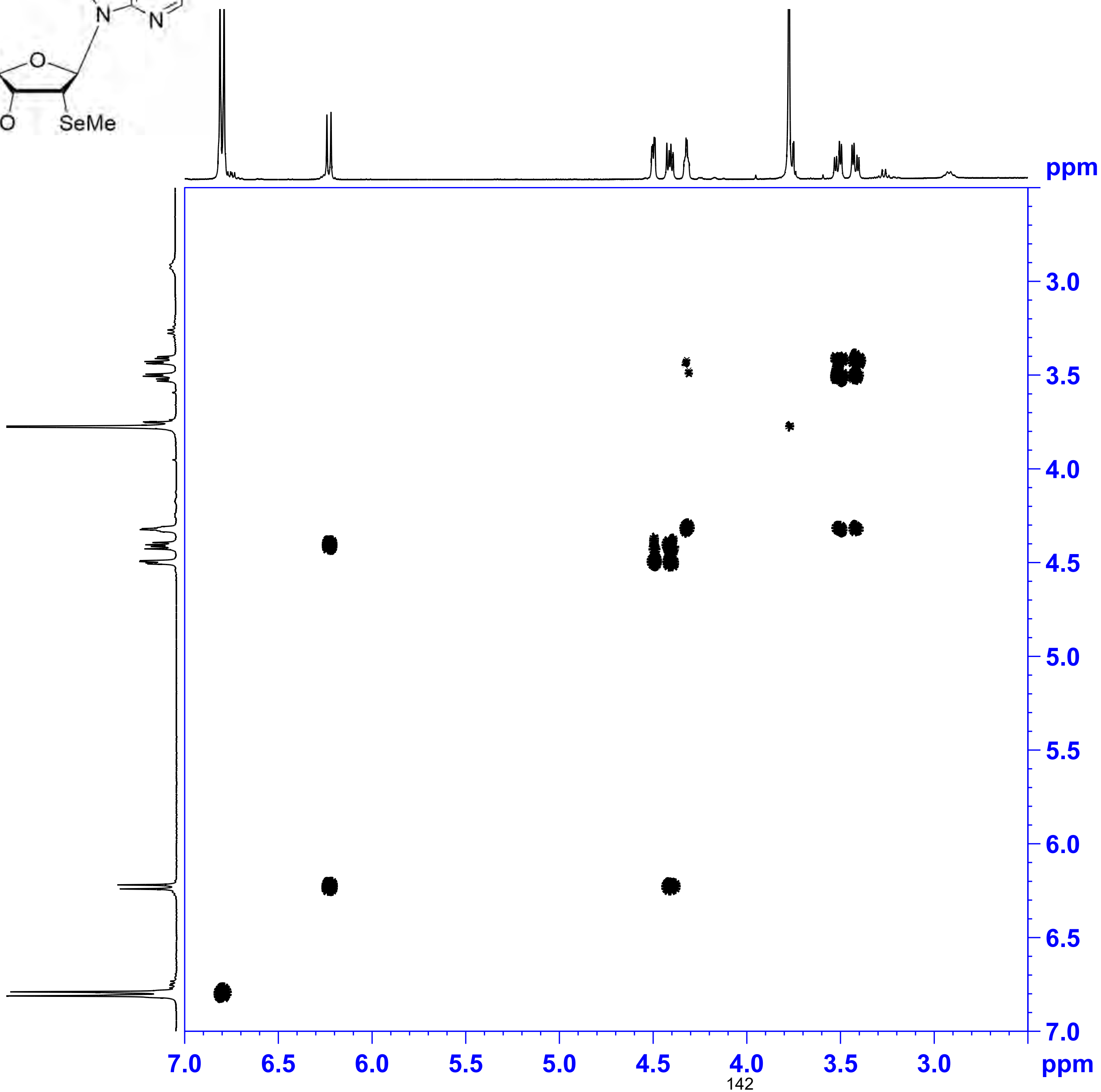

Current Data Parameters	
NAME	LH-II-72 NEW NMR
EXPNO	11
PROCNO	1

F2 - Acquisition Parameters	
Date_	20230625
Time	20.32 h
INSTRUM	AVIII_400
PROBHD	Z108618_0817 (
PULPROG	cosygpmfqf
TD	2048
SOLVENT	CDCl3
NS	3
DS	8
SWH	4629.629 Hz
FIDRES	4.521122 Hz
AQ	0.2211840 sec
RG	1620
DW	108.000 usec
DE	6.50 usec
TE	294.7 K
D0	0.00000300 sec
D1	1.97077799 sec
D13	0.00000400 sec
D16	0.00020000 sec
IN0	0.00021600 sec
TDav	1
SFO1	399.9118735 MHz
NUC1	1H
P1	500.00 usec
PLW1	31.62299919 W
GPNAM[1]	SINE.100
GPZ1	16.00 %
GPNAM[2]	SINE.100
GPZ2	12.00 %
GPNAM[3]	SINE.100
GPZ3	40.00 %
P16	1000.00 usec

F1 - Acquisition parameters	
TD	256
SFO1	399.9119 MHz
FIDRES	36.168980 Hz
SW	11.577 ppm
FnMODE	QF

F2 - Processing parameters	
SI	1024
SF	399.9100084 MHz
WDW	SINE
SSB	0
LB	0 Hz
GB	0
PC	1.40

F1 - Processing parameters	
SI	1024
MC2	QF
SF	399.9100084 MHz
WDW	SINE
SSB	0
LB	0 Hz
GB	0

^1H-^{13}C HSQC NMR spectrum of compound 18

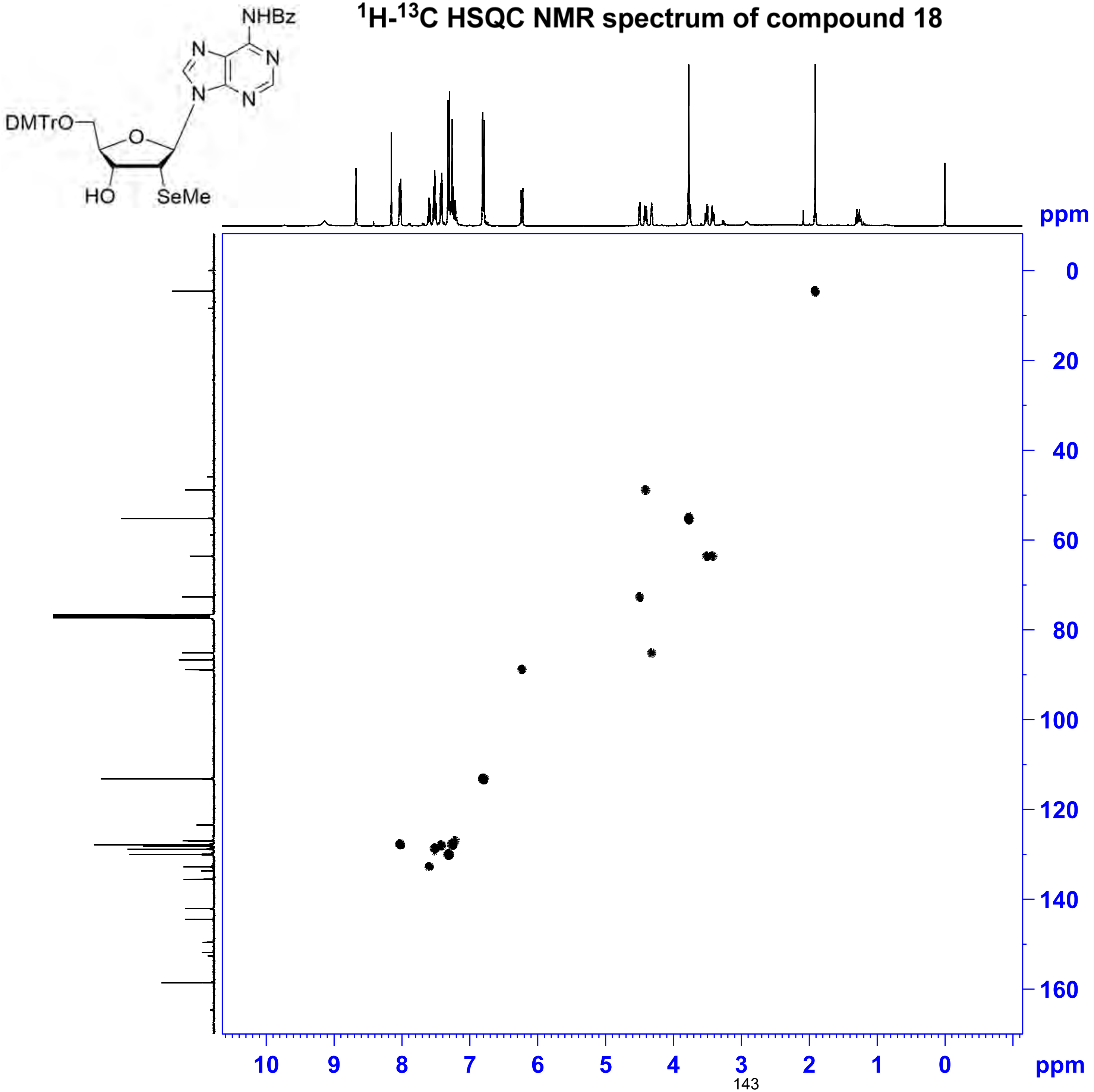


```
Current Data Parameters
NAME      LH-II-72 OLD NMR
EXPNO                   13
PROCNO                   1

F2 - Acquisition Parameters
Date_             20230625
Time                 18.59 h
INSTRUM          AVIII_400
PROBHD    Z108618_0146 (
PULPROG         hsqcedetgp
TD                    1024
SOLVENT              CDCl3
NS                       4
DS                      16
SWH               4716.981 Hz
FIDRES            9.212853 Hz
AQ               0.1085440 sec
RG                    1290
DW                 106.000 usec
DE                    6.50 usec
TE                   300.0 K
CNST2          145.0000000
D0              0.00000300 sec
D1              1.45965397 sec
D4              0.00172414 sec
D11             0.03000000 sec
D16             0.00020000 sec
D21             0.00345000 sec
IN0             0.00003000 sec
TDav                     1
ZGOPTNS
SFO1           400.1119099 MHz
NUC1                    1H
P1                   15.00 usec
P2                   30.00 usec
PLW1           17.29199982 W
SFO2           100.6152434 MHz
NUC2                   13C
CPDPRG[2              garp
P3                    8.70 usec
P4                   17.40 usec
PCPD2                56.50 usec
PLW2           96.68000031 W
PLW12           3.16230011 W
GPNAM[1]        SMSQ10.100
GPZ1                 80.00 %
GPNAM[2]        SMSQ10.100
GPZ2                 20.10 %
P16                1000.00 usec

F1 - Acquisition parameters
TD                     256
SFO1              100.6152 MHz
FIDRES          130.208328 Hz
SW                 165.648 ppm
FnMODE        Echo-Antiecho

F2 - Processing parameters
SI                    1024
SF             400.1100086 MHz
WDW                  QSINE
SSB                      2
LB                       0 Hz
GB                       0
PC                    1.40

F1 - Processing parameters
SI                    1024
MC2          echo-antiecho
SF             100.6077400 MHz
WDW                  QSINE
SSB                      2
LB                       0 Hz
GB                       0
```

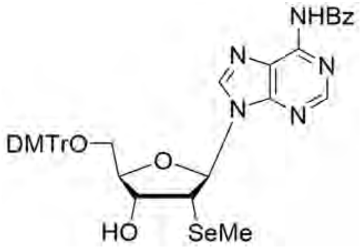

Expanded region of ^{1}H-^{13}C HSQC NMR spectrum of compound 18

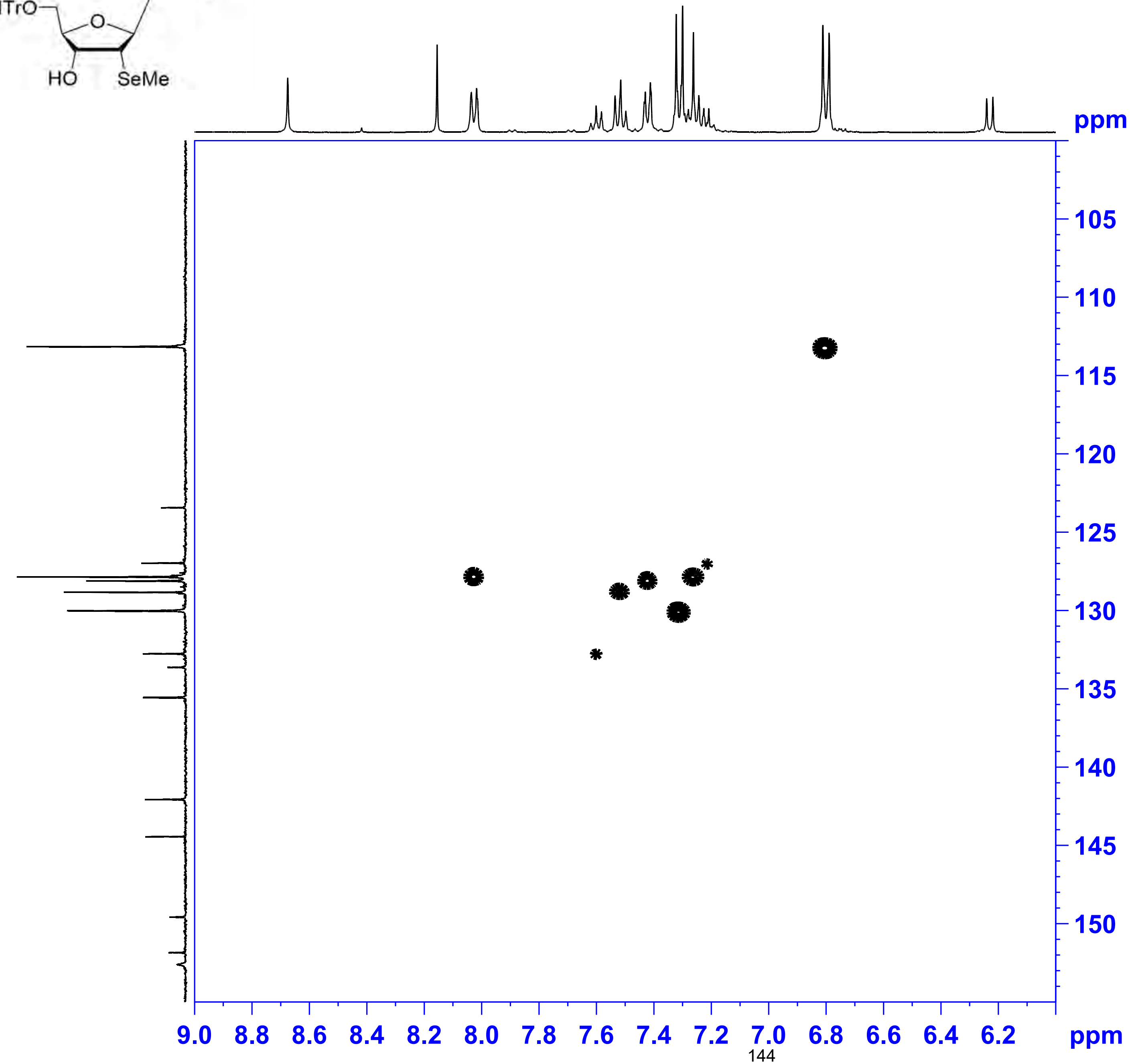


```
Current Data Parameters
NAME      LH-II-72 OLD NMR
EXPNO                   13
PROCNO                   1

F2 - Acquisition Parameters
Date_             20230625
Time                 18.59 h
INSTRUM          AVIII_400
PROBHD    Z108618_0146 (
PULPROG         hsqcedetgp
TD                    1024
SOLVENT              CDCl3
NS                       4
DS                      16
SWH               4716.981 Hz
FIDRES            9.212853 Hz
AQ               0.1085440 sec
RG                    1290
DW                 106.000 usec
DE                    6.50 usec
TE                   300.0 K
CNST2          145.0000000
D0              0.00000300 sec
D1              1.45965397 sec
D4              0.00172414 sec
D11             0.03000000 sec
D16             0.00020000 sec
D21             0.00345000 sec
IN0             0.00003000 sec
TDav                     1
ZGOPTNS
SFO1           400.1119099 MHz
NUC1                    1H
P1                   15.00 usec
P2                   30.00 usec
PLW1           17.29199982 W
SFO2           100.6152434 MHz
NUC2                   13C
CPDPRG[2              garp
P3                    8.70 usec
P4                   17.40 usec
PCPD2                56.50 usec
PLW2           96.68000031 W
PLW12           3.16230011 W
GPNAM[1]        SMSQ10.100
GPZ1                 80.00 %
GPNAM[2]        SMSQ10.100
GPZ2                 20.10 %
P16                1000.00 usec

F1 - Acquisition parameters
TD                     256
SFO1              100.6152 MHz
FIDRES          130.208328 Hz
SW                 165.648 ppm
FnMODE        Echo-Antiecho

F2 - Processing parameters
SI                    1024
SF             400.1100086 MHz
WDW                  QSINE
SSB                      2
LB            0 Hz
GB            0
PC                    1.40

F1 - Processing parameters
SI                    1024
MC2           echo-antiecho
SF             100.6077400 MHz
WDW                  QSINE
SSB                      2
LB            0 Hz
GB            0
```

^{31}P NMR spectrum of partially purified compound 8

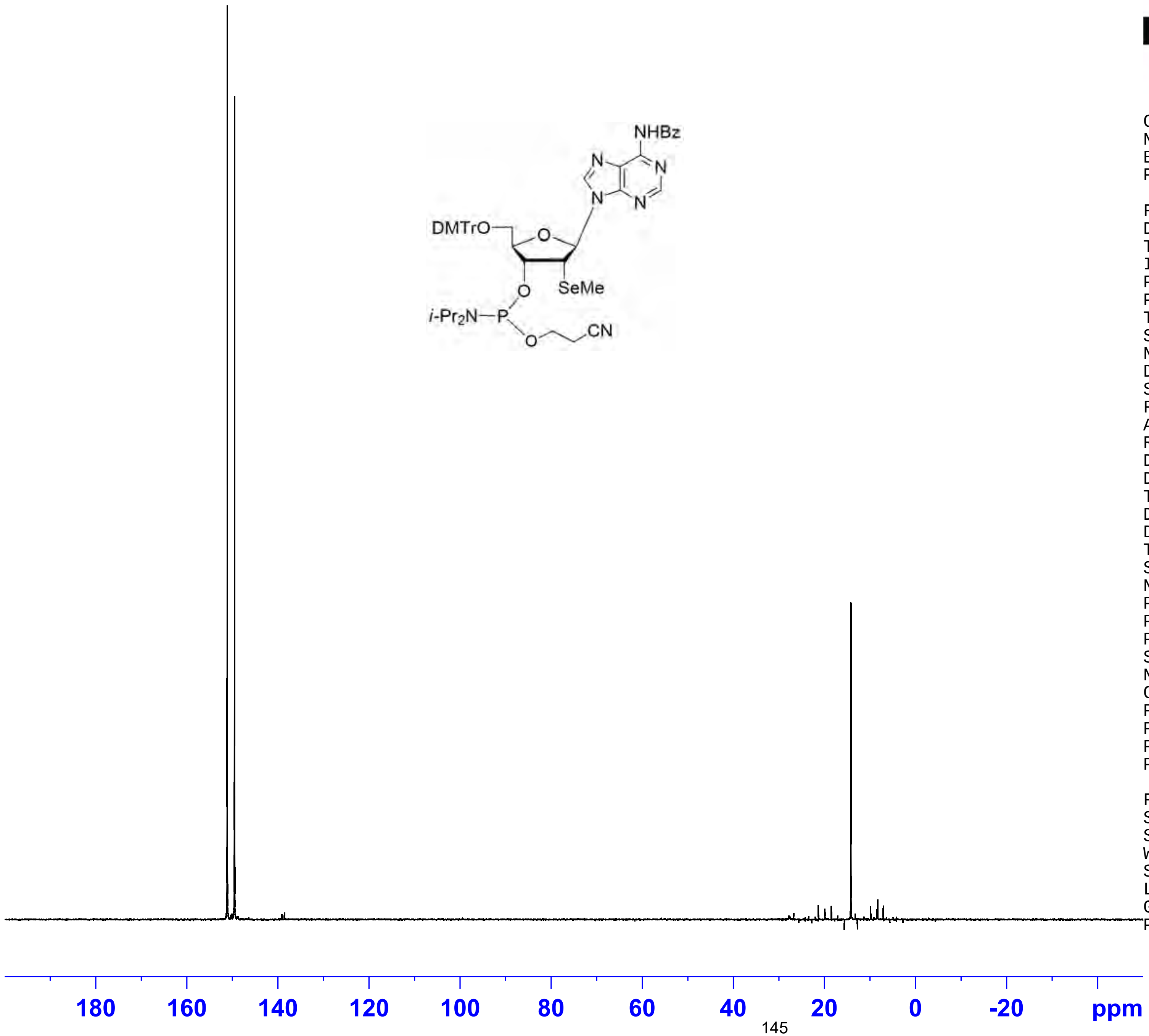


```
Current Data Parameters
NAME              LH-II-73
EXPNO                   10
PROCNO                   1

F2 - Acquisition Parameters
Date_             20230616
Time                 17.30 h
INSTRUM           AVIII_400
PROBHD    Z108618_0146 (
PULPROG            zgpg30
TD                   65536
SOLVENT              CDCl3
NS                    1000
DS                       4
SWH              49019.609 Hz
FIDRES            1.495960 Hz
AQ               0.6684672 sec
RG                    2050
DW                  10.200 usec
DE                    6.50 usec
TE                   300.0 K
D1              2.00000000 sec
D11             0.03000000 sec
TD0                      1
SFO1           161.9755954 MHz
NUC1                   31P
P0                    2.80 usec
P1                    8.40 usec
PLW1           41.93299866 W
SFO2           400.1116004 MHz
NUC2                    1H
CPDPRG[2           waltz16
PCPD2                90.00 usec
PLW2           17.29199982 W
PLW12           0.48032999 W
PLW13           0.24160001 W

F2 - Processing parameters
SI                   32768
SF             161.9674970 MHz
WDW                     EM
SSB       0
LB                    2.00 Hz
GB        0
PC                    1.40
```

Expanded region of ^{31}P NMR spectrum of partially purified compound 8

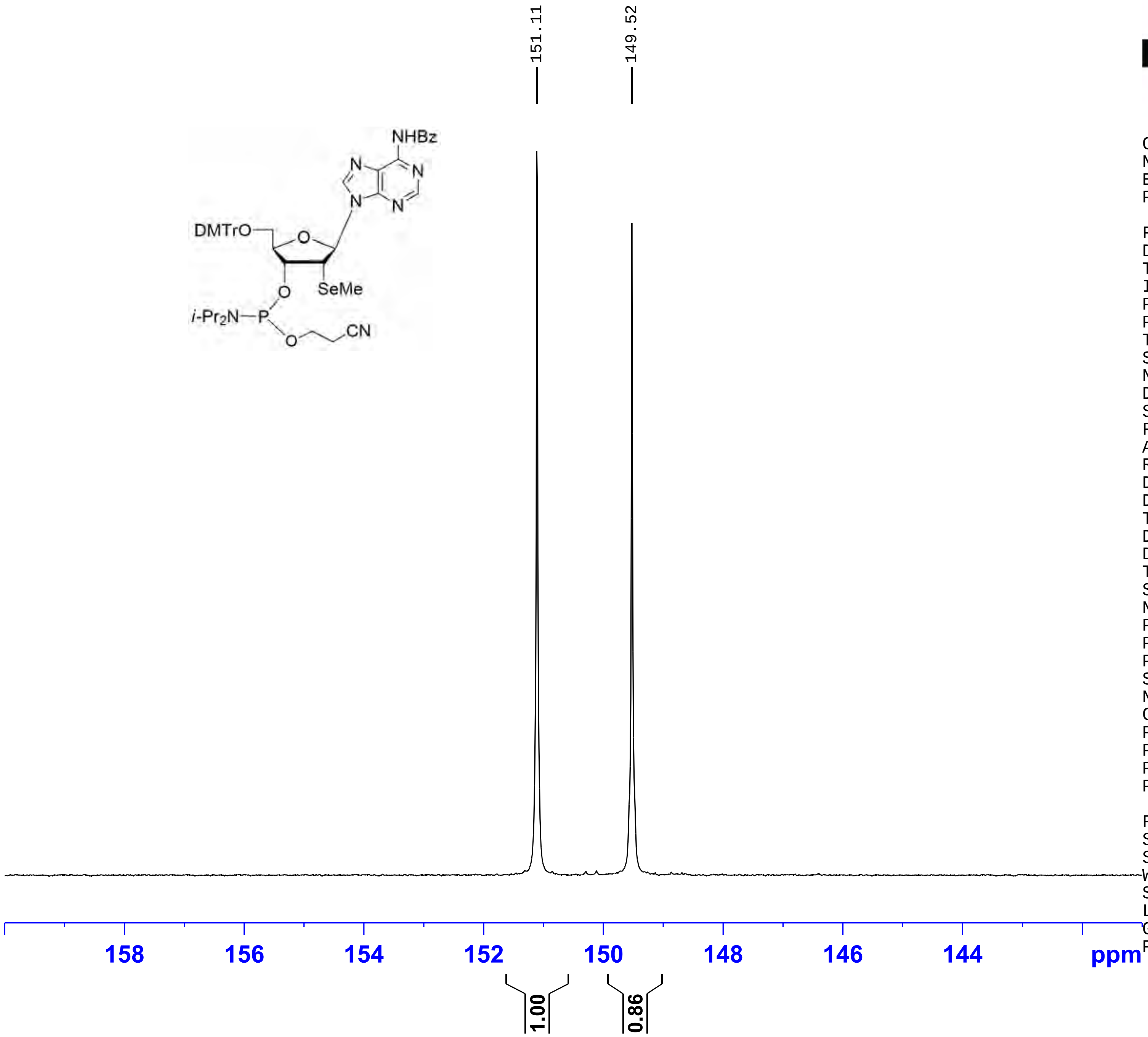

Current Data Parameters
NAME LH-II-73
EXPNO 10
PROCNO 1

F2 - Acquisition Parameters
Date_ 20230616
Time 17.30 h
INSTRUM AVIII_400
PROBHD Z108618_0146 (
PULPROG zgpg30
TD 65536
SOLVENT CDCl3
NS 1000
DS 4
SWH 49019.609 Hz
FIDRES 1.495960 Hz
AQ 0.6684672 sec
RG 2050
DW 10.200 usec
DE 6.50 usec
TE 300.0 K
D1 2.00000000 sec
D11 0.03000000 sec
TD0 1
SFO1 161.9755954 MHz
NUC1 31P
P0 2.80 usec
P1 8.40 usec
PLW1 41.93299866 W
SFO2 400.1116004 MHz
NUC2 1H
CPDPRG[2 waltz16
PCPD2 90.00 usec
PLW2 17.29199982 W
PLW12 0.48032999 W
PLW13 0.24160001 W

F2 - Processing parameters
SI 32768
SF 161.9674970 MHz
WDW EM
SSB 0
LB 2.00 Hz
GB 0
PC 1.40

Expanded region of ^{31}P NMR spectrum of partially purified compound 8

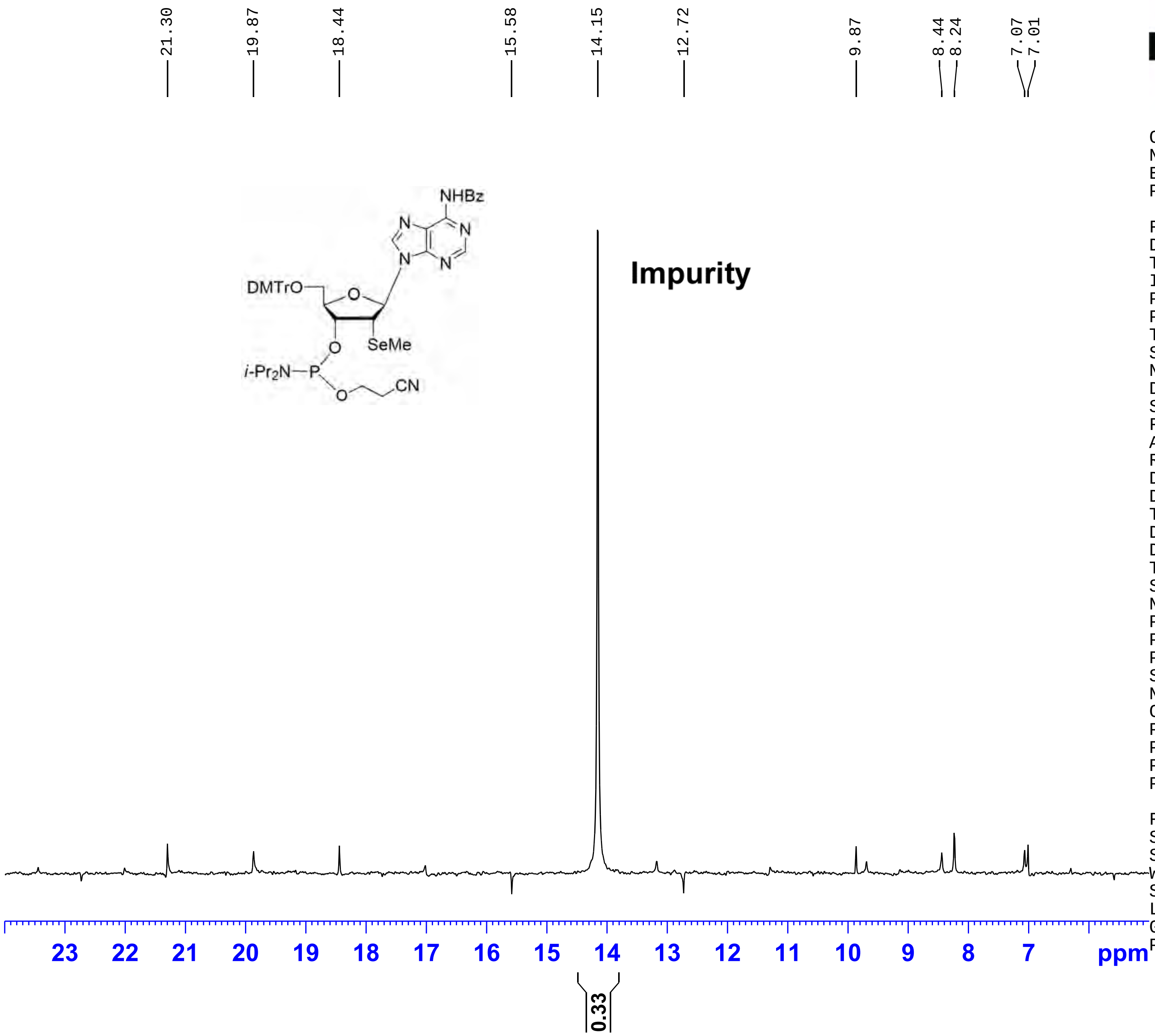


```
Current Data Parameters
NAME              LH-II-73
EXPNO                   10
PROCNO                   1

F2 - Acquisition Parameters
Date_             20230616
Time                 17.30 h
INSTRUM           AVIII_400
PROBHD     Z108618_0146 (
PULPROG            zgpg30
TD                  65536
SOLVENT             CDCl3
NS                   1000
DS                      4
SWH             49019.609 Hz
FIDRES           1.495960 Hz
AQ              0.6684672 sec
RG                   2050
DW                 10.200 usec
DE                   6.50 usec
TE                  300.0 K
D1             2.00000000 sec
D11            0.03000000 sec
TD0                     1
SFO1          161.9755954 MHz
NUC1                  31P
P0                   2.80 usec
P1                   8.40 usec
PLW1          41.93299866 W
SFO2          400.1116004 MHz
NUC2                   1H
CPDPRG[2          waltz16
PCPD2               90.00 usec
PLW2          17.29199982 W
PLW12          0.48032999 W
PLW13          0.24160001 W

F2 - Processing parameters
SI                  32768
SF            161.9674970 MHz
WDW                    EM
SSB      0
LB                   2.00 Hz
GB       0
PC                   1.40
```

Expanded region of ^{31}P NMR spectrum of partially purified compound 8 before precipitation

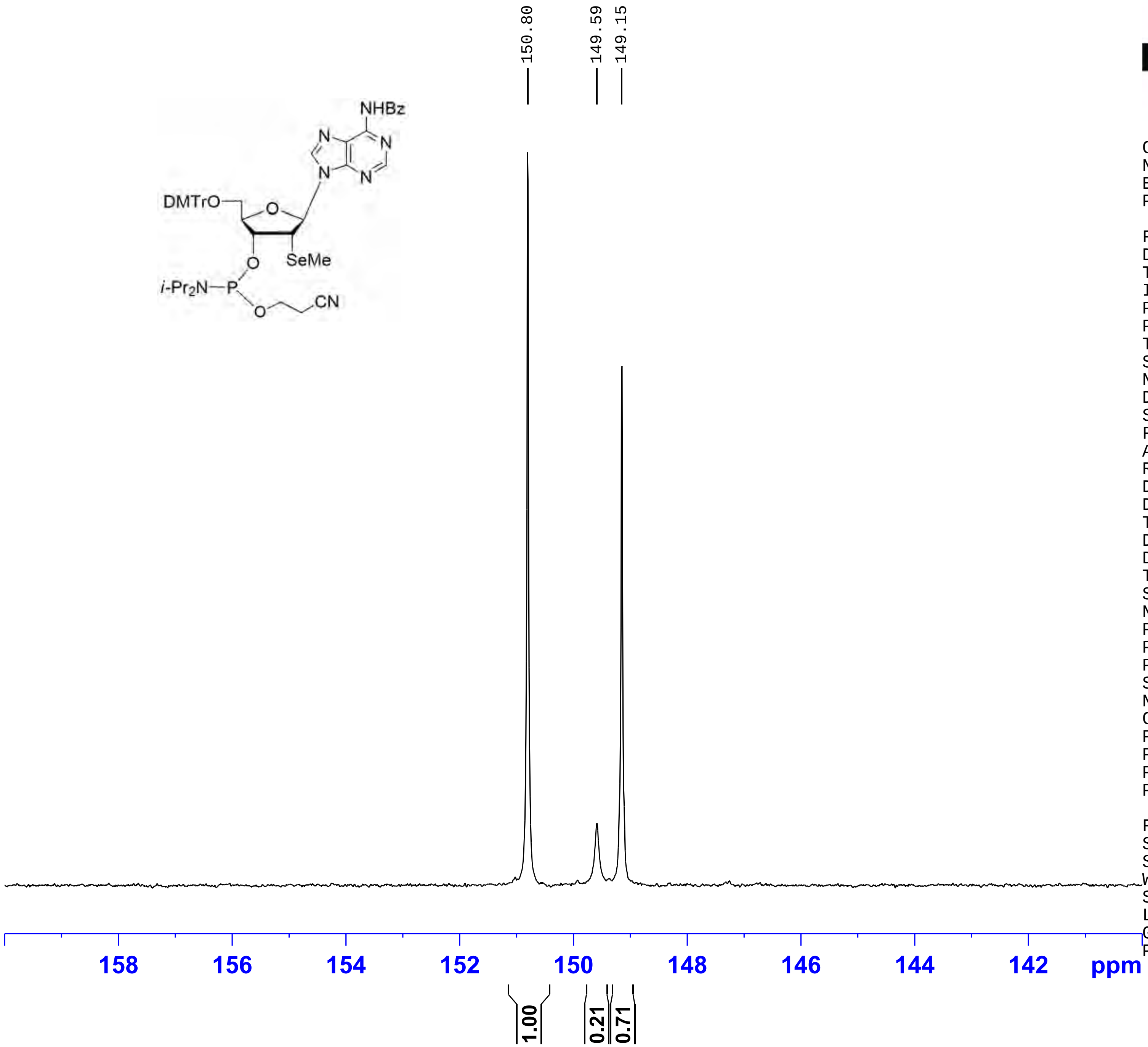


```
Current Data Parameters
NAME              LH-II-60
EXPNO                   10
PROCNO                   1

F2 - Acquisition Parameters
Date_             20230517
Time                 14.14 h
INSTRUM           AVIII_400
PROBHD    Z108618_0146 (
PULPROG             zgpg30
TD                   65536
SOLVENT                D2O
NS                    1000
DS                       4
SWH              49019.609 Hz
FIDRES            1.495960 Hz
AQ               0.6684672 sec
RG                    2050
DW                  10.200 usec
DE                    6.50 usec
TE                   300.0 K
D1              2.00000000 sec
D11             0.03000000 sec
TD0                      1
SFO1           161.9755954 MHz
NUC1                   31P
P0                    2.80 usec
P1                    8.40 usec
PLW1           41.93299866 W
SFO2           400.1116004 MHz
NUC2                    1H
CPDPRG[2           waltz16
PCPD2                90.00 usec
PLW2           17.29199982 W
PLW12           0.48032999 W
PLW13           0.24160001 W

F2 - Processing parameters
SI                   32768
SF             161.9674970 MHz
WDW                     EM
SSB       0
LB                    2.00 Hz
GB        0
PC                    1.40
```

Expanded region of ^{31}P NMR spectrum of partially purified compound 8 after precipitation

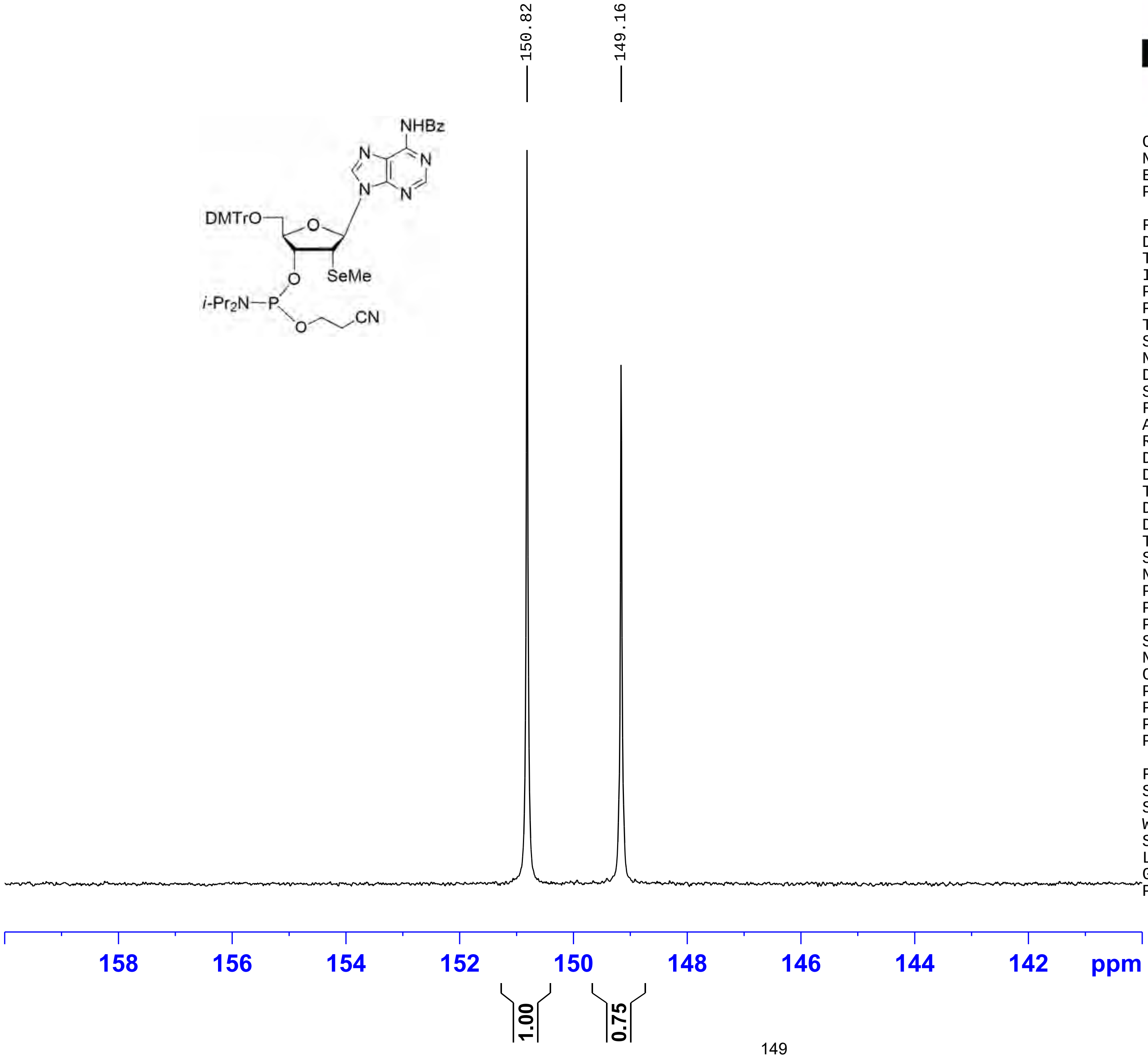


```
Current Data Parameters
NAME      LH-II-60 AFTER PRECIP
EXPNO                  10
PROCNO                  1

F2 - Acquisition Parameters
Date_            20230517
Time                17.12 h
INSTRUM          AVIII_400
PROBHD   Z108618_0146 (
PULPROG            zgpg30
TD                  65536
SOLVENT               D2O
NS                   1000
DS                      4
SWH             49019.609 Hz
FIDRES           1.495960 Hz
AQ              0.6684672 sec
RG                   2050
DW                 10.200 usec
DE                   6.50 usec
TE                  300.0 K
D1             2.00000000 sec
D11            0.03000000 sec
TD0                     1
SFO1          161.9755954 MHz
NUC1                  31P
P0                   2.80 usec
P1                   8.40 usec
PLW1          41.93299866 W
SFO2          400.1116004 MHz
NUC2                   1H
CPDPRG[2          waltz16
PCPD2               90.00 usec
PLW2          17.29199982 W
PLW12          0.48032999 W
PLW13          0.24160001 W

F2 - Processing parameters
SI                  32768
SF            161.9674970 MHz
WDW                    EM
SSB      0
LB                   2.00 Hz
GB       0
PC                   1.40
```

Expanded region of ^{31}P NMR spectrum of partially purified compound 8 after precipitation

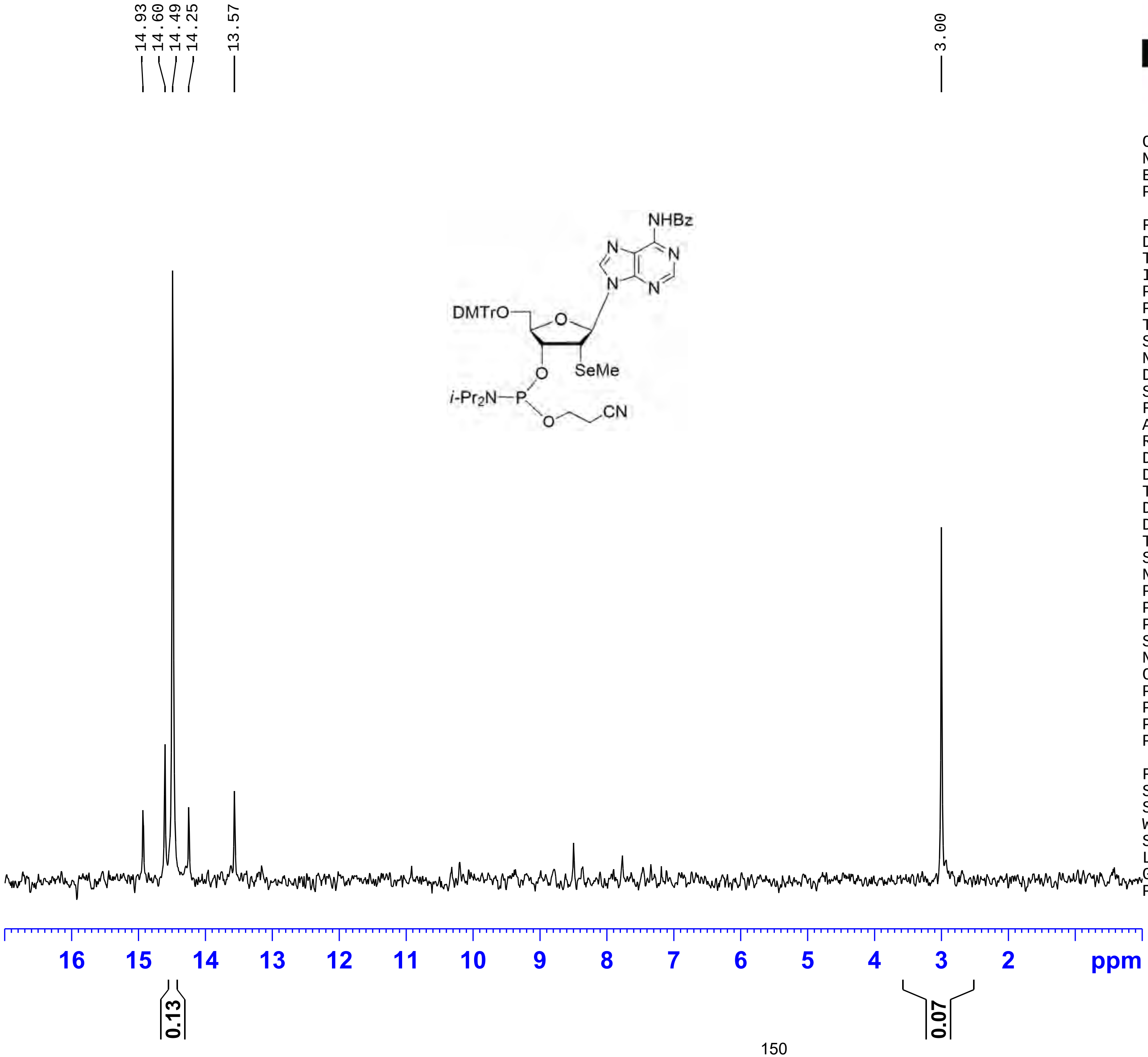


```
Current Data Parameters
NAME      LH-II-60 AFTER PRECIP
EXPNO                      10
PROCNO                      1

F2 - Acquisition Parameters
Date_                20230517
Time                  17.12 h
INSTRUM             AVIII_400
PROBHD    Z108618_0146 (
PULPROG               zgpg30
TD                     65536
SOLVENT                  D2O
NS                      1000
DS                         4
SWH            49019.609 Hz
FIDRES          1.495960 Hz
AQ             0.6684672 sec
RG                      2050
DW                10.200 usec
DE                  6.50 usec
TE                 300.0 K
D1            2.00000000 sec
D11           0.03000000 sec
TD0                        1
SFO1         161.9755954 MHz
NUC1                     31P
P0                  2.80 usec
P1                  8.40 usec
PLW1         41.93299866 W
SFO2         400.1116004 MHz
NUC2                      1H
CPDPRG[2             waltz16
PCPD2              90.00 usec
PLW2         17.29199982 W
PLW12         0.48032999 W
PLW13         0.24160001 W

F2 - Processing parameters
SI                     32768
SF           161.9674970 MHz
WDW                       EM
SSB       0
LB                    2.00 Hz
GB        0
PC                      1.40
```

^{31}P NMR spectrum of compound 7

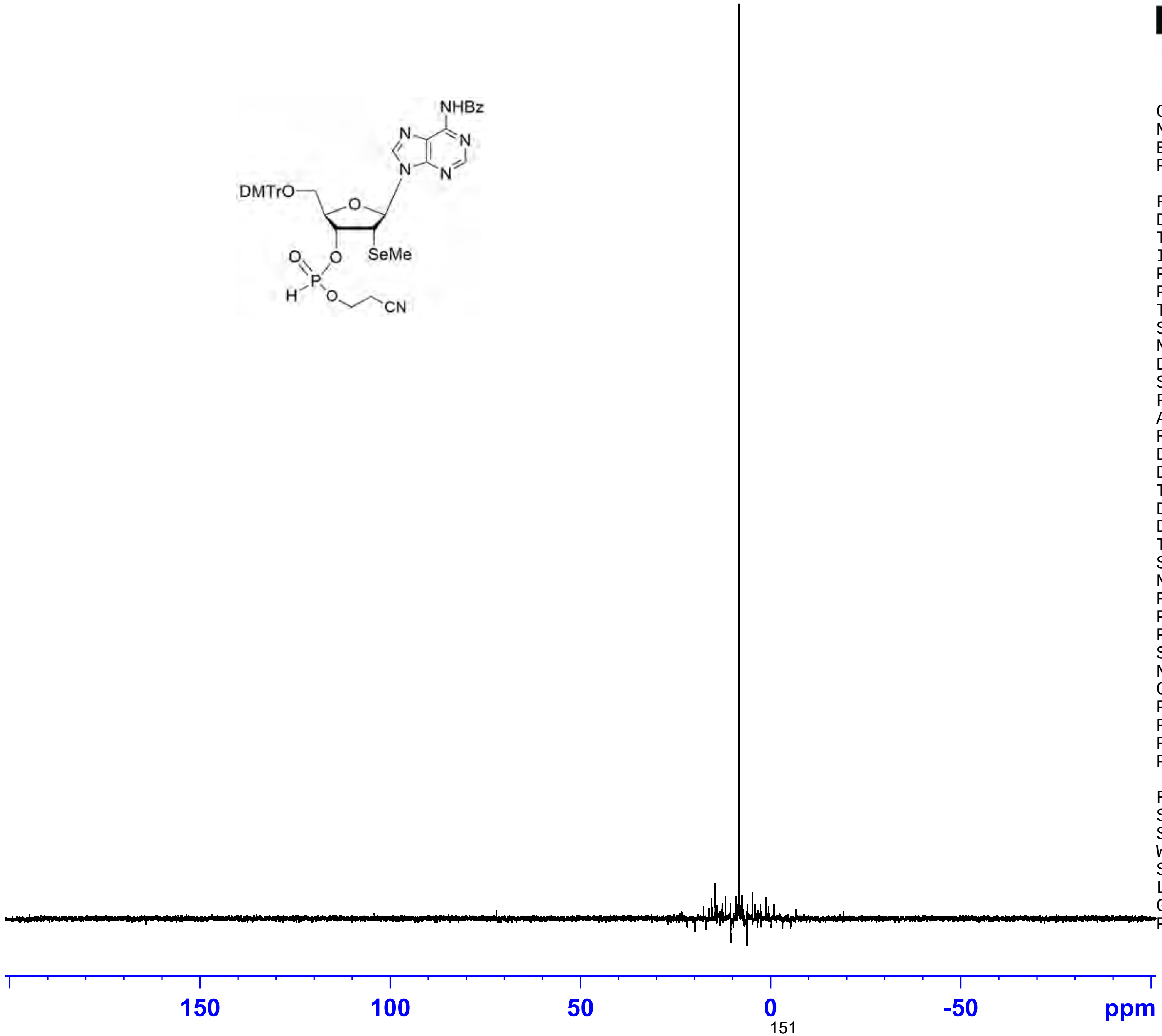

Current Data Parameters

NAME	LH-II-55
EXPNO	20
PROCNO	1

F2 - Acquisition Parameters

Date_	20230504
Time	11.50 h
INSTRUM	AVIII_400
PROBHD	Z108618_0146 (
PULPROG	zgpg30
TD	65536
SOLVENT	D2O
NS	1000
DS	4
SWH	49019.609 Hz
FIDRES	1.495960 Hz
AQ	0.6684672 sec
RG	2050
DW	10.200 usec
DE	6.50 usec
TE	300.0 K
D1	2.00000000 sec
D11	0.03000000 sec
TD0	1
SFO1	161.9755954 MHz
NUC1	31P
P0	2.80 usec
P1	8.40 usec
PLW1	41.93299866 W
SFO2	400.1116004 MHz
NUC2	1H
CPDPRG[2	waltz16
PCPD2	90.00 usec
PLW2	17.29199982 W
PLW12	0.48032999 W
PLW13	0.24160001 W

F2 - Processing parameters

SI	32768
SF	161.9674970 MHz
WDW	EM
SSB	0
LB	2.00 Hz
GB	0
PC	1.40

Expanded region of ^{31}P NMR spectrum of compound 7

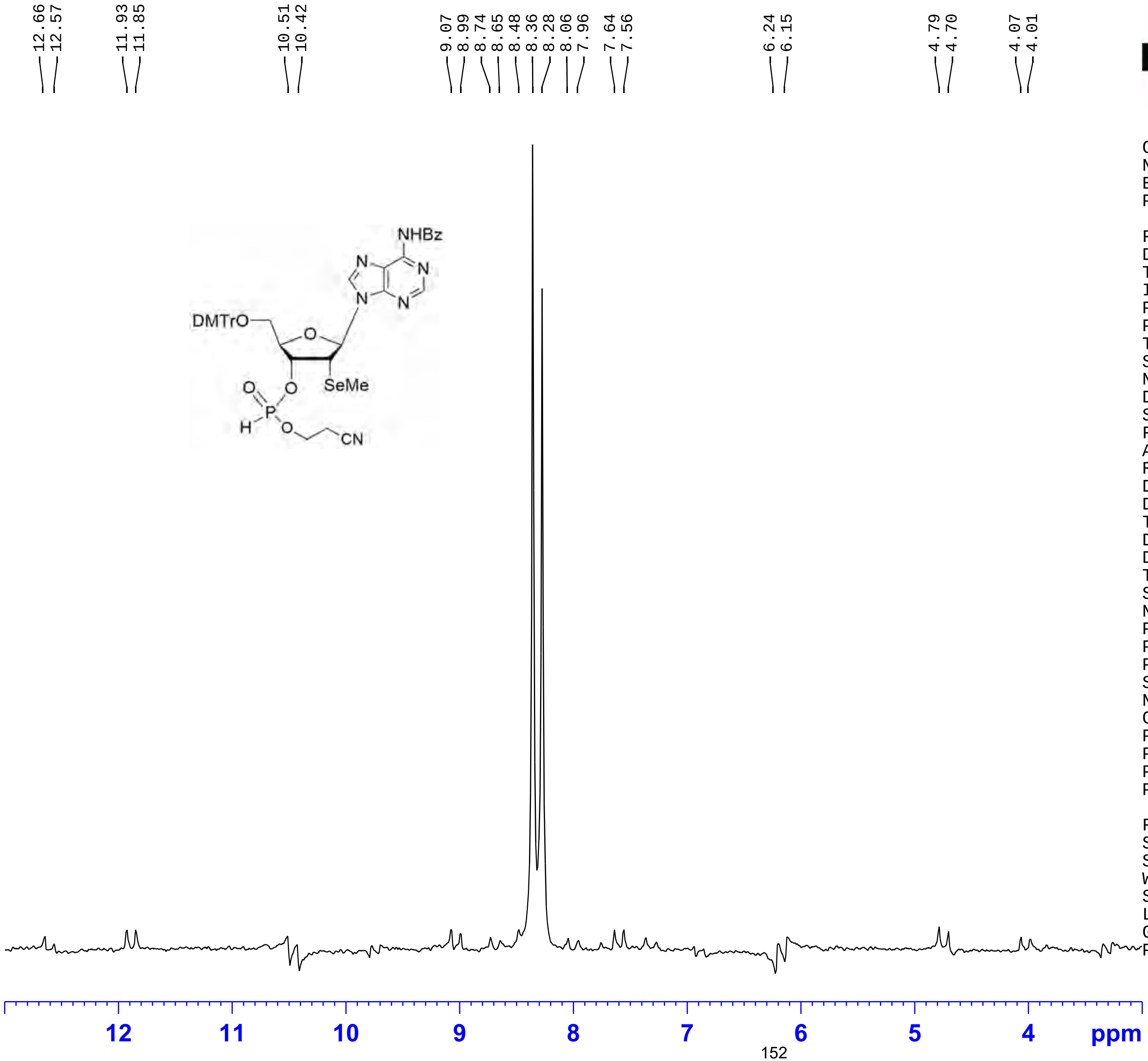

Current Data Parameters
NAME LH-II-55
EXPNO 20
PROCNO 1

F2 - Acquisition Parameters

Date_	20230504	
Time	11.50	h
INSTRUM	AVIII_400	
PROBHD	Z108618_0146 (	
PULPROG	zgpg30	
TD	65536	
SOLVENT	D2O	
NS	1000	
DS	4	
SWH	49019.609	Hz
FIDRES	1.495960	Hz
AQ	0.6684672	sec
RG	2050	
DW	10.200	usec
DE	6.50	usec
TE	300.0	K
D1	2.00000000	sec
D11	0.03000000	sec
TD0	1	
SFO1	161.9755954	MHz
NUC1	31P	
P0	2.80	usec
P1	8.40	usec
PLW1	41.93299866	W
SFO2	400.1116004	MHz
NUC2	1H	
CPDPRG[2	waltz16	
PCPD2	90.00	usec
PLW2	17.29199982	W
PLW12	0.48032999	W
PLW13	0.24160001	W

F2 - Processing parameters

SI	32768	
SF	161.9674970	MHz
WDW	EM	
SSB	0	
LB	2.00	Hz
GB	0	
PC	1.40	

Expanded region of ^{31}P NMR spectrum of compound 7

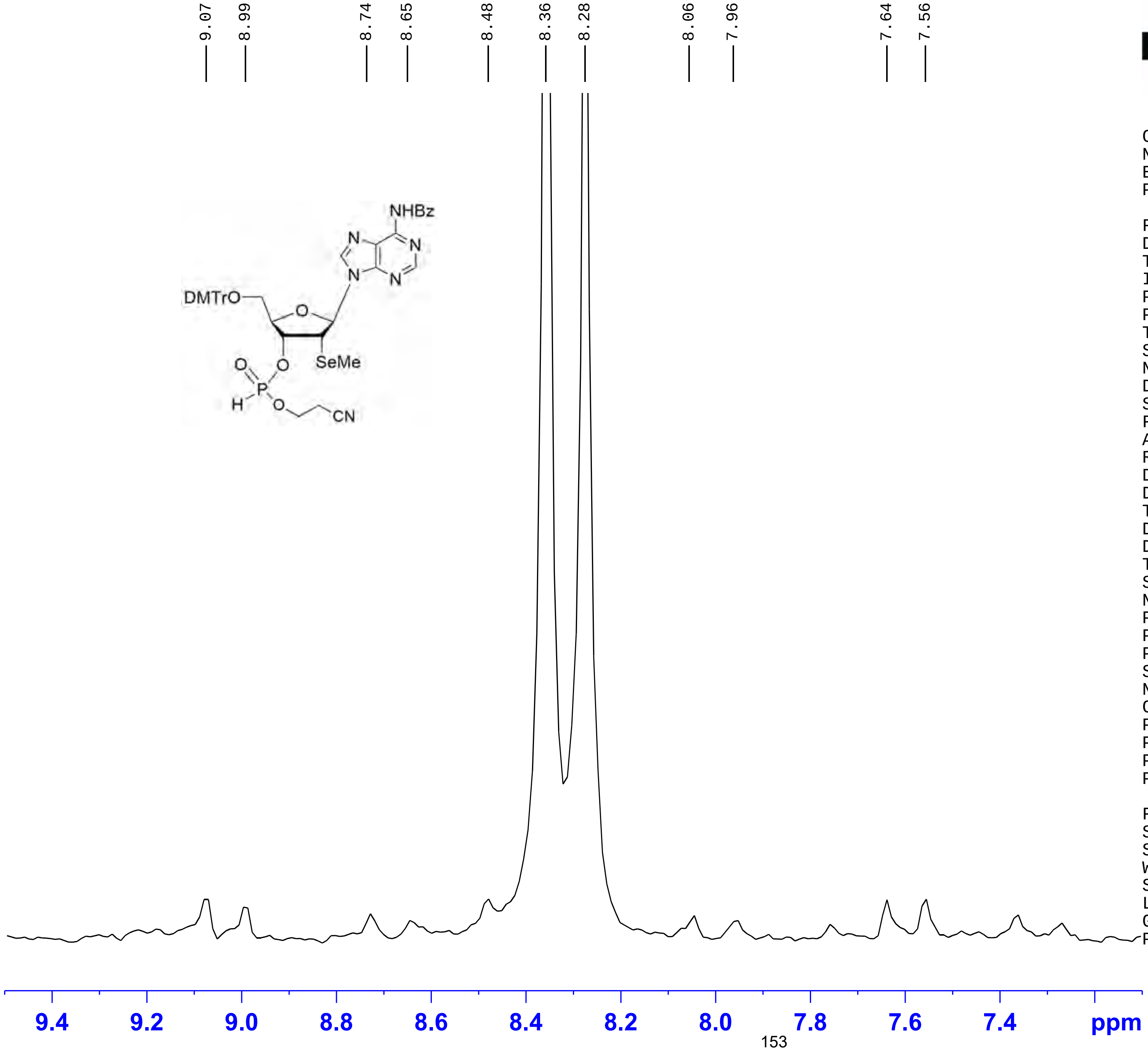


```
Current Data Parameters
NAME              LH-II-55
EXPNO                   20
PROCNO                   1

F2 - Acquisition Parameters
Date_             20230504
Time                 11.50 h
INSTRUM           AVIII_400
PROBHD     Z108618_0146 (
PULPROG             zgpg30
TD                   65536
SOLVENT                D2O
NS                    1000
DS                       4
SWH              49019.609 Hz
FIDRES            1.495960 Hz
AQ               0.6684672 sec
RG                    2050
DW                  10.200 usec
DE                    6.50 usec
TE                   300.0 K
D1              2.00000000 sec
D11             0.03000000 sec
TD0                      1
SFO1           161.9755954 MHz
NUC1                   31P
P0                    2.80 usec
P1                    8.40 usec
PLW1           41.93299866 W
SFO2           400.1116004 MHz
NUC2                    1H
CPDPRG[2           waltz16
PCPD2                90.00 usec
PLW2           17.29199982 W
PLW12           0.48032999 W
PLW13           0.24160001 W

F2 - Processing parameters
SI                   32768
SF             161.9674970 MHz
WDW                     EM
SSB      0
LB                    2.00 Hz
GB       0
PC                    1.40
```

^{1}H NMR spectrum of compound 12

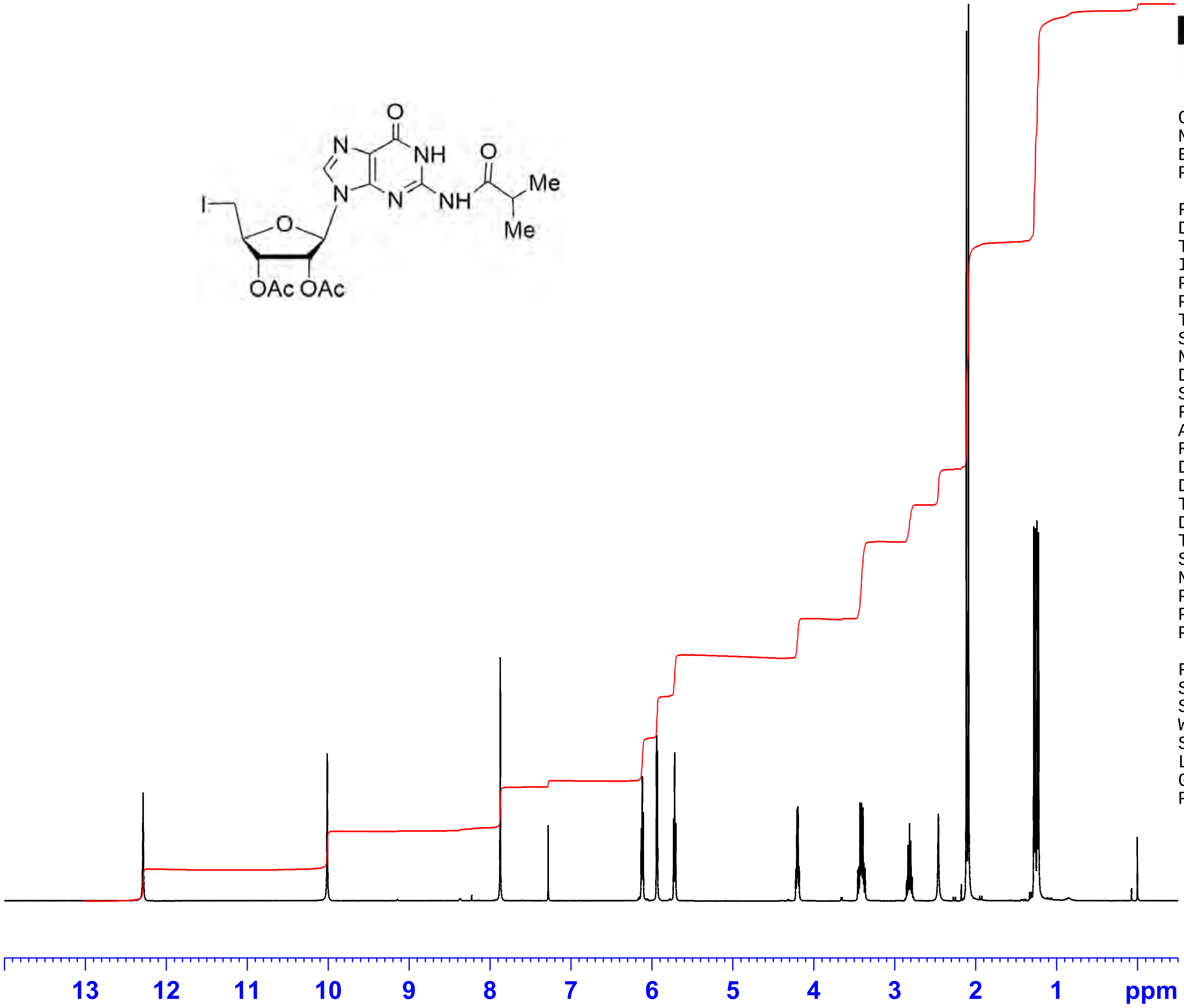

Current Data Parameters

NAME	LH-II-74 NEW NMR
EXPNO	10
PROCNO	1

F2 - Acquisition Parameters

Date_	20230624
Time	13.12 h
INSTRUM	AVIII_400
PROBHD	Z108618_0817 (
PULPROG	zg30
TD	65536
SOLVENT	CDCl3
NS	16
DS	2
SWH	8223.685 Hz
FIDRES	0.250967 Hz
AQ	3.9845889 sec
RG	144
DW	60.800 usec
DE	6.50 usec
TE	296.1 K
D1	1.00000000 sec
TD0	1
SFO1	399.9124696 MHz
NUC1	1H
P0	166.67 usec
P1	500.00 usec
PLW1	31.62299919 W

F2 - Processing parameters

SI	32768
SF	399.9100014 MHz
WDW	EM
SSB	0
LB	0.30 Hz
GB	0
PC	1.00

Expanded region of the ^{1}H NMR spectrum of compound 12

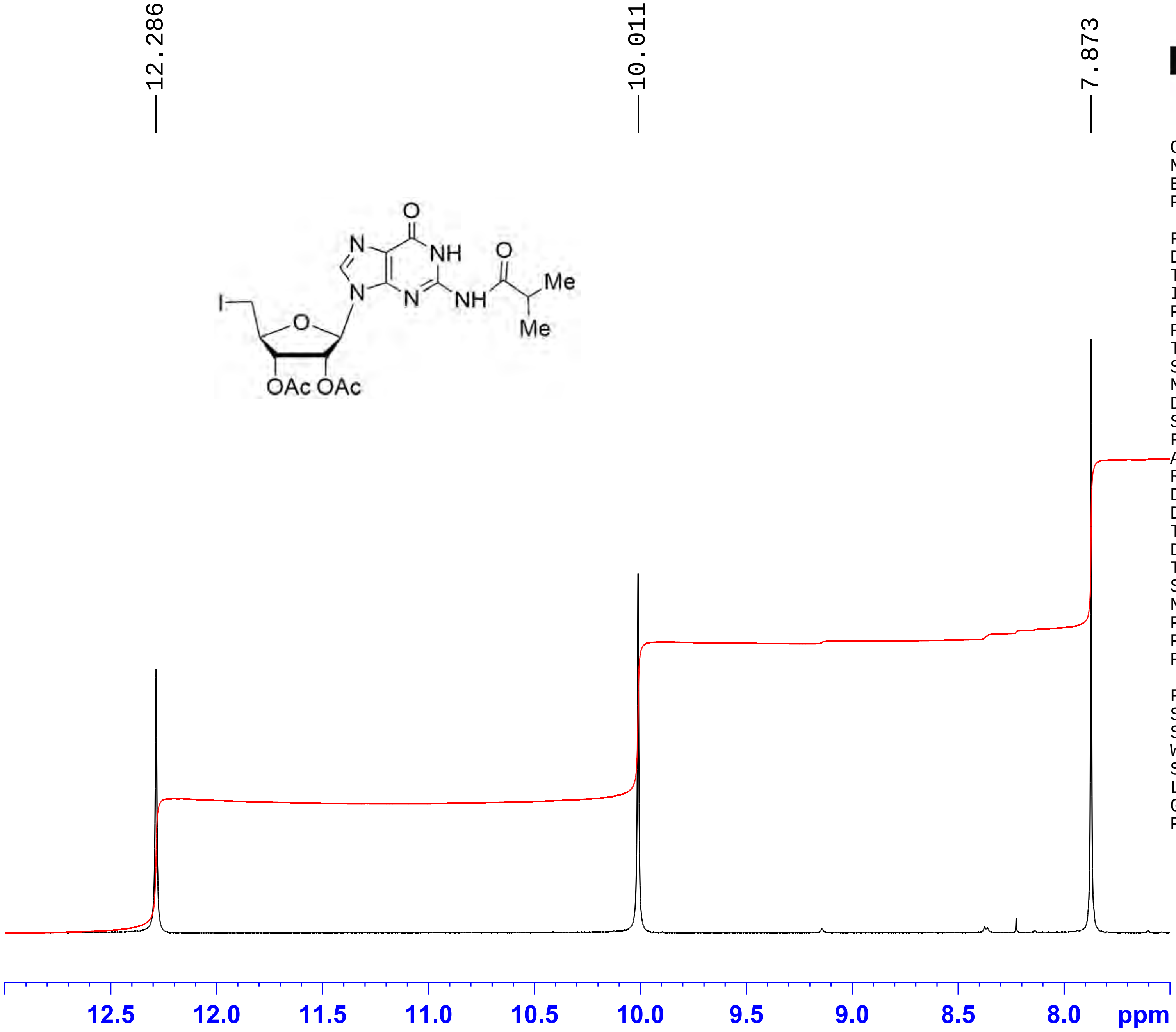

Current Data Parameters
NAME LH-II-74 NEW NMR
EXPNO 10
PROCNO 1

F2 - Acquisition Parameters

Date_	20230624
Time	13.12 h
INSTRUM	AVIII_400
PROBHD	Z108618_0817 (
PULPROG	zg30
TD	65536
SOLVENT	CDCl3
NS	16
DS	2
SWH	8223.685 Hz
FIDRES	0.250967 Hz
AQ	3.9845889 sec
RG	144
DW	60.800 usec
DE	6.50 usec
TE	296.1 K
D1	1.00000000 sec
TD0	1
SFO1	399.9124696 MHz
NUC1	1H
P0	166.67 usec
P1	500.00 usec
PLW1	31.62299919 W

F2 - Processing parameters

SI	32768
SF	399.9100014 MHz
WDW	EM
SSB	0
LB	0.30 Hz
GB	0
PC	1.00

Expanded region of the ^{1}H NMR spectrum of compound 12

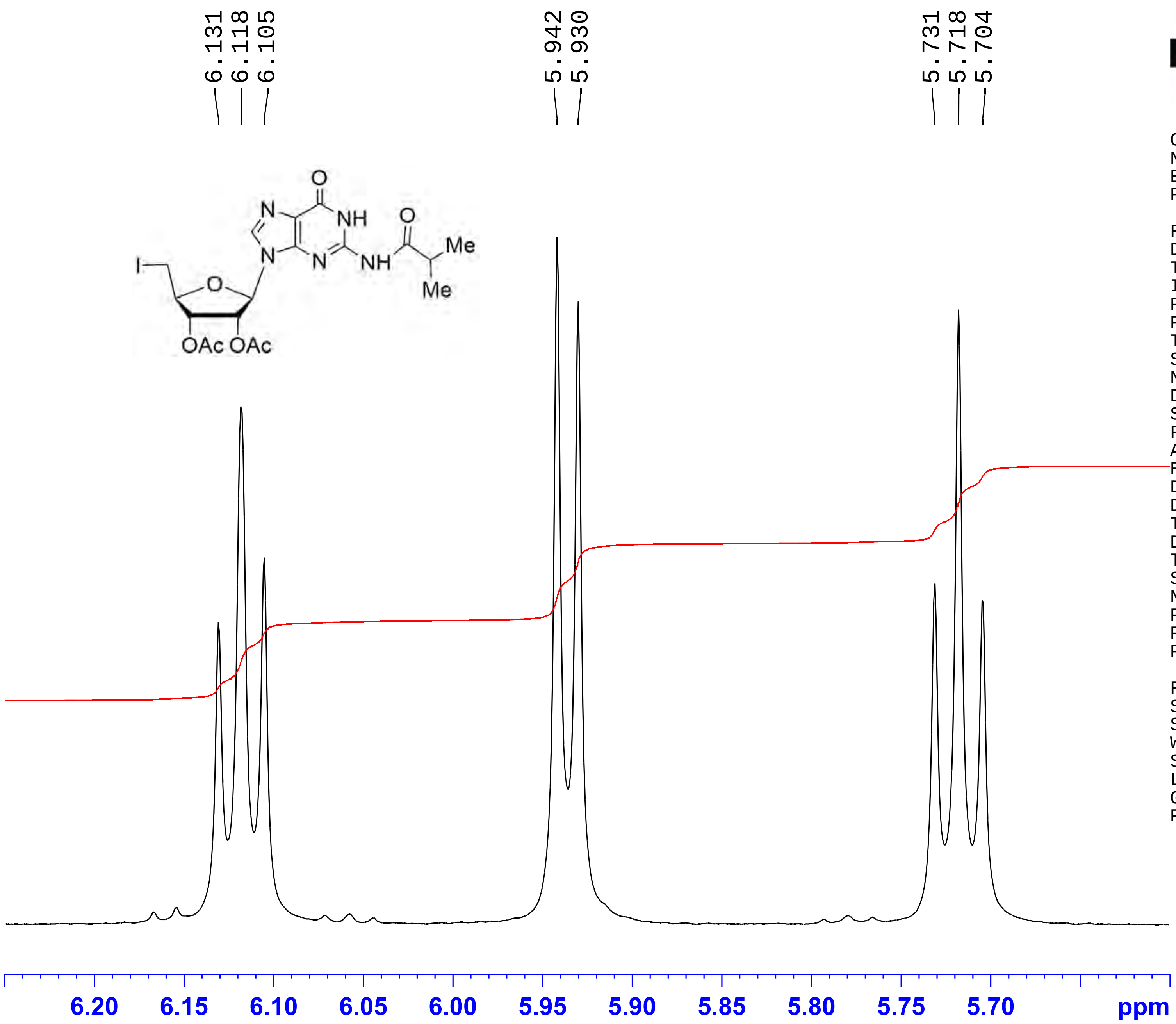

Current Data Parameters
NAME LH-II-74 NEW NMR
EXPNO 10
PROCNO 1

F2 - Acquisition Parameters
Date_ 20230624
Time 13.12 h
INSTRUM AVIII_400
PROBHD Z108618_0817 (
PULPROG zg30
TD 65536
SOLVENT CDCl3
NS 16
DS 2
SWH 8223.685 Hz
FIDRES 0.250967 Hz
AQ 3.9845889 sec
RG 144
DW 60.800 usec
DE 6.50 usec
TE 296.1 K
D1 1.00000000 sec
TD0 1
SFO1 399.9124696 MHz
NUC1 1H
P0 166.67 usec
P1 500.00 usec
PLW1 31.62299919 W

F2 - Processing parameters
SI 32768
SF 399.9100014 MHz
WDW EM
SSB 0
LB 0.30 Hz
GB 0
PC 1.00

Expanded region of the ^{1}H NMR spectrum of compound 12

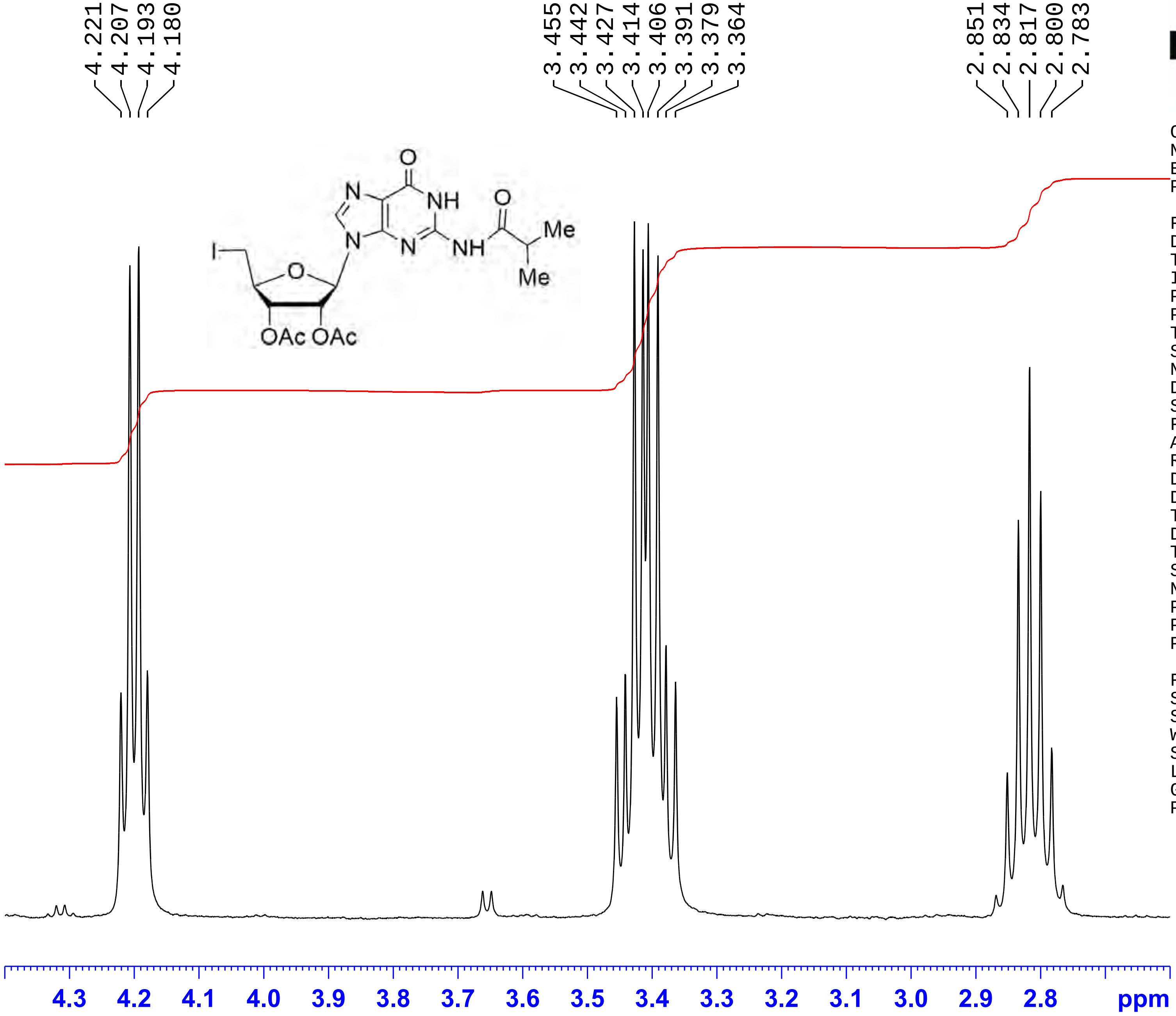

Current Data Parameters
NAME LH-II-74 NEW NMR
EXPNO 10
PROCNO 1

F2 - Acquisition Parameters
Date_ 20230624
Time 13.12 h
INSTRUM AVIII_400
PROBHD Z108618_0817 (
PULPROG zg30
TD 65536
SOLVENT CDCl3
NS 16
DS 2
SWH 8223.685 Hz
FIDRES 0.250967 Hz
AQ 3.9845889 sec
RG 144
DW 60.800 usec
DE 6.50 usec
TE 296.1 K
D1 1.00000000 sec
TD0 1
SFO1 399.9124696 MHz
NUC1 1H
P0 166.67 usec
P1 500.00 usec
PLW1 31.62299919 W

F2 - Processing parameters
SI 32768
SF 399.9100014 MHz
WDW EM
SSB 0
LB 0.30 Hz
GB 0
PC 1.00

Expanded region of the ^{1}H NMR spectrum of compound 12

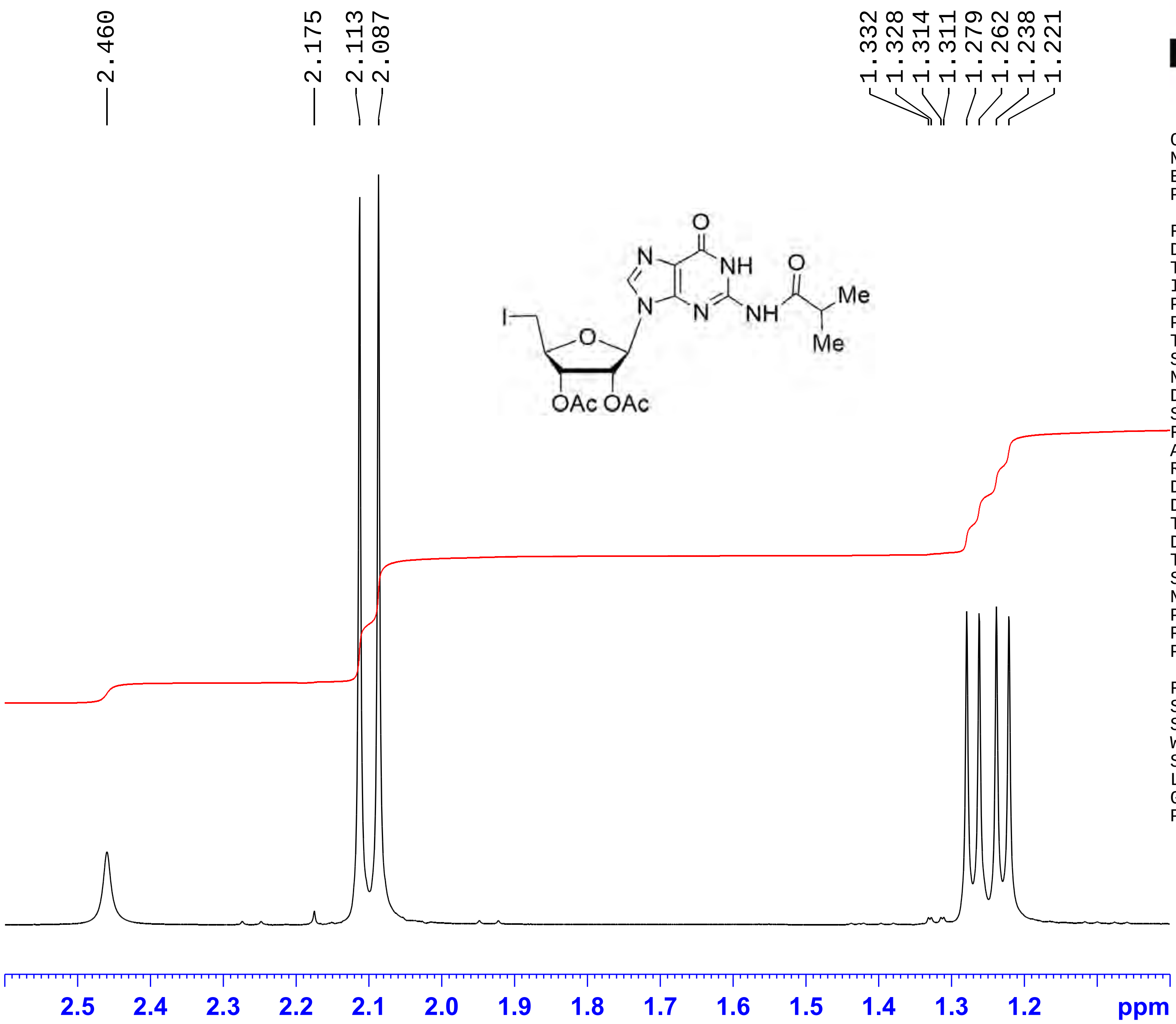

BRUKER

```
Current Data Parameters
NAME       LH-II-74 NEW NMR
EXPNO                    10
PROCNO                    1

F2 - Acquisition Parameters
Date_              20230624
Time                  13.12 h
INSTRUM            AVIII_400
PROBHD     Z108618_0817 (
PULPROG                zg30
TD                    65536
SOLVENT               CDCl3
NS                       16
DS                        2
SWH                8223.685 Hz
FIDRES             0.250967 Hz
AQ                3.9845889 sec
RG                      144
DW                   60.800 usec
DE                     6.50 usec
TE                    296.1 K
D1               1.00000000 sec
TD0                       1
SFO1            399.9124696 MHz
NUC1                     1H
P0                   166.67 usec
P1                   500.00 usec
PLW1            31.62299919 W

F2 - Processing parameters
SI                    32768
SF              399.9100014 MHz
WDW                      EM
SSB       0
LB                     0.30 Hz
GB        0
PC                     1.00
```

^{13}C NMR spectrum of compound 12

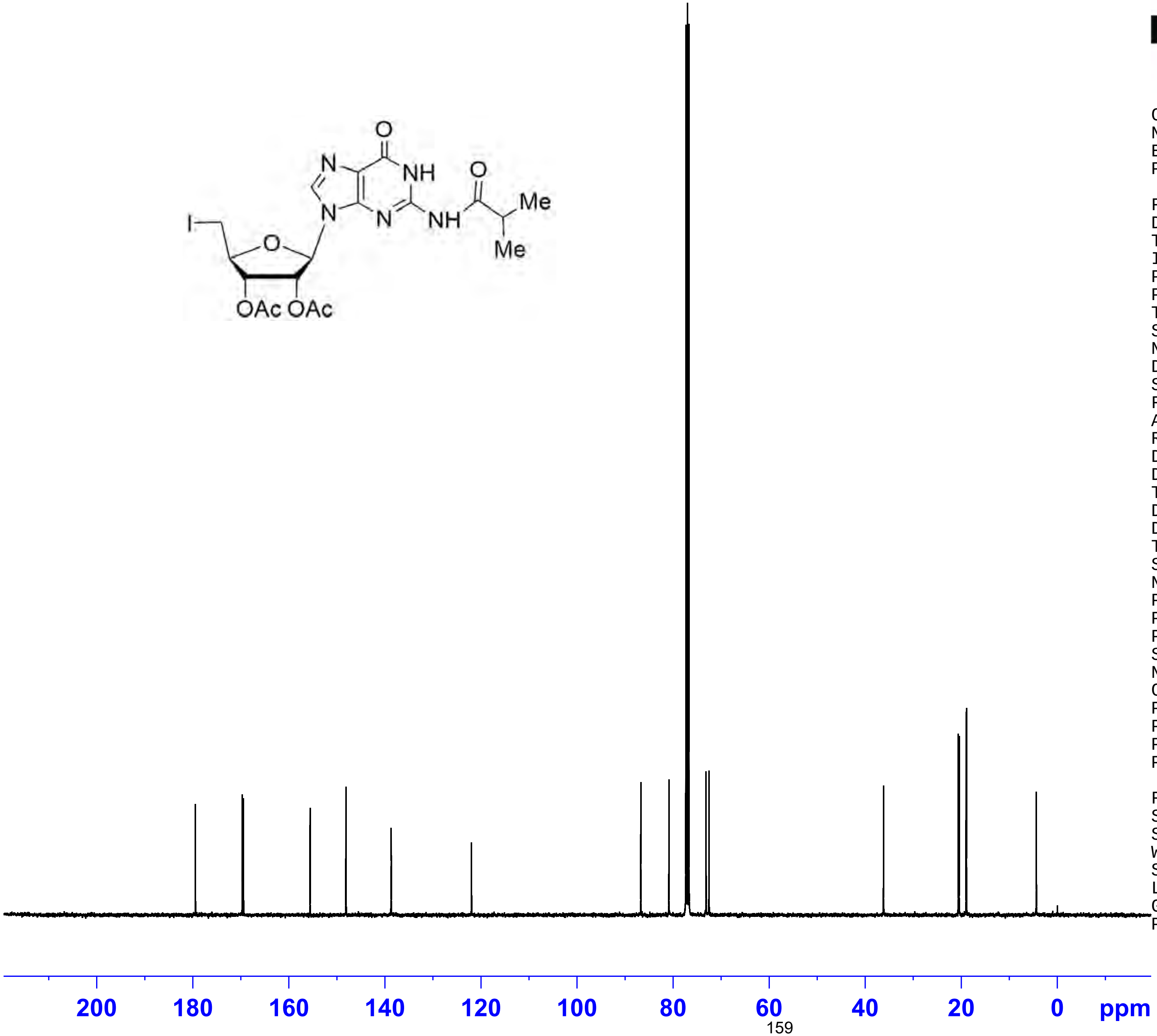

Current Data Parameters
NAME LH-II-74
EXPNO 11
PROCNO 1

F2 - Acquisition Parameters
Date_ 20230624
Time 11.03 h
INSTRUM AVIII_400
PROBHD Z108618_0146 (
PULPROG zgpg30
TD 96150
SOLVENT CDCl3
NS 3000
DS 4
SWH 24038.461 Hz
FIDRES 0.500020 Hz
AQ 1.9999200 sec
RG 2050
DW 20.800 usec
DE 6.50 usec
TE 300.0 K
D1 1.00000000 sec
D11 0.03000000 sec
TD0 1
SFO1 100.6178003 MHz
NUC1 13C
P0 2.90 usec
P1 8.70 usec
PLW1 96.68000031 W
SFO2 400.1116004 MHz
NUC2 1H
CPDPRG[2 waltz64
PCPD2 90.00 usec
PLW2 17.29199982 W
PLW12 0.48032999 W
PLW13 0.24160001 W

F2 - Processing parameters
SI 131072
SF 100.6077464 MHz
WDW EM
SSB 0
LB 1.00 Hz
GB 0
PC 1.40

Expanded region of the ^{13}C NMR spectrum of compound 12

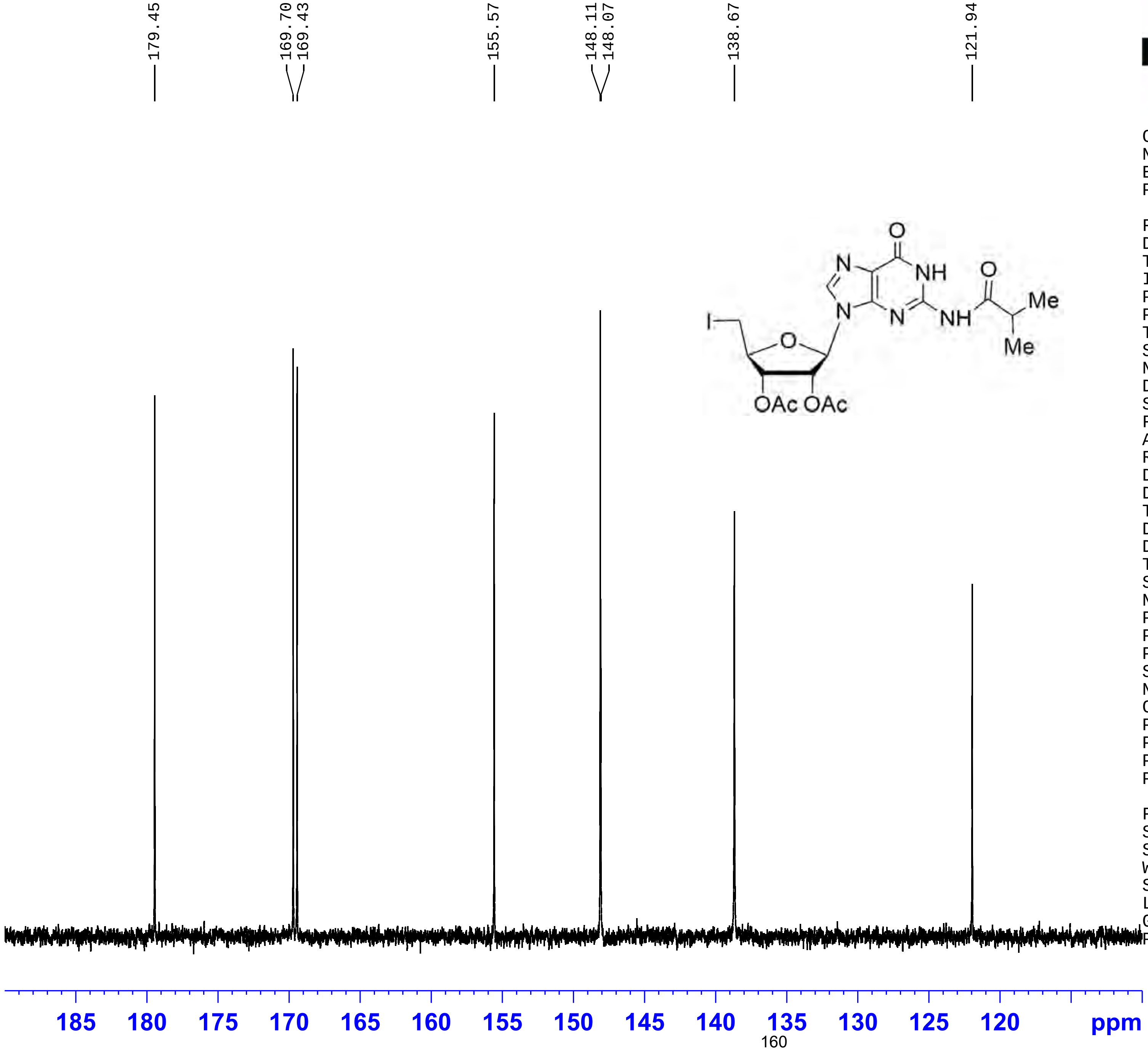

Current Data Parameters

NAME	LH-II-74
EXPNO	11
PROCNO	1

F2 - Acquisition Parameters

Date_	20230624
Time	11.03 h
INSTRUM	AVIII_400
PROBHD	Z108618_0146 (
PULPROG	zgpg30
TD	96150
SOLVENT	CDCl3
NS	3000
DS	4
SWH	24038.461 Hz
FIDRES	0.500020 Hz
AQ	1.9999200 sec
RG	2050
DW	20.800 usec
DE	6.50 usec
TE	300.0 K
D1	1.00000000 sec
D11	0.03000000 sec
TD0	1
SFO1	100.6178003 MHz
NUC1	13C
P0	2.90 usec
P1	8.70 usec
PLW1	96.68000031 W
SFO2	400.1116004 MHz
NUC2	1H
CPDPRG[2	waltz64
PCPD2	90.00 usec
PLW2	17.29199982 W
PLW12	0.48032999 W
PLW13	0.24160001 W

F2 - Processing parameters

SI	131072
SF	100.6077464 MHz
WDW	EM
SSB	0
LB	1.00 Hz
GB	0
PC	1.40

Expanded region of the ^{13}C NMR spectrum of compound 12

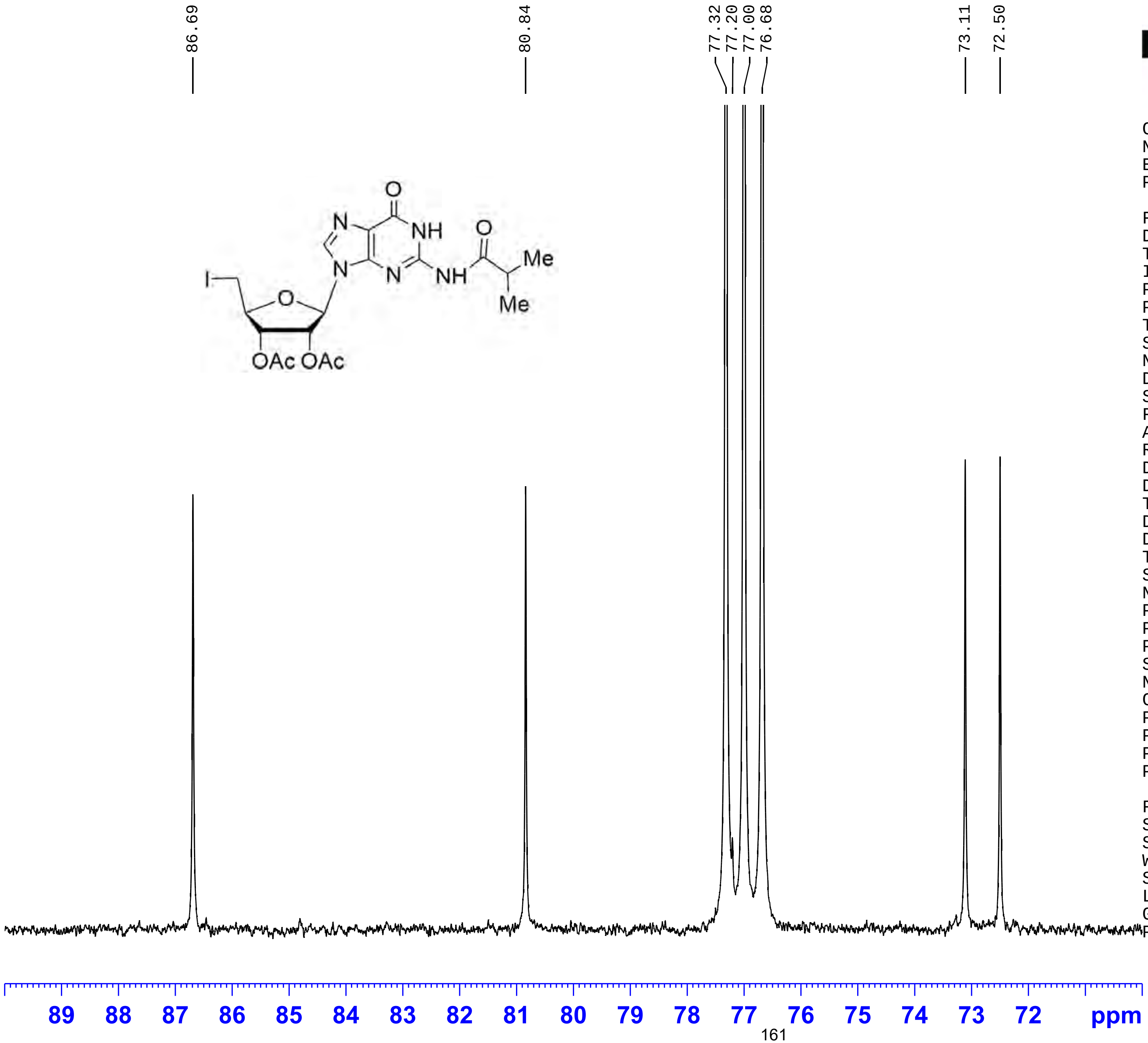


```
Current Data Parameters
NAME              LH-II-74
EXPNO                   11
PROCNO                   1

F2 - Acquisition Parameters
Date_             20230624
Time                 11.03 h
INSTRUM          AVIII_400
PROBHD   Z108618_0146 (
PULPROG            zgpg30
TD                  96150
SOLVENT             CDCl3
NS                   3000
DS                      4
SWH             24038.461 Hz
FIDRES           0.500020 Hz
AQ              1.9999200 sec
RG                   2050
DW                 20.800 usec
DE                   6.50 usec
TE                  300.0 K
D1             1.00000000 sec
D11            0.03000000 sec
TD0                     1
SFO1          100.6178003 MHz
NUC1                  13C
P0                   2.90 usec
P1                   8.70 usec
PLW1          96.68000031 W
SFO2          400.1116004 MHz
NUC2                   1H
CPDPRG[2          waltz64
PCPD2               90.00 usec
PLW2          17.29199982 W
PLW12          0.48032999 W
PLW13          0.24160001 W

F2 - Processing parameters
SI                 131072
SF            100.6077464 MHz
WDW                    EM
SSB      0
LB                   1.00 Hz
GB       0
PC                   1.40
```

Expanded region of the ^{13}C NMR spectrum of compound 12

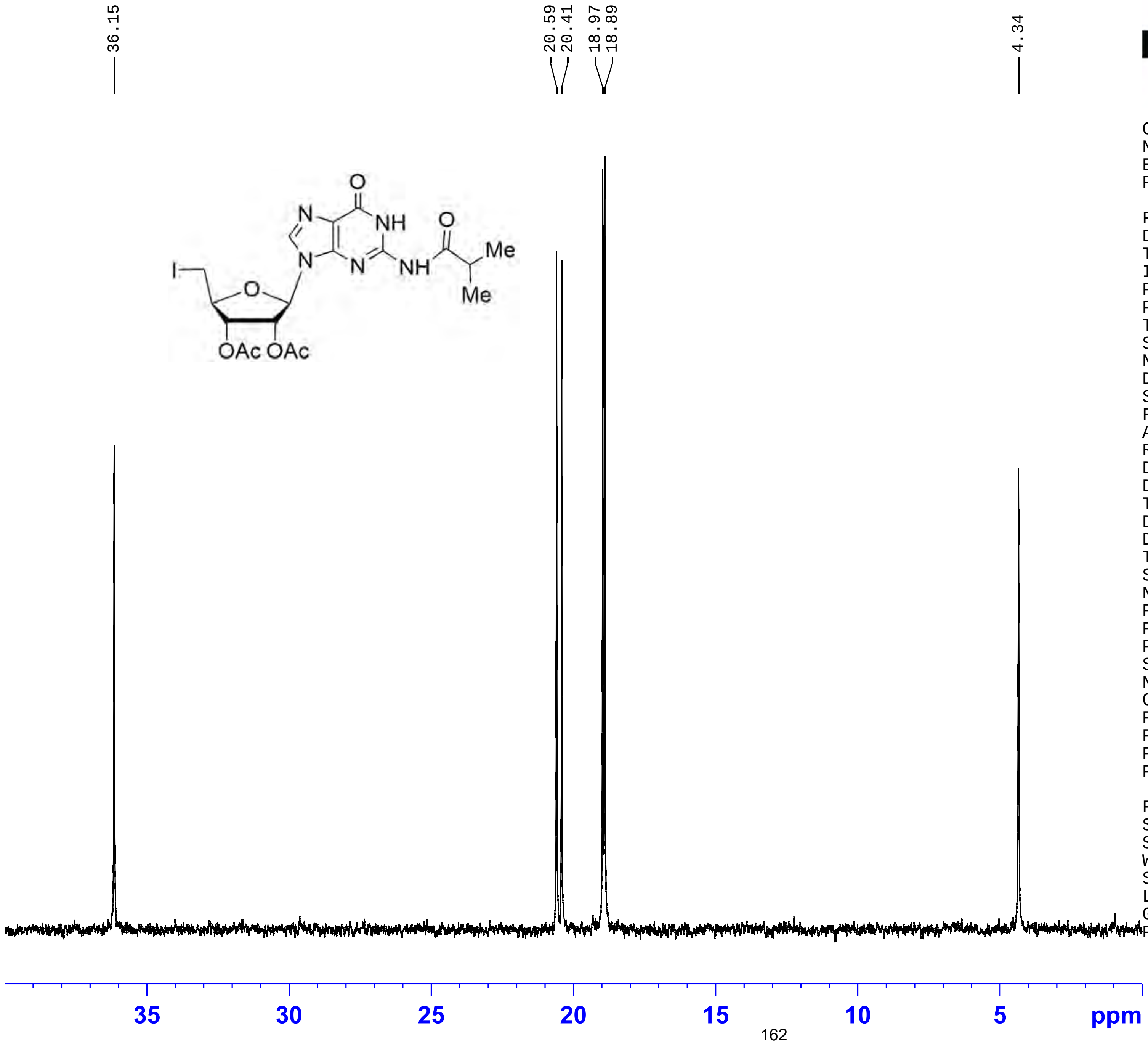

Current Data Parameters
NAME LH-II-74
EXPNO 11
PROCNO 1

F2 - Acquisition Parameters
Date_ 20230624
Time 11.03 h
INSTRUM AVIII_400
PROBHD Z108618_0146 (
PULPROG zgpg30
TD 96150
SOLVENT CDCl3
NS 3000
DS 4
SWH 24038.461 Hz
FIDRES 0.500020 Hz
AQ 1.9999200 sec
RG 2050
DW 20.800 usec
DE 6.50 usec
TE 300.0 K
D1 1.00000000 sec
D11 0.03000000 sec
TD0 1
SFO1 100.6178003 MHz
NUC1 13C
P0 2.90 usec
P1 8.70 usec
PLW1 96.68000031 W
SFO2 400.1116004 MHz
NUC2 1H
CPDPRG[2 waltz64
PCPD2 90.00 usec
PLW2 17.29199982 W
PLW12 0.48032999 W
PLW13 0.24160001 W

F2 - Processing parameters
SI 131072
SF 100.6077464 MHz
WDW EM
SSB 0
LB 1.00 Hz
GB 0
PC 1.40

^{13}C DEPT-135 NMR spectrum of compound 12

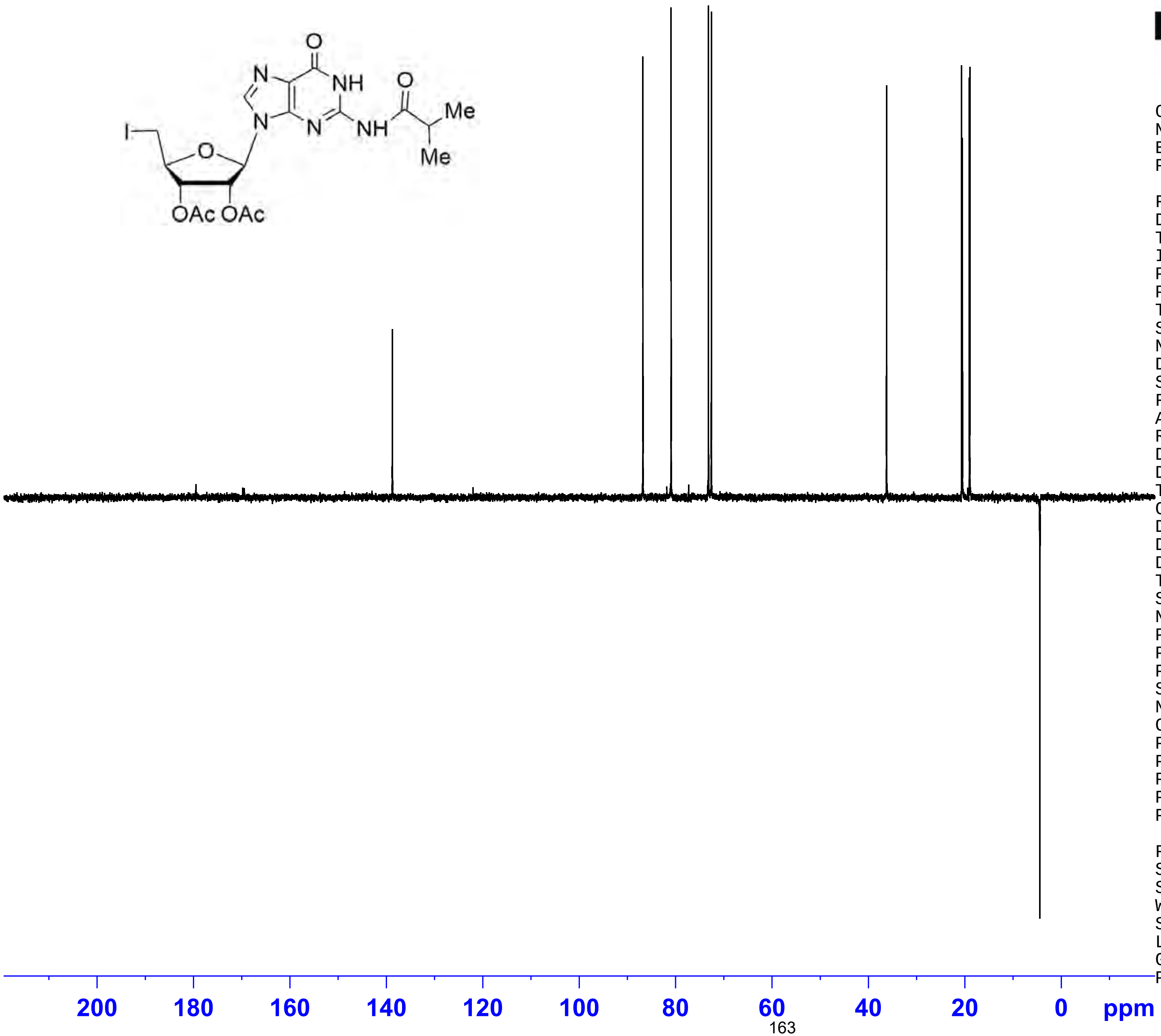

Current Data Parameters
NAME LH-II-74
EXPNO 12
PROCNO 1

F2 - Acquisition Parameters
Date_ 20230624
Time 12.32 h
INSTRUM AVIII_400
PROBHD Z108618_0146 (
PULPROG dept135
TD 65536
SOLVENT CDCl3
NS 1500
DS 4
SWH 24038.461 Hz
FIDRES 0.733596 Hz
AQ 1.3631488 sec
RG 2050
DW 20.800 usec
DE 6.50 usec
TE 300.0 K
CNST2 145.0000000
D1 2.00000000 sec
D2 0.00344828 sec
D12 0.00002000 sec
TD0 1
SFO1 100.6178003 MHz
NUC1 13C
P1 8.70 usec
P2 17.40 usec
PLW1 96.68000031 W
SFO2 400.1116004 MHz
NUC2 1H
CPDPRG[2 waltz64
P3 15.00 usec
P4 30.00 usec
PCPD2 90.00 usec
PLW2 17.29199982 W
PLW12 0.48032999 W

F2 - Processing parameters
SI 32768
SF 100.6077400 MHz
WDW EM
SSB 0
LB 1.00 Hz
GB 0
PC 1.40

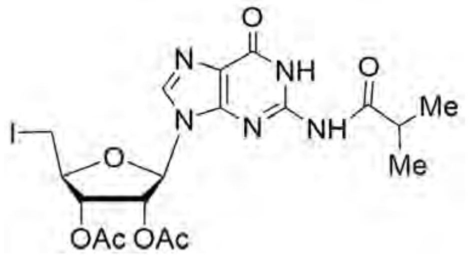

^{1}H-^{1}H COSY NMR spectrum of compound 12

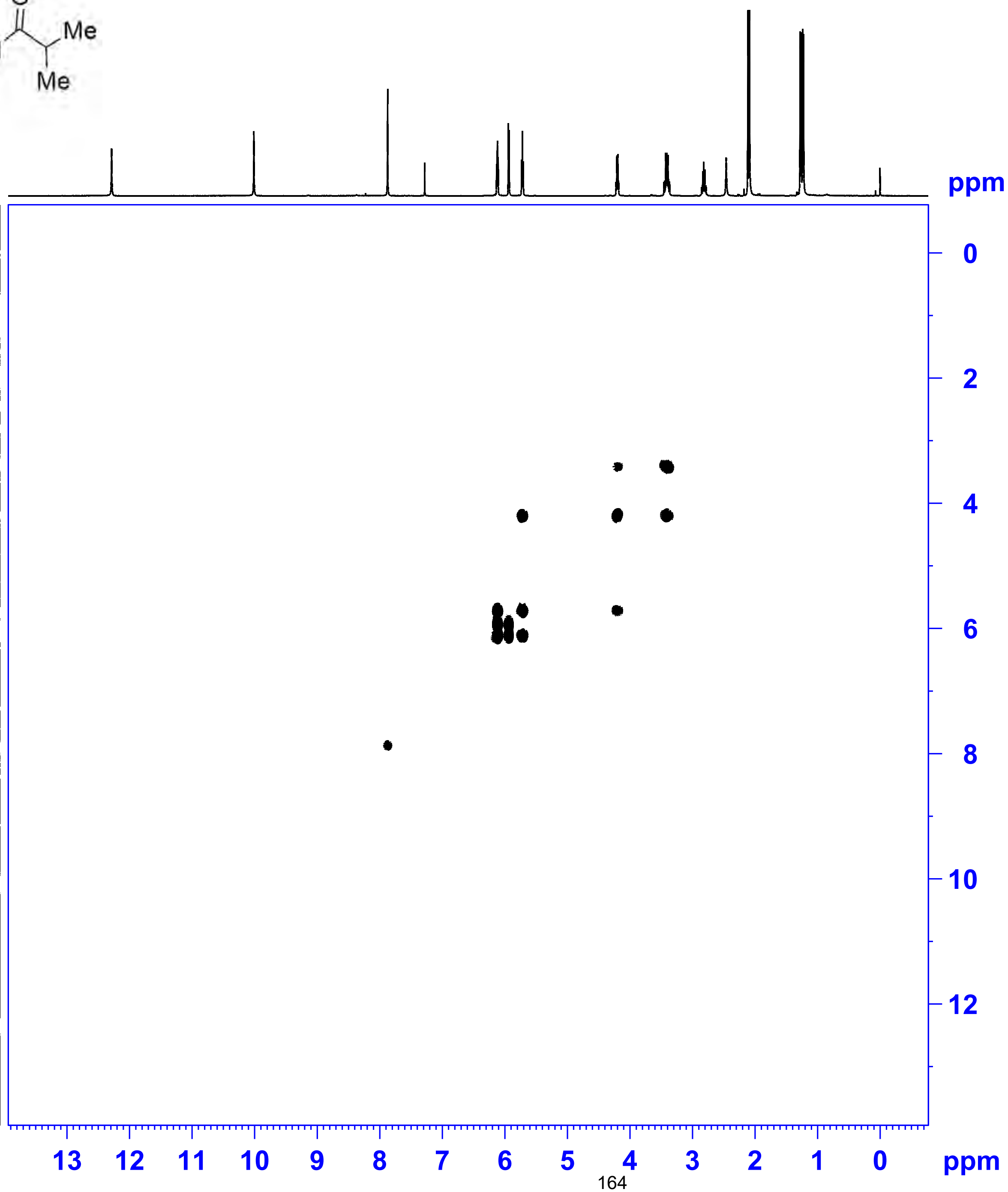


```
Current Data Parameters
NAME      LH-II-74 NEW NMR
EXPNO                   11
PROCNO                   1

F2 - Acquisition Parameters
Date_             20230624
Time                 13.42 h
INSTRUM           AVIII_400
PROBHD      Z108618_0817 (
PULPROG          cosygpmfqf
TD                    2048
SOLVENT              CDCl3
NS                       3
DS                       8
SWH               5882.353 Hz
FIDRES            5.744485 Hz
AQ               0.1740800 sec
RG                    1620
DW                  85.000 usec
DE                    6.50 usec
TE                   296.1 K
D0              0.00000300 sec
D1              2.01788306 sec
D13             0.00000400 sec
D16             0.00020000 sec
IN0             0.00017000 sec
TDav                     1
SFO1           399.9126345 MHz
NUC1                    1H
P1                  500.00 usec
PLW1           31.62299919 W
GPNAM[1]          SINE.100
GPZ1                 16.00 %
GPNAM[2]          SINE.100
GPZ2                 12.00 %
GPNAM[3]          SINE.100
GPZ3                 40.00 %
P16                1000.00 usec

F1 - Acquisition parameters
TD                     256
SFO1              399.9126 MHz
FIDRES           45.955883 Hz
SW                  14.709 ppm
FnMODE                  QF

F2 - Processing parameters
SI                    1024
SF             399.9100014 MHz
WDW                   SINE
SSB       0
LB        0 Hz
GB        0
PC                    1.40

F1 - Processing parameters
SI                    1024
MC2                     QF
SF             399.9100014 MHz
WDW                   SINE
SSB       0
LB        0 Hz
GB        0
```

Expanded region of ^1H-^1H COSY NMR spectrum of compound 12

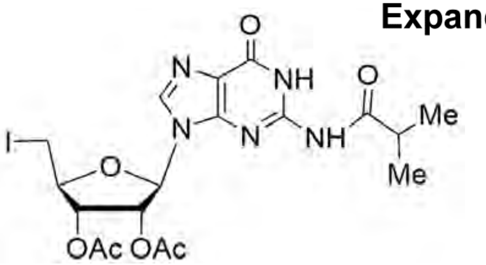

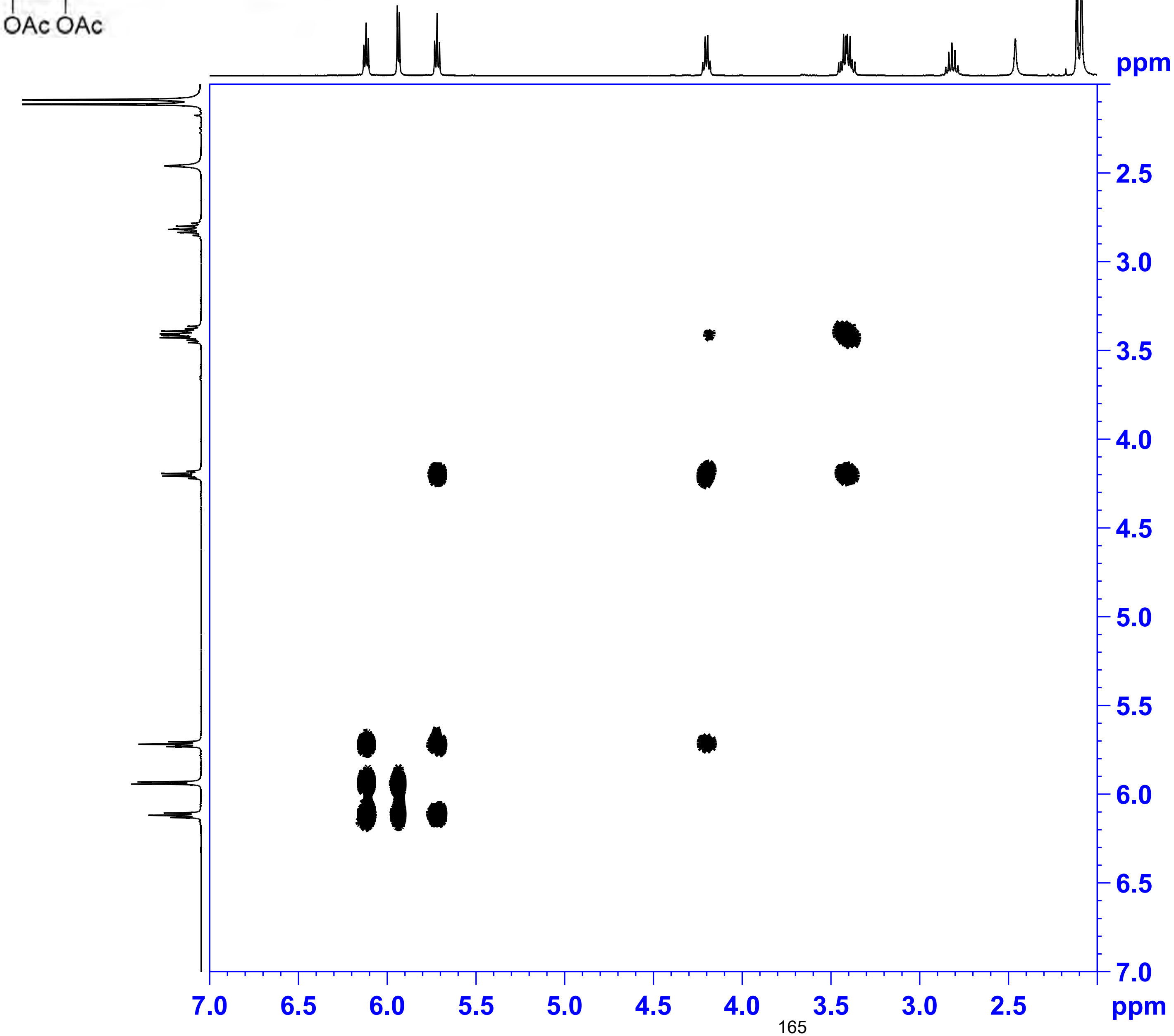


```
Current Data Parameters
NAME      LH-II-74 NEW NMR
EXPNO                   11
PROCNO                   1

F2 - Acquisition Parameters
Date_             20230624
Time                 13.42 h
INSTRUM          AVIII_400
PROBHD    Z108618_0817 (
PULPROG          cosygpmfqf
TD                    2048
SOLVENT              CDCl3
NS                       3
DS                       8
SWH               5882.353 Hz
FIDRES            5.744485 Hz
AQ               0.1740800 sec
RG                    1620
DW                  85.000 usec
DE                    6.50 usec
TE                   296.1 K
D0             0.00000300 sec
D1             2.01788306 sec
D13            0.00000400 sec
D16            0.00020000 sec
IN0            0.00017000 sec
TDav                     1
SFO1           399.9126345 MHz
NUC1                    1H
P1                  500.00 usec
PLW1           31.62299919 W
GPNAM[1]         SINE.100
GPZ1                16.00 %
GPNAM[2]         SINE.100
GPZ2                12.00 %
GPNAM[3]         SINE.100
GPZ3                40.00 %
P16               1000.00 usec

F1 - Acquisition parameters
TD                     256
SFO1              399.9126 MHz
FIDRES           45.955883 Hz
SW                  14.709 ppm
FnMODE                  QF

F2 - Processing parameters
SI                    1024
SF             399.9100014 MHz
WDW                   SINE
SSB      0
LB       0 Hz
GB       0
PC                    1.40

F1 - Processing parameters
SI                    1024
MC2                     QF
SF             399.9100014 MHz
WDW                   SINE
SSB      0
LB       0 Hz
GB       0
```

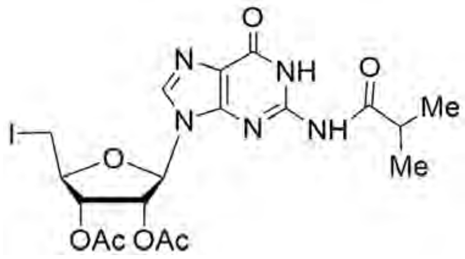

^{1}H-^{13}C HSQC NMR spectrum of compound 12

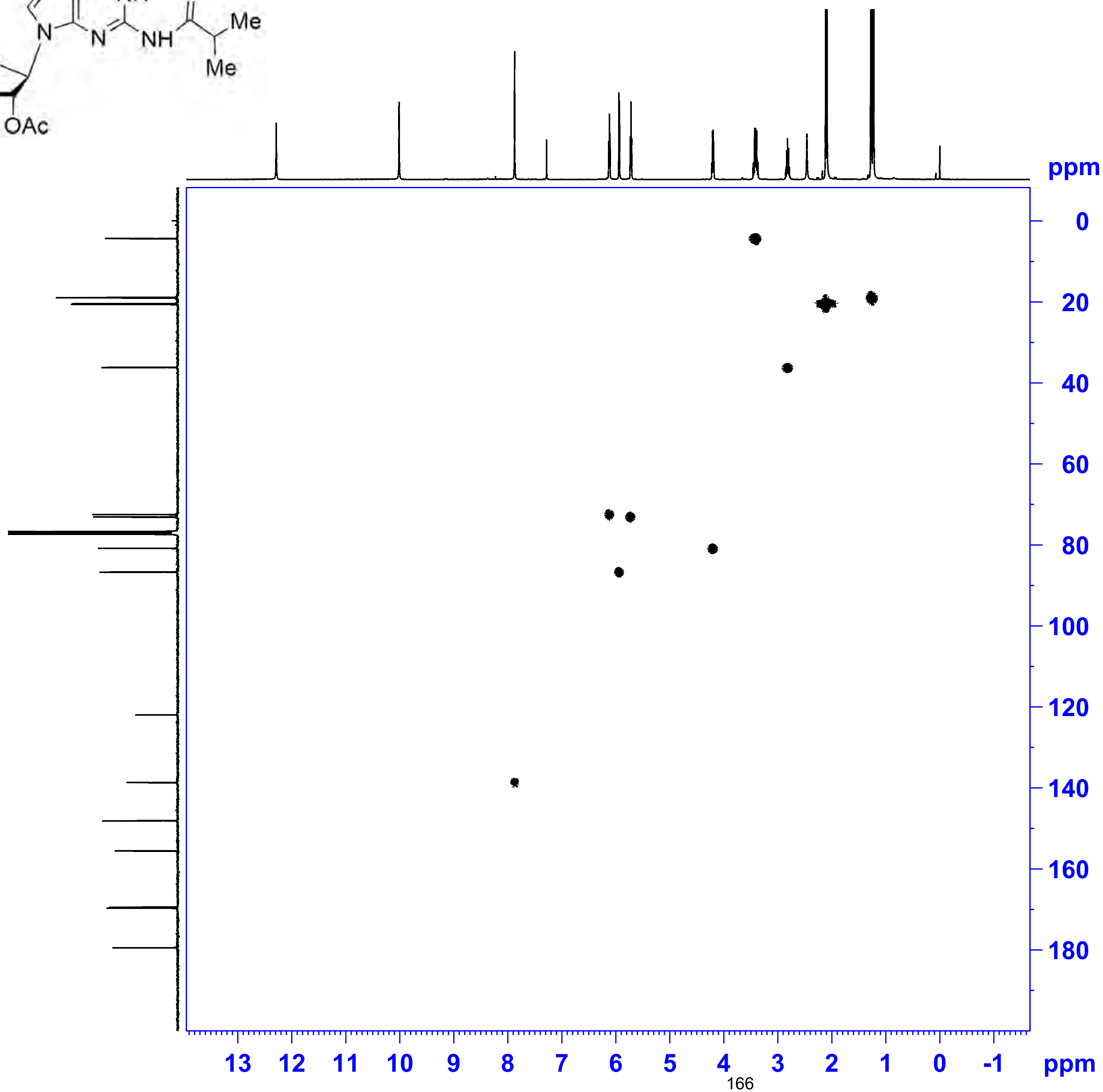


```
Current Data Parameters
NAME              LH-II-74
EXPNO                   13
PROCNO                   1

F2 - Acquisition Parameters
Date_             20230624
Time                 13.01 h
INSTRUM           AVIII_400
PROBHD   Z108618_0146 (
PULPROG          hsqcedetgp
TD                    1024
SOLVENT              CDCl3
NS                       4
DS                      16
SWH               6250.000 Hz
FIDRES           12.207031 Hz
AQ               0.0819200 sec
RG                    1290
DW                  80.000 usec
DE                    6.50 usec
TE                   300.0 K
CNST2        145.0000000
D0            0.00000300 sec
D1            1.45965397 sec
D4            0.00172414 sec
D11           0.03000000 sec
D16           0.00020000 sec
D21           0.00345000 sec
IN0           0.00003000 sec
TDav                     1
ZGOPTNS
SFO1        400.1124568 MHz
NUC1                    1H
P1                   15.00 usec
P2                   30.00 usec
PLW1          17.29199982 W
SFO2        100.6152434 MHz
NUC2                   13C
CPDPRG[2              garp
P3                    8.70 usec
P4                   17.40 usec
PCPD2                56.50 usec
PLW2          96.68000031 W
PLW12          3.16230011 W
GPNAM[1]         SMSQ10.100
GPZ1                 80.00 %
GPNAM[2]         SMSQ10.100
GPZ2                 20.10 %
P16                1000.00 usec

F1 - Acquisition parameters
TD                     256
SFO1              100.6152 MHz
FIDRES          130.208328 Hz
SW                 165.648 ppm
FnMODE        Echo-Antiecho

F2 - Processing parameters
SI                    1024
SF           400.1099995 MHz
WDW                  QSINE
SSB                      2
LB                0 Hz
GB                0
PC                    1.40

F1 - Processing parameters
SI                    1024
MC2            echo-antiecho
SF           100.6077400 MHz
WDW                  QSINE
SSB                      2
LB                0 Hz
GB                0
```

^{1}H NMR spectrum of compound 11

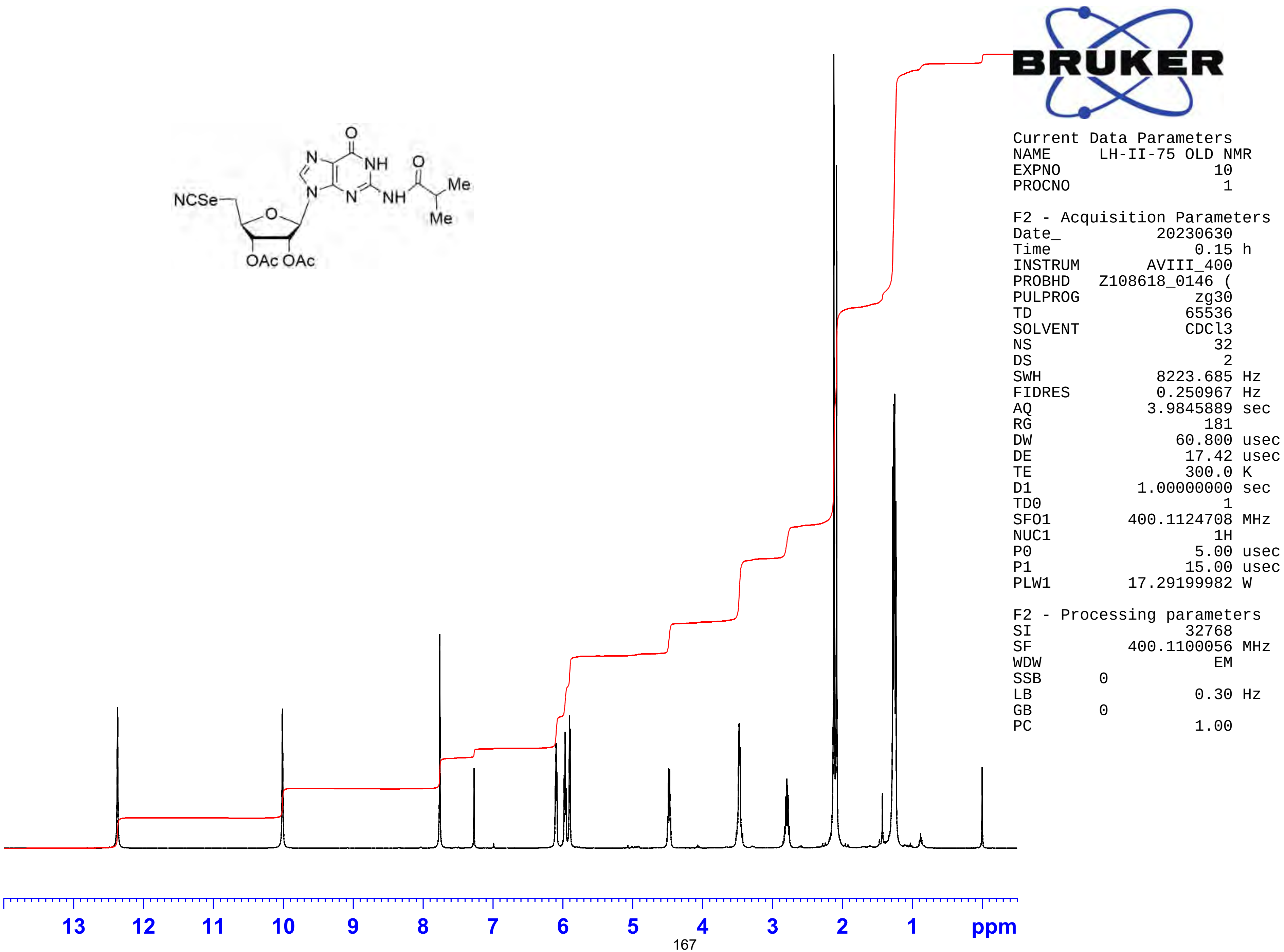

Expanded region of the ^{1}H NMR spectrum of compound 11

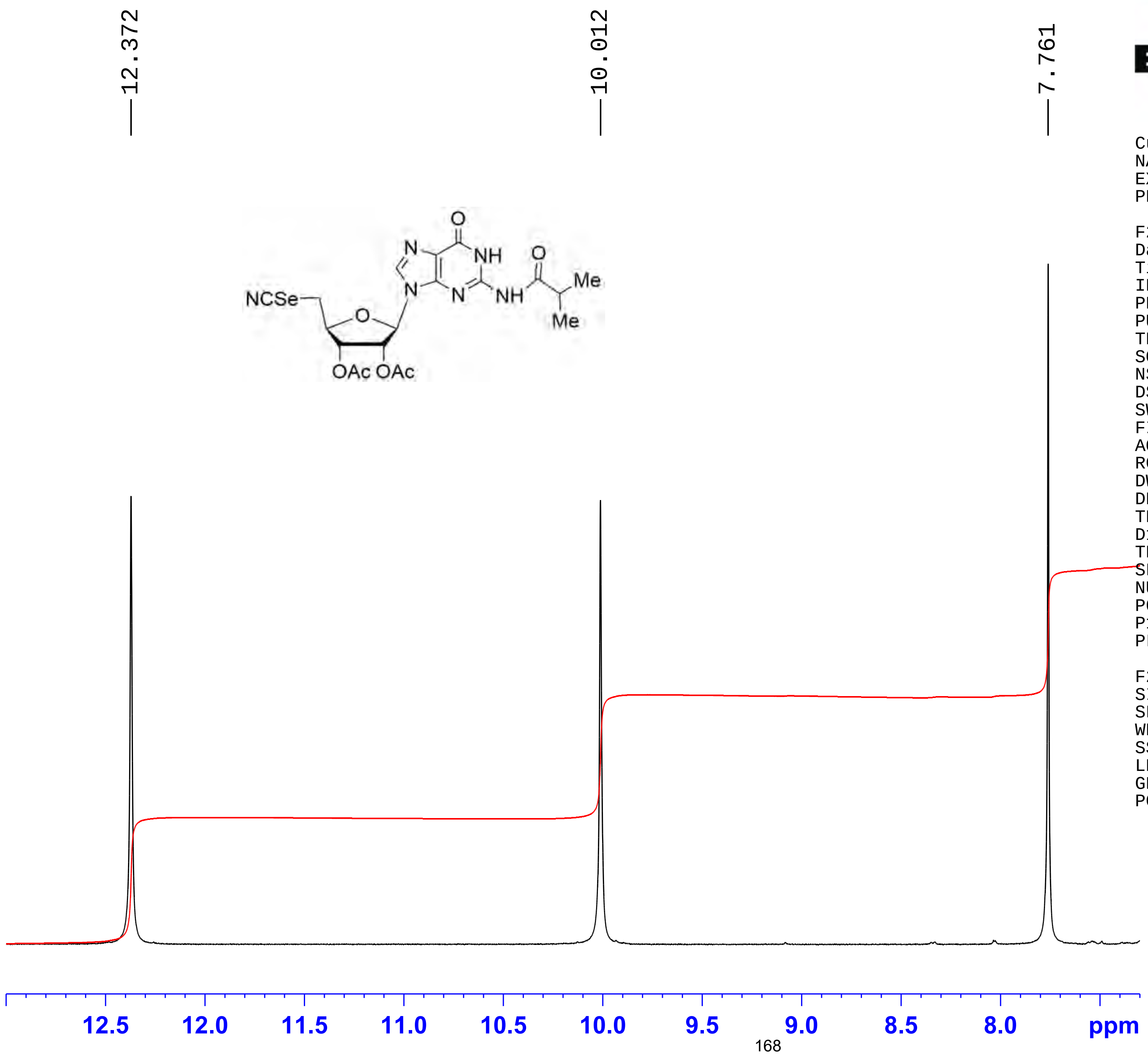


```
Current Data Parameters
NAME       LH-II-75 OLD NMR
EXPNO                    10
PROCNO                    1

F2 - Acquisition Parameters
Date_              20230630
Time                   0.15 h
INSTRUM           AVIII_400
PROBHD    Z108618_0146 (
PULPROG                zg30
TD                    65536
SOLVENT               CDCl3
NS                       32
DS                        2
SWH                8223.685 Hz
FIDRES             0.250967 Hz
AQ                3.9845889 sec
RG                      181
DW                   60.800 usec
DE                    17.42 usec
TE                    300.0 K
D1               1.00000000 sec
TD0                       1
SFO1            400.1124708 MHz
NUC1                     1H
P0                     5.00 usec
P1                    15.00 usec
PLW1            17.29199982 W

F2 - Processing parameters
SI                    32768
SF              400.1100056 MHz
WDW                      EM
SSB       0
LB                     0.30 Hz
GB        0
PC                     1.00
```

Expanded region of the ^{1}H NMR spectrum of compound 11

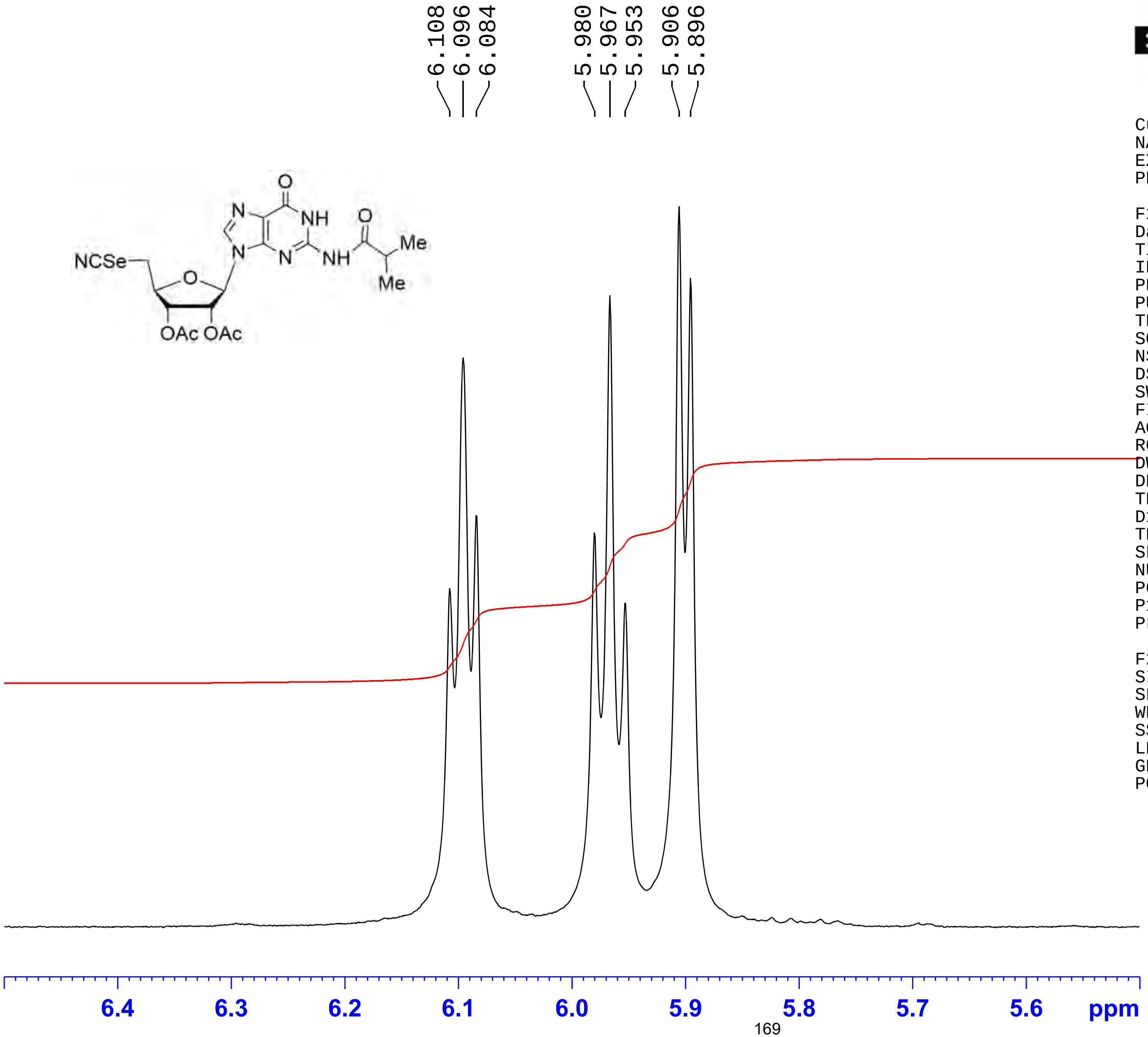

Current Data Parameters
NAME LH-II-75 OLD NMR
EXPNO 10
PROCNO 1

F2 - Acquisition Parameters
Date_ 20230630
Time 0.15 h
INSTRUM AVIII_400
PROBHD Z108618_0146 (
PULPROG zg30
TD 65536
SOLVENT CDCl3
NS 32
DS 2
SWH 8223.685 Hz
FIDRES 0.250967 Hz
AQ 3.9845889 sec
RG 181
DW 60.800 usec
DE 17.42 usec
TE 300.0 K
D1 1.00000000 sec
TD0 1
SFO1 400.1124708 MHz
NUC1 1H
P0 5.00 usec
P1 15.00 usec
PLW1 17.29199982 W

F2 - Processing parameters
SI 32768
SF 400.1100056 MHz
WDW EM
SSB 0
LB 0.30 Hz
GB 0
PC 1.00

Expanded region of the ^{1}H NMR spectrum of compound 11

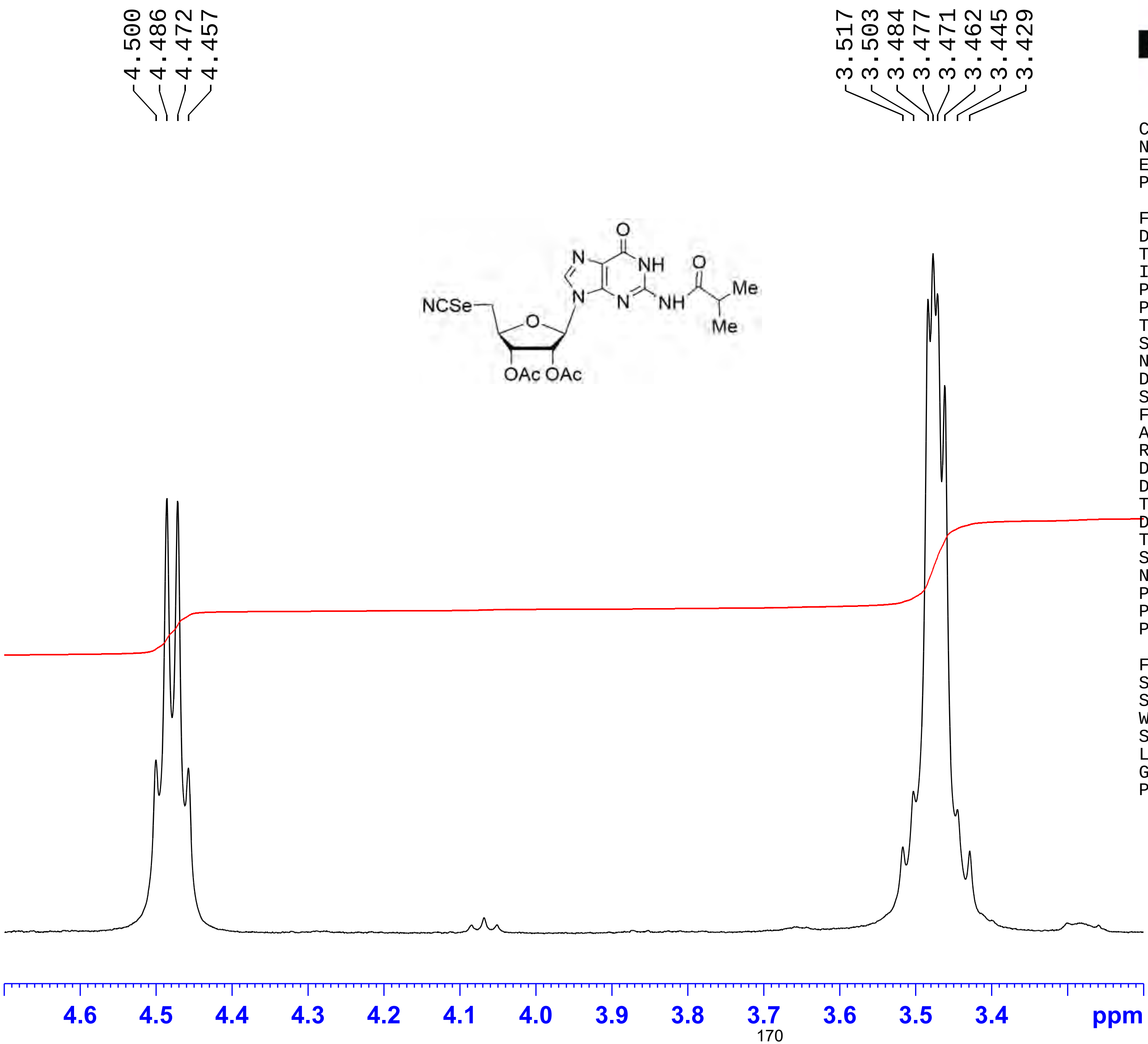

Current Data Parameters
NAME LH-II-75 OLD NMR
EXPNO 10
PROCNO 1

F2 - Acquisition Parameters
Date_ 20230630
Time 0.15 h
INSTRUM AVIII_400
PROBHD Z108618_0146 (
PULPROG zg30
TD 65536
SOLVENT CDCl3
NS 32
DS 2
SWH 8223.685 Hz
FIDRES 0.250967 Hz
AQ 3.9845889 sec
RG 181
DW 60.800 usec
DE 17.42 usec
TE 300.0 K
D1 1.00000000 sec
TD0 1
SFO1 400.1124708 MHz
NUC1 1H
P0 5.00 usec
P1 15.00 usec
PLW1 17.29199982 W

F2 - Processing parameters
SI 32768
SF 400.1100056 MHz
WDW EM
SSB 0
LB 0.30 Hz
GB 0
PC 1.00

Expanded region of the ^{1}H NMR spectrum of compound 11

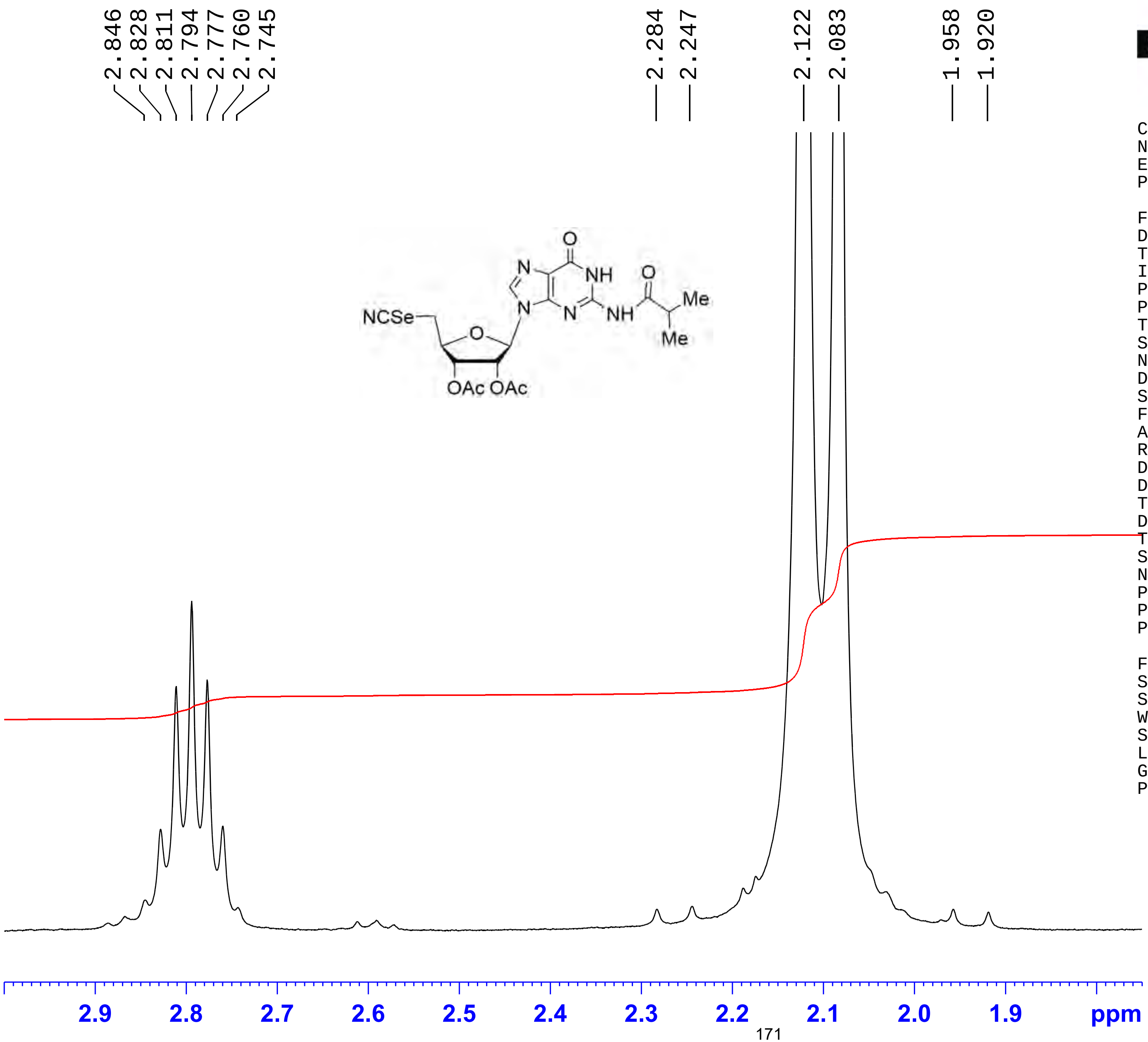

Current Data Parameters
NAME LH-II-75 OLD NMR
EXPNO 10
PROCNO 1

F2 - Acquisition Parameters
Date_ 20230630
Time 0.15 h
INSTRUM AVIII_400
PROBHD Z108618_0146 (
PULPROG zg30
TD 65536
SOLVENT CDCl3
NS 32
DS 2
SWH 8223.685 Hz
FIDRES 0.250967 Hz
AQ 3.9845889 sec
RG 181
DW 60.800 usec
DE 17.42 usec
TE 300.0 K
D1 1.00000000 sec
TD0 1
SFO1 400.1124708 MHz
NUC1 1H
P0 5.00 usec
P1 15.00 usec
PLW1 17.29199982 W

F2 - Processing parameters
SI 32768
SF 400.1100056 MHz
WDW EM
SSB 0
LB 0.30 Hz
GB 0
PC 1.00

Expanded region of the ^{1}H NMR spectrum of compound 11

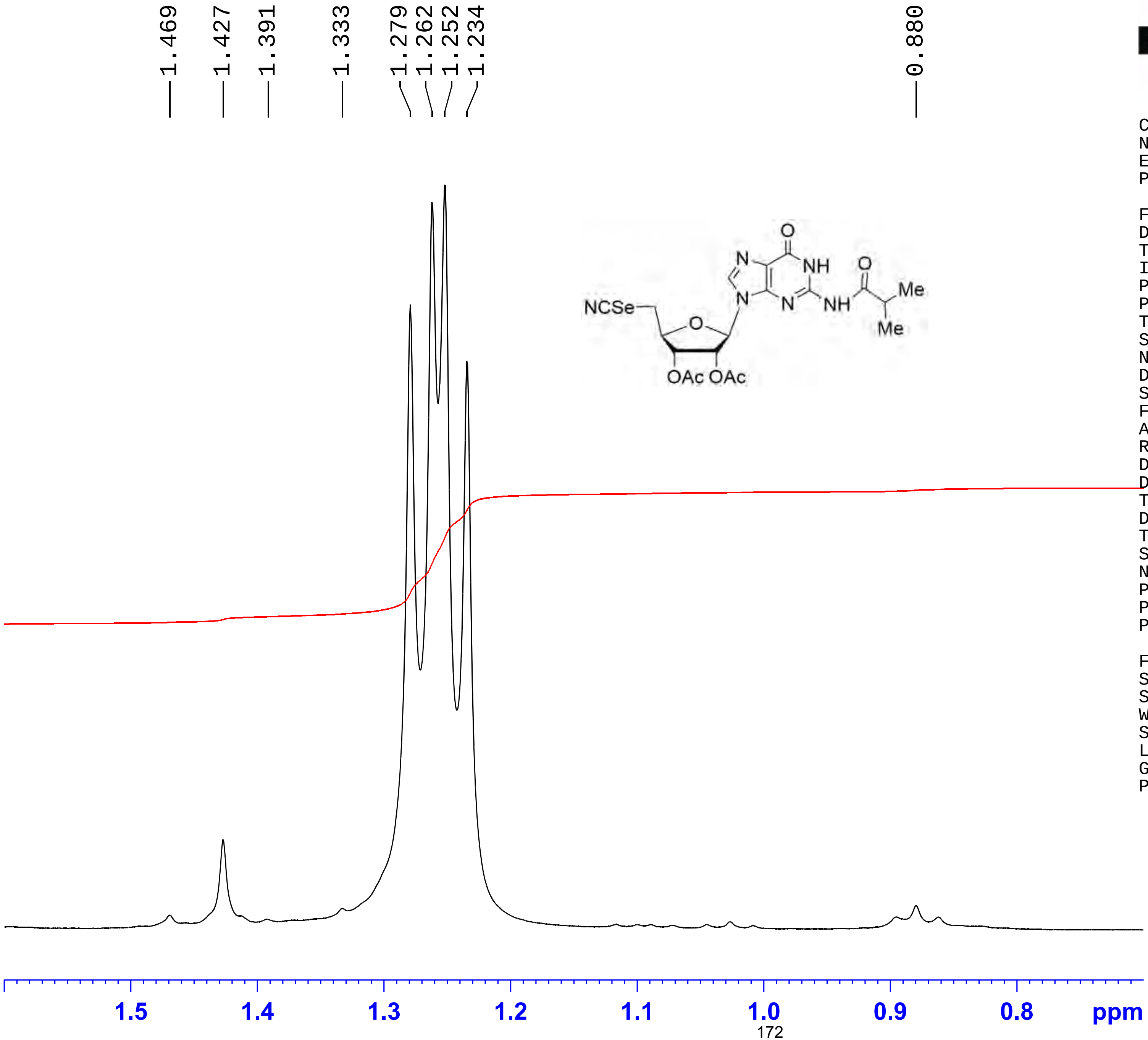

Current Data Parameters
NAME LH-II-75 OLD NMR
EXPNO 10
PROCNO 1

F2 - Acquisition Parameters
Date_ 20230630
Time 0.15 h
INSTRUM AVIII_400
PROBHD Z108618_0146 (
PULPROG zg30
TD 65536
SOLVENT CDCl3
NS 32
DS 2
SWH 8223.685 Hz
FIDRES 0.250967 Hz
AQ 3.9845889 sec
RG 181
DW 60.800 usec
DE 17.42 usec
TE 300.0 K
D1 1.00000000 sec
TD0 1
SFO1 400.1124708 MHz
NUC1 1H
P0 5.00 usec
P1 15.00 usec
PLW1 17.29199982 W

F2 - Processing parameters
SI 32768
SF 400.1100056 MHz
WDW EM
SSB 0
LB 0.30 Hz
GB 0
PC 1.00

^{13}C NMR spectrum of compound 11

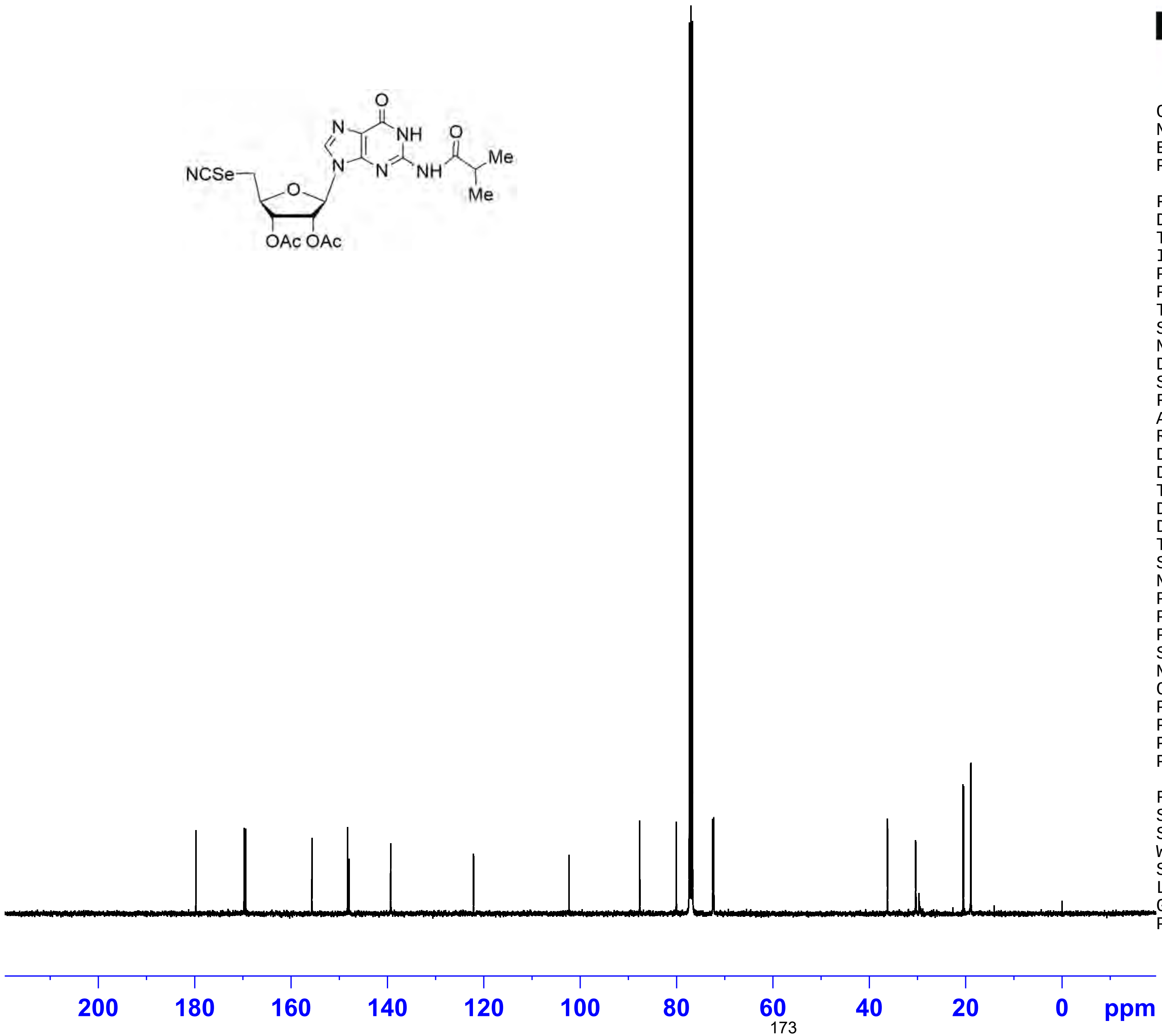


```
Current Data Parameters
NAME       LH-II-75 OLD NMR
EXPNO                    11
PROCNO                    1

F2 - Acquisition Parameters
Date_              20230630
Time                   2.52 h
INSTRUM           AVIII_400
PROBHD     Z108618_0146 (
PULPROG             zgpg30
TD                    96150
SOLVENT               CDCl3
NS                     3000
DS                        4
SWH               24038.461 Hz
FIDRES             0.500020 Hz
AQ                1.9999200 sec
RG                     2050
DW                   20.800 usec
DE                     6.50 usec
TE                    300.0 K
D1               1.00000000 sec
D11              0.03000000 sec
TD0                       1
SFO1            100.6178003 MHz
NUC1                    13C
P0                     2.90 usec
P1                     8.70 usec
PLW1            96.68000031 W
SFO2            400.1116004 MHz
NUC2                     1H
CPDPRG[2            waltz64
PCPD2                 90.00 usec
PLW2            17.29199982 W
PLW12            0.48032999 W
PLW13            0.24160001 W

F2 - Processing parameters
SI                   131072
SF              100.6077445 MHz
WDW                      EM
SSB        0
LB                     1.00 Hz
GB         0
PC                     1.40
```

Expanded region of the ^{13}C NMR spectrum of compound 11

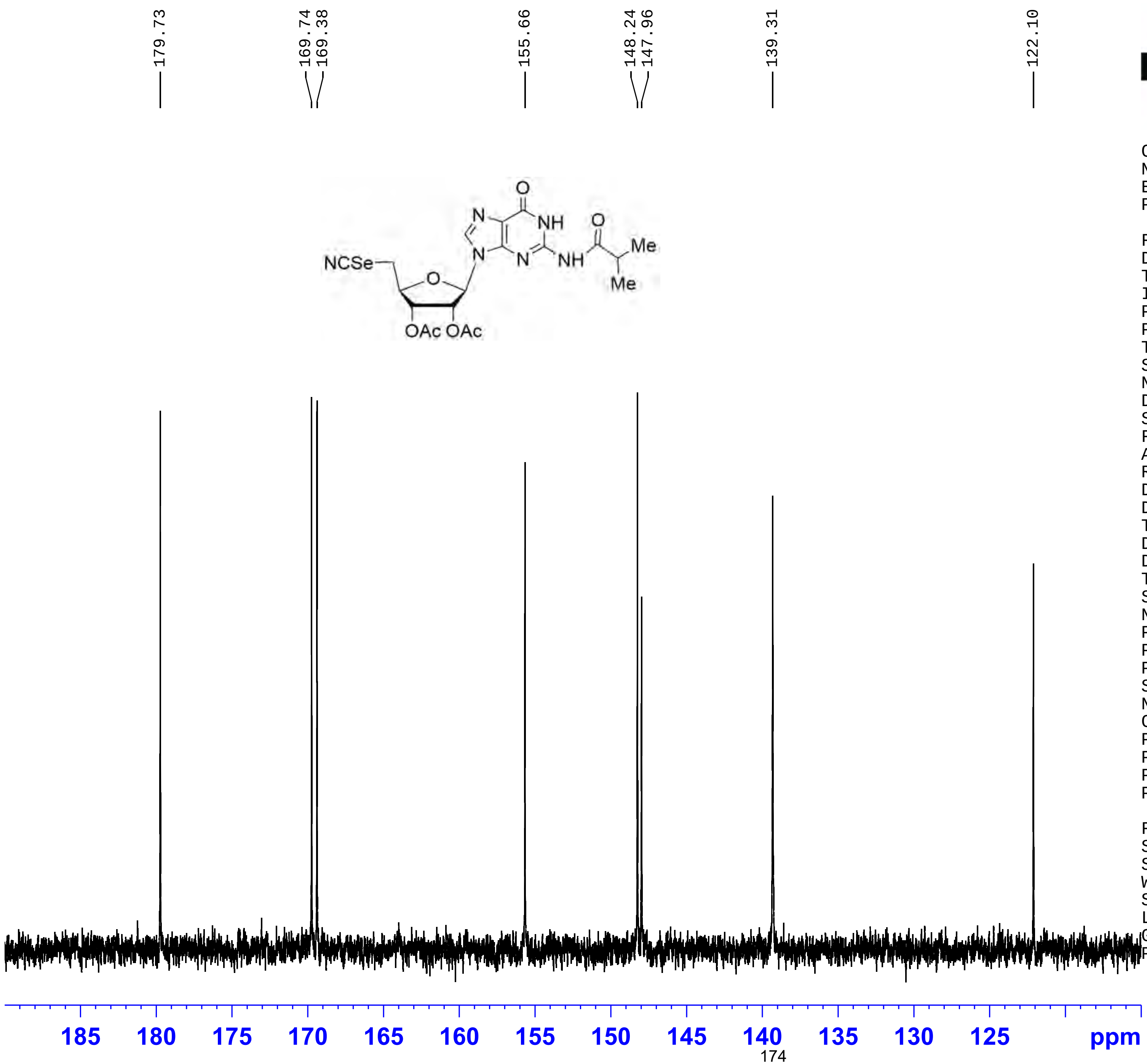


```
Current Data Parameters
NAME       LH-II-75 OLD NMR
EXPNO                    11
PROCNO                    1

F2 - Acquisition Parameters
Date_              20230630
Time                   2.52 h
INSTRUM           AVIII_400
PROBHD    Z108618_0146 (
PULPROG             zgpg30
TD                    96150
SOLVENT               CDCl3
NS                     3000
DS                        4
SWH               24038.461 Hz
FIDRES             0.500020 Hz
AQ                1.9999200 sec
RG                     2050
DW                   20.800 usec
DE                     6.50 usec
TE                    300.0 K
D1               1.00000000 sec
D11              0.03000000 sec
TD0                       1
SFO1            100.6178003 MHz
NUC1                    13C
P0                     2.90 usec
P1                     8.70 usec
PLW1            96.68000031 W
SFO2            400.1116004 MHz
NUC2                     1H
CPDPRG[2            waltz64
PCPD2                 90.00 usec
PLW2            17.29199982 W
PLW12            0.48032999 W
PLW13            0.24160001 W

F2 - Processing parameters
SI                   131072
SF              100.6077445 MHz
WDW                      EM
SSB          0
LB                     1.00 Hz
GB           0
PC                     1.40
```

Expanded region of the ^{13}C NMR spectrum of compound 11

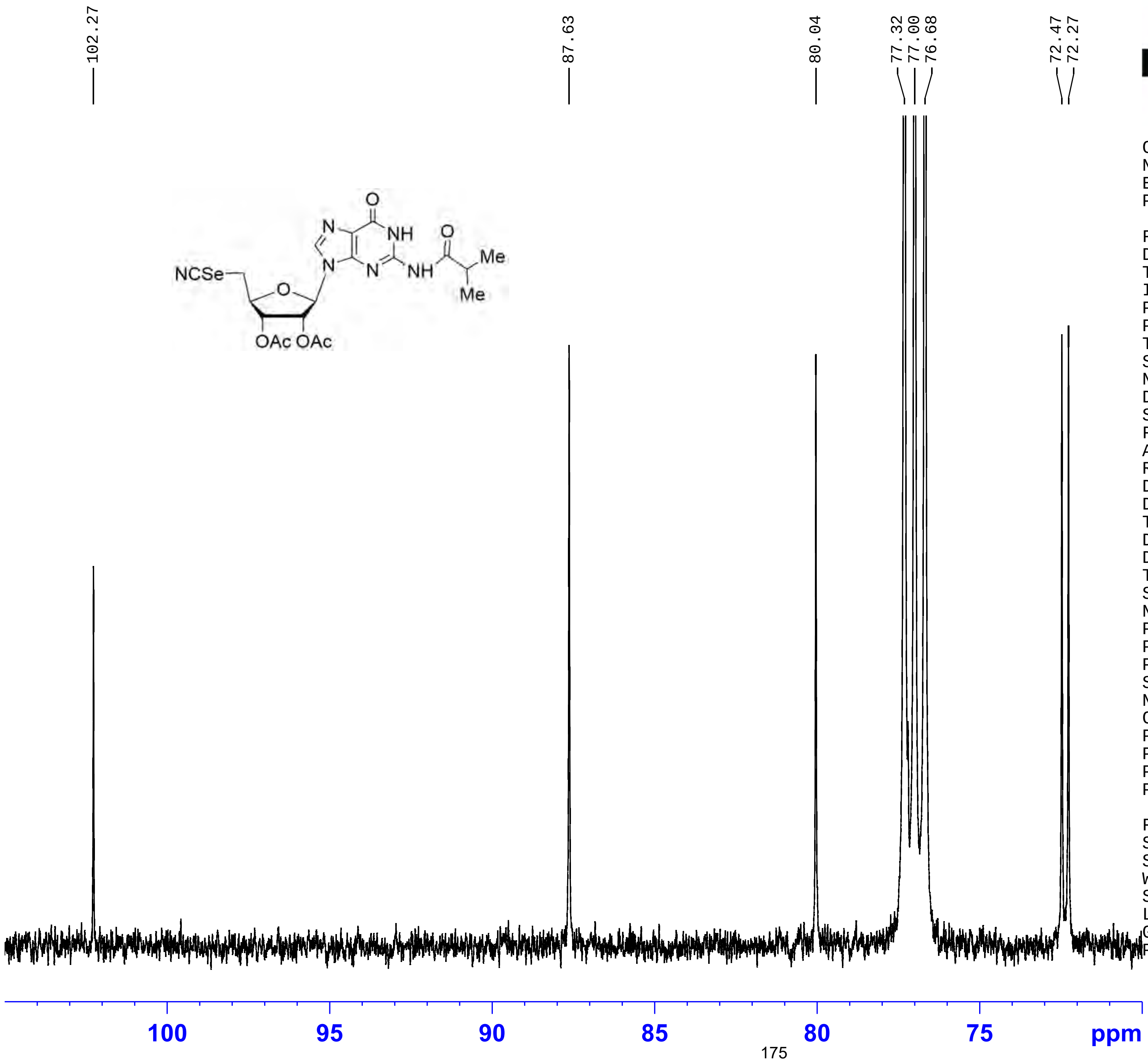

Current Data Parameters
NAME LH-II-75 OLD NMR
EXPNO 11
PROCNO 1

F2 - Acquisition Parameters
Date_ 20230630
Time 2.52 h
INSTRUM AVIII_400
PROBHD Z108618_0146 (
PULPROG zgpg30
TD 96150
SOLVENT CDCl3
NS 3000
DS 4
SWH 24038.461 Hz
FIDRES 0.500020 Hz
AQ 1.9999200 sec
RG 2050
DW 20.800 usec
DE 6.50 usec
TE 300.0 K
D1 1.00000000 sec
D11 0.03000000 sec
TD0 1
SFO1 100.6178003 MHz
NUC1 13C
P0 2.90 usec
P1 8.70 usec
PLW1 96.68000031 W
SFO2 400.1116004 MHz
NUC2 1H
CPDPRG[2 waltz64
PCPD2 90.00 usec
PLW2 17.29199982 W
PLW12 0.48032999 W
PLW13 0.24160001 W

F2 - Processing parameters
SI 131072
SF 100.6077445 MHz
WDW EM
SSB 0
LB 1.00 Hz
GB 0
PC 1.40

Expanded region of the ^{13}C NMR spectrum of compound 11

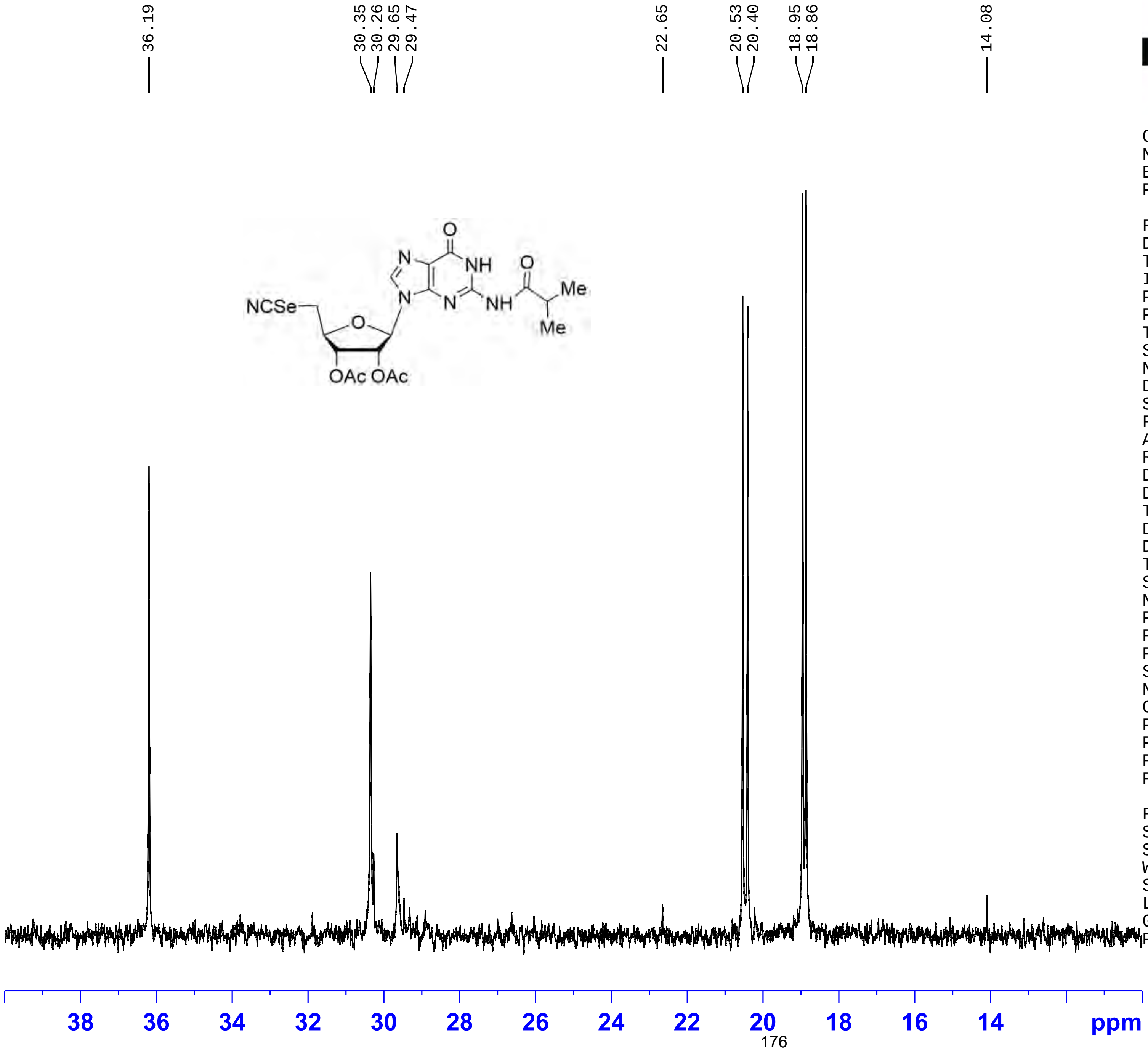

Current Data Parameters
NAME LH-II-75 OLD NMR
EXPNO 11
PROCNO 1

F2 - Acquisition Parameters
Date_ 20230630
Time 2.52 h
INSTRUM AVIII_400
PROBHD Z108618_0146 (
PULPROG zgpg30
TD 96150
SOLVENT CDCl3
NS 3000
DS 4
SWH 24038.461 Hz
FIDRES 0.500020 Hz
AQ 1.9999200 sec
RG 2050
DW 20.800 usec
DE 6.50 usec
TE 300.0 K
D1 1.00000000 sec
D11 0.03000000 sec
TD0 1
SFO1 100.6178003 MHz
NUC1 13C
P0 2.90 usec
P1 8.70 usec
PLW1 96.68000031 W
SFO2 400.1116004 MHz
NUC2 1H
CPDPRG[2 waltz64
PCPD2 90.00 usec
PLW2 17.29199982 W
PLW12 0.48032999 W
PLW13 0.24160001 W

F2 - Processing parameters
SI 131072
SF 100.6077445 MHz
WDW EM
SSB 0
LB 1.00 Hz
GB 0
PC 1.40

^{13}C DEPT-135 NMR spectrum of compound 11

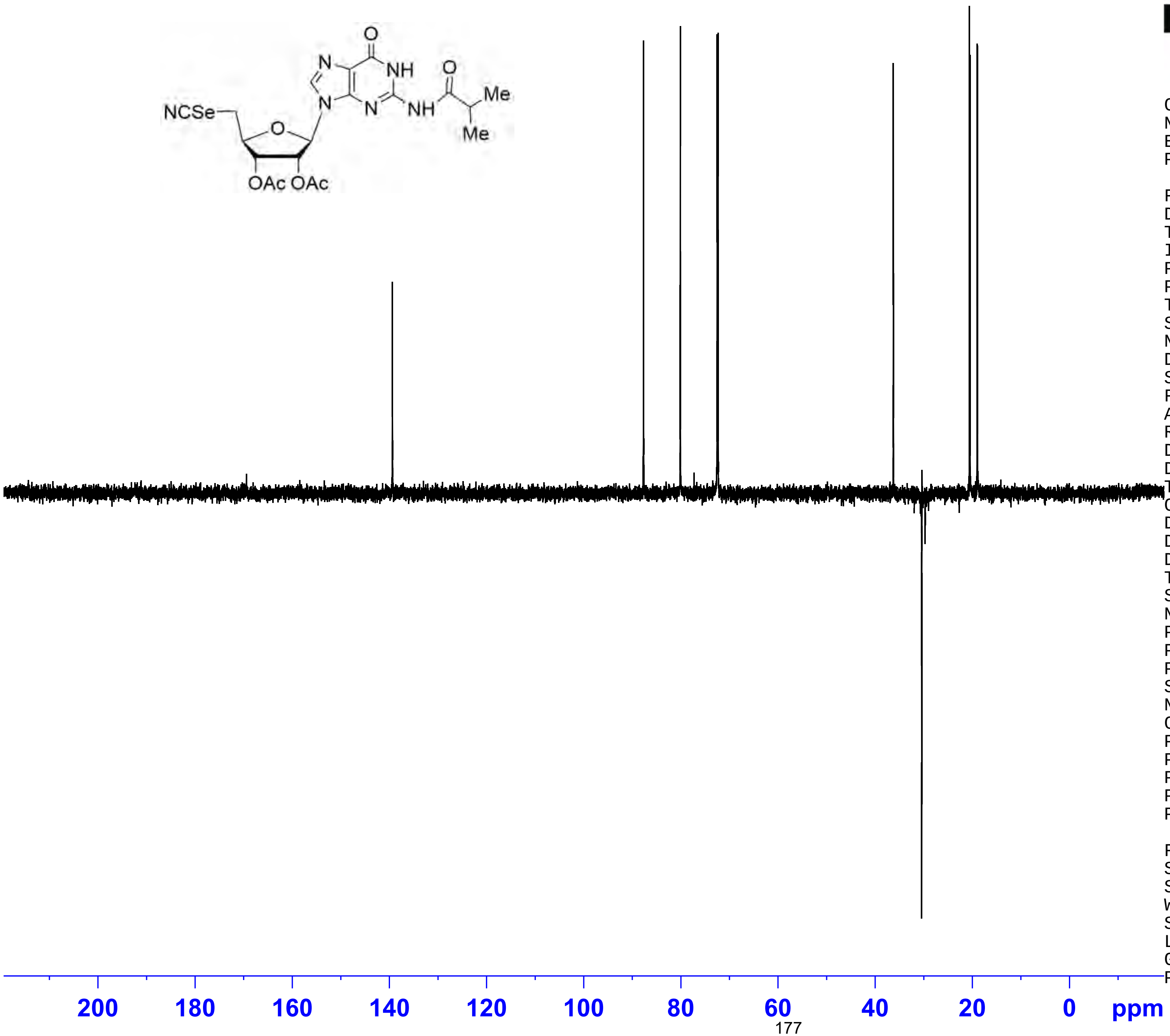


```
Current Data Parameters
NAME        LH-II-75 OLD NMR
EXPNO                     12
PROCNO                     1

F2 - Acquisition Parameters
Date_               20230630
Time                    4.21 h
INSTRUM             AVIII_400
PROBHD        Z108618_0146 (
PULPROG              dept135
TD                     65536
SOLVENT                CDCl3
NS                      1500
DS                         4
SWH                24038.461 Hz
FIDRES              0.733596 Hz
AQ                 1.3631488 sec
RG                      2050
DW                    20.800 usec
DE                      6.50 usec
TE                     300.0 K
CNST2            145.0000000
D1                2.00000000 sec
D2                0.00344828 sec
D12               0.00002000 sec
TD0                        1
SFO1             100.6178003 MHz
NUC1                     13C
P1                      8.70 usec
P2                     17.40 usec
PLW1             96.68000031 W
SFO2             400.1116004 MHz
NUC2                      1H
CPDPRG[2            waltz64
P3                     15.00 usec
P4                     30.00 usec
PCPD2                  90.00 usec
PLW2             17.29199982 W
PLW12             0.48032999 W

F2 - Processing parameters
SI                     32768
SF               100.6077400 MHz
WDW                       EM
SSB       0
LB                      1.00 Hz
GB        0
PC                      1.40
```

^{1}H-^{1}H COSY NMR spectrum of compound 11

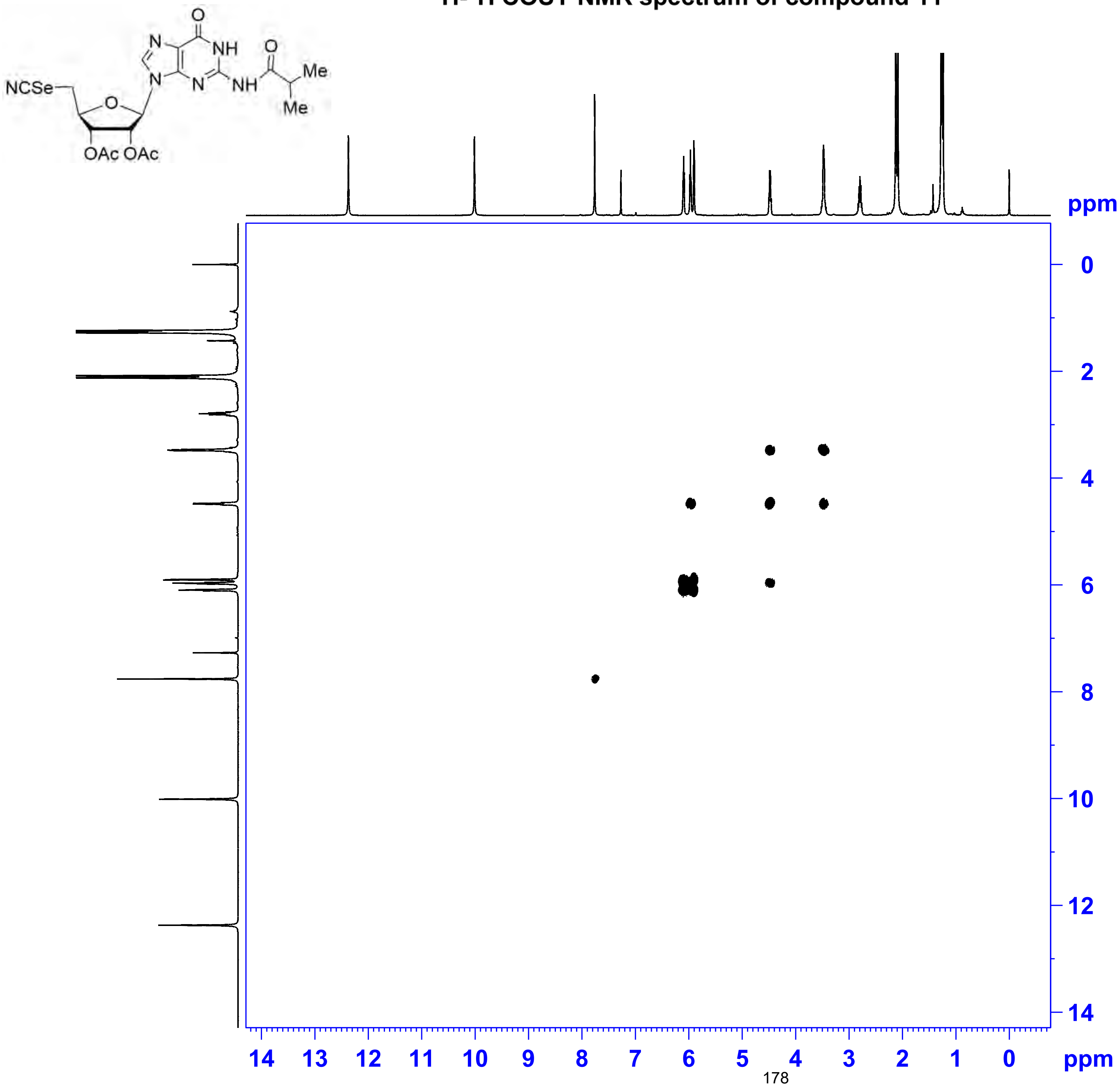


```
Current Data Parameters
NAME        LH-II-75 NEW NMR
EXPNO                     11
PROCNO                     1

F2 - Acquisition Parameters
Date_               20230630
Time                   11.52 h
INSTRUM            AVIII_400
PROBHD       Z108618_0817 (
PULPROG           cosygpmfqf
TD                      2048
SOLVENT                CDCl3
NS                         3
DS                         8
SWH                 6024.096 Hz
FIDRES              5.882906 Hz
AQ                 0.1699840 sec
RG                      1620
DW                    83.000 usec
DE                      6.50 usec
TE                     295.0 K
D0                0.00000300 sec
D1                2.02197909 sec
D13               0.00000400 sec
D16               0.00020000 sec
IN0               0.00016600 sec
TDav                       1
SFO1             399.9127083 MHz
NUC1                      1H
P1                    500.00 usec
PLW1             31.62299919 W
GPNAM[1]            SINE.100
GPZ1                   16.00 %
GPNAM[2]            SINE.100
GPZ2                   12.00 %
GPNAM[3]            SINE.100
GPZ3                   40.00 %
P16                  1000.00 usec

F1 - Acquisition parameters
TD                       256
SFO1                399.9127 MHz
FIDRES             47.063251 Hz
SW                    15.064 ppm
FnMODE                    QF

F2 - Processing parameters
SI                      1024
SF               399.9100053 MHz
WDW                     SINE
SSB         0
LB          0 Hz
GB          0
PC                      1.40

F1 - Processing parameters
SI                      1024
MC2                       QF
SF               399.9100053 MHz
WDW                     SINE
SSB         0
LB          0 Hz
GB          0
```

Expanded region of ^{1}H-^{1}H COSY NMR spectrum of compound 11

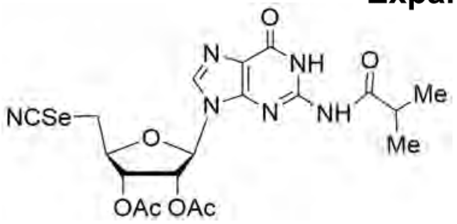

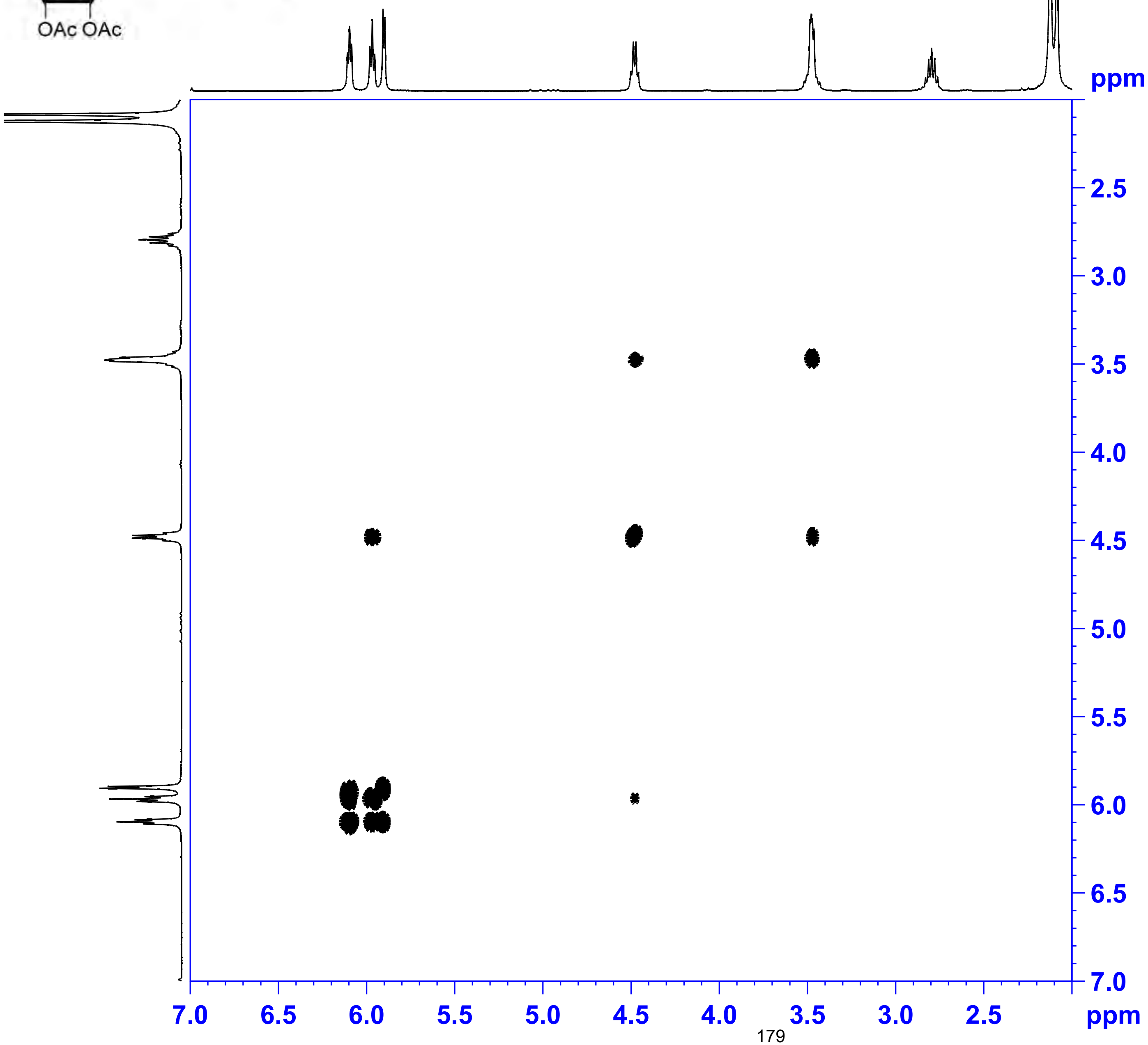


```
Current Data Parameters
NAME      LH-II-75 NEW NMR
EXPNO                   11
PROCNO                   1

F2 - Acquisition Parameters
Date_             20230630
Time               11.52 h
INSTRUM          AVIII_400
PROBHD    Z108618_0817 (
PULPROG         cosygpmfqf
TD                    2048
SOLVENT              CDCl3
NS                       3
DS                       8
SWH             6024.096 Hz
FIDRES          5.882906 Hz
AQ             0.1699840 sec
RG                    1620
DW                83.000 usec
DE                  6.50 usec
TE                 295.0 K
D0            0.00000300 sec
D1            2.02197909 sec
D13           0.00000400 sec
D16           0.00020000 sec
IN0           0.00016600 sec
TDav                     1
SFO1         399.9127083 MHz
NUC1                    1H
P1                500.00 usec
PLW1         31.62299919 W
GPNAM[1]          SINE.100
GPZ1               16.00 %
GPNAM[2]          SINE.100
GPZ2               12.00 %
GPNAM[3]          SINE.100
GPZ3               40.00 %
P16              1000.00 usec

F1 - Acquisition parameters
TD                     256
SFO1           399.9127 MHz
FIDRES         47.063251 Hz
SW                15.064 ppm
FnMODE                  QF

F2 - Processing parameters
SI                    1024
SF           399.9100053 MHz
WDW                   SINE
SSB       0
LB        0 Hz
GB        0
PC                    1.40

F1 - Processing parameters
SI                    1024
MC2                     QF
SF           399.9100053 MHz
WDW                   SINE
SSB       0
LB        0 Hz
GB        0
```

^{1}H-^{13}C HSQC NMR spectrum of compound 11

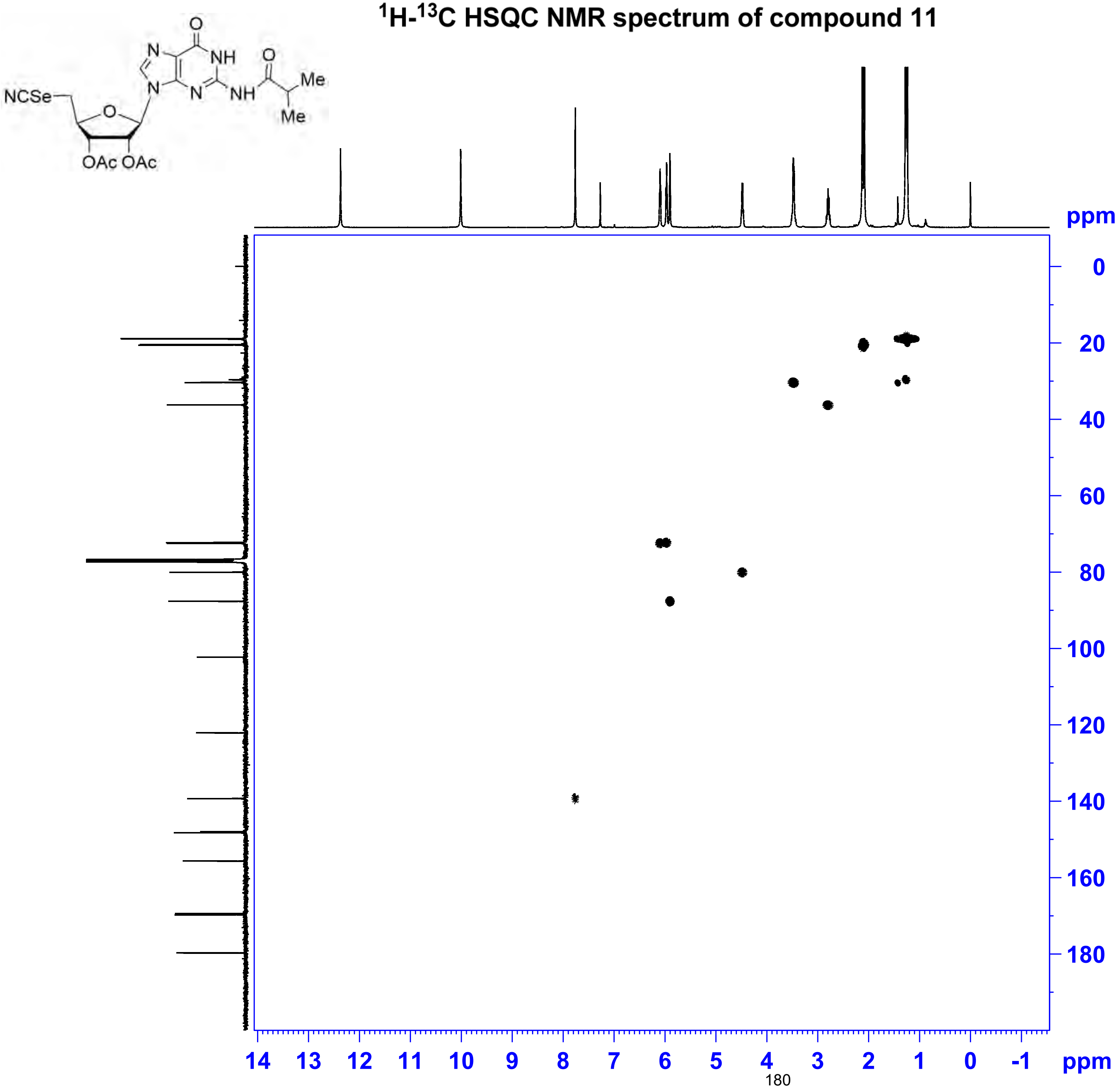


```
Current Data Parameters
NAME      LH-II-75 OLD NMR
EXPNO                   13
PROCNO                   1

F2 - Acquisition Parameters
Date_             20230630
Time                4.50 h
INSTRUM           AVIII_400
PROBHD    Z108618_0146 (
PULPROG          hsqcedetgp
TD                    1024
SOLVENT              CDCl3
NS                       4
DS                      16
SWH               6250.000 Hz
FIDRES           12.207031 Hz
AQ               0.0819200 sec
RG                    1440
DW                  80.000 usec
DE                    6.50 usec
TE                   300.0 K
CNST2          145.0000000
D0              0.00000300 sec
D1              1.45965397 sec
D4              0.00172414 sec
D11             0.03000000 sec
D16             0.00020000 sec
D21             0.00345000 sec
IN0             0.00003000 sec
TDav                     1
ZGOPTNS
SFO1           400.1125093 MHz
NUC1                    1H
P1                   15.00 usec
P2                   30.00 usec
PLW1           17.29199982 W
SFO2           100.6152434 MHz
NUC2                   13C
CPDPRG[2              garp
P3                    8.70 usec
P4                   17.40 usec
PCPD2                56.50 usec
PLW2           96.68000031 W
PLW12           3.16230011 W
GPNAM[1]        SMSQ10.100
GPZ1                 80.00 %
GPNAM[2]        SMSQ10.100
GPZ2                 20.10 %
P16                1000.00 usec

F1 - Acquisition parameters
TD                     256
SFO1              100.6152 MHz
FIDRES          130.208328 Hz
SW                 165.648 ppm
FnMODE        Echo-Antiecho

F2 - Processing parameters
SI                    1024
SF             400.1100056 MHz
WDW                  QSINE
SSB                      2
LB        0 Hz
GB        0
PC                    1.40

F1 - Processing parameters
SI                    1024
MC2           echo-antiecho
SF             100.6077400 MHz
WDW                  QSINE
SSB                      2
LB        0 Hz
GB        0
```

^{31}P NMR spectrum of compound 22

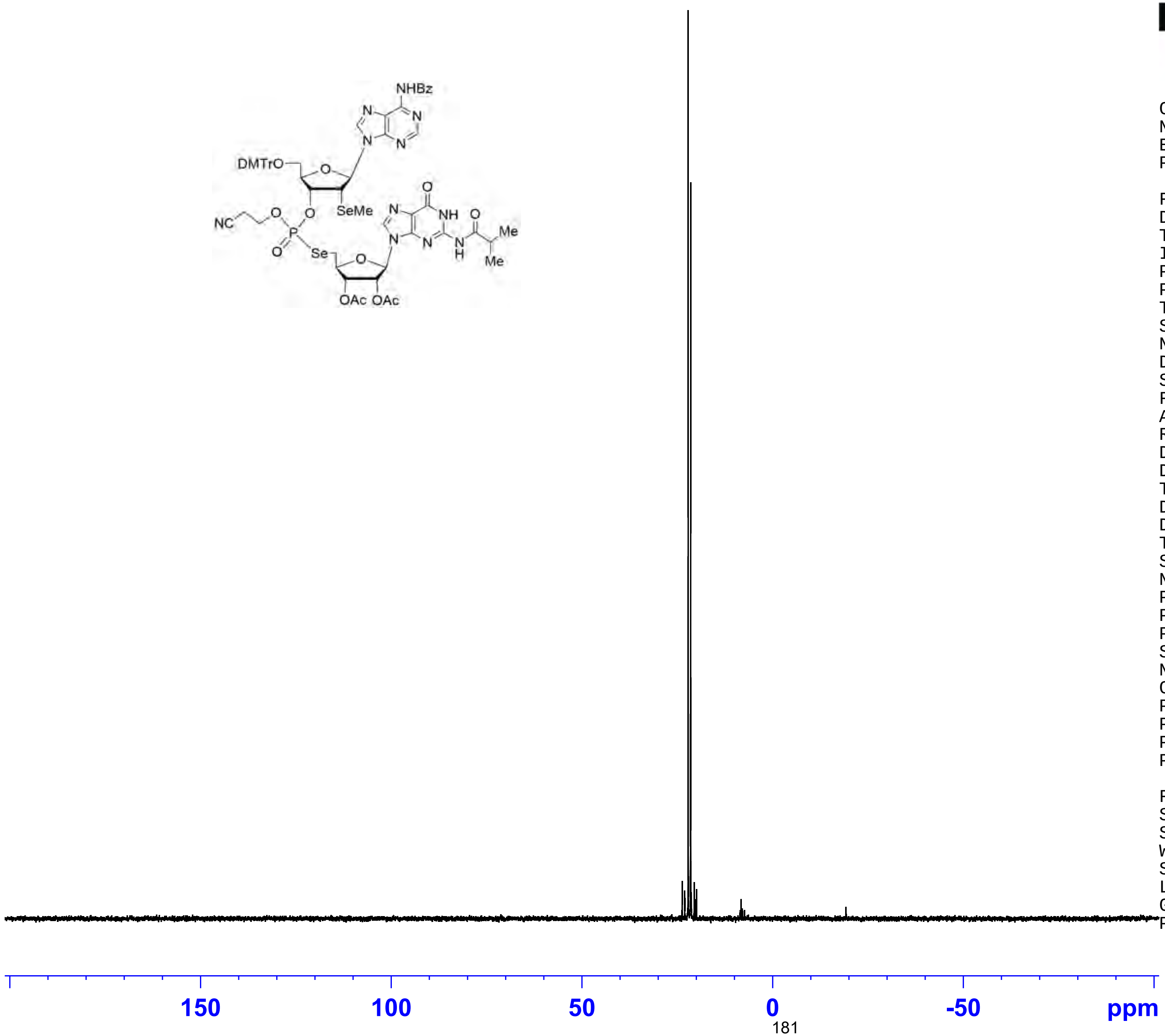

Current Data Parameters
NAME LH-II-77
EXPNO 10
PROCNO 1

F2 - Acquisition Parameters
Date_ 20230630
Time 15.40 h
INSTRUM AVIII_400
PROBHD Z108618_0146 (
PULPROG zgpg30
TD 65536
SOLVENT D2O
NS 539
DS 4
SWH 49019.609 Hz
FIDRES 1.495960 Hz
AQ 0.6684672 sec
RG 2050
DW 10.200 usec
DE 6.50 usec
TE 300.0 K
D1 2.00000000 sec
D11 0.03000000 sec
TD0 1
SFO1 161.9755954 MHz
NUC1 31P
P0 2.80 usec
P1 8.40 usec
PLW1 41.93299866 W
SFO2 400.1116004 MHz
NUC2 1H
CPDPRG[2 waltz16
PCPD2 90.00 usec
PLW2 17.29199982 W
PLW12 0.48032999 W
PLW13 0.24160001 W

F2 - Processing parameters
SI 32768
SF 161.9674970 MHz
WDW EM
SSB 0
LB 2.00 Hz
GB 0
PC 1.40

Expanded region of ^{31}P NMR spectrum of compound 22

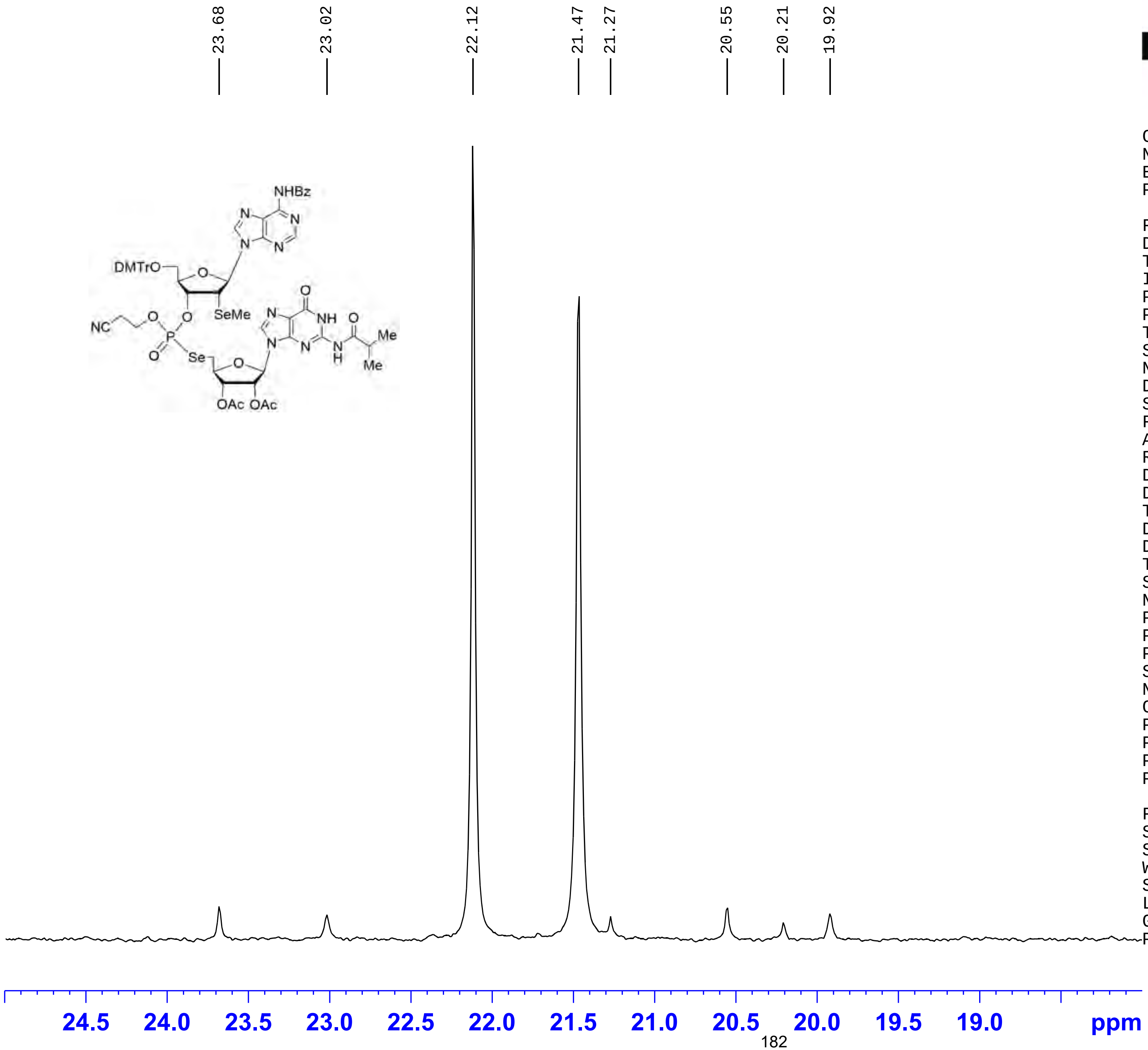

BRUKER

```
Current Data Parameters
NAME              LH-II-77
EXPNO                   10
PROCNO                   1

F2 - Acquisition Parameters
Date_             20230630
Time                 15.40 h
INSTRUM          AVIII_400
PROBHD   Z108618_0146 (
PULPROG            zgpg30
TD                   65536
SOLVENT                D2O
NS                     539
DS                       4
SWH              49019.609 Hz
FIDRES            1.495960 Hz
AQ               0.6684672 sec
RG                    2050
DW                  10.200 usec
DE                    6.50 usec
TE                   300.0 K
D1              2.00000000 sec
D11             0.03000000 sec
TD0                      1
SFO1           161.9755954 MHz
NUC1                   31P
P0                    2.80 usec
P1                    8.40 usec
PLW1           41.93299866 W
SFO2           400.1116004 MHz
NUC2                    1H
CPDPRG[2           waltz16
PCPD2                90.00 usec
PLW2           17.29199982 W
PLW12           0.48032999 W
PLW13           0.24160001 W

F2 - Processing parameters
SI                   32768
SF             161.9674970 MHz
WDW                     EM
SSB      0
LB                    2.00 Hz
GB       0
PC                    1.40
```

^{77}Se NMR spectrum of compound 22

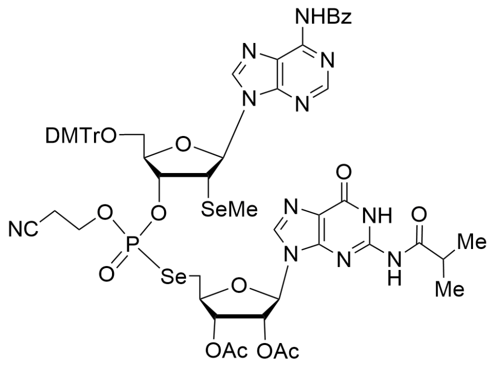

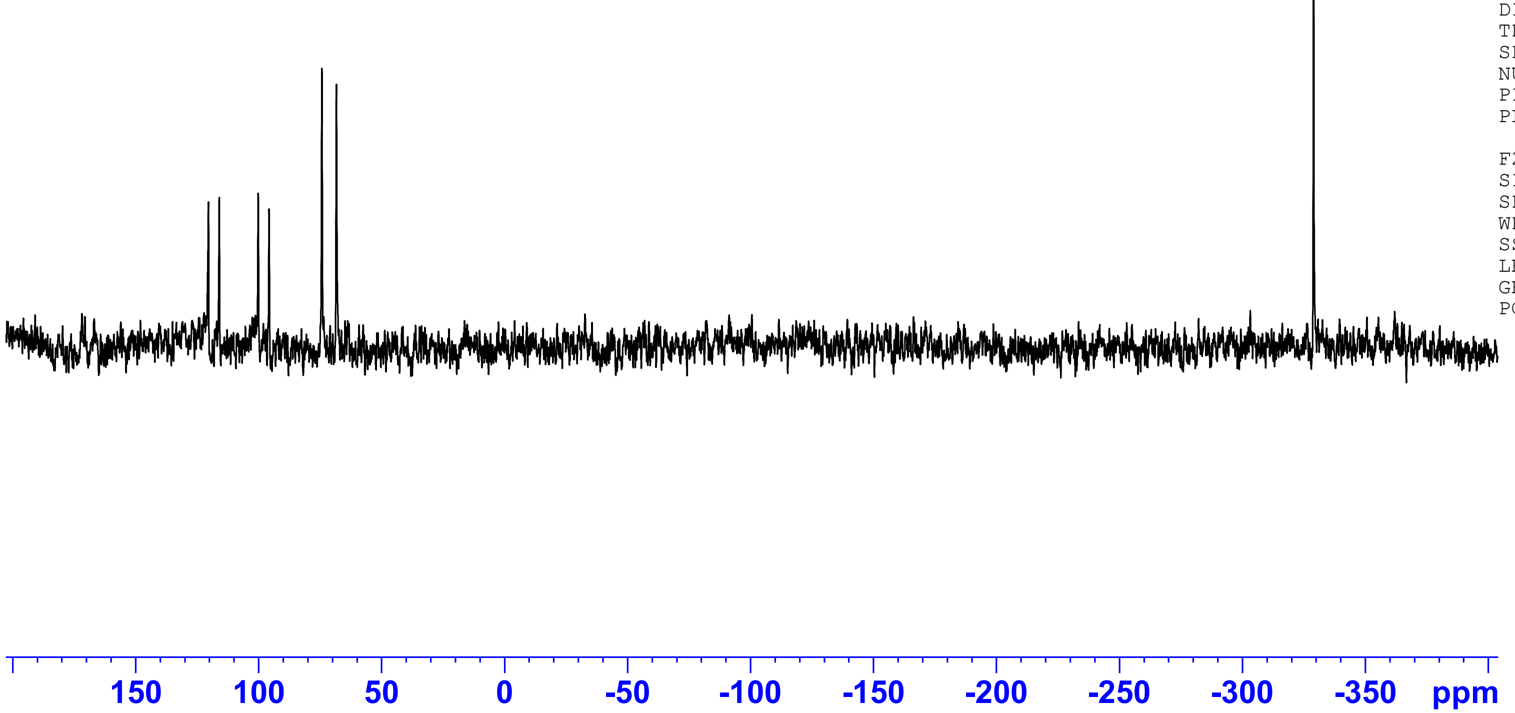

Current Data Parameters

NAME	LH-II-77-Pure
EXPNO	15
PROCNO	1

F2 - Acquisition Parameters

Date_	20231105
Time	21.50
INSTRUM	spect
PROBHD	Z114607_0188 (PA BBO
PULPROG	zg
TD	27770
SOLVENT	D2O
NS	20480
DS	4
SWH	69444.445
FIDRES	5.001400
AQ	0.1999440
RG	186.92
DW	7.200
DE	6.50
TE	298.0
D1	1.00000000
TD0	1
SFO1	114.4424256
NUC1	77Se
P1	4.00
PLW1	60.00000000

F2 - Processing parameters

SI	32768
SF	114.4539373
WDW	EM
SSB	0
LB	20.00
GB	0
PC	1.40

^{31}P NMR spectrum of compound 6 in $CDCl_3$

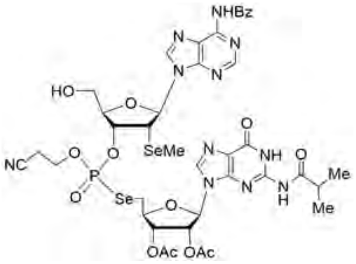

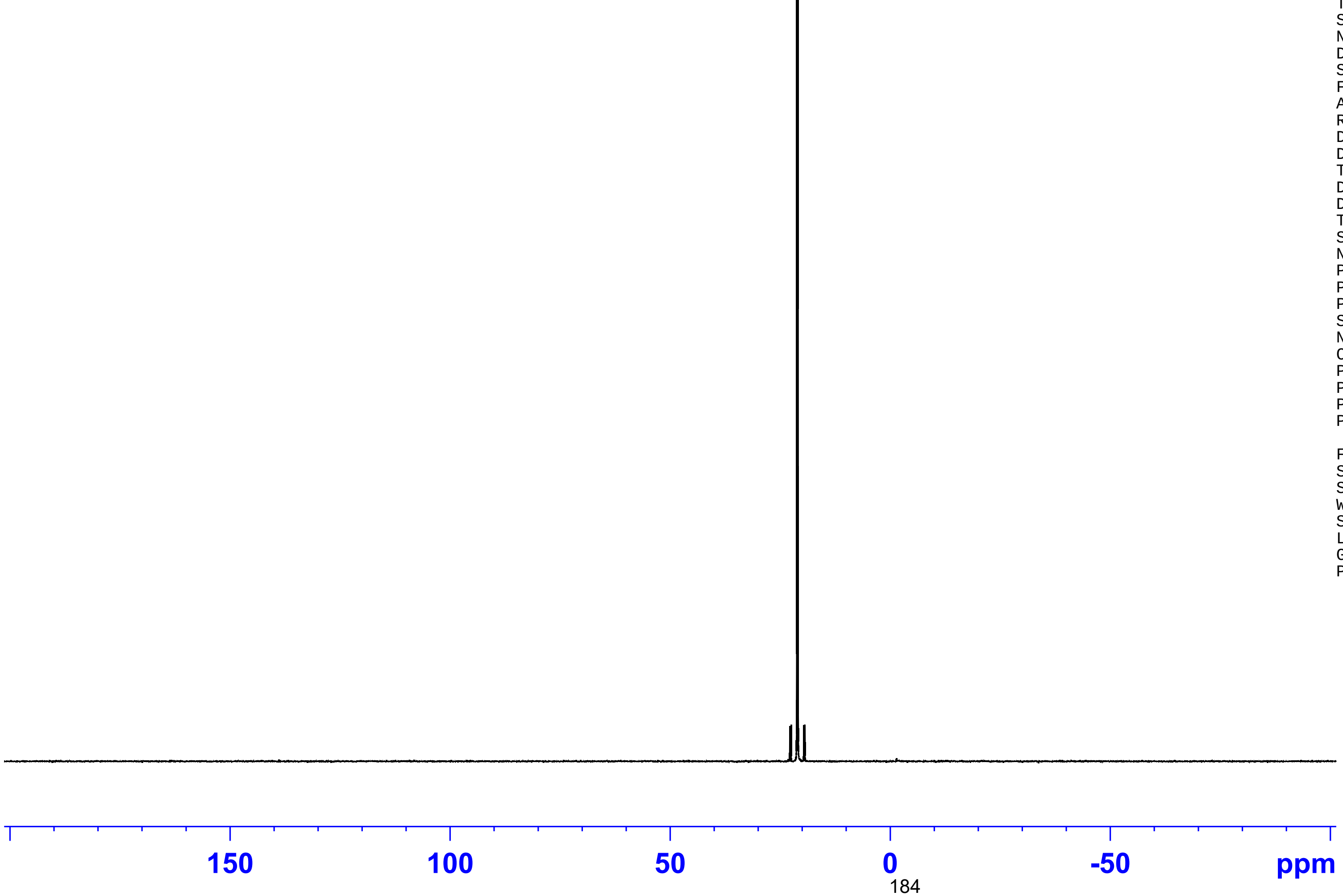

Current Data Parameters
NAME LH-II-64 OLD NMR 31P CDCl3
EXPNO 10
PROCNO 1

F2 - Acquisition Parameters
Date_ 20230720
Time 11.11 h
INSTRUM AVIII_400
PROBHD Z108618_0146 (
PULPROG zgpg30
TD 65536
SOLVENT CDCl3
NS 1024
DS 4
SWH 49019.609 Hz
FIDRES 1.495960 Hz
AQ 0.6684672 sec
RG 2050
DW 10.200 usec
DE 6.50 usec
TE 300.0 K
D1 2.00000000 sec
D11 0.03000000 sec
TD0 1
SFO1 161.9755954 MHz
NUC1 31P
P0 2.80 usec
P1 8.40 usec
PLW1 41.93299866 W
SFO2 400.1116004 MHz
NUC2 1H
CPDPRG[2 waltz16
PCPD2 90.00 usec
PLW2 17.29199982 W
PLW12 0.48032999 W
PLW13 0.24160001 W

F2 - Processing parameters
SI 32768
SF 161.9674970 MHz
WDW EM
SSB 0
LB 2.00 Hz
GB 0
PC 1.40

Expanded region of ^{31}P NMR spectrum of compound 6 in $CDCl_3$

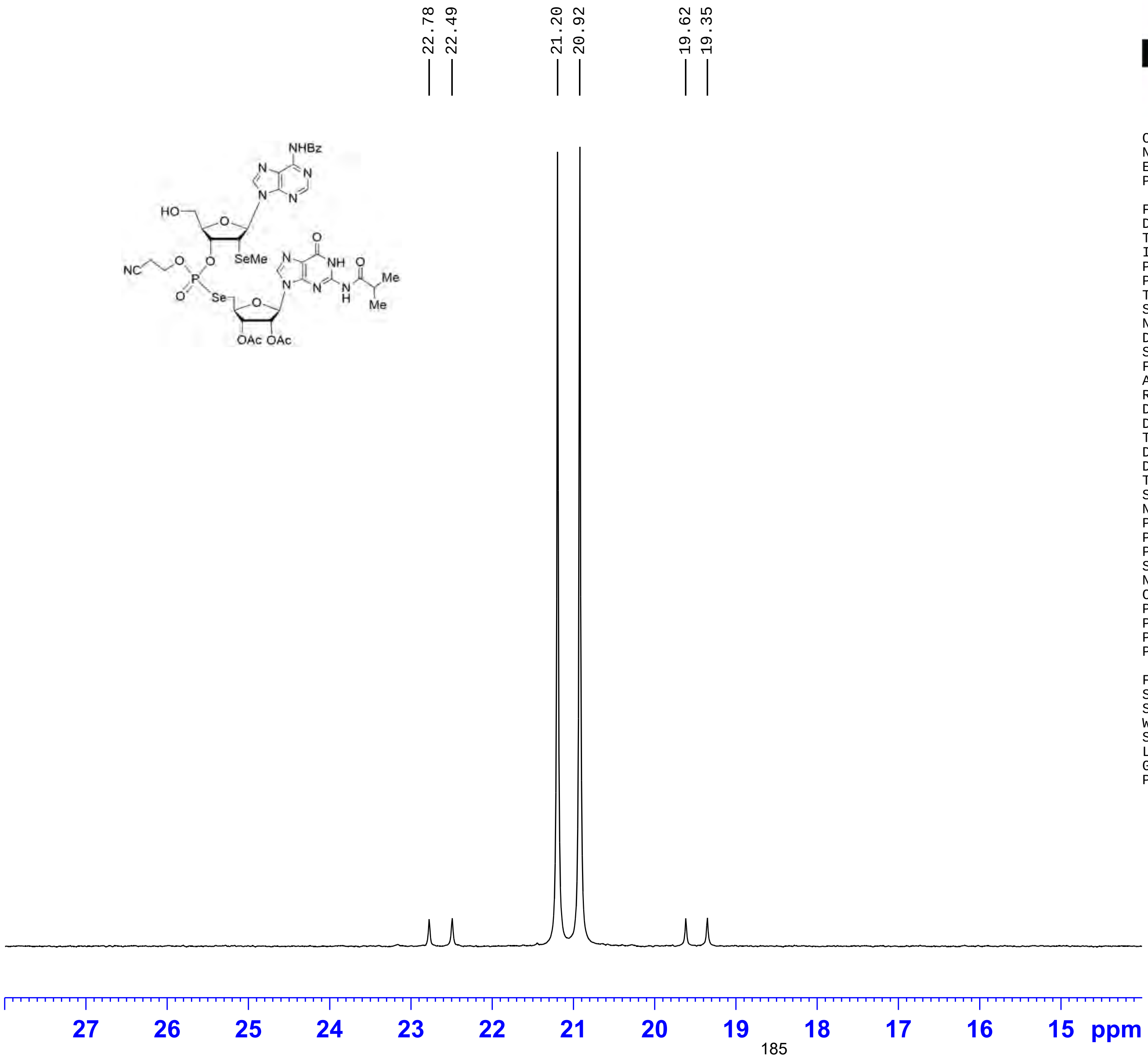


```
Current Data Parameters
NAME      LH-II-64 OLD NMR 31P CDCl3
EXPNO                     10
PROCNO                     1

F2 - Acquisition Parameters
Date_               20230720
Time                   11.11 h
INSTRUM             AVIII_400
PROBHD     Z108618_0146 (
PULPROG               zgpg30
TD                     65536
SOLVENT                CDCl3
NS                      1024
DS                         4
SWH               49019.609 Hz
FIDRES             1.495960 Hz
AQ                0.6684672 sec
RG                      2050
DW                    10.200 usec
DE                      6.50 usec
TE                     300.0 K
D1                2.00000000 sec
D11               0.03000000 sec
TD0                        1
SFO1             161.9755954 MHz
NUC1                     31P
P0                      2.80 usec
P1                      8.40 usec
PLW1             41.93299866 W
SFO2             400.1116004 MHz
NUC2                      1H
CPDPRG[2             waltz16
PCPD2                  90.00 usec
PLW2             17.29199982 W
PLW12             0.48032999 W
PLW13             0.24160001 W

F2 - Processing parameters
SI                     32768
SF               161.9674970 MHz
WDW                       EM
SSB        0
LB                      2.00 Hz
GB         0
PC                      1.40
```

^{31}P NMR spectrum of compound 6 in MeCN (D_2O External Lock)

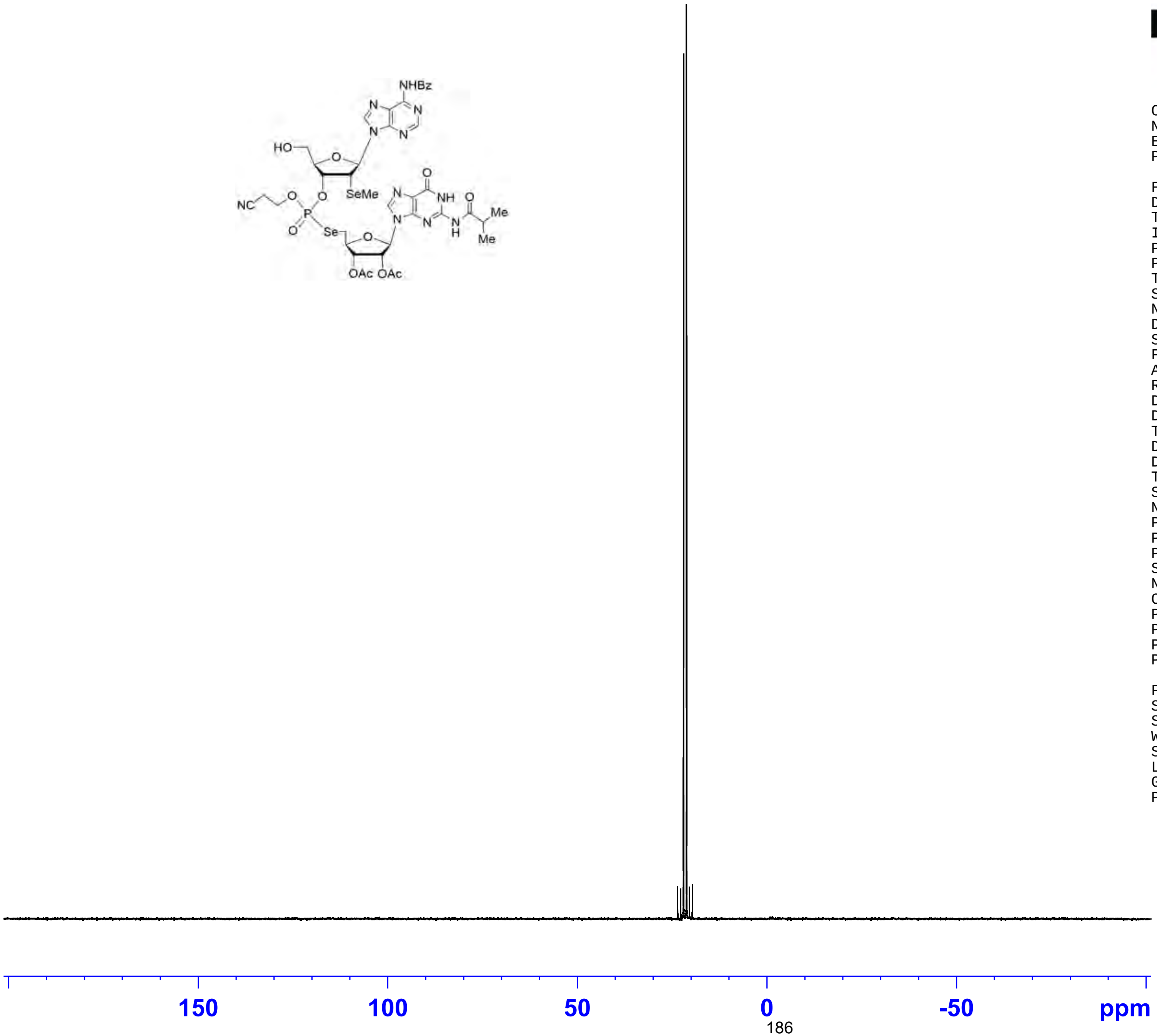

Current Data Parameters
NAME LH-II-64 OLD NMR 31P D2O
EXPNO 10
PROCNO 1

F2 - Acquisition Parameters
Date_ 20230720
Time 12.36 h
INSTRUM AVIII_400
PROBHD Z108618_0146 (
PULPROG zgpg30
TD 65536
SOLVENT D2O
NS 1024
DS 4
SWH 49019.609 Hz
FIDRES 1.495960 Hz
AQ 0.6684672 sec
RG 2050
DW 10.200 usec
DE 6.50 usec
TE 300.0 K
D1 2.00000000 sec
D11 0.03000000 sec
TD0 1
SFO1 161.9755954 MHz
NUC1 31P
P0 2.80 usec
P1 8.40 usec
PLW1 41.93299866 W
SFO2 400.1116004 MHz
NUC2 1H
CPDPRG[2 waltz16
PCPD2 90.00 usec
PLW2 17.29199982 W
PLW12 0.48032999 W
PLW13 0.24160001 W

F2 - Processing parameters
SI 32768
SF 161.9674970 MHz
WDW EM
SSB 0
LB 2.00 Hz
GB 0
PC 1.40

Expansion of the ^{31}P NMR spectrum of compound 6 in MeCN (D_2O External Lock)

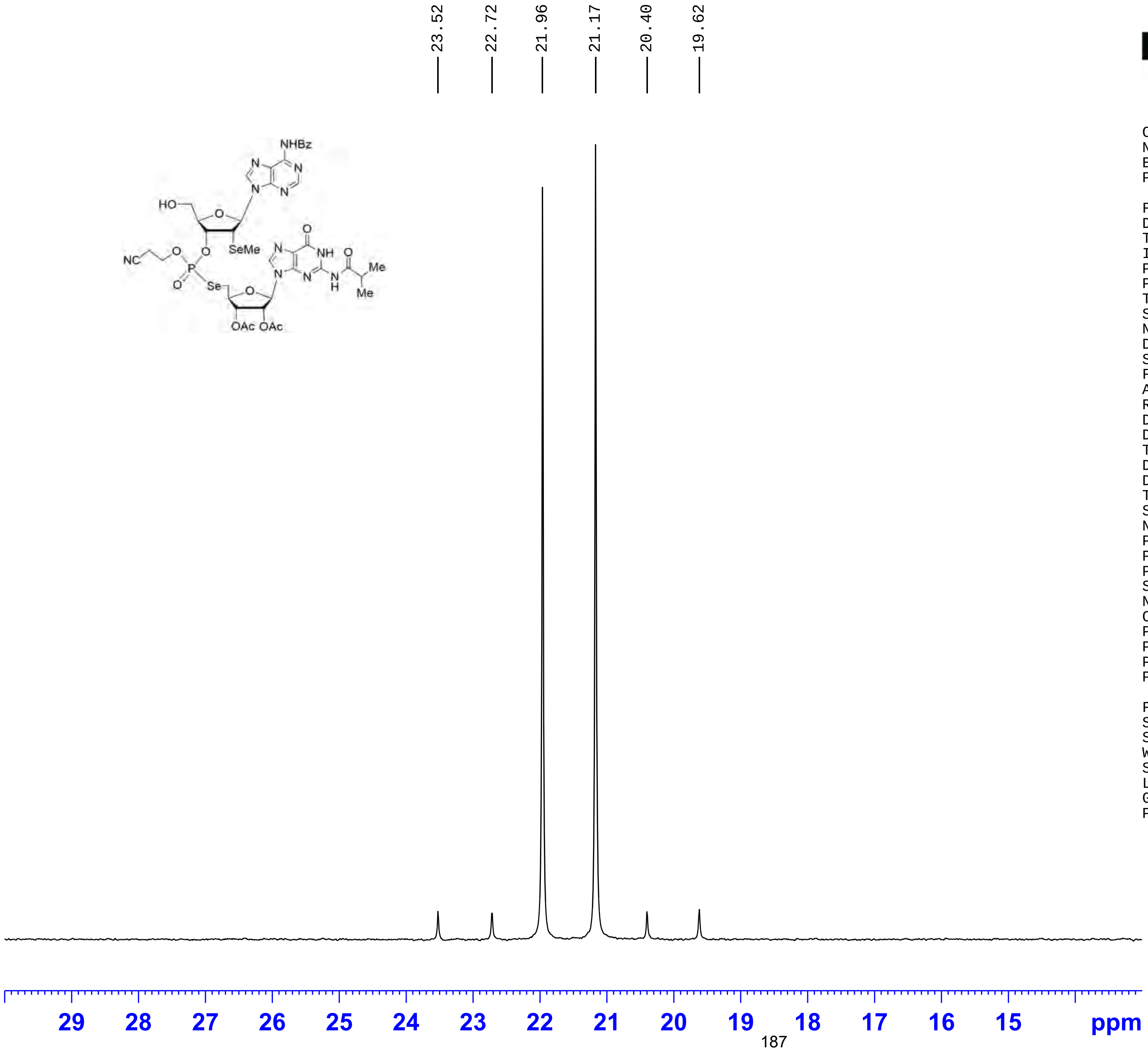

BRUKER

Current Data Parameters
NAME LH-II-64 OLD NMR 31P D2O
EXPNO 10
PROCNO 1

F2 - Acquisition Parameters
Date_ 20230720
Time 12.36 h
INSTRUM AVIII_400
PROBHD Z108618_0146 (
PULPROG zgpg30
TD 65536
SOLVENT D2O
NS 1024
DS 4
SWH 49019.609 Hz
FIDRES 1.495960 Hz
AQ 0.6684672 sec
RG 2050
DW 10.200 usec
DE 6.50 usec
TE 300.0 K
D1 2.00000000 sec
D11 0.03000000 sec
TD0 1
SFO1 161.9755954 MHz
NUC1 31P
P0 2.80 usec
P1 8.40 usec
PLW1 41.93299866 W
SFO2 400.1116004 MHz
NUC2 1H
CPDPRG[2 waltz16
PCPD2 90.00 usec
PLW2 17.29199982 W
PLW12 0.48032999 W
PLW13 0.24160001 W

F2 - Processing parameters
SI 32768
SF 161.9674970 MHz
WDW EM
SSB 0
LB 2.00 Hz
GB 0
PC 1.40

^{1}H NMR spectrum of compound 6

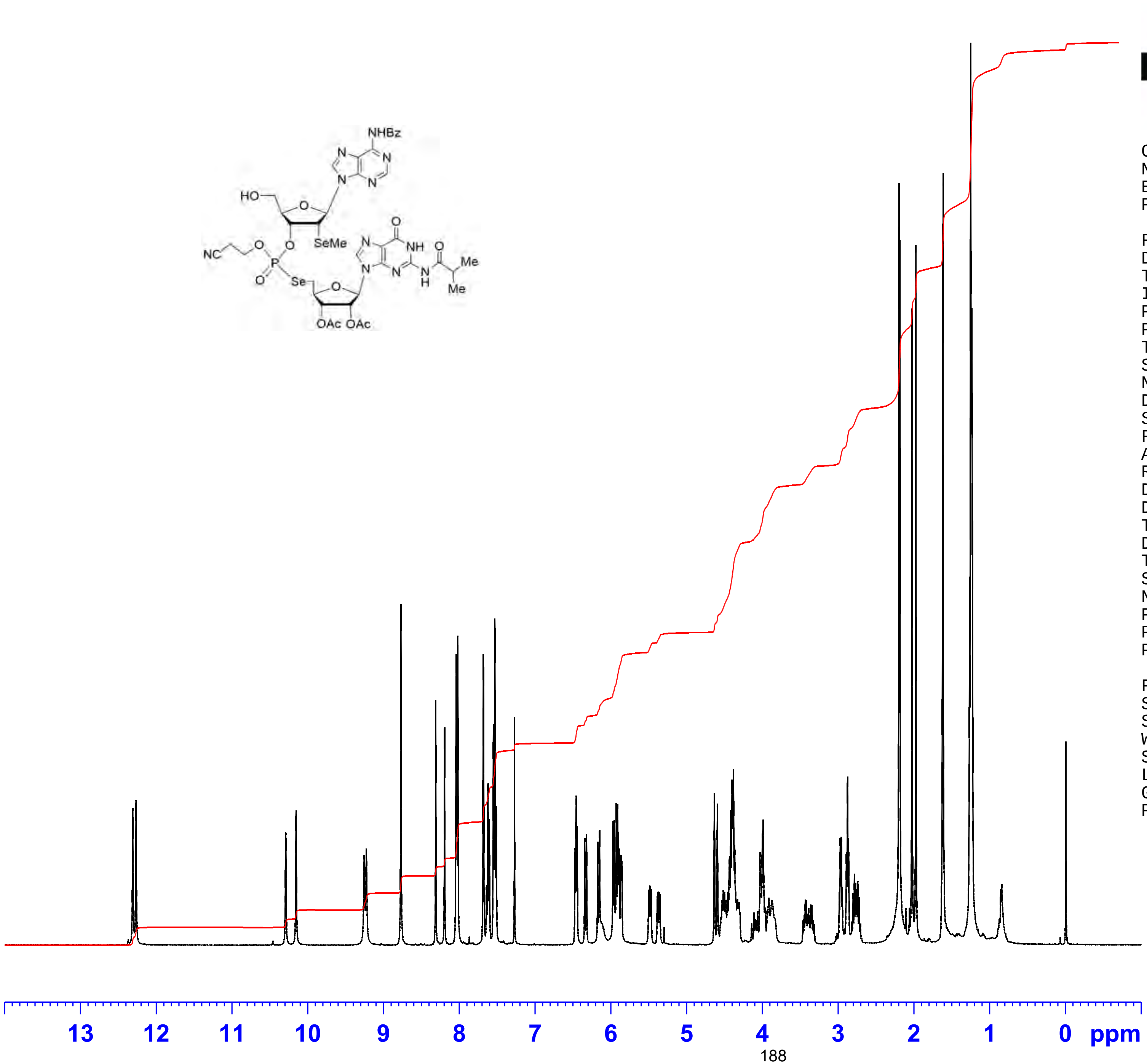

Expanded region of the ^{1}H NMR spectrum of compound 6

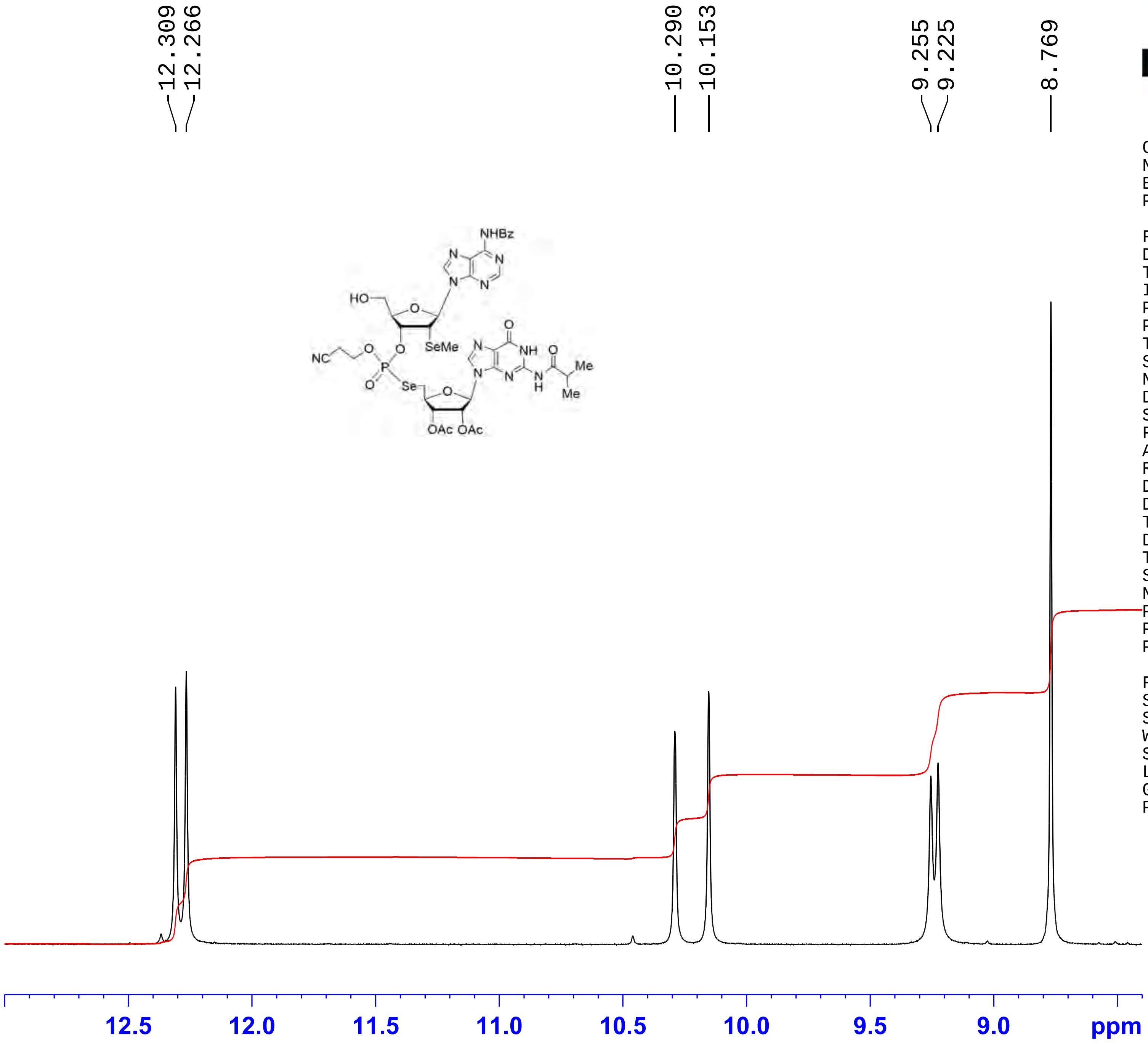

BRUKER

```
Current Data Parameters
NAME       LH-II-64 OLD NMR
EXPNO                    10
PROCNO                    1

F2 - Acquisition Parameters
Date_              20230719
Time                  21.46 h
INSTRUM            AVIII_400
PROBHD     Z108618_0146 (
PULPROG                zg30
TD                    65536
SOLVENT               CDCl3
NS                       32
DS                        2
SWH                8223.685 Hz
FIDRES             0.250967 Hz
AQ                3.9845889 sec
RG                     80.6
DW                   60.800 usec
DE                    17.42 usec
TE                    300.0 K
D1               1.00000000 sec
TD0                       1
SFO1            400.1124708 MHz
NUC1                     1H
P0                     5.00 usec
P1                    15.00 usec
PLW1            17.29199982 W

F2 - Processing parameters
SI                    32768
SF              400.1100053 MHz
WDW                      EM
SSB        0
LB                     0.30 Hz
GB         0
PC                     1.00
```

Expanded region of the ^{1}H NMR spectrum of compound 6

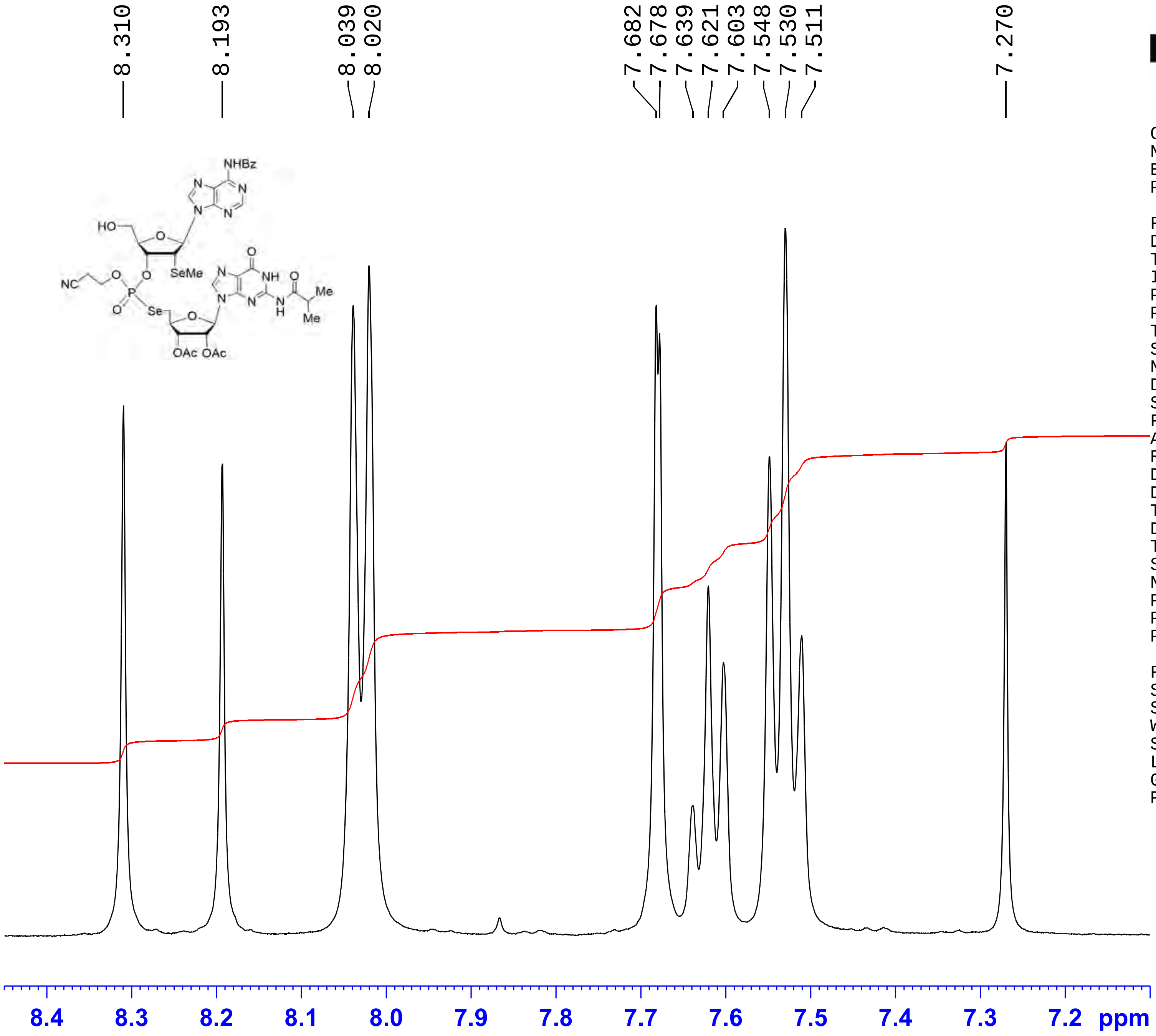

BRUKER

```
Current Data Parameters
NAME      LH-II-64 OLD NMR
EXPNO                   10
PROCNO                   1

F2 - Acquisition Parameters
Date_             20230719
Time                 21.46 h
INSTRUM          AVIII_400
PROBHD    Z108618_0146 (
PULPROG               zg30
TD                   65536
SOLVENT              CDCl3
NS                      32
DS                       2
SWH               8223.685 Hz
FIDRES            0.250967 Hz
AQ               3.9845889 sec
RG                    80.6
DW                  60.800 usec
DE                   17.42 usec
TE                   300.0 K
D1              1.00000000 sec
TD0                      1
SFO1           400.1124708 MHz
NUC1                    1H
P0                    5.00 usec
P1                   15.00 usec
PLW1           17.29199982 W

F2 - Processing parameters
SI                   32768
SF             400.1100053 MHz
WDW                     EM
SSB       0
LB                    0.30 Hz
GB        0
PC                    1.00
```

Expanded region of the ^{1}H NMR spectrum of compound 6

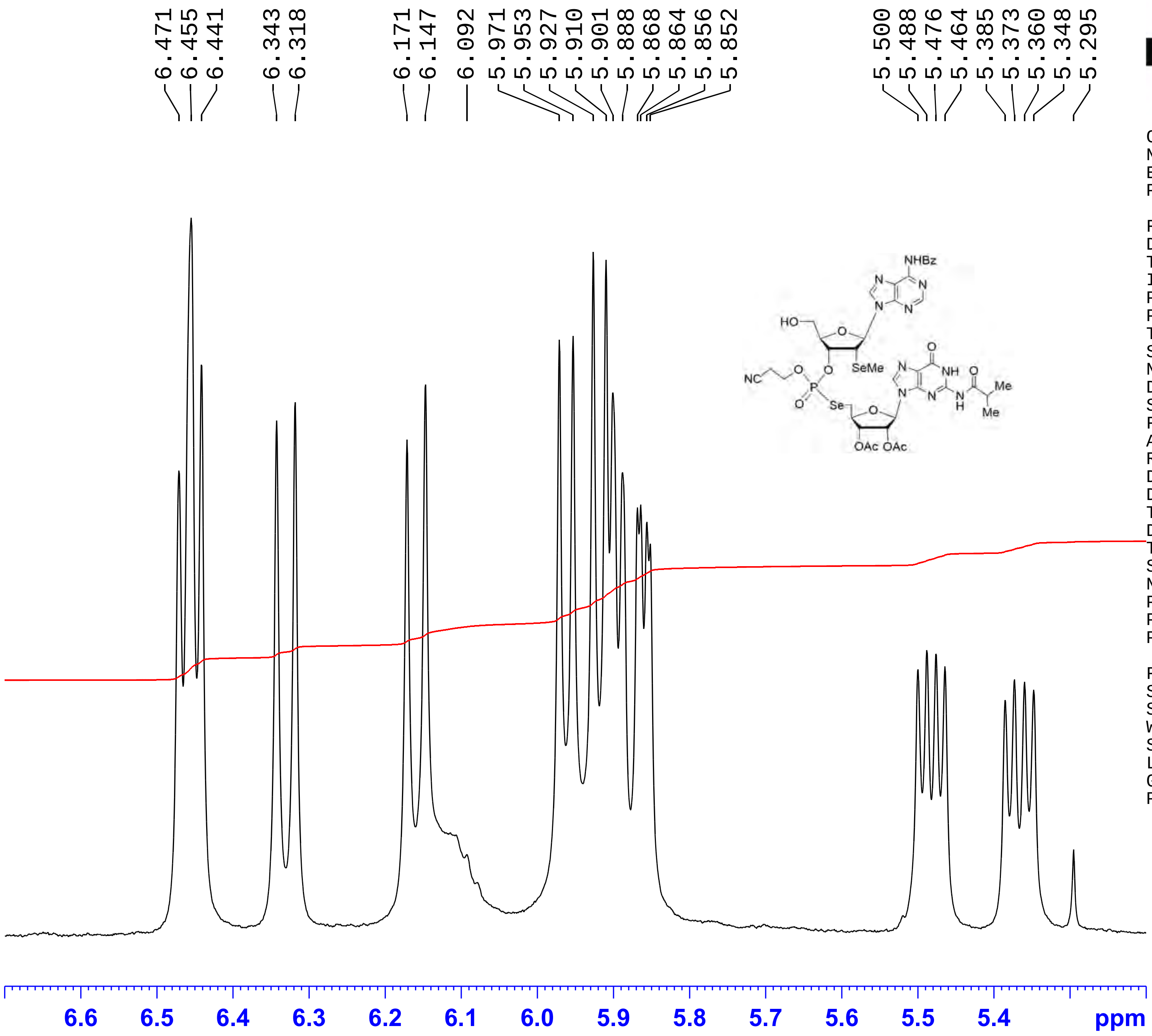


```
Current Data Parameters
NAME      LH-II-64 OLD NMR
EXPNO                   10
PROCNO                   1

F2 - Acquisition Parameters
Date_             20230719
Time                 21.46 h
INSTRUM           AVIII_400
PROBHD     Z108618_0146 (
PULPROG               zg30
TD                   65536
SOLVENT              CDCl3
NS                      32
DS                       2
SWH               8223.685 Hz
FIDRES            0.250967 Hz
AQ               3.9845889 sec
RG                    80.6
DW                  60.800 usec
DE                   17.42 usec
TE                   300.0 K
D1              1.00000000 sec
TD0                      1
SFO1           400.1124708 MHz
NUC1                    1H
P0                    5.00 usec
P1                   15.00 usec
PLW1           17.29199982 W

F2 - Processing parameters
SI                   32768
SF             400.1100053 MHz
WDW                     EM
SSB       0
LB                    0.30 Hz
GB        0
PC                    1.00
```

Expanded region of the ^{1}H NMR spectrum of compound 6

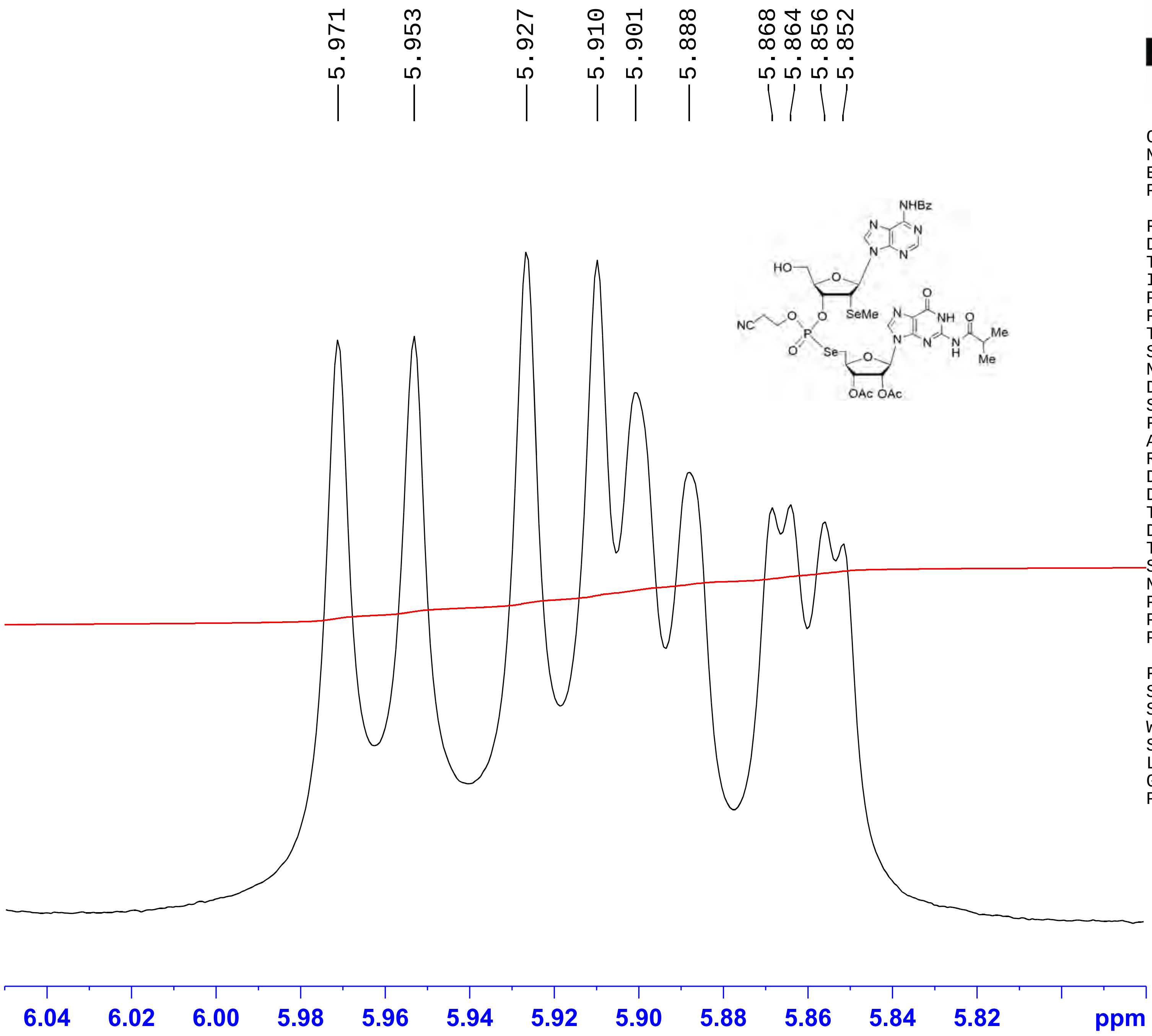

BRUKER

Current Data Parameters
NAME LH-II-64 OLD NMR
EXPNO 10
PROCNO 1

F2 - Acquisition Parameters
Date_ 20230719
Time 21.46 h
INSTRUM AVIII_400
PROBHD Z108618_0146 (
PULPROG zg30
TD 65536
SOLVENT CDCl3
NS 32
DS 2
SWH 8223.685 Hz
FIDRES 0.250967 Hz
AQ 3.9845889 sec
RG 80.6
DW 60.800 usec
DE 17.42 usec
TE 300.0 K
D1 1.00000000 sec
TD0 1
SFO1 400.1124708 MHz
NUC1 1H
P0 5.00 usec
P1 15.00 usec
PLW1 17.29199982 W

F2 - Processing parameters
SI 32768
SF 400.1100053 MHz
WDW EM
SSB 0
LB 0.30 Hz
GB 0
PC 1.00

Expanded region of the ^{1}H NMR spectrum of compound 6

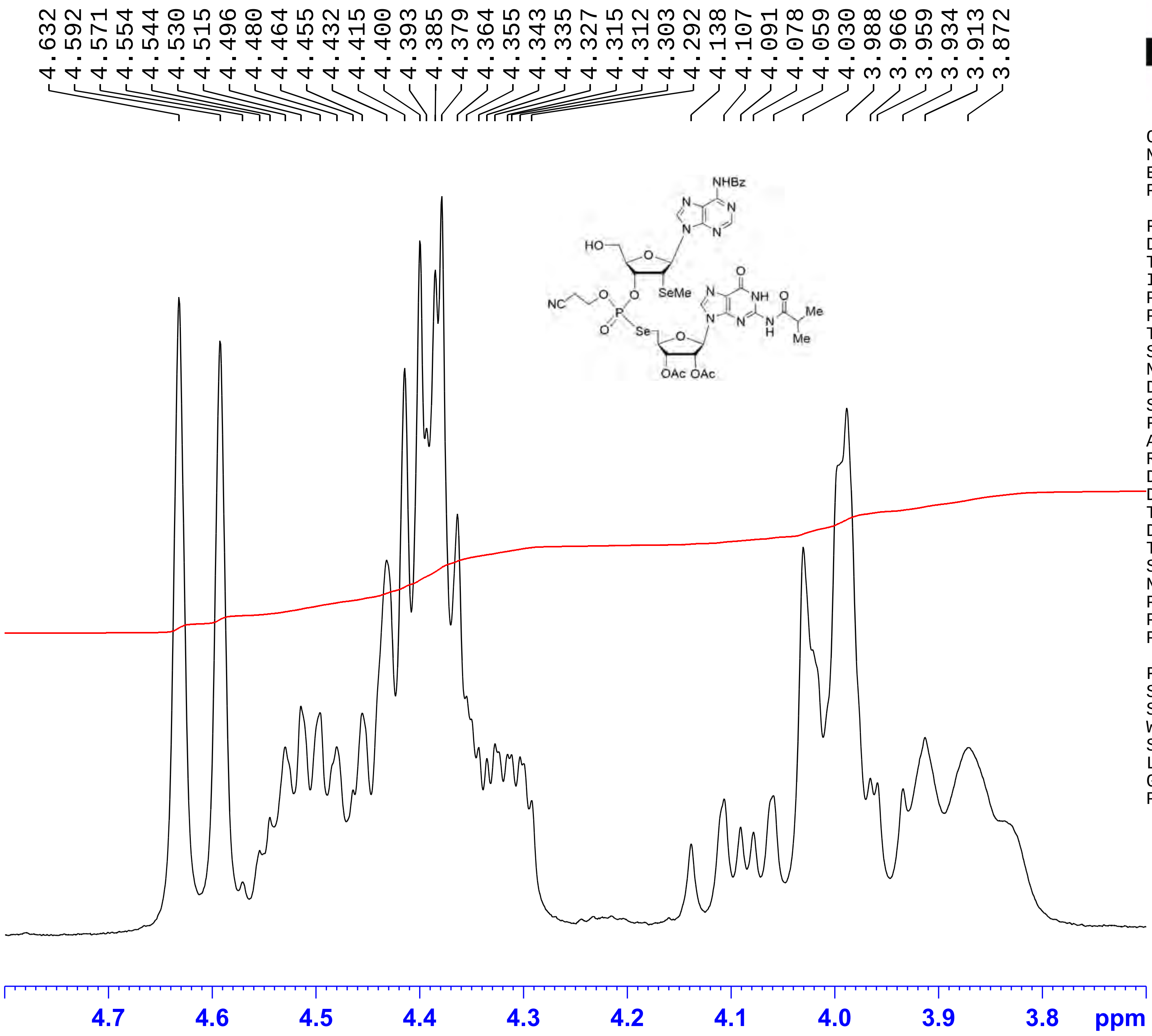


```
Current Data Parameters
NAME      LH-II-64 OLD NMR
EXPNO                   10
PROCNO                   1

F2 - Acquisition Parameters
Date_             20230719
Time                 21.46 h
INSTRUM           AVIII_400
PROBHD    Z108618_0146 (
PULPROG               zg30
TD                   65536
SOLVENT              CDCl3
NS                      32
DS                       2
SWH               8223.685 Hz
FIDRES            0.250967 Hz
AQ               3.9845889 sec
RG                    80.6
DW                  60.800 usec
DE                   17.42 usec
TE                   300.0 K
D1              1.00000000 sec
TD0                      1
SFO1           400.1124708 MHz
NUC1                    1H
P0                    5.00 usec
P1                   15.00 usec
PLW1           17.29199982 W

F2 - Processing parameters
SI                   32768
SF             400.1100053 MHz
WDW                     EM
SSB      0
LB                    0.30 Hz
GB       0
PC                    1.00
```

Expanded region of the ^{1}H NMR spectrum of compound 6

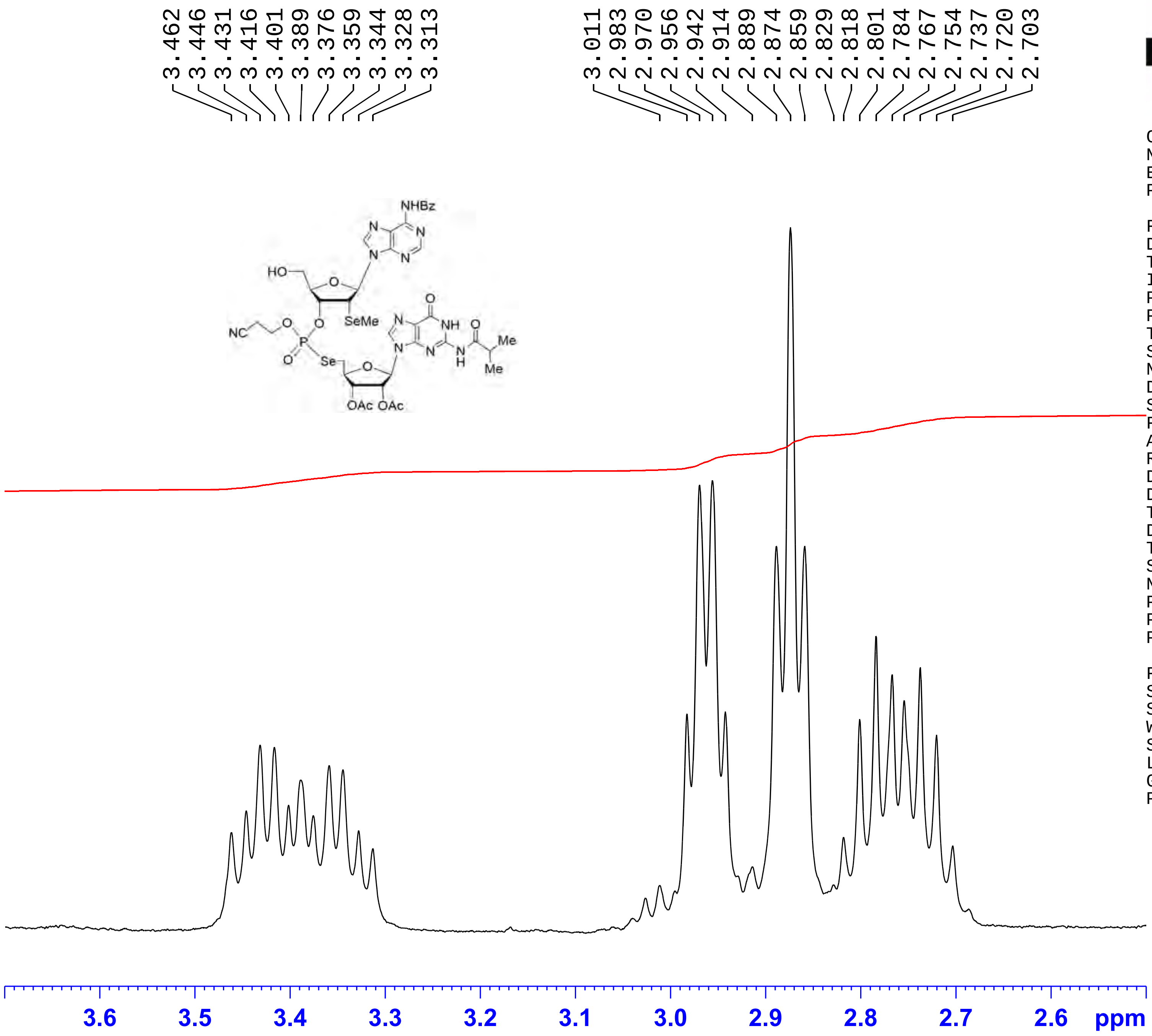

Current Data Parameters
NAME LH-II-64 OLD NMR
EXPNO 10
PROCNO 1

F2 - Acquisition Parameters
Date_ 20230719
Time 21.46 h
INSTRUM AVIII_400
PROBHD Z108618_0146 (
PULPROG zg30
TD 65536
SOLVENT CDCl3
NS 32
DS 2
SWH 8223.685 Hz
FIDRES 0.250967 Hz
AQ 3.9845889 sec
RG 80.6
DW 60.800 usec
DE 17.42 usec
TE 300.0 K
D1 1.00000000 sec
TD0 1
SFO1 400.1124708 MHz
NUC1 1H
P0 5.00 usec
P1 15.00 usec
PLW1 17.29199982 W

F2 - Processing parameters
SI 32768
SF 400.1100053 MHz
WDW EM
SSB 0
LB 0.30 Hz
GB 0
PC 1.00

Expanded region of the ^{1}H NMR spectrum of compound 6

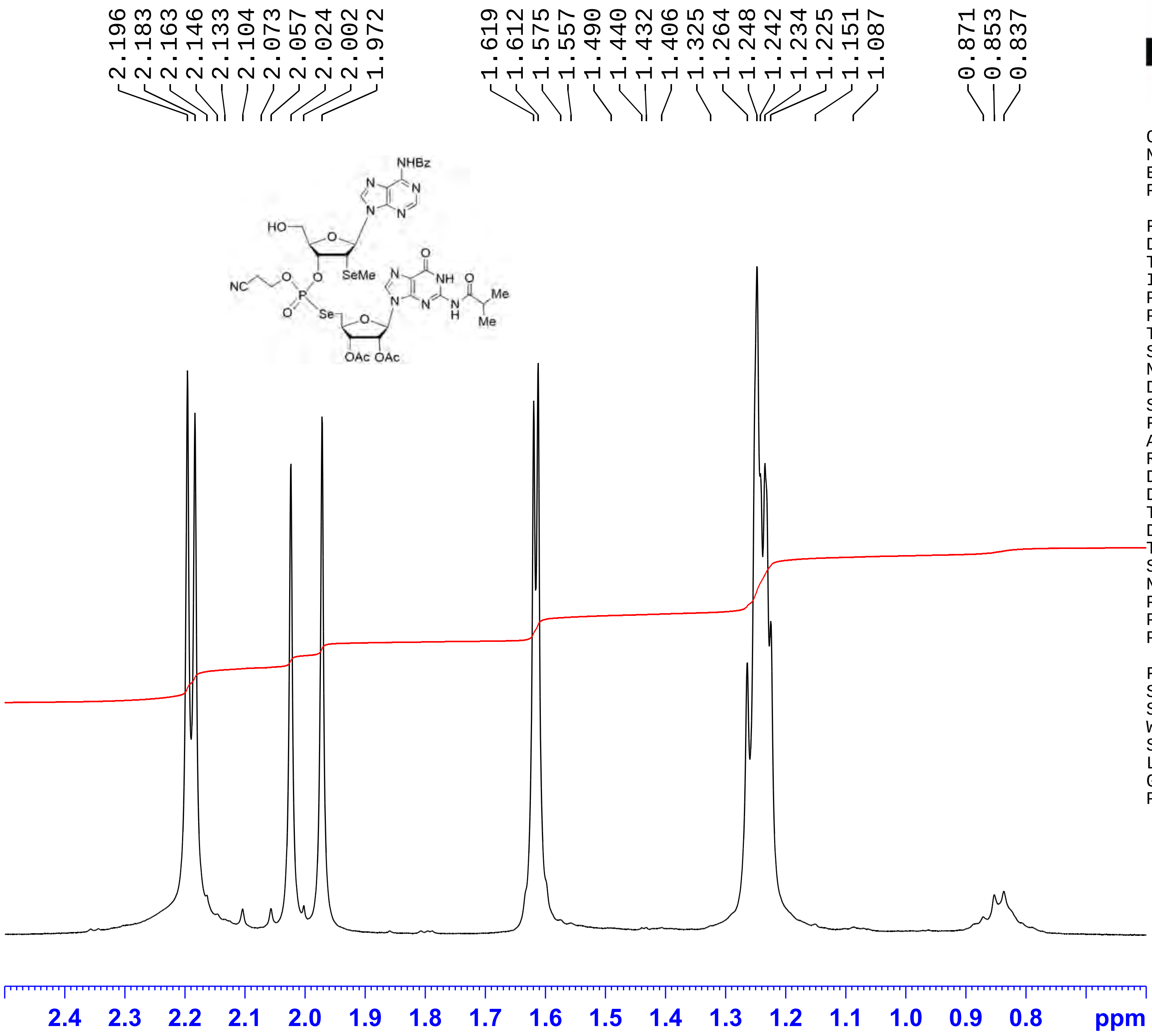

BRUKER

Current Data Parameters
NAME LH-II-64 OLD NMR
EXPNO 10
PROCNO 1

F2 - Acquisition Parameters
Date_ 20230719
Time 21.46 h
INSTRUM AVIII_400
PROBHD Z108618_0146 (
PULPROG zg30
TD 65536
SOLVENT CDCl3
NS 32
DS 2
SWH 8223.685 Hz
FIDRES 0.250967 Hz
AQ 3.9845889 sec
RG 80.6
DW 60.800 usec
DE 17.42 usec
TE 300.0 K
D1 1.00000000 sec
TD0 1
SFO1 400.1124708 MHz
NUC1 1H
P0 5.00 usec
P1 15.00 usec
PLW1 17.29199982 W

F2 - Processing parameters
SI 32768
SF 400.1100053 MHz
WDW EM
SSB 0
LB 0.30 Hz
GB 0
PC 1.00

^{13}C NMR spectrum of compound 6

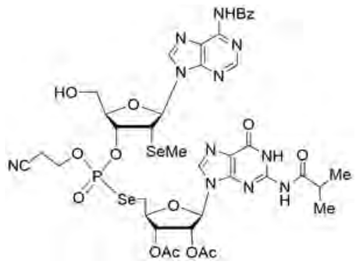

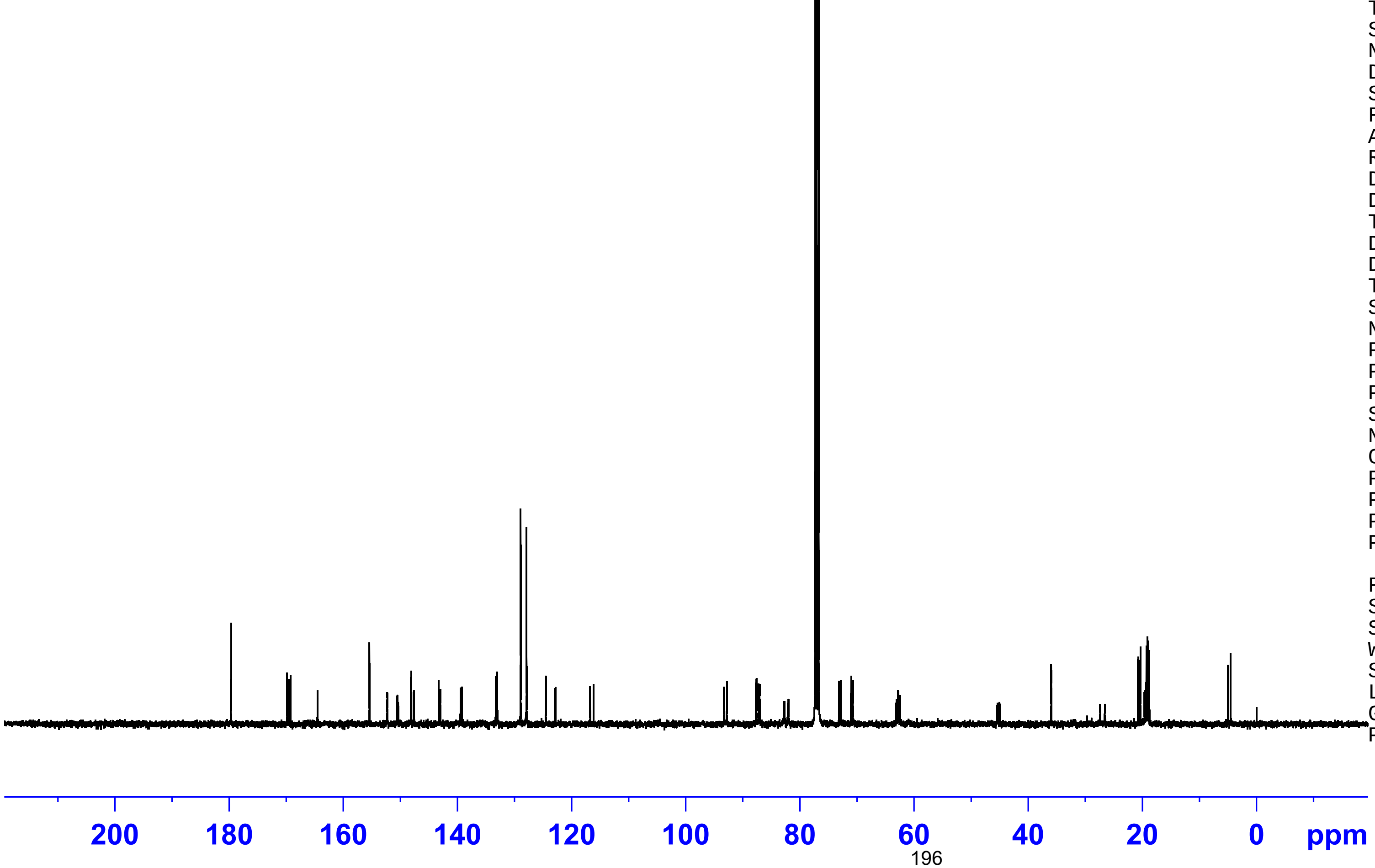

Current Data Parameters
NAME LH-II-64 OLD NMR
EXPNO 11
PROCNO 1

F2 - Acquisition Parameters
Date_ 20230720
Time 0.22 h
INSTRUM AVIII_400
PROBHD Z108618_0146 (
PULPROG zgpg30
TD 96150
SOLVENT CDCl3
NS 3000
DS 4
SWH 24038.461 Hz
FIDRES 0.500020 Hz
AQ 1.9999200 sec
RG 2050
DW 20.800 usec
DE 6.50 usec
TE 300.0 K
D1 1.00000000 sec
D11 0.03000000 sec
TD0 1
SFO1 100.6178003 MHz
NUC1 13C
P0 2.90 usec
P1 8.70 usec
PLW1 96.68000031 W
SFO2 400.1116004 MHz
NUC2 1H
CPDPRG[2 waltz64
PCPD2 90.00 usec
PLW2 17.29199982 W
PLW12 0.48032999 W
PLW13 0.24160001 W

F2 - Processing parameters
SI 131072
SF 100.6077468 MHz
WDW EM
SSB 0
LB 1.00 Hz
GB 0
PC 1.40

Expanded region of the ^{13}C NMR spectrum of compound 6

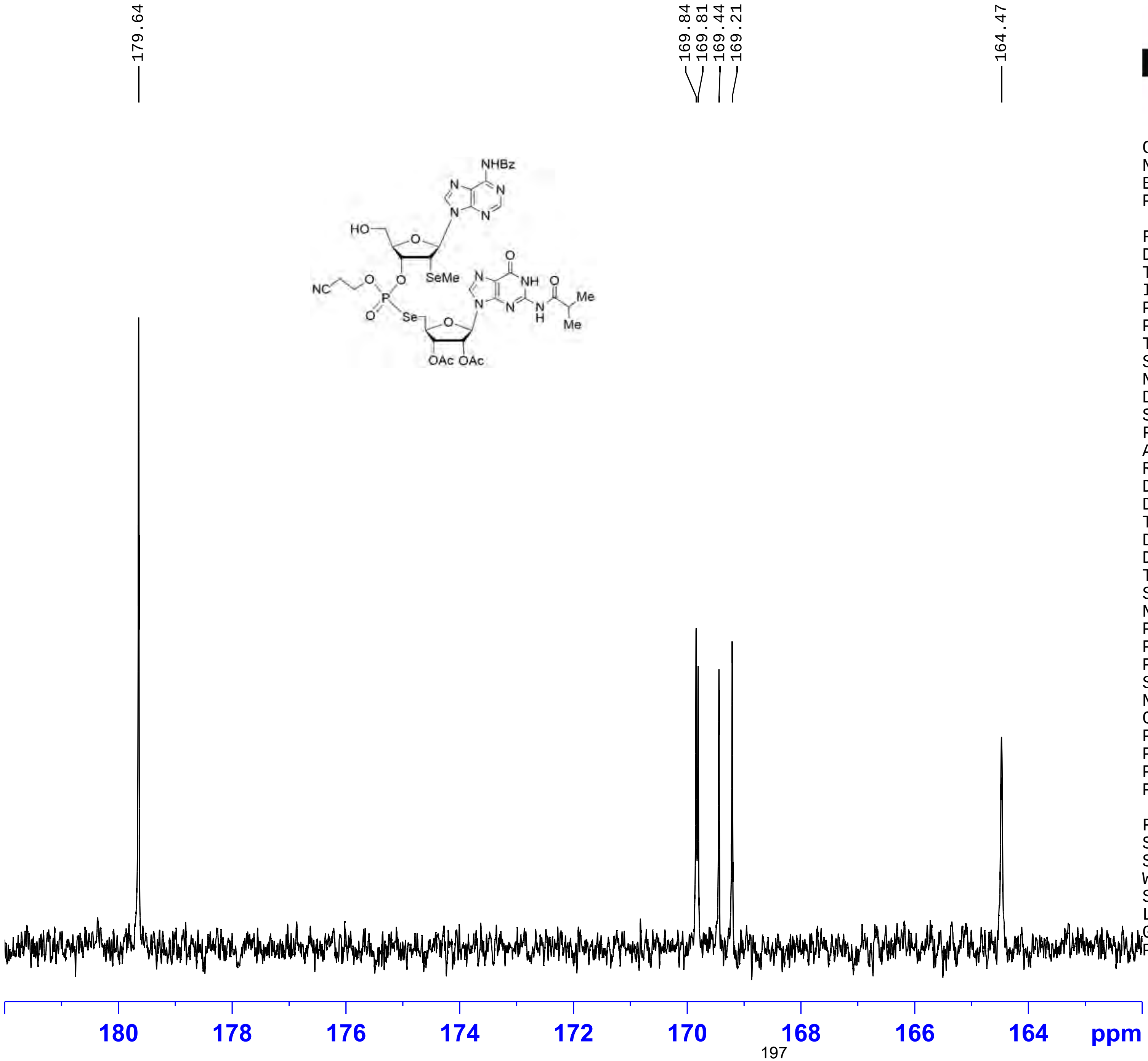

Current Data Parameters
NAME LH-II-64 OLD NMR
EXPNO 11
PROCNO 1

F2 - Acquisition Parameters
Date_ 20230720
Time 0.22 h
INSTRUM AVIII_400
PROBHD Z108618_0146 (
PULPROG zgpg30
TD 96150
SOLVENT CDCl3
NS 3000
DS 4
SWH 24038.461 Hz
FIDRES 0.500020 Hz
AQ 1.9999200 sec
RG 2050
DW 20.800 usec
DE 6.50 usec
TE 300.0 K
D1 1.00000000 sec
D11 0.03000000 sec
TD0 1
SFO1 100.6178003 MHz
NUC1 13C
P0 2.90 usec
P1 8.70 usec
PLW1 96.68000031 W
SFO2 400.1116004 MHz
NUC2 1H
CPDPRG[2 waltz64
PCPD2 90.00 usec
PLW2 17.29199982 W
PLW12 0.48032999 W
PLW13 0.24160001 W

F2 - Processing parameters
SI 131072
SF 100.6077468 MHz
WDW EM
SSB 0
LB 1.00 Hz
GB 0
PC 1.40

Expanded region of the ^{13}C NMR spectrum of compound 6

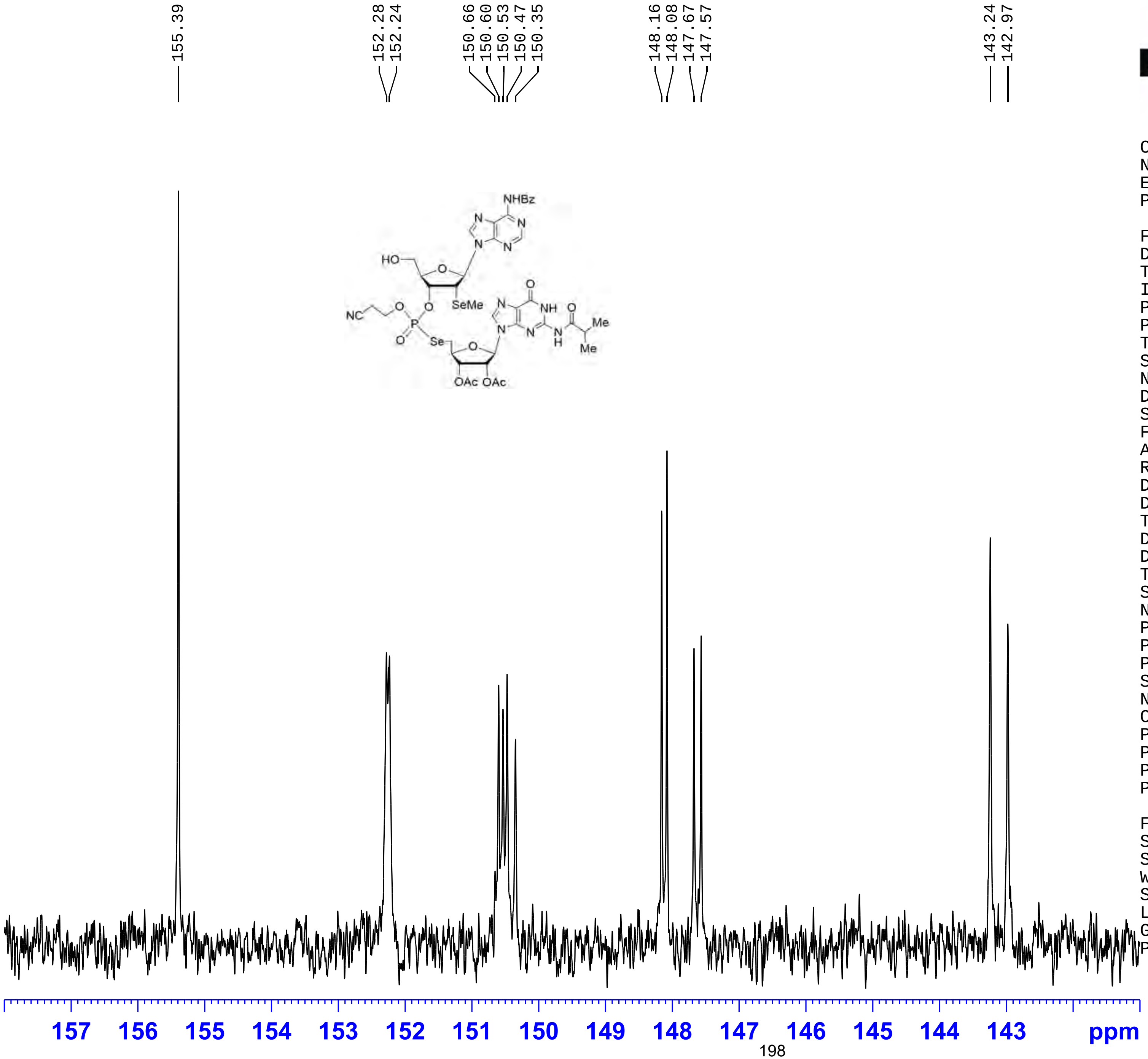

Current Data Parameters
NAME LH-II-64 OLD NMR
EXPNO 11
PROCNO 1

F2 - Acquisition Parameters
Date_ 20230720
Time 0.22 h
INSTRUM AVIII_400
PROBHD Z108618_0146 (
PULPROG zgpg30
TD 96150
SOLVENT CDCl3
NS 3000
DS 4
SWH 24038.461 Hz
FIDRES 0.500020 Hz
AQ 1.9999200 sec
RG 2050
DW 20.800 usec
DE 6.50 usec
TE 300.0 K
D1 1.00000000 sec
D11 0.03000000 sec
TD0 1
SFO1 100.6178003 MHz
NUC1 13C
P0 2.90 usec
P1 8.70 usec
PLW1 96.68000031 W
SFO2 400.1116004 MHz
NUC2 1H
CPDPRG[2 waltz64
PCPD2 90.00 usec
PLW2 17.29199982 W
PLW12 0.48032999 W
PLW13 0.24160001 W

F2 - Processing parameters
SI 131072
SF 100.6077468 MHz
WDW EM
SSB 0
LB 1.00 Hz
GB 0
PC 1.40

Expanded region of the ^{13}C NMR spectrum of compound 6

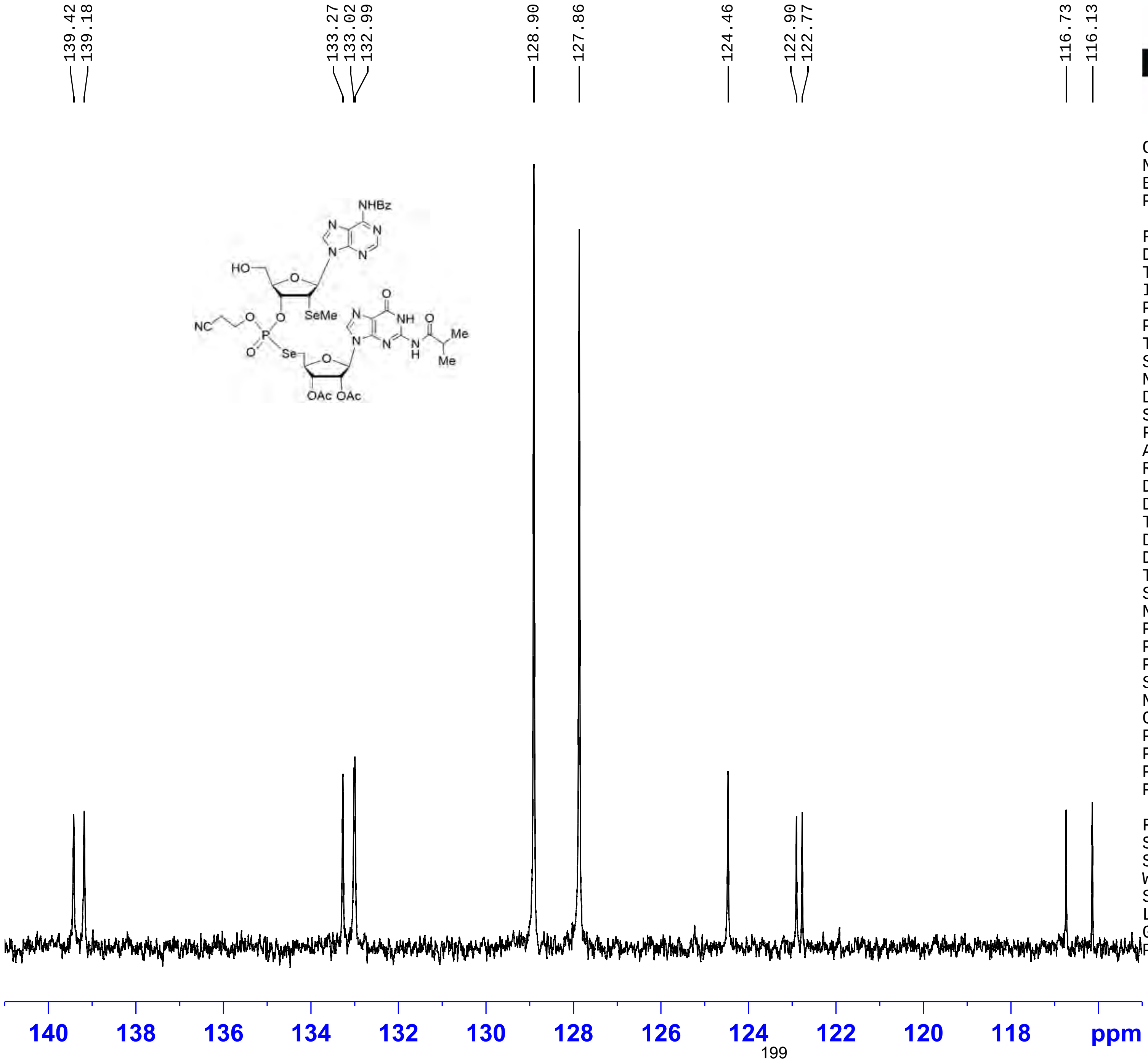

Current Data Parameters
NAME LH-II-64 OLD NMR
EXPNO 11
PROCNO 1

F2 - Acquisition Parameters
Date_ 20230720
Time 0.22 h
INSTRUM AVIII_400
PROBHD Z108618_0146 (
PULPROG zgpg30
TD 96150
SOLVENT CDCl3
NS 3000
DS 4
SWH 24038.461 Hz
FIDRES 0.500020 Hz
AQ 1.9999200 sec
RG 2050
DW 20.800 usec
DE 6.50 usec
TE 300.0 K
D1 1.00000000 sec
D11 0.03000000 sec
TD0 1
SFO1 100.6178003 MHz
NUC1 13C
P0 2.90 usec
P1 8.70 usec
PLW1 96.68000031 W
SFO2 400.1116004 MHz
NUC2 1H
CPDPRG[2 waltz64
PCPD2 90.00 usec
PLW2 17.29199982 W
PLW12 0.48032999 W
PLW13 0.24160001 W

F2 - Processing parameters
SI 131072
SF 100.6077468 MHz
WDW EM
SSB 0
LB 1.00 Hz
GB 0
PC 1.40

Expanded region of the ^{13}C NMR spectrum of compound 6

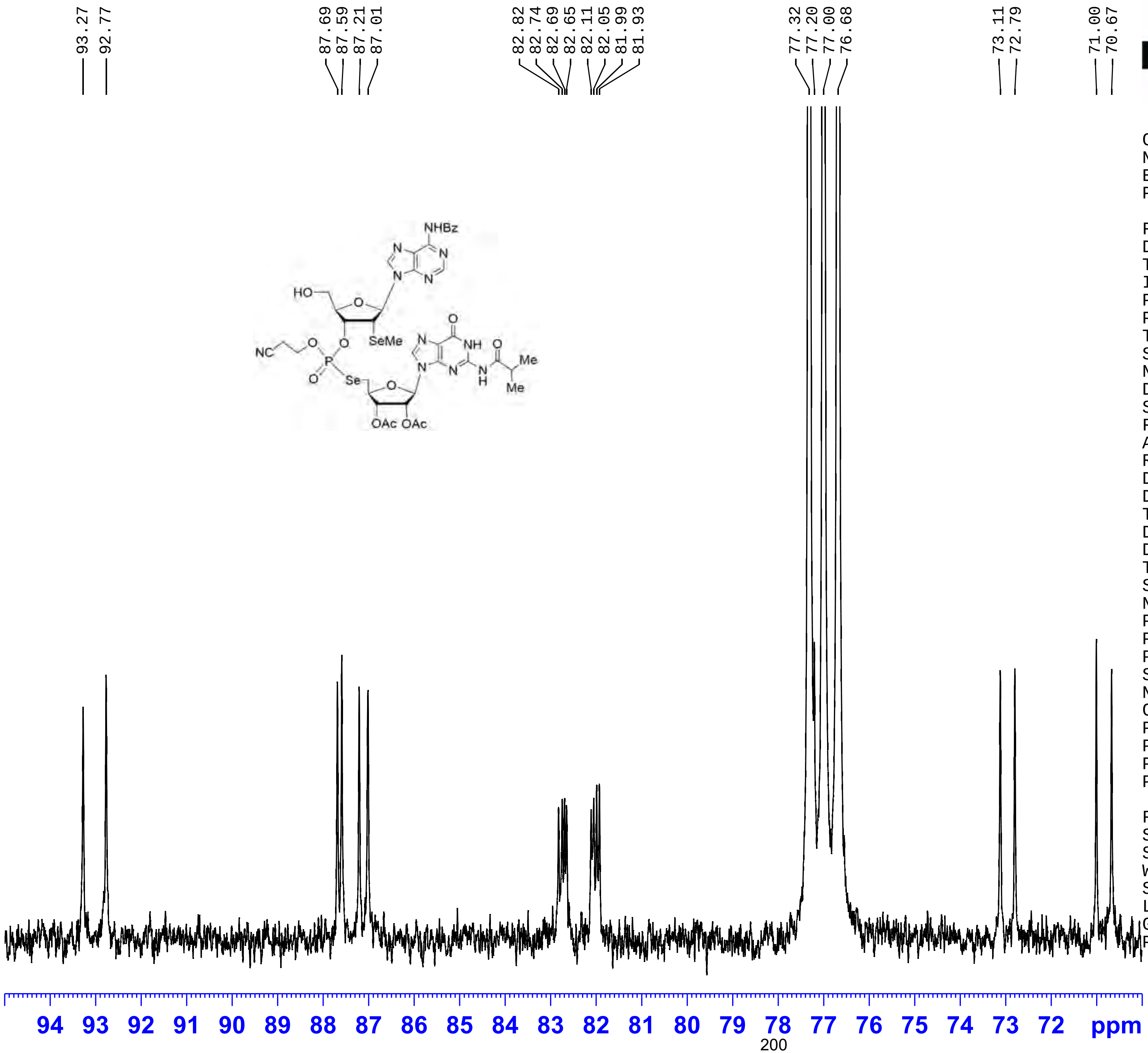

BRUKER

Current Data Parameters
NAME LH-II-64 OLD NMR
EXPNO 11
PROCNO 1

F2 - Acquisition Parameters
Date_ 20230720
Time 0.22 h
INSTRUM AVIII_400
PROBHD Z108618_0146 (
PULPROG zgpg30
TD 96150
SOLVENT CDCl3
NS 3000
DS 4
SWH 24038.461 Hz
FIDRES 0.500020 Hz
AQ 1.9999200 sec
RG 2050
DW 20.800 usec
DE 6.50 usec
TE 300.0 K
D1 1.00000000 sec
D11 0.03000000 sec
TD0 1
SFO1 100.6178003 MHz
NUC1 13C
P0 2.90 usec
P1 8.70 usec
PLW1 96.68000031 W
SFO2 400.1116004 MHz
NUC2 1H
CPDPRG[2 waltz64
PCPD2 90.00 usec
PLW2 17.29199982 W
PLW12 0.48032999 W
PLW13 0.24160001 W

F2 - Processing parameters
SI 131072
SF 100.6077468 MHz
WDW EM
SSB 0
LB 1.00 Hz
GB 0
PC 1.40

Expanded region of the ^{13}C NMR spectrum of compound 6

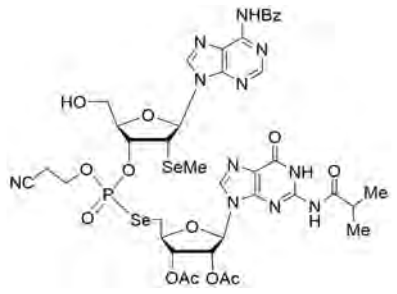

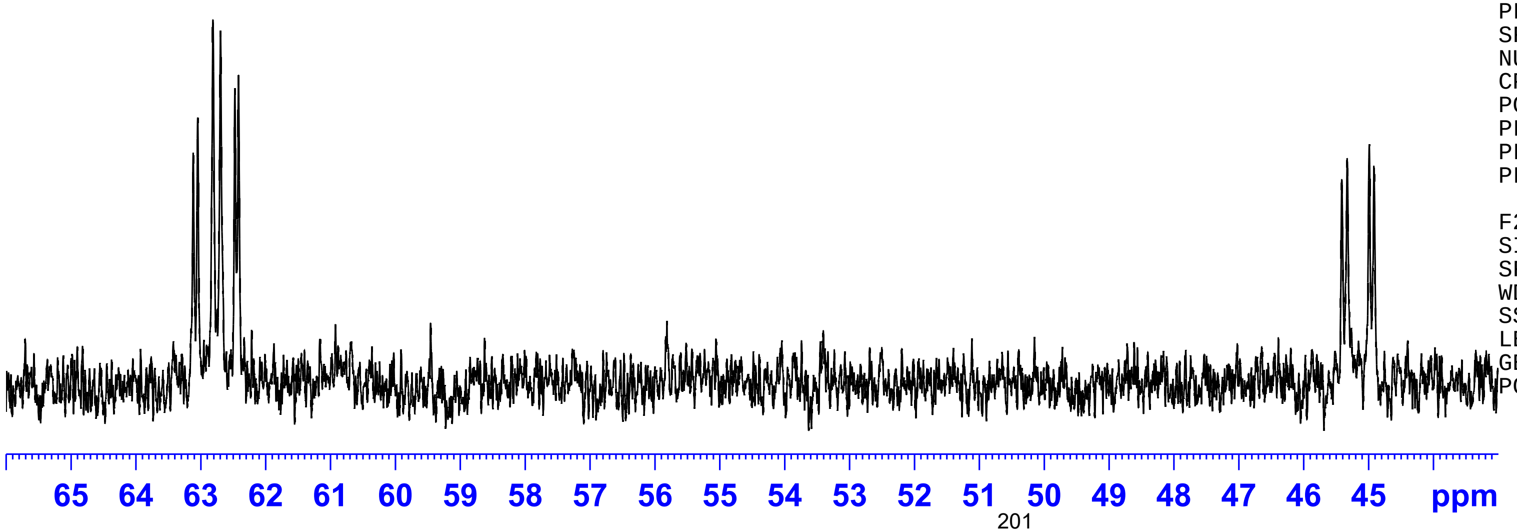


```
Current Data Parameters
NAME       LH-II-64 OLD NMR
EXPNO                    11
PROCNO                    1

F2 - Acquisition Parameters
Date_              20230720
Time                  0.22 h
INSTRUM           AVIII_400
PROBHD      Z108618_0146 (
PULPROG             zgpg30
TD                   96150
SOLVENT              CDCl3
NS                    3000
DS                       4
SWH              24038.461 Hz
FIDRES            0.500020 Hz
AQ               1.9999200 sec
RG                    2050
DW                  20.800 usec
DE                    6.50 usec
TE                   300.0 K
D1              1.00000000 sec
D11             0.03000000 sec
TD0                      1
SFO1           100.6178003 MHz
NUC1                   13C
P0                    2.90 usec
P1                    8.70 usec
PLW1           96.68000031 W
SFO2           400.1116004 MHz
NUC2                    1H
CPDPRG[2           waltz64
PCPD2                90.00 usec
PLW2           17.29199982 W
PLW12           0.48032999 W
PLW13           0.24160001 W

F2 - Processing parameters
SI                  131072
SF             100.6077468 MHz
WDW                     EM
SSB        0
LB                    1.00 Hz
GB         0
PC                    1.40
```

Expanded region of the ^{13}C NMR spectrum of compound 6

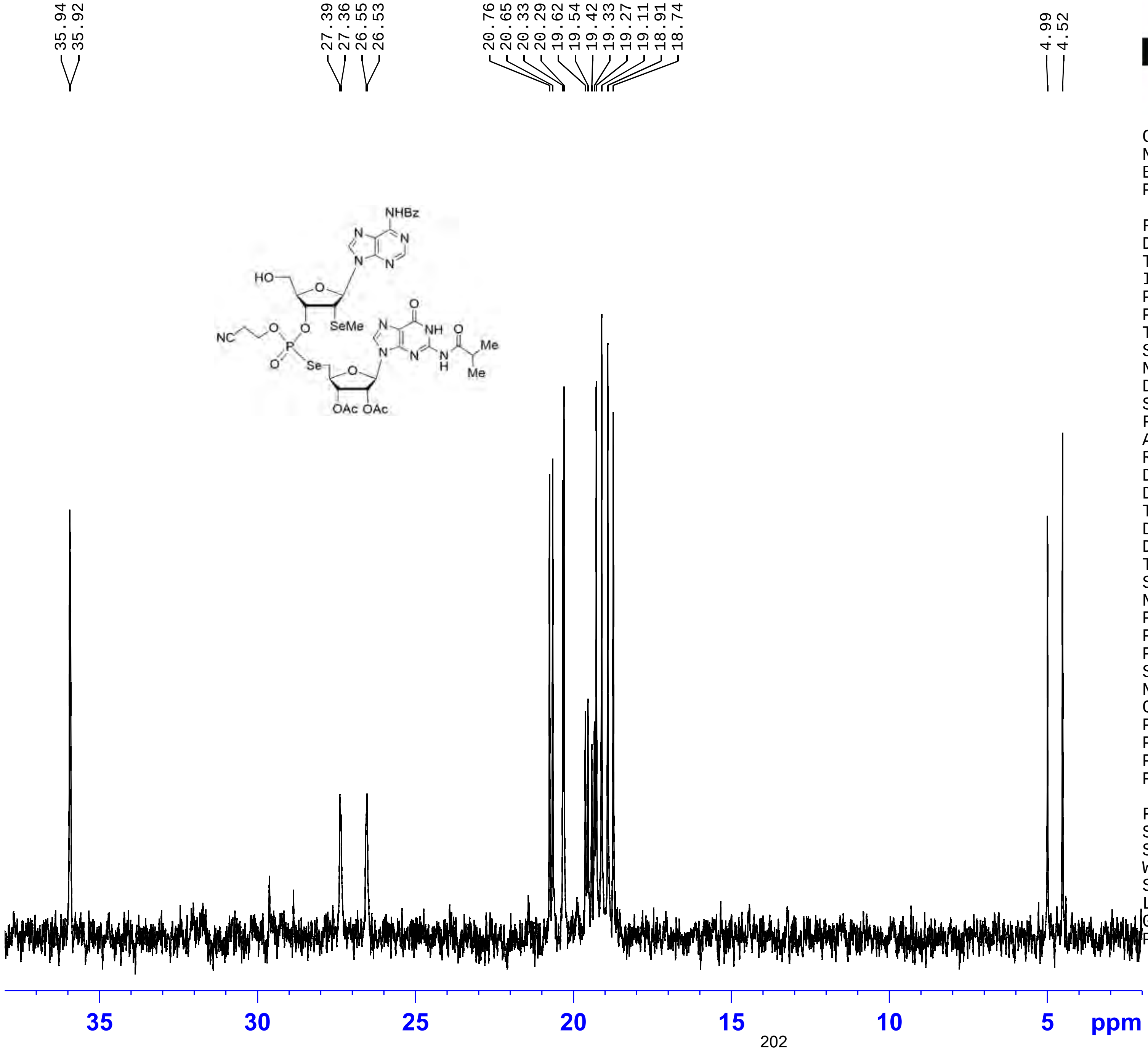


```
Current Data Parameters
NAME      LH-II-64 OLD NMR
EXPNO                   11
PROCNO                   1

F2 - Acquisition Parameters
Date_           20230720
Time                0.22 h
INSTRUM         AVIII_400
PROBHD   Z108618_0146 (
PULPROG           zgpg30
TD                 96150
SOLVENT            CDCl3
NS                  3000
DS                     4
SWH            24038.461 Hz
FIDRES          0.500020 Hz
AQ             1.9999200 sec
RG                  2050
DW                20.800 usec
DE                  6.50 usec
TE                 300.0 K
D1            1.00000000 sec
D11           0.03000000 sec
TD0                    1
SFO1         100.6178003 MHz
NUC1                 13C
P0                  2.90 usec
P1                  8.70 usec
PLW1         96.68000031 W
SFO2         400.1116004 MHz
NUC2                  1H
CPDPRG[2         waltz64
PCPD2              90.00 usec
PLW2         17.29199982 W
PLW12         0.48032999 W
PLW13         0.24160001 W

F2 - Processing parameters
SI                131072
SF           100.6077468 MHz
WDW                   EM
SSB      0
LB                  1.00 Hz
GB       0
PC                  1.40
```

Expanded region of the ^{13}C NMR spectrum of compound 6

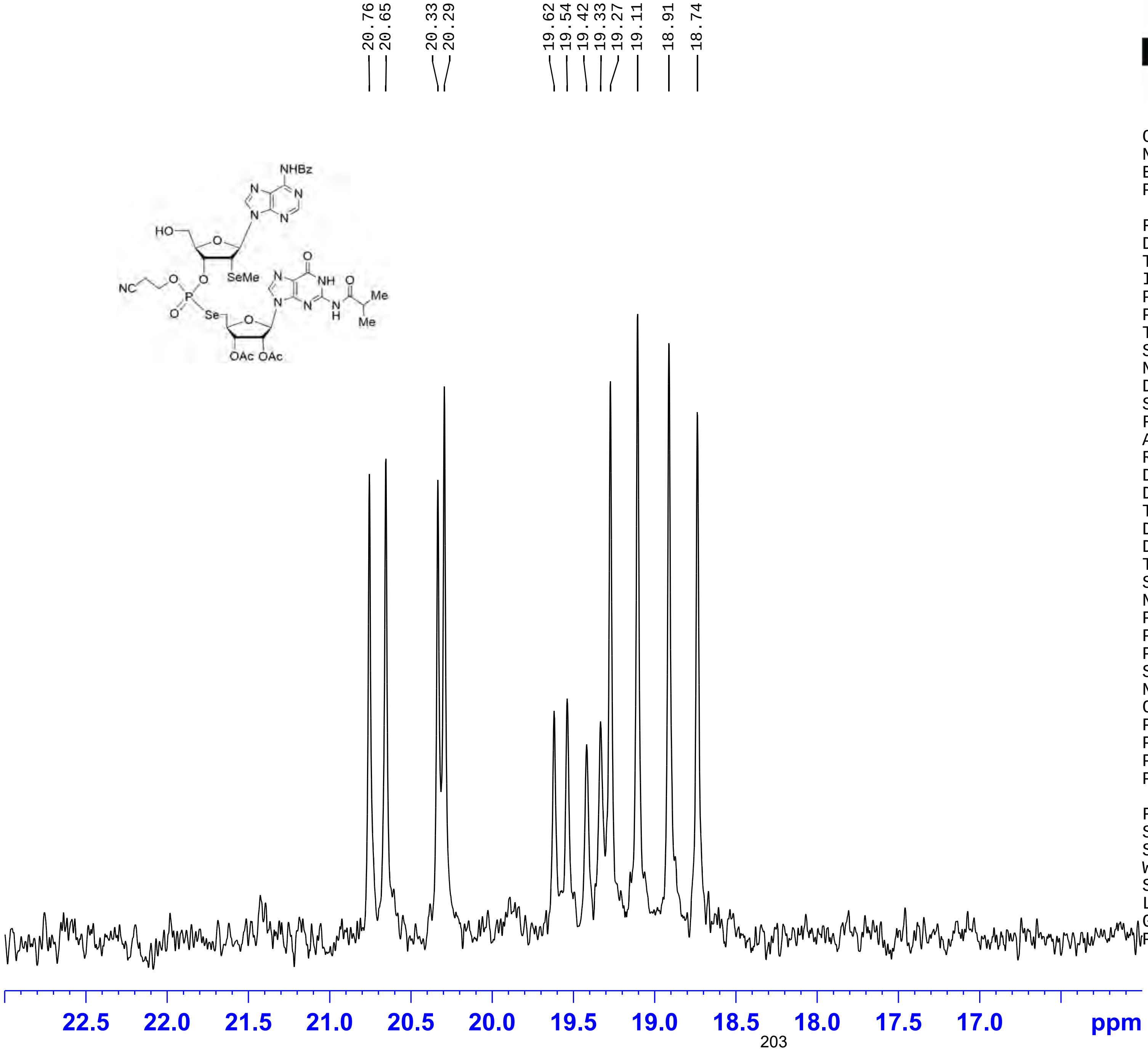

Current Data Parameters
NAME LH-II-64 OLD NMR
EXPNO 11
PROCNO 1

F2 - Acquisition Parameters

Date_	20230720
Time	0.22 h
INSTRUM	AVIII_400
PROBHD	Z108618_0146 (
PULPROG	zgpg30
TD	96150
SOLVENT	CDCl3
NS	3000
DS	4
SWH	24038.461 Hz
FIDRES	0.500020 Hz
AQ	1.9999200 sec
RG	2050
DW	20.800 usec
DE	6.50 usec
TE	300.0 K
D1	1.00000000 sec
D11	0.03000000 sec
TD0	1
SFO1	100.6178003 MHz
NUC1	13C
P0	2.90 usec
P1	8.70 usec
PLW1	96.68000031 W
SFO2	400.1116004 MHz
NUC2	1H
CPDPRG[2	waltz64
PCPD2	90.00 usec
PLW2	17.29199982 W
PLW12	0.48032999 W
PLW13	0.24160001 W

F2 - Processing parameters

SI	131072
SF	100.6077468 MHz
WDW	EM
SSB	0
LB	1.00 Hz
GB	0
PC	1.40

^{13}C DEPT-135 NMR spectrum of compound 6

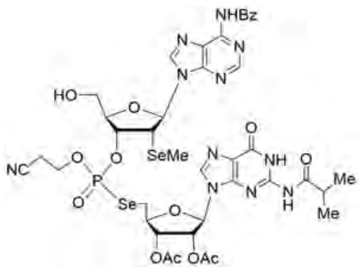

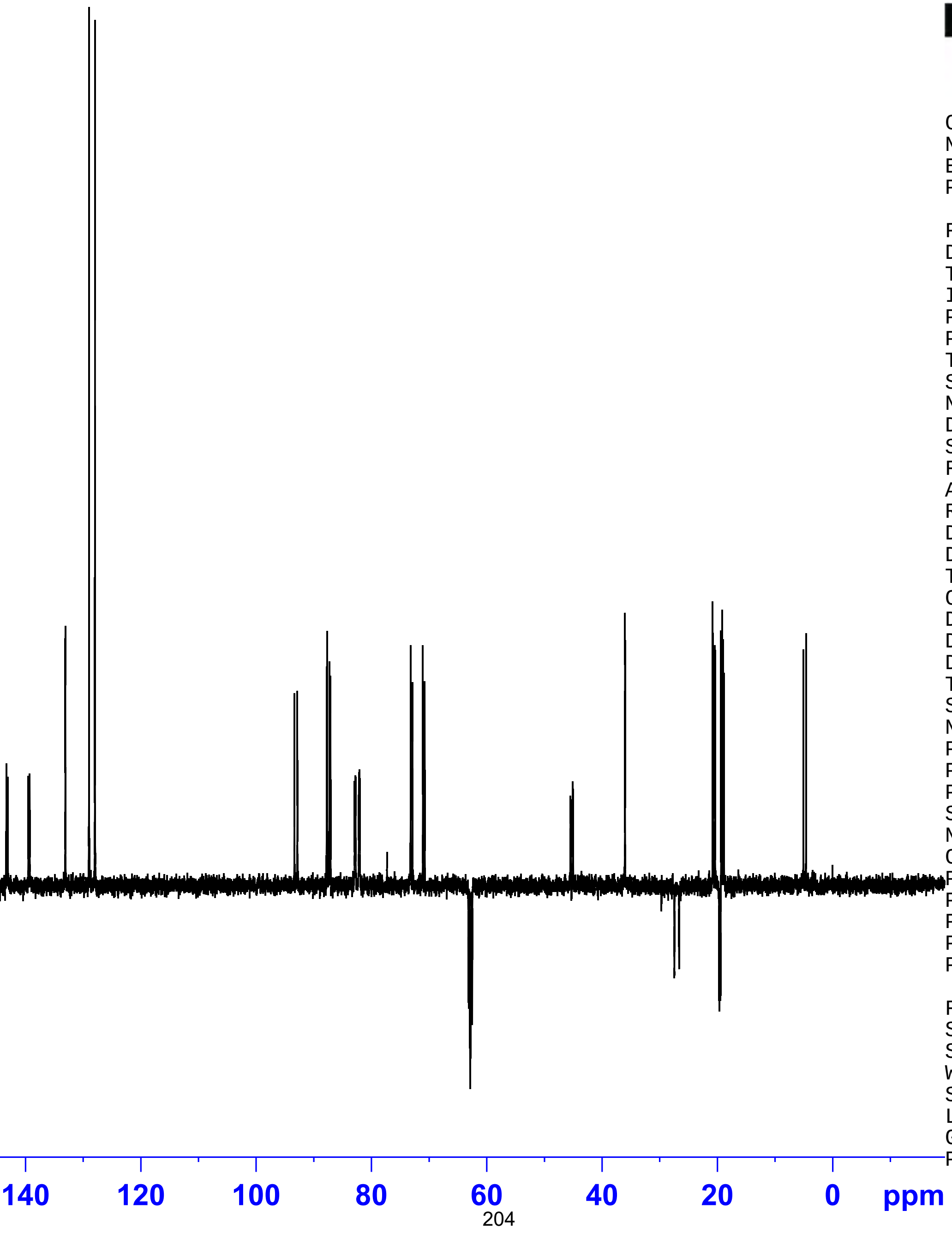

Current Data Parameters
NAME LH-II-64 OLD NMR
EXPNO 12
PROCNO 1

F2 - Acquisition Parameters
Date_ 20230720
Time 1.51 h
INSTRUM AVIII_400
PROBHD Z108618_0146 (
PULPROG dept135
TD 65536
SOLVENT CDCl3
NS 1500
DS 4
SWH 24038.461 Hz
FIDRES 0.733596 Hz
AQ 1.3631488 sec
RG 2050
DW 20.800 usec
DE 6.50 usec
TE 300.0 K
CNST2 145.0000000
D1 2.00000000 sec
D2 0.00344828 sec
D12 0.00002000 sec
TD0 1
SFO1 100.6178003 MHz
NUC1 13C
P1 8.70 usec
P2 17.40 usec
PLW1 96.68000031 W
SFO2 400.1116004 MHz
NUC2 1H
CPDPRG[2 waltz64
P3 15.00 usec
P4 30.00 usec
PCPD2 90.00 usec
PLW2 17.29199982 W
PLW12 0.48032999 W

F2 - Processing parameters
SI 32768
SF 100.6077400 MHz
WDW EM
SSB 0
LB 1.00 Hz
GB 0
PC 1.40

Expanded region of the ^{13}C DEPT-135 NMR spectrum of compound 6

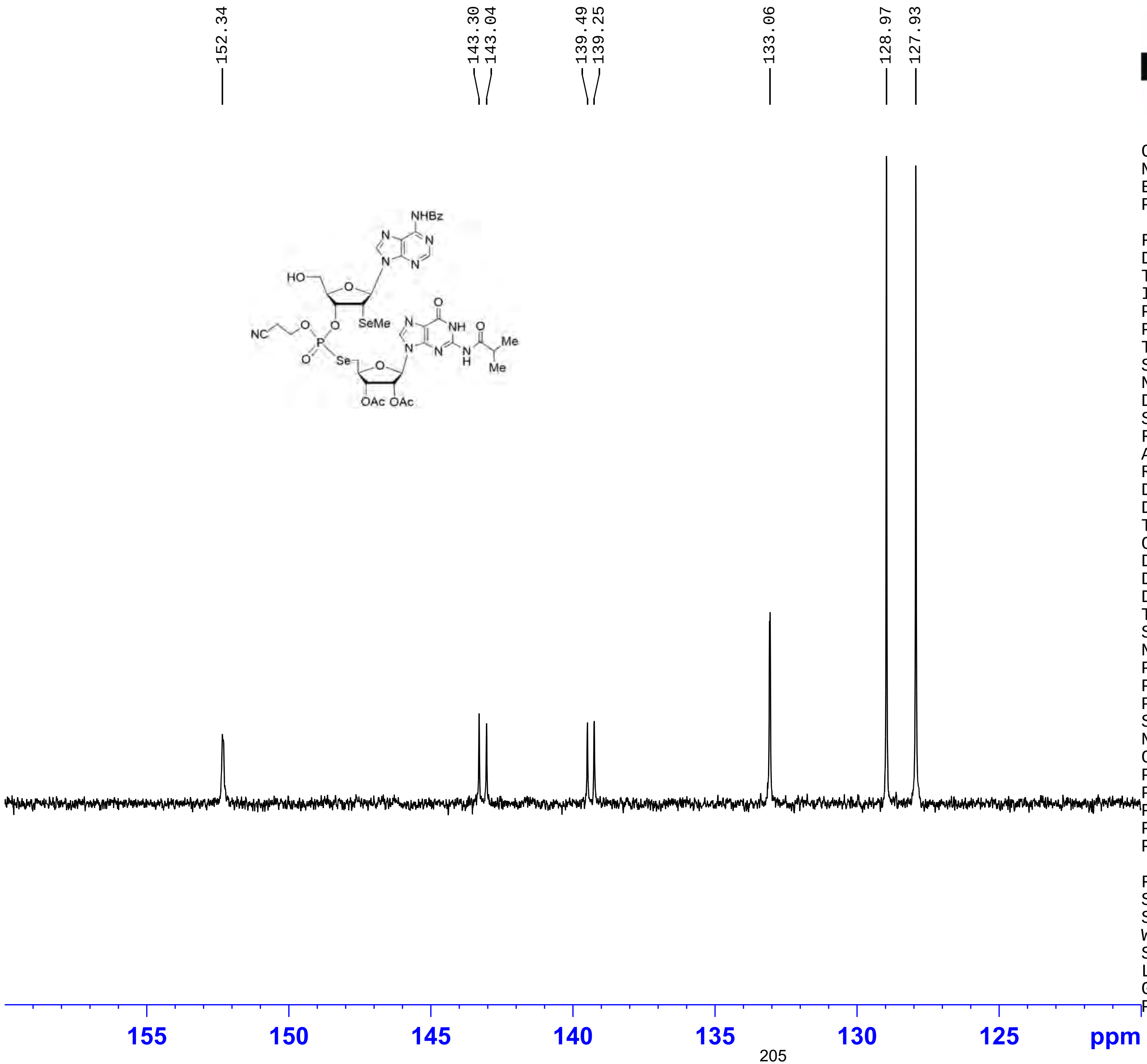

BRUKER

Current Data Parameters
NAME LH-II-64 OLD NMR
EXPNO 12
PROCNO 1

F2 - Acquisition Parameters
Date_ 20230720
Time 1.51 h
INSTRUM AVIII_400
PROBHD Z108618_0146 (
PULPROG dept135
TD 65536
SOLVENT CDCl3
NS 1500
DS 4
SWH 24038.461 Hz
FIDRES 0.733596 Hz
AQ 1.3631488 sec
RG 2050
DW 20.800 usec
DE 6.50 usec
TE 300.0 K
CNST2 145.0000000
D1 2.00000000 sec
D2 0.00344828 sec
D12 0.00002000 sec
TD0 1
SFO1 100.6178003 MHz
NUC1 13C
P1 8.70 usec
P2 17.40 usec
PLW1 96.68000031 W
SFO2 400.1116004 MHz
NUC2 1H
CPDPRG[2 waltz64
P3 15.00 usec
P4 30.00 usec
PCPD2 90.00 usec
PLW2 17.29199982 W
PLW12 0.48032999 W

F2 - Processing parameters
SI 32768
SF 100.6077400 MHz
WDW EM
SSB 0
LB 1.00 Hz
GB 0
PC 1.40

Expanded region of the ^{13}C DEPT-135 NMR spectrum of compound 6

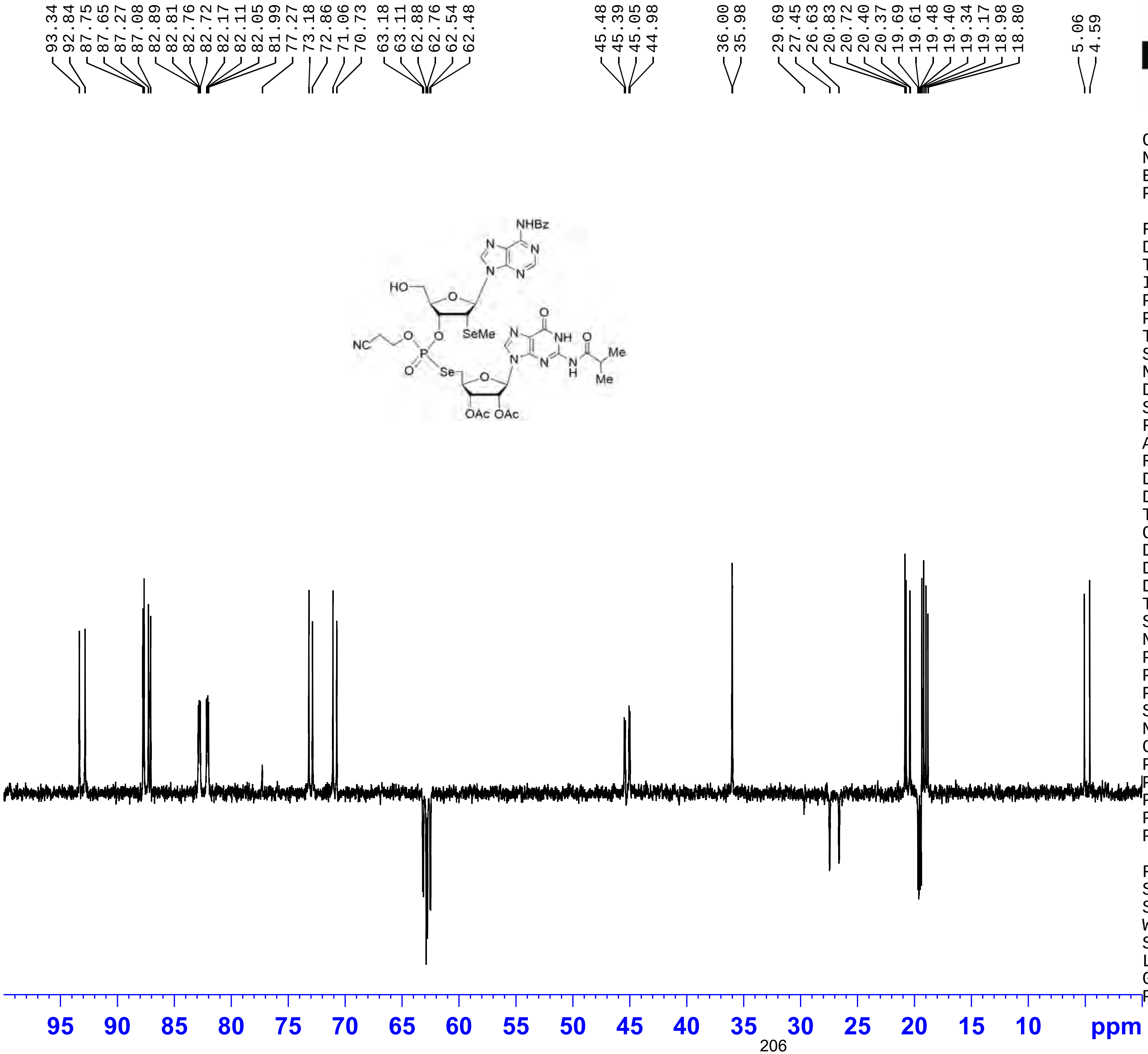


```
Current Data Parameters
NAME       LH-II-64 OLD NMR
EXPNO                    12
PROCNO                    1

F2 - Acquisition Parameters
Date_              20230720
Time                   1.51 h
INSTRUM           AVIII_400
PROBHD       Z108618_0146 (
PULPROG             dept135
TD                    65536
SOLVENT               CDCl3
NS                     1500
DS                        4
SWH               24038.461 Hz
FIDRES             0.733596 Hz
AQ                1.3631488 sec
RG                     2050
DW                   20.800 usec
DE                     6.50 usec
TE                    300.0 K
CNST2           145.0000000
D1               2.00000000 sec
D2               0.00344828 sec
D12              0.00002000 sec
TD0                       1
SFO1            100.6178003 MHz
NUC1                    13C
P1                     8.70 usec
P2                    17.40 usec
PLW1            96.68000031 W
SFO2            400.1116004 MHz
NUC2                     1H
CPDPRG[2            waltz64
P3                    15.00 usec
P4                    30.00 usec
PCPD2                 90.00 usec
PLW2            17.29199982 W
PLW12            0.48032999 W

F2 - Processing parameters
SI                    32768
SF              100.6077400 MHz
WDW                      EM
SSB      0
LB                     1.00 Hz
GB       0
PC                     1.40
```

Expanded region of the ^{13}C DEPT-135 NMR spectrum of compound 6

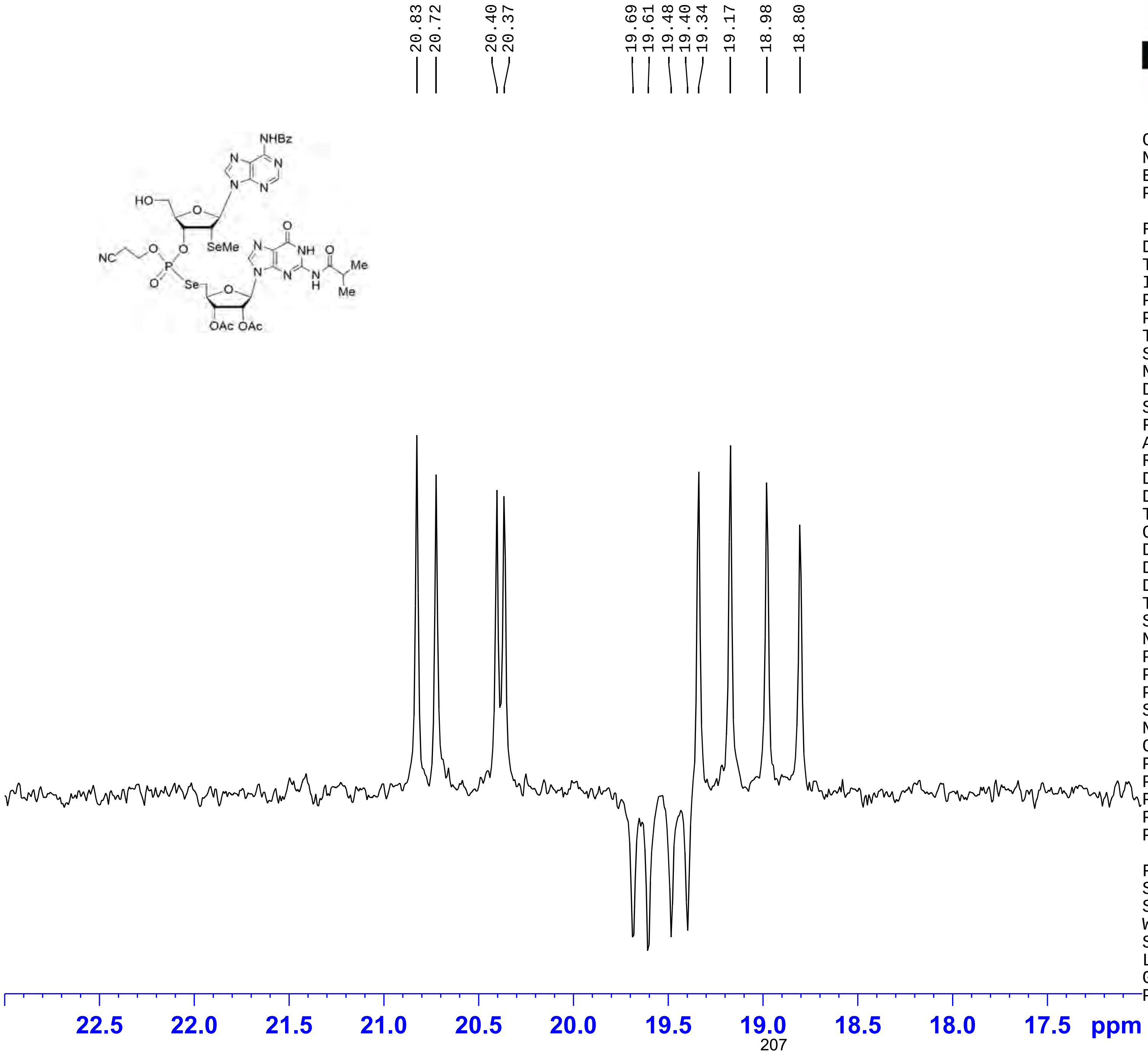


```
Current Data Parameters
NAME       LH-II-64 OLD NMR
EXPNO                    12
PROCNO                    1

F2 - Acquisition Parameters
Date_              20230720
Time                   1.51 h
INSTRUM           AVIII_400
PROBHD      Z108618_0146 (
PULPROG             dept135
TD                    65536
SOLVENT               CDCl3
NS                     1500
DS                        4
SWH               24038.461 Hz
FIDRES             0.733596 Hz
AQ                1.3631488 sec
RG                     2050
DW                   20.800 usec
DE                     6.50 usec
TE                    300.0 K
CNST2        145.0000000
D1            2.00000000 sec
D2            0.00344828 sec
D12           0.00002000 sec
TD0                       1
SFO1        100.6178003 MHz
NUC1                    13C
P1                     8.70 usec
P2                    17.40 usec
PLW1         96.68000031 W
SFO2        400.1116004 MHz
NUC2                     1H
CPDPRG[2            waltz64
P3                    15.00 usec
P4                    30.00 usec
PCPD2                 90.00 usec
PLW2         17.29199982 W
PLW12         0.48032999 W

F2 - Processing parameters
SI                    32768
SF          100.6077400 MHz
WDW                      EM
SSB       0
LB                     1.00 Hz
GB        0
PC                     1.40
```

^{1}H-^{1}H COSY NMR spectrum of compound 6

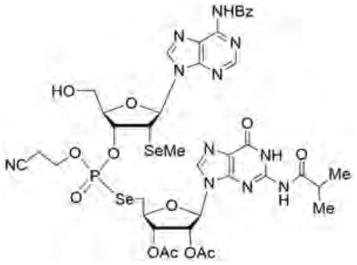

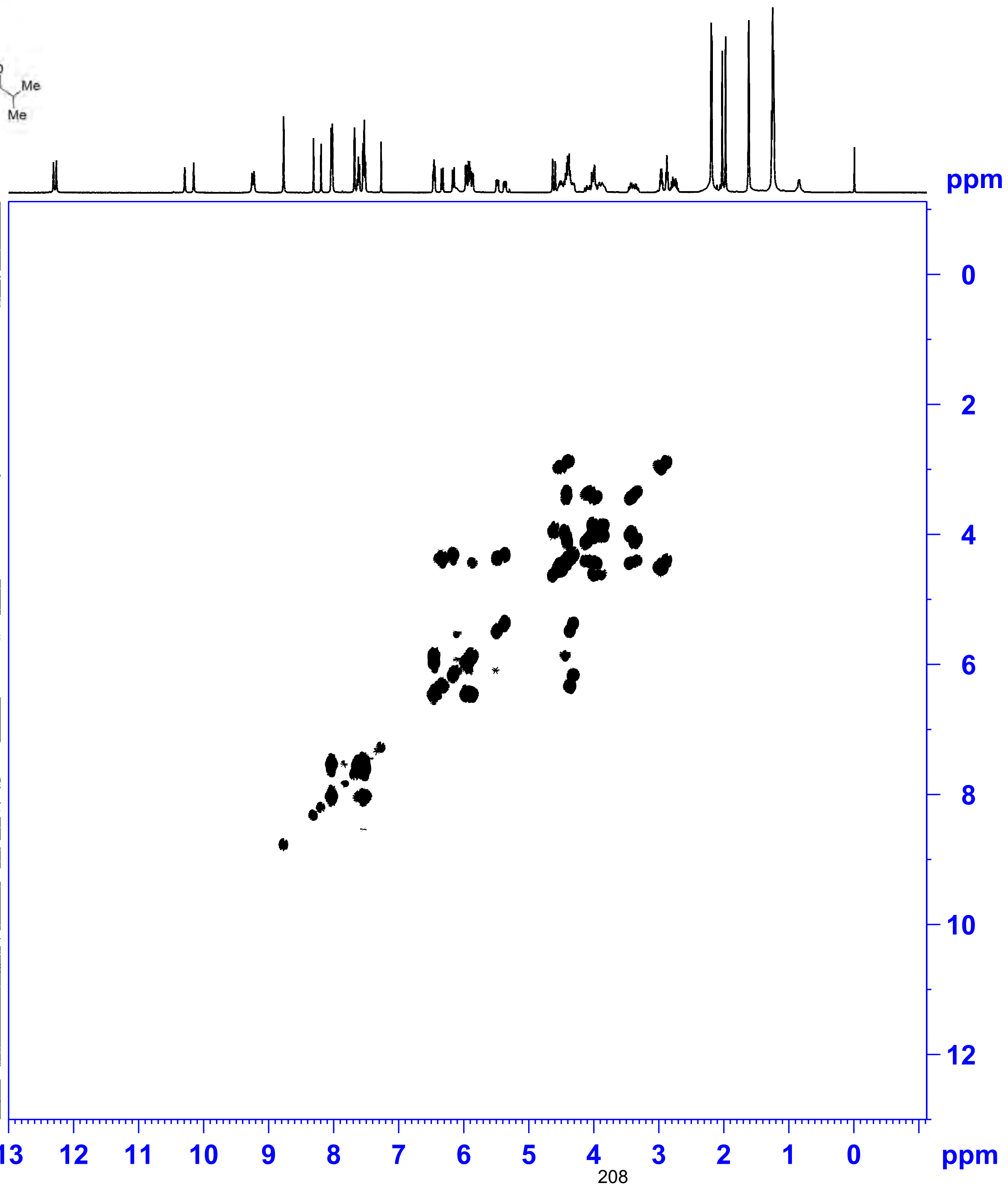


```
Current Data Parameters
NAME      LH-II-64 NEW NMR
EXPNO                   11
PROCNO                   1

F2 - Acquisition Parameters
Date_             20230720
Time                 12.33 h
INSTRUM           AVIII_400
PROBHD   Z108618_0817 (
PULPROG        cosygpmfqf
TD                    2048
SOLVENT              CDCl3
NS                       3
DS                       8
SWH               6172.839 Hz
FIDRES            6.028163 Hz
AQ               0.1658880 sec
RG                    2050
DW                  81.000 usec
DE                    6.50 usec
TE                   295.3 K
D0             0.00000300 sec
D1             2.02607393 sec
D13            0.00000400 sec
D16            0.00020000 sec
IN0            0.00016200 sec
TDav                     1
SFO1           399.9124600 MHz
NUC1                    1H
P1                  500.00 usec
PLW1           31.62299919 W
GPNAM[1]          SINE.100
GPZ1                 16.00 %
GPNAM[2]          SINE.100
GPZ2                 12.00 %
GPNAM[3]          SINE.100
GPZ3                 40.00 %
P16                1000.00 usec

F1 - Acquisition parameters
TD                     256
SFO1              399.9125 MHz
FIDRES           48.225307 Hz
SW                  15.435 ppm
FnMODE                  QF

F2 - Processing parameters
SI                    1024
SF             399.9100018 MHz
WDW                   SINE
SSB      0
LB       0 Hz
GB       0
PC                    1.40

F1 - Processing parameters
SI                    1024
MC2                     QF
SF             399.9100018 MHz
WDW                   SINE
SSB      0
LB       0 Hz
GB       0
```

Expanded region of ^{1}H-^{1}H COSY NMR spectrum of compound 6

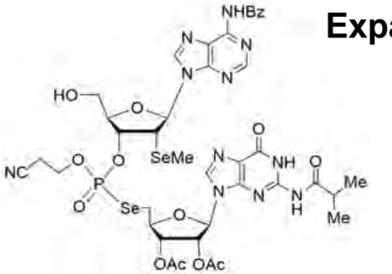

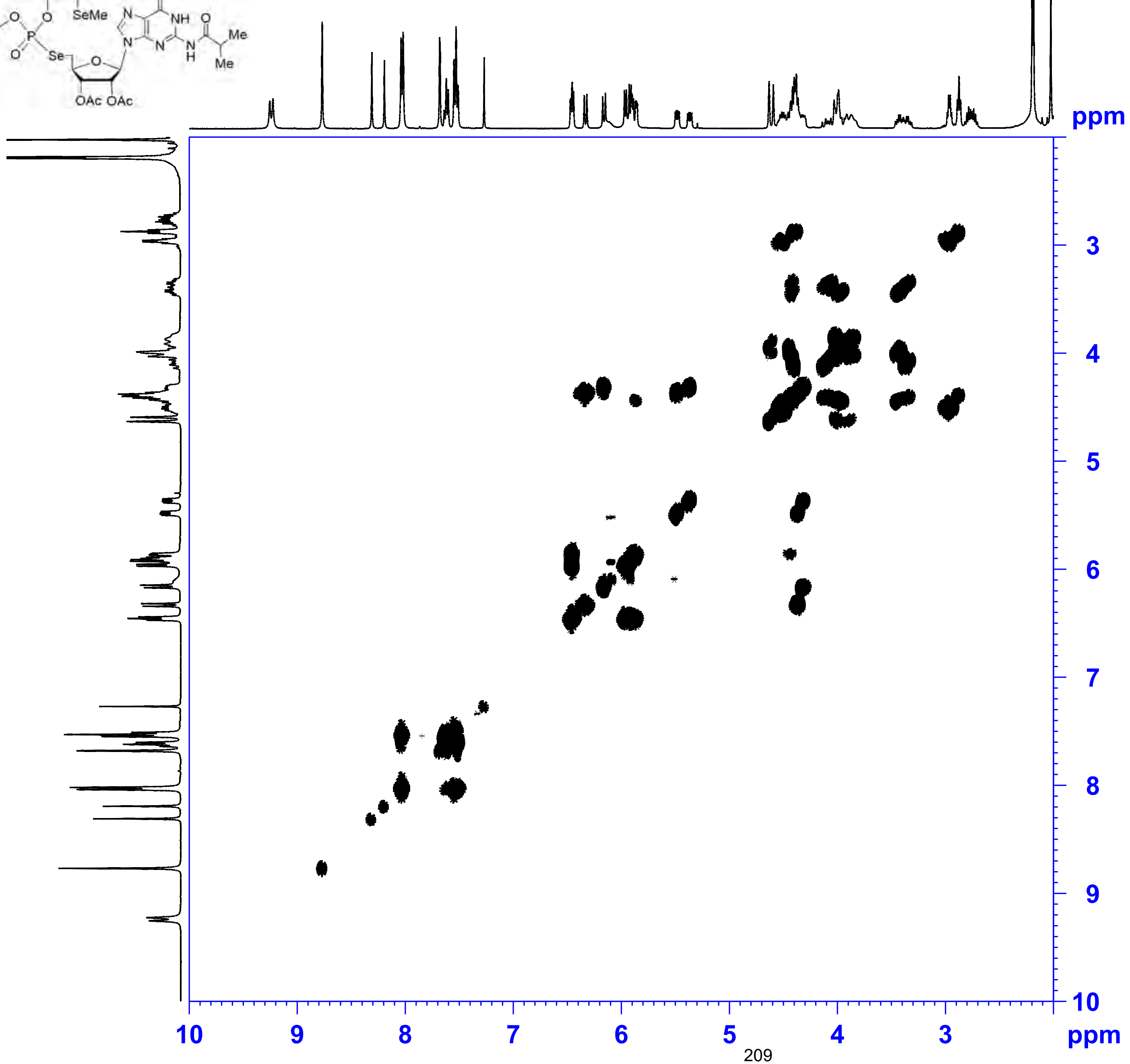


```
Current Data Parameters
NAME      LH-II-64 NEW NMR
EXPNO                   11
PROCNO                   1

F2 - Acquisition Parameters
Date_             20230720
Time                 12.33 h
INSTRUM          AVIII_400
PROBHD    Z108618_0817 (
PULPROG       cosygpmfqf
TD                    2048
SOLVENT              CDCl3
NS                       3
DS                       8
SWH               6172.839 Hz
FIDRES            6.028163 Hz
AQ               0.1658880 sec
RG                    2050
DW                  81.000 usec
DE                    6.50 usec
TE                   295.3 K
D0             0.00000300 sec
D1             2.02607393 sec
D13            0.00000400 sec
D16            0.00020000 sec
IN0            0.00016200 sec
TDav                     1
SFO1           399.9124600 MHz
NUC1                    1H
P1                  500.00 usec
PLW1           31.62299919 W
GPNAM[1]          SINE.100
GPZ1                 16.00 %
GPNAM[2]          SINE.100
GPZ2                 12.00 %
GPNAM[3]          SINE.100
GPZ3                 40.00 %
P16                1000.00 usec

F1 - Acquisition parameters
TD                     256
SFO1              399.9125 MHz
FIDRES           48.225307 Hz
SW                  15.435 ppm
FnMODE                  QF

F2 - Processing parameters
SI                    1024
SF             399.9100018 MHz
WDW                   SINE
SSB       0
LB        0 Hz
GB        0
PC                    1.40

F1 - Processing parameters
SI                    1024
MC2                     QF
SF             399.9100018 MHz
WDW                   SINE
SSB       0
LB        0 Hz
GB        0
```

Expanded region of ^{1}H-^{1}H COSY NMR spectrum of compound 6

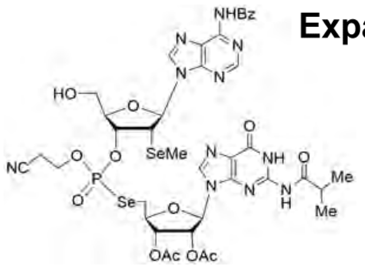

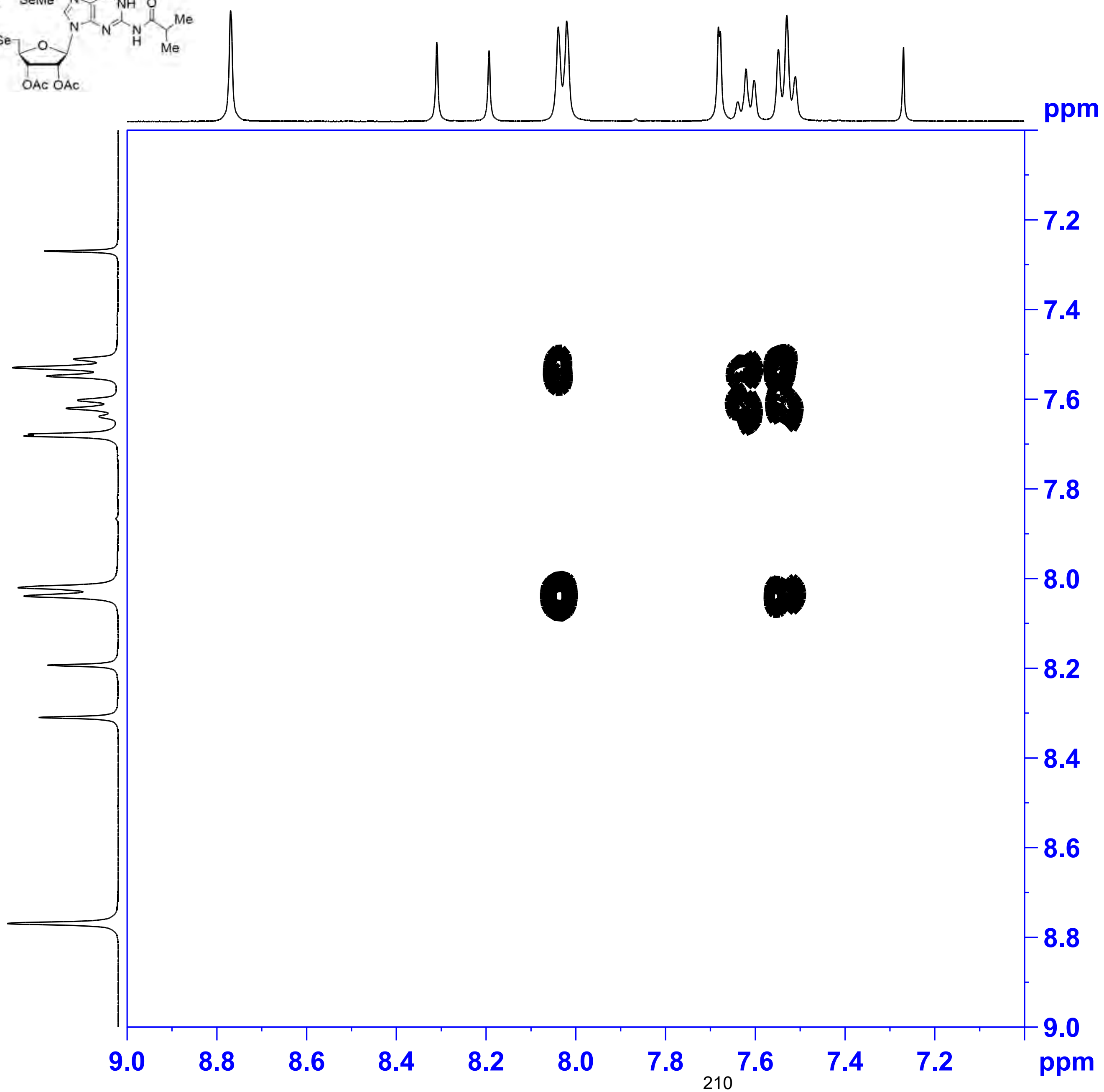

Current Data Parameters
NAME LH-II-64 NEW NMR
EXPNO 11
PROCNO 1

F2 - Acquisition Parameters
Date_ 20230720
Time 12.33 h
INSTRUM AVIII_400
PROBHD Z108618_0817 (
PULPROG cosygpmfqf
TD 2048
SOLVENT CDCl3
NS 3
DS 8
SWH 6172.839 Hz
FIDRES 6.028163 Hz
AQ 0.1658880 sec
RG 2050
DW 81.000 usec
DE 6.50 usec
TE 295.3 K
D0 0.00000300 sec
D1 2.02607393 sec
D13 0.00000400 sec
D16 0.00020000 sec
IN0 0.00016200 sec
TDav 1
SFO1 399.9124600 MHz
NUC1 1H
P1 500.00 usec
PLW1 31.62299919 W
GPNAM[1] SINE.100
GPZ1 16.00 %
GPNAM[2] SINE.100
GPZ2 12.00 %
GPNAM[3] SINE.100
GPZ3 40.00 %
P16 1000.00 usec

F1 - Acquisition parameters
TD 256
SFO1 399.9125 MHz
FIDRES 48.225307 Hz
SW 15.435 ppm
FnMODE QF

F2 - Processing parameters
SI 1024
SF 399.9100018 MHz
WDW SINE
SSB 0
LB 0 Hz
GB 0
PC 1.40

F1 - Processing parameters
SI 1024
MC2 QF
SF 399.9100018 MHz
WDW SINE
SSB 0
LB 0 Hz
GB 0

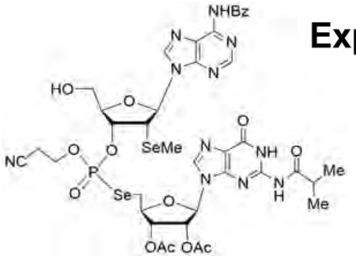

Expanded region of ^{1}H-^{1}H COSY NMR spectrum of compound 6

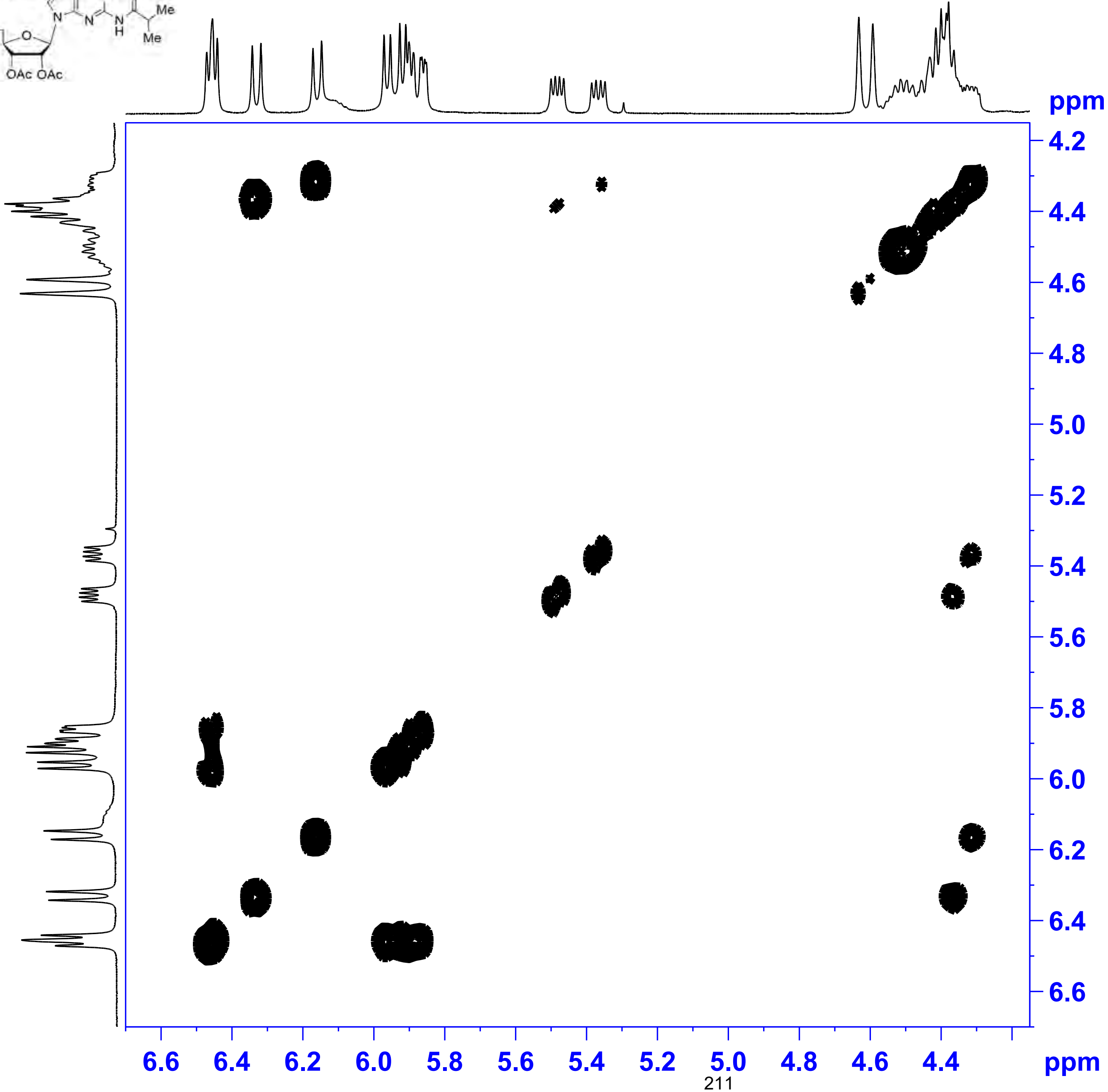


```
Current Data Parameters
NAME      LH-II-64 NEW NMR
EXPNO                   11
PROCNO                   1

F2 - Acquisition Parameters
Date_             20230720
Time                 12.33 h
INSTRUM           AVIII_400
PROBHD    Z108618_0817 (
PULPROG          cosygpmfqf
TD                    2048
SOLVENT              CDCl3
NS                       3
DS                       8
SWH               6172.839 Hz
FIDRES            6.028163 Hz
AQ               0.1658880 sec
RG                    2050
DW                  81.000 usec
DE                    6.50 usec
TE                   295.3 K
D0              0.00000300 sec
D1              2.02607393 sec
D13             0.00000400 sec
D16             0.00020000 sec
IN0             0.00016200 sec
TDav                     1
SFO1           399.9124600 MHz
NUC1                    1H
P1                  500.00 usec
PLW1           31.62299919 W
GPNAM[1]         SINE.100
GPZ1                 16.00 %
GPNAM[2]         SINE.100
GPZ2                 12.00 %
GPNAM[3]         SINE.100
GPZ3                 40.00 %
P16                1000.00 usec

F1 - Acquisition parameters
TD                     256
SFO1              399.9125 MHz
FIDRES           48.225307 Hz
SW                  15.435 ppm
FnMODE                  QF

F2 - Processing parameters
SI                    1024
SF             399.9100018 MHz
WDW                   SINE
SSB       0
LB        0 Hz
GB        0
PC                    1.40

F1 - Processing parameters
SI                    1024
MC2                     QF
SF             399.9100018 MHz
WDW                   SINE
SSB       0
LB        0 Hz
GB        0
```

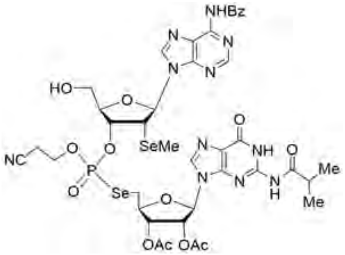

Expanded region of ¹H-¹H COSY NMR spectrum of compound 6

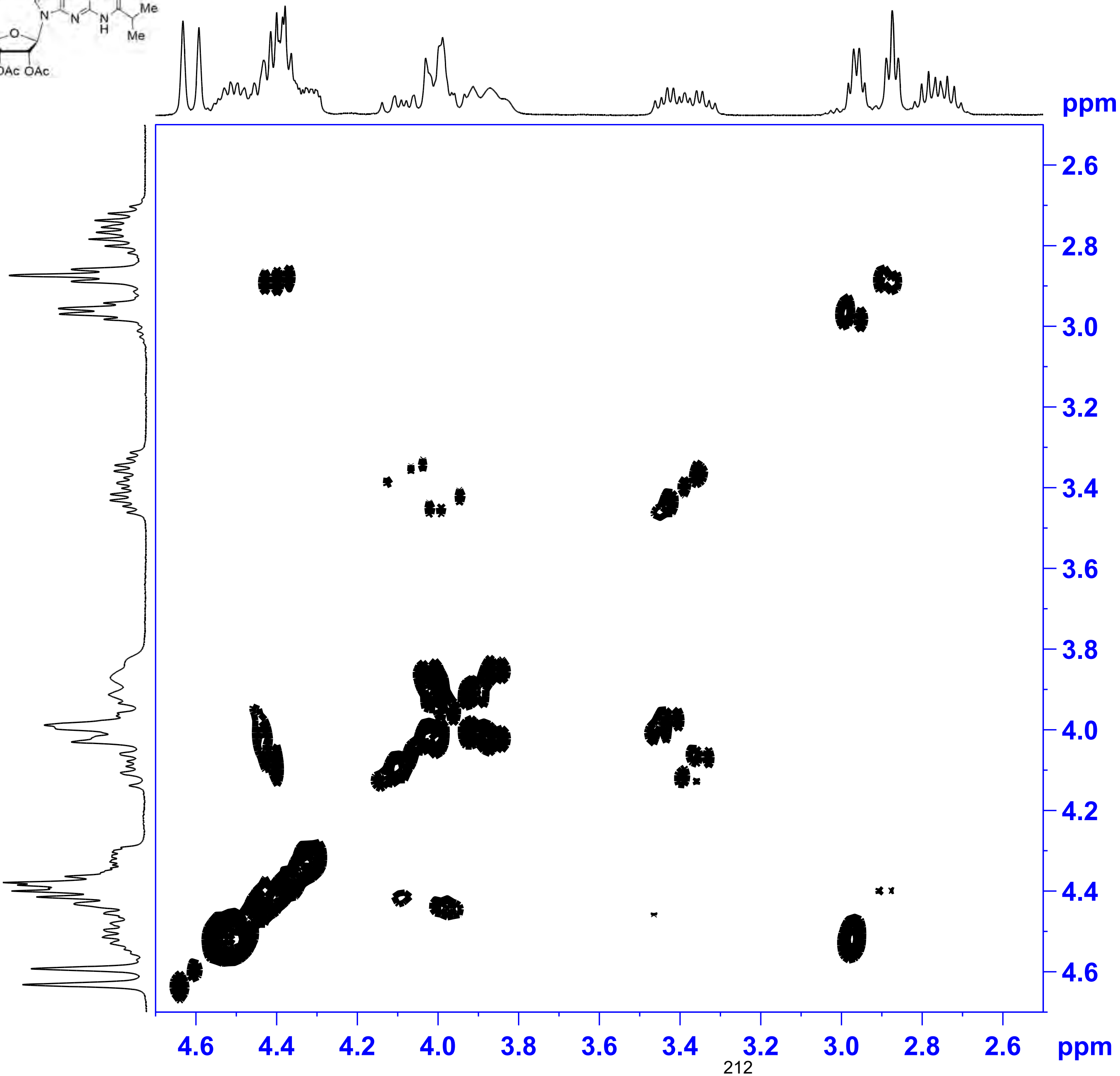


```
Current Data Parameters
NAME      LH-II-64 NEW NMR
EXPNO                   11
PROCNO                   1

F2 - Acquisition Parameters
Date_             20230720
Time                 12.33 h
INSTRUM          AVIII_400
PROBHD    Z108618_0817 (
PULPROG        cosygpmfqf
TD                    2048
SOLVENT              CDCl3
NS                       3
DS                       8
SWH               6172.839 Hz
FIDRES            6.028163 Hz
AQ               0.1658880 sec
RG                    2050
DW                  81.000 usec
DE                    6.50 usec
TE                   295.3 K
D0              0.00000300 sec
D1              2.02607393 sec
D13             0.00000400 sec
D16             0.00020000 sec
IN0             0.00016200 sec
TDav                     1
SFO1           399.9124600 MHz
NUC1                    1H
P1                  500.00 usec
PLW1           31.62299919 W
GPNAM[1]          SINE.100
GPZ1                 16.00 %
GPNAM[2]          SINE.100
GPZ2                 12.00 %
GPNAM[3]          SINE.100
GPZ3                 40.00 %
P16                1000.00 usec

F1 - Acquisition parameters
TD                     256
SFO1              399.9125 MHz
FIDRES           48.225307 Hz
SW                  15.435 ppm
FnMODE                  QF

F2 - Processing parameters
SI                    1024
SF             399.9100018 MHz
WDW                   SINE
SSB      0
LB       0 Hz
GB       0
PC                    1.40

F1 - Processing parameters
SI                    1024
MC2                     QF
SF             399.9100018 MHz
WDW                   SINE
SSB      0
LB       0 Hz
GB       0
```

Expanded region of the ^{1}H-^{13}C HSQC NMR spectrum of compound 6

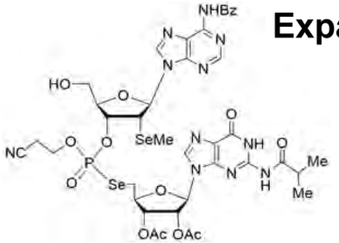

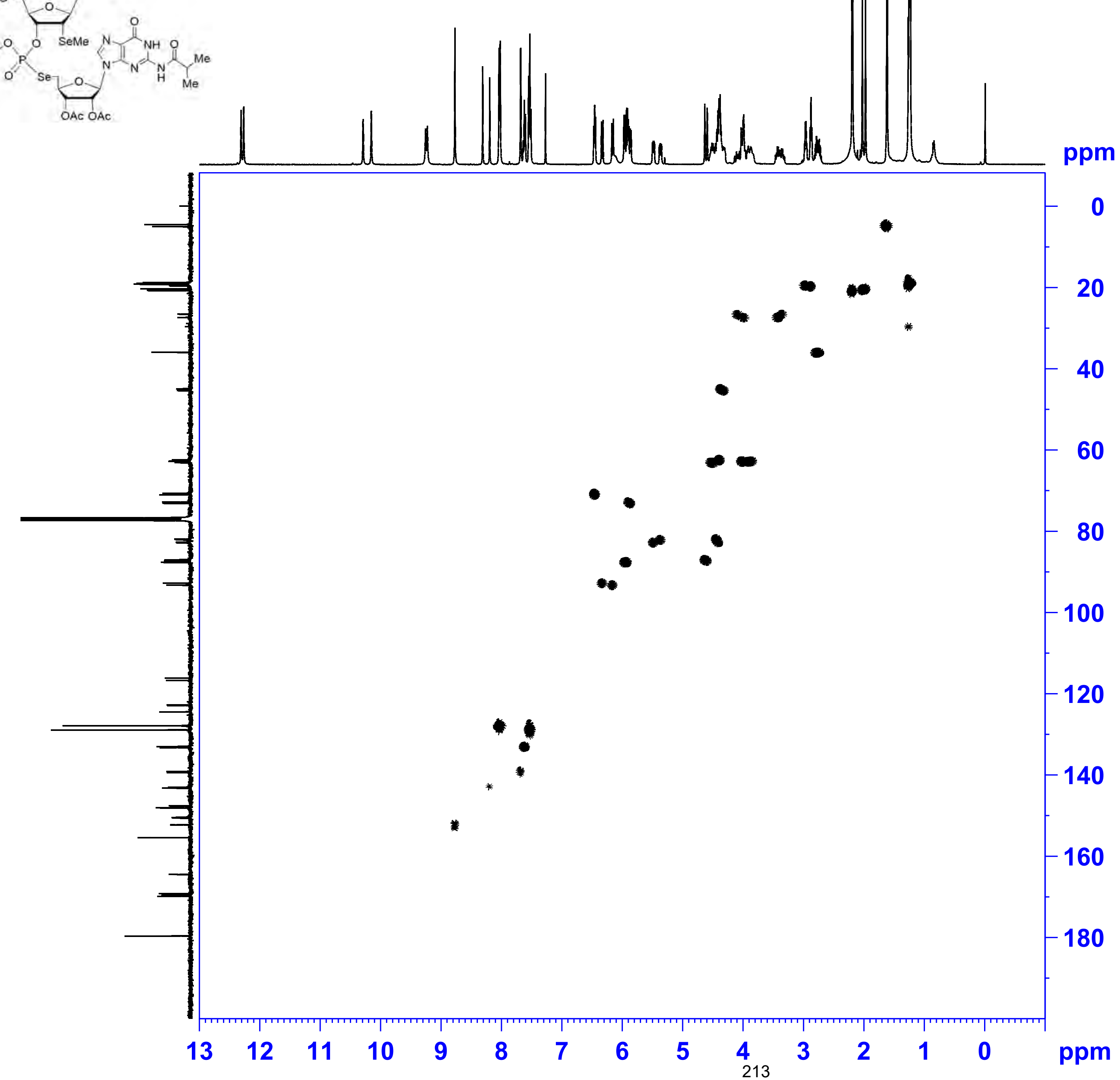


```
Current Data Parameters
NAME      LH-II-64 OLD NMR
EXPNO                   13
PROCNO                   1

F2 - Acquisition Parameters
Date_             20230720
Time                  2.20 h
INSTRUM          AVIII_400
PROBHD    Z108618_0146 (
PULPROG         hsqcedetgp
TD                    1024
SOLVENT              CDCl3
NS                       4
DS                      16
SWH               6250.000 Hz
FIDRES           12.207031 Hz
AQ               0.0819200 sec
RG                    1620
DW                  80.000 usec
DE                    6.50 usec
TE                   300.0 K
CNST2          145.0000000
D0              0.00000300 sec
D1              1.45965397 sec
D4              0.00172414 sec
D11             0.03000000 sec
D16             0.00020000 sec
D21             0.00345000 sec
IN0             0.00003000 sec
TDav                     1
ZGOPTNS
SFO1           400.1124337 MHz
NUC1                    1H
P1                   15.00 usec
P2                   30.00 usec
PLW1           17.29199982 W
SFO2           100.6152434 MHz
NUC2                   13C
CPDPRG[2              garp
P3                    8.70 usec
P4                   17.40 usec
PCPD2                56.50 usec
PLW2           96.68000031 W
PLW12           3.16230011 W
GPNAM[1]       SMSQ10.100
GPZ1                 80.00 %
GPNAM[2]       SMSQ10.100
GPZ2                 20.10 %
P16                1000.00 usec

F1 - Acquisition parameters
TD                     256
SFO1              100.6152 MHz
FIDRES          130.208328 Hz
SW                 165.648 ppm
FnMODE         Echo-Antiecho

F2 - Processing parameters
SI                    1024
SF             400.1100018 MHz
WDW                  QSINE
SSB                      2
LB                       0 Hz
GB                       0
PC                    1.40

F1 - Processing parameters
SI                    1024
MC2          echo-antiecho
SF             100.6077400 MHz
WDW                  QSINE
SSB                      2
LB                       0 Hz
GB                       0
```

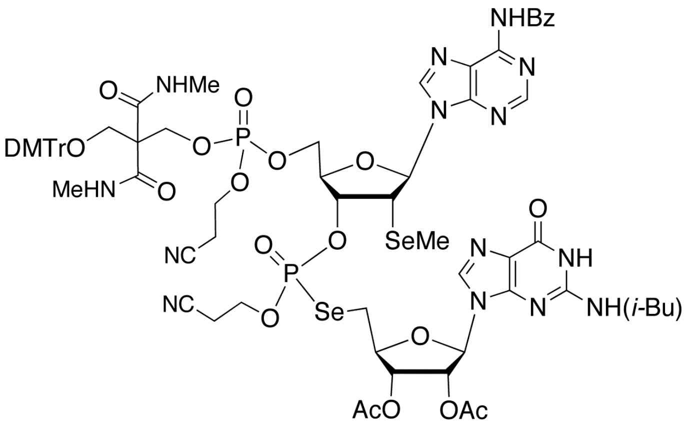

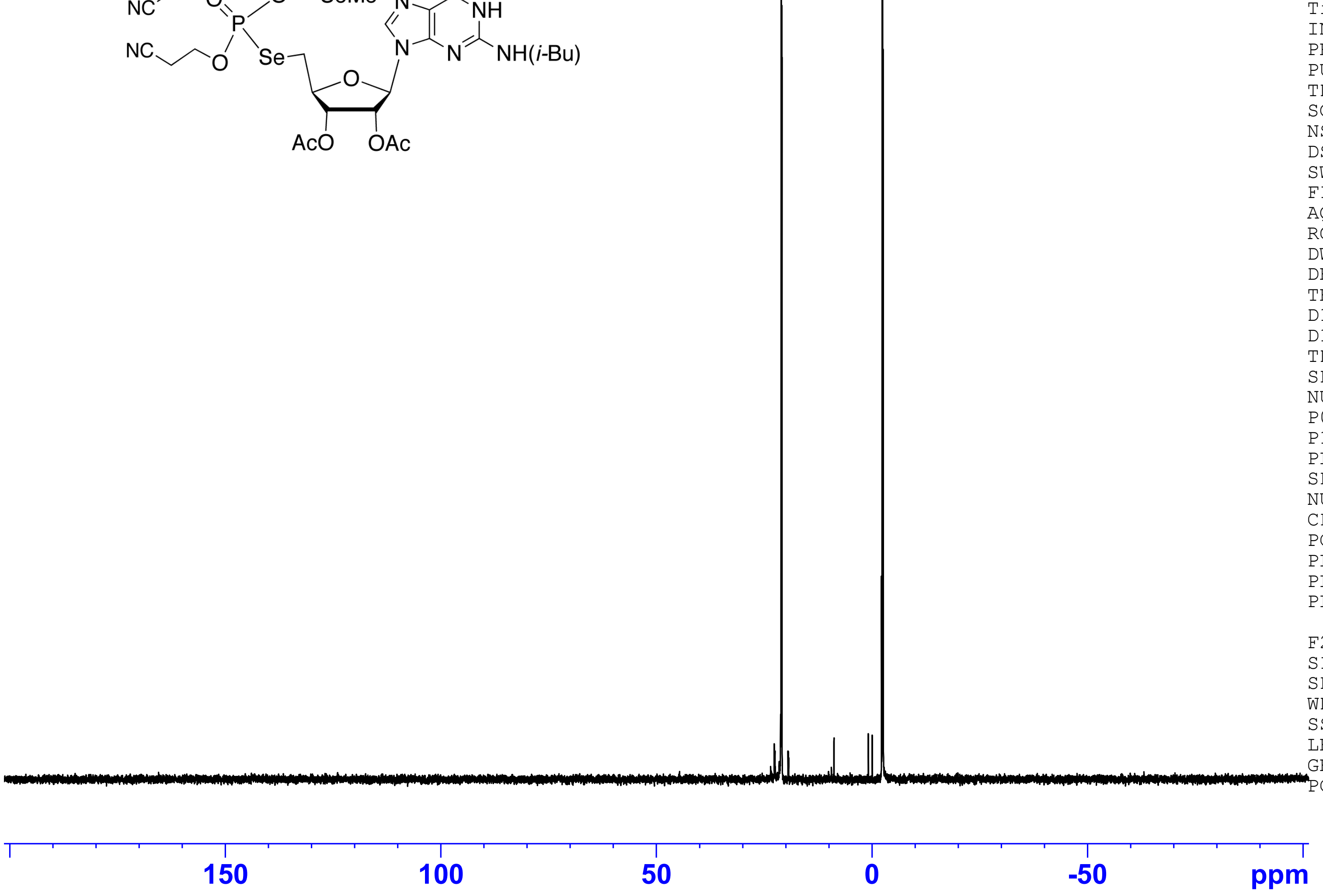

Current Data Parameters
NAME LH-II-80 OLD NMR
EXPNO 10
PROCNO 1

F2 - Acquisition Parameters

Date_	20230907	
Time	19.46	h
INSTRUM	AVIII_400	
PROBHD	Z108618_0146 (	
PULPROG	zgpg30	
TD	65536	
SOLVENT	CDCl3	
NS	1500	
DS	4	
SWH	49019.609	Hz
FIDRES	1.495960	Hz
AQ	0.6684672	sec
RG	2050	
DW	10.200	usec
DE	6.50	usec
TE	300.0	K
D1	2.00000000	sec
D11	0.03000000	sec
TD0	1	
SFO1	161.9755954	MHz
NUC1	31P	
P0	2.80	usec
P1	8.40	usec
PLW1	41.93299866	W
SFO2	400.1116004	MHz
NUC2	1H	
CPDPRG[2	waltz16	
PCPD2	90.00	usec
PLW2	17.29199982	W
PLW12	0.48032999	W
PLW13	0.24160001	W

F2 - Processing parameters

SI	32768	
SF	161.9674970	MHz
WDW	EM	
SSB	0	
LB	2.00	Hz
GB	0	
PC	1.40	

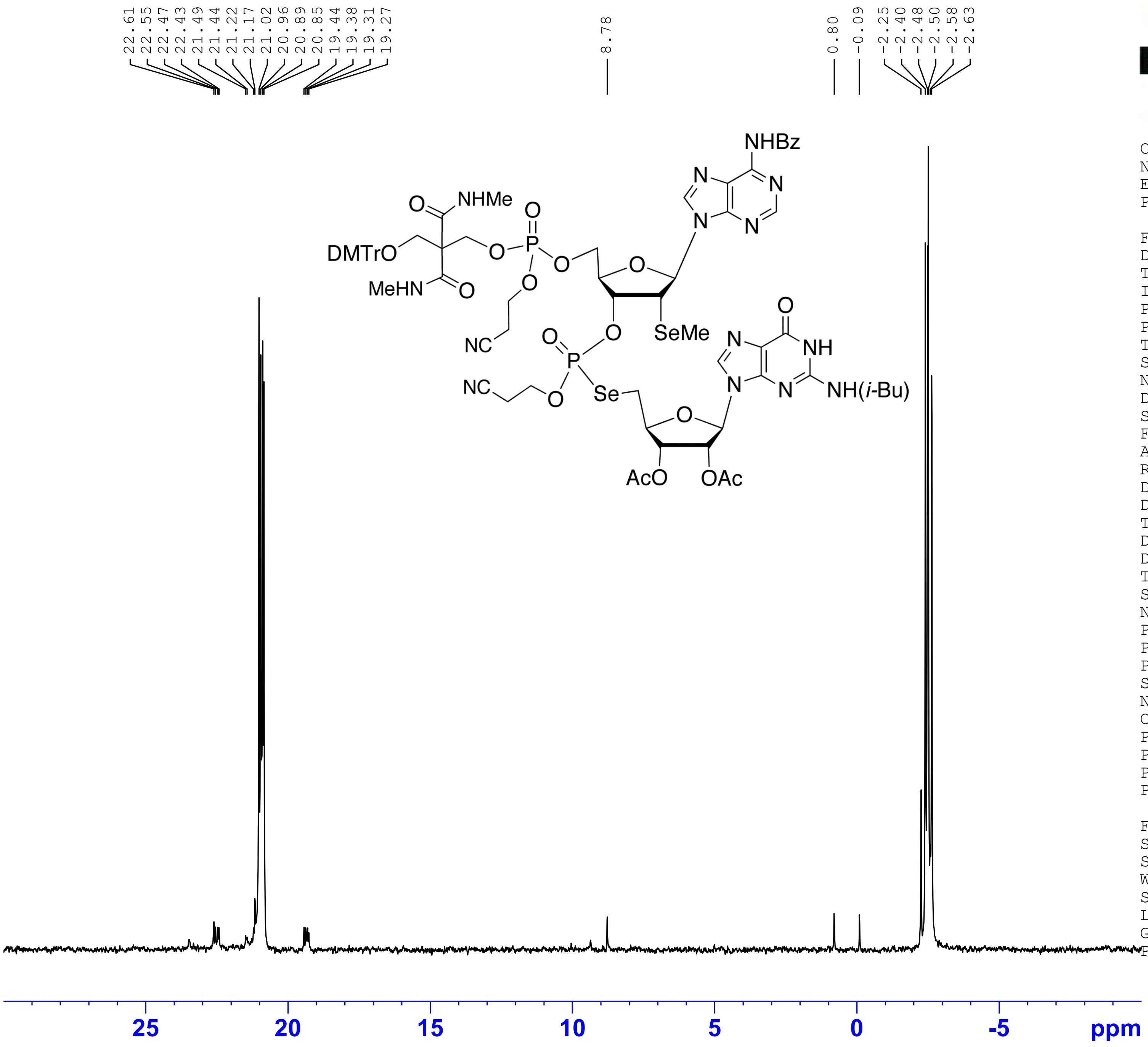

Current Data Parameters
NAME LH-II-80 OLD NMR
EXPNO 10
PROCNO 1

F2 - Acquisition Parameters
Date_ 20230907
Time 19.46 h
INSTRUM AVIII_400
PROBHD Z108618_0146 (
PULPROG zgpg30
TD 65536
SOLVENT CDCl3
NS 1500
DS 4
SWH 49019.609 Hz
FIDRES 1.495960 Hz
AQ 0.6684672 sec
RG 2050
DW 10.200 usec
DE 6.50 usec
TE 300.0 K
D1 2.00000000 sec
D11 0.03000000 sec
TD0 1
SFO1 161.9755954 MHz
NUC1 31P
P0 2.80 usec
P1 8.40 usec
PLW1 41.93299866 W
SFO2 400.1116004 MHz
NUC2 1H
CPDPRG[2 waltz16
PCPD2 90.00 usec
PLW2 17.29199982 W
PLW12 0.48032999 W
PLW13 0.24160001 W

F2 - Processing parameters
SI 32768
SF 161.9674970 MHz
WDW EM
SSB 0
LB 2.00 Hz
GB 0
PC 1.40

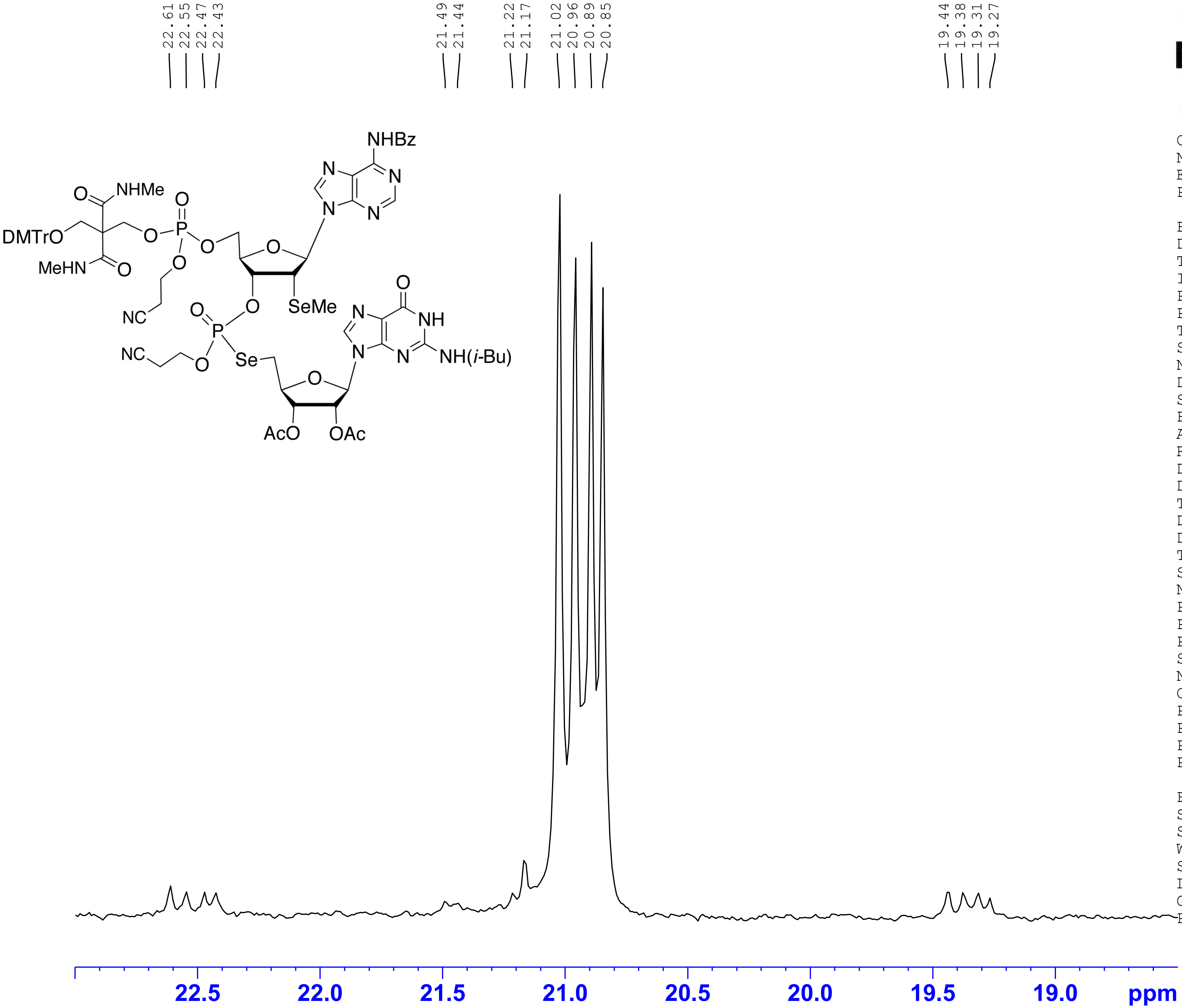


```
Current Data Parameters
NAME       LH-II-80 OLD NMR
EXPNO                    10
PROCNO                    1

F2 - Acquisition Parameters
Date_              20230907
Time                  19.46 h
INSTRUM           AVIII_400
PROBHD     Z108618_0146 (
PULPROG             zgpg30
TD                    65536
SOLVENT               CDCl3
NS                     1500
DS                        4
SWH               49019.609 Hz
FIDRES             1.495960 Hz
AQ                0.6684672 sec
RG                     2050
DW                   10.200 usec
DE                     6.50 usec
TE                    300.0 K
D1               2.00000000 sec
D11              0.03000000 sec
TD0                       1
SFO1            161.9755954 MHz
NUC1                    31P
P0                     2.80 usec
P1                     8.40 usec
PLW1            41.93299866 W
SFO2            400.1116004 MHz
NUC2                     1H
CPDPRG[2            waltz16
PCPD2                 90.00 usec
PLW2            17.29199982 W
PLW12            0.48032999 W
PLW13            0.24160001 W

F2 - Processing parameters
SI                    32768
SF              161.9674970 MHz
WDW                      EM
SSB                       0
LB                     2.00 Hz
GB                        0
PC                     1.40
```

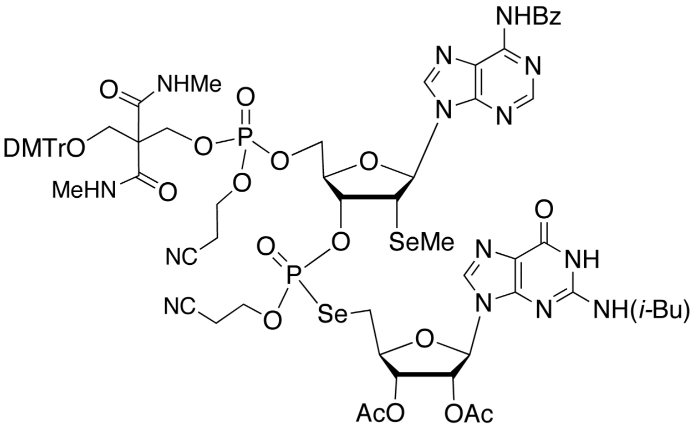

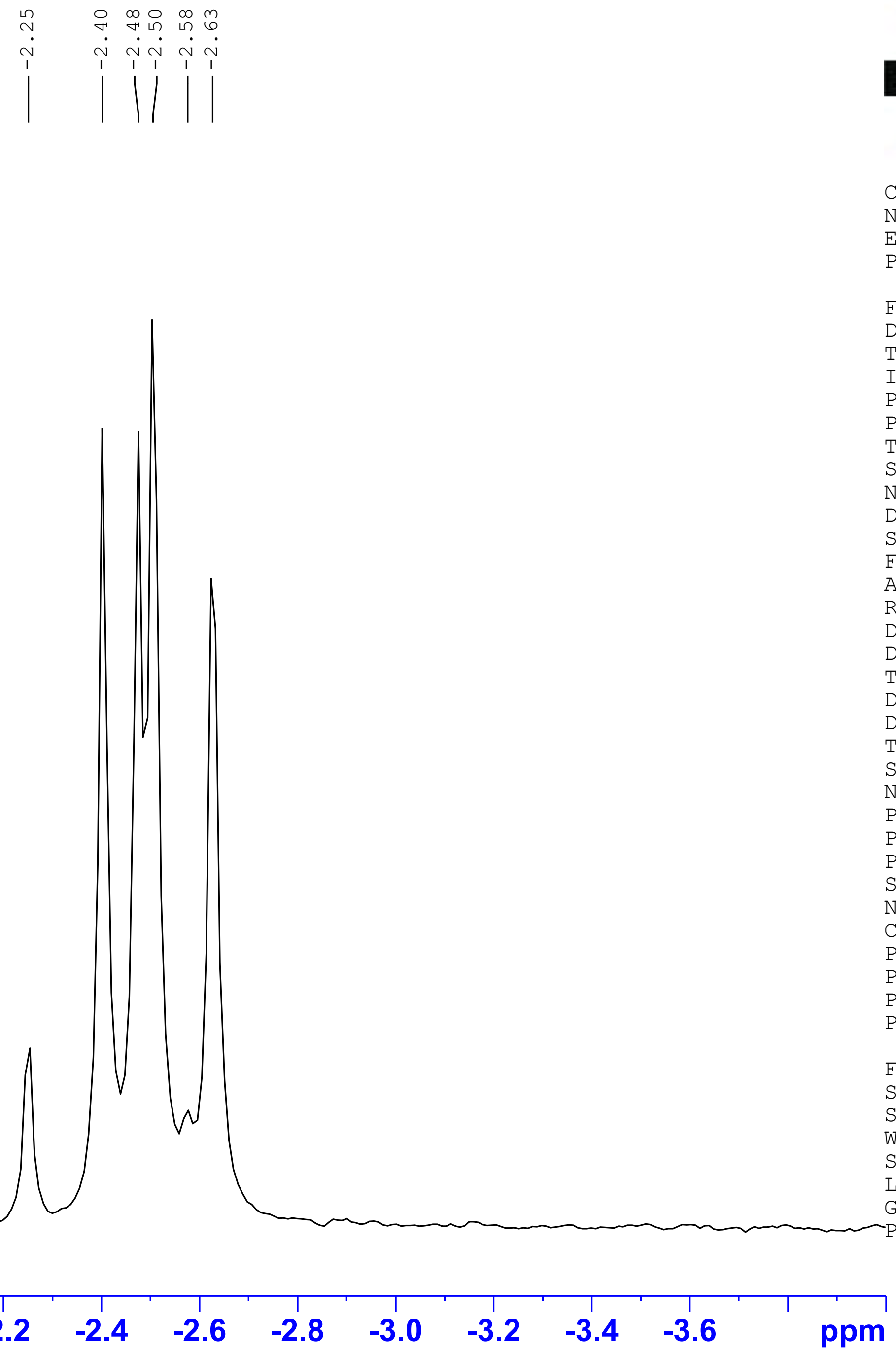

Current Data Parameters
NAME LH-II-80 OLD NMR
EXPNO 10
PROCNO 1

F2 - Acquisition Parameters
Date_ 20230907
Time 19.46 h
INSTRUM AVIII_400
PROBHD Z108618_0146 (
PULPROG zgpg30
TD 65536
SOLVENT CDCl3
NS 1500
DS 4
SWH 49019.609 Hz
FIDRES 1.495960 Hz
AQ 0.6684672 sec
RG 2050
DW 10.200 usec
DE 6.50 usec
TE 300.0 K
D1 2.00000000 sec
D11 0.03000000 sec
TD0 1
SFO1 161.9755954 MHz
NUC1 31P
P0 2.80 usec
P1 8.40 usec
PLW1 41.93299866 W
SFO2 400.1116004 MHz
NUC2 1H
CPDPRG[2 waltz16
PCPD2 90.00 usec
PLW2 17.29199982 W
PLW12 0.48032999 W
PLW13 0.24160001 W

F2 - Processing parameters
SI 32768
SF 161.9674970 MHz
WDW EM
SSB 0
LB 2.00 Hz
GB 0
PC 1.40

^{1}H NMR spectrum of compound 4

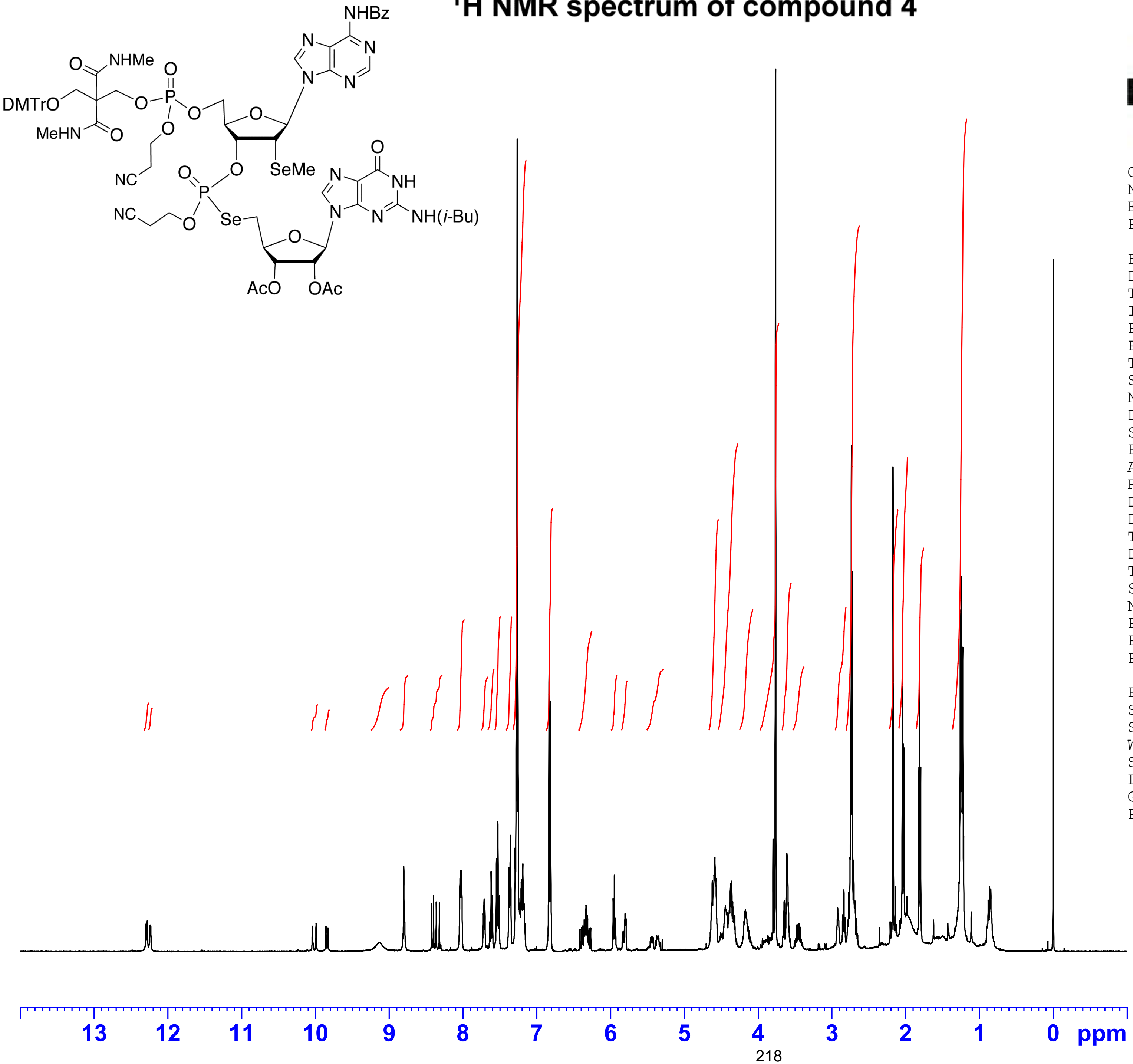


```
Current Data Parameters
NAME      LH-II-80 OLD NMR
EXPNO                   11
PROCNO                   1

F2 - Acquisition Parameters
Date_             20230907
Time                 19.50 h
INSTRUM           AVIII_400
PROBHD   Z108618_0146 (
PULPROG               zg30
TD                   65536
SOLVENT              CDCl3
NS                      32
DS                       2
SWH               8223.685 Hz
FIDRES            0.250967 Hz
AQ               3.9845889 sec
RG                     203
DW                  60.800 usec
DE                   17.42 usec
TE                   300.0 K
D1              1.00000000 sec
TD0                      1
SFO1           400.1124708 MHz
NUC1                    1H
P0                    5.00 usec
P1                   15.00 usec
PLW1           17.29199982 W

F2 - Processing parameters
SI                   32768
SF             400.1100072 MHz
WDW                     EM
SSB                      0
LB                    0.30 Hz
GB                       0
PC                    1.00
```

Expanded region of the ^{1}H NMR spectrum of compound 4

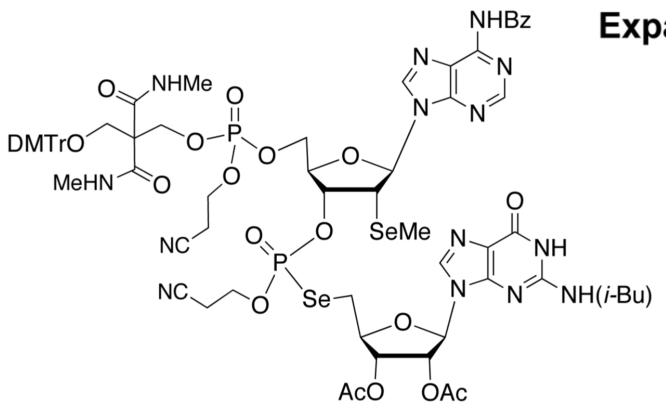

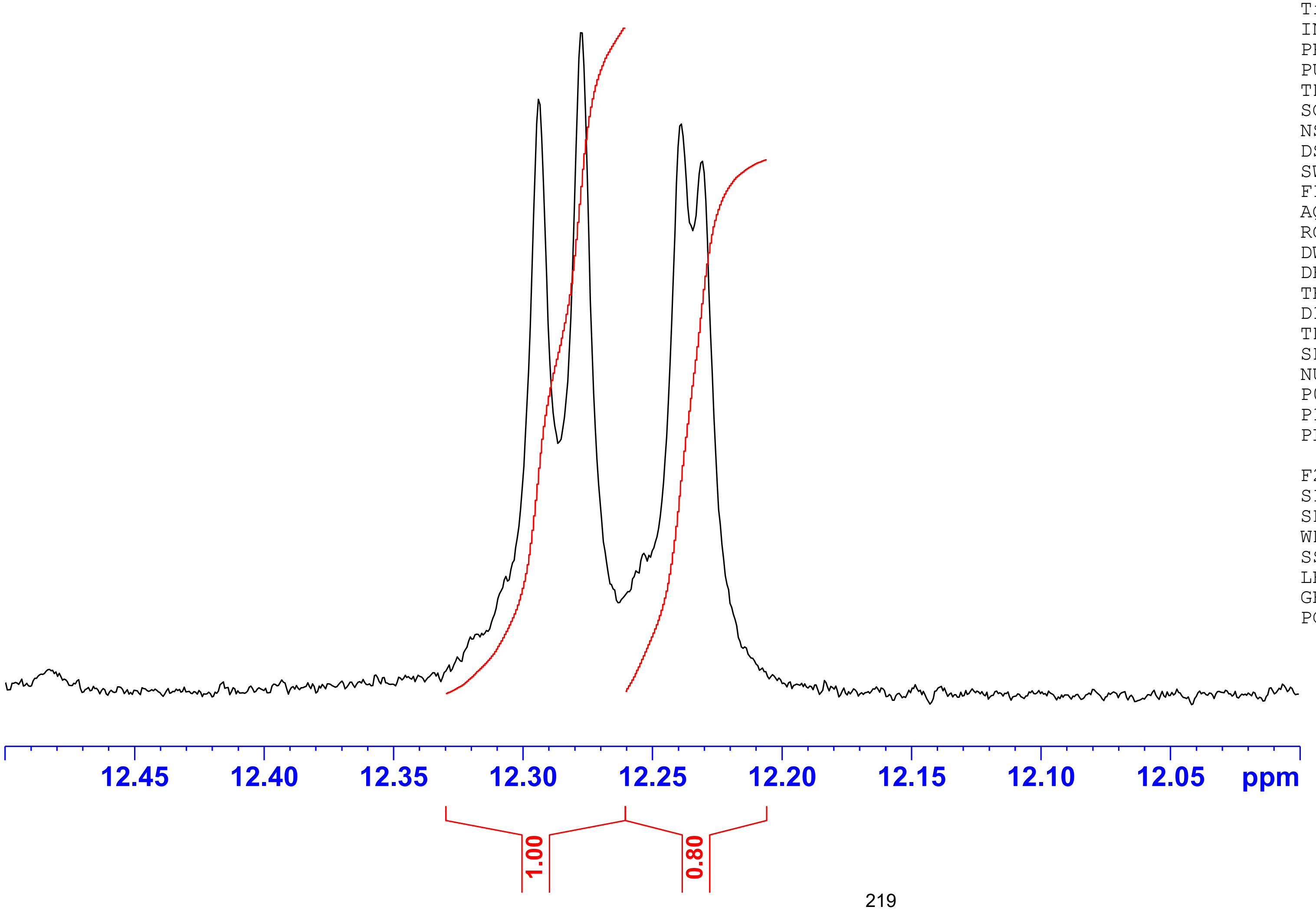


```
Current Data Parameters
NAME        LH-II-80 OLD NMR
EXPNO                     11
PROCNO                     1

F2 - Acquisition Parameters
Date_              20230907
Time                  19.50 h
INSTRUM           AVIII_400
PROBHD      Z108618_0146 (
PULPROG                zg30
TD                    65536
SOLVENT               CDCl3
NS                       32
DS                        2
SWH                8223.685 Hz
FIDRES             0.250967 Hz
AQ                3.9845889 sec
RG                      203
DW                   60.800 usec
DE                    17.42 usec
TE                    300.0 K
D1               1.00000000 sec
TD0                       1
SFO1            400.1124708 MHz
NUC1                     1H
P0                     5.00 usec
P1                    15.00 usec
PLW1            17.29199982 W

F2 - Processing parameters
SI                    32768
SF              400.1100072 MHz
WDW                      EM
SSB                       0
LB                     0.30 Hz
GB                        0
PC                     1.00
```

Expanded region of the ^{1}H NMR spectrum of compound 4

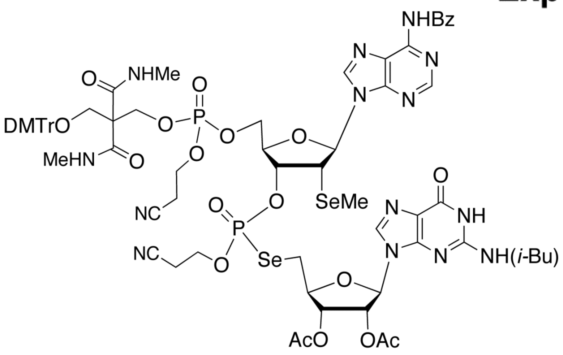

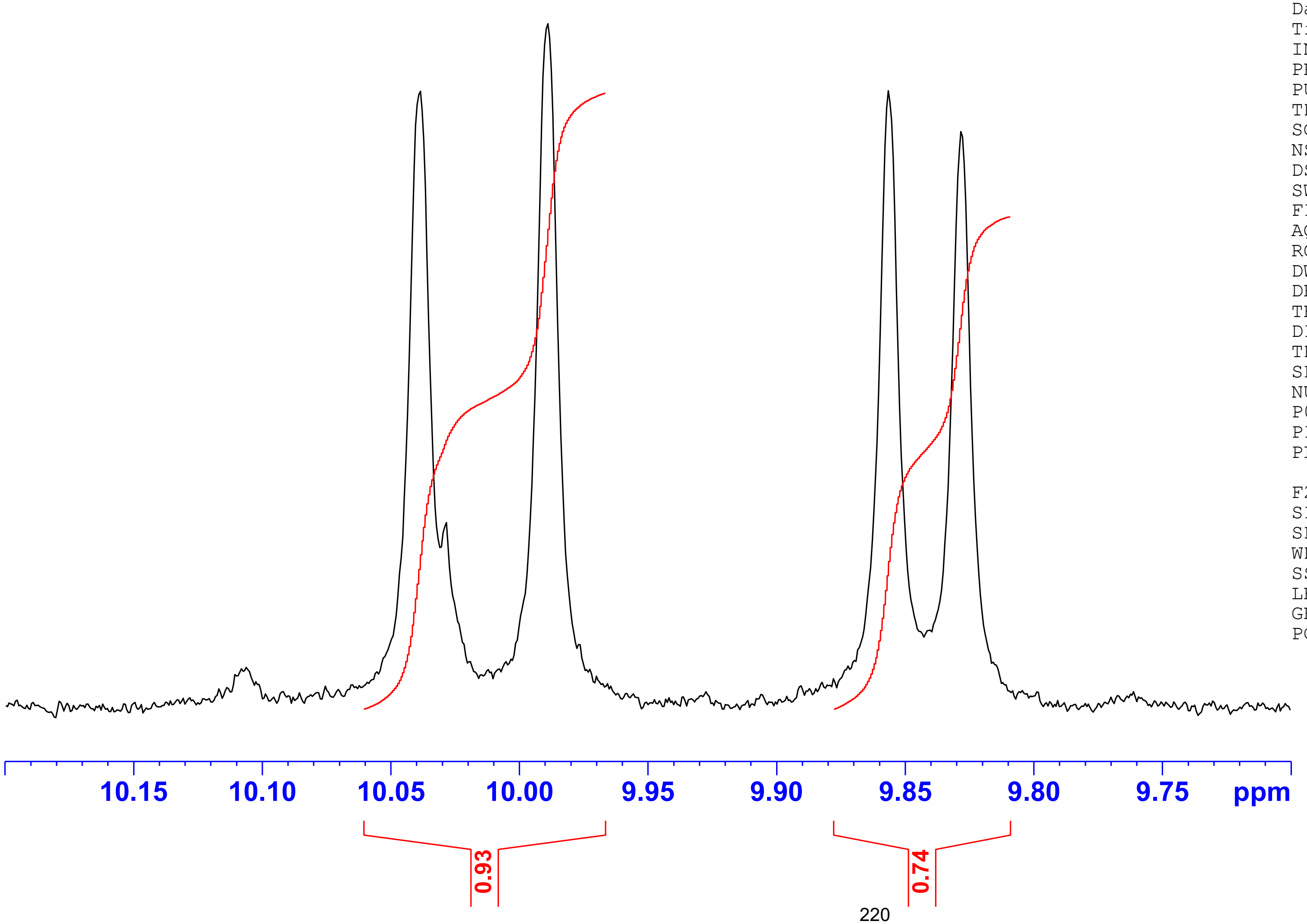


```
Current Data Parameters
NAME       LH-II-80 OLD NMR
EXPNO                    11
PROCNO                    1

F2 - Acquisition Parameters
Date_              20230907
Time                  19.50 h
INSTRUM           AVIII_400
PROBHD    Z108618_0146 (
PULPROG                zg30
TD                    65536
SOLVENT               CDCl3
NS                       32
DS                        2
SWH                8223.685 Hz
FIDRES             0.250967 Hz
AQ                3.9845889 sec
RG                      203
DW                   60.800 usec
DE                    17.42 usec
TE                    300.0 K
D1               1.00000000 sec
TD0                       1
SFO1            400.1124708 MHz
NUC1                     1H
P0                     5.00 usec
P1                    15.00 usec
PLW1            17.29199982 W

F2 - Processing parameters
SI                    32768
SF              400.1100072 MHz
WDW                      EM
SSB                       0
LB                     0.30 Hz
GB                        0
PC                     1.00
```

Expanded region of the ^{1}H NMR spectrum of compound 4

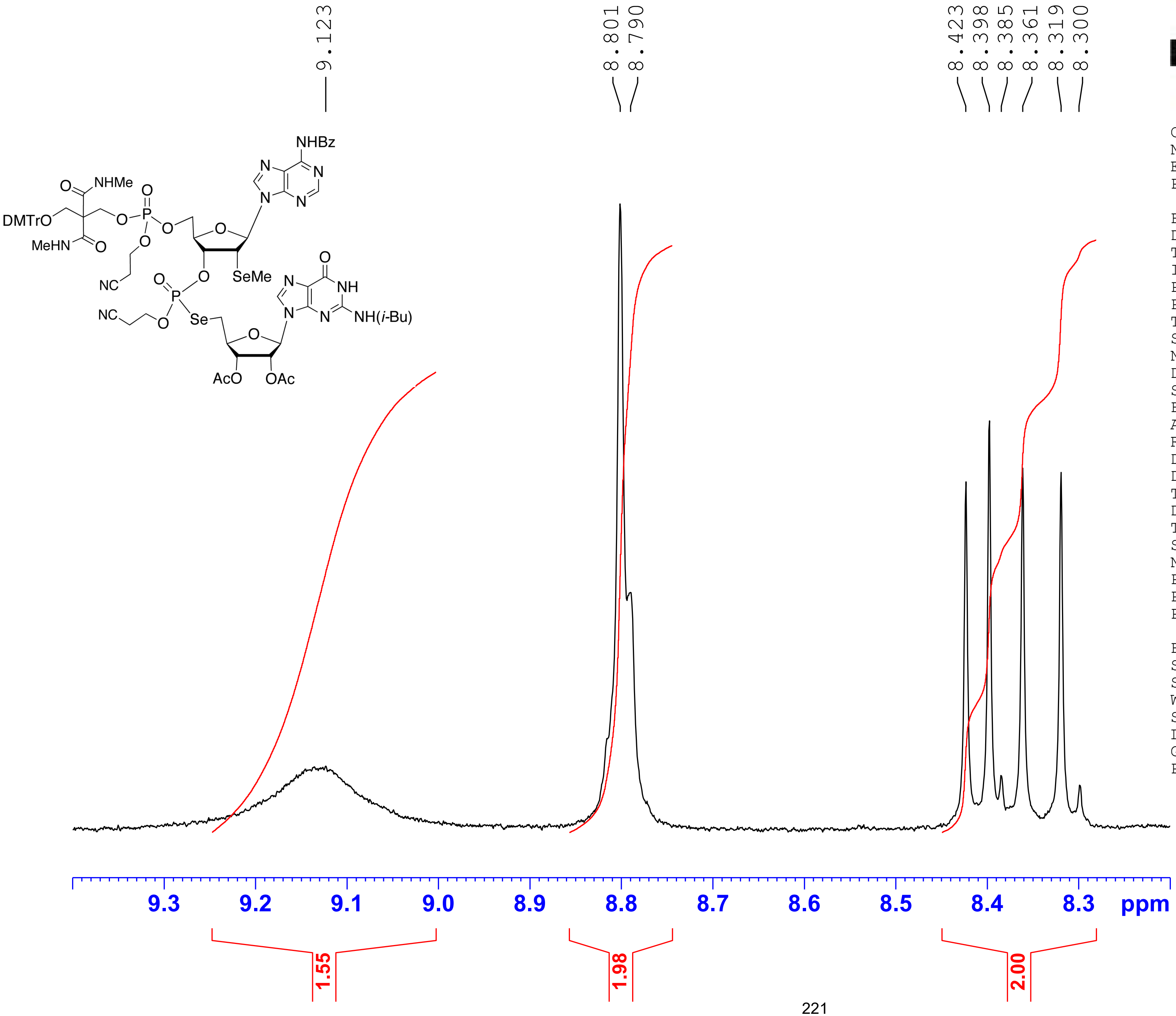


```
Current Data Parameters
NAME        LH-II-80 OLD NMR
EXPNO                     11
PROCNO                     1

F2 - Acquisition Parameters
Date_               20230907
Time                   19.50 h
INSTRUM             AVIII_400
PROBHD      Z108618_0146 (
PULPROG                 zg30
TD                     65536
SOLVENT                CDCl3
NS                        32
DS                         2
SWH                 8223.685 Hz
FIDRES              0.250967 Hz
AQ                 3.9845889 sec
RG                       203
DW                    60.800 usec
DE                     17.42 usec
TE                     300.0 K
D1                1.00000000 sec
TD0                        1
SFO1             400.1124708 MHz
NUC1                      1H
P0                      5.00 usec
P1                     15.00 usec
PLW1             17.29199982 W

F2 - Processing parameters
SI                     32768
SF               400.1100072 MHz
WDW                       EM
SSB                        0
LB                      0.30 Hz
GB                         0
PC                      1.00
```

Expanded region of the ^{1}H NMR spectrum of compound 4

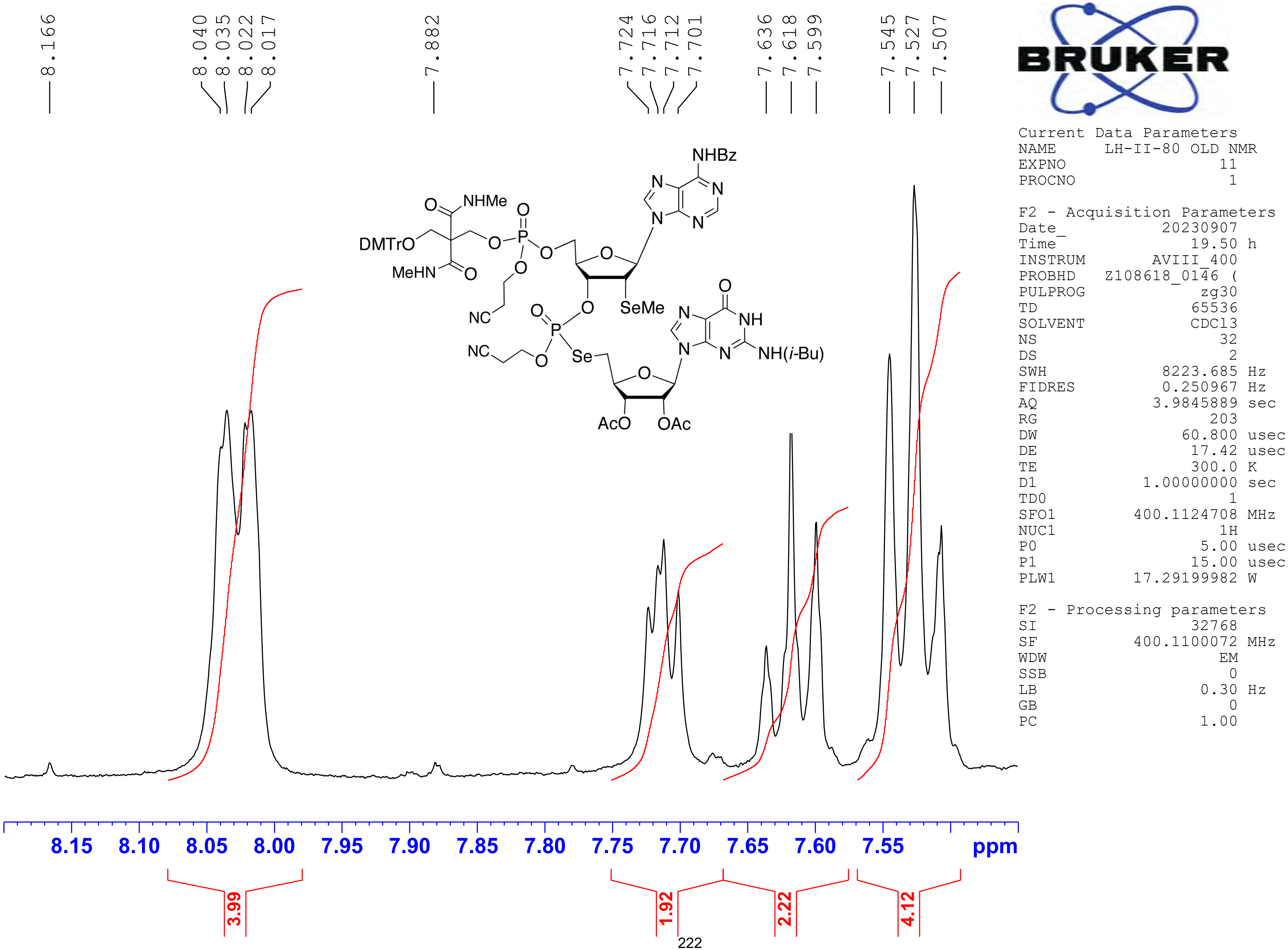

Expanded region of the ^{1}H NMR spectrum of compound 4

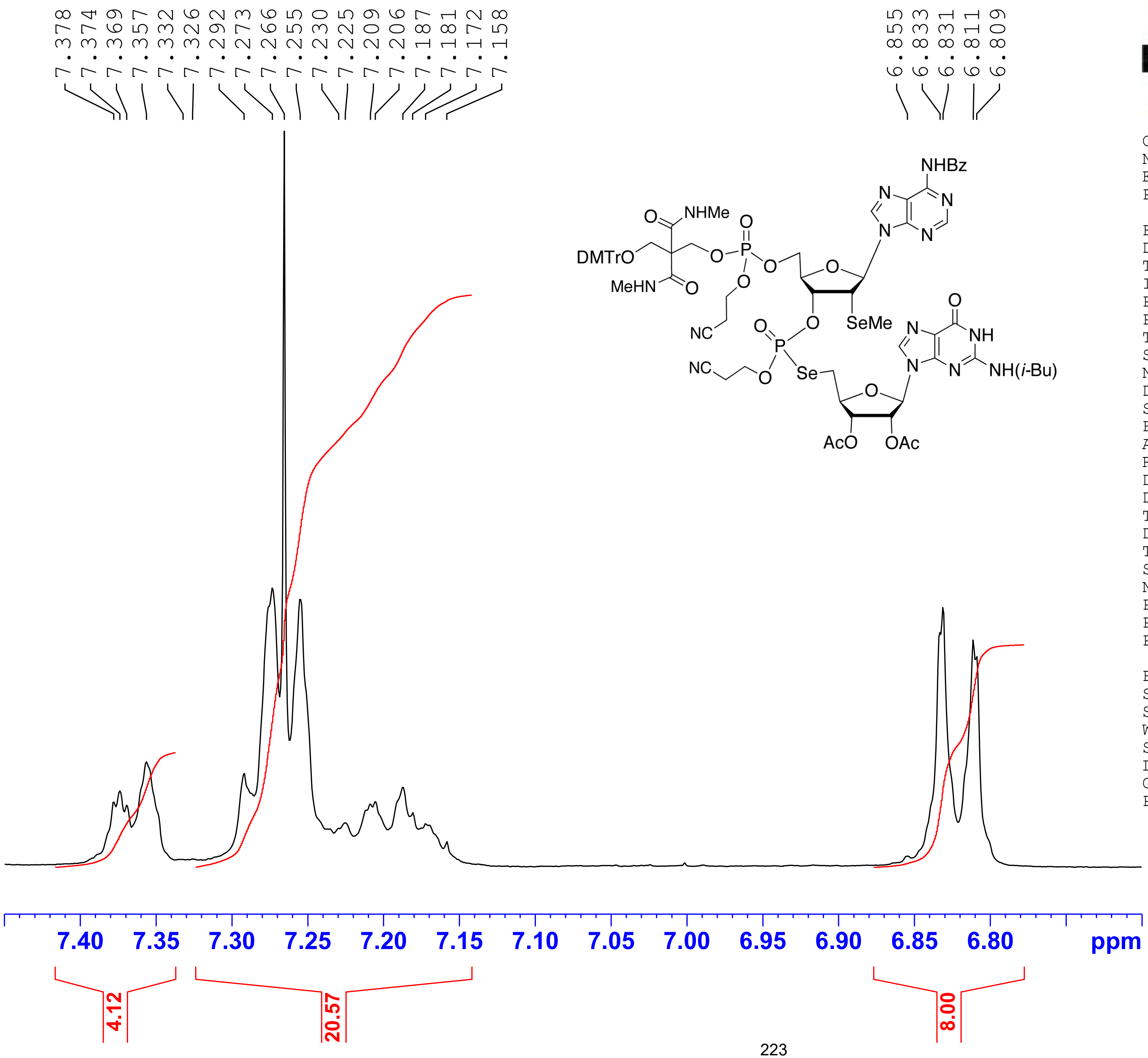

Current Data Parameters		
NAME	LH-II-80 OLD NMR	
EXPNO	11	
PROCNO	1	
F2 - Acquisition Parameters		
Date_	20230907	
Time	19.50	h
INSTRUM	AVIII_400	
PROBHD	Z108618_0146 (	
PULPROG	zg30	
TD	65536	
SOLVENT	CDCl3	
NS	32	
DS	2	
SWH	8223.685	Hz
FIDRES	0.250967	Hz
AQ	3.9845889	sec
RG	203	
DW	60.800	usec
DE	17.42	usec
TE	300.0	K
D1	1.00000000	sec
TD0	1	
SFO1	400.1124708	MHz
NUC1	1H	
P0	5.00	usec
P1	15.00	usec
PLW1	17.29199982	W
F2 - Processing parameters		
SI	32768	
SF	400.1100072	MHz
WDW	EM	
SSB	0	
LB	0.30	Hz
GB	0	
PC	1.00	

Expanded region of the ^{1}H NMR spectrum of compound 4

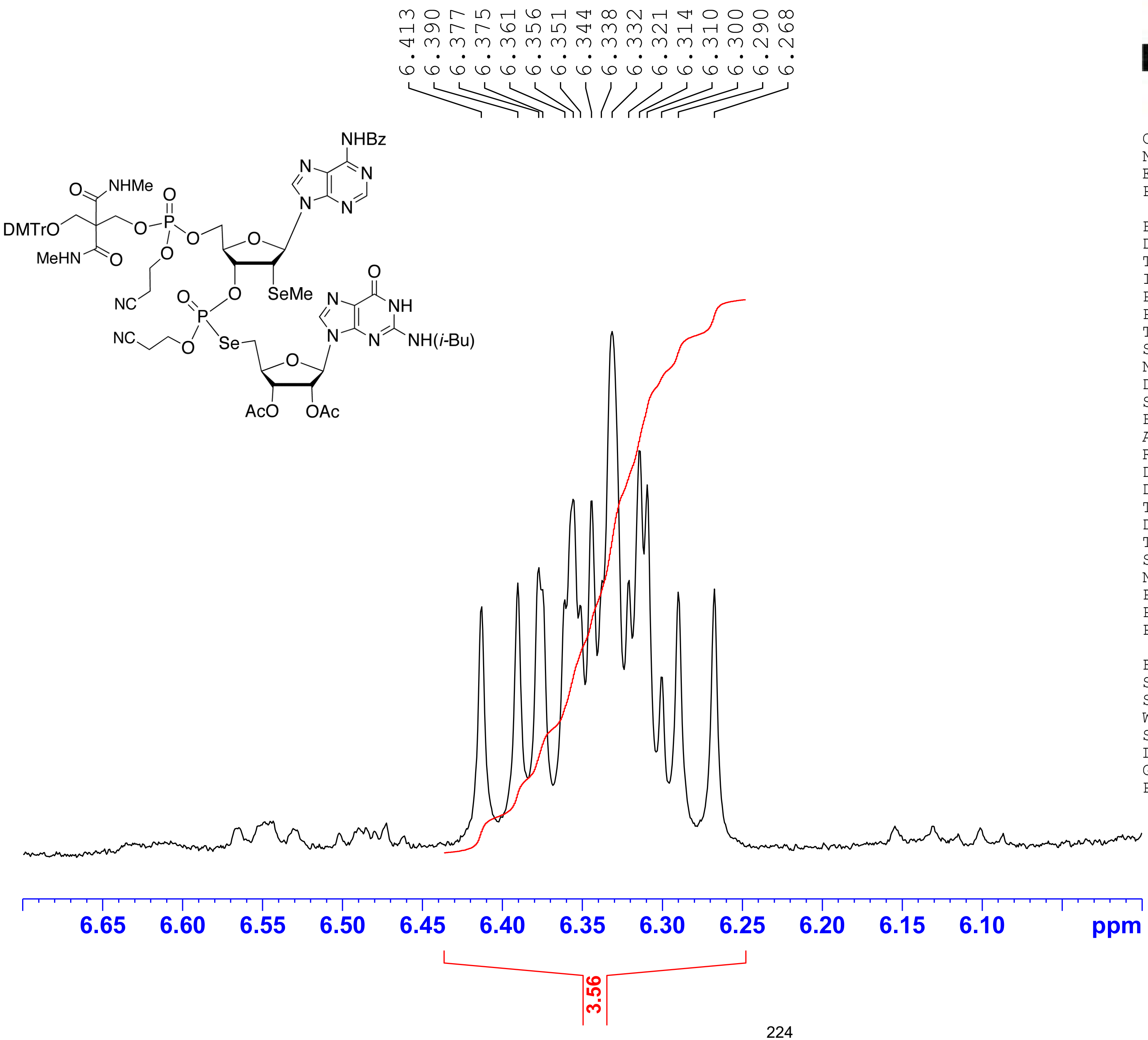

Current Data Parameters
NAME LH-II-80 OLD NMR
EXPNO 11
PROCNO 1

F2 - Acquisition Parameters
Date_ 20230907
Time 19.50 h
INSTRUM AVIII_400
PROBHD Z108618_0146 (
PULPROG zg30
TD 65536
SOLVENT CDCl3
NS 32
DS 2
SWH 8223.685 Hz
FIDRES 0.250967 Hz
AQ 3.9845889 sec
RG 203
DW 60.800 usec
DE 17.42 usec
TE 300.0 K
D1 1.00000000 sec
TD0 1
SFO1 400.1124708 MHz
NUC1 1H
P0 5.00 usec
P1 15.00 usec
PLW1 17.29199982 W

F2 - Processing parameters
SI 32768
SF 400.1100072 MHz
WDW EM
SSB 0
LB 0.30 Hz
GB 0
PC 1.00

Expanded region of the ^{1}H NMR spectrum of compound 4

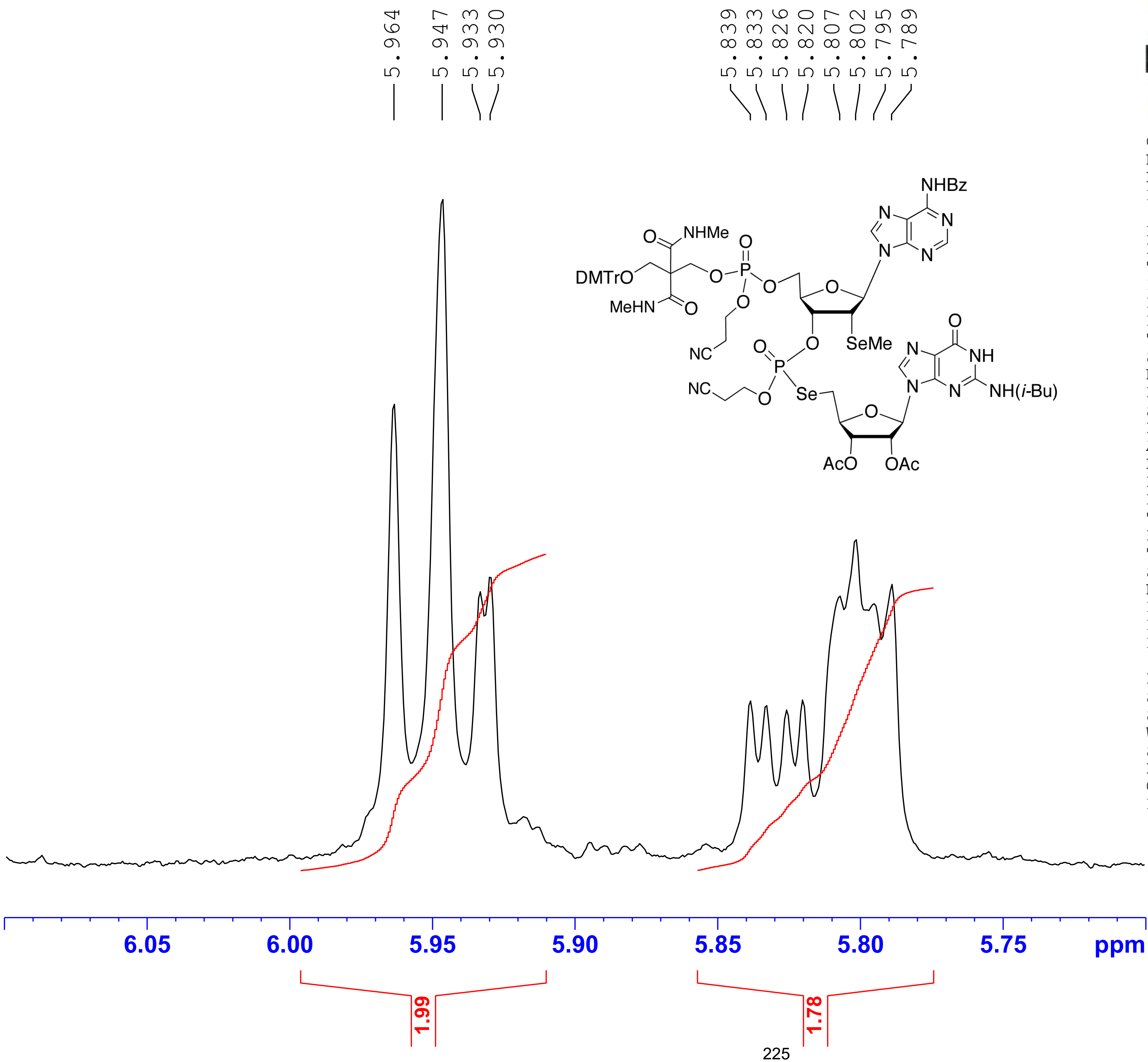

Current Data Parameters
NAME LH-II-80 OLD NMR
EXPNO 11
PROCNO 1

F2 - Acquisition Parameters
Date_ 20230907
Time 19.50 h
INSTRUM AVIII_400
PROBHD Z108618_0146 (
PULPROG zg30
TD 65536
SOLVENT CDCl3
NS 32
DS 2
SWH 8223.685 Hz
FIDRES 0.250967 Hz
AQ 3.9845889 sec
RG 203
DW 60.800 usec
DE 17.42 usec
TE 300.0 K
D1 1.00000000 sec
TD0 1
SFO1 400.1124708 MHz
NUC1 1H
P0 5.00 usec
P1 15.00 usec
PLW1 17.29199982 W

F2 - Processing parameters
SI 32768
SF 400.1100072 MHz
WDW EM
SSB 0
LB 0.30 Hz
GB 0
PC 1.00

Expanded region of the ^{1}H NMR spectrum of compound 4

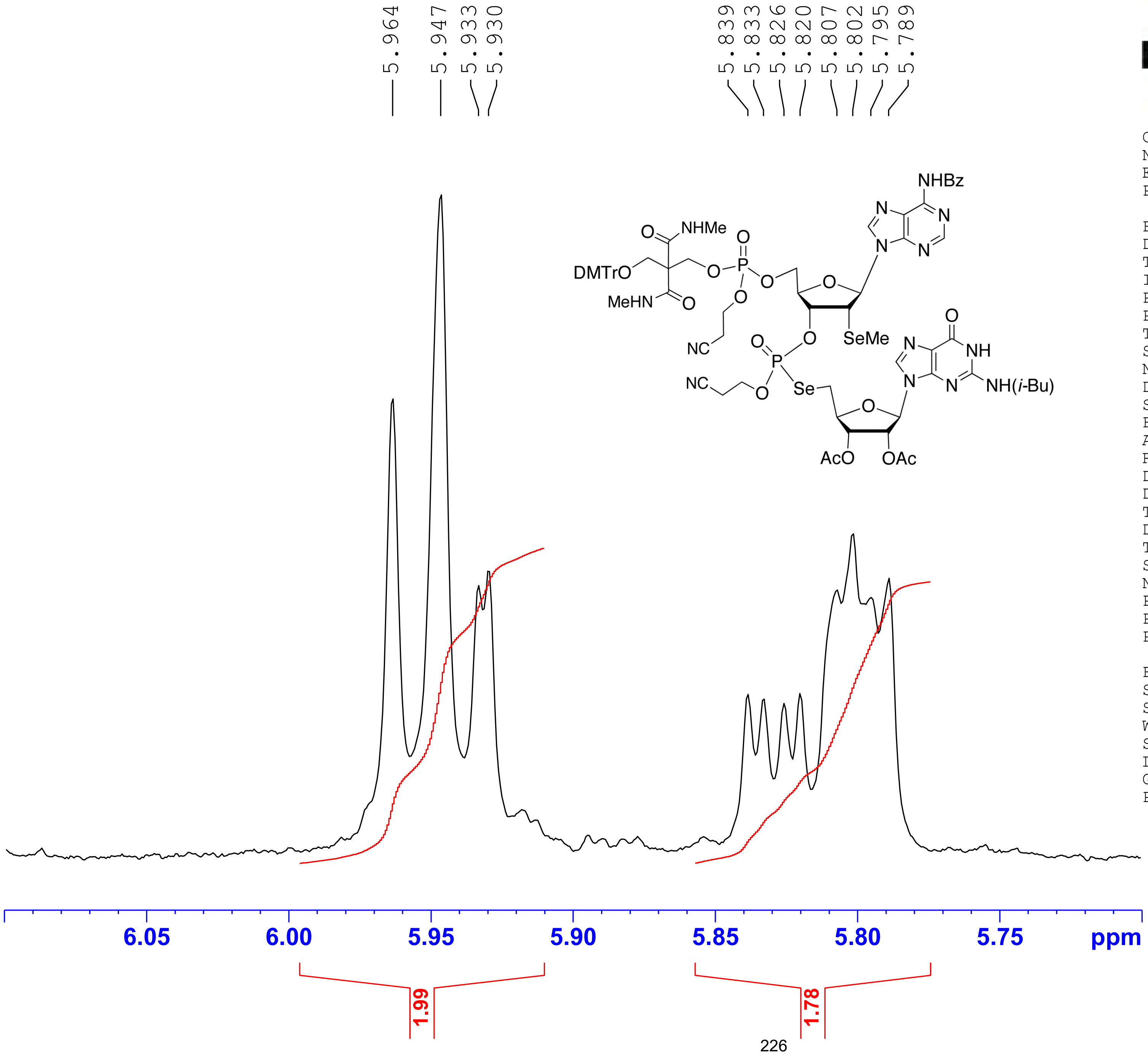


```
Current Data Parameters
NAME      LH-II-80 OLD NMR
EXPNO                   11
PROCNO                   1

F2 - Acquisition Parameters
Date_             20230907
Time                 19.50 h
INSTRUM          AVIII_400
PROBHD   Z108618_0146 (
PULPROG              zg30
TD                   65536
SOLVENT              CDCl3
NS                      32
DS                       2
SWH               8223.685 Hz
FIDRES            0.250967 Hz
AQ               3.9845889 sec
RG                     203
DW                  60.800 usec
DE                   17.42 usec
TE                   300.0 K
D1              1.00000000 sec
TD0                      1
SFO1           400.1124708 MHz
NUC1                    1H
P0                    5.00 usec
P1                   15.00 usec
PLW1           17.29199982 W

F2 - Processing parameters
SI                   32768
SF             400.1100072 MHz
WDW                     EM
SSB                      0
LB                    0.30 Hz
GB                       0
PC                    1.00
```

Expanded region of the ^{1}H NMR spectrum of compound 4

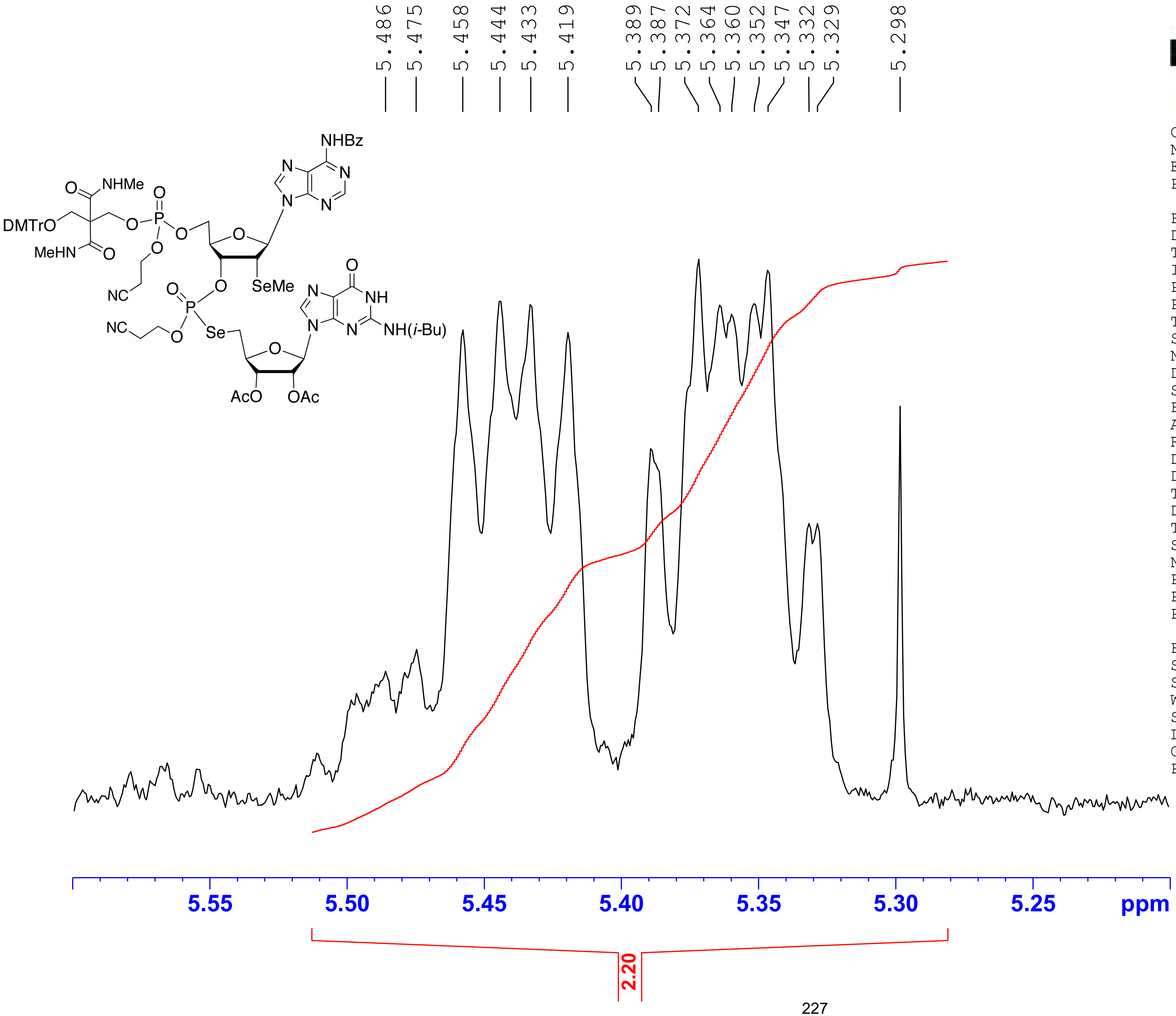

Current Data Parameters
NAME LH-II-80 OLD NMR
EXPNO 11
PROCNO 1

F2 - Acquisition Parameters
Date_ 20230907
Time 19.50 h
INSTRUM AVIII_400
PROBHD Z108618_0146 (
PULPROG zg30
TD 65536
SOLVENT CDCl3
NS 32
DS 2
SWH 8223.685 Hz
FIDRES 0.250967 Hz
AQ 3.9845889 sec
RG 203
DW 60.800 usec
DE 17.42 usec
TE 300.0 K
D1 1.00000000 sec
TD0 1
SFO1 400.1124708 MHz
NUC1 1H
P0 5.00 usec
P1 15.00 usec
PLW1 17.29199982 W

F2 - Processing parameters
SI 32768
SF 400.1100072 MHz
WDW EM
SSB 0
LB 0.30 Hz
GB 0
PC 1.00

Expanded region of the ^{1}H NMR spectrum of compound 4

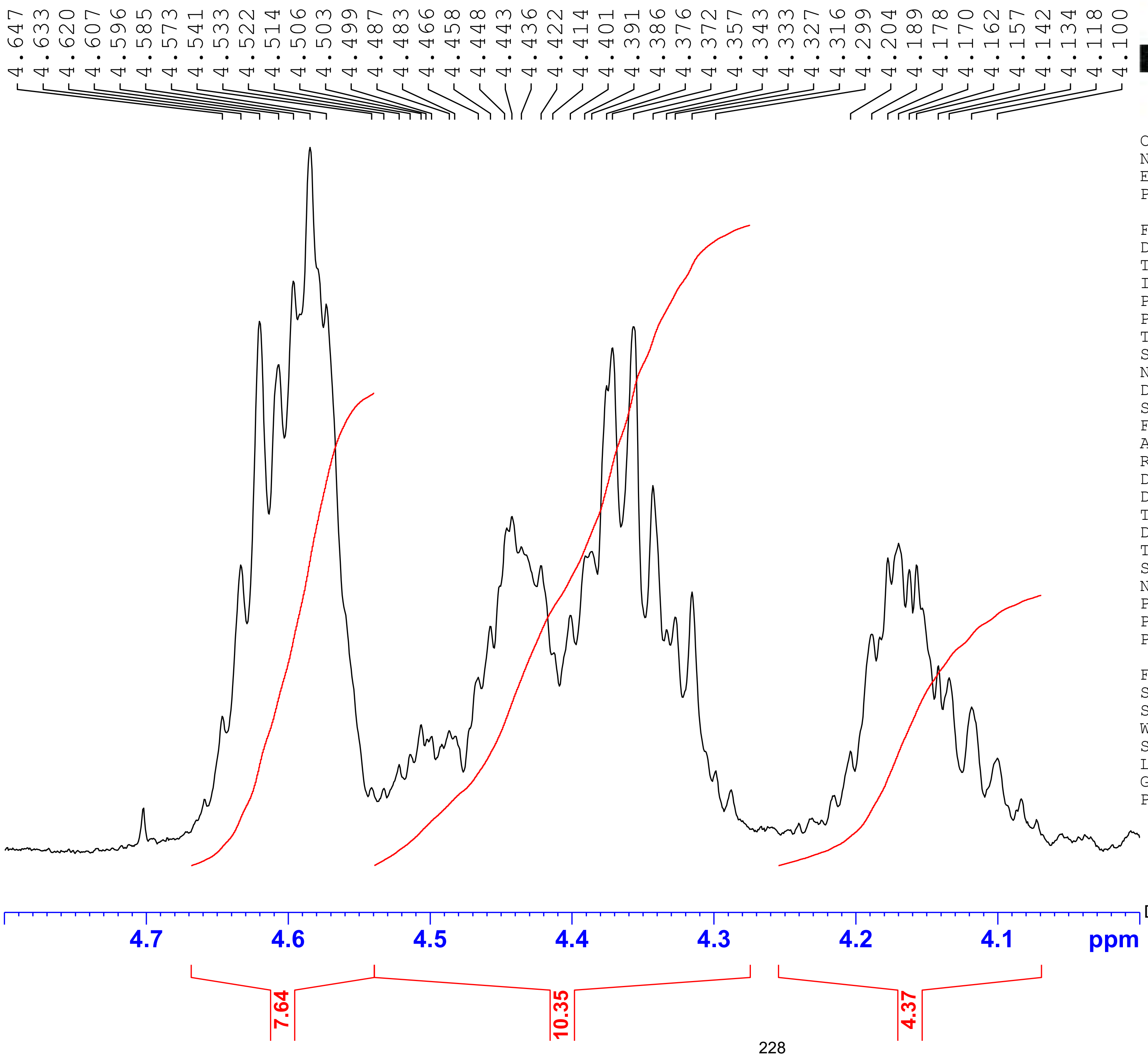


```
Current Data Parameters
NAME      LH-II-80 OLD NMR
EXPNO                   11
PROCNO                   1

F2 - Acquisition Parameters
Date_             20230907
Time                 19.50 h
INSTRUM          AVIII_400
PROBHD    Z108618_0146 (
PULPROG               zg30
TD                   65536
SOLVENT              CDCl3
NS                      32
DS                       2
SWH               8223.685 Hz
FIDRES            0.250967 Hz
AQ               3.9845889 sec
RG                     203
DW                  60.800 usec
DE                   17.42 usec
TE                   300.0 K
D1              1.00000000 sec
TD0                      1
SFO1           400.1124708 MHz
NUC1                    1H
P0                    5.00 usec
P1                   15.00 usec
PLW1           17.29199982 W

F2 - Processing parameters
SI                   32768
SF             400.1100072 MHz
WDW                     EM
SSB                      0
LB                    0.30 Hz
GB                       0
PC                    1.00
```

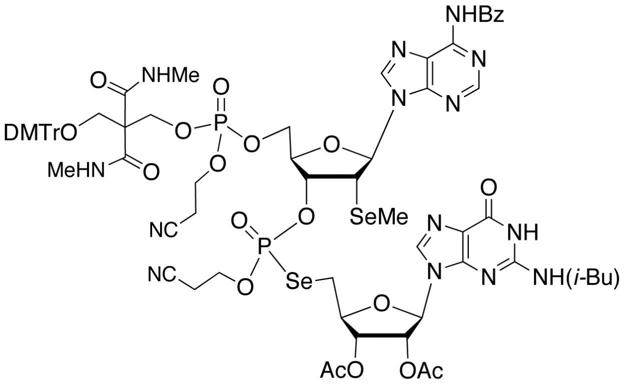

Expanded region of the ^{1}H NMR spectrum of compound 4

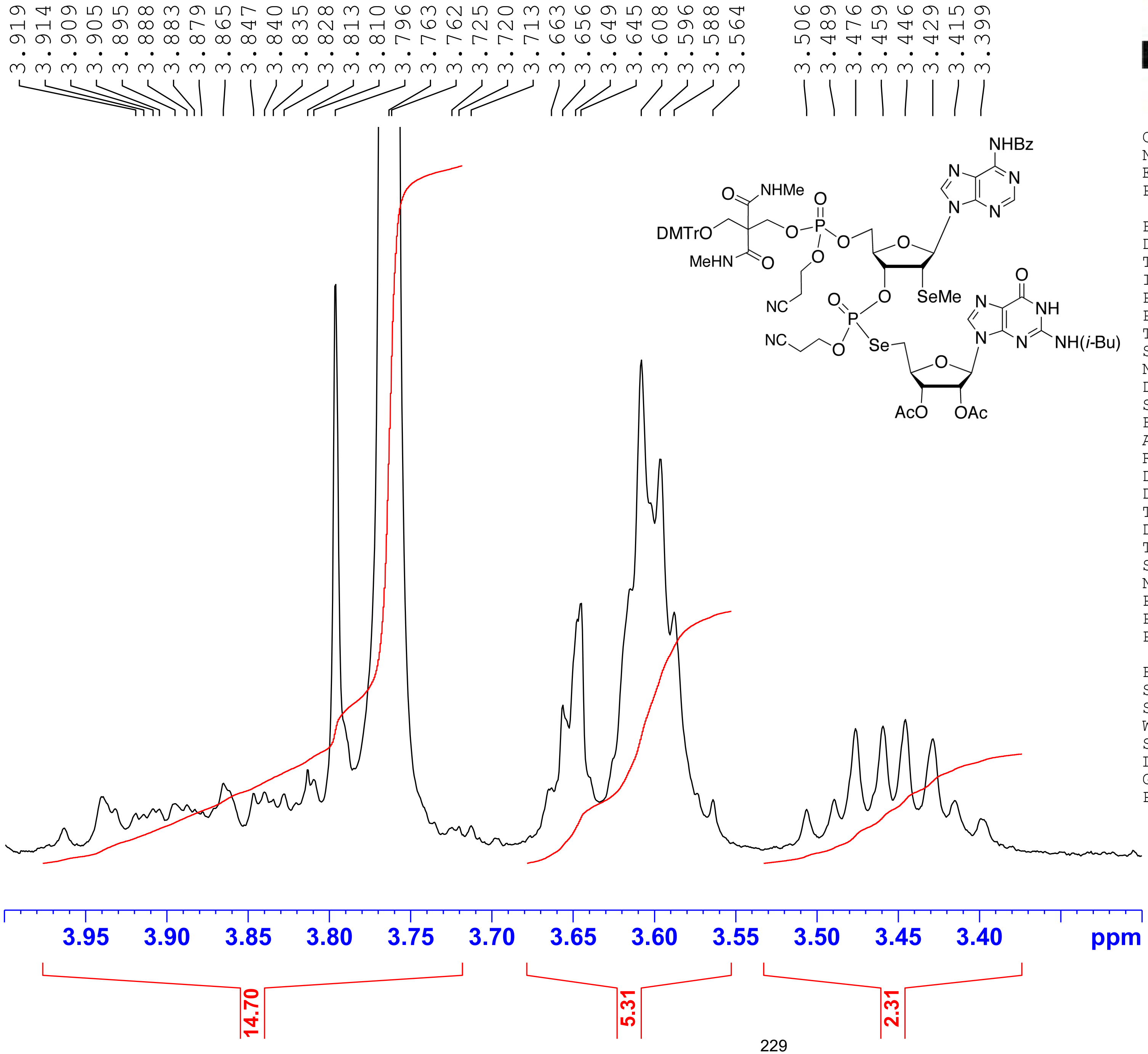


```
Current Data Parameters
NAME      LH-II-80 OLD NMR
EXPNO                   11
PROCNO                   1

F2 - Acquisition Parameters
Date_             20230907
Time                 19.50 h
INSTRUM          AVIII_400
PROBHD   Z108618_0146 (
PULPROG               zg30
TD                   65536
SOLVENT              CDCl3
NS                      32
DS                       2
SWH               8223.685 Hz
FIDRES            0.250967 Hz
AQ               3.9845889 sec
RG                     203
DW                  60.800 usec
DE                   17.42 usec
TE                   300.0 K
D1              1.00000000 sec
TD0                      1
SFO1           400.1124708 MHz
NUC1                    1H
P0                    5.00 usec
P1                   15.00 usec
PLW1           17.29199982 W

F2 - Processing parameters
SI                   32768
SF             400.1100072 MHz
WDW                     EM
SSB                      0
LB                    0.30 Hz
GB                       0
PC                    1.00
```

Expanded region of the ^{1}H NMR spectrum of compound 4

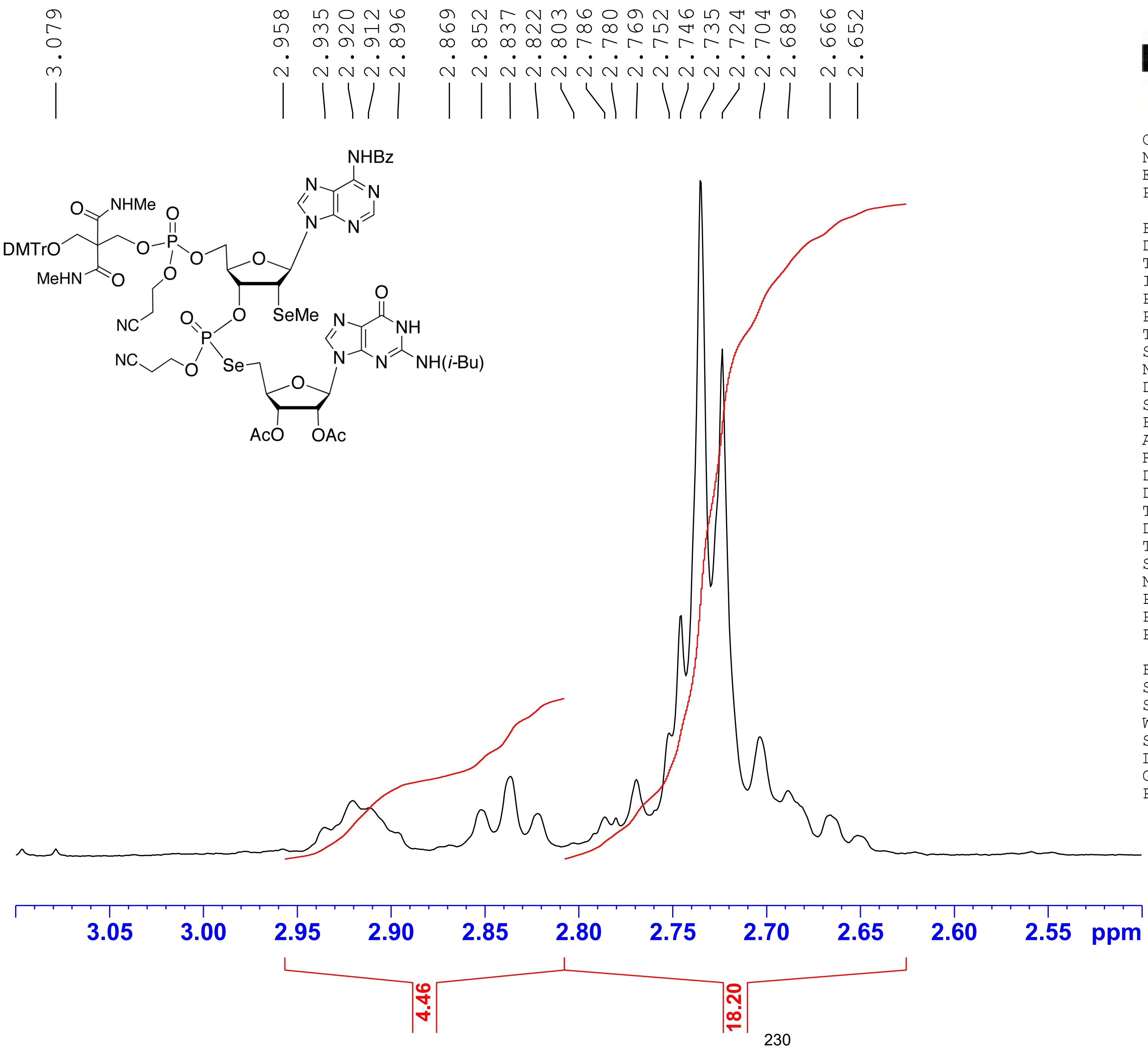

Current Data Parameters
NAME LH-II-80 OLD NMR
EXPNO 11
PROCNO 1

F2 - Acquisition Parameters
Date_ 20230907
Time 19.50 h
INSTRUM AVIII_400
PROBHD Z108618_0146 (
PULPROG zg30
TD 65536
SOLVENT CDCl3
NS 32
DS 2
SWH 8223.685 Hz
FIDRES 0.250967 Hz
AQ 3.9845889 sec
RG 203
DW 60.800 usec
DE 17.42 usec
TE 300.0 K
D1 1.00000000 sec
TD0 1
SFO1 400.1124708 MHz
NUC1 1H
P0 5.00 usec
P1 15.00 usec
PLW1 17.29199982 W

F2 - Processing parameters
SI 32768
SF 400.1100072 MHz
WDW EM
SSB 0
LB 0.30 Hz
GB 0
PC 1.00

Expanded region of the ^{1}H NMR spectrum of compound 4

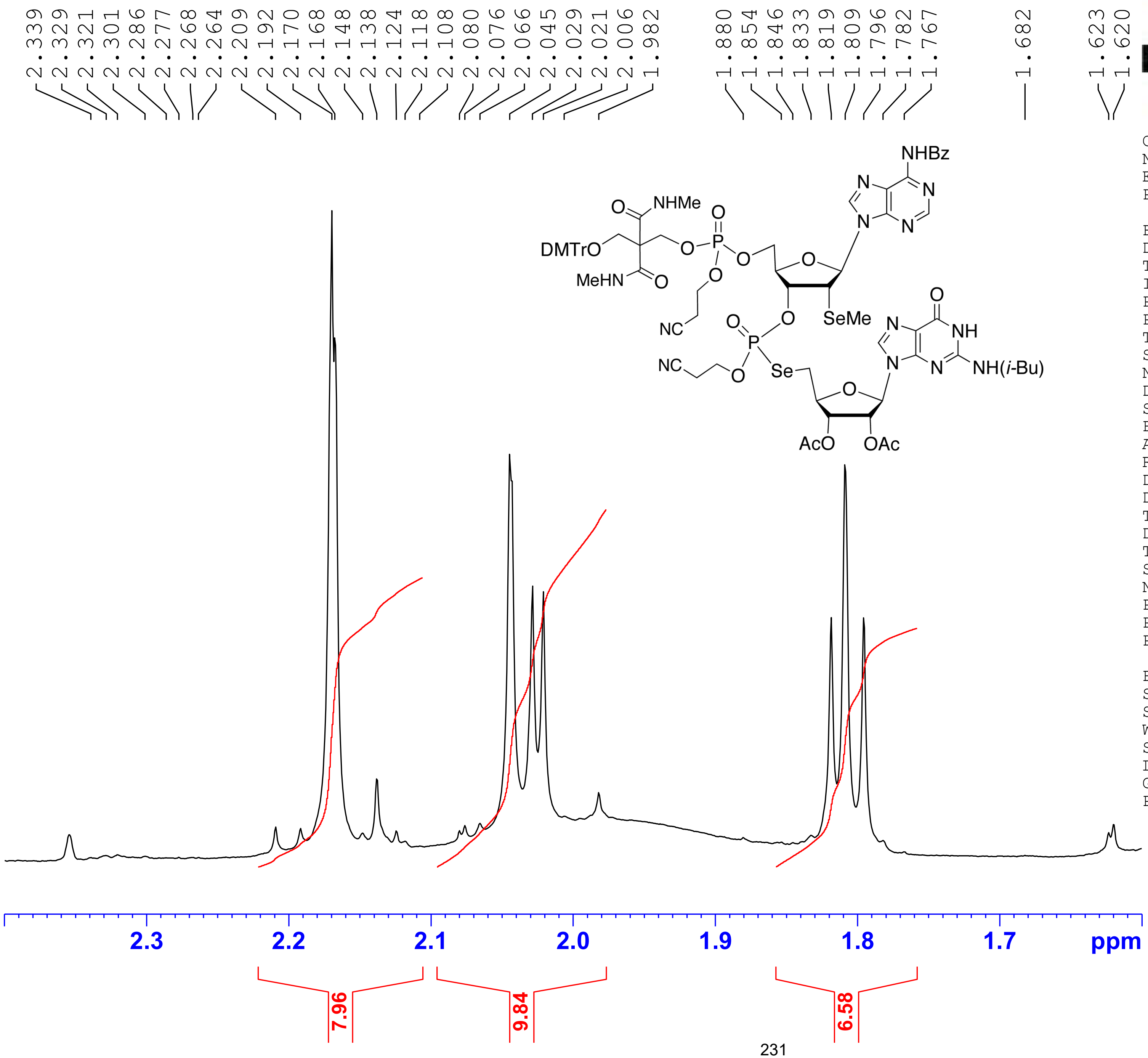

Current Data Parameters
NAME LH-II-80 OLD NMR
EXPNO 11
PROCNO 1

F2 - Acquisition Parameters
Date_ 20230907
Time 19.50 h
INSTRUM AVIII_400
PROBHD Z108618_0146 (
PULPROG zg30
TD 65536
SOLVENT CDCl3
NS 32
DS 2
SWH 8223.685 Hz
FIDRES 0.250967 Hz
AQ 3.9845889 sec
RG 203
DW 60.800 usec
DE 17.42 usec
TE 300.0 K
D1 1.00000000 sec
TD0 1
SFO1 400.1124708 MHz
NUC1 1H
P0 5.00 usec
P1 15.00 usec
PLW1 17.29199982 W

F2 - Processing parameters
SI 32768
SF 400.1100072 MHz
WDW EM
SSB 0
LB 0.30 Hz
GB 0
PC 1.00

Expanded region of the ^{1}H NMR spectrum of compound 4

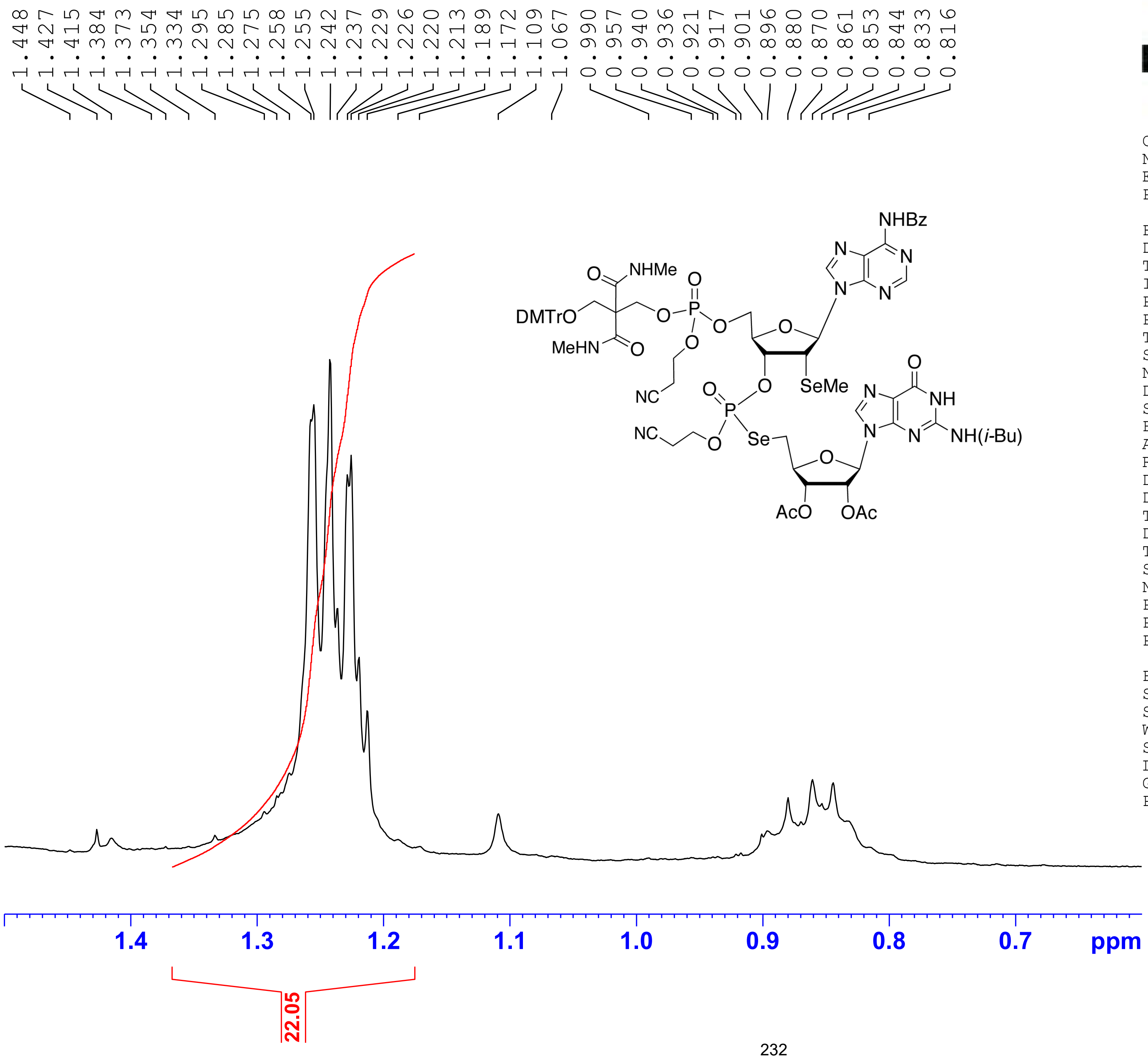

BRUKER

Current Data Parameters
NAME LH-II-80 OLD NMR
EXPNO 11
PROCNO 1

F2 - Acquisition Parameters
Date_ 20230907
Time 19.50 h
INSTRUM AVIII_400
PROBHD Z108618_0146 (
PULPROG zg30
TD 65536
SOLVENT CDCl3
NS 32
DS 2
SWH 8223.685 Hz
FIDRES 0.250967 Hz
AQ 3.9845889 sec
RG 203
DW 60.800 usec
DE 17.42 usec
TE 300.0 K
D1 1.00000000 sec
TD0 1
SFO1 400.1124708 MHz
NUC1 1H
P0 5.00 usec
P1 15.00 usec
PLW1 17.29199982 W

F2 - Processing parameters
SI 32768
SF 400.1100072 MHz
WDW EM
SSB 0
LB 0.30 Hz
GB 0
PC 1.00

^{13}C NMR spectrum of compound 4

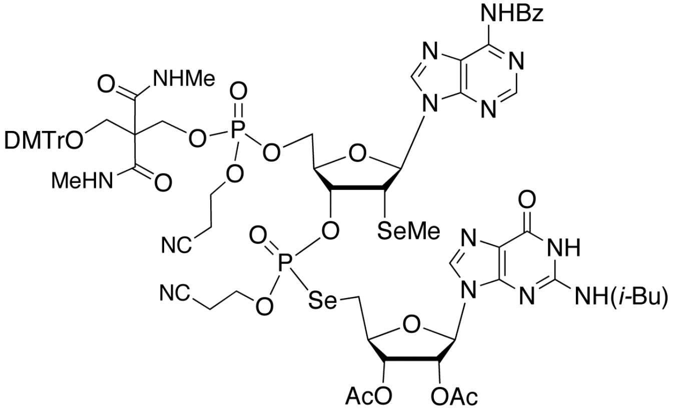

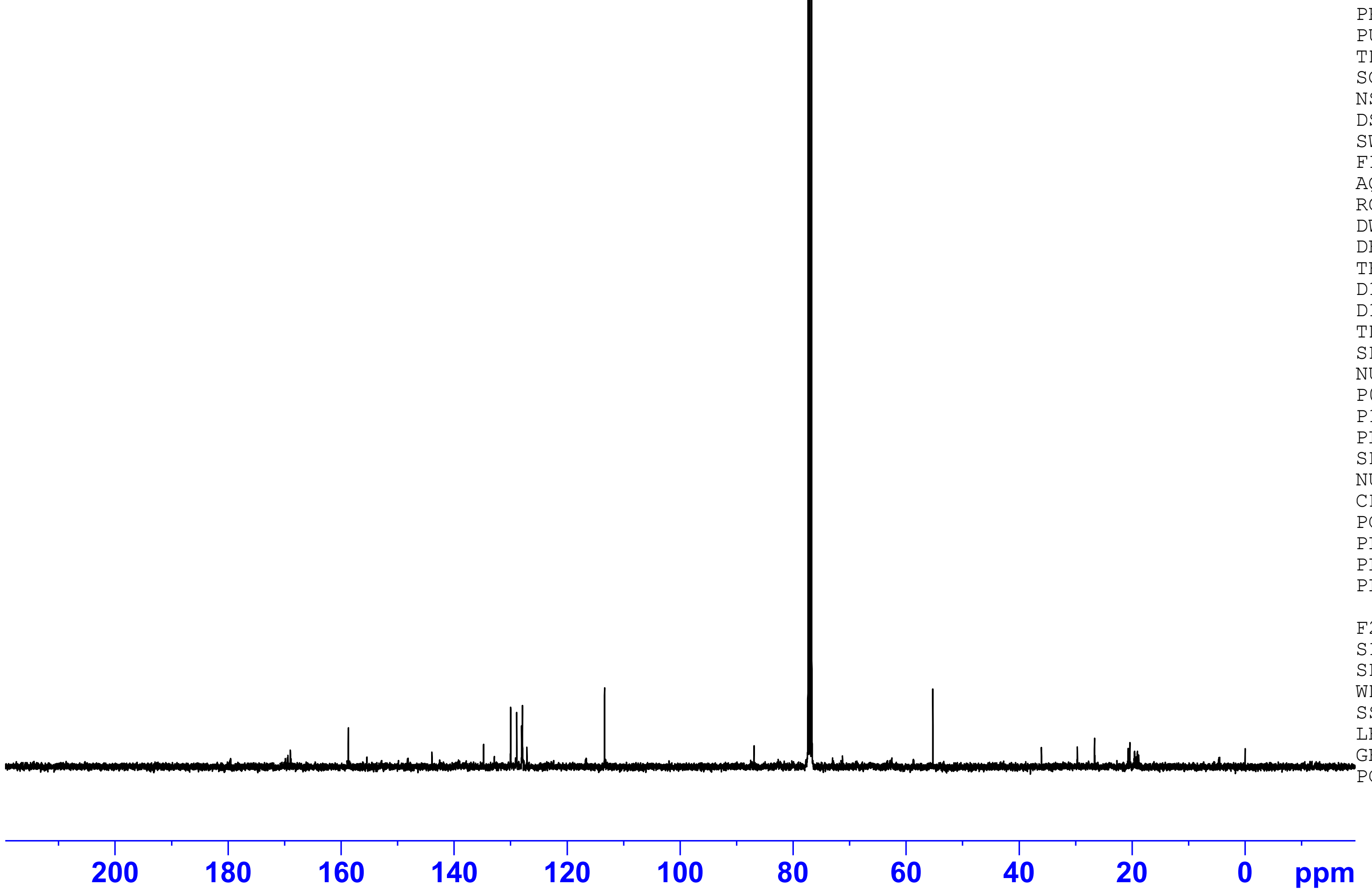

Current Data Parameters
NAME LH-II-80 OLD NMR
EXPNO 12
PROCNO 1

F2 - Acquisition Parameters
Date_ 20230907
Time 21.34 h
INSTRUM AVIII_400
PROBHD Z108618_0146 (
PULPROG zgpg30
TD 96150
SOLVENT CDCl3
NS 2000
DS 4
SWH 24038.461 Hz
FIDRES 0.500020 Hz
AQ 1.9999200 sec
RG 2050
DW 20.800 usec
DE 6.50 usec
TE 300.0 K
D1 1.00000000 sec
D11 0.03000000 sec
TD0 1
SFO1 100.6178003 MHz
NUC1 13C
P0 2.90 usec
P1 8.70 usec
PLW1 96.68000031 W
SFO2 400.1116004 MHz
NUC2 1H
CPDPRG[2 waltz64
PCPD2 90.00 usec
PLW2 17.29199982 W
PLW12 0.48032999 W
PLW13 0.24160001 W

F2 - Processing parameters
SI 131072
SF 100.6077432 MHz
WDW EM
SSB 0
LB 1.00 Hz
GB 0
PC 1.40

Expanded region of the ^{13}C NMR spectrum of compound 4

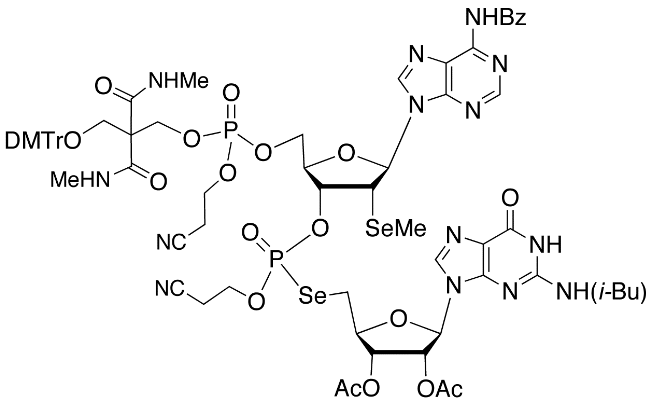

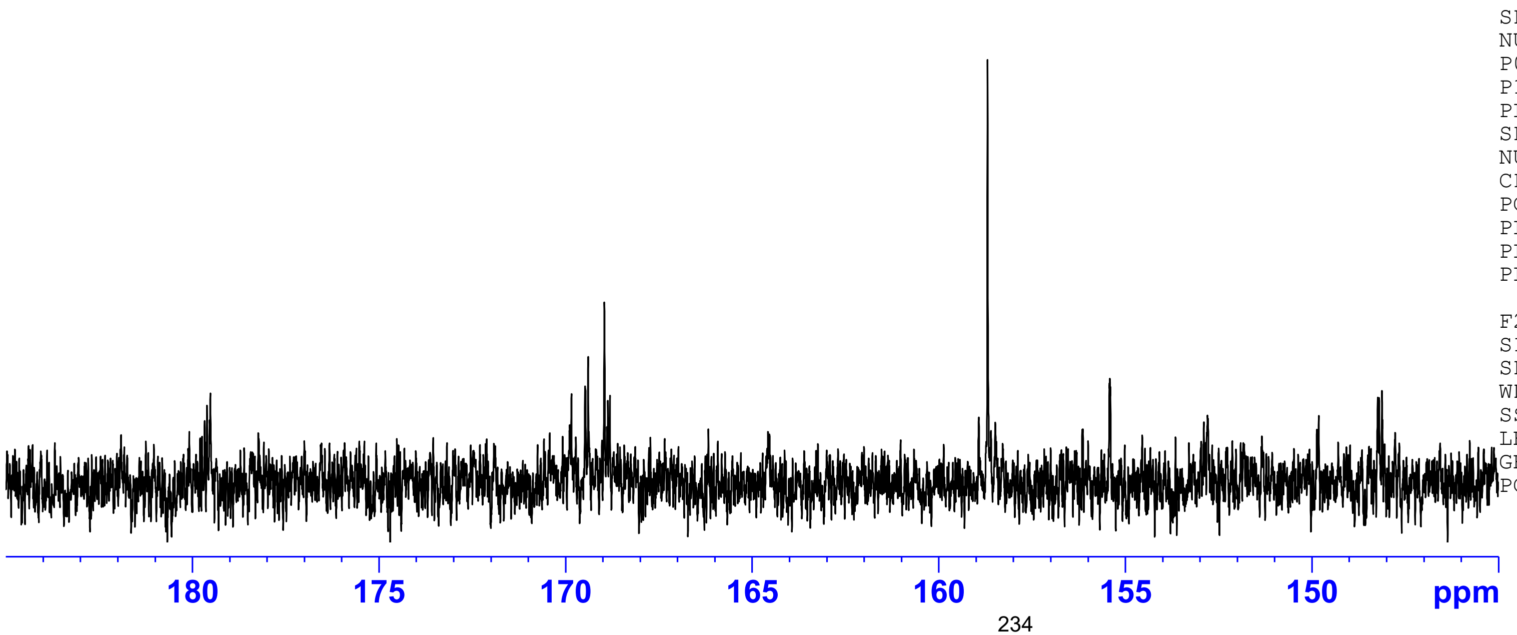

Current Data Parameters

NAME	LH-II-80 OLD NMR
EXPNO	12
PROCNO	1

F2 - Acquisition Parameters

Date_	20230907
Time	21.34 h
INSTRUM	AVIII_400
PROBHD	Z108618_0146 (
PULPROG	zgpg30
TD	96150
SOLVENT	CDCl3
NS	2000
DS	4
SWH	24038.461 Hz
FIDRES	0.500020 Hz
AQ	1.9999200 sec
RG	2050
DW	20.800 usec
DE	6.50 usec
TE	300.0 K
D1	1.00000000 sec
D11	0.03000000 sec
TD0	1
SFO1	100.6178003 MHz
NUC1	13C
P0	2.90 usec
P1	8.70 usec
PLW1	96.68000031 W
SFO2	400.1116004 MHz
NUC2	1H
CPDPRG[2	waltz64
PCPD2	90.00 usec
PLW2	17.29199982 W
PLW12	0.48032999 W
PLW13	0.24160001 W

F2 - Processing parameters

SI	131072
SF	100.6077432 MHz
WDW	EM
SSB	0
LB	1.00 Hz
GB	0
PC	1.40

Expanded region of the ${}^{13}C$ NMR spectrum of compound 4

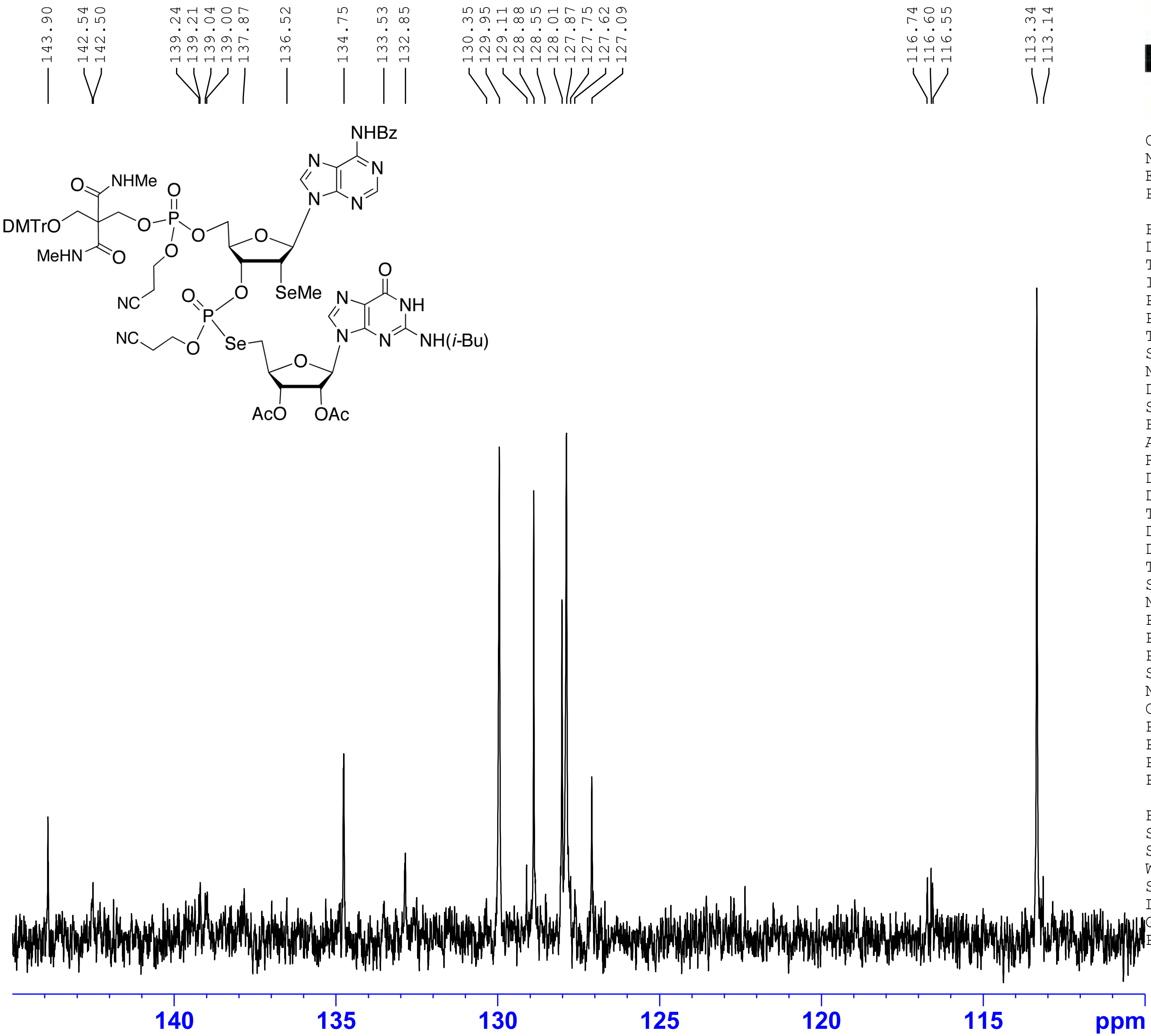

Current Data Parameters
NAME LH-II-80 OLD NMR
EXPNO 12
PROCNO 1

F2 - Acquisition Parameters
Date_ 20230907
Time 21.34 h
INSTRUM AVIII_400
PROBHD Z108618_0146 (
PULPROG zgpg30
TD 96150
SOLVENT CDCl3
NS 2000
DS 4
SWH 24038.461 Hz
FIDRES 0.500020 Hz
AQ 1.9999200 sec
RG 2050
DW 20.800 usec
DE 6.50 usec
TE 300.0 K
D1 1.00000000 sec
D11 0.03000000 sec
TD0 1
SFO1 100.6178003 MHz
NUC1 13C
P0 2.90 usec
P1 8.70 usec
PLW1 96.68000031 W
SFO2 400.1116004 MHz
NUC2 1H
CPDPRG[2 waltz64
PCPD2 90.00 usec
PLW2 17.29199982 W
PLW12 0.48032999 W
PLW13 0.24160001 W

F2 - Processing parameters
SI 131072
SF 100.6077432 MHz
WDW EM
SSB 0
LB 1.00 Hz
GB 0
PC 1.40

Expanded region of the ^{13}C NMR spectrum of compound 4

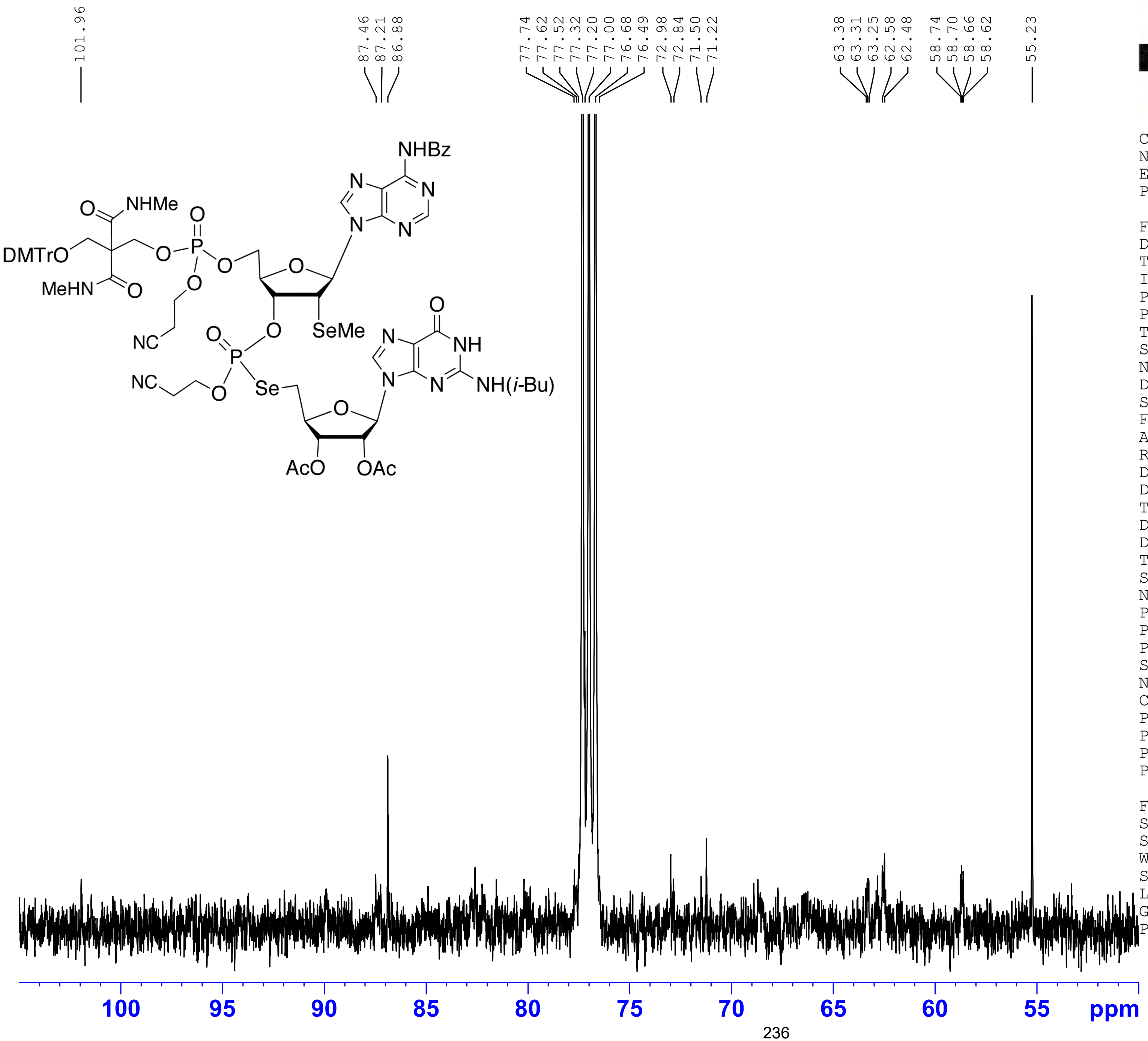


```
Current Data Parameters
NAME      LH-II-80 OLD NMR
EXPNO                   12
PROCNO                   1

F2 - Acquisition Parameters
Date_             20230907
Time                 21.34 h
INSTRUM           AVIII_400
PROBHD     Z108618_0146 (
PULPROG             zgpg30
TD                   96150
SOLVENT              CDCl3
NS                    2000
DS                       4
SWH              24038.461 Hz
FIDRES            0.500020 Hz
AQ               1.9999200 sec
RG                    2050
DW                  20.800 usec
DE                    6.50 usec
TE                   300.0 K
D1              1.00000000 sec
D11             0.03000000 sec
TD0                      1
SFO1           100.6178003 MHz
NUC1                   13C
P0                    2.90 usec
P1                    8.70 usec
PLW1           96.68000031 W
SFO2           400.1116004 MHz
NUC2                    1H
CPDPRG[2           waltz64
PCPD2                90.00 usec
PLW2           17.29199982 W
PLW12           0.48032999 W
PLW13           0.24160001 W

F2 - Processing parameters
SI                  131072
SF             100.6077432 MHz
WDW                     EM
SSB                      0
LB                    1.00 Hz
GB                       0
PC                    1.40
```

Expanded region of the ^{13}C NMR spectrum of compound 4

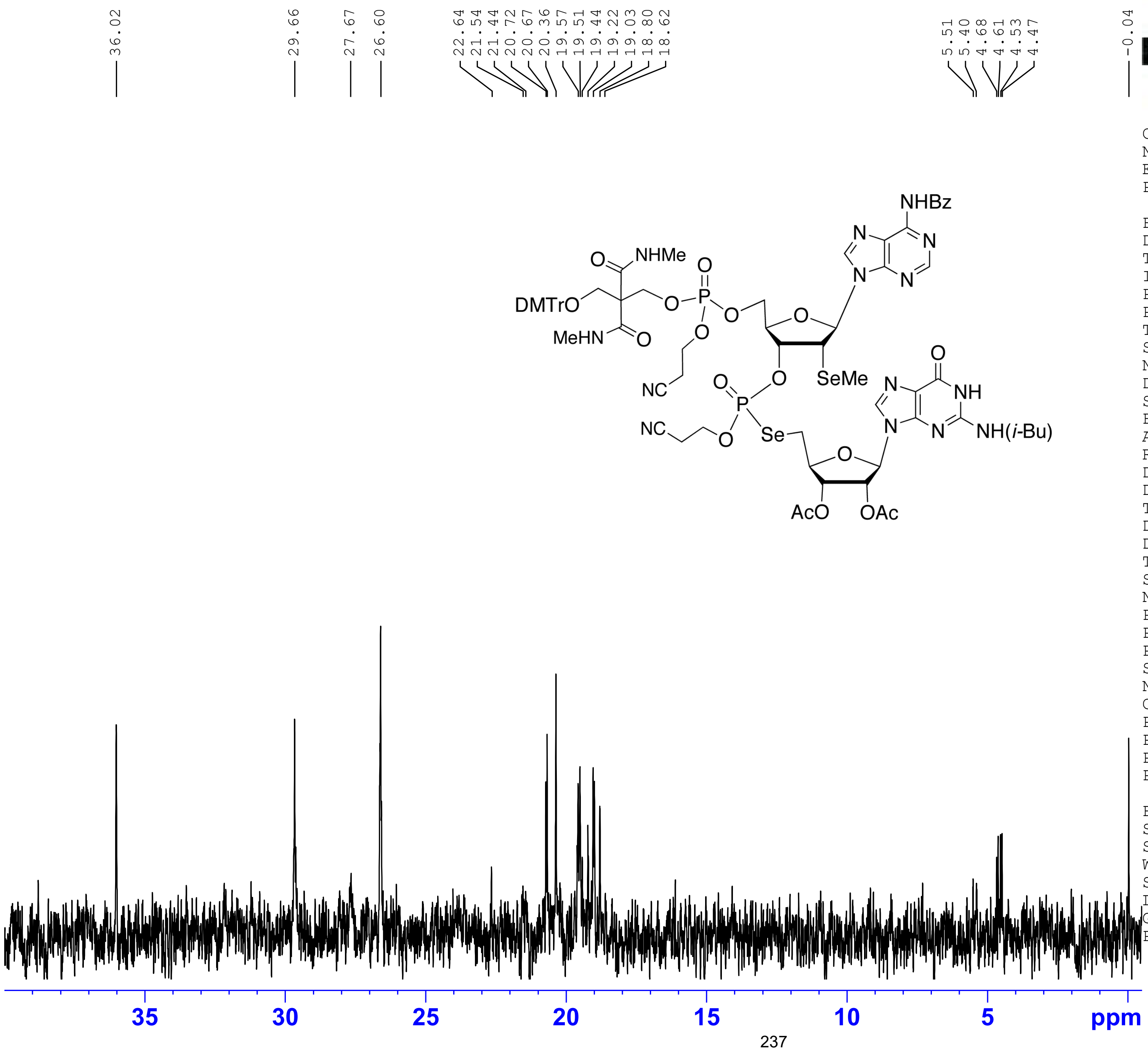

Current Data Parameters
NAME LH-II-80 OLD NMR
EXPNO 12
PROCNO 1

F2 - Acquisition Parameters

Date_	20230907	
Time	21.34	h
INSTRUM	AVIII_400	
PROBHD	Z108618_0146 (	
PULPROG	zgpg30	
TD	96150	
SOLVENT	CDCl3	
NS	2000	
DS	4	
SWH	24038.461	Hz
FIDRES	0.500020	Hz
AQ	1.9999200	sec
RG	2050	
DW	20.800	usec
DE	6.50	usec
TE	300.0	K
D1	1.00000000	sec
D11	0.03000000	sec
TD0	1	
SFO1	100.6178003	MHz
NUC1	13C	
P0	2.90	usec
P1	8.70	usec
PLW1	96.68000031	W
SFO2	400.1116004	MHz
NUC2	1H	
CPDPRG[2	waltz64	
PCPD2	90.00	usec
PLW2	17.29199982	W
PLW12	0.48032999	W
PLW13	0.24160001	W

F2 - Processing parameters

SI	131072	
SF	100.6077432	MHz
WDW	EM	
SSB	0	
LB	1.00	Hz
GB	0	
PC	1.40	

^{13}C DEPT-135 NMR spectrum of compound 4

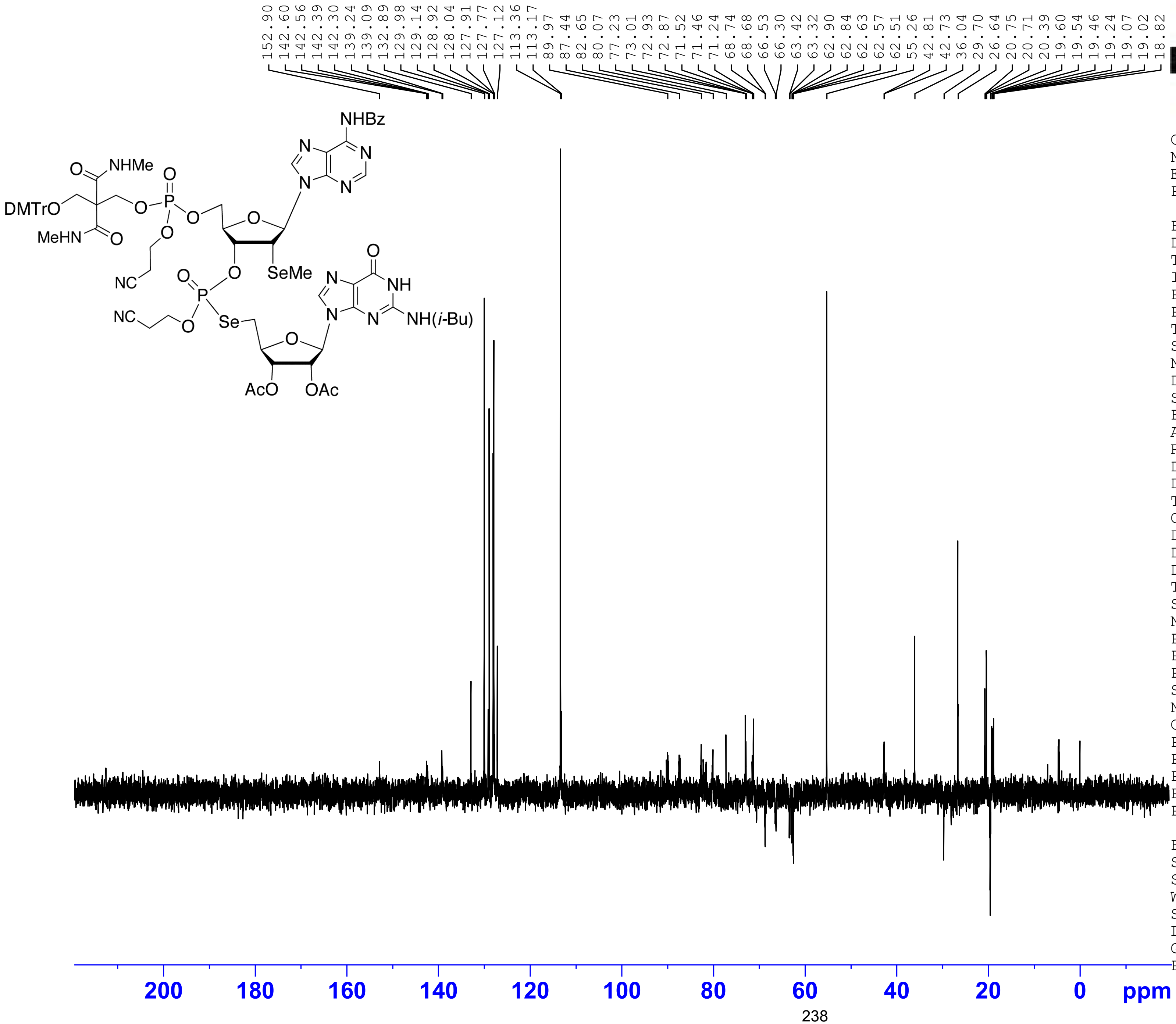

Current Data Parameters
NAME LH-II-80 OLD NMR
EXPNO 13
PROCNO 1

F2 - Acquisition Parameters

Date_	20230907	
Time	23.01	h
INSTRUM	AVIII_400	
PROBHD	Z108618_0146 (	
PULPROG	dept135	
TD	65536	
SOLVENT	CDCl3	
NS	1500	
DS	4	
SWH	24038.461	Hz
FIDRES	0.733596	Hz
AQ	1.3631488	sec
RG	2050	
DW	20.800	usec
DE	6.50	usec
TE	300.0	K
CNST2	145.0000000	
D1	2.00000000	sec
D2	0.00344828	sec
D12	0.00002000	sec
TD0	1	
SFO1	100.6178003	MHz
NUC1	13C	
P1	8.70	usec
P2	17.40	usec
PLW1	96.68000031	W
SFO2	400.1116004	MHz
NUC2	1H	
CPDPRG[2	waltz64	
P3	15.00	usec
P4	30.00	usec
PCPD2	90.00	usec
PLW2	17.29199982	W
PLW12	0.48032999	W

F2 - Processing parameters

SI	32768	
SF	100.6077400	MHz
WDW	EM	
SSB	0	
LB	1.00	Hz
GB	0	
PC	1.40	

Expanded region of the ^{13}C DEPT-135 NMR spectrum of compound 4

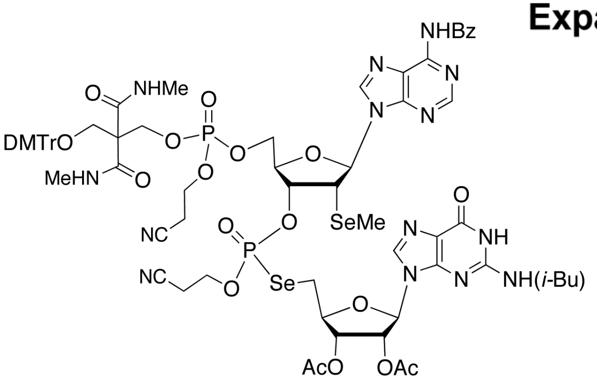

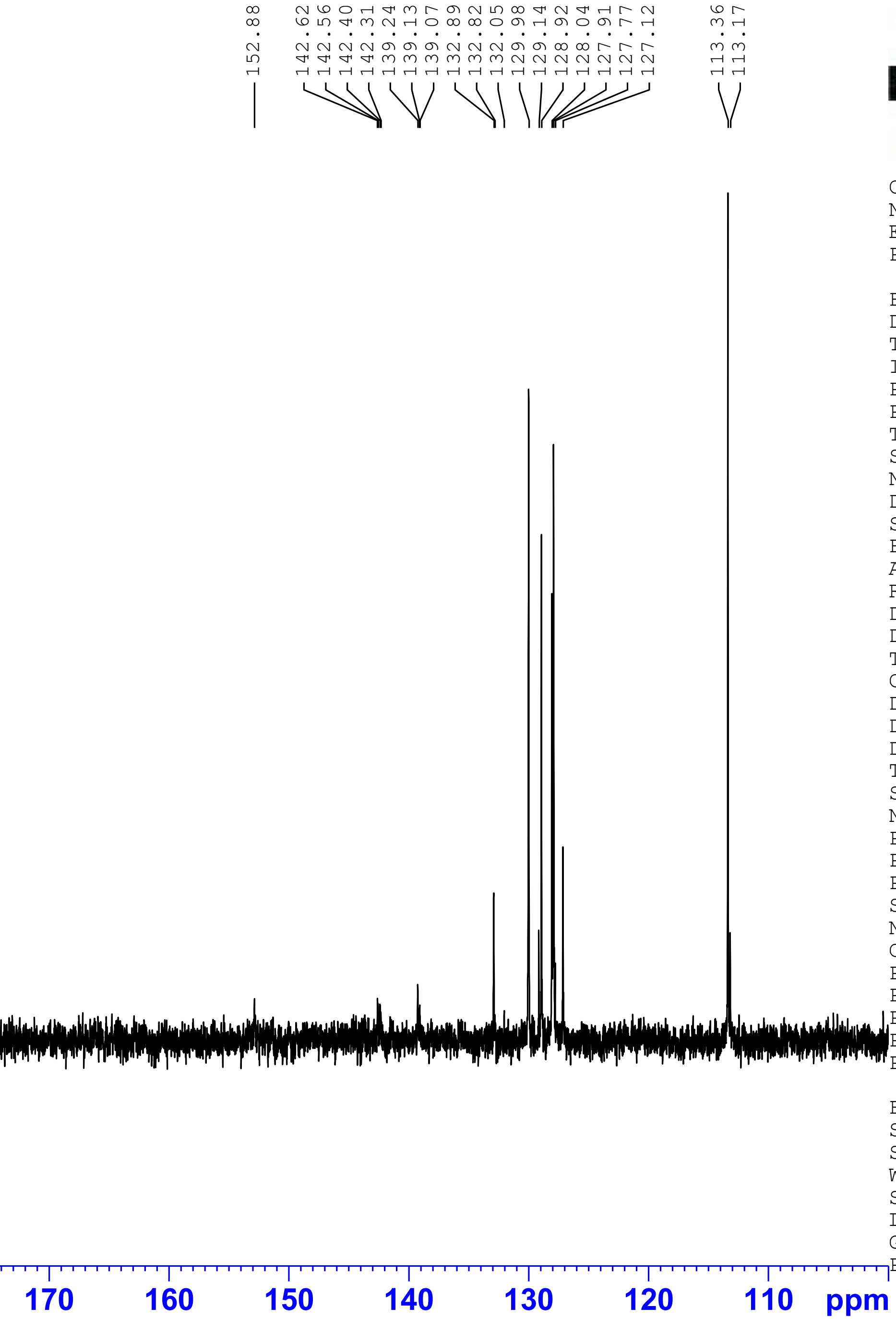


```
Current Data Parameters
NAME       LH-II-80 OLD NMR
EXPNO                    13
PROCNO                    1

F2 - Acquisition Parameters
Date_              20230907
Time                  23.01 h
INSTRUM            AVIII_400
PROBHD     Z108618_0146 (
PULPROG            dept135
TD                   65536
SOLVENT              CDCl3
NS                    1500
DS                       4
SWH              24038.461 Hz
FIDRES            0.733596 Hz
AQ               1.3631488 sec
RG                    2050
DW                  20.800 usec
DE                    6.50 usec
TE                   300.0 K
CNST2          145.0000000
D1              2.00000000 sec
D2              0.00344828 sec
D12             0.00002000 sec
TD0                      1
SFO1           100.6178003 MHz
NUC1                   13C
P1                    8.70 usec
P2                   17.40 usec
PLW1           96.68000031 W
SFO2           400.1116004 MHz
NUC2                    1H
CPDPRG[2           waltz64
P3                   15.00 usec
P4                   30.00 usec
PCPD2                90.00 usec
PLW2           17.29199982 W
PLW12           0.48032999 W

F2 - Processing parameters
SI                   32768
SF             100.6077400 MHz
WDW                     EM
SSB                      0
LB                    1.00 Hz
GB                       0
PC                    1.40
```

Expanded region of the ^{13}C DEPT-135 NMR spectrum of compound 4

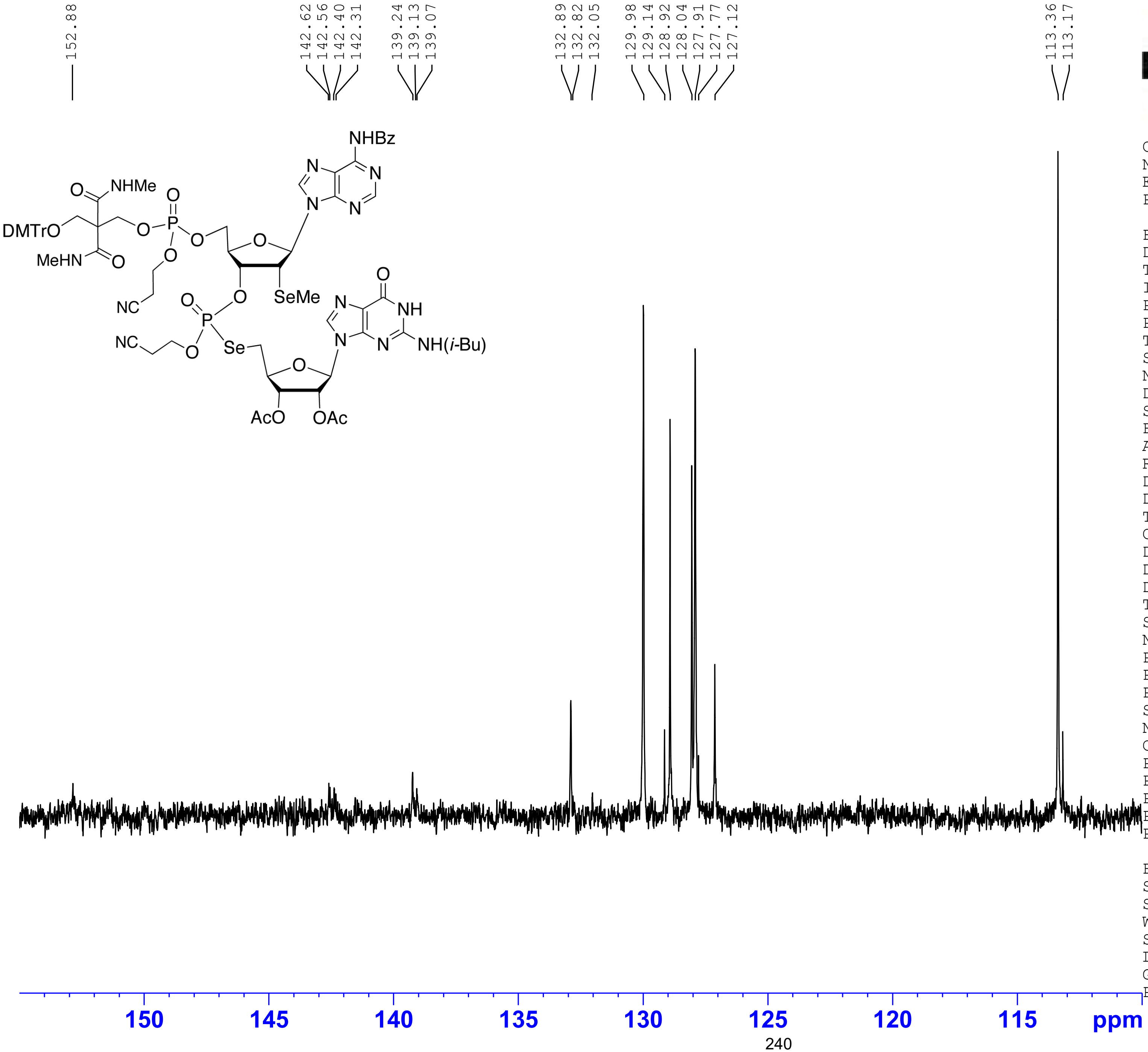

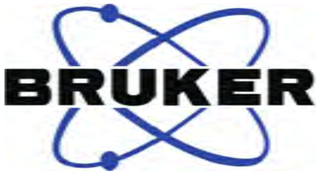

Current Data Parameters
NAME LH-II-80 OLD NMR
EXPNO 13
PROCNO 1

F2 - Acquisition Parameters
Date_ 20230907
Time 23.01 h
INSTRUM AVIII_400
PROBHD Z108618_0146 (
PULPROG dept135
TD 65536
SOLVENT CDCl3
NS 1500
DS 4
SWH 24038.461 Hz
FIDRES 0.733596 Hz
AQ 1.3631488 sec
RG 2050
DW 20.800 usec
DE 6.50 usec
TE 300.0 K
CNST2 145.0000000
D1 2.00000000 sec
D2 0.00344828 sec
D12 0.00002000 sec
TD0 1
SFO1 100.6178003 MHz
NUC1 13C
P1 8.70 usec
P2 17.40 usec
PLW1 96.68000031 W
SFO2 400.1116004 MHz
NUC2 1H
CPDPRG[2 waltz64
P3 15.00 usec
P4 30.00 usec
PCPD2 90.00 usec
PLW2 17.29199982 W
PLW12 0.48032999 W

F2 - Processing parameters
SI 32768
SF 100.6077400 MHz
WDW EM
SSB 0
LB 1.00 Hz
GB 0
PC 1.40

Expanded region of the ^{13}C DEPT-135 NMR spectrum of compound 4

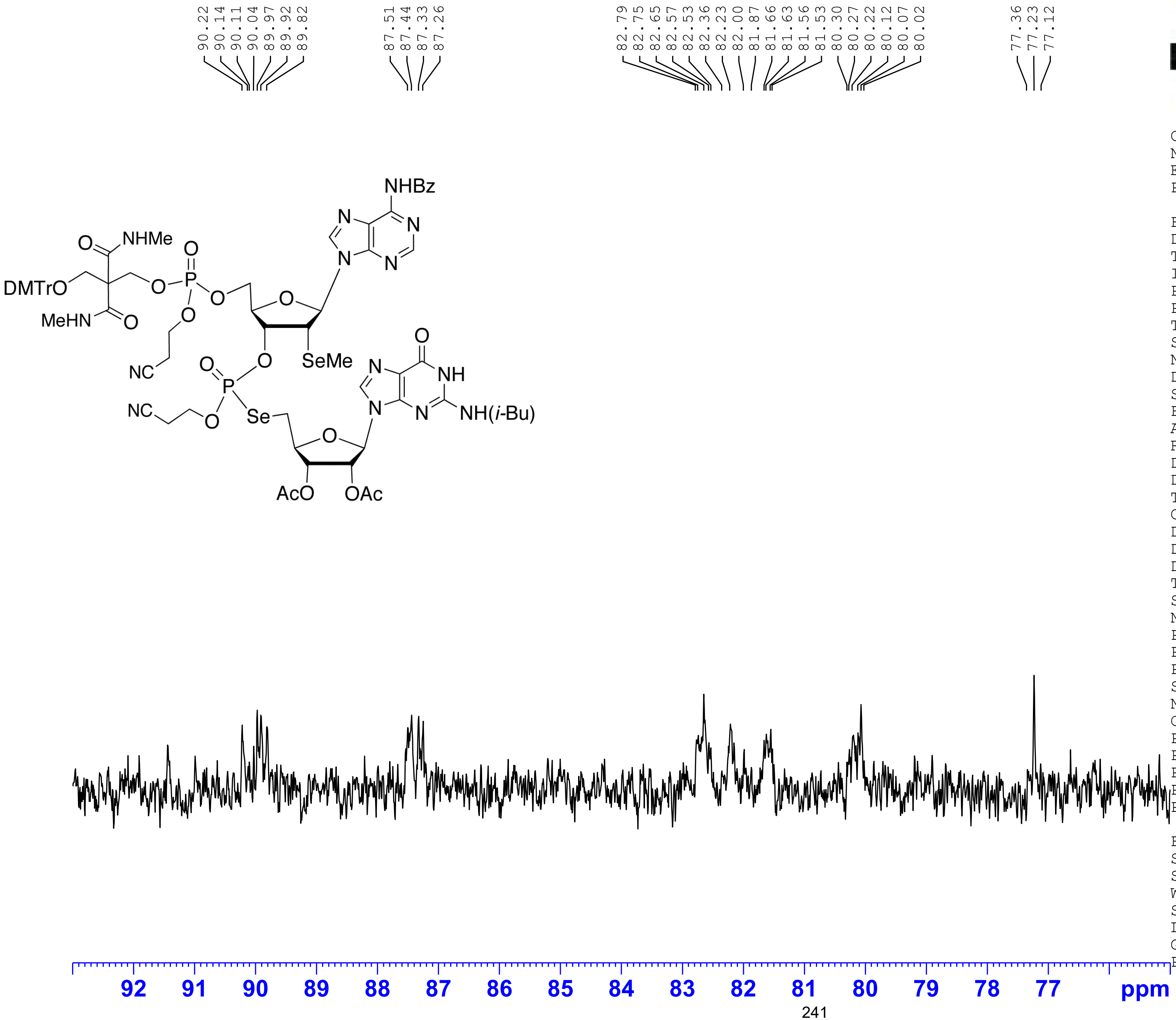


```
Current Data Parameters
NAME       LH-II-80 OLD NMR
EXPNO                    13
PROCNO                    1

F2 - Acquisition Parameters
Date_              20230907
Time                  23.01 h
INSTRUM            AVIII_400
PROBHD    Z108618_0146 (
PULPROG            dept135
TD                   65536
SOLVENT              CDCl3
NS                    1500
DS                       4
SWH              24038.461 Hz
FIDRES            0.733596 Hz
AQ               1.3631488 sec
RG                    2050
DW                  20.800 usec
DE                    6.50 usec
TE                   300.0 K
CNST2          145.0000000
D1              2.00000000 sec
D2              0.00344828 sec
D12             0.00002000 sec
TD0                      1
SFO1           100.6178003 MHz
NUC1                   13C
P1                    8.70 usec
P2                   17.40 usec
PLW1           96.68000031 W
SFO2           400.1116004 MHz
NUC2                    1H
CPDPRG[2           waltz64
P3                   15.00 usec
P4                   30.00 usec
PCPD2                90.00 usec
PLW2           17.29199982 W
PLW12           0.48032999 W

F2 - Processing parameters
SI                   32768
SF             100.6077400 MHz
WDW                     EM
SSB                      0
LB                    1.00 Hz
GB                       0
PC                    1.40
```

Expanded region of the ^{13}C DEPT-135 NMR spectrum of compound 4

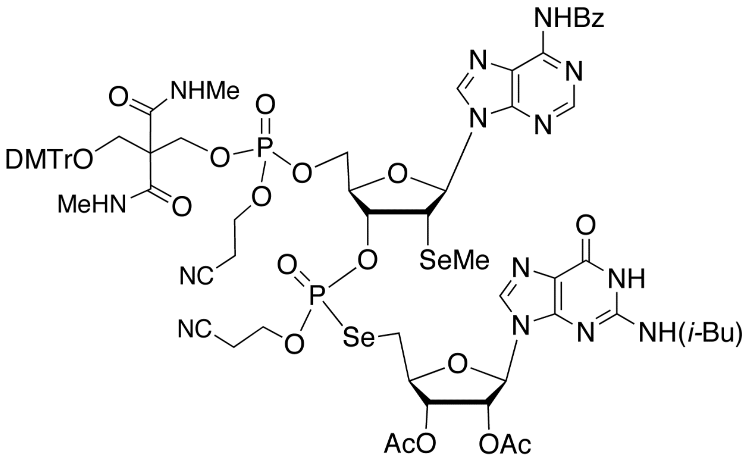

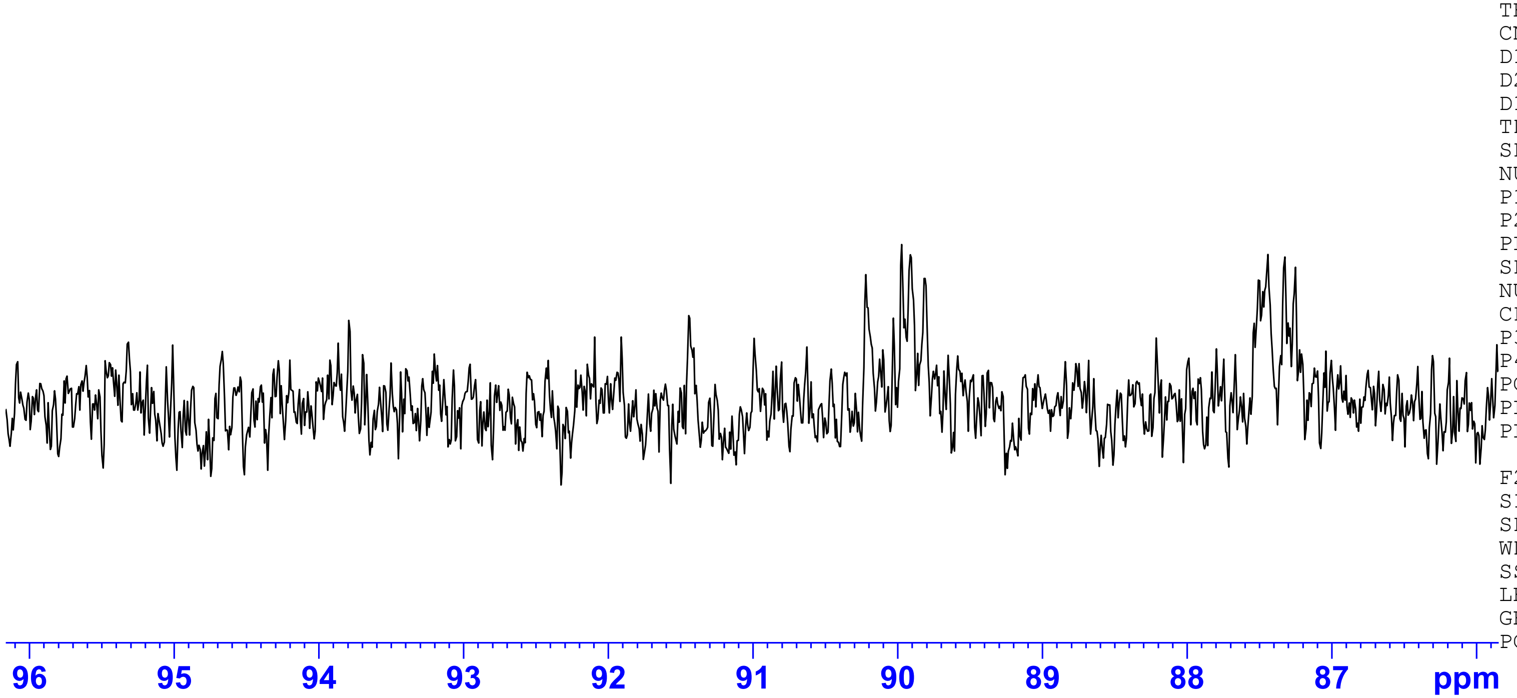


```
Current Data Parameters
NAME       LH-II-80 OLD NMR
EXPNO                    13
PROCNO                    1

F2 - Acquisition Parameters
Date_              20230907
Time                  23.01 h
INSTRUM            AVIII_400
PROBHD    Z108618_0146 (
PULPROG            dept135
TD                   65536
SOLVENT              CDCl3
NS                    1500
DS                       4
SWH              24038.461 Hz
FIDRES            0.733596 Hz
AQ               1.3631488 sec
RG                    2050
DW                  20.800 usec
DE                    6.50 usec
TE                   300.0 K
CNST2          145.0000000
D1              2.00000000 sec
D2              0.00344828 sec
D12             0.00002000 sec
TD0                      1
SFO1           100.6178003 MHz
NUC1                   13C
P1                    8.70 usec
P2                   17.40 usec
PLW1           96.68000031 W
SFO2           400.1116004 MHz
NUC2                    1H
CPDPRG[2           waltz64
P3                   15.00 usec
P4                   30.00 usec
PCPD2                90.00 usec
PLW2           17.29199982 W
PLW12           0.48032999 W

F2 - Processing parameters
SI                   32768
SF             100.6077400 MHz
WDW                     EM
SSB                      0
LB                    1.00 Hz
GB                       0
PC                    1.40
```

Expanded region of the ^{13}C DEPT-135 NMR spectrum of compound 4

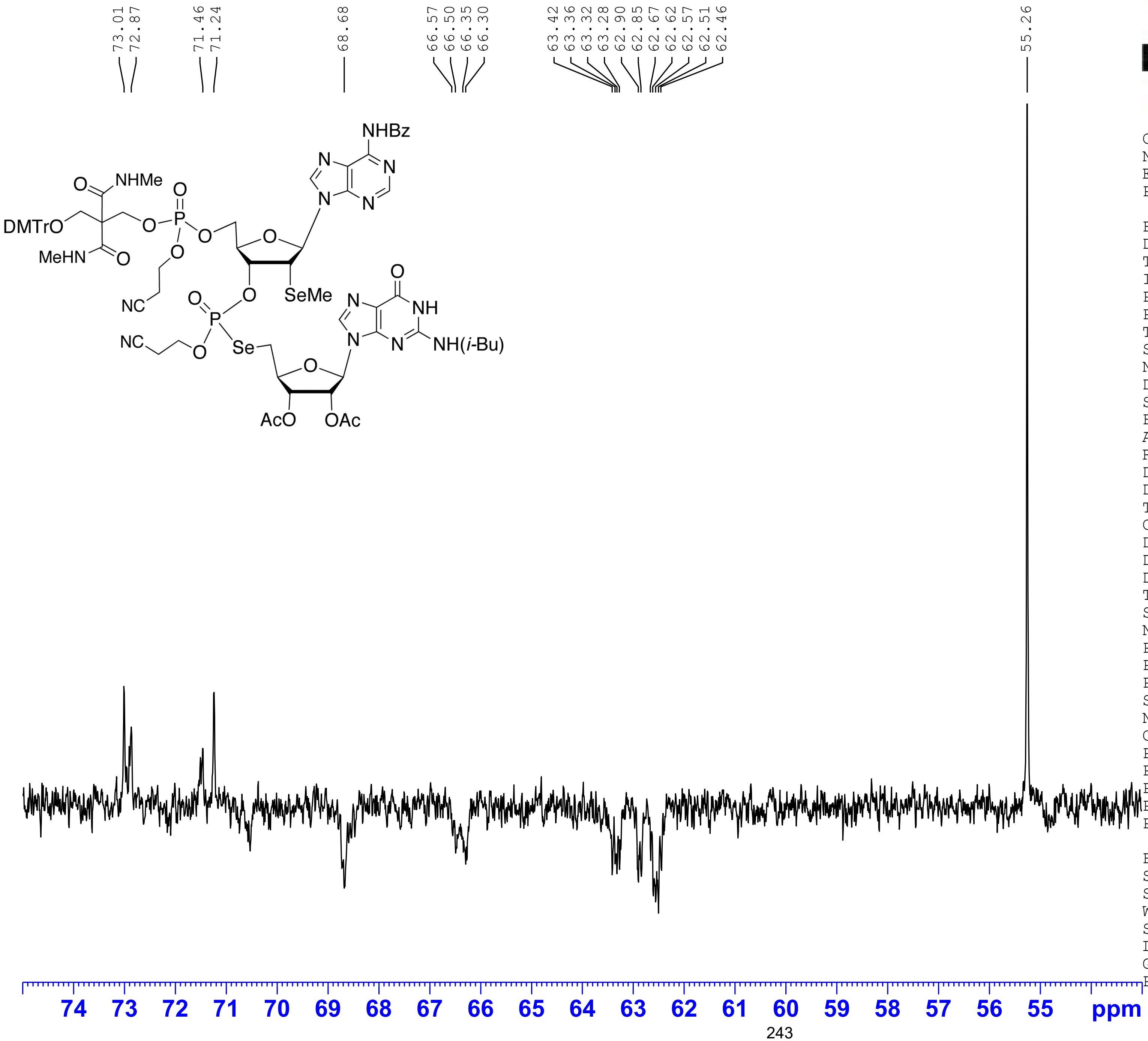

BRUKER

```
Current Data Parameters
NAME      LH-II-80 OLD NMR
EXPNO                   13
PROCNO                   1

F2 - Acquisition Parameters
Date_             20230907
Time                 23.01 h
INSTRUM           AVIII_400
PROBHD     Z108618_0146 (
PULPROG           dept135
TD                  65536
SOLVENT             CDCl3
NS                   1500
DS                      4
SWH             24038.461 Hz
FIDRES           0.733596 Hz
AQ              1.3631488 sec
RG                   2050
DW                 20.800 usec
DE                   6.50 usec
TE                  300.0 K
CNST2         145.0000000
D1             2.00000000 sec
D2             0.00344828 sec
D12            0.00002000 sec
TD0                     1
SFO1          100.6178003 MHz
NUC1                  13C
P1                   8.70 usec
P2                  17.40 usec
PLW1          96.68000031 W
SFO2          400.1116004 MHz
NUC2                   1H
CPDPRG[2          waltz64
P3                  15.00 usec
P4                  30.00 usec
PCPD2               90.00 usec
PLW2          17.29199982 W
PLW12          0.48032999 W

F2 - Processing parameters
SI                  32768
SF            100.6077400 MHz
WDW                    EM
SSB                     0
LB                   1.00 Hz
GB                      0
PC                   1.40
```

Expanded region of the ^{13}C DEPT-135 NMR spectrum of compound 4

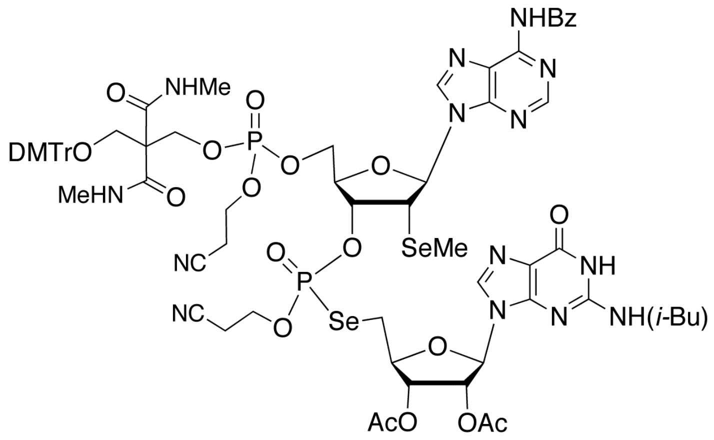

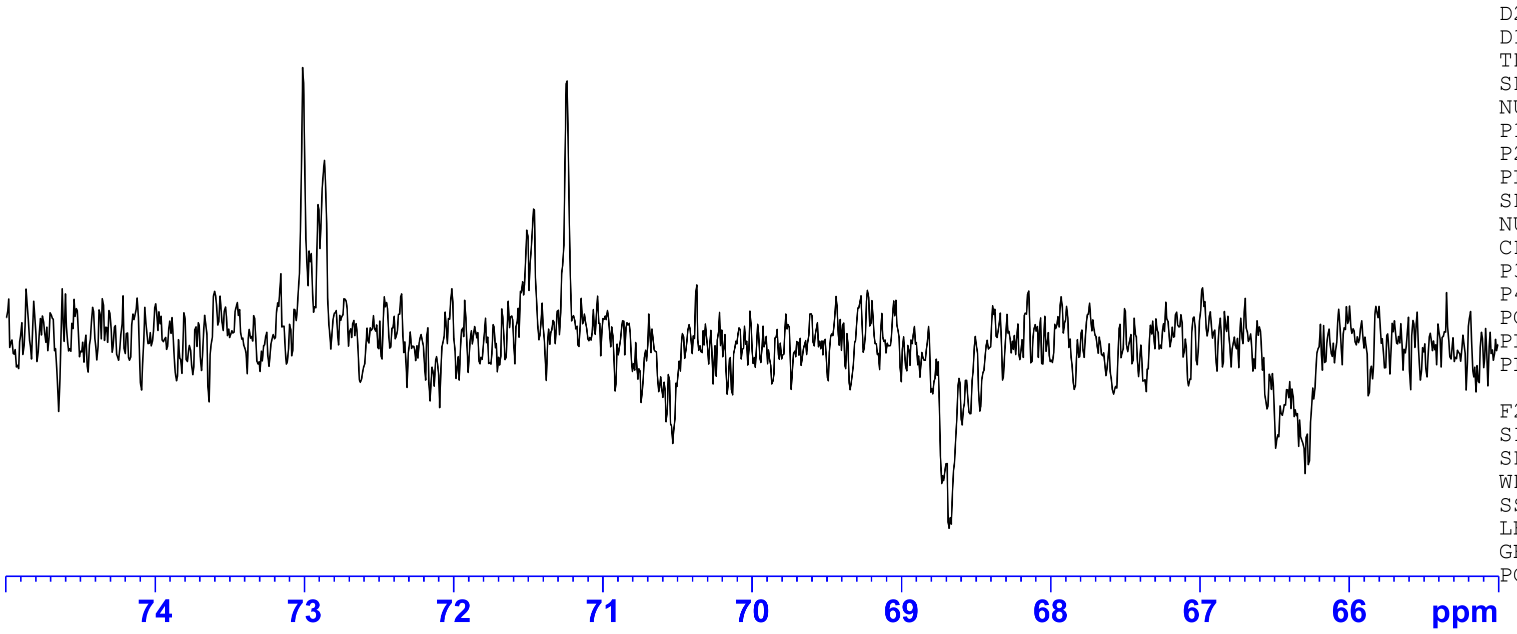

Current Data Parameters
NAME LH-II-80 OLD NMR
EXPNO 13
PROCNO 1

F2 - Acquisition Parameters
Date_ 20230907
Time 23.01 h
INSTRUM AVIII_400
PROBHD Z108618_0146 (
PULPROG dept135
TD 65536
SOLVENT CDCl3
NS 1500
DS 4
SWH 24038.461 Hz
FIDRES 0.733596 Hz
AQ 1.3631488 sec
RG 2050
DW 20.800 usec
DE 6.50 usec
TE 300.0 K
CNST2 145.0000000
D1 2.00000000 sec
D2 0.00344828 sec
D12 0.00002000 sec
TD0 1
SFO1 100.6178003 MHz
NUC1 13C
P1 8.70 usec
P2 17.40 usec
PLW1 96.68000031 W
SFO2 400.1116004 MHz
NUC2 1H
CPDPRG[2 waltz64
P3 15.00 usec
P4 30.00 usec
PCPD2 90.00 usec
PLW2 17.29199982 W
PLW12 0.48032999 W

F2 - Processing parameters
SI 32768
SF 100.6077400 MHz
WDW EM
SSB 0
LB 1.00 Hz
GB 0
PC 1.40

Expanded region of the ^{13}C DEPT-135 NMR spectrum of compound 4

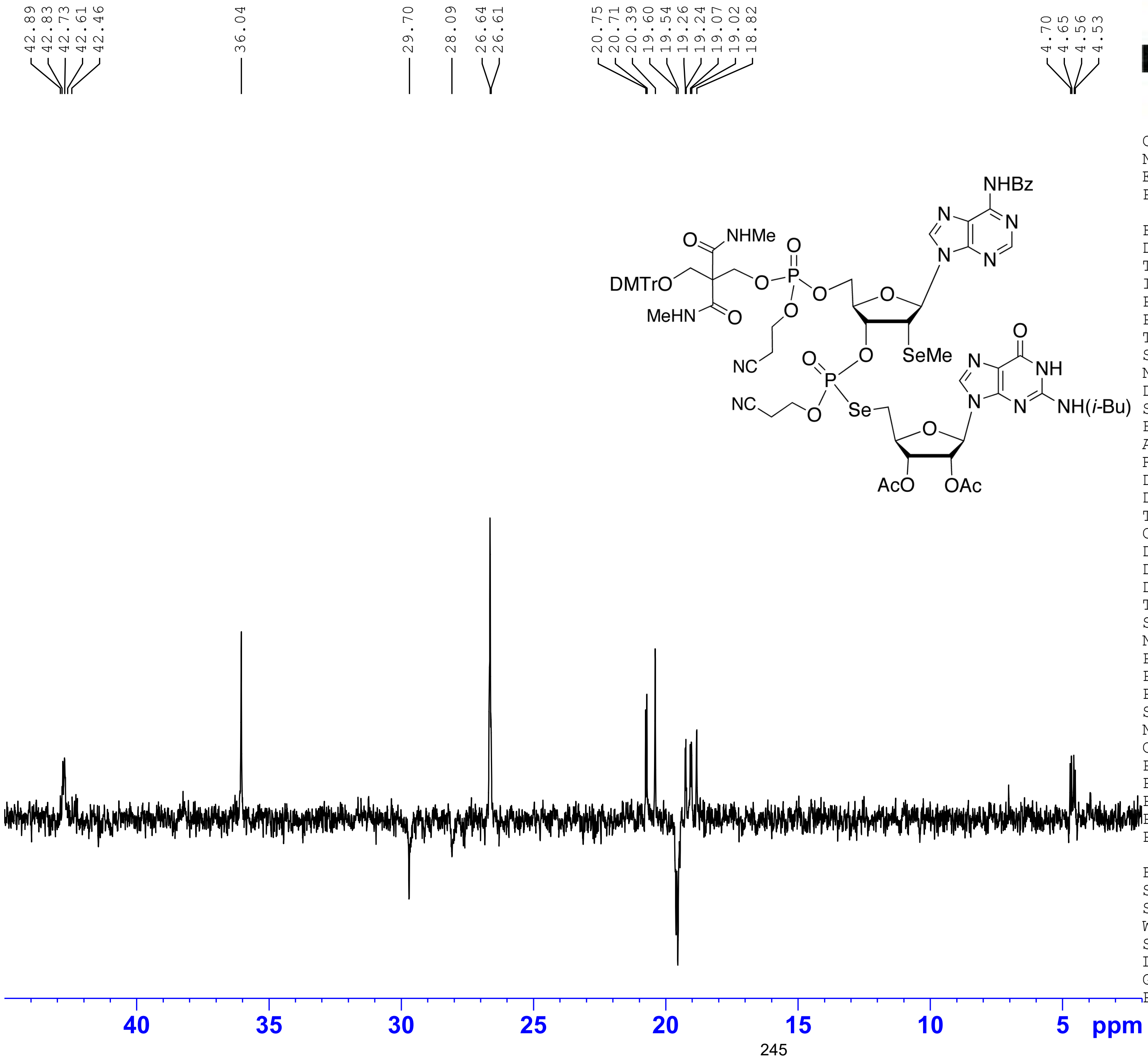

Current Data Parameters
NAME LH-II-80 OLD NMR
EXPNO 13
PROCNO 1

F2 - Acquisition Parameters
Date_ 20230907
Time 23.01 h
INSTRUM AVIII_400
PROBHD Z108618_0146 (
PULPROG dept135
TD 65536
SOLVENT CDCl3
NS 1500
DS 4
SWH 24038.461 Hz
FIDRES 0.733596 Hz
AQ 1.3631488 sec
RG 2050
DW 20.800 usec
DE 6.50 usec
TE 300.0 K
CNST2 145.0000000
D1 2.00000000 sec
D2 0.00344828 sec
D12 0.00002000 sec
TD0 1
SFO1 100.6178003 MHz
NUC1 13C
P1 8.70 usec
P2 17.40 usec
PLW1 96.68000031 W
SFO2 400.1116004 MHz
NUC2 1H
CPDPRG[2 waltz64
P3 15.00 usec
P4 30.00 usec
PCPD2 90.00 usec
PLW2 17.29199982 W
PLW12 0.48032999 W

F2 - Processing parameters
SI 32768
SF 100.6077400 MHz
WDW EM
SSB 0
LB 1.00 Hz
GB 0
PC 1.40

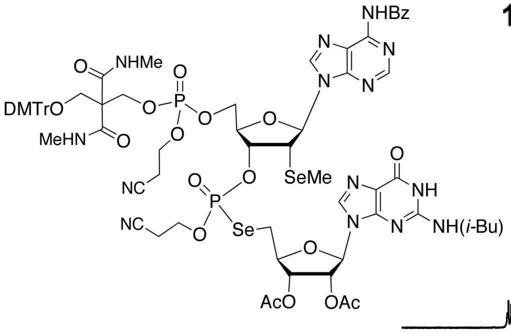

^{1}H-^{13}C HSQC NMR spectrum of compound 4

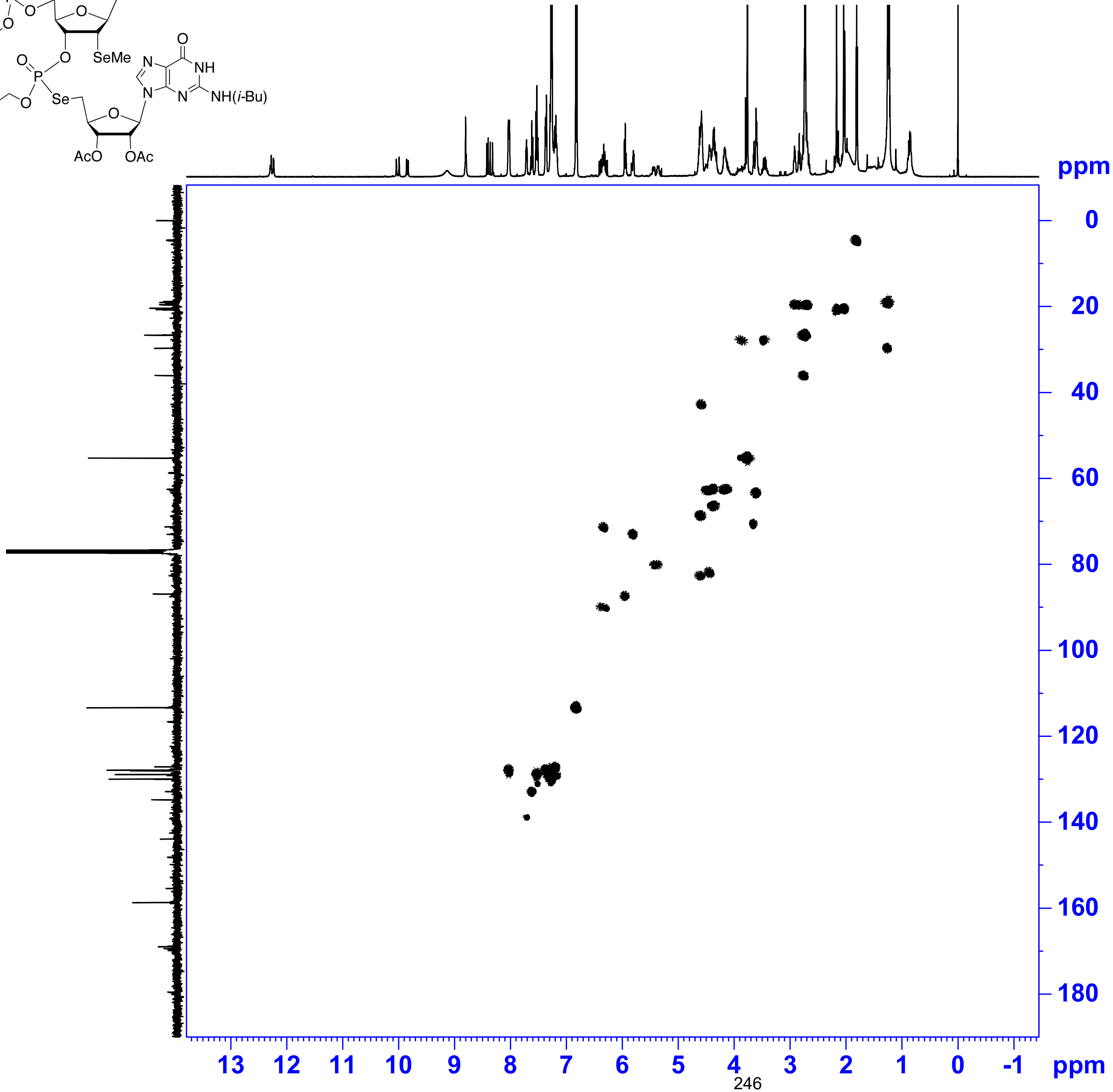


```
Current Data Parameters
NAME      LH-II-80 OLD NMR
EXPNO                   14
PROCNO                   1

F2 - Acquisition Parameters
Date_             20230907
Time                 23.29 h
INSTRUM          AVIII_400
PROBHD   Z108618_0146 (
PULPROG         hsqcedetgp
TD                    1024
SOLVENT              CDCl3
NS                       4
DS                      16
SWH               6097.561 Hz
FIDRES           11.909299 Hz
AQ               0.0839680 sec
RG                    1820
DW                  82.000 usec
DE                    6.50 usec
TE                   300.0 K
CNST2          145.0000000
D0              0.00000300 sec
D1              1.45965397 sec
D4              0.00172414 sec
D11             0.03000000 sec
D16             0.00020000 sec
D21             0.00345000 sec
IN0             0.00003000 sec
TDav                     1
ZGOPTNS
SFO1           400.1124771 MHz
NUC1                    1H
P1                   15.00 usec
P2                   30.00 usec
PLW1           17.29199982 W
SFO2           100.6152434 MHz
NUC2                   13C
CPDPRG[2              garp
P3                    8.70 usec
P4                   17.40 usec
PCPD2                56.50 usec
PLW2           96.68000031 W
PLW12           3.16230011 W
GPNAM[1]       SMSQ10.100
GPZ1                 80.00 %
GPNAM[2]       SMSQ10.100
GPZ2                 20.10 %
P16                1000.00 usec

F1 - Acquisition parameters
TD                     256
SFO1              100.6152 MHz
FIDRES          130.208328 Hz
SW                 165.648 ppm
FnMODE      Echo-Antiecho

F2 - Processing parameters
SI                    1024
SF             400.1100072 MHz
WDW                  QSINE
SSB                      2
LB                       0 Hz
GB                       0
PC                    1.40

F1 - Processing parameters
SI                    1024
MC2          echo-antiecho
SF             100.6077400 MHz
WDW                  QSINE
SSB                      2
LB                       0 Hz
GB                       0
```

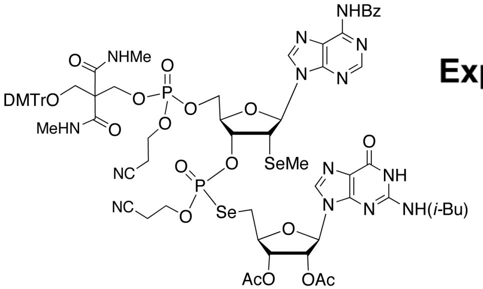

Expanded region of the ^{1}H-^{13}C HSQC NMR spectrum of compound 4

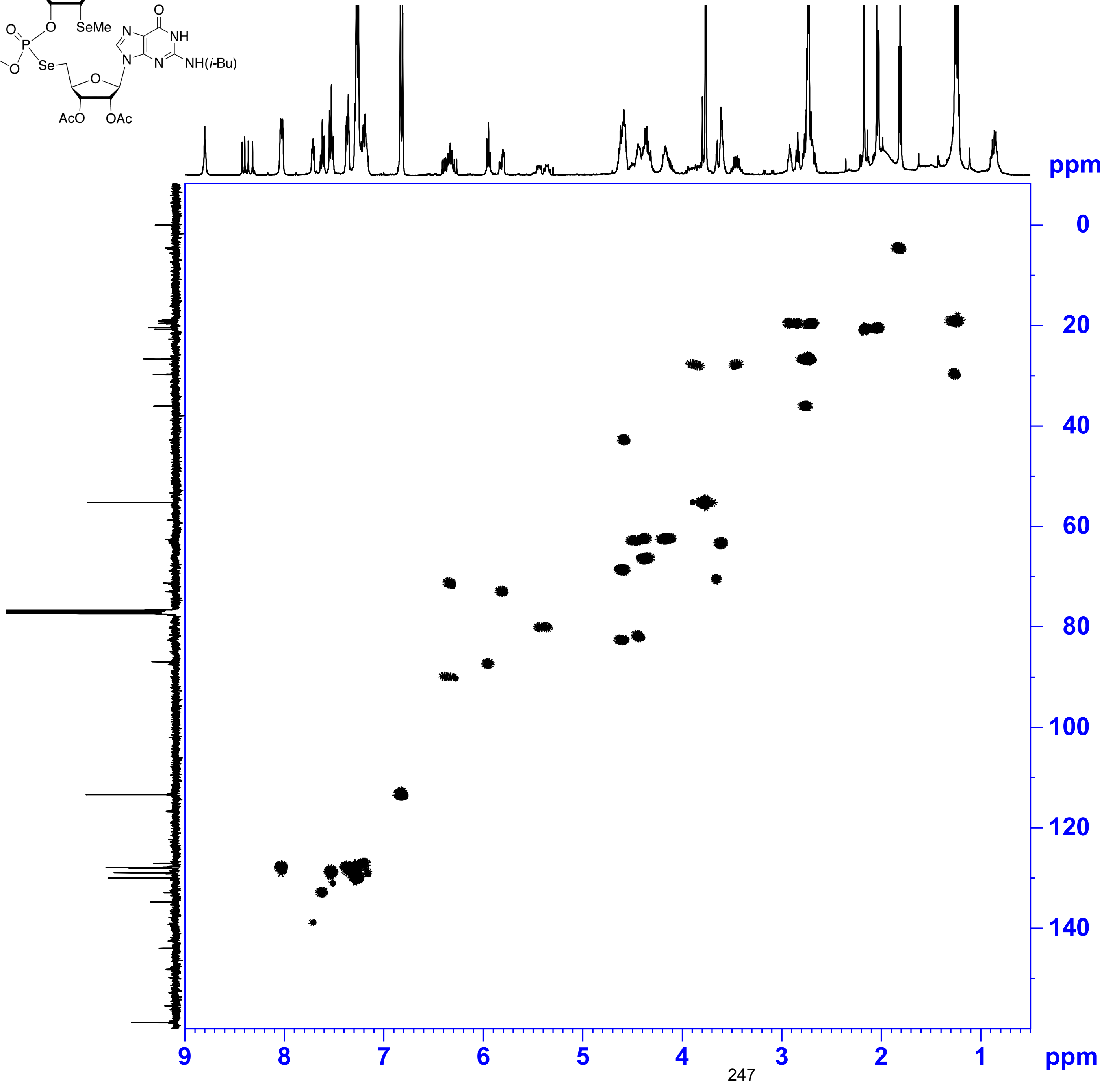


```
Current Data Parameters
NAME      LH-II-80 OLD NMR
EXPNO                   14
PROCNO                   1

F2 - Acquisition Parameters
Date_             20230907
Time                 23.29 h
INSTRUM           AVIII_400
PROBHD    Z108618_0146 (
PULPROG          hsqcedetgp
TD                    1024
SOLVENT              CDCl3
NS                       4
DS                      16
SWH               6097.561 Hz
FIDRES           11.909299 Hz
AQ               0.0839680 sec
RG                    1820
DW                  82.000 usec
DE                    6.50 usec
TE                   300.0 K
CNST2          145.0000000
D0              0.00000300 sec
D1              1.45965397 sec
D4              0.00172414 sec
D11             0.03000000 sec
D16             0.00020000 sec
D21             0.00345000 sec
IN0             0.00003000 sec
TDav                     1
ZGOPTNS
SFO1           400.1124771 MHz
NUC1                    1H
P1                   15.00 usec
P2                   30.00 usec
PLW1           17.29199982 W
SFO2           100.6152434 MHz
NUC2                   13C
CPDPRG[2              garp
P3                    8.70 usec
P4                   17.40 usec
PCPD2                56.50 usec
PLW2           96.68000031 W
PLW12           3.16230011 W
GPNAM[1]        SMSQ10.100
GPZ1                 80.00 %
GPNAM[2]        SMSQ10.100
GPZ2                 20.10 %
P16                1000.00 usec

F1 - Acquisition parameters
TD                     256
SFO1              100.6152 MHz
FIDRES          130.208328 Hz
SW                 165.648 ppm
FnMODE      Echo-Antiecho

F2 - Processing parameters
SI                    1024
SF             400.1100072 MHz
WDW                  QSINE
SSB                      2
LB                       0 Hz
GB                       0
PC                    1.40

F1 - Processing parameters
SI                    1024
MC2            echo-antiecho
SF             100.6077400 MHz
WDW                  QSINE
SSB                      2
LB                       0 Hz
GB                       0
```

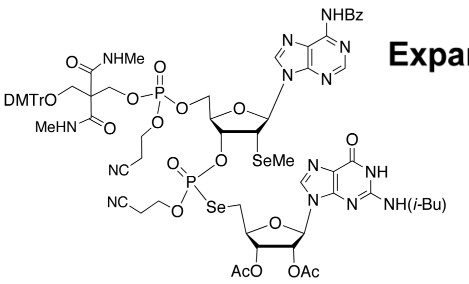

Expanded region of the ^{1}H-^{13}C HSQC NMR spectrum of compound 4

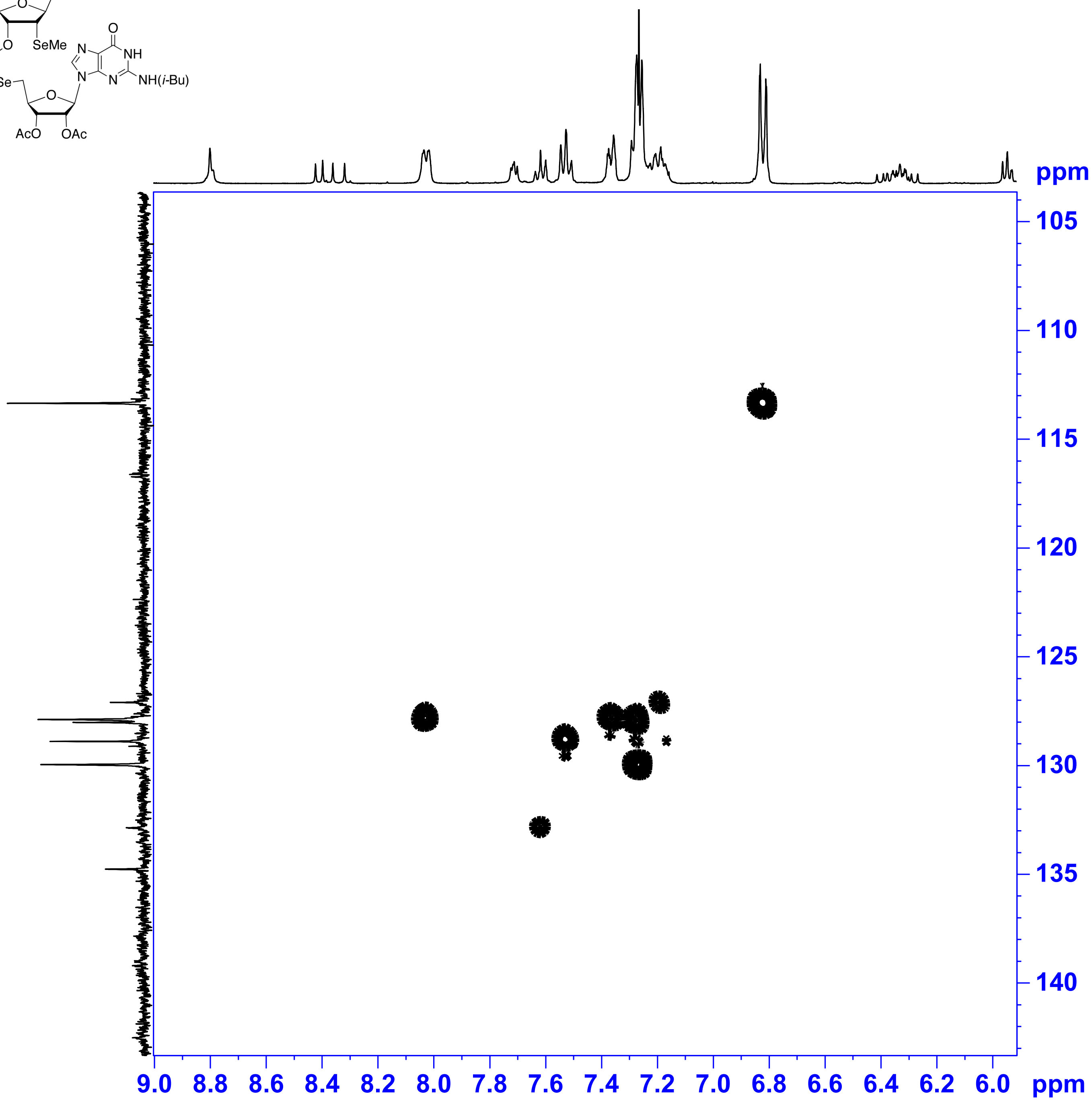

Current Data Parameters

NAME	LH-II-80 OLD NMR
EXPNO	14
PROCNO	1

F2 - Acquisition Parameters

Date_	20230907
Time	23.29 h
INSTRUM	AVIII_400
PROBHD	Z108618_0146 (
PULPROG	hsqcedetgp
TD	1024
SOLVENT	CDCl3
NS	4
DS	16
SWH	6097.561 Hz
FIDRES	11.909299 Hz
AQ	0.0839680 sec
RG	1820
DW	82.000 usec
DE	6.50 usec
TE	300.0 K
CNST2	145.0000000
D0	0.00000300 sec
D1	1.45965397 sec
D4	0.00172414 sec
D11	0.03000000 sec
D16	0.00020000 sec
D21	0.00345000 sec
IN0	0.00003000 sec
TDav	1
ZGOPTNS	
SFO1	400.1124771 MHz
NUC1	1H
P1	15.00 usec
P2	30.00 usec
PLW1	17.29199982 W
SFO2	100.6152434 MHz
NUC2	13C
CPDPRG[2	garp
P3	8.70 usec
P4	17.40 usec
PCPD2	56.50 usec
PLW2	96.68000031 W
PLW12	3.16230011 W
GPNAM[1]	SMSQ10.100
GPZ1	80.00 %
GPNAM[2]	SMSQ10.100
GPZ2	20.10 %
P16	1000.00 usec

F1 - Acquisition parameters

TD	256
SFO1	100.6152 MHz
FIDRES	130.208328 Hz
SW	165.648 ppm
FnMODE	Echo-Antiecho

F2 - Processing parameters

SI	1024
SF	400.1100072 MHz
WDW	QSINE
SSB	2
LB	0 Hz
GB	0
PC	1.40

F1 - Processing parameters

SI	1024
MC2	echo-antiecho
SF	100.6077400 MHz
WDW	QSINE
SSB	2
LB	0 Hz
GB	0

Expanded region of the ^{1}H-^{13}C HSQC NMR spectrum of compound 4

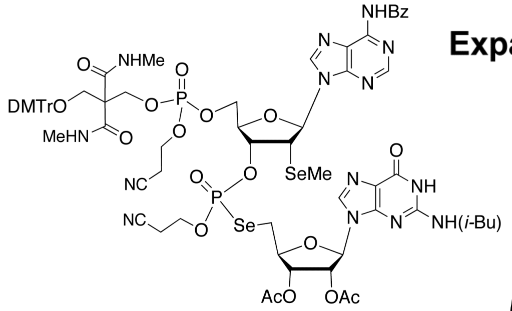

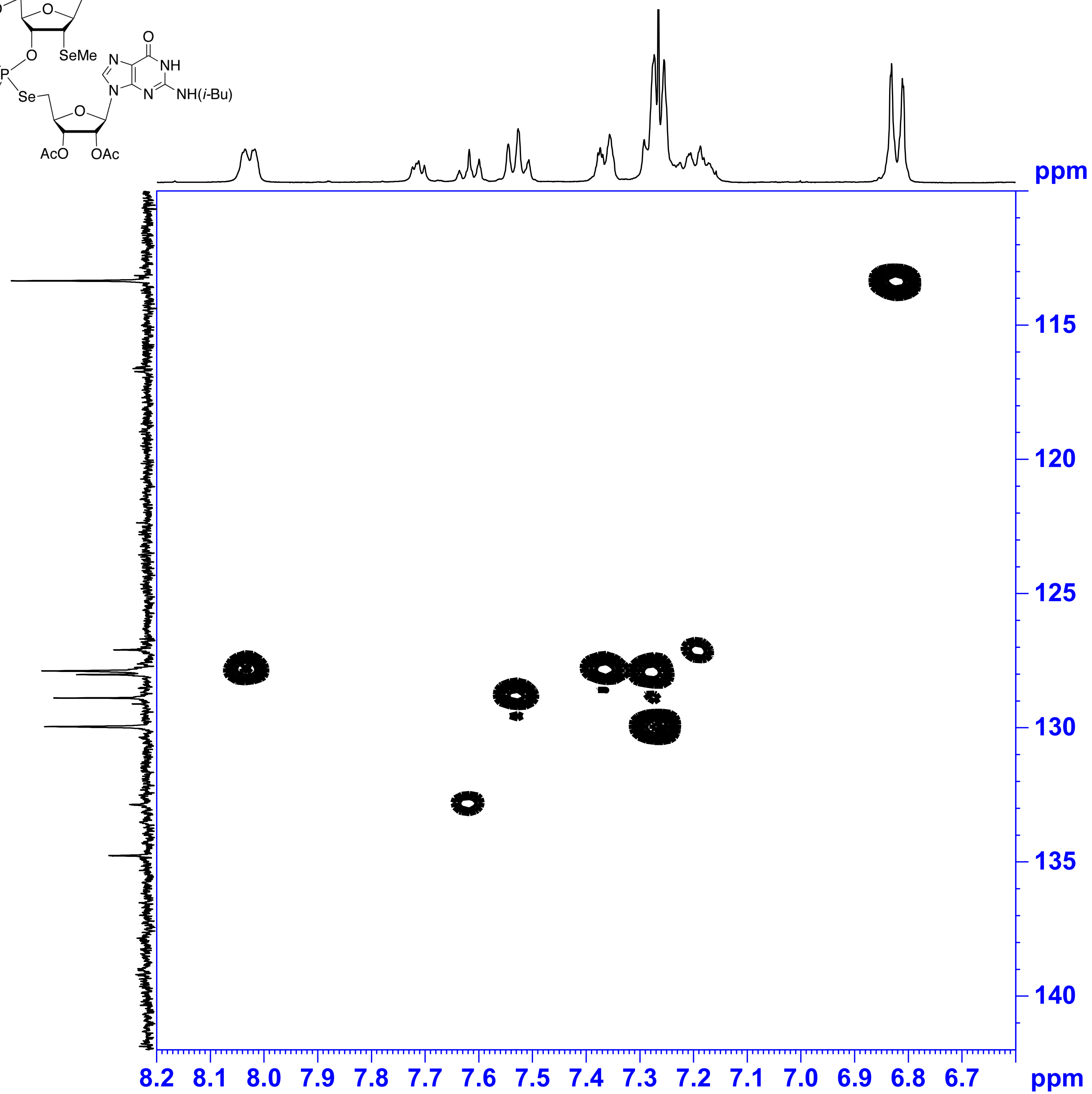


```
Current Data Parameters
NAME      LH-II-80 OLD NMR
EXPNO                   14
PROCNO                   1

F2 - Acquisition Parameters
Date_             20230907
Time                 23.29 h
INSTRUM           AVIII_400
PROBHD     Z108618_0146 (
PULPROG         hsqcedetgp
TD                    1024
SOLVENT              CDCl3
NS                       4
DS                      16
SWH               6097.561 Hz
FIDRES           11.909299 Hz
AQ               0.0839680 sec
RG                    1820
DW                  82.000 usec
DE                    6.50 usec
TE                   300.0 K
CNST2        145.0000000
D0              0.00000300 sec
D1              1.45965397 sec
D4              0.00172414 sec
D11             0.03000000 sec
D16             0.00020000 sec
D21             0.00345000 sec
IN0             0.00003000 sec
TDav                     1
ZGOPTNS
SFO1           400.1124771 MHz
NUC1                    1H
P1                   15.00 usec
P2                   30.00 usec
PLW1           17.29199982 W
SFO2           100.6152434 MHz
NUC2                   13C
CPDPRG[2              garp
P3                    8.70 usec
P4                   17.40 usec
PCPD2                56.50 usec
PLW2           96.68000031 W
PLW12           3.16230011 W
GPNAM[1]        SMSQ10.100
GPZ1                 80.00 %
GPNAM[2]        SMSQ10.100
GPZ2                 20.10 %
P16                1000.00 usec

F1 - Acquisition parameters
TD                     256
SFO1              100.6152 MHz
FIDRES          130.208328 Hz
SW                 165.648 ppm
FnMODE        Echo-Antiecho

F2 - Processing parameters
SI                    1024
SF             400.1100072 MHz
WDW                  QSINE
SSB                      2
LB                       0 Hz
GB                       0
PC                    1.40

F1 - Processing parameters
SI                    1024
MC2           echo-antiecho
SF             100.6077400 MHz
WDW                  QSINE
SSB                      2
LB                       0 Hz
GB                       0
```

^{31}P NMR spectrum of compound 28

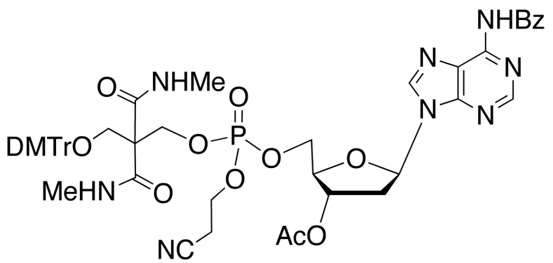

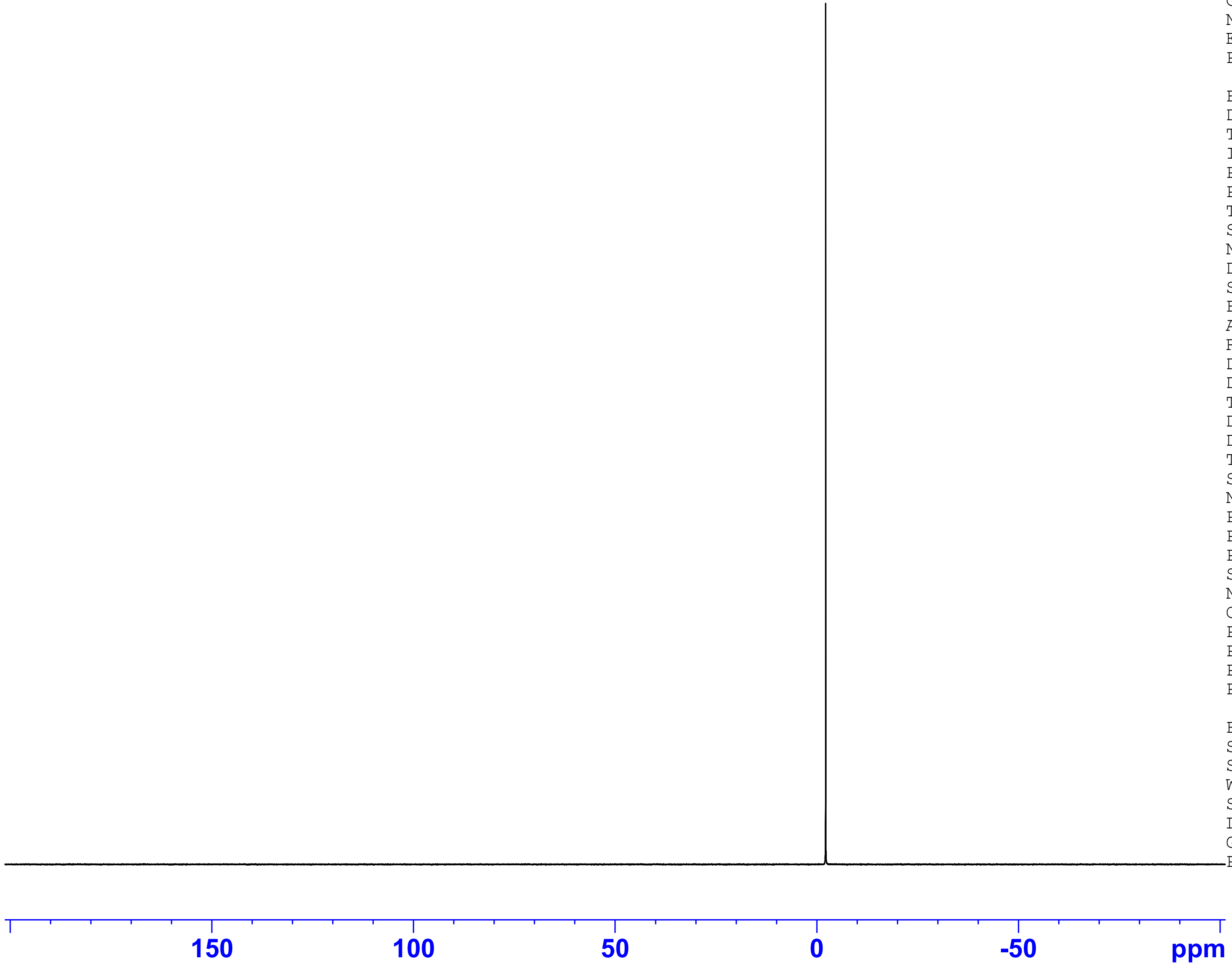

Current Data Parameters
NAME LH-II-79
EXPNO 11
PROCNO 1

F2 - Acquisition Parameters
Date_ 20230920
Time 20.52 h
INSTRUM AVIII_400
PROBHD Z108618_0146 (
PULPROG zgpg30
TD 65536
SOLVENT CDCl3
NS 2000
DS 4
SWH 49019.609 Hz
FIDRES 1.495960 Hz
AQ 0.6684672 sec
RG 2050
DW 10.200 usec
DE 6.50 usec
TE 300.0 K
D1 2.00000000 sec
D11 0.03000000 sec
TD0 1
SFO1 161.9755954 MHz
NUC1 31P
P0 2.80 usec
P1 8.40 usec
PLW1 41.93299866 W
SFO2 400.1116004 MHz
NUC2 1H
CPDPRG[2 waltz16
PCPD2 90.00 usec
PLW2 17.29199982 W
PLW12 0.48032999 W
PLW13 0.24160001 W

F2 - Processing parameters
SI 32768
SF 161.9674970 MHz
WDW EM
SSB 0
LB 2.00 Hz
GB 0
PC 1.40

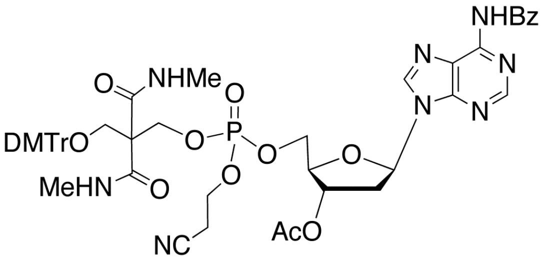

Expanded region of ^{31}P NMR spectrum of compound 28

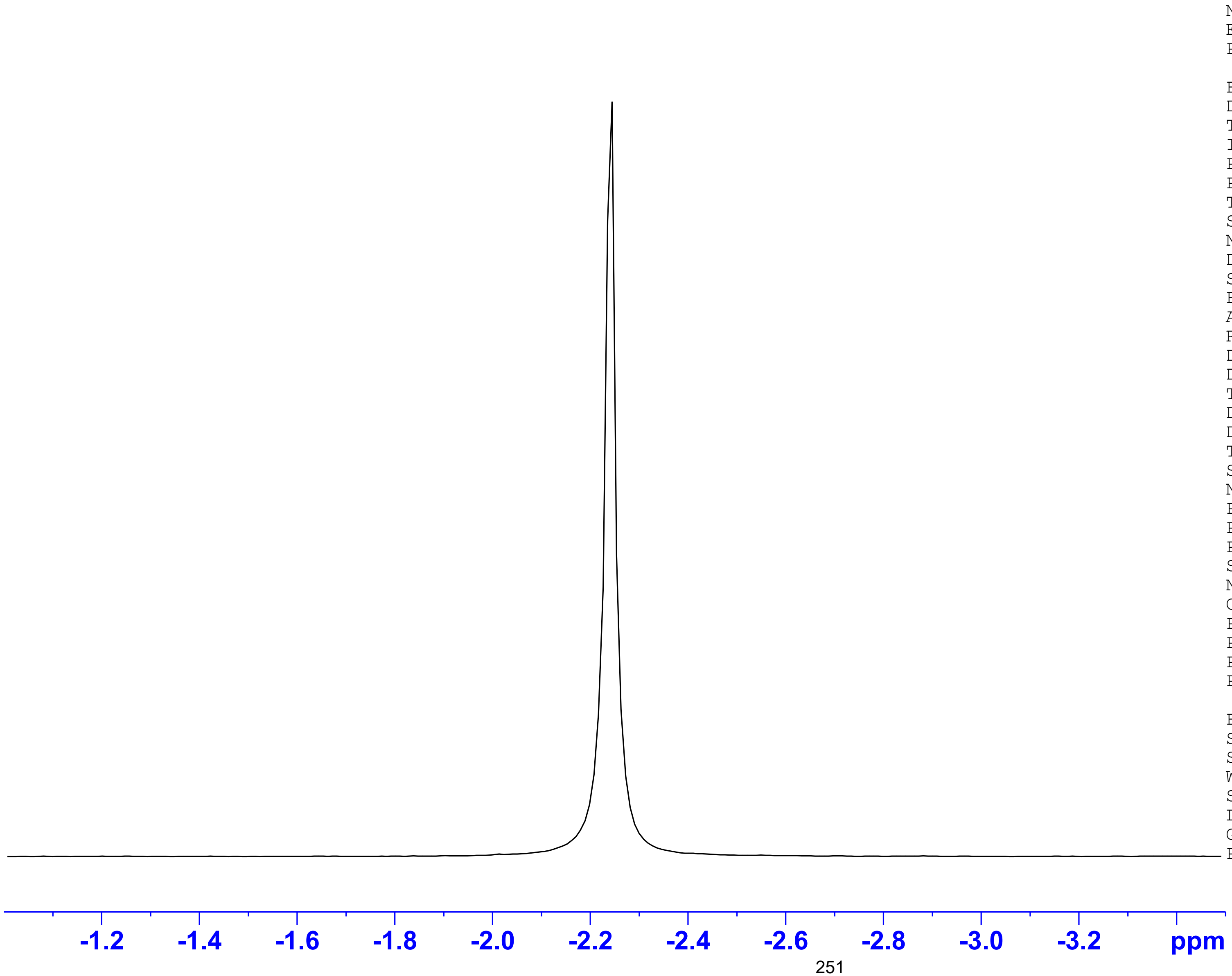

BRUKER

Current Data Parameters

NAME	LH-II-79
EXPNO	11
PROCNO	1

F2 - Acquisition Parameters

Date_	20230920
Time	20.52 h
INSTRUM	AVIII_400
PROBHD	Z108618_0146 (
PULPROG	zgpg30
TD	65536
SOLVENT	CDCl3
NS	2000
DS	4
SWH	49019.609 Hz
FIDRES	1.495960 Hz
AQ	0.6684672 sec
RG	2050
DW	10.200 usec
DE	6.50 usec
TE	300.0 K
D1	2.00000000 sec
D11	0.03000000 sec
TD0	1
SFO1	161.9755954 MHz
NUC1	31P
P0	2.80 usec
P1	8.40 usec
PLW1	41.93299866 W
SFO2	400.1116004 MHz
NUC2	1H
CPDPRG[2	waltz16
PCPD2	90.00 usec
PLW2	17.29199982 W
PLW12	0.48032999 W
PLW13	0.24160001 W

F2 - Processing parameters

SI	32768
SF	161.9674970 MHz
WDW	EM
SSB	0
LB	2.00 Hz
GB	0
PC	1.40

^{1}H NMR spectrum of compound 28

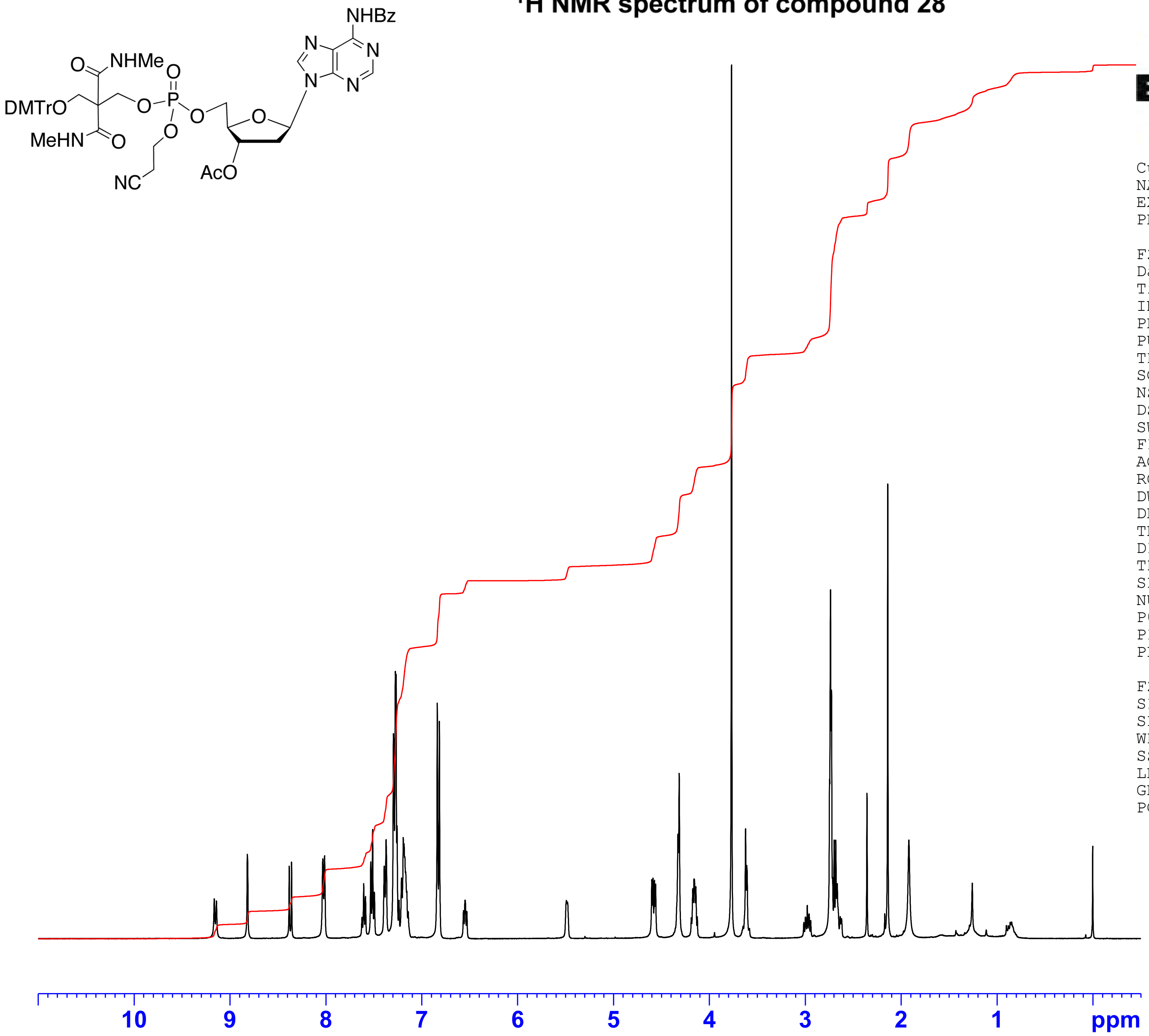


```
Current Data Parameters
NAME             LH-II-79
EXPNO                  10
PROCNO                  1

F2 - Acquisition Parameters
Date_            20230920
Time                19.18 h
INSTRUM          AVIII_400
PROBHD   Z108618_0146 (
PULPROG              zg30
TD                  65536
SOLVENT             CDCl3
NS                     32
DS                      2
SWH              8223.685 Hz
FIDRES           0.250967 Hz
AQ              3.9845889 sec
RG                    161
DW                 60.800 usec
DE                  17.42 usec
TE                  300.0 K
D1             1.00000000 sec
TD0                     1
SFO1          400.1124708 MHz
NUC1                   1H
P0                   5.00 usec
P1                  15.00 usec
PLW1          17.29199982 W

F2 - Processing parameters
SI                  32768
SF            400.1100073 MHz
WDW                    EM
SSB                     0
LB                   0.30 Hz
GB                      0
PC                   1.00
```

Expanded region of the ^{1}H NMR spectrum of compound 28

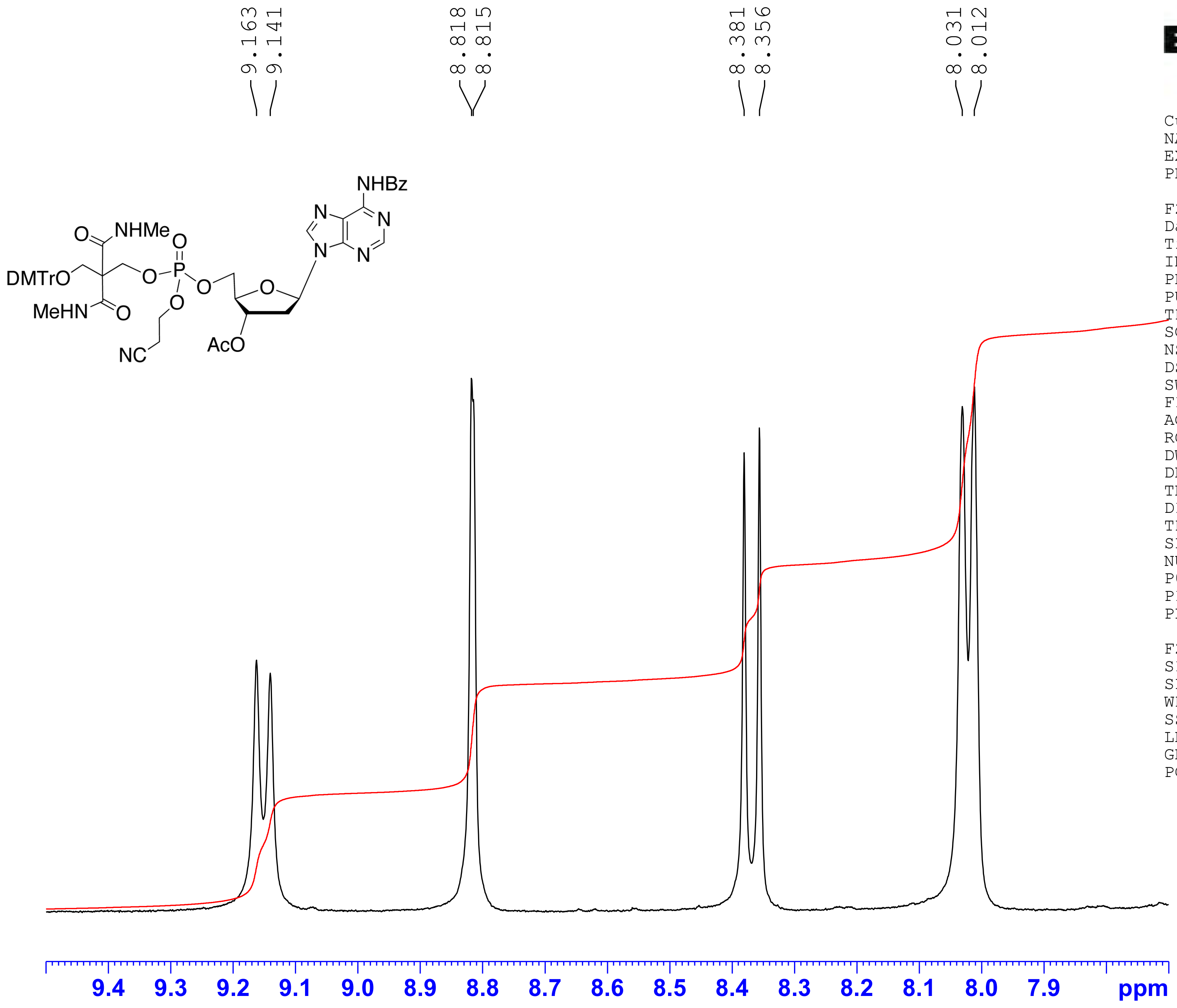


```
Current Data Parameters
NAME             LH-II-79
EXPNO                  10
PROCNO                  1

F2 - Acquisition Parameters
Date_            20230920
Time                19.18 h
INSTRUM         AVIII_400
PROBHD   Z108618_0146 (
PULPROG              zg30
TD                  65536
SOLVENT             CDCl3
NS                     32
DS                      2
SWH              8223.685 Hz
FIDRES           0.250967 Hz
AQ              3.9845889 sec
RG                    161
DW                 60.800 usec
DE                  17.42 usec
TE                  300.0 K
D1             1.00000000 sec
TD0                     1
SFO1          400.1124708 MHz
NUC1                   1H
P0                   5.00 usec
P1                  15.00 usec
PLW1          17.29199982 W

F2 - Processing parameters
SI                  32768
SF            400.1100073 MHz
WDW                    EM
SSB                     0
LB                   0.30 Hz
GB                      0
PC                   1.00
```

Expanded region of the ^{1}H NMR spectrum of compound 28

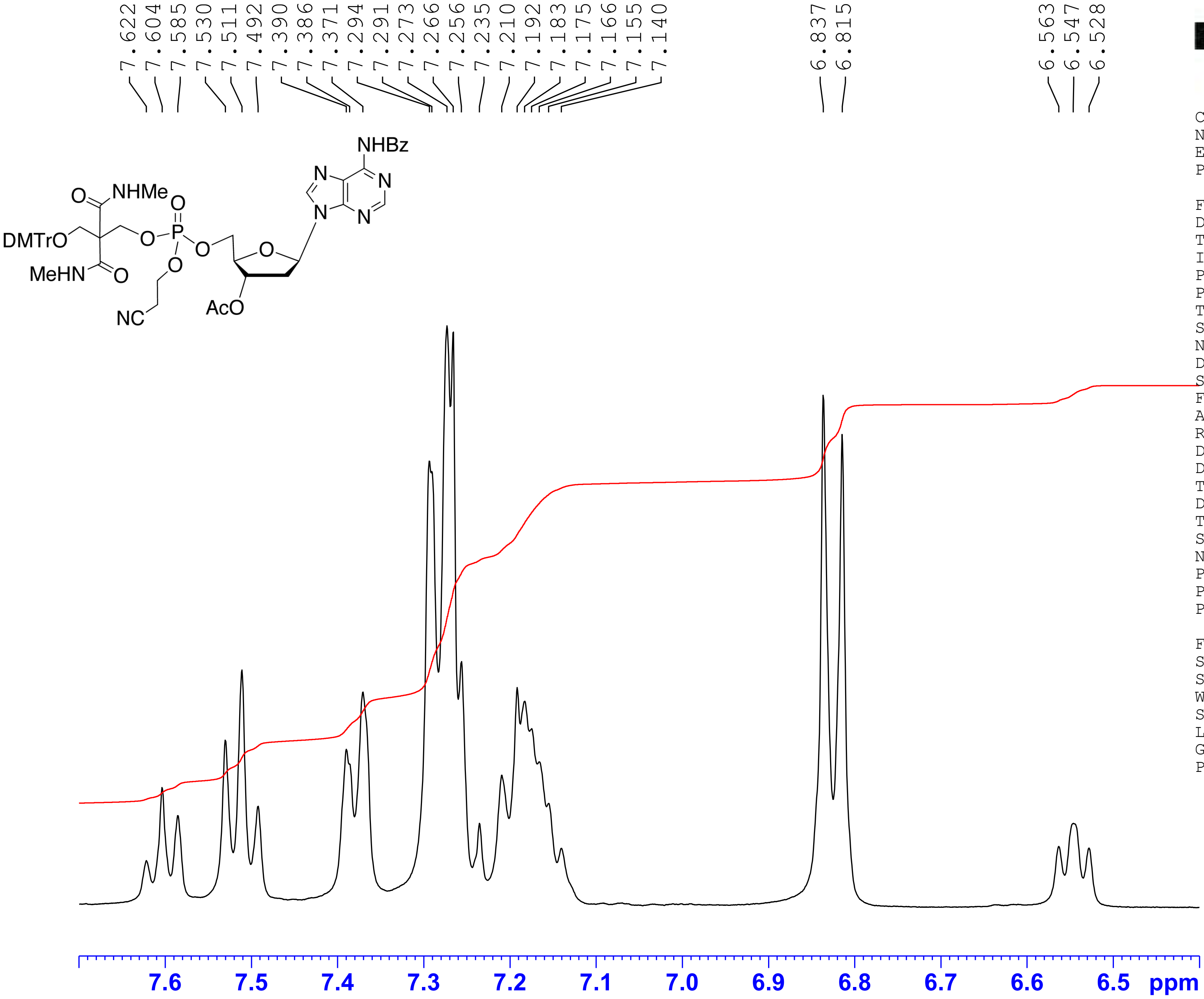

Current Data Parameters
NAME LH-II-79
EXPNO 10
PROCNO 1

F2 - Acquisition Parameters
Date_ 20230920
Time 19.18 h
INSTRUM AVIII_400
PROBHD Z108618_0146 (
PULPROG zg30
TD 65536
SOLVENT CDCl3
NS 32
DS 2
SWH 8223.685 Hz
FIDRES 0.250967 Hz
AQ 3.9845889 sec
RG 161
DW 60.800 usec
DE 17.42 usec
TE 300.0 K
D1 1.00000000 sec
TD0 1
SFO1 400.1124708 MHz
NUC1 1H
P0 5.00 usec
P1 15.00 usec
PLW1 17.29199982 W

F2 - Processing parameters
SI 32768
SF 400.1100073 MHz
WDW EM
SSB 0
LB 0.30 Hz
GB 0
PC 1.00

Expanded region of the ^{1}H NMR spectrum of compound 28

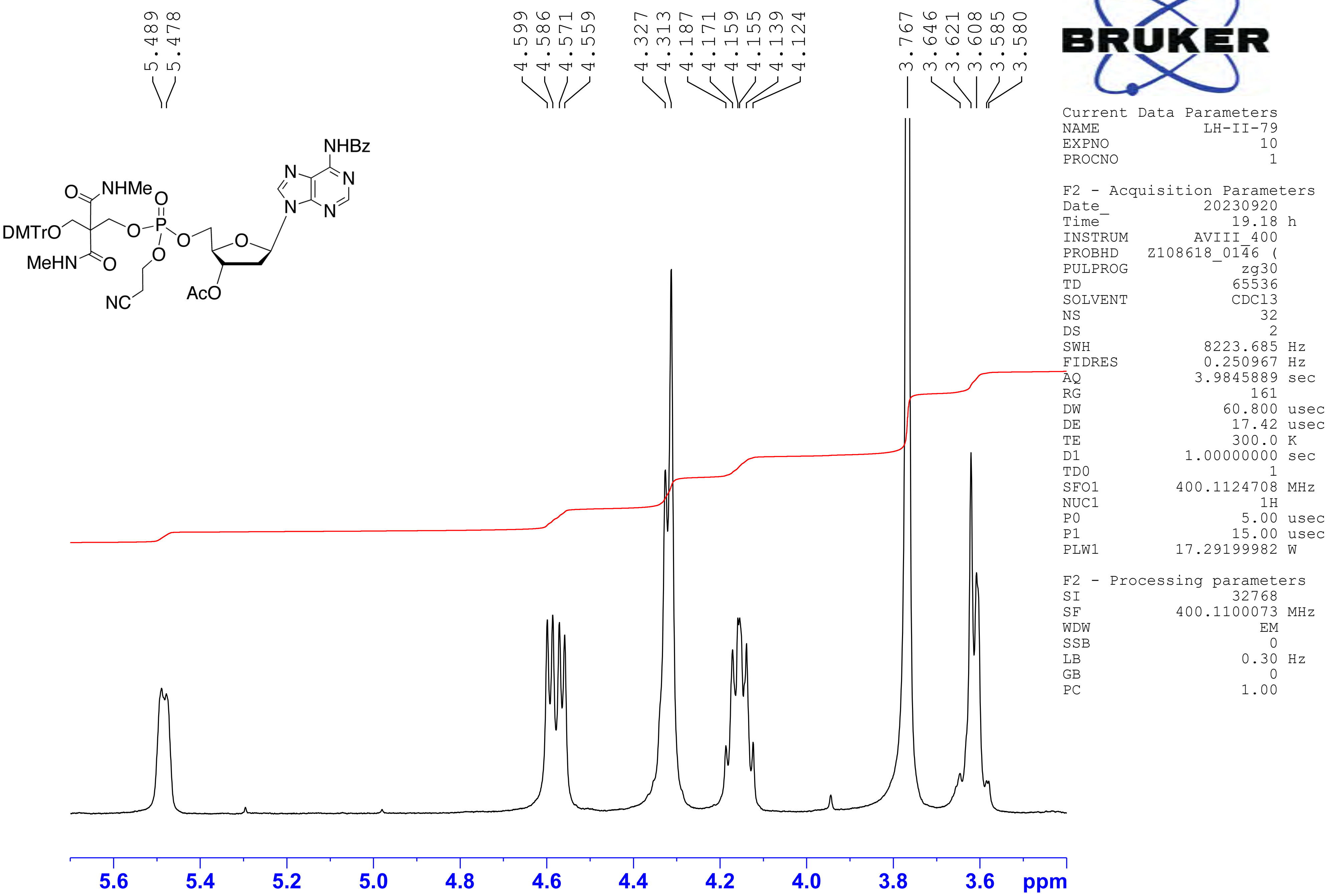

Expanded region of the ^{1}H NMR spectrum of compound 28

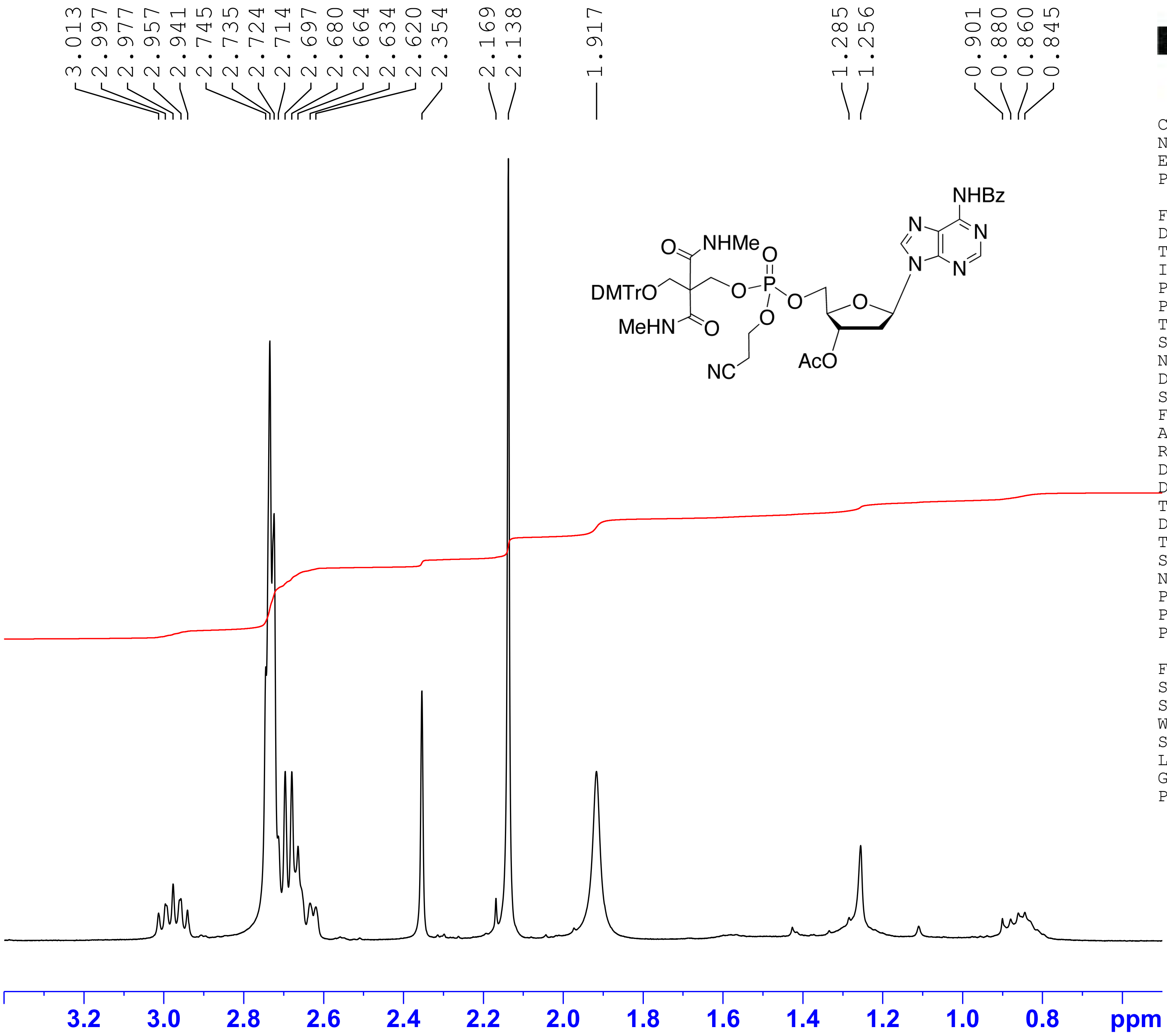


```
Current Data Parameters
NAME                LH-II-79
EXPNO                     10
PROCNO                     1

F2 - Acquisition Parameters
Date_               20230920
Time                   19.18 h
INSTRUM            AVIII_400
PROBHD   Z108618_0146 (
PULPROG                 zg30
TD                     65536
SOLVENT                CDCl3
NS                        32
DS                         2
SWH                 8223.685 Hz
FIDRES              0.250967 Hz
AQ                 3.9845889 sec
RG                       161
DW                    60.800 usec
DE                     17.42 usec
TE                     300.0 K
D1                1.00000000 sec
TD0                        1
SFO1             400.1124708 MHz
NUC1                      1H
P0                      5.00 usec
P1                     15.00 usec
PLW1             17.29199982 W

F2 - Processing parameters
SI                     32768
SF               400.1100073 MHz
WDW                       EM
SSB                        0
LB                      0.30 Hz
GB                         0
PC                      1.00
```

^{1}H NMR spectrum of compound 29

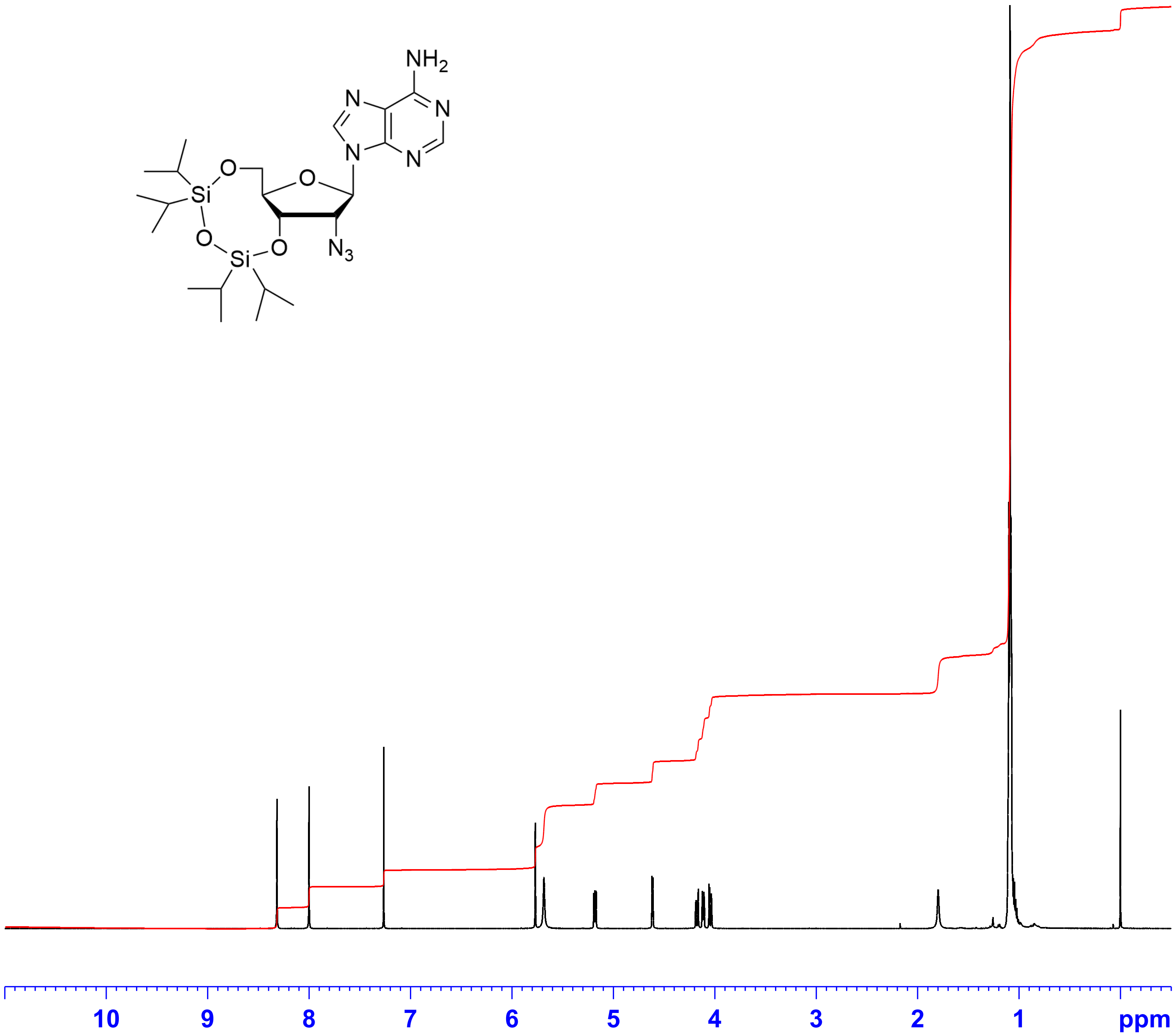

Current Data Parameters
NAME LH-I-61
EXPNO 10
PROCNO 1

F2 - Acquisition Parameters
Date_ 20220214
Time 15.37 h
INSTRUM spect
PROBHD Z114607_0188 (
PULPROG zg30
TD 180286
SOLVENT CDCl3
NS 16
DS 0
SWH 18028.846 Hz
FIDRES 0.200003 Hz
AQ 4.9999318 sec
RG 97.5
DW 27.733 usec
DE 8.00 usec
TE 300.0 K
D1 0.10000000 sec
TD0 1
SFO1 600.1337060 MHz
NUC1 1H
P0 3.33 usec
P1 10.00 usec
PLW1 26.60000038 W

F2 - Processing parameters
SI 262144
SF 600.1300121 MHz
WDW EM
SSB 0
LB 0.10 Hz
GB 0
PC 1.00

Expanded region of the ^{1}H NMR spectrum of compound 29

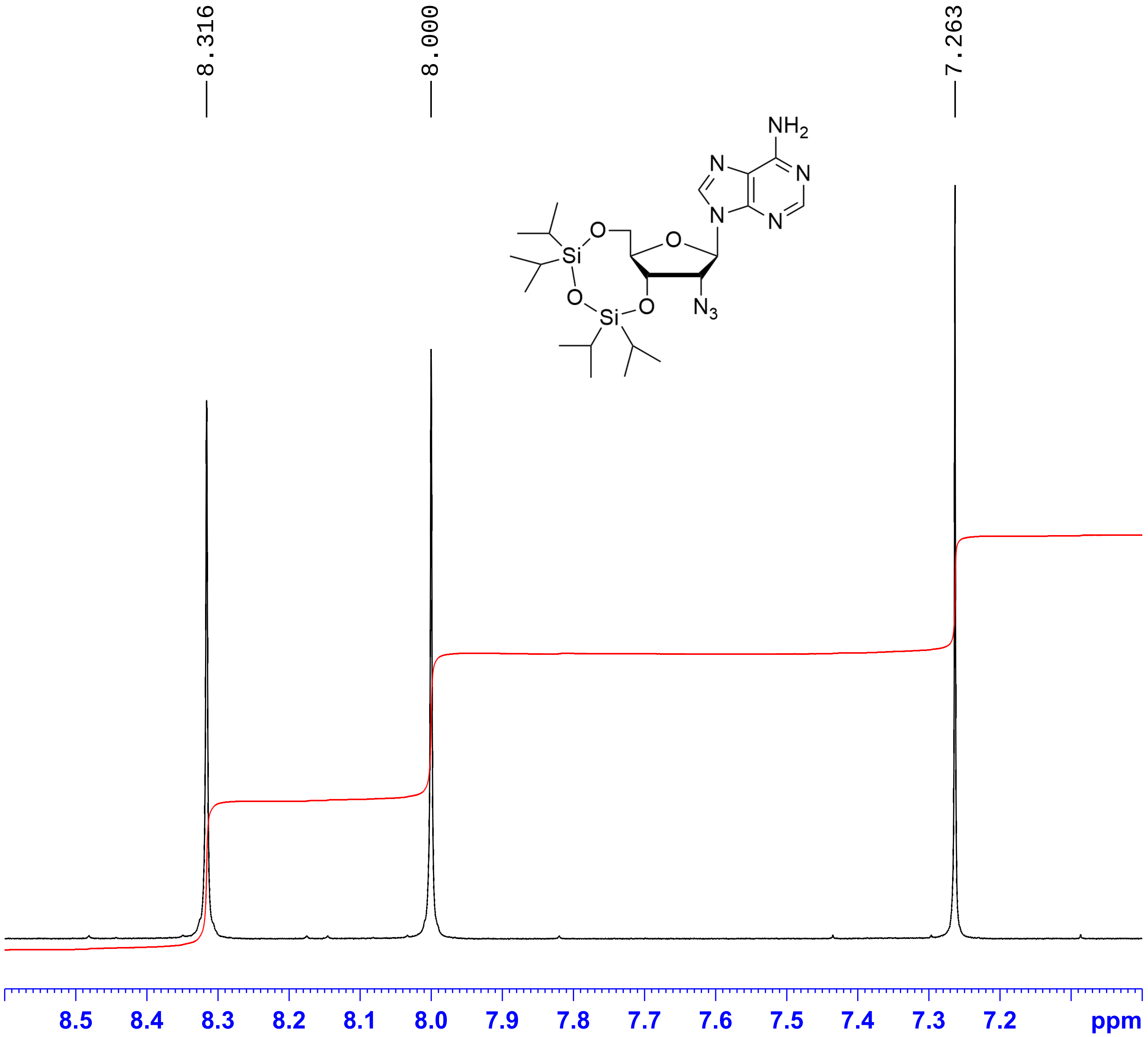


```
Current Data Parameters
NAME              LH-I-61
EXPNO                  10
PROCNO                  1

F2 - Acquisition Parameters
Date_            20220214
Time                15.37 h
INSTRUM             spect
PROBHD     Z114607_0188 (
PULPROG              zg30
TD                 180286
SOLVENT             CDCl3
NS                     16
DS                      0
SWH             18028.846 Hz
FIDRES           0.200003 Hz
AQ              4.9999318 sec
RG                   97.5
DW                 27.733 usec
DE                   8.00 usec
TE                  300.0 K
D1             0.10000000 sec
TD0                     1
SFO1          600.1337060 MHz
NUC1                   1H
P0                   3.33 usec
P1                  10.00 usec
PLW1          26.60000038 W

F2 - Processing parameters
SI                 262144
SF            600.1300121 MHz
WDW                    EM
SSB       0
LB                   0.10 Hz
GB        0
PC                   1.00
```

Expanded region of the ^{1}H NMR spectrum of compound 29

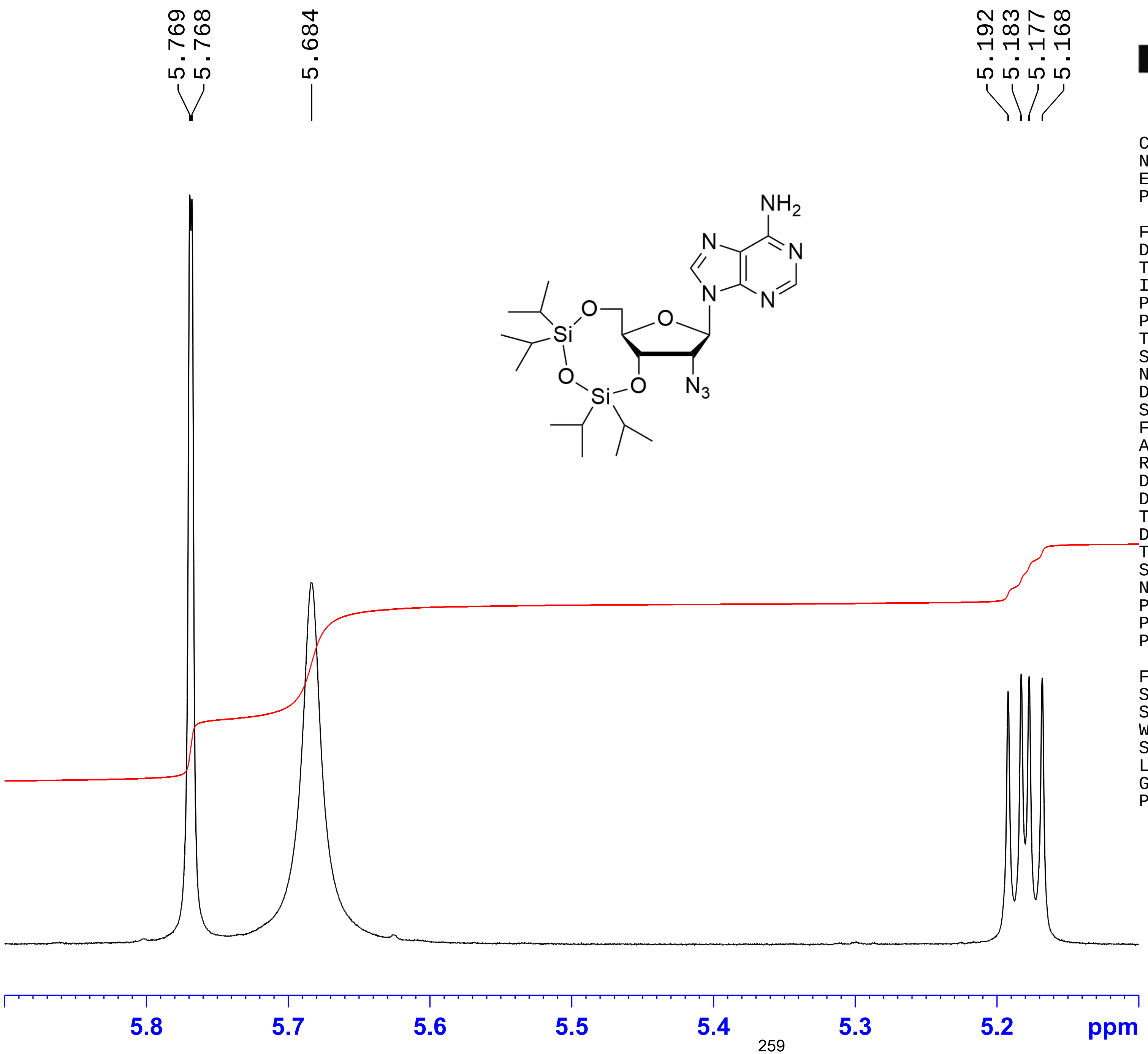


```
Current Data Parameters
NAME              LH-I-61
EXPNO                  10
PROCNO                  1

F2 - Acquisition Parameters
Date_            20220214
Time                15.37 h
INSTRUM             spect
PROBHD   Z114607_0188 (
PULPROG              zg30
TD                 180286
SOLVENT             CDCl3
NS                     16
DS                      0
SWH             18028.846 Hz
FIDRES           0.200003 Hz
AQ              4.9999318 sec
RG                   97.5
DW                 27.733 usec
DE                   8.00 usec
TE                  300.0 K
D1             0.10000000 sec
TD0                     1
SFO1          600.1337060 MHz
NUC1                   1H
P0                   3.33 usec
P1                  10.00 usec
PLW1          26.60000038 W

F2 - Processing parameters
SI                 262144
SF            600.1300121 MHz
WDW                    EM
SSB      0
LB                   0.10 Hz
GB       0
PC                   1.00
```

Expanded region of the ^{1}H NMR spectrum of compound 29

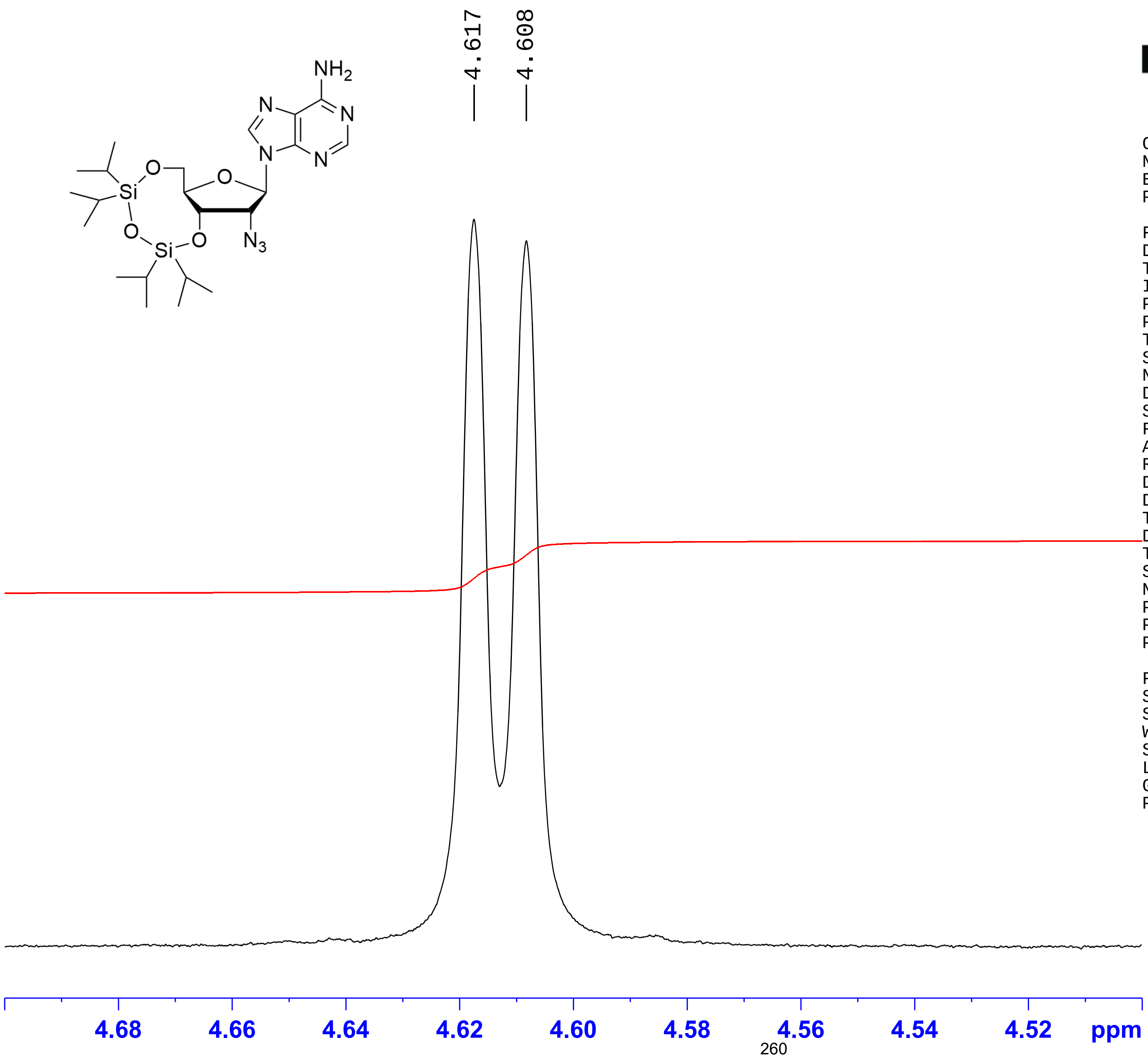

Current Data Parameters

NAME	LH-I-61
EXPNO	10
PROCNO	1

F2 - Acquisition Parameters

Date_	20220214
Time	15.37 h
INSTRUM	spect
PROBHD	Z114607_0188 (
PULPROG	zg30
TD	180286
SOLVENT	CDCl3
NS	16
DS	0
SWH	18028.846 Hz
FIDRES	0.200003 Hz
AQ	4.9999318 sec
RG	97.5
DW	27.733 usec
DE	8.00 usec
TE	300.0 K
D1	0.10000000 sec
TD0	1
SFO1	600.1337060 MHz
NUC1	1H
P0	3.33 usec
P1	10.00 usec
PLW1	26.60000038 W

F2 - Processing parameters

SI	262144
SF	600.1300121 MHz
WDW	EM
SSB	0
LB	0.10 Hz
GB	0
PC	1.00

Expanded region of the ^{1}H NMR spectrum of compound 29

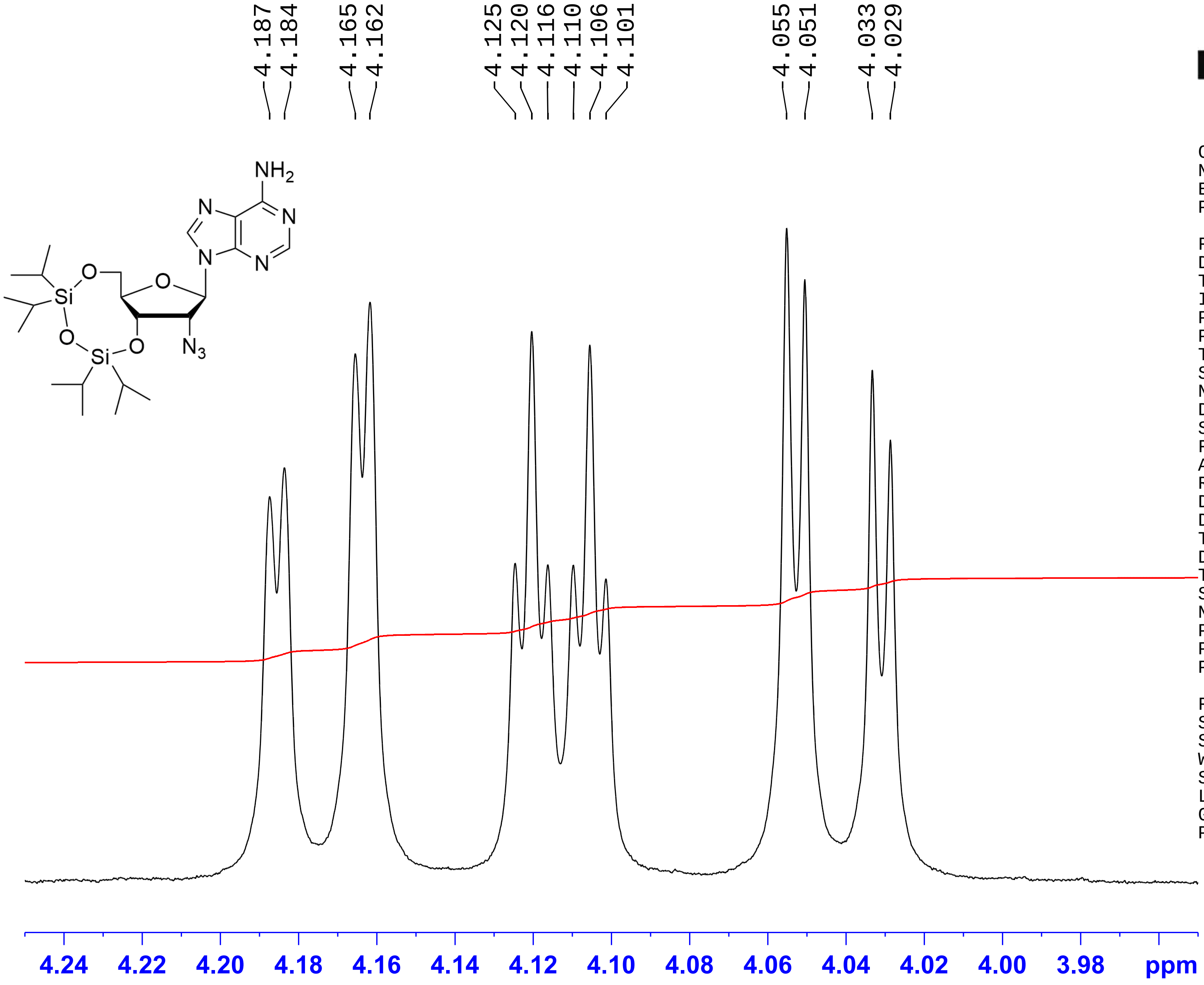

BRUKER

```
Current Data Parameters
NAME              LH-I-61
EXPNO                  10
PROCNO                  1

F2 - Acquisition Parameters
Date_            20220214
Time                15.37 h
INSTRUM             spect
PROBHD    Z114607_0188 (
PULPROG              zg30
TD                 180286
SOLVENT             CDCl3
NS                     16
DS                      0
SWH             18028.846 Hz
FIDRES           0.200003 Hz
AQ              4.9999318 sec
RG                   97.5
DW                 27.733 usec
DE                   8.00 usec
TE                  300.0 K
D1             0.10000000 sec
TD0                     1
SFO1          600.1337060 MHz
NUC1                   1H
P0                   3.33 usec
P1                  10.00 usec
PLW1          26.60000038 W

F2 - Processing parameters
SI                 262144
SF            600.1300121 MHz
WDW                    EM
SSB       0
LB                   0.10 Hz
GB        0
PC                   1.00
```

^{13}C NMR spectrum of compound 29

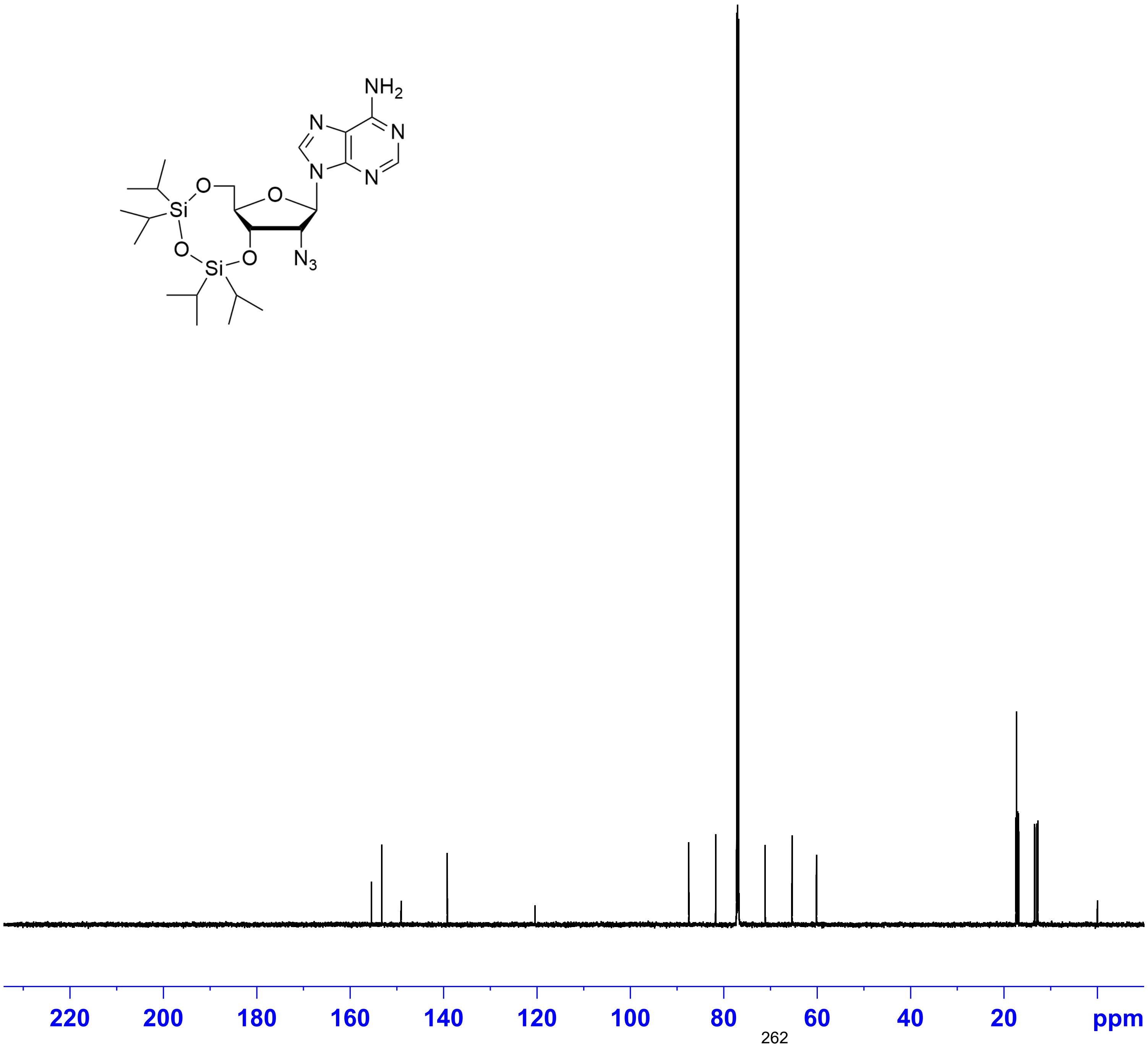


```
Current Data Parameters
NAME              LH-I-61
EXPNO                  11
PROCNO                  1

F2 - Acquisition Parameters
Date_            20220214
Time                17.07 h
INSTRUM             spect
PROBHD   Z114607_0188 (
PULPROG           zgpg30
TD                119044
SOLVENT            CDCl3
NS                  2000
DS                     4
SWH            37500.000 Hz
FIDRES          0.630019 Hz
AQ             1.5872533 sec
RG                186.92
DW                13.333 usec
DE                  6.53 usec
TE                 300.0 K
D1            1.00000000 sec
D11           0.03000000 sec
TD0                    1
SFO1         150.9194058 MHz
NUC1                 13C
P0                  3.93 usec
P1                 11.80 usec
PLW1         85.00000000 W
SFO2         600.1324005 MHz
NUC2                  1H
CPDPRG[2         waltz64
PCPD2              70.00 usec
PLW2         27.00000000 W
PLW12         0.57327998 W
PLW13         0.28836000 W

F2 - Processing parameters
SI                131072
SF           150.9028113 MHz
WDW                   EM
SSB      0
LB                  1.00 Hz
GB       0
PC                  1.40
```

Expanded region of the ^{13}C NMR spectrum of compound 29

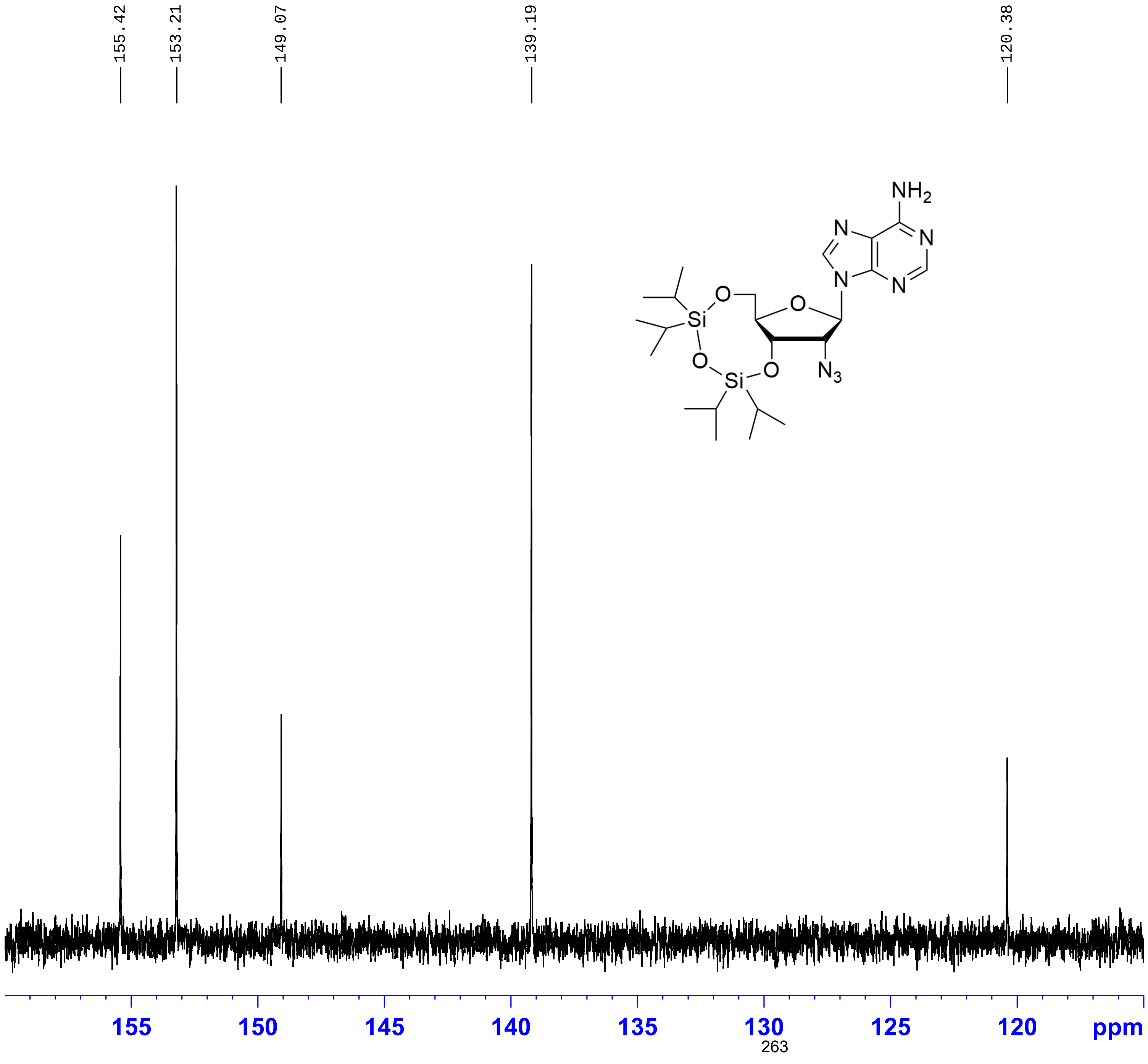

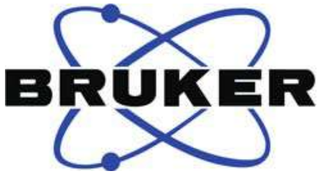

Current Data Parameters
NAME LH-I-61
EXPNO 11
PROCNO 1

F2 - Acquisition Parameters
Date_ 20220214
Time 17.07 h
INSTRUM spect
PROBHD Z114607_0188 (
PULPROG zgpg30
TD 119044
SOLVENT CDCl3
NS 2000
DS 4
SWH 37500.000 Hz
FIDRES 0.630019 Hz
AQ 1.5872533 sec
RG 186.92
DW 13.333 usec
DE 6.53 usec
TE 300.0 K
D1 1.00000000 sec
D11 0.03000000 sec
TD0 1
SFO1 150.9194058 MHz
NUC1 13C
P0 3.93 usec
P1 11.80 usec
PLW1 85.00000000 W
SFO2 600.1324005 MHz
NUC2 1H
CPDPRG[2 waltz64
PCPD2 70.00 usec
PLW2 27.00000000 W
PLW12 0.57327998 W
PLW13 0.28836000 W

F2 - Processing parameters
SI 131072
SF 150.9028113 MHz
WDW EM
SSB 0
LB 1.00 Hz
GB 0
PC 1.40

Expanded region of the ^{13}C NMR spectrum of compound 29

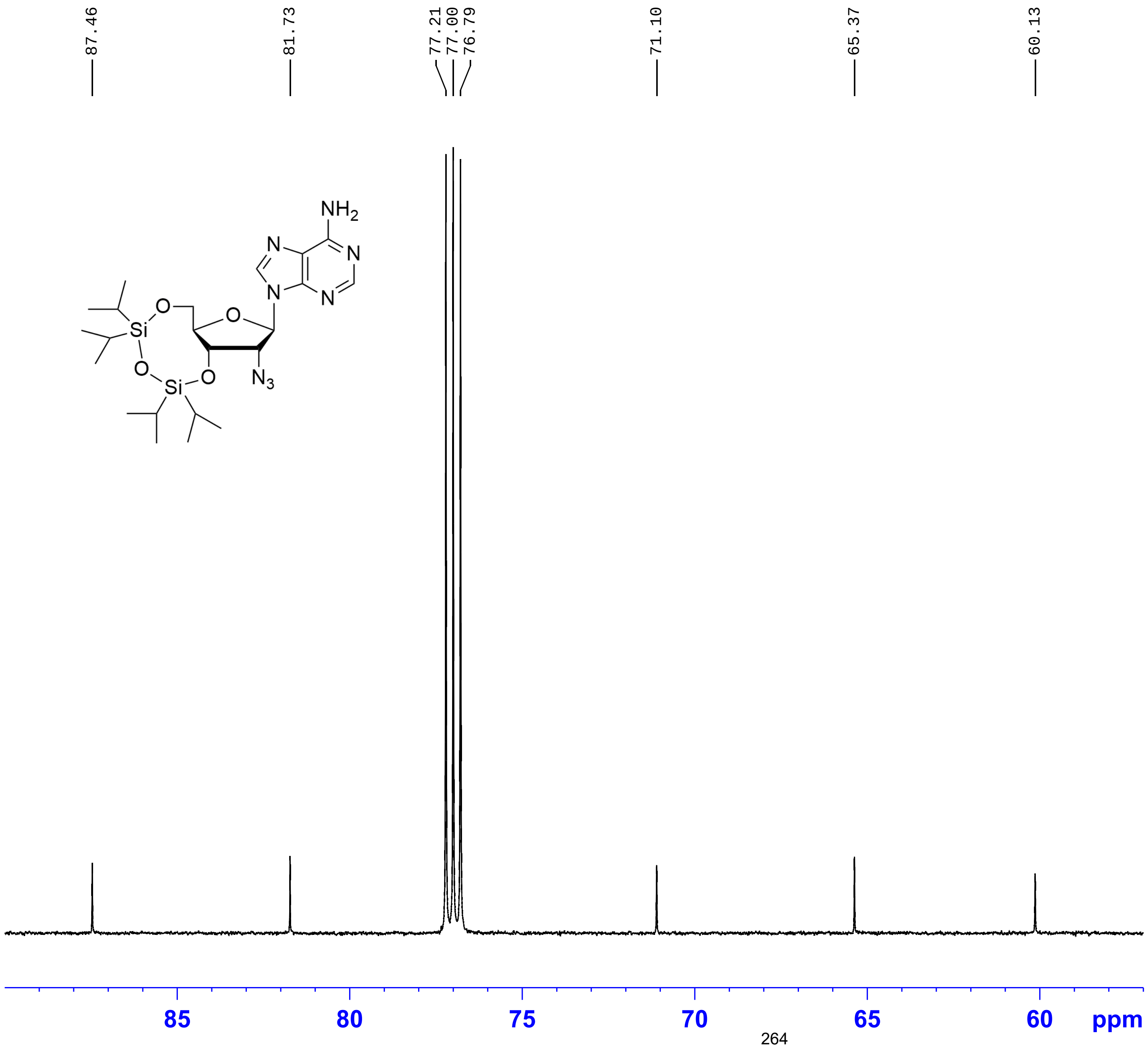


```
Current Data Parameters
NAME              LH-I-61
EXPNO                  11
PROCNO                  1

F2 - Acquisition Parameters
Date_            20220214
Time                17.07 h
INSTRUM             spect
PROBHD    Z114607_0188 (
PULPROG           zgpg30
TD                119044
SOLVENT            CDCl3
NS                  2000
DS                     4
SWH            37500.000 Hz
FIDRES          0.630019 Hz
AQ             1.5872533 sec
RG                186.92
DW                13.333 usec
DE                  6.53 usec
TE                 300.0 K
D1            1.00000000 sec
D11           0.03000000 sec
TD0                    1
SFO1         150.9194058 MHz
NUC1                 13C
P0                  3.93 usec
P1                 11.80 usec
PLW1         85.00000000 W
SFO2         600.1324005 MHz
NUC2                  1H
CPDPRG[2         waltz64
PCPD2              70.00 usec
PLW2         27.00000000 W
PLW12         0.57327998 W
PLW13         0.28836000 W

F2 - Processing parameters
SI                131072
SF           150.9028113 MHz
WDW                   EM
SSB       0
LB                  1.00 Hz
GB        0
PC                  1.40
```

Expanded region of the ^{13}C NMR spectrum of compound 29

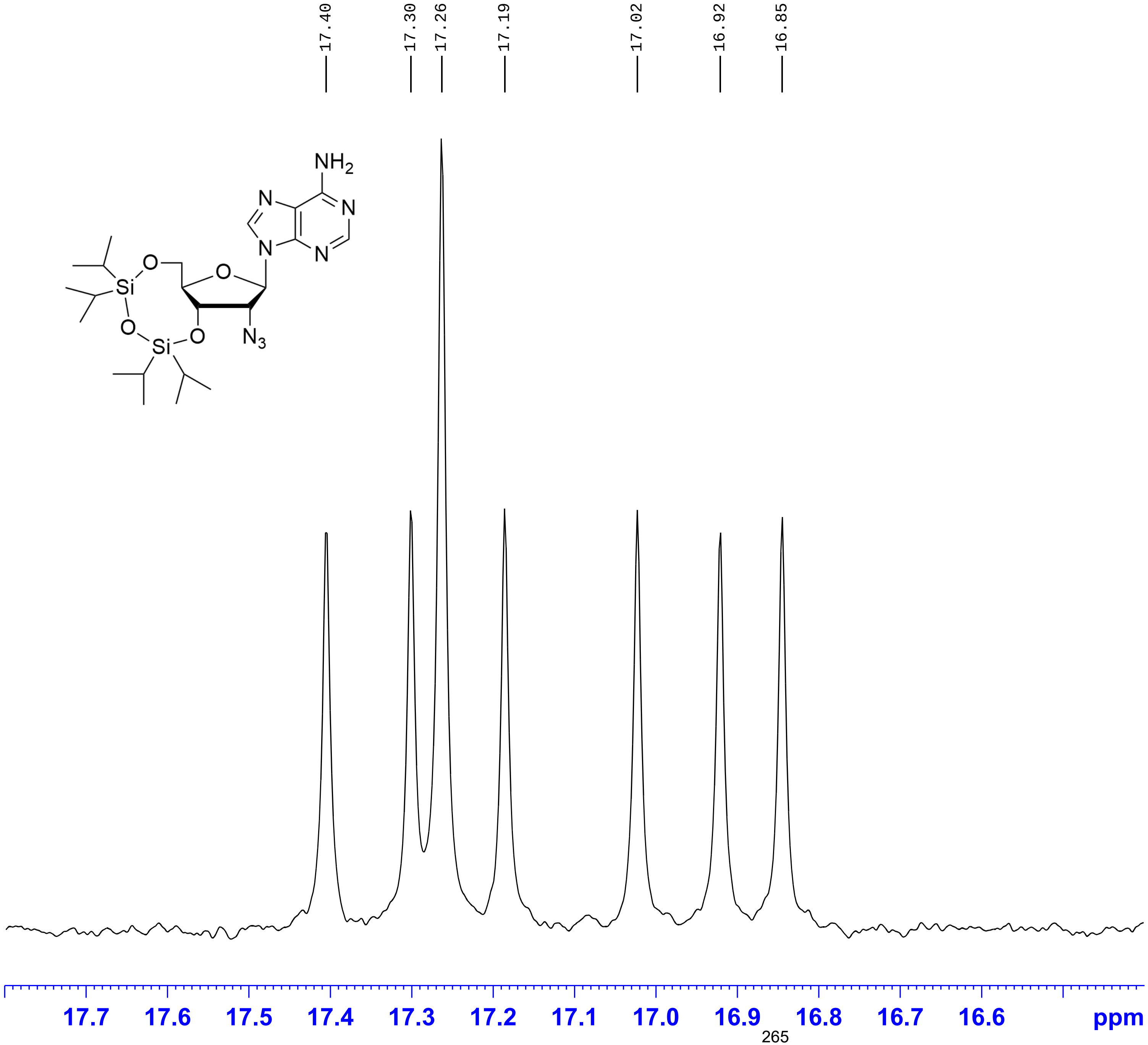


```
Current Data Parameters
NAME              LH-I-61
EXPNO                  11
PROCNO                  1

F2 - Acquisition Parameters
Date_            20220214
Time                17.07 h
INSTRUM             spect
PROBHD     Z114607_0188 (
PULPROG            zgpg30
TD                 119044
SOLVENT             CDCl3
NS                   2000
DS                      4
SWH             37500.000 Hz
FIDRES           0.630019 Hz
AQ              1.5872533 sec
RG                 186.92
DW                 13.333 usec
DE                   6.53 usec
TE                  300.0 K
D1             1.00000000 sec
D11            0.03000000 sec
TD0                     1
SFO1          150.9194058 MHz
NUC1                  13C
P0                   3.93 usec
P1                  11.80 usec
PLW1          85.00000000 W
SFO2          600.1324005 MHz
NUC2                   1H
CPDPRG[2          waltz64
PCPD2               70.00 usec
PLW2          27.00000000 W
PLW12          0.57327998 W
PLW13          0.28836000 W

F2 - Processing parameters
SI                 131072
SF            150.9028113 MHz
WDW                    EM
SSB      0
LB                   1.00 Hz
GB       0
PC                   1.40
```

Expanded region of the ^{13}C NMR spectrum of compound 29

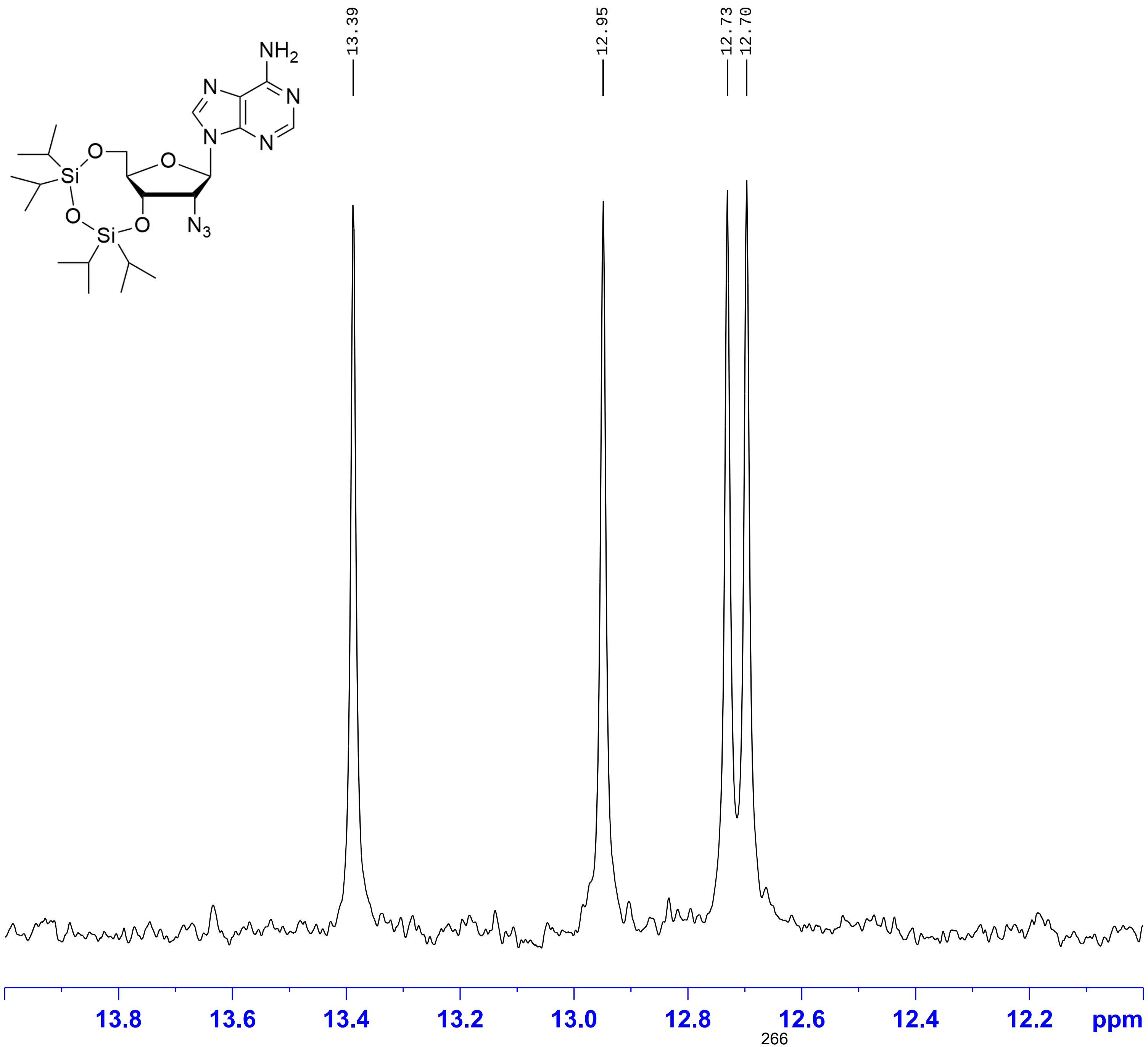


```
Current Data Parameters
NAME              LH-I-61
EXPNO                  11
PROCNO                  1

F2 - Acquisition Parameters
Date_            20220214
Time                17.07 h
INSTRUM             spect
PROBHD     Z114607_0188 (
PULPROG           zgpg30
TD                 119044
SOLVENT             CDCl3
NS                   2000
DS                      4
SWH             37500.000 Hz
FIDRES           0.630019 Hz
AQ              1.5872533 sec
RG                 186.92
DW                 13.333 usec
DE                   6.53 usec
TE                  300.0 K
D1             1.00000000 sec
D11            0.03000000 sec
TD0                     1
SFO1          150.9194058 MHz
NUC1                  13C
P0                   3.93 usec
P1                  11.80 usec
PLW1          85.00000000 W
SFO2          600.1324005 MHz
NUC2                   1H
CPDPRG[2          waltz64
PCPD2               70.00 usec
PLW2          27.00000000 W
PLW12          0.57327998 W
PLW13          0.28836000 W

F2 - Processing parameters
SI                 131072
SF            150.9028113 MHz
WDW                    EM
SSB      0
LB                   1.00 Hz
GB       0
PC                   1.40
```

^{13}C DEPT-135 NMR spectrum of compound 29

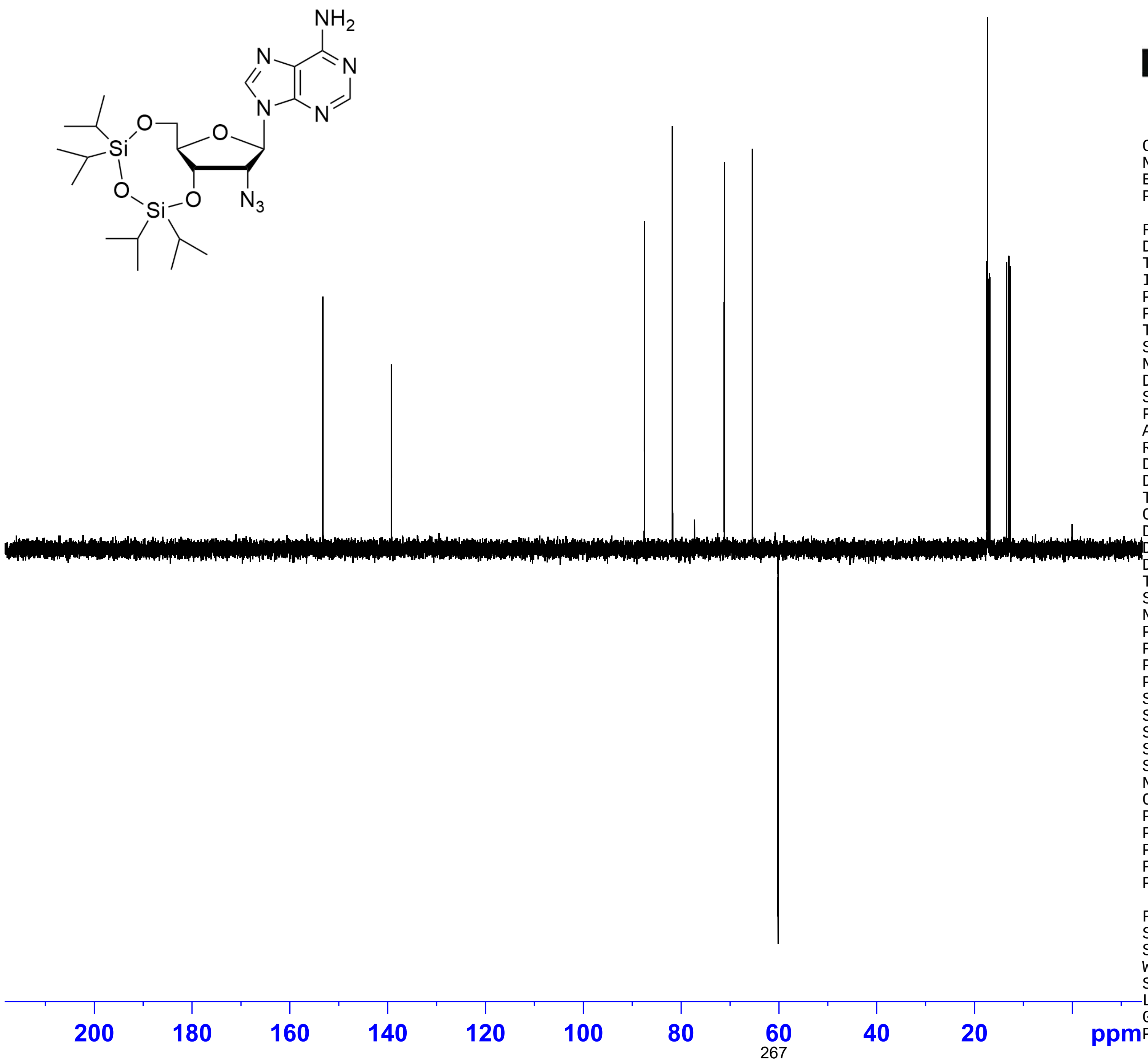

Current Data Parameters
NAME LH-I-61
EXPNO 12
PROCNO 1

F2 - Acquisition Parameters
Date_ 20220214
Time 17.52 h
INSTRUM spect
PROBHD Z114607_0188 (
PULPROG deptsp135.b
TD 119044
SOLVENT CDCl3
NS 1000
DS 4
SWH 35714.285 Hz
FIDRES 0.600018 Hz
AQ 1.6666160 sec
RG 186.92
DW 14.000 usec
DE 7.44 usec
TE 300.0 K
CNST2 145.0000000
D1 1.00000000 sec
D2 0.00344828 sec
D12 0.00002000 sec
TD0 1
SFO1 150.9178962 MHz
NUC1 13C
P1 11.80 usec
P13 2000.00 usec
PLW0 0 W
PLW1 85.00000000 W
SPNAM[5] Crp60comp.4
SPOAL5 0.500
SPOFFS5 0 Hz
SPW5 18.08300018 W
SFO2 600.1324005 MHz
NUC2 1H
CPDPRG[2 waltz64
P3 10.20 usec
P4 20.40 usec
PCPD2 70.00 usec
PLW2 27.00000000 W
PLW12 0.57327998 W

F2 - Processing parameters
SI 131072
SF 150.9028085 MHz
WDW EM
SSB 0
LB 1.00 Hz
GB 0
PC 1.40

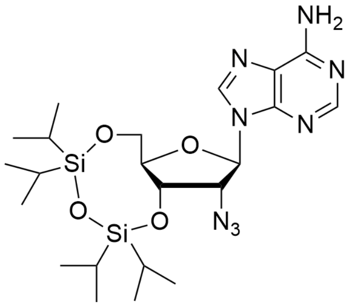

^{1}H-^{1}H COSY NMR spectrum of compound 29

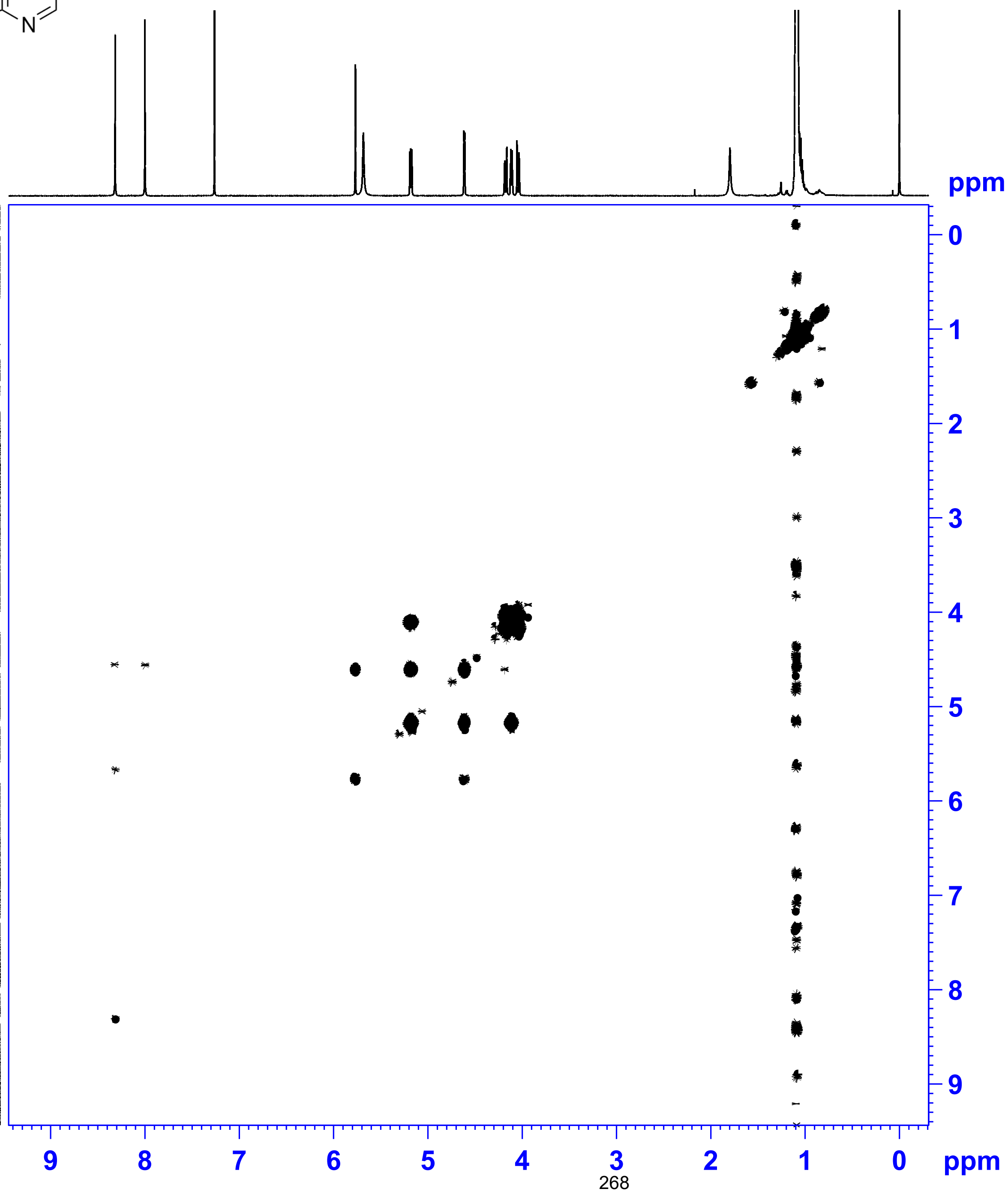


```
Current Data Parameters
NAME               LH-I-61
EXPNO                   13
PROCNO                   1

F2 - Acquisition Parameters
Date_             20220214
Time                 18.03 h
INSTRUM              spect
PROBHD    Z114607_0188 (
PULPROG      cosygpmfppqf
TD                    2048
SOLVENT              CDCl3
NS                       2
DS                       8
SWH               5854.801 Hz
FIDRES            5.717579 Hz
AQ               0.1748992 sec
RG                  186.92
DW                  85.400 usec
DE                    6.50 usec
TE                   300.0 K
D0              0.00000300 sec
D1              0.88531131 sec
D11             0.03000000 sec
D12             0.00002000 sec
D13             0.00000400 sec
D16             0.00020000 sec
IN0             0.00017080 sec
TDav                     1
SFO1           600.1327525 MHz
NUC1                    1H
P1                   10.00 usec
P17                2500.00 usec
PLW1           26.60000038 W
PLW10           4.25600004 W
GPNAM[1]       SMSQ10.100
GPZ1                 16.00 %
GPNAM[2]       SMSQ10.100
GPZ2                 12.00 %
GPNAM[3]       SMSQ10.100
GPZ3                 40.00 %
P16                1000.00 usec

F1 - Acquisition parameters
TD                     256
SFO1              600.1328 MHz
FIDRES           45.740631 Hz
SW                   9.756 ppm
FnMODE                  QF

F2 - Processing parameters
SI                    1024
SF             600.1300112 MHz
WDW                   SINE
SSB      0
LB       0 Hz
GB       0
PC                    1.40

F1 - Processing parameters
SI                    1024
MC2                     QF
SF             600.1300160 MHz
WDW                   SINE
SSB      0
LB       0 Hz
GB       0
```

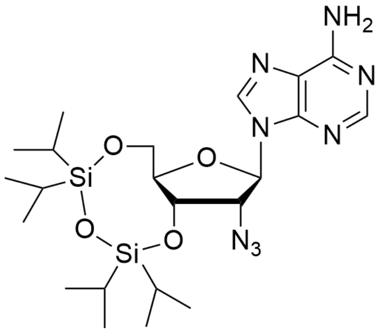

^{1}H-^{13}C HSQC NMR spectrum of compound 29

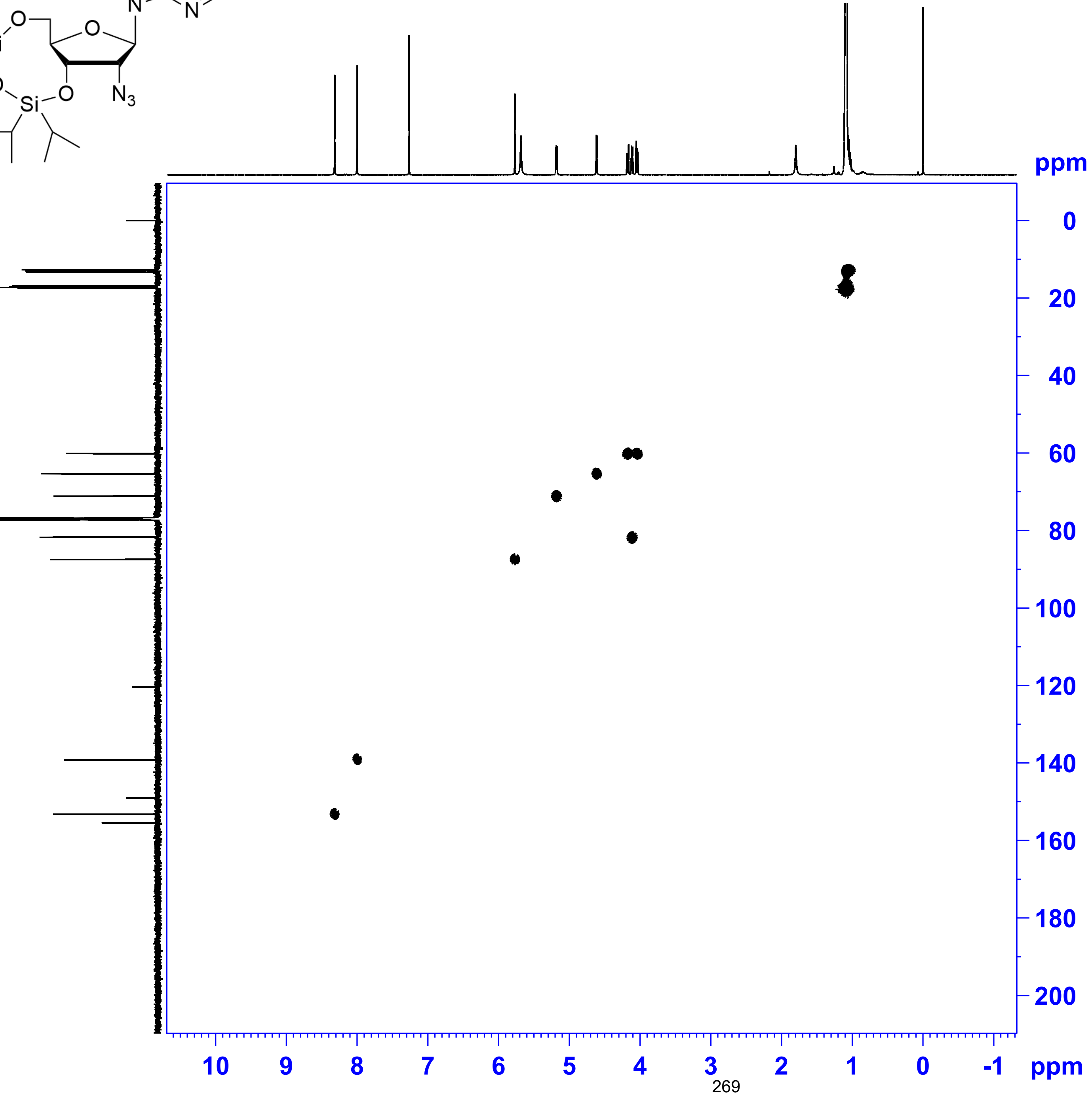

Current Data Parameters

NAME	LH-I-61
EXPNO	14
PROCNO	1

F2 - Acquisition Parameters

Date_	20220214
Time	18.12 h
INSTRUM	spect
PROBHD	Z114607_0188 (
PULPROG	hsqcedetgpsp.3
TD	1024
SOLVENT	CDCl3
NS	2
DS	32
SWH	7211.539 Hz
FIDRES	14.085036 Hz
AQ	0.0709973 sec
RG	186.92
DW	69.333 usec
DE	6.50 usec
TE	300.3 K
CNST2	145.0000000
D0	0.00000300 sec
D1	0.80000001 sec
D4	0.00172414 sec
D11	0.03000000 sec
D16	0.00020000 sec
D21	0.00360000 sec
IN0	0.00001510 sec
TDav	1
ZGOPTNS	
SFO1	600.1328223 MHz
NUC1	1H
P1	10.00 usec
P2	20.00 usec
PLW1	26.60000038 W
SFO2	150.9178988 MHz
NUC2	13C
CPDPRG[2	garp4
P3	11.80 usec
P14	500.00 usec
P31	1730.00 usec
PCPD2	60.00 usec
PLW0	0 W
PLW2	85.00000000 W
PLW12	3.28760004 W
SPNAM[3]	Crp60,0.5,20.1
SPOAL3	0.500
SPOFFS3	0 Hz
SPW3	18.08300018 W
SPNAM[18	Crp60_xfilt.2
SPOAL18	0.500
SPOFFS18	0 Hz
SPW18	5.22629976 W
GPNAM[1]	SMSQ10.100
GPZ1	80.00 %
GPNAM[2]	SMSQ10.100
GPZ2	20.10 %
P16	1000.00 usec

F1 - Acquisition parameters

TD	256
SFO1	150.9179 MHz
FIDRES	258.692047 Hz
SW	219.408 ppm
FnMODE	Echo-Antiecho

F2 - Processing parameters

SI	1024
SF	600.1300119 MHz
WDW	QSINE
SSB	2
LB	0 Hz
GB	0
PC	1.40

F1 - Processing parameters

SI	1024
MC2	echo-antiecho
SF	150.9027969 MHz
WDW	QSINE
SSB	2
LB	0 Hz
GB	0

^{1}H NMR spectrum of compound 30

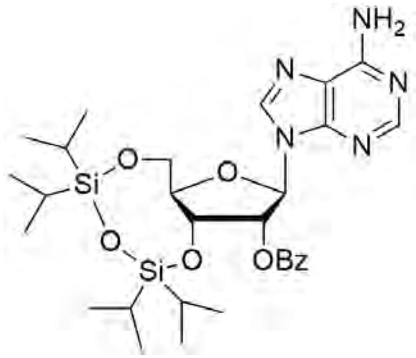

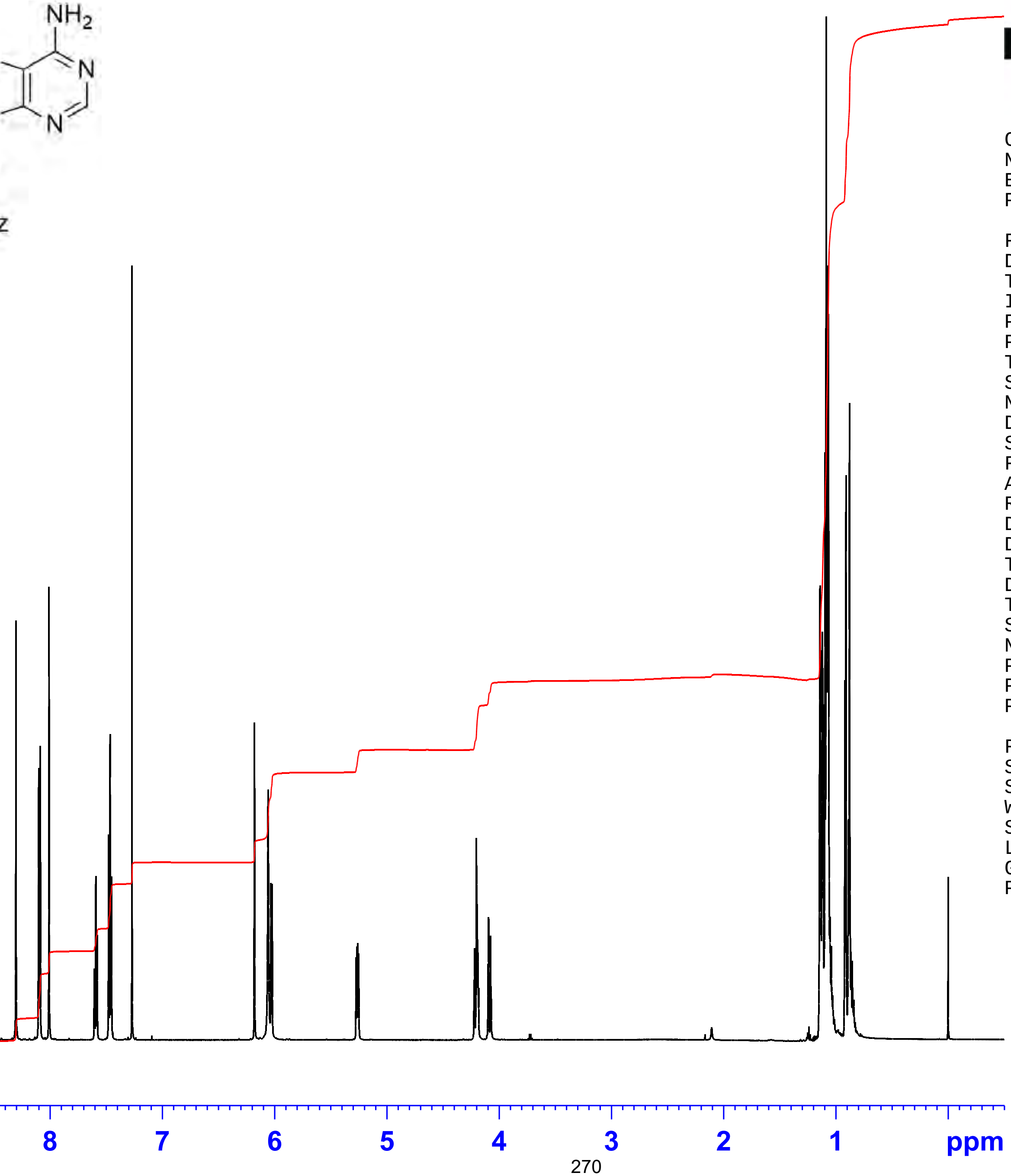


```
Current Data Parameters
NAME              LH-I-58
EXPNO                  20
PROCNO                  1

F2 - Acquisition Parameters
Date_            20220307
Time                12.41 h
INSTRUM             spect
PROBHD   Z114607_0188 (
PULPROG              zg30
TD                 180286
SOLVENT             CDCl3
NS                     16
DS                      0
SWH             18028.846 Hz
FIDRES           0.200003 Hz
AQ              4.9999318 sec
RG                  34.91
DW                 27.733 usec
DE                   8.00 usec
TE                  300.0 K
D1             0.10000000 sec
TD0                     1
SFO1          600.1337060 MHz
NUC1                   1H
P0                   3.33 usec
P1                  10.00 usec
PLW1          26.60000038 W

F2 - Processing parameters
SI                 262144
SF            600.1300082 MHz
WDW                    EM
SSB      0
LB                   0.10 Hz
GB       0
PC                   1.00
```

Expanded region of the ¹H NMR spectrum of compound 30

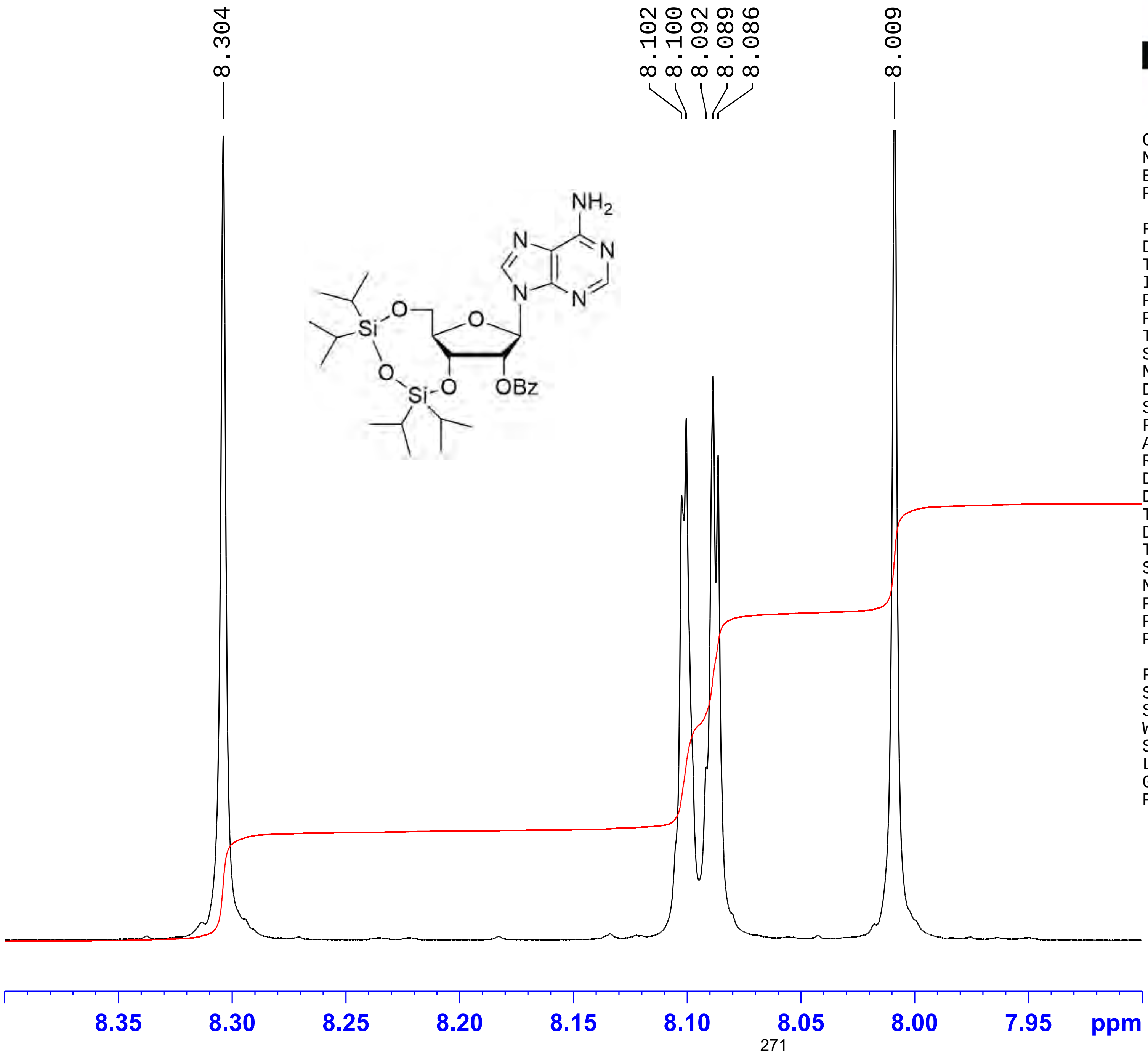

Current Data Parameters
NAME LH-I-58
EXPNO 20
PROCNO 1

F2 - Acquisition Parameters
Date_ 20220307
Time 12.41 h
INSTRUM spect
PROBHD Z114607_0188 (
PULPROG zg30
TD 180286
SOLVENT CDCl3
NS 16
DS 0
SWH 18028.846 Hz
FIDRES 0.200003 Hz
AQ 4.9999318 sec
RG 34.91
DW 27.733 usec
DE 8.00 usec
TE 300.0 K
D1 0.10000000 sec
TD0 1
SFO1 600.1337060 MHz
NUC1 1H
P0 3.33 usec
P1 10.00 usec
PLW1 26.60000038 W

F2 - Processing parameters
SI 262144
SF 600.1300082 MHz
WDW EM
SSB 0
LB 0.10 Hz
GB 0
PC 1.00

Expanded region of the ^{1}H NMR spectrum of compound 30

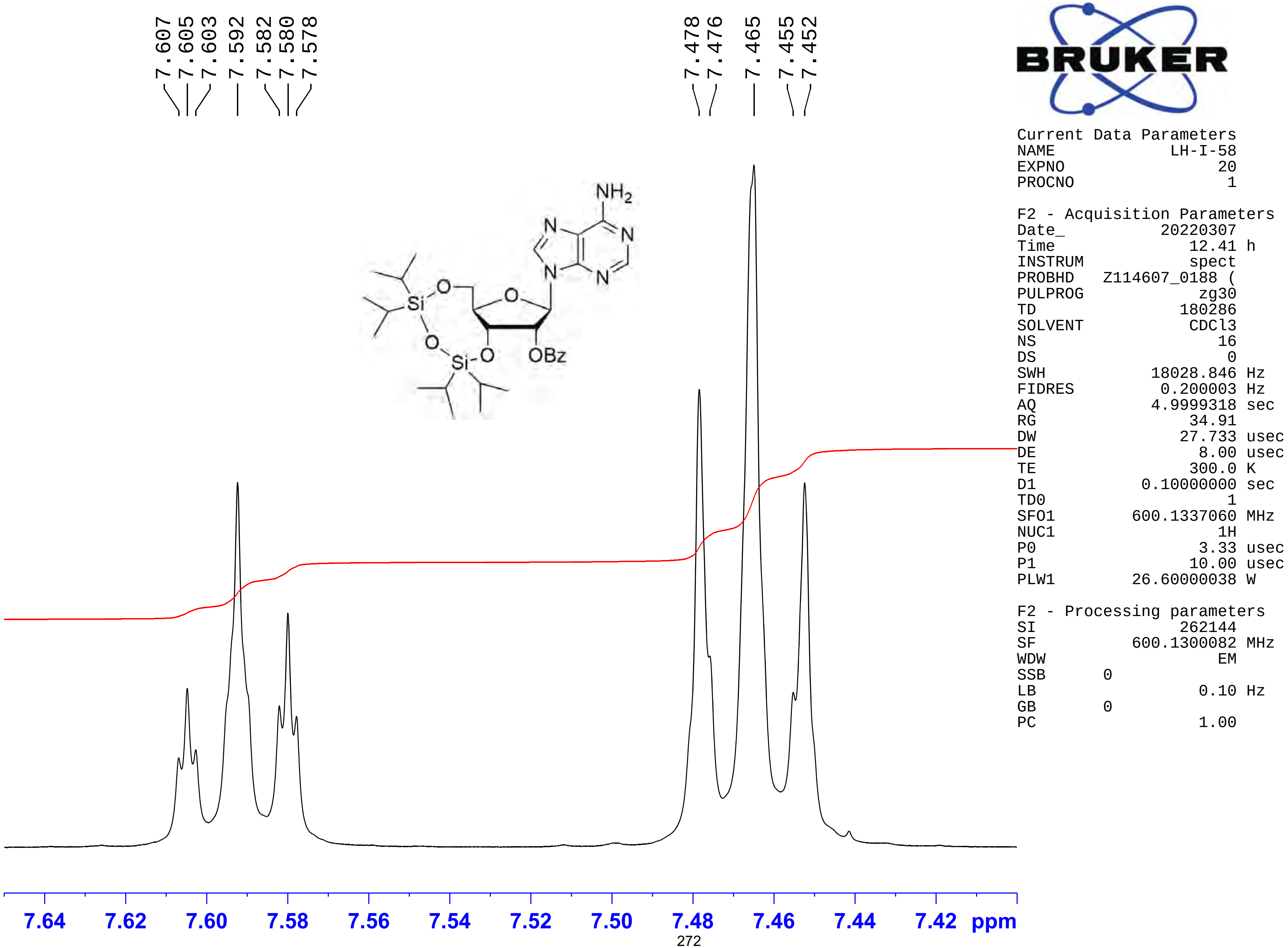

Expanded region of the ^{1}H NMR spectrum of compound 30

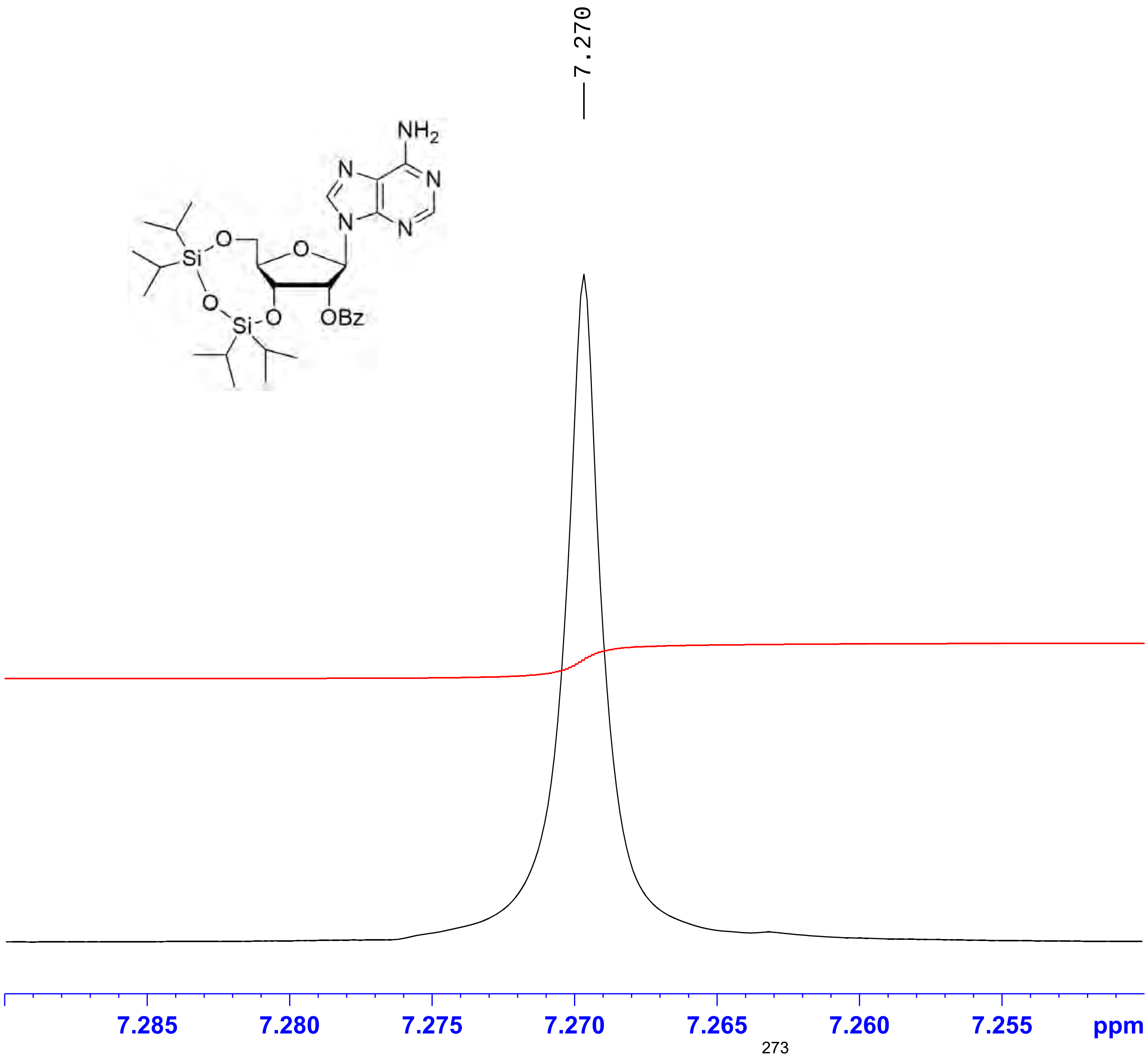

BRUKER

Current Data Parameters
NAME LH-I-58
EXPNO 20
PROCNO 1

F2 - Acquisition Parameters
Date_ 20220307
Time 12.41 h
INSTRUM spect
PROBHD Z114607_0188 (
PULPROG zg30
TD 180286
SOLVENT CDCl3
NS 16
DS 0
SWH 18028.846 Hz
FIDRES 0.200003 Hz
AQ 4.9999318 sec
RG 34.91
DW 27.733 usec
DE 8.00 usec
TE 300.0 K
D1 0.10000000 sec
TD0 1
SFO1 600.1337060 MHz
NUC1 1H
P0 3.33 usec
P1 10.00 usec
PLW1 26.60000038 W

F2 - Processing parameters
SI 262144
SF 600.1300082 MHz
WDW EM
SSB 0
LB 0.10 Hz
GB 0
PC 1.00

Expanded region of the ^{1}H NMR spectrum of compound 30

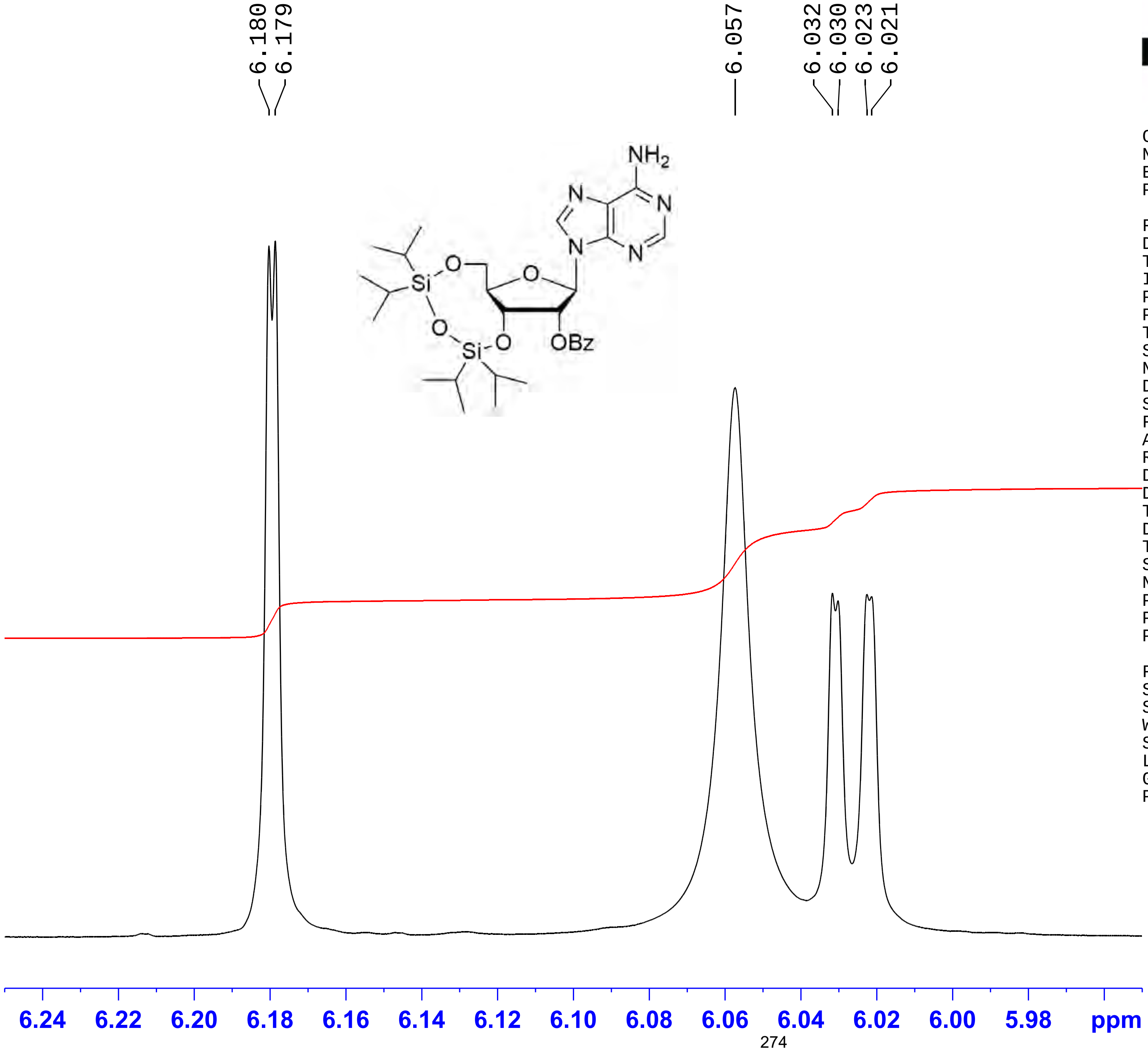

Current Data Parameters
NAME LH-I-58
EXPNO 20
PROCNO 1

F2 - Acquisition Parameters
Date_ 20220307
Time 12.41 h
INSTRUM spect
PROBHD Z114607_0188 (
PULPROG zg30
TD 180286
SOLVENT CDCl3
NS 16
DS 0
SWH 18028.846 Hz
FIDRES 0.200003 Hz
AQ 4.9999318 sec
RG 34.91
DW 27.733 usec
DE 8.00 usec
TE 300.0 K
D1 0.10000000 sec
TD0 1
SFO1 600.1337060 MHz
NUC1 1H
P0 3.33 usec
P1 10.00 usec
PLW1 26.60000038 W

F2 - Processing parameters
SI 262144
SF 600.1300082 MHz
WDW EM
SSB 0
LB 0.10 Hz
GB 0
PC 1.00

Expanded region of the ^{1}H NMR spectrum of compound 30

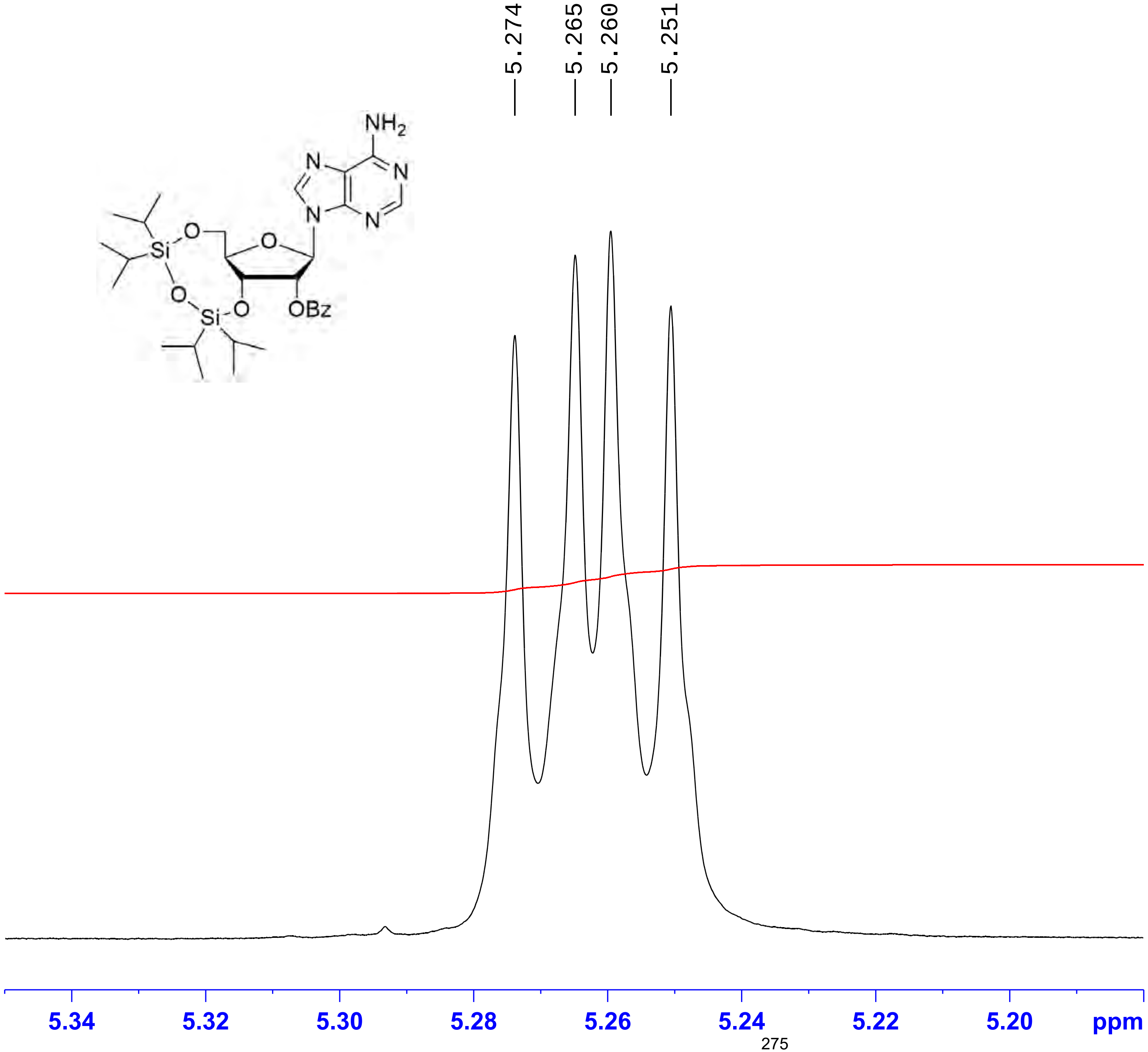

Current Data Parameters
NAME LH-I-58
EXPNO 20
PROCNO 1

F2 - Acquisition Parameters
Date_ 20220307
Time 12.41 h
INSTRUM spect
PROBHD Z114607_0188 (
PULPROG zg30
TD 180286
SOLVENT CDCl3
NS 16
DS 0
SWH 18028.846 Hz
FIDRES 0.200003 Hz
AQ 4.9999318 sec
RG 34.91
DW 27.733 usec
DE 8.00 usec
TE 300.0 K
D1 0.10000000 sec
TD0 1
SFO1 600.1337060 MHz
NUC1 1H
P0 3.33 usec
P1 10.00 usec
PLW1 26.60000038 W

F2 - Processing parameters
SI 262144
SF 600.1300082 MHz
WDW EM
SSB 0
LB 0.10 Hz
GB 0
PC 1.00

Expanded region of the ^{1}H NMR spectrum of compound 30

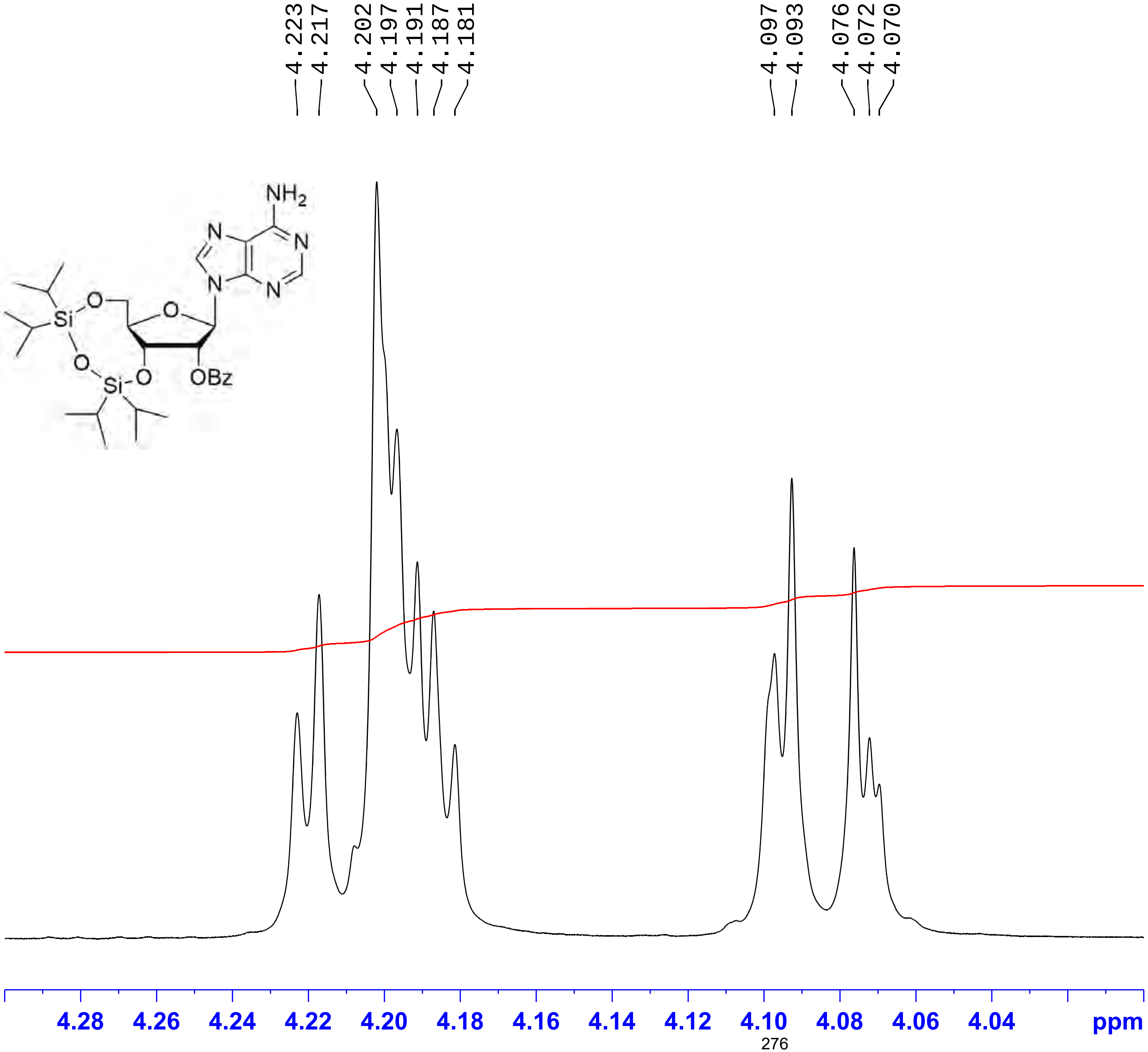


```
Current Data Parameters
NAME              LH-I-58
EXPNO                  20
PROCNO                  1

F2 - Acquisition Parameters
Date_            20220307
Time                12.41 h
INSTRUM             spect
PROBHD   Z114607_0188 (
PULPROG              zg30
TD                 180286
SOLVENT             CDCl3
NS                     16
DS                      0
SWH             18028.846 Hz
FIDRES           0.200003 Hz
AQ              4.9999318 sec
RG                  34.91
DW                 27.733 usec
DE                   8.00 usec
TE                  300.0 K
D1             0.10000000 sec
TD0                     1
SFO1          600.1337060 MHz
NUC1                   1H
P0                   3.33 usec
P1                  10.00 usec
PLW1          26.60000038 W

F2 - Processing parameters
SI                 262144
SF            600.1300082 MHz
WDW                    EM
SSB      0
LB                   0.10 Hz
GB       0
PC                   1.00
```

^{13}C NMR spectrum of compound 30

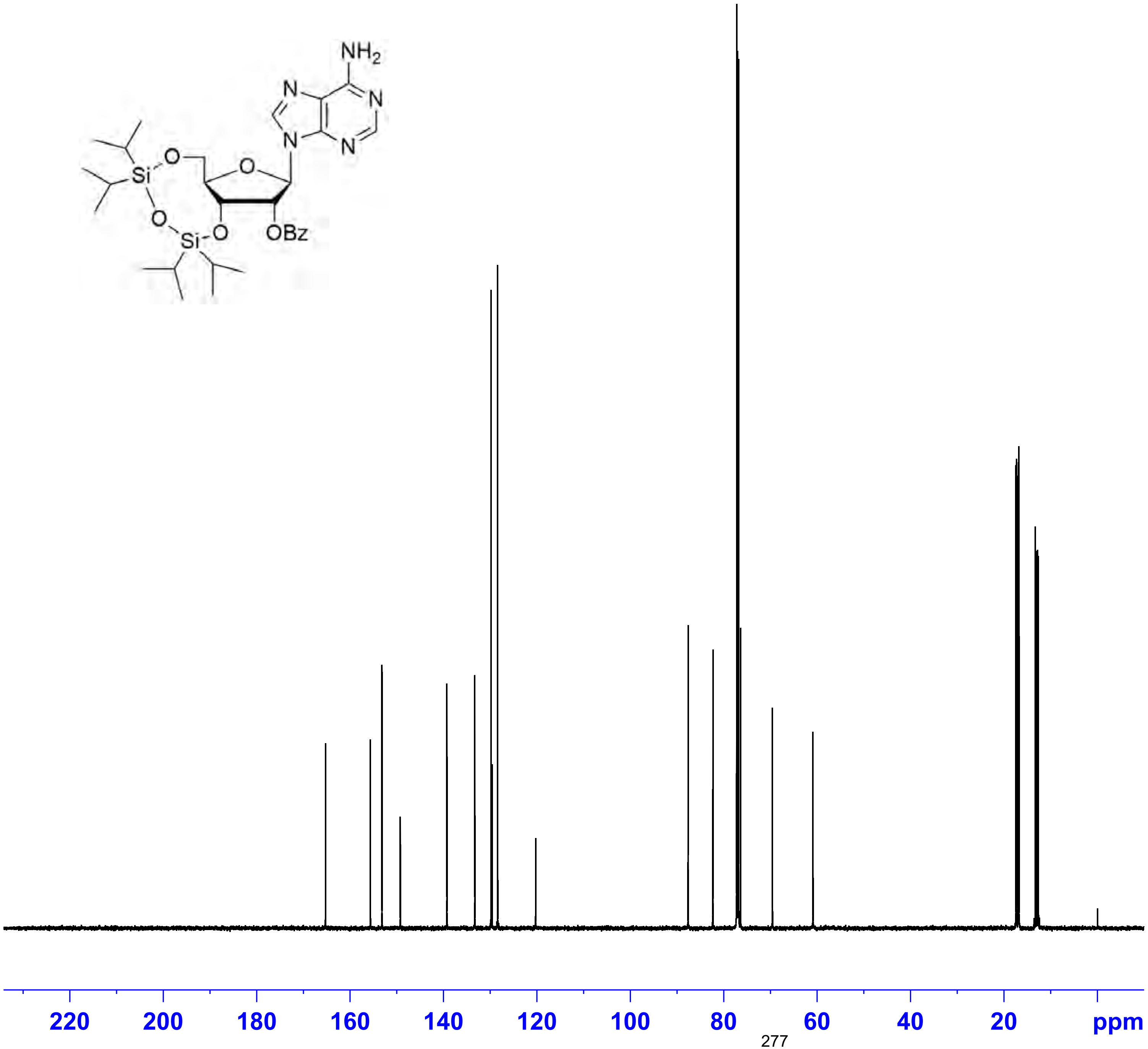

Current Data Parameters
NAME LH-I-58
EXPNO 21
PROCNO 1

F2 - Acquisition Parameters
Date_ 20220307
Time 14.10 h
INSTRUM spect
PROBHD Z114607_0188 (
PULPROG zgpg30
TD 119044
SOLVENT CDCl3
NS 2000
DS 4
SWH 37500.000 Hz
FIDRES 0.630019 Hz
AQ 1.5872533 sec
RG 186.92
DW 13.333 usec
DE 6.53 usec
TE 300.0 K
D1 1.00000000 sec
D11 0.03000000 sec
TD0 1
SFO1 150.9194058 MHz
NUC1 13C
P0 3.93 usec
P1 11.80 usec
PLW1 85.00000000 W
SFO2 600.1324005 MHz
NUC2 1H
CPDPRG[2 waltz64
PCPD2 70.00 usec
PLW2 27.00000000 W
PLW12 0.57327998 W
PLW13 0.28836000 W

F2 - Processing parameters
SI 131072
SF 150.9028155 MHz
WDW EM
SSB 0
LB 1.00 Hz
GB 0
PC 1.40

Expanded region of the ^{13}C NMR spectrum of compound 30

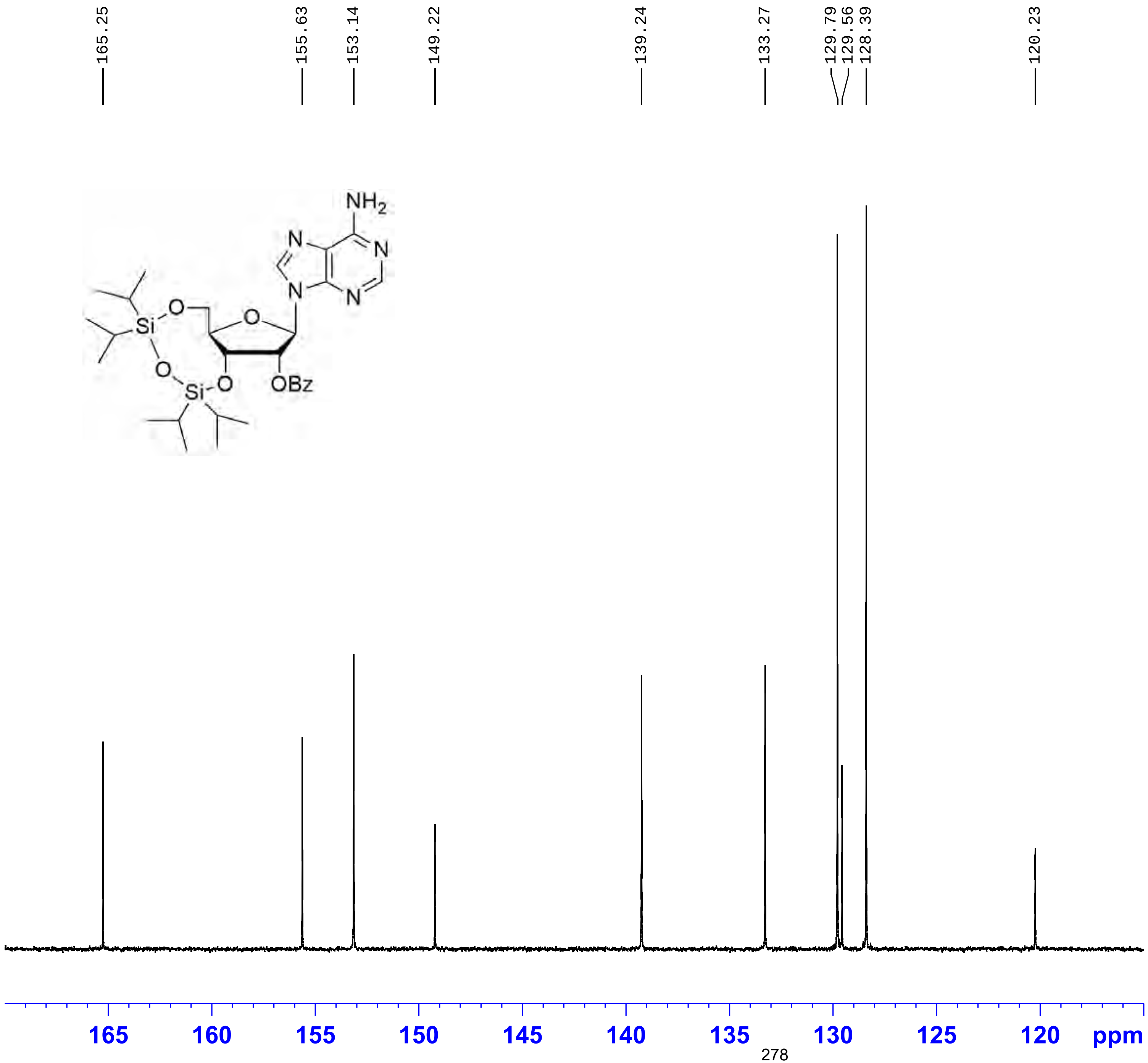

BRUKER

Current Data Parameters
NAME LH-I-58
EXPNO 21
PROCNO 1

F2 - Acquisition Parameters
Date_ 20220307
Time 14.10 h
INSTRUM spect
PROBHD Z114607_0188 (
PULPROG zgpg30
TD 119044
SOLVENT CDCl3
NS 2000
DS 4
SWH 37500.000 Hz
FIDRES 0.630019 Hz
AQ 1.5872533 sec
RG 186.92
DW 13.333 usec
DE 6.53 usec
TE 300.0 K
D1 1.00000000 sec
D11 0.03000000 sec
TD0 1
SFO1 150.9194058 MHz
NUC1 13C
P0 3.93 usec
P1 11.80 usec
PLW1 85.00000000 W
SFO2 600.1324005 MHz
NUC2 1H
CPDPRG[2 waltz64
PCPD2 70.00 usec
PLW2 27.00000000 W
PLW12 0.57327998 W
PLW13 0.28836000 W

F2 - Processing parameters
SI 131072
SF 150.9028155 MHz
WDW EM
SSB 0
LB 1.00 Hz
GB 0
PC 1.40

Expanded region of the ^{13}C NMR spectrum of compound 30

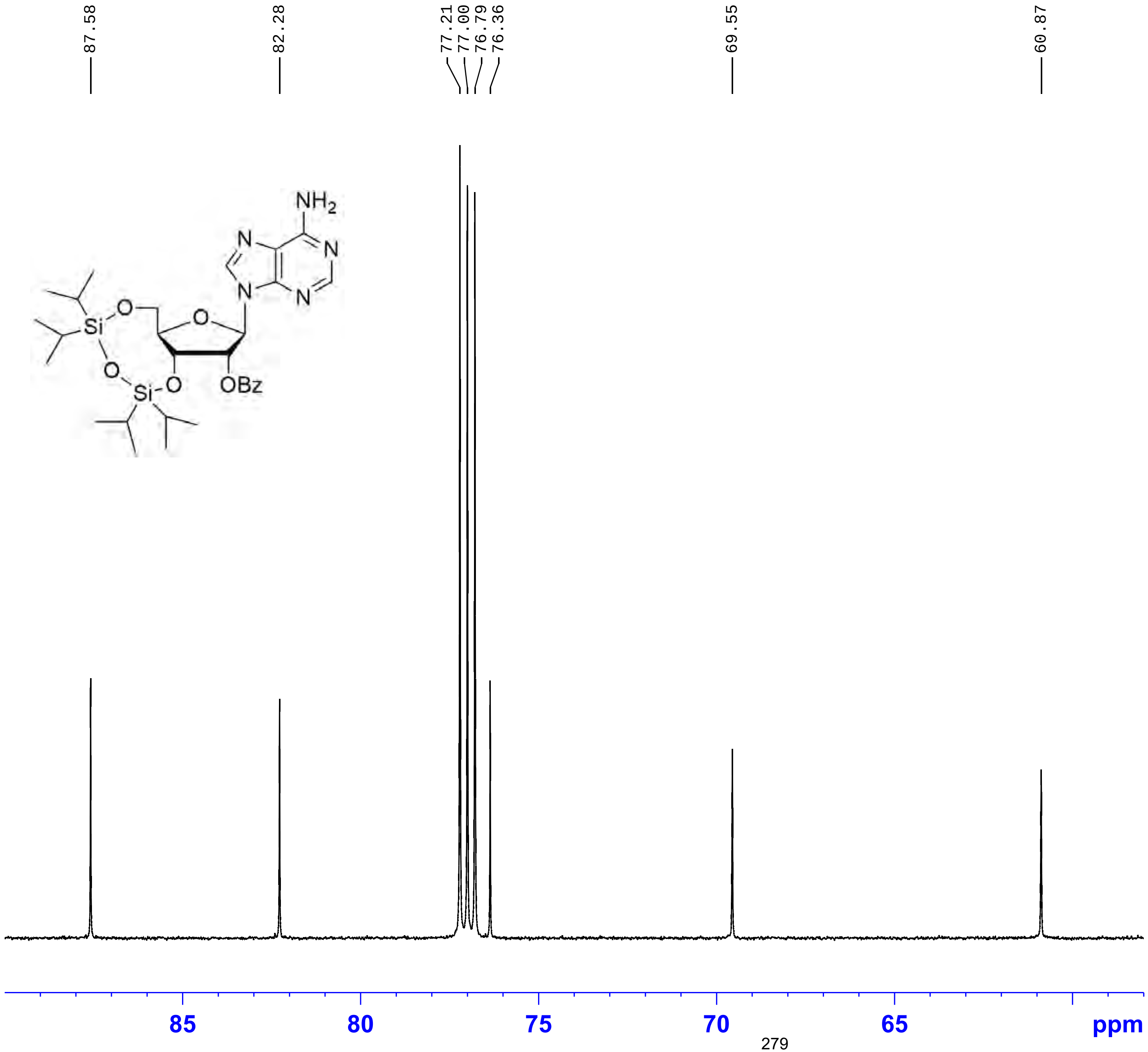

Current Data Parameters
NAME LH-I-58
EXPNO 21
PROCNO 1

F2 - Acquisition Parameters
Date_ 20220307
Time 14.10 h
INSTRUM spect
PROBHD Z114607_0188 (
PULPROG zgpg30
TD 119044
SOLVENT CDCl3
NS 2000
DS 4
SWH 37500.000 Hz
FIDRES 0.630019 Hz
AQ 1.5872533 sec
RG 186.92
DW 13.333 usec
DE 6.53 usec
TE 300.0 K
D1 1.00000000 sec
D11 0.03000000 sec
TD0 1
SFO1 150.9194058 MHz
NUC1 13C
P0 3.93 usec
P1 11.80 usec
PLW1 85.00000000 W
SFO2 600.1324005 MHz
NUC2 1H
CPDPRG[2 waltz64
PCPD2 70.00 usec
PLW2 27.00000000 W
PLW12 0.57327998 W
PLW13 0.28836000 W

F2 - Processing parameters
SI 131072
SF 150.9028155 MHz
WDW EM
SSB 0
LB 1.00 Hz
GB 0
PC 1.40

Expanded region of the ^{13}C NMR spectrum of compound 30

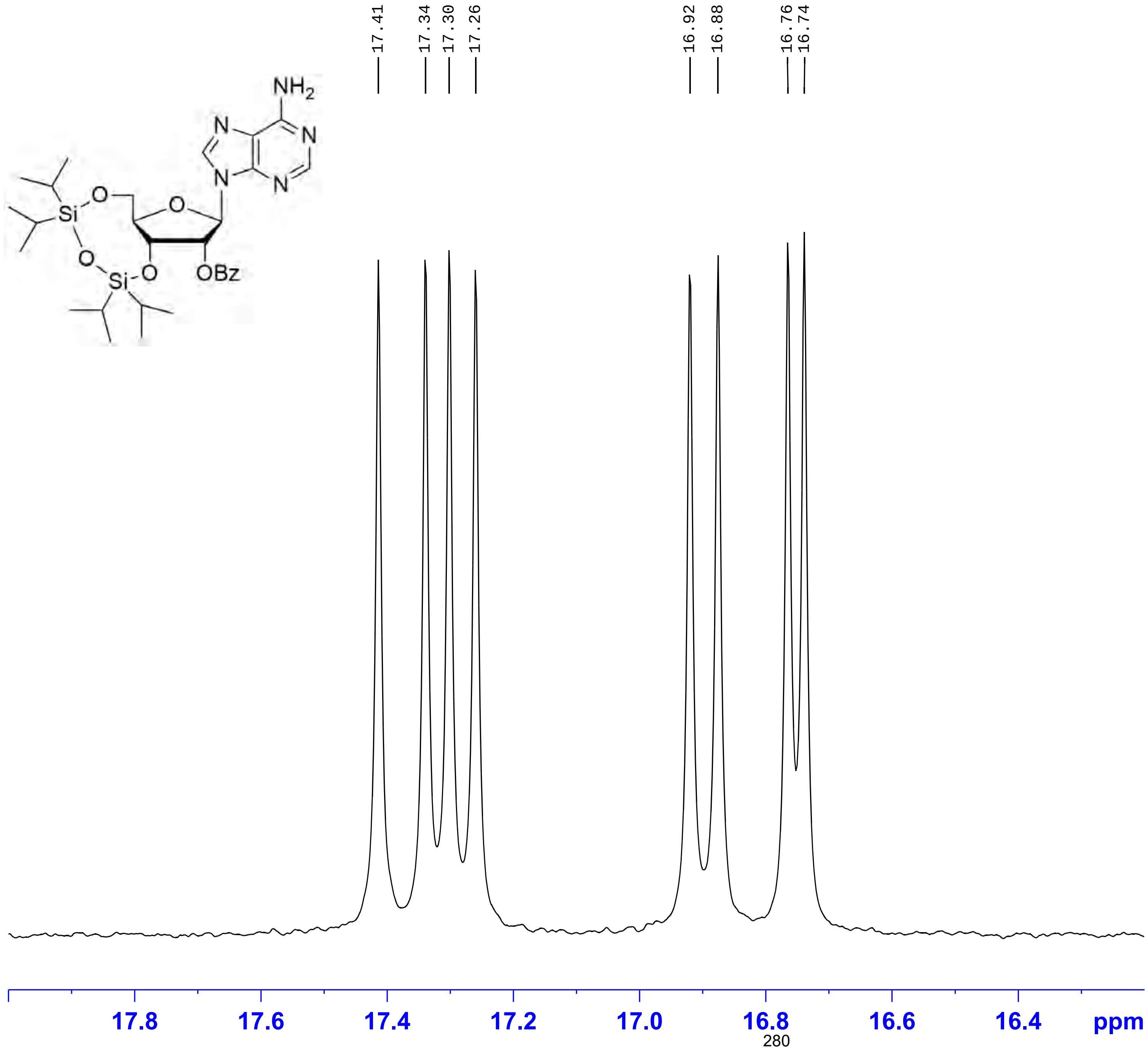

Current Data Parameters
NAME LH-I-58
EXPNO 21
PROCNO 1

F2 - Acquisition Parameters
Date_ 20220307
Time 14.10 h
INSTRUM spect
PROBHD Z114607_0188 (
PULPROG zgpg30
TD 119044
SOLVENT CDCl3
NS 2000
DS 4
SWH 37500.000 Hz
FIDRES 0.630019 Hz
AQ 1.5872533 sec
RG 186.92
DW 13.333 usec
DE 6.53 usec
TE 300.0 K
D1 1.00000000 sec
D11 0.03000000 sec
TD0 1
SFO1 150.9194058 MHz
NUC1 13C
P0 3.93 usec
P1 11.80 usec
PLW1 85.00000000 W
SFO2 600.1324005 MHz
NUC2 1H
CPDPRG[2 waltz64
PCPD2 70.00 usec
PLW2 27.00000000 W
PLW12 0.57327998 W
PLW13 0.28836000 W

F2 - Processing parameters
SI 131072
SF 150.9028155 MHz
WDW EM
SSB 0
LB 1.00 Hz
GB 0
PC 1.40

Expanded region of the ^{13}C NMR spectrum of compound 30

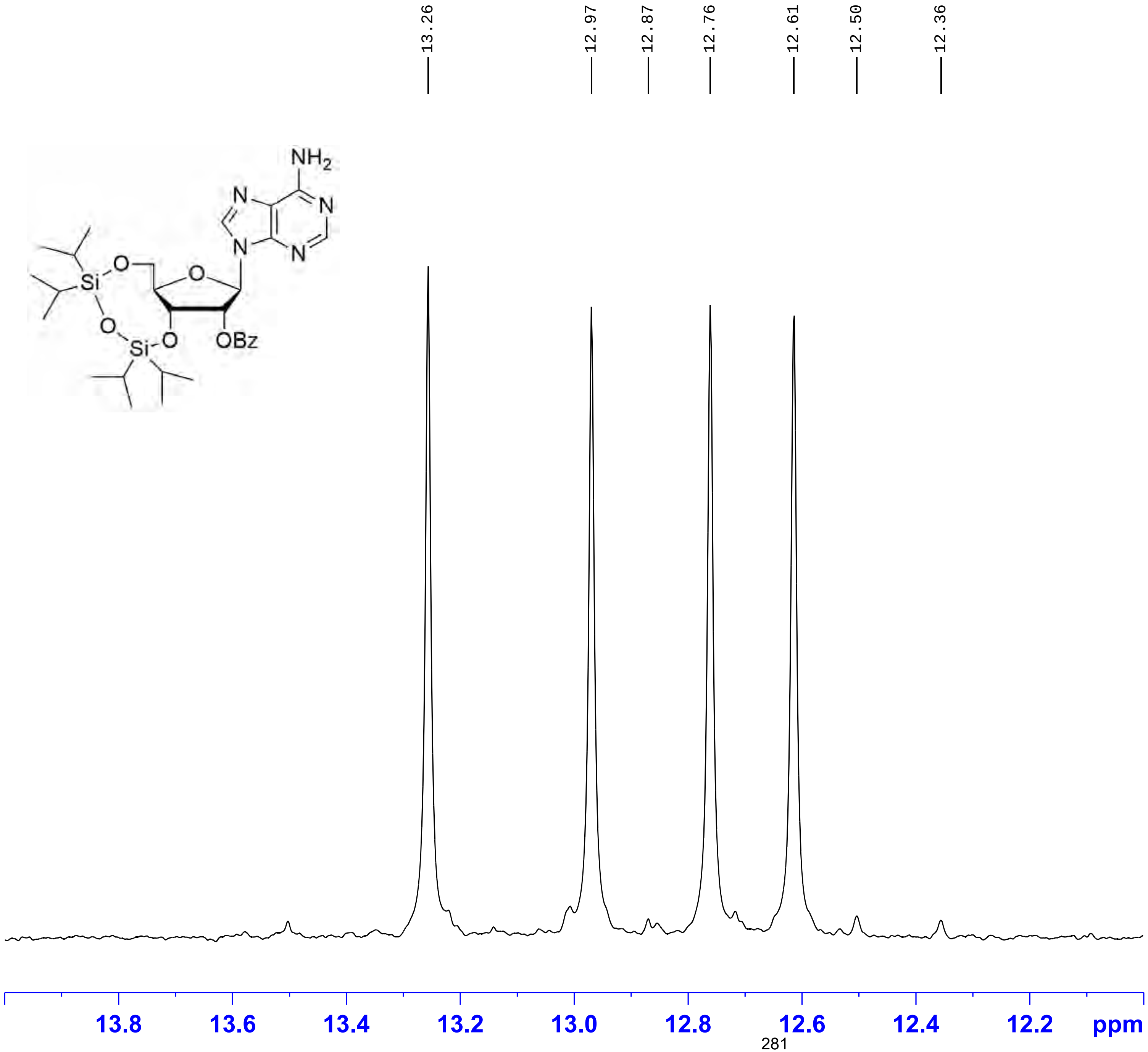

Current Data Parameters

NAME	LH-I-58
EXPNO	21
PROCNO	1

F2 - Acquisition Parameters

Date_	20220307
Time	14.10 h
INSTRUM	spect
PROBHD	Z114607_0188 (
PULPROG	zgpg30
TD	119044
SOLVENT	CDCl3
NS	2000
DS	4
SWH	37500.000 Hz
FIDRES	0.630019 Hz
AQ	1.5872533 sec
RG	186.92
DW	13.333 usec
DE	6.53 usec
TE	300.0 K
D1	1.00000000 sec
D11	0.03000000 sec
TD0	1
SFO1	150.9194058 MHz
NUC1	13C
P0	3.93 usec
P1	11.80 usec
PLW1	85.00000000 W
SFO2	600.1324005 MHz
NUC2	1H
CPDPRG[2	waltz64
PCPD2	70.00 usec
PLW2	27.00000000 W
PLW12	0.57327998 W
PLW13	0.28836000 W

F2 - Processing parameters

SI	131072
SF	150.9028155 MHz
WDW	EM
SSB	0
LB	1.00 Hz
GB	0
PC	1.40

^{13}C DEPT-135 NMR spectrum of compound 30

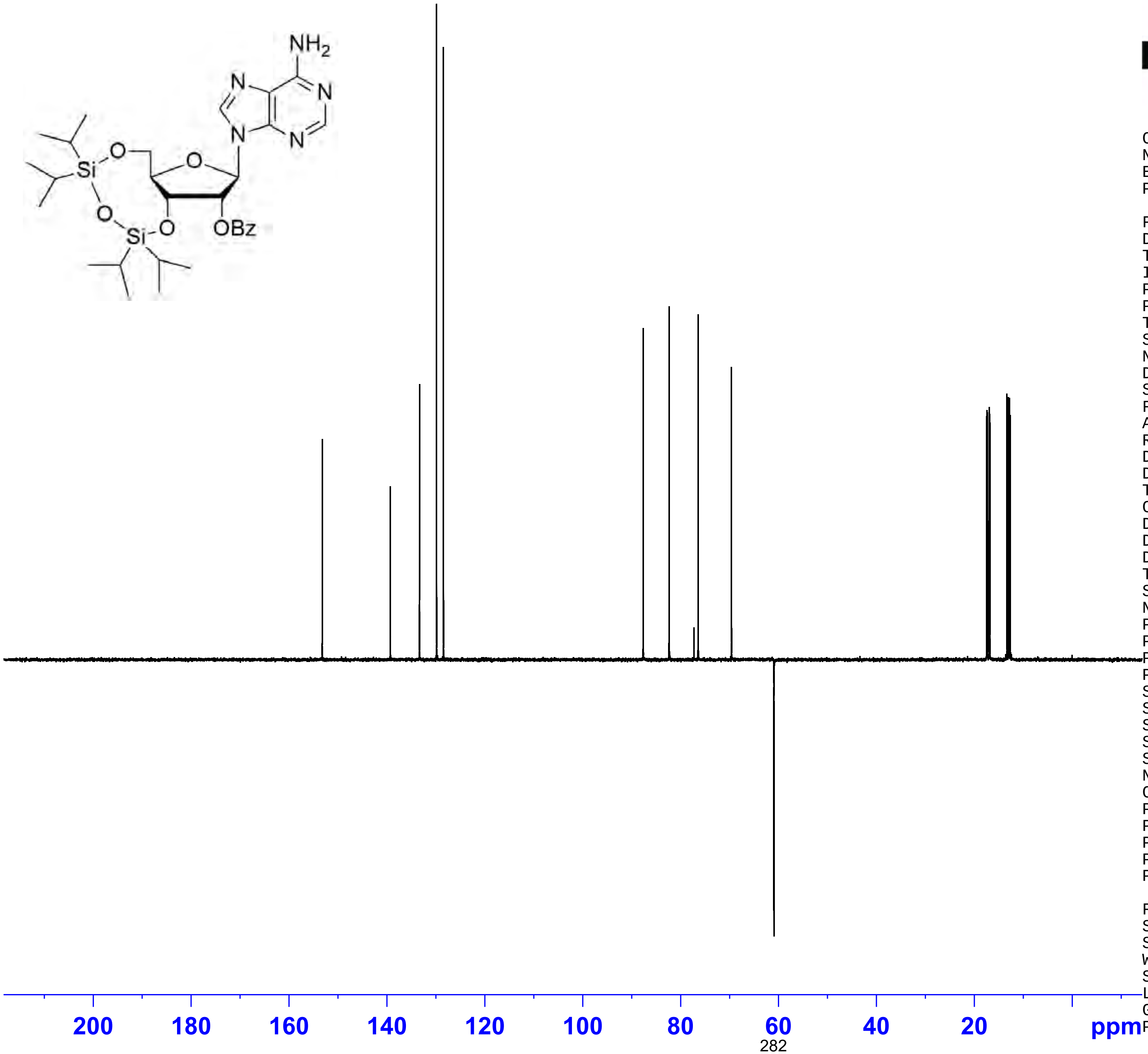

Current Data Parameters
NAME LH-I-58
EXPNO 22
PROCNO 1

F2 - Acquisition Parameters
Date_ 20220307
Time 14.56 h
INSTRUM spect
PROBHD Z114607_0188 (
PULPROG deptsp135.b
TD 119044
SOLVENT CDCl3
NS 1000
DS 4
SWH 35714.285 Hz
FIDRES 0.600018 Hz
AQ 1.6666160 sec
RG 186.92
DW 14.000 usec
DE 7.44 usec
TE 300.0 K
CNST2 145.0000000
D1 1.00000000 sec
D2 0.00344828 sec
D12 0.00002000 sec
TD0 1
SFO1 150.9178962 MHz
NUC1 13C
P1 11.80 usec
P13 2000.00 usec
PLW0 0 W
PLW1 85.00000000 W
SPNAM[5] Crp60comp.4
SPOAL5 0.500
SPOFFS5 0 Hz
SPW5 18.08300018 W
SFO2 600.1324005 MHz
NUC2 1H
CPDPRG[2 waltz64
P3 10.20 usec
P4 20.40 usec
PCPD2 70.00 usec
PLW2 27.00000000 W
PLW12 0.57327998 W

F2 - Processing parameters
SI 131072
SF 150.9028085 MHz
WDW EM
SSB 0
LB 1.00 Hz
GB 0
PC 1.40

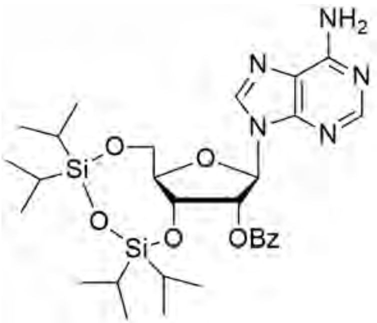

^1H-^1H COSY NMR spectrum of compound 30

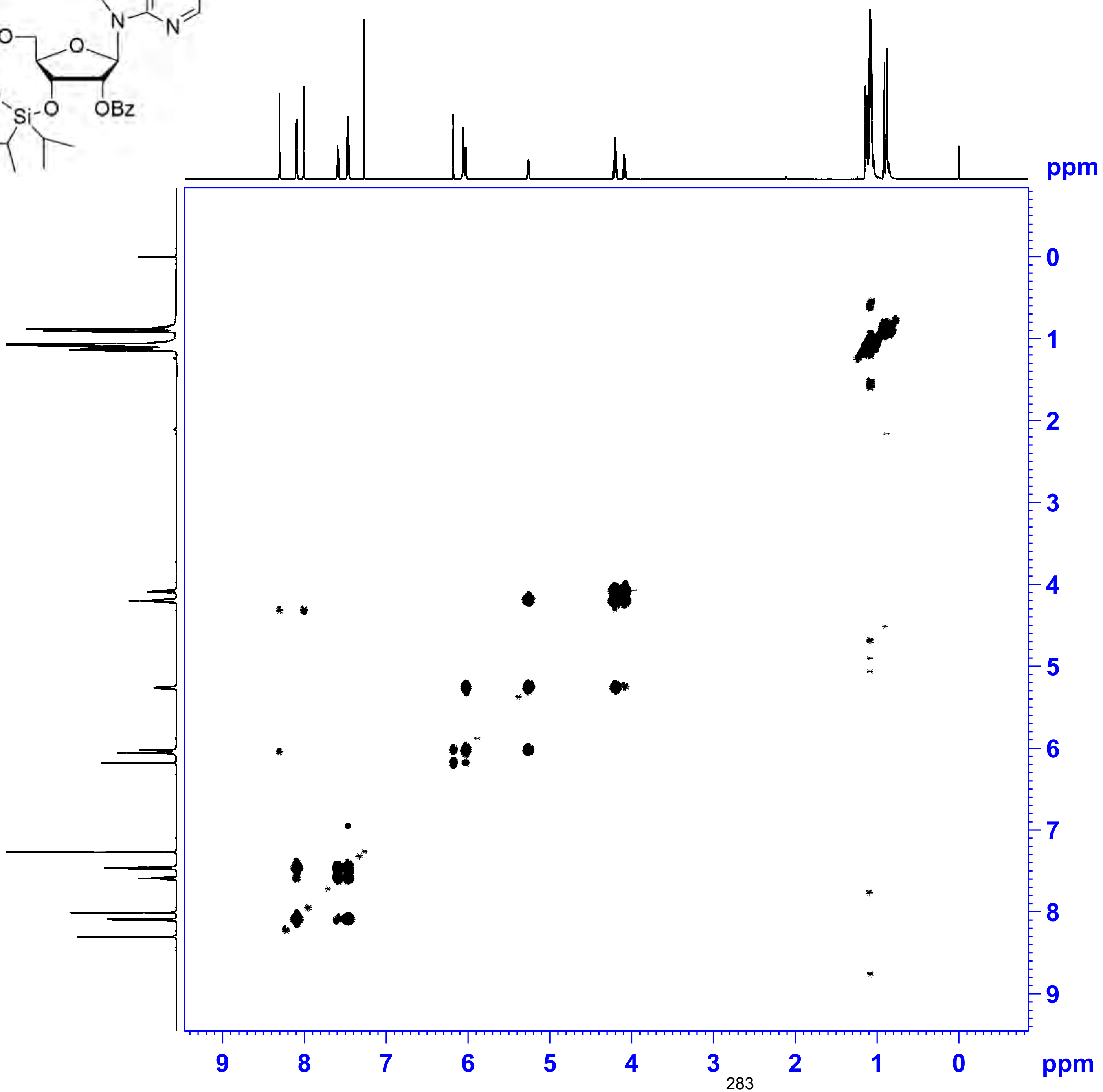

BRUKER

Current Data Parameters
NAME LH-I-58
EXPNO 23
PROCNO 1

F2 - Acquisition Parameters
Date_ 20220307
Time 15.06 h
INSTRUM spect
PROBHD Z114607_0188 (
PULPROG cosygpmfppqf
TD 2048
SOLVENT CDCl3
NS 2
DS 8
SWH 6188.119 Hz
FIDRES 6.043085 Hz
AQ 0.1654784 sec
RG 186.92
DW 80.800 usec
DE 6.50 usec
TE 300.0 K
D0 0.00000300 sec
D1 0.89555132 sec
D11 0.03000000 sec
D12 0.00002000 sec
D13 0.00000400 sec
D16 0.00020000 sec
IN0 0.00016180 sec
TDav 1
SFO1 600.1325943 MHz
NUC1 1H
P1 10.00 usec
P17 2500.00 usec
PLW1 26.60000038 W
PLW10 4.25600004 W
GPNAM[1] SMSQ10.100
GPZ1 16.00 %
GPNAM[2] SMSQ10.100
GPZ2 12.00 %
GPNAM[3] SMSQ10.100
GPZ3 40.00 %
P16 1000.00 usec

F1 - Acquisition parameters
TD 256
SFO1 600.1326 MHz
FIDRES 48.284920 Hz
SW 10.299 ppm
FnMODE QF

F2 - Processing parameters
SI 1024
SF 600.1300094 MHz
WDW SINE
SSB 0
LB 0 Hz
GB 0
PC 1.40

F1 - Processing parameters
SI 1024
MC2 QF
SF 600.1300112 MHz
WDW SINE
SSB 0
LB 0 Hz
GB 0

^1H-^{13}C HSQC NMR spectrum of compound 30

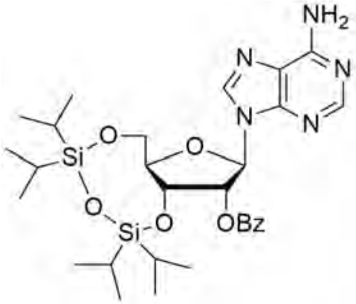

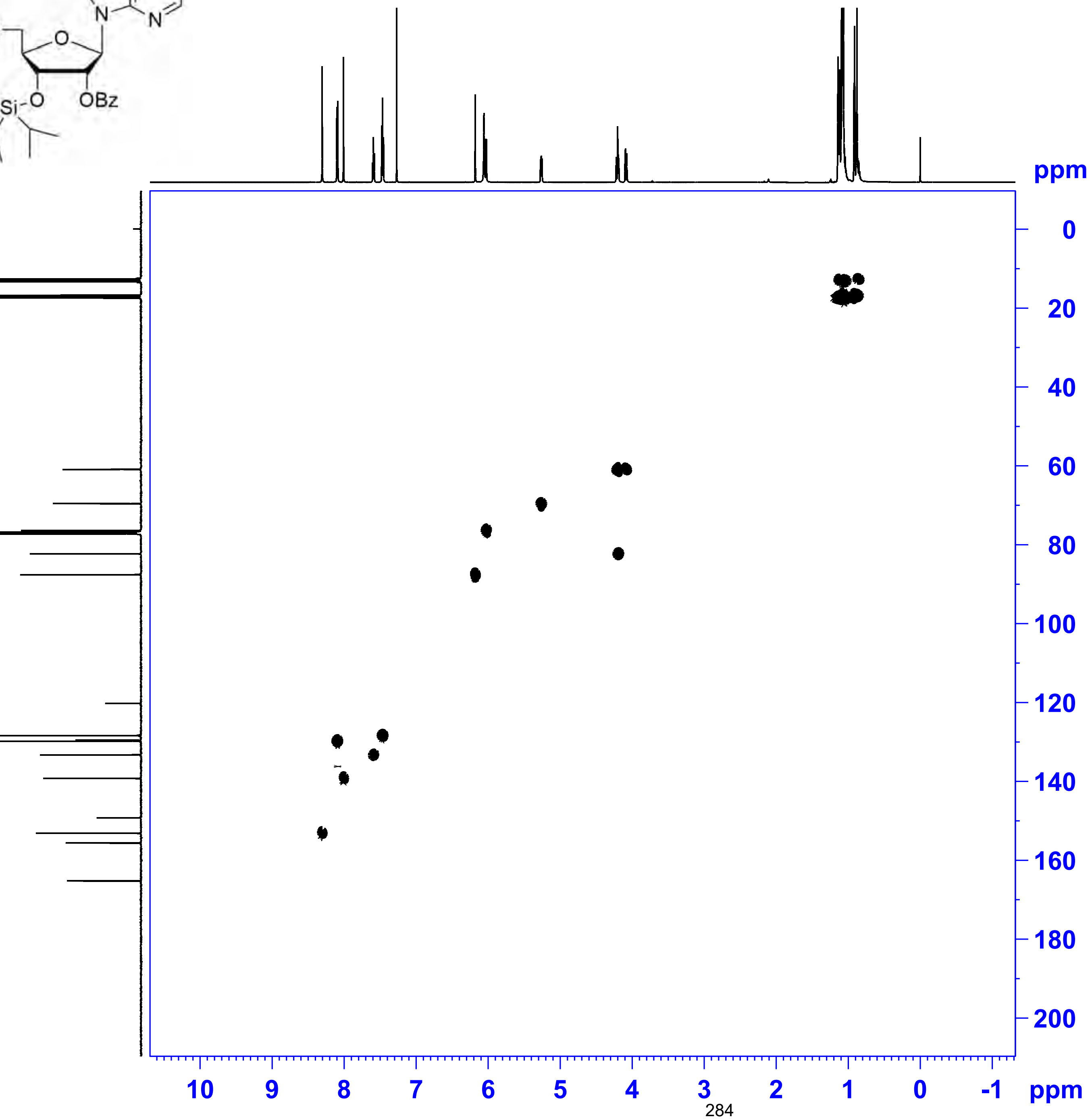


```
Current Data Parameters
NAME                LH-I-58
EXPNO                    24
PROCNO                    1

F2 - Acquisition Parameters
Date_              20220307
Time                  15.15 h
INSTRUM               spect
PROBHD   Z114607_0188 (
PULPROG  hsqcedetgpsp.3
TD                     1024
SOLVENT               CDCl3
NS                        2
DS                       32
SWH                7211.539 Hz
FIDRES            14.085036 Hz
AQ                0.0709973 sec
RG                   186.92
DW                   69.333 usec
DE                     6.50 usec
TE                    300.2 K
CNST2           145.0000000
D0               0.00000300 sec
D1               0.80000001 sec
D4               0.00172414 sec
D11              0.03000000 sec
D16              0.00020000 sec
D21              0.00360000 sec
IN0              0.00001510 sec
TDav                      1
ZGOPTNS
SFO1            600.1328223 MHz
NUC1                     1H
P1                    10.00 usec
P2                    20.00 usec
PLW1            26.60000038 W
SFO2            150.9178988 MHz
NUC2                    13C
CPDPRG[2              garp4
P3                    11.80 usec
P14                  500.00 usec
P31                 1730.00 usec
PCPD2                 60.00 usec
PLW0     0 W
PLW2            85.00000000 W
PLW12            3.28760004 W
SPNAM[3] Crp60,0.5,20.1
SPOAL3                0.500
SPOFFS3  0 Hz
SPW3            18.08300018 W
SPNAM[18  Crp60_xfilt.2
SPOAL18               0.500
SPOFFS18 0 Hz
SPW18            5.22629976 W
GPNAM[1]         SMSQ10.100
GPZ1                  80.00 %
GPNAM[2]         SMSQ10.100
GPZ2                  20.10 %
P16                 1000.00 usec

F1 - Acquisition parameters
TD                      256
SFO1               150.9179 MHz
FIDRES           258.692047 Hz
SW                  219.408 ppm
FnMODE         Echo-Antiecho

F2 - Processing parameters
SI                     1024
SF              600.1300091 MHz
WDW                   QSINE
SSB                       2
LB       0 Hz
GB       0
PC                     1.40

F1 - Processing parameters
SI                     1024
MC2            echo-antiecho
SF              150.9028106 MHz
WDW                   QSINE
SSB                       2
LB       0 Hz
GB       0
```

^{1}H NMR spectrum of compound 31

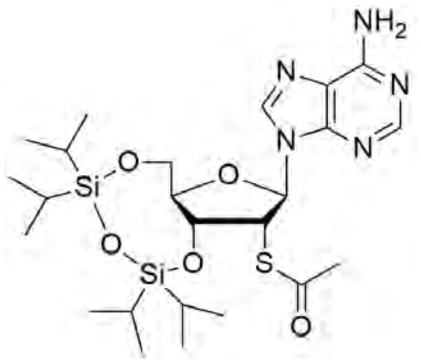

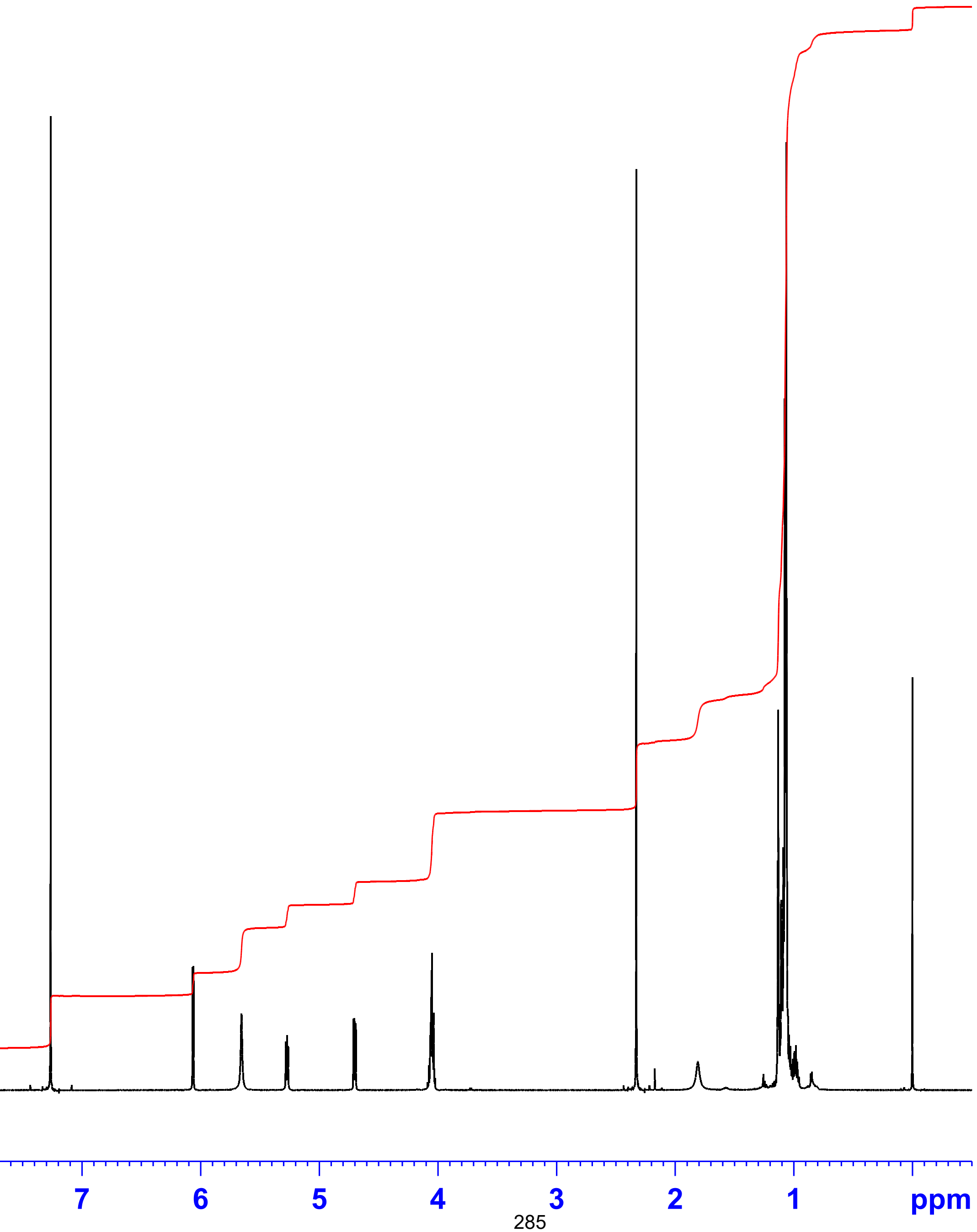


```
Current Data Parameters
NAME                LH-I-59
EXPNO                    20
PROCNO                    1

F2 - Acquisition Parameters
Date_              20220210
Time                  11.21 h
INSTRUM               spect
PROBHD    Z114607_0188 (
PULPROG                zg30
TD                   180286
SOLVENT               CDCl3
NS                       16
DS                        0
SWH               18028.846 Hz
FIDRES             0.200003 Hz
AQ                4.9999318 sec
RG                     97.5
DW                   27.733 usec
DE                     8.00 usec
TE                    300.0 K
D1               0.10000000 sec
TD0                       1
SFO1            600.1337060 MHz
NUC1                     1H
P0                     3.33 usec
P1                    10.00 usec
PLW1            26.60000038 W

F2 - Processing parameters
SI                   262144
SF              600.1300124 MHz
WDW                      EM
SSB      0
LB                     0.10 Hz
GB       0
PC                     1.00
```

Expanded region of the ^{1}H NMR spectrum of compound 31

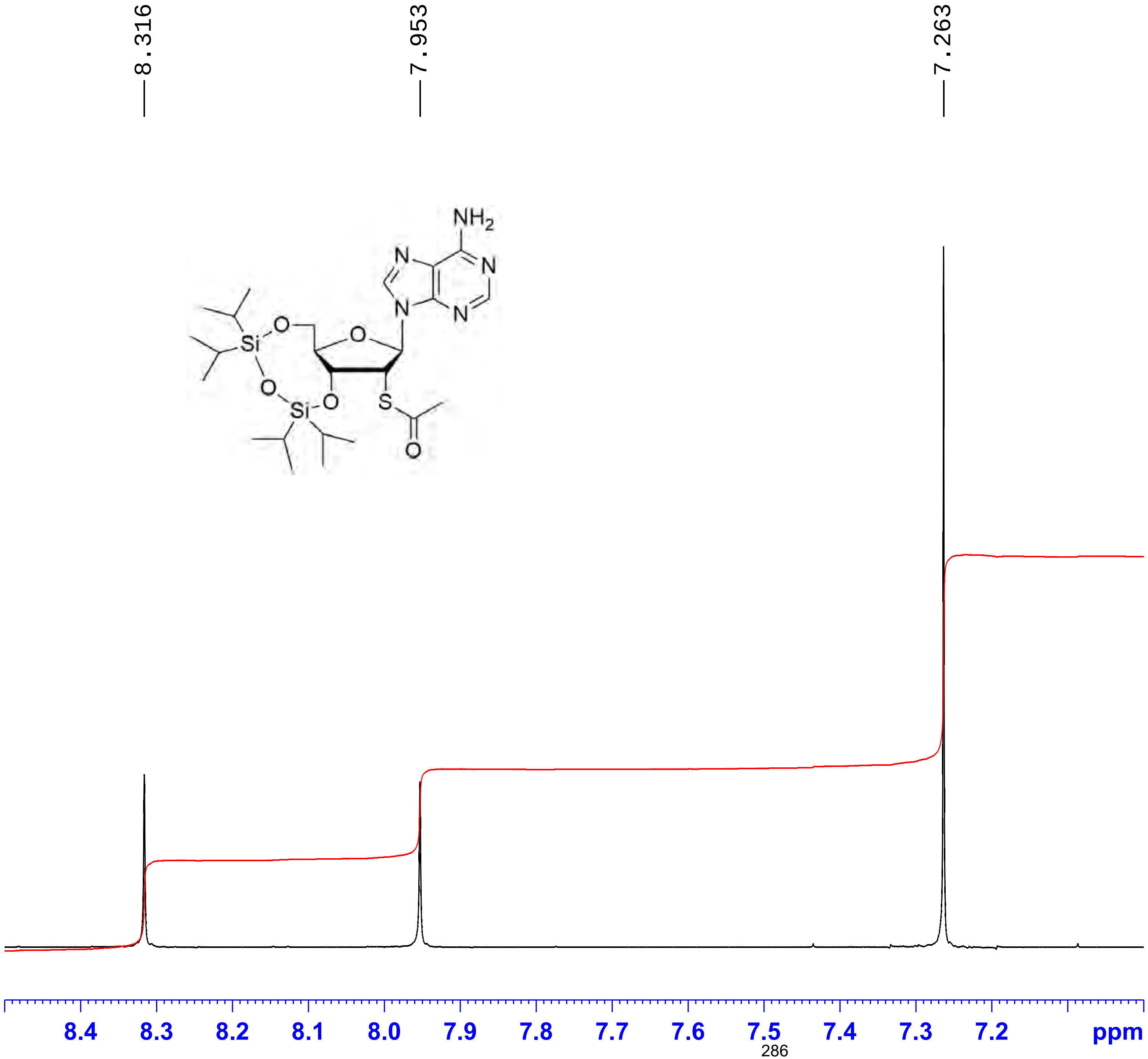


```
Current Data Parameters
NAME              LH-I-59
EXPNO                  20
PROCNO                  1

F2 - Acquisition Parameters
Date_            20220210
Time                11.21 h
INSTRUM             spect
PROBHD   Z114607_0188 (
PULPROG              zg30
TD                 180286
SOLVENT             CDCl3
NS                     16
DS                      0
SWH             18028.846 Hz
FIDRES           0.200003 Hz
AQ              4.9999318 sec
RG                   97.5
DW                 27.733 usec
DE                   8.00 usec
TE                  300.0 K
D1             0.10000000 sec
TD0                     1
SFO1          600.1337060 MHz
NUC1                   1H
P0                   3.33 usec
P1                  10.00 usec
PLW1          26.60000038 W

F2 - Processing parameters
SI                 262144
SF            600.1300124 MHz
WDW                    EM
SSB      0
LB                   0.10 Hz
GB       0
PC                   1.00
```

Expanded region of the ^{1}H NMR spectrum of compound 31

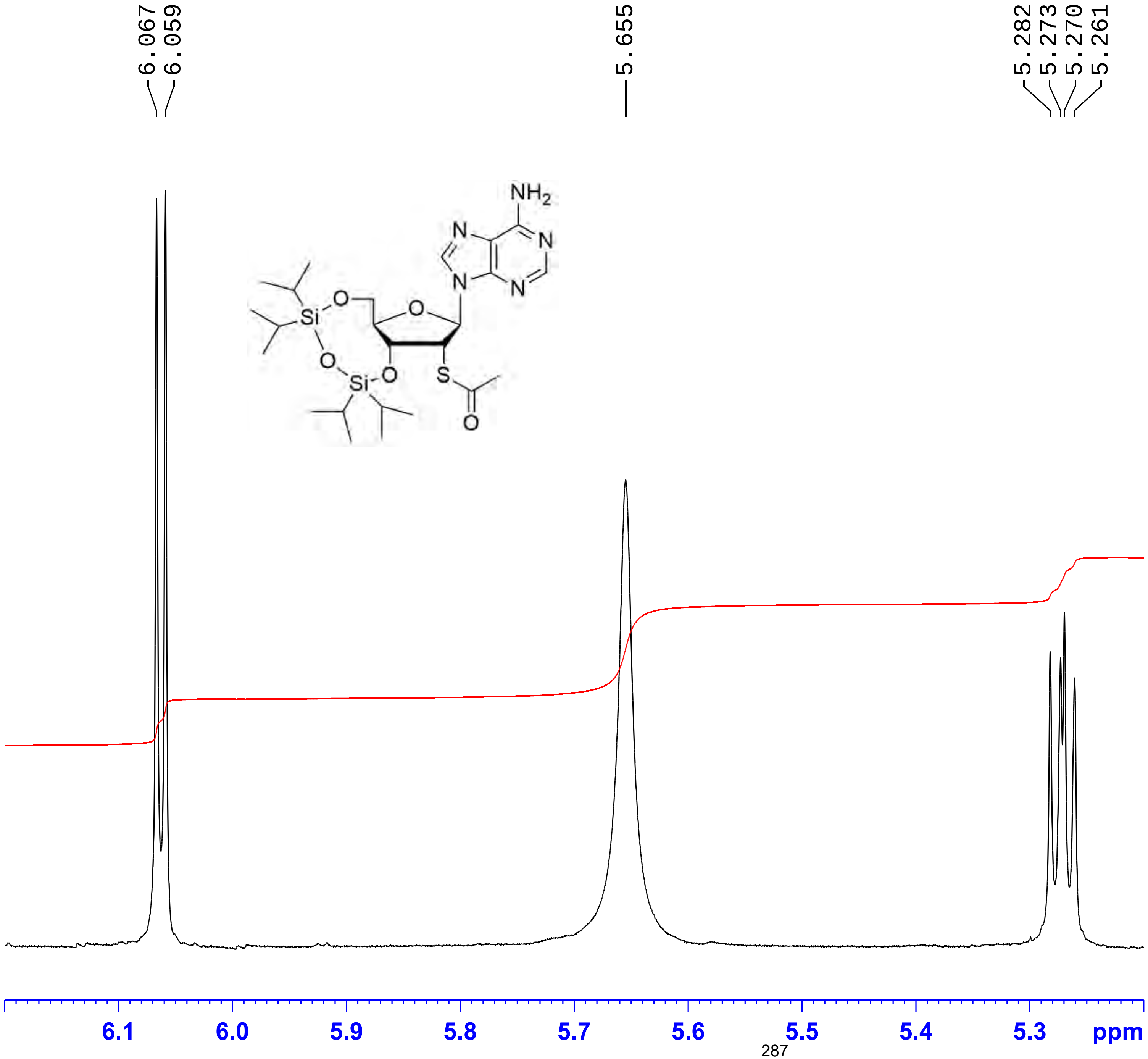

BRUKER

Current Data Parameters
NAME LH-I-59
EXPNO 20
PROCNO 1

F2 - Acquisition Parameters
Date_ 20220210
Time 11.21 h
INSTRUM spect
PROBHD Z114607_0188 (
PULPROG zg30
TD 180286
SOLVENT CDCl3
NS 16
DS 0
SWH 18028.846 Hz
FIDRES 0.200003 Hz
AQ 4.9999318 sec
RG 97.5
DW 27.733 usec
DE 8.00 usec
TE 300.0 K
D1 0.10000000 sec
TD0 1
SFO1 600.1337060 MHz
NUC1 1H
P0 3.33 usec
P1 10.00 usec
PLW1 26.60000038 W

F2 - Processing parameters
SI 262144
SF 600.1300124 MHz
WDW EM
SSB 0
LB 0.10 Hz
GB 0
PC 1.00

Expanded region of the ^{1}H NMR spectrum of compound 31

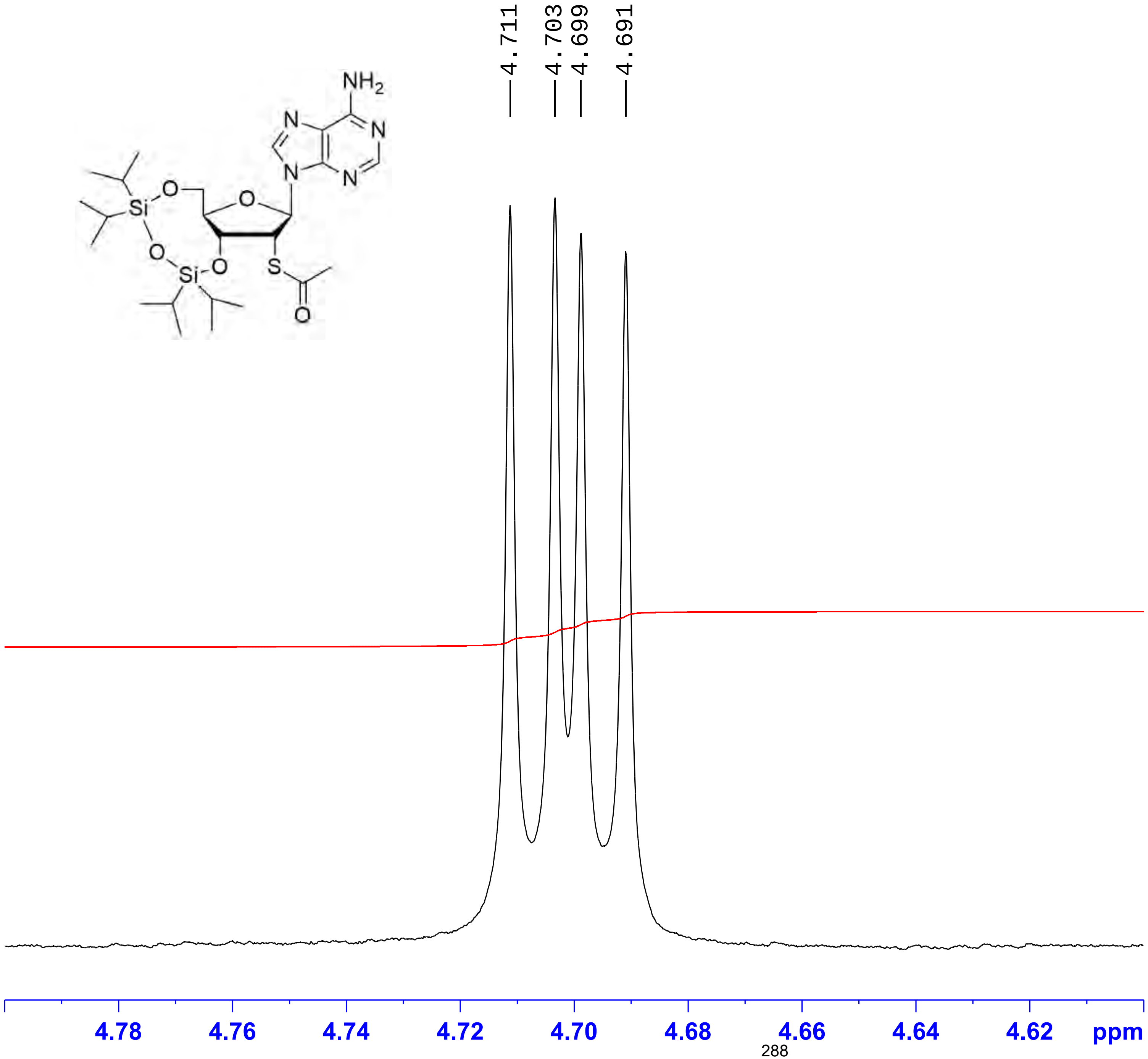

BRUKER

Current Data Parameters
NAME LH-I-59
EXPNO 20
PROCNO 1

F2 - Acquisition Parameters
Date_ 20220210
Time 11.21 h
INSTRUM spect
PROBHD Z114607_0188 (
PULPROG zg30
TD 180286
SOLVENT CDCl3
NS 16
DS 0
SWH 18028.846 Hz
FIDRES 0.200003 Hz
AQ 4.9999318 sec
RG 97.5
DW 27.733 usec
DE 8.00 usec
TE 300.0 K
D1 0.10000000 sec
TD0 1
SFO1 600.1337060 MHz
NUC1 1H
P0 3.33 usec
P1 10.00 usec
PLW1 26.60000038 W

F2 - Processing parameters
SI 262144
SF 600.1300124 MHz
WDW EM
SSB 0
LB 0.10 Hz
GB 0
PC 1.00

Expanded region of the ^{1}H NMR spectrum of compound 31

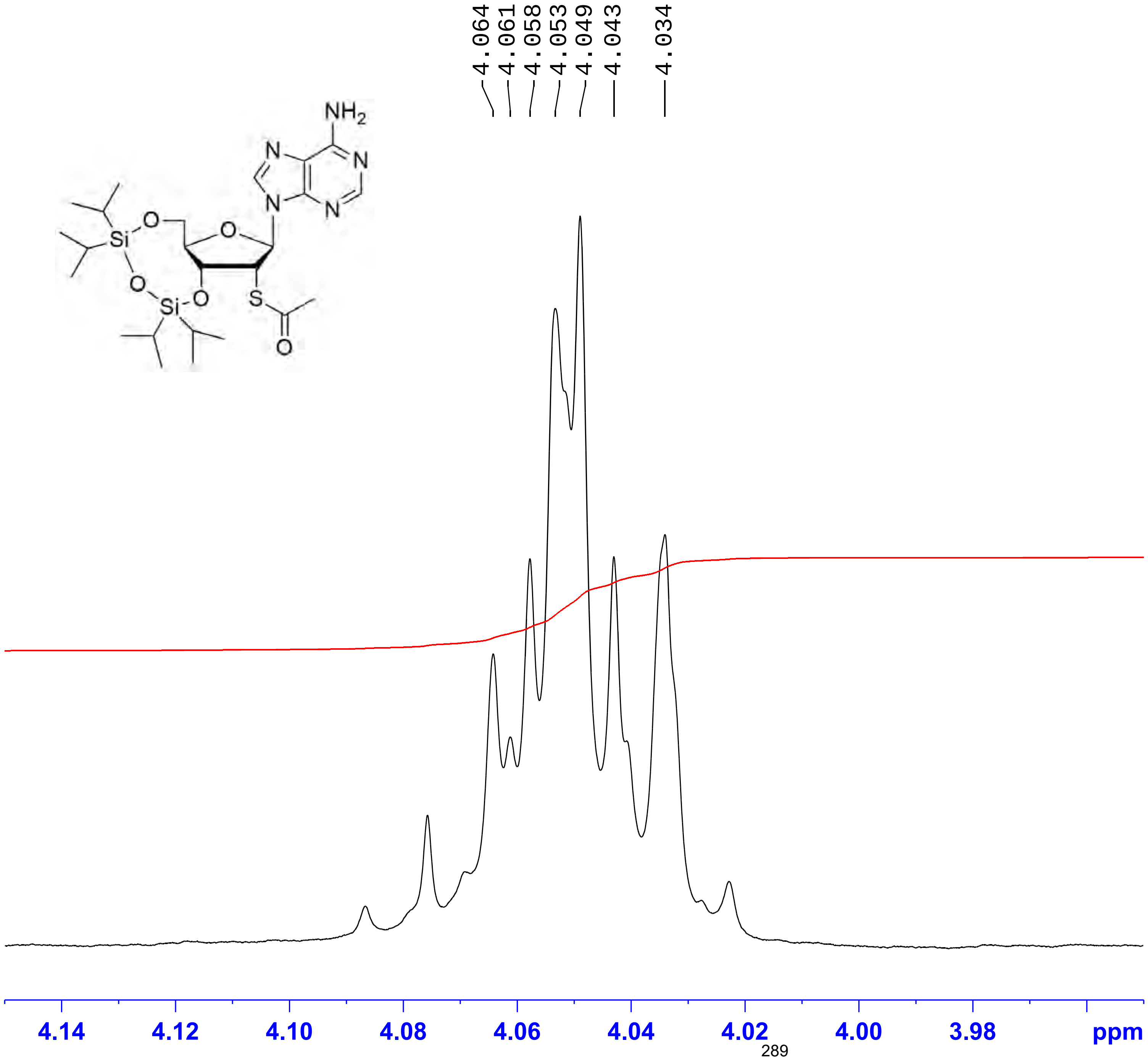


```
Current Data Parameters
NAME              LH-I-59
EXPNO                  20
PROCNO                  1

F2 - Acquisition Parameters
Date_            20220210
Time                11.21 h
INSTRUM             spect
PROBHD   Z114607_0188 (
PULPROG              zg30
TD                 180286
SOLVENT             CDCl3
NS                     16
DS                      0
SWH             18028.846 Hz
FIDRES           0.200003 Hz
AQ              4.9999318 sec
RG                   97.5
DW                 27.733 usec
DE                   8.00 usec
TE                  300.0 K
D1             0.10000000 sec
TD0                     1
SFO1          600.1337060 MHz
NUC1                   1H
P0                   3.33 usec
P1                  10.00 usec
PLW1          26.60000038 W

F2 - Processing parameters
SI                 262144
SF            600.1300124 MHz
WDW                    EM
SSB      0
LB                   0.10 Hz
GB       0
PC                   1.00
```

Expanded region of the ¹H NMR spectrum of compound 31

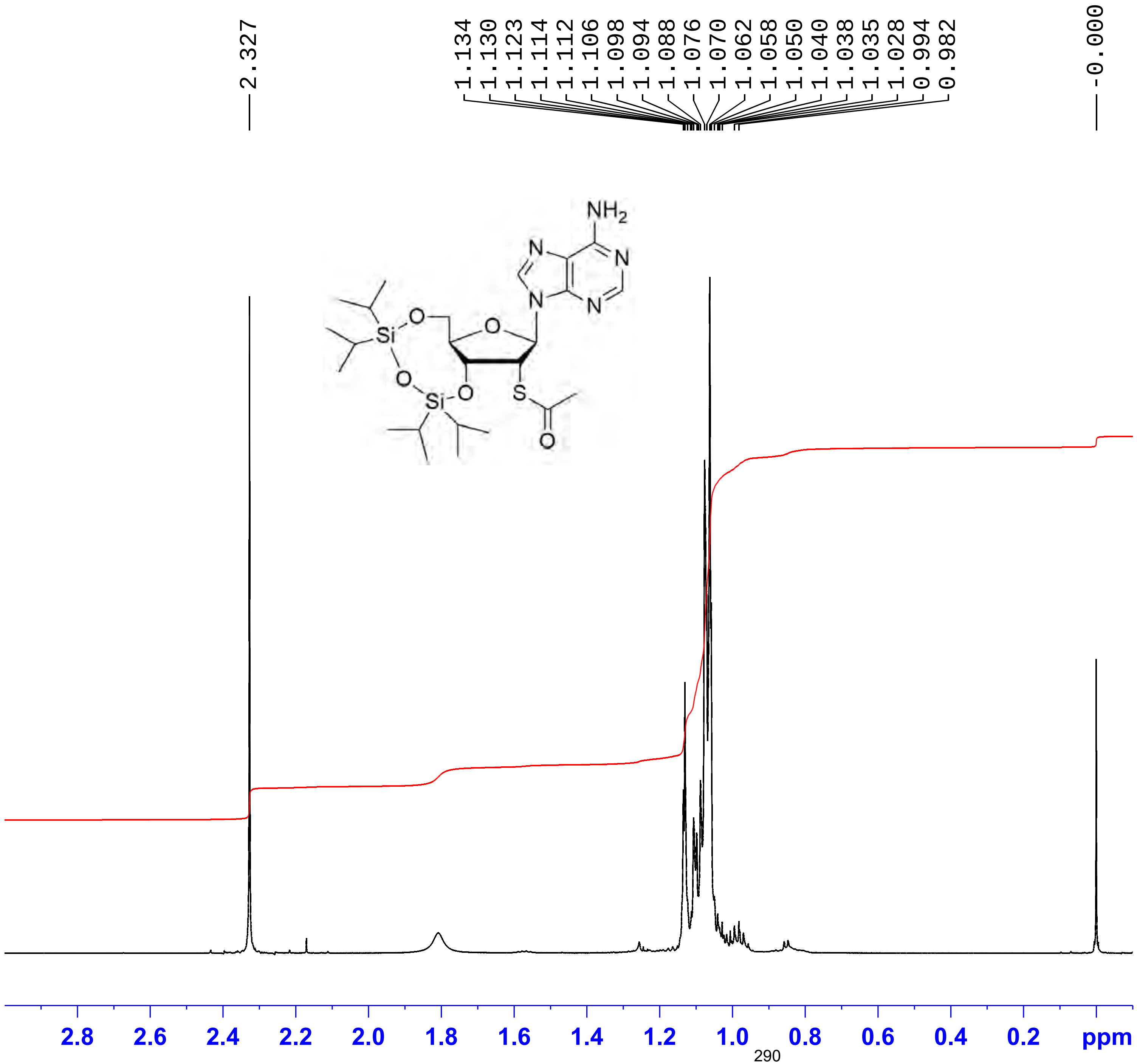

BRUKER

```
Current Data Parameters
NAME              LH-I-59
EXPNO                  20
PROCNO                  1

F2 - Acquisition Parameters
Date_            20220210
Time                11.21 h
INSTRUM             spect
PROBHD   Z114607_0188 (
PULPROG              zg30
TD                 180286
SOLVENT             CDCl3
NS                     16
DS                      0
SWH             18028.846 Hz
FIDRES           0.200003 Hz
AQ              4.9999318 sec
RG                   97.5
DW                 27.733 usec
DE                   8.00 usec
TE                  300.0 K
D1             0.10000000 sec
TD0                     1
SFO1          600.1337060 MHz
NUC1                   1H
P0                   3.33 usec
P1                  10.00 usec
PLW1          26.60000038 W

F2 - Processing parameters
SI                 262144
SF            600.1300124 MHz
WDW                    EM
SSB      0
LB                   0.10 Hz
GB       0
PC                   1.00
```

^{13}C NMR spectrum of compound 31

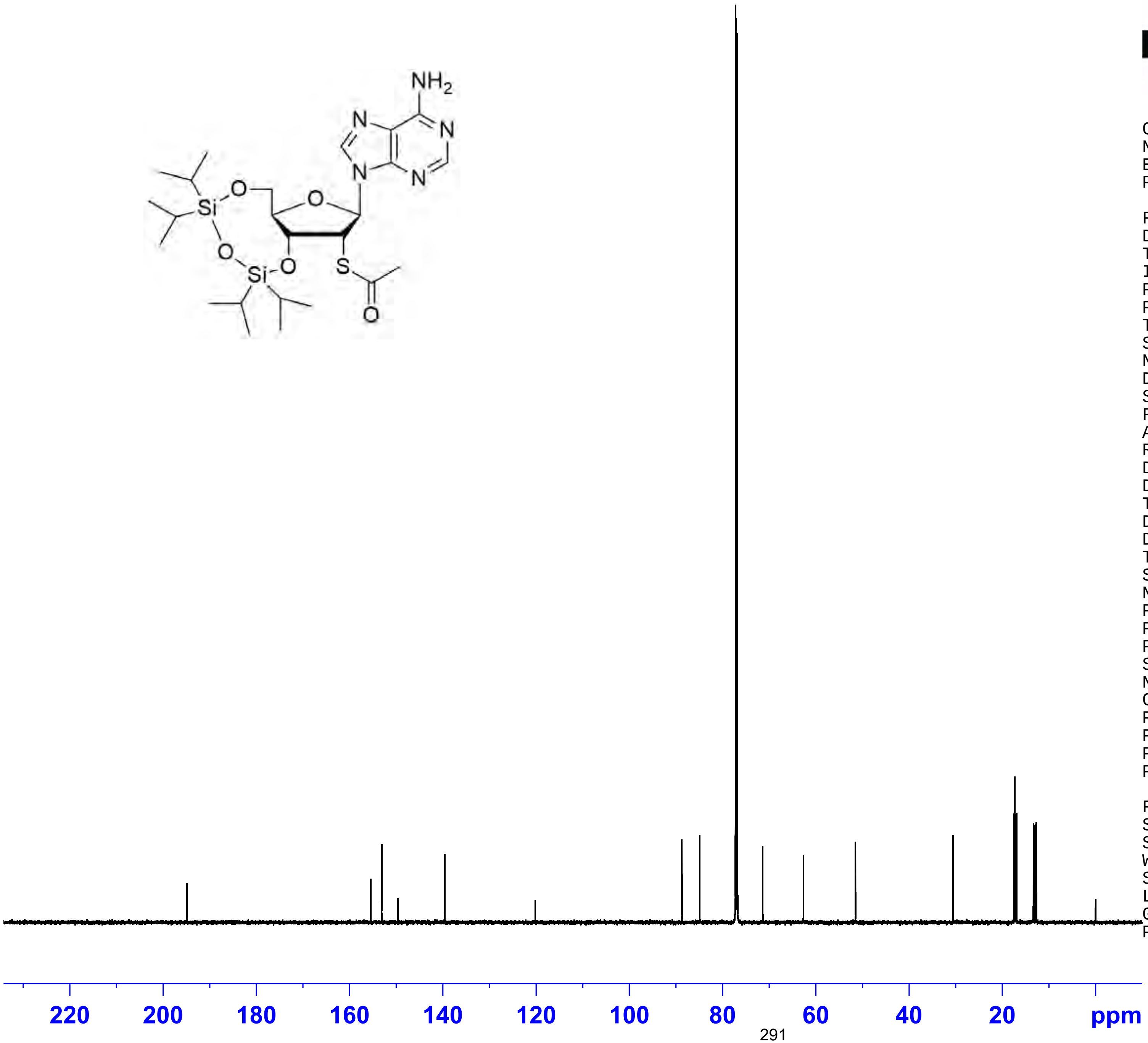


```
Current Data Parameters
NAME                LH-I-59
EXPNO                    21
PROCNO                    1

F2 - Acquisition Parameters
Date_              20220210
Time                  13.12 h
INSTRUM               spect
PROBHD   Z114607_0188 (
PULPROG             zgpg30
TD                  119044
SOLVENT              CDCl3
NS                     2500
DS                        4
SWH              37500.000 Hz
FIDRES            0.630019 Hz
AQ               1.5872533 sec
RG                   186.92
DW                   13.333 usec
DE                     6.53 usec
TE                    300.0 K
D1              1.00000000 sec
D11             0.03000000 sec
TD0                       1
SFO1           150.9194058 MHz
NUC1                    13C
P0                     3.93 usec
P1                    11.80 usec
PLW1           85.00000000 W
SFO2           600.1324005 MHz
NUC2                     1H
CPDPRG[2            waltz64
PCPD2                 70.00 usec
PLW2           27.00000000 W
PLW12           0.57327998 W
PLW13           0.28836000 W

F2 - Processing parameters
SI                   131072
SF             150.9028115 MHz
WDW                      EM
SSB      0
LB                     1.00 Hz
GB       0
PC                     1.40
```

Expanded region of the ^{13}C NMR spectrum of compound 31

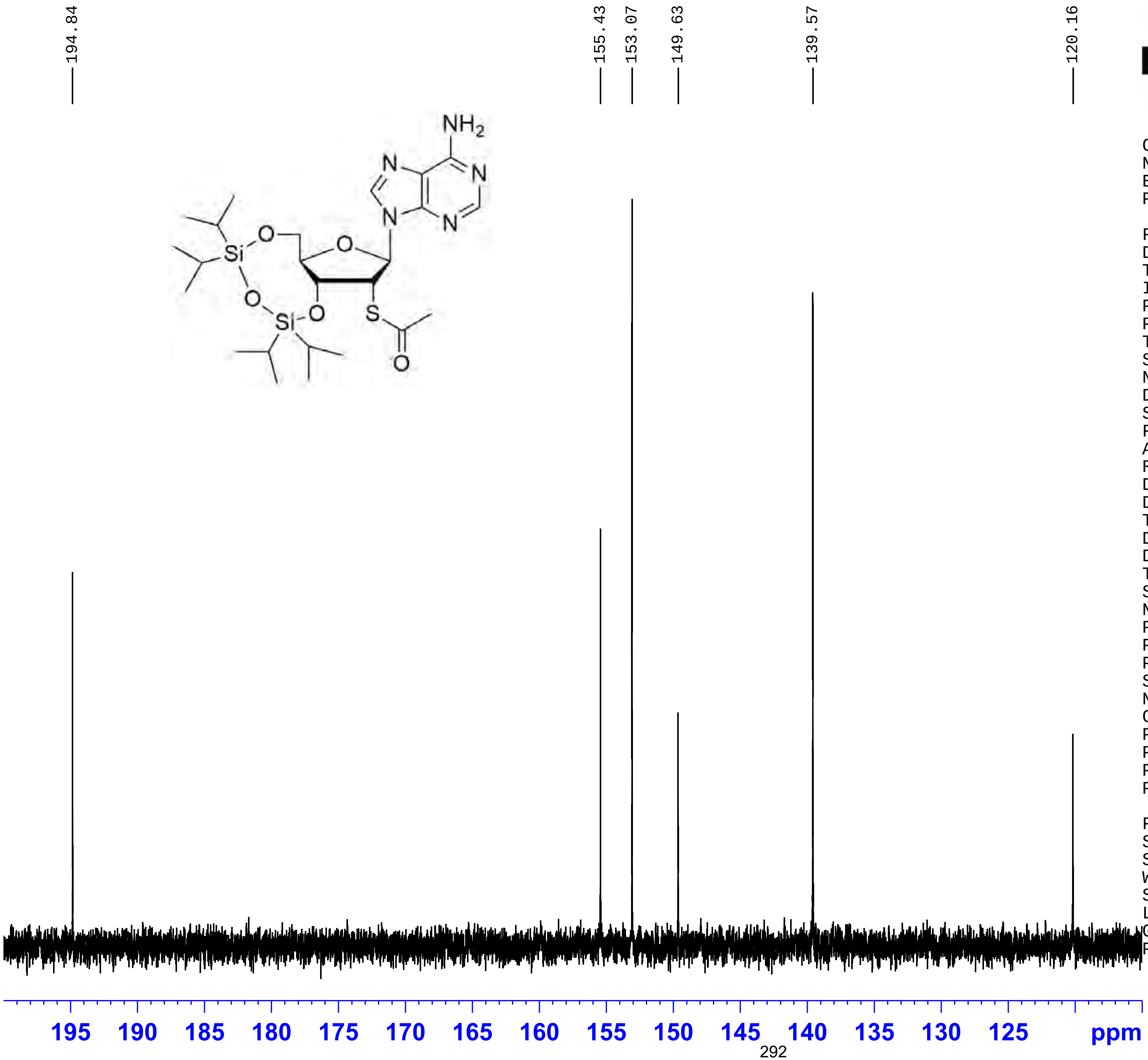

Current Data Parameters
NAME LH-I-59
EXPNO 21
PROCNO 1

F2 - Acquisition Parameters
Date_ 20220210
Time 13.12 h
INSTRUM spect
PROBHD Z114607_0188 (
PULPROG zgpg30
TD 119044
SOLVENT CDCl3
NS 2500
DS 4
SWH 37500.000 Hz
FIDRES 0.630019 Hz
AQ 1.5872533 sec
RG 186.92
DW 13.333 usec
DE 6.53 usec
TE 300.0 K
D1 1.00000000 sec
D11 0.03000000 sec
TD0 1
SFO1 150.9194058 MHz
NUC1 13C
P0 3.93 usec
P1 11.80 usec
PLW1 85.00000000 W
SFO2 600.1324005 MHz
NUC2 1H
CPDPRG[2 waltz64
PCPD2 70.00 usec
PLW2 27.00000000 W
PLW12 0.57327998 W
PLW13 0.28836000 W

F2 - Processing parameters
SI 131072
SF 150.9028115 MHz
WDW EM
SSB 0
LB 1.00 Hz
GB 0
PC 1.40

Expanded region of the ^{13}C NMR spectrum of compound 31

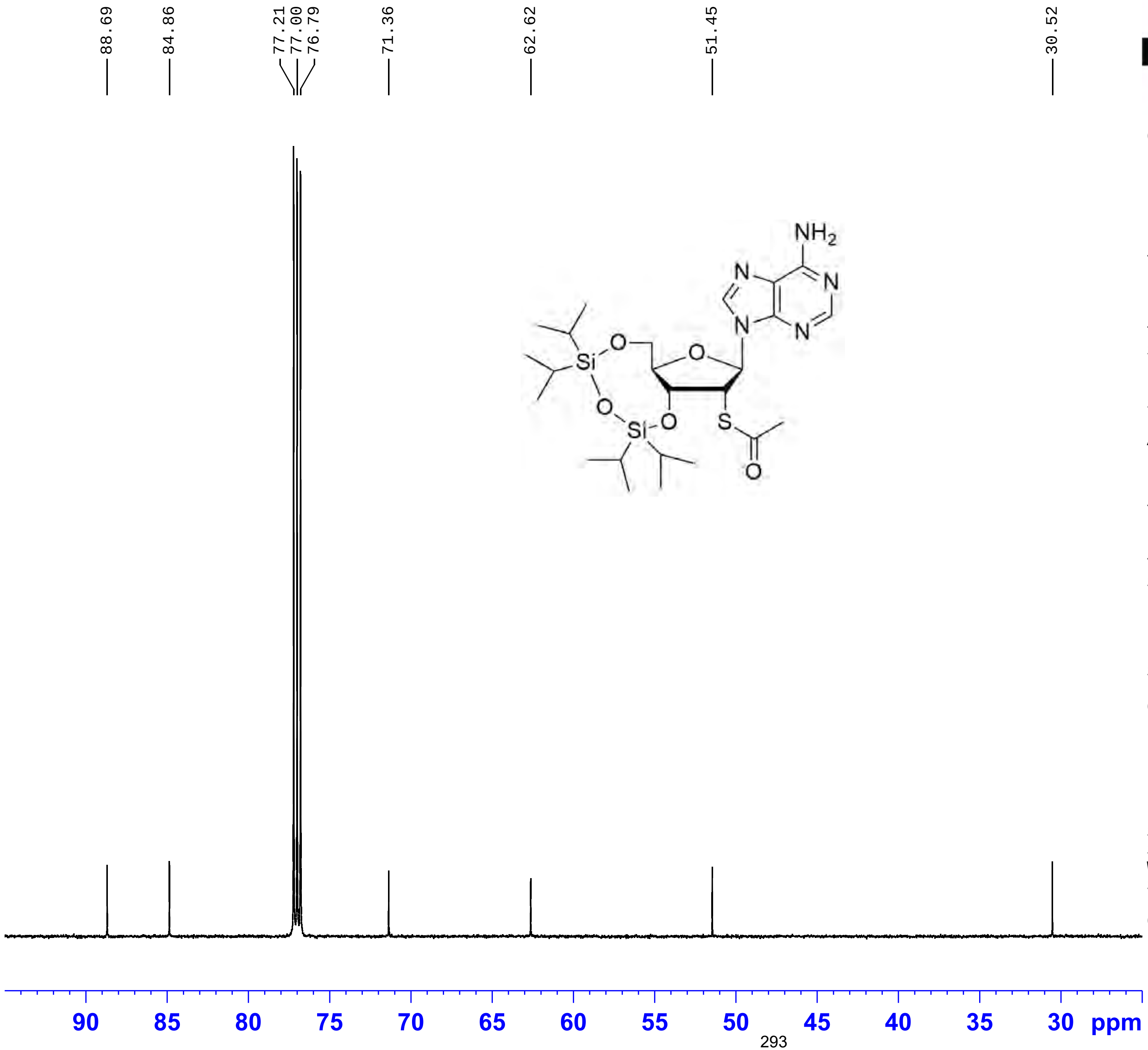


```
Current Data Parameters
NAME              LH-I-59
EXPNO                  21
PROCNO                  1

F2 - Acquisition Parameters
Date_            20220210
Time                13.12 h
INSTRUM             spect
PROBHD     Z114607_0188 (
PULPROG           zgpg30
TD                119044
SOLVENT             CDCl3
NS                   2500
DS                      4
SWH             37500.000 Hz
FIDRES           0.630019 Hz
AQ              1.5872533 sec
RG                 186.92
DW                 13.333 usec
DE                   6.53 usec
TE                  300.0 K
D1             1.00000000 sec
D11            0.03000000 sec
TD0                     1
SFO1          150.9194058 MHz
NUC1                  13C
P0                   3.93 usec
P1                  11.80 usec
PLW1          85.00000000 W
SFO2          600.1324005 MHz
NUC2                   1H
CPDPRG[2          waltz64
PCPD2               70.00 usec
PLW2          27.00000000 W
PLW12          0.57327998 W
PLW13          0.28836000 W

F2 - Processing parameters
SI                 131072
SF            150.9028115 MHz
WDW                    EM
SSB          0
LB                   1.00 Hz
GB           0
PC                   1.40
```

Expanded region of the ^{13}C NMR spectrum of compound 31

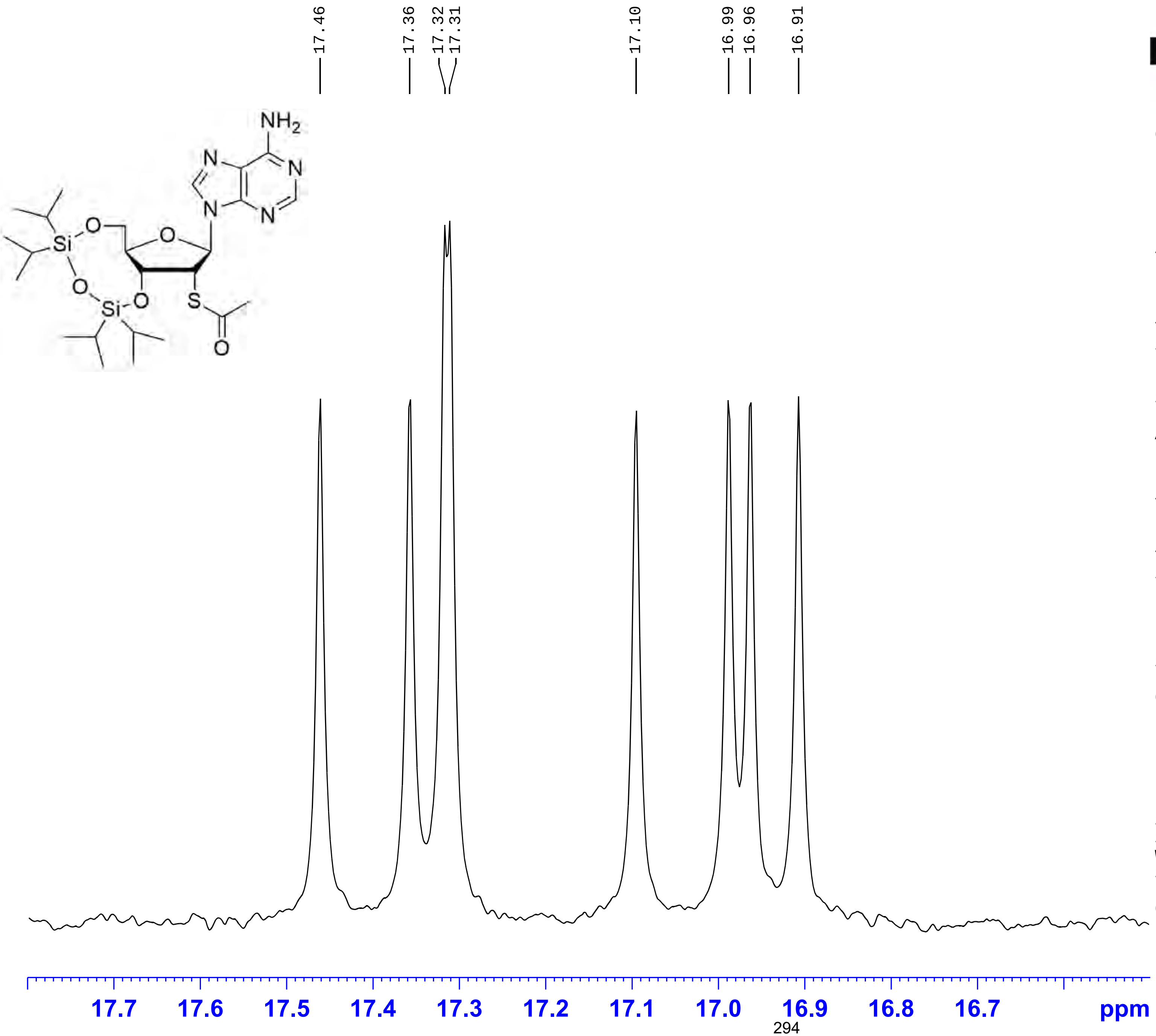

Current Data Parameters
NAME LH-I-59
EXPNO 21
PROCNO 1

F2 - Acquisition Parameters
Date_ 20220210
Time 13.12 h
INSTRUM spect
PROBHD Z114607_0188 (
PULPROG zgpg30
TD 119044
SOLVENT CDCl3
NS 2500
DS 4
SWH 37500.000 Hz
FIDRES 0.630019 Hz
AQ 1.5872533 sec
RG 186.92
DW 13.333 usec
DE 6.53 usec
TE 300.0 K
D1 1.00000000 sec
D11 0.03000000 sec
TD0 1
SFO1 150.9194058 MHz
NUC1 13C
P0 3.93 usec
P1 11.80 usec
PLW1 85.00000000 W
SFO2 600.1324005 MHz
NUC2 1H
CPDPRG[2 waltz64
PCPD2 70.00 usec
PLW2 27.00000000 W
PLW12 0.57327998 W
PLW13 0.28836000 W

F2 - Processing parameters
SI 131072
SF 150.9028115 MHz
WDW EM
SSB 0
LB 1.00 Hz
GB 0
PC 1.40

Expanded region of the ^{13}C NMR spectrum of compound 31

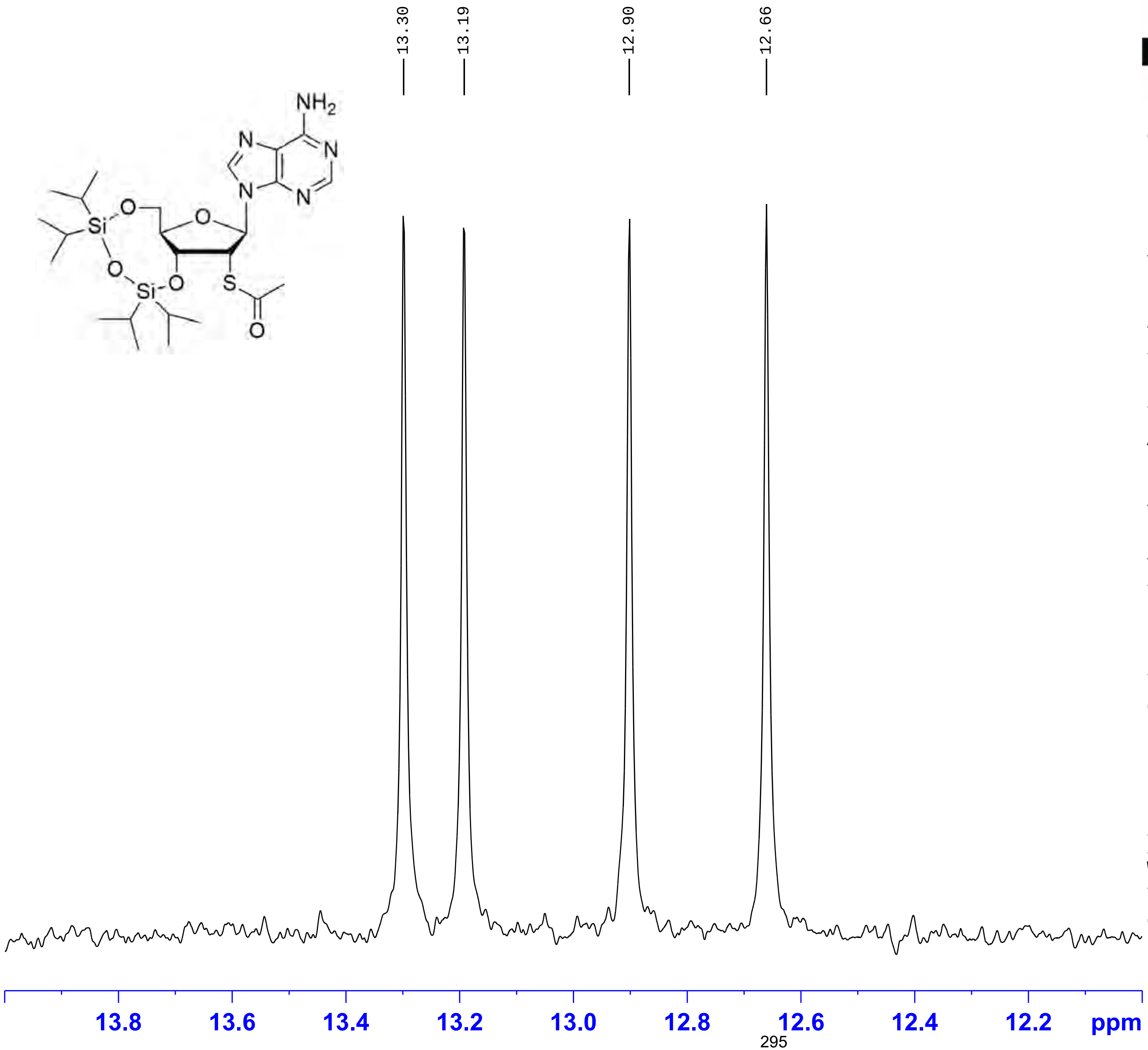


```
Current Data Parameters
NAME              LH-I-59
EXPNO                  21
PROCNO                  1

F2 - Acquisition Parameters
Date_            20220210
Time                13.12 h
INSTRUM             spect
PROBHD   Z114607_0188 (
PULPROG           zgpg30
TD                119044
SOLVENT            CDCl3
NS                  2500
DS                     4
SWH            37500.000 Hz
FIDRES          0.630019 Hz
AQ             1.5872533 sec
RG                186.92
DW                13.333 usec
DE                  6.53 usec
TE                 300.0 K
D1            1.00000000 sec
D11           0.03000000 sec
TD0                    1
SFO1         150.9194058 MHz
NUC1                 13C
P0                  3.93 usec
P1                 11.80 usec
PLW1         85.00000000 W
SFO2         600.1324005 MHz
NUC2                  1H
CPDPRG[2         waltz64
PCPD2              70.00 usec
PLW2         27.00000000 W
PLW12         0.57327998 W
PLW13         0.28836000 W

F2 - Processing parameters
SI                131072
SF           150.9028115 MHz
WDW                   EM
SSB        0
LB                  1.00 Hz
GB         0
PC                  1.40
```

^{13}C DEPT-135 NMR spectrum of compound 31

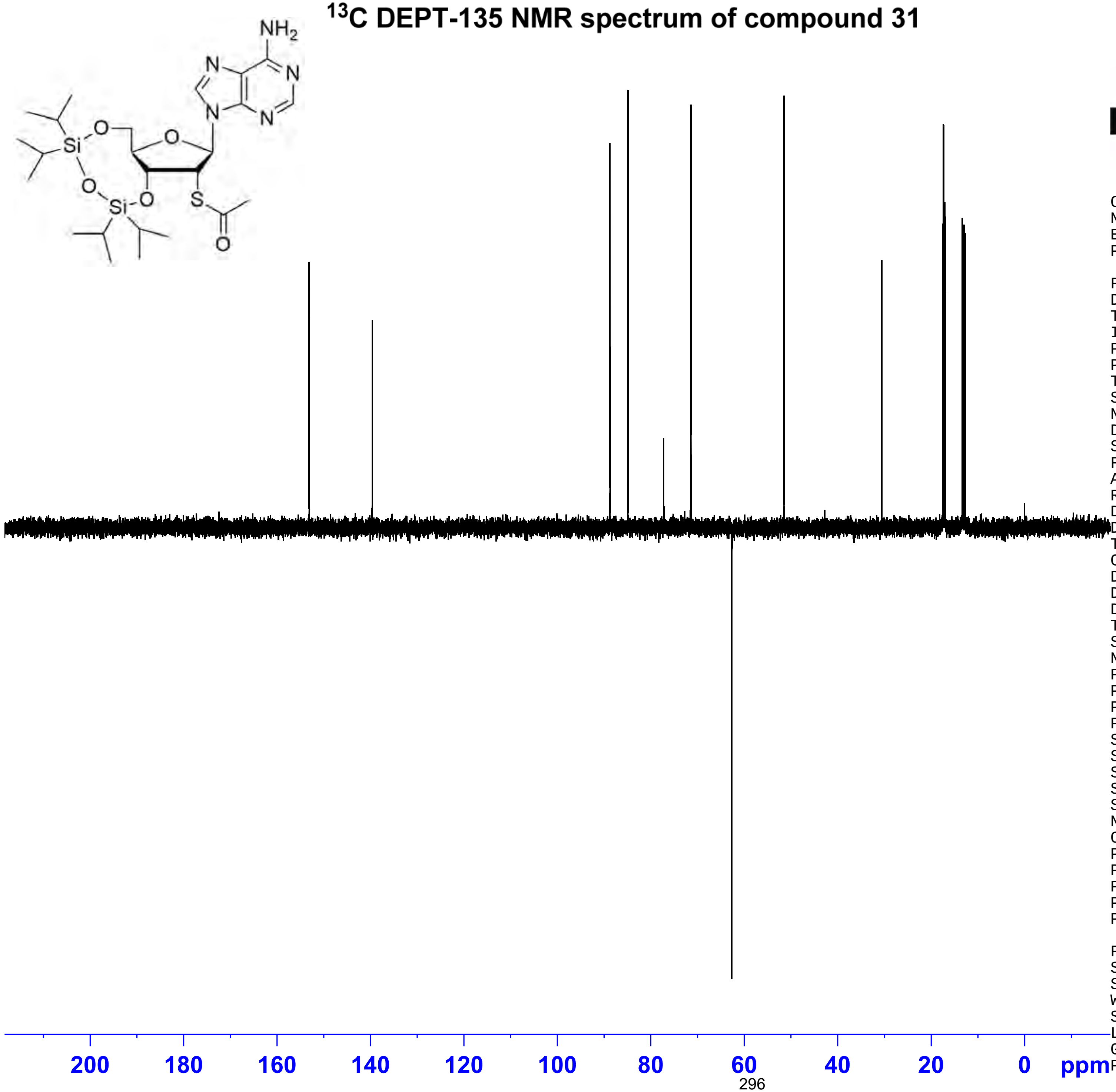

Current Data Parameters
NAME LH-I-59
EXPNO 22
PROCNO 1

F2 - Acquisition Parameters

Date_	20220210	
Time	13.58	h
INSTRUM	spect	
PROBHD	Z114607_0188 (	
PULPROG	deptsp135.b	
TD	119044	
SOLVENT	CDCl3	
NS	1000	
DS	4	
SWH	35714.285	Hz
FIDRES	0.600018	Hz
AQ	1.6666160	sec
RG	186.92	
DW	14.000	usec
DE	7.44	usec
TE	300.0	K
CNST2	145.0000000	
D1	1.00000000	sec
D2	0.00344828	sec
D12	0.00002000	sec
TD0	1	
SFO1	150.9178962	MHz
NUC1	13C	
P1	11.80	usec
P13	2000.00	usec
PLW0	0 W	
PLW1	85.00000000	W
SPNAM[5]	Crp60comp.4	
SPOAL5	0.500	
SPOFFS5	0 Hz	
SPW5	18.08300018	W
SFO2	600.1324005	MHz
NUC2	1H	
CPDPRG[2	waltz64	
P3	10.20	usec
P4	20.40	usec
PCPD2	70.00	usec
PLW2	27.00000000	W
PLW12	0.57327998	W

F2 - Processing parameters

SI	131072	
SF	150.9028085	MHz
WDW	EM	
SSB	0	
LB	1.00	Hz
GB	0	
PC	1.40	

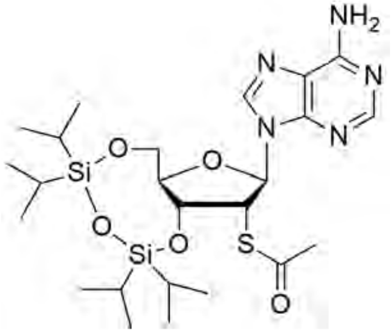

^{1}H-^{1}H COSY NMR spectrum of compound 31

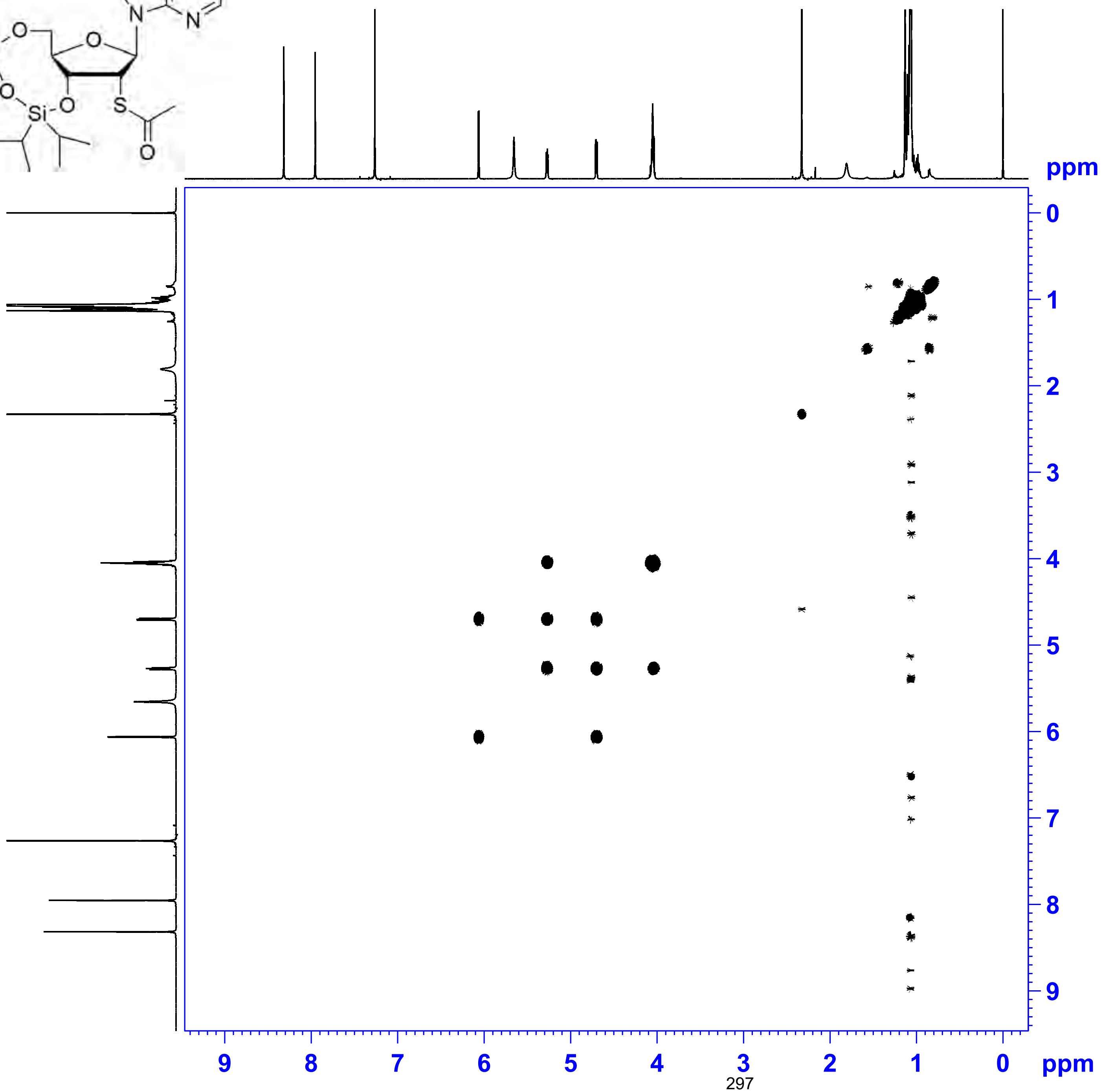

BRUKER

Current Data Parameters
NAME LH-I-59
EXPNO 23
PROCNO 1

F2 - Acquisition Parameters
Date_ 20220210
Time 14.09 h
INSTRUM spect
PROBHD Z114607_0188 (
PULPROG cosygpmfppqf
TD 2048
SOLVENT CDCl3
NS 2
DS 8
SWH 5854.801 Hz
FIDRES 5.717579 Hz
AQ 0.1748992 sec
RG 186.92
DW 85.400 usec
DE 6.50 usec
TE 300.0 K
D0 0.00000300 sec
D1 0.88531131 sec
D11 0.03000000 sec
D12 0.00002000 sec
D13 0.00000400 sec
D16 0.00020000 sec
IN0 0.00017080 sec
TDav 1
SFO1 600.1327645 MHz
NUC1 1H
P1 10.00 usec
P17 2500.00 usec
PLW1 26.60000038 W
PLW10 4.25600004 W
GPNAM[1] SMSQ10.100
GPZ1 16.00 %
GPNAM[2] SMSQ10.100
GPZ2 12.00 %
GPNAM[3] SMSQ10.100
GPZ3 40.00 %
P16 1000.00 usec

F1 - Acquisition parameters
TD 256
SFO1 600.1328 MHz
FIDRES 45.740631 Hz
SW 9.756 ppm
FnMODE QF

F2 - Processing parameters
SI 1024
SF 600.1300129 MHz
WDW SINE
SSB 0
LB 0 Hz
GB 0
PC 1.40

F1 - Processing parameters
SI 1024
MC2 QF
SF 600.1300128 MHz
WDW SINE
SSB 0
LB 0 Hz
GB 0

^{1}H-^{13}C HSQC NMR spectrum of compound 31

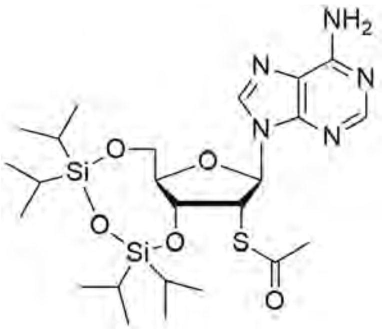

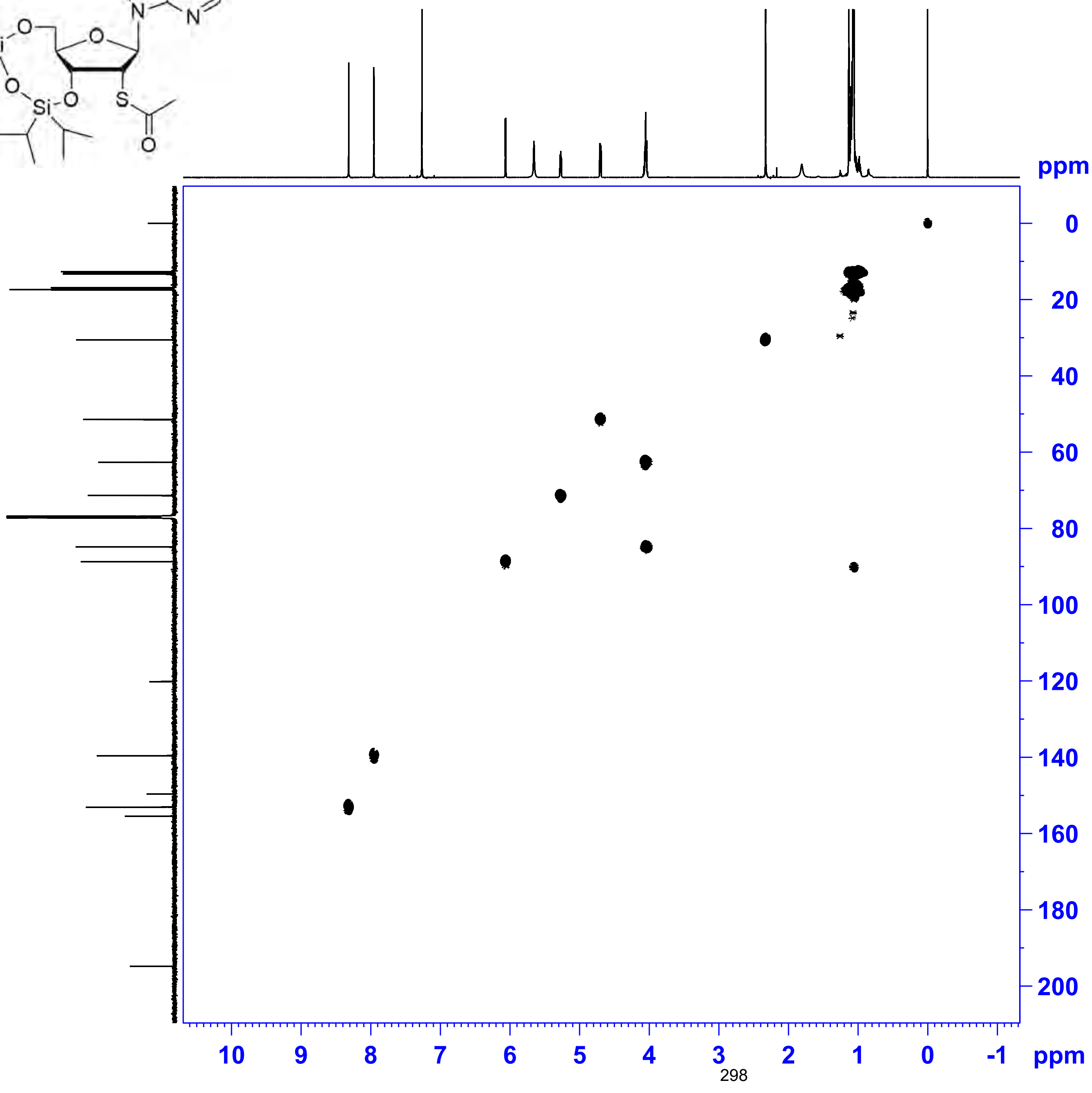


```
Current Data Parameters
NAME              LH-I-59
EXPNO                  24
PROCNO                  1

F2 - Acquisition Parameters
Date_            20220210
Time                14.18 h
INSTRUM             spect
PROBHD   Z114607_0188 (
PULPROG  hsqcedetgpsp.3
TD                   1024
SOLVENT             CDCl3
NS                      2
DS                     32
SWH              7211.539 Hz
FIDRES          14.085036 Hz
AQ              0.0709973 sec
RG                 186.92
DW                 69.333 usec
DE                   6.50 usec
TE                  300.3 K
CNST2         145.0000000
D0             0.00000300 sec
D1             0.80000001 sec
D4             0.00172414 sec
D11            0.03000000 sec
D16            0.00020000 sec
D21            0.00360000 sec
IN0            0.00001510 sec
TDav                    1
ZGOPTNS
SFO1          600.1328223 MHz
NUC1                   1H
P1                  10.00 usec
P2                  20.00 usec
PLW1          26.60000038 W
SFO2          150.9178988 MHz
NUC2                  13C
CPDPRG[2            garp4
P3                  11.80 usec
P14                500.00 usec
P31               1730.00 usec
PCPD2               60.00 usec
PLW0     0 W
PLW2          85.00000000 W
PLW12          3.28760004 W
SPNAM[3] Crp60,0.5,20.1
SPOAL3              0.500
SPOFFS3  0 Hz
SPW3          18.08300018 W
SPNAM[18  Crp60_xfilt.2
SPOAL18             0.500
SPOFFS18 0 Hz
SPW18          5.22629976 W
GPNAM[1]       SMSQ10.100
GPZ1                80.00 %
GPNAM[2]       SMSQ10.100
GPZ2                20.10 %
P16               1000.00 usec

F1 - Acquisition parameters
TD                    256
SFO1             150.9179 MHz
FIDRES         258.692047 Hz
SW                219.408 ppm
FnMODE     Echo-Antiecho

F2 - Processing parameters
SI                   1024
SF            600.1300105 MHz
WDW                 QSINE
SSB                     2
LB       0 Hz
GB       0
PC                   1.40

F1 - Processing parameters
SI                   1024
MC2          echo-antiecho
SF            150.9028135 MHz
WDW                 QSINE
SSB                     2
LB       0 Hz
GB       0
```

^{1}H NMR spectrum of compound 32

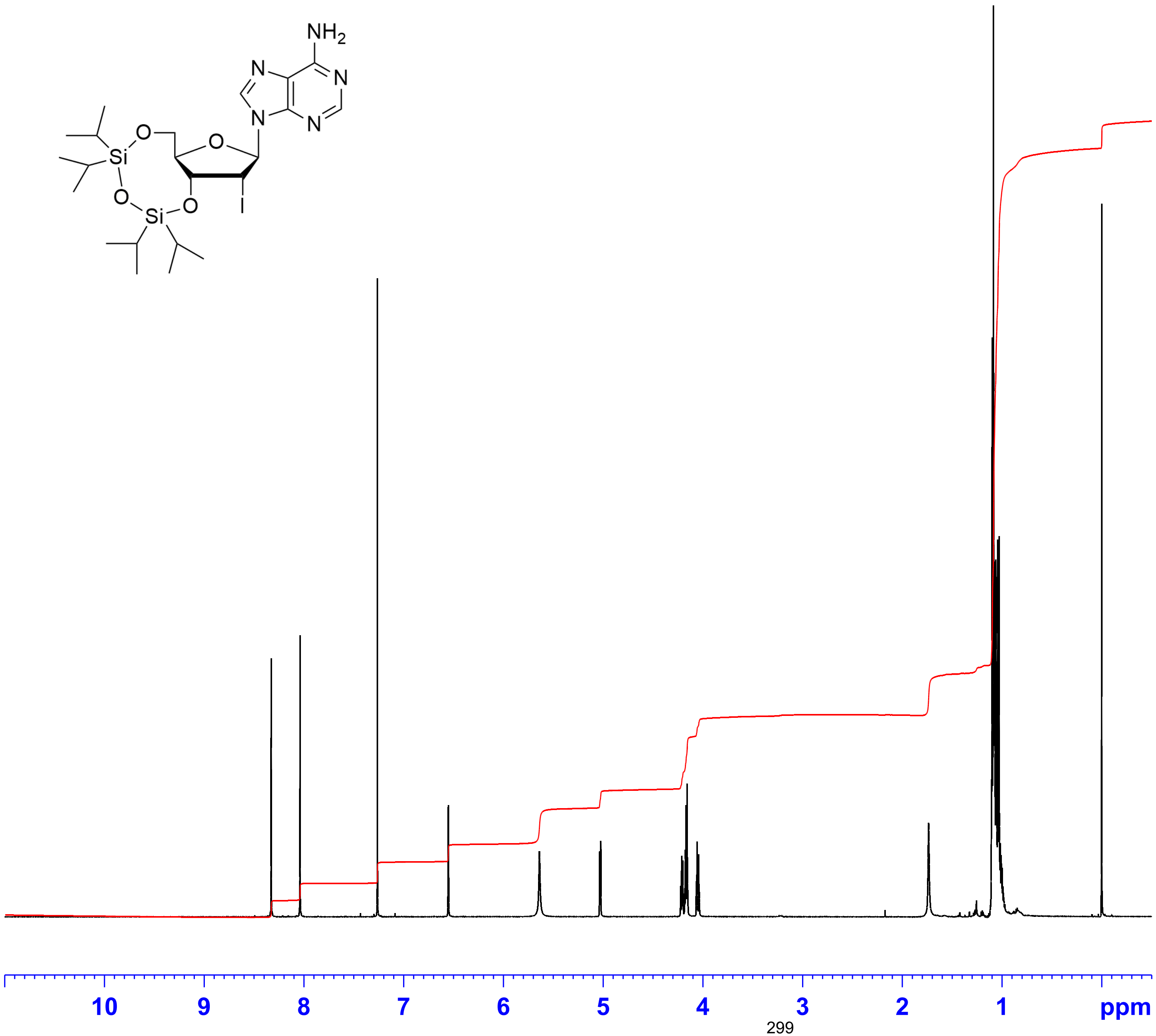

BRUKER

Current Data Parameters	
NAME	LH-I-60
EXPNO	10
PROCNO	1
F2 - Acquisition Parameters	
Date_	20220210
Time	17.11 h
INSTRUM	spect
PROBHD	Z114607_0188 (
PULPROG	zg30
TD	180286
SOLVENT	CDCl3
NS	16
DS	0
SWH	18028.846 Hz
FIDRES	0.200003 Hz
AQ	4.9999318 sec
RG	97.5
DW	27.733 usec
DE	8.00 usec
TE	300.0 K
D1	0.10000000 sec
TD0	1
SFO1	600.1337060 MHz
NUC1	1H
P0	3.33 usec
P1	10.00 usec
PLW1	26.60000038 W
F2 - Processing parameters	
SI	262144
SF	600.1300127 MHz
WDW	EM
SSB	0
LB	0.10 Hz
GB	0
PC	1.00

Expanded region of the ^{1}H NMR spectrum of compound 32

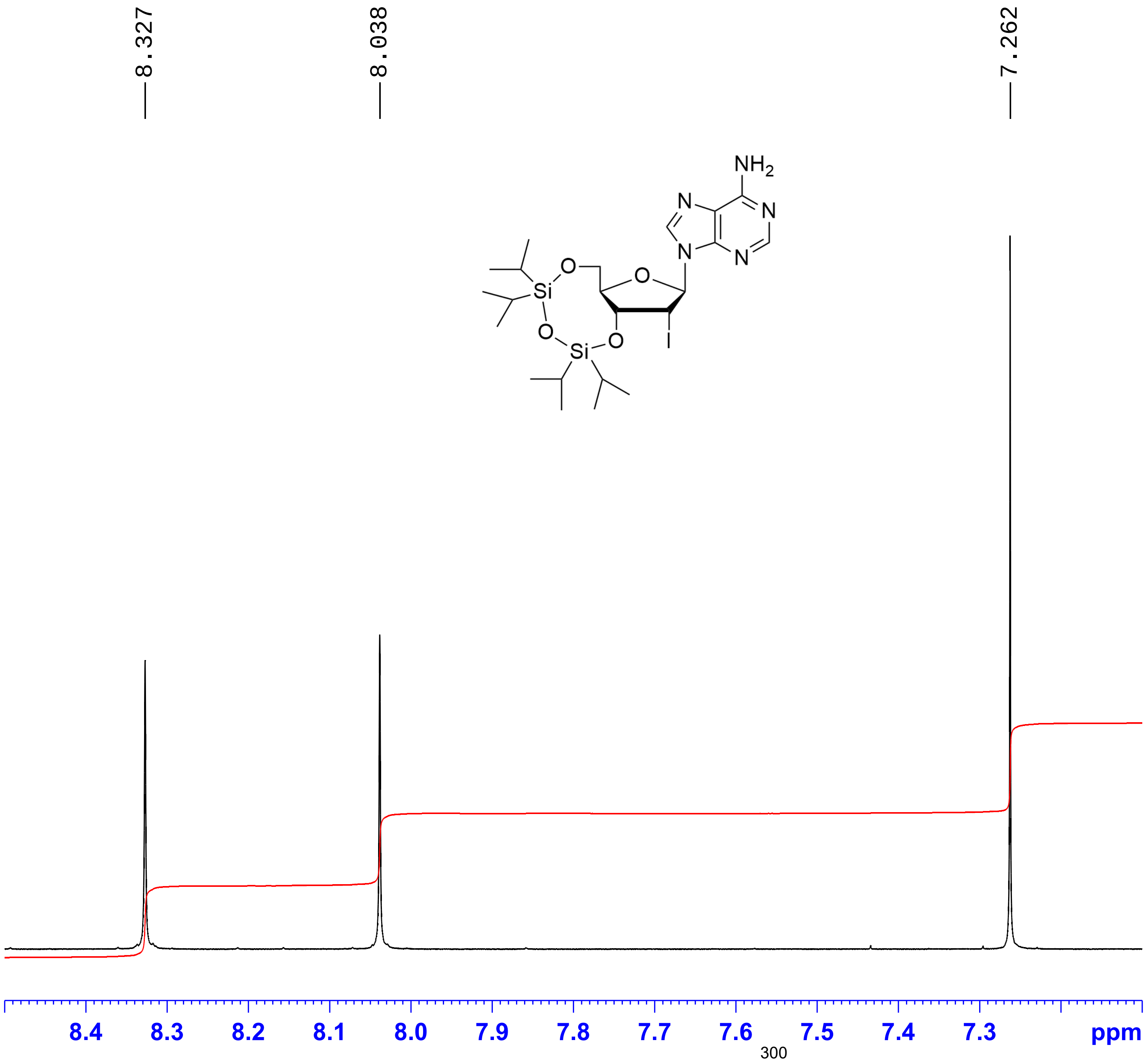


```
Current Data Parameters
NAME              LH-I-60
EXPNO                  10
PROCNO                  1

F2 - Acquisition Parameters
Date_            20220210
Time                17.11 h
INSTRUM             spect
PROBHD    Z114607_0188 (
PULPROG              zg30
TD                 180286
SOLVENT             CDCl3
NS                     16
DS                      0
SWH             18028.846 Hz
FIDRES           0.200003 Hz
AQ              4.9999318 sec
RG                   97.5
DW                 27.733 usec
DE                   8.00 usec
TE                  300.0 K
D1             0.10000000 sec
TD0                     1
SFO1          600.1337060 MHz
NUC1                   1H
P0                   3.33 usec
P1                  10.00 usec
PLW1          26.60000038 W

F2 - Processing parameters
SI                 262144
SF            600.1300127 MHz
WDW                    EM
SSB         0
LB                   0.10 Hz
GB          0
PC                   1.00
```

Expanded region of the ^{1}H NMR spectrum of compound 32

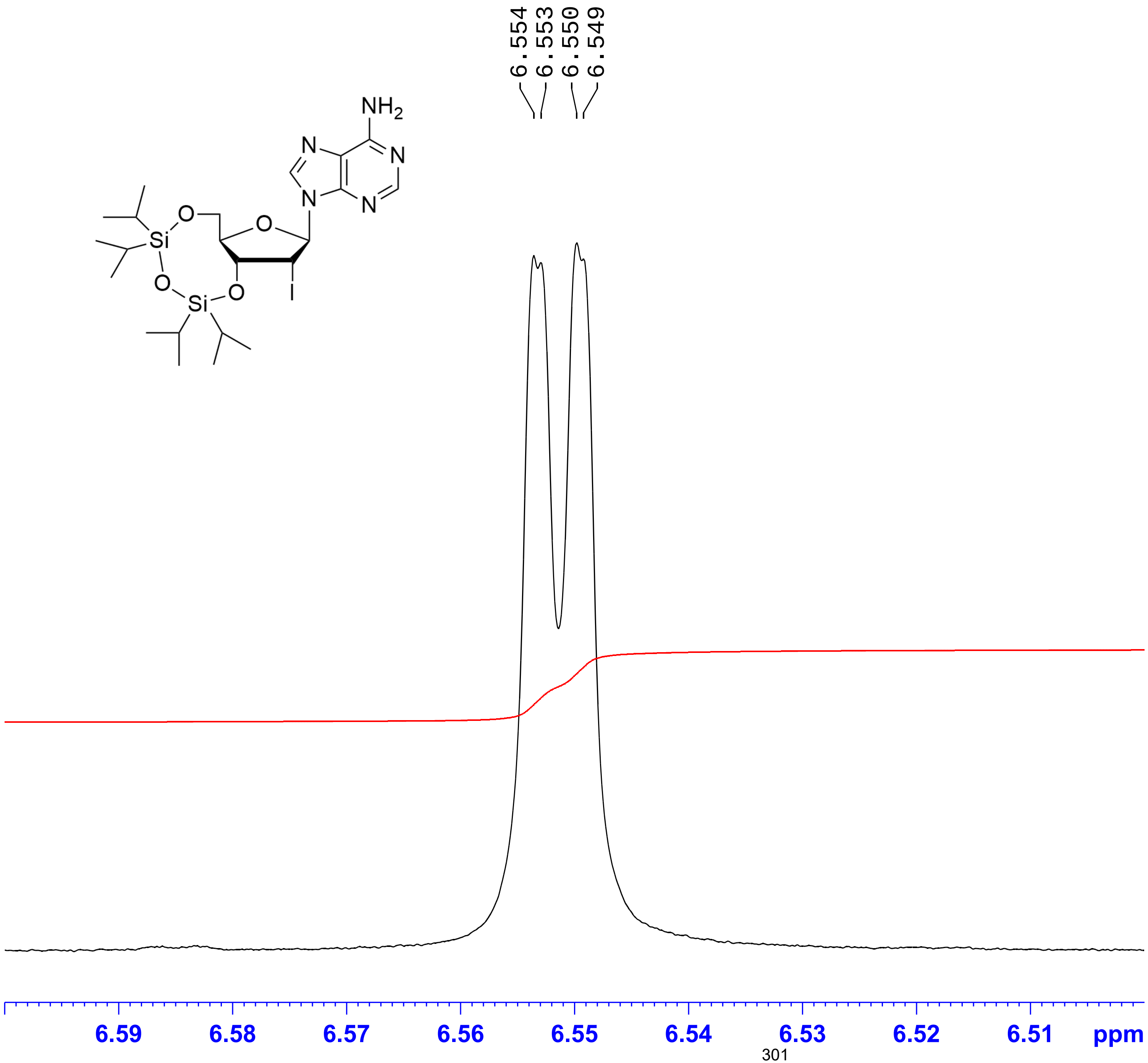

Current Data Parameters		
NAME	LH-I-60	
EXPNO	10	
PROCNO	1	
F2 - Acquisition Parameters		
Date_	20220210	
Time	17.11	h
INSTRUM	spect	
PROBHD	Z114607_0188 (	
PULPROG	zg30	
TD	180286	
SOLVENT	CDCl3	
NS	16	
DS	0	
SWH	18028.846	Hz
FIDRES	0.200003	Hz
AQ	4.9999318	sec
RG	97.5	
DW	27.733	usec
DE	8.00	usec
TE	300.0	K
D1	0.10000000	sec
TD0	1	
SFO1	600.1337060	MHz
NUC1	1H	
P0	3.33	usec
P1	10.00	usec
PLW1	26.60000038	W
F2 - Processing parameters		
SI	262144	
SF	600.1300127	MHz
WDW	EM	
SSB	0	
LB	0.10	Hz
GB	0	
PC	1.00	

Expanded region of the ^{1}H NMR spectrum of compound 32

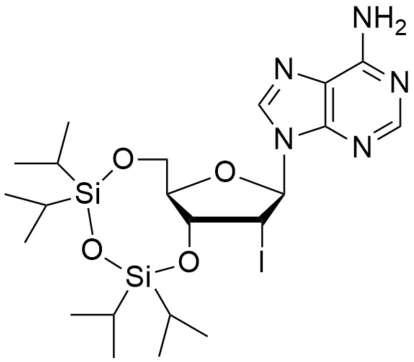

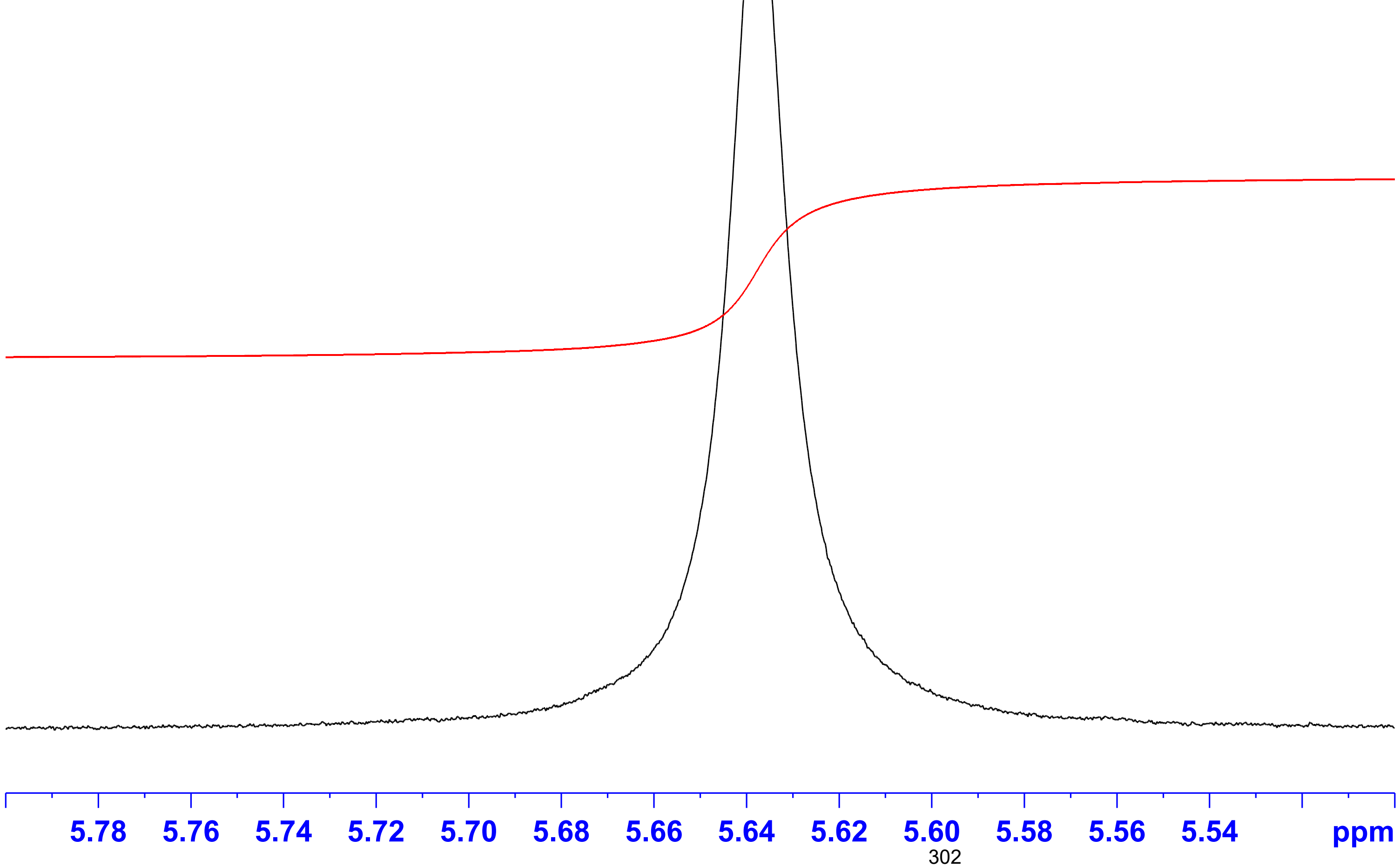

Current Data Parameters
NAME LH-I-60
EXPNO 10
PROCNO 1

F2 - Acquisition Parameters
Date_ 20220210
Time 17.11 h
INSTRUM spect
PROBHD Z114607_0188 (
PULPROG zg30
TD 180286
SOLVENT CDCl3
NS 16
DS 0
SWH 18028.846 Hz
FIDRES 0.200003 Hz
AQ 4.9999318 sec
RG 97.5
DW 27.733 usec
DE 8.00 usec
TE 300.0 K
D1 0.10000000 sec
TD0 1
SFO1 600.1337060 MHz
NUC1 1H
P0 3.33 usec
P1 10.00 usec
PLW1 26.60000038 W

F2 - Processing parameters
SI 262144
SF 600.1300127 MHz
WDW EM
SSB 0
LB 0.10 Hz
GB 0
PC 1.00

Expanded region of the ^{1}H NMR spectrum of compound 32

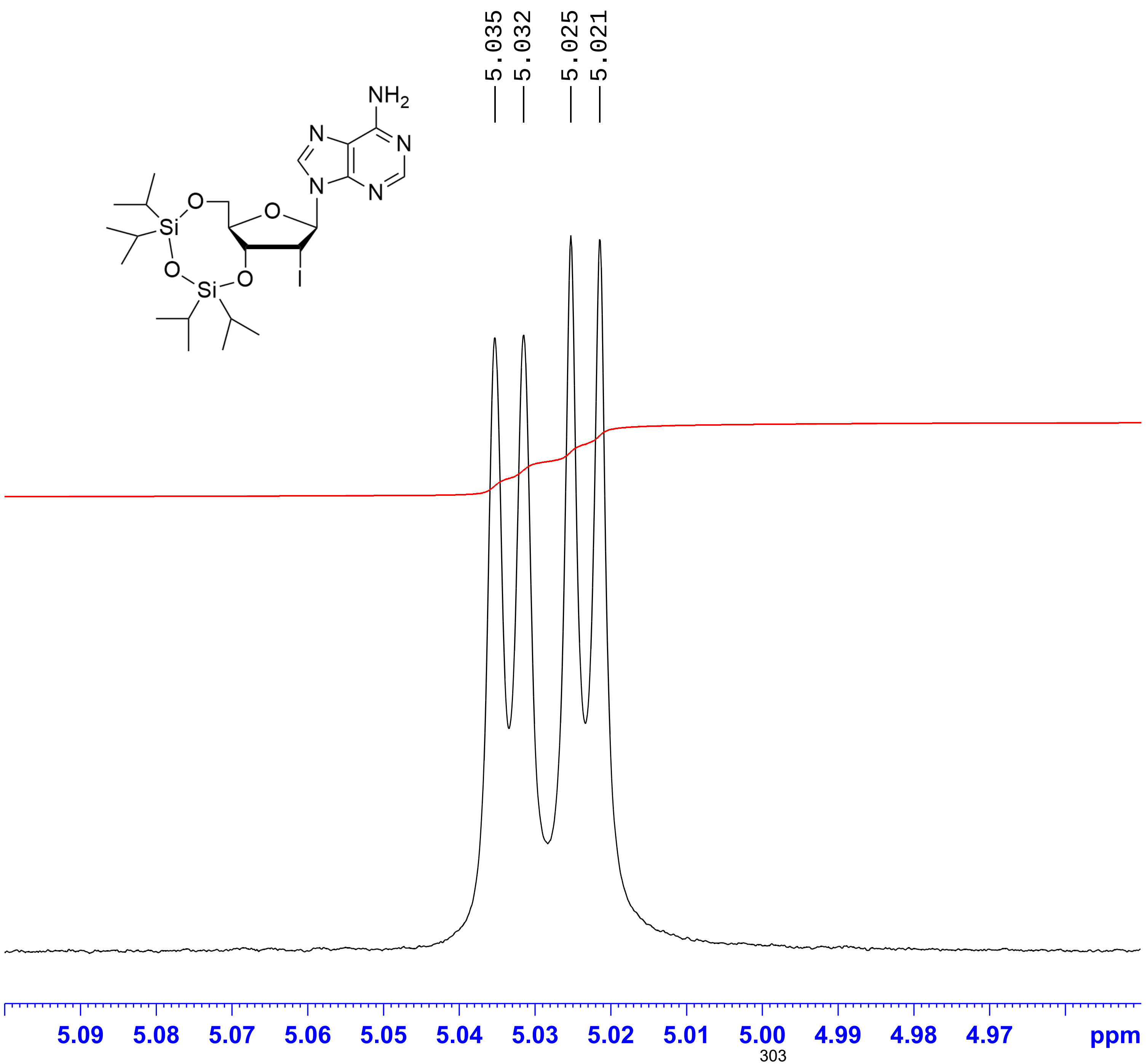

Current Data Parameters
NAME LH-I-60
EXPNO 10
PROCNO 1

F2 - Acquisition Parameters
Date_ 20220210
Time 17.11 h
INSTRUM spect
PROBHD Z114607_0188 (
PULPROG zg30
TD 180286
SOLVENT CDCl3
NS 16
DS 0
SWH 18028.846 Hz
FIDRES 0.200003 Hz
AQ 4.9999318 sec
RG 97.5
DW 27.733 usec
DE 8.00 usec
TE 300.0 K
D1 0.10000000 sec
TD0 1
SFO1 600.1337060 MHz
NUC1 1H
P0 3.33 usec
P1 10.00 usec
PLW1 26.60000038 W

F2 - Processing parameters
SI 262144
SF 600.1300127 MHz
WDW EM
SSB 0
LB 0.10 Hz
GB 0
PC 1.00

Expanded region of the ^{1}H NMR spectrum of compound 32

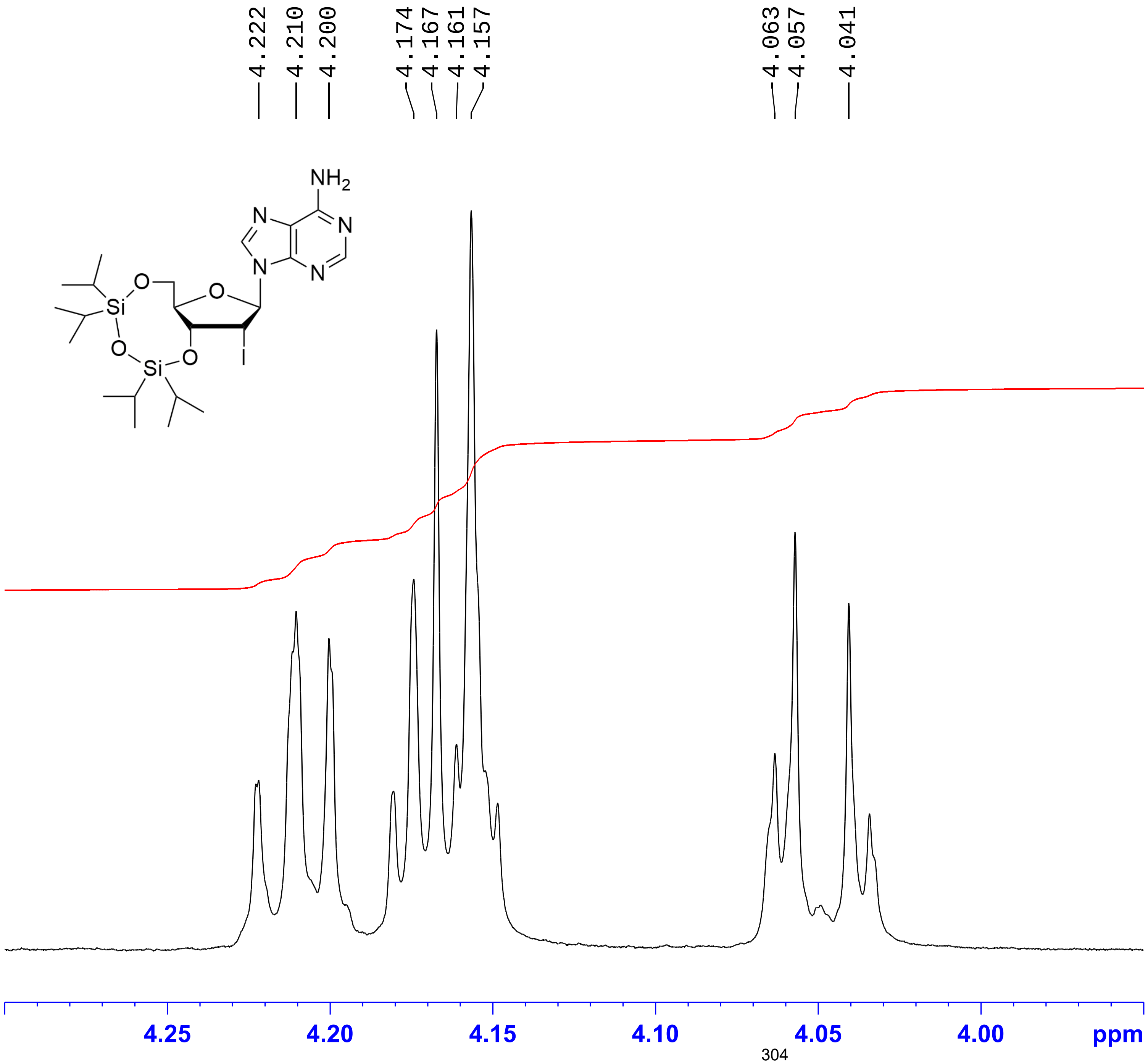


```
Current Data Parameters
NAME              LH-I-60
EXPNO                  10
PROCNO                  1

F2 - Acquisition Parameters
Date_            20220210
Time                17.11 h
INSTRUM             spect
PROBHD   Z114607_0188 (
PULPROG              zg30
TD                 180286
SOLVENT             CDCl3
NS                     16
DS                      0
SWH             18028.846 Hz
FIDRES           0.200003 Hz
AQ              4.9999318 sec
RG                   97.5
DW                 27.733 usec
DE                   8.00 usec
TE                  300.0 K
D1             0.10000000 sec
TD0                     1
SFO1          600.1337060 MHz
NUC1                   1H
P0                   3.33 usec
P1                  10.00 usec
PLW1          26.60000038 W

F2 - Processing parameters
SI                 262144
SF            600.1300127 MHz
WDW                    EM
SSB      0
LB                   0.10 Hz
GB       0
PC                   1.00
```

Expanded region of the ^{1}H NMR spectrum of compound 32

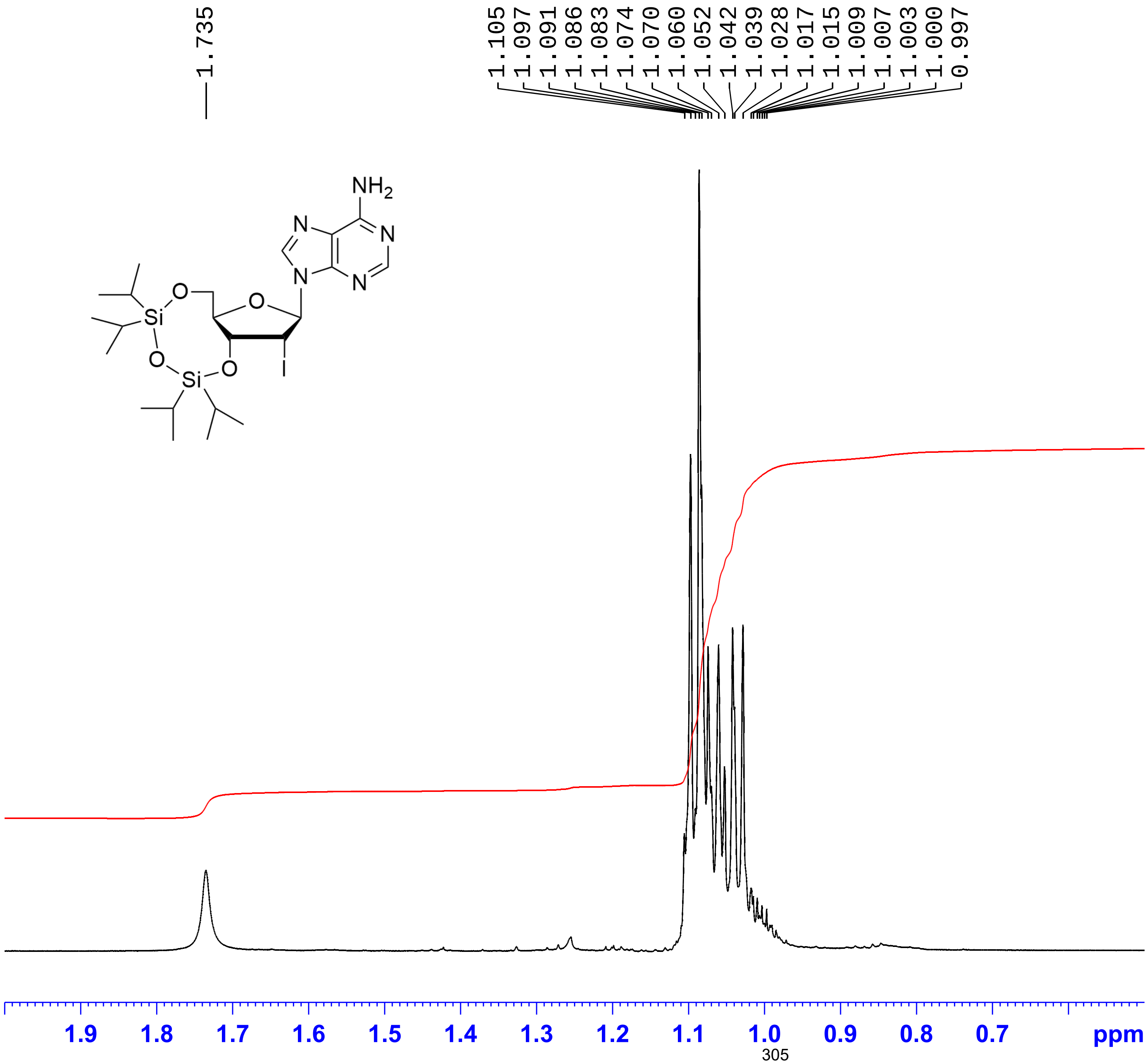


```
Current Data Parameters
NAME              LH-I-60
EXPNO                  10
PROCNO                  1

F2 - Acquisition Parameters
Date_            20220210
Time                17.11 h
INSTRUM             spect
PROBHD     Z114607_0188 (
PULPROG              zg30
TD                 180286
SOLVENT             CDCl3
NS                     16
DS                      0
SWH             18028.846 Hz
FIDRES           0.200003 Hz
AQ              4.9999318 sec
RG                   97.5
DW                 27.733 usec
DE                   8.00 usec
TE                  300.0 K
D1             0.10000000 sec
TD0                     1
SFO1         600.1337060 MHz
NUC1                   1H
P0                   3.33 usec
P1                  10.00 usec
PLW1          26.60000038 W

F2 - Processing parameters
SI                 262144
SF           600.1300127 MHz
WDW                    EM
SSB       0
LB                   0.10 Hz
GB        0
PC                   1.00
```

^{13}C NMR spectrum of compound 32

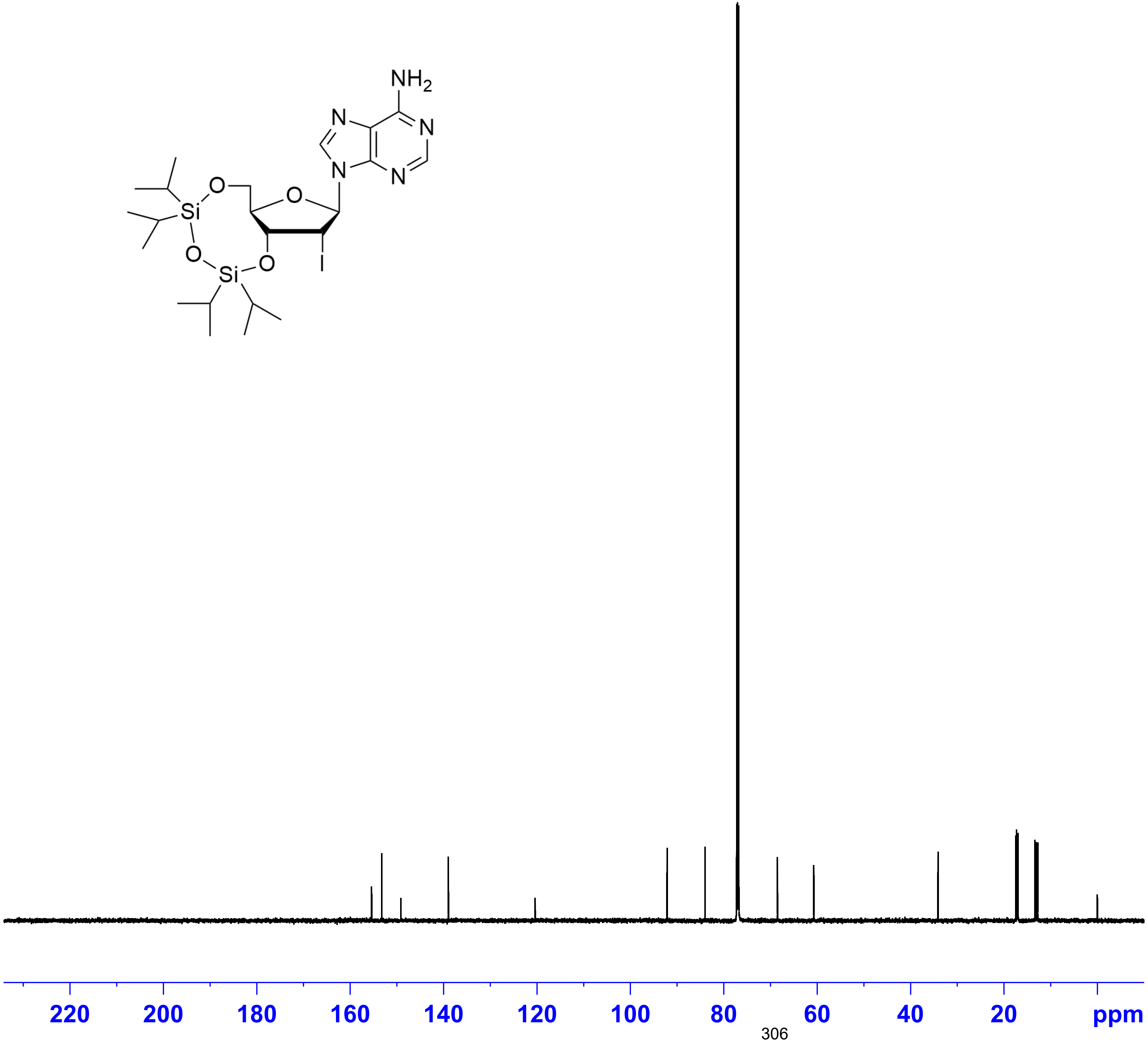

Current Data Parameters

NAME	LH-I-60
EXPNO	11
PROCNO	1

F2 - Acquisition Parameters

Date_	20220210
Time	18.40 h
INSTRUM	spect
PROBHD	Z114607_0188 (
PULPROG	zgpg30
TD	119044
SOLVENT	CDCl3
NS	2000
DS	4
SWH	37500.000 Hz
FIDRES	0.630019 Hz
AQ	1.5872533 sec
RG	186.92
DW	13.333 usec
DE	6.53 usec
TE	300.0 K
D1	1.00000000 sec
D11	0.03000000 sec
TD0	1
SFO1	150.9194058 MHz
NUC1	13C
P0	3.93 usec
P1	11.80 usec
PLW1	85.00000000 W
SFO2	600.1324005 MHz
NUC2	1H
CPDPRG[2	waltz64
PCPD2	70.00 usec
PLW2	27.00000000 W
PLW12	0.57327998 W
PLW13	0.28836000 W

F2 - Processing parameters

SI	131072
SF	150.9028112 MHz
WDW	EM
SSB	0
LB	1.00 Hz
GB	0
PC	1.40

Expanded region of the ^{13}C NMR spectrum of compound 32

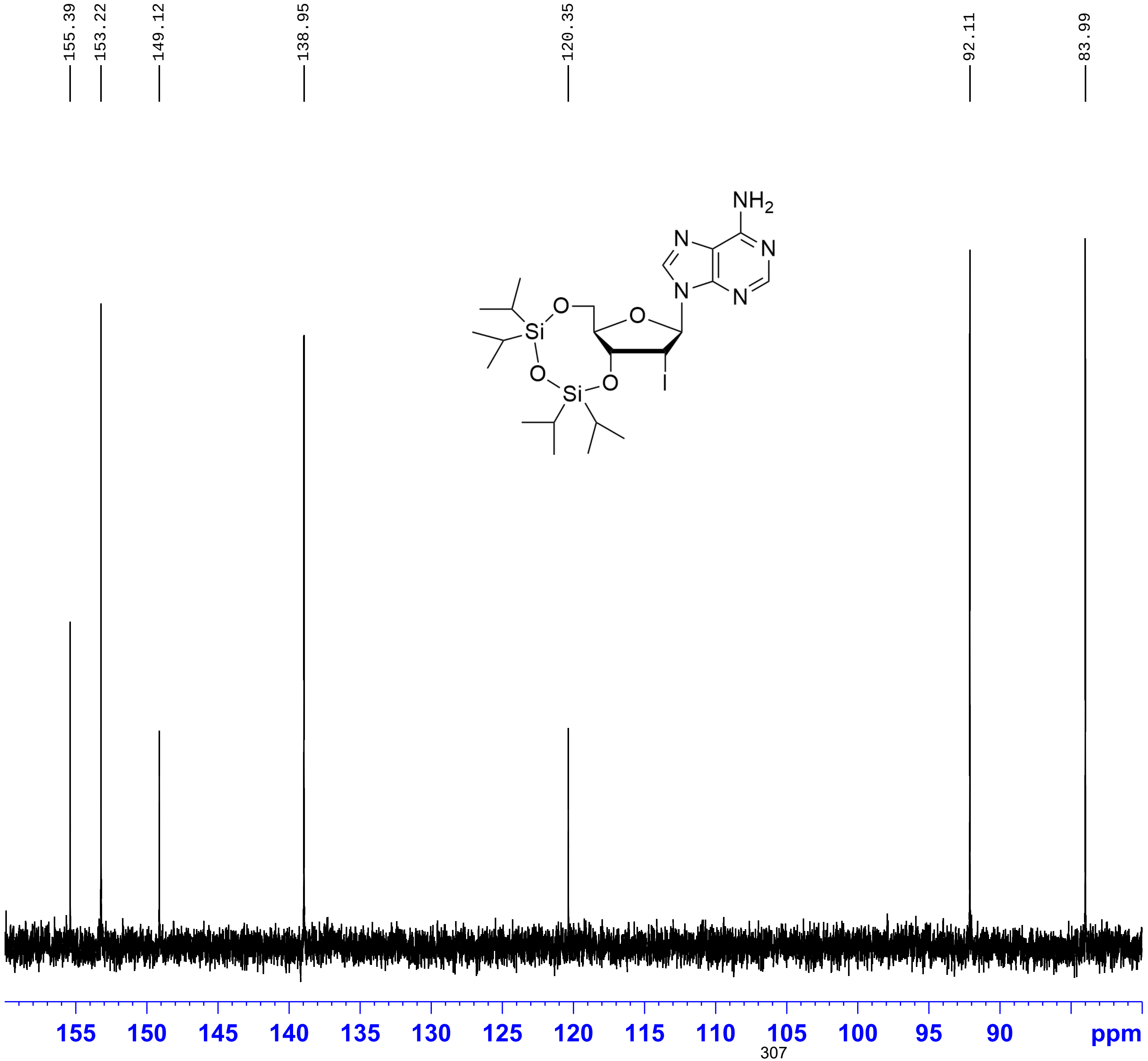

Current Data Parameters
NAME LH-I-60
EXPNO 11
PROCNO 1

F2 - Acquisition Parameters
Date_ 20220210
Time 18.40 h
INSTRUM spect
PROBHD Z114607_0188 (
PULPROG zgpg30
TD 119044
SOLVENT CDCl3
NS 2000
DS 4
SWH 37500.000 Hz
FIDRES 0.630019 Hz
AQ 1.5872533 sec
RG 186.92
DW 13.333 usec
DE 6.53 usec
TE 300.0 K
D1 1.00000000 sec
D11 0.03000000 sec
TD0 1
SFO1 150.9194058 MHz
NUC1 13C
P0 3.93 usec
P1 11.80 usec
PLW1 85.00000000 W
SFO2 600.1324005 MHz
NUC2 1H
CPDPRG[2 waltz64
PCPD2 70.00 usec
PLW2 27.00000000 W
PLW12 0.57327998 W
PLW13 0.28836000 W

F2 - Processing parameters
SI 131072
SF 150.9028112 MHz
WDW EM
SSB 0
LB 1.00 Hz
GB 0
PC 1.40

Expanded region of the ^{13}C NMR spectrum of compound 32

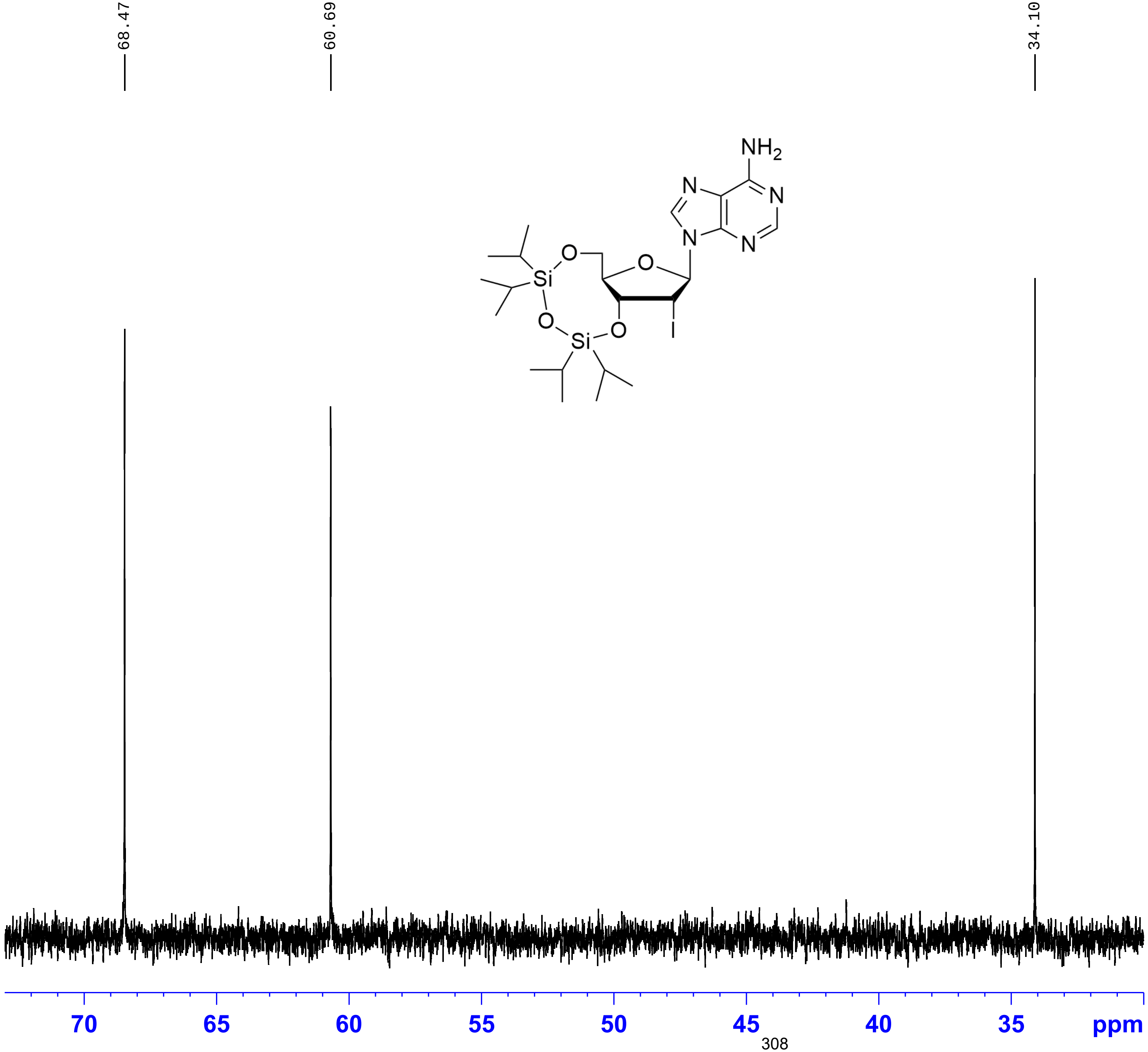

Current Data Parameters
NAME LH-I-60
EXPNO 11
PROCNO 1

F2 - Acquisition Parameters

Date_	20220210	
Time	18.40	h
INSTRUM	spect	
PROBHD	Z114607_0188 (	
PULPROG	zgpg30	
TD	119044	
SOLVENT	CDCl3	
NS	2000	
DS	4	
SWH	37500.000	Hz
FIDRES	0.630019	Hz
AQ	1.5872533	sec
RG	186.92	
DW	13.333	usec
DE	6.53	usec
TE	300.0	K
D1	1.00000000	sec
D11	0.03000000	sec
TD0	1	
SFO1	150.9194058	MHz
NUC1	13C	
P0	3.93	usec
P1	11.80	usec
PLW1	85.00000000	W
SFO2	600.1324005	MHz
NUC2	1H	
CPDPRG[2	waltz64	
PCPD2	70.00	usec
PLW2	27.00000000	W
PLW12	0.57327998	W
PLW13	0.28836000	W

F2 - Processing parameters

SI	131072	
SF	150.9028112	MHz
WDW	EM	
SSB	0	
LB	1.00	Hz
GB	0	
PC	1.40	

Expanded region of the ^{13}C NMR spectrum of compound 32

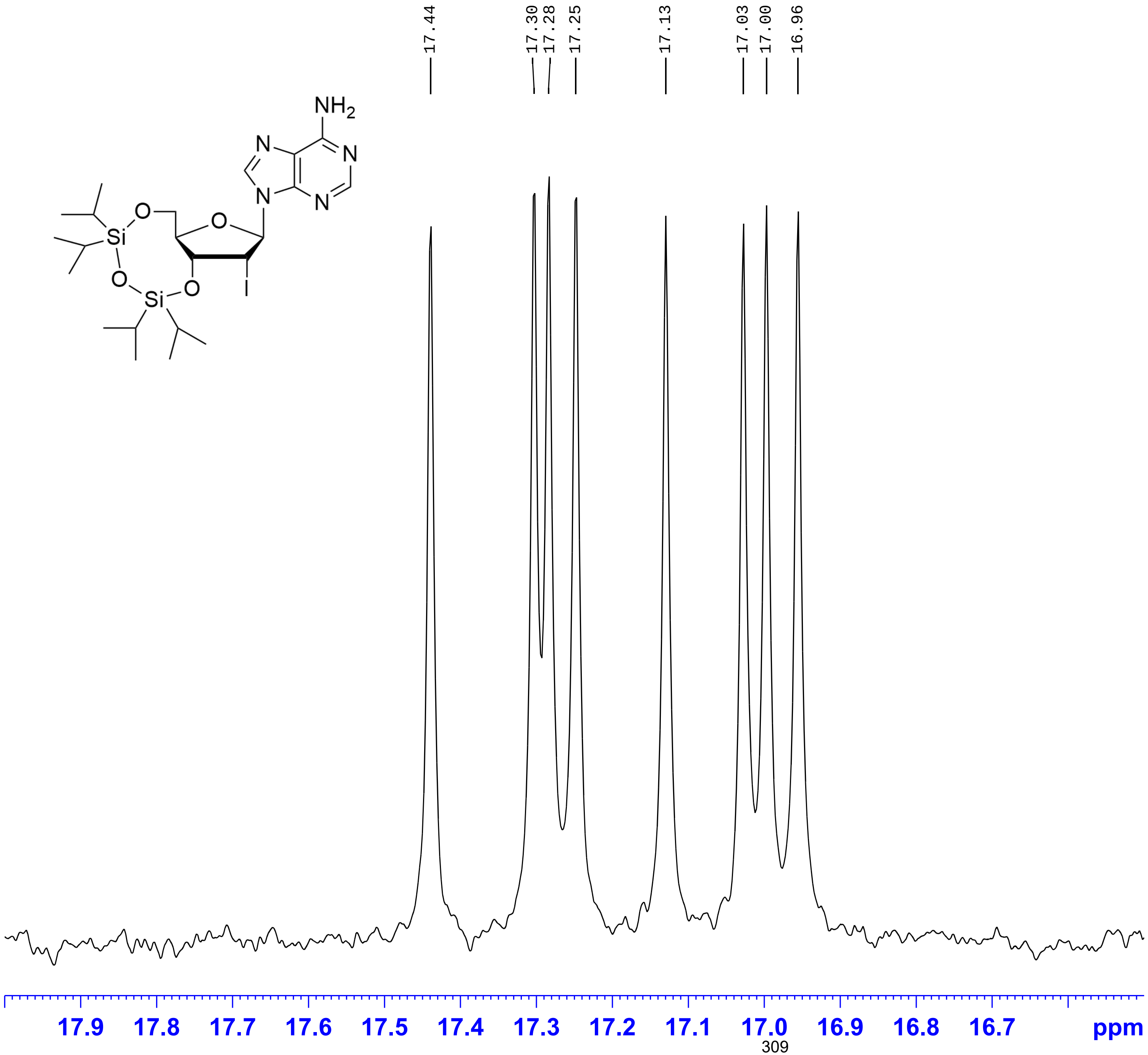

Current Data Parameters		
NAME	LH-I-60	
EXPNO	11	
PROCNO	1	
F2 - Acquisition Parameters		
Date_	20220210	
Time	18.40	h
INSTRUM	spect	
PROBHD	Z114607_0188 (	
PULPROG	zgpg30	
TD	119044	
SOLVENT	CDCl3	
NS	2000	
DS	4	
SWH	37500.000	Hz
FIDRES	0.630019	Hz
AQ	1.5872533	sec
RG	186.92	
DW	13.333	usec
DE	6.53	usec
TE	300.0	K
D1	1.00000000	sec
D11	0.03000000	sec
TD0	1	
SFO1	150.9194058	MHz
NUC1	13C	
P0	3.93	usec
P1	11.80	usec
PLW1	85.00000000	W
SFO2	600.1324005	MHz
NUC2	1H	
CPDPRG[2	waltz64	
PCPD2	70.00	usec
PLW2	27.00000000	W
PLW12	0.57327998	W
PLW13	0.28836000	W
F2 - Processing parameters		
SI	131072	
SF	150.9028112	MHz
WDW	EM	
SSB	0	
LB	1.00	Hz
GB	0	
PC	1.40	

Expanded region of the ^{13}C NMR spectrum of compound 32

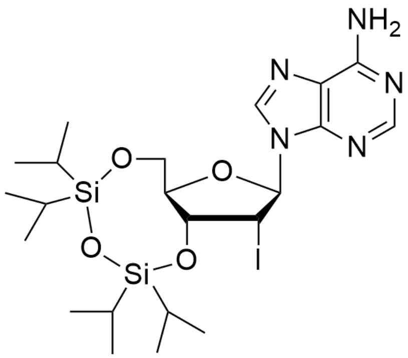

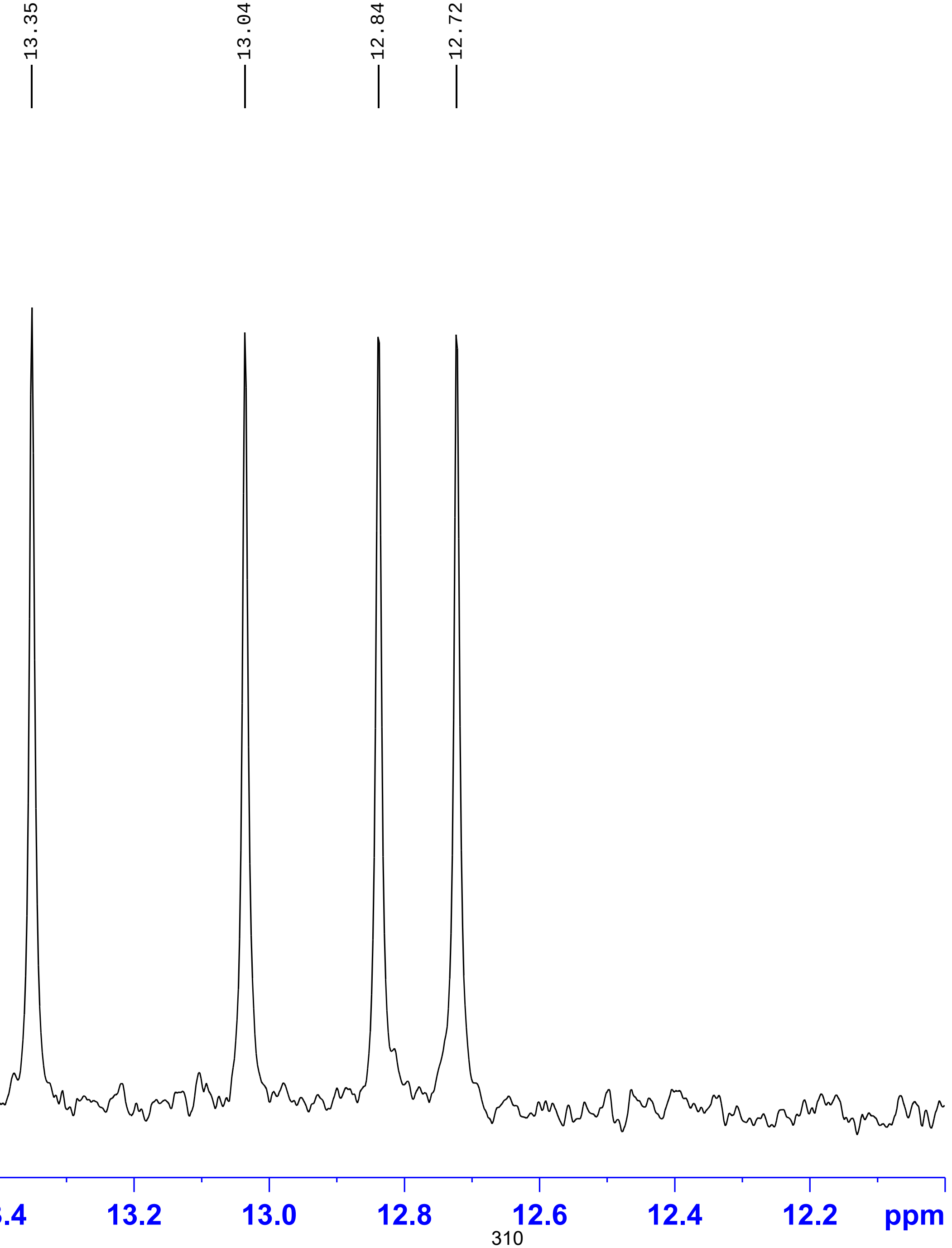

Current Data Parameters
NAME LH-I-60
EXPNO 11
PROCNO 1

F2 - Acquisition Parameters
Date_ 20220210
Time 18.40 h
INSTRUM spect
PROBHD Z114607_0188 (
PULPROG zgpg30
TD 119044
SOLVENT CDCl3
NS 2000
DS 4
SWH 37500.000 Hz
FIDRES 0.630019 Hz
AQ 1.5872533 sec
RG 186.92
DW 13.333 usec
DE 6.53 usec
TE 300.0 K
D1 1.00000000 sec
D11 0.03000000 sec
TD0 1
SFO1 150.9194058 MHz
NUC1 13C
P0 3.93 usec
P1 11.80 usec
PLW1 85.00000000 W
SFO2 600.1324005 MHz
NUC2 1H
CPDPRG[2 waltz64
PCPD2 70.00 usec
PLW2 27.00000000 W
PLW12 0.57327998 W
PLW13 0.28836000 W

F2 - Processing parameters
SI 131072
SF 150.9028112 MHz
WDW EM
SSB 0
LB 1.00 Hz
GB 0
PC 1.40

^{13}C DEPT-135 NMR spectrum of compound 32

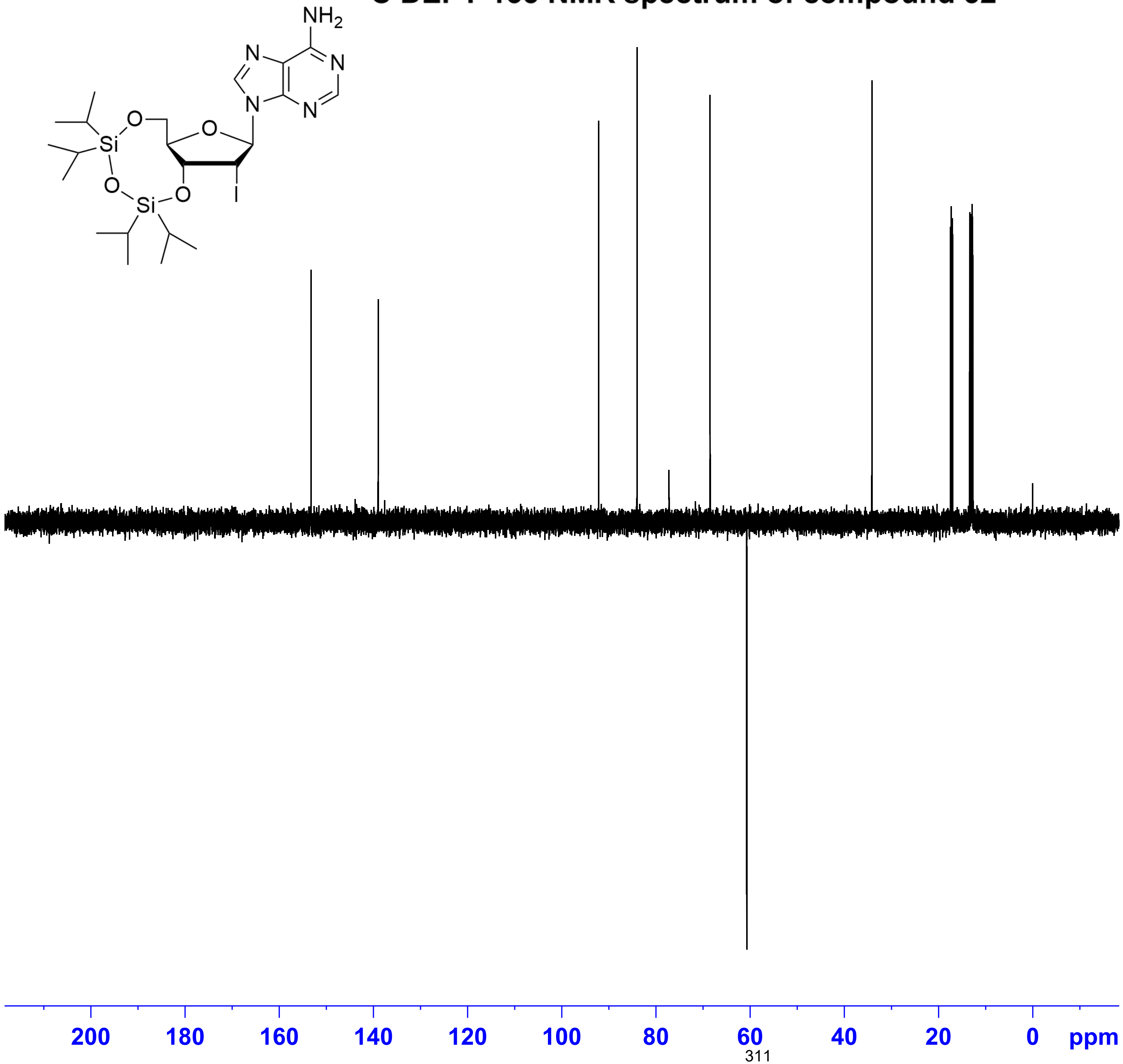

Current Data Parameters
NAME LH-I-60
EXPNO 12
PROCNO 1

F2 - Acquisition Parameters
Date_ 20220210
Time 19.25 h
INSTRUM spect
PROBHD Z114607_0188 (
PULPROG deptsp135.b
TD 119044
SOLVENT CDCl3
NS 1000
DS 4
SWH 35714.285 Hz
FIDRES 0.600018 Hz
AQ 1.6666160 sec
RG 186.92
DW 14.000 usec
DE 7.44 usec
TE 300.0 K
CNST2 145.0000000
D1 1.00000000 sec
D2 0.00344828 sec
D12 0.00002000 sec
TD0 1
SFO1 150.9178962 MHz
NUC1 13C
P1 11.80 usec
P13 2000.00 usec
PLW0 0 W
PLW1 85.00000000 W
SPNAM[5] Crp60comp.4
SPOAL5 0.500
SPOFFS5 0 Hz
SPW5 18.08300018 W
SFO2 600.1324005 MHz
NUC2 1H
CPDPRG[2 waltz64
P3 10.20 usec
P4 20.40 usec
PCPD2 70.00 usec
PLW2 27.00000000 W
PLW12 0.57327998 W

F2 - Processing parameters
SI 131072
SF 150.9028085 MHz
WDW EM
SSB 0
LB 1.00 Hz
GB 0
PC 1.40

^{1}H-^{1}H COSY NMR spectrum of compound 32

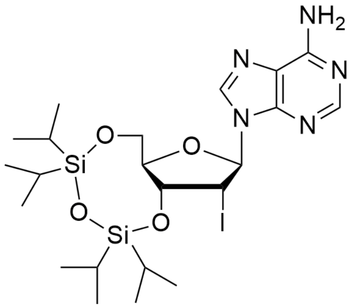

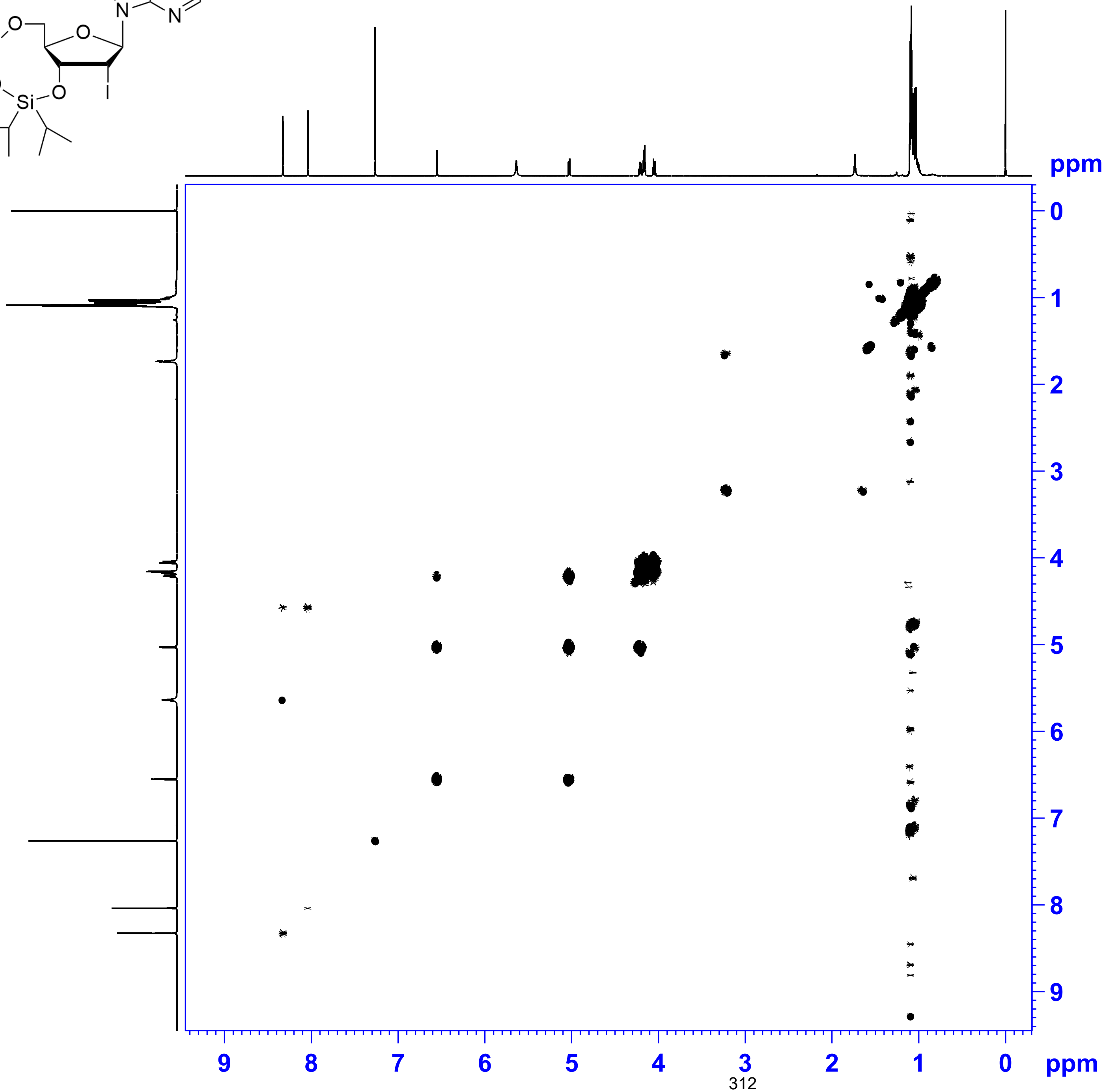


```
Current Data Parameters
NAME              LH-I-60
EXPNO                  13
PROCNO                  1

F2 - Acquisition Parameters
Date_            20220210
Time                19.36 h
INSTRUM             spect
PROBHD   Z114607_0188 (
PULPROG      cosygpmfppqf
TD                   2048
SOLVENT             CDCl3
NS                      2
DS                      8
SWH              5854.801 Hz
FIDRES           5.717579 Hz
AQ              0.1748992 sec
RG                 186.92
DW                 85.400 usec
DE                   6.50 usec
TE                  300.0 K
D0             0.00000300 sec
D1             0.88531131 sec
D11            0.03000000 sec
D12            0.00002000 sec
D13            0.00000400 sec
D16            0.00020000 sec
IN0            0.00017080 sec
TDav                    1
SFO1          600.1327554 MHz
NUC1                   1H
P1                  10.00 usec
P17               2500.00 usec
PLW1          26.60000038 W
PLW10          4.25600004 W
GPNAM[1]       SMSQ10.100
GPZ1                16.00 %
GPNAM[2]       SMSQ10.100
GPZ2                12.00 %
GPNAM[3]       SMSQ10.100
GPZ3                40.00 %
P16               1000.00 usec

F1 - Acquisition parameters
TD                    256
SFO1             600.1328 MHz
FIDRES          45.740631 Hz
SW                  9.756 ppm
FnMODE                 QF

F2 - Processing parameters
SI                   1024
SF            600.1300110 MHz
WDW                  SINE
SSB      0
LB       0 Hz
GB       0
PC                   1.40

F1 - Processing parameters
SI                   1024
MC2                    QF
SF            600.1300115 MHz
WDW                  SINE
SSB      0
LB       0 Hz
GB       0
```

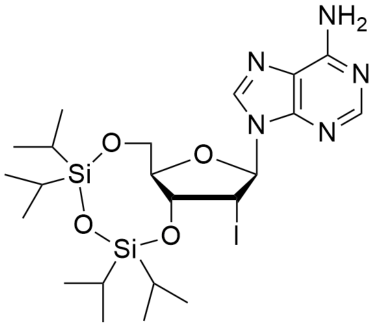

^{1}H-^{13}C HSQC NMR spectrum of compound 32

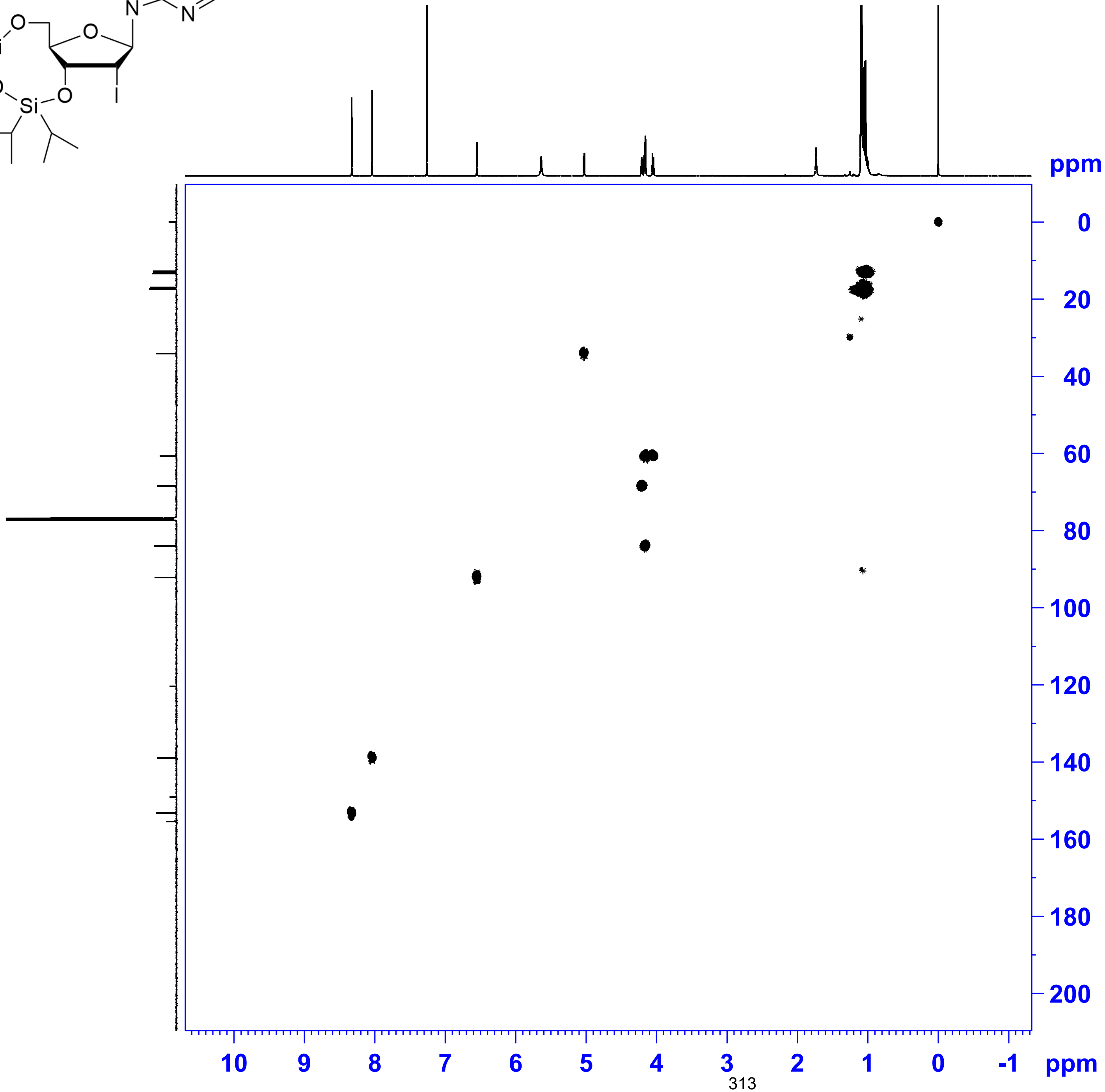


```
Current Data Parameters
NAME              LH-I-60
EXPNO                  14
PROCNO                  1

F2 - Acquisition Parameters
Date_            20220210
Time                19.45 h
INSTRUM             spect
PROBHD   Z114607_0188 (
PULPROG  hsqcedetgpsp.3
TD                   1024
SOLVENT             CDCl3
NS                      2
DS                     32
SWH              7211.539 Hz
FIDRES          14.085036 Hz
AQ              0.0709973 sec
RG                 186.92
DW                 69.333 usec
DE                   6.50 usec
TE                  300.2 K
CNST2         145.0000000
D0             0.00000300 sec
D1             0.80000001 sec
D4             0.00172414 sec
D11            0.03000000 sec
D16            0.00020000 sec
D21            0.00360000 sec
IN0            0.00001510 sec
TDav                    1
ZGOPTNS
SFO1          600.1328223 MHz
NUC1                   1H
P1                  10.00 usec
P2                  20.00 usec
PLW1          26.60000038 W
SFO2          150.9178988 MHz
NUC2                  13C
CPDPRG[2            garp4
P3                  11.80 usec
P14                500.00 usec
P31               1730.00 usec
PCPD2               60.00 usec
PLW0     0 W
PLW2          85.00000000 W
PLW12          3.28760004 W
SPNAM[3] Crp60,0.5,20.1
SPOAL3              0.500
SPOFFS3  0 Hz
SPW3          18.08300018 W
SPNAM[18  Crp60_xfilt.2
SPOAL18             0.500
SPOFFS18 0 Hz
SPW18          5.22629976 W
GPNAM[1]       SMSQ10.100
GPZ1                80.00 %
GPNAM[2]       SMSQ10.100
GPZ2                20.10 %
P16               1000.00 usec

F1 - Acquisition parameters
TD                    256
SFO1             150.9179 MHz
FIDRES         258.692047 Hz
SW                219.408 ppm
FnMODE       Echo-Antiecho

F2 - Processing parameters
SI                   1024
SF            600.1300120 MHz
WDW                 QSINE
SSB                     2
LB       0 Hz
GB       0
PC                   1.40

F1 - Processing parameters
SI                   1024
MC2          echo-antiecho
SF            150.9028179 MHz
WDW                 QSINE
SSB                     2
LB       0 Hz
GB       0
```

^{1}H NMR spectrum of compound 33

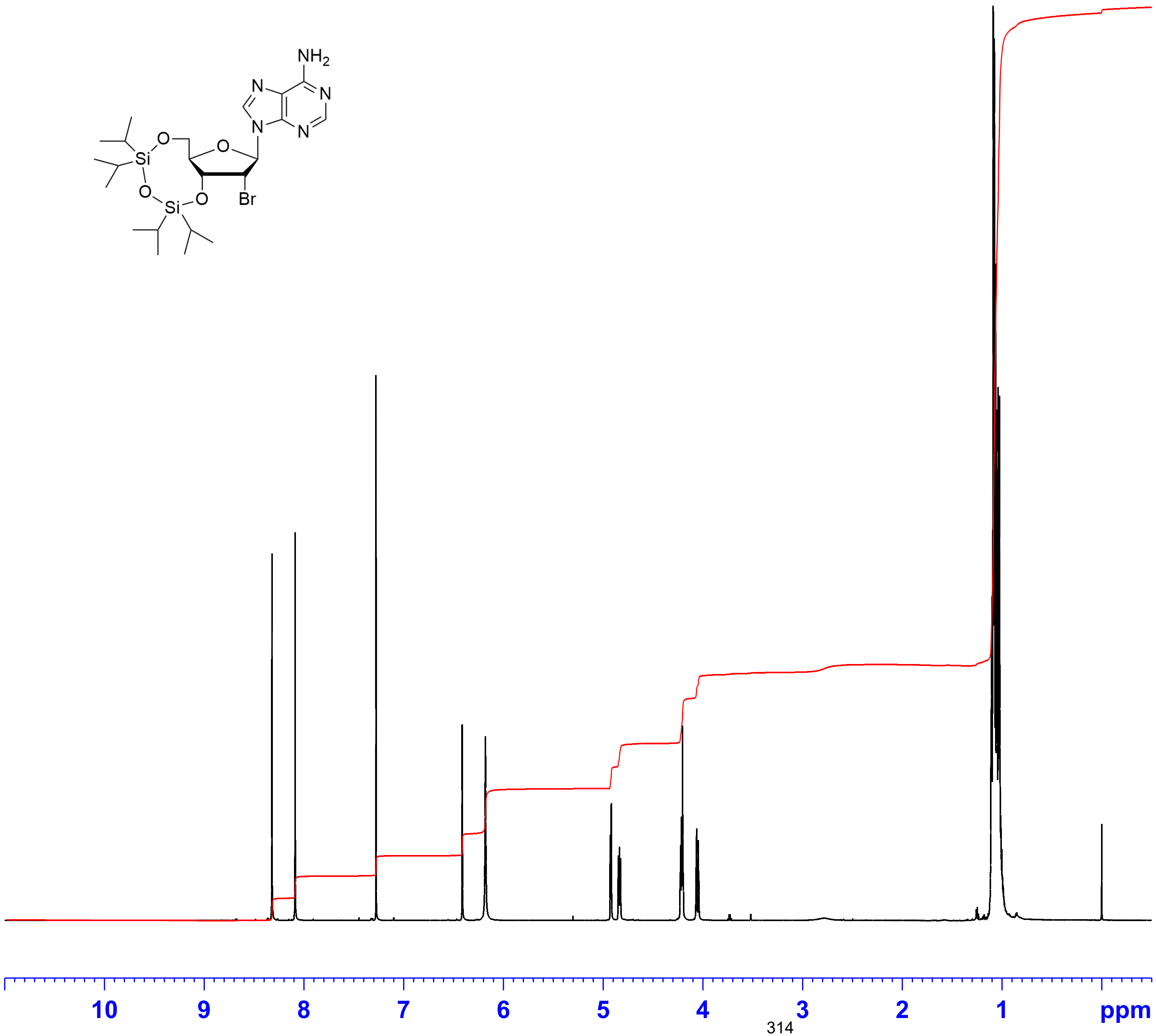

BRUKER

```
Current Data Parameters
NAME               LH-I-66
EXPNO                   10
PROCNO                   1

F2 - Acquisition Parameters
Date_             20220301
Time                 16.52 h
INSTRUM              spect
PROBHD    Z114607_0188 (
PULPROG               zg30
TD                  180286
SOLVENT              CDCl3
NS                      16
DS                       0
SWH              18028.846 Hz
FIDRES            0.200003 Hz
AQ               4.9999318 sec
RG                   31.58
DW                  27.733 usec
DE                    8.00 usec
TE                   300.0 K
D1              0.10000000 sec
TD0                      1
SFO1           600.1337060 MHz
NUC1                    1H
P0                    3.33 usec
P1                   10.00 usec
PLW1           26.60000038 W

F2 - Processing parameters
SI                  262144
SF             600.1300047 MHz
WDW                     EM
SSB      0
LB                    0.10 Hz
GB       0
PC                    1.00
```

Expanded region of the ^1H NMR spectrum of compound 33

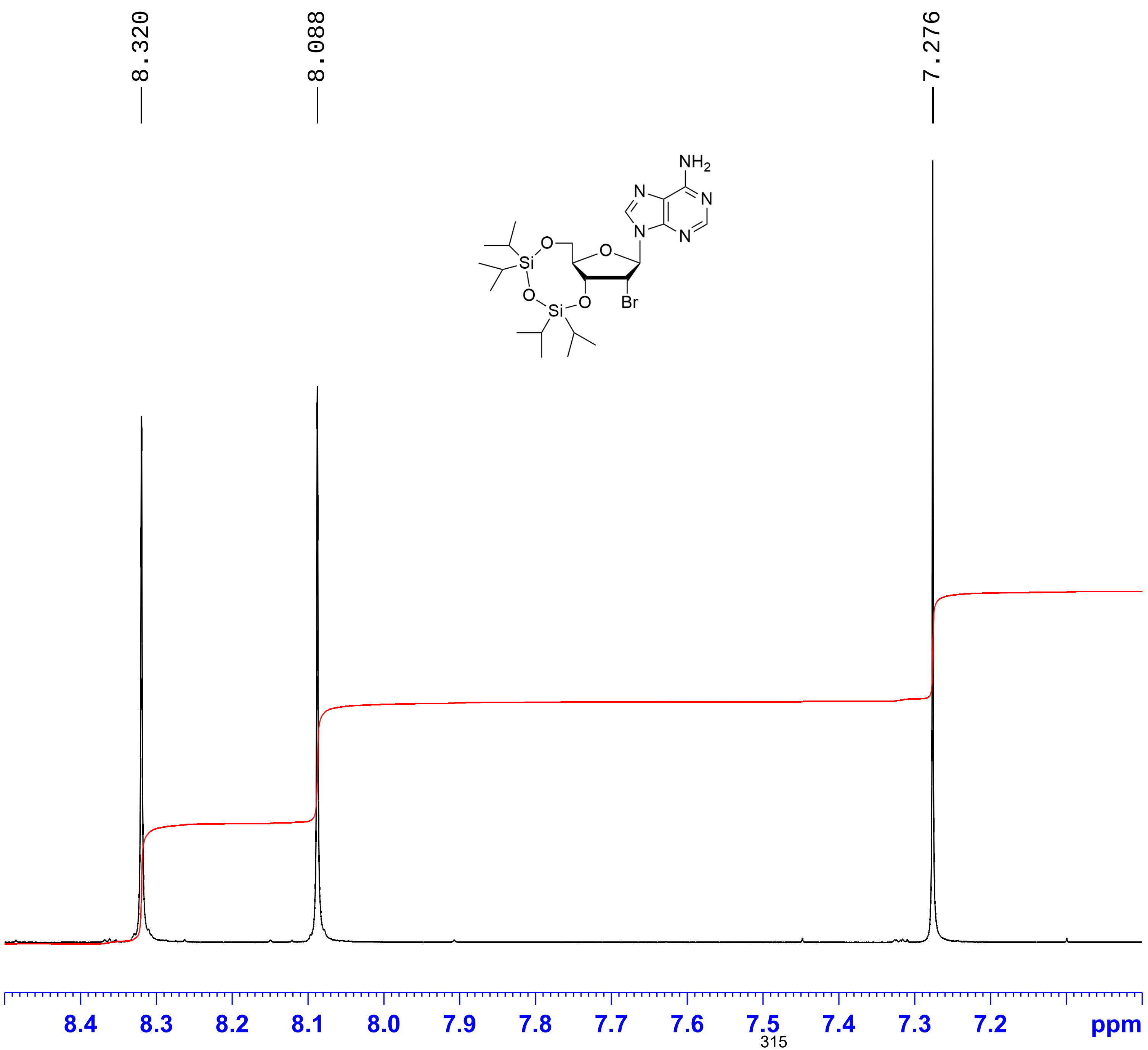

Current Data Parameters
NAME LH-I-66
EXPNO 10
PROCNO 1

F2 - Acquisition Parameters
Date_ 20220301
Time 16.52 h
INSTRUM spect
PROBHD Z114607_0188 (
PULPROG zg30
TD 180286
SOLVENT CDCl3
NS 16
DS 0
SWH 18028.846 Hz
FIDRES 0.200003 Hz
AQ 4.9999318 sec
RG 31.58
DW 27.733 usec
DE 8.00 usec
TE 300.0 K
D1 0.10000000 sec
TD0 1
SFO1 600.1337060 MHz
NUC1 1H
P0 3.33 usec
P1 10.00 usec
PLW1 26.60000038 W

F2 - Processing parameters
SI 262144
SF 600.1300047 MHz
WDW EM
SSB 0
LB 0.10 Hz
GB 0
PC 1.00

Expanded region of the ^{1}H NMR spectrum of compound 33

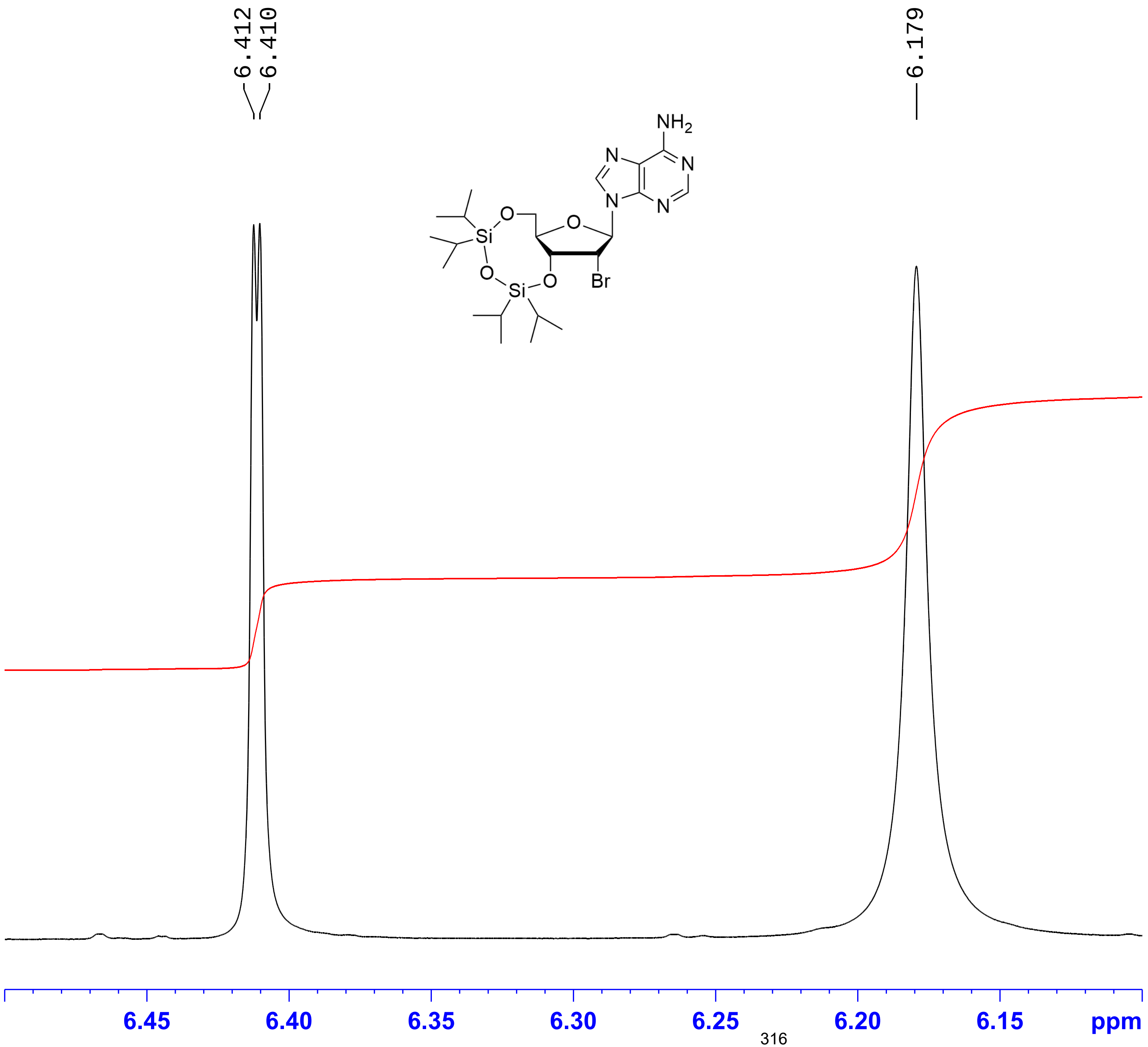

Current Data Parameters
NAME LH-I-66
EXPNO 10
PROCNO 1

F2 - Acquisition Parameters
Date_ 20220301
Time 16.52 h
INSTRUM spect
PROBHD Z114607_0188 (
PULPROG zg30
TD 180286
SOLVENT CDCl3
NS 16
DS 0
SWH 18028.846 Hz
FIDRES 0.200003 Hz
AQ 4.9999318 sec
RG 31.58
DW 27.733 usec
DE 8.00 usec
TE 300.0 K
D1 0.10000000 sec
TD0 1
SFO1 600.1337060 MHz
NUC1 1H
P0 3.33 usec
P1 10.00 usec
PLW1 26.60000038 W

F2 - Processing parameters
SI 262144
SF 600.1300047 MHz
WDW EM
SSB 0
LB 0.10 Hz
GB 0
PC 1.00

Expanded region of the ^{1}H NMR spectrum of compound 33

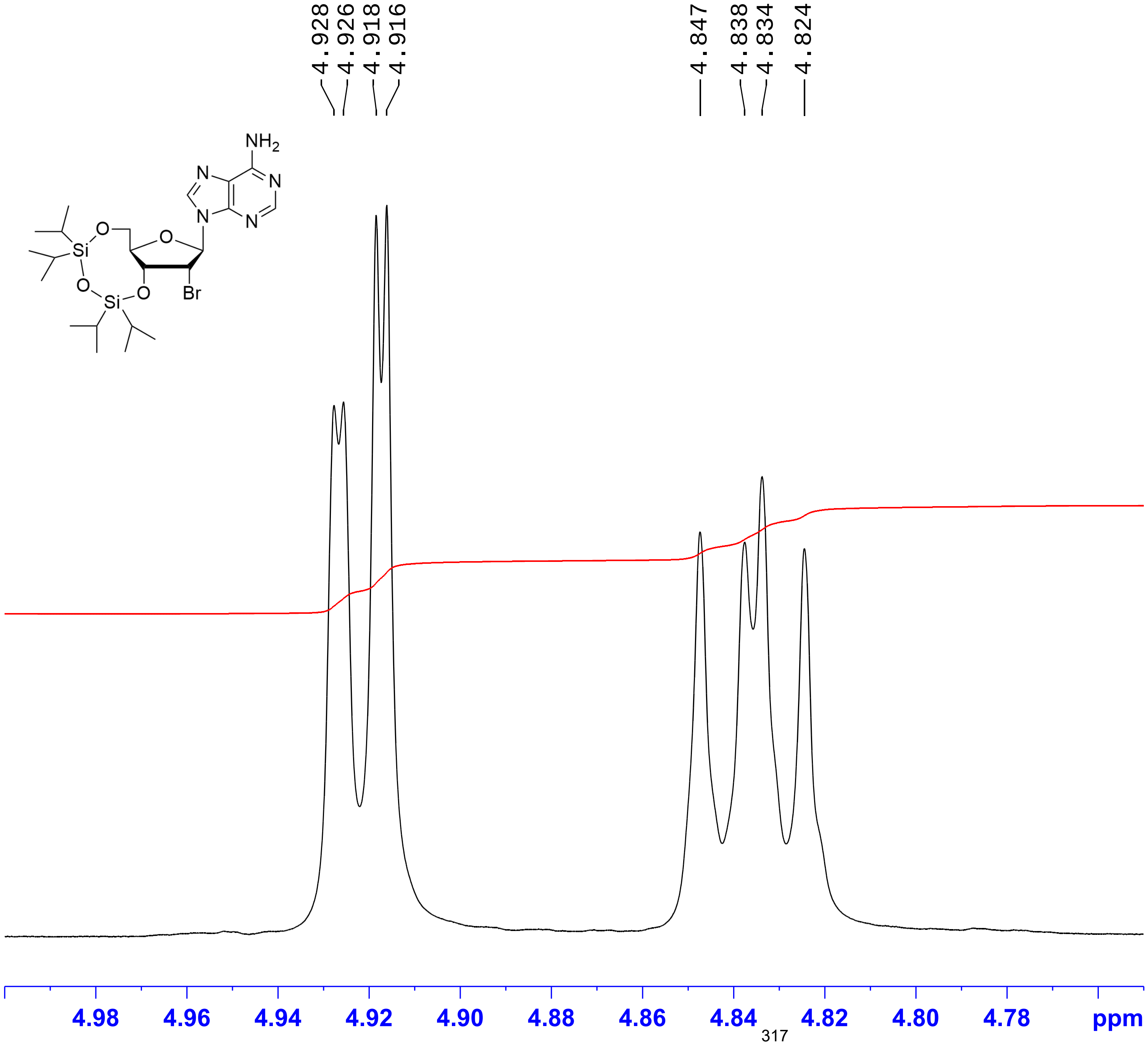


```
Current Data Parameters
NAME              LH-I-66
EXPNO                  10
PROCNO                  1

F2 - Acquisition Parameters
Date_            20220301
Time                16.52 h
INSTRUM             spect
PROBHD   Z114607_0188 (
PULPROG              zg30
TD                 180286
SOLVENT             CDCl3
NS                     16
DS                      0
SWH             18028.846 Hz
FIDRES           0.200003 Hz
AQ              4.9999318 sec
RG                  31.58
DW                 27.733 usec
DE                   8.00 usec
TE                  300.0 K
D1             0.10000000 sec
TD0                     1
SFO1          600.1337060 MHz
NUC1                   1H
P0                   3.33 usec
P1                  10.00 usec
PLW1          26.60000038 W

F2 - Processing parameters
SI                 262144
SF            600.1300047 MHz
WDW                    EM
SSB      0
LB                   0.10 Hz
GB       0
PC                   1.00
```

Expanded region of the ^{1}H NMR spectrum of compound 33

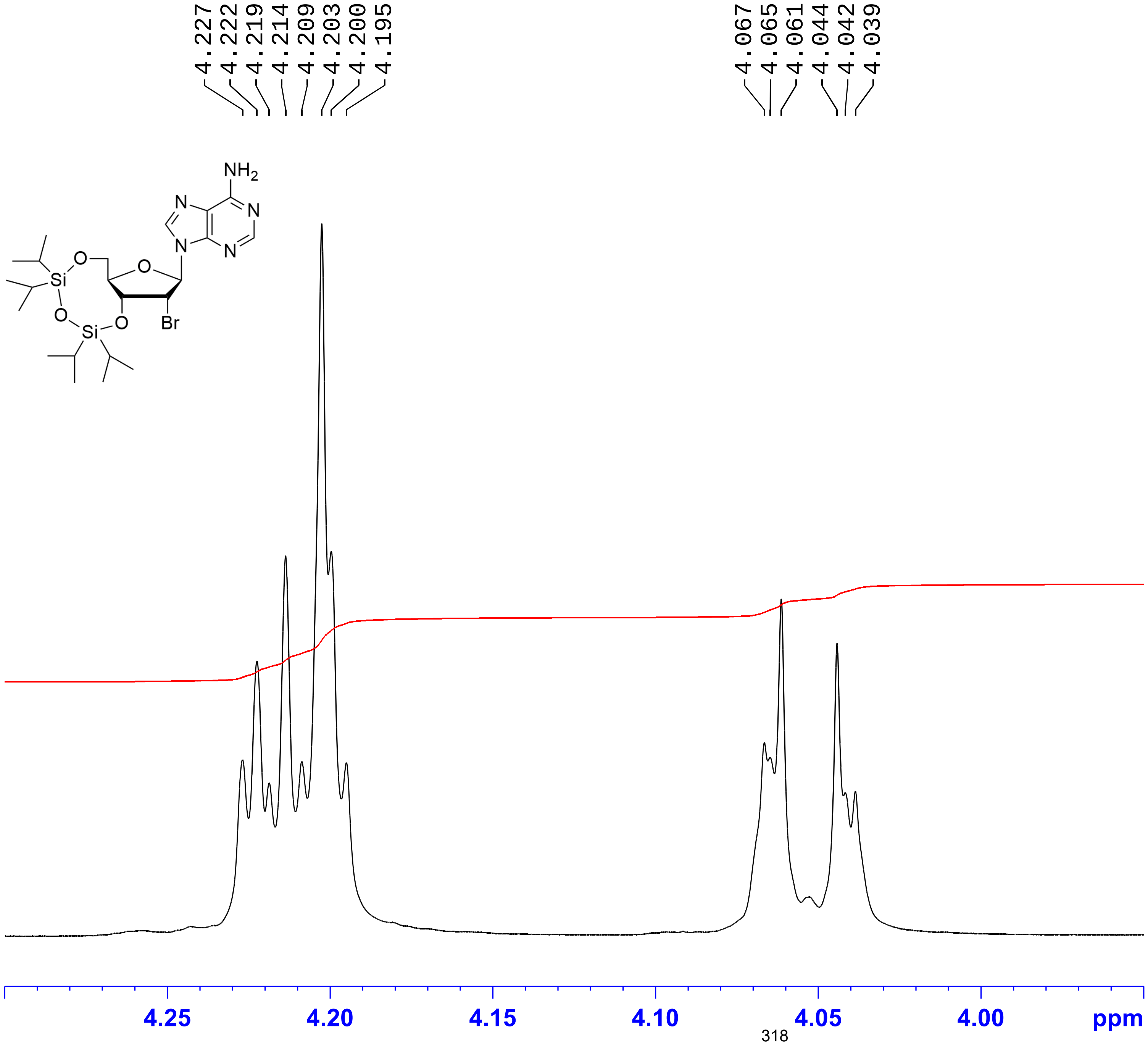


```
Current Data Parameters
NAME              LH-I-66
EXPNO                  10
PROCNO                  1

F2 - Acquisition Parameters
Date_           20220301
Time               16.52 h
INSTRUM            spect
PROBHD    Z114607_0188 (
PULPROG             zg30
TD                180286
SOLVENT            CDCl3
NS                    16
DS                     0
SWH            18028.846 Hz
FIDRES          0.200003 Hz
AQ             4.9999318 sec
RG                 31.58
DW                27.733 usec
DE                  8.00 usec
TE                 300.0 K
D1            0.10000000 sec
TD0                    1
SFO1         600.1337060 MHz
NUC1                  1H
P0                  3.33 usec
P1                 10.00 usec
PLW1         26.60000038 W

F2 - Processing parameters
SI                262144
SF           600.1300047 MHz
WDW                   EM
SSB      0
LB                  0.10 Hz
GB       0
PC                  1.00
```

^{13}C NMR spectrum of compound 33

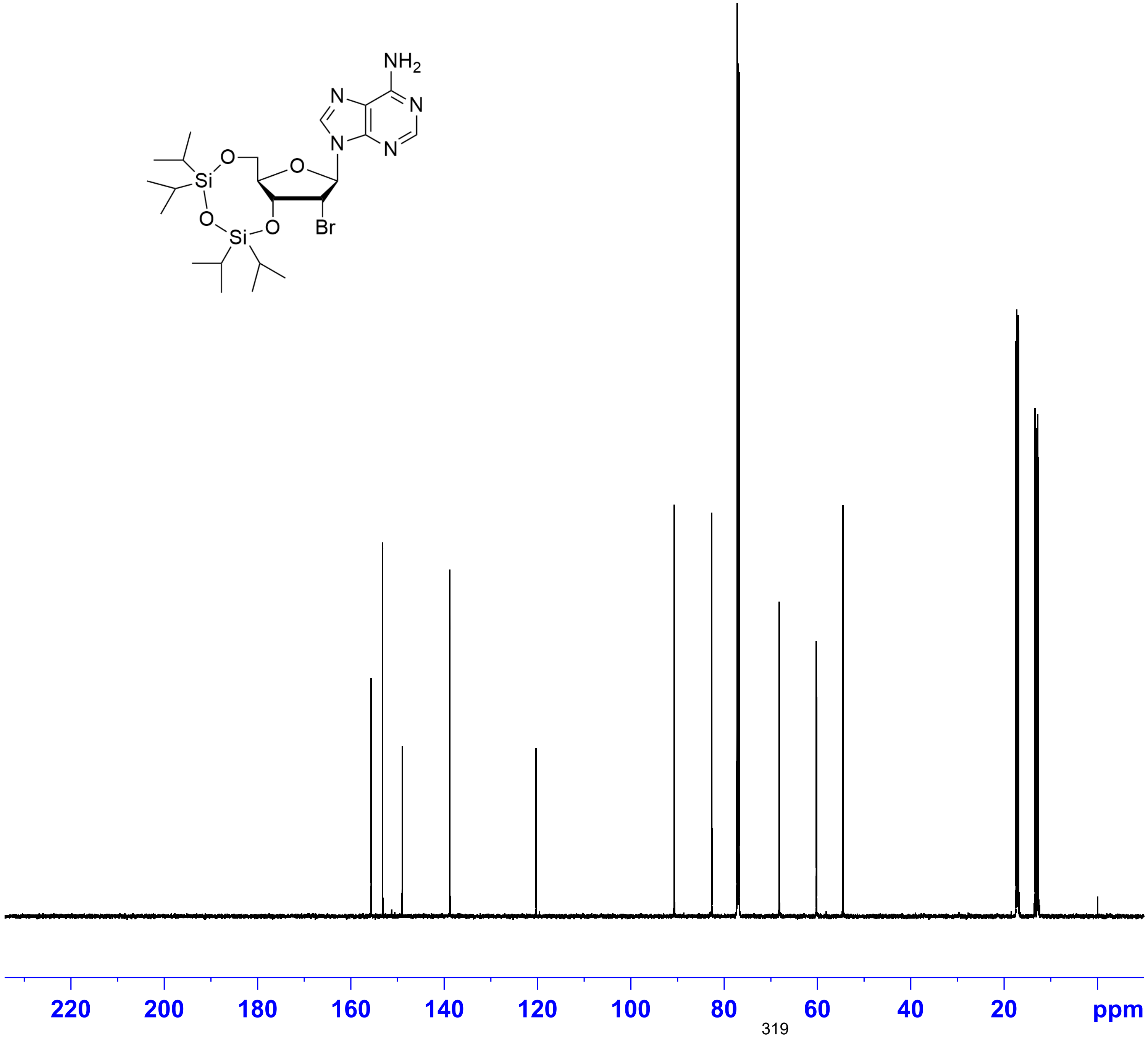

Current Data Parameters
NAME LH-I-66
EXPNO 11
PROCNO 1

F2 - Acquisition Parameters
Date_ 20220301
Time 18.21 h
INSTRUM spect
PROBHD Z114607_0188 (
PULPROG zgpg30
TD 119044
SOLVENT CDCl3
NS 2000
DS 4
SWH 37500.000 Hz
FIDRES 0.630019 Hz
AQ 1.5872533 sec
RG 186.92
DW 13.333 usec
DE 6.53 usec
TE 300.0 K
D1 1.00000000 sec
D11 0.03000000 sec
TD0 1
SFO1 150.9194058 MHz
NUC1 13C
P0 3.93 usec
P1 11.80 usec
PLW1 85.00000000 W
SFO2 600.1324005 MHz
NUC2 1H
CPDPRG[2 waltz64
PCPD2 70.00 usec
PLW2 27.00000000 W
PLW12 0.57327998 W
PLW13 0.28836000 W

F2 - Processing parameters
SI 131072
SF 150.9028152 MHz
WDW EM
SSB 0
LB 1.00 Hz
GB 0
PC 1.40

Expanded region of the ^{13}C NMR spectrum of compound 33

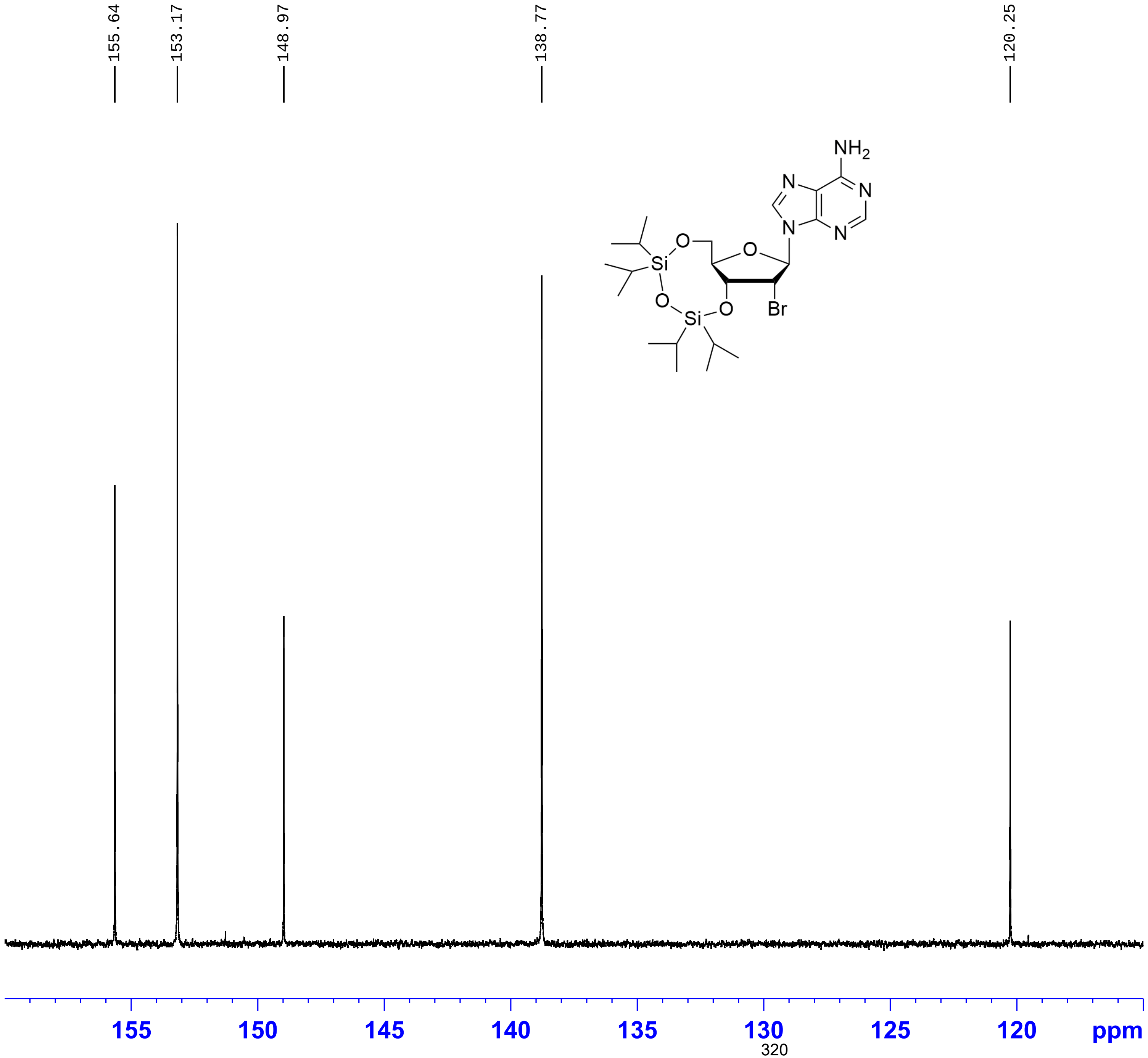


```
Current Data Parameters
NAME             LH-I-66
EXPNO                 11
PROCNO                 1

F2 - Acquisition Parameters
Date_           20220301
Time               18.21 h
INSTRUM            spect
PROBHD    Z114607_0188 (
PULPROG           zgpg30
TD                119044
SOLVENT            CDCl3
NS                  2000
DS                     4
SWH            37500.000 Hz
FIDRES          0.630019 Hz
AQ             1.5872533 sec
RG                186.92
DW                13.333 usec
DE                  6.53 usec
TE                 300.0 K
D1            1.00000000 sec
D11           0.03000000 sec
TD0                    1
SFO1         150.9194058 MHz
NUC1                 13C
P0                  3.93 usec
P1                 11.80 usec
PLW1         85.00000000 W
SFO2         600.1324005 MHz
NUC2                  1H
CPDPRG[2         waltz64
PCPD2              70.00 usec
PLW2         27.00000000 W
PLW12         0.57327998 W
PLW13         0.28836000 W

F2 - Processing parameters
SI                131072
SF           150.9028152 MHz
WDW                   EM
SSB       0
LB                  1.00 Hz
GB        0
PC                  1.40
```

Expanded region of the ^{13}C NMR spectrum of compound 33

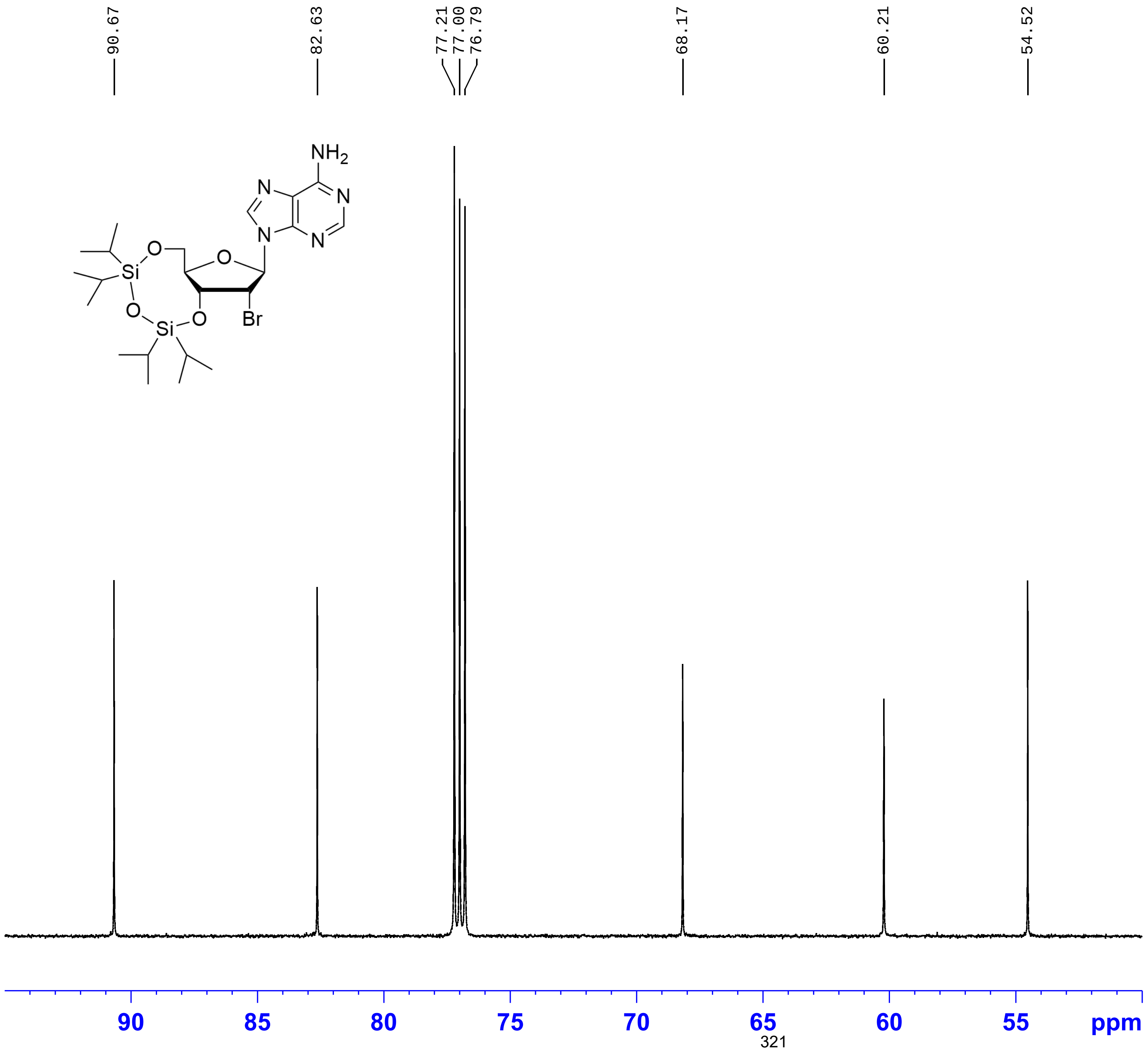

Current Data Parameters
NAME LH-I-66
EXPNO 11
PROCNO 1

F2 - Acquisition Parameters
Date_ 20220301
Time 18.21 h
INSTRUM spect
PROBHD Z114607_0188 (
PULPROG zgpg30
TD 119044
SOLVENT CDCl3
NS 2000
DS 4
SWH 37500.000 Hz
FIDRES 0.630019 Hz
AQ 1.5872533 sec
RG 186.92
DW 13.333 usec
DE 6.53 usec
TE 300.0 K
D1 1.00000000 sec
D11 0.03000000 sec
TD0 1
SFO1 150.9194058 MHz
NUC1 13C
P0 3.93 usec
P1 11.80 usec
PLW1 85.00000000 W
SFO2 600.1324005 MHz
NUC2 1H
CPDPRG[2 waltz64
PCPD2 70.00 usec
PLW2 27.00000000 W
PLW12 0.57327998 W
PLW13 0.28836000 W

F2 - Processing parameters
SI 131072
SF 150.9028152 MHz
WDW EM
SSB 0
LB 1.00 Hz
GB 0
PC 1.40

Expanded region of the ^{13}C NMR spectrum of compound 33

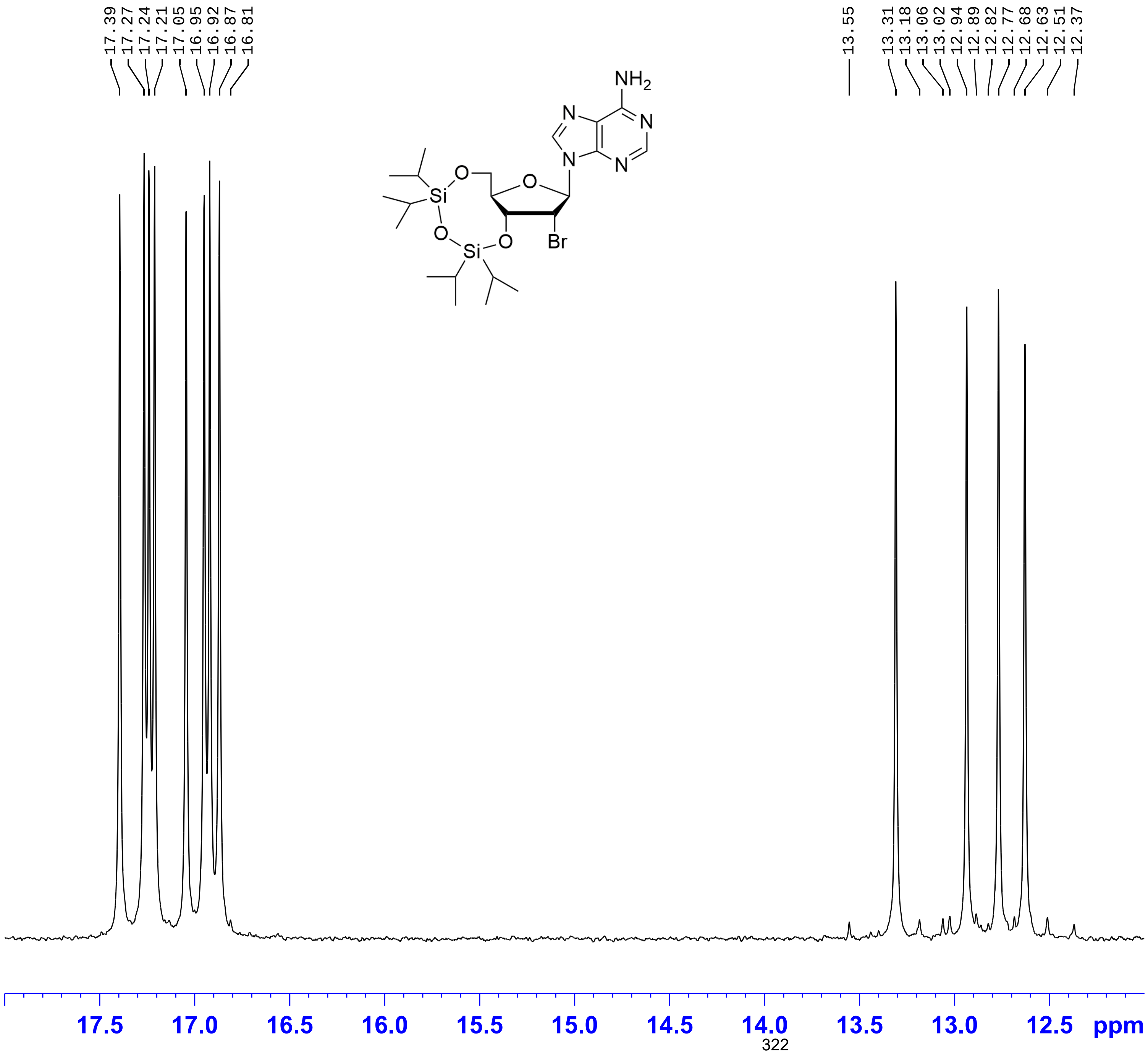


```
Current Data Parameters
NAME                LH-I-66
EXPNO                    11
PROCNO                    1

F2 - Acquisition Parameters
Date_              20220301
Time                  18.21 h
INSTRUM               spect
PROBHD       Z114607_0188 (
PULPROG             zgpg30
TD                   119044
SOLVENT               CDCl3
NS                     2000
DS                        4
SWH              37500.000 Hz
FIDRES            0.630019 Hz
AQ               1.5872533 sec
RG                   186.92
DW                   13.333 usec
DE                     6.53 usec
TE                    300.0 K
D1               1.00000000 sec
D11              0.03000000 sec
TD0                       1
SFO1            150.9194058 MHz
NUC1                    13C
P0                     3.93 usec
P1                    11.80 usec
PLW1            85.00000000 W
SFO2            600.1324005 MHz
NUC2                     1H
CPDPRG[2            waltz64
PCPD2                 70.00 usec
PLW2            27.00000000 W
PLW12            0.57327998 W
PLW13            0.28836000 W

F2 - Processing parameters
SI                   131072
SF              150.9028152 MHz
WDW                      EM
SSB      0
LB                     1.00 Hz
GB       0
PC                     1.40
```

^{13}C DEPT-135 NMR spectrum of compound 33

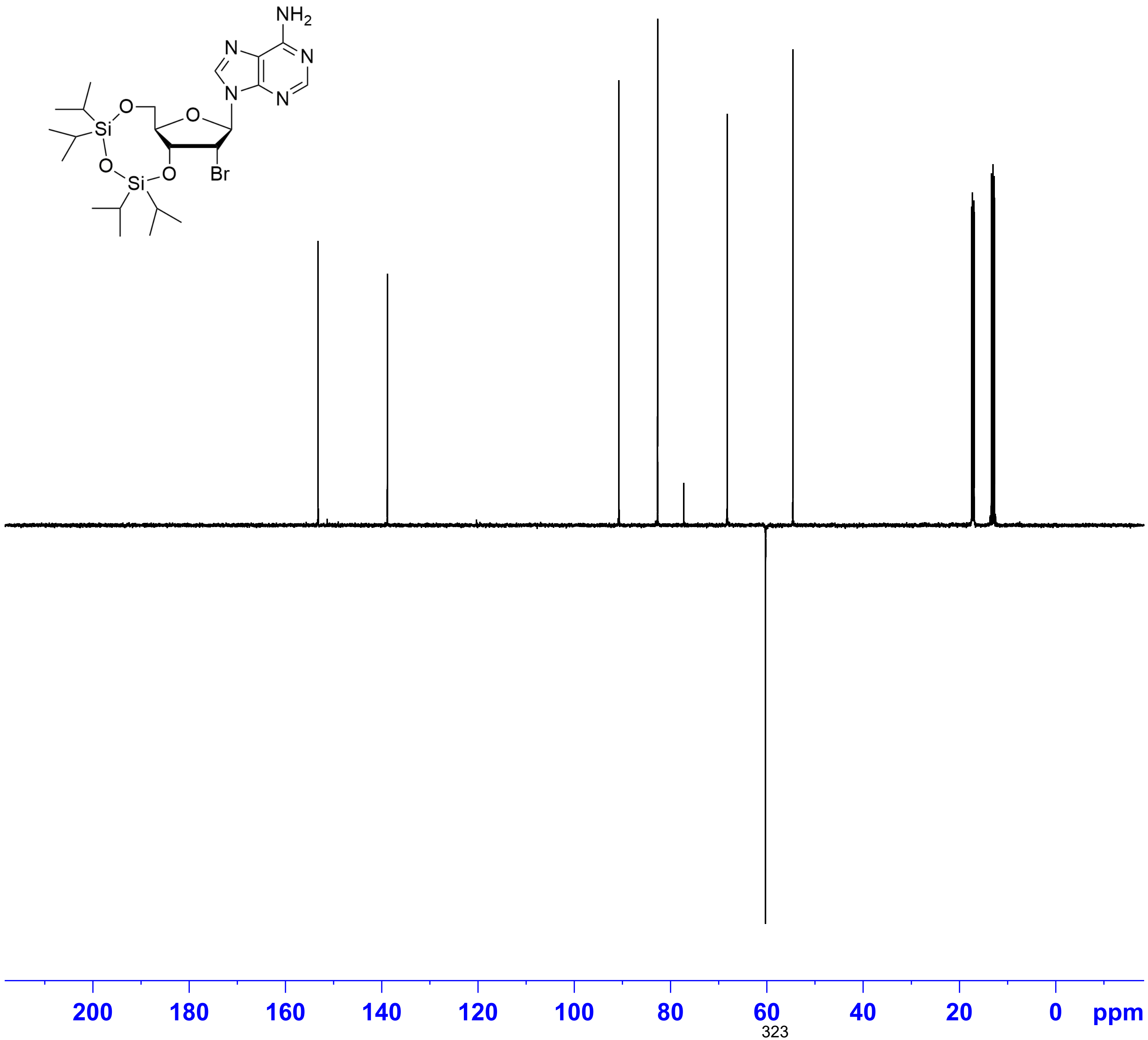


```
Current Data Parameters
NAME              LH-I-66
EXPNO                  12
PROCNO                  1

F2 - Acquisition Parameters
Date_            20220301
Time                19.06 h
INSTRUM             spect
PROBHD      Z114607_0188 (
PULPROG        deptsp135.b
TD                 119044
SOLVENT             CDCl3
NS                   1000
DS                      4
SWH            35714.285 Hz
FIDRES          0.600018 Hz
AQ             1.6666160 sec
RG                 186.92
DW                 14.000 usec
DE                   7.44 usec
TE                  300.0 K
CNST2         145.0000000
D1             1.00000000 sec
D2             0.00344828 sec
D12            0.00002000 sec
TD0                     1
SFO1          150.9178962 MHz
NUC1                  13C
P1                  11.80 usec
P13               2000.00 usec
PLW0         0 W
PLW1          85.00000000 W
SPNAM[5]      Crp60comp.4
SPOAL5              0.500
SPOFFS5      0 Hz
SPW5          18.08300018 W
SFO2          600.1324005 MHz
NUC2                   1H
CPDPRG[2          waltz64
P3                  10.20 usec
P4                  20.40 usec
PCPD2               70.00 usec
PLW2          27.00000000 W
PLW12          0.57327998 W

F2 - Processing parameters
SI                 131072
SF            150.9028085 MHz
WDW                    EM
SSB          0
LB                   1.00 Hz
GB           0
PC                   1.40
```

^{1}H-^{1}H COSY NMR spectrum of compound 33

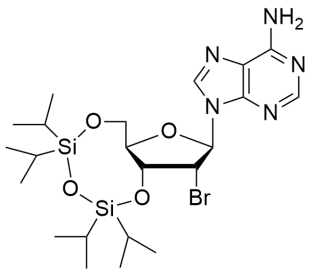

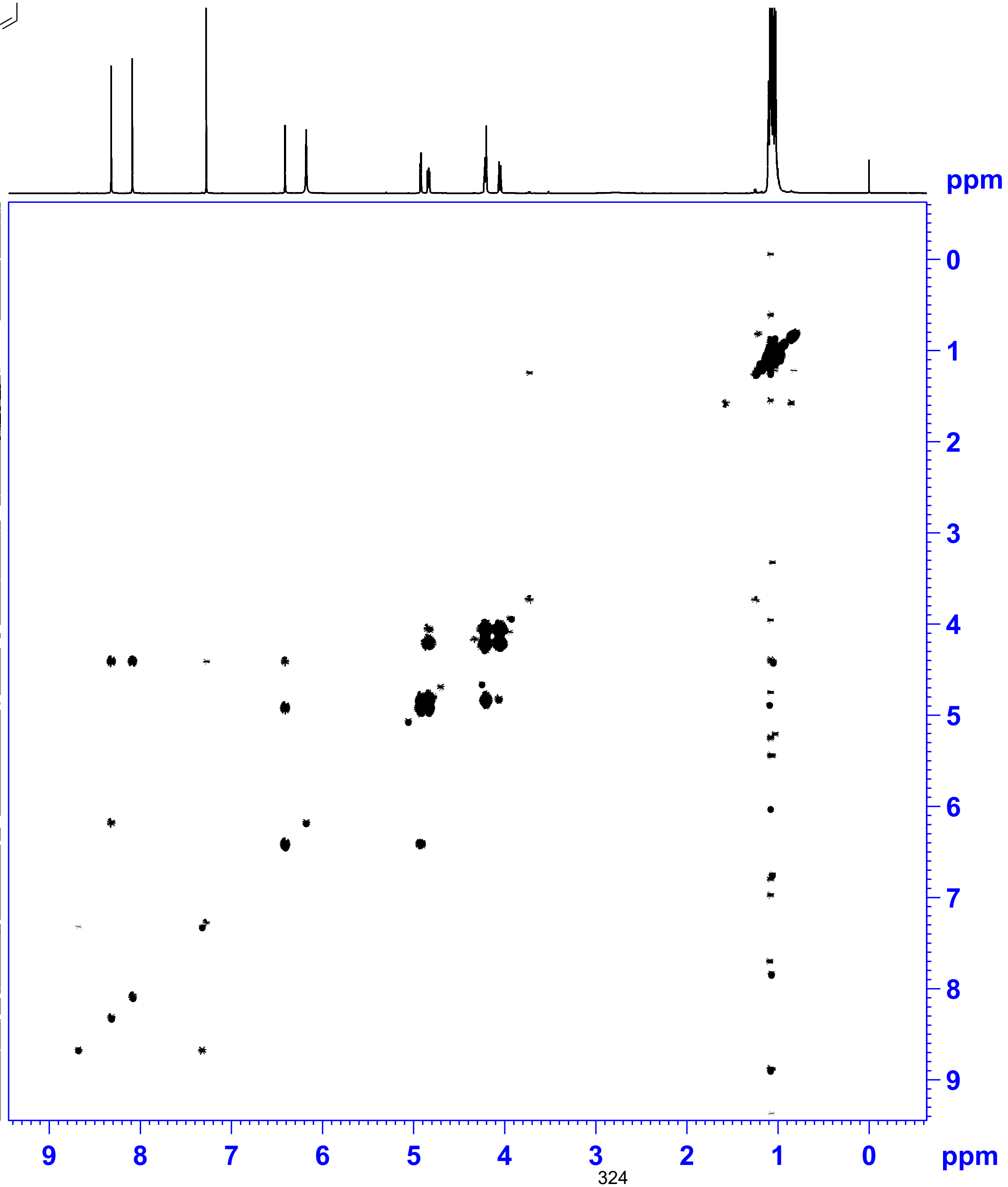


```
Current Data Parameters
NAME              LH-I-66
EXPNO                  13
PROCNO                  1

F2 - Acquisition Parameters
Date_            20220301
Time                19.17 h
INSTRUM             spect
PROBHD    Z114607_0188 (
PULPROG     cosygpmfppqf
TD                   2048
SOLVENT             CDCl3
NS                      2
DS                      8
SWH              6048.387 Hz
FIDRES           5.906628 Hz
AQ              0.1693013 sec
RG                 186.92
DW                 82.667 usec
DE                   6.50 usec
TE                  300.0 K
D0             0.00000300 sec
D1             0.89145529 sec
D11            0.03000000 sec
D12            0.00002000 sec
D13            0.00000400 sec
D16            0.00020000 sec
IN0            0.00016540 sec
TDav                    1
SFO1          600.1326500 MHz
NUC1                   1H
P1                  10.00 usec
P17               2500.00 usec
PLW1          26.60000038 W
PLW10          4.25600004 W
GPNAM[1]       SMSQ10.100
GPZ1                16.00 %
GPNAM[2]       SMSQ10.100
GPZ2                12.00 %
GPNAM[3]       SMSQ10.100
GPZ3                40.00 %
P16               1000.00 usec

F1 - Acquisition parameters
TD                    256
SFO1             600.1327 MHz
FIDRES          47.233978 Hz
SW                 10.074 ppm
FnMODE                 QF

F2 - Processing parameters
SI                   1024
SF            600.1300052 MHz
WDW                  SINE
SSB      0
LB       0 Hz
GB       0
PC                   1.40

F1 - Processing parameters
SI                   1024
MC2                    QF
SF            600.1300044 MHz
WDW                  SINE
SSB      0
LB       0 Hz
GB       0
```

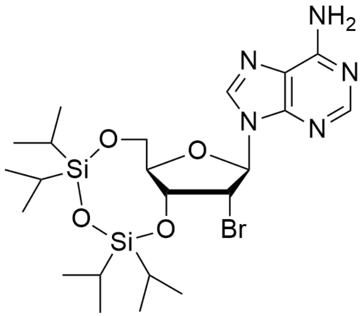

^{1}H-^{13}C HSQC NMR spectrum of compound 33

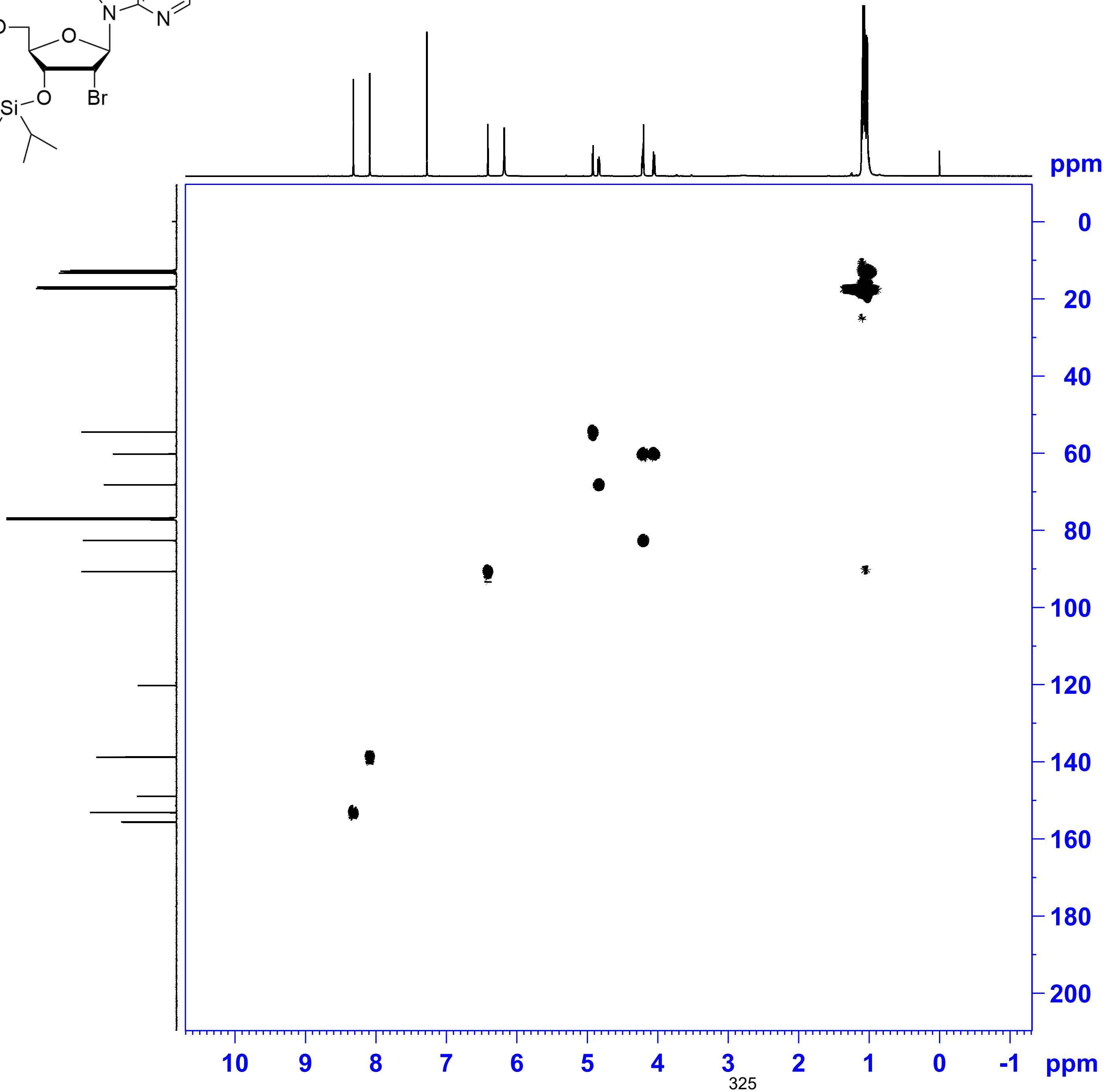


```
Current Data Parameters
NAME              LH-I-66
EXPNO                  14
PROCNO                  1

F2 - Acquisition Parameters
Date_            20220301
Time                19.26 h
INSTRUM             spect
PROBHD   Z114607_0188 (
PULPROG  hsqcedetgpsp.3
TD                   1024
SOLVENT             CDCl3
NS                      2
DS                     32
SWH              7211.539 Hz
FIDRES          14.085036 Hz
AQ              0.0709973 sec
RG                 186.92
DW                 69.333 usec
DE                   6.50 usec
TE                  300.3 K
CNST2         145.0000000
D0             0.00000300 sec
D1             0.80000001 sec
D4             0.00172414 sec
D11            0.03000000 sec
D16            0.00020000 sec
D21            0.00360000 sec
IN0            0.00001510 sec
TDav                    1
ZGOPTNS
SFO1       600.1328223 MHz
NUC1                   1H
P1                  10.00 usec
P2                  20.00 usec
PLW1          26.60000038 W
SFO2       150.9178988 MHz
NUC2                  13C
CPDPRG[2            garp4
P3                  11.80 usec
P14                500.00 usec
P31               1730.00 usec
PCPD2               60.00 usec
PLW0     0 W
PLW2          85.00000000 W
PLW12          3.28760004 W
SPNAM[3] Crp60,0.5,20.1
SPOAL3              0.500
SPOFFS3  0 Hz
SPW3          18.08300018 W
SPNAM[18  Crp60_xfilt.2
SPOAL18             0.500
SPOFFS18 0 Hz
SPW18          5.22629976 W
GPNAM[1]       SMSQ10.100
GPZ1                80.00 %
GPNAM[2]       SMSQ10.100
GPZ2                20.10 %
P16               1000.00 usec

F1 - Acquisition parameters
TD                    256
SFO1             150.9179 MHz
FIDRES         258.692047 Hz
SW                219.408 ppm
FnMODE      Echo-Antiecho

F2 - Processing parameters
SI                   1024
SF         600.1300038 MHz
WDW                 QSINE
SSB                     2
LB       0 Hz
GB       0
PC                   1.40

F1 - Processing parameters
SI                   1024
MC2          echo-antiecho
SF         150.9028085 MHz
WDW                 QSINE
SSB                     2
LB       0 Hz
GB       0
```

S4. Copies of High Resolution Mass Spectra of Compounds Reported

LH-1-49

ASEP_25JUL22_113 (0.053) Is (1.00,1.00) C22H39N5Si2O5H

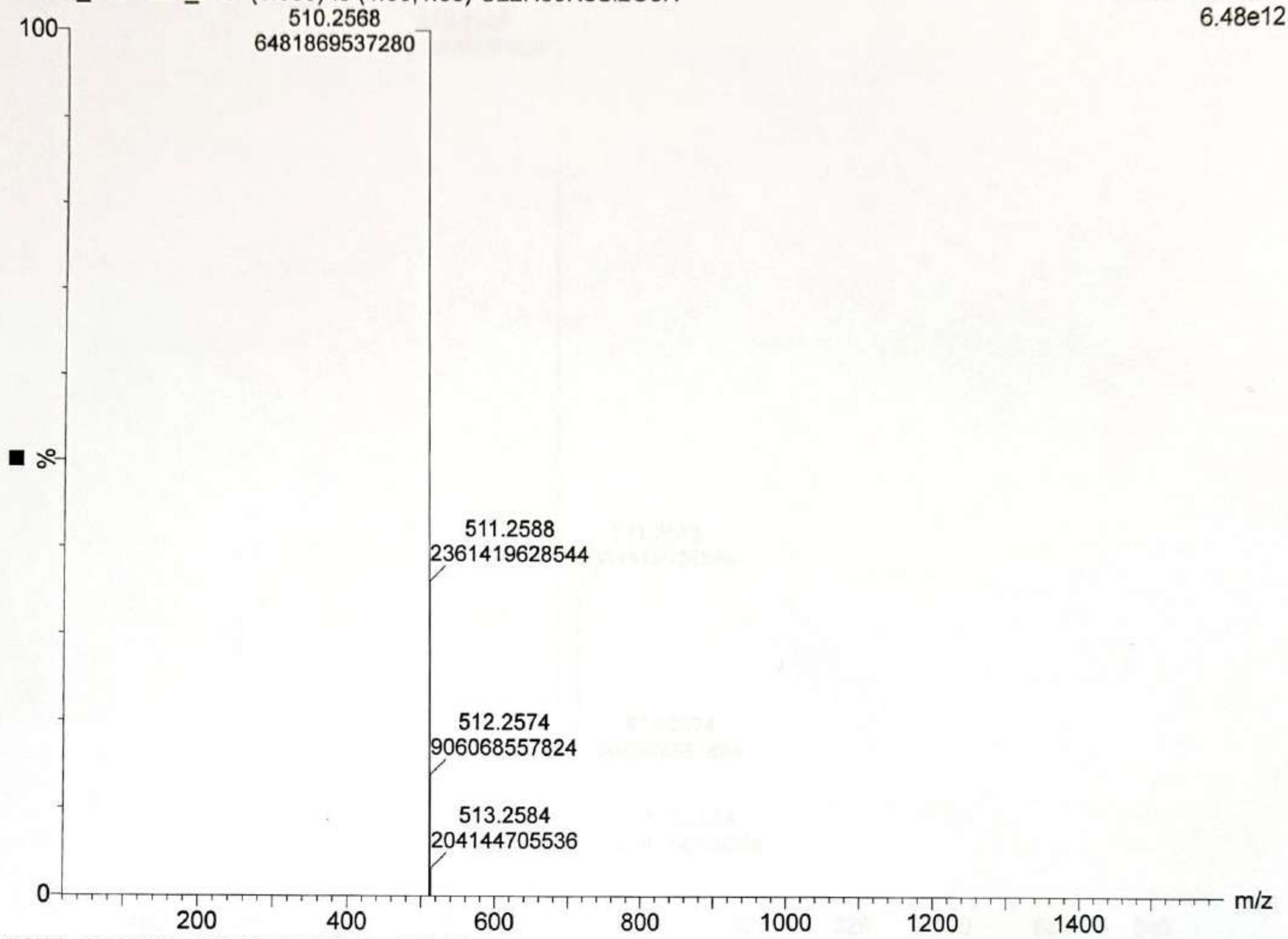

ASEP_25JUL22_113 29 (0.586) Cm (29:42)

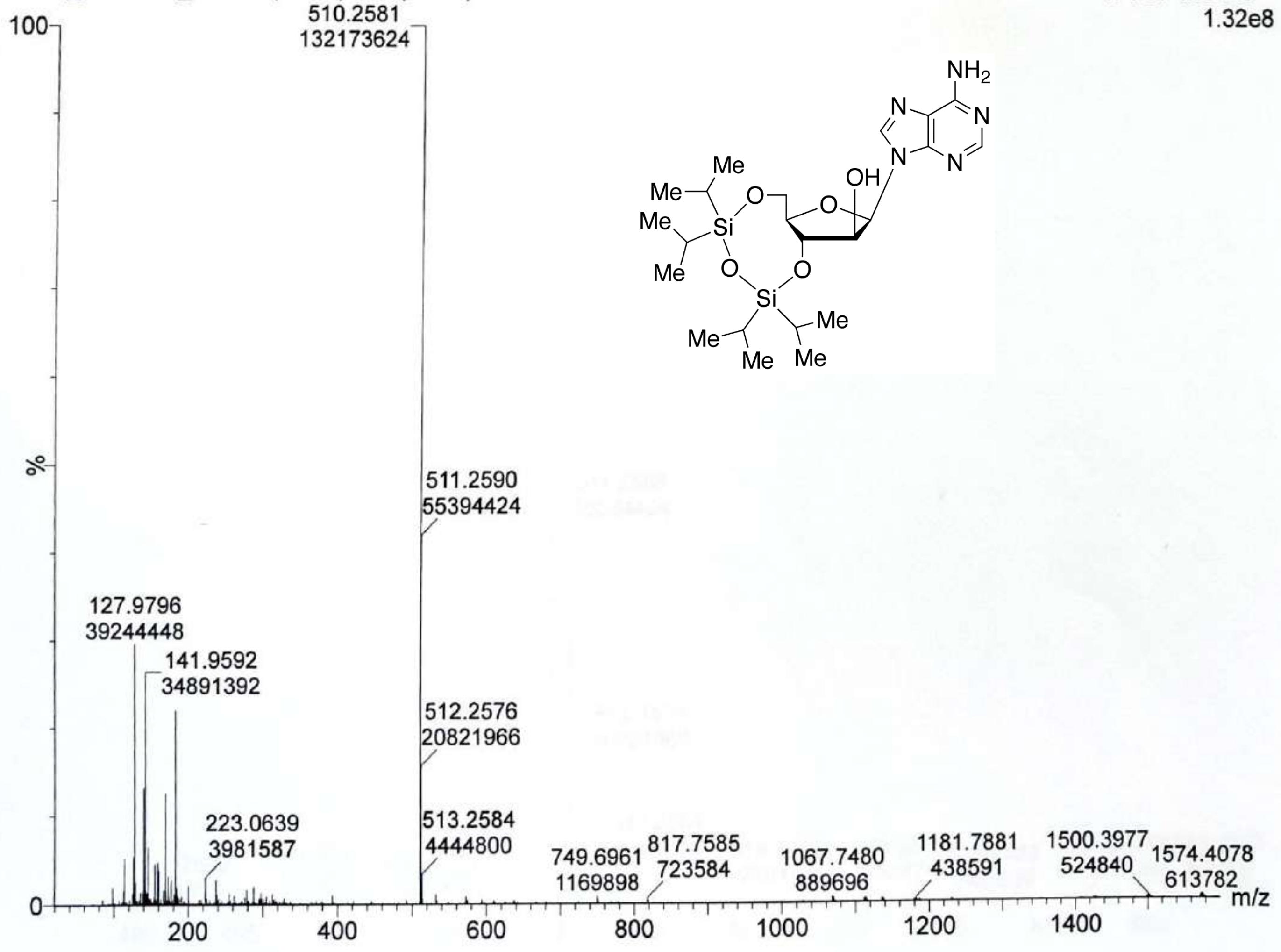

LH-11-67

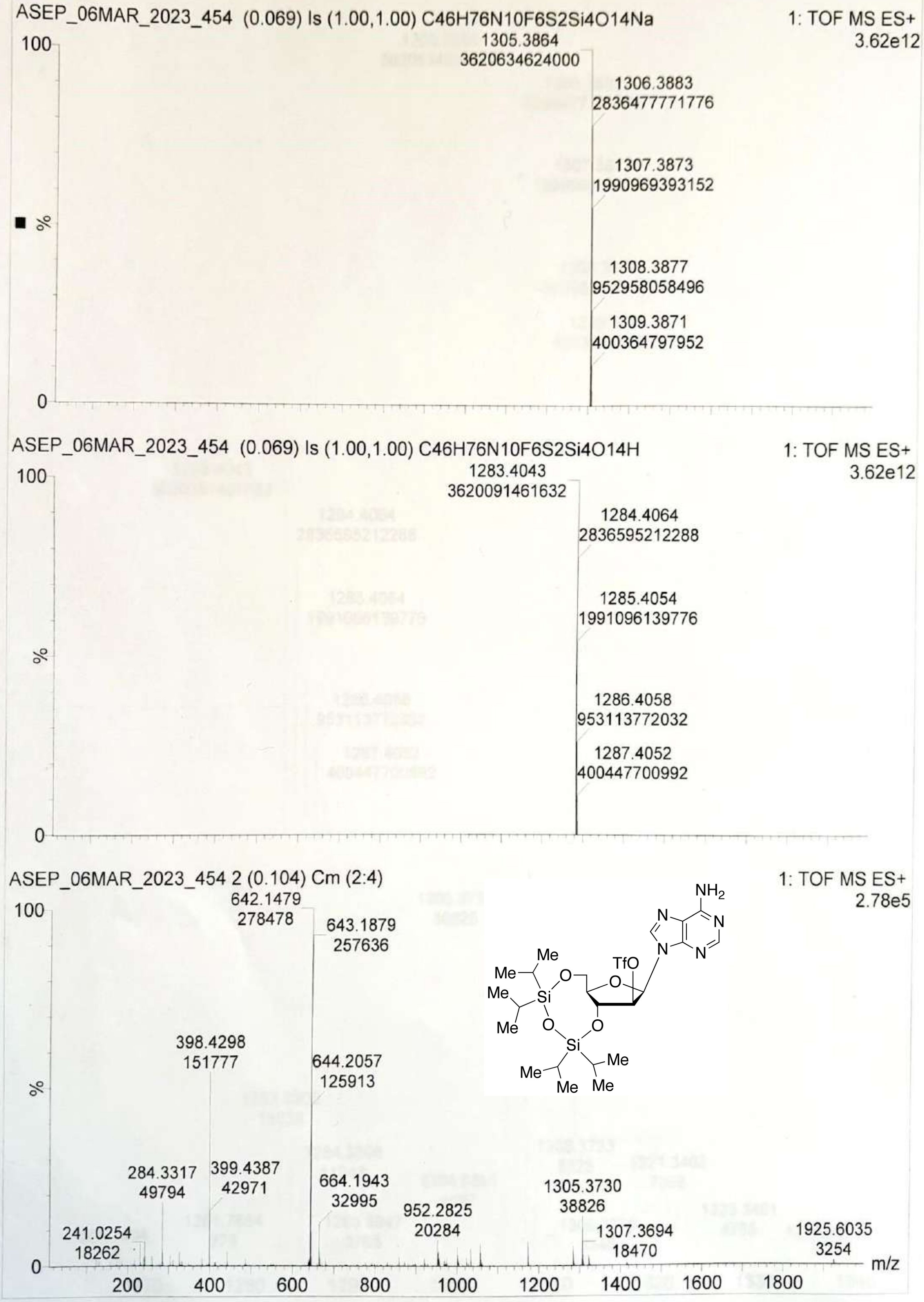
ASEP_06MAR_2023_454 (0.069) Is (1.00,1.00) C46H76N10F6S2Si4O14Na
1: TOF MS ES+
3.62e12
100
%
0
1305.3864
3620634624000
1306.3883
2836477771776
1307.3873
1990969393152
1308.3877
952958058496
1309.3871
400364797952
ASEP_06MAR_2023_454 (0.069) Is (1.00,1.00) C46H76N10F6S2Si4O14H
1: TOF MS ES+
3.62e12
1283.4043
3620091461632
1284.4064
2836595212288
1285.4054
1991096139776
1286.4058
953113772032
1287.4052
400447700992
ASEP_06MAR_2023_454 2 (0.104) Cm (2:4)
1: TOF MS ES+
2.78e5
642.1479
278478
643.1879
257636
398.4298
151777
644.2057
125913
284.3317
49794
399.4387
42971
664.1943
32995
952.2825
20284
1305.3730
38826
241.0254
18262
1307.3694
18470
1925.6035
3254
m/z
200
400
600
800
1000
1200
1400
1600
1800
NH2
N
TfO
O
Me
Si
Me

LH-1-55

ASEP_25JUL22_114 (0.053) Is (1.00,1.00) C23H38N6Si2SeO4H — 1: TOF MS ES+ 3.42e12

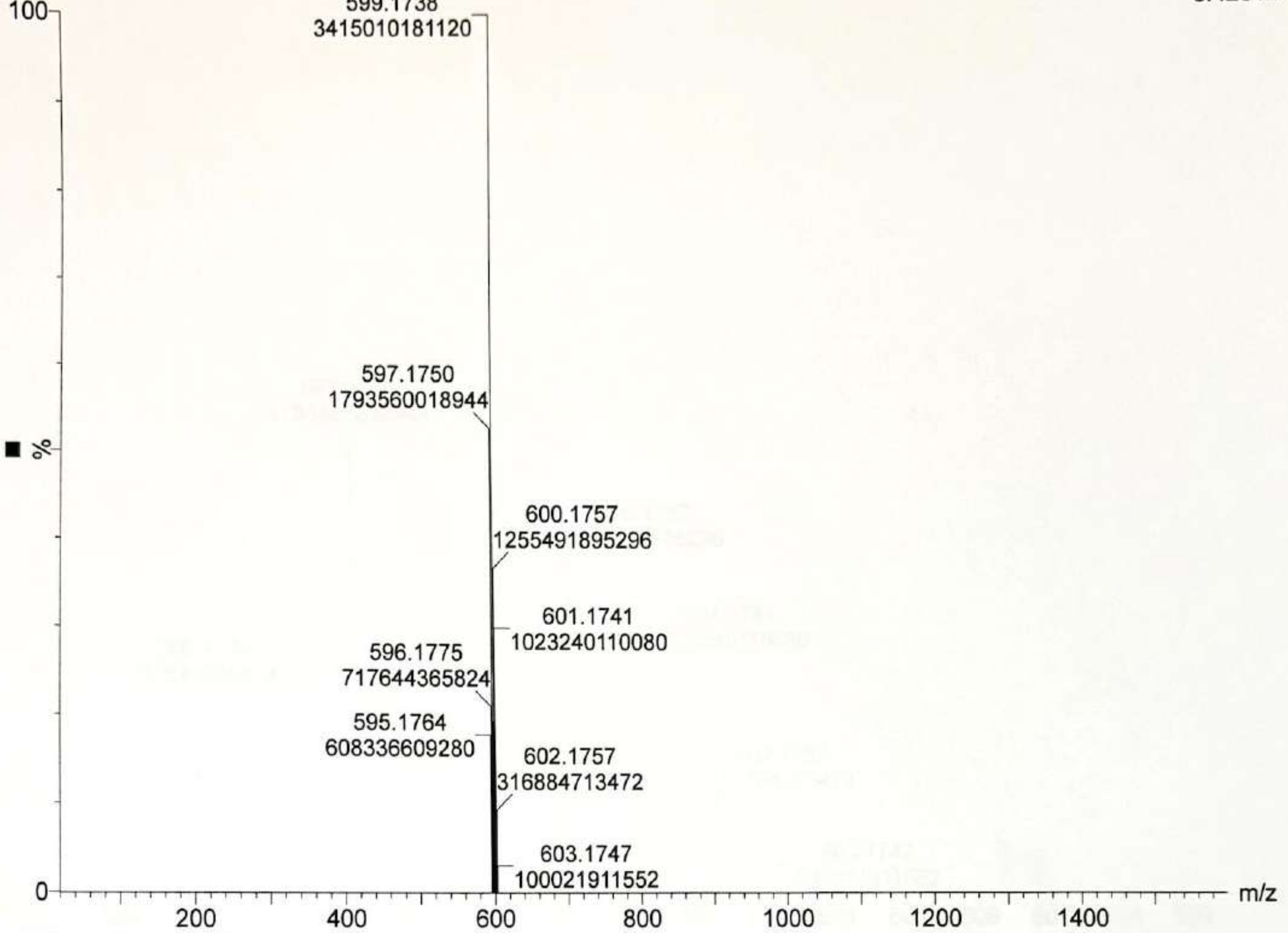

ASEP_25JUL22_114 26 (0.536) Cm (26:40) — 1: TOF MS ES+ 3.31e7

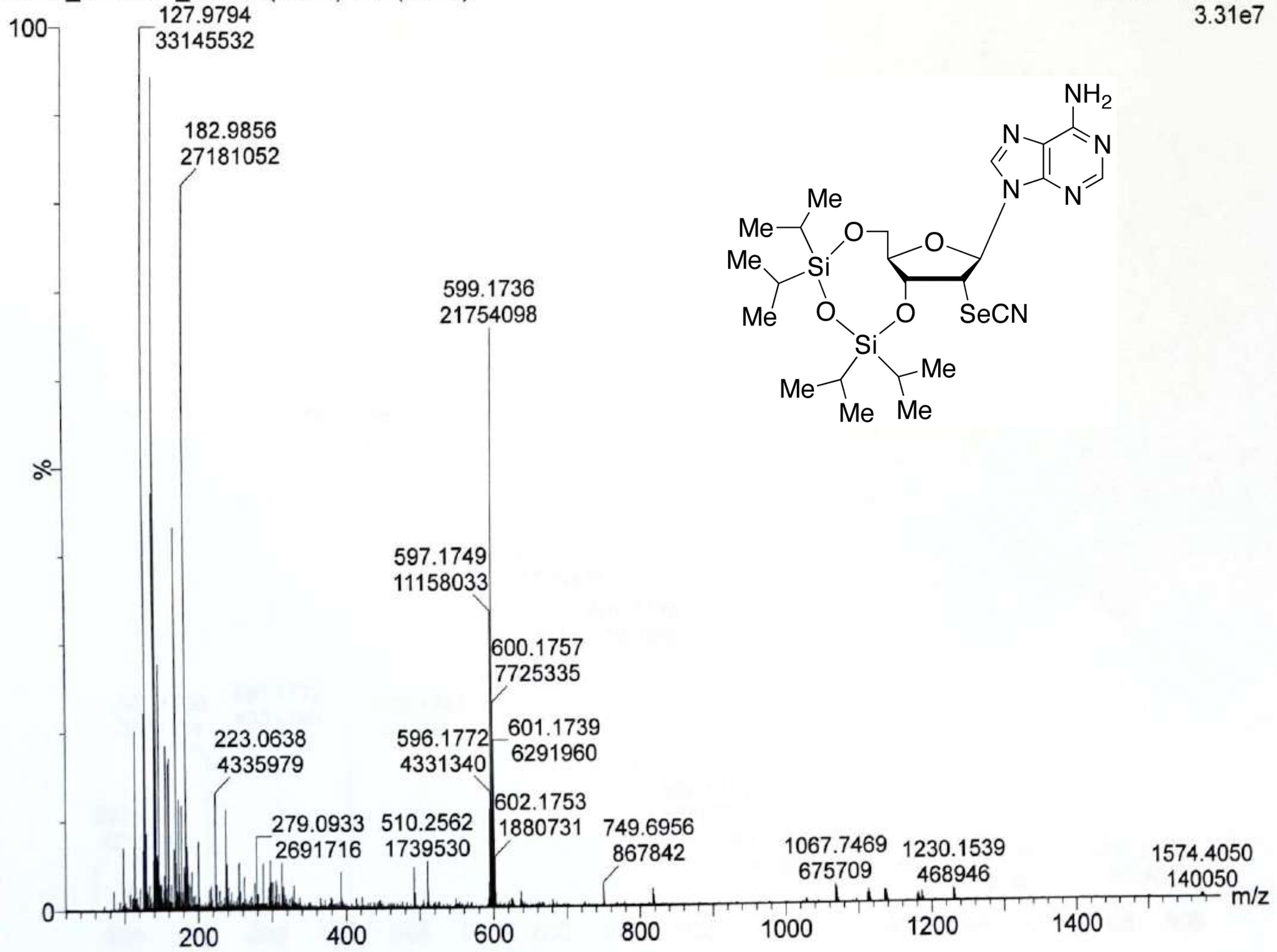

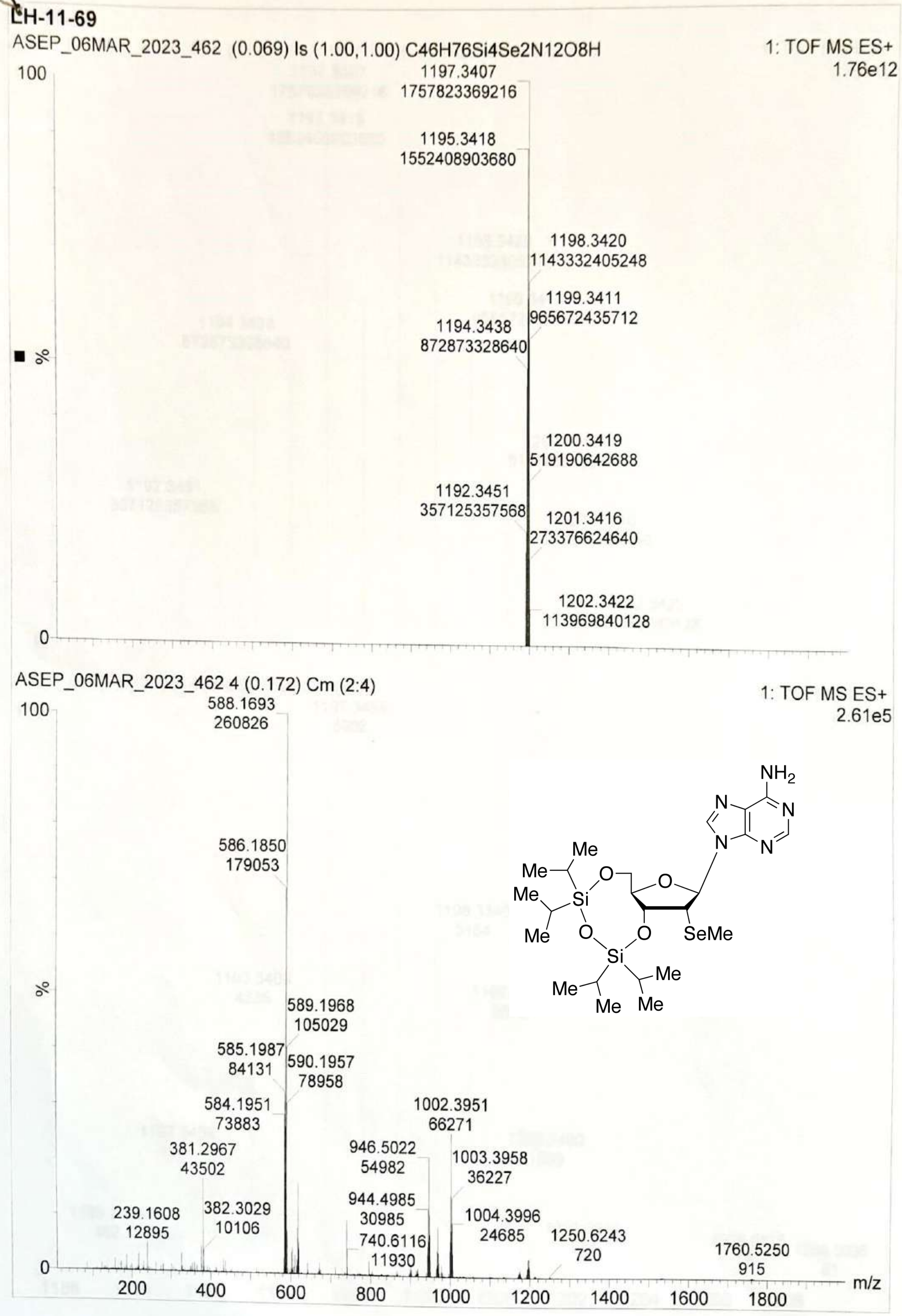
LH-11-69
ASEP_06MAR_2023_462 (0.069) Is (1.00,1.00) C46H76Si4Se2N12O8H
1: TOF MS ES+
1.76e12
100
%
0
1197.3407
1757823369216
1195.3418
1552408903680
1198.3420
1143332405248
1199.3411
965672435712
1194.3438
872873328640
1200.3419
519190642688
1192.3451
357125357568
1201.3416
273376624640
1202.3422
113969840128
ASEP_06MAR_2023_462 4 (0.172) Cm (2:4)
1: TOF MS ES+
2.61e5
588.1693
260826
586.1850
179053
589.1968
105029
585.1987
84131
590.1957
78958
584.1951
73883
1002.3951
66271
381.2967
43502
946.5022
54982
1003.3958
36227
944.4985
30985
239.1608
12895
382.3029
10106
1004.3996
24685
740.6116
11930
1250.6243
720
1760.5250
915
200
400
600
800
1000
1200
1400
1600
1800
m/z
NH2
N
N
N
N
Me
Me
O
O
Me
Si
Me
O
O
SeMe
Si
Me
Me
Me
Me

LH-11-70

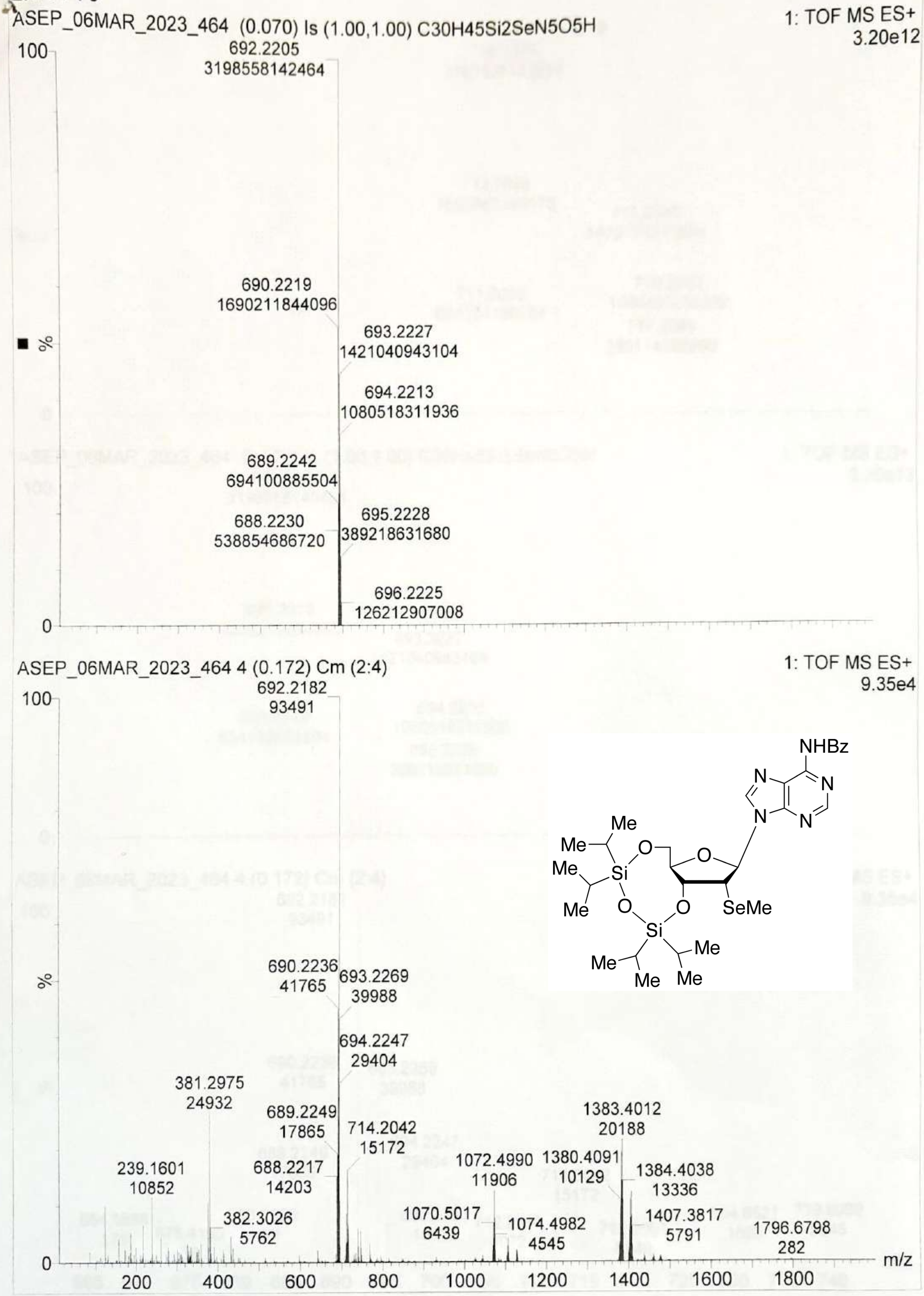
ASEP_06MAR_2023_464 (0.070) Is (1.00,1.00) C30H45Si2SeN5O5H
1: TOF MS ES+
3.20e12
692.2205
3198558142464
690.2219
1690211844096
693.2227
1421040943104
694.2213
1080518311936
689.2242
694100885504
695.2228
389218631680
688.2230
538854686720
696.2225
126212907008
ASEP_06MAR_2023_464 4 (0.172) Cm (2:4)
1: TOF MS ES+
9.35e4
692.2182
93491
690.2236
41765
693.2269
39988
694.2247
29404
381.2975
24932
689.2249
17865
714.2042
15172
1383.4012
20188
239.1601
10852
688.2217
14203
1072.4990
11906
1380.4091
10129
1384.4038
13336
382.3026
5762
1070.5017
6439
1074.4982
4545
1407.3817
5791
1796.6798
282
%
m/z
NHBz
N
Me
O
SeMe
Si
Me

LH-11-71

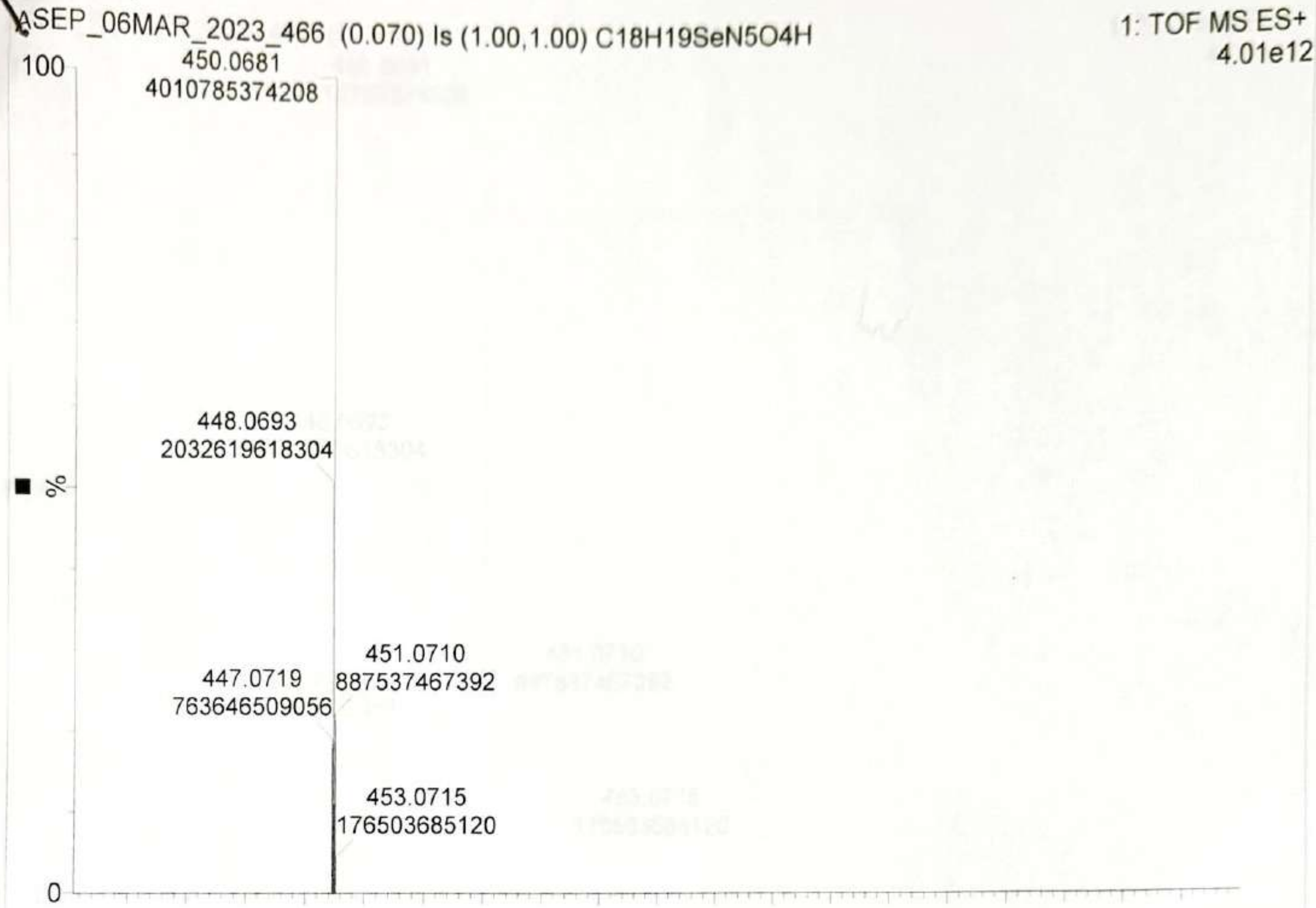
ASEP_06MAR_2023_466 (0.070) Is (1.00,1.00) C18H19SeN5O4H
1: TOF MS ES+
4.01e12
100
450.0681
4010785374208
448.0693
2032619618304
%
447.0719
763646509056
451.0710
887537467392
453.0715
176503685120
0

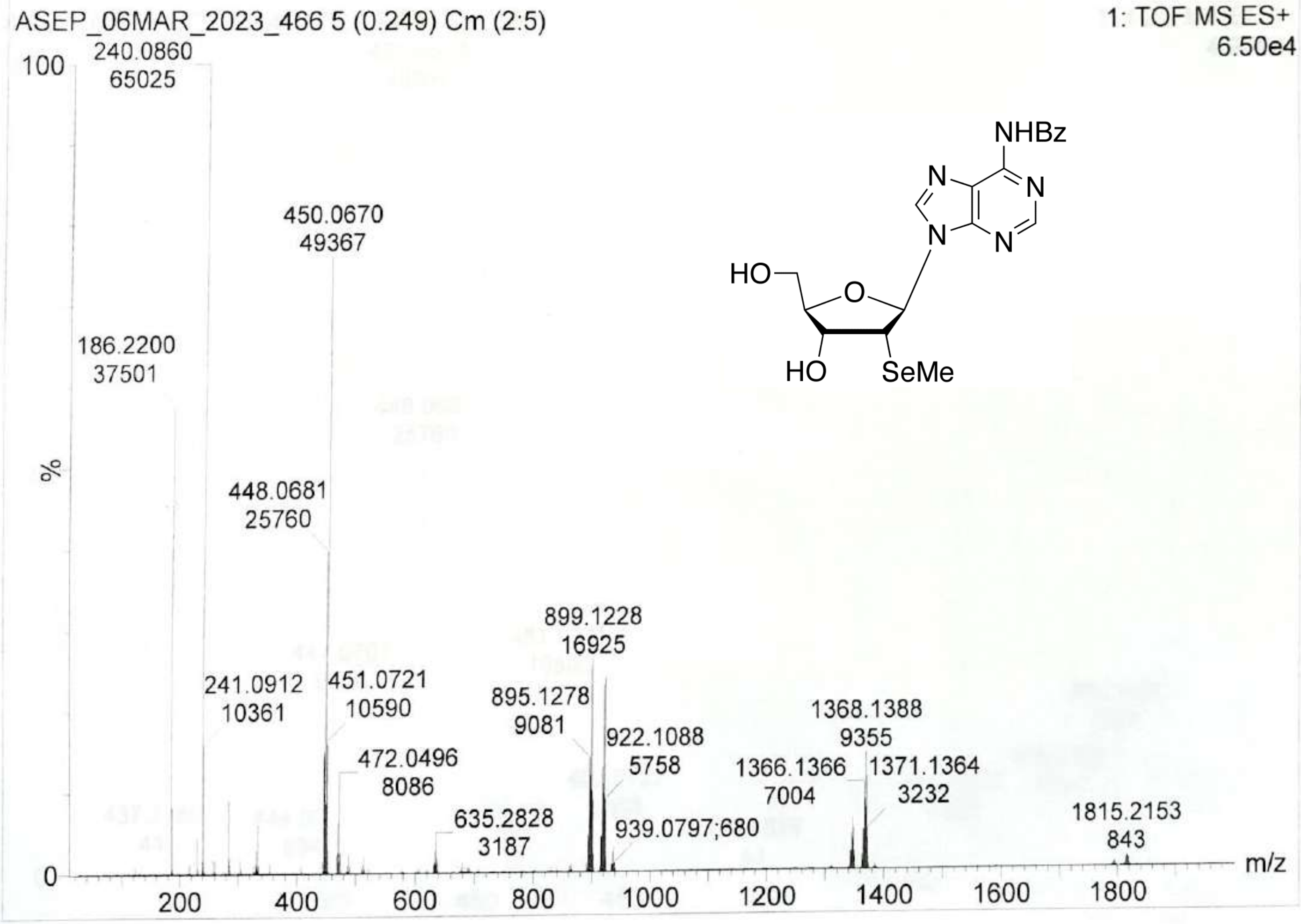
ASEP_06MAR_2023_466 5 (0.249) Cm (2:5)
1: TOF MS ES+
6.50e4
100
240.0860
65025
450.0670
49367
186.2200
37501
%
448.0681
25760
899.1228
16925
241.0912
10361
451.0721
10590
895.1278
9081
472.0496
8086
922.1088
5758
1368.1388
9355
1366.1366
7004
1371.1364
3232
635.2828
3187
939.0797;680
1815.2153
843
0
200
400
600
800
1000
1200
1400
1600
1800
m/z
NHBz
N
N
N
N
HO
O
HO
SeMe

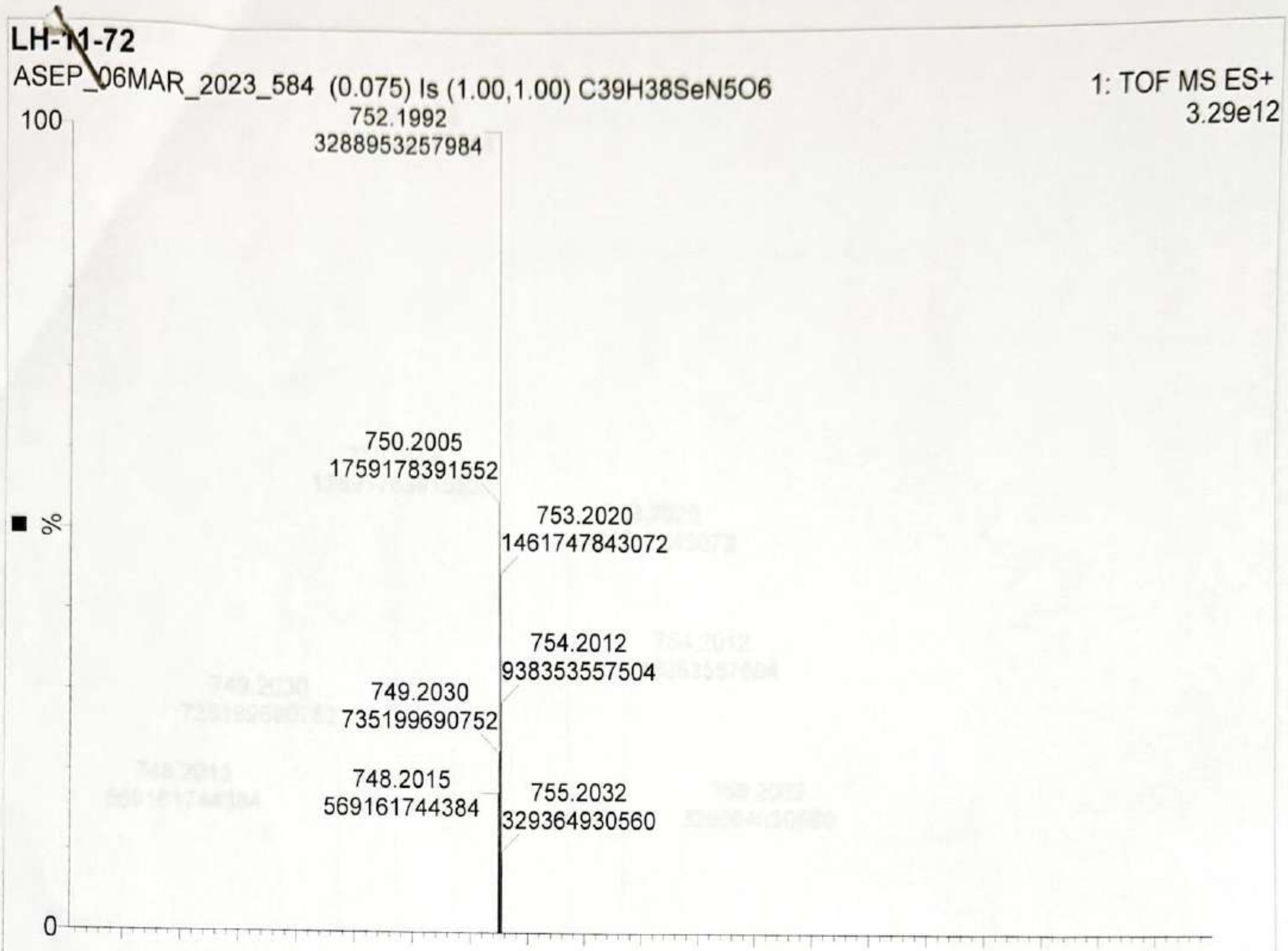
LH-11-72
ASEP_06MAR_2023_584 (0.075) Is (1.00,1.00) C39H38SeN5O6
1: TOF MS ES+
3.29e12
100
%
0
752.1992
3288953257984
750.2005
1759178391552
753.2020
1461747843072
754.2012
938353557504
749.2030
735199690752
748.2015
569161744384
755.2032
329364930560

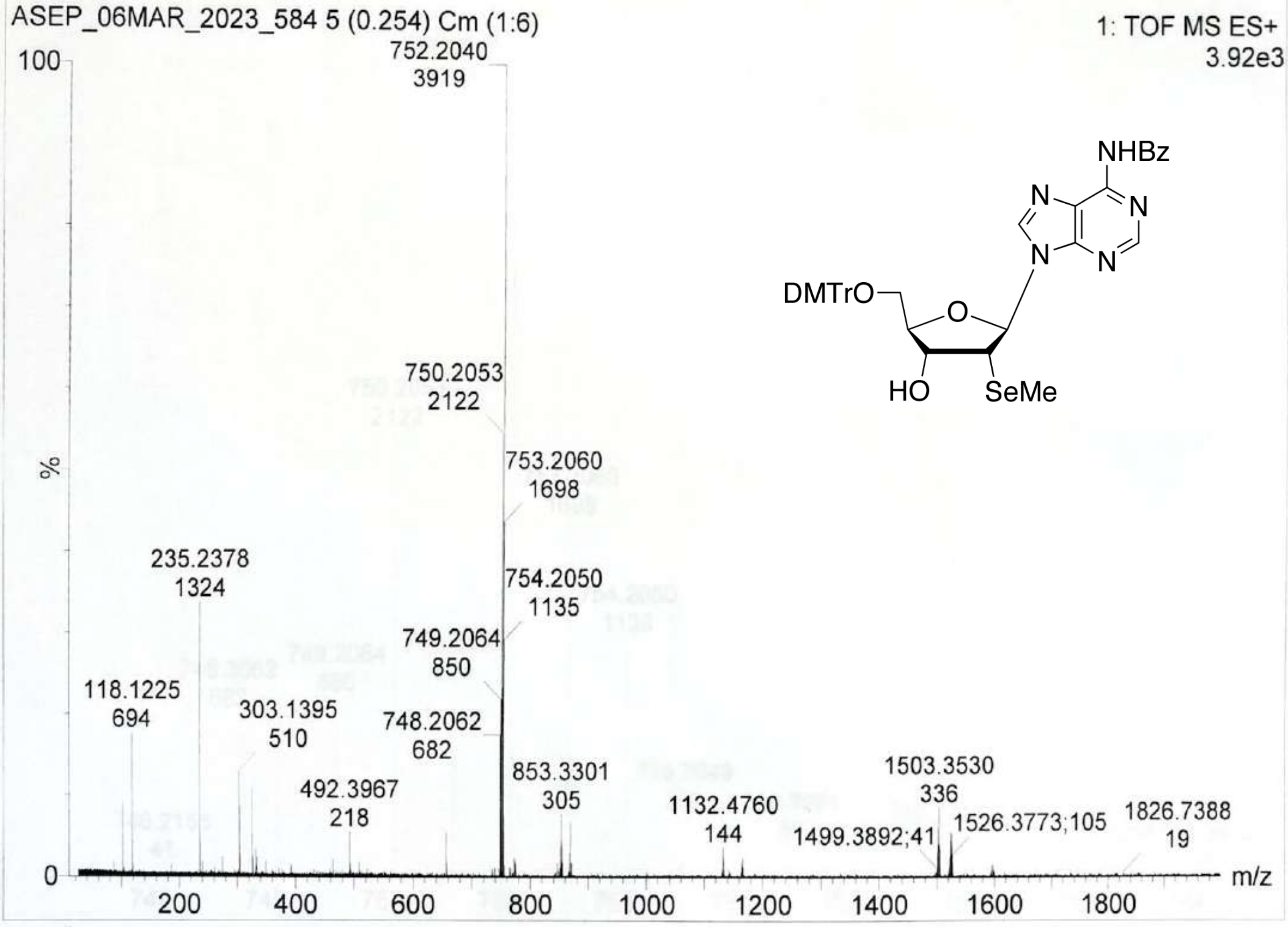
ASEP_06MAR_2023_584 5 (0.254) Cm (1:6)
1: TOF MS ES+
3.92e3
100
%
0
752.2040
3919
750.2053
2122
753.2060
1698
235.2378
1324
754.2050
1135
749.2064
850
118.1225
694
303.1395
510
748.2062
682
853.3301
305
492.3967
218
1132.4760
144
1503.3530
336
1499.3892;41
1526.3773;105
1826.7388
19
200
400
600
800
1000
1200
1400
1600
1800
m/z
NHBz
N
N
N
N
DMTrO
O
HO
SeMe

LH-11-73

ASEP_06MAR_2023_574 (0.075) Is (1.00,1.00) C48H54PSeN7O7H

1: TOF MS ES+ 3.03e12

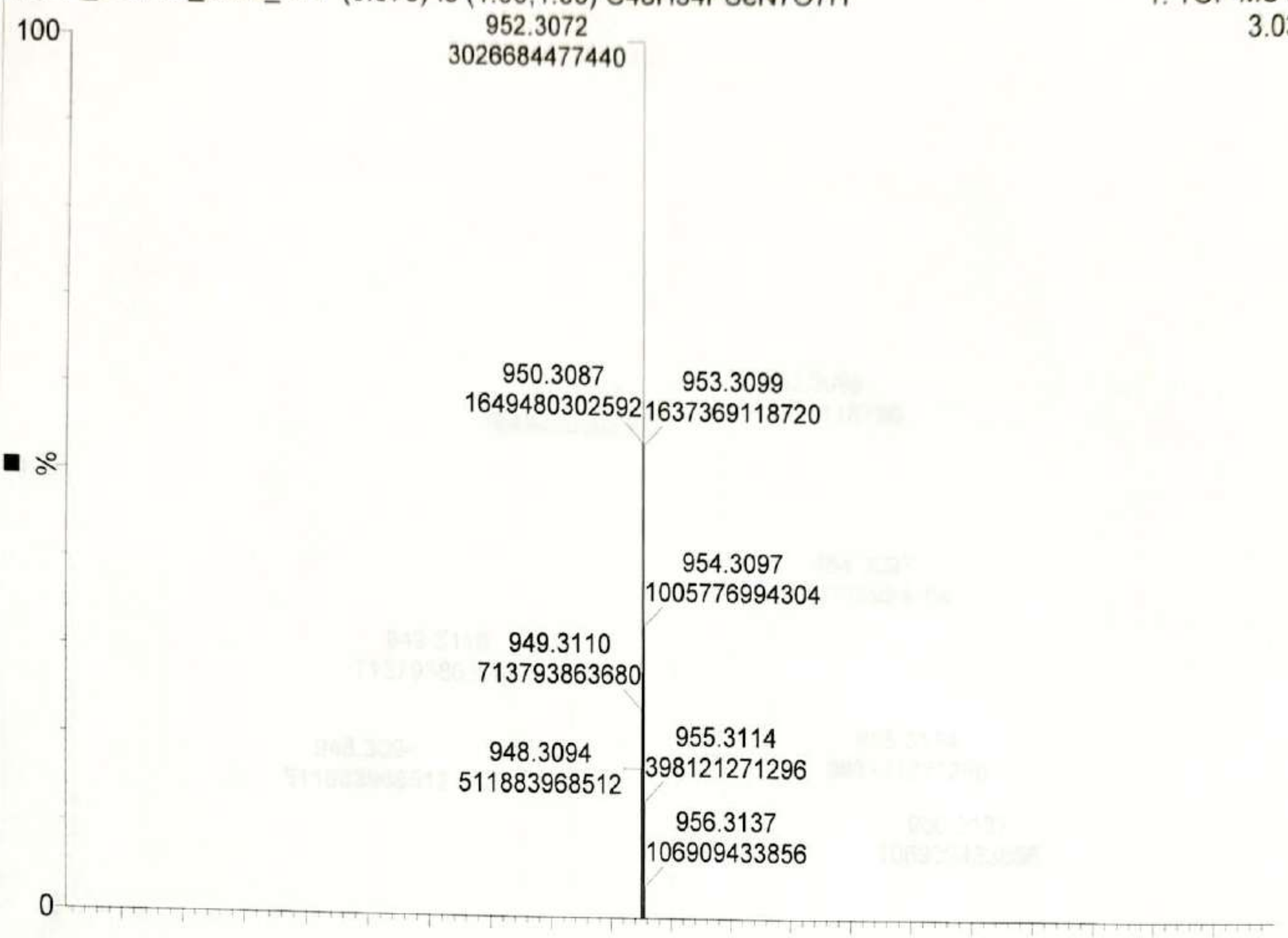

ASEP_06MAR_2023_574 4 (0.177) Cm (1:4)

1: TOF MS ES+ 6.43e4

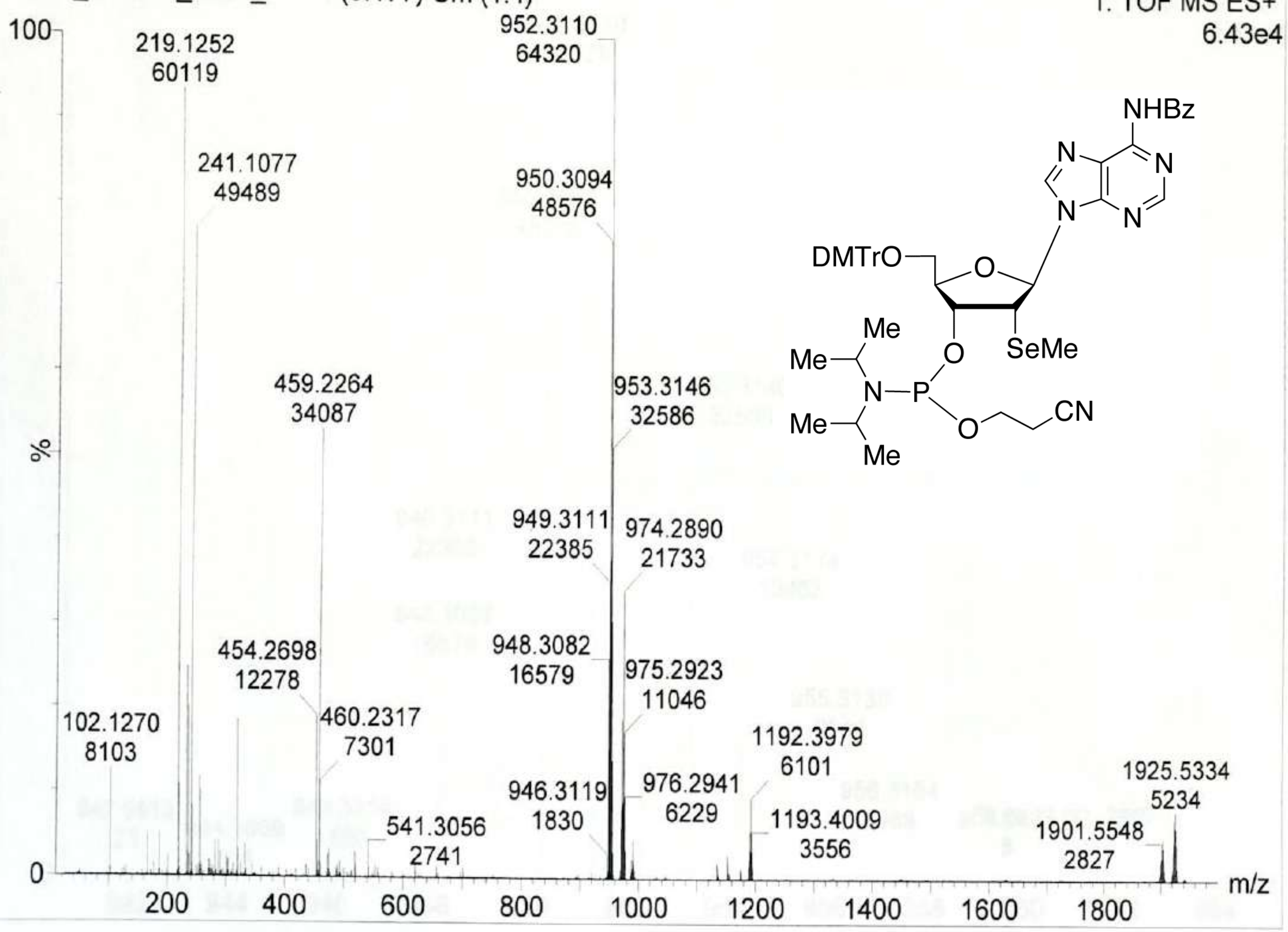

LH-11-76

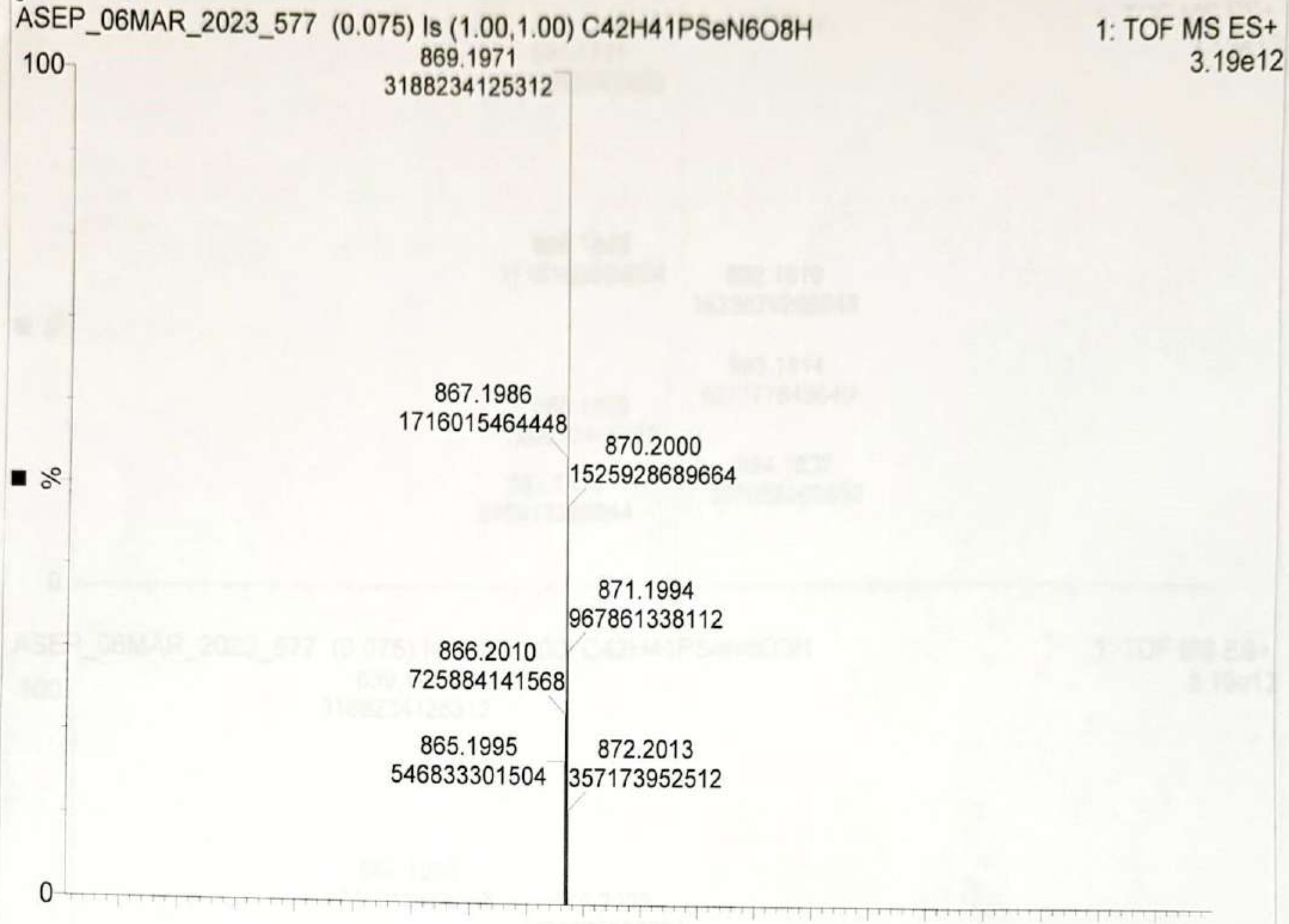
ASEP_06MAR_2023_577 (0.075) Is (1.00,1.00) C42H41PSeN6O8H
1: TOF MS ES+
3.19e12
100
%
0
869.1971
3188234125312
867.1986
1716015464448
870.2000
1525928689664
871.1994
967861338112
866.2010
725884141568
865.1995
546833301504
872.2013
357173952512

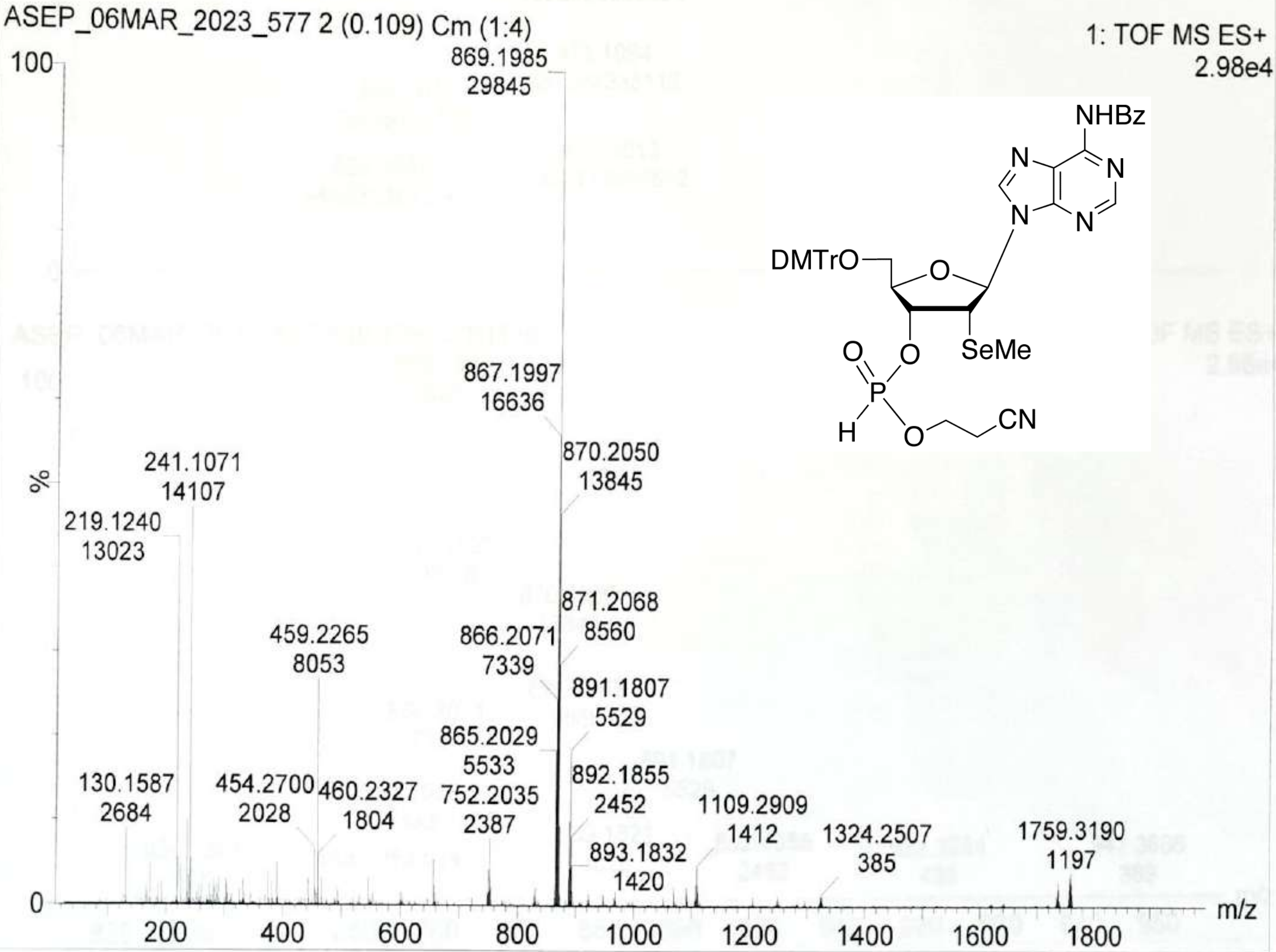
ASEP_06MAR_2023_577 2 (0.109) Cm (1:4)
1: TOF MS ES+
2.98e4
100
%
0
869.1985
29845
867.1997
16636
870.2050
13845
241.1071
14107
219.1240
13023
871.2068
8560
459.2265
8053
866.2071
7339
891.1807
5529
865.2029
5533
130.1587
2684
454.2700
2028
460.2327
1804
752.2035
2387
892.1855
2452
1109.2909
1412
1324.2507
385
1759.3190
1197
893.1832
1420
200
400
600
800
1000
1200
1400
1600
1800
m/z
NHBz
N
N
N
N
DMTrO
O
O
O
P
H
O
SeMe
CN

LH-11-74

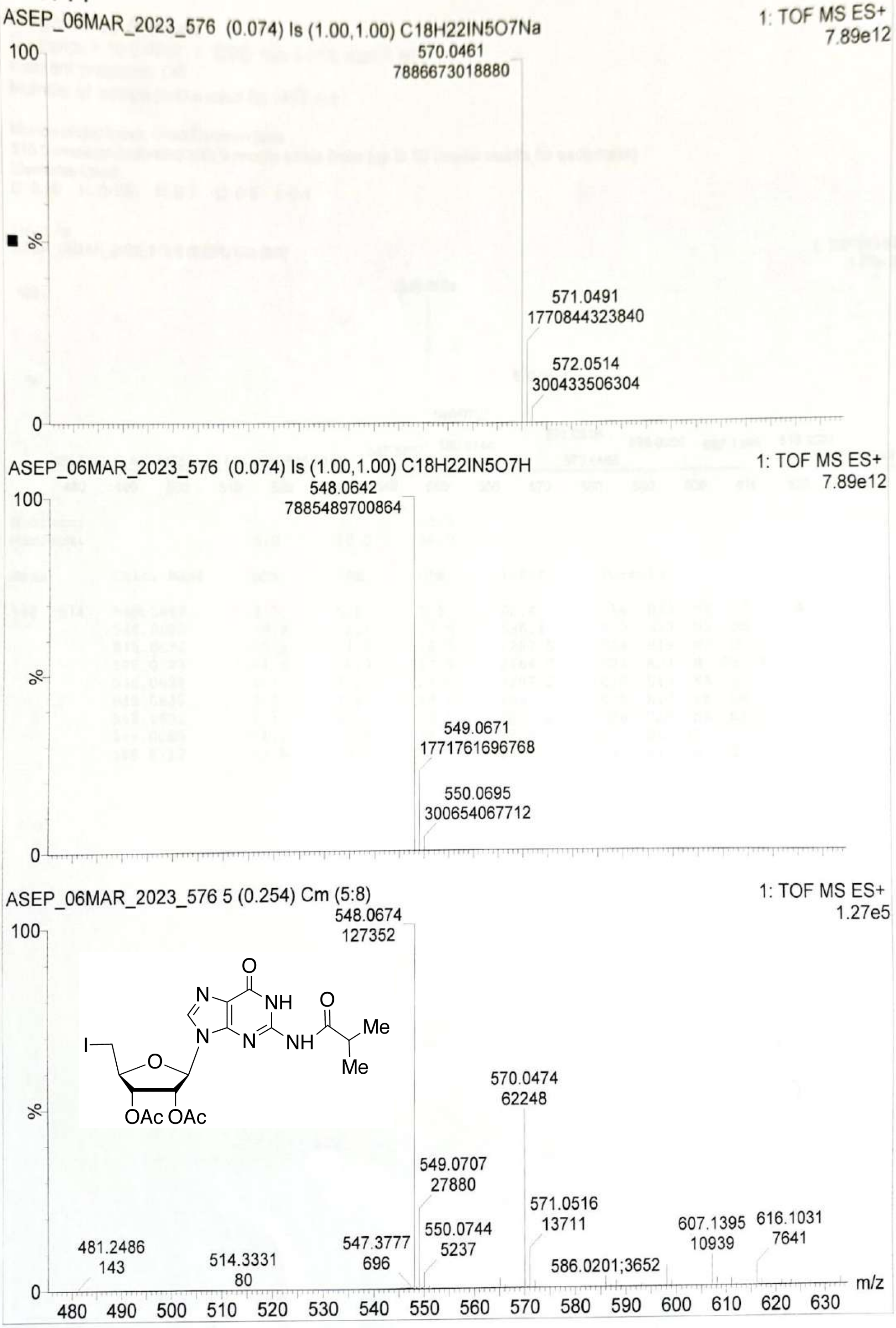
ASEP_06MAR_2023_576 (0.074) Is (1.00,1.00) C18H22IN5O7Na
1: TOF MS ES+
7.89e12
570.0461
7886673018880
571.0491
1770844323840
572.0514
300433506304
ASEP_06MAR_2023_576 (0.074) Is (1.00,1.00) C18H22IN5O7H
1: TOF MS ES+
7.89e12
548.0642
7885489700864
549.0671
1771761696768
550.0695
300654067712
ASEP_06MAR_2023_576 5 (0.254) Cm (5:8)
1: TOF MS ES+
1.27e5
548.0674
127352
570.0474
62248
549.0707
27880
571.0516
13711
550.0744
5237
607.1395
10939
616.1031
7641
481.2486
143
514.3331
80
547.3777
696
586.0201;3652
m/z
%
100
0
480
490
500
510
520
530
540
550
560
570
580
590
600
610
620
630
I
O
N
NH
NH
Me
Me
OAc OAc

LH-11-74

ASEP_06MAR_2023_576 (0.074) Is (1.00,1.00) C18H22IN5O7Na

1: TOF MS ES+ 7.89e12

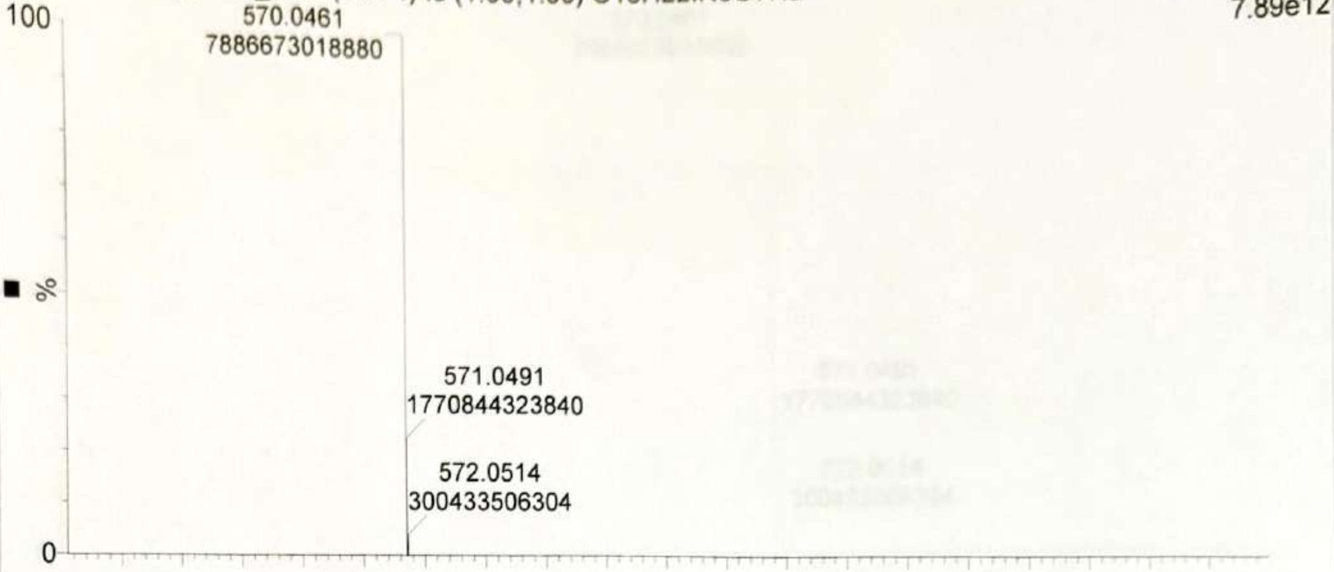

ASEP_06MAR_2023_576 (0.074) Is (1.00,1.00) C18H22IN5O7H

1: TOF MS ES+ 7.89e12

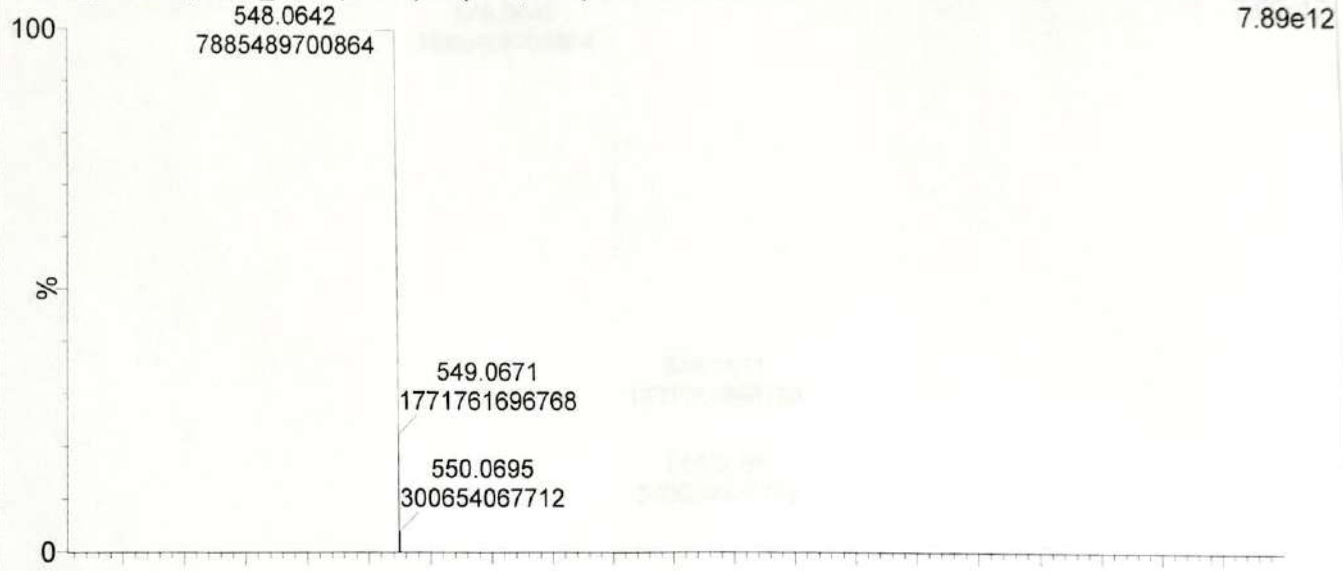

ASEP_06MAR_2023_576 5 (0.254) Cm (5:8)

1: TOF MS ES+ 1.78e5

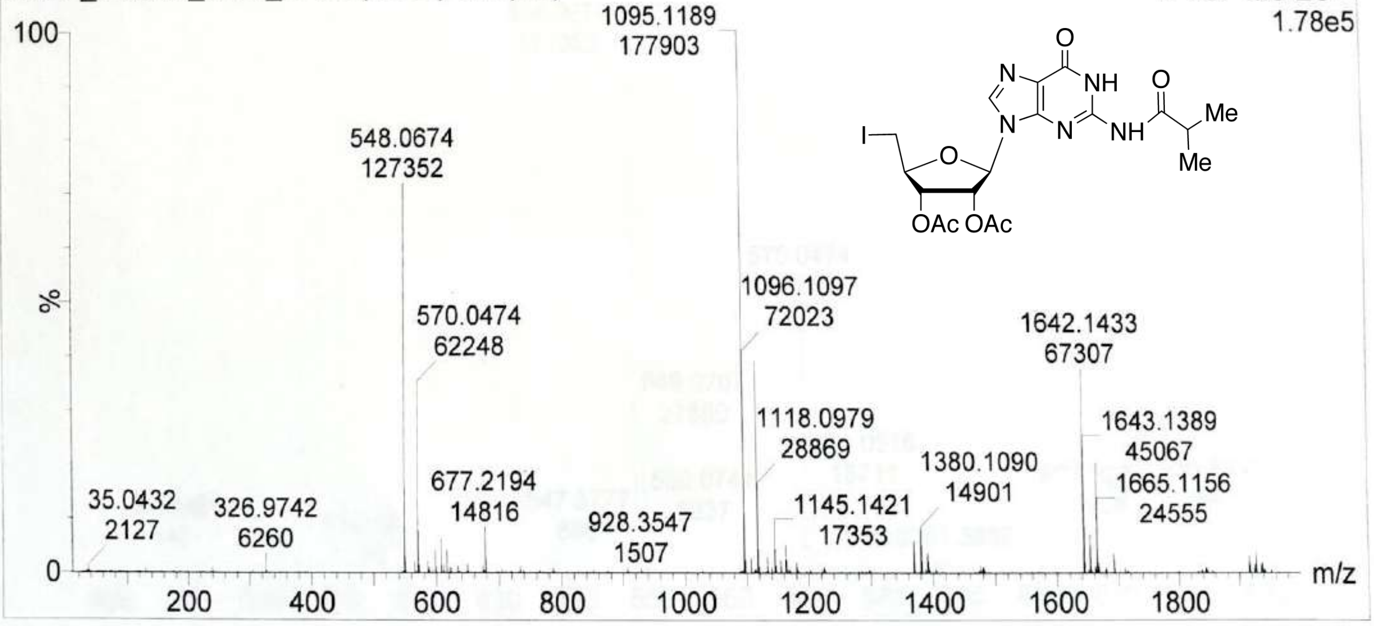

LH-11-53

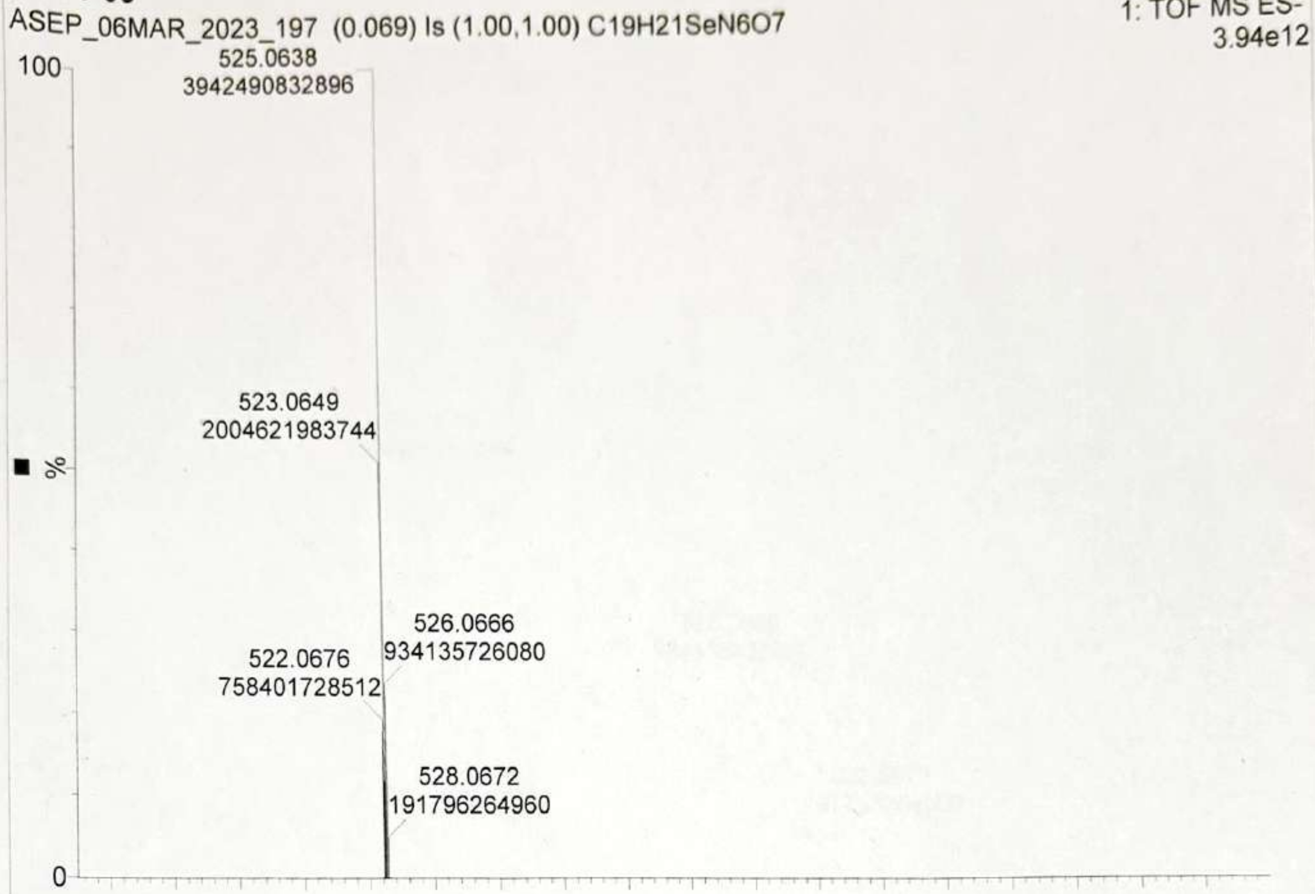
ASEP_06MAR_2023_197 (0.069) Is (1.00,1.00) C19H21SeN6O7
1: TOF MS ES-
3.94e12
525.0638
3942490832896
523.0649
2004621983744
526.0666
934135726080
522.0676
758401728512
528.0672
191796264960
100
%
0

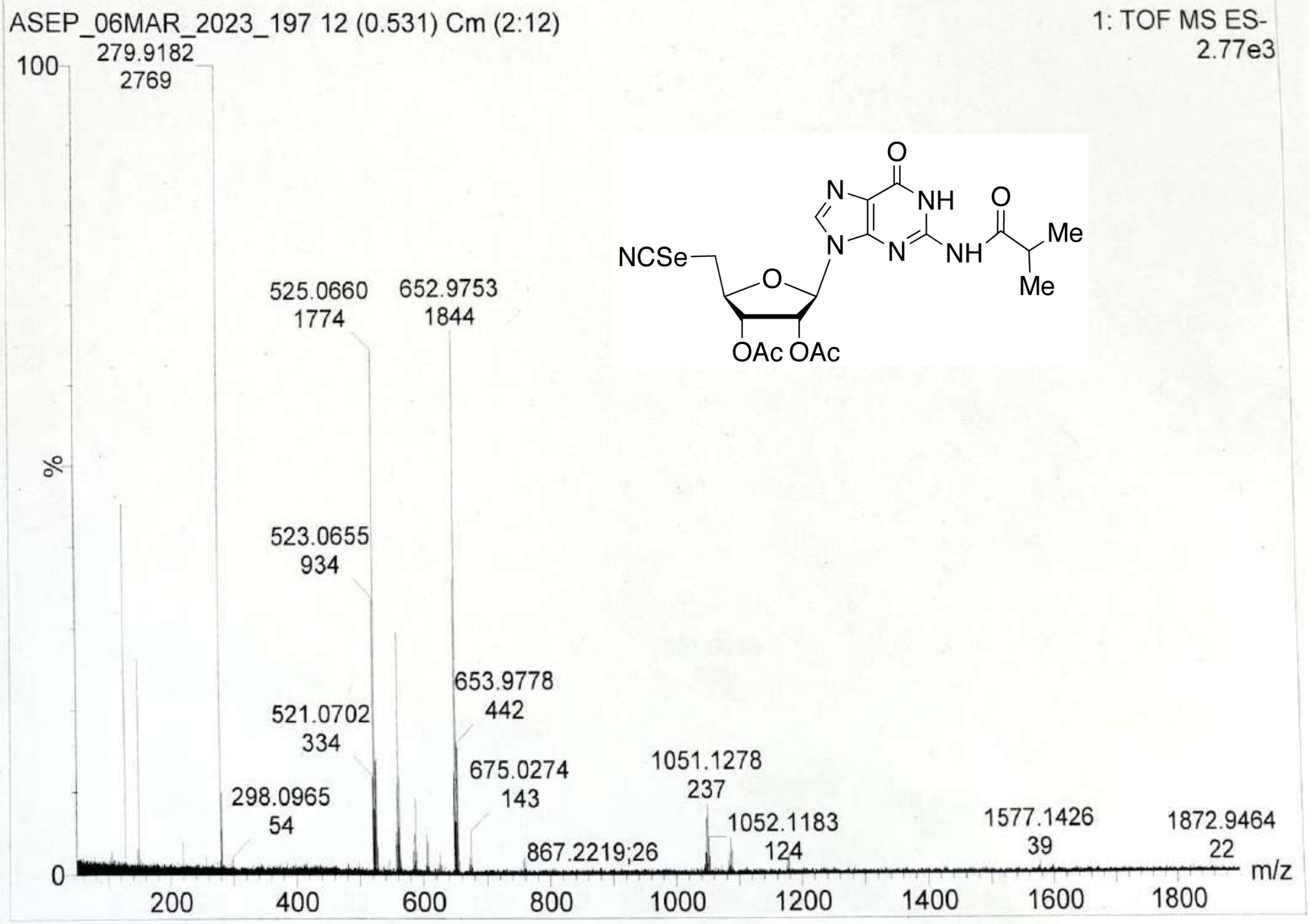
ASEP_06MAR_2023_197 12 (0.531) Cm (2:12)
1: TOF MS ES-
2.77e3
279.9182
2769
525.0660
1774
652.9753
1844
523.0655
934
653.9778
442
521.0702
334
675.0274
143
1051.1278
237
298.0965
54
1052.1183
124
867.2219;26
1577.1426
39
1872.9464
22
100
%
0
200
400
600
800
1000
1200
1400
1600
1800
m/z
NCSe
O
OAc
OAc
N
N
N
NH
O
NH
O
Me
Me

LH-11-77

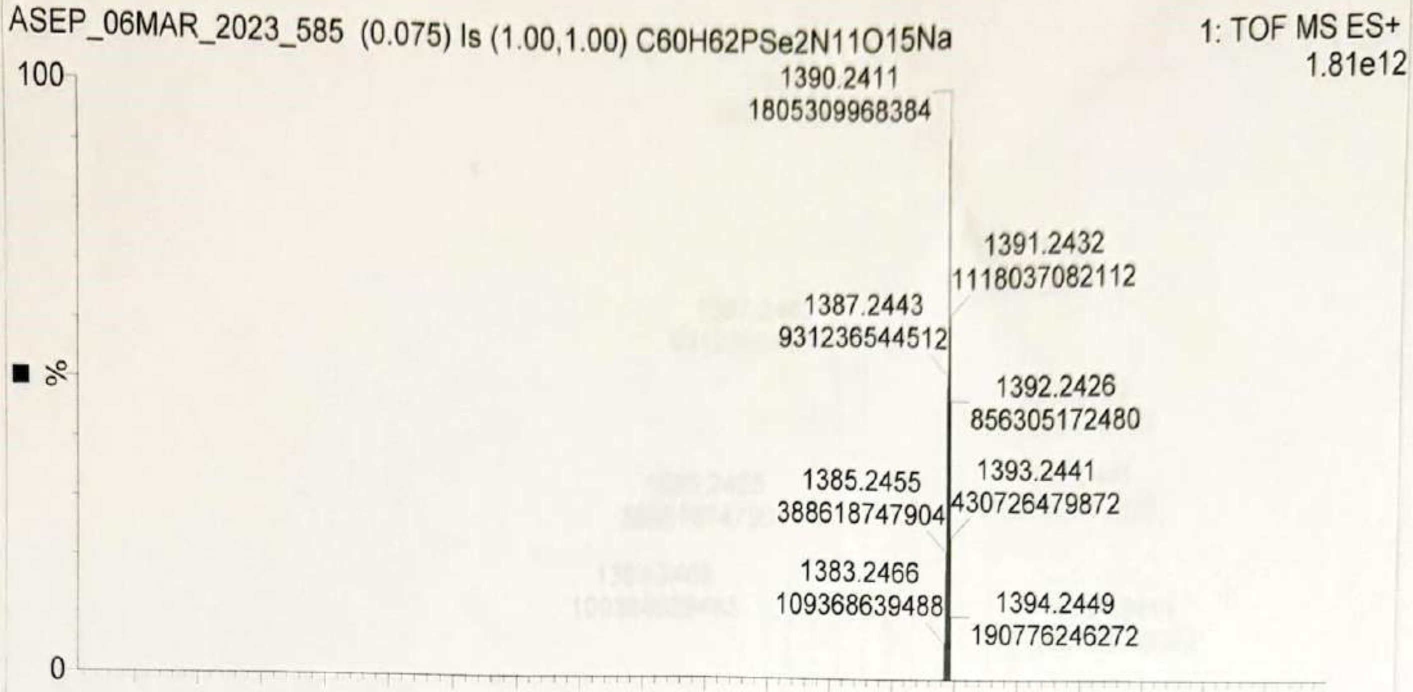

ASEP_06MAR_2023_585 (0.075) Is (1.00,1.00) C60H62PSe2N11O15Na
1: TOF MS ES+
1.81e12
100
%
0
1390.2411
1805309968384
1391.2432
1118037082112
1387.2443
931236544512
1392.2426
856305172480
1385.2455
388618747904
1393.2441
430726479872
1383.2466
109368639488
1394.2449
190776246272

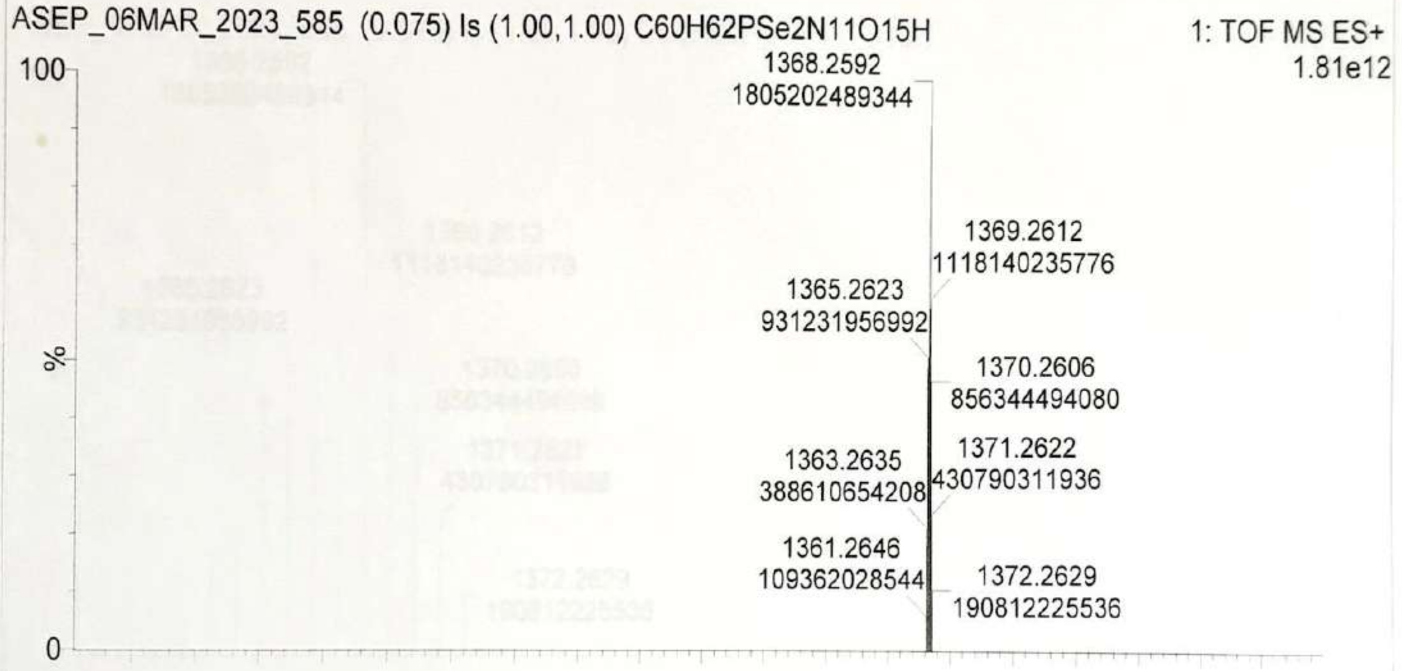

ASEP_06MAR_2023_585 (0.075) Is (1.00,1.00) C60H62PSe2N11O15H
1: TOF MS ES+
1.81e12
100
%
0
1368.2592
1805202489344
1369.2612
1118140235776
1365.2623
931231956992
1370.2606
856344494080
1363.2635
388610654208
1371.2622
430790311936
1361.2646
109362028544
1372.2629
190812225536

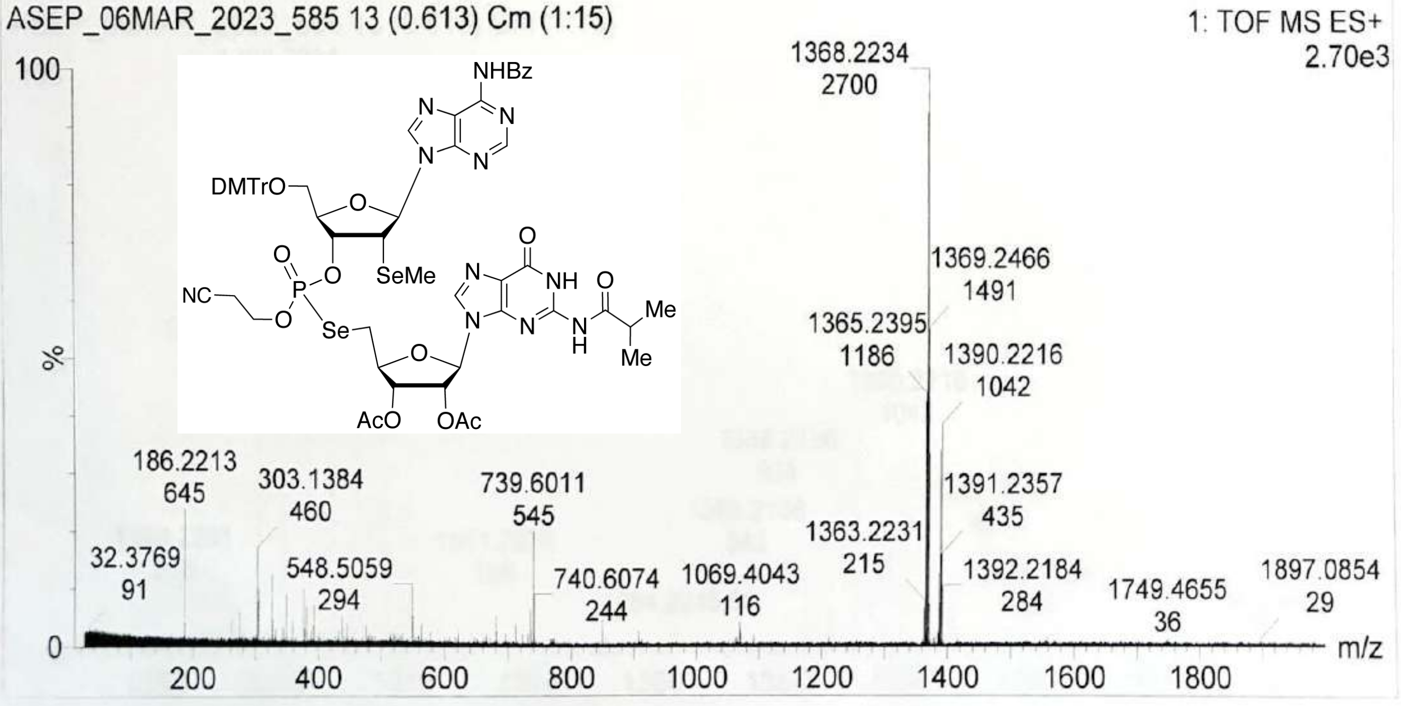

ASEP_06MAR_2023_585 13 (0.613) Cm (1:15)
1: TOF MS ES+
2.70e3
100
%
0
NHBz
N
DMTrO
O
SeMe
NC
O
P
Se
NH
Me
H
Me
AcO
OAc
1368.2234
2700
1369.2466
1491
1365.2395
1186
1390.2216
1042
1391.2357
435
1363.2231
215
1392.2184
284
1749.4655
36
1897.0854
29
186.2213
645
303.1384
460
739.6011
545
32.3769
91
548.5059
294
740.6074
244
1069.4043
116
200
400
600
800
1000
1200
1400
1600
1800
m/z

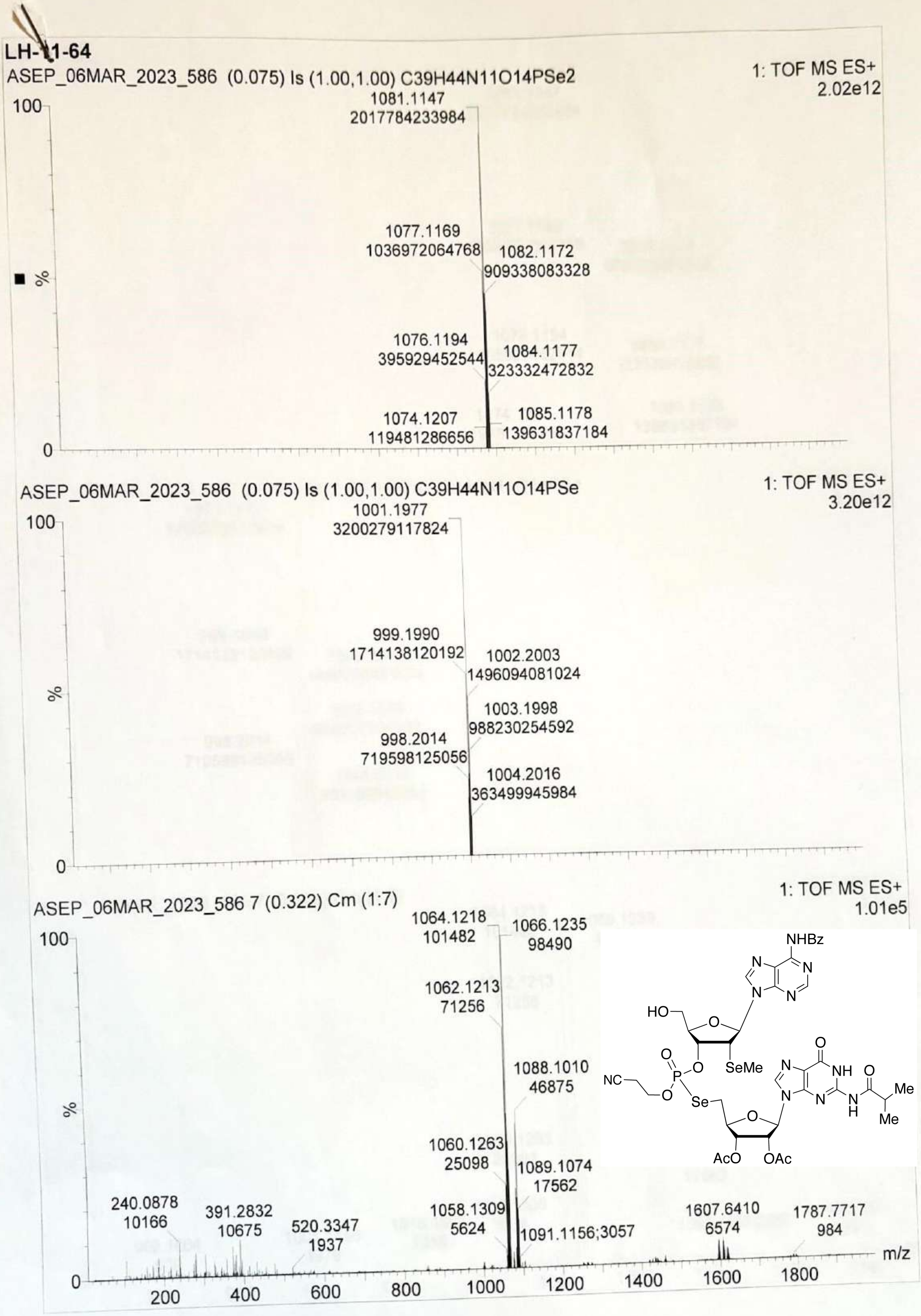
LH-11-64
ASEP_06MAR_2023_586 (0.075) Is (1.00,1.00) C39H44N11O14PSe2
1: TOF MS ES+
2.02e12
1081.1147
2017784233984
1077.1169
1036972064768
1082.1172
909338083328
1076.1194
395929452544
1084.1177
323332472832
1074.1207
119481286656
1085.1178
139631837184
ASEP_06MAR_2023_586 (0.075) Is (1.00,1.00) C39H44N11O14PSe
1: TOF MS ES+
3.20e12
1001.1977
3200279117824
999.1990
1714138120192
1002.2003
1496094081024
1003.1998
988230254592
998.2014
719598125056
1004.2016
363499945984
ASEP_06MAR_2023_586 7 (0.322) Cm (1:7)
1: TOF MS ES+
1.01e5
1064.1218
101482
1066.1235
98490
1062.1213
71256
1088.1010
46875
1060.1263
25098
1089.1074
17562
240.0878
10166
391.2832
10675
520.3347
1937
1058.1309
5624
1091.1156;3057
1607.6410
6574
1787.7717
984
m/z
%
NHBz
HO
SeMe
NC
Se
AcO
OAc
NH
Me
Me

LH-[illegible]-80

asep_06_july2023_291 (0.070) Is (1.00,1.00) C70H75Se2P2N14O21H

1: TOF MS ES+
1.72e12

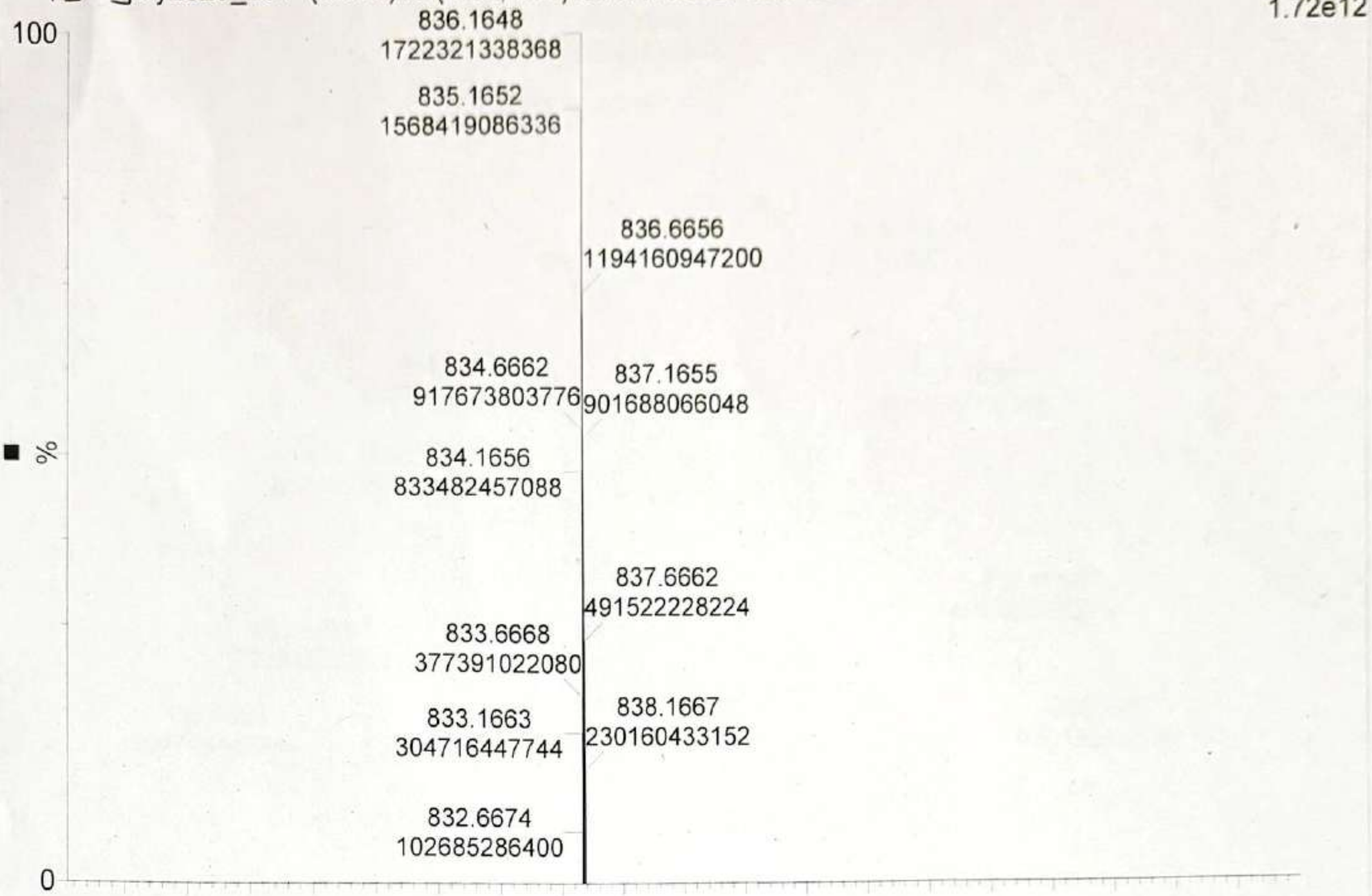

asep_06_july2023_291 15 (0.677) Cm (1:16)

1: TOF MS ES+
2.48e4

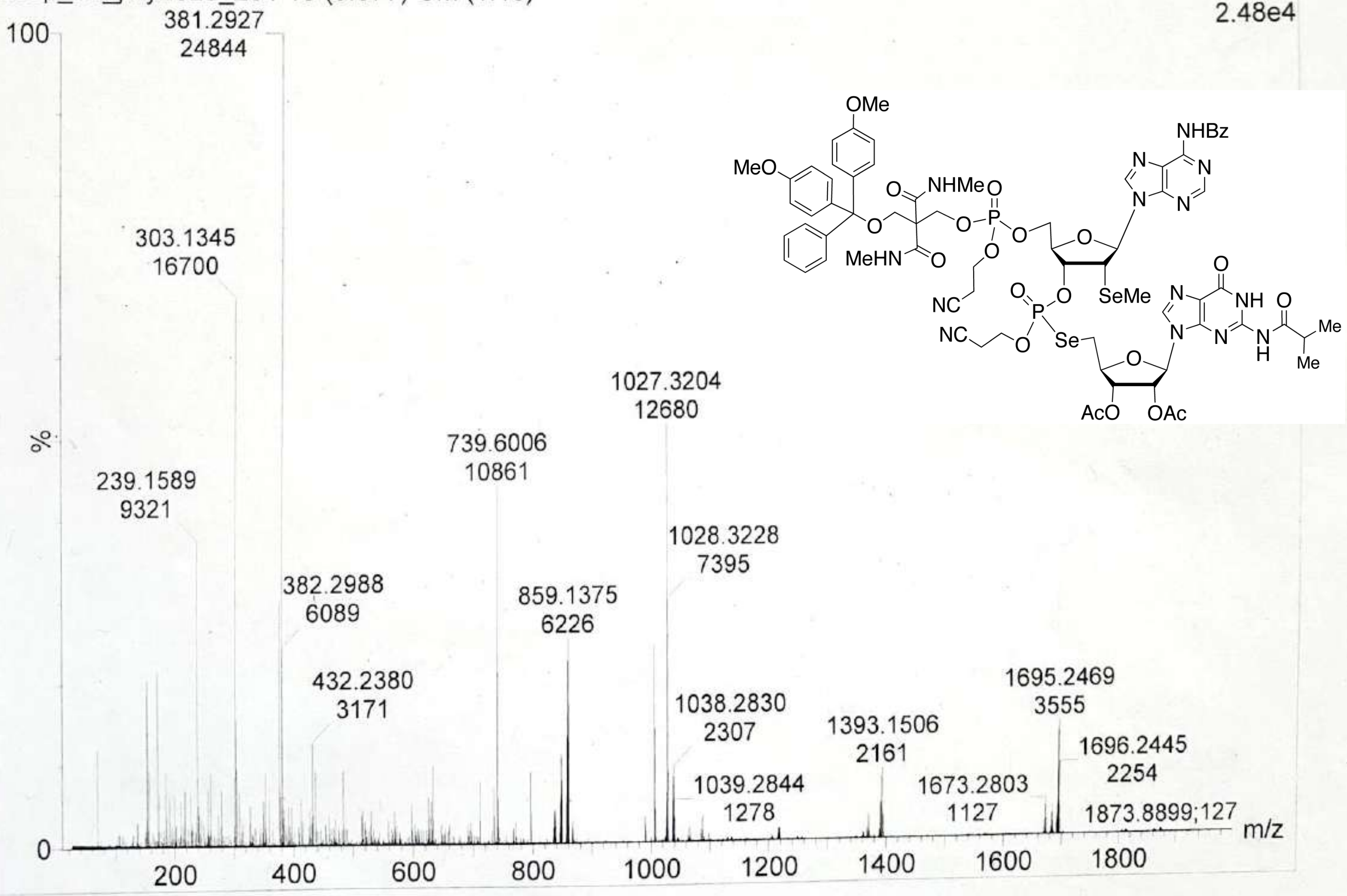

LH-11-79

asep_06_july2023_288 (0.070) Is (1.00,1.00) C50H53PN8O13Na

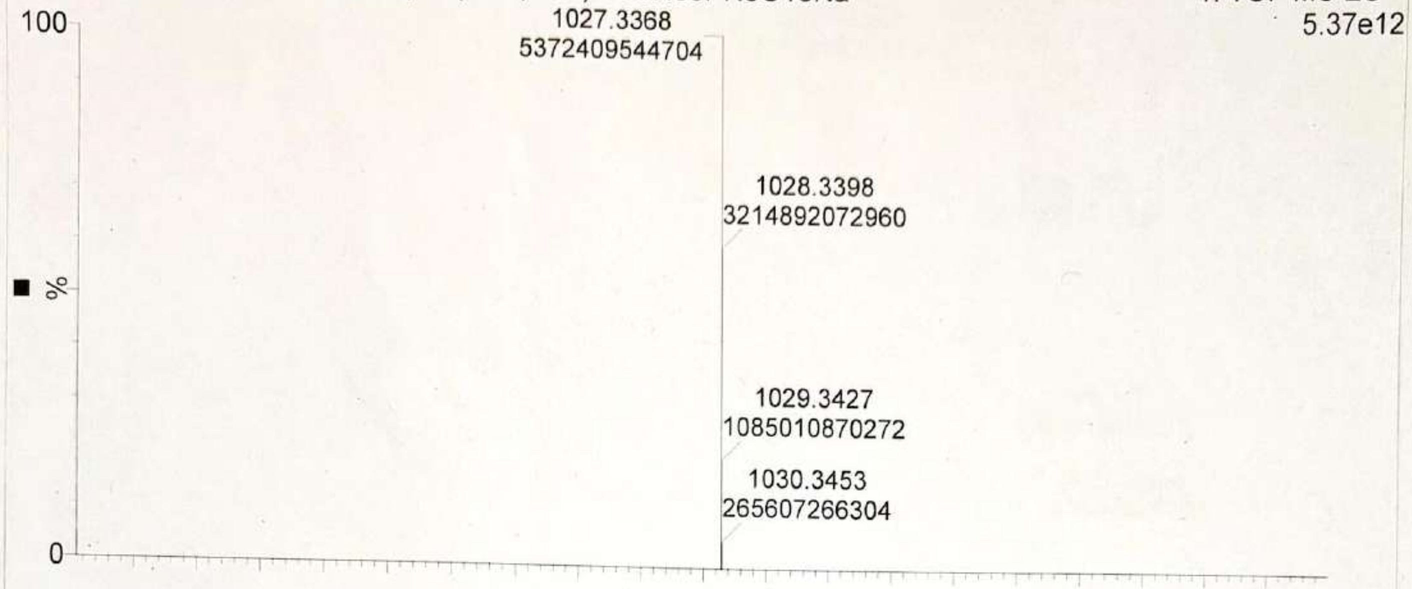

asep_06_july2023_288 (0.070) Is (1.00,1.00) C50H53PN8O13H

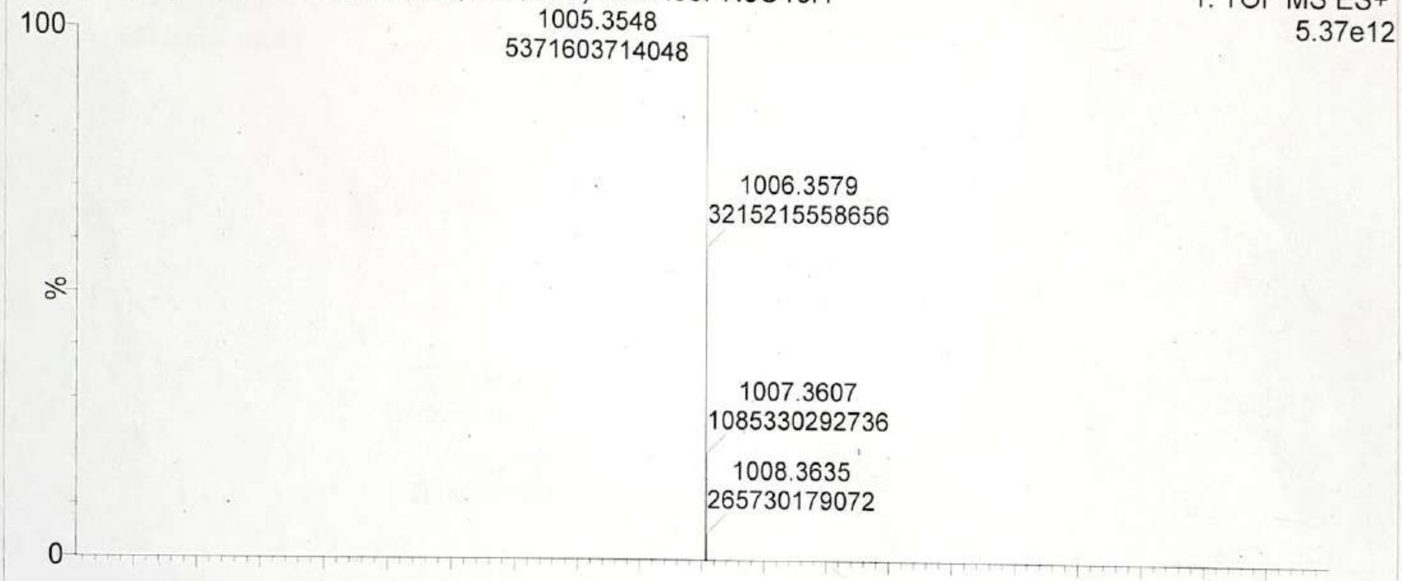

asep_06_july2023_288 6 (0.284) Cm (5:6)

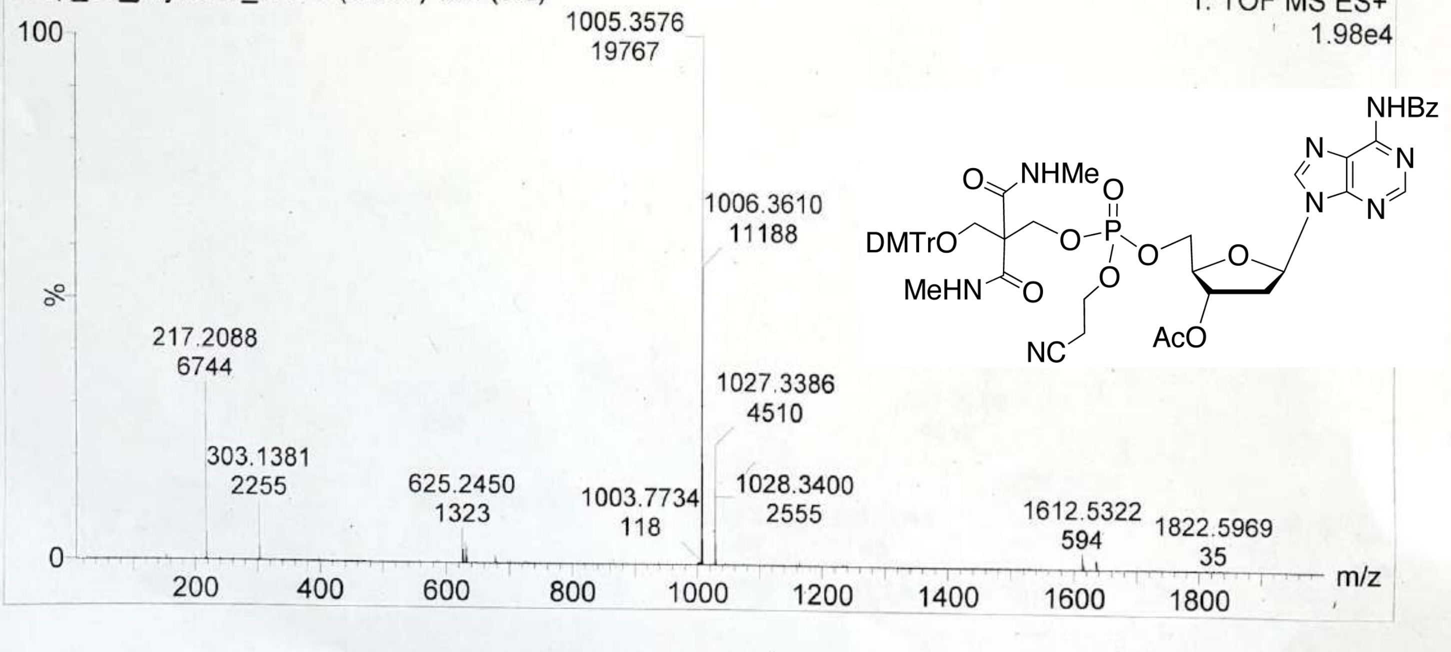

LH-1-64

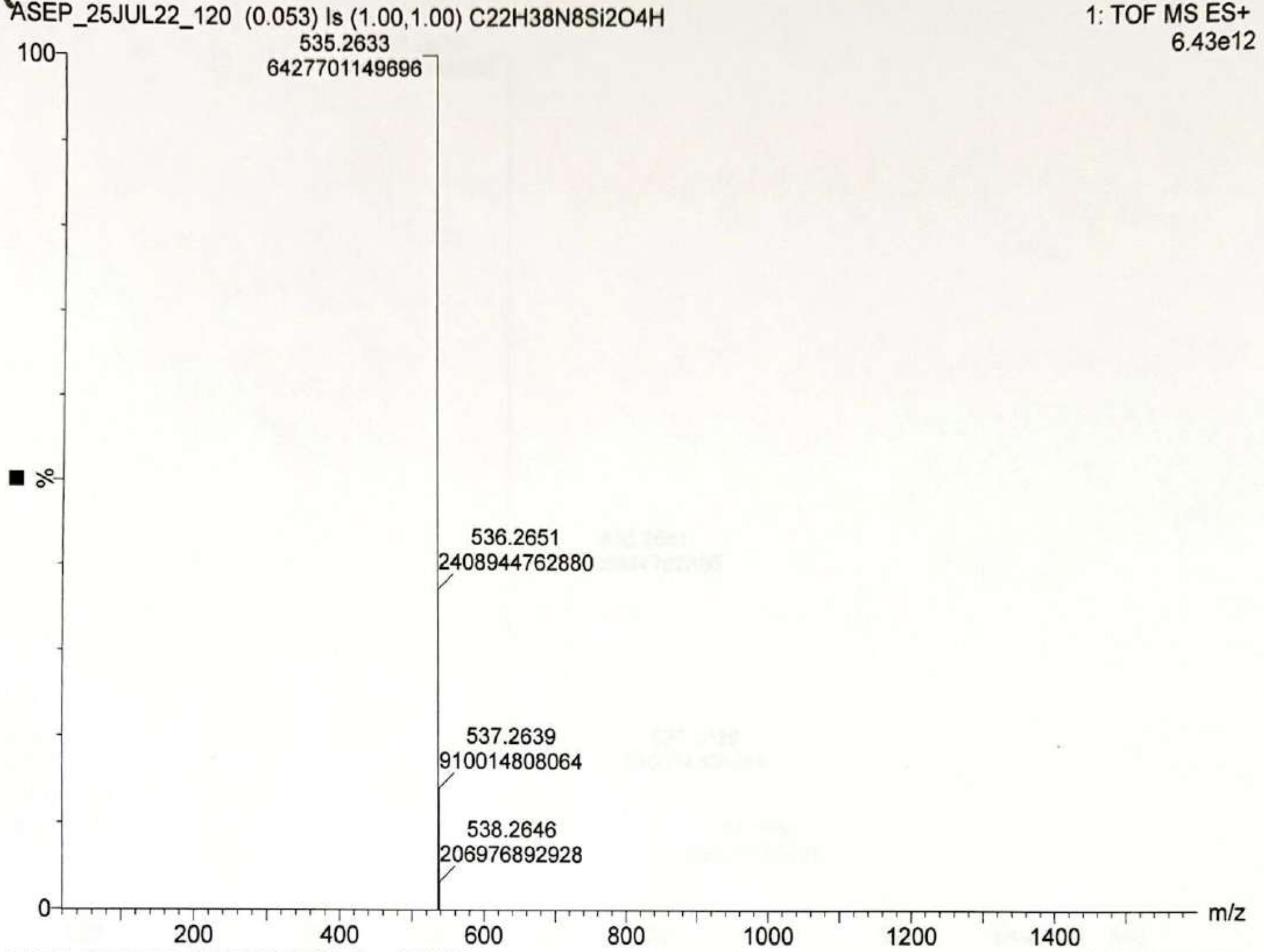

ASEP_25JUL22_120 (0.053) Is (1.00,1.00) C22H38N8Si2O4H
1: TOF MS ES+
6.43e12
535.2633
6427701149696
536.2651
2408944762880
537.2639
910014808064
538.2646
206976892928
100
%
0
200
400
600
800
1000
1200
1400
m/z

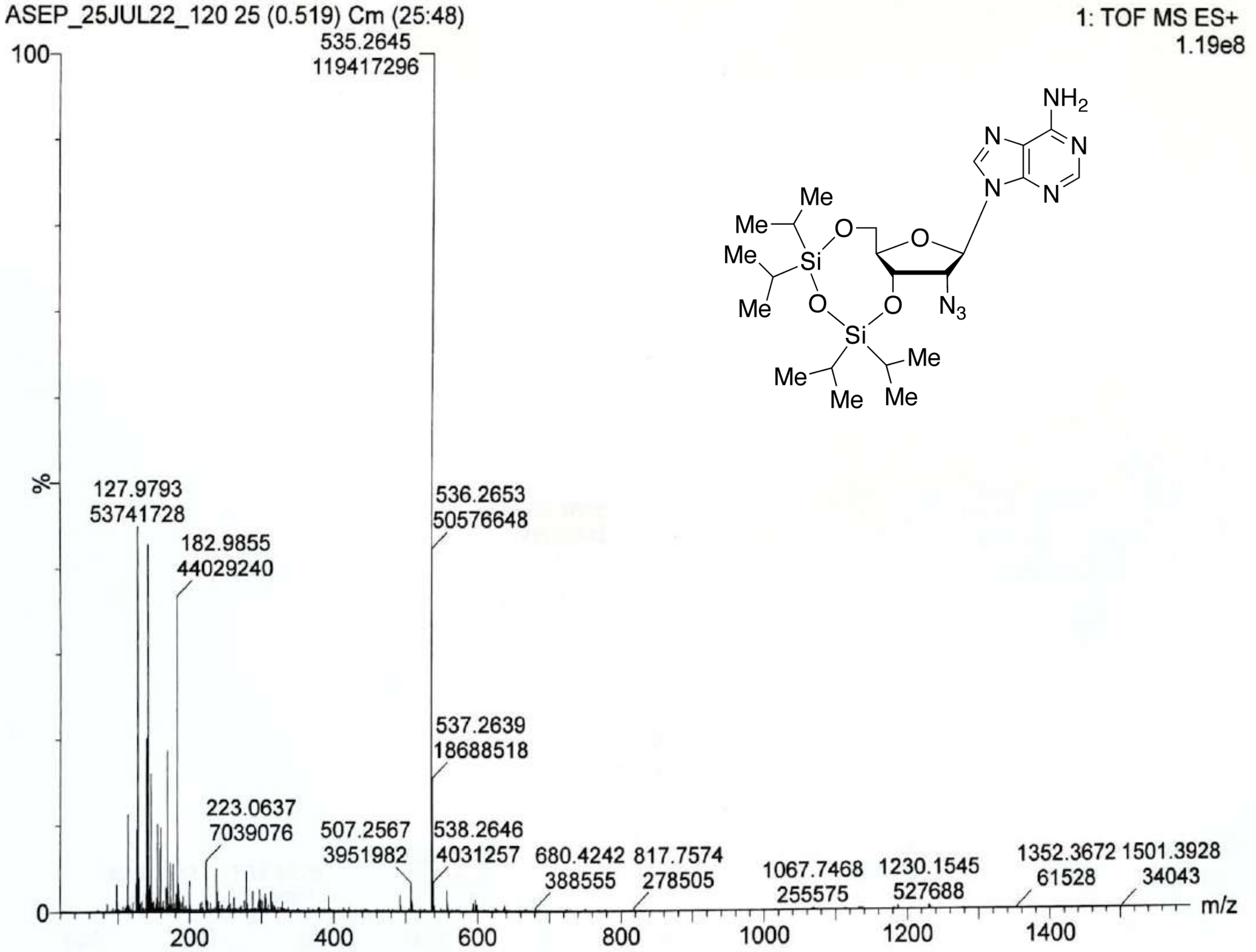

ASEP_25JUL22_120 25 (0.519) Cm (25:48)
1: TOF MS ES+
1.19e8
535.2645
119417296
127.9793
53741728
182.9855
44029240
536.2653
50576648
537.2639
18688518
223.0637
7039076
507.2567
3951982
538.2646
4031257
680.4242
388555
817.7574
278505
1067.7468
255575
1230.1545
527688
1352.3672
61528
1501.3928
34043
100
%
0
200
400
600
800
1000
1200
1400
m/z
NH2
N
N
N
N
O
Me
Me
Me
Me
Me
Me
Me
Me
O
O
O
O
Si
Si
N3

LH-1-58

ASEP_25JUL22_116 (0.053) Is (1.00,1.00) C29H43Si2N5O6H — 1: TOF MS ES+

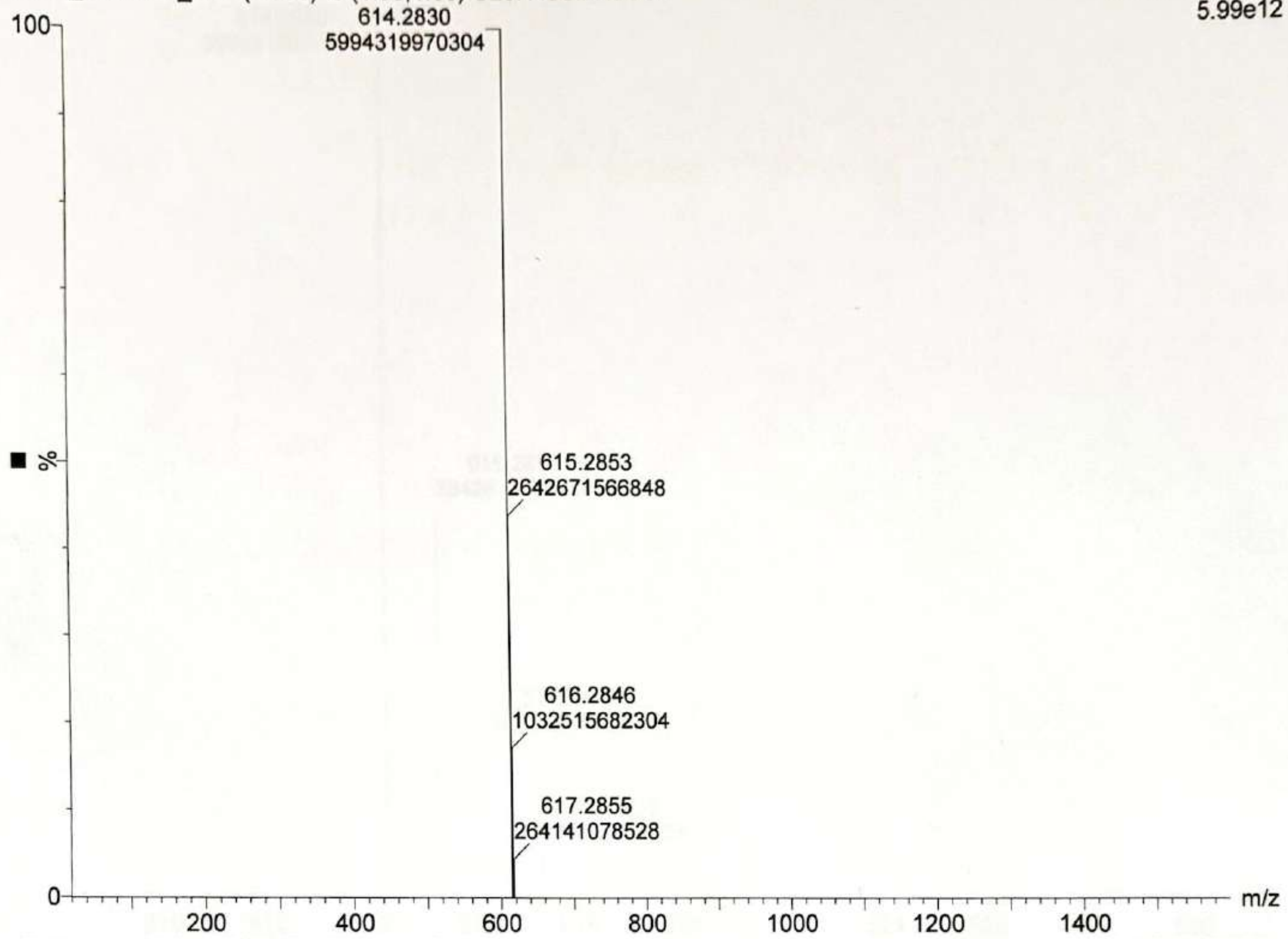

ASEP_25JUL22_116 23 (0.465) Cm (23:47) — 1: TOF MS ES+

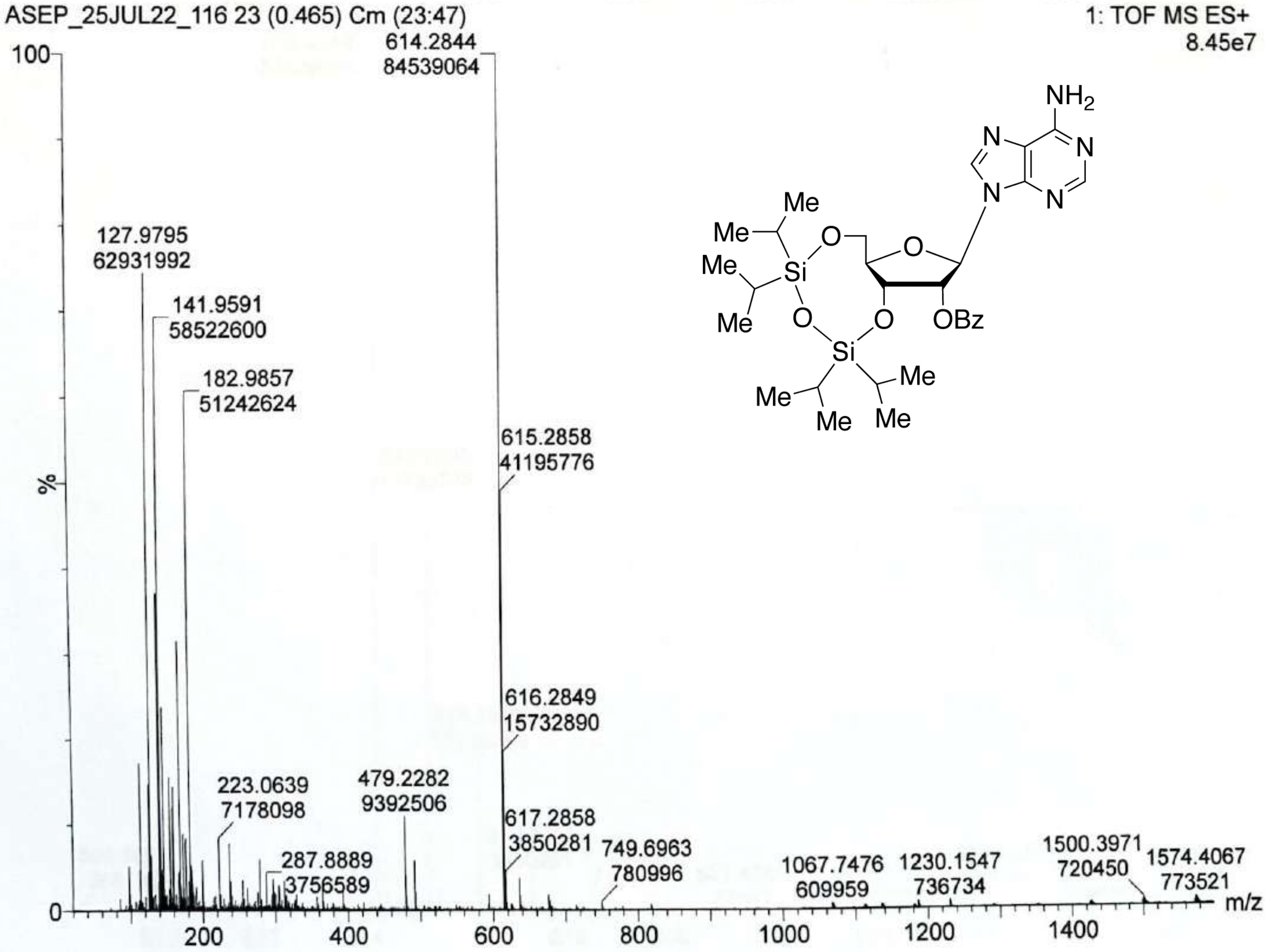

LH-1-59

ASEP_25JUL22_117 (0.053) Is (1.00,1.00) C24H41Si2SN5O5H

1: TOF MS ES+ 6.02e12

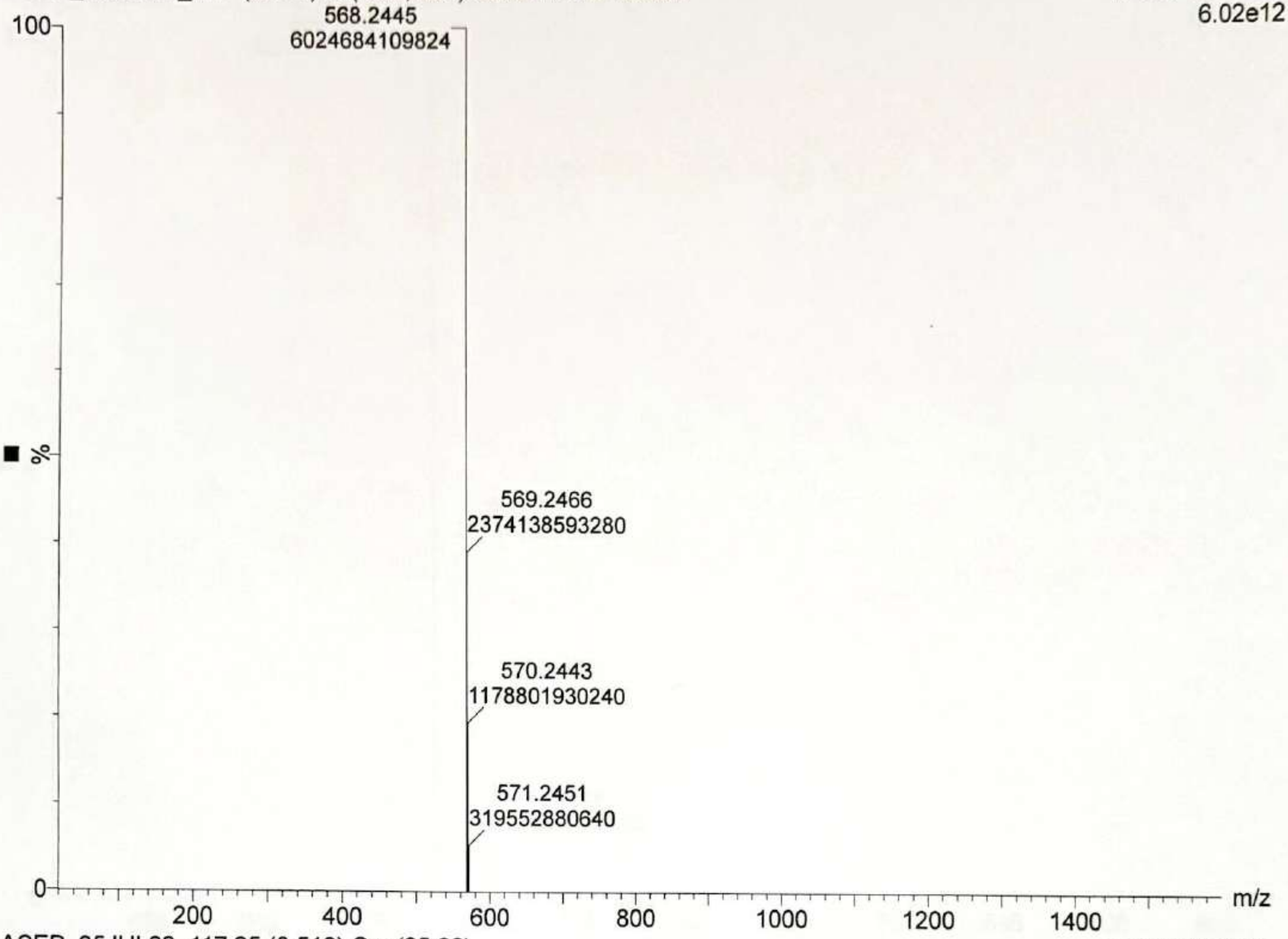

ASEP_25JUL22_117 25 (0.519) Cm (25:38)

1: TOF MS ES+ 3.14e7

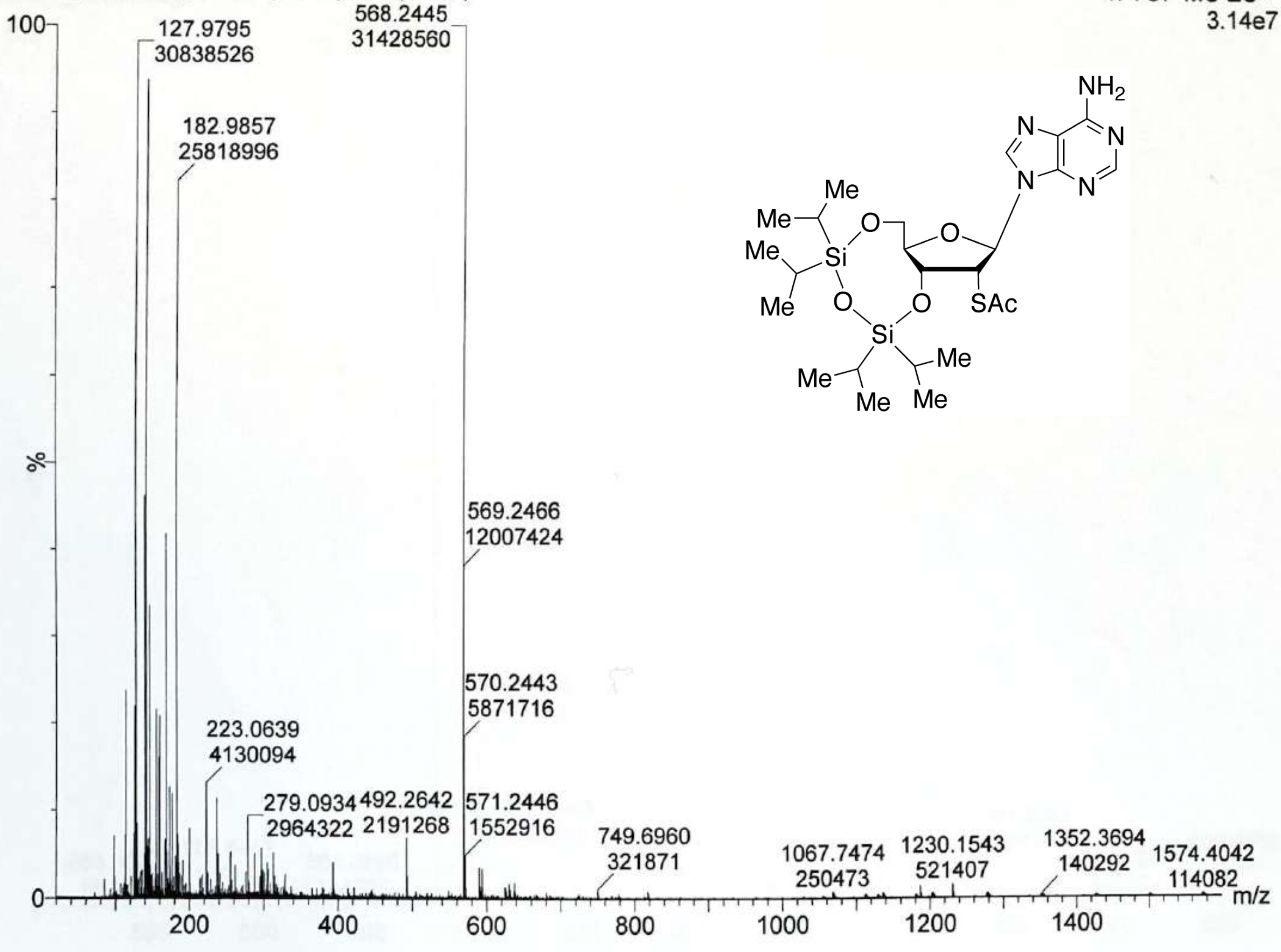

LH-1-66

ASEP_25JUL22_122 (0.053) Is (1.00,1.00) C22H38N5Si2BrO4H

1: TOF MS ES+
3.66e12

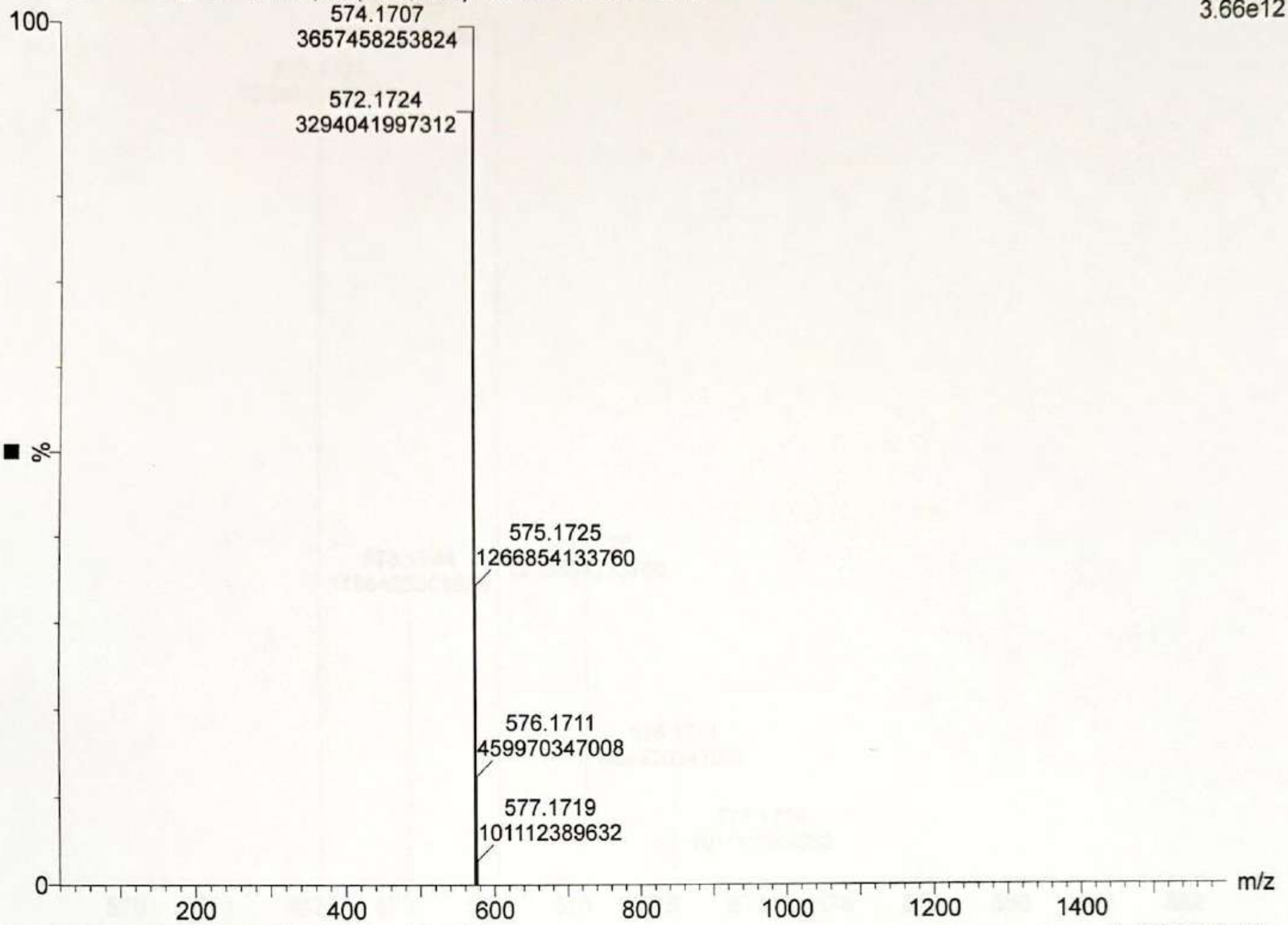

ASEP_25JUL22_122 25 (0.519) Cm (25:41)

1: TOF MS ES+
3.52e7

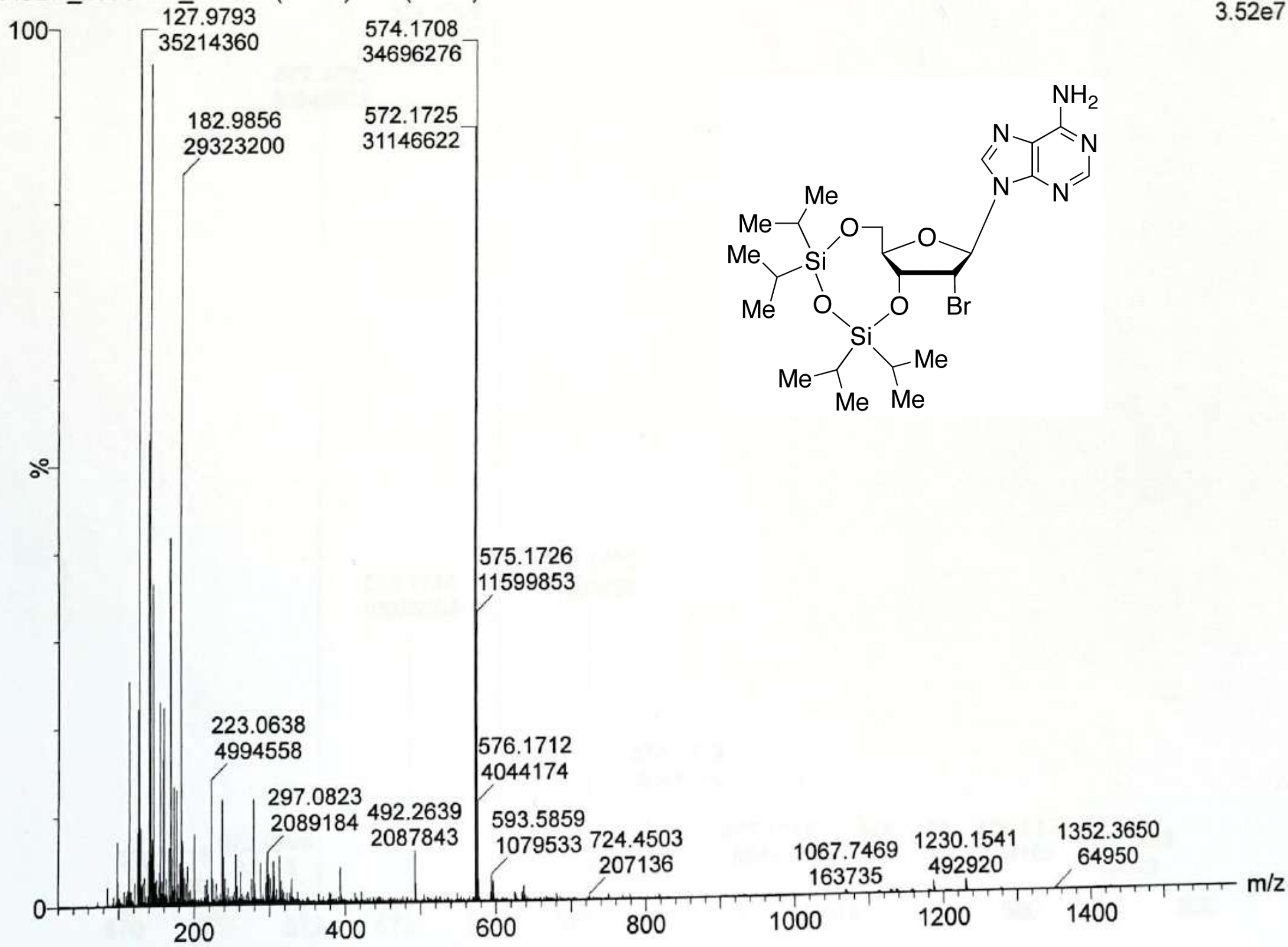

Supplement: Supplementary file 1 — Experimental procedures and full characterization data including spectra are given in the Supporting Information. Supporting File: chem70615‐sup‐0001‐SuppMat.pdf. [file CHEM-32-e02321-s001.pdf]
